# Supplementary material for: Biochemical and structural characterization of two cif-like epoxide hydrolases from Burkholderia cenocepacia
Source: Curr Res Struct Biol. 2021 Feb 21;3:72–84. doi: 10.1016/j.crstbi.2021.02.002 (PMC8244358; doi:10.1016/j.crstbi.2021.02.002)
Supplement: Multimedia component 1 [file mmc1.docx]

Supplemental Materials for Taher *et al.*

The following supplemental materials are included:

- **Table S1** Preliminary screen for Cfl1 and Cfl2 hydrolysis activity against a panel of epoxides.
- **Table S2** Mean ConSurf conservation scores of various interfaces.
- **FIG S1** Alignment of Cfl1 sequences from *B. cenocepacia* strains HI2424 and J2315.
- **FIG S2** Electron microscopy images of negatively stained single particles of Cfl2.
- **FIG S3** Quaternary structure differences between Cfl1 and Cfl2.
- **FIG S4** Structural alignment of the Cfl1-D123S monomer to the Cfl1-WT monomer.
- **Supplemental Movie 1** Comparison of the first non-trivial normal mode of Cif-like proteins.
- **Figure S5** Evolutionary conservation of the interfaces of the Cfls calculated using Cif-like homologs only
- **Supplementary Files** Rosetta xml protocols and multiple sequence alignments related to Figure 8 and Figure S5

**Table S1** Preliminary screen for Cfl1 and Cfl2 hydrolysis activity against a panel of epoxides.

|  | **ΔA_490_** | | |
| --- | --- | --- | --- |
| **xenobiotic substrates** | **Cfl1** | **Cfl2** | **aCif** |
| *S*-styrene oxide | -0.086 | 0.038 | -0.200 |
| *R*-styrene oxide | -0.239 | 0.041 |  |
| 1,2-epoxyoctane | -0.037 | -0.037 |  |
| 1,2-epoxyhexane | -0.022 | -0.038 |  |
| cyclohexene oxide | -0.029 | -0.044 |  |
| epibromohydrin | -0.032 | -0.035 |  |
| propylene oxide | -0.016 | -0.036 |  |
| glycidol | nd | -0.059 |  |
| *cis*-stilbene oxide | nd | -0.078 |  |
| *trans*-stilbene oxide | nd | 0.006 |  |
| 1,2-epoxy-4-vinylcyclohexane | nd | -0.041 |  |
| allyl glycidyl ether | nd | -0.036 |  |
| glycidyl methacrylate | nd | -0.026 |  |
| (+)-limonene oxide | nd | -0.045 |  |
|  |  |  |  |
| **biological substrates** |  |  |  |
| 11(12)-EET | nd | -0.039 |  |
| 8(9)-EET | nd | -0.039 |  |
| 16(17)-EpDPA | nd | -0.048 |  |
| 14(15)-EpETE | nd | -0.029 |  |
| 5(6)-EET | -0.001 | -0.034 |  |
| 14(15)-EET | 0.004 | -0.032 |  |
| 17(18)-EpETE | 0.016 | -0.026 |  |

The adrenochrome end-point assay was performed as described (see *Materials and Methods* section). ΔA_490_ values represent the difference in colorimetric readouts between the experimental reactions and either the no-substrate reaction, or the D123S catalytic mutant reaction in the case of Cfl1 vs. biological substrates (n=1). Substrate hydrolysis should result in negative values. nd: not determined.

**Table S2** Mean ConSurf conservation scores of the non-interface surface residues and of the residues in each monomer:monomer interface of Cfl1 and Cfl2.

|  | **residue location** | **<ConSurf score>** |
| --- | --- | --- |
| **Cfl1** | non-interface | 0.45 |
|  | 1:2 | 0.07 |
|  | 1:3 | 0.18 |
|  | 1:4 | 0.21 |
|  | 1:7 | 0.70 |
|  |  |  |
| **Cfl2** | non-interface | 0.37 |
|  | 1:2 | 0.20 |
|  | 1:3 | 0.39 |
|  | 1:4 | 0.10 |
|  | 1:9 | 0.87 |

Higher ConSurf scores indicate more variability.


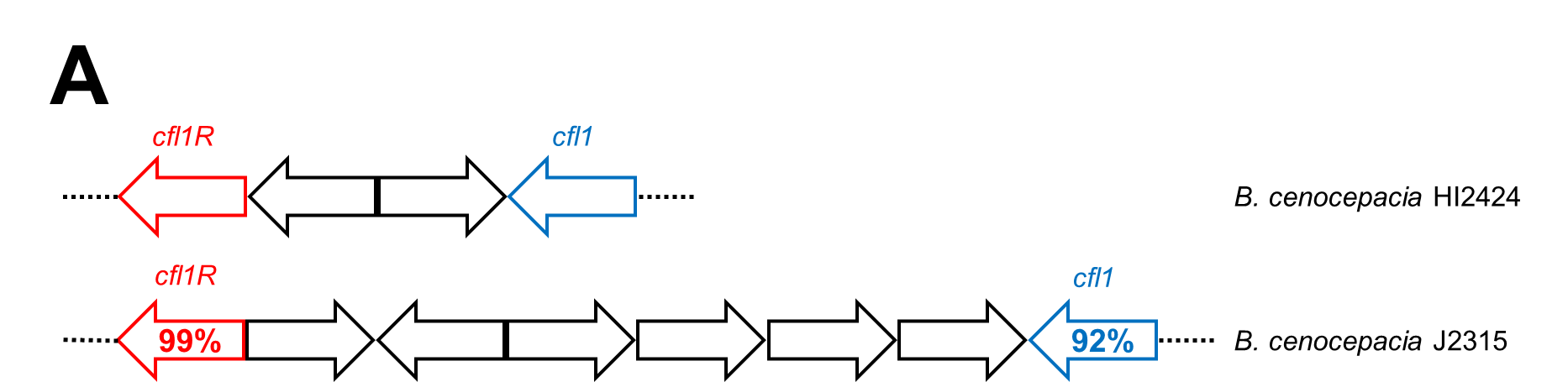


**B**

Cfl1_(H)_ MQN---EPSMSGMPAPGLPAGFDRRFSRRYAQVDDVRLHYVTGGPDDGEL 47

||| | || |||||||||||:|||||||||:||||||||||||||||:

Cfl1_(J)_ MQNERSEQSMPGMPAPGLPAGFERRFSRRYAQLDDVRLHYVTGGPDDGEM 50

Cfl1_(H)_ VVLL**HGWP**QTWYTWRHVMPVLAQEGYRVVAVDYRGAGESDKPLGGYDKAS 97

||||||||||||||||||| ||::||||||||||||||||||||||||||

Cfl1_(J)_ VVLL**HGWP**QTWYTWRHVMPALAEDGYRVVAVDYRGAGESDKPLGGYDKAS 100

Cfl1_(H)_ MAGDIRALVRQLGATRIHLVGR**D**IGVMVAYAYAAQRPAEIVKLAML**D**VPV 147

||||||||| ||||||||||||||||||||||||| | ||||||||||||

Cfl1_(J)_ MAGDIRALVHQLGATRIHLVGR**D**IGVMVAYAYAAQWPTEIVKLAML**D**VPV 150

Cfl1_(H)_ PGTRIWDEAKARADPQIW**H**FGLHQQRDIAELLIAGKEHAYILDFYKKRAH 197

||||||||||| ||||||||||||||||||:|||||| |||||||||| |

Cfl1_(J)_ PGTRIWDEAKASADPQIW**H**FGLHQQRDIAEMLIAGKERAYILDFYKKRTH 200

Cfl1_(H)_ VALSNDDIAVYADAYAAPGALRAGFEL**Y**RAFPQDETQFKAFMKHKLPMPV 247

||||||||||||||||||||||||||||||||||||:|||||||||||||

Cfl1_(J)_ VALSNDDIAVYADAYAAPGALRAGFEL**Y**RAFPQDETRFKAFMKHKLPMPV 250

Cfl1_(H)_ LALAGDKSNGAKEFDMAKELALDVRGAVAPNTG**H**WLPDENPAFLTRQLLD 297

||||||||||||| |||:||||||||||||||||||||||||||||||||

Cfl1_(J)_ LALAGDKSNGAKELDMARELALDVRGAVAPNTG**H**WLPDENPAFLTRQLLD 300

Cfl1_(H)_ FFREPAPNR 306

|||| | |

Cfl1_(J)_ FFREAASGR 309

**Figure S1.** Alignment of Cfl1 sequences from *B. cenocepacia* strains HI2424 (Cfl1_(H)_) and J2315 (Cfl1_(J)_). (A) The genomic regions that encompass *cfl1*-*cfl1R* genes are shown. The percent sequence identities of Cfl1 (blue) and Cfl1R (red) homologs in strains HI2424 and J2315 calculated using EMBOSS Needle are indicated on each corresponding gene. (B) Pair-wise amino-acid sequence alignment of Cfl1_(H)_ and Cfl1_(J)_ calculated using EMBOSS Needle is shown. The subscripts (H) and (J) refer to *B. cenocepacia* strains HI2424 and J2315, respectively. Vertical lines and colons indicate sequence identity and similarity, respectively. The canonical epoxide hydrolase H-G-X-P motif and catalytic residues are highlighted in bold blue and bold red, respectively.


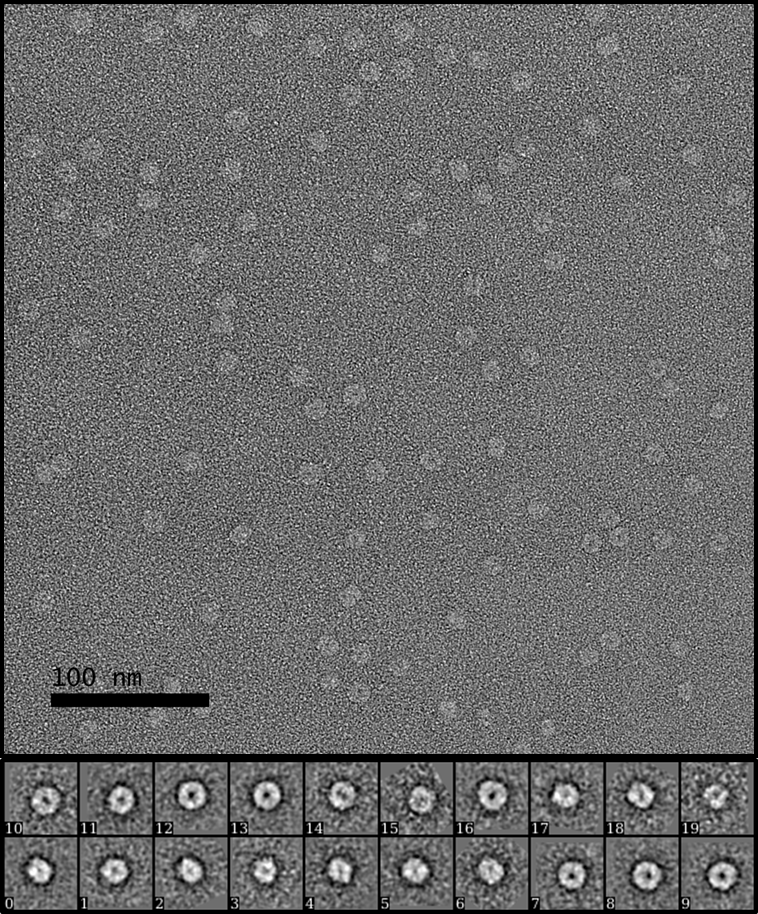


**Figure S2.** Electron microscopy images of negatively stained single particles of Cfl2. Top: a representative raw electron micrograph of Cfl2 particles negatively stained with uranyl formate. Bottom: twenty class-average profiles of Cfl2 particles. For experimental details, see *Materials and Methods* section.


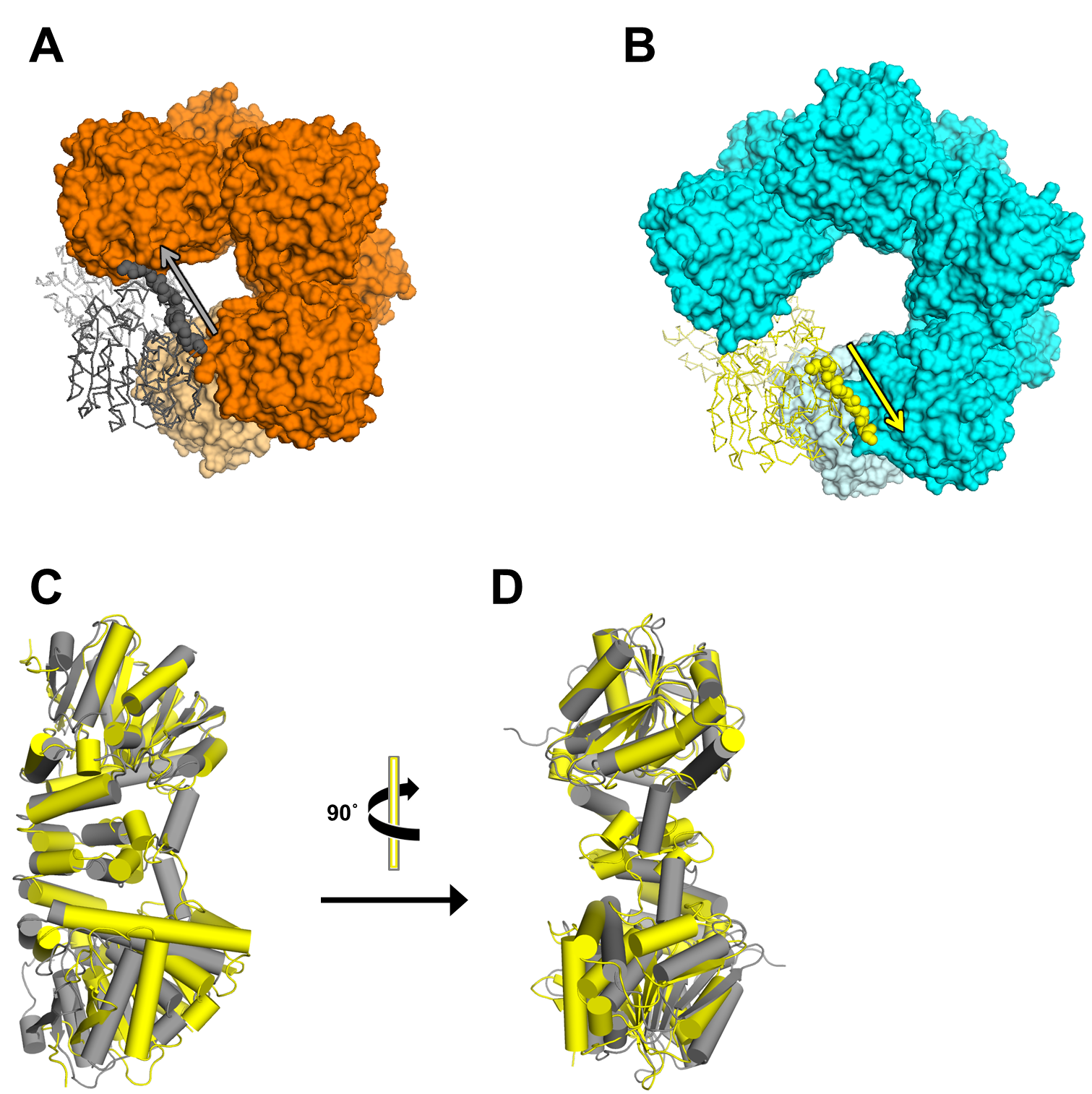


**Figure S3.** Quaternary structure differences between Cfl1 and Cfl2. (A) Top view of the Cfl1 oligomer. Six subunits are shown in surface representation and colored orange, and two subunits are shown as C_α_ traces and colored grey. The N-terminus of one of the subunits is shown as spheres. An arrow indicates the direction the N-terminus extends from one subunit to the other. (B) Top view of the Cfl2 oligomer. Eight subunits are shown in surface representation and colored cyan, and two subunits are shown as C_α_ traces and colored yellow. The N-terminus of one of the subunits is shown as spheres. An arrow indicates the direction the N-terminus extends from one subunit to the other. (C) View from an adjacent ring dimer of the Cfl1 (grey) and Cfl2 (yelow) dimers aligned using the top subunit and shown as cartoons with cylindrical α-helices. (D) View from outside the ring of the dimers shown in (C), with a 90˚ rotation about the long axis of the dimers as indicated.


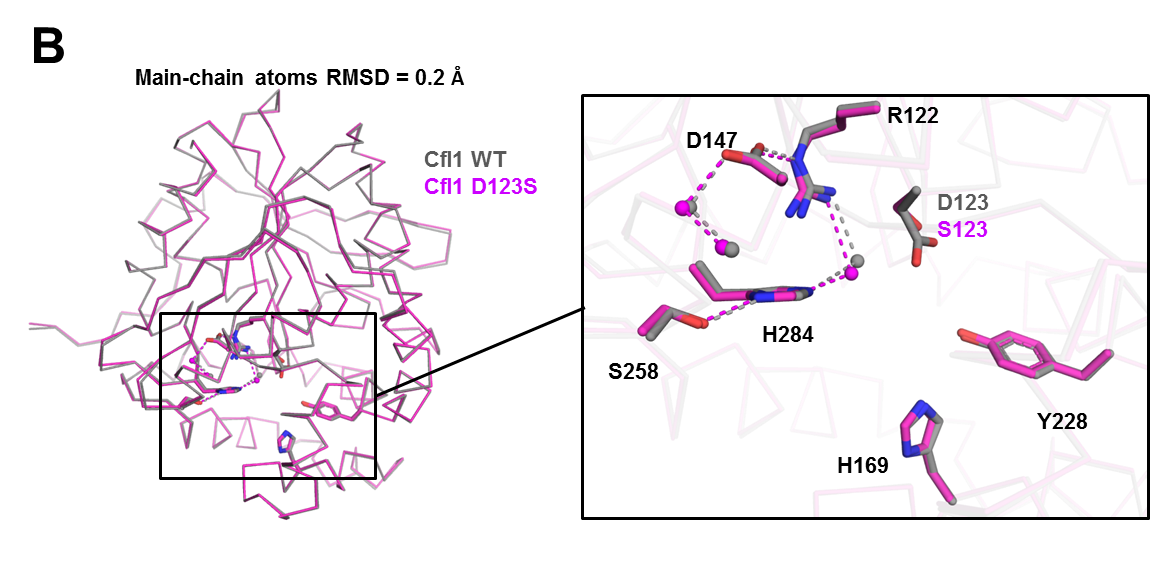


**Figure S4.** Structural alignment of the Cfl1-D123S monomer to the Cfl1-WT monomer. The main-chain atoms of the Cfl1-WT (grey) and Cfl1-D123S (magenta) monomers were aligned using PyMol. The inset shows a closer view of the presumed catalytic residues (sticks) and waters (spheres). Electrostatic interactions are depicted as dashed lines.

**Supplemental Movie 1** Comparison of the first non-trivial normal mode of Cif-like proteins, including Cif, aCif, and the extracted dimers of Cfl1 and Cfl2. From left to right: Cif, aCif, Cfl1, and Cfl2. The trajectories of mode 7 as calculated by Bio3D are shown for each protein. In the case of Cfl1 and Cfl2, the calculation was performed on the dimer subunit after it was extracted from its respective oligomer (See Materials and Methods for details).


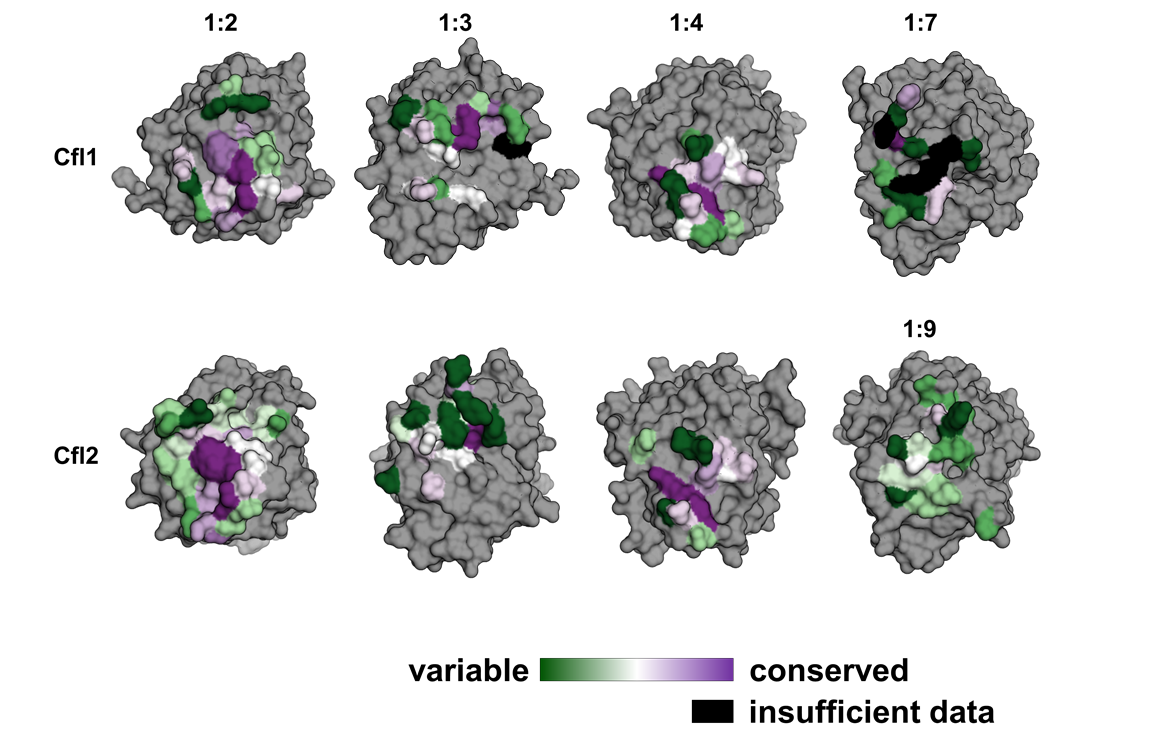


**Figure S5.** Evolutionary conservation of the interfaces of the Cfls calculated using Cif-like homologs only. The interfaces of Cfl1 and Cfl2 are shown on each protein’s monomer subunit as surfaces. The interfaces are labeled at the top according to the monomer numbering used in Figure 7. The interface residues calculated by Rosetta are colored according to the ConSurf conservation score as indicated by the color legend, and all other residues are colored grey.
