# Supplementary material for: Biochemical and structural characterization of two cif-like epoxide hydrolases from Burkholderia cenocepacia
Source: Curr Res Struct Biol. 2021 Feb 21;3:72–84. doi: 10.1016/j.crstbi.2021.02.002 (PMC8244358; doi:10.1016/j.crstbi.2021.02.002)
Supplement: Multimedia component 3 [file mmc3.zip › Cfl1_Cif-like-only_MSA.html]

ConSurf Color-Coded MSA

# ConSurf Color-Coded MSA

|  |  |  |  |  |  |  |  |  |  |  |  |  |  |  |  |  |  |  |  |  |  |  |  |  |  |  |  |  |  |  |  |  |  |  |  |  |  |  |  |  |  |  |  |  |  |  |  |  |  |  |
| --- | --- | --- | --- | --- | --- | --- | --- | --- | --- | --- | --- | --- | --- | --- | --- | --- | --- | --- | --- | --- | --- | --- | --- | --- | --- | --- | --- | --- | --- | --- | --- | --- | --- | --- | --- | --- | --- | --- | --- | --- | --- | --- | --- | --- | --- | --- | --- | --- | --- | --- |
| 001 UniRef90\_L9WLS3\_16\_284 | - | - | - | - | - | - | - | - | - | - | - | - | - | - | - | - | - | - | - | - | - | - | - | - | - | - | - | - | - | - | - | - | - | - | - | - | - | - | - | - | - | - | - | - | - | R | V | N | G | - |
| 002 UniRef90\_A0A0M4FVH2\_24\_312 | - | - | - | - | - | - | - | - | - | - | - | - | - | - | - | - | - | - | - | - | - | R | P | E | P | S | - | - | - | L | D | G | - | - | - | - | - | - | - | F | K | H | R | F | Q | T | V | D | G | - |
| 003 UniRef90\_UPI0016621A44\_4\_265 | - | - | - | - | - | - | - | - | - | - | - | - | - | - | - | - | - | - | - | - | - | - | - | - | - | - | - | - | - | - | - | - | - | - | - | - | - | - | - | - | - | - | - | - | - | - | - | S | G | - |
| 004 UniRef90\_A0A447J1A9\_28\_317 | - | - | - | - | - | - | - | - | - | - | P | K | P | P | V | - | - | - | - | - | - | - | - | - | - | - | - | - | - | - | - | T | A | Q | V | N | E | A | D | F | Q | H | H | Y | T | D | V | A | G | - |
| 005 UniRef90\_A0A1Y6D2S0\_29\_302 | - | - | - | - | - | - | - | - | - | - | - | - | - | - | - | - | - | - | - | - | - | - | - | - | - | - | - | - | - | - | - | - | - | - | - | - | - | - | - | - | K | S | G | F | A | Q | V | N | G | - |
| 006 UniRef90\_A0A0G2FGE6\_47\_303 | - | - | - | - | - | - | - | - | - | - | - | - | - | - | - | - | - | - | - | - | - | - | - | - | - | - | - | - | - | - | - | - | - | - | - | - | - | - | - | - | - | - | - | - | - | - | - | - | - | - |
| 007 UniRef90\_F8JLL5\_12\_309 | - | - | - | - | - | - | - | - | - | - | - | G | N | P | V | - | - | - | - | R | D | L | P | L | P | D | - | - | - | L | A | G | - | - | - | - | - | - | - | F | T | H | R | W | V | D | A | D | G | - |
| 008 UniRef90\_A0A4R2Z7A8\_3\_305 | - | - | - | - | - | - | - | Q | N | M | P | S | T | P | V | A | T | A | L | F | G | I | P | D | P | T | - | - | - | L | K | G | - | - | - | - | - | - | - | F | H | H | H | Y | A | T | V | D | G | - |
| 009 UniRef90\_UPI0016149F5E\_5\_284 | - | - | - | - | - | - | - | - | - | - | - | - | - | - | - | - | - | - | - | - | - | - | - | - | - | - | - | - | - | - | - | - | - | - | - | - | E | N | H | I | T | H | E | Y | V | R | I | A | G | - |
| 010 UniRef90\_A0A327RPK7\_10\_284 | - | - | - | - | - | - | - | - | - | - | - | - | - | - | - | - | - | - | - | - | - | - | - | - | - | - | - | - | - | - | - | - | - | - | - | - | - | - | - | Y | E | H | R | I | A | R | V | N | N | - |
| 011 UniRef90\_UPI00131EC7B5\_30\_311 | - | - | - | - | - | - | - | - | - | - | - | - | - | - | - | - | - | - | - | - | - | - | - | - | - | - | - | - | - | - | - | G | - | - | - | - | - | - | - | F | E | H | R | Y | A | T | V | D | G | - |
| 012 UniRef90\_A0A1N6RLL9\_1\_202 | - | - | - | - | - | - | - | - | - | - | - | - | - | - | - | - | - | - | - | - | - | - | - | - | - | - | - | - | - | - | - | - | - | - | - | - | - | - | - | - | - | - | - | - | - | - | - | - | - | - |
| 013 UniRef90\_A0A0N1GDE2\_28\_316 | - | - | - | - | - | - | - | - | - | - | L | R | L | P | N | - | - | - | - | - | - | - | - | - | - | - | - | - | - | - | - | G | F | - | - | - | T | D | V | F | T | S | R | L | V | E | L | N | G | - |
| 014 UniRef90\_A0A1I6BK09\_10\_296 | - | - | - | - | - | - | - | - | - | - | - | - | - | - | - | - | - | - | - | - | - | - | - | - | - | - | - | - | - | - | - | - | - | - | - | - | - | - | - | - | T | H | H | R | A | Q | V | G | E | - |
| 015 UniRef90\_UPI00158A2D6D\_3\_306 | - | - | - | - | - | N | E | H | S | I | S | G | M | P | A | A | G | - | - | - | - | - | - | - | - | - | - | - | L | P | A | G | F | - | - | - | D | R | R | F | S | R | R | Y | V | H | V | N | D | - |
| 016 UniRef90\_UPI00160CFF45\_55\_340 | - | - | - | - | - | - | - | - | - | - | - | - | - | - | - | - | - | - | - | - | - | - | - | - | - | - | - | - | - | - | - | Q | F | - | - | - | T | S | T | F | R | H | E | F | A | D | V | N | G | - |
| 017 UniRef90\_A0A2N3KZL1\_30\_325 | - | - | - | K | P | Q | S | Q | P | E | S | E | F | P | - | - | - | - | - | - | - | - | - | - | - | - | - | - | - | - | - | - | - | - | - | V | P | A | G | F | E | S | S | Y | A | E | I | N | G | - |
| 018 UniRef90\_A0A2M9M9Q1\_18\_305 | - | - | - | - | - | - | - | - | - | V | P | W | L | P | D | - | - | - | - | - | - | - | - | - | - | - | - | - | - | - | - | G | F | - | - | - | T | D | V | F | E | S | S | L | V | Q | V | G | R | - |
| 019 UniRef90\_UPI000361D127\_26\_313 | - | - | - | - | - | - | - | - | - | V | P | D | L | P | A | - | - | - | - | - | - | - | - | - | - | - | - | - | - | - | - | G | F | - | - | - | A | D | V | F | S | S | S | Y | V | D | T | G | S | - |
| 020 UniRef90\_UPI001473614E\_45\_322 | - | - | - | - | - | - | - | - | A | T | P | K | E | P | A | E | G | - | - | - | - | - | - | - | - | - | - | - | - | P | A | R | F | - | - | - | A | P | G | F | R | H | G | K | V | A | V | E | G | G |
| 021 UniRef90\_E6WJ64\_19\_301 | - | - | - | - | - | - | - | - | - | - | - | - | - | - | - | - | - | - | - | - | - | - | - | - | - | - | - | - | - | - | - | G | - | - | - | - | - | - | - | F | A | H | H | Y | A | T | V | D | G | - |
| 022 UniRef90\_A0A6L3SWG4\_20\_294 | - | - | - | - | - | - | - | - | - | - | - | - | - | - | - | - | - | - | - | - | - | - | - | - | - | - | - | - | - | - | - | - | - | - | - | - | - | - | - | - | - | - | - | - | - | - | - | - | G | - |
| 023 UniRef90\_A0A109IGY4\_11\_301 | - | - | - | - | - | - | - | - | S | S | L | R | L | P | A | - | - | - | - | - | - | - | - | - | - | - | - | - | - | - | - | G | F | - | - | - | T | D | V | F | T | S | Q | L | V | E | L | D | G | - |
| 024 UniRef90\_A0A0Q4UQ58\_27\_305 | - | - | - | - | - | - | - | - | - | - | - | A | V | P | D | S | G | - | - | - | - | - | - | - | - | - | - | L | D | A | G | V | L | - | - | - | P | P | G | A | A | S | R | F | A | Q | L | N | G | - |
| 025 UniRef90\_A0A239MTD5\_1\_271 | - | - | - | - | - | - | - | - | - | - | - | - | - | - | - | - | - | - | - | - | - | - | - | - | - | - | - | - | - | - | - | - | - | - | - | - | - | - | - | - | - | - | - | - | - | - | - | - | - | - |
| 026 UniRef90\_UPI00049086FA\_57\_348 | - | - | - | - | - | - | - | - | - | - | - | - | - | - | - | - | - | - | - | - | - | - | - | - | - | - | - | - | - | - | - | E | F | - | - | - | N | L | D | F | R | H | C | F | S | T | V | D | G | - |
| 027 UniRef90\_A0A517LKB1\_23\_304 | - | - | - | - | - | - | - | - | - | - | - | - | - | - | - | - | - | - | - | - | - | - | - | - | - | - | - | - | - | - | - | - | - | - | - | - | - | - | - | - | - | - | - | - | - | - | P | E | E | - |
| 028 UniRef90\_UPI0004CCFD44\_55\_340 | - | - | - | - | - | - | - | - | - | - | - | - | - | - | - | - | - | - | - | - | - | - | - | - | - | - | - | - | - | - | - | E | F | - | - | - | E | G | T | F | R | H | E | F | A | D | V | D | G | - |
| 029 UniRef90\_A0A285BKA7\_8\_299 | - | - | - | - | - | - | - | - | - | V | P | G | L | S | A | - | - | - | - | - | - | - | - | - | - | - | - | - | - | - | - | A | F | - | - | - | T | D | T | F | T | S | R | T | V | T | T | D | E | G |
| 030 UniRef90\_A0A4R7C9R7\_33\_310 | - | - | - | - | - | S | R | L | P | P | P | A | D | P | P | - | - | - | - | - | - | - | - | - | - | - | - | - | - | - | - | - | L | - | - | - | P | T | G | A | E | S | R | F | A | T | V | N | G | - |
| 031 UniRef90\_A0A4D4LDP7\_7\_304 | - | - | - | - | - | - | - | - | - | - | - | P | R | P | G | - | - | - | - | I | G | A | E | I | S | D | A | E | Q | L | E | G | - | - | - | - | - | - | - | F | T | H | L | S | A | D | A | D | G | - |
| 032 UniRef90\_J4PG95\_17\_290 | - | - | - | - | - | - | - | - | - | - | - | - | - | - | - | - | - | - | - | - | - | - | - | - | - | - | - | - | - | - | - | - | - | - | - | - | - | - | - | - | - | H | Q | Y | P | L | I | D | G | - |
| 033 UniRef90\_A0A1Q8KTW7\_17\_294 | - | - | - | - | - | - | - | - | - | - | - | - | - | - | - | - | - | - | - | - | - | - | - | - | - | - | - | - | - | - | - | - | - | - | - | - | - | - | - | - | - | - | - | - | A | T | V | N | G | - |
| 034 UniRef90\_UPI00076E3DF2\_55\_340 | - | - | - | - | - | - | - | - | - | - | - | - | - | - | - | - | - | - | - | - | - | - | - | - | - | - | - | - | - | - | - | E | F | - | - | - | E | S | T | F | R | H | E | F | A | D | V | D | G | - |
| 035 UniRef90\_A0A542JC81\_20\_314 | - | - | - | - | - | - | - | - | Q | A | P | N | L | P | E | - | - | - | - | - | - | - | - | - | - | - | - | - | - | - | - | G | F | - | - | - | T | D | T | F | T | S | R | Y | I | D | A | N | G | - |
| 036 UniRef90\_UPI00156E1DFF\_8\_289 | - | - | - | - | - | - | - | - | - | - | - | - | - | - | - | - | - | - | - | - | - | - | - | - | - | - | - | - | - | - | - | - | - | - | - | - | P | D | G | F | E | S | I | T | V | S | V | N | G | - |
| 037 UniRef90\_UPI00161A7EC4\_21\_297 | - | - | - | - | - | - | - | - | - | - | P | R | E | P | S | T | G | - | - | - | - | - | - | - | - | - | - | - | - | Q | A | R | F | - | - | - | S | P | I | F | T | H | G | R | V | A | V | D | G | G |
| 038 UniRef90\_UPI0012B05B7D\_16\_287 | - | - | - | - | - | - | - | - | - | - | - | - | - | - | - | - | - | - | - | - | - | - | - | - | - | - | - | - | - | - | - | - | - | - | - | - | - | - | - | - | - | - | - | - | - | - | F | D | N | - |
| 039 UniRef90\_A0A2I8F4B9\_53\_324 | - | - | - | - | - | - | - | - | - | - | - | - | - | - | - | - | - | - | - | - | - | - | - | - | - | - | - | - | - | - | - | - | - | - | - | - | - | - | - | - | - | - | - | - | - | - | V | N | G | - |
| 040 UniRef90\_A0A4D7B7N1\_7\_276 | - | - | - | - | - | - | - | - | - | - | - | - | - | - | - | - | - | - | - | - | - | - | - | - | - | - | - | - | - | - | - | - | - | - | - | - | - | - | - | - | - | - | - | - | - | - | - | - | - | - |
| 041 UniRef90\_UPI000E275A28\_18\_308 | - | - | - | - | - | - | - | - | - | V | P | E | L | P | N | - | - | - | - | - | - | - | - | - | - | - | - | - | - | - | - | G | F | - | - | - | L | D | V | F | E | S | Y | R | I | P | V | G | D | - |
| 042 UniRef90\_S3CY91\_25\_307 | - | - | - | - | - | - | - | - | - | - | - | - | - | - | - | - | - | - | - | - | - | - | - | - | - | - | - | - | - | - | - | - | - | - | - | - | - | - | - | - | - | - | - | - | - | - | - | Q | E | - |
| 043 UniRef90\_A0A316VRQ3\_1\_263 | - | - | - | - | - | - | - | - | - | - | - | - | - | - | - | - | - | - | - | - | - | - | - | - | - | - | - | - | - | - | - | - | - | - | - | - | - | - | - | - | - | - | - | - | - | - | - | - | - | - |
| 044 UniRef90\_A0A4R6HBQ4\_11\_285 | - | - | - | - | - | - | - | - | - | - | - | - | - | - | - | - | - | - | - | - | - | - | - | - | - | - | - | - | - | - | - | - | - | - | - | - | - | - | - | - | - | - | - | - | - | - | - | A | D | - |
| 045 UniRef90\_UPI0015F81933\_16\_310 | - | - | - | - | - | - | - | - | - | - | - | - | - | P | I | - | - | - | - | R | D | L | P | L | R | H | - | - | - | L | A | G | - | - | - | - | - | - | - | F | T | H | R | W | V | D | A | D | G | - |
| 046 UniRef90\_UPI0009E804D9\_8\_293 | - | - | - | - | - | - | - | - | - | - | - | - | - | P | G | T | - | - | - | - | - | - | - | - | - | - | - | - | D | Y | H | W | L | - | - | - | P | A | G | F | T | V | H | E | V | D | T | N | G | - |
| 047 UniRef90\_A0A401ZLI5\_4\_287 | - | - | - | - | - | - | - | - | - | - | - | - | - | P | L | - | - | - | - | - | - | - | - | - | - | - | - | - | - | P | A | P | L | - | - | - | E | P | L | F | T | H | Q | T | A | Q | V | N | G | - |
| 048 UniRef90\_A0A6A6C1N5\_26\_297 | - | - | - | - | - | - | - | - | - | - | - | - | - | - | - | - | - | - | - | - | - | - | - | - | - | - | - | - | - | - | - | - | - | - | - | - | - | - | - | - | - | - | - | T | N | V | P | S | A | - |
| 049 UniRef90\_A0A329J6I4\_30\_314 | - | - | - | - | - | - | - | - | - | - | - | - | - | - | - | - | - | - | - | - | - | - | - | - | - | - | - | - | - | - | - | - | - | - | - | - | P | K | G | F | T | S | E | F | R | T | V | D | G | - |
| 050 UniRef90\_A0A1B2HH26\_4\_280 | - | - | - | - | - | - | - | - | - | - | - | - | - | P | A | P | A | - | - | - | - | - | - | - | - | - | - | - | - | - | - | - | - | - | - | - | P | E | G | F | E | H | A | Y | A | E | V | N | G | - |
| 051 UniRef90\_A0A385B2U0\_45\_333 | - | - | - | - | - | - | - | - | - | - | - | R | L | P | Y | - | - | - | - | - | - | - | - | - | - | - | - | - | - | - | - | - | - | - | R | L | P | D | G | F | R | S | E | Y | A | Q | V | N | D | - |
| 052 UniRef90\_A0A2E5PJQ7\_5\_284 | - | - | - | - | - | - | - | - | - | - | - | - | - | - | - | - | - | - | - | - | - | - | - | - | - | - | - | - | - | - | - | - | - | - | - | - | - | - | - | - | A | H | H | Y | A | D | L | G | D | - |
| 053 UniRef90\_A0A5C8T429\_14\_295 | - | - | - | - | - | - | - | - | - | - | - | - | - | - | - | - | - | - | - | - | - | - | - | - | - | - | - | - | - | - | F | G | R | - | - | - | - | - | I | F | Q | H | R | V | Q | G | A | V | G | - |
| 054 UniRef90\_A0A261TYY2\_43\_315 | - | - | - | - | - | - | - | - | - | - | - | - | - | - | - | - | - | - | - | - | - | - | - | - | - | - | - | - | - | - | - | - | - | - | - | - | - | - | - | - | - | - | R | M | Q | G | E | P | G | - |
| 055 UniRef90\_A0A6N7ZB66\_18\_297 | - | - | - | - | - | - | - | - | - | - | - | - | - | - | - | - | - | - | - | - | - | - | - | - | - | - | - | - | - | - | - | - | - | - | - | - | - | - | G | F | T | V | R | E | V | Q | T | N | G | - |
| 056 UniRef90\_A0A2T6L0M5\_16\_299 | - | - | - | - | - | - | - | - | - | - | - | - | - | - | - | - | - | - | - | - | - | - | - | - | - | - | - | - | - | - | - | - | - | - | - | V | P | A | G | Y | E | S | H | Y | A | H | V | N | G | - |
| 057 UniRef90\_G0FSK7\_17\_310 | - | - | - | - | - | - | - | - | G | T | P | G | L | P | A | - | - | - | - | - | - | - | - | - | - | - | - | - | - | - | - | G | F | - | - | - | D | E | V | F | T | S | R | W | I | A | V | G | E | - |
| 058 UniRef90\_A0A1Q8IR73\_6\_294 | - | - | - | - | - | - | - | - | - | L | P | A | A | P | T | - | - | - | - | - | - | - | - | - | - | - | - | - | - | - | - | G | - | - | - | - | - | - | - | F | E | H | K | F | A | T | A | E | G | - |
| 059 UniRef90\_A0A1I2MT44\_20\_306 | - | - | - | - | - | - | - | - | - | V | P | R | L | P | A | - | - | - | - | - | - | - | - | - | - | - | - | - | - | - | - | G | F | - | - | - | T | D | T | F | T | S | R | Y | V | D | V | D | G | - |
| 060 UniRef90\_UPI0010F95BB2\_18\_294 | - | - | - | - | - | - | - | - | - | - | - | - | - | - | - | - | - | - | - | - | - | - | - | - | - | - | - | - | - | - | - | - | - | - | - | - | - | - | - | - | - | - | - | - | - | - | - | N | G | - |
| 061 UniRef90\_A0A4P8X818\_19\_291 | - | - | - | - | - | - | - | - | - | - | - | - | - | - | - | - | - | - | - | - | - | - | - | - | - | - | - | - | - | - | - | - | - | - | - | - | - | - | - | - | - | - | - | - | - | T | V | N | G | - |
| 062 UniRef90\_A0A0M3UDU6\_18\_309 | - | - | - | - | - | - | - | - | - | - | - | - | - | - | - | - | - | - | - | - | - | - | - | - | - | - | - | - | - | R | T | D | F | - | - | - | W | K | C | F | H | H | A | S | A | R | V | G | D | - |
| 063 UniRef90\_A0A2P2CCU5\_19\_306 | - | - | - | - | - | - | - | - | R | A | P | G | L | P | A | - | - | - | - | - | - | - | - | - | - | - | - | - | - | - | - | G | F | - | - | - | S | T | T | F | S | S | Q | T | V | T | A | N | G | - |
| 064 UniRef90\_UPI0009781B11\_10\_287 | - | - | - | - | - | - | - | - | - | - | - | - | - | - | - | - | - | - | - | - | - | - | - | - | - | - | - | - | - | - | - | - | - | - | - | - | - | - | - | - | - | - | R | V | I | D | A | Q | G | - |
| 065 UniRef90\_UPI000561A6EF\_30\_315 | - | - | - | - | - | - | A | S | G | A | P | E | L | P | A | - | - | - | - | - | - | - | - | - | - | - | - | - | - | - | - | G | F | - | - | - | G | E | T | F | T | S | R | Y | V | D | T | G | A | - |
| 066 UniRef90\_A0A1Z4J856\_27\_318 | - | - | - | - | - | - | - | - | - | - | - | - | - | P | F | - | - | - | - | - | - | - | - | - | - | - | - | - | N | K | A | A | F | - | - | - | I | K | T | F | A | H | H | T | A | K | V | N | D | - |
| 067 UniRef90\_A0A5C5SVV1\_11\_281 | - | - | - | - | - | - | - | - | - | - | - | - | - | - | - | - | - | - | - | - | - | - | - | - | - | - | - | - | - | - | - | - | - | - | - | - | - | - | - | - | - | - | - | - | V | R | V | G | E | - |
| **068 Input\_protein\_seq** | M | Q | N | E | R | S | E | Q | S | M | P | G | M | P | A | P | G | - | - | - | - | - | - | - | - | - | - | - | L | P | A | G | F | - | - | - | E | R | R | F | S | R | R | Y | A | Q | L | D | D | - |
| 069 UniRef90\_A0A1Q7W147\_16\_308 | - | - | - | - | - | - | - | - | - | - | - | - | - | P | V | - | - | - | - | R | D | L | P | L | H | D | - | - | - | L | A | G | - | - | - | - | - | - | - | F | T | H | R | W | V | D | A | D | G | - |
| 070 UniRef90\_A0A0N0TCD7\_1\_288 | - | - | - | - | - | - | - | - | - | - | - | - | M | P | A | - | - | - | - | - | - | - | - | - | - | - | - | - | - | - | - | G | F | - | - | - | T | D | T | F | T | S | H | T | V | E | T | G | D | - |
| 071 UniRef90\_UPI0004DF415F\_15\_308 | - | - | - | - | - | - | - | - | - | - | - | - | N | P | I | - | - | - | - | S | G | L | P | L | H | D | - | - | - | L | A | G | - | - | - | - | - | - | - | F | T | H | R | W | V | D | A | A | G | - |
| 072 UniRef90\_G7LVZ3\_7\_288 | - | - | - | - | - | - | - | - | - | - | - | - | - | - | - | - | - | - | - | - | - | - | - | - | - | - | - | - | - | - | - | - | - | - | - | - | - | - | - | F | T | H | H | T | T | V | V | N | G | - |

  
  

|  |  |  |  |  |  |  |  |  |  |  |  |  |  |  |  |  |  |  |  |  |  |  |  |  |  |  |  |  |  |  |  |  |  |  |  |  |  |  |  |  |  |  |  |  |  |  |  |  |  |  |
| --- | --- | --- | --- | --- | --- | --- | --- | --- | --- | --- | --- | --- | --- | --- | --- | --- | --- | --- | --- | --- | --- | --- | --- | --- | --- | --- | --- | --- | --- | --- | --- | --- | --- | --- | --- | --- | --- | --- | --- | --- | --- | --- | --- | --- | --- | --- | --- | --- | --- | --- |
| 001 UniRef90\_L9WLS3\_16\_284 | V | S | I | H | Y | V | T | A | - | - | - | - | - | - | - | - | - | - | - | G | S | - | - | - | - | G | P | P | - | L | V | L | L | H | G | W | P | Q | T | W | Y | E | W | R | D | V | I | P | S | F |
| 002 UniRef90\_A0A0M4FVH2\_24\_312 | V | R | L | H | Y | V | V | G | - | - | - | - | - | - | - | - | - | - | - | G | K | D | - | - | D | G | D | V | - | V | V | L | L | P | G | F | P | E | S | W | F | T | W | H | K | V | M | P | L | L |
| 003 UniRef90\_UPI0016621A44\_4\_265 | - | - | - | - | - | - | - | - | - | - | - | - | - | - | - | - | - | - | - | G | D | - | - | - | - | G | P | V | - | V | L | L | L | H | G | W | P | V | T | S | Y | H | W | R | L | T | V | P | A | L |
| 004 UniRef90\_A0A447J1A9\_28\_317 | Q | R | I | H | Y | V | T | A | - | - | - | - | - | - | - | - | - | - | - | G | K | - | - | - | - | G | E | P | - | V | L | L | I | P | G | W | P | Q | T | W | Y | T | W | R | Y | V | M | T | G | L |
| 005 UniRef90\_A0A1Y6D2S0\_29\_302 | I | Q | M | H | Y | V | K | M | - | - | - | - | - | - | - | - | - | - | - | G | K | - | - | - | - | G | P | L | - | L | L | L | L | H | G | W | P | Q | T | W | Y | E | W | H | R | I | M | P | M | L |
| 006 UniRef90\_A0A0G2FGE6\_47\_303 | - | - | - | - | - | - | - | - | - | - | - | - | - | - | - | - | - | - | - | - | - | - | - | - | - | - | - | T | - | I | L | L | I | H | G | Y | P | Q | C | S | Y | Q | F | R | H | V | I | T | P | I |
| 007 UniRef90\_F8JLL5\_12\_309 | V | L | L | H | A | V | E | G | - | - | - | - | - | - | - | - | - | - | - | G | R | P | - | - | G | G | P | A | - | V | V | L | L | A | G | F | P | Q | T | W | W | A | W | R | K | V | M | P | D | L |
| 008 UniRef90\_A0A4R2Z7A8\_3\_305 | V | R | I | H | Y | V | C | G | - | - | - | - | - | - | - | - | - | - | - | G | N | P | - | - | D | G | E | V | - | L | L | L | L | A | G | F | P | Q | S | W | F | A | W | R | H | V | M | Q | D | L |
| 009 UniRef90\_UPI0016149F5E\_5\_284 | Q | R | L | H | C | A | I | T | - | - | - | - | - | - | - | - | - | - | - | G | S | - | - | - | - | G | K | P | - | V | L | L | I | P | G | W | P | Q | T | W | Y | A | W | R | H | V | M | A | A | L |
| 010 UniRef90\_A0A327RPK7\_10\_284 | I | N | L | H | Y | V | I | A | - | - | - | - | - | - | - | - | - | - | - | G | S | - | - | - | - | G | D | P | - | I | V | L | L | H | G | W | P | Q | T | W | Y | E | W | K | D | I | I | P | T | L |
| 011 UniRef90\_UPI00131EC7B5\_30\_311 | I | R | M | H | Y | V | T | G | - | - | - | - | - | - | - | - | - | - | - | G | N | P | - | - | D | G | E | T | - | V | V | L | L | A | G | F | P | Q | S | W | Y | A | W | R | S | V | M | Q | Q | L |
| 012 UniRef90\_A0A1N6RLL9\_1\_202 | - | - | - | - | - | - | - | - | - | - | - | - | - | - | - | - | - | - | - | - | - | - | - | - | - | - | - | - | - | - | - | - | - | - | - | - | - | - | - | - | - | - | - | - | - | - | - | - | - | - |
| 013 UniRef90\_A0A0N1GDE2\_28\_316 | L | R | L | H | A | V | T | G | - | - | - | - | - | - | - | - | - | - | - | G | D | - | - | - | - | G | P | P | - | L | L | L | V | G | G | W | P | Q | T | W | Y | A | W | R | E | V | M | P | A | L |
| 014 UniRef90\_A0A1I6BK09\_10\_296 | V | S | L | H | Y | L | Q | A | - | - | - | - | - | - | - | - | - | - | - | G | E | P | R | P | G | R | A | P | - | L | V | L | L | H | G | F | P | Q | H | S | H | M | W | R | R | L | M | P | A | L |
| 015 UniRef90\_UPI00158A2D6D\_3\_306 | V | R | L | H | Y | V | T | G | - | - | - | - | - | - | - | - | - | G | P | D | D | - | - | - | - | G | E | L | - | V | I | L | L | H | G | W | P | Q | T | W | Y | T | W | R | H | V | M | P | A | L |
| 016 UniRef90\_UPI00160CFF45\_55\_340 | V | R | M | H | Y | V | A | G | - | - | - | - | - | - | - | - | - | - | - | G | S | - | - | - | - | G | P | P | - | V | V | L | L | H | G | W | P | Q | T | W | Y | G | W | W | P | I | M | P | E | L |
| 017 UniRef90\_A0A2N3KZL1\_30\_325 | V | R | L | H | Y | V | K | G | - | - | - | - | - | - | - | - | - | - | - | G | S | - | - | - | - | G | P | L | - | A | F | L | V | H | G | F | G | Q | S | W | Y | E | W | H | Q | L | M | P | L | L |
| 018 UniRef90\_A0A2M9M9Q1\_18\_305 | V | G | L | H | T | V | Q | G | - | - | - | - | - | - | - | - | - | - | - | G | T | - | - | - | - | G | A | P | - | L | L | L | V | G | G | W | P | Q | N | W | Y | V | W | R | F | V | M | P | R | L |
| 019 UniRef90\_UPI000361D127\_26\_313 | V | R | L | H | T | V | V | G | - | - | - | - | - | - | - | - | - | - | - | G | R | - | - | - | - | G | P | A | - | L | L | L | I | G | G | W | P | Q | F | W | Y | Q | W | R | H | V | M | L | P | L |
| 020 UniRef90\_UPI001473614E\_45\_322 | A | - | V | H | Y | V | I | G | - | - | - | - | - | - | - | - | - | - | - | G | S | - | - | - | - | G | P | A | - | V | V | L | L | H | G | W | P | Q | T | W | L | M | W | R | K | L | M | P | D | L |
| 021 UniRef90\_E6WJ64\_19\_301 | V | R | L | H | Y | V | S | G | - | - | - | - | - | - | - | - | - | - | - | G | H | P | - | - | Q | G | D | V | - | L | L | L | L | A | G | F | P | Q | S | W | Y | A | W | H | Q | V | M | A | Q | L |
| 022 UniRef90\_A0A6L3SWG4\_20\_294 | V | R | L | H | Y | G | V | A | - | - | - | - | - | - | - | - | - | G | A | G | D | - | - | - | - | - | R | T | - | V | M | L | V | H | G | Y | P | Q | T | A | Y | A | W | R | R | V | V | P | L | L |
| 023 UniRef90\_A0A109IGY4\_11\_301 | L | H | L | H | A | V | T | G | - | - | - | - | - | - | - | - | - | - | - | G | D | - | - | - | - | G | P | P | - | L | L | L | V | G | G | W | P | Q | T | W | Y | A | W | R | E | V | M | P | A | L |
| 024 UniRef90\_A0A0Q4UQ58\_27\_305 | I | R | M | H | Y | V | V | A | - | - | - | - | - | - | - | - | - | - | - | G | S | - | - | - | - | G | P | P | - | L | V | L | L | H | G | W | P | Q | T | W | A | A | W | K | A | T | M | A | A | L |
| 025 UniRef90\_A0A239MTD5\_1\_271 | - | - | M | H | Y | M | E | G | - | - | - | - | - | - | - | - | - | - | - | G | Q | - | - | - | - | G | S | P | - | V | V | M | I | H | G | F | P | Q | T | W | A | E | W | R | Q | Q | M | G | P | L |
| 026 UniRef90\_UPI00049086FA\_57\_348 | V | Q | M | H | Y | V | I | G | - | - | - | - | - | - | - | - | - | - | - | G | T | - | - | - | - | G | P | S | T | L | V | L | L | H | G | W | P | E | S | W | Y | E | Y | R | A | V | M | P | R | L |
| 027 UniRef90\_A0A517LKB1\_23\_304 | V | R | I | H | Y | R | I | A | Q | P | P | - | - | - | - | - | - | - | S | Q | K | - | - | - | - | K | G | V | - | I | L | L | I | H | G | Y | P | E | S | S | Y | Q | F | R | H | V | I | P | L | L |
| 028 UniRef90\_UPI0004CCFD44\_55\_340 | V | R | M | H | Y | V | T | G | - | - | - | - | - | - | - | - | - | - | - | G | S | - | - | - | - | G | P | P | - | V | V | L | V | H | G | W | P | Q | T | W | F | G | W | W | P | I | M | P | A | L |
| 029 UniRef90\_A0A285BKA7\_8\_299 | M | T | L | H | A | V | V | G | - | - | - | - | - | - | - | - | - | - | - | G | D | - | - | - | - | G | P | P | - | L | L | L | L | P | G | W | P | Q | F | W | Y | S | W | R | L | V | M | P | A | L |
| 030 UniRef90\_A0A4R7C9R7\_33\_310 | I | R | M | H | Y | V | V | A | - | - | - | - | - | - | - | - | - | - | - | G | Q | - | - | - | - | G | P | T | - | V | I | L | L | H | G | W | P | Q | T | W | F | A | W | H | G | Q | I | E | R | L |
| 031 UniRef90\_A0A4D4LDP7\_7\_304 | I | R | I | H | A | V | V | G | - | - | - | - | - | - | - | - | - | - | - | G | S | T | - | - | G | S | D | P | - | V | V | L | L | A | G | F | P | Q | T | W | Y | A | W | H | K | V | M | P | A | L |
| 032 UniRef90\_J4PG95\_17\_290 | H | R | I | H | C | A | I | A | - | - | - | - | - | - | - | - | - | - | - | G | D | - | - | - | - | G | P | P | - | V | L | L | I | P | G | W | P | Q | T | W | F | T | W | R | H | I | M | K | S | L |
| 033 UniRef90\_A0A1Q8KTW7\_17\_294 | V | R | L | H | Y | V | T | A | - | - | - | - | - | - | - | - | - | - | - | G | S | - | - | - | - | G | P | P | - | L | V | L | L | H | G | V | P | K | S | W | Y | Y | W | H | R | V | I | P | L | L |
| 034 UniRef90\_UPI00076E3DF2\_55\_340 | V | R | M | H | Y | V | T | G | - | - | - | - | - | - | - | - | - | - | - | G | S | - | - | - | - | G | T | P | - | V | V | L | L | H | G | W | P | Q | T | W | F | G | W | W | S | I | M | P | E | L |
| 035 UniRef90\_A0A542JC81\_20\_314 | V | R | L | H | A | V | I | G | - | - | - | - | - | - | - | - | - | - | - | G | Q | - | - | - | - | G | P | A | - | L | L | L | V | H | G | W | P | E | T | W | Y | A | W | R | L | V | M | P | E | L |
| 036 UniRef90\_UPI00156E1DFF\_8\_289 | T | R | L | H | A | V | V | G | - | - | - | - | - | - | - | - | - | - | - | G | S | - | - | - | - | G | E | P | - | V | L | L | L | H | G | W | P | Q | T | W | R | A | W | R | Y | L | M | P | T | L |
| 037 UniRef90\_UPI00161A7EC4\_21\_297 | S | T | L | H | Y | V | R | G | - | - | - | - | - | - | - | - | - | - | - | G | S | - | - | - | - | G | P | A | - | I | V | L | L | H | G | W | P | Q | T | W | W | I | W | R | N | V | M | P | E | L |
| 038 UniRef90\_UPI0012B05B7D\_16\_287 | L | R | L | H | Y | L | E | S | - | - | - | - | - | - | - | - | - | - | - | G | T | - | - | - | - | G | P | V | - | I | L | L | V | A | G | F | P | Q | S | C | Y | A | W | R | K | V | I | P | L | L |
| 039 UniRef90\_A0A2I8F4B9\_53\_324 | V | R | Y | H | Y | L | L | A | Q | - | - | - | - | - | - | - | - | G | D | K | K | - | - | - | - | G | T | P | - | V | V | L | L | H | G | W | G | S | T | S | Y | M | W | R | Y | V | M | P | Q | L |
| 040 UniRef90\_A0A4D7B7N1\_7\_276 | L | R | F | H | T | V | E | G | - | - | - | - | - | - | - | - | - | - | - | G | S | - | - | - | - | G | A | P | - | V | V | L | L | A | G | F | P | Q | S | W | Y | A | W | R | R | V | M | P | L | I |
| 041 UniRef90\_UPI000E275A28\_18\_308 | V | S | L | H | A | V | I | G | - | - | - | - | - | - | - | - | - | - | - | G | E | - | - | - | - | G | P | P | - | L | L | L | L | T | G | W | P | Q | N | W | Y | A | W | R | D | M | M | L | P | L |
| 042 UniRef90\_S3CY91\_25\_307 | V | R | I | H | Y | - | I | D | C | P | P | Y | P | D | K | P | D | R | T | E | E | - | - | - | - | T | N | T | - | I | L | L | I | H | G | F | P | N | T | S | Y | Q | W | R | H | V | I | T | P | L |
| 043 UniRef90\_A0A316VRQ3\_1\_263 | - | - | - | - | - | - | - | - | - | - | - | - | - | - | - | - | - | - | - | - | - | - | - | - | - | - | - | - | - | I | L | L | I | H | G | Y | P | Q | T | V | Y | A | M | R | Y | L | L | P | L | F |
| 044 UniRef90\_A0A4R6HBQ4\_11\_285 | L | R | L | H | A | V | F | A | - | - | - | - | - | - | - | - | - | - | - | G | P | E | - | - | H | G | E | T | - | V | L | L | L | H | G | W | P | Q | T | S | H | A | W | R | K | V | I | P | L | L |
| 045 UniRef90\_UPI0015F81933\_16\_310 | V | R | L | H | A | V | E | G | - | - | - | - | - | - | - | - | - | - | - | G | R | P | - | - | S | G | P | A | - | V | V | L | L | A | G | F | P | Q | T | W | W | A | W | R | K | V | M | P | G | L |
| 046 UniRef90\_UPI0009E804D9\_8\_293 | T | T | I | S | A | A | V | G | - | - | - | - | - | - | - | - | - | - | - | G | R | - | - | - | - | G | P | T | - | M | I | L | L | H | G | W | P | Q | T | G | R | A | W | R | H | V | M | G | P | L |
| 047 UniRef90\_A0A401ZLI5\_4\_287 | V | R | L | H | Y | V | I | G | - | - | - | - | - | - | - | - | - | - | - | G | Q | - | - | - | - | G | S | P | - | V | L | L | L | H | G | W | P | Q | T | W | Y | A | W | R | K | I | M | P | A | L |
| 048 UniRef90\_A0A6A6C1N5\_26\_297 | A | R | - | - | - | - | - | - | - | - | - | - | - | - | - | - | - | - | - | - | - | - | - | - | - | - | - | T | - | L | L | L | L | H | G | F | P | Q | T | S | H | Q | F | R | H | V | I | P | L | L |
| 049 UniRef90\_A0A329J6I4\_30\_314 | V | K | L | H | Y | V | K | G | - | - | - | - | - | - | - | - | - | - | - | G | S | - | - | - | - | G | P | L | - | V | F | L | A | H | G | F | G | Q | A | W | Y | E | W | H | N | L | M | P | E | L |
| 050 UniRef90\_A0A1B2HH26\_4\_280 | V | Q | M | H | Y | V | T | G | - | - | - | - | - | - | - | - | - | - | - | G | E | - | - | - | - | G | P | L | - | V | V | L | V | H | G | W | P | F | S | W | I | E | F | R | E | L | L | P | L | M |
| 051 UniRef90\_A0A385B2U0\_45\_333 | F | R | M | H | Y | L | R | G | - | - | - | - | - | - | - | - | - | - | - | G | S | - | - | - | - | G | S | P | - | V | V | L | L | H | G | F | P | Q | T | S | A | E | W | E | P | Q | L | E | A | L |
| 052 UniRef90\_A0A2E5PJQ7\_5\_284 | V | K | I | H | Y | I | T | A | - | - | - | - | - | - | - | - | - | - | - | G | R | - | - | - | - | G | P | P | - | V | I | L | L | H | G | W | P | E | T | W | Y | E | W | R | H | I | I | P | K | L |
| 053 UniRef90\_A0A5C8T429\_14\_295 | N | R | L | H | Y | V | T | G | - | - | - | - | - | - | - | - | - | - | - | G | Q | - | - | - | - | G | A | P | - | V | L | L | V | P | G | W | P | Q | T | W | Y | A | W | R | H | V | M | P | L | L |
| 054 UniRef90\_A0A261TYY2\_43\_315 | Q | R | L | H | Y | V | I | G | - | - | - | - | - | - | - | - | - | - | - | G | Q | - | - | - | - | G | E | P | - | V | L | L | V | P | G | W | P | Q | T | W | Y | A | W | R | K | T | M | R | A | L |
| 055 UniRef90\_A0A6N7ZB66\_18\_297 | V | R | L | S | A | A | V | G | - | - | - | - | - | - | - | - | - | - | - | G | S | - | - | - | - | G | P | V | - | L | V | L | L | H | G | W | P | Q | T | G | R | A | W | A | R | V | M | P | T | L |
| 056 UniRef90\_A0A2T6L0M5\_16\_299 | F | R | M | H | Y | L | R | G | - | - | - | - | - | - | - | - | - | - | - | G | S | - | - | - | - | G | S | P | - | V | L | L | L | H | G | F | P | Q | T | W | A | E | W | H | H | Q | L | G | P | L |
| 057 UniRef90\_G0FSK7\_17\_310 | L | R | L | H | A | V | V | G | - | - | - | - | - | - | - | - | - | - | - | G | D | - | - | - | - | G | P | P | - | L | L | L | I | P | G | W | P | Q | T | W | Y | A | W | R | L | L | M | P | E | L |
| 058 UniRef90\_A0A1Q8IR73\_6\_294 | L | R | Y | H | Y | V | E | G | - | - | - | - | - | - | - | - | - | - | - | G | A | N | - | - | A | E | R | T | - | I | V | L | I | A | G | F | P | E | S | W | Y | A | W | R | K | V | M | P | L | L |
| 059 UniRef90\_A0A1I2MT44\_20\_306 | L | R | L | H | A | V | T | G | - | - | - | - | - | - | - | - | - | - | - | G | E | - | - | - | - | G | P | P | - | L | L | L | L | A | G | W | P | Q | T | W | Y | A | W | R | L | V | M | P | A | L |
| 060 UniRef90\_UPI0010F95BB2\_18\_294 | L | R | L | H | L | T | E | S | - | - | - | - | - | - | - | - | - | - | - | G | S | Q | - | - | D | A | E | A | - | I | L | L | L | H | G | W | P | Q | T | S | Y | A | W | R | H | V | A | P | L | L |
| 061 UniRef90\_A0A4P8X818\_19\_291 | V | R | L | H | Y | Y | M | A | - | - | - | - | - | - | - | - | - | - | - | G | S | - | - | - | - | G | E | P | - | L | M | L | L | H | G | V | P | K | T | S | Y | Y | W | R | K | V | L | P | L | L |
| 062 UniRef90\_A0A0M3UDU6\_18\_309 | V | N | L | H | Y | V | E | G | - | - | - | - | - | - | - | - | - | - | - | G | E | - | - | - | - | G | P | P | - | V | L | L | I | P | G | W | P | Q | S | W | Y | A | W | R | Y | V | M | P | Q | L |
| 063 UniRef90\_A0A2P2CCU5\_19\_306 | I | R | Q | H | V | V | I | G | - | - | - | - | - | - | - | - | - | - | - | G | E | - | - | - | - | G | P | P | - | L | L | L | V | H | G | W | P | E | N | W | Y | A | W | R | H | V | M | P | D | L |
| 064 UniRef90\_UPI0009781B11\_10\_287 | A | K | L | H | F | V | E | K | - | - | - | - | - | - | - | - | - | - | - | G | N | - | - | - | - | G | P | L | - | L | V | L | V | S | G | W | P | Q | T | W | L | A | W | R | K | V | I | P | R | L |
| 065 UniRef90\_UPI000561A6EF\_30\_315 | L | R | L | H | A | V | V | G | - | - | - | - | - | - | - | - | - | - | - | G | R | - | - | - | - | G | P | A | - | L | L | L | I | C | G | W | P | Q | T | W | Y | A | W | R | L | L | M | P | A | L |
| 066 UniRef90\_A0A1Z4J856\_27\_318 | V | R | L | H | Y | V | I | G | - | - | - | - | - | - | - | - | - | - | - | G | K | - | - | - | - | G | E | P | - | L | V | L | L | H | G | F | P | T | T | W | Y | E | W | R | H | V | M | P | T | L |
| 067 UniRef90\_A0A5C5SVV1\_11\_281 | V | N | L | H | T | V | I | A | - | - | - | - | - | - | - | - | - | - | - | G | Q | - | - | - | - | G | A | P | - | L | V | L | L | H | G | F | P | Q | T | W | W | E | W | R | K | M | I | P | L | L |
| **068 Input\_protein\_seq** | V | R | L | H | Y | V | T | G | - | - | - | - | - | - | - | - | - | G | P | D | D | - | - | - | - | G | E | M | - | V | V | L | L | H | G | W | P | Q | T | W | Y | T | W | R | H | V | M | P | A | L |
| 069 UniRef90\_A0A1Q7W147\_16\_308 | I | R | L | H | A | V | E | G | - | - | - | - | - | - | - | - | - | - | - | G | R | P | - | - | S | G | P | A | - | L | V | L | L | A | G | F | P | Q | T | W | W | A | W | R | K | V | M | P | S | L |
| 070 UniRef90\_A0A0N0TCD7\_1\_288 | L | T | L | H | A | L | A | G | - | - | - | - | - | - | - | - | - | - | - | G | D | - | - | - | - | G | P | P | - | L | L | L | L | P | G | W | P | Q | F | W | Y | S | W | R | L | V | M | P | A | L |
| 071 UniRef90\_UPI0004DF415F\_15\_308 | I | R | L | H | A | V | E | G | - | - | - | - | - | - | - | - | - | - | - | G | R | P | - | - | G | G | P | A | - | V | V | L | L | A | G | F | P | Q | T | W | W | A | W | R | Q | T | M | P | G | L |
| 072 UniRef90\_G7LVZ3\_7\_288 | V | R | L | H | Y | V | M | A | - | - | - | - | - | - | - | - | - | - | - | G | S | - | - | - | - | G | D | P | - | L | V | L | L | H | G | W | P | Q | S | W | R | E | W | H | H | L | I | P | I | L |

  
  

|  |  |  |  |  |  |  |  |  |  |  |  |  |  |  |  |  |  |  |  |  |  |  |  |  |  |  |  |  |  |  |  |  |  |  |  |  |  |  |  |  |  |  |  |  |  |  |  |  |  |  |
| --- | --- | --- | --- | --- | --- | --- | --- | --- | --- | --- | --- | --- | --- | --- | --- | --- | --- | --- | --- | --- | --- | --- | --- | --- | --- | --- | --- | --- | --- | --- | --- | --- | --- | --- | --- | --- | --- | --- | --- | --- | --- | --- | --- | --- | --- | --- | --- | --- | --- | --- |
| 001 UniRef90\_L9WLS3\_16\_284 | A | A | E | - | H | T | V | I | A | P | D | L | R | G | L | G | D | S | E | T | P | A | S | G | - | - | - | - | Y | D | K | D | T | V | A | T | D | V | R | E | L | V | H | A | - | L | G | H | G | - |
| 002 UniRef90\_A0A0M4FVH2\_24\_312 | A | P | T | - | Y | K | L | I | V | L | D | L | P | G | Q | G | D | S | D | R | P | A | D | G | - | - | - | - | Y | D | T | K | T | L | A | T | T | V | H | K | F | L | Q | Q | - | L | G | - | - | - |
| 003 UniRef90\_UPI0016621A44\_4\_265 | A | E | A | G | F | R | A | V | A | A | D | L | R | G | L | G | N | S | T | A | G | G | G | R | - | - | - | - | F | D | K | E | L | L | A | G | D | V | I | A | L | A | D | R | - | L | D | - | - | - |
| 004 UniRef90\_A0A447J1A9\_28\_317 | A | A | Q | G | Y | M | A | I | A | V | D | P | P | G | T | G | F | S | A | R | P | D | S | G | - | - | - | - | Y | D | T | G | A | V | A | T | T | L | H | T | M | M | N | Q | - | L | G | - | - | - |
| 005 UniRef90\_A0A1Y6D2S0\_29\_302 | A | G | K | - | Y | T | V | V | A | P | D | L | R | G | L | G | L | S | E | K | T | R | T | G | - | - | - | - | Y | D | K | P | T | I | A | N | D | I | A | A | L | I | Q | H | - | L | G | R | - | - |
| 006 UniRef90\_A0A0G2FGE6\_47\_303 | A | D | A | G | Y | T | V | I | A | P | D | Y | R | G | A | G | H | S | S | K | P | R | D | G | - | - | - | - | Y | E | K | T | Q | M | A | A | D | I | H | K | L | L | T | E | H | L | G | I | - | - |
| 007 UniRef90\_F8JLL5\_12\_309 | A | D | R | - | F | H | V | I | A | I | D | L | P | G | Q | G | H | S | E | R | P | D | I | S | - | - | - | - | Y | D | T | H | T | V | A | A | H | V | H | A | A | V | E | A | - | L | G | - | - | - |
| 008 UniRef90\_A0A4R2Z7A8\_3\_305 | K | D | T | - | F | W | L | V | A | P | D | L | P | G | Q | G | D | S | D | R | P | E | T | G | - | - | - | - | Y | D | T | Q | S | L | A | Q | K | T | H | E | L | M | Q | S | - | L | G | - | - | - |
| 009 UniRef90\_UPI0016149F5E\_5\_284 | A | S | Q | G | Y | Q | A | I | A | I | D | P | P | G | S | G | Y | S | D | R | P | S | G | G | - | - | - | - | Y | D | T | G | A | V | A | A | T | L | H | R | A | M | L | A | - | L | G | - | - | - |
| 010 UniRef90\_A0A327RPK7\_10\_284 | A | K | S | - | Y | T | V | I | A | P | D | L | R | G | A | G | L | S | D | K | P | K | T | G | - | - | - | - | Y | D | K | L | T | L | A | Q | D | I | Y | L | L | V | E | Q | - | L | G | F | - | - |
| 011 UniRef90\_UPI00131EC7B5\_30\_311 | A | P | T | - | Y | R | I | I | A | P | D | L | P | G | Q | G | D | S | D | R | P | E | N | G | - | - | - | - | Y | D | T | K | S | L | A | A | K | V | H | G | L | L | A | Q | - | L | S | - | - | - |
| 012 UniRef90\_A0A1N6RLL9\_1\_202 | - | - | - | - | - | - | - | - | - | - | - | - | - | - | - | - | - | - | - | - | - | - | - | - | - | - | - | - | - | - | - | - | - | M | A | E | D | I | H | Q | L | S | L | Q | - | L | Q | - | - | - |
| 013 UniRef90\_A0A0N1GDE2\_28\_316 | A | R | E | - | H | T | V | V | A | V | D | S | R | G | A | G | L | S | D | K | P | D | D | G | - | - | - | - | Y | D | A | G | T | L | A | A | D | L | V | A | L | M | A | A | - | L | G | - | - | - |
| 014 UniRef90\_A0A1I6BK09\_10\_296 | A | E | H | - | Y | L | V | I | A | P | D | L | R | G | I | G | G | S | S | I | L | P | T | G | - | - | - | - | Y | D | K | R | T | L | A | A | D | I | Y | G | L | M | Q | Q | - | L | G | - | - | - |
| 015 UniRef90\_UPI00158A2D6D\_3\_306 | A | E | E | G | Y | R | V | V | A | V | D | Y | R | G | A | G | E | S | D | K | P | L | G | G | - | - | - | - | Y | D | K | A | S | M | A | G | D | I | R | A | L | A | Q | Q | - | L | G | A | - | - |
| 016 UniRef90\_UPI00160CFF45\_55\_340 | A | K | H | - | H | T | V | Y | A | V | D | L | P | G | L | G | D | S | T | G | S | P | T | G | - | - | - | - | Y | D | K | A | T | L | A | R | Y | V | H | T | L | M | A | D | R | L | G | - | - | - |
| 017 UniRef90\_A0A2N3KZL1\_30\_325 | A | K | T | - | H | S | V | V | A | V | D | L | P | G | L | G | Q | S | A | V | P | K | S | - | - | - | - | - | Y | V | G | Q | D | I | S | P | I | L | Y | G | L | A | K | Q | - | F | S | P | - | - |
| 018 UniRef90\_A0A2M9M9Q1\_18\_305 | A | E | R | - | F | R | V | I | A | V | D | P | R | G | V | G | R | S | D | K | P | H | G | G | - | - | - | - | Y | D | S | G | T | V | A | A | E | L | R | L | L | M | R | Q | - | L | G | - | - | - |
| 019 UniRef90\_UPI000361D127\_26\_313 | A | E | Q | - | Y | T | V | V | V | A | D | P | R | G | T | G | L | S | D | K | P | V | T | G | - | - | - | - | Y | D | S | A | T | C | A | Q | D | F | H | R | L | M | E | R | - | L | G | - | - | - |
| 020 UniRef90\_UPI001473614E\_45\_322 | A | R | D | - | H | T | V | I | A | V | D | L | P | G | L | G | D | S | S | V | P | A | S | G | - | - | - | - | Y | D | K | V | T | T | A | Q | R | V | R | Q | A | V | R | R | - | L | G | - | - | - |
| 021 UniRef90\_E6WJ64\_19\_301 | A | D | R | - | Y | F | I | I | A | P | D | L | P | G | Q | G | D | S | D | K | P | Q | A | G | - | - | - | - | Y | D | T | E | A | L | A | E | K | V | H | R | L | M | Q | Q | - | L | G | - | - | - |
| 022 UniRef90\_A0A6L3SWG4\_20\_294 | V | Q | A | G | L | R | V | V | M | P | D | Y | R | G | A | G | G | S | S | K | P | S | G | G | - | - | - | - | Y | D | K | H | T | M | A | G | D | L | H | A | L | L | Y | E | H | L | G | L | - | - |
| 023 UniRef90\_A0A109IGY4\_11\_301 | A | R | T | - | H | T | V | V | A | V | D | Q | R | G | T | G | L | S | D | K | P | D | A | G | - | - | - | - | Y | D | A | G | T | L | A | A | D | L | V | A | L | M | A | A | - | L | G | - | - | - |
| 024 UniRef90\_A0A0Q4UQ58\_27\_305 | S | D | R | - | F | T | V | I | A | P | D | L | R | G | L | G | L | S | E | R | T | V | S | G | - | - | - | - | Y | D | K | R | T | I | A | E | D | I | R | A | L | I | D | H | - | E | A | G | - | - |
| 025 UniRef90\_A0A239MTD5\_1\_271 | S | R | T | - | H | T | V | I | A | V | D | L | R | G | T | G | N | S | Q | V | T | E | S | G | - | - | - | - | Y | Q | A | A | Q | L | A | E | D | V | H | E | L | L | K | Q | - | L | G | L | - | - |
| 026 UniRef90\_UPI00049086FA\_57\_348 | L | A | G | - | R | T | V | I | A | V | D | L | P | G | M | G | D | S | T | G | E | P | P | A | - | - | - | - | Y | T | K | T | T | M | A | G | Y | V | H | A | L | L | G | A | I | G | R | - | - | - |
| 027 UniRef90\_A0A517LKB1\_23\_304 | A | T | A | G | Y | K | V | I | A | P | D | Y | R | G | H | G | H | S | N | P | P | T | N | S | T | V | D | M | Y | T | K | R | Q | L | A | A | D | L | H | D | L | V | T | K | H | L | D | I | - | - |
| 028 UniRef90\_UPI0004CCFD44\_55\_340 | A | E | H | - | H | T | V | Y | A | V | D | L | P | G | L | G | D | S | T | G | S | P | T | G | - | - | - | - | Y | D | K | A | T | L | A | R | Y | V | H | T | L | I | A | D | R | L | G | - | - | - |
| 029 UniRef90\_A0A285BKA7\_8\_299 | A | E | H | - | F | T | V | I | A | P | D | L | R | G | M | G | A | S | D | K | P | A | T | G | - | - | - | - | Y | D | A | A | T | P | A | D | D | M | A | E | L | M | T | A | - | L | G | - | - | - |
| 030 UniRef90\_A0A4R7C9R7\_33\_310 | A | R | R | - | F | R | V | V | A | A | D | L | R | G | T | G | L | S | E | V | T | P | S | G | - | - | - | - | Y | D | K | R | T | I | A | E | D | I | R | A | L | I | G | H | - | L | G | V | - | - |
| 031 UniRef90\_A0A4D4LDP7\_7\_304 | A | R | H | - | H | R | V | I | A | L | D | L | P | G | Q | G | L | S | D | R | P | A | D | G | - | - | - | - | Y | D | T | E | T | A | A | A | R | V | H | E | A | V | V | R | - | L | A | - | - | - |
| 032 UniRef90\_J4PG95\_17\_290 | A | E | A | G | Y | T | A | I | A | V | D | P | P | G | T | G | D | S | G | R | P | S | Q | G | - | - | - | - | Y | D | T | G | A | V | A | S | V | L | H | R | L | M | E | Q | - | L | G | - | - | - |
| 033 UniRef90\_A0A1Q8KTW7\_17\_294 | S | E | R | - | F | T | V | V | A | P | D | V | R | G | F | G | D | S | A | R | P | D | D | G | - | - | - | - | Y | D | M | G | M | I | A | R | D | I | G | A | L | M | T | E | - | L | G | H | - | - |
| 034 UniRef90\_UPI00076E3DF2\_55\_340 | A | E | H | - | H | T | V | Y | A | V | D | L | P | G | L | G | D | S | T | G | T | P | T | G | - | - | - | - | Y | D | K | A | T | L | A | R | Y | V | H | T | L | V | A | D | Q | L | G | - | - | - |
| 035 UniRef90\_A0A542JC81\_20\_314 | A | K | E | - | F | T | V | I | A | V | D | Q | R | G | I | G | L | S | E | K | P | A | S | G | - | - | - | - | Y | D | S | T | T | I | A | A | D | L | V | A | L | M | D | A | - | L | G | - | - | - |
| 036 UniRef90\_UPI00156E1DFF\_8\_289 | A | E | H | G | Y | R | V | I | A | P | D | L | R | G | I | G | A | S | D | R | P | L | S | G | - | - | - | - | Y | D | K | D | T | Q | A | E | D | M | R | E | L | L | A | Q | - | L | G | I | - | - |
| 037 UniRef90\_UPI00161A7EC4\_21\_297 | A | R | T | - | H | T | V | I | A | F | D | L | P | G | L | G | D | S | T | A | Q | S | G | G | - | - | - | - | Y | D | K | A | T | T | A | K | R | I | R | Q | A | V | N | K | - | L | G | - | - | - |
| 038 UniRef90\_UPI0012B05B7D\_16\_287 | A | N | K | - | Y | R | V | I | A | L | D | L | P | G | Q | G | D | S | D | K | P | P | G | G | - | - | - | - | Y | D | T | Q | T | T | A | E | R | I | H | G | F | V | E | K | - | L | G | - | - | - |
| 039 UniRef90\_A0A2I8F4B9\_53\_324 | V | A | R | G | Y | T | V | L | A | P | D | L | R | G | L | G | D | T | S | K | P | A | T | G | - | - | - | - | Y | D | K | A | N | V | A | E | D | I | R | A | L | V | A | K | - | L | N | L | - | - |
| 040 UniRef90\_A0A4D7B7N1\_7\_276 | A | E | R | - | H | K | V | I | A | I | D | L | P | G | Q | G | D | S | D | K | P | V | D | G | - | - | - | - | Y | D | T | R | T | T | G | E | R | I | H | A | L | L | Q | T | - | L | G | - | - | - |
| 041 UniRef90\_UPI000E275A28\_18\_308 | A | K | H | - | Y | T | L | I | V | P | D | P | R | G | L | G | L | S | D | K | P | A | T | G | - | - | - | - | Y | D | T | G | T | L | G | G | D | L | F | A | L | M | T | A | - | L | D | - | - | - |
| 042 UniRef90\_S3CY91\_25\_307 | A | K | A | G | Y | R | V | I | A | P | D | Y | R | G | A | G | E | S | S | H | P | A | N | G | - | - | - | - | F | D | K | L | T | I | A | T | D | L | H | K | L | L | H | D | H | L | N | I | - | - |
| 043 UniRef90\_A0A316VRQ3\_1\_263 | A | E | K | G | F | F | V | V | A | P | D | Y | R | G | A | G | G | S | S | M | P | L | G | G | - | - | - | - | Y | D | K | M | T | M | A | T | D | L | H | K | L | M | V | D | K | L | N | - | - | - |
| 044 UniRef90\_A0A4R6HBQ4\_11\_285 | A | A | A | G | F | R | V | I | A | P | D | L | R | G | F | G | D | S | S | K | P | E | G | I | - | - | - | - | H | S | K | L | V | V | A | G | D | L | V | A | M | L | D | R | - | L | G | - | - | - |
| 045 UniRef90\_UPI0015F81933\_16\_310 | A | H | R | - | F | H | V | I | A | I | D | L | P | G | Q | G | H | S | E | R | P | E | R | G | - | - | - | - | Y | D | T | H | T | V | A | A | H | V | H | A | A | V | K | A | - | L | G | - | - | - |
| 046 UniRef90\_UPI0009E804D9\_8\_293 | A | E | H | - | H | T | V | V | V | P | D | L | R | G | S | G | A | S | A | R | P | E | D | G | - | - | - | - | Y | H | K | T | N | Q | A | E | D | M | R | G | V | L | Q | H | - | L | G | L | - | - |
| 047 UniRef90\_A0A401ZLI5\_4\_287 | A | E | K | - | Y | T | V | I | A | P | D | S | R | G | I | G | D | S | E | R | T | D | S | G | - | - | - | - | Y | D | A | S | T | L | A | E | D | T | F | S | L | V | R | S | - | L | G | F | - | - |
| 048 UniRef90\_A0A6A6C1N5\_26\_297 | A | A | Q | G | Y | R | C | I | A | P | D | L | R | G | T | G | S | S | T | A | P | T | N | D | - | - | - | - | F | L | K | T | T | L | A | Q | D | L | L | A | L | L | - | D | H | L | H | L | - | - |
| 049 UniRef90\_A0A329J6I4\_30\_314 | A | R | N | - | H | T | V | V | A | V | D | L | P | G | L | G | E | S | Q | P | P | K | T | G | - | - | - | - | Y | S | G | T | A | V | S | K | Y | L | F | D | L | A | T | Q | - | L | S | G | - | - |
| 050 UniRef90\_A0A1B2HH26\_4\_280 | A | A | R | G | F | S | V | L | A | P | D | L | R | G | S | G | D | S | E | V | P | T | G | H | - | - | - | - | W | T | K | Q | D | E | A | D | D | L | H | E | L | L | H | H | - | L | G | - | - | - |
| 051 UniRef90\_A0A385B2U0\_45\_333 | A | K | D | - | H | T | V | I | A | V | D | L | R | G | T | G | D | S | S | V | P | Q | Q | G | - | - | - | - | Y | D | T | A | Q | L | A | D | D | V | H | E | L | L | T | R | - | L | N | L | - | - |
| 052 UniRef90\_A0A2E5PJQ7\_5\_284 | A | P | Y | - | Y | T | V | I | A | P | D | L | R | G | L | G | D | S | S | R | P | A | D | G | - | - | - | - | Y | D | K | Q | T | V | A | N | D | I | W | R | V | A | H | D | E | L | G | - | - | - |
| 053 UniRef90\_A0A5C8T429\_14\_295 | A | K | R | - | F | T | V | V | A | V | D | L | P | G | M | G | D | S | D | K | P | I | D | G | - | - | - | - | Y | D | T | G | T | V | A | M | R | L | H | D | L | T | R | A | - | L | G | - | - | - |
| 054 UniRef90\_A0A261TYY2\_43\_315 | A | R | H | - | Y | T | V | V | A | I | D | P | P | G | L | G | D | S | D | R | P | P | G | G | - | - | - | - | Y | D | T | D | A | A | A | D | R | L | H | A | F | T | Q | S | - | L | G | - | - | - |
| 055 UniRef90\_A0A6N7ZB66\_18\_297 | A | E | N | - | H | T | V | V | V | P | D | L | R | G | T | G | S | S | E | R | P | A | D | G | - | - | - | - | Y | L | K | T | N | Q | V | K | D | M | R | G | L | V | E | G | - | L | G | L | - | - |
| 056 UniRef90\_A0A2T6L0M5\_16\_299 | A | A | D | - | H | T | V | I | A | V | D | L | R | G | T | G | D | S | E | V | T | S | D | G | - | - | - | - | Y | D | T | V | Q | M | A | D | D | V | H | A | L | L | T | Q | - | L | G | L | - | - |
| 057 UniRef90\_G0FSK7\_17\_310 | A | R | D | - | F | T | V | V | A | V | D | P | R | G | T | G | L | S | G | Q | P | G | D | G | - | - | - | - | Y | D | T | A | T | L | A | A | D | L | T | G | L | M | A | A | - | L | G | - | - | - |
| 058 UniRef90\_A0A1Q8IR73\_6\_294 | G | S | R | - | F | R | V | V | A | I | D | L | P | G | Q | G | D | S | D | R | P | I | D | G | - | - | - | - | Y | D | T | Q | T | V | A | Q | R | V | H | D | L | V | S | H | - | L | G | - | - | - |
| 059 UniRef90\_A0A1I2MT44\_20\_306 | A | R | E | - | F | R | V | V | A | V | D | A | R | G | V | G | L | S | D | K | P | A | T | G | - | - | - | - | Y | D | T | G | T | L | A | R | D | M | A | G | L | M | T | A | - | L | G | - | - | - |
| 060 UniRef90\_UPI0010F95BB2\_18\_294 | A | R | A | G | Y | R | A | I | A | V | D | L | R | G | F | G | H | S | S | K | P | P | D | G | - | - | - | - | Y | D | K | K | T | V | A | R | D | L | H | E | L | L | D | S | - | L | A | - | - | - |
| 061 UniRef90\_A0A4P8X818\_19\_291 | S | S | K | - | F | T | V | I | V | P | D | L | R | G | F | G | D | S | T | R | P | K | D | G | - | - | - | - | Y | D | M | R | T | M | A | E | D | V | V | Q | L | A | S | R | - | L | G | F | - | - |
| 062 UniRef90\_A0A0M3UDU6\_18\_309 | V | D | A | G | Y | R | V | I | A | V | D | P | R | G | M | G | E | S | D | A | P | I | D | D | - | - | - | - | Y | D | L | G | T | V | A | A | E | L | R | S | F | A | E | T | - | I | G | L | F | E |
| 063 UniRef90\_A0A2P2CCU5\_19\_306 | A | Q | R | - | H | T | V | I | A | V | D | Q | R | G | M | G | L | T | D | K | P | D | D | G | - | - | - | - | Y | D | A | G | T | L | A | R | D | L | A | A | L | M | D | E | - | L | G | - | - | - |
| 064 UniRef90\_UPI0009781B11\_10\_287 | A | D | T | - | F | R | V | L | A | I | D | P | P | G | L | G | D | S | G | P | S | V | S | G | - | - | - | - | Y | D | T | R | A | I | A | L | H | L | D | A | L | F | A | Y | - | T | G | - | - | - |
| 065 UniRef90\_UPI000561A6EF\_30\_315 | A | E | H | - | F | S | V | V | A | V | D | P | R | G | V | G | L | S | D | K | P | A | T | G | - | - | - | - | Y | D | T | G | T | L | A | A | D | L | A | A | L | M | D | A | - | L | G | - | - | - |
| 066 UniRef90\_A0A1Z4J856\_27\_318 | A | E | R | - | Y | T | V | I | V | P | D | M | R | G | L | G | D | S | S | K | P | L | T | G | - | - | - | - | Y | D | A | R | T | V | A | D | D | I | Y | Q | L | V | G | K | - | L | G | F | - | - |
| 067 UniRef90\_A0A5C5SVV1\_11\_281 | A | P | H | - | H | T | I | V | M | P | D | L | R | G | A | G | Q | S | D | C | P | Q | G | G | - | - | - | - | Y | D | K | A | S | L | A | A | D | I | A | G | L | M | H | A | - | L | G | - | - | - |
| **068 Input\_protein\_seq** | A | E | D | G | Y | R | V | V | A | V | D | Y | R | G | A | G | E | S | D | K | P | L | G | G | - | - | - | - | Y | D | K | A | S | M | A | G | D | I | R | A | L | V | H | Q | - | L | G | A | - | - |
| 069 UniRef90\_A0A1Q7W147\_16\_308 | A | G | R | - | F | R | V | I | A | I | D | L | P | G | Q | G | H | S | E | R | P | V | R | S | - | - | - | - | Y | D | T | H | T | V | A | A | H | V | H | A | A | V | N | A | - | L | G | - | - | - |
| 070 UniRef90\_A0A0N0TCD7\_1\_288 | A | E | H | - | F | T | V | I | A | P | D | L | R | G | M | G | A | S | D | K | P | A | T | G | - | - | - | - | Y | D | A | A | T | L | A | D | D | M | A | T | L | M | T | A | - | L | G | - | - | - |
| 071 UniRef90\_UPI0004DF415F\_15\_308 | A | E | R | - | F | R | V | I | A | I | D | L | P | G | Q | G | H | S | D | R | P | Q | D | S | - | - | - | - | Y | D | T | H | T | V | A | S | R | V | Q | A | A | V | T | A | - | L | D | - | - | - |
| 072 UniRef90\_G7LVZ3\_7\_288 | A | S | R | - | F | T | V | I | A | P | D | M | R | G | F | G | D | S | D | K | P | T | G | G | - | - | - | - | Y | D | K | R | T | V | A | Q | D | I | R | Q | L | V | Q | H | - | L | G | F | - | - |

  
  

|  |  |  |  |  |  |  |  |  |  |  |  |  |  |  |  |  |  |  |  |  |  |  |  |  |  |  |  |  |  |  |  |  |  |  |  |  |  |  |  |  |  |  |  |  |  |  |  |  |  |  |
| --- | --- | --- | --- | --- | --- | --- | --- | --- | --- | --- | --- | --- | --- | --- | --- | --- | --- | --- | --- | --- | --- | --- | --- | --- | --- | --- | --- | --- | --- | --- | --- | --- | --- | --- | --- | --- | --- | --- | --- | --- | --- | --- | --- | --- | --- | --- | --- | --- | --- | --- |
| 001 UniRef90\_L9WLS3\_16\_284 | D | E | R | I | A | L | V | G | H | D | W | G | M | P | T | A | Y | A | Y | A | A | Q | Y | R | E | E | V | A | A | L | C | V | L | E | - | A | G | L | P | G | V | R | E | D | E | - | - | - | - | - |
| 002 UniRef90\_A0A0M4FVH2\_24\_312 | T | K | R | Y | F | L | A | A | H | D | V | G | A | W | V | A | Y | T | Y | A | A | L | F | G | D | E | V | R | R | L | A | L | L | D | - | A | G | I | P | G | I | T | M | P | D | A | L | P | - | - |
| 003 UniRef90\_UPI0016621A44\_4\_265 | V | A | R | F | A | V | V | G | H | D | W | G | G | T | V | G | Y | L | V | A | A | A | H | P | E | R | V | W | A | L | A | V | E | E | - | E | V | L | P | G | I | D | A | E | - | - | - | - | - | - |
| 004 UniRef90\_A0A447J1A9\_28\_317 | Y | K | T | Y | S | V | V | G | H | D | I | G | M | W | V | G | Y | A | L | A | G | D | Y | P | A | D | I | K | K | I | V | L | T | E | - | A | V | I | P | G | L | A | P | A | P | G | I | F | - | - |
| 005 UniRef90\_A0A1Y6D2S0\_29\_302 | - | G | P | A | F | V | V | G | H | D | M | G | G | K | A | A | Y | V | L | G | L | V | H | P | E | L | V | A | K | L | V | L | V | D | - | C | M | P | P | G | - | - | T | E | N | M | - | - | - | - |
| 006 UniRef90\_A0A0G2FGE6\_47\_303 | T | E | P | V | H | V | V | G | H | D | I | G | G | M | V | A | H | A | Y | A | S | R | F | A | N | E | T | A | S | V | A | L | G | E | - | F | P | M | P | G | T | K | V | Y | D | N | F | - | - | C |
| 007 UniRef90\_F8JLL5\_12\_309 | V | S | T | Y | W | L | A | A | H | D | V | G | A | W | V | A | F | S | L | A | L | T | Y | R | S | H | L | R | G | L | A | L | L | D | - | A | G | I | P | G | I | T | L | P | D | A | I | P | - | - |
| 008 UniRef90\_A0A4R2Z7A8\_3\_305 | H | T | R | Y | L | L | V | A | H | D | V | G | A | W | V | A | Y | P | Y | A | A | L | Y | P | N | E | V | K | R | L | A | L | L | D | - | A | G | I | P | G | I | T | L | P | D | A | L | P | - | - |
| 009 UniRef90\_UPI0016149F5E\_5\_284 | H | T | Q | Y | D | V | V | G | H | D | I | G | M | W | V | G | Y | A | L | A | S | D | F | P | Q | A | V | T | K | L | A | L | T | E | - | A | V | I | P | G | L | A | P | A | P | P | I | F | - | - |
| 010 UniRef90\_A0A327RPK7\_10\_284 | - | K | E | I | S | L | V | G | H | D | I | G | A | M | V | A | F | T | Y | A | A | E | Y | R | E | Y | V | K | K | L | V | L | L | D | - | V | L | L | P | G | F | G | L | E | K | L | M | - | - | - |
| 011 UniRef90\_UPI00131EC7B5\_30\_311 | V | S | R | Y | F | L | A | A | H | D | V | G | A | W | V | A | W | P | Y | A | S | L | Y | G | S | E | V | R | K | L | V | L | L | D | - | A | G | I | P | G | V | T | L | P | D | A | L | P | - | - |
| 012 UniRef90\_A0A1N6RLL9\_1\_202 | L | K | Q | V | Y | I | V | G | H | D | I | G | G | M | V | T | Y | A | L | L | R | L | Y | P | E | T | I | R | G | V | M | I | L | D | - | S | G | L | P | G | L | S | P | W | E | E | N | K | - | - |
| 013 UniRef90\_A0A0N1GDE2\_28\_316 | H | D | R | F | D | V | V | G | H | D | I | G | M | W | T | G | Y | A | L | A | A | D | H | P | E | R | V | S | R | L | A | V | V | D | - | A | I | I | P | G | L | T | P | T | P | S | V | F | - | - |
| 014 UniRef90\_A0A1I6BK09\_10\_296 | H | A | R | I | N | L | V | G | Y | D | L | G | A | G | V | A | Y | A | Y | A | A | A | H | P | E | A | V | R | R | L | V | F | M | E | - | F | A | L | P | G | F | G | V | W | E | Q | G | I | - | T |
| 015 UniRef90\_UPI00158A2D6D\_3\_306 | T | - | R | I | K | L | V | G | R | D | I | G | V | M | V | A | Y | A | Y | A | A | Q | W | P | A | E | V | L | K | L | A | M | L | D | - | V | P | V | P | G | T | R | I | W | D | E | A | - | - | - |
| 016 UniRef90\_UPI00160CFF45\_55\_340 | V | R | D | A | S | V | I | G | H | D | L | G | A | A | V | A | F | Q | Y | A | A | Q | F | P | A | D | T | A | R | L | G | Y | L | D | - | L | P | L | P | G | P | G | I | D | A | R | T | - | - | - |
| 017 UniRef90\_A0A2N3KZL1\_30\_325 | D | A | P | F | D | L | V | A | H | D | I | G | I | W | N | T | Y | P | M | A | V | S | H | Q | K | D | I | R | K | L | I | F | M | E | - | A | P | I | P | D | H | R | L | Y | D | F | P | A | - | - |
| 018 UniRef90\_A0A2M9M9Q1\_18\_305 | H | E | R | F | A | M | A | G | H | D | V | G | M | W | L | G | Y | A | L | A | A | D | H | G | D | A | V | T | R | L | A | L | L | D | - | A | T | L | P | G | L | A | P | D | L | P | L | F | - | - |
| 019 UniRef90\_UPI000361D127\_26\_313 | H | E | R | F | A | V | V | G | H | D | I | G | M | W | T | A | Y | A | M | A | V | D | T | P | G | R | V | Q | R | M | A | L | V | D | - | A | I | I | P | G | V | S | P | S | P | P | L | L | - | - |
| 020 UniRef90\_UPI001473614E\_45\_322 | F | D | Q | V | S | I | I | G | H | D | L | G | V | L | V | A | Y | P | W | A | R | D | H | P | G | E | V | T | R | L | V | M | I | E | - | T | P | L | S | G | F | G | L | E | E | L | Y | G | - | - |
| 021 UniRef90\_E6WJ64\_19\_301 | H | P | R | Y | Y | L | A | A | H | D | V | G | A | W | V | A | W | P | H | A | M | R | Y | S | Q | Q | V | I | K | L | A | L | L | D | - | A | G | I | P | G | I | T | L | P | E | A | L | P | - | - |
| 022 UniRef90\_A0A6L3SWG4\_20\_294 | T | G | P | V | T | V | A | G | H | D | I | G | M | M | V | A | Y | A | F | A | R | R | F | P | D | R | T | D | G | L | V | V | M | E | - | A | P | L | P | G | T | A | A | Y | E | R | S | - | - | - |
| 023 UniRef90\_A0A109IGY4\_11\_301 | H | D | R | F | D | V | V | G | H | D | I | G | M | W | T | G | Y | A | L | A | A | D | H | P | E | R | V | G | R | L | A | V | V | D | - | A | I | I | P | G | L | T | P | S | P | S | V | F | - | - |
| 024 UniRef90\_A0A0Q4UQ58\_27\_305 | - | G | R | A | G | V | V | G | H | D | M | G | G | K | A | A | Y | V | L | A | H | L | H | P | D | Q | I | S | R | L | V | L | A | D | - | C | L | L | P | G | - | - | T | E | N | M | - | - | - | - |
| 025 UniRef90\_A0A239MTD5\_1\_271 | N | N | D | I | Q | V | V | A | H | D | V | G | V | W | V | A | Y | A | Y | A | A | Q | W | P | S | E | V | R | R | M | A | V | M | E | - | A | P | I | A | D | G | S | A | Y | S | Y | P | A | - | - |
| 026 UniRef90\_UPI00049086FA\_57\_348 | P | H | G | V | R | I | A | A | H | D | F | G | V | G | V | A | Y | A | L | A | A | Q | H | R | E | Q | V | A | G | L | L | L | M | D | - | F | P | L | V | G | K | A | L | A | F | D | A | - | - | - |
| 027 UniRef90\_A0A517LKB1\_23\_304 | K | E | K | I | H | V | V | G | H | D | I | G | G | M | I | A | H | A | Y | V | A | Q | F | P | A | D | T | E | S | L | I | W | G | E | - | C | P | L | P | G | S | T | V | Y | E | K | - | - | - | T |
| 028 UniRef90\_UPI0004CCFD44\_55\_340 | V | R | D | A | R | V | V | G | H | D | F | G | A | A | V | A | F | Q | Y | A | T | Q | F | P | A | D | T | A | R | L | G | Y | L | D | - | L | P | L | P | G | P | A | I | D | A | S | T | - | - | - |
| 029 UniRef90\_A0A285BKA7\_8\_299 | H | E | R | F | A | V | V | G | Y | D | L | G | M | L | V | A | Y | A | L | A | A | S | H | R | N | R | V | T | H | F | V | G | A | E | - | S | I | L | P | G | L | T | P | F | P | P | M | L | - | - |
| 030 UniRef90\_A0A4R7C9R7\_33\_310 | - | P | Q | A | H | V | V | G | H | D | M | G | G | K | A | A | Y | I | L | A | H | L | H | P | G | V | V | S | K | L | V | L | V | D | - | C | L | L | P | G | - | - | T | E | N | M | - | - | - | - |
| 031 UniRef90\_A0A4D4LDP7\_7\_304 | G | G | R | Y | W | L | F | A | H | D | I | G | S | W | V | A | F | T | Y | A | L | R | Y | A | D | E | L | A | G | L | A | L | L | D | - | A | G | I | P | G | V | T | L | P | E | T | L | P | - | - |
| 032 UniRef90\_J4PG95\_17\_290 | H | A | A | Y | Q | V | V | G | H | D | I | G | M | W | V | A | Y | A | L | A | S | D | Y | P | E | A | V | R | R | L | V | L | T | E | - | A | V | I | P | G | L | A | P | A | P | P | I | F | - | - |
| 033 UniRef90\_A0A1Q8KTW7\_17\_294 | - | Q | R | F | A | I | A | G | E | D | W | G | A | A | F | A | Y | A | V | A | A | T | Y | P | E | R | V | T | K | L | S | F | A | E | - | M | L | L | P | G | F | G | L | E | D | W | S | A | L | T |
| 034 UniRef90\_UPI00076E3DF2\_55\_340 | V | R | D | A | R | V | V | G | H | D | F | G | A | A | V | A | F | Q | Y | A | A | Q | F | P | G | D | T | S | S | L | G | Y | L | D | - | L | P | L | P | G | P | E | L | D | A | S | T | - | - | - |
| 035 UniRef90\_A0A542JC81\_20\_314 | H | E | R | F | A | V | A | G | H | D | T | G | M | V | S | A | Y | A | L | A | A | D | Y | S | E | R | V | D | R | V | V | L | A | E | I | P | A | P | P | G | A | T | P | A | P | P | L | F | - | - |
| 036 UniRef90\_UPI00156E1DFF\_8\_289 | H | G | G | V | R | I | I | G | H | D | I | G | G | M | V | A | F | A | Y | A | R | R | H | P | E | G | V | E | R | L | V | L | V | E | - | L | A | L | P | G | L | G | L | E | Q | A | M | - | - | - |
| 037 UniRef90\_UPI00161A7EC4\_21\_297 | F | R | Q | V | G | L | I | G | H | D | L | G | A | L | I | A | Y | P | Y | A | R | D | F | P | N | E | V | T | K | L | A | V | L | E | - | T | P | L | A | G | F | G | L | E | N | L | Y | G | - | - |
| 038 UniRef90\_UPI0012B05B7D\_16\_287 | L | E | N | F | L | Y | V | G | H | D | I | G | A | W | V | G | Y | A | F | G | H | L | Y | A | S | S | L | R | G | I | V | L | L | D | - | A | N | I | P | G | V | T | L | Q | D | T | I | T | - | - |
| 039 UniRef90\_A0A2I8F4B9\_53\_324 | G | P | Q | V | N | V | V | G | H | D | M | G | G | M | V | A | Y | A | Y | A | A | Q | H | P | D | E | V | R | T | L | A | I | L | D | - | V | P | L | P | G | I | E | P | W | D | Q | L | - | - | - |
| 040 UniRef90\_A0A4D7B7N1\_7\_276 | H | D | R | Y | F | I | G | A | H | D | I | G | A | W | V | A | Y | P | Y | V | A | R | Y | A | D | E | V | R | R | L | V | L | L | D | - | A | N | I | P | G | V | T | L | R | P | T | V | D | - | - |
| 041 UniRef90\_UPI000E275A28\_18\_308 | H | E | H | F | A | M | V | G | H | D | C | G | M | W | V | G | Y | A | M | A | A | D | Q | P | Q | R | I | L | R | I | A | L | G | E | - | A | I | I | P | G | V | A | D | S | P | P | L | I | - | - |
| 042 UniRef90\_S3CY91\_25\_307 | K | K | S | I | H | V | V | G | H | D | I | G | G | M | I | A | H | A | Y | A | A | K | F | P | D | H | T | L | S | V | T | W | G | E | - | C | P | L | P | G | S | T | P | Y | E | S | G | - | - | F |
| 043 UniRef90\_A0A316VRQ3\_1\_263 | V | Q | A | Y | S | V | L | G | H | D | I | G | S | M | V | A | T | A | Q | A | L | K | F | R | D | H | V | K | A | L | I | I | M | E | - | C | P | Q | P | G | T | S | V | Y | K | A | F | T | - | - |
| 044 UniRef90\_A0A4R6HBQ4\_11\_285 | I | A | T | A | W | I | V | G | H | D | L | G | G | Q | V | A | Y | P | L | A | A | N | W | P | Q | R | T | R | G | L | V | F | I | E | - | S | G | L | P | G | F | G | Q | E | R | A | M | D | V | - |
| 045 UniRef90\_UPI0015F81933\_16\_310 | V | S | T | Y | W | L | V | A | H | D | I | G | A | W | V | A | F | S | L | A | L | K | Y | Q | S | R | L | R | G | L | A | L | L | D | - | A | G | I | P | G | I | T | L | P | D | A | I | P | - | - |
| 046 UniRef90\_UPI0009E804D9\_8\_293 | S | G | P | S | V | V | V | G | H | D | I | G | A | M | V | S | F | A | W | A | A | A | H | P | D | E | V | A | A | L | I | A | I | D | - | V | I | F | P | G | L | G | L | E | E | A | M | - | - | - |
| 047 UniRef90\_A0A401ZLI5\_4\_287 | - | Q | Q | V | F | L | V | G | H | D | L | G | V | L | T | A | Y | A | Y | A | A | R | Y | R | E | A | V | Q | R | L | V | I | L | D | - | S | P | V | E | G | F | G | S | E | D | F | V | - | - | - |
| 048 UniRef90\_A0A6A6C1N5\_26\_297 | P | S | P | I | H | I | I | G | H | D | I | G | G | M | V | A | Y | A | L | A | S | R | H | P | H | R | V | R | S | V | I | W | G | E | - | C | P | L | P | G | T | E | T | Y | R | L | - | - | - | D |
| 049 UniRef90\_A0A329J6I4\_30\_314 | N | Q | P | F | D | L | V | A | H | D | I | G | I | W | N | T | Y | P | L | A | V | M | H | Q | S | Q | I | K | K | L | V | F | M | E | - | A | P | I | P | D | K | S | V | Y | D | F | P | A | - | - |
| 050 UniRef90\_A0A1B2HH26\_4\_280 | H | D | H | A | F | V | L | G | T | D | V | G | T | M | T | V | H | A | W | A | Q | R | H | P | R | D | V | T | R | L | V | L | S | E | - | C | F | L | P | G | F | G | L | E | E | H | L | A | - | - |
| 051 UniRef90\_A0A385B2U0\_45\_333 | N | K | G | V | Q | I | V | A | H | D | I | G | A | W | I | A | Y | P | Y | A | A | M | W | P | N | E | V | S | R | M | V | V | M | E | - | G | P | I | P | D | R | S | L | Y | T | F | P | A | - | - |
| 052 UniRef90\_A0A2E5PJQ7\_5\_284 | H | E | R | F | L | L | V | G | H | D | W | G | G | P | T | A | F | A | L | A | A | T | H | P | E | A | V | E | K | L | A | I | L | D | - | V | V | I | P | G | - | - | - | - | - | - | - | - | - | D |
| 053 UniRef90\_A0A5C8T429\_14\_295 | W | R | Q | F | D | F | V | G | H | D | I | G | C | W | L | G | Y | P | F | V | A | T | Y | P | D | S | V | R | K | L | A | L | I | D | - | A | T | V | P | G | L | A | P | A | E | A | Y | A | - | - |
| 054 UniRef90\_A0A261TYY2\_43\_315 | W | D | R | F | H | Y | V | G | H | D | V | G | V | W | I | G | Y | A | Y | A | S | R | H | G | H | T | L | N | K | L | A | L | I | D | - | A | T | I | P | G | L | T | P | P | E | A | Y | A | - | - |
| 055 UniRef90\_A0A6N7ZB66\_18\_297 | S | G | P | I | V | V | V | G | H | D | I | G | A | M | V | A | F | A | W | A | V | H | R | P | E | D | L | T | A | A | V | V | L | D | - | C | F | L | P | G | V | D | L | E | D | S | M | - | - | - |
| 056 UniRef90\_A0A2T6L0M5\_16\_299 | N | D | G | I | Q | I | V | A | H | D | I | G | A | W | V | A | Y | P | Y | A | A | R | W | P | S | E | V | D | R | M | A | L | L | E | - | G | P | L | P | D | E | T | V | Y | D | Y | R | A | - | - |
| 057 UniRef90\_G0FSK7\_17\_310 | H | E | R | F | A | V | A | G | H | D | V | G | M | W | I | G | Y | A | M | A | A | D | H | P | G | R | V | A | R | L | A | L | A | E | - | A | L | I | P | G | L | S | P | S | P | P | L | F | - | - |
| 058 UniRef90\_A0A1Q8IR73\_6\_294 | L | K | R | Y | S | L | A | A | H | D | V | G | A | W | V | A | F | P | Y | A | H | L | F | G | E | E | V | E | A | L | A | L | M | D | - | A | G | I | P | G | I | T | L | P | D | M | L | P | - | - |
| 059 UniRef90\_A0A1I2MT44\_20\_306 | H | G | T | F | A | V | V | G | H | D | I | G | M | W | T | G | Y | A | L | A | A | D | H | G | E | R | V | E | R | L | A | V | A | E | - | A | A | V | P | G | L | T | P | S | P | P | L | F | - | - |
| 060 UniRef90\_UPI0010F95BB2\_18\_294 | I | Q | S | C | W | V | V | G | H | D | M | G | G | Q | V | A | Y | P | F | V | A | Q | W | P | E | R | A | K | G | L | V | F | I | E | - | S | G | L | P | G | F | G | Q | E | N | A | M | N | V | - |
| 061 UniRef90\_A0A4P8X818\_19\_291 | - | E | K | F | H | V | A | G | E | D | W | G | A | A | C | G | Y | A | L | A | A | A | Y | P | E | K | V | K | K | F | A | F | V | E | - | M | I | L | P | G | F | G | L | E | D | W | S | F | L | T |
| 062 UniRef90\_A0A0M3UDU6\_18\_309 | R | G | P | I | D | V | V | G | H | D | V | G | A | W | I | A | Y | A | W | A | A | D | W | R | S | D | I | R | R | I | A | L | L | D | - | A | L | I | P | G | V | S | A | P | R | T | D | L | - | - |
| 063 UniRef90\_A0A2P2CCU5\_19\_306 | H | D | R | F | A | L | V | G | H | D | T | G | M | V | I | S | Y | A | L | A | A | D | F | P | Q | R | V | D | R | V | A | L | A | E | V | P | G | P | P | T | P | D | H | S | P | P | L | F | - | - |
| 064 UniRef90\_UPI0009781B11\_10\_287 | D | T | Q | C | R | L | V | G | H | D | V | G | S | W | I | S | Y | A | Y | A | A | Q | R | P | E | R | V | T | H | L | A | L | I | D | - | A | A | V | P | G | L | A | P | D | D | I | Y | R | - | - |
| 065 UniRef90\_UPI000561A6EF\_30\_315 | H | A | R | F | S | V | A | G | H | D | V | G | M | W | T | G | Y | A | L | A | A | D | H | R | E | R | V | E | R | L | A | L | A | E | - | A | L | V | A | G | I | S | P | S | P | P | L | L | - | - |
| 066 UniRef90\_A0A1Z4J856\_27\_318 | - | K | R | I | F | L | V | G | H | D | I | G | A | P | V | A | Y | A | Y | A | A | A | H | P | E | D | V | R | R | L | A | V | L | E | - | L | V | L | A | G | A | G | L | E | E | L | I | - | - | - |
| 067 UniRef90\_A0A5C5SVV1\_11\_281 | H | P | R | Y | A | V | C | G | H | D | I | G | G | M | V | G | M | A | L | A | M | T | R | R | Q | A | V | T | H | L | A | I | L | D | - | V | P | L | P | G | W | S | R | W | E | T | L | F | - | - |
| **068 Input\_protein\_seq** | T | - | R | I | H | L | V | G | R | D | I | G | V | M | V | A | Y | A | Y | A | A | Q | W | P | T | E | I | V | K | L | A | M | L | D | - | V | P | V | P | G | T | R | I | W | D | E | A | - | - | - |
| 069 UniRef90\_A0A1Q7W147\_16\_308 | A | P | T | Y | W | L | A | G | H | D | V | G | A | W | V | A | F | S | L | A | L | N | Y | E | S | Q | L | R | G | L | A | L | L | D | - | A | G | I | P | G | V | T | L | P | E | A | I | P | - | - |
| 070 UniRef90\_A0A0N0TCD7\_1\_288 | H | D | R | F | A | V | V | G | Y | D | L | G | M | L | V | G | Y | A | L | A | S | R | H | R | V | R | V | T | R | L | A | V | S | E | - | A | V | I | P | G | L | S | P | S | P | P | L | M | - | - |
| 071 UniRef90\_UPI0004DF415F\_15\_308 | V | P | K | Y | W | L | V | A | H | D | I | G | A | W | V | A | F | S | L | A | L | K | Y | E | E | Q | L | H | G | V | A | L | L | D | - | A | G | I | P | G | I | T | L | P | D | T | I | P | - | - |
| 072 UniRef90\_G7LVZ3\_7\_288 | - | C | E | I | N | L | V | G | H | D | I | G | M | M | V | A | Y | E | Y | A | S | A | H | P | K | E | I | R | R | L | A | V | L | E | - | A | G | L | P | G | L | G | L | E | A | L | M | - | - | - |

  
  

|  |  |  |  |  |  |  |  |  |  |  |  |  |  |  |  |  |  |  |  |  |  |  |  |  |  |  |  |  |  |  |  |  |  |  |  |  |  |  |  |  |  |  |  |  |  |  |  |  |  |  |
| --- | --- | --- | --- | --- | --- | --- | --- | --- | --- | --- | --- | --- | --- | --- | --- | --- | --- | --- | --- | --- | --- | --- | --- | --- | --- | --- | --- | --- | --- | --- | --- | --- | --- | --- | --- | --- | --- | --- | --- | --- | --- | --- | --- | --- | --- | --- | --- | --- | --- | --- |
| 001 UniRef90\_L9WLS3\_16\_284 | - | - | - | - | - | - | K | R | - | H | F | W | H | T | R | - | F | H | - | G | V | R | D | L | P | E | R | L | V | A | G | R | E | - | R | M | Y | L | E | W | F | Y | - | - | - | - | - | - | - | K |
| 002 UniRef90\_A0A0M4FVH2\_24\_312 | - | T | A | P | E | - | R | A | W | K | T | W | H | F | A | - | F | H | - | V | I | P | D | L | P | E | M | L | I | A | G | R | E | - | R | E | Y | L | D | W | F | L | - | - | - | - | - | - | - | R |
| 003 UniRef90\_UPI0016621A44\_4\_265 | I | P | E | P | G | R | S | H | Y | P | D | W | H | G | P | - | F | N | - | R | R | A | G | L | A | E | T | L | V | P | G | R | E | - | D | A | Y | Y | G | T | F | L | - | - | - | - | - | - | - | R |
| 004 UniRef90\_A0A447J1A9\_28\_317 | - | V | D | P | E | - | E | N | I | F | L | W | H | F | M | - | F | N | - | Q | V | Q | D | L | P | E | T | L | A | A | G | K | E | - | R | E | Y | L | N | F | I | F | - | - | - | - | - | - | - | D |
| 005 UniRef90\_A0A1Y6D2S0\_29\_302 | - | - | D | S | A | - | K | G | - | G | M | W | H | Y | G | - | F | H | - | M | A | A | G | F | P | E | M | L | T | K | N | R | E | - | R | E | Y | I | A | A | Q | I | - | - | - | - | - | - | - | K |
| 006 UniRef90\_A0A0G2FGE6\_47\_303 | R | E | N | P | G | - | - | - | - | - | V | W | H | F | H | - | F | H | - | W | Q | T | D | L | P | E | L | L | T | Q | G | R | E | - | R | Q | Y | I | K | H | F | Y | - | - | - | - | - | - | - | D |
| 007 UniRef90\_F8JLL5\_12\_309 | - | T | D | P | E | - | Q | A | W | K | T | W | H | F | A | - | F | H | - | L | V | P | D | L | P | E | T | L | L | A | G | R | E | - | R | E | Y | V | G | W | F | L | - | - | - | - | - | - | - | K |
| 008 UniRef90\_A0A4R2Z7A8\_3\_305 | - | V | T | P | D | - | K | S | W | K | T | W | H | F | A | - | F | H | - | T | L | P | D | L | P | E | A | L | I | T | G | N | E | - | R | I | Y | L | D | W | F | L | - | - | - | - | - | - | - | R |
| 009 UniRef90\_UPI0016149F5E\_5\_284 | - | V | A | P | A | - | D | N | I | F | L | W | H | F | M | - | F | N | - | Q | V | P | D | L | P | E | M | L | T | A | G | K | E | - | R | E | Y | I | R | F | I | F | - | - | - | - | - | - | - | D |
| 010 UniRef90\_A0A327RPK7\_10\_284 | - | - | D | V | A | - | N | G | - | G | M | W | H | F | G | - | F | H | - | M | V | H | D | L | P | E | A | L | I | T | G | R | E | - | H | I | Y | L | D | H | F | F | - | - | - | - | - | - | - | K |
| 011 UniRef90\_UPI00131EC7B5\_30\_311 | - | V | T | P | D | - | K | A | W | K | T | W | H | F | A | - | F | H | - | A | I | P | D | L | P | E | A | L | I | A | G | K | E | - | L | V | Y | L | D | W | F | L | - | - | - | - | - | - | - | K |
| 012 UniRef90\_A0A1N6RLL9\_1\_202 | - | - | - | - | - | - | A | N | P | F | L | W | H | F | G | - | F | H | - | Q | T | P | D | V | P | E | K | L | I | T | G | R | Q | F | I | Y | F | R | E | G | M | F | - | - | - | - | - | - | - | N |
| 013 UniRef90\_A0A0N1GDE2\_28\_316 | - | S | P | A | A | - | V | N | Q | R | L | W | H | F | G | - | F | N | - | R | L | T | D | L | N | E | E | L | V | R | G | R | E | - | R | L | F | F | G | Y | Q | F | - | - | - | - | - | - | - | A |
| 014 UniRef90\_A0A1I6BK09\_10\_296 | P | G | P | D | W | - | H | N | G | V | N | W | H | A | A | - | L | F | - | T | L | P | D | V | A | E | S | F | M | G | G | Q | E | - | R | K | F | L | S | W | I | F | - | - | - | - | - | - | - | W |
| 015 UniRef90\_UPI00158A2D6D\_3\_306 | - | - | K | A | R | - | S | D | P | Q | I | W | H | F | G | - | L | H | - | Q | Q | R | D | I | A | E | M | L | I | A | G | K | E | - | H | A | Y | I | L | D | F | Y | K | K | R | I | - | - | H | V |
| 016 UniRef90\_UPI00160CFF45\_55\_340 | - | - | - | - | - | - | Y | R | S | L | S | W | H | I | A | - | F | H | - | S | Q | P | K | V | P | E | T | V | V | G | N | D | V | - | R | D | Y | L | A | L | F | Y | P | Q | V | S | F | G | G | T |
| 017 UniRef90\_A0A2N3KZL1\_30\_325 | - | F | T | P | E | - | G | E | S | L | V | W | H | F | S | F | F | A | - | A | G | N | N | L | A | E | T | L | V | T | G | H | E | - | R | M | F | L | E | H | F | I | - | - | - | - | - | - | - | K |
| 018 UniRef90\_A0A2M9M9Q1\_18\_305 | - | G | S | E | A | - | Q | N | D | L | L | W | H | F | A | - | F | N | - | R | K | R | S | I | N | E | L | L | V | R | G | R | E | - | H | L | Y | Y | G | D | Q | F | - | - | - | - | - | - | - | R |
| 019 UniRef90\_UPI000361D127\_26\_313 | - | G | D | R | R | - | T | S | D | F | L | W | H | F | N | - | F | N | - | R | A | H | D | V | N | E | Q | L | V | Q | G | R | E | - | D | V | Y | F | G | Y | Q | F | - | - | - | - | - | - | - | A |
| 020 UniRef90\_UPI001473614E\_45\_322 | - | - | - | - | - | - | - | - | - | V | S | W | H | F | R | - | F | N | M | S | P | A | P | I | P | E | R | I | M | D | N | D | D | V | S | T | Y | L | G | M | I | F | D | F | S | - | - | - | - | - |
| 021 UniRef90\_E6WJ64\_19\_301 | - | A | T | P | D | - | K | A | W | K | T | W | H | F | A | - | F | H | - | L | L | P | D | L | P | E | A | L | I | S | G | R | E | - | E | I | Y | L | E | W | F | L | - | - | - | - | - | - | - | R |
| 022 UniRef90\_A0A6L3SWG4\_20\_294 | - | - | L | G | D | - | T | K | - | R | V | W | H | F | H | - | F | H | - | R | A | P | D | L | P | E | L | L | T | A | G | R | E | - | Q | L | Y | L | E | R | F | Y | - | - | - | - | - | - | - | L |
| 023 UniRef90\_A0A109IGY4\_11\_301 | - | S | P | A | A | - | A | N | Q | R | F | W | H | F | G | - | F | N | - | R | L | D | D | L | N | E | E | L | V | R | G | R | E | - | R | L | F | F | G | Y | Q | F | - | - | - | - | - | - | - | A |
| 024 UniRef90\_A0A0Q4UQ58\_27\_305 | - | - | D | P | L | - | R | G | - | G | A | W | H | Y | G | - | F | H | - | M | A | P | D | I | P | E | K | L | T | A | G | R | E | - | R | G | Y | I | R | D | Q | I | - | - | - | - | - | - | - | R |
| 025 UniRef90\_A0A239MTD5\_1\_271 | - | L | N | A | D | P | E | K | P | S | P | W | H | W | G | M | F | Q | - | M | - | - | P | L | A | E | H | L | I | A | G | H | E | - | R | V | F | I | Q | E | M | I | - | - | - | - | - | - | - | T |
| 026 UniRef90\_UPI00049086FA\_57\_348 | - | - | - | - | - | - | V | R | P | L | S | W | H | F | S | - | F | N | - | L | Q | E | P | L | D | E | Q | L | V | T | G | R | E | - | G | A | F | L | R | Y | F | F | Q | H | S | R | I | - | - | S |
| 027 UniRef90\_A0A517LKB1\_23\_304 | K | H | D | P | D | - | - | - | - | - | L | W | H | F | D | - | F | Q | S | H | H | P | D | L | A | V | S | L | V | T | G | K | E | - | E | L | Y | L | T | H | F | Y | - | - | - | - | - | - | - | D |
| 028 UniRef90\_UPI0004CCFD44\_55\_340 | - | - | - | - | - | - | Y | R | S | L | S | W | H | I | A | - | F | H | - | S | Q | R | R | V | P | E | A | V | V | G | D | D | V | - | R | E | Y | L | A | L | F | Y | P | Q | V | S | F | G | G | T |
| 029 UniRef90\_A0A285BKA7\_8\_299 | - | T | D | P | T | - | V | N | E | V | M | A | H | F | V | - | I | N | - | R | L | A | D | L | N | E | R | L | I | A | G | R | E | - | E | I | Y | F | G | H | Q | F | - | - | - | - | - | - | - | A |
| 030 UniRef90\_A0A4R7C9R7\_33\_310 | - | - | D | A | L | - | R | G | - | G | A | W | H | Y | G | - | F | H | - | M | A | P | D | F | P | E | M | L | T | K | G | R | E | - | R | D | Y | I | A | A | Q | I | - | - | - | - | - | - | - | R |
| 031 UniRef90\_A0A4D4LDP7\_7\_304 | - | L | N | P | A | - | G | A | W | K | M | W | H | F | A | - | F | H | - | Q | V | P | D | L | P | E | V | L | L | E | G | K | E | - | R | E | Y | V | S | W | F | L | - | - | - | - | - | - | - | R |
| 032 UniRef90\_J4PG95\_17\_290 | - | V | P | P | A | - | E | N | I | F | L | W | H | F | M | - | F | N | - | Q | L | Q | D | L | P | E | A | L | I | T | G | R | E | - | R | A | Y | L | S | F | I | F | - | - | - | - | - | - | - | D |
| 033 UniRef90\_A0A1Q8KTW7\_17\_294 | A | D | N | V | F | - | S | D | H | F | L | W | H | V | G | - | F | F | - | H | V | R | D | F | P | E | M | L | I | S | G | R | E | - | E | Q | F | W | S | T | W | M | - | - | - | - | - | - | - | K |
| 034 UniRef90\_UPI00076E3DF2\_55\_340 | - | - | - | - | - | - | Y | R | S | M | S | W | H | I | A | - | F | H | - | S | Q | P | E | I | P | E | A | V | V | G | D | D | V | - | R | D | Y | L | A | L | F | Y | P | Q | V | S | Y | E | G | S |
| 035 UniRef90\_A0A542JC81\_20\_314 | - | V | P | A | P | - | L | N | A | K | L | W | H | I | P | - | F | N | - | R | A | E | G | L | A | E | Q | L | I | A | G | R | E | - | D | V | Y | F | G | Y | E | F | - | - | - | - | - | - | - | A |
| 036 UniRef90\_UPI00156E1DFF\_8\_289 | - | - | D | V | A | - | S | G | - | G | L | F | H | F | G | - | L | F | - | M | T | E | E | L | P | E | L | L | L | D | G | R | E | - | S | D | F | L | T | W | W | F | - | - | - | - | - | - | - | N |
| 037 UniRef90\_UPI00161A7EC4\_21\_297 | - | - | - | - | - | - | - | - | - | I | S | F | H | F | K | - | F | N | M | A | P | A | P | V | P | E | S | I | L | D | N | D | D | V | P | T | Y | L | G | F | M | F | G | F | S | - | - | - | - | - |
| 038 UniRef90\_UPI0012B05B7D\_16\_287 | - | L | G | P | D | - | N | - | W | R | N | W | H | F | L | - | F | N | - | P | I | A | D | L | P | E | A | L | L | A | G | R | E | - | R | I | L | I | E | W | F | F | - | - | - | - | - | - | - | K |
| 039 UniRef90\_A0A2I8F4B9\_53\_324 | - | - | V | - | - | - | Q | T | P | R | T | W | H | F | R | - | F | Y | - | S | V | Q | D | V | P | E | M | L | I | A | G | H | E | - | L | E | Y | L | K | W | F | H | - | - | - | - | - | - | - | N |
| 040 UniRef90\_A0A4D7B7N1\_7\_276 | - | V | G | P | D | - | N | - | W | K | S | W | H | F | F | - | F | H | - | P | V | P | D | L | P | E | A | L | I | T | G | R | E | - | R | L | Y | I | E | W | F | F | - | - | - | - | - | - | - | Q |
| 041 UniRef90\_UPI000E275A28\_18\_308 | P | D | D | R | K | - | I | S | D | F | L | W | H | N | N | - | F | C | - | R | A | R | G | I | N | E | E | M | V | A | G | R | E | - | E | I | F | F | G | Y | Q | F | - | - | - | - | - | - | - | T |
| 042 UniRef90\_S3CY91\_25\_307 | K | N | S | P | G | - | - | - | - | - | M | W | H | F | T | - | F | H | - | Q | Q | T | D | L | P | E | A | L | V | A | G | R | E | - | R | I | Y | L | K | H | F | Y | - | - | - | - | - | - | - | D |
| 043 UniRef90\_A0A316VRQ3\_1\_263 | T | E | P | E | L | - | T | L | G | P | T | F | H | F | F | - | F | H | - | H | A | D | N | L | P | E | Q | L | T | Y | G | R | E | - | D | L | Y | L | Q | H | L | Y | - | - | - | - | - | - | - | D |
| 044 UniRef90\_A0A4R6HBQ4\_11\_285 | - | - | - | - | - | - | A | N | G | G | S | W | H | F | G | - | F | N | - | R | A | G | D | I | A | E | E | L | V | R | G | R | E | - | H | L | F | I | K | A | M | I | R | R | E | - | - | - | - | N |
| 045 UniRef90\_UPI0015F81933\_16\_310 | - | T | D | P | E | - | Q | A | W | K | T | W | H | F | A | - | F | H | - | L | V | P | D | L | P | E | T | L | L | A | G | R | E | - | R | E | Y | V | G | W | F | L | - | - | - | - | - | - | - | K |
| 046 UniRef90\_UPI0009E804D9\_8\_293 | - | - | N | V | A | - | T | G | - | G | M | W | H | F | G | - | F | F | - | M | Q | P | H | I | P | E | M | L | F | D | G | H | E | - | L | E | F | F | T | T | T | F | - | - | - | - | - | - | - | T |
| 047 UniRef90\_A0A401ZLI5\_4\_287 | - | - | T | K | L | - | K | - | - | - | I | W | H | F | G | L | F | Q | - | A | P | R | N | L | A | E | S | L | A | E | G | R | E | - | H | I | L | L | Q | W | F | F | - | - | - | - | - | - | - | S |
| 048 UniRef90\_A0A6A6C1N5\_26\_297 | R | T | V | P | S | - | R | T | T | Q | Q | F | H | F | V | - | F | H | - | T | V | P | D | L | A | A | M | L | V | Q | G | K | E | - | K | G | Y | V | E | H | F | L | - | - | - | - | - | - | - | R |
| 049 UniRef90\_A0A329J6I4\_30\_314 | - | F | S | P | E | - | G | E | S | L | V | W | H | F | S | F | F | A | - | A | G | D | H | M | A | E | T | L | I | K | G | H | E | - | K | F | F | L | K | R | F | I | - | - | - | - | - | - | - | Q |
| 050 UniRef90\_A0A1B2HH26\_4\_280 | - | - | - | - | - | - | - | - | - | S | L | W | H | F | G | - | F | H | - | A | Q | S | E | F | A | A | Q | L | V | A | G | R | E | - | E | Q | Y | L | T | W | F | W | - | R | Q | - | - | - | - | - |
| 051 UniRef90\_A0A385B2U0\_45\_333 | - | F | P | A | E | - | G | G | L | S | T | W | H | L | G | F | F | Q | - | K | - | - | D | F | A | Q | D | L | V | R | G | H | E | - | R | D | L | I | K | G | F | V | - | - | - | - | - | - | - | E |
| 052 UniRef90\_A0A2E5PJQ7\_5\_284 | G | G | D | F | S | - | Q | G | G | R | R | W | H | H | P | - | F | H | - | I | T | P | D | L | P | E | A | L | T | K | G | R | E | - | D | I | Y | L | G | W | F | Y | - | - | - | - | - | - | - | S |
| 053 UniRef90\_A0A5C8T429\_14\_295 | - | F | A | P | E | - | R | I | G | R | N | W | H | F | F | - | F | N | - | A | L | S | D | L | P | E | T | L | L | A | G | R | E | - | R | E | F | L | S | W | L | F | - | - | - | - | - | - | - | Q |
| 054 UniRef90\_A0A261TYY2\_43\_315 | - | F | E | P | Q | - | R | I | S | K | N | W | H | F | Y | - | F | F | - | A | M | P | D | L | A | E | A | L | L | T | G | R | E | - | R | E | F | L | G | W | L | F | - | - | - | - | - | - | - | Q |
| 055 UniRef90\_A0A6N7ZB66\_18\_297 | - | - | N | V | V | - | K | G | - | G | S | W | H | F | G | - | F | F | - | A | A | P | H | I | P | E | M | L | F | A | G | H | E | - | L | E | F | V | Q | A | T | F | - | - | - | - | - | - | - | G |
| 056 UniRef90\_A0A2T6L0M5\_16\_299 | - | L | D | P | D | - | G | G | P | S | I | W | H | Y | G | F | F | Q | - | A | - | - | D | I | A | E | G | L | I | A | G | N | E | - | R | A | L | V | E | G | F | I | - | - | - | - | - | - | - | G |
| 057 UniRef90\_G0FSK7\_17\_310 | - | A | P | R | D | - | V | V | E | R | L | W | H | F | A | - | F | N | - | Q | L | D | D | L | N | E | Q | L | V | A | G | R | E | - | D | V | F | L | R | W | Q | F | - | - | - | - | - | - | - | T |
| 058 UniRef90\_A0A1Q8IR73\_6\_294 | - | S | S | S | D | - | R | S | W | K | T | W | H | F | S | - | F | H | - | A | V | P | D | L | P | E | I | L | L | A | G | R | E | - | R | A | Y | L | E | W | F | F | - | - | - | - | - | - | - | W |
| 059 UniRef90\_A0A1I2MT44\_20\_306 | - | S | A | A | E | - | V | N | D | R | L | W | H | F | A | - | F | N | - | R | L | G | G | I | N | E | K | L | V | E | G | R | E | - | H | L | F | F | G | H | Q | F | - | - | - | - | - | - | - | T |
| 060 UniRef90\_UPI0010F95BB2\_18\_294 | - | - | - | - | - | - | A | Q | G | G | S | W | H | F | G | - | F | N | - | A | A | G | D | I | A | E | A | L | V | A | G | R | E | - | H | M | F | I | R | H | M | F | H | R | D | - | - | - | - | K |
| 061 UniRef90\_A0A4P8X818\_19\_291 | T | E | N | V | N | - | S | N | R | W | L | W | H | I | N | - | F | Y | - | S | V | P | D | F | P | E | L | L | I | T | G | R | E | - | R | Q | Y | F | D | K | F | F | - | - | - | - | - | - | - | K |
| 062 UniRef90\_A0A0M3UDU6\_18\_309 | - | S | V | D | E | - | A | N | R | R | S | W | H | F | A | - | F | N | - | Q | L | D | D | L | P | E | L | L | I | S | G | R | E | - | D | V | F | L | T | W | L | F | - | - | - | - | - | - | - | R |
| 063 UniRef90\_A0A2P2CCU5\_19\_306 | - | V | P | R | A | - | L | N | D | R | I | W | H | I | A | - | F | N | - | R | A | G | A | V | A | E | E | L | V | A | G | R | E | - | A | L | F | F | G | Y | E | F | - | - | - | - | - | - | - | A |
| 064 UniRef90\_UPI0009781B11\_10\_287 | - | L | T | P | Q | - | T | L | E | K | T | W | H | F | A | - | F | N | - | F | L | P | E | L | S | E | L | L | V | T | G | R | E | - | R | E | F | L | A | W | L | F | - | - | - | - | - | - | - | R |
| 065 UniRef90\_UPI000561A6EF\_30\_315 | - | G | D | H | E | - | A | N | R | R | L | W | H | F | A | - | F | N | - | R | L | D | G | L | N | E | E | L | V | R | G | R | E | - | R | L | Y | F | G | H | Q | F | - | - | - | - | - | - | - | A |
| 066 UniRef90\_A0A1Z4J856\_27\_318 | - | - | N | K | A | - | S | A | - | A | L | W | H | F | S | - | F | Q | - | A | T | R | D | L | P | E | A | L | V | E | G | K | E | - | R | L | Y | L | S | V | F | L | - | - | - | - | - | - | - | R |
| 067 UniRef90\_A0A5C5SVV1\_11\_281 | - | G | D | P | - | - | - | - | - | R | V | W | H | F | S | - | F | H | - | M | K | E | N | L | P | E | R | L | L | Y | G | R | E | - | Y | D | Y | V | S | T | F | I | - | - | - | - | - | - | - | F |
| **068 Input\_protein\_seq** | - | - | K | A | S | - | A | D | P | Q | I | W | H | F | G | - | L | H | - | Q | Q | R | D | I | A | E | M | L | I | A | G | K | E | - | R | A | Y | I | L | D | F | Y | K | K | R | T | - | - | H | V |
| 069 UniRef90\_A0A1Q7W147\_16\_308 | - | T | D | P | N | - | L | A | W | K | T | W | H | F | A | - | F | H | - | L | V | P | D | L | P | E | T | L | L | A | G | R | E | - | R | E | Y | V | D | W | F | L | - | - | - | - | - | - | - | T |
| 070 UniRef90\_A0A0N0TCD7\_1\_288 | - | S | D | P | A | - | T | N | E | M | L | W | H | F | A | - | F | N | - | R | L | P | D | I | N | E | R | M | V | A | G | R | E | - | E | I | Y | F | G | H | T | F | - | - | - | - | - | - | - | T |
| 071 UniRef90\_UPI0004DF415F\_15\_308 | - | T | D | P | D | - | H | A | W | K | T | W | H | F | A | - | F | H | - | L | V | T | D | L | P | E | T | L | L | T | G | R | E | - | R | E | Y | L | E | W | F | L | - | - | - | - | - | - | - | N |
| 072 UniRef90\_G7LVZ3\_7\_288 | - | - | N | T | A | - | K | F | P | Q | F | W | H | F | G | - | F | F | - | S | A | P | G | V | A | E | T | L | I | A | G | R | E | - | K | M | F | L | A | H | F | I | - | - | - | - | - | - | - | R |

  
  

|  |  |  |  |  |  |  |  |  |  |  |  |  |  |  |  |  |  |  |  |  |  |  |  |  |  |  |  |  |  |  |  |  |  |  |  |  |  |  |  |  |  |  |  |  |  |  |  |  |  |  |
| --- | --- | --- | --- | --- | --- | --- | --- | --- | --- | --- | --- | --- | --- | --- | --- | --- | --- | --- | --- | --- | --- | --- | --- | --- | --- | --- | --- | --- | --- | --- | --- | --- | --- | --- | --- | --- | --- | --- | --- | --- | --- | --- | --- | --- | --- | --- | --- | --- | --- | --- |
| 001 UniRef90\_L9WLS3\_16\_284 | E | G | A | - | Y | D | P | - | A | A | I | - | - | D | S | D | A | R | D | E | Y | V | R | C | Y | - | S | Q | P | G | G | L | R | G | G | F | E | Y | Y | R | A | Y | - | - | - | D | D | D | A | E |
| 002 UniRef90\_A0A0M4FVH2\_24\_312 | R | K | A | - | A | N | P | - | E | A | F | - | - | S | E | A | D | I | E | E | Y | L | R | V | F | - | K | K | A | G | G | L | R | A | G | L | A | Y | Y | R | A | A | - | - | - | S | L | S | A | Q |
| 003 UniRef90\_UPI0016621A44\_4\_265 | Q | S | A | G | P | A | P | - | L | A | L | - | - | P | G | D | A | E | Q | V | Y | L | D | A | Y | - | R | R | P | G | T | L | A | A | G | L | G | Y | Y | R | T | A | - | - | - | Q | A | D | A | A |
| 004 UniRef90\_A0A447J1A9\_28\_317 | H | W | A | - | Y | C | - | - | - | - | R | - | - | D | R | V | A | A | Q | T | Y | I | D | A | Y | - | S | S | P | G | G | L | R | A | G | F | A | Y | Y | R | A | I | - | - | - | P | Q | T | I | A |
| 005 UniRef90\_A0A1Y6D2S0\_29\_302 | Q | W | A | - | H | R | K | - | Q | A | I | - | - | T | P | Q | A | I | D | E | Y | A | K | H | Y | - | A | R | P | G | G | M | T | A | G | F | N | Y | Y | R | A | L | - | - | - | L | D | D | A | Q |
| 006 UniRef90\_A0A0G2FGE6\_47\_303 | R | L | C | - | I | N | P | - | S | A | I | - | - | S | P | A | D | V | D | Y | Y | A | S | M | F | - | E | K | A | G | A | M | R | A | G | F | D | V | Y | R | A | F | - | - | - | H | K | D | A | E |
| 007 UniRef90\_F8JLL5\_12\_309 | A | K | T | - | L | S | S | - | D | T | F | - | - | D | D | T | E | T | E | H | Y | A | A | S | V | - | A | A | D | G | G | L | R | A | S | L | A | Y | Y | R | D | A | - | - | - | A | E | S | A | R |
| 008 UniRef90\_A0A4R2Z7A8\_3\_305 | R | K | T | - | A | A | P | - | D | A | F | - | - | T | D | E | D | I | S | E | Y | L | R | V | F | - | L | K | S | G | G | L | R | A | G | L | A | Y | Y | R | S | V | - | - | - | T | V | S | A | E |
| 009 UniRef90\_UPI0016149F5E\_5\_284 | R | W | S | - | Y | R | - | - | - | - | R | - | - | D | K | V | A | V | D | V | Y | A | E | A | Y | - | A | T | P | G | A | L | R | A | G | F | A | Y | Y | R | A | I | - | - | - | P | E | T | I | R |
| 010 UniRef90\_A0A327RPK7\_10\_284 | T | L | A | - | Y | N | P | - | T | S | I | - | - | K | E | E | A | I | A | E | Y | V | R | Q | Y | - | S | A | P | G | A | L | R | A | G | F | E | Y | Y | R | C | L | - | - | - | L | E | D | G | K |
| 011 UniRef90\_UPI00131EC7B5\_30\_311 | R | K | T | - | A | C | P | - | D | V | F | - | - | S | A | E | D | M | A | E | Y | L | R | I | L | - | M | L | N | G | G | L | R | A | G | L | A | Y | Y | R | A | A | - | - | - | A | L | S | A | S |
| 012 UniRef90\_A0A1N6RLL9\_1\_202 | R | F | A | - | L | H | K | - | D | S | I | - | - | T | D | S | D | V | N | H | Y | V | N | S | Y | - | G | T | S | E | K | L | R | A | G | L | E | F | Y | R | A | F | - | - | - | P | A | N | E | K |
| 013 UniRef90\_A0A0N1GDE2\_28\_316 | K | K | A | - | A | T | P | - | D | A | I | - | - | P | A | Y | A | V | D | V | Y | V | D | A | I | V | A | D | P | R | G | L | R | A | S | F | A | Y | Y | R | A | L | - | - | - | D | E | T | I | A |
| 014 UniRef90\_A0A1I6BK09\_10\_296 | H | L | S | - | C | N | P | - | D | A | V | - | - | S | P | E | D | F | E | V | Y | A | R | Q | L | - | S | K | P | G | A | F | R | A | G | I | N | L | Y | A | A | V | - | - | - | W | T | D | A | E |
| 015 UniRef90\_UPI00158A2D6D\_3\_306 | P | L | S | - | D | D | - | - | - | - | - | - | - | - | - | - | D | I | A | V | Y | A | D | A | Y | - | A | A | P | G | A | L | R | A | G | F | E | L | Y | R | A | F | - | - | - | P | Q | D | E | T |
| 016 UniRef90\_UPI00160CFF45\_55\_340 | A | F | G | G | T | S | T | R | S | P | F | - | - | T | E | A | E | I | N | E | Y | A | R | T | Y | - | S | R | P | S | A | L | S | G | G | F | E | L | Y | R | S | L | - | - | - | D | K | D | - | - |
| 017 UniRef90\_A0A2N3KZL1\_30\_325 | E | H | A | - | T | N | R | - | A | A | F | - | - | T | D | E | L | L | D | L | Y | G | A | S | Y | - | A | K | P | H | T | L | H | A | S | F | E | Y | Y | R | A | L | - | - | - | N | E | T | A | A |
| 018 UniRef90\_A0A2M9M9Q1\_18\_305 | L | K | A | - | A | R | P | - | - | - | L | - | - | P | E | S | A | V | D | F | Y | V | E | T | L | A | R | D | P | E | A | L | R | A | S | F | D | Y | Y | R | A | T | - | - | - | D | E | N | I | A |
| 019 UniRef90\_UPI000361D127\_26\_313 | T | K | A | - | A | T | P | - | T | A | I | - | - | P | S | T | A | V | D | V | Y | V | E | A | I | - | R | L | P | G | A | L | R | A | S | F | E | Y | Y | R | A | I | - | - | - | D | E | T | M | T |
| 020 UniRef90\_UPI001473614E\_45\_322 | - | - | - | - | Y | R | R | - | D | A | V | - | - | - | - | - | E | R | E | P | Y | Y | R | A | Y | - | A | D | P | A | R | R | T | A | G | Y | E | Y | Y | R | A | F | - | - | - | A | A | D | A | E |
| 021 UniRef90\_E6WJ64\_19\_301 | R | K | T | - | A | S | P | - | M | V | F | - | - | S | D | A | D | M | A | E | Y | V | R | L | L | - | R | Q | N | G | A | L | R | A | G | L | A | P | Y | R | V | V | - | - | - | T | Q | S | A | A |
| 022 UniRef90\_A0A6L3SWG4\_20\_294 | D | L | A | - | F | D | I | - | E | A | I | - | - | G | A | E | A | V | A | R | Y | V | R | A | F | - | T | R | P | G | A | M | R | A | G | F | E | L | Y | R | A | F | - | - | - | P | A | D | A | E |
| 023 UniRef90\_A0A109IGY4\_11\_301 | R | K | A | - | A | T | P | - | T | T | I | - | - | P | G | Y | A | V | D | V | Y | V | D | A | I | V | A | D | P | R | G | L | R | A | S | F | G | Y | Y | R | A | L | - | - | - | D | E | T | I | A |
| 024 UniRef90\_A0A0Q4UQ58\_27\_305 | A | W | S | - | H | R | K | - | E | A | V | - | - | T | D | E | A | I | A | E | Y | A | R | H | Y | - | A | L | P | G | G | M | K | A | G | F | N | Y | Y | R | A | L | - | - | - | K | D | D | A | A |
| 025 UniRef90\_A0A239MTD5\_1\_271 | A | Y | L | V | G | N | K | - | S | P | F | - | - | T | P | S | D | Y | D | Y | Y | A | H | F | L | - | K | E | P | G | R | T | T | A | W | M | N | V | Y | R | Q | F | - | - | - | R | T | N | V | Q |
| 026 UniRef90\_UPI00049086FA\_57\_348 | T | R | G | G | A | S | E | A | E | P | L | P | V | S | E | R | A | L | A | E | Y | T | R | V | Y | - | A | R | P | Q | V | L | H | A | G | F | E | L | Y | R | A | W | - | - | - | A | Q | D | E | A |
| 027 UniRef90\_A0A517LKB1\_23\_304 | R | L | A | - | Q | K | P | - | S | A | F | - | - | T | N | E | D | V | Q | T | Y | V | R | Q | Y | - | S | R | P | G | A | L | R | A | G | F | L | S | Y | K | A | F | - | - | - | E | T | D | G | E |
| 028 UniRef90\_UPI0004CCFD44\_55\_340 | A | F | G | G | T | S | D | R | S | P | F | - | - | T | D | A | E | I | N | E | Y | A | R | T | Y | - | R | R | P | E | V | L | S | G | G | F | E | L | Y | R | A | L | - | - | - | D | K | D | - | - |
| 029 UniRef90\_A0A285BKA7\_8\_299 | S | K | T | - | A | T | P | - | G | A | I | - | - | P | Q | D | A | V | D | L | Y | V | D | H | L | - | R | D | P | A | A | R | H | A | S | F | E | Y | Y | R | T | L | - | - | - | D | T | T | A | E |
| 030 UniRef90\_A0A4R7C9R7\_33\_310 | A | W | S | - | H | R | K | - | D | A | I | - | - | G | E | D | A | I | T | V | F | A | A | H | Y | - | A | Q | P | G | R | M | T | A | G | F | N | Y | Y | R | A | L | - | - | - | R | E | D | A | P |
| 031 UniRef90\_A0A4D4LDP7\_7\_304 | A | K | T | - | A | T | G | - | N | V | F | - | - | D | G | A | E | I | D | L | Y | T | K | A | L | - | I | R | D | G | G | L | H | A | A | M | E | Y | Y | R | A | V | - | - | - | P | E | S | A | A |
| 032 UniRef90\_J4PG95\_17\_290 | K | W | S | - | H | R | - | - | - | - | R | - | - | D | R | V | A | A | D | V | Y | I | D | A | Y | - | S | A | P | G | A | L | R | S | G | F | A | Y | Y | R | A | I | - | - | - | P | E | T | I | R |
| 033 UniRef90\_A0A1Q8KTW7\_17\_294 | N | E | T | - | Y | N | P | - | A | A | I | - | - | T | D | D | C | V | R | E | W | A | R | C | S | - | A | A | P | G | G | L | R | A | I | F | E | V | Y | R | A | T | - | - | - | F | T | N | V | E |
| 034 UniRef90\_UPI00076E3DF2\_55\_340 | A | F | G | G | A | S | E | Q | S | P | F | - | - | T | D | A | E | I | D | E | Y | A | R | T | Y | - | D | D | P | E | V | L | A | G | G | F | E | L | Y | R | A | L | - | - | - | D | E | D | - | - |
| 035 UniRef90\_A0A542JC81\_20\_314 | I | Q | G | - | G | G | - | - | - | - | V | - | - | P | A | E | A | I | E | Y | Y | I | G | L | V | - | S | D | P | V | A | L | T | G | S | L | G | F | Y | R | A | W | - | - | - | E | T | T | M | G |
| 036 UniRef90\_UPI00156E1DFF\_8\_289 | W | L | S | - | A | V | P | - | G | T | F | - | - | P | P | K | E | V | A | A | V | A | S | S | Y | - | R | G | Y | E | A | L | R | A | G | F | A | H | Y | R | T | L | - | - | - | L | D | D | G | R |
| 037 UniRef90\_UPI00161A7EC4\_21\_297 | - | - | - | - | Q | H | P | - | E | L | I | - | - | - | - | - | D | K | N | T | Y | Y | R | A | Y | - | A | D | P | A | K | R | S | A | G | Y | E | Y | Y | R | A | F | - | - | - | A | E | D | G | T |
| 038 UniRef90\_UPI0012B05B7D\_16\_287 | N | K | A | - | L | N | Y | R | D | T | F | - | - | T | E | L | D | L | D | E | Y | T | R | V | Y | - | S | A | L | G | G | M | R | G | M | L | G | Y | Y | R | S | V | - | - | - | L | E | D | M | E |
| 039 UniRef90\_A0A2I8F4B9\_53\_324 | S | E | A | - | V | N | A | - | R | A | F | - | - | T | N | E | V | E | E | T | Y | A | R | E | Y | - | A | M | P | G | A | L | R | A | G | F | E | Y | Y | R | A | F | - | - | - | P | Q | D | V | K |
| 040 UniRef90\_A0A4D7B7N1\_7\_276 | R | K | T | - | A | N | P | A | A | T | F | - | - | S | A | Q | D | V | D | E | Y | E | R | V | Y | - | R | M | T | G | N | L | R | G | A | L | G | Y | Y | R | A | V | - | - | - | F | E | D | I | E |
| 041 UniRef90\_UPI000E275A28\_18\_308 | - | K | I | - | D | T | P | - | H | P | L | - | - | P | E | Y | A | R | N | F | F | I | E | L | L | K | R | D | R | N | A | L | R | A | S | F | E | Y | Y | R | A | I | - | - | - | D | D | D | L | P |
| 042 UniRef90\_S3CY91\_25\_307 | R | L | C | - | V | N | P | - | A | A | I | - | - | T | P | A | D | L | D | H | Y | V | T | S | F | - | A | Q | P | G | G | M | R | C | G | F | E | L | Y | R | A | F | - | - | - | P | R | D | A | R |
| 043 UniRef90\_A0A316VRQ3\_1\_263 | R | L | S | - | Y | K | P | - | Y | F | L | - | - | T | N | E | E | R | Q | V | Y | Y | E | S | F | - | R | R | S | G | R | M | R | A | G | F | G | V | Y | R | A | F | R | Q | D | D | K | D | V | K |
| 044 UniRef90\_A0A4R6HBQ4\_11\_285 | V | G | T | - | F | D | P | - | T | S | I | - | - | T | E | E | D | I | G | H | Y | A | R | A | A | - | A | A | P | G | G | L | R | G | M | F | A | H | Y | R | A | L | I | - | - | P | Q | D | R | D |
| 045 UniRef90\_UPI0015F81933\_16\_310 | A | K | A | - | L | S | S | - | D | T | F | - | - | D | D | A | E | I | E | H | Y | A | A | S | V | - | A | A | D | G | G | L | R | A | S | L | A | Y | Y | R | D | A | - | - | - | A | E | S | A | Q |
| 046 UniRef90\_UPI0009E804D9\_8\_293 | A | L | S | - | N | - | P | - | G | T | F | - | - | T | D | K | D | L | A | F | Y | A | R | A | Y | - | Q | G | R | E | R | L | S | G | G | F | L | H | Y | R | D | L | - | - | - | L | E | D | G | R |
| 047 UniRef90\_A0A401ZLI5\_4\_287 | R | - | A | - | R | N | S | - | A | A | F | - | - | T | Q | E | D | I | D | E | Y | V | R | C | Y | - | S | G | R | D | A | L | R | A | G | F | E | Y | Y | R | S | F | - | - | - | S | S | N | A | Q |
| 048 UniRef90\_A0A6A6C1N5\_26\_297 | K | I | S | - | F | R | R | - | D | V | F | P | - | A | E | E | D | I | S | F | Y | A | D | E | Y | - | A | K | E | G | V | M | R | A | A | M | G | W | Y | A | A | F | - | - | - | E | T | D | A | E |
| 049 UniRef90\_A0A329J6I4\_30\_314 | S | H | A | - | A | S | M | - | D | A | F | - | - | T | P | K | L | I | D | M | Y | A | K | S | Y | - | A | K | P | Q | T | L | N | A | S | F | E | Y | Y | R | A | L | - | - | - | N | T | S | I | A |
| 050 UniRef90\_A0A1B2HH26\_4\_280 | - | - | - | - | M | E | R | - | G | G | I | - | - | T | D | A | D | R | A | D | L | L | R | T | L | - | T | R | P | D | G | M | R | G | G | F | E | H | Y | A | S | V | - | - | - | A | Q | D | A | V |
| 051 UniRef90\_A0A385B2U0\_45\_333 | Q | Y | L | - | A | V | D | - | G | A | F | - | - | D | D | R | D | Y | E | F | Y | A | R | Y | L | - | R | E | P | G | R | F | K | A | W | M | N | M | Y | Q | A | L | - | - | - | H | T | D | I | A |
| 052 UniRef90\_A0A2E5PJQ7\_5\_284 | N | F | A | - | W | K | T | - | D | A | F | - | - | E | Q | P | V | I | D | E | Y | L | R | T | Y | - | T | Q | P | G | A | M | R | A | G | F | A | Y | Y | R | A | L | - | - | - | P | Q | D | V | A |
| 053 UniRef90\_A0A5C8T429\_14\_295 | A | K | A | - | S | N | P | - | A | A | I | - | - | S | Q | Q | A | M | D | E | Y | V | R | C | Y | - | E | A | P | G | A | W | R | C | A | N | S | Y | Y | R | A | Y | - | - | - | F | D | D | M | A |
| 054 UniRef90\_A0A261TYY2\_43\_315 | S | K | S | - | A | G | V | - | Q | W | I | - | - | E | P | H | A | L | D | E | Y | A | R | C | Y | - | G | G | L | G | G | W | R | A | G | A | S | Y | Y | R | A | L | - | - | - | F | Q | D | M | E |
| 055 UniRef90\_A0A6N7ZB66\_18\_297 | A | R | T | - | T | - | P | - | G | T | F | - | - | T | D | E | E | L | E | F | Y | A | R | S | Y | - | T | G | H | D | R | A | R | G | G | F | E | H | Y | R | A | L | - | - | - | L | E | D | G | R |
| 056 UniRef90\_A0A2T6L0M5\_16\_299 | Q | F | L | - | G | D | Q | - | T | A | F | - | - | A | A | V | D | Y | E | F | Y | A | E | R | L | - | R | R | P | G | R | V | E | A | W | V | K | M | Y | R | S | L | - | - | - | A | V | N | I | Q |
| 057 UniRef90\_G0FSK7\_17\_310 | H | K | A | - | A | R | P | - | - | - | L | - | - | A | E | P | A | I | E | H | Y | V | D | S | I | R | R | D | P | R | A | L | H | A | S | F | G | C | Y | R | A | I | - | - | - | G | D | T | V | A |
| 058 UniRef90\_A0A1Q8IR73\_6\_294 | S | K | T | - | A | N | P | - | A | C | Y | - | - | G | E | E | E | I | A | E | Y | L | R | T | Y | - | C | A | P | G | G | M | R | S | G | L | A | F | Y | R | A | A | - | - | - | A | L | S | V | E |
| 059 UniRef90\_A0A1I2MT44\_20\_306 | A | K | A | - | A | R | P | - | - | - | L | - | - | P | D | H | A | V | Q | H | Y | V | D | T | L | A | A | D | P | A | A | L | R | A | S | F | A | F | Y | R | S | L | - | - | - | D | E | T | I | A |
| 060 UniRef90\_UPI0010F95BB2\_18\_294 | V | G | L | - | W | D | R | - | T | A | I | - | - | L | E | A | D | I | A | V | Y | A | A | A | A | - | A | A | P | G | G | L | R | S | M | F | A | Y | Y | R | T | L | L | - | - | S | H | D | L | Q |
| 061 UniRef90\_A0A4P8X818\_19\_291 | N | E | T | - | Y | D | P | - | - | N | I | - | - | P | N | D | A | M | D | E | Y | I | R | C | Y | - | S | Q | P | G | G | L | R | S | M | F | E | V | Y | R | A | T | - | - | - | L | E | D | G | E |
| 062 UniRef90\_A0A0M3UDU6\_18\_309 | V | K | S | - | L | Q | P | - | W | T | I | - | - | T | A | E | D | I | A | V | Y | A | R | Q | L | - | A | A | P | G | A | L | R | A | A | T | R | Y | Y | Q | C | A | L | - | S | P | E | G | V | A |
| 063 UniRef90\_A0A2P2CCU5\_19\_306 | T | Q | G | - | G | Q | - | - | - | - | V | - | - | P | D | D | A | I | A | Y | Y | V | E | V | L | - | S | R | P | G | V | L | S | G | S | F | G | F | Y | R | S | W | - | - | - | D | E | T | M | A |
| 064 UniRef90\_UPI0009781B11\_10\_287 | T | K | S | - | V | D | W | N | I | A | F | - | - | D | D | R | T | I | D | E | Y | A | S | A | Y | - | A | R | P | G | R | W | T | A | G | L | A | Y | Y | R | S | I | - | - | - | F | E | S | I | A |
| 065 UniRef90\_UPI000561A6EF\_30\_315 | T | K | A | - | A | R | P | - | - | - | L | - | - | P | E | H | A | V | R | Q | Y | T | D | P | I | A | A | G | P | E | A | L | S | A | A | F | G | F | Y | R | E | L | - | - | - | D | R | T | M | A |
| 066 UniRef90\_A0A1Z4J856\_27\_318 | P | F | T | - | Y | N | P | - | A | A | I | - | - | T | E | D | A | M | D | E | Y | V | R | T | Y | - | S | A | P | G | G | M | R | A | G | F | E | Y | Y | R | A | I | - | - | - | P | Q | V | A | K |
| 067 UniRef90\_A0A5C5SVV1\_11\_281 | D | R | A | - | F | D | H | - | G | A | H | - | - | A | L | E | D | I | E | V | F | A | R | A | F | - | A | Q | P | G | R | T | R | G | G | L | E | W | Y | R | A | F | - | - | - | R | K | D | H | A |
| **068 Input\_protein\_seq** | A | L | S | - | N | D | - | - | - | - | - | - | - | - | - | - | D | I | A | V | Y | A | D | A | Y | - | A | A | P | G | A | L | R | A | G | F | E | L | Y | R | A | F | - | - | - | P | Q | D | E | T |
| 069 UniRef90\_A0A1Q7W147\_16\_308 | A | K | T | - | L | S | P | - | G | T | F | - | - | D | D | A | E | R | D | H | Y | A | A | A | L | - | A | T | D | G | A | L | R | A | S | L | A | Y | Y | R | D | A | - | - | - | A | E | S | A | R |
| 070 UniRef90\_A0A0N0TCD7\_1\_288 | S | K | T | - | A | T | P | - | G | A | I | - | - | P | Q | H | A | V | D | V | Y | V | D | S | L | - | R | D | P | A | A | L | R | A | S | F | A | Y | Y | R | S | L | - | - | - | D | E | S | A | R |
| 071 UniRef90\_UPI0004DF415F\_15\_308 | V | K | A | - | L | S | P | - | D | T | F | - | - | D | S | T | E | I | E | H | Y | A | A | A | I | - | A | A | E | G | G | L | R | A | S | L | A | Y | Y | R | D | A | - | - | - | A | E | S | A | R |
| 072 UniRef90\_G7LVZ3\_7\_288 | H | L | A | - | Y | D | T | - | Y | A | V | - | - | T | E | D | D | L | N | E | Y | S | E | R | M | - | S | A | P | G | A | L | R | A | S | F | E | H | Y | R | A | F | - | - | - | P | V | D | A | R |

  
  

|  |  |  |  |  |  |  |  |  |  |  |  |  |  |  |  |  |  |  |  |  |  |  |  |  |  |  |  |  |  |  |  |  |  |  |  |  |  |  |  |  |  |  |  |  |  |  |  |  |  |  |
| --- | --- | --- | --- | --- | --- | --- | --- | --- | --- | --- | --- | --- | --- | --- | --- | --- | --- | --- | --- | --- | --- | --- | --- | --- | --- | --- | --- | --- | --- | --- | --- | --- | --- | --- | --- | --- | --- | --- | --- | --- | --- | --- | --- | --- | --- | --- | --- | --- | --- | --- |
| 001 UniRef90\_L9WLS3\_16\_284 | N | N | A | V | H | A | E | T | - | - | P | - | - | L | E | M | P | V | L | A | L | - | - | G | G | A | A | S | - | - | - | - | F | R | S | L | P | I | - | E | - | D | M | N | A | V | A | T | D | - |
| 002 UniRef90\_A0A0M4FVH2\_24\_312 | Q | N | R | E | L | C | A | M | G | - | K | - | - | L | R | T | P | V | L | A | L | - | - | G | A | D | Q | G | - | - | - | - | - | S | I | A | D | M | V | T | - | P | L | K | A | F | A | E | D | - |
| 003 UniRef90\_UPI0016621A44\_4\_265 | A | V | R | T | R | S | H | H | P | - | - | - | - | L | D | T | P | V | L | A | I | - | - | G | G | R | Y | G | - | - | - | - | - | M | G | T | A | V | T | K | - | C | L | A | Q | L | A | T | D | - |
| 004 UniRef90\_A0A447J1A9\_28\_317 | Q | N | K | R | - | R | A | E | K | - | K | - | - | L | T | M | P | V | L | A | I | - | - | G | A | D | H | A | - | - | - | - | - | T | R | D | A | P | Q | L | - | T | M | Q | G | R | A | V | N | - |
| 005 UniRef90\_A0A1Y6D2S0\_29\_302 | F | V | A | A | Y | A | D | Q | - | - | K | - | - | F | A | M | P | V | L | A | V | - | - | A | G | R | Y | G | - | - | - | - | - | V | A | D | K | L | S | K | - | A | L | Q | A | K | A | D | I | - |
| 006 UniRef90\_A0A0G2FGE6\_47\_303 | E | N | R | D | W | V | A | N | N | G | K | - | - | C | T | V | P | C | M | S | L | - | - | N | G | E | G | S | - | - | - | - | - | F | L | A | N | I | A | E | E | Q | N | L | E | A | Y | Q | A | - |
| 007 UniRef90\_F8JLL5\_12\_309 | K | N | H | E | A | L | Q | Q | Q | - | R | - | - | L | T | V | P | V | L | G | I | - | - | S | S | S | H | G | - | - | - | - | - | S | I | R | D | M | A | A | - | S | I | K | P | W | A | D | H | - |
| 008 UniRef90\_A0A4R2Z7A8\_3\_305 | Q | N | R | K | L | N | S | Q | G | - | K | - | - | L | Q | M | P | L | L | A | V | - | - | S | A | D | Q | G | - | - | - | - | - | S | I | P | D | M | A | V | - | P | L | R | D | F | A | E | N | - |
| 009 UniRef90\_UPI0016149F5E\_5\_284 | Q | N | L | E | - | R | A | K | R | - | S | - | - | L | A | M | P | V | L | A | I | - | - | G | A | D | H | A | - | - | - | - | - | T | N | D | A | P | L | L | - | T | M | Q | G | K | A | S | I | - |
| 010 UniRef90\_A0A327RPK7\_10\_284 | H | N | Q | K | Y | K | E | Q | - | - | K | - | - | L | D | I | P | V | L | A | Y | - | - | G | G | E | T | S | - | - | - | - | - | T | G | D | N | F | R | K | - | S | L | S | L | I | A | N | H | - |
| 011 UniRef90\_UPI00131EC7B5\_30\_311 | Q | N | R | E | R | V | K | K | G | - | K | - | - | L | A | M | P | I | L | A | I | - | - | S | A | E | Q | G | - | - | - | - | - | S | I | A | D | M | A | T | - | P | L | R | P | F | A | E | D | - |
| 012 UniRef90\_A0A1N6RLL9\_1\_202 | F | N | A | A | K | R | N | V | - | - | - | - | - | L | N | N | P | I | V | L | A | - | - | G | G | D | K | A | A | G | P | - | - | T | L | L | K | M | A | E | - | S | L | R | K | Y | G | C | T | N |
| 013 UniRef90\_A0A0N1GDE2\_28\_316 | Q | N | E | Q | - | R | K | K | T | - | R | - | - | L | T | L | P | V | L | A | I | - | - | G | G | A | R | Y | - | - | - | - | - | T | G | V | M | V | A | E | - | T | M | R | L | A | A | D | D | - |
| 014 UniRef90\_A0A1I6BK09\_10\_296 | H | N | R | A | S | A | Q | R | - | - | K | - | - | L | P | M | P | V | L | A | V | - | - | G | G | A | C | S | - | - | - | - | - | G | G | A | Y | I | A | Q | - | A | F | A | V | A | A | E | D | - |
| 015 UniRef90\_UPI00158A2D6D\_3\_306 | Q | F | K | A | F | M | K | H | - | - | Q | - | - | L | P | M | P | V | L | A | L | - | - | A | G | D | K | S | - | - | - | - | - | N | G | T | R | E | L | D | - | M | A | R | E | L | A | V | D | - |
| 016 UniRef90\_UPI00160CFF45\_55\_340 | - | - | - | - | V | S | D | A | K | A | A | K | P | V | R | V | P | A | L | V | M | S | A | Q | G | - | - | - | - | Q | I | K | - | - | - | - | - | - | - | - | - | A | I | R | A | T | V | S | P | R |
| 017 UniRef90\_A0A2N3KZL1\_30\_325 | R | N | K | S | L | - | A | K | K | - | K | - | - | L | S | M | P | V | L | A | I | G | G | G | G | H | G | G | - | - | - | - | - | M | G | Q | L | E | A | D | - | Q | L | G | E | Y | G | T | H | - |
| 018 UniRef90\_A0A2M9M9Q1\_18\_305 | Q | N | G | R | - | R | G | R | R | - | P | - | - | L | P | M | P | V | L | G | V | - | - | A | G | A | R | G | - | - | - | - | - | Q | G | D | R | L | A | A | - | L | L | G | P | V | A | E | R | - |
| 019 UniRef90\_UPI000361D127\_26\_313 | Q | T | V | L | - | R | K | Q | N | - | K | - | - | L | T | I | P | V | L | A | V | - | - | A | G | D | A | L | - | - | - | - | - | G | G | E | N | V | E | R | - | E | V | R | S | L | A | D | D | - |
| 020 UniRef90\_UPI001473614E\_45\_322 | N | N | T | A | N | A | A | K | R | - | - | - | - | L | P | M | P | V | L | G | I | - | - | G | G | Q | Y | S | - | - | - | - | - | F | G | P | G | V | A | D | - | S | F | R | Q | V | A | D | D | - |
| 021 UniRef90\_E6WJ64\_19\_301 | Q | N | R | A | L | R | E | Q | G | - | K | - | - | L | T | L | P | L | L | A | I | - | - | S | A | D | Q | G | - | - | - | - | - | S | I | P | D | M | A | T | - | P | L | R | Q | F | A | D | D | - |
| 022 UniRef90\_A0A6L3SWG4\_20\_294 | R | N | R | A | D | L | E | R | E | G | K | - | - | L | P | M | P | V | L | A | V | - | - | A | G | A | H | S | - | - | - | - | - | T | F | A | R | S | M | D | G | M | M | R | E | V | A | Q | D | - |
| 023 UniRef90\_A0A109IGY4\_11\_301 | Q | N | E | R | - | R | A | K | T | - | R | - | - | L | T | L | P | V | L | A | I | - | - | G | G | A | L | Y | - | - | - | - | - | S | G | A | L | V | A | R | - | T | M | R | L | A | A | A | D | - |
| 024 UniRef90\_A0A0Q4UQ58\_27\_305 | L | A | A | S | F | A | G | R | - | - | R | - | - | L | A | M | P | V | L | A | I | - | - | T | G | R | Y | G | - | - | - | - | - | V | G | S | R | L | S | D | - | A | L | S | R | E | S | D | R | - |
| 025 UniRef90\_A0A239MTD5\_1\_271 | Q | N | K | E | F | L | A | R | G | - | K | - | - | L | K | M | P | I | L | A | I | - | - | G | A | Q | D | S | - | - | - | - | - | F | G | G | I | V | V | D | - | Q | W | R | D | Y | A | V | N | - |
| 026 UniRef90\_UPI00049086FA\_57\_348 | E | N | A | R | L | Q | D | T | - | - | - | - | P | L | T | I | P | V | R | L | L | A | Q | D | G | F | A | S | V | M | L | P | - | - | - | - | - | - | - | - | - | A | V | R | A | A | A | P | A | - |
| 027 UniRef90\_A0A517LKB1\_23\_304 | D | N | Q | T | W | R | E | K | N | G | K | - | - | V | K | V | R | N | M | V | L | - | - | S | G | E | G | S | - | - | - | - | - | F | L | K | G | G | A | E | S | I | A | R | E | F | Y | E | N | - |
| 028 UniRef90\_UPI0004CCFD44\_55\_340 | - | - | - | - | V | R | D | T | T | A | A | A | P | I | R | V | P | T | L | L | M | T | A | Q | G | - | - | - | - | Q | L | A | - | - | - | - | - | - | - | - | - | P | V | R | A | T | A | A | Q | R |
| 029 UniRef90\_A0A285BKA7\_8\_299 | H | I | Q | R | W | R | D | Q | G | - | P | - | - | L | A | I | P | V | L | A | I | - | - | G | G | Q | Y | S | - | - | - | - | - | T | G | T | M | P | A | D | - | T | M | R | L | V | A | T | D | - |
| 030 UniRef90\_A0A4R7C9R7\_33\_310 | L | A | A | E | L | R | G | R | - | - | R | - | - | L | P | M | P | V | M | A | V | - | - | A | G | R | Q | S | - | - | - | - | - | G | G | S | R | L | A | D | - | S | L | R | A | E | A | P | S | - |
| 031 UniRef90\_A0A4D4LDP7\_7\_304 | R | N | R | R | L | L | A | D | R | - | E | - | - | L | T | M | P | V | L | A | I | - | - | D | S | E | H | G | - | - | - | - | - | S | I | P | D | M | A | A | - | P | L | T | P | F | V | P | H | - |
| 032 UniRef90\_J4PG95\_17\_290 | Q | N | Q | E | - | R | S | K | T | - | R | - | - | L | R | M | P | T | L | A | I | - | - | G | T | E | H | A | - | - | - | - | - | T | R | D | A | P | L | I | - | T | M | R | D | N | A | T | D | - |
| 033 UniRef90\_A0A1Q8KTW7\_17\_294 | L | N | T | E | W | A | R | T | - | - | P | - | - | L | P | M | P | V | L | T | I | - | - | G | S | S | H | F | - | - | - | - | - | I | G | E | E | S | R | R | - | Q | M | E | R | T | A | T | D | - |
| 034 UniRef90\_UPI00076E3DF2\_55\_340 | - | - | - | - | V | R | D | T | G | E | A | A | P | V | D | V | P | T | L | L | M | T | A | E | G | - | - | - | - | Q | L | D | - | - | - | - | - | - | - | - | - | T | V | R | A | T | V | D | S | H |
| 035 UniRef90\_A0A542JC81\_20\_314 | Q | N | A | E | - | R | A | K | S | - | K | - | - | L | P | M | P | V | L | A | V | - | - | G | G | E | R | S | - | - | - | - | - | Y | G | H | H | V | E | E | - | N | M | R | L | L | A | D | D | - |
| 036 UniRef90\_UPI00156E1DFF\_8\_289 | T | N | R | T | W | R | E | T | - | - | G | G | I | L | P | M | P | V | L | A | V | - | - | G | G | E | H | S | - | - | - | - | - | T | G | T | Q | L | A | D | - | S | L | N | T | V | A | Q | R | - |
| 037 UniRef90\_UPI00161A7EC4\_21\_297 | N | N | T | A | N | A | S | R | R | - | - | - | - | L | T | Q | P | V | L | A | L | - | - | G | G | A | A | S | - | - | - | - | - | F | G | P | G | V | A | D | - | S | F | R | L | V | A | D | D | - |
| 038 UniRef90\_UPI0012B05B7D\_16\_287 | Q | N | R | V | F | - | G | Q | Q | - | L | - | - | L | K | I | P | V | L | A | L | - | - | G | G | D | K | G | - | - | - | - | - | S | A | P | D | L | H | D | - | R | I | K | Q | L | A | I | D | - |
| 039 UniRef90\_A0A2I8F4B9\_53\_324 | A | N | Q | A | F | S | A | T | - | - | K | - | - | L | T | M | P | V | L | G | I | - | - | G | G | A | G | S | - | - | - | - | - | F | G | P | I | I | G | D | - | H | L | R | H | V | A | T | N | - |
| 040 UniRef90\_A0A4D7B7N1\_7\_276 | Q | N | K | H | L | - | A | N | I | - | R | - | - | L | K | T | P | I | L | A | L | - | - | G | G | D | V | G | - | - | - | - | - | M | S | P | N | I | Y | D | - | A | M | K | P | L | G | E | N | - |
| 041 UniRef90\_UPI000E275A28\_18\_308 | Q | N | Y | A | - | R | K | K | T | - | K | - | - | L | P | M | P | V | L | G | F | - | - | A | G | A | L | A | - | - | - | - | - | C | G | E | L | V | E | E | - | Q | L | R | R | V | A | T | D | - |
| 042 UniRef90\_S3CY91\_25\_307 | D | N | R | R | M | L | E | E | R | G | R | - | - | S | K | V | P | A | C | S | L | - | - | S | G | E | G | S | - | - | - | - | - | L | L | L | Q | V | A | E | E | Q | T | R | E | F | Y | E | S | - |
| 043 UniRef90\_A0A316VRQ3\_1\_263 | E | N | I | A | S | K | G | K | - | - | - | - | - | L | S | I | P | I | L | A | T | - | - | G | G | S | E | S | V | - | - | - | - | F | C | K | Y | I | E | Q | - | I | G | K | E | I | G | G | N | - |
| 044 UniRef90\_A0A4R6HBQ4\_11\_285 | D | N | L | R | L | G | A | S | - | - | P | - | - | L | L | C | P | V | L | A | V | - | - | G | G | D | H | G | - | - | - | - | - | Y | G | E | A | A | L | N | - | T | M | K | R | V | A | T | E | - |
| 045 UniRef90\_UPI0015F81933\_16\_310 | K | N | H | E | A | L | E | R | Q | - | R | - | - | L | T | V | P | I | L | G | I | - | - | S | S | S | H | G | - | - | - | - | - | S | I | P | D | M | A | A | - | S | I | R | P | W | A | D | H | - |
| 046 UniRef90\_UPI0009E804D9\_8\_293 | E | N | R | T | L | L | Q | N | - | - | R | P | - | L | T | M | P | V | L | A | I | - | - | G | G | G | D | R | - | - | - | - | - | M | G | T | G | V | A | D | - | A | L | R | P | H | A | P | R | - |
| 047 UniRef90\_A0A401ZLI5\_4\_287 | L | F | K | E | Y | S | K | E | - | - | K | - | - | L | R | I | P | T | L | A | L | - | - | G | G | E | Y | S | - | - | - | - | G | A | G | W | P | F | - | Y | - | S | L | A | Q | L | A | E | N | - |
| 048 UniRef90\_A0A6A6C1N5\_26\_297 | E | N | L | E | W | V | R | R | E | G | K | - | - | C | A | V | P | T | M | V | L | - | - | S | G | E | R | S | - | - | - | - | - | W | Q | R | E | E | A | E | G | M | V | R | E | V | T | E | D | G |
| 049 UniRef90\_A0A329J6I4\_30\_314 | E | N | A | E | L | - | S | K | T | - | S | - | - | L | N | M | P | V | L | A | I | G | G | G | G | R | N | G | - | - | - | - | - | L | G | Q | F | Q | I | D | - | Q | T | K | K | Y | A | T | N | - |
| 050 UniRef90\_A0A1B2HH26\_4\_280 | N | A | R | A | G | R | - | - | - | - | K | - | - | V | E | V | P | V | L | V | L | - | - | H | G | E | H | G | L | - | - | - | - | P | A | D | V | L | L | T | - | G | A | R | E | A | A | T | D | - |
| 051 UniRef90\_A0A385B2U0\_45\_333 | Q | N | E | K | F | R | E | A | G | - | L | - | - | L | Q | M | P | I | L | A | V | - | - | G | G | E | E | A | - | - | - | - | - | L | S | S | A | V | G | T | - | Q | W | Q | G | Y | A | A | N | - |
| 052 UniRef90\_A0A2E5PJQ7\_5\_284 | A | N | R | A | L | L | E | T | G | F | R | - | - | L | P | M | P | V | L | A | M | - | - | G | G | A | M | W | - | E | A | R | G | R | G | E | E | P | E | K | - | S | M | R | R | V | A | E | N | - |
| 053 UniRef90\_A0A5C8T429\_14\_295 | Q | N | R | E | H | - | A | R | R | - | K | - | - | I | R | T | P | I | L | T | V | - | - | G | G | D | V | G | - | - | - | - | - | L | G | S | M | M | E | P | - | M | M | R | P | V | A | E | D | - |
| 054 UniRef90\_A0A261TYY2\_43\_315 | Q | N | R | A | H | - | A | R | A | - | R | - | - | L | T | M | P | V | L | A | M | - | - | G | G | E | A | A | - | - | - | - | - | L | G | G | M | M | E | T | - | M | L | S | R | V | A | D | D | - |
| 055 UniRef90\_A0A6N7ZB66\_18\_297 | E | N | T | A | V | L | E | K | - | - | G | K | - | I | T | T | P | L | L | L | I | - | - | G | G | K | D | S | - | - | - | - | - | T | G | D | Q | T | I | K | - | A | L | A | P | H | A | E | K | - |
| 056 UniRef90\_A0A2T6L0M5\_16\_299 | Q | N | K | Q | L | Q | A | A | G | - | P | - | - | I | G | M | P | I | L | A | V | - | - | G | G | E | K | S | - | - | - | - | - | L | G | K | S | I | G | T | - | Q | L | E | R | Y | A | T | Q | - |
| 057 UniRef90\_G0FSK7\_17\_310 | Q | N | A | E | - | R | V | K | N | - | R | - | - | L | D | L | P | I | L | T | I | - | - | A | G | E | R | S | - | - | - | - | - | T | G | P | L | V | E | R | - | T | M | I | P | A | A | A | D | - |
| 058 UniRef90\_A0A1Q8IR73\_6\_294 | Q | N | R | A | L | S | R | D | R | - | K | - | - | L | A | M | P | V | L | G | L | - | - | S | A | D | Q | G | - | - | - | - | - | S | I | P | D | M | S | A | - | A | L | R | P | F | A | N | E | - |
| 059 UniRef90\_A0A1I2MT44\_20\_306 | Q | N | E | Q | - | R | A | A | R | - | R | - | - | L | T | L | P | V | L | A | V | - | - | G | G | A | Q | N | - | - | - | - | - | L | G | A | A | V | A | A | - | T | M | D | R | A | A | D | D | - |
| 060 UniRef90\_UPI0010F95BB2\_18\_294 | D | N | L | A | L | G | R | R | - | - | K | - | - | L | S | L | P | V | L | A | I | - | - | G | A | E | H | G | - | - | - | - | - | Y | R | A | A | S | L | S | - | T | M | E | R | V | A | T | N | - |
| 061 UniRef90\_A0A4P8X818\_19\_291 | W | N | K | K | A | A | E | T | - | - | K | - | - | L | P | M | P | V | L | A | V | - | - | G | S | K | H | F | - | - | - | - | - | I | A | D | E | V | Q | R | - | Q | M | E | R | V | A | S | N | - |
| 062 UniRef90\_A0A0M3UDU6\_18\_309 | A | N | R | L | - | R | A | E | K | - | P | - | - | L | D | I | P | V | L | A | L | - | - | G | A | D | R | G | - | - | - | - | - | V | G | D | H | I | V | K | - | A | L | Q | A | L | A | T | N | - |
| 063 UniRef90\_A0A2P2CCU5\_19\_306 | Q | N | G | E | - | R | A | T | R | - | M | - | - | L | T | M | P | V | L | A | I | - | - | G | G | E | T | S | - | - | - | - | - | W | G | G | A | V | G | G | - | A | M | S | A | L | A | G | H | - |
| 064 UniRef90\_UPI0009781B11\_10\_287 | Q | N | K | V | S | - | S | R | I | - | P | - | - | L | P | M | P | V | L | A | I | - | - | G | G | D | F | G | - | - | - | - | - | V | G | E | N | M | H | R | - | S | I | V | G | A | A | L | D | - |
| 065 UniRef90\_UPI000561A6EF\_30\_315 | Q | N | Q | R | - | R | K | E | R | - | P | - | - | L | T | I | P | V | L | T | I | - | - | A | G | A | D | S | - | - | - | - | - | L | G | G | A | V | G | G | - | A | M | R | L | A | A | E | D | - |
| 066 UniRef90\_A0A1Z4J856\_27\_318 | Q | T | K | E | N | M | K | T | - | - | K | - | - | L | R | I | P | V | L | A | L | - | - | A | G | E | H | S | L | G | N | P | A | L | G | D | P | A | R | T | - | S | I | Q | L | L | A | E | N | - |
| 067 UniRef90\_A0A5C5SVV1\_11\_281 | D | A | L | A | W | K | R | E | R | - | - | - | - | L | T | M | P | V | L | G | L | - | - | G | G | D | G | R | - | - | - | - | - | W | G | P | Q | I | V | A | - | I | L | Q | E | F | A | E | D | - |
| **068 Input\_protein\_seq** | R | F | K | A | F | M | K | H | - | - | K | - | - | L | P | M | P | V | L | A | L | - | - | A | G | D | K | S | - | - | - | - | - | N | G | A | K | E | L | D | - | M | A | R | E | L | A | L | D | - |
| 069 UniRef90\_A0A1Q7W147\_16\_308 | K | N | H | Q | A | L | R | R | R | - | H | - | - | L | T | L | P | V | L | G | I | - | - | S | S | S | H | G | - | - | - | - | - | S | I | P | D | M | A | A | - | S | I | S | P | W | A | K | N | - |
| 070 UniRef90\_A0A0N0TCD7\_1\_288 | Q | A | Q | R | W | R | D | E | G | - | P | - | - | L | S | V | P | V | L | A | I | - | - | G | G | E | H | S | - | - | - | - | - | T | G | T | M | P | E | E | - | V | M | R | M | V | A | T | D | - |
| 071 UniRef90\_UPI0004DF415F\_15\_308 | K | N | H | E | A | L | E | R | Q | - | H | - | - | L | T | V | P | V | M | G | I | - | - | S | G | S | H | G | - | - | - | - | - | S | T | P | D | M | A | A | - | S | L | G | P | W | A | K | N | - |
| 072 UniRef90\_G7LVZ3\_7\_288 | N | N | Q | E | H | A | R | T | - | - | K | - | - | L | P | M | P | V | L | G | I | - | - | G | G | E | Y | C | - | - | - | - | - | M | G | D | Q | V | G | H | - | I | M | R | P | L | A | D | N | - |

  
  

|  |  |  |  |  |  |  |  |  |  |  |  |  |  |  |  |  |  |  |  |  |  |  |  |  |  |  |  |  |  |  |  |  |  |  |  |  |  |  |  |  |
| --- | --- | --- | --- | --- | --- | --- | --- | --- | --- | --- | --- | --- | --- | --- | --- | --- | --- | --- | --- | --- | --- | --- | --- | --- | --- | --- | --- | --- | --- | --- | --- | --- | --- | --- | --- | --- | --- | --- | --- | --- |
| 001 UniRef90\_L9WLS3\_16\_284 | - | - | - | - | V | E | G | E | V | L | E | R | A | G | H | W | I | P | E | E | R | P | E | Y | F | V | E | R | V | E | S | F | L | E | S | A | - | - | - | - |
| 002 UniRef90\_A0A0M4FVH2\_24\_312 | - | - | - | - | V | Q | G | G | T | I | S | F | C | G | H | F | L | P | E | E | Q | P | E | A | V | S | R | E | L | K | A | F | F | - | - | - | - | - | - | - |
| 003 UniRef90\_UPI0016621A44\_4\_265 | - | - | - | - | V | Q | D | L | Q | V | A | Q | A | G | H | Y | P | A | E | Q | D | P | A | T | V | N | R | E | L | T | S | F | L | - | - | - | - | - | - | - |
| 004 UniRef90\_A0A447J1A9\_28\_317 | - | - | - | - | L | Q | G | A | I | L | S | E | C | G | H | F | V | T | E | E | C | P | E | Q | L | M | D | V | V | L | P | F | L | R | - | - | - | - | - | - |
| 005 UniRef90\_A0A1Y6D2S0\_29\_302 | - | - | - | - | I | K | S | A | I | A | E | D | S | G | H | F | V | P | E | E | A | P | A | F | L | V | E | Q | L | S | S | F | L | A | P | A | - | - | - | - |
| 006 UniRef90\_A0A0G2FGE6\_47\_303 | - | - | - | - | T | E | T | A | T | I | P | G | A | G | H | W | C | A | E | E | N | P | G | A | F | T | Q | T | V | L | A | - | - | - | - | - | - | - | - | - |
| 007 UniRef90\_F8JLL5\_12\_309 | - | - | - | - | A | T | G | I | V | V | P | D | A | G | H | F | I | P | D | E | Q | P | D | A | V | A | A | A | L | T | D | F | V | T | E | G | - | - | - | - |
| 008 UniRef90\_A0A4R2Z7A8\_3\_305 | - | - | - | - | V | T | G | I | I | I | A | N | S | G | H | F | I | P | E | E | Q | P | E | A | L | A | R | E | L | H | T | F | F | - | - | - | - | - | - | - |
| 009 UniRef90\_UPI0016149F5E\_5\_284 | - | - | - | - | L | Q | G | A | I | V | T | E | C | G | H | F | I | M | E | E | Q | P | E | A | F | I | A | H | L | L | P | F | L | A | - | - | - | - | - | - |
| 010 UniRef90\_A0A327RPK7\_10\_284 | - | - | - | - | V | E | G | G | S | I | P | E | C | G | H | Y | I | A | E | E | Q | P | E | F | L | I | K | V | L | T | E | F | L | - | - | - | - | - | - | - |
| 011 UniRef90\_UPI00131EC7B5\_30\_311 | - | - | - | - | V | A | G | V | L | V | T | H | C | G | H | F | L | P | E | E | Q | P | Q | V | I | A | R | E | L | S | G | F | F | - | - | - | - | - | - | - |
| 012 UniRef90\_A0A1N6RLL9\_1\_202 | - | - | - | - | V | T | T | K | I | I | Q | N | S | G | H | F | V | S | E | E | Q | P | E | I | V | I | G | L | I | - | - | - | - | - | - | - | - | - | - | - |
| 013 UniRef90\_A0A0N1GDE2\_28\_316 | - | - | - | - | V | T | G | V | V | L | D | D | C | G | H | Y | A | A | E | E | Q | P | A | R | F | T | E | I | L | E | D | F | L | - | - | - | - | - | - | - |
| 014 UniRef90\_A0A1I6BK09\_10\_296 | - | - | - | - | V | R | P | L | V | L | E | G | A | G | H | W | L | A | E | E | Q | P | A | A | L | A | T | H | F | L | D | F | F | G | E | A | D | - | - | - |
| 015 UniRef90\_UPI00158A2D6D\_3\_306 | - | - | - | - | V | R | G | A | V | A | P | N | T | G | H | W | L | P | D | E | N | P | A | F | L | T | R | Q | L | I | A | F | F | R | E | A | V | P | G | R |
| 016 UniRef90\_UPI00160CFF45\_55\_340 | L | T | N | I | V | R | A | V | D | V | P | K | A | G | H | W | L | I | E | E | N | P | Q | F | V | T | K | E | L | L | R | F | L | - | - | - | - | - | - | - |
| 017 UniRef90\_A0A2N3KZL1\_30\_325 | - | - | - | - | V | K | G | L | V | I | P | D | C | G | H | W | L | P | E | E | C | A | K | P | L | N | D | A | V | L | D | F | L | - | - | - | - | - | - | - |
| 018 UniRef90\_A0A2M9M9Q1\_18\_305 | - | - | - | - | F | T | G | A | V | L | P | D | C | G | H | Y | V | P | E | E | C | P | D | A | L | L | D | H | L | L | P | F | L | - | - | - | - | - | - | - |
| 019 UniRef90\_UPI000361D127\_26\_313 | - | - | - | - | V | T | G | V | V | I | A | N | C | G | H | F | V | A | E | E | A | P | E | A | F | L | N | A | L | R | P | F | - | - | - | - | - | - | - | - |
| 020 UniRef90\_UPI001473614E\_45\_322 | - | - | - | - | V | R | T | V | V | A | P | D | S | G | H | F | V | P | E | E | N | P | R | F | M | A | - | - | - | - | - | - | - | - | - | - | - | - | - | - |
| 021 UniRef90\_E6WJ64\_19\_301 | - | - | - | - | V | T | G | I | T | L | A | H | S | G | H | F | I | P | D | E | Q | P | L | A | L | A | A | A | L | A | R | F | F | R | - | - | - | - | - | - |
| 022 UniRef90\_A0A6L3SWG4\_20\_294 | - | - | - | - | V | T | F | Q | V | I | E | Q | A | N | H | W | I | P | E | E | N | A | E | A | L | A | A | A | V | R | S | F | L | S | E | - | - | - | - | - |
| 023 UniRef90\_A0A109IGY4\_11\_301 | - | - | - | - | V | T | E | A | I | I | E | D | C | G | H | Y | A | A | E | E | Q | P | A | R | F | T | G | I | L | A | D | F | L | - | - | - | - | - | - | - |
| 024 UniRef90\_A0A0Q4UQ58\_27\_305 | - | - | - | - | L | E | T | V | I | V | E | E | S | G | H | F | V | A | E | E | A | P | - | - | - | - | - | - | - | E | T | F | - | - | - | - | - | - | - | - |
| 025 UniRef90\_A0A239MTD5\_1\_271 | - | - | - | - | V | D | G | R | V | L | K | N | S | G | H | F | V | T | E | E | K | P | Q | E | V | T | A | M | L | Q | S | F | L | Q | E | - | - | - | - | - |
| 026 UniRef90\_UPI00049086FA\_57\_348 | - | - | - | - | A | T | G | A | D | V | P | G | A | G | H | W | M | L | D | Q | H | P | D | Q | V | V | A | E | I | N | A | F | Y | P | A | A | A | - | - | - |
| 027 UniRef90\_A0A517LKB1\_23\_304 | - | - | - | - | P | V | V | G | L | V | E | G | S | G | H | W | L | A | E | E | N | P | E | G | F | A | K | E | V | L | K | F | V | - | - | - | - | - | - | - |
| 028 UniRef90\_UPI0004CCFD44\_55\_340 | M | T | N | I | V | R | A | V | D | V | P | N | A | G | H | W | L | V | E | E | N | P | Q | F | V | T | T | E | L | L | R | F | L | - | - | - | - | - | - | - |
| 029 UniRef90\_A0A285BKA7\_8\_299 | - | - | - | - | V | T | E | L | V | I | P | Q | A | G | H | F | L | P | E | E | V | P | Q | E | L | T | K | H | L | L | D | F | L | R | - | - | - | - | - | - |
| 030 UniRef90\_A0A4R7C9R7\_33\_310 | - | - | - | - | L | T | S | V | I | I | D | D | C | G | H | F | V | A | E | E | A | P | - | - | - | - | - | - | - | D | P | F | - | - | - | - | - | - | - | - |
| 031 UniRef90\_A0A4D4LDP7\_7\_304 | - | - | - | - | V | R | R | A | S | I | A | G | A | G | H | F | I | P | D | E | Q | P | A | A | L | A | Q | I | L | L | R | F | A | - | - | - | - | - | - | - |
| 032 UniRef90\_J4PG95\_17\_290 | - | - | - | - | L | T | G | V | I | I | A | D | C | G | H | F | V | T | E | E | C | H | E | A | F | S | E | H | L | L | A | F | L | - | - | - | - | - | - | - |
| 033 UniRef90\_A0A1Q8KTW7\_17\_294 | - | - | - | - | V | R | Y | V | E | I | A | E | C | G | H | S | L | A | L | E | R | P | A | E | L | A | R | L | L | G | E | F | F | A | D | - | - | - | - | - |
| 034 UniRef90\_UPI00076E3DF2\_55\_340 | I | T | N | V | V | R | A | V | D | V | P | N | A | G | H | W | L | I | E | E | N | P | E | Y | V | A | E | E | L | L | L | F | L | - | - | - | - | - | - | - |
| 035 UniRef90\_A0A542JC81\_20\_314 | - | - | - | - | V | E | G | V | V | I | A | G | A | G | H | W | V | A | E | E | A | P | E | Q | L | L | A | A | L | I | S | F | L | E | P | F | R | A | A | A |
| 036 UniRef90\_UPI00156E1DFF\_8\_289 | - | - | - | - | L | A | G | V | V | I | P | G | S | G | H | F | V | P | E | E | C | P | D | A | F | A | R | E | L | L | H | F | L | - | - | - | - | - | - | - |
| 037 UniRef90\_UPI00161A7EC4\_21\_297 | - | - | - | - | V | R | P | V | V | V | P | D | S | G | H | Y | I | A | E | E | N | S | S | Y | L | G | - | - | - | - | - | - | - | - | - | - | - | - | - | - |
| 038 UniRef90\_UPI0012B05B7D\_16\_287 | - | - | - | - | V | Y | G | G | H | I | K | D | S | G | H | Y | I | P | E | E | Q | P | L | A | L | V | E | E | I | I | E | F | D | R | - | - | - | - | - | - |
| 039 UniRef90\_A0A2I8F4B9\_53\_324 | - | - | - | - | V | E | A | I | N | V | Q | G | S | G | H | W | V | A | E | E | Q | P | A | V | V | T | G | A | L | L | R | F | L | - | - | - | - | - | - | - |
| 040 UniRef90\_A0A4D7B7N1\_7\_276 | - | - | - | - | V | Q | G | G | I | V | A | D | C | G | H | Y | M | P | E | E | Q | P | E | V | I | A | E | R | M | L | A | F | F | G | E | - | - | - | - | - |
| 041 UniRef90\_UPI000E275A28\_18\_308 | - | - | - | - | V | Q | C | T | I | I | P | D | C | G | H | F | I | P | E | E | C | P | E | D | L | L | A | V | L | K | P | F | F | E | - | - | - | - | - | - |
| 042 UniRef90\_S3CY91\_25\_307 | - | - | - | - | V | E | V | A | T | V | E | G | S | G | H | W | C | A | E | E | N | P | G | G | L | V | R | K | V | M | E | F | V | - | - | - | - | - | - | - |
| 043 UniRef90\_A0A316VRQ3\_1\_263 | - | - | - | - | M | Q | F | K | P | V | E | D | A | S | H | W | V | P | E | E | N | P | V | G | L | A | N | L | T | V | K | F | L | Q | E | - | - | - | - | - |
| 044 UniRef90\_A0A4R6HBQ4\_11\_285 | - | - | - | - | P | E | G | A | I | I | K | D | C | G | H | Y | V | P | E | E | R | P | R | E | L | V | E | T | M | V | S | F | F | A | - | - | - | - | - | - |
| 045 UniRef90\_UPI0015F81933\_16\_310 | - | - | - | - | A | T | G | T | V | V | P | D | A | G | H | F | I | P | D | E | Q | P | D | A | V | A | A | A | L | T | D | F | I | T | E | - | - | - | - | - |
| 046 UniRef90\_UPI0009E804D9\_8\_293 | - | - | - | - | L | A | G | L | V | A | P | - | T | G | H | F | V | V | E | E | D | P | T | W | F | L | Q | A | L | D | G | F | L | - | - | - | - | - | - | - |
| 047 UniRef90\_A0A401ZLI5\_4\_287 | - | - | - | - | V | S | S | G | I | I | S | Q | C | G | H | Y | I | A | E | E | Q | P | E | E | L | L | Q | R | L | N | A | F | F | S | E | - | - | - | - | - |
| 048 UniRef90\_A0A6A6C1N5\_26\_297 | - | - | - | V | V | E | V | G | V | V | E | G | V | G | H | Y | L | A | E | E | G | P | E | E | F | V | E | V | V | L | E | F | L | - | - | - | - | - | - | - |
| 049 UniRef90\_A0A329J6I4\_30\_314 | - | - | - | - | V | K | G | E | I | L | P | G | C | G | H | W | L | P | E | E | C | A | A | K | L | N | A | V | V | V | D | F | L | - | - | - | - | - | - | - |
| 050 UniRef90\_A0A1B2HH26\_4\_280 | - | - | - | - | V | R | A | E | I | V | P | D | A | A | H | T | F | A | A | D | N | P | S | A | T | A | D | V | L | T | H | F | F | R | - | - | - | - | - | - |
| 051 UniRef90\_A0A385B2U0\_45\_333 | - | - | - | - | V | E | T | Q | V | M | T | D | T | G | H | W | L | T | E | E | R | P | Q | E | L | T | T | M | L | L | K | F | L | R | - | - | - | - | - | - |
| 052 UniRef90\_A0A2E5PJQ7\_5\_284 | - | - | - | - | V | T | G | M | V | A | A | E | S | G | H | F | I | P | E | E | Q | P | D | F | V | A | E | K | L | L | E | F | F | K | D | - | - | - | - | - |
| 053 UniRef90\_A0A5C8T429\_14\_295 | - | - | - | - | V | T | G | L | V | V | P | D | C | G | H | Y | V | P | E | E | A | P | N | F | L | A | E | H | L | I | N | F | L | - | - | - | - | - | - | - |
| 054 UniRef90\_A0A261TYY2\_43\_315 | - | - | - | - | V | T | G | V | I | A | P | - | C | G | H | Y | V | P | E | E | A | P | E | F | L | V | A | Q | L | R | A | F | - | - | - | - | - | - | - | - |
| 055 UniRef90\_A0A6N7ZB66\_18\_297 | - | - | - | - | L | T | S | Y | V | A | D | - | T | S | H | F | V | A | E | E | A | P | E | W | F | V | E | K | L | T | D | F | L | A | N | A | G | - | - | - |
| 056 UniRef90\_A0A2T6L0M5\_16\_299 | - | - | - | - | V | D | T | R | V | L | A | G | A | G | H | W | V | T | E | E | R | P | H | E | V | T | E | L | L | R | T | F | L | R | - | - | - | - | - | - |
| 057 UniRef90\_G0FSK7\_17\_310 | - | - | - | - | V | R | G | V | V | L | P | G | C | G | H | Y | P | A | E | E | A | P | Q | A | M | L | A | A | L | S | E | F | L | A | P | W | A | D | - | - |
| 058 UniRef90\_A0A1Q8IR73\_6\_294 | - | - | - | - | V | K | G | E | T | I | A | N | C | G | H | F | Q | P | E | E | Q | P | E | A | V | A | D | A | L | A | R | F | F | A | - | - | - | - | - | - |
| 059 UniRef90\_A0A1I2MT44\_20\_306 | - | - | - | - | V | T | G | V | V | I | P | G | C | G | H | F | P | P | E | E | T | P | Q | E | L | L | S | A | L | R | P | F | - | - | - | - | - | - | - | - |
| 060 UniRef90\_UPI0010F95BB2\_18\_294 | - | - | - | - | P | E | G | W | I | V | P | N | C | G | H | Y | V | P | E | E | C | P | D | Q | L | A | E | R | L | V | S | F | F | R | N | S | - | - | - | - |
| 061 UniRef90\_A0A4P8X818\_19\_291 | - | - | - | - | V | T | G | V | - | I | L | D | Y | G | H | Q | L | A | E | E | C | P | E | D | F | A | H | V | L | D | E | F | F | - | - | - | - | - | - | - |
| 062 UniRef90\_A0A0M3UDU6\_18\_309 | - | - | - | - | V | Q | G | D | V | I | L | D | A | G | H | Y | L | P | E | E | A | A | N | R | I | A | D | L | L | V | D | F | L | K | - | - | - | - | - | - |
| 063 UniRef90\_A0A2P2CCU5\_19\_306 | - | - | - | - | V | E | T | L | V | V | P | G | T | G | H | W | V | A | E | A | A | P | E | A | V | I | E | A | L | T | T | F | L | - | - | - | - | - | - | - |
| 064 UniRef90\_UPI0009781B11\_10\_287 | - | - | - | - | V | R | G | T | V | I | A | N | C | G | H | Y | V | P | E | E | K | P | S | E | L | I | D | A | M | L | P | F | L | S | E | - | - | - | - | - |
| 065 UniRef90\_UPI000561A6EF\_30\_315 | - | - | - | - | V | R | E | V | I | L | P | Q | C | G | H | Y | P | A | E | E | A | P | E | A | T | L | A | A | - | - | - | - | - | - | - | - | - | - | - | - |
| 066 UniRef90\_A0A1Z4J856\_27\_318 | - | - | - | - | V | R | Y | S | A | I | E | N | C | G | H | W | I | P | E | E | R | P | A | Y | L | V | Q | Q | L | L | N | F | F | G | E | - | - | - | - | - |
| 067 UniRef90\_A0A5C5SVV1\_11\_281 | - | - | - | - | V | R | G | G | S | I | V | D | C | N | H | W | L | P | E | E | R | P | R | E | T | A | D | A | L | L | A | F | L | R | G | - | - | - | - | - |
| **068 Input\_protein\_seq** | - | - | - | - | V | R | G | A | V | A | P | N | T | G | H | W | L | P | D | E | N | P | A | F | L | T | R | Q | L | L | D | F | F | R | E | A | A | S | G | R |
| 069 UniRef90\_A0A1Q7W147\_16\_308 | - | - | - | - | A | T | G | I | V | V | P | D | A | G | H | F | I | P | D | E | Q | P | E | A | V | A | A | A | L | A | D | F | V | - | - | - | - | - | - | - |
| 070 UniRef90\_A0A0N0TCD7\_1\_288 | - | - | - | - | V | T | G | L | I | I | P | A | A | G | H | F | L | P | E | E | A | P | Q | A | L | T | T | A | L | L | D | F | L | R | - | - | - | - | - | - |
| 071 UniRef90\_UPI0004DF415F\_15\_308 | - | - | - | - | T | T | G | T | V | I | P | D | S | G | H | F | I | P | D | E | Q | P | G | A | L | V | D | A | L | T | A | F | I | - | - | - | - | - | - | - |
| 072 UniRef90\_G7LVZ3\_7\_288 | - | - | - | - | V | Q | T | A | V | V | E | R | S | G | H | W | I | P | E | E | Q | P | E | Q | L | A | A | L | L | V | E | F | F | T | T | T | S | A | G | - |

  
  

|  |  |  |  |  |  |  |  |  |  |  |  |  |  |  |  |  |  |
| --- | --- | --- | --- | --- | --- | --- | --- | --- | --- | --- | --- | --- | --- | --- | --- | --- | --- |
| |  |  |  |  |  |  |  |  |  | | --- | --- | --- | --- | --- | --- | --- | --- | --- | | 1 | 2 | 3 | 4 | 5 | 6 | 7 | 8 | 9 |   |  |  |  |  |  |  | | --- | --- | --- | --- | --- | --- | | **Variable** |  | **Average** |  | **Conserved** | | | |  |  |  | | --- | --- | | **X | - Insufficient data - the calculation for this site was performed on less than 10% of the sequences. |** |
