# Supplementary material for: Biochemical and structural characterization of two cif-like epoxide hydrolases from Burkholderia cenocepacia
Source: Curr Res Struct Biol. 2021 Feb 21;3:72–84. doi: 10.1016/j.crstbi.2021.02.002 (PMC8244358; doi:10.1016/j.crstbi.2021.02.002)
Supplement: Multimedia component 4 [file mmc4.zip › Cfl1_MSA.html]

ConSurf Color-Coded MSA

# ConSurf Color-Coded MSA

|  |  |  |  |  |  |  |  |  |  |  |  |  |  |  |  |  |  |  |  |  |  |  |  |  |  |  |  |  |  |  |  |  |  |  |  |  |  |  |  |  |  |  |  |  |  |  |  |  |  |  |
| --- | --- | --- | --- | --- | --- | --- | --- | --- | --- | --- | --- | --- | --- | --- | --- | --- | --- | --- | --- | --- | --- | --- | --- | --- | --- | --- | --- | --- | --- | --- | --- | --- | --- | --- | --- | --- | --- | --- | --- | --- | --- | --- | --- | --- | --- | --- | --- | --- | --- | --- |
| **001 Input\_protein\_seq** | M | Q | N | E | R | S | E | Q | S | M | P | G | M | P | A | P | G | - | - | - | - | L | P | A | G | F | E | R | R | F | S | R | R | Y | A | Q | L | D | - | D | V | R | L | H | Y | V | T | G | G | P |
| 002 UniRef90\_UPI00158A2D6D\_3\_306 | - | - | - | - | - | N | E | H | S | I | S | G | M | P | A | A | G | - | - | - | - | L | P | A | G | F | D | R | R | F | S | R | R | Y | V | H | V | N | - | D | V | R | L | H | Y | V | T | G | G | P |
| 003 UniRef90\_A0A1Z4J856\_27\_318 | - | - | - | - | - | - | - | - | - | - | - | - | - | P | F | - | - | - | - | - | - | N | K | A | A | F | I | K | T | F | A | H | H | T | A | K | V | N | - | D | V | R | L | H | Y | V | I | G | G | - |
| 004 UniRef90\_G7LVZ3\_7\_288 | - | - | - | - | - | - | - | - | - | - | - | - | - | - | - | - | - | - | - | - | - | - | - | - | - | - | - | - | - | F | T | H | H | T | T | V | V | N | - | G | V | R | L | H | Y | V | M | A | G | - |
| 005 UniRef90\_A0A2I8F4B9\_53\_324 | - | - | - | - | - | - | - | - | - | - | - | - | - | - | - | - | - | - | - | - | - | - | - | - | - | - | - | - | - | - | - | - | - | - | - | - | V | N | - | G | V | R | Y | H | Y | L | L | A | Q | - |
| 006 UniRef90\_A0A327RPK7\_10\_284 | - | - | - | - | - | - | - | - | - | - | - | - | - | - | - | - | - | - | - | - | - | - | - | - | - | - | - | - | - | Y | E | H | R | I | A | R | V | N | - | N | I | N | L | H | Y | V | I | A | G | - |
| 007 UniRef90\_A0A6L3SWG4\_20\_294 | - | - | - | - | - | - | - | - | - | - | - | - | - | - | - | - | - | - | - | - | - | - | - | - | - | - | - | - | - | - | - | - | - | - | - | - | - | - | - | G | V | R | L | H | Y | G | V | A | G | - |
| 008 UniRef90\_A0A401ZLI5\_4\_287 | - | - | - | - | - | - | - | - | - | - | - | - | - | P | - | - | - | - | - | - | - | L | P | A | P | L | E | P | L | F | T | H | Q | T | A | Q | V | N | - | G | V | R | L | H | Y | V | I | G | G | - |
| 009 UniRef90\_A0A4V2U6R1\_12\_297 | A | - | - | - | - | - | - | - | - | - | - | - | - | - | - | - | - | - | - | - | - | - | - | - | - | - | - | - | - | - | - | - | - | - | - | - | - | - | - | D | V | R | I | H | Y | V | T | A | G | M |
| 010 UniRef90\_A0A5A5T922\_6\_282 | - | - | - | - | - | - | - | - | - | - | - | - | - | - | - | - | - | - | - | - | - | - | - | - | - | - | - | - | A | F | E | H | H | T | A | Q | V | G | - | D | V | T | I | H | Y | V | R | M | G | - |
| 011 UniRef90\_A0A2V6UIN9\_3\_273 | - | - | - | - | - | - | - | - | - | - | - | - | - | - | - | - | - | - | - | - | - | - | - | - | - | - | - | - | - | - | - | S | R | F | D | E | V | N | - | G | V | R | I | H | Y | L | I | S | G | - |
| 012 UniRef90\_UPI001669B3C5\_41\_314 | - | - | - | - | - | - | - | - | - | - | - | - | - | - | - | - | - | - | - | - | - | - | - | - | - | - | - | - | - | F | S | A | H | D | V | K | V | N | - | G | V | Q | L | H | Y | Q | I | S | G | - |
| 013 UniRef90\_A0A4R2Z7A8\_3\_305 | - | - | - | - | - | - | - | Q | N | M | P | S | T | P | V | A | T | A | L | F | G | I | P | D | P | T | L | K | G | F | H | H | H | Y | A | T | V | D | - | G | V | R | I | H | Y | V | C | G | G | - |
| 014 UniRef90\_A0A5C5SVV1\_11\_281 | - | - | - | - | - | - | - | - | - | - | - | - | - | - | - | - | - | - | - | - | - | - | - | - | - | - | - | - | - | - | - | - | - | - | V | R | V | G | - | E | V | N | L | H | T | V | I | A | G | - |
| 015 UniRef90\_L9WLS3\_16\_284 | - | - | - | - | - | - | - | - | - | - | - | - | - | - | - | - | - | - | - | - | - | - | - | - | - | - | - | - | - | - | - | - | - | - | - | R | V | N | - | G | V | S | I | H | Y | V | T | A | G | - |
| 016 UniRef90\_UPI00131EC7B5\_30\_311 | - | - | - | - | - | - | - | - | - | - | - | - | - | - | - | - | - | - | - | - | - | - | - | - | - | - | - | - | G | F | E | H | R | Y | A | T | V | D | - | G | I | R | M | H | Y | V | T | G | G | - |
| 017 UniRef90\_C7QAM5\_50\_332 | - | - | - | - | - | - | - | - | - | - | - | - | - | - | - | - | - | - | - | - | - | - | - | - | - | M | P | A | G | F | S | E | H | K | T | D | V | G | - | G | I | G | I | D | Y | V | I | G | G | - |
| 018 UniRef90\_A0A1Q3SXP6\_11\_288 | N | - | - | - | - | - | - | - | - | - | - | - | - | - | - | - | - | - | - | - | - | - | - | - | - | - | E | K | M | M | K | S | H | F | A | D | V | N | - | G | L | K | I | H | Y | L | T | E | G | - |
| 019 UniRef90\_UPI00149248BF\_39\_315 | - | - | - | - | - | - | - | - | - | - | - | - | - | - | - | - | - | - | - | - | - | - | - | - | - | - | - | - | D | F | K | S | Q | Y | A | D | V | N | - | G | V | K | L | H | Y | V | E | G | G | - |
| 020 UniRef90\_UPI000361D127\_26\_313 | - | - | - | - | - | - | - | - | - | - | - | - | V | P | D | - | - | - | - | - | - | L | P | A | G | F | A | D | V | F | S | S | S | Y | V | D | T | G | - | S | V | R | L | H | T | V | V | G | G | - |
| 021 UniRef90\_A0A0M4FVH2\_24\_312 | - | - | - | - | - | - | - | - | - | - | - | - | - | - | - | - | - | - | - | - | - | R | P | E | P | S | L | D | G | F | K | H | R | F | Q | T | V | D | - | G | V | R | L | H | Y | V | V | G | G | - |
| 022 UniRef90\_A0A1Q4ZL08\_9\_290 | - | - | - | - | - | - | - | - | - | - | - | - | - | - | - | - | - | - | - | - | - | - | - | - | - | - | - | - | G | F | T | E | Q | V | A | R | V | G | - | E | I | T | I | N | Y | V | R | G | G | - |
| 023 UniRef90\_S3CY91\_25\_307 | Q | - | - | - | - | - | - | - | - | - | - | - | - | - | - | - | - | - | - | - | - | - | - | - | - | - | - | - | - | - | - | - | - | - | - | - | - | - | - | E | V | R | I | H | Y | I | D | C | P | P |
| 024 UniRef90\_UPI0010F95BB2\_18\_294 | - | - | - | - | - | - | - | - | - | - | - | - | - | - | - | - | - | - | - | - | - | - | - | - | - | - | - | - | - | - | - | - | - | - | - | - | - | N | - | G | L | R | L | H | L | T | E | S | G | S |
| 025 UniRef90\_A0A0N1GDE2\_28\_316 | - | - | - | - | - | - | - | - | - | - | - | - | - | L | R | - | - | - | - | - | - | L | P | N | G | F | T | D | V | F | T | S | R | L | V | E | L | N | - | G | L | R | L | H | A | V | T | G | G | - |
| 026 UniRef90\_A0A2E5PJQ7\_5\_284 | - | - | - | - | - | - | - | - | - | - | - | - | - | - | - | - | - | - | - | - | - | - | - | - | - | - | - | - | - | - | A | H | H | Y | A | D | L | G | - | D | V | K | I | H | Y | I | T | A | G | - |
| 027 UniRef90\_A0A1M7IC97\_44\_328 | - | - | - | - | - | - | - | - | - | - | - | - | - | - | - | - | - | - | - | - | - | - | - | E | R | M | P | A | G | F | S | E | H | K | T | R | V | A | - | G | I | G | L | H | Y | V | I | G | G | - |
| 028 UniRef90\_UPI0016149F5E\_5\_284 | - | - | - | - | - | - | - | - | - | - | - | - | - | - | - | - | - | - | - | - | - | - | - | - | - | - | E | N | H | I | T | H | E | Y | V | R | I | A | - | G | Q | R | L | H | C | A | I | T | G | - |
| 029 UniRef90\_UPI00156E1DFF\_8\_289 | - | - | - | - | - | - | - | - | - | - | - | - | - | - | - | - | - | - | - | - | - | - | - | - | - | - | P | D | G | F | E | S | I | T | V | S | V | N | - | G | T | R | L | H | A | V | V | G | G | - |
| 030 UniRef90\_A0A3A8HRU0\_15\_300 | - | - | - | - | - | - | - | - | - | - | - | - | A | P | G | A | - | - | - | - | - | I | R | P | F | F | P | E | G | F | R | E | G | D | E | D | V | N | - | G | T | R | I | H | F | V | I | G | G | - |
| 031 UniRef90\_A0A0M3UDU6\_18\_309 | - | - | - | - | - | - | - | - | - | - | - | - | - | - | - | - | - | - | - | - | - | - | R | T | D | F | W | K | C | F | H | H | A | S | A | R | V | G | - | D | V | N | L | H | Y | V | E | G | G | - |
| 032 UniRef90\_A0A447J1A9\_28\_317 | - | - | - | - | - | - | - | - | - | - | P | K | P | P | V | - | - | - | - | - | - | T | A | Q | V | N | E | A | D | F | Q | H | H | Y | T | D | V | A | - | G | Q | R | I | H | Y | V | T | A | G | - |
| 033 UniRef90\_A0A5C8T429\_14\_295 | - | - | - | - | - | - | - | - | - | - | - | - | - | - | - | - | - | - | - | - | - | - | - | - | - | F | G | R | I | F | Q | H | R | V | Q | G | A | V | - | G | N | R | L | H | Y | V | T | G | G | - |
| 034 UniRef90\_A0A0N0TCD7\_1\_288 | - | - | - | - | - | - | - | - | - | - | - | - | - | - | - | - | - | - | - | - | - | M | P | A | G | F | T | D | T | F | T | S | H | T | V | E | T | G | - | D | L | T | L | H | A | L | A | G | G | - |
| 035 UniRef90\_W9ARP2\_16\_295 | - | - | - | - | - | - | - | - | - | - | - | - | - | - | - | - | - | - | - | - | - | - | - | - | - | - | - | - | - | - | - | - | - | - | V | D | A | D | E | S | V | R | I | G | Y | T | V | A | T | P |
| 036 UniRef90\_A0A109IGY4\_11\_301 | - | - | - | - | - | - | - | - | - | - | - | S | S | L | R | - | - | - | - | - | - | L | P | A | G | F | T | D | V | F | T | S | Q | L | V | E | L | D | - | G | L | H | L | H | A | V | T | G | G | - |
| 037 UniRef90\_K9DQK6\_24\_306 | - | - | - | - | - | - | - | - | - | - | - | - | E | P | S | - | - | - | - | - | - | - | - | - | - | M | P | A | G | F | K | T | A | S | V | Q | T | A | D | G | A | T | I | H | V | R | S | G | G | - |
| 038 UniRef90\_A0A1Q8IR73\_6\_294 | - | - | - | - | - | - | - | - | - | - | - | - | - | - | - | - | - | - | - | - | - | L | P | A | - | A | P | T | G | F | E | H | K | F | A | T | A | E | - | G | L | R | Y | H | Y | V | E | G | G | - |
| 039 UniRef90\_A0A1Y6D2S0\_29\_302 | - | - | - | - | - | - | - | - | - | - | - | - | - | - | - | - | - | - | - | - | - | - | - | - | - | - | - | - | - | - | K | S | G | F | A | Q | V | N | - | G | I | Q | M | H | Y | V | K | M | G | - |
| 040 UniRef90\_E6WJ64\_19\_301 | - | - | - | - | - | - | - | - | - | - | - | - | - | - | - | - | - | - | - | - | - | - | - | - | - | - | - | - | G | F | A | H | H | Y | A | T | V | D | - | G | V | R | L | H | Y | V | S | G | G | - |
| 041 UniRef90\_UPI000489A94A\_43\_316 | - | - | - | - | - | - | - | - | - | - | - | - | - | - | - | - | - | - | - | - | - | - | - | - | - | - | - | - | - | - | - | - | - | - | - | - | A | N | - | G | L | R | L | H | Y | L | Q | A | G | - |
| 042 UniRef90\_A0A4R6HBQ4\_11\_285 | - | - | - | - | - | - | - | - | - | - | - | - | - | - | - | - | - | - | - | - | - | - | - | - | - | - | - | - | - | - | - | - | - | - | - | - | - | A | - | D | L | R | L | H | A | V | F | A | G | P |
| 043 UniRef90\_A0A1M7QJX9\_32\_311 | - | - | - | - | - | - | - | - | - | - | - | - | - | - | - | - | - | - | - | - | - | - | P | A | S | P | S | P | G | F | K | H | Q | Y | A | Q | V | N | - | G | V | R | I | H | Y | V | I | G | G | - |
| 044 UniRef90\_A0A5B8WA72\_22\_300 | - | - | - | - | - | - | - | - | - | - | - | - | - | - | - | - | - | - | - | - | - | - | P | A | N | P | P | S | N | F | K | H | Q | Y | A | T | V | N | - | N | V | K | I | H | Y | V | I | G | G | - |
| 045 UniRef90\_A0A0G2FGE6\_47\_303 | - | - | - | - | - | - | - | - | - | - | - | - | - | - | - | - | - | - | - | - | - | - | - | - | - | - | - | - | - | - | - | - | - | - | - | - | - | - | - | - | - | - | - | - | - | - | - | - | - | - |
| 046 UniRef90\_A0A1I6BK09\_10\_296 | - | - | - | - | - | - | - | - | - | - | - | - | - | - | - | - | - | - | - | - | - | - | - | - | - | - | - | - | - | - | T | H | H | R | A | Q | V | G | - | E | V | S | L | H | Y | L | Q | A | G | - |
| 047 UniRef90\_A0A4R7C9R7\_33\_310 | - | - | - | - | - | - | - | - | - | - | S | R | L | P | P | P | - | - | - | - | - | A | D | P | P | L | P | T | G | A | E | S | R | F | A | T | V | N | - | G | I | R | M | H | Y | V | V | A | G | - |
| 048 UniRef90\_A0A2M9M9Q1\_18\_305 | - | - | - | - | - | - | - | - | - | - | - | - | V | P | W | - | - | - | - | - | - | L | P | D | G | F | T | D | V | F | E | S | S | L | V | Q | V | G | - | R | V | G | L | H | T | V | Q | G | G | - |
| 049 UniRef90\_A0A4V3T343\_11\_295 | - | - | - | - | - | - | - | - | - | - | - | - | - | - | - | - | - | - | - | - | A | L | A | A | S | L | D | G | G | F | T | S | H | H | A | D | V | N | - | G | T | R | L | H | Y | V | D | G | G | - |
| 050 UniRef90\_UPI001616534D\_11\_303 | - | - | - | - | - | - | - | - | - | - | - | - | - | - | - | - | - | - | - | - | A | L | A | A | S | L | E | G | N | F | S | S | R | H | A | E | V | N | - | G | V | R | L | H | Y | V | E | G | G | - |
| 051 UniRef90\_J4PG95\_17\_290 | - | - | - | - | - | - | - | - | - | - | - | - | - | - | - | - | - | - | - | - | - | - | - | - | - | - | - | - | - | - | - | H | Q | Y | P | L | I | D | - | G | H | R | I | H | C | A | I | A | G | - |
| 052 UniRef90\_A0A0T1T741\_1\_295 | - | - | - | - | - | - | - | M | T | G | L | S | S | L | R | - | - | - | - | - | - | L | P | D | G | F | L | D | V | F | T | S | R | L | V | E | V | N | - | G | L | R | L | H | A | V | T | G | G | - |
| 053 UniRef90\_G0FSK7\_17\_310 | - | - | - | - | - | - | - | - | - | - | - | G | T | P | G | - | - | - | - | - | - | L | P | A | G | F | D | E | V | F | T | S | R | W | I | A | V | G | - | E | L | R | L | H | A | V | V | G | G | - |
| 054 UniRef90\_UPI00164AAB2D\_18\_303 | - | - | - | - | - | - | - | - | - | - | - | - | A | P | A | W | A | - | - | - | - | A | P | L | P | Y | P | A | T | F | K | I | E | N | I | K | T | P | - | E | V | T | I | H | V | R | V | G | G | - |
| 055 UniRef90\_A0A1I2MT44\_20\_306 | - | - | - | - | - | - | - | - | - | - | - | - | V | P | R | - | - | - | - | - | - | L | P | A | G | F | T | D | T | F | T | S | R | Y | V | D | V | D | - | G | L | R | L | H | A | V | T | G | G | - |
| 056 UniRef90\_A0A2P2CCU5\_19\_306 | - | - | - | - | - | - | - | - | - | - | - | R | A | P | G | - | - | - | - | - | - | L | P | A | G | F | S | T | T | F | S | S | Q | T | V | T | A | N | - | G | I | R | Q | H | V | V | I | G | G | - |
| 057 UniRef90\_A0A2V9H5C8\_12\_298 | - | - | - | - | - | - | - | - | - | L | P | F | R | P | R | P | T | - | - | - | - | - | L | Q | T | P | D | N | T | F | A | S | H | T | T | E | I | D | - | G | L | K | L | H | Y | T | T | G | G | - |
| 058 UniRef90\_A0A0P4V0Y0\_7\_291 | - | - | - | - | - | - | - | - | - | - | - | - | - | - | - | - | - | - | - | - | - | - | - | - | - | - | - | - | T | F | S | H | H | T | T | E | A | N | - | G | V | K | L | H | Y | V | I | G | G | - |
| 059 UniRef90\_A0A0Q4UQ58\_27\_305 | - | - | - | - | - | - | - | - | - | - | - | A | V | P | D | S | G | - | - | - | L | D | A | G | V | L | P | P | G | A | A | S | R | F | A | Q | L | N | - | G | I | R | M | H | Y | V | V | A | G | - |
| 060 UniRef90\_A0A6M4IM28\_20\_309 | - | - | - | - | - | - | - | - | - | - | - | S | T | P | S | A | A | - | - | - | - | Q | P | P | A | F | P | P | A | F | R | V | E | R | I | A | T | N | - | S | T | M | L | H | V | R | V | G | G | - |
| 061 UniRef90\_A0A537J419\_20\_301 | - | - | - | - | - | - | - | - | - | - | - | - | - | P | S | V | S | - | - | - | - | - | - | V | S | A | A | A | S | F | V | S | R | T | A | V | V | D | - | G | V | T | L | H | Y | L | T | G | G | - |
| 062 UniRef90\_A0A1V2PGN8\_6\_275 | - | - | - | - | - | - | - | - | - | - | - | - | - | - | - | - | - | - | - | - | - | - | - | - | - | - | - | - | - | - | - | - | - | - | A | T | V | N | - | G | I | R | T | H | Y | L | R | G | G | - |
| 063 UniRef90\_A0A542JC81\_20\_314 | - | - | - | - | - | - | - | - | - | - | - | Q | A | P | N | - | - | - | - | - | - | L | P | E | G | F | T | D | T | F | T | S | R | Y | I | D | A | N | - | G | V | R | L | H | A | V | I | G | G | - |
| 064 UniRef90\_UPI000424F353\_33\_308 | - | - | - | - | - | - | - | - | - | - | - | - | - | - | - | - | - | - | - | - | - | - | - | - | - | - | P | A | N | F | R | M | Q | T | I | P | I | N | - | S | T | Q | I | F | V | R | V | G | G | - |
| 065 UniRef90\_A0A2V9W2E9\_22\_299 | - | - | - | - | - | - | - | - | - | - | - | - | - | P | S | - | - | - | - | - | - | - | - | - | - | L | P | S | S | F | Q | A | K | T | I | H | S | P | A | G | A | D | I | F | V | R | W | G | G | - |
| 066 UniRef90\_A0A2T6L0M5\_16\_299 | - | - | - | - | - | - | - | - | - | - | - | - | - | - | - | - | - | - | - | - | - | - | - | - | - | V | P | A | G | Y | E | S | H | Y | A | H | V | N | - | G | F | R | M | H | Y | L | R | G | G | - |
| 067 UniRef90\_A0A261TYY2\_43\_315 | - | - | - | - | - | - | - | - | - | - | - | - | - | - | - | - | - | - | - | - | - | - | - | - | - | - | - | - | - | - | - | - | R | M | Q | G | E | P | - | G | Q | R | L | H | Y | V | I | G | G | - |
| 068 UniRef90\_UPI0009781B11\_10\_287 | - | - | - | - | - | - | - | - | - | - | - | - | - | - | - | - | - | - | - | - | - | - | - | - | - | - | - | - | - | - | - | - | R | V | I | D | A | Q | - | G | A | K | L | H | F | V | E | K | G | - |
| 069 UniRef90\_A0A2V9TPC1\_35\_307 | - | - | - | - | - | - | - | - | - | - | - | - | - | - | - | - | - | - | - | - | - | - | - | - | - | - | - | - | T | I | V | S | R | T | A | E | I | N | - | G | V | K | L | H | Y | I | T | A | G | - |
| 070 UniRef90\_UPI00145FA88A\_6\_279 | - | - | - | - | - | - | - | - | - | - | - | - | - | - | - | - | - | - | - | - | - | - | - | - | - | - | - | - | - | - | - | - | - | - | A | K | V | N | - | G | I | R | M | H | Y | L | R | G | G | - |
| 071 UniRef90\_UPI0003FADEBE\_21\_306 | - | - | - | - | - | - | - | - | - | - | - | - | L | P | A | L | A | - | - | - | - | Q | P | R | P | Y | P | A | S | F | K | A | Q | R | I | Q | A | A | G | G | A | T | L | H | V | R | V | G | G | - |
| 072 UniRef90\_A0A2V8Y3P4\_78\_329 | - | - | - | - | - | - | - | - | - | - | - | - | - | - | - | - | - | - | - | - | - | - | - | - | - | - | - | - | - | - | - | - | - | - | - | - | - | - | - | - | - | - | - | - | - | - | S | G | G | - |
| 073 UniRef90\_A0A285BKA7\_8\_299 | - | - | - | - | - | - | - | - | - | - | - | - | V | P | G | - | - | - | - | - | - | L | S | A | A | F | T | D | T | F | T | S | R | T | V | T | T | D | E | G | M | T | L | H | A | V | V | G | G | - |
| 074 UniRef90\_A0A0K3AVW3\_44\_319 | - | - | - | - | - | - | - | - | - | - | - | - | - | - | - | - | - | - | - | - | - | - | - | - | - | - | - | - | - | F | R | N | G | Y | A | K | V | N | - | G | I | R | M | H | Y | V | T | G | G | - |
| 075 UniRef90\_A0A4D7B7N1\_7\_276 | - | - | - | - | - | - | - | - | - | - | - | - | - | - | - | - | - | - | - | - | - | - | - | - | - | - | - | - | - | - | - | - | - | - | - | - | - | - | - | - | L | R | F | H | T | V | E | G | G | - |
| 076 UniRef90\_A0A2U0WF37\_17\_293 | - | - | - | - | - | - | - | - | - | - | - | - | - | P | A | H | A | - | - | - | - | G | V | T | P | F | P | A | S | F | Q | T | K | D | I | A | V | D | - | G | A | T | L | H | V | R | V | G | G | - |
| 077 UniRef90\_UPI00135C2D49\_17\_309 | - | - | - | - | - | - | - | - | - | - | - | - | - | - | - | - | - | - | - | - | - | - | - | - | S | L | D | G | S | F | S | S | H | H | A | D | V | N | - | G | V | R | L | H | Y | V | E | G | G | - |
| 078 UniRef90\_UPI001473614E\_45\_322 | - | - | - | - | - | - | - | - | - | - | - | A | T | P | K | E | P | A | - | - | E | G | P | A | R | F | A | P | G | F | R | H | G | K | V | A | V | E | - | G | G | A | V | H | Y | V | I | G | G | - |
| 079 UniRef90\_J3F862\_22\_299 | - | - | - | - | - | - | - | - | - | - | - | - | - | - | - | - | - | - | - | - | - | - | V | T | P | F | P | A | S | F | R | T | Q | D | I | A | V | D | - | G | A | K | I | H | T | R | I | G | G | - |
| 080 UniRef90\_A0A1A9NC01\_62\_345 | - | - | - | - | - | - | - | - | - | - | - | - | - | - | - | - | - | - | - | - | - | - | - | - | - | - | - | - | - | F | S | N | G | Y | A | E | V | N | - | G | V | R | L | H | Y | V | V | G | G | - |
| 081 UniRef90\_UPI00131AC11B\_45\_327 | - | - | - | - | - | - | - | - | - | - | - | - | - | - | - | - | - | - | - | - | - | - | - | - | - | - | - | - | - | F | K | N | G | Y | A | D | V | D | - | G | I | R | L | H | Y | V | T | G | G | - |
| 082 UniRef90\_A0A4R8HC20\_22\_306 | - | - | - | - | - | - | - | - | - | - | - | - | - | - | - | A | - | - | - | - | A | L | A | A | S | L | D | G | G | F | T | S | H | H | A | D | V | N | - | G | T | R | L | H | Y | V | E | G | G | - |
| 083 UniRef90\_A0A1B2HH26\_4\_280 | - | - | - | - | - | - | - | - | - | - | - | - | - | - | - | - | - | - | - | - | - | - | P | A | P | A | P | E | G | F | E | H | A | Y | A | E | V | N | - | G | V | Q | M | H | Y | V | T | G | G | - |
| 084 UniRef90\_A0A517LKB1\_23\_304 | - | - | - | - | - | - | - | - | - | - | - | - | - | P | E | - | - | - | - | - | - | - | - | - | - | - | - | - | - | - | - | - | - | - | - | - | - | - | - | E | V | R | I | H | Y | R | I | A | Q | P |
| 085 UniRef90\_A0A0N1F3N8\_17\_298 | - | - | - | - | - | - | - | - | - | - | - | - | - | P | A | A | A | - | - | - | - | Q | L | V | A | F | P | P | S | F | K | T | Q | E | I | K | A | N | - | G | T | T | I | H | V | R | I | G | G | - |
| 086 UniRef90\_UPI0004CCFD44\_55\_340 | - | - | - | - | - | - | - | - | - | - | - | - | - | - | - | - | - | - | - | - | - | - | - | - | E | F | E | G | T | F | R | H | E | F | A | D | V | D | - | G | V | R | M | H | Y | V | T | G | G | - |
| 087 UniRef90\_A0A4D4LDP7\_7\_304 | - | - | - | - | - | - | - | - | - | - | - | P | R | P | G | I | G | A | E | - | I | S | D | A | E | Q | L | E | G | F | T | H | L | S | A | D | A | D | - | G | I | R | I | H | A | V | V | G | G | - |
| 088 UniRef90\_A0A2V4B948\_8\_279 | - | - | - | - | - | - | - | - | - | - | - | - | - | - | - | - | - | - | - | - | - | - | - | - | - | - | - | - | - | - | - | - | - | - | - | - | V | N | - | G | I | R | M | H | Y | R | I | A | G | - |
| 089 UniRef90\_A0A2P2FV90\_3\_282 | - | - | - | - | - | - | - | - | - | - | - | - | - | - | - | - | - | - | - | - | - | - | - | - | - | - | - | - | R | L | E | S | K | T | A | S | V | N | - | G | I | R | L | H | Y | L | R | A | G | - |
| 090 UniRef90\_A0A4Q2J292\_17\_304 | - | - | - | - | - | - | - | - | - | - | H | A | M | P | A | A | A | - | - | - | - | A | V | P | P | F | P | S | G | F | K | T | Q | T | I | A | V | N | - | G | A | K | M | Y | V | R | V | G | G | - |
| 091 UniRef90\_UPI0012B05B7D\_16\_287 | - | - | - | - | - | - | - | - | - | - | - | - | - | - | - | - | - | - | - | - | - | - | - | - | - | - | - | - | - | - | - | - | - | - | - | - | F | D | - | N | L | R | L | H | Y | L | E | S | G | - |
| 092 UniRef90\_UPI00048D4AD4\_4\_301 | - | - | - | - | - | - | - | - | - | - | - | - | - | P | D | A | - | - | - | - | A | L | A | A | S | L | D | G | G | F | T | S | H | H | A | D | L | D | - | G | V | R | L | H | Y | V | D | G | G | - |
| 093 UniRef90\_UPI0015F81933\_16\_310 | - | - | - | - | - | - | - | - | - | - | - | - | - | P | I | R | D | - | - | - | - | L | P | L | R | H | L | A | G | F | T | H | R | W | V | D | A | D | - | G | V | R | L | H | A | V | E | G | G | - |
| 094 UniRef90\_UPI00160CFF45\_55\_340 | - | - | - | - | - | - | - | - | - | - | - | - | - | - | - | - | - | - | - | - | - | - | - | - | Q | F | T | S | T | F | R | H | E | F | A | D | V | N | - | G | V | R | M | H | Y | V | A | G | G | - |
| 095 UniRef90\_A0A1Q8KTW7\_17\_294 | - | - | - | - | - | - | - | - | - | - | - | - | - | - | - | - | - | - | - | - | - | - | - | - | - | - | - | - | - | - | - | - | - | - | A | T | V | N | - | G | V | R | L | H | Y | V | T | A | G | - |
| 096 UniRef90\_UPI00161A7EC4\_21\_297 | - | - | - | - | - | - | - | - | - | - | - | - | - | P | R | E | P | S | - | - | T | G | Q | A | R | F | S | P | I | F | T | H | G | R | V | A | V | D | G | G | S | T | L | H | Y | V | R | G | G | - |
| 097 UniRef90\_A0A1B4WXA8\_16\_299 | - | - | - | - | - | - | - | - | - | - | - | - | L | P | A | H | A | - | - | - | - | A | I | A | P | F | P | A | S | F | H | T | Q | D | I | P | V | E | - | G | A | T | M | H | V | R | V | G | G | - |
| 098 UniRef90\_UPI000E46D2C1\_48\_330 | - | - | - | - | - | - | - | - | - | - | - | - | - | - | - | - | - | - | - | - | - | - | - | - | - | - | - | - | G | F | A | S | R | F | A | E | V | N | - | G | I | R | M | H | Y | V | T | G | G | - |
| 099 UniRef90\_A0A2N5CFI4\_23\_293 | - | - | - | - | - | - | - | - | - | - | - | - | - | - | - | - | - | - | - | - | - | - | V | F | S | F | P | A | D | F | K | K | E | I | I | S | T | N | - | G | V | K | M | Y | V | R | V | G | G | - |
| 100 UniRef90\_A0A385B2U0\_45\_333 | - | - | - | - | - | - | - | - | - | - | - | - | - | - | - | - | - | - | - | - | R | L | P | Y | R | L | P | D | G | F | R | S | E | Y | A | Q | V | N | - | D | F | R | M | H | Y | L | R | G | G | - |
| 101 UniRef90\_A0A1Q7W147\_16\_308 | - | - | - | - | - | - | - | - | - | - | - | - | - | P | V | R | D | - | - | - | - | L | P | L | H | D | L | A | G | F | T | H | R | W | V | D | A | D | - | G | I | R | L | H | A | V | E | G | G | - |
| 102 UniRef90\_A0A239MTD5\_1\_271 | - | - | - | - | - | - | - | - | - | - | - | - | - | - | - | - | - | - | - | - | - | - | - | - | - | - | - | - | - | - | - | - | - | - | - | - | - | - | - | - | - | - | M | H | Y | M | E | G | G | - |
| 103 UniRef90\_UPI000561A6EF\_30\_315 | - | - | - | - | - | - | - | - | - | A | S | G | A | P | E | - | - | - | - | - | - | L | P | A | G | F | G | E | T | F | T | S | R | Y | V | D | T | G | - | A | L | R | L | H | A | V | V | G | G | - |
| 104 UniRef90\_A0A4Y8RHA3\_28\_294 | - | - | - | - | - | - | - | - | - | - | - | - | - | - | - | - | - | - | - | - | - | - | - | - | - | - | - | - | S | F | T | T | S | D | I | A | T | N | - | G | T | S | L | H | V | R | I | G | G | - |
| 105 UniRef90\_UPI0004DF415F\_15\_308 | - | - | - | - | - | - | - | - | - | - | - | - | N | P | I | S | G | - | - | - | - | L | P | L | H | D | L | A | G | F | T | H | R | W | V | D | A | A | - | G | I | R | L | H | A | V | E | G | G | - |
| 106 UniRef90\_A0A2N3KZL1\_30\_325 | - | - | - | - | - | - | - | - | - | - | - | - | K | P | Q | S | Q | P | - | - | E | S | E | F | P | V | P | A | G | F | E | S | S | Y | A | E | I | N | - | G | V | R | L | H | Y | V | K | G | G | - |
| 107 UniRef90\_UPI00076E3DF2\_55\_340 | - | - | - | - | - | - | - | - | - | - | - | - | - | - | - | - | - | - | - | - | - | - | - | - | E | F | E | S | T | F | R | H | E | F | A | D | V | D | - | G | V | R | M | H | Y | V | T | G | G | - |
| 108 UniRef90\_UPI00130E1FA7\_66\_348 | - | - | - | - | - | - | - | - | - | - | - | - | - | - | - | - | - | - | - | - | - | - | - | - | - | - | - | - | G | F | K | N | G | F | A | E | V | N | - | G | T | R | L | H | Y | V | E | G | G | - |
| 109 UniRef90\_A0A1I3FUH2\_46\_328 | - | - | - | - | - | - | - | - | - | - | - | - | - | - | - | - | - | - | - | - | - | - | - | - | - | - | - | - | G | F | E | R | G | F | A | E | V | N | - | G | V | R | L | H | Y | V | E | G | G | - |
| 110 UniRef90\_UPI000E2836BA\_28\_313 | - | - | - | - | - | - | - | - | - | - | - | - | - | - | - | - | - | - | - | - | - | L | A | A | S | L | D | G | A | F | S | S | R | Y | A | K | V | N | - | G | V | G | L | H | Y | V | T | G | G | - |
| 111 UniRef90\_A0A653WNW3\_25\_297 | - | - | - | - | - | - | - | - | - | - | - | - | - | - | - | - | - | - | - | - | - | - | - | - | - | F | P | T | D | F | K | T | Q | E | I | A | T | N | - | G | T | T | L | H | V | R | I | G | G | - |
| 112 UniRef90\_A0A5C1I8U5\_39\_321 | - | - | - | - | - | - | - | - | - | - | - | - | - | - | - | - | - | - | - | - | - | - | - | - | - | - | - | - | - | F | K | N | G | F | A | T | V | N | - | G | V | K | L | H | Y | V | A | G | G | - |
| 113 UniRef90\_UPI00165059AC\_4\_267 | - | - | - | - | - | - | - | - | - | - | - | - | - | - | - | - | - | - | - | - | - | - | - | - | - | - | - | - | - | - | - | - | - | - | A | R | V | N | - | G | I | R | M | Y | Y | H | V | A | G | - |
| 114 UniRef90\_F8JLL5\_12\_309 | - | - | - | - | - | - | - | - | - | - | - | G | N | P | V | R | D | - | - | - | - | L | P | L | P | D | L | A | G | F | T | H | R | W | V | D | A | D | - | G | V | L | L | H | A | V | E | G | G | - |
| 115 UniRef90\_A0A329J6I4\_30\_314 | - | - | - | - | - | - | - | - | - | - | - | - | - | - | - | - | - | - | - | - | - | - | - | - | - | - | P | K | G | F | T | S | E | F | R | T | V | D | - | G | V | K | L | H | Y | V | K | G | G | - |
| 116 UniRef90\_A0A3N1H0W7\_60\_340 | - | - | - | - | - | - | - | - | - | - | - | - | - | - | - | - | - | - | - | - | - | - | - | - | - | - | - | - | - | F | R | S | D | H | V | T | A | D | - | G | L | R | L | H | Y | V | A | G | G | - |
| 117 UniRef90\_A0A316VRQ3\_1\_263 | - | - | - | - | - | - | - | - | - | - | - | - | - | - | - | - | - | - | - | - | - | - | - | - | - | - | - | - | - | - | - | - | - | - | - | - | - | - | - | - | - | - | - | - | - | - | - | - | - | - |
| 118 UniRef90\_A0A4P8X818\_19\_291 | - | - | - | - | - | - | - | - | - | - | - | - | - | - | - | - | - | - | - | - | - | - | - | - | - | - | - | - | - | - | - | - | - | - | - | T | V | N | - | G | V | R | L | H | Y | Y | M | A | G | - |
| 119 UniRef90\_UPI0008405A69\_11\_309 | - | - | - | - | - | - | - | - | - | - | - | - | - | - | - | - | - | - | - | - | - | L | A | A | S | L | A | S | D | F | T | S | A | Y | A | E | V | N | - | G | I | R | L | H | H | V | T | G | G | - |
| 120 UniRef90\_A0A6I8M538\_3\_275 | - | - | - | - | - | - | - | - | - | - | - | - | - | - | - | - | - | - | - | - | - | - | - | - | - | - | - | - | - | - | - | - | - | - | - | T | V | N | - | G | I | R | M | H | F | V | K | A | G | - |
| 121 UniRef90\_UPI000B5CCBFE\_21\_300 | - | - | - | - | - | - | - | - | - | - | - | - | - | - | - | - | - | - | - | - | - | - | - | - | - | - | - | G | D | F | H | S | E | F | A | E | V | N | - | G | T | R | L | H | Y | V | V | G | G | - |
| 122 UniRef90\_A0A0M9ZDW3\_1\_266 | - | - | - | - | - | - | - | - | - | - | - | - | - | - | - | - | - | - | - | - | - | - | - | - | - | - | - | - | - | - | - | - | - | - | - | - | - | - | - | - | - | - | M | H | Y | V | I | G | G | - |
| 123 UniRef90\_UPI000E275A28\_18\_308 | - | - | - | - | - | - | - | - | - | - | - | - | V | P | E | - | - | - | - | - | - | L | P | N | G | F | L | D | V | F | E | S | Y | R | I | P | V | G | - | D | V | S | L | H | A | V | I | G | G | - |
| 124 UniRef90\_A0A3L8K0H7\_13\_298 | - | - | - | - | - | - | - | - | - | - | - | - | - | - | - | - | - | - | - | - | - | L | A | A | S | L | D | G | G | F | S | S | R | Y | A | E | V | D | - | G | V | R | L | H | Y | V | T | G | G | - |
| 125 UniRef90\_A0A6N7ZB66\_18\_297 | - | - | - | - | - | - | - | - | - | - | - | - | - | - | - | - | - | - | - | - | - | - | - | - | - | - | - | - | G | F | T | V | R | E | V | Q | T | N | - | G | V | R | L | S | A | A | V | G | G | - |
| 126 UniRef90\_A0A2W7GM08\_52\_337 | - | - | - | - | - | - | - | - | - | - | - | - | - | - | - | - | - | - | - | - | - | - | - | - | - | - | - | - | - | F | K | N | G | T | A | Q | V | N | - | G | I | D | I | H | Y | V | V | G | G | - |
| 127 UniRef90\_A0A258JI33\_23\_300 | - | - | - | - | - | - | - | - | - | - | - | - | - | - | - | - | - | - | - | - | - | K | I | E | R | F | P | R | G | F | H | I | Q | R | I | N | T | G | - | D | A | T | I | Y | V | R | V | G | G | - |
| 128 UniRef90\_A0A6A6C1N5\_26\_297 | - | - | - | - | - | - | - | - | - | - | - | - | - | - | - | - | - | - | - | - | - | - | - | - | - | - | - | - | - | - | - | - | - | - | - | - | T | N | - | - | - | - | - | - | - | - | V | P | S | - |
| 129 UniRef90\_UPI00160A92C2\_5\_284 | - | - | - | - | - | - | - | - | - | - | - | - | - | - | - | - | - | - | - | - | - | - | - | - | - | - | - | - | - | L | K | R | R | T | V | R | V | N | - | G | I | R | M | H | F | A | E | A | G | - |
| 130 UniRef90\_UPI0009E804D9\_8\_293 | - | - | - | - | - | - | - | - | - | - | - | - | - | P | G | T | - | - | - | - | - | D | Y | H | W | L | P | A | G | F | T | V | H | E | V | D | T | N | - | G | T | T | I | S | A | A | V | G | G | - |
| 131 UniRef90\_UPI00049086FA\_57\_348 | - | - | - | - | - | - | - | - | - | - | - | - | - | - | - | - | - | - | - | - | - | - | - | - | E | F | N | L | D | F | R | H | C | F | S | T | V | D | - | G | V | Q | M | H | Y | V | I | G | G | - |
| 132 UniRef90\_A5FF96\_16\_286 | - | - | - | - | - | - | - | - | - | - | - | - | - | - | - | - | - | - | - | - | - | - | - | - | - | - | - | - | - | F | K | N | N | Y | T | T | V | N | - | G | V | N | L | H | Y | V | T | G | G | - |
| 133 UniRef90\_UPI0016621A44\_4\_265 | - | - | - | - | - | - | - | - | - | - | - | - | - | - | - | - | - | - | - | - | - | - | - | - | - | - | - | - | - | - | - | - | - | - | - | - | - | - | - | - | - | - | - | - | - | - | S | G | G | - |
| 134 UniRef90\_UPI000B83B944\_2\_264 | - | - | - | - | - | - | - | - | - | - | - | - | - | - | - | - | - | - | - | - | - | - | - | - | - | - | - | - | - | - | - | - | - | - | - | - | - | - | - | - | - | - | - | - | - | - | - | - | - | - |
| 135 UniRef90\_A0A0B4DHQ5\_15\_286 | - | - | - | - | - | - | - | - | - | - | - | - | - | - | - | - | - | - | - | - | - | - | - | - | - | - | - | - | - | - | - | - | - | - | - | - | - | N | - | G | I | R | Q | N | Y | I | D | A | G | - |
| 136 UniRef90\_UPI0009B31CF7\_18\_283 | - | - | - | - | - | - | - | - | - | - | - | - | - | - | - | - | - | - | - | - | - | - | - | - | - | - | - | - | - | - | - | - | - | - | - | - | - | - | - | - | - | - | H | Q | V | T | V | A | G | - |
| 137 UniRef90\_UPI001661B99F\_34\_324 | - | - | - | - | - | - | - | - | - | - | - | - | - | - | - | - | - | - | - | - | A | T | P | P | G | G | L | P | G | F | T | D | G | Y | A | T | S | D | - | G | L | R | I | H | Y | A | V | A | G | - |
| 138 UniRef90\_UPI00104123D8\_9\_274 | - | - | - | - | - | - | - | - | - | - | - | - | - | - | - | - | - | - | - | - | - | - | - | - | - | - | - | - | - | - | - | - | - | H | L | P | V | G | - | D | L | S | L | H | V | V | E | A | G | - |
| 139 UniRef90\_A0A1M7Z6I5\_10\_283 | - | - | - | - | - | - | - | - | - | - | - | - | - | - | - | - | - | - | - | - | - | - | - | - | - | - | - | - | - | - | - | - | - | - | - | R | A | N | - | G | I | R | Q | H | Y | L | E | A | G | - |
| 140 UniRef90\_A0A431M1N9\_22\_292 | - | - | - | - | - | - | - | - | - | - | - | - | - | - | - | - | - | - | - | - | - | - | - | - | P | F | P | S | S | F | H | A | Q | Q | M | V | V | S | - | D | G | T | Q | Y | V | R | I | G | G | - |
| 141 UniRef90\_UPI0013D28676\_49\_329 | - | - | - | - | - | - | - | - | - | - | - | - | - | - | - | - | - | - | - | - | - | - | - | - | - | - | - | - | - | F | Q | D | G | Y | A | T | S | G | - | V | V | R | V | H | Y | V | I | G | G | - |
| 142 UniRef90\_A0A402BD85\_4\_199 | - | - | - | - | - | - | - | - | - | - | - | - | - | - | - | - | - | - | - | - | - | - | - | - | - | - | - | - | - | - | - | - | - | - | - | - | - | - | - | G | I | H | M | H | Y | V | S | G | G | - |
| 143 UniRef90\_A0A2V5S4I0\_55\_307 | - | - | - | - | - | - | - | - | - | - | - | - | - | P | - | - | S | - | - | - | - | - | - | H | G | A | P | A | E | I | A | S | R | T | A | S | I | D | - | G | G | Q | L | H | Y | L | I | A | G | - |
| 144 UniRef90\_A0A328AN40\_21\_303 | - | - | - | - | - | - | - | - | - | - | - | - | - | - | - | - | - | - | - | - | - | - | - | - | - | - | - | - | E | F | Q | T | R | K | V | R | A | G | - | A | L | D | L | N | V | A | E | A | G | P |
| 145 UniRef90\_A0A0M9AP20\_25\_244 | - | - | - | - | - | - | - | - | - | - | - | - | - | - | - | - | - | - | - | - | - | - | - | - | - | - | - | - | - | - | - | - | E | L | A | D | T | N | - | G | V | R | L | H | T | V | T | A | G | P |
| 146 UniRef90\_A0A1N6RLL9\_1\_202 | - | - | - | - | - | - | - | - | - | - | - | - | - | - | - | - | - | - | - | - | - | - | - | - | - | - | - | - | - | - | - | - | - | - | - | - | - | - | - | - | - | - | - | - | - | - | - | - | - | - |
| 147 UniRef90\_A0A4R3LTR2\_48\_312 | - | - | - | - | - | - | - | - | - | - | - | - | - | - | - | - | - | - | - | - | - | - | - | - | - | - | - | - | - | - | - | - | - | - | - | - | - | - | - | - | - | - | - | - | - | - | - | - | G | - |
| 148 UniRef90\_UPI0002ED95C2\_14\_285 | - | - | - | - | - | - | - | - | - | - | - | - | - | - | - | - | - | - | - | - | - | - | - | - | - | - | - | - | - | - | - | - | - | - | - | P | V | G | - | D | V | A | L | N | V | G | V | A | G | - |
| 149 UniRef90\_A0A1Q7MBV0\_16\_212 | - | - | - | - | - | - | - | - | - | - | - | - | - | - | - | - | - | - | - | - | - | - | - | - | - | - | - | - | - | - | - | - | - | - | - | - | A | G | - | D | V | R | L | H | C | A | A | I | G | P |
| 150 UniRef90\_A0A1A0KI05\_7\_212 | - | - | - | - | - | - | - | - | - | - | - | - | - | - | - | - | - | - | - | - | - | - | - | - | - | - | - | - | - | - | - | - | - | - | A | A | V | D | - | G | F | R | L | A | F | D | R | F | G | - |

  
  

|  |  |  |  |  |  |  |  |  |  |  |  |  |  |  |  |  |  |  |  |  |  |  |  |  |  |  |  |  |  |  |  |  |  |  |  |  |  |  |  |  |  |  |  |  |  |  |  |  |  |  |
| --- | --- | --- | --- | --- | --- | --- | --- | --- | --- | --- | --- | --- | --- | --- | --- | --- | --- | --- | --- | --- | --- | --- | --- | --- | --- | --- | --- | --- | --- | --- | --- | --- | --- | --- | --- | --- | --- | --- | --- | --- | --- | --- | --- | --- | --- | --- | --- | --- | --- | --- |
| **001 Input\_protein\_seq** | - | - | - | - | - | - | - | - | D | D | - | - | - | - | - | - | - | - | G | E | M | - | V | V | L | L | H | G | W | P | Q | T | W | Y | T | W | R | H | V | M | P | A | L | A | - | E | D | G | Y | R |
| 002 UniRef90\_UPI00158A2D6D\_3\_306 | - | - | - | - | - | - | - | - | D | D | - | - | - | - | - | - | - | - | G | E | L | - | V | I | L | L | H | G | W | P | Q | T | W | Y | T | W | R | H | V | M | P | A | L | A | - | E | E | G | Y | R |
| 003 UniRef90\_A0A1Z4J856\_27\_318 | - | - | - | - | - | - | - | - | - | K | - | - | - | - | - | - | - | - | G | E | P | - | L | V | L | L | H | G | F | P | T | T | W | Y | E | W | R | H | V | M | P | T | L | A | - | E | R | - | Y | T |
| 004 UniRef90\_G7LVZ3\_7\_288 | - | - | - | - | - | - | - | - | - | S | - | - | - | - | - | - | - | - | G | D | P | - | L | V | L | L | H | G | W | P | Q | S | W | R | E | W | H | H | L | I | P | I | L | A | - | S | R | - | F | T |
| 005 UniRef90\_A0A2I8F4B9\_53\_324 | - | - | - | - | - | - | G | D | K | K | - | - | - | - | - | - | - | - | G | T | P | - | V | V | L | L | H | G | W | G | S | T | S | Y | M | W | R | Y | V | M | P | Q | L | V | - | A | R | G | Y | T |
| 006 UniRef90\_A0A327RPK7\_10\_284 | - | - | - | - | - | - | - | - | - | S | - | - | - | - | - | - | - | - | G | D | P | - | I | V | L | L | H | G | W | P | Q | T | W | Y | E | W | K | D | I | I | P | T | L | A | - | K | S | - | Y | T |
| 007 UniRef90\_A0A6L3SWG4\_20\_294 | - | - | - | - | - | - | - | - | - | A | - | - | - | - | - | - | - | - | G | D | R | T | V | M | L | V | H | G | Y | P | Q | T | A | Y | A | W | R | R | V | V | P | L | L | V | - | Q | A | G | L | R |
| 008 UniRef90\_A0A401ZLI5\_4\_287 | - | - | - | - | - | - | - | - | - | Q | - | - | - | - | - | - | - | - | G | S | P | - | V | L | L | L | H | G | W | P | Q | T | W | Y | A | W | R | K | I | M | P | A | L | A | - | E | K | - | Y | T |
| 009 UniRef90\_A0A4V2U6R1\_12\_297 | S | P | - | - | - | R | D | G | Q | D | - | - | - | - | - | - | - | - | V | R | T | - | M | V | L | L | H | G | W | P | Q | T | W | W | E | W | R | H | I | I | E | P | L | R | - | A | D | G | W | F |
| 010 UniRef90\_A0A5A5T922\_6\_282 | - | - | - | - | - | - | - | - | - | N | - | - | - | - | - | - | - | - | G | A | P | - | L | V | L | L | H | G | W | P | Q | T | W | F | A | W | R | H | M | I | P | L | L | A | - | Q | H | - | Y | T |
| 011 UniRef90\_A0A2V6UIN9\_3\_273 | - | - | - | - | - | - | - | - | - | K | - | - | - | - | - | - | - | - | N | E | P | - | I | I | L | L | H | G | Y | A | Q | T | S | H | M | W | R | P | L | I | P | E | L | A | - | K | T | - | H | T |
| 012 UniRef90\_UPI001669B3C5\_41\_314 | - | - | - | - | - | - | - | - | - | K | - | - | - | - | - | - | - | - | G | S | P | - | V | V | L | I | H | G | Y | T | D | T | G | Y | M | W | T | P | V | M | P | D | L | A | - | K | N | - | H | T |
| 013 UniRef90\_A0A4R2Z7A8\_3\_305 | - | - | - | - | - | - | - | - | - | N | - | - | - | - | - | - | P | D | G | E | V | - | L | L | L | L | A | G | F | P | Q | S | W | F | A | W | R | H | V | M | Q | D | L | K | - | D | T | - | F | W |
| 014 UniRef90\_A0A5C5SVV1\_11\_281 | - | - | - | - | - | - | - | - | - | Q | - | - | - | - | - | - | - | - | G | A | P | - | L | V | L | L | H | G | F | P | Q | T | W | W | E | W | R | K | M | I | P | L | L | A | - | P | H | - | H | T |
| 015 UniRef90\_L9WLS3\_16\_284 | - | - | - | - | - | - | - | - | - | S | - | - | - | - | - | - | - | - | G | P | P | - | L | V | L | L | H | G | W | P | Q | T | W | Y | E | W | R | D | V | I | P | S | F | A | - | A | E | - | H | T |
| 016 UniRef90\_UPI00131EC7B5\_30\_311 | - | - | - | - | - | - | - | - | - | N | - | - | - | - | - | - | P | D | G | E | T | - | V | V | L | L | A | G | F | P | Q | S | W | Y | A | W | R | S | V | M | Q | Q | L | A | - | P | T | - | Y | R |
| 017 UniRef90\_C7QAM5\_50\_332 | - | - | - | - | - | - | - | - | - | H | - | - | - | - | - | - | - | - | G | P | T | - | L | V | L | L | H | G | Y | P | Q | T | W | Y | E | W | R | D | V | M | P | A | L | A | - | E | H | - | Y | T |
| 018 UniRef90\_A0A1Q3SXP6\_11\_288 | - | - | - | - | - | - | - | - | - | K | - | - | - | - | - | - | - | - | G | N | P | - | I | V | L | L | H | G | Y | T | Q | T | S | H | M | W | L | P | L | I | E | T | L | A | - | K | T | - | H | T |
| 019 UniRef90\_UPI00149248BF\_39\_315 | - | - | - | - | - | - | - | - | - | K | - | - | - | - | - | - | - | - | G | Q | Q | T | L | V | L | I | P | G | W | P | Q | T | W | Y | A | W | R | K | I | M | P | E | L | A | - | K | T | - | Y | R |
| 020 UniRef90\_UPI000361D127\_26\_313 | - | - | - | - | - | - | - | - | - | R | - | - | - | - | - | - | - | - | G | P | A | - | L | L | L | I | G | G | W | P | Q | F | W | Y | Q | W | R | H | V | M | L | P | L | A | - | E | Q | - | Y | T |
| 021 UniRef90\_A0A0M4FVH2\_24\_312 | - | - | - | - | - | - | - | - | - | K | - | - | - | - | - | - | D | D | G | D | V | - | V | V | L | L | P | G | F | P | E | S | W | F | T | W | H | K | V | M | P | L | L | A | - | P | T | - | Y | K |
| 022 UniRef90\_A0A1Q4ZL08\_9\_290 | - | - | - | - | - | - | - | - | - | Q | - | - | - | - | - | - | - | - | G | G | T | - | L | V | L | L | H | G | Y | P | Q | T | W | F | M | W | R | K | V | L | P | E | L | A | - | K | R | - | Y | T |
| 023 UniRef90\_S3CY91\_25\_307 | Y | P | D | K | P | D | R | T | E | E | - | - | - | - | - | - | - | - | T | N | T | - | I | L | L | I | H | G | F | P | N | T | S | Y | Q | W | R | H | V | I | T | P | L | A | - | K | A | G | Y | R |
| 024 UniRef90\_UPI0010F95BB2\_18\_294 | - | - | - | - | - | - | - | - | Q | D | - | - | - | - | - | - | - | - | A | E | A | - | I | L | L | L | H | G | W | P | Q | T | S | Y | A | W | R | H | V | A | P | L | L | A | - | R | A | G | Y | R |
| 025 UniRef90\_A0A0N1GDE2\_28\_316 | - | - | - | - | - | - | - | - | - | D | - | - | - | - | - | - | - | - | G | P | P | - | L | L | L | V | G | G | W | P | Q | T | W | Y | A | W | R | E | V | M | P | A | L | A | - | R | E | - | H | T |
| 026 UniRef90\_A0A2E5PJQ7\_5\_284 | - | - | - | - | - | - | - | - | - | R | - | - | - | - | - | - | - | - | G | P | P | - | V | I | L | L | H | G | W | P | E | T | W | Y | E | W | R | H | I | I | P | K | L | A | - | P | Y | - | Y | T |
| 027 UniRef90\_A0A1M7IC97\_44\_328 | - | - | - | - | - | - | - | - | - | H | - | - | - | - | - | - | - | - | G | P | T | - | L | M | L | I | H | G | Y | P | Q | T | W | Y | E | W | H | G | I | M | S | A | L | A | - | E | H | - | Y | T |
| 028 UniRef90\_UPI0016149F5E\_5\_284 | - | - | - | - | - | - | - | - | - | S | - | - | - | - | - | - | - | - | G | K | P | - | V | L | L | I | P | G | W | P | Q | T | W | Y | A | W | R | H | V | M | A | A | L | A | - | S | Q | G | Y | Q |
| 029 UniRef90\_UPI00156E1DFF\_8\_289 | - | - | - | - | - | - | - | - | - | S | - | - | - | - | - | - | - | - | G | E | P | - | V | L | L | L | H | G | W | P | Q | T | W | R | A | W | R | Y | L | M | P | T | L | A | - | E | H | G | Y | R |
| 030 UniRef90\_A0A3A8HRU0\_15\_300 | - | - | - | - | - | - | - | - | - | K | - | - | - | - | - | - | - | - | G | S | P | - | V | L | L | L | H | G | Y | T | Q | T | H | L | M | W | W | R | L | A | P | E | L | A | - | K | Q | - | H | T |
| 031 UniRef90\_A0A0M3UDU6\_18\_309 | - | - | - | - | - | - | - | - | - | E | - | - | - | - | - | - | - | - | G | P | P | - | V | L | L | I | P | G | W | P | Q | S | W | Y | A | W | R | Y | V | M | P | Q | L | V | - | D | A | G | Y | R |
| 032 UniRef90\_A0A447J1A9\_28\_317 | - | - | - | - | - | - | - | - | - | K | - | - | - | - | - | - | - | - | G | E | P | - | V | L | L | I | P | G | W | P | Q | T | W | Y | T | W | R | Y | V | M | T | G | L | A | - | A | Q | G | Y | M |
| 033 UniRef90\_A0A5C8T429\_14\_295 | - | - | - | - | - | - | - | - | - | Q | - | - | - | - | - | - | - | - | G | A | P | - | V | L | L | V | P | G | W | P | Q | T | W | Y | A | W | R | H | V | M | P | L | L | A | - | K | R | - | F | T |
| 034 UniRef90\_A0A0N0TCD7\_1\_288 | - | - | - | - | - | - | - | - | - | D | - | - | - | - | - | - | - | - | G | P | P | - | L | L | L | L | P | G | W | P | Q | F | W | Y | S | W | R | L | V | M | P | A | L | A | - | E | H | - | F | T |
| 035 UniRef90\_W9ARP2\_16\_295 | P | - | - | - | - | - | - | - | - | E | - | - | - | - | - | - | P | - | T | M | T | - | V | V | L | L | H | G | A | P | Q | T | R | Y | E | W | R | K | V | M | M | P | L | A | - | A | A | G | Y | R |
| 036 UniRef90\_A0A109IGY4\_11\_301 | - | - | - | - | - | - | - | - | - | D | - | - | - | - | - | - | - | - | G | P | P | - | L | L | L | V | G | G | W | P | Q | T | W | Y | A | W | R | E | V | M | P | A | L | A | - | R | T | - | H | T |
| 037 UniRef90\_K9DQK6\_24\_306 | - | - | - | - | - | - | - | - | - | K | - | - | - | - | - | - | - | - | G | P | A | - | V | V | L | I | H | G | F | G | D | T | G | D | M | W | G | P | L | A | A | R | L | A | - | R | N | - | H | T |
| 038 UniRef90\_A0A1Q8IR73\_6\_294 | - | - | - | - | - | - | - | - | - | A | - | - | - | - | - | - | N | A | E | R | T | - | I | V | L | I | A | G | F | P | E | S | W | Y | A | W | R | K | V | M | P | L | L | G | - | S | R | - | F | R |
| 039 UniRef90\_A0A1Y6D2S0\_29\_302 | - | - | - | - | - | - | - | - | - | K | - | - | - | - | - | - | - | - | G | P | L | - | L | L | L | L | H | G | W | P | Q | T | W | Y | E | W | H | R | I | M | P | M | L | A | - | G | K | - | Y | T |
| 040 UniRef90\_E6WJ64\_19\_301 | - | - | - | - | - | - | - | - | - | H | - | - | - | - | - | - | P | Q | G | D | V | - | L | L | L | L | A | G | F | P | Q | S | W | Y | A | W | H | Q | V | M | A | Q | L | A | - | D | R | - | Y | F |
| 041 UniRef90\_UPI000489A94A\_43\_316 | - | - | - | - | - | - | - | - | - | Q | Q | A | D | R | E | S | S | D | A | T | A | - | I | V | L | L | H | G | Y | T | E | T | S | H | M | W | R | P | L | M | T | R | L | A | - | A | S | - | G | P |
| 042 UniRef90\_A0A4R6HBQ4\_11\_285 | - | - | - | - | - | - | - | - | E | H | - | - | - | - | - | - | - | - | G | E | T | - | V | L | L | L | H | G | W | P | Q | T | S | H | A | W | R | K | V | I | P | L | L | A | - | A | A | G | F | R |
| 043 UniRef90\_A0A1M7QJX9\_32\_311 | - | - | - | - | - | - | - | - | - | K | - | - | - | - | - | - | - | - | G | E | P | - | L | L | L | V | H | G | F | G | Q | N | W | Y | M | W | N | R | L | L | P | E | L | S | - | K | H | - | F | T |
| 044 UniRef90\_A0A5B8WA72\_22\_300 | - | - | - | - | - | - | - | - | - | K | - | - | - | - | - | - | - | - | G | E | P | - | L | L | L | I | H | G | F | G | Q | N | W | Y | M | W | N | R | L | L | P | E | L | S | - | K | H | - | Y | T |
| 045 UniRef90\_A0A0G2FGE6\_47\_303 | - | - | - | - | - | - | - | - | - | T | - | - | - | - | - | - | - | - | - | - | - | - | I | L | L | I | H | G | Y | P | Q | C | S | Y | Q | F | R | H | V | I | T | P | I | A | - | D | A | G | Y | T |
| 046 UniRef90\_A0A1I6BK09\_10\_296 | - | - | - | - | - | - | - | - | - | E | P | R | - | - | - | - | P | G | R | A | P | - | L | V | L | L | H | G | F | P | Q | H | S | H | M | W | R | R | L | M | P | A | L | A | - | E | H | - | Y | L |
| 047 UniRef90\_A0A4R7C9R7\_33\_310 | - | - | - | - | - | - | - | - | - | Q | - | - | - | - | - | - | - | - | G | P | T | - | V | I | L | L | H | G | W | P | Q | T | W | F | A | W | H | G | Q | I | E | R | L | A | - | R | R | - | F | R |
| 048 UniRef90\_A0A2M9M9Q1\_18\_305 | - | - | - | - | - | - | - | - | - | T | - | - | - | - | - | - | - | - | G | A | P | - | L | L | L | V | G | G | W | P | Q | N | W | Y | V | W | R | F | V | M | P | R | L | A | - | E | R | - | F | R |
| 049 UniRef90\_A0A4V3T343\_11\_295 | - | - | - | - | - | - | - | - | - | A | - | - | - | - | - | - | - | - | G | R | P | - | L | V | L | L | G | G | W | P | Q | T | W | W | Q | W | H | K | V | M | P | A | L | A | - | R | T | - | H | R |
| 050 UniRef90\_UPI001616534D\_11\_303 | - | - | - | - | - | - | - | - | - | A | - | - | - | - | - | - | - | - | G | E | P | - | L | L | L | L | G | G | W | P | Q | T | W | W | Q | W | N | K | V | M | P | A | L | A | - | R | R | - | H | R |
| 051 UniRef90\_J4PG95\_17\_290 | - | - | - | - | - | - | - | - | - | D | - | - | - | - | - | - | - | - | G | P | P | - | V | L | L | I | P | G | W | P | Q | T | W | F | T | W | R | H | I | M | K | S | L | A | - | E | A | G | Y | T |
| 052 UniRef90\_A0A0T1T741\_1\_295 | - | - | - | - | - | - | - | - | - | D | - | - | - | - | - | - | - | - | G | P | A | - | L | L | L | I | A | G | W | P | Q | T | W | Y | A | W | R | E | V | M | P | A | L | A | - | R | R | - | H | T |
| 053 UniRef90\_G0FSK7\_17\_310 | - | - | - | - | - | - | - | - | - | D | - | - | - | - | - | - | - | - | G | P | P | - | L | L | L | I | P | G | W | P | Q | T | W | Y | A | W | R | L | L | M | P | E | L | A | - | R | D | - | F | T |
| 054 UniRef90\_UPI00164AAB2D\_18\_303 | - | - | - | - | - | - | - | - | - | S | - | - | - | - | - | - | - | - | G | P | A | - | V | V | L | L | H | G | F | G | D | T | G | D | M | W | T | P | L | A | I | A | L | A | - | K | D | - | H | K |
| 055 UniRef90\_A0A1I2MT44\_20\_306 | - | - | - | - | - | - | - | - | - | E | - | - | - | - | - | - | - | - | G | P | P | - | L | L | L | L | A | G | W | P | Q | T | W | Y | A | W | R | L | V | M | P | A | L | A | - | R | E | - | F | R |
| 056 UniRef90\_A0A2P2CCU5\_19\_306 | - | - | - | - | - | - | - | - | - | E | - | - | - | - | - | - | - | - | G | P | P | - | L | L | L | V | H | G | W | P | E | N | W | Y | A | W | R | H | V | M | P | D | L | A | - | Q | R | - | H | T |
| 057 UniRef90\_A0A2V9H5C8\_12\_298 | - | - | - | - | - | - | - | - | - | H | - | - | - | - | - | - | - | - | G | P | A | - | L | I | L | L | H | G | Y | A | E | T | S | R | M | W | T | P | I | L | P | L | F | G | - | E | K | - | F | T |
| 058 UniRef90\_A0A0P4V0Y0\_7\_291 | - | - | - | - | - | - | - | - | - | S | - | - | - | - | - | - | - | - | G | E | P | - | I | L | L | W | H | G | F | L | E | T | W | Y | C | W | R | K | I | M | P | A | L | A | - | E | R | - | Y | T |
| 059 UniRef90\_A0A0Q4UQ58\_27\_305 | - | - | - | - | - | - | - | - | - | S | - | - | - | - | - | - | - | - | G | P | P | - | L | V | L | L | H | G | W | P | Q | T | W | A | A | W | K | A | T | M | A | A | L | S | - | D | R | - | F | T |
| 060 UniRef90\_A0A6M4IM28\_20\_309 | - | - | - | - | - | - | - | - | - | S | - | - | - | - | - | - | - | - | G | P | A | - | V | L | L | L | H | G | Y | G | E | T | G | D | M | W | S | P | L | A | A | E | L | V | - | T | N | - | H | T |
| 061 UniRef90\_A0A537J419\_20\_301 | - | - | - | - | - | - | - | - | - | H | - | - | - | - | - | - | - | - | G | A | P | - | L | I | L | L | H | G | Y | A | E | T | S | R | M | W | R | P | I | I | P | V | L | A | - | K | R | - | F | M |
| 062 UniRef90\_A0A1V2PGN8\_6\_275 | - | - | - | - | - | - | - | - | - | D | - | - | - | - | - | - | - | - | G | P | P | - | V | V | L | L | H | G | W | P | Q | T | S | H | C | W | H | K | I | F | G | A | L | A | - | E | E | - | H | T |
| 063 UniRef90\_A0A542JC81\_20\_314 | - | - | - | - | - | - | - | - | - | Q | - | - | - | - | - | - | - | - | G | P | A | - | L | L | L | V | H | G | W | P | E | T | W | Y | A | W | R | L | V | M | P | E | L | A | - | K | E | - | F | T |
| 064 UniRef90\_UPI000424F353\_33\_308 | - | - | - | - | - | - | - | - | - | K | - | - | - | - | - | - | - | - | G | P | A | - | V | V | L | L | H | G | Y | G | E | T | G | D | M | W | E | P | L | A | V | K | L | A | - | S | N | - | H | T |
| 065 UniRef90\_A0A2V9W2E9\_22\_299 | - | - | - | - | - | - | - | - | - | K | - | - | - | - | - | - | - | - | G | S | V | - | V | L | L | I | H | G | Y | A | E | N | S | D | S | W | A | P | L | A | G | D | L | M | - | K | D | - | H | T |
| 066 UniRef90\_A0A2T6L0M5\_16\_299 | - | - | - | - | - | - | - | - | - | S | - | - | - | - | - | - | - | - | G | S | P | - | V | L | L | L | H | G | F | P | Q | T | W | A | E | W | H | H | Q | L | G | P | L | A | - | A | D | - | H | T |
| 067 UniRef90\_A0A261TYY2\_43\_315 | - | - | - | - | - | - | - | - | - | Q | - | - | - | - | - | - | - | - | G | E | P | - | V | L | L | V | P | G | W | P | Q | T | W | Y | A | W | R | K | T | M | R | A | L | A | - | R | H | - | Y | T |
| 068 UniRef90\_UPI0009781B11\_10\_287 | - | - | - | - | - | - | - | - | - | N | - | - | - | - | - | - | - | - | G | P | L | - | L | V | L | V | S | G | W | P | Q | T | W | L | A | W | R | K | V | I | P | R | L | A | - | D | T | - | F | R |
| 069 UniRef90\_A0A2V9TPC1\_35\_307 | - | - | - | - | - | - | - | - | - | H | - | - | - | - | - | - | - | - | G | A | P | - | L | I | L | L | H | G | Y | A | E | T | S | L | M | W | K | P | I | I | P | V | L | A | - | E | R | - | F | T |
| 070 UniRef90\_UPI00145FA88A\_6\_279 | - | - | - | - | - | - | - | - | - | D | - | - | - | - | - | - | - | - | G | P | P | - | L | V | L | L | H | G | W | P | Q | T | S | H | C | W | R | F | I | A | G | P | L | C | - | E | T | - | H | T |
| 071 UniRef90\_UPI0003FADEBE\_21\_306 | - | - | - | - | - | - | - | - | - | S | - | - | - | - | - | - | - | - | G | P | A | - | V | V | L | L | H | G | F | G | D | T | G | D | M | W | A | L | M | A | A | A | L | A | - | R | D | - | H | R |
| 072 UniRef90\_A0A2V8Y3P4\_78\_329 | - | - | - | - | - | - | - | - | - | S | - | - | - | - | - | - | - | - | G | P | V | - | V | V | L | L | H | G | Y | A | E | N | S | D | S | W | A | P | L | A | A | D | L | I | - | T | D | - | H | T |
| 073 UniRef90\_A0A285BKA7\_8\_299 | - | - | - | - | - | - | - | - | - | D | - | - | - | - | - | - | - | - | G | P | P | - | L | L | L | L | P | G | W | P | Q | F | W | Y | S | W | R | L | V | M | P | A | L | A | - | E | H | - | F | T |
| 074 UniRef90\_A0A0K3AVW3\_44\_319 | - | - | - | - | - | - | - | - | - | R | - | - | - | - | - | - | - | - | G | R | P | - | L | V | L | V | P | G | W | P | Q | T | W | W | E | F | H | K | I | M | P | A | L | A | - | T | R | - | Y | R |
| 075 UniRef90\_A0A4D7B7N1\_7\_276 | - | - | - | - | - | - | - | - | - | S | - | - | - | - | - | - | - | - | G | A | P | - | V | V | L | L | A | G | F | P | Q | S | W | Y | A | W | R | R | V | M | P | L | I | A | - | E | R | - | H | K |
| 076 UniRef90\_A0A2U0WF37\_17\_293 | - | - | - | - | - | - | - | - | - | K | - | - | - | - | - | - | - | - | G | P | A | - | V | V | L | L | H | G | F | G | D | T | G | D | M | W | A | P | L | A | A | D | L | V | - | K | D | - | H | T |
| 077 UniRef90\_UPI00135C2D49\_17\_309 | - | - | - | - | - | - | - | - | - | A | - | - | - | - | - | - | - | - | G | D | P | - | L | L | L | L | G | G | W | P | Q | T | W | W | Q | W | H | K | V | M | P | A | L | A | - | R | R | - | R | R |
| 078 UniRef90\_UPI001473614E\_45\_322 | - | - | - | - | - | - | - | - | - | S | - | - | - | - | - | - | - | - | G | P | A | - | V | V | L | L | H | G | W | P | Q | T | W | L | M | W | R | K | L | M | P | D | L | A | - | R | D | - | H | T |
| 079 UniRef90\_J3F862\_22\_299 | - | - | - | - | - | - | - | - | - | K | - | - | - | - | - | - | - | - | G | P | A | - | V | V | L | L | H | G | F | G | D | T | G | D | M | W | A | P | L | A | A | D | L | A | - | K | D | - | H | T |
| 080 UniRef90\_A0A1A9NC01\_62\_345 | - | - | - | - | - | - | - | - | - | K | - | - | - | - | - | - | - | - | G | E | P | - | L | V | L | L | P | G | W | P | E | T | W | W | T | F | H | K | I | M | P | A | L | A | - | E | H | - | Y | T |
| 081 UniRef90\_UPI00131AC11B\_45\_327 | - | - | - | - | - | - | - | - | - | E | - | - | - | - | - | - | - | - | G | S | P | - | V | L | L | L | P | G | W | P | E | T | W | W | G | Y | H | K | I | M | P | E | L | A | - | K | T | - | H | R |
| 082 UniRef90\_A0A4R8HC20\_22\_306 | - | - | - | - | - | - | - | - | - | R | - | - | - | - | - | - | - | - | G | E | P | - | L | V | L | L | G | G | W | P | Q | T | W | W | Q | W | R | K | V | L | P | A | L | A | - | A | R | - | H | R |
| 083 UniRef90\_A0A1B2HH26\_4\_280 | - | - | - | - | - | - | - | - | - | E | - | - | - | - | - | - | - | - | G | P | L | - | V | V | L | V | H | G | W | P | F | S | W | I | E | F | R | E | L | L | P | L | M | A | - | A | R | G | F | S |
| 084 UniRef90\_A0A517LKB1\_23\_304 | P | - | - | - | - | - | - | S | Q | K | - | - | - | - | - | - | - | - | K | G | V | - | I | L | L | I | H | G | Y | P | E | S | S | Y | Q | F | R | H | V | I | P | L | L | A | - | T | A | G | Y | K |
| 085 UniRef90\_A0A0N1F3N8\_17\_298 | - | - | - | - | - | - | - | - | - | K | - | - | - | - | - | - | - | - | G | P | A | - | V | V | L | L | H | G | Y | G | E | T | G | D | M | W | G | A | M | A | A | D | L | A | - | R | D | - | H | T |
| 086 UniRef90\_UPI0004CCFD44\_55\_340 | - | - | - | - | - | - | - | - | - | S | - | - | - | - | - | - | - | - | G | P | P | - | V | V | L | V | H | G | W | P | Q | T | W | F | G | W | W | P | I | M | P | A | L | A | - | E | H | - | H | T |
| 087 UniRef90\_A0A4D4LDP7\_7\_304 | - | - | - | - | - | - | - | - | - | S | - | - | - | - | - | - | T | G | S | D | P | - | V | V | L | L | A | G | F | P | Q | T | W | Y | A | W | H | K | V | M | P | A | L | A | - | R | H | - | H | R |
| 088 UniRef90\_A0A2V4B948\_8\_279 | - | - | - | - | - | - | - | - | - | S | - | - | - | - | - | - | - | - | G | P | A | - | V | V | L | L | H | G | W | P | Q | T | W | Y | C | W | R | T | V | I | E | P | L | A | - | R | D | - | H | T |
| 089 UniRef90\_A0A2P2FV90\_3\_282 | - | - | - | - | - | - | - | - | - | E | - | - | - | - | - | - | - | - | G | P | A | - | L | F | L | L | H | G | W | P | Q | T | S | F | C | W | H | E | I | I | G | E | L | A | - | E | T | - | H | T |
| 090 UniRef90\_A0A4Q2J292\_17\_304 | - | - | - | - | - | - | - | - | - | H | - | - | - | - | - | - | - | - | G | P | A | - | V | V | M | L | H | G | Y | G | E | S | G | D | M | W | A | P | L | A | A | R | L | A | - | G | S | - | H | T |
| 091 UniRef90\_UPI0012B05B7D\_16\_287 | - | - | - | - | - | - | - | - | - | T | - | - | - | - | - | - | - | - | G | P | V | - | I | L | L | V | A | G | F | P | Q | S | C | Y | A | W | R | K | V | I | P | L | L | A | - | N | K | - | Y | R |
| 092 UniRef90\_UPI00048D4AD4\_4\_301 | - | - | - | - | - | - | - | - | - | S | - | - | - | - | - | - | - | - | G | A | P | - | L | L | L | L | G | G | W | P | Q | T | W | W | Q | W | N | K | V | M | P | A | L | A | - | R | H | - | H | R |
| 093 UniRef90\_UPI0015F81933\_16\_310 | - | - | - | - | - | - | - | - | - | R | - | - | - | - | - | - | P | S | G | P | A | - | V | V | L | L | A | G | F | P | Q | T | W | W | A | W | R | K | V | M | P | G | L | A | - | H | R | - | F | H |
| 094 UniRef90\_UPI00160CFF45\_55\_340 | - | - | - | - | - | - | - | - | - | S | - | - | - | - | - | - | - | - | G | P | P | - | V | V | L | L | H | G | W | P | Q | T | W | Y | G | W | W | P | I | M | P | E | L | A | - | K | H | - | H | T |
| 095 UniRef90\_A0A1Q8KTW7\_17\_294 | - | - | - | - | - | - | - | - | - | S | - | - | - | - | - | - | - | - | G | P | P | - | L | V | L | L | H | G | V | P | K | S | W | Y | Y | W | H | R | V | I | P | L | L | S | - | E | R | - | F | T |
| 096 UniRef90\_UPI00161A7EC4\_21\_297 | - | - | - | - | - | - | - | - | - | S | - | - | - | - | - | - | - | - | G | P | A | - | I | V | L | L | H | G | W | P | Q | T | W | W | I | W | R | N | V | M | P | E | L | A | - | R | T | - | H | T |
| 097 UniRef90\_A0A1B4WXA8\_16\_299 | - | - | - | - | - | - | - | - | - | K | - | - | - | - | - | - | - | - | G | P | A | - | V | V | L | L | H | G | F | G | D | T | G | D | M | W | A | P | L | A | A | D | L | A | - | R | D | - | H | T |
| 098 UniRef90\_UPI000E46D2C1\_48\_330 | - | - | - | - | - | - | - | - | - | E | - | - | - | - | - | - | - | - | G | P | P | - | V | I | M | M | P | G | W | P | Q | T | W | W | A | F | N | K | I | M | P | D | L | A | - | E | D | - | H | T |
| 099 UniRef90\_A0A2N5CFI4\_23\_293 | - | - | - | - | - | - | - | - | - | H | - | - | - | - | - | - | - | - | G | P | A | - | V | V | L | L | H | G | Y | G | E | T | G | D | M | W | I | P | L | A | T | K | L | A | - | V | N | - | H | T |
| 100 UniRef90\_A0A385B2U0\_45\_333 | - | - | - | - | - | - | - | - | - | S | - | - | - | - | - | - | - | - | G | S | P | - | V | V | L | L | H | G | F | P | Q | T | S | A | E | W | E | P | Q | L | E | A | L | A | - | K | D | - | H | T |
| 101 UniRef90\_A0A1Q7W147\_16\_308 | - | - | - | - | - | - | - | - | - | R | - | - | - | - | - | - | P | S | G | P | A | - | L | V | L | L | A | G | F | P | Q | T | W | W | A | W | R | K | V | M | P | S | L | A | - | G | R | - | F | R |
| 102 UniRef90\_A0A239MTD5\_1\_271 | - | - | - | - | - | - | - | - | - | Q | - | - | - | - | - | - | - | - | G | S | P | - | V | V | M | I | H | G | F | P | Q | T | W | A | E | W | R | Q | Q | M | G | P | L | S | - | R | T | - | H | T |
| 103 UniRef90\_UPI000561A6EF\_30\_315 | - | - | - | - | - | - | - | - | - | R | - | - | - | - | - | - | - | - | G | P | A | - | L | L | L | I | C | G | W | P | Q | T | W | Y | A | W | R | L | L | M | P | A | L | A | - | E | H | - | F | S |
| 104 UniRef90\_A0A4Y8RHA3\_28\_294 | - | - | - | - | - | - | - | - | - | E | - | - | - | - | - | - | - | - | G | P | A | - | V | V | L | L | H | G | Y | G | E | T | G | D | M | W | A | P | L | A | A | D | L | A | - | K | D | - | H | K |
| 105 UniRef90\_UPI0004DF415F\_15\_308 | - | - | - | - | - | - | - | - | - | R | - | - | - | - | - | - | P | G | G | P | A | - | V | V | L | L | A | G | F | P | Q | T | W | W | A | W | R | Q | T | M | P | G | L | A | - | E | R | - | F | R |
| 106 UniRef90\_A0A2N3KZL1\_30\_325 | - | - | - | - | - | - | - | - | - | S | - | - | - | - | - | - | - | - | G | P | L | - | A | F | L | V | H | G | F | G | Q | S | W | Y | E | W | H | Q | L | M | P | L | L | A | - | K | T | - | H | S |
| 107 UniRef90\_UPI00076E3DF2\_55\_340 | - | - | - | - | - | - | - | - | - | S | - | - | - | - | - | - | - | - | G | T | P | - | V | V | L | L | H | G | W | P | Q | T | W | F | G | W | W | S | I | M | P | E | L | A | - | E | H | - | H | T |
| 108 UniRef90\_UPI00130E1FA7\_66\_348 | - | - | - | - | - | - | - | - | - | Q | - | - | - | - | - | - | - | - | G | T | P | - | V | V | L | L | P | G | W | P | Q | T | W | W | A | F | H | Q | I | M | P | K | L | A | - | E | K | - | H | H |
| 109 UniRef90\_A0A1I3FUH2\_46\_328 | - | - | - | - | - | - | - | - | - | E | - | - | - | - | - | - | - | - | G | T | P | - | I | I | L | M | P | G | W | P | Q | T | W | W | A | F | H | K | I | M | P | A | L | A | - | Q | K | - | H | H |
| 110 UniRef90\_UPI000E2836BA\_28\_313 | - | - | - | - | - | - | - | - | - | E | - | - | - | - | - | - | - | - | G | T | P | - | L | V | L | L | P | G | W | P | E | T | W | W | E | Y | R | K | V | M | P | A | L | A | - | E | R | - | H | Q |
| 111 UniRef90\_A0A653WNW3\_25\_297 | - | - | - | - | - | - | - | - | - | N | - | - | - | - | - | - | - | - | G | P | A | - | V | V | L | L | H | G | Y | G | E | T | G | D | M | W | A | P | M | A | A | D | L | A | - | R | E | - | R | T |
| 112 UniRef90\_A0A5C1I8U5\_39\_321 | - | - | - | - | - | - | - | - | - | T | - | - | - | - | - | - | - | - | G | K | A | - | L | V | L | L | P | G | W | P | E | T | W | W | S | Y | S | K | I | M | P | A | L | A | - | S | K | - | Y | H |
| 113 UniRef90\_UPI00165059AC\_4\_267 | - | - | - | - | - | - | - | - | - | E | - | - | - | - | - | - | - | - | G | P | L | - | I | V | L | L | H | G | W | P | Q | T | G | H | C | W | R | H | L | M | G | P | L | S | - | E | N | - | H | T |
| 114 UniRef90\_F8JLL5\_12\_309 | - | - | - | - | - | - | - | - | - | R | - | - | - | - | - | - | P | G | G | P | A | - | V | V | L | L | A | G | F | P | Q | T | W | W | A | W | R | K | V | M | P | D | L | A | - | D | R | - | F | H |
| 115 UniRef90\_A0A329J6I4\_30\_314 | - | - | - | - | - | - | - | - | - | S | - | - | - | - | - | - | - | - | G | P | L | - | V | F | L | A | H | G | F | G | Q | A | W | Y | E | W | H | N | L | M | P | E | L | A | - | R | N | - | H | T |
| 116 UniRef90\_A0A3N1H0W7\_60\_340 | - | - | - | - | - | - | - | - | - | T | - | - | - | - | - | - | - | - | G | K | P | - | L | L | L | L | P | G | W | P | E | T | W | W | E | F | R | K | V | M | P | A | L | A | - | A | A | G | R | R |
| 117 UniRef90\_A0A316VRQ3\_1\_263 | - | - | - | - | - | - | - | - | - | - | - | - | - | - | - | - | - | - | - | - | - | - | I | L | L | I | H | G | Y | P | Q | T | V | Y | A | M | R | Y | L | L | P | L | F | A | - | E | K | G | F | F |
| 118 UniRef90\_A0A4P8X818\_19\_291 | - | - | - | - | - | - | - | - | - | S | - | - | - | - | - | - | - | - | G | E | P | - | L | M | L | L | H | G | V | P | K | T | S | Y | Y | W | R | K | V | L | P | L | L | S | - | S | K | - | F | T |
| 119 UniRef90\_UPI0008405A69\_11\_309 | - | - | - | - | - | - | - | - | - | T | - | - | - | - | - | - | - | - | G | E | P | - | V | I | L | L | G | G | W | P | Q | T | W | W | Q | F | R | K | I | M | P | E | L | A | - | L | R | - | H | E |
| 120 UniRef90\_A0A6I8M538\_3\_275 | - | - | - | - | - | - | - | - | - | E | - | - | - | - | - | - | - | - | G | P | P | - | L | F | L | L | H | G | W | P | Q | T | S | H | C | W | H | R | V | V | G | P | L | A | - | E | T | - | H | T |
| 121 UniRef90\_UPI000B5CCBFE\_21\_300 | - | - | - | - | - | - | - | - | - | S | - | - | - | - | - | - | - | - | G | E | P | - | V | V | L | L | H | G | W | P | E | T | W | W | A | Y | R | K | L | M | P | R | L | A | - | E | Q | - | Y | R |
| 122 UniRef90\_A0A0M9ZDW3\_1\_266 | - | - | - | - | - | - | - | - | - | Q | - | - | - | - | - | - | - | - | G | D | D | L | M | V | L | L | P | G | W | P | R | T | W | W | Q | F | R | K | L | M | P | K | L | A | - | E | R | - | F | R |
| 123 UniRef90\_UPI000E275A28\_18\_308 | - | - | - | - | - | - | - | - | - | E | - | - | - | - | - | - | - | - | G | P | P | - | L | L | L | L | T | G | W | P | Q | N | W | Y | A | W | R | D | M | M | L | P | L | A | - | K | H | - | Y | T |
| 124 UniRef90\_A0A3L8K0H7\_13\_298 | - | - | - | - | - | - | - | - | - | Q | - | - | - | - | - | - | - | - | G | S | P | - | L | I | L | L | P | G | W | P | E | T | W | W | E | Y | R | K | V | L | P | A | L | A | - | A | R | - | H | Q |
| 125 UniRef90\_A0A6N7ZB66\_18\_297 | - | - | - | - | - | - | - | - | - | S | - | - | - | - | - | - | - | - | G | P | V | - | L | V | L | L | H | G | W | P | Q | T | G | R | A | W | A | R | V | M | P | T | L | A | - | E | N | - | H | T |
| 126 UniRef90\_A0A2W7GM08\_52\_337 | - | - | - | - | - | - | - | - | - | Q | - | - | - | - | - | - | - | - | G | D | A | - | V | V | L | L | P | G | W | P | Q | T | W | W | E | Y | H | K | V | M | P | A | L | A | - | K | T | - | H | R |
| 127 UniRef90\_A0A258JI33\_23\_300 | - | - | - | - | - | - | - | - | - | K | - | - | - | - | - | - | - | - | G | P | A | - | V | V | L | L | H | G | F | G | D | T | G | D | M | W | A | P | A | A | R | A | L | Y | - | K | N | - | H | T |
| 128 UniRef90\_A0A6A6C1N5\_26\_297 | - | - | - | - | - | - | - | - | - | A | - | - | - | - | - | - | - | - | A | R | T | - | L | L | L | L | H | G | F | P | Q | T | S | H | Q | F | R | H | V | I | P | L | L | A | - | A | Q | G | Y | R |
| 129 UniRef90\_UPI00160A92C2\_5\_284 | - | - | - | - | - | - | - | - | - | D | - | - | - | - | - | - | - | - | G | P | P | - | V | V | L | L | H | G | Y | P | E | T | H | H | A | W | R | D | Q | W | P | E | L | A | - | R | D | - | Y | H |
| 130 UniRef90\_UPI0009E804D9\_8\_293 | - | - | - | - | - | - | - | - | - | R | - | - | - | - | - | - | - | - | G | P | T | - | M | I | L | L | H | G | W | P | Q | T | G | R | A | W | R | H | V | M | G | P | L | A | - | E | H | - | H | T |
| 131 UniRef90\_UPI00049086FA\_57\_348 | - | - | - | - | - | - | - | - | - | T | - | - | - | - | - | - | - | - | G | P | S | T | L | V | L | L | H | G | W | P | E | S | W | Y | E | Y | R | A | V | M | P | R | L | L | - | A | G | - | R | T |
| 132 UniRef90\_A5FF96\_16\_286 | - | - | - | - | - | - | - | - | - | Q | - | - | - | - | - | - | - | - | G | S | P | - | L | V | L | I | P | G | W | P | Q | T | W | W | S | Y | R | K | I | M | P | I | L | A | - | E | K | - | H | S |
| 133 UniRef90\_UPI0016621A44\_4\_265 | - | - | - | - | - | - | - | - | - | D | - | - | - | - | - | - | - | - | G | P | V | - | V | L | L | L | H | G | W | P | V | T | S | Y | H | W | R | L | T | V | P | A | L | A | - | E | A | G | F | R |
| 134 UniRef90\_UPI000B83B944\_2\_264 | - | - | - | - | - | - | - | - | - | - | - | - | - | - | - | - | - | - | - | - | - | - | - | F | L | L | P | G | W | P | Q | N | W | Y | A | Y | H | K | I | M | P | E | L | V | - | K | K | - | Y | H |
| 135 UniRef90\_A0A0B4DHQ5\_15\_286 | - | - | - | - | - | - | - | - | - | E | - | - | - | - | - | - | - | - | G | A | P | - | V | V | L | L | H | G | F | P | E | T | N | Y | A | W | R | H | Q | I | P | V | L | S | - | K | K | - | Y | R |
| 136 UniRef90\_UPI0009B31CF7\_18\_283 | - | - | - | - | - | - | - | - | - | S | - | - | - | - | - | - | - | - | G | Q | A | - | V | L | L | L | H | G | W | P | L | T | S | H | M | W | R | K | V | T | P | A | L | V | A | A | G | - | F | R |
| 137 UniRef90\_UPI001661B99F\_34\_324 | - | - | - | - | - | - | - | - | - | H | - | - | - | - | - | - | - | - | G | P | A | - | L | V | L | L | H | G | W | P | E | T | L | Q | T | W | E | Q | V | A | P | A | L | T | - | A | D | - | H | T |
| 138 UniRef90\_UPI00104123D8\_9\_274 | - | - | - | - | - | - | - | - | - | D | - | - | - | - | - | - | P | R | G | R | P | - | Y | L | L | L | H | G | W | P | E | S | W | R | T | W | E | G | V | M | D | A | - | A | - | T | D | - | A | R |
| 139 UniRef90\_A0A1M7Z6I5\_10\_283 | - | - | - | - | - | - | - | - | - | S | - | - | - | - | - | - | - | - | G | P | P | - | V | V | L | L | H | G | F | P | E | T | S | Y | A | W | R | H | Q | I | P | V | L | A | - | E | R | - | Y | R |
| 140 UniRef90\_A0A431M1N9\_22\_292 | - | - | - | - | - | - | - | - | - | K | - | - | - | - | - | - | - | - | G | P | A | - | V | L | L | L | H | G | F | G | D | T | G | D | M | W | E | P | L | A | K | V | L | V | - | K | D | - | H | T |
| 141 UniRef90\_UPI0013D28676\_49\_329 | - | - | - | - | - | - | - | - | - | H | - | - | - | - | - | - | - | - | G | P | A | - | L | V | L | L | H | G | W | P | E | T | W | Y | A | W | A | D | L | M | P | A | L | A | - | R | T | - | H | T |
| 142 UniRef90\_A0A402BD85\_4\_199 | - | - | - | - | - | - | - | - | - | N | - | - | - | - | - | - | - | - | G | Q | V | - | L | M | L | L | H | G | F | P | E | T | W | Y | A | W | H | K | V | L | P | D | L | A | - | K | H | - | Y | L |
| 143 UniRef90\_A0A2V5S4I0\_55\_307 | - | - | - | - | - | - | - | - | - | S | - | - | - | - | - | - | - | - | G | P | A | - | V | L | L | L | H | G | Y | T | Q | T | S | R | M | W | R | P | I | I | P | L | L | A | - | E | K | - | F | T |
| 144 UniRef90\_A0A328AN40\_21\_303 | - | - | - | - | - | - | - | - | V | D | - | - | - | - | - | - | - | - | G | P | L | - | V | I | L | L | H | G | F | P | E | F | W | F | E | W | R | E | L | I | G | P | L | A | - | E | A | G | F | R |
| 145 UniRef90\_A0A0M9AP20\_25\_244 | - | - | - | - | - | - | - | - | P | D | - | - | - | - | - | - | - | - | G | D | L | - | V | V | L | L | H | G | F | P | E | F | W | Y | A | W | K | H | Q | I | P | A | L | A | - | D | A | G | Y | R |
| 146 UniRef90\_A0A1N6RLL9\_1\_202 | - | - | - | - | - | - | - | - | - | - | - | - | - | - | - | - | - | - | - | - | - | - | - | - | - | - | - | - | - | - | - | - | - | - | - | - | - | - | - | - | - | - | - | - | - | - | - | - | - | - |
| 147 UniRef90\_A0A4R3LTR2\_48\_312 | - | - | - | - | - | - | - | - | - | D | - | - | - | - | - | - | - | - | G | P | P | - | L | L | L | L | H | G | H | P | Q | T | H | V | T | W | R | K | I | A | P | A | L | A | - | R | R | - | F | T |
| 148 UniRef90\_UPI0002ED95C2\_14\_285 | - | - | - | - | - | - | - | - | - | A | - | - | - | - | - | - | - | - | G | A | P | - | I | V | L | L | H | G | F | P | Q | T | H | L | A | W | R | H | V | A | T | D | L | A | - | R | D | - | H | L |
| 149 UniRef90\_A0A1Q7MBV0\_16\_212 | - | - | - | - | - | - | - | - | R | D | - | - | - | - | - | - | - | - | G | P | L | - | V | L | L | M | H | G | F | P | E | C | W | L | S | W | R | Y | Q | L | P | A | L | A | - | A | A | G | F | R |
| 150 UniRef90\_A0A1A0KI05\_7\_212 | - | - | - | - | - | - | - | - | - | S | - | - | - | - | - | - | P | G | A | Q | S | - | V | V | L | L | H | G | W | P | G | H | R | H | D | Y | R | L | V | A | P | D | L | A | - | R | V | - | A | D |

  
  

|  |  |  |  |  |  |  |  |  |  |  |  |  |  |  |  |  |  |  |  |  |  |  |  |  |  |  |  |  |  |  |  |  |  |  |  |  |  |  |  |  |  |  |  |  |  |  |  |  |  |  |
| --- | --- | --- | --- | --- | --- | --- | --- | --- | --- | --- | --- | --- | --- | --- | --- | --- | --- | --- | --- | --- | --- | --- | --- | --- | --- | --- | --- | --- | --- | --- | --- | --- | --- | --- | --- | --- | --- | --- | --- | --- | --- | --- | --- | --- | --- | --- | --- | --- | --- | --- |
| **001 Input\_protein\_seq** | V | V | A | V | D | Y | R | G | A | G | E | S | D | - | - | - | - | K | P | L | G | G | - | - | - | - | - | Y | D | K | A | S | M | A | G | D | I | R | A | L | V | - | H | Q | L | G | A | - | - | - |
| 002 UniRef90\_UPI00158A2D6D\_3\_306 | V | V | A | V | D | Y | R | G | A | G | E | S | D | - | - | - | - | K | P | L | G | G | - | - | - | - | - | Y | D | K | A | S | M | A | G | D | I | R | A | L | A | - | Q | Q | L | G | A | - | - | - |
| 003 UniRef90\_A0A1Z4J856\_27\_318 | V | I | V | P | D | M | R | G | L | G | D | S | S | - | - | - | - | K | P | L | T | G | - | - | - | - | - | Y | D | A | R | T | V | A | D | D | I | Y | Q | L | V | - | G | K | L | G | F | - | - | - |
| 004 UniRef90\_G7LVZ3\_7\_288 | V | I | A | P | D | M | R | G | F | G | D | S | D | - | - | - | - | K | P | T | G | G | - | - | - | - | - | Y | D | K | R | T | V | A | Q | D | I | R | Q | L | V | - | Q | H | L | G | F | - | - | - |
| 005 UniRef90\_A0A2I8F4B9\_53\_324 | V | L | A | P | D | L | R | G | L | G | D | T | S | - | - | - | - | K | P | A | T | G | - | - | - | - | - | Y | D | K | A | N | V | A | E | D | I | R | A | L | V | - | A | K | L | N | L | G | - | - |
| 006 UniRef90\_A0A327RPK7\_10\_284 | V | I | A | P | D | L | R | G | A | G | L | S | D | - | - | - | - | K | P | K | T | G | - | - | - | - | - | Y | D | K | L | T | L | A | Q | D | I | Y | L | L | V | - | E | Q | L | G | F | - | - | - |
| 007 UniRef90\_A0A6L3SWG4\_20\_294 | V | V | M | P | D | Y | R | G | A | G | G | S | S | - | - | - | - | K | P | S | G | G | - | - | - | - | - | Y | D | K | H | T | M | A | G | D | L | H | A | L | L | Y | E | H | L | G | L | T | - | - |
| 008 UniRef90\_A0A401ZLI5\_4\_287 | V | I | A | P | D | S | R | G | I | G | D | S | E | - | - | - | - | R | T | D | S | G | - | - | - | - | - | Y | D | A | S | T | L | A | E | D | T | F | S | L | V | - | R | S | L | G | F | - | - | - |
| 009 UniRef90\_A0A4V2U6R1\_12\_297 | V | V | A | P | D | Y | R | G | A | G | G | S | S | - | - | - | - | K | P | Q | A | G | - | - | - | - | - | Y | D | K | I | T | M | A | K | D | I | K | A | L | L | - | D | H | L | G | A | R | - | - |
| 010 UniRef90\_A0A5A5T922\_6\_282 | V | I | A | P | D | L | R | G | L | G | D | S | S | - | - | - | - | K | P | V | N | D | - | - | - | - | - | Y | D | K | R | T | I | A | E | D | V | Y | Q | L | T | - | L | Q | L | G | F | - | - | - |
| 011 UniRef90\_A0A2V6UIN9\_3\_273 | I | I | A | P | D | L | R | G | F | G | D | S | S | - | - | - | - | R | P | E | G | G | - | - | - | - | - | Y | D | K | K | T | M | A | Q | D | I | H | A | L | A | - | A | S | L | G | Y | - | - | - |
| 012 UniRef90\_UPI001669B3C5\_41\_314 | V | I | V | P | D | L | R | G | A | G | Q | S | E | - | - | - | - | K | A | A | G | G | - | - | - | - | - | Y | D | K | K | T | M | A | Q | D | I | H | A | L | V | - | K | S | L | G | Y | - | - | - |
| 013 UniRef90\_A0A4R2Z7A8\_3\_305 | L | V | A | P | D | L | P | G | Q | G | D | S | D | - | - | - | - | R | P | E | T | G | - | - | - | - | - | Y | D | T | Q | S | L | A | Q | K | T | H | E | L | M | - | Q | S | L | G | H | - | - | - |
| 014 UniRef90\_A0A5C5SVV1\_11\_281 | I | V | M | P | D | L | R | G | A | G | Q | S | D | - | - | - | - | C | P | Q | G | G | - | - | - | - | - | Y | D | K | A | S | L | A | A | D | I | A | G | L | M | - | H | A | L | G | H | - | - | - |
| 015 UniRef90\_L9WLS3\_16\_284 | V | I | A | P | D | L | R | G | L | G | D | S | E | - | - | - | - | T | P | A | S | G | - | - | - | - | - | Y | D | K | D | T | V | A | T | D | V | R | E | L | V | - | H | A | L | G | H | G | D | - |
| 016 UniRef90\_UPI00131EC7B5\_30\_311 | I | I | A | P | D | L | P | G | Q | G | D | S | D | - | - | - | - | R | P | E | N | G | - | - | - | - | - | Y | D | T | K | S | L | A | A | K | V | H | G | L | L | - | A | Q | L | S | V | - | - | - |
| 017 UniRef90\_C7QAM5\_50\_332 | V | I | A | P | D | L | P | G | A | G | R | S | D | - | - | - | - | A | P | A | T | G | - | - | - | - | - | Y | D | K | K | D | L | A | A | D | I | H | G | L | L | - | A | E | I | G | H | D | - | - |
| 018 UniRef90\_A0A1Q3SXP6\_11\_288 | V | I | A | P | D | L | R | G | F | G | Q | S | S | - | - | - | - | K | P | L | E | G | - | - | - | - | - | Y | D | K | K | I | M | A | Q | D | I | H | A | L | I | - | T | S | L | G | L | - | - | - |
| 019 UniRef90\_UPI00149248BF\_39\_315 | V | I | A | V | D | V | R | G | M | G | D | S | S | - | - | - | - | R | P | E | N | G | - | - | - | - | - | Y | D | T | Q | T | A | A | N | D | I | R | A | L | M | - | S | E | L | G | V | - | - | - |
| 020 UniRef90\_UPI000361D127\_26\_313 | V | V | V | A | D | P | R | G | T | G | L | S | D | - | - | - | - | K | P | V | T | G | - | - | - | - | - | Y | D | S | A | T | C | A | Q | D | F | H | R | L | M | - | E | R | L | G | H | - | - | - |
| 021 UniRef90\_A0A0M4FVH2\_24\_312 | L | I | V | L | D | L | P | G | Q | G | D | S | D | - | - | - | - | R | P | A | D | G | - | - | - | - | - | Y | D | T | K | T | L | A | T | T | V | H | K | F | L | - | Q | Q | L | G | T | - | - | - |
| 022 UniRef90\_A0A1Q4ZL08\_9\_290 | V | I | A | P | D | L | R | G | A | G | G | S | D | - | - | - | - | A | P | A | G | G | - | - | - | - | - | Y | D | K | K | T | L | A | R | D | V | H | D | L | L | - | A | Q | L | G | L | E | - | - |
| 023 UniRef90\_S3CY91\_25\_307 | V | I | A | P | D | Y | R | G | A | G | E | S | S | - | - | - | - | H | P | A | N | G | - | - | - | - | - | F | D | K | L | T | I | A | T | D | L | H | K | L | L | H | D | H | L | N | I | K | - | - |
| 024 UniRef90\_UPI0010F95BB2\_18\_294 | A | I | A | V | D | L | R | G | F | G | H | S | S | - | - | - | - | K | P | P | D | G | - | - | - | - | - | Y | D | K | K | T | V | A | R | D | L | H | E | L | L | - | D | S | L | A | I | - | - | - |
| 025 UniRef90\_A0A0N1GDE2\_28\_316 | V | V | A | V | D | S | R | G | A | G | L | S | D | - | - | - | - | K | P | D | D | G | - | - | - | - | - | Y | D | A | G | T | L | A | A | D | L | V | A | L | M | - | A | A | L | G | H | - | - | - |
| 026 UniRef90\_A0A2E5PJQ7\_5\_284 | V | I | A | P | D | L | R | G | L | G | D | S | S | - | - | - | - | R | P | A | D | G | - | - | - | - | - | Y | D | K | Q | T | V | A | N | D | I | W | R | V | A | H | D | E | L | G | H | - | - | - |
| 027 UniRef90\_A0A1M7IC97\_44\_328 | V | I | A | P | D | L | P | G | A | G | Q | S | D | - | - | - | - | A | P | K | S | G | - | - | - | - | - | Y | D | K | K | T | M | A | A | E | L | H | A | L | L | - | A | S | L | G | K | D | - | - |
| 028 UniRef90\_UPI0016149F5E\_5\_284 | A | I | A | I | D | P | P | G | S | G | Y | S | D | - | - | - | - | R | P | S | G | G | - | - | - | - | - | Y | D | T | G | A | V | A | A | T | L | H | R | A | M | - | L | A | L | G | H | - | - | - |
| 029 UniRef90\_UPI00156E1DFF\_8\_289 | V | I | A | P | D | L | R | G | I | G | A | S | D | - | - | - | - | R | P | L | S | G | - | - | - | - | - | Y | D | K | D | T | Q | A | E | D | M | R | E | L | L | - | A | Q | L | G | I | H | - | - |
| 030 UniRef90\_A0A3A8HRU0\_15\_300 | V | L | I | P | D | L | R | G | A | G | A | S | A | - | - | - | - | A | P | A | K | G | - | - | - | - | - | Y | D | K | E | T | M | A | R | D | M | R | A | L | V | - | K | K | L | G | F | - | - | - |
| 031 UniRef90\_A0A0M3UDU6\_18\_309 | V | I | A | V | D | P | R | G | M | G | E | S | D | - | - | - | - | A | P | I | D | D | - | - | - | - | - | Y | D | L | G | T | V | A | A | E | L | R | S | F | A | - | E | T | I | G | L | F | E | R |
| 032 UniRef90\_A0A447J1A9\_28\_317 | A | I | A | V | D | P | P | G | T | G | F | S | A | - | - | - | - | R | P | D | S | G | - | - | - | - | - | Y | D | T | G | A | V | A | T | T | L | H | T | M | M | - | N | Q | L | G | Y | - | - | - |
| 033 UniRef90\_A0A5C8T429\_14\_295 | V | V | A | V | D | L | P | G | M | G | D | S | D | - | - | - | - | K | P | I | D | G | - | - | - | - | - | Y | D | T | G | T | V | A | M | R | L | H | D | L | T | - | R | A | L | G | W | - | - | - |
| 034 UniRef90\_A0A0N0TCD7\_1\_288 | V | I | A | P | D | L | R | G | M | G | A | S | D | - | - | - | - | K | P | A | T | G | - | - | - | - | - | Y | D | A | A | T | L | A | D | D | M | A | T | L | M | - | T | A | L | G | H | - | - | - |
| 035 UniRef90\_W9ARP2\_16\_295 | V | I | M | P | D | Y | R | G | A | G | A | S | D | - | - | - | - | K | P | R | D | G | - | - | - | - | - | Y | D | K | W | T | M | A | G | D | I | H | T | L | V | R | E | K | L | G | V | A | - | - |
| 036 UniRef90\_A0A109IGY4\_11\_301 | V | V | A | V | D | Q | R | G | T | G | L | S | D | - | - | - | - | K | P | D | A | G | - | - | - | - | - | Y | D | A | G | T | L | A | A | D | L | V | A | L | M | - | A | A | L | G | H | - | - | - |
| 037 UniRef90\_K9DQK6\_24\_306 | V | I | V | P | D | L | R | G | L | G | L | S | S | - | - | - | - | K | A | A | G | G | - | - | - | - | - | Y | D | K | K | S | Q | A | A | D | I | R | A | V | V | - | E | R | L | G | Q | - | - | - |
| 038 UniRef90\_A0A1Q8IR73\_6\_294 | V | V | A | I | D | L | P | G | Q | G | D | S | D | - | - | - | - | R | P | I | D | G | - | - | - | - | - | Y | D | T | Q | T | V | A | Q | R | V | H | D | L | V | - | S | H | L | G | L | - | - | - |
| 039 UniRef90\_A0A1Y6D2S0\_29\_302 | V | V | A | P | D | L | R | G | L | G | L | S | E | - | - | - | - | K | T | R | T | G | - | - | - | - | - | Y | D | K | P | T | I | A | N | D | I | A | A | L | I | - | Q | H | L | G | R | - | - | - |
| 040 UniRef90\_E6WJ64\_19\_301 | I | I | A | P | D | L | P | G | Q | G | D | S | D | - | - | - | - | K | P | Q | A | G | - | - | - | - | - | Y | D | T | E | A | L | A | E | K | V | H | R | L | M | - | Q | Q | L | G | H | - | - | - |
| 041 UniRef90\_UPI000489A94A\_43\_316 | V | I | A | P | D | L | P | G | A | G | D | S | A | - | - | - | - | I | P | A | N | G | - | - | - | - | - | Y | D | K | K | A | L | A | Q | D | I | H | A | M | V | - | H | A | L | G | Y | - | - | - |
| 042 UniRef90\_A0A4R6HBQ4\_11\_285 | V | I | A | P | D | L | R | G | F | G | D | S | S | - | - | - | - | K | P | E | G | I | - | - | - | - | - | H | S | K | L | V | V | A | G | D | L | V | A | M | L | - | D | R | L | G | I | - | - | - |
| 043 UniRef90\_A0A1M7QJX9\_32\_311 | V | I | A | P | D | M | R | G | V | G | E | S | G | - | - | - | - | K | P | T | G | G | - | - | - | - | - | Y | D | K | K | N | M | A | V | D | L | H | D | L | M | - | K | Q | L | G | Y | - | - | - |
| 044 UniRef90\_A0A5B8WA72\_22\_300 | V | I | A | P | D | M | R | G | V | G | E | S | G | - | - | - | - | K | P | T | A | G | - | - | - | - | - | Y | D | K | K | N | M | A | V | D | M | H | E | L | M | - | K | K | L | G | Y | - | - | - |
| 045 UniRef90\_A0A0G2FGE6\_47\_303 | V | I | A | P | D | Y | R | G | A | G | H | S | S | - | - | - | - | K | P | R | D | G | - | - | - | - | - | Y | E | K | T | Q | M | A | A | D | I | H | K | L | L | T | E | H | L | G | I | T | - | - |
| 046 UniRef90\_A0A1I6BK09\_10\_296 | V | I | A | P | D | L | R | G | I | G | G | S | S | - | - | - | - | I | L | P | T | G | - | - | - | - | - | Y | D | K | R | T | L | A | A | D | I | Y | G | L | M | - | Q | Q | L | G | H | - | - | - |
| 047 UniRef90\_A0A4R7C9R7\_33\_310 | V | V | A | A | D | L | R | G | T | G | L | S | E | - | - | - | - | V | T | P | S | G | - | - | - | - | - | Y | D | K | R | T | I | A | E | D | I | R | A | L | I | - | G | H | L | G | V | - | - | - |
| 048 UniRef90\_A0A2M9M9Q1\_18\_305 | V | I | A | V | D | P | R | G | V | G | R | S | D | - | - | - | - | K | P | H | G | G | - | - | - | - | - | Y | D | S | G | T | V | A | A | E | L | R | L | L | M | - | R | Q | L | G | H | - | - | - |
| 049 UniRef90\_A0A4V3T343\_11\_295 | V | I | A | V | D | L | R | G | M | G | G | S | A | - | - | - | - | K | P | E | G | G | - | - | - | - | - | Y | D | K | K | T | L | A | A | D | V | H | A | L | L | - | A | H | L | G | I | - | - | - |
| 050 UniRef90\_UPI001616534D\_11\_303 | V | I | A | V | D | L | R | G | M | G | G | S | G | - | - | - | - | K | P | A | G | G | - | - | - | - | - | Y | D | K | K | T | M | A | A | D | V | H | A | L | V | - | R | H | L | G | L | - | - | - |
| 051 UniRef90\_J4PG95\_17\_290 | A | I | A | V | D | P | P | G | T | G | D | S | G | - | - | - | - | R | P | S | Q | G | - | - | - | - | - | Y | D | T | G | A | V | A | S | V | L | H | R | L | M | - | E | Q | L | G | H | - | - | - |
| 052 UniRef90\_A0A0T1T741\_1\_295 | V | V | A | V | D | S | R | G | A | G | L | S | D | - | - | - | - | K | P | D | D | G | - | - | - | - | - | Y | D | A | G | T | L | A | A | D | L | V | A | L | M | - | A | A | L | G | H | - | - | - |
| 053 UniRef90\_G0FSK7\_17\_310 | V | V | A | V | D | P | R | G | T | G | L | S | G | - | - | - | - | Q | P | G | D | G | - | - | - | - | - | Y | D | T | A | T | L | A | A | D | L | T | G | L | M | - | A | A | L | G | H | - | - | - |
| 054 UniRef90\_UPI00164AAB2D\_18\_303 | V | I | V | P | D | L | R | G | L | G | L | S | S | - | - | - | - | R | P | E | T | G | - | - | - | - | - | Y | E | K | K | N | Q | A | G | D | L | R | A | V | L | - | D | K | L | G | V | - | - | - |
| 055 UniRef90\_A0A1I2MT44\_20\_306 | V | V | A | V | D | A | R | G | V | G | L | S | D | - | - | - | - | K | P | A | T | G | - | - | - | - | - | Y | D | T | G | T | L | A | R | D | M | A | G | L | M | - | T | A | L | G | H | - | - | - |
| 056 UniRef90\_A0A2P2CCU5\_19\_306 | V | I | A | V | D | Q | R | G | M | G | L | T | D | - | - | - | - | K | P | D | D | G | - | - | - | - | - | Y | D | A | G | T | L | A | R | D | L | A | A | L | M | - | D | E | L | G | H | - | - | - |
| 057 UniRef90\_A0A2V9H5C8\_12\_298 | V | I | A | P | D | L | P | G | I | G | D | S | A | - | - | - | - | I | P | T | D | G | - | - | - | - | - | L | D | M | K | T | A | A | M | R | I | H | A | L | A | - | R | S | L | G | V | - | - | - |
| 058 UniRef90\_A0A0P4V0Y0\_7\_291 | V | I | V | P | D | M | R | G | Y | G | D | S | D | - | - | - | - | K | P | E | T | G | - | - | - | - | - | Y | D | A | R | S | L | S | E | D | F | R | Q | L | I | - | Q | Q | L | G | F | - | - | - |
| 059 UniRef90\_A0A0Q4UQ58\_27\_305 | V | I | A | P | D | L | R | G | L | G | L | S | E | - | - | - | - | R | T | V | S | G | - | - | - | - | - | Y | D | K | R | T | I | A | E | D | I | R | A | L | I | - | D | H | E | A | G | - | - | - |
| 060 UniRef90\_A0A6M4IM28\_20\_309 | V | I | V | P | D | L | R | G | M | G | H | S | A | - | - | - | - | R | P | A | S | G | - | - | - | - | - | Y | D | K | K | T | Q | G | T | D | I | A | G | M | L | - | D | A | L | H | I | - | - | - |
| 061 UniRef90\_A0A537J419\_20\_301 | V | I | A | P | D | L | P | G | I | G | D | S | E | - | - | - | - | I | P | A | S | G | - | - | - | - | - | L | D | M | K | A | A | A | I | R | I | H | A | L | A | - | R | S | L | G | V | - | - | - |
| 062 UniRef90\_A0A1V2PGN8\_6\_275 | V | I | A | P | D | L | R | G | Y | G | R | T | D | - | - | - | - | K | P | R | D | G | - | - | - | - | - | Y | D | K | R | T | M | A | A | D | V | S | E | L | V | - | R | T | L | G | F | - | - | - |
| 063 UniRef90\_A0A542JC81\_20\_314 | V | I | A | V | D | Q | R | G | I | G | L | S | E | - | - | - | - | K | P | A | S | G | - | - | - | - | - | Y | D | S | T | T | I | A | A | D | L | V | A | L | M | - | D | A | L | G | H | - | - | - |
| 064 UniRef90\_UPI000424F353\_33\_308 | V | I | V | P | D | L | R | G | M | G | L | S | S | - | - | - | - | H | P | S | G | G | - | - | - | - | - | Y | D | K | K | T | Q | G | G | D | I | A | G | V | L | - | D | A | L | K | I | - | - | - |
| 065 UniRef90\_A0A2V9W2E9\_22\_299 | V | V | V | P | D | L | R | G | I | G | K | S | S | - | - | - | - | K | P | E | G | G | - | - | - | - | - | Y | D | K | K | T | Q | A | K | D | M | R | A | V | V | - | T | G | L | G | Y | - | - | - |
| 066 UniRef90\_A0A2T6L0M5\_16\_299 | V | I | A | V | D | L | R | G | T | G | D | S | E | - | - | - | - | V | T | S | D | G | - | - | - | - | - | Y | D | T | V | Q | M | A | D | D | V | H | A | L | L | - | T | Q | L | G | L | N | - | - |
| 067 UniRef90\_A0A261TYY2\_43\_315 | V | V | A | I | D | P | P | G | L | G | D | S | D | - | - | - | - | R | P | P | G | G | - | - | - | - | - | Y | D | T | D | A | A | A | D | R | L | H | A | F | T | - | Q | S | L | G | W | - | - | - |
| 068 UniRef90\_UPI0009781B11\_10\_287 | V | L | A | I | D | P | P | G | L | G | D | S | G | - | - | - | - | P | S | V | S | G | - | - | - | - | - | Y | D | T | R | A | I | A | L | H | L | D | A | L | F | - | A | Y | T | G | D | - | - | - |
| 069 UniRef90\_A0A2V9TPC1\_35\_307 | V | I | A | P | D | L | P | G | I | G | D | S | D | - | - | - | - | I | P | A | H | G | - | - | - | - | - | L | D | M | K | S | A | A | I | R | I | H | D | L | A | - | K | S | L | G | V | - | - | - |
| 070 UniRef90\_UPI00145FA88A\_6\_279 | V | I | V | P | D | L | R | G | Y | G | R | S | D | - | - | - | - | K | P | L | G | G | - | - | - | - | - | Y | D | K | R | T | M | A | T | D | V | A | E | L | V | - | G | S | L | G | F | - | - | - |
| 071 UniRef90\_UPI0003FADEBE\_21\_306 | V | V | V | P | D | L | R | G | M | G | L | S | S | - | - | - | - | R | P | A | G | G | - | - | - | - | - | Y | D | K | K | T | Q | A | A | D | I | R | A | V | L | - | D | K | L | G | I | - | - | - |
| 072 UniRef90\_A0A2V8Y3P4\_78\_329 | V | V | V | P | D | L | R | G | I | G | R | S | S | - | - | - | - | K | P | D | G | G | - | - | - | - | - | Y | D | K | K | T | Q | A | K | D | I | R | A | V | V | - | T | A | L | G | Y | - | - | - |
| 073 UniRef90\_A0A285BKA7\_8\_299 | V | I | A | P | D | L | R | G | M | G | A | S | D | - | - | - | - | K | P | A | T | G | - | - | - | - | - | Y | D | A | A | T | P | A | D | D | M | A | E | L | M | - | T | A | L | G | H | - | - | - |
| 074 UniRef90\_A0A0K3AVW3\_44\_319 | V | I | A | V | D | M | R | G | Q | G | G | T | D | - | - | - | - | K | P | A | G | G | - | - | - | - | - | Y | D | K | K | T | M | A | R | D | I | H | E | L | V | - | R | H | L | G | H | - | - | - |
| 075 UniRef90\_A0A4D7B7N1\_7\_276 | V | I | A | I | D | L | P | G | Q | G | D | S | D | - | - | - | - | K | P | V | D | G | - | - | - | - | - | Y | D | T | R | T | T | G | E | R | I | H | A | L | L | - | Q | T | L | G | H | - | - | - |
| 076 UniRef90\_A0A2U0WF37\_17\_293 | V | V | V | P | D | L | R | G | L | G | L | S | S | - | - | - | - | I | P | Q | G | G | - | - | - | - | - | Y | D | K | K | T | Q | A | G | D | I | R | G | V | L | - | A | A | L | G | I | - | - | - |
| 077 UniRef90\_UPI00135C2D49\_17\_309 | V | I | S | V | D | L | R | G | M | G | S | S | A | - | - | - | - | K | P | A | G | G | - | - | - | - | - | Y | D | K | R | T | M | A | D | D | I | R | A | L | V | - | R | H | L | G | L | - | - | - |
| 078 UniRef90\_UPI001473614E\_45\_322 | V | I | A | V | D | L | P | G | L | G | D | S | S | - | - | - | - | V | P | A | S | G | - | - | - | - | - | Y | D | K | V | T | T | A | Q | R | V | R | Q | A | V | - | R | R | L | G | F | - | - | - |
| 079 UniRef90\_J3F862\_22\_299 | V | V | V | P | D | L | R | G | M | G | L | S | S | - | - | - | - | I | P | A | D | G | - | - | - | - | - | Y | D | K | K | T | Q | A | G | D | I | R | A | V | L | - | A | S | L | G | I | - | - | - |
| 080 UniRef90\_A0A1A9NC01\_62\_345 | V | I | A | V | D | L | R | G | M | G | A | S | S | - | - | - | - | K | P | D | N | G | - | - | - | - | - | Y | D | K | K | T | M | A | A | D | I | H | E | L | V | - | K | S | L | G | Y | - | - | - |
| 081 UniRef90\_UPI00131AC11B\_45\_327 | V | Y | A | I | D | I | R | G | M | G | S | S | D | - | - | - | - | K | P | A | G | G | - | - | - | - | - | Y | D | K | K | T | M | A | N | D | L | S | E | L | I | - | H | Q | L | G | F | - | - | - |
| 082 UniRef90\_A0A4R8HC20\_22\_306 | V | I | A | V | D | L | R | G | M | G | G | S | A | - | - | - | - | K | P | A | T | G | - | - | - | - | - | Y | D | K | K | T | L | A | R | D | I | H | A | L | L | - | G | R | L | G | I | - | - | - |
| 083 UniRef90\_A0A1B2HH26\_4\_280 | V | L | A | P | D | L | R | G | S | G | D | S | E | - | - | - | - | V | P | T | G | H | - | - | - | - | - | W | T | K | Q | D | E | A | D | D | L | H | E | L | L | - | H | H | L | G | H | - | - | - |
| 084 UniRef90\_A0A517LKB1\_23\_304 | V | I | A | P | D | Y | R | G | H | G | H | S | N | - | - | - | - | P | P | T | N | S | - | T | V | D | M | Y | T | K | R | Q | L | A | A | D | L | H | D | L | V | T | K | H | L | D | I | K | - | - |
| 085 UniRef90\_A0A0N1F3N8\_17\_298 | V | V | V | P | D | L | R | G | M | G | L | S | S | - | - | - | - | R | A | P | G | G | - | - | - | - | - | Y | D | K | K | T | Q | G | A | D | V | A | G | V | L | - | D | A | L | K | I | - | - | - |
| 086 UniRef90\_UPI0004CCFD44\_55\_340 | V | Y | A | V | D | L | P | G | L | G | D | S | T | - | - | - | - | G | S | P | T | G | - | - | - | - | - | Y | D | K | A | T | L | A | R | Y | V | H | T | L | I | A | D | R | L | G | V | - | - | - |
| 087 UniRef90\_A0A4D4LDP7\_7\_304 | V | I | A | L | D | L | P | G | Q | G | L | S | D | - | - | - | - | R | P | A | D | G | - | - | - | - | - | Y | D | T | E | T | A | A | A | R | V | H | E | A | V | - | V | R | L | A | G | - | - | - |
| 088 UniRef90\_A0A2V4B948\_8\_279 | V | I | A | P | D | L | R | G | Y | G | L | T | D | - | - | - | - | K | P | A | H | G | - | - | - | - | - | Y | D | K | R | T | M | A | A | D | V | A | A | L | V | - | T | A | L | G | F | - | - | - |
| 089 UniRef90\_A0A2P2FV90\_3\_282 | V | I | A | P | D | L | R | G | Y | G | K | S | D | - | - | - | - | K | P | R | D | G | - | - | - | - | - | Y | D | K | R | T | M | A | A | D | V | A | A | L | A | - | T | H | L | G | F | - | - | - |
| 090 UniRef90\_A0A4Q2J292\_17\_304 | V | I | V | P | D | L | R | G | M | G | L | S | D | - | - | - | - | H | P | A | T | G | - | - | - | - | - | Y | D | K | K | N | E | A | R | D | I | A | G | V | L | - | D | A | L | K | I | - | - | - |
| 091 UniRef90\_UPI0012B05B7D\_16\_287 | V | I | A | L | D | L | P | G | Q | G | D | S | D | - | - | - | - | K | P | P | G | G | - | - | - | - | - | Y | D | T | Q | T | T | A | E | R | I | H | G | F | V | - | E | K | L | G | L | - | - | - |
| 092 UniRef90\_UPI00048D4AD4\_4\_301 | V | I | A | V | D | L | R | G | M | G | D | S | S | - | - | - | - | K | P | E | A | G | - | - | - | - | - | Y | D | K | R | T | M | A | A | D | I | H | R | L | V | - | D | H | L | G | L | - | - | - |
| 093 UniRef90\_UPI0015F81933\_16\_310 | V | I | A | I | D | L | P | G | Q | G | H | S | E | - | - | - | - | R | P | E | R | G | - | - | - | - | - | Y | D | T | H | T | V | A | A | H | V | H | A | A | V | - | K | A | L | G | V | - | - | - |
| 094 UniRef90\_UPI00160CFF45\_55\_340 | V | Y | A | V | D | L | P | G | L | G | D | S | T | - | - | - | - | G | S | P | T | G | - | - | - | - | - | Y | D | K | A | T | L | A | R | Y | V | H | T | L | M | A | D | R | L | G | V | - | - | - |
| 095 UniRef90\_A0A1Q8KTW7\_17\_294 | V | V | A | P | D | V | R | G | F | G | D | S | A | - | - | - | - | R | P | D | D | G | - | - | - | - | - | Y | D | M | G | M | I | A | R | D | I | G | A | L | M | - | T | E | L | G | H | - | - | - |
| 096 UniRef90\_UPI00161A7EC4\_21\_297 | V | I | A | F | D | L | P | G | L | G | D | S | T | - | - | - | - | A | Q | S | G | G | - | - | - | - | - | Y | D | K | A | T | T | A | K | R | I | R | Q | A | V | - | N | K | L | G | F | - | - | - |
| 097 UniRef90\_A0A1B4WXA8\_16\_299 | V | V | V | P | D | L | R | G | M | G | L | S | S | - | - | - | - | I | P | D | G | G | - | - | - | - | - | Y | D | K | K | T | Q | A | G | D | I | R | G | V | L | - | A | A | L | K | I | - | - | - |
| 098 UniRef90\_UPI000E46D2C1\_48\_330 | V | I | A | V | D | I | R | G | M | G | E | T | D | - | - | - | - | K | P | E | G | G | - | - | - | - | - | Y | D | K | K | T | M | A | A | D | I | H | A | L | I | - | G | E | L | G | Y | - | - | - |
| 099 UniRef90\_A0A2N5CFI4\_23\_293 | V | V | V | P | D | L | R | G | M | G | L | S | N | - | - | - | - | H | P | A | T | G | - | - | - | - | - | Y | D | K | K | N | Q | A | R | D | I | A | G | V | L | - | D | A | L | Q | I | - | - | - |
| 100 UniRef90\_A0A385B2U0\_45\_333 | V | I | A | V | D | L | R | G | T | G | D | S | S | - | - | - | - | V | P | Q | Q | G | - | - | - | - | - | Y | D | T | A | Q | L | A | D | D | V | H | E | L | L | - | T | R | L | N | L | N | - | - |
| 101 UniRef90\_A0A1Q7W147\_16\_308 | V | I | A | I | D | L | P | G | Q | G | H | S | E | - | - | - | - | R | P | V | R | S | - | - | - | - | - | Y | D | T | H | T | V | A | A | H | V | H | A | A | V | - | N | A | L | G | A | - | - | - |
| 102 UniRef90\_A0A239MTD5\_1\_271 | V | I | A | V | D | L | R | G | T | G | N | S | Q | - | - | - | - | V | T | E | S | G | - | - | - | - | - | Y | Q | A | A | Q | L | A | E | D | V | H | E | L | L | - | K | Q | L | G | L | N | - | - |
| 103 UniRef90\_UPI000561A6EF\_30\_315 | V | V | A | V | D | P | R | G | V | G | L | S | D | - | - | - | - | K | P | A | T | G | - | - | - | - | - | Y | D | T | G | T | L | A | A | D | L | A | A | L | M | - | D | A | L | G | H | - | - | - |
| 104 UniRef90\_A0A4Y8RHA3\_28\_294 | V | I | V | P | D | L | R | G | L | G | L | S | Q | - | - | - | - | R | P | E | G | G | - | - | - | - | - | Y | D | K | K | T | Q | G | Q | D | I | A | G | L | L | - | D | A | L | D | V | - | - | - |
| 105 UniRef90\_UPI0004DF415F\_15\_308 | V | I | A | I | D | L | P | G | Q | G | H | S | D | - | - | - | - | R | P | Q | D | S | - | - | - | - | - | Y | D | T | H | T | V | A | S | R | V | Q | A | A | V | - | T | A | L | D | V | - | - | - |
| 106 UniRef90\_A0A2N3KZL1\_30\_325 | V | V | A | V | D | L | P | G | L | G | Q | S | A | - | - | - | - | V | P | K | S | - | - | - | - | - | - | Y | V | G | Q | D | I | S | P | I | L | Y | G | L | A | - | K | Q | F | S | P | D | - | - |
| 107 UniRef90\_UPI00076E3DF2\_55\_340 | V | Y | A | V | D | L | P | G | L | G | D | S | T | - | - | - | - | G | T | P | T | G | - | - | - | - | - | Y | D | K | A | T | L | A | R | Y | V | H | T | L | V | A | D | Q | L | G | V | - | - | - |
| 108 UniRef90\_UPI00130E1FA7\_66\_348 | V | I | A | V | D | I | R | G | M | G | S | S | D | - | - | - | - | K | P | K | S | G | - | - | - | - | - | Y | D | K | K | N | M | A | R | D | I | Y | E | L | I | - | K | K | L | G | Y | - | - | - |
| 109 UniRef90\_A0A1I3FUH2\_46\_328 | V | V | A | V | D | I | R | G | M | G | A | S | E | - | - | - | - | K | P | E | G | G | - | - | - | - | - | Y | D | K | K | T | M | A | R | D | I | Q | E | L | I | - | H | A | L | G | Y | - | - | - |
| 110 UniRef90\_UPI000E2836BA\_28\_313 | V | I | A | V | D | I | R | G | M | G | G | S | E | - | - | - | - | K | P | R | S | G | - | - | - | - | - | F | T | K | K | N | M | A | R | D | V | H | E | L | A | - | R | H | L | G | H | - | - | - |
| 111 UniRef90\_A0A653WNW3\_25\_297 | V | I | V | P | D | L | R | G | M | G | L | S | A | - | - | - | - | Q | P | S | G | G | - | - | - | - | - | F | D | K | K | N | Q | A | H | D | I | A | G | V | L | - | D | K | L | G | I | - | - | - |
| 112 UniRef90\_A0A5C1I8U5\_39\_321 | V | I | A | I | D | Y | R | G | M | G | T | S | D | - | - | - | - | K | P | A | E | G | - | - | - | - | - | Y | D | K | K | T | I | A | G | D | I | Y | G | L | L | - | H | Q | L | G | Y | - | - | - |
| 113 UniRef90\_UPI00165059AC\_4\_267 | V | V | A | P | D | L | R | G | Y | G | R | T | D | - | - | - | - | K | P | A | G | G | - | - | - | - | - | Y | D | K | R | T | M | A | D | D | V | H | R | L | V | - | G | S | L | G | F | - | - | - |
| 114 UniRef90\_F8JLL5\_12\_309 | V | I | A | I | D | L | P | G | Q | G | H | S | E | - | - | - | - | R | P | D | I | S | - | - | - | - | - | Y | D | T | H | T | V | A | A | H | V | H | A | A | V | - | E | A | L | G | V | - | - | - |
| 115 UniRef90\_A0A329J6I4\_30\_314 | V | V | A | V | D | L | P | G | L | G | E | S | Q | - | - | - | - | P | P | K | T | G | - | - | - | - | - | Y | S | G | T | A | V | S | K | Y | L | F | D | L | A | - | T | Q | L | S | G | N | - | - |
| 116 UniRef90\_A0A3N1H0W7\_60\_340 | V | I | A | A | D | L | P | G | M | G | T | S | A | - | - | - | - | K | P | A | G | G | - | - | - | - | - | Y | D | K | K | S | M | A | T | A | V | R | G | L | V | - | R | A | L | G | Y | - | - | - |
| 117 UniRef90\_A0A316VRQ3\_1\_263 | V | V | A | P | D | Y | R | G | A | G | G | S | S | - | - | - | - | M | P | L | G | G | - | - | - | - | - | Y | D | K | M | T | M | A | T | D | L | H | K | L | M | V | D | K | L | N | V | - | - | - |
| 118 UniRef90\_A0A4P8X818\_19\_291 | V | I | V | P | D | L | R | G | F | G | D | S | T | - | - | - | - | R | P | K | D | G | - | - | - | - | - | Y | D | M | R | T | M | A | E | D | V | V | Q | L | A | - | S | R | L | G | F | - | - | - |
| 119 UniRef90\_UPI0008405A69\_11\_309 | V | T | A | V | D | L | R | G | M | G | G | S | A | - | - | - | - | K | P | E | S | G | - | - | - | - | - | Y | D | K | K | T | M | A | R | D | I | H | E | L | I | - | R | H | L | G | H | - | - | - |
| 120 UniRef90\_A0A6I8M538\_3\_275 | V | I | T | P | D | L | R | G | Y | G | R | T | D | - | - | - | - | K | P | R | T | G | - | - | - | - | - | Y | D | K | R | T | M | A | A | D | I | A | A | L | A | - | E | H | L | G | Y | - | - | - |
| 121 UniRef90\_UPI000B5CCBFE\_21\_300 | V | I | A | V | D | H | R | G | I | G | I | P | D | E | - | - | L | K | P | T | D | G | - | - | - | - | - | F | T | K | K | N | M | A | R | D | V | Y | E | L | I | - | C | S | L | G | Y | - | - | - |
| 122 UniRef90\_A0A0M9ZDW3\_1\_266 | V | V | A | V | D | I | R | G | M | G | D | S | D | - | - | - | - | K | P | G | C | G | - | - | - | - | - | Y | D | K | K | N | M | A | R | D | I | Y | E | L | V | - | R | S | L | G | Y | - | - | - |
| 123 UniRef90\_UPI000E275A28\_18\_308 | L | I | V | P | D | P | R | G | L | G | L | S | D | - | - | - | - | K | P | A | T | G | - | - | - | - | - | Y | D | T | G | T | L | G | G | D | L | F | A | L | M | - | T | A | L | D | H | - | - | - |
| 124 UniRef90\_A0A3L8K0H7\_13\_298 | V | Y | A | V | D | I | R | G | M | G | G | S | A | - | - | - | - | K | P | A | A | G | - | - | - | - | - | F | E | K | K | N | M | A | R | D | I | R | E | L | V | - | R | L | L | G | H | - | - | - |
| 125 UniRef90\_A0A6N7ZB66\_18\_297 | V | V | V | P | D | L | R | G | T | G | S | S | E | - | - | - | - | R | P | A | D | G | - | - | - | - | - | Y | L | K | T | N | Q | V | K | D | M | R | G | L | V | - | E | G | L | G | L | S | - | - |
| 126 UniRef90\_A0A2W7GM08\_52\_337 | V | V | S | V | D | L | R | G | M | G | A | S | G | - | - | - | - | K | P | A | D | G | - | - | - | - | - | Y | D | K | K | T | M | A | A | D | I | A | A | L | V | - | R | H | L | E | L | - | - | - |
| 127 UniRef90\_A0A258JI33\_23\_300 | V | I | V | P | D | L | R | G | M | G | L | S | S | - | - | - | - | Q | P | K | S | G | - | - | - | - | - | Y | D | K | K | T | Q | G | Q | D | I | A | K | V | M | - | D | A | L | K | V | - | - | - |
| 128 UniRef90\_A0A6A6C1N5\_26\_297 | C | I | A | P | D | L | R | G | T | G | S | S | T | - | - | - | - | A | P | T | N | D | - | - | - | - | - | F | L | K | T | T | L | A | Q | D | L | L | A | L | L | - | D | H | L | H | L | P | - | - |
| 129 UniRef90\_UPI00160A92C2\_5\_284 | V | I | M | P | D | L | R | G | Y | G | E | T | D | - | - | - | - | K | P | D | Q | G | - | - | - | - | - | Y | D | K | R | N | M | A | E | D | V | R | Q | L | V | - | A | H | L | G | Y | - | - | - |
| 130 UniRef90\_UPI0009E804D9\_8\_293 | V | V | V | P | D | L | R | G | S | G | A | S | A | - | - | - | - | R | P | E | D | G | - | - | - | - | - | Y | H | K | T | N | Q | A | E | D | M | R | G | V | L | - | Q | H | L | G | L | S | - | - |
| 131 UniRef90\_UPI00049086FA\_57\_348 | V | I | A | V | D | L | P | G | M | G | D | S | T | - | - | - | - | G | E | P | P | A | - | - | - | - | - | Y | T | K | T | T | M | A | G | Y | V | H | A | L | L | - | G | A | I | G | R | P | - | - |
| 132 UniRef90\_A5FF96\_16\_286 | L | I | V | V | D | L | R | G | M | G | S | S | E | - | - | - | - | K | P | L | D | G | - | - | - | - | - | Y | T | K | K | N | M | A | Q | D | I | Q | L | L | I | - | A | H | L | G | Y | - | - | - |
| 133 UniRef90\_UPI0016621A44\_4\_265 | A | V | A | A | D | L | R | G | L | G | N | S | T | - | - | - | - | A | G | G | G | R | - | - | - | - | - | F | D | K | E | L | L | A | G | D | V | I | A | L | A | - | D | R | L | D | V | - | - | - |
| 134 UniRef90\_UPI000B83B944\_2\_264 | V | Y | V | V | E | Y | R | G | M | G | S | S | D | - | - | - | - | K | P | P | T | G | - | - | - | - | - | Y | D | K | K | T | L | S | S | D | I | H | A | L | V | - | K | E | L | G | Y | - | - | - |
| 135 UniRef90\_A0A0B4DHQ5\_15\_286 | V | I | A | P | D | L | R | G | Y | G | E | T | E | - | - | - | - | K | P | A | T | G | - | - | - | - | - | Y | D | K | R | N | M | A | K | D | L | A | A | L | L | - | D | K | L | G | I | - | - | - |
| 136 UniRef90\_UPI0009B31CF7\_18\_283 | T | I | A | P | D | L | R | G | I | G | G | S | D | - | - | - | - | R | P | E | S | G | - | - | - | - | - | Y | D | V | Q | N | L | A | D | D | A | V | A | L | L | - | D | A | L | D | A | - | - | - |
| 137 UniRef90\_UPI001661B99F\_34\_324 | V | I | A | V | D | L | R | G | L | G | R | S | Q | - | - | - | - | P | S | D | S | D | - | A | Y | G | T | Y | T | A | L | T | L | A | A | D | V | H | A | V | V | - | D | R | L | G | F | G | - | D |
| 138 UniRef90\_UPI00104123D8\_9\_274 | V | I | A | I | D | L | P | G | I | G | E | S | V | - | - | - | - | G | A | V | T | G | - | - | - | - | - | G | T | K | L | R | L | A | E | A | I | H | G | L | V | - | Q | R | L | A | L | - | - | - |
| 139 UniRef90\_A0A1M7Z6I5\_10\_283 | V | I | A | P | D | L | R | G | Y | G | E | T | D | - | - | - | - | K | P | A | T | G | - | - | - | - | - | Y | D | K | R | N | M | A | L | D | L | V | E | L | L | - | K | T | L | D | I | - | - | - |
| 140 UniRef90\_A0A431M1N9\_22\_292 | V | I | V | P | D | L | R | G | M | G | L | S | S | - | - | - | - | H | P | S | A | G | - | - | - | - | - | Y | E | K | V | N | Q | A | R | D | L | V | G | I | L | - | D | Q | L | K | I | - | - | - |
| 141 UniRef90\_UPI0013D28676\_49\_329 | V | V | A | I | D | L | R | G | L | G | G | S | S | P | - | - | - | A | P | T | D | A | - | - | - | G | Q | Y | T | A | L | A | L | A | G | D | V | R | A | V | V | - | T | R | L | G | L | D | R | T |
| 142 UniRef90\_A0A402BD85\_4\_199 | V | I | V | P | D | M | R | G | A | G | Q | I | D | - | - | - | - | A | L | P | T | G | - | - | - | - | - | Y | D | K | V | T | M | A | R | D | I | H | G | L | V | - | S | Q | L | S | P | - | - | - |
| 143 UniRef90\_A0A2V5S4I0\_55\_307 | V | I | A | V | D | L | P | G | I | G | D | S | S | - | - | - | - | I | P | A | D | N | - | - | - | - | K | V | D | M | I | I | A | A | K | Q | I | H | D | L | V | - | R | S | L | K | I | - | - | - |
| 144 UniRef90\_A0A328AN40\_21\_303 | V | V | A | P | D | Q | R | G | Y | N | L | S | D | - | - | - | - | K | P | K | G | V | - | - | - | D | A | Y | R | L | D | L | L | A | G | D | I | F | A | L | A | - | A | A | L | G | R | - | - | - |
| 145 UniRef90\_A0A0M9AP20\_25\_244 | V | V | A | P | D | L | R | G | Y | N | H | S | D | - | - | - | - | K | P | D | G | V | - | - | - | A | A | Y | H | I | D | E | L | V | A | D | V | A | G | L | V | - | S | E | L | G | R | - | - | - |
| 146 UniRef90\_A0A1N6RLL9\_1\_202 | - | - | - | - | - | - | - | - | - | - | - | - | - | - | - | - | - | - | - | - | - | - | - | - | - | - | - | - | - | - | - | - | M | A | E | D | I | H | Q | L | S | - | L | Q | L | Q | L | - | - | - |
| 147 UniRef90\_A0A4R3LTR2\_48\_312 | V | V | A | A | D | L | R | G | Y | G | D | S | S | - | - | - | - | K | P | A | G | G | P | D | H | I | A | Y | S | K | R | E | M | A | K | D | Q | V | A | L | M | - | A | R | L | G | F | - | - | - |
| 148 UniRef90\_UPI0002ED95C2\_14\_285 | V | V | C | P | D | L | R | G | Y | G | A | S | D | - | - | - | - | K | P | A | G | G | P | D | G | D | A | Y | S | K | R | V | M | A | A | D | I | V | G | L | V | - | R | S | L | G | H | - | - | - |
| 149 UniRef90\_A0A1Q7MBV0\_16\_212 | A | V | A | P | D | L | R | G | Y | G | G | S | D | - | - | - | - | K | P | S | G | V | - | - | - | A | A | Y | R | I | E | L | L | V | R | D | A | S | E | L | I | - | A | A | L | G | R | - | - | - |
| 150 UniRef90\_A0A1A0KI05\_7\_212 | V | V | V | P | D | L | R | G | F | G | E | S | D | K | H | L | A | D | P | Q | Q | F | - | - | - | - | - | Y | S | A | A | A | Q | A | R | S | V | V | G | L | I | - | D | E | L | G | L | - | - | - |

  
  

|  |  |  |  |  |  |  |  |  |  |  |  |  |  |  |  |  |  |  |  |  |  |  |  |  |  |  |  |  |  |  |  |  |  |  |  |  |  |  |  |  |  |  |  |  |  |  |  |  |  |  |
| --- | --- | --- | --- | --- | --- | --- | --- | --- | --- | --- | --- | --- | --- | --- | --- | --- | --- | --- | --- | --- | --- | --- | --- | --- | --- | --- | --- | --- | --- | --- | --- | --- | --- | --- | --- | --- | --- | --- | --- | --- | --- | --- | --- | --- | --- | --- | --- | --- | --- | --- |
| **001 Input\_protein\_seq** | T | R | I | H | L | V | G | R | D | I | G | V | M | V | A | Y | A | Y | A | A | Q | W | P | T | E | I | V | K | L | A | M | L | D | V | P | V | - | P | G | T | R | I | W | - | D | E | A | - | - | - |
| 002 UniRef90\_UPI00158A2D6D\_3\_306 | T | R | I | K | L | V | G | R | D | I | G | V | M | V | A | Y | A | Y | A | A | Q | W | P | A | E | V | L | K | L | A | M | L | D | V | P | V | - | P | G | T | R | I | W | - | D | E | A | - | - | - |
| 003 UniRef90\_A0A1Z4J856\_27\_318 | K | R | I | F | L | V | G | H | D | I | G | A | P | V | A | Y | A | Y | A | A | A | H | P | E | D | V | R | R | L | A | V | L | E | L | V | L | - | A | G | A | G | L | E | - | E | L | I | - | - | - |
| 004 UniRef90\_G7LVZ3\_7\_288 | C | E | I | N | L | V | G | H | D | I | G | M | M | V | A | Y | E | Y | A | S | A | H | P | K | E | I | R | R | L | A | V | L | E | A | G | L | - | P | G | L | G | L | E | - | A | L | M | - | - | - |
| 005 UniRef90\_A0A2I8F4B9\_53\_324 | P | Q | V | N | V | V | G | H | D | M | G | G | M | V | A | Y | A | Y | A | A | Q | H | P | D | E | V | R | T | L | A | I | L | D | V | P | L | - | P | G | I | E | P | W | - | D | Q | L | - | - | - |
| 006 UniRef90\_A0A327RPK7\_10\_284 | K | E | I | S | L | V | G | H | D | I | G | A | M | V | A | F | T | Y | A | A | E | Y | R | E | Y | V | K | K | L | V | L | L | D | V | L | L | - | P | G | F | G | L | E | - | K | L | M | - | - | - |
| 007 UniRef90\_A0A6L3SWG4\_20\_294 | G | P | V | T | V | A | G | H | D | I | G | M | M | V | A | Y | A | F | A | R | R | F | P | D | R | T | D | G | L | V | V | M | E | A | P | L | - | P | G | T | A | A | Y | - | E | R | S | - | - | - |
| 008 UniRef90\_A0A401ZLI5\_4\_287 | Q | Q | V | F | L | V | G | H | D | L | G | V | L | T | A | Y | A | Y | A | A | R | Y | R | E | A | V | Q | R | L | V | I | L | D | S | P | V | - | E | G | F | G | S | E | - | D | F | V | - | - | - |
| 009 UniRef90\_A0A4V2U6R1\_12\_297 | K | R | I | T | V | V | S | H | D | L | G | M | Q | V | G | Y | A | F | A | A | L | F | P | E | D | I | D | R | L | V | V | M | E | A | P | I | - | A | G | T | T | G | M | - | E | S | V | - | - | - |
| 010 UniRef90\_A0A5A5T922\_6\_282 | E | H | I | L | L | V | G | H | D | W | G | G | H | I | A | Y | A | Y | A | A | G | H | R | D | G | V | Q | R | L | A | I | V | E | S | L | V | - | R | S | G | D | T | V | - | R | D | A | - | - | - |
| 011 UniRef90\_A0A2V6UIN9\_3\_273 | A | R | A | G | I | V | G | H | D | I | G | L | M | V | A | Y | A | Y | A | A | Q | Y | P | S | E | V | D | R | I | V | L | M | D | A | F | L | - | P | G | V | G | D | W | - | T | Q | V | - | - | - |
| 012 UniRef90\_UPI001669B3C5\_41\_314 | Q | H | A | T | I | V | G | H | D | I | G | L | M | V | A | Y | A | Y | A | A | Q | Y | P | Q | E | T | D | R | V | V | L | M | D | A | F | L | - | P | G | I | G | N | W | - | K | D | M | - | - | - |
| 013 UniRef90\_A0A4R2Z7A8\_3\_305 | T | R | Y | L | L | V | A | H | D | V | G | A | W | V | A | Y | P | Y | A | A | L | Y | P | N | E | V | K | R | L | A | L | L | D | A | G | I | - | P | G | I | T | L | P | - | D | A | L | - | - | - |
| 014 UniRef90\_A0A5C5SVV1\_11\_281 | P | R | Y | A | V | C | G | H | D | I | G | G | M | V | G | M | A | L | A | M | T | R | R | Q | A | V | T | H | L | A | I | L | D | V | P | L | - | P | G | W | S | R | W | - | E | T | L | - | - | - |
| 015 UniRef90\_L9WLS3\_16\_284 | E | R | I | A | L | V | G | H | D | W | G | M | P | T | A | Y | A | Y | A | A | Q | Y | R | E | E | V | A | A | L | C | V | L | E | A | G | L | - | P | G | V | R | E | D | - | - | - | - | - | - | - |
| 016 UniRef90\_UPI00131EC7B5\_30\_311 | S | R | Y | F | L | A | A | H | D | V | G | A | W | V | A | W | P | Y | A | S | L | Y | G | S | E | V | R | K | L | V | L | L | D | A | G | I | - | P | G | V | T | L | P | - | D | A | L | - | - | - |
| 017 UniRef90\_C7QAM5\_50\_332 | H | D | I | R | L | V | G | H | D | I | G | T | M | V | A | Y | S | Y | A | A | A | Y | P | R | D | V | T | K | L | V | L | S | E | A | P | I | - | P | D | P | S | I | Y | - | T | Y | P | - | - | - |
| 018 UniRef90\_A0A1Q3SXP6\_11\_288 | S | H | I | Q | I | V | G | H | D | I | G | L | M | V | A | Y | A | Y | A | A | Q | F | P | E | T | V | D | R | I | V | L | M | D | A | F | L | - | P | G | I | G | N | W | - | K | A | V | - | - | - |
| 019 UniRef90\_UPI00149248BF\_39\_315 | S | R | Y | T | V | I | G | H | D | V | G | M | W | I | A | Y | P | L | A | A | E | H | G | E | S | I | D | K | L | V | M | I | E | A | T | I | - | P | G | V | T | P | W | - | P | P | M | - | - | - |
| 020 UniRef90\_UPI000361D127\_26\_313 | E | R | F | A | V | V | G | H | D | I | G | M | W | T | A | Y | A | M | A | V | D | T | P | G | R | V | Q | R | M | A | L | V | D | A | I | I | - | P | G | V | S | P | S | - | P | P | L | - | - | - |
| 021 UniRef90\_A0A0M4FVH2\_24\_312 | K | R | Y | F | L | A | A | H | D | V | G | A | W | V | A | Y | T | Y | A | A | L | F | G | D | E | V | R | R | L | A | L | L | D | A | G | I | - | P | G | I | T | M | P | - | D | A | L | - | - | - |
| 022 UniRef90\_A0A1Q4ZL08\_9\_290 | R | D | I | C | L | V | G | H | D | I | G | T | M | V | A | Y | A | Y | A | A | A | Y | G | N | Q | V | S | K | L | V | L | T | E | A | P | I | - | P | D | Q | G | L | Y | - | Q | A | P | - | - | - |
| 023 UniRef90\_S3CY91\_25\_307 | K | S | I | H | V | V | G | H | D | I | G | G | M | I | A | H | A | Y | A | A | K | F | P | D | H | T | L | S | V | T | W | G | E | C | P | L | - | P | G | S | T | P | Y | - | E | S | G | - | F | K |
| 024 UniRef90\_UPI0010F95BB2\_18\_294 | Q | S | C | W | V | V | G | H | D | M | G | G | Q | V | A | Y | P | F | V | A | Q | W | P | E | R | A | K | G | L | V | F | I | E | S | G | L | - | P | G | F | G | Q | E | - | N | A | M | - | - | - |
| 025 UniRef90\_A0A0N1GDE2\_28\_316 | D | R | F | D | V | V | G | H | D | I | G | M | W | T | G | Y | A | L | A | A | D | H | P | E | R | V | S | R | L | A | V | V | D | A | I | I | - | P | G | L | T | P | T | - | P | S | V | - | - | - |
| 026 UniRef90\_A0A2E5PJQ7\_5\_284 | E | R | F | L | L | V | G | H | D | W | G | G | P | T | A | F | A | L | A | A | T | H | P | E | A | V | E | K | L | A | I | L | D | V | V | I | - | P | G | D | G | G | D | - | - | - | - | - | - | - |
| 027 UniRef90\_A0A1M7IC97\_44\_328 | R | D | V | R | I | V | G | H | D | I | G | T | M | V | G | Y | S | Y | A | A | Q | Y | P | S | T | V | T | K | L | V | L | S | E | A | P | I | - | P | D | L | G | I | Y | - | S | I | P | - | - | - |
| 028 UniRef90\_UPI0016149F5E\_5\_284 | T | Q | Y | D | V | V | G | H | D | I | G | M | W | V | G | Y | A | L | A | S | D | F | P | Q | A | V | T | K | L | A | L | T | E | A | V | I | - | P | G | L | A | P | A | - | P | P | I | - | - | - |
| 029 UniRef90\_UPI00156E1DFF\_8\_289 | G | G | V | R | I | I | G | H | D | I | G | G | M | V | A | F | A | Y | A | R | R | H | P | E | G | V | E | R | L | V | L | V | E | L | A | L | - | P | G | L | G | L | E | - | Q | A | M | - | - | - |
| 030 UniRef90\_A0A3A8HRU0\_15\_300 | D | K | V | S | V | V | G | H | D | I | G | L | M | V | A | Y | A | Y | A | G | L | F | P | D | E | V | E | R | L | A | L | L | D | A | F | L | - | P | G | I | E | P | W | S | D | Q | V | - | - | - |
| 031 UniRef90\_A0A0M3UDU6\_18\_309 | G | P | I | D | V | V | G | H | D | V | G | A | W | I | A | Y | A | W | A | A | D | W | R | S | D | I | R | R | I | A | L | L | D | A | L | I | - | P | G | V | S | A | P | - | R | T | D | - | - | - |
| 032 UniRef90\_A0A447J1A9\_28\_317 | K | T | Y | S | V | V | G | H | D | I | G | M | W | V | G | Y | A | L | A | G | D | Y | P | A | D | I | K | K | I | V | L | T | E | A | V | I | - | P | G | L | A | P | A | - | P | G | I | - | - | - |
| 033 UniRef90\_A0A5C8T429\_14\_295 | R | Q | F | D | F | V | G | H | D | I | G | C | W | L | G | Y | P | F | V | A | T | Y | P | D | S | V | R | K | L | A | L | I | D | A | T | V | - | P | G | L | A | P | A | - | E | A | Y | - | - | - |
| 034 UniRef90\_A0A0N0TCD7\_1\_288 | D | R | F | A | V | V | G | Y | D | L | G | M | L | V | G | Y | A | L | A | S | R | H | R | V | R | V | T | R | L | A | V | S | E | A | V | I | - | P | G | L | S | P | S | - | P | P | L | - | - | - |
| 035 UniRef90\_W9ARP2\_16\_295 | E | P | I | S | L | V | G | H | D | L | G | S | T | L | A | L | S | F | A | L | R | Y | R | A | D | V | V | S | V | T | F | M | E | A | P | L | - | P | G | T | D | Y | Y | - | R | R | R | - | M | - |
| 036 UniRef90\_A0A109IGY4\_11\_301 | D | R | F | D | V | V | G | H | D | I | G | M | W | T | G | Y | A | L | A | A | D | H | P | E | R | V | G | R | L | A | V | V | D | A | I | I | - | P | G | L | T | P | S | - | P | S | V | - | - | - |
| 037 UniRef90\_K9DQK6\_24\_306 | D | R | A | D | V | V | G | H | D | I | G | T | M | V | A | Y | A | Y | A | V | R | Y | P | D | K | V | T | R | L | V | V | M | D | A | P | V | - | P | G | V | A | P | W | - | D | E | L | - | - | - |
| 038 UniRef90\_A0A1Q8IR73\_6\_294 | K | R | Y | S | L | A | A | H | D | V | G | A | W | V | A | F | P | Y | A | H | L | F | G | E | E | V | E | A | L | A | L | M | D | A | G | I | - | P | G | I | T | L | P | - | D | M | L | - | - | - |
| 039 UniRef90\_A0A1Y6D2S0\_29\_302 | G | P | A | F | V | V | G | H | D | M | G | G | K | A | A | Y | V | L | G | L | V | H | P | E | L | V | A | K | L | V | L | V | D | C | M | P | - | P | G | T | E | N | M | - | D | S | - | - | - | - |
| 040 UniRef90\_E6WJ64\_19\_301 | P | R | Y | Y | L | A | A | H | D | V | G | A | W | V | A | W | P | H | A | M | R | Y | S | Q | Q | V | I | K | L | A | L | L | D | A | G | I | - | P | G | I | T | L | P | - | E | A | L | - | - | - |
| 041 UniRef90\_UPI000489A94A\_43\_316 | R | K | V | K | L | V | G | H | D | I | G | L | M | V | A | Y | A | Y | A | A | Q | Y | P | D | E | V | Q | S | I | V | L | M | D | A | F | L | - | P | G | V | G | D | W | - | Q | Q | V | - | - | - |
| 042 UniRef90\_A0A4R6HBQ4\_11\_285 | A | T | A | W | I | V | G | H | D | L | G | G | Q | V | A | Y | P | L | A | A | N | W | P | Q | R | T | R | G | L | V | F | I | E | S | G | L | - | P | G | F | G | Q | E | - | R | A | M | - | - | - |
| 043 UniRef90\_A0A1M7QJX9\_32\_311 | K | H | I | N | L | A | G | H | D | I | G | L | M | V | A | Y | A | Y | A | A | Q | F | Q | S | D | V | K | K | I | A | L | M | D | A | L | L | - | P | G | V | E | P | V | - | - | - | - | - | - | - |
| 044 UniRef90\_A0A5B8WA72\_22\_300 | Q | H | V | N | V | V | G | H | D | I | G | L | M | V | A | Y | A | Y | A | A | Q | F | P | D | D | V | K | K | L | A | L | M | D | A | L | L | - | P | G | I | E | P | V | - | - | - | - | - | - | - |
| 045 UniRef90\_A0A0G2FGE6\_47\_303 | E | P | V | H | V | V | G | H | D | I | G | G | M | V | A | H | A | Y | A | S | R | F | A | N | E | T | A | S | V | A | L | G | E | F | P | M | - | P | G | T | K | V | Y | - | D | N | F | - | C | R |
| 046 UniRef90\_A0A1I6BK09\_10\_296 | A | R | I | N | L | V | G | Y | D | L | G | A | G | V | A | Y | A | Y | A | A | A | H | P | E | A | V | R | R | L | V | F | M | E | F | A | L | - | P | G | F | G | V | W | - | E | Q | G | - | I | T |
| 047 UniRef90\_A0A4R7C9R7\_33\_310 | P | Q | A | H | V | V | G | H | D | M | G | G | K | A | A | Y | I | L | A | H | L | H | P | G | V | V | S | K | L | V | L | V | D | C | L | L | - | P | G | T | E | N | M | - | D | A | - | - | - | - |
| 048 UniRef90\_A0A2M9M9Q1\_18\_305 | E | R | F | A | M | A | G | H | D | V | G | M | W | L | G | Y | A | L | A | A | D | H | G | D | A | V | T | R | L | A | L | L | D | A | T | L | - | P | G | L | A | P | D | - | L | P | L | - | - | - |
| 049 UniRef90\_A0A4V3T343\_11\_295 | G | R | A | H | L | V | G | H | D | I | G | A | M | V | A | Y | A | H | A | A | N | H | P | G | A | T | D | R | I | A | L | L | D | V | S | H | - | P | D | P | G | W | Y | - | E | G | T | - | L | L |
| 050 UniRef90\_UPI001616534D\_11\_303 | S | A | V | S | I | A | G | H | D | I | G | A | M | V | A | Y | A | F | A | A | N | H | P | E | A | T | T | K | I | A | L | L | D | V | P | H | - | P | D | R | T | W | S | - | V | F | G | - | L | L |
| 051 UniRef90\_J4PG95\_17\_290 | A | A | Y | Q | V | V | G | H | D | I | G | M | W | V | A | Y | A | L | A | S | D | Y | P | E | A | V | R | R | L | V | L | T | E | A | V | I | - | P | G | L | A | P | A | - | P | P | I | - | - | - |
| 052 UniRef90\_A0A0T1T741\_1\_295 | D | R | F | D | V | V | G | H | D | I | G | T | W | T | G | Y | A | L | A | A | D | H | P | E | R | V | G | R | L | A | I | V | E | A | V | I | - | P | G | L | T | P | S | - | P | P | L | - | - | - |
| 053 UniRef90\_G0FSK7\_17\_310 | E | R | F | A | V | A | G | H | D | V | G | M | W | I | G | Y | A | M | A | A | D | H | P | G | R | V | A | R | L | A | L | A | E | A | L | I | - | P | G | L | S | P | S | - | P | P | L | - | - | - |
| 054 UniRef90\_UPI00164AAB2D\_18\_303 | G | Q | A | V | I | I | G | H | D | I | G | T | M | V | A | Y | A | Y | A | A | R | Y | P | D | Q | T | T | K | L | V | V | M | D | A | P | V | - | P | G | I | P | P | W | - | E | Q | I | - | - | - |
| 055 UniRef90\_A0A1I2MT44\_20\_306 | G | T | F | A | V | V | G | H | D | I | G | M | W | T | G | Y | A | L | A | A | D | H | G | E | R | V | E | R | L | A | V | A | E | A | A | V | - | P | G | L | T | P | S | - | P | P | L | - | - | - |
| 056 UniRef90\_A0A2P2CCU5\_19\_306 | D | R | F | A | L | V | G | H | D | T | G | M | V | I | S | Y | A | L | A | A | D | F | P | Q | R | V | D | R | V | A | L | A | E | V | P | G | - | P | P | T | P | D | H | S | P | P | L | - | - | - |
| 057 UniRef90\_A0A2V9H5C8\_12\_298 | A | K | A | R | V | V | G | H | D | I | G | L | M | V | A | Y | A | Y | A | A | Q | F | P | S | E | V | E | K | L | V | V | M | D | A | F | L | - | P | G | V | G | E | W | - | E | A | V | - | - | - |
| 058 UniRef90\_A0A0P4V0Y0\_7\_291 | E | K | I | H | I | V | A | H | D | M | G | A | P | P | A | L | I | Y | T | N | D | Y | P | D | E | V | L | S | L | T | Y | L | E | E | P | V | - | L | L | Q | A | H | L | - | Q | Q | I | F | Q | F |
| 059 UniRef90\_A0A0Q4UQ58\_27\_305 | G | R | A | G | V | V | G | H | D | M | G | G | K | A | A | Y | V | L | A | H | L | H | P | D | Q | I | S | R | L | V | L | A | D | C | L | L | - | P | G | T | E | N | M | - | D | P | - | - | - | - |
| 060 UniRef90\_A0A6M4IM28\_20\_309 | T | Q | V | D | L | V | T | H | D | I | G | N | M | V | G | Y | A | F | A | A | Q | Q | P | A | R | V | R | R | F | A | L | I | D | A | P | L | - | P | G | V | G | P | W | - | D | D | L | - | - | - |
| 061 UniRef90\_A0A537J419\_20\_301 | Q | K | A | E | V | V | G | H | D | I | G | L | M | V | A | Y | A | Y | A | A | Q | F | P | T | E | V | E | K | L | V | V | M | D | A | F | L | - | P | G | V | G | A | W | - | D | A | V | - | - | - |
| 062 UniRef90\_A0A1V2PGN8\_6\_275 | D | Q | V | S | V | V | G | H | D | R | G | G | R | V | A | H | R | W | A | L | D | R | P | D | Q | V | T | R | L | A | V | L | D | I | I | - | - | P | T | R | A | M | W | - | Q | R | M | - | - | - |
| 063 UniRef90\_A0A542JC81\_20\_314 | E | R | F | A | V | A | G | H | D | T | G | M | V | S | A | Y | A | L | A | A | D | Y | S | E | R | V | D | R | V | V | L | A | E | I | P | A | P | P | G | A | T | P | A | - | P | P | L | - | - | - |
| 064 UniRef90\_UPI000424F353\_33\_308 | G | K | I | D | L | V | T | H | D | I | G | N | M | V | G | Y | A | F | A | A | E | Q | P | D | R | V | T | K | F | I | L | M | D | A | P | L | - | P | G | V | G | P | W | - | D | E | V | - | - | - |
| 065 UniRef90\_A0A2V9W2E9\_22\_299 | D | K | T | F | V | V | A | H | D | I | G | N | M | V | A | Y | A | Y | A | A | T | Y | P | D | K | V | E | R | L | V | V | M | D | A | P | I | - | P | G | V | E | P | W | - | K | E | I | - | - | - |
| 066 UniRef90\_A0A2T6L0M5\_16\_299 | D | G | I | Q | I | V | A | H | D | I | G | A | W | V | A | Y | P | Y | A | A | R | W | P | S | E | V | D | R | M | A | L | L | E | G | P | L | - | P | D | E | T | V | Y | - | D | Y | R | - | - | - |
| 067 UniRef90\_A0A261TYY2\_43\_315 | D | R | F | H | Y | V | G | H | D | V | G | V | W | I | G | Y | A | Y | A | S | R | H | G | H | T | L | N | K | L | A | L | I | D | A | T | I | - | P | G | L | T | P | P | - | E | A | Y | - | - | - |
| 068 UniRef90\_UPI0009781B11\_10\_287 | T | Q | C | R | L | V | G | H | D | V | G | S | W | I | S | Y | A | Y | A | A | Q | R | P | E | R | V | T | H | L | A | L | I | D | A | A | V | - | P | G | L | A | P | D | - | D | I | Y | - | - | - |
| 069 UniRef90\_A0A2V9TPC1\_35\_307 | Q | K | A | E | V | V | G | H | D | I | G | L | M | V | A | Y | A | Y | A | A | Q | F | P | T | E | V | T | K | L | V | L | M | D | A | F | L | - | P | G | V | E | G | W | - | E | A | V | - | - | - |
| 070 UniRef90\_UPI00145FA88A\_6\_279 | D | R | V | S | V | V | G | H | D | R | G | A | R | V | G | H | R | W | A | L | D | R | P | D | Q | V | E | R | L | S | V | L | D | I | V | - | - | P | T | R | E | M | W | - | R | R | W | - | - | - |
| 071 UniRef90\_UPI0003FADEBE\_21\_306 | D | K | A | H | V | V | G | H | D | I | G | T | M | V | A | Y | A | Y | A | A | S | Y | P | D | K | T | A | T | L | V | V | M | D | A | L | V | - | P | G | V | P | P | W | - | E | Q | I | - | - | - |
| 072 UniRef90\_A0A2V8Y3P4\_78\_329 | D | R | T | F | V | V | A | H | D | I | G | N | M | V | A | Y | A | Y | A | A | T | Y | P | D | T | V | E | R | L | V | V | M | D | A | P | I | - | P | G | I | D | P | W | - | N | E | I | - | - | - |
| 073 UniRef90\_A0A285BKA7\_8\_299 | E | R | F | A | V | V | G | Y | D | L | G | M | L | V | A | Y | A | L | A | A | S | H | R | N | R | V | T | H | F | V | G | A | E | S | I | L | - | P | G | L | T | P | F | - | P | P | M | - | - | - |
| 074 UniRef90\_A0A0K3AVW3\_44\_319 | N | Q | V | D | I | V | G | H | D | I | G | A | M | V | A | F | S | F | A | A | N | H | P | A | A | T | R | S | V | S | L | L | D | V | G | H | - | P | D | E | S | L | F | - | H | I | P | - | L | L |
| 075 UniRef90\_A0A4D7B7N1\_7\_276 | D | R | Y | F | I | G | A | H | D | I | G | A | W | V | A | Y | P | Y | V | A | R | Y | A | D | E | V | R | R | L | V | L | L | D | A | N | I | - | P | G | V | T | L | R | - | P | T | V | - | - | - |
| 076 UniRef90\_A0A2U0WF37\_17\_293 | E | H | A | V | V | V | G | H | D | I | G | T | M | V | A | F | A | Y | A | S | R | Y | P | Q | L | T | D | R | L | I | V | M | D | A | P | V | - | P | G | I | P | P | W | - | N | D | I | - | - | - |
| 077 UniRef90\_UPI00135C2D49\_17\_309 | E | S | V | D | I | A | G | H | D | I | G | A | M | V | A | Y | A | Y | A | A | N | H | P | E | A | T | R | R | I | C | L | L | D | V | P | H | - | P | D | G | T | W | S | - | D | L | R | - | L | L |
| 078 UniRef90\_UPI001473614E\_45\_322 | D | Q | V | S | I | I | G | H | D | L | G | V | L | V | A | Y | P | W | A | R | D | H | P | G | E | V | T | R | L | V | M | I | E | T | P | L | - | S | G | F | G | L | E | - | E | L | Y | - | - | - |
| 079 UniRef90\_J3F862\_22\_299 | E | H | S | V | V | V | G | H | D | I | G | T | M | V | A | F | A | Y | A | S | R | Y | P | Q | Q | T | D | R | L | V | V | M | D | A | P | V | - | P | G | I | P | P | W | - | N | E | I | - | - | - |
| 080 UniRef90\_A0A1A9NC01\_62\_345 | E | K | A | S | V | A | G | H | D | I | G | S | A | V | A | F | A | Y | A | A | N | Y | P | Q | A | T | D | R | L | V | M | L | E | L | P | H | - | P | D | D | S | L | L | - | S | F | P | - | L | L |
| 081 UniRef90\_UPI00131AC11B\_45\_327 | E | K | V | D | V | V | G | H | D | I | G | A | M | V | A | F | S | L | A | A | N | H | P | E | Q | V | R | K | L | V | M | L | D | V | A | H | - | P | S | P | G | Y | F | - | K | L | A | - | L | L |
| 082 UniRef90\_A0A4R8HC20\_22\_306 | A | S | A | H | V | V | G | H | D | I | G | A | M | V | A | Y | A | F | A | A | N | H | P | E | A | T | R | K | I | A | L | L | D | V | S | H | - | P | E | E | S | W | A | - | R | Q | T | - | L | L |
| 083 UniRef90\_A0A1B2HH26\_4\_280 | D | H | A | F | V | L | G | T | D | V | G | T | M | T | V | H | A | W | A | Q | R | H | P | R | D | V | T | R | L | V | L | S | E | C | F | L | - | P | G | F | G | L | E | - | E | H | L | - | - | - |
| 084 UniRef90\_A0A517LKB1\_23\_304 | E | K | I | H | V | V | G | H | D | I | G | G | M | I | A | H | A | Y | V | A | Q | F | P | A | D | T | E | S | L | I | W | G | E | C | P | L | - | P | G | S | T | V | Y | - | E | K | T | - | - | - |
| 085 UniRef90\_A0A0N1F3N8\_17\_298 | G | K | V | D | L | V | T | H | D | I | G | N | M | V | G | Y | A | F | A | A | Q | N | R | D | R | V | T | K | F | V | L | I | D | A | P | L | - | P | G | V | G | P | W | - | E | E | I | - | - | - |
| 086 UniRef90\_UPI0004CCFD44\_55\_340 | R | D | A | R | V | V | G | H | D | F | G | A | A | V | A | F | Q | Y | A | T | Q | F | P | A | D | T | A | R | L | G | Y | L | D | L | P | L | - | P | G | P | A | I | D | - | A | S | T | - | - | - |
| 087 UniRef90\_A0A4D4LDP7\_7\_304 | G | R | Y | W | L | F | A | H | D | I | G | S | W | V | A | F | T | Y | A | L | R | Y | A | D | E | L | A | G | L | A | L | L | D | A | G | I | - | P | G | V | T | L | P | - | E | T | L | - | - | - |
| 088 UniRef90\_A0A2V4B948\_8\_279 | D | R | V | A | V | A | G | H | D | R | G | G | R | V | A | H | R | W | A | L | D | R | P | D | Q | V | H | R | L | A | V | L | D | I | I | - | - | P | T | R | E | M | W | - | R | R | L | - | - | - |
| 089 UniRef90\_A0A2P2FV90\_3\_282 | E | R | V | S | V | V | G | H | D | R | G | G | R | V | A | H | R | W | A | L | D | R | P | D | Q | V | D | R | L | A | A | L | D | I | I | - | - | P | T | R | E | L | W | - | K | R | L | - | - | - |
| 090 UniRef90\_A0A4Q2J292\_17\_304 | G | K | V | D | V | V | A | H | D | I | G | N | M | V | A | Y | A | F | T | A | E | Q | P | D | R | V | T | K | L | V | L | M | D | A | P | L | - | P | G | V | G | P | W | - | D | E | I | - | - | - |
| 091 UniRef90\_UPI0012B05B7D\_16\_287 | E | N | F | L | Y | V | G | H | D | I | G | A | W | V | G | Y | A | F | G | H | L | Y | A | S | S | L | R | G | I | V | L | L | D | A | N | I | - | P | G | V | T | L | Q | - | D | T | I | - | - | - |
| 092 UniRef90\_UPI00048D4AD4\_4\_301 | G | A | V | S | I | A | G | H | D | I | G | A | M | V | A | Y | A | F | A | A | N | H | P | E | S | T | S | K | I | A | I | L | D | V | A | H | - | P | D | E | S | W | S | - | E | F | R | - | L | L |
| 093 UniRef90\_UPI0015F81933\_16\_310 | S | T | Y | W | L | V | A | H | D | I | G | A | W | V | A | F | S | L | A | L | K | Y | Q | S | R | L | R | G | L | A | L | L | D | A | G | I | - | P | G | I | T | L | P | - | D | A | I | - | - | - |
| 094 UniRef90\_UPI00160CFF45\_55\_340 | R | D | A | S | V | I | G | H | D | L | G | A | A | V | A | F | Q | Y | A | A | Q | F | P | A | D | T | A | R | L | G | Y | L | D | L | P | L | - | P | G | P | G | I | D | - | A | R | T | - | - | - |
| 095 UniRef90\_A0A1Q8KTW7\_17\_294 | Q | R | F | A | I | A | G | E | D | W | G | A | A | F | A | Y | A | V | A | A | T | Y | P | E | R | V | T | K | L | S | F | A | E | M | L | L | - | P | G | F | G | L | E | - | D | W | S | - | A | L |
| 096 UniRef90\_UPI00161A7EC4\_21\_297 | R | Q | V | G | L | I | G | H | D | L | G | A | L | I | A | Y | P | Y | A | R | D | F | P | N | E | V | T | K | L | A | V | L | E | T | P | L | - | A | G | F | G | L | E | - | N | L | Y | - | - | - |
| 097 UniRef90\_A0A1B4WXA8\_16\_299 | E | H | S | V | V | I | G | H | D | I | G | T | M | V | A | F | A | Y | A | S | R | Y | P | E | R | T | D | R | L | V | V | M | D | A | P | V | - | P | G | I | P | P | W | - | N | E | I | - | - | - |
| 098 UniRef90\_UPI000E46D2C1\_48\_330 | D | A | V | H | L | V | G | H | D | I | G | S | Q | V | A | Y | A | F | A | A | N | Y | P | E | A | T | T | S | V | S | F | L | D | V | S | A | - | Q | P | Q | S | T | R | - | D | M | R | - | L | V |
| 099 UniRef90\_A0A2N5CFI4\_23\_293 | A | K | A | D | V | V | A | H | D | I | G | N | M | V | A | Y | A | F | A | A | E | Q | P | E | R | V | T | K | L | V | L | M | D | A | P | I | - | P | G | V | G | P | W | - | E | D | I | - | - | - |
| 100 UniRef90\_A0A385B2U0\_45\_333 | K | G | V | Q | I | V | A | H | D | I | G | A | W | I | A | Y | P | Y | A | A | M | W | P | N | E | V | S | R | M | V | V | M | E | G | P | I | - | P | D | R | S | L | Y | - | T | F | P | - | - | - |
| 101 UniRef90\_A0A1Q7W147\_16\_308 | P | T | Y | W | L | A | G | H | D | V | G | A | W | V | A | F | S | L | A | L | N | Y | E | S | Q | L | R | G | L | A | L | L | D | A | G | I | - | P | G | V | T | L | P | - | E | A | I | - | - | - |
| 102 UniRef90\_A0A239MTD5\_1\_271 | N | D | I | Q | V | V | A | H | D | V | G | V | W | V | A | Y | A | Y | A | A | Q | W | P | S | E | V | R | R | M | A | V | M | E | A | P | I | - | A | D | G | S | A | Y | - | S | Y | P | - | - | - |
| 103 UniRef90\_UPI000561A6EF\_30\_315 | A | R | F | S | V | A | G | H | D | V | G | M | W | T | G | Y | A | L | A | A | D | H | R | E | R | V | E | R | L | A | L | A | E | A | L | V | - | A | G | I | S | P | S | - | P | P | L | - | - | - |
| 104 UniRef90\_A0A4Y8RHA3\_28\_294 | G | Q | V | E | V | V | A | H | D | I | G | N | M | V | A | Y | A | F | A | A | E | H | R | D | R | V | T | K | L | V | L | M | D | A | P | L | - | P | G | V | G | P | W | - | D | E | I | - | - | - |
| 105 UniRef90\_UPI0004DF415F\_15\_308 | P | K | Y | W | L | V | A | H | D | I | G | A | W | V | A | F | S | L | A | L | K | Y | E | E | Q | L | H | G | V | A | L | L | D | A | G | I | - | P | G | I | T | L | P | - | D | T | I | - | - | - |
| 106 UniRef90\_A0A2N3KZL1\_30\_325 | A | P | F | D | L | V | A | H | D | I | G | I | W | N | T | Y | P | M | A | V | S | H | Q | K | D | I | R | K | L | I | F | M | E | A | P | I | - | P | D | H | R | L | Y | - | D | F | P | - | - | - |
| 107 UniRef90\_UPI00076E3DF2\_55\_340 | R | D | A | R | V | V | G | H | D | F | G | A | A | V | A | F | Q | Y | A | A | Q | F | P | G | D | T | S | S | L | G | Y | L | D | L | P | L | - | P | G | P | E | L | D | - | A | S | T | - | - | - |
| 108 UniRef90\_UPI00130E1FA7\_66\_348 | D | R | A | H | I | V | G | H | D | I | G | S | H | M | A | Y | P | F | A | A | M | Y | P | E | A | T | I | S | T | T | Y | L | D | V | A | G | - | L | P | D | Y | V | R | - | D | I | K | - | L | L |
| 109 UniRef90\_A0A1I3FUH2\_46\_328 | E | K | A | H | I | V | G | H | D | I | G | S | H | V | A | Y | A | F | A | A | N | H | P | E | A | A | W | S | T | T | Y | L | D | V | A | G | - | L | P | A | S | I | K | - | D | L | K | - | L | L |
| 110 UniRef90\_UPI000E2836BA\_28\_313 | E | R | I | N | I | A | G | H | G | I | G | A | M | V | A | F | A | H | A | A | N | H | P | A | A | T | A | K | V | A | I | L | N | T | T | H | - | I | D | D | S | Y | Y | - | D | F | P | - | M | V |
| 111 UniRef90\_A0A653WNW3\_25\_297 | A | K | A | D | L | V | T | H | D | I | G | N | M | V | G | F | A | L | A | A | Q | Y | R | D | R | I | S | R | F | V | L | I | D | A | P | V | - | P | G | V | G | P | W | - | E | E | I | - | - | - |
| 112 UniRef90\_A0A5C1I8U5\_39\_321 | E | K | A | F | I | A | G | H | D | I | G | A | Q | V | A | F | S | V | A | A | N | H | S | E | L | V | E | K | L | I | I | M | D | V | P | H | - | P | D | E | S | F | A | - | A | V | L | - | M | L |
| 113 UniRef90\_UPI00165059AC\_4\_267 | G | S | A | T | V | I | G | H | D | R | G | A | R | V | A | H | R | W | A | L | D | H | P | G | D | V | E | R | L | V | V | M | D | I | I | - | - | P | T | R | E | M | W | - | R | R | M | - | - | - |
| 114 UniRef90\_F8JLL5\_12\_309 | S | T | Y | W | L | A | A | H | D | V | G | A | W | V | A | F | S | L | A | L | T | Y | R | S | H | L | R | G | L | A | L | L | D | A | G | I | - | P | G | I | T | L | P | - | D | A | I | - | - | - |
| 115 UniRef90\_A0A329J6I4\_30\_314 | Q | P | F | D | L | V | A | H | D | I | G | I | W | N | T | Y | P | L | A | V | M | H | Q | S | Q | I | K | K | L | V | F | M | E | A | P | I | - | P | D | K | S | V | Y | - | D | F | P | - | - | - |
| 116 UniRef90\_A0A3N1H0W7\_60\_340 | D | R | V | D | I | A | G | H | D | I | G | A | Q | I | A | F | S | F | A | A | N | H | P | E | A | T | G | R | I | A | M | L | D | I | L | H | - | P | D | E | S | V | Y | - | E | L | R | - | M | V |
| 117 UniRef90\_A0A316VRQ3\_1\_263 | Q | A | Y | S | V | L | G | H | D | I | G | S | M | V | A | T | A | Q | A | L | K | F | R | D | H | V | K | A | L | I | I | M | E | C | P | Q | - | P | G | T | S | V | Y | - | K | A | F | - | - | - |
| 118 UniRef90\_A0A4P8X818\_19\_291 | E | K | F | H | V | A | G | E | D | W | G | A | A | C | G | Y | A | L | A | A | A | Y | P | E | K | V | K | K | F | A | F | V | E | M | I | L | - | P | G | F | G | L | E | - | D | W | S | - | F | L |
| 119 UniRef90\_UPI0008405A69\_11\_309 | E | S | A | F | V | V | G | H | D | I | G | A | S | L | G | Y | H | L | A | A | N | F | P | Q | S | V | R | R | L | I | T | V | D | M | G | V | - | P | D | A | A | W | Q | - | A | I | S | - | M | L |
| 120 UniRef90\_A0A6I8M538\_3\_275 | P | K | V | A | V | A | G | H | D | R | G | G | R | V | A | H | R | W | A | L | D | R | P | D | Q | V | E | R | V | A | V | L | D | I | A | - | - | P | T | R | A | M | W | - | Q | R | L | - | - | - |
| 121 UniRef90\_UPI000B5CCBFE\_21\_300 | S | S | A | S | F | V | G | H | D | M | G | A | M | V | G | Y | S | F | A | A | N | H | P | E | A | T | R | R | L | A | L | L | D | V | V | H | - | P | D | E | S | Y | Y | - | S | R | P | - | M | L |
| 122 UniRef90\_A0A0M9ZDW3\_1\_266 | S | K | A | H | I | C | G | E | D | I | G | G | M | V | A | Y | S | Y | A | V | N | H | P | E | A | T | D | K | L | A | L | W | E | T | I | H | - | P | N | E | G | F | Y | - | G | I | P | - | M | L |
| 123 UniRef90\_UPI000E275A28\_18\_308 | E | H | F | A | M | V | G | H | D | C | G | M | W | V | G | Y | A | M | A | A | D | Q | P | Q | R | I | L | R | I | A | L | G | E | A | I | I | - | P | G | V | A | D | S | - | P | P | L | - | - | - |
| 124 UniRef90\_A0A3L8K0H7\_13\_298 | E | R | V | D | I | A | G | H | G | I | G | A | M | V | A | F | A | F | A | A | N | H | P | E | A | T | R | K | V | A | I | L | N | T | T | H | - | I | D | D | S | Y | Y | - | D | F | S | - | L | V |
| 125 UniRef90\_A0A6N7ZB66\_18\_297 | G | P | I | V | V | V | G | H | D | I | G | A | M | V | A | F | A | W | A | V | H | R | P | E | D | L | T | A | A | V | V | L | D | C | F | L | - | P | G | V | D | L | E | - | D | S | M | - | - | - |
| 126 UniRef90\_A0A2W7GM08\_52\_337 | D | K | V | D | I | V | G | H | D | I | G | S | M | V | A | Y | A | F | A | A | N | H | P | E | L | T | R | R | L | V | M | M | D | V | P | H | - | P | D | P | Q | L | A | - | T | W | P | - | L | L |
| 127 UniRef90\_A0A258JI33\_23\_300 | D | K | A | D | L | V | T | H | D | I | G | N | M | V | G | Y | A | L | T | A | Q | Y | P | A | R | I | T | K | W | V | I | I | D | A | P | L | - | P | G | I | G | P | W | - | D | E | I | - | - | - |
| 128 UniRef90\_A0A6A6C1N5\_26\_297 | S | P | I | H | I | I | G | H | D | I | G | G | M | V | A | Y | A | L | A | S | R | H | P | H | R | V | R | S | V | I | W | G | E | C | P | L | - | P | G | T | E | T | Y | - | R | L | D | - | - | - |
| 129 UniRef90\_UPI00160A92C2\_5\_284 | E | R | I | A | L | V | G | H | D | R | G | A | R | V | A | T | R | F | A | K | D | H | R | A | M | V | D | R | L | V | V | L | D | N | I | - | - | P | T | R | V | V | F | - | E | T | T | - | - | - |
| 130 UniRef90\_UPI0009E804D9\_8\_293 | G | P | S | V | V | V | G | H | D | I | G | A | M | V | S | F | A | W | A | A | A | H | P | D | E | V | A | A | L | I | A | I | D | V | I | F | - | P | G | L | G | L | E | - | E | A | M | - | - | - |
| 131 UniRef90\_UPI00049086FA\_57\_348 | H | G | V | R | I | A | A | H | D | F | G | V | G | V | A | Y | A | L | A | A | Q | H | R | E | Q | V | A | G | L | L | L | M | D | F | P | L | - | V | G | K | A | L | A | - | F | D | A | - | - | - |
| 132 UniRef90\_A5FF96\_16\_286 | K | K | I | N | I | A | G | H | D | I | G | A | A | V | A | F | S | Y | A | A | N | F | P | E | N | T | D | K | L | I | I | L | D | T | P | H | - | P | D | E | N | I | Y | - | K | L | P | - | M | M |
| 133 UniRef90\_UPI0016621A44\_4\_265 | A | R | F | A | V | V | G | H | D | W | G | G | T | V | G | Y | L | V | A | A | A | H | P | E | R | V | W | A | L | A | V | E | E | E | V | L | - | P | G | I | D | A | E | - | - | - | - | - | - | I |
| 134 UniRef90\_UPI000B83B944\_2\_264 | D | K | V | H | M | A | G | H | D | I | G | A | Q | V | A | Y | A | Y | A | A | Q | F | P | E | A | T | T | K | L | V | M | M | D | V | P | H | - | P | F | E | G | F | L | - | S | I | P | - | L | L |
| 135 UniRef90\_A0A0B4DHQ5\_15\_286 | E | K | I | S | L | I | G | H | D | R | G | A | R | V | A | T | R | F | A | K | D | Y | P | E | R | V | D | R | L | V | V | M | D | N | V | - | - | P | T | R | I | V | A | - | R | D | L | - | - | - |
| 136 UniRef90\_UPI0009B31CF7\_18\_283 | A | D | A | F | V | V | G | I | D | L | G | V | P | V | A | W | T | L | A | M | R | H | S | K | R | I | L | R | L | A | V | M | E | G | L | L | - | G | R | L | P | G | A | - | E | A | F | - | L | - |
| 137 UniRef90\_UPI001661B99F\_34\_324 | A | R | I | S | V | A | G | H | D | V | G | G | G | V | A | L | A | Y | A | A | K | Y | R | D | R | V | D | H | L | A | I | L | E | A | - | - | - | P | P | T | T | W | Y | - | L | Q | L | - | - | - |
| 138 UniRef90\_UPI00104123D8\_9\_274 | T | D | L | T | L | I | G | H | D | A | G | G | M | V | T | Y | A | Y | L | R | Q | Y | G | D | - | L | A | R | A | V | I | M | N | T | V | I | - | P | G | V | D | P | W | - | T | E | V | - | L | - |
| 139 UniRef90\_A0A1M7Z6I5\_10\_283 | G | R | I | A | L | V | G | H | D | R | G | A | R | V | A | T | R | F | A | K | D | H | P | G | L | L | D | R | L | V | V | M | D | N | V | - | - | P | T | R | I | V | A | - | R | S | M | - | - | - |
| 140 UniRef90\_A0A431M1N9\_22\_292 | Q | S | V | V | L | V | T | H | D | I | G | N | M | V | G | Y | A | L | A | A | G | Y | P | G | R | V | T | K | W | I | V | M | D | A | P | L | - | P | G | L | G | T | W | - | D | K | Q | - | - | - |
| 141 UniRef90\_UPI0013D28676\_49\_329 | G | P | I | D | L | A | G | H | D | W | G | G | N | I | A | L | A | Y | A | L | A | Y | R | S | D | V | R | Q | L | A | V | L | E | A | - | - | - | P | A | A | E | D | Y | - | T | T | L | - | - | - |
| 142 UniRef90\_A0A402BD85\_4\_199 | G | P | I | N | L | V | G | H | D | I | V | L | M | V | A | Y | A | Y | E | A | L | Y | P | S | T | V | Q | H | L | A | L | L | E | A | P | I | - | P | D | A | S | I | Y | - | T | L | P | - | - | - |
| 143 UniRef90\_A0A2V5S4I0\_55\_307 | D | K | A | R | V | V | G | H | D | I | G | L | M | V | A | Y | A | Y | A | A | Q | F | P | G | E | T | E | K | L | A | V | M | D | A | F | L | - | P | G | V | P | G | W | - | E | P | I | - | - | - |
| 144 UniRef90\_A0A328AN40\_21\_303 | D | R | F | S | V | V | G | H | D | W | G | A | S | V | A | W | W | M | A | T | I | R | P | Q | P | I | E | R | L | A | I | M | N | A | P | H | - | P | A | V | W | K | Q | - | A | M | T | - | - | - |
| 145 UniRef90\_A0A0M9AP20\_25\_244 | E | Q | A | H | I | V | G | H | D | W | G | G | V | V | A | W | Q | A | A | I | D | R | P | E | V | V | D | Q | L | A | V | L | N | A | P | H | - | P | S | A | Y | E | R | - | E | L | R | - | - | - |
| 146 UniRef90\_A0A1N6RLL9\_1\_202 | K | Q | V | Y | I | V | G | H | D | I | G | G | M | V | T | Y | A | L | L | R | L | Y | P | E | T | I | R | G | V | M | I | L | D | S | G | L | - | P | G | L | S | P | W | - | E | E | N | - | - | K |
| 147 UniRef90\_A0A4R3LTR2\_48\_312 | D | R | F | A | L | V | G | H | D | R | G | G | R | V | A | H | R | L | A | L | D | W | P | E | A | V | E | K | L | C | V | I | D | I | A | - | - | P | T | A | T | M | Y | - | A | M | T | - | - | - |
| 148 UniRef90\_UPI0002ED95C2\_14\_285 | D | R | F | A | V | A | G | H | D | R | G | G | L | V | A | F | R | A | G | L | D | H | P | E | A | I | T | H | L | A | I | L | D | V | L | - | - | P | A | A | D | T | W | - | R | A | L | - | - | - |
| 149 UniRef90\_A0A1Q7MBV0\_16\_212 | D | R | A | D | V | V | G | H | D | W | G | A | H | I | A | W | H | L | A | M | W | H | P | Q | R | V | R | R | L | A | I | L | N | V | P | H | - | P | A | R | M | W | R | - | G | L | R | - | - | - |
| 150 UniRef90\_A0A1A0KI05\_7\_212 | D | R | V | V | L | G | G | Y | D | V | G | S | R | I | A | Q | A | L | A | R | Q | H | P | E | R | I | V | G | L | V | V | - | S | P | P | F | - | P | G | V | G | D | R | - | - | - | - | - | - | - |

  
  

|  |  |  |  |  |  |  |  |  |  |  |  |  |  |  |  |  |  |  |  |  |  |  |  |  |  |  |  |  |  |  |  |  |  |  |  |  |  |  |  |  |  |  |  |  |  |  |  |  |  |  |
| --- | --- | --- | --- | --- | --- | --- | --- | --- | --- | --- | --- | --- | --- | --- | --- | --- | --- | --- | --- | --- | --- | --- | --- | --- | --- | --- | --- | --- | --- | --- | --- | --- | --- | --- | --- | --- | --- | --- | --- | --- | --- | --- | --- | --- | --- | --- | --- | --- | --- | --- |
| **001 Input\_protein\_seq** | - | - | - | - | - | - | - | - | - | - | - | K | A | S | - | A | D | P | Q | I | W | H | - | F | G | L | H | Q | - | Q | R | - | D | I | A | E | M | L | - | I | A | - | G | K | - | - | - | E | R | A |
| 002 UniRef90\_UPI00158A2D6D\_3\_306 | - | - | - | - | - | - | - | - | - | - | - | K | A | R | - | S | D | P | Q | I | W | H | - | F | G | L | H | Q | - | Q | R | - | D | I | A | E | M | L | - | I | A | - | G | K | - | - | - | E | H | A |
| 003 UniRef90\_A0A1Z4J856\_27\_318 | - | - | - | - | - | - | - | - | - | - | - | - | N | K | - | A | S | A | A | L | W | H | - | F | S | F | Q | A | - | T | R | - | D | L | P | E | A | L | - | V | E | - | G | K | - | - | - | E | R | L |
| 004 UniRef90\_G7LVZ3\_7\_288 | - | - | - | - | - | - | - | - | - | - | - | N | T | A | - | K | F | P | Q | F | W | H | - | F | G | F | F | S | - | A | P | - | G | V | A | E | T | L | - | I | A | - | G | R | - | - | - | E | K | M |
| 005 UniRef90\_A0A2I8F4B9\_53\_324 | V | - | - | - | - | - | - | - | - | - | - | - | - | - | - | Q | T | P | R | T | W | H | - | F | R | F | Y | S | - | V | Q | - | D | V | P | E | M | L | - | I | A | - | G | H | - | - | - | E | L | E |
| 006 UniRef90\_A0A327RPK7\_10\_284 | - | - | - | - | - | - | - | - | - | - | - | - | D | V | - | A | N | G | G | M | W | H | - | F | G | F | H | M | - | V | H | - | D | L | P | E | A | L | - | I | T | - | G | R | - | - | - | E | H | I |
| 007 UniRef90\_A0A6L3SWG4\_20\_294 | L | G | - | - | - | - | - | - | - | - | - | - | - | - | - | D | T | K | R | V | W | H | - | F | H | F | H | R | - | A | P | - | D | L | P | E | L | L | - | T | A | - | G | R | - | - | - | E | Q | L |
| 008 UniRef90\_A0A401ZLI5\_4\_287 | - | - | - | - | - | - | - | - | - | - | - | - | - | - | - | T | K | L | K | I | W | H | - | F | G | L | F | Q | - | A | P | R | N | L | A | E | S | L | - | A | E | - | G | R | - | - | - | E | H | I |
| 009 UniRef90\_A0A4V2U6R1\_12\_297 | A | - | - | - | - | - | - | - | - | - | T | T | K | F | - | S | Q | A | K | L | W | H | - | F | H | L | H | G | A | A | G | - | N | V | A | E | M | L | - | I | C | - | G | N | - | - | - | E | F | A |
| 010 UniRef90\_A0A5A5T922\_6\_282 | A | - | - | - | - | - | - | - | - | - | - | - | - | - | - | L | G | N | D | A | W | F | - | A | T | F | H | R | - | I | P | - | N | L | P | E | T | L | - | I | S | - | G | R | - | - | - | E | R | V |
| 011 UniRef90\_A0A2V6UIN9\_3\_273 | - | - | - | - | - | - | - | - | - | - | - | - | - | W | - | L | L | R | D | L | W | H | - | F | H | F | F | G | - | - | - | - | K | T | P | L | A | L | - | V | E | - | G | R | - | - | - | E | R | I |
| 012 UniRef90\_UPI001669B3C5\_41\_314 | - | - | - | - | - | - | - | - | - | - | - | - | - | W | - | L | L | R | D | L | W | H | - | F | H | F | Y | G | - | - | - | - | E | V | P | L | A | L | - | V | E | - | G | R | - | - | - | E | R | I |
| 013 UniRef90\_A0A4R2Z7A8\_3\_305 | P | - | - | - | - | - | - | - | - | - | V | T | P | D | - | K | S | W | K | T | W | H | - | F | A | F | H | T | - | L | P | - | D | L | P | E | A | L | - | I | T | - | G | N | - | - | - | E | R | I |
| 014 UniRef90\_A0A5C5SVV1\_11\_281 | F | - | - | - | - | - | - | - | - | - | - | - | - | - | - | G | D | P | R | V | W | H | - | F | S | F | H | M | - | K | E | - | N | L | P | E | R | L | - | L | Y | - | G | R | - | - | - | E | Y | D |
| 015 UniRef90\_L9WLS3\_16\_284 | - | - | - | - | - | - | - | - | - | - | - | - | - | - | - | E | K | R | H | F | W | H | - | T | R | F | H | G | - | V | R | - | D | L | P | E | R | L | - | V | A | - | G | R | - | - | - | E | R | M |
| 016 UniRef90\_UPI00131EC7B5\_30\_311 | P | - | - | - | - | - | - | - | - | - | V | T | P | D | - | K | A | W | K | T | W | H | - | F | A | F | H | A | - | I | P | - | D | L | P | E | A | L | - | I | A | - | G | K | - | - | - | E | L | V |
| 017 UniRef90\_C7QAM5\_50\_332 | - | - | - | - | - | - | - | - | - | - | S | L | T | A | - | D | G | P | G | A | W | H | - | F | G | F | F | A | - | L | T | N | G | L | P | E | Q | L | - | I | S | - | G | R | - | - | - | E | E | L |
| 018 UniRef90\_A0A1Q3SXP6\_11\_288 | - | - | - | - | - | - | - | - | - | - | - | - | - | W | - | L | L | R | D | L | W | H | - | F | H | F | Y | G | - | - | - | - | E | V | P | L | K | L | - | V | E | - | G | R | - | - | - | E | R | I |
| 019 UniRef90\_UPI00149248BF\_39\_315 | L | - | - | - | - | - | - | - | - | - | L | P | P | E | - | A | N | A | G | M | A | Q | - | F | M | F | N | Q | - | L | R | - | D | L | P | E | F | L | - | V | A | - | G | R | - | - | - | E | D | P |
| 020 UniRef90\_UPI000361D127\_26\_313 | L | - | - | - | - | - | - | - | - | - | G | D | R | R | - | T | S | D | F | L | W | H | - | F | N | F | N | R | - | A | H | - | D | V | N | E | Q | L | - | V | Q | - | G | R | - | - | - | E | D | V |
| 021 UniRef90\_A0A0M4FVH2\_24\_312 | P | - | - | - | - | - | - | - | - | - | T | A | P | E | - | R | A | W | K | T | W | H | - | F | A | F | H | V | - | I | P | - | D | L | P | E | M | L | - | I | A | - | G | R | - | - | - | E | R | E |
| 022 UniRef90\_A0A1Q4ZL08\_9\_290 | - | - | - | - | - | - | - | - | - | - | S | L | T | P | - | S | G | P | G | L | W | N | - | F | G | F | F | S | - | L | P | N | G | L | P | E | Q | I | - | V | A | - | G | R | - | - | - | E | E | L |
| 023 UniRef90\_S3CY91\_25\_307 | - | - | - | - | - | - | - | - | - | - | - | - | - | - | - | N | S | P | G | M | W | H | - | F | T | F | H | Q | - | Q | T | - | D | L | P | E | A | L | - | V | A | - | G | R | - | - | - | E | R | I |
| 024 UniRef90\_UPI0010F95BB2\_18\_294 | - | - | - | - | - | - | - | - | - | - | - | - | N | V | - | A | Q | G | G | S | W | H | - | F | G | F | N | A | - | A | G | - | D | I | A | E | A | L | - | V | A | - | G | R | - | - | - | E | H | M |
| 025 UniRef90\_A0A0N1GDE2\_28\_316 | F | - | - | - | - | - | - | - | - | - | S | P | A | A | - | V | N | Q | R | L | W | H | - | F | G | F | N | R | - | L | T | - | D | L | N | E | E | L | - | V | R | - | G | R | - | - | - | E | R | L |
| 026 UniRef90\_A0A2E5PJQ7\_5\_284 | - | - | - | - | - | - | - | - | - | - | - | - | F | S | - | Q | G | G | R | R | W | H | - | H | P | F | H | I | - | T | P | - | D | L | P | E | A | L | - | T | K | - | G | R | - | - | - | E | D | I |
| 027 UniRef90\_A0A1M7IC97\_44\_328 | - | - | - | - | - | - | - | - | - | - | S | L | T | A | - | R | G | P | G | V | W | N | - | F | G | F | F | N | - | L | T | D | G | L | P | E | N | M | - | I | K | - | G | R | - | - | - | E | V | A |
| 028 UniRef90\_UPI0016149F5E\_5\_284 | F | - | - | - | - | - | - | - | - | - | V | A | P | A | - | D | N | I | F | L | W | H | - | F | M | F | N | Q | - | V | P | - | D | L | P | E | M | L | - | T | A | - | G | K | - | - | - | E | R | E |
| 029 UniRef90\_UPI00156E1DFF\_8\_289 | - | - | - | - | - | - | - | - | - | - | - | - | D | V | - | A | S | G | G | L | F | H | - | F | G | L | F | M | - | T | E | - | E | L | P | E | L | L | - | L | D | - | G | R | - | - | - | E | S | D |
| 030 UniRef90\_A0A3A8HRU0\_15\_300 | - | - | - | - | - | - | - | - | - | - | - | - | - | M | - | S | S | L | Q | V | W | H | - | F | T | F | N | G | - | - | - | - | P | T | A | E | K | L | - | V | Q | - | G | R | - | - | - | E | R | I |
| 031 UniRef90\_A0A0M3UDU6\_18\_309 | L | - | - | - | - | - | - | - | - | - | S | V | D | E | - | A | N | R | R | S | W | H | - | F | A | F | N | Q | - | L | D | - | D | L | P | E | L | L | - | I | S | - | G | R | - | - | - | E | D | V |
| 032 UniRef90\_A0A447J1A9\_28\_317 | F | - | - | - | - | - | - | - | - | - | V | D | P | E | - | E | N | I | F | L | W | H | - | F | M | F | N | Q | - | V | Q | - | D | L | P | E | T | L | - | A | A | - | G | K | - | - | - | E | R | E |
| 033 UniRef90\_A0A5C8T429\_14\_295 | A | - | - | - | - | - | - | - | - | - | F | A | P | E | - | R | I | G | R | N | W | H | - | F | F | F | N | A | - | L | S | - | D | L | P | E | T | L | - | L | A | - | G | R | - | - | - | E | R | E |
| 034 UniRef90\_A0A0N0TCD7\_1\_288 | M | - | - | - | - | - | - | - | - | - | S | D | P | A | - | T | N | E | M | L | W | H | - | F | A | F | N | R | - | L | P | - | D | I | N | E | R | M | - | V | A | - | G | R | - | - | - | E | E | I |
| 035 UniRef90\_W9ARP2\_16\_295 | - | - | - | - | - | - | - | - | - | - | - | - | - | - | - | A | Q | K | S | A | W | Q | - | F | S | F | H | A | - | N | P | - | D | I | A | V | Y | L | - | T | H | - | G | R | - | - | - | E | R | W |
| 036 UniRef90\_A0A109IGY4\_11\_301 | F | - | - | - | - | - | - | - | - | - | S | P | A | A | - | A | N | Q | R | F | W | H | - | F | G | F | N | R | - | L | D | - | D | L | N | E | E | L | - | V | R | - | G | R | - | - | - | E | R | L |
| 037 UniRef90\_K9DQK6\_24\_306 | - | - | - | - | - | - | - | - | - | - | - | - | - | V | - | L | D | P | R | L | W | H | - | F | N | F | G | G | - | - | - | - | K | E | A | E | R | L | - | V | A | - | G | R | - | - | - | E | R | I |
| 038 UniRef90\_A0A1Q8IR73\_6\_294 | P | - | - | - | - | - | - | - | - | - | S | S | S | D | - | R | S | W | K | T | W | H | - | F | S | F | H | A | - | V | P | - | D | L | P | E | I | L | - | L | A | - | G | R | - | - | - | E | R | A |
| 039 UniRef90\_A0A1Y6D2S0\_29\_302 | - | - | - | - | - | - | - | - | - | - | - | - | - | - | - | A | K | G | G | M | W | H | - | Y | G | F | H | M | - | A | A | - | G | F | P | E | M | L | - | T | K | - | N | R | - | - | - | E | R | E |
| 040 UniRef90\_E6WJ64\_19\_301 | P | - | - | - | - | - | - | - | - | - | A | T | P | D | - | K | A | W | K | T | W | H | - | F | A | F | H | L | - | L | P | - | D | L | P | E | A | L | - | I | S | - | G | R | - | - | - | E | E | I |
| 041 UniRef90\_UPI000489A94A\_43\_316 | - | - | - | - | - | - | - | - | - | - | - | - | - | F | - | L | L | R | D | K | W | H | - | F | N | F | Y | G | - | - | - | - | A | T | P | L | A | L | - | V | H | - | G | R | - | - | - | E | R | V |
| 042 UniRef90\_A0A4R6HBQ4\_11\_285 | - | - | - | - | - | - | - | - | - | - | - | - | D | V | - | A | N | G | G | S | W | H | - | F | G | F | N | R | - | A | G | - | D | I | A | E | E | L | - | V | R | - | G | R | - | - | - | E | H | L |
| 043 UniRef90\_A0A1M7QJX9\_32\_311 | - | - | - | - | - | - | - | - | - | - | - | W | S | Q | - | V | R | A | Q | A | W | W | - | F | G | F | F | A | - | M | P | - | N | S | G | E | - | I | - | V | A | - | G | K | - | - | - | A | G | L |
| 044 UniRef90\_A0A5B8WA72\_22\_300 | - | - | - | - | - | - | - | - | - | - | - | W | S | Q | - | V | K | A | Q | A | W | W | - | F | G | F | F | A | - | Q | P | - | H | A | G | E | - | L | - | V | S | - | G | K | - | - | - | V | G | L |
| 045 UniRef90\_A0A0G2FGE6\_47\_303 | - | - | - | - | - | - | - | - | - | - | - | - | - | - | - | E | N | P | G | V | W | H | - | F | H | F | H | W | - | Q | T | - | D | L | P | E | L | L | - | T | Q | - | G | R | - | - | - | E | R | Q |
| 046 UniRef90\_A0A1I6BK09\_10\_296 | P | - | - | - | - | - | - | - | - | - | G | P | D | W | - | H | N | G | V | N | W | H | - | A | A | L | F | T | - | L | P | - | D | V | A | E | S | F | - | M | G | - | G | Q | - | - | - | E | R | K |
| 047 UniRef90\_A0A4R7C9R7\_33\_310 | - | - | - | - | - | - | - | - | - | - | - | - | - | - | - | L | R | G | G | A | W | H | - | Y | G | F | H | M | - | A | P | - | D | F | P | E | M | L | - | T | K | - | G | R | - | - | - | E | R | D |
| 048 UniRef90\_A0A2M9M9Q1\_18\_305 | F | - | - | - | - | - | - | - | - | - | G | S | E | A | - | Q | N | D | L | L | W | H | - | F | A | F | N | R | - | K | R | - | S | I | N | E | L | L | - | V | R | - | G | R | - | - | - | E | H | L |
| 049 UniRef90\_A0A4V3T343\_11\_295 | P | R | P | G | - | - | - | - | - | - | - | - | - | - | - | - | R | F | H | K | W | W | - | F | A | F | N | Q | - | V | E | - | D | L | P | E | Q | L | - | L | A | - | G | R | - | - | - | Y | H | H |
| 050 UniRef90\_UPI001616534D\_11\_303 | P | E | P | D | Q | H | L | D | S | V | I | E | A | G | - | A | R | S | Y | L | W | W | - | F | A | F | N | Q | - | V | R | - | G | L | P | E | R | L | - | L | E | - | G | R | - | - | - | S | R | L |
| 051 UniRef90\_J4PG95\_17\_290 | F | - | - | - | - | - | - | - | - | - | V | P | P | A | - | E | N | I | F | L | W | H | - | F | M | F | N | Q | - | L | Q | - | D | L | P | E | A | L | - | I | T | - | G | R | - | - | - | E | R | A |
| 052 UniRef90\_A0A0T1T741\_1\_295 | F | - | - | - | - | - | - | - | - | - | G | P | A | A | - | A | N | L | K | L | W | Q | - | F | G | F | N | R | - | L | T | - | D | L | N | E | E | L | - | V | R | - | G | R | - | - | - | E | R | L |
| 053 UniRef90\_G0FSK7\_17\_310 | F | - | - | - | - | - | - | - | - | - | A | P | R | D | - | V | V | E | R | L | W | H | - | F | A | F | N | Q | - | L | D | - | D | L | N | E | Q | L | - | V | A | - | G | R | - | - | - | E | D | V |
| 054 UniRef90\_UPI00164AAB2D\_18\_303 | - | - | - | - | - | - | - | - | - | - | - | - | - | V | - | R | L | P | A | L | W | H | - | F | D | F | G | G | - | - | - | - | K | D | A | L | R | L | - | V | Q | - | G | R | - | - | - | E | R | I |
| 055 UniRef90\_A0A1I2MT44\_20\_306 | F | - | - | - | - | - | - | - | - | - | S | A | A | E | - | V | N | D | R | L | W | H | - | F | A | F | N | R | - | L | G | - | G | I | N | E | K | L | - | V | E | - | G | R | - | - | - | E | H | L |
| 056 UniRef90\_A0A2P2CCU5\_19\_306 | F | - | - | - | - | - | - | - | - | - | V | P | R | A | - | L | N | D | R | I | W | H | - | I | A | F | N | R | - | A | G | - | A | V | A | E | E | L | - | V | A | - | G | R | - | - | - | E | A | L |
| 057 UniRef90\_A0A2V9H5C8\_12\_298 | - | - | - | - | - | - | - | - | - | - | - | - | - | Y | - | N | N | P | G | F | W | H | - | F | R | F | H | G | - | - | - | - | P | T | P | E | A | L | - | V | E | - | G | R | - | - | - | E | R | T |
| 058 UniRef90\_A0A0P4V0Y0\_7\_291 | S | - | - | - | - | - | - | - | - | - | P | Q | S | T | - | Q | N | G | G | L | W | W | - | W | T | F | F | L | - | A | P | - | E | L | P | E | T | L | - | I | A | - | G | R | - | - | - | E | Q | E |
| 059 UniRef90\_A0A0Q4UQ58\_27\_305 | - | - | - | - | - | - | - | - | - | - | - | - | - | - | - | L | R | G | G | A | W | H | - | Y | G | F | H | M | - | A | P | - | D | I | P | E | K | L | - | T | A | - | G | R | - | - | - | E | R | G |
| 060 UniRef90\_A0A6M4IM28\_20\_309 | - | - | - | - | - | - | - | - | - | - | - | - | - | I | - | R | S | H | Q | L | W | H | - | F | S | F | W | G | - | - | - | - | P | D | A | E | R | L | - | V | A | - | G | R | - | - | - | E | R | I |
| 061 UniRef90\_A0A537J419\_20\_301 | - | - | - | - | - | - | - | - | - | - | - | - | - | Y | - | N | N | P | A | N | W | H | - | F | R | F | N | G | - | - | - | - | P | I | P | E | A | L | - | V | H | - | G | R | - | - | - | E | R | I |
| 062 UniRef90\_A0A1V2PGN8\_6\_275 | - | - | - | - | - | - | - | - | - | - | - | N | A | E | - | V | G | R | G | Y | W | H | - | W | L | F | H | L | - | Q | P | - | D | L | P | E | L | L | - | V | G | - | Q | N | - | - | - | I | A | A |
| 063 UniRef90\_A0A542JC81\_20\_314 | F | - | - | - | - | - | - | - | - | - | V | P | A | P | - | L | N | A | K | L | W | H | - | I | P | F | N | R | - | A | E | - | G | L | A | E | Q | L | - | I | A | - | G | R | - | - | - | E | D | V |
| 064 UniRef90\_UPI000424F353\_33\_308 | - | - | - | - | - | - | - | - | - | - | - | - | - | L | - | K | N | P | L | L | W | H | - | F | R | F | G | G | - | - | - | - | P | D | M | E | R | L | - | V | K | - | G | R | - | - | - | E | R | I |
| 065 UniRef90\_A0A2V9W2E9\_22\_299 | - | - | - | - | - | - | - | - | - | - | - | - | - | L | - | Q | N | P | G | V | W | H | - | F | N | F | H | G | - | - | - | - | P | D | A | E | R | L | - | V | A | - | G | R | - | - | - | E | R | I |
| 066 UniRef90\_A0A2T6L0M5\_16\_299 | A | - | - | - | - | - | - | - | - | - | L | D | P | D | - | G | G | P | S | I | W | H | - | Y | G | F | F | Q | - | A | - | - | D | I | A | E | G | L | - | I | A | - | G | N | - | - | - | E | R | A |
| 067 UniRef90\_A0A261TYY2\_43\_315 | A | - | - | - | - | - | - | - | - | - | F | E | P | Q | - | R | I | S | K | N | W | H | - | F | Y | F | F | A | - | M | P | - | D | L | A | E | A | L | - | L | T | - | G | R | - | - | - | E | R | E |
| 068 UniRef90\_UPI0009781B11\_10\_287 | R | - | - | - | - | - | - | - | - | - | L | T | P | Q | - | T | L | E | K | T | W | H | - | F | A | F | N | F | - | L | P | - | E | L | S | E | L | L | - | V | T | - | G | R | - | - | - | E | R | E |
| 069 UniRef90\_A0A2V9TPC1\_35\_307 | - | - | - | - | - | - | - | - | - | - | - | - | - | Y | - | N | N | P | D | I | W | H | - | F | R | F | N | G | - | - | - | - | P | T | P | E | A | L | - | V | Q | - | G | R | - | - | - | E | R | T |
| 070 UniRef90\_UPI00145FA88A\_6\_279 | - | - | - | - | - | - | - | - | - | - | - | N | T | E | - | V | G | A | A | Y | W | H | - | W | M | F | H | L | - | Q | P | - | D | L | P | E | L | L | - | A | G | - | K | D | - | - | - | V | A | G |
| 071 UniRef90\_UPI0003FADEBE\_21\_306 | - | - | - | - | - | - | - | - | - | - | - | - | - | V | - | R | L | P | L | L | W | H | - | F | D | F | G | G | - | - | - | - | K | D | A | L | R | L | - | V | R | - | G | R | - | - | - | E | R | I |
| 072 UniRef90\_A0A2V8Y3P4\_78\_329 | - | - | - | - | - | - | - | - | - | - | - | - | - | L | - | L | N | P | G | V | W | H | - | F | N | F | H | G | - | - | - | - | P | D | A | E | R | L | - | V | V | - | G | R | - | - | - | E | R | I |
| 073 UniRef90\_A0A285BKA7\_8\_299 | L | - | - | - | - | - | - | - | - | - | T | D | P | T | - | V | N | E | V | M | A | H | - | F | V | I | N | R | - | L | A | - | D | L | N | E | R | L | - | I | A | - | G | R | - | - | - | E | E | I |
| 074 UniRef90\_A0A0K3AVW3\_44\_319 | A | P | A | G | - | - | - | - | - | - | - | - | - | - | - | V | P | V | H | V | W | W | - | F | A | F | N | Q | - | V | R | - | T | L | P | E | Q | L | - | L | T | - | G | R | - | - | - | F | R | Y |
| 075 UniRef90\_A0A4D7B7N1\_7\_276 | D | - | - | - | - | - | - | - | - | - | V | G | P | D | - | - | N | W | K | S | W | H | - | F | F | F | H | P | - | V | P | - | D | L | P | E | A | L | - | I | T | - | G | R | - | - | - | E | R | L |
| 076 UniRef90\_A0A2U0WF37\_17\_293 | - | - | - | - | - | - | - | - | - | - | - | - | - | V | - | R | S | P | M | L | W | H | - | F | D | F | G | G | - | - | - | - | P | D | A | E | R | L | - | V | A | - | G | R | - | - | - | E | R | I |
| 077 UniRef90\_UPI00135C2D49\_17\_309 | P | G | P | D | Q | H | V | N | T | V | I | E | D | G | - | D | R | A | F | L | W | W | - | F | A | F | N | Q | - | V | G | - | G | L | P | E | K | L | - | L | E | - | G | R | - | - | - | F | R | L |
| 078 UniRef90\_UPI001473614E\_45\_322 | - | - | - | - | - | - | - | - | - | - | - | - | - | - | - | - | - | G | V | S | W | H | - | F | R | F | N | M | - | S | P | A | P | I | P | E | R | I | - | M | D | - | N | D | - | - | - | D | V | S |
| 079 UniRef90\_J3F862\_22\_299 | - | - | - | - | - | - | - | - | - | - | - | - | - | V | - | R | S | P | M | L | W | H | - | F | D | F | G | G | - | - | - | - | P | D | A | E | R | L | - | V | A | - | G | R | - | - | - | E | R | I |
| 080 UniRef90\_A0A1A9NC01\_62\_345 | P | P | Q | G | N | V | G | D | K | - | L | - | G | S | - | A | R | P | Y | L | W | W | - | F | A | F | N | Q | - | I | H | - | G | L | P | E | K | L | - | L | A | - | G | R | - | - | - | V | G | L |
| 081 UniRef90\_UPI00131AC11B\_45\_327 | P | E | A | G | T | F | G | D | K | - | I | - | D | E | - | D | H | P | Y | L | W | W | - | F | A | F | H | Q | - | V | K | - | G | L | P | E | D | L | - | L | E | - | G | R | - | - | - | A | G | L |
| 082 UniRef90\_A0A4R8HC20\_22\_306 | P | R | H | G | - | - | - | - | - | - | - | - | - | - | - | - | Q | R | H | K | W | W | - | F | A | F | N | Q | - | V | H | - | G | L | P | E | Q | L | - | L | A | - | G | R | - | - | - | F | H | L |
| 083 UniRef90\_A0A1B2HH26\_4\_280 | - | - | - | - | - | - | - | - | - | - | - | - | - | - | - | - | - | A | S | L | W | H | - | F | G | F | H | A | - | Q | S | - | E | F | A | A | Q | L | - | V | A | - | G | R | - | - | - | E | E | Q |
| 084 UniRef90\_A0A517LKB1\_23\_304 | K | - | - | - | - | - | - | - | - | - | - | - | - | - | - | H | D | P | D | L | W | H | - | F | D | F | Q | S | H | H | P | - | D | L | A | V | S | L | - | V | T | - | G | K | - | - | - | E | E | L |
| 085 UniRef90\_A0A0N1F3N8\_17\_298 | - | - | - | - | - | - | - | - | - | - | - | - | - | L | - | K | N | P | L | L | W | H | - | F | R | L | G | G | - | - | - | - | P | D | M | E | R | L | - | V | A | - | G | R | - | - | - | E | R | I |
| 086 UniRef90\_UPI0004CCFD44\_55\_340 | - | - | - | - | - | - | - | - | - | - | - | - | - | - | - | Y | R | S | L | S | W | H | - | I | A | F | H | S | - | Q | R | - | R | V | P | E | A | V | - | V | G | - | D | D | - | - | - | V | R | E |
| 087 UniRef90\_A0A4D4LDP7\_7\_304 | P | - | - | - | - | - | - | - | - | - | L | N | P | A | - | G | A | W | K | M | W | H | - | F | A | F | H | Q | - | V | P | - | D | L | P | E | V | L | - | L | E | - | G | K | - | - | - | E | R | E |
| 088 UniRef90\_A0A2V4B948\_8\_279 | - | - | - | - | - | - | - | - | - | - | - | D | S | G | - | L | G | R | A | Y | W | H | - | W | L | F | H | L | - | Q | P | - | D | L | P | E | R | L | - | A | G | - | Q | D | - | - | - | V | A | G |
| 089 UniRef90\_A0A2P2FV90\_3\_282 | - | - | - | - | - | - | - | - | - | - | - | D | T | E | - | I | G | R | A | Y | W | H | - | W | L | F | H | L | - | Q | P | - | D | L | P | E | L | L | - | A | G | - | R | D | - | - | - | I | A | A |
| 090 UniRef90\_A0A4Q2J292\_17\_304 | - | - | - | - | - | - | - | - | - | - | - | - | - | L | - | K | N | P | L | L | W | H | - | F | R | F | G | G | - | - | - | - | P | D | M | E | R | L | - | V | K | - | G | R | - | - | - | E | R | I |
| 091 UniRef90\_UPI0012B05B7D\_16\_287 | T | - | - | - | - | - | - | - | - | - | L | G | P | D | - | - | N | W | R | N | W | H | - | F | L | F | N | P | - | I | A | - | D | L | P | E | A | L | - | L | A | - | G | R | - | - | - | E | R | I |
| 092 UniRef90\_UPI00048D4AD4\_4\_301 | P | Q | P | D | Q | H | V | D | G | D | I | L | R | G | - | Q | R | S | Y | L | W | W | - | F | A | F | N | Q | - | V | R | - | A | L | P | E | K | L | - | L | D | - | G | R | - | - | - | T | R | P |
| 093 UniRef90\_UPI0015F81933\_16\_310 | P | - | - | - | - | - | - | - | - | - | T | D | P | E | - | Q | A | W | K | T | W | H | - | F | A | F | H | L | - | V | P | - | D | L | P | E | T | L | - | L | A | - | G | R | - | - | - | E | R | E |
| 094 UniRef90\_UPI00160CFF45\_55\_340 | - | - | - | - | - | - | - | - | - | - | - | - | - | - | - | Y | R | S | L | S | W | H | - | I | A | F | H | S | - | Q | P | - | K | V | P | E | T | V | - | V | G | - | N | D | - | - | - | V | R | D |
| 095 UniRef90\_A0A1Q8KTW7\_17\_294 | T | A | - | - | - | - | - | - | - | - | D | N | V | F | - | S | D | H | F | L | W | H | - | V | G | F | F | H | - | V | R | - | D | F | P | E | M | L | - | I | S | - | G | R | - | - | - | E | E | Q |
| 096 UniRef90\_UPI00161A7EC4\_21\_297 | - | - | - | - | - | - | - | - | - | - | - | - | - | - | - | - | - | G | I | S | F | H | - | F | K | F | N | M | - | A | P | A | P | V | P | E | S | I | - | L | D | - | N | D | - | - | - | D | V | P |
| 097 UniRef90\_A0A1B4WXA8\_16\_299 | - | - | - | - | - | - | - | - | - | - | - | - | - | V | - | R | S | P | M | L | W | H | - | F | D | F | G | G | - | - | - | - | P | D | M | E | R | L | - | V | A | - | G | R | - | - | - | E | R | I |
| 098 UniRef90\_UPI000E46D2C1\_48\_330 | P | S | A | P | L | T | - | - | - | - | G | E | F | R | - | Q | D | F | F | L | W | W | - | F | A | F | N | Q | - | V | H | - | D | M | P | E | E | L | - | I | E | - | G | R | - | - | - | A | H | I |
| 099 UniRef90\_A0A2N5CFI4\_23\_293 | - | - | - | - | - | - | - | - | - | - | - | - | - | L | - | K | S | P | L | L | W | H | - | F | R | F | G | G | - | - | - | - | P | D | M | E | R | L | - | V | K | - | G | R | - | - | - | E | R | I |
| 100 UniRef90\_A0A385B2U0\_45\_333 | A | - | - | - | - | - | - | - | - | - | F | P | A | E | - | G | G | L | S | T | W | H | - | L | G | F | F | Q | - | - | K | - | D | F | A | Q | D | L | - | V | R | - | G | H | - | - | - | E | R | D |
| 101 UniRef90\_A0A1Q7W147\_16\_308 | P | - | - | - | - | - | - | - | - | - | T | D | P | N | - | L | A | W | K | T | W | H | - | F | A | F | H | L | - | V | P | - | D | L | P | E | T | L | - | L | A | - | G | R | - | - | - | E | R | E |
| 102 UniRef90\_A0A239MTD5\_1\_271 | A | - | - | - | - | - | - | - | - | - | L | N | A | D | P | E | K | P | S | P | W | H | - | W | G | M | F | Q | - | M | P | - | - | L | A | E | H | L | - | I | A | - | G | H | - | - | - | E | R | V |
| 103 UniRef90\_UPI000561A6EF\_30\_315 | L | - | - | - | - | - | - | - | - | - | G | D | H | E | - | A | N | R | R | L | W | H | - | F | A | F | N | R | - | L | D | - | G | L | N | E | E | L | - | V | R | - | G | R | - | - | - | E | R | L |
| 104 UniRef90\_A0A4Y8RHA3\_28\_294 | - | - | - | - | - | - | - | - | - | - | - | - | - | L | - | K | N | P | L | L | W | H | - | F | R | F | G | G | - | - | - | - | P | D | M | E | R | L | - | V | A | - | G | R | - | - | - | E | R | I |
| 105 UniRef90\_UPI0004DF415F\_15\_308 | P | - | - | - | - | - | - | - | - | - | T | D | P | D | - | H | A | W | K | T | W | H | - | F | A | F | H | L | - | V | T | - | D | L | P | E | T | L | - | L | T | - | G | R | - | - | - | E | R | E |
| 106 UniRef90\_A0A2N3KZL1\_30\_325 | A | - | - | - | - | - | - | - | - | - | F | T | P | E | - | G | E | S | L | V | W | H | - | F | S | F | F | A | - | A | G | N | N | L | A | E | T | L | - | V | T | - | G | H | - | - | - | E | R | M |
| 107 UniRef90\_UPI00076E3DF2\_55\_340 | - | - | - | - | - | - | - | - | - | - | - | - | - | - | - | Y | R | S | M | S | W | H | - | I | A | F | H | S | - | Q | P | - | E | I | P | E | A | V | - | V | G | - | D | D | - | - | - | V | R | D |
| 108 UniRef90\_UPI00130E1FA7\_66\_348 | P | E | K | D | T | S | - | - | - | - | T | P | F | R | - | E | N | F | F | I | W | W | - | F | A | F | H | Q | - | V | H | - | D | M | P | A | E | L | - | I | E | - | G | R | - | - | - | A | H | I |
| 109 UniRef90\_A0A1I3FUH2\_46\_328 | P | E | P | G | S | S | - | - | - | - | G | D | F | A | - | D | T | F | F | L | W | W | - | F | A | F | H | Q | - | V | N | - | G | M | P | E | K | L | - | I | E | - | G | R | - | - | - | A | H | I |
| 110 UniRef90\_UPI000E2836BA\_28\_313 | P | R | P | G | D | Q | - | - | - | - | - | - | - | - | - | - | G | P | H | R | W | W | - | L | A | F | Q | Q | - | V | Q | - | D | L | P | E | Q | L | - | L | A | D | G | R | - | - | - | Y | R | H |
| 111 UniRef90\_A0A653WNW3\_25\_297 | - | - | - | - | - | - | - | - | - | - | - | - | - | L | - | K | N | P | L | L | W | H | - | F | R | F | G | G | - | - | - | - | P | D | M | E | R | L | - | V | A | - | G | R | - | - | - | E | R | I |
| 112 UniRef90\_A0A5C1I8U5\_39\_321 | P | A | L | G | T | P | T | D | K | - | L | - | D | P | - | A | R | P | F | L | W | W | - | F | A | F | N | Q | - | M | R | - | G | L | P | E | D | L | - | L | E | - | G | R | - | - | - | F | E | M |
| 113 UniRef90\_UPI00165059AC\_4\_267 | - | - | - | - | - | - | - | - | - | - | - | D | A | D | - | L | A | Q | R | T | W | H | - | W | L | F | H | L | - | Q | P | - | D | L | P | E | L | I | - | - | - | - | - | D | - | - | - | I | G | A |
| 114 UniRef90\_F8JLL5\_12\_309 | P | - | - | - | - | - | - | - | - | - | T | D | P | E | - | Q | A | W | K | T | W | H | - | F | A | F | H | L | - | V | P | - | D | L | P | E | T | L | - | L | A | - | G | R | - | - | - | E | R | E |
| 115 UniRef90\_A0A329J6I4\_30\_314 | A | - | - | - | - | - | - | - | - | - | F | S | P | E | - | G | E | S | L | V | W | H | - | F | S | F | F | A | - | A | G | D | H | M | A | E | T | L | - | I | K | - | G | H | - | - | - | E | K | F |
| 116 UniRef90\_A0A3N1H0W7\_60\_340 | P | L | P | G | - | - | - | - | - | - | - | - | - | - | - | Q | P | Y | H | P | W | W | - | F | A | F | N | Q | - | V | A | - | G | L | P | E | R | L | - | L | A | - | G | R | - | - | - | S | R | L |
| 117 UniRef90\_A0A316VRQ3\_1\_263 | T | - | - | - | - | - | - | - | - | - | T | E | P | E | L | T | L | G | P | T | F | H | - | F | F | F | H | H | - | A | D | - | N | L | P | E | Q | L | - | T | Y | - | G | R | - | - | - | E | D | L |
| 118 UniRef90\_A0A4P8X818\_19\_291 | T | T | - | - | - | - | - | - | - | - | E | N | V | N | - | S | N | R | W | L | W | H | - | I | N | F | Y | S | - | V | P | - | D | F | P | E | L | L | - | I | T | - | G | R | - | - | - | E | R | Q |
| 119 UniRef90\_UPI0008405A69\_11\_309 | P | G | S | D | E | Q | V | E | A | - | L | A | H | G | - | L | G | G | Y | P | W | W | - | F | A | F | N | Q | - | L | R | - | G | L | P | E | A | L | - | L | A | - | D | R | - | - | - | F | R | L |
| 120 UniRef90\_A0A6I8M538\_3\_275 | - | - | - | - | - | - | - | - | - | - | - | D | T | G | - | V | A | Q | A | Y | W | H | - | W | L | F | H | L | - | Q | P | - | D | L | P | E | L | L | - | A | G | - | Q | N | - | - | - | I | A | A |
| 121 UniRef90\_UPI000B5CCBFE\_21\_300 | R | R | P | N | - | - | - | - | - | - | - | - | - | - | - | T | G | F | N | M | W | W | - | M | A | F | N | Q | - | V | R | - | G | L | P | E | Q | V | - | F | A | - | D | R | - | - | - | G | W | Y |
| 122 UniRef90\_A0A0M9ZDW3\_1\_266 | P | Q | P | A | - | - | - | - | - | - | - | - | - | - | - | Q | D | G | L | Q | W | W | - | L | A | F | N | M | - | V | K | - | D | L | P | E | A | V | - | L | T | - | G | R | - | - | - | F | R | T |
| 123 UniRef90\_UPI000E275A28\_18\_308 | I | P | - | - | - | - | - | - | - | - | D | D | R | K | - | I | S | D | F | L | W | H | - | N | N | F | C | R | - | A | R | - | G | I | N | E | E | M | - | V | A | - | G | R | - | - | - | E | E | I |
| 124 UniRef90\_A0A3L8K0H7\_13\_298 | P | R | P | D | H | Q | - | - | - | - | - | - | - | - | - | - | G | P | H | R | W | W | - | L | A | F | Q | Q | - | V | E | - | N | L | P | E | K | L | - | L | T | D | G | R | - | - | - | Y | R | H |
| 125 UniRef90\_A0A6N7ZB66\_18\_297 | - | - | - | - | - | - | - | - | - | - | - | - | N | V | - | V | K | G | G | S | W | H | - | F | G | F | F | A | - | A | P | - | H | I | P | E | M | L | - | F | A | - | G | H | - | - | - | E | L | E |
| 126 UniRef90\_A0A2W7GM08\_52\_337 | P | G | V | G | Q | F | E | D | K | - | V | G | D | G | - | S | K | A | Y | P | W | W | - | F | A | Y | H | Q | - | V | K | - | G | L | P | E | Q | L | G | A | D | - | G | R | - | - | - | I | R | L |
| 127 UniRef90\_A0A258JI33\_23\_300 | - | - | - | - | - | - | - | - | - | - | - | - | - | L | - | K | S | P | L | L | W | H | - | F | N | F | R | G | - | - | - | - | P | D | V | D | R | L | - | V | K | - | G | R | - | - | - | E | R | I |
| 128 UniRef90\_A0A6A6C1N5\_26\_297 | - | - | - | - | - | - | - | - | R | - | T | V | P | S | - | R | T | T | Q | Q | F | H | - | F | V | F | H | T | - | V | P | - | D | L | A | A | M | L | - | V | Q | - | G | K | - | - | - | E | K | G |
| 129 UniRef90\_UPI00160A92C2\_5\_284 | - | - | - | - | - | - | - | - | - | - | - | D | A | K | - | I | A | K | L | Y | W | F | - | F | Y | F | Q | Q | - | V | P | - | H | L | P | E | A | L | - | V | Q | - | G | R | - | - | - | E | E | M |
| 130 UniRef90\_UPI0009E804D9\_8\_293 | - | - | - | - | - | - | - | - | - | - | - | - | N | V | - | A | T | G | G | M | W | H | - | F | G | F | F | M | - | Q | P | - | H | I | P | E | M | L | - | F | D | - | G | H | - | - | - | E | L | E |
| 131 UniRef90\_UPI00049086FA\_57\_348 | - | - | - | - | - | - | - | - | - | - | - | - | - | - | - | V | R | P | L | S | W | H | - | F | S | F | N | L | - | Q | E | - | P | L | D | E | Q | L | - | V | T | - | G | R | - | - | - | E | G | A |
| 132 UniRef90\_A5FF96\_16\_286 | P | I | G | - | - | - | - | - | - | - | - | - | - | - | - | N | P | V | F | P | W | W | - | L | A | F | N | Q | - | V | K | - | Q | L | P | E | E | L | - | L | E | - | G | R | - | - | - | Y | D | I |
| 133 UniRef90\_UPI0016621A44\_4\_265 | P | - | - | - | - | - | - | - | - | - | E | P | G | R | - | S | H | Y | P | D | W | H | - | G | P | F | N | R | - | R | A | - | G | L | A | E | T | L | - | V | P | - | G | R | - | - | - | E | D | A |
| 134 UniRef90\_UPI000B83B944\_2\_264 | P | P | P | G | V | Y | V | M | - | - | S | D | K | E | - | R | P | I | Y | P | W | W | - | F | S | L | N | S | - | V | P | - | D | L | P | E | K | L | - | L | Q | - | G | K | Q | - | - | M | S | I |
| 135 UniRef90\_A0A0B4DHQ5\_15\_286 | - | - | - | - | - | - | - | - | - | - | - | N | A | E | - | I | A | K | A | Y | W | F | - | F | L | F | H | L | - | V | P | - | D | L | P | E | V | L | - | I | S | - | G | K | - | - | - | E | E | Q |
| 136 UniRef90\_UPI0009B31CF7\_18\_283 | - | - | - | - | - | - | - | - | - | - | - | - | - | - | - | A | G | G | P | P | W | W | - | F | G | F | H | G | - | V | P | - | G | L | A | E | R | V | - | V | E | - | G | R | - | - | - | E | A | E |
| 137 UniRef90\_UPI001661B99F\_34\_324 | - | - | - | - | - | - | - | - | - | - | V | A | D | Q | - | P | N | Y | L | W | W | D | S | F | V | N | G | Q | - | K | A | - | G | M | A | E | Q | L | - | V | A | - | G | R | - | - | - | E | K | V |
| 138 UniRef90\_UPI00104123D8\_9\_274 | - | - | - | - | - | - | - | - | - | - | - | - | - | - | - | A | N | P | Y | I | W | H | - | F | G | F | H | A | - | V | A | - | A | L | P | E | A | L | - | V | Q | - | G | H | - | - | - | Q | A | E |
| 139 UniRef90\_A0A1M7Z6I5\_10\_283 | - | - | - | - | - | - | - | - | - | - | - | N | A | Q | - | I | A | R | A | Y | W | F | - | F | M | F | H | L | - | V | P | - | D | L | P | E | A | L | - | I | Y | - | G | R | - | - | - | E | D | I |
| 140 UniRef90\_A0A431M1N9\_22\_292 | - | - | - | - | - | - | - | - | - | - | - | - | - | L | - | V | N | P | K | T | W | H | - | F | N | F | R | G | - | - | - | - | P | D | V | E | R | L | - | V | A | - | G | R | - | - | - | E | R | I |
| 141 UniRef90\_UPI0013D28676\_49\_329 | - | - | - | - | - | - | - | - | - | - | V | R | Q | Q | - | P | G | T | F | W | W | D | W | F | I | N | G | S | - | N | P | - | G | V | A | E | R | L | - | V | D | - | G | Q | - | - | - | P | G | T |
| 142 UniRef90\_A0A402BD85\_4\_199 | - | - | - | - | - | - | - | - | - | - | A | L | T | A | - | K | G | P | G | V | W | W | - | F | G | F | F | S | - | T | P | - | Q | M | P | E | K | L | - | I | Q | - | G | R | - | - | - | E | L | T |
| 143 UniRef90\_A0A2V5S4I0\_55\_307 | - | - | - | - | - | - | - | - | - | - | - | - | - | Y | - | N | N | P | N | T | W | H | - | F | R | F | N | G | - | - | - | - | E | Y | P | E | K | L | - | V | R | - | G | R | - | - | - | E | R | T |
| 144 UniRef90\_A0A328AN40\_21\_303 | - | - | - | - | - | - | - | - | - | - | T | D | P | E | - | Q | R | N | K | S | R | Y | - | V | Q | M | L | R | - | I | P | - | A | L | P | E | L | M | - | V | R | - | A | G | - | - | - | G | Y | K |
| 145 UniRef90\_A0A0M9AP20\_25\_244 | - | - | - | - | - | - | - | - | - | - | C | S | A | D | - | Q | L | L | R | S | W | Y | - | V | L | F | F | Q | - | L | P | - | V | L | P | E | A | S | - | L | R | - | W | N | - | - | - | D | F | T |
| 146 UniRef90\_A0A1N6RLL9\_1\_202 | - | - | - | - | - | - | - | - | - | - | - | - | - | - | - | A | N | P | F | L | W | H | - | F | G | F | H | Q | - | T | P | - | D | V | P | E | K | L | - | I | T | - | G | R | - | - | - | Q | F | I |
| 147 UniRef90\_A0A4R3LTR2\_48\_312 | - | - | - | - | - | - | - | - | - | - | - | D | K | A | F | A | T | R | Y | F | W | W | - | F | F | L | I | Q | - | P | Y | - | P | L | P | E | R | M | - | I | G | - | A | D | - | - | - | P | D | Y |
| 148 UniRef90\_UPI0002ED95C2\_14\_285 | - | - | - | - | - | - | - | - | - | - | - | H | G | T | - | S | G | I | F | A | F | H | L | Y | L | L | A | Q | - | R | T | - | D | L | A | E | R | M | - | I | R | - | A | D | - | - | - | S | D | L |
| 149 UniRef90\_A0A1Q7MBV0\_16\_212 | - | - | - | - | - | - | - | - | - | - | - | T | L | R | - | Q | L | R | K | S | W | Y | - | M | F | F | F | Q | - | L | P | - | L | L | P | E | W | L | - | M | S | - | P | R | - | - | - | - | - | - |
| 150 UniRef90\_A0A1A0KI05\_7\_212 | V | - | - | - | - | - | - | - | - | - | L | G | E | G | - | P | Q | R | E | F | W | Y | - | Q | A | F | H | Q | - | L | P | - | - | V | A | D | A | L | - | I | D | - | G | K | P | D | A | V | R | A |

  
  

|  |  |  |  |  |  |  |  |  |  |  |  |  |  |  |  |  |  |  |  |  |  |  |  |  |  |  |  |  |  |  |  |  |  |  |  |  |  |  |  |  |  |  |  |  |  |  |  |  |  |  |
| --- | --- | --- | --- | --- | --- | --- | --- | --- | --- | --- | --- | --- | --- | --- | --- | --- | --- | --- | --- | --- | --- | --- | --- | --- | --- | --- | --- | --- | --- | --- | --- | --- | --- | --- | --- | --- | --- | --- | --- | --- | --- | --- | --- | --- | --- | --- | --- | --- | --- | --- |
| **001 Input\_protein\_seq** | Y | I | - | L | D | F | Y | K | K | R | T | H | - | - | - | - | - | - | - | - | - | - | - | - | - | V | A | L | - | S | N | D | D | I | A | V | Y | A | D | A | Y | - | A | A | P | G | A | L | R | A |
| 002 UniRef90\_UPI00158A2D6D\_3\_306 | Y | I | - | L | D | F | Y | K | K | R | I | H | - | - | - | - | - | - | - | - | - | - | - | - | - | V | P | L | - | S | D | D | D | I | A | V | Y | A | D | A | Y | - | A | A | P | G | A | L | R | A |
| 003 UniRef90\_A0A1Z4J856\_27\_318 | Y | L | - | S | V | F | L | R | P | F | T | Y | - | - | - | - | - | - | - | - | - | N | - | P | - | A | A | I | - | T | E | D | A | M | D | E | Y | V | R | T | Y | - | S | A | P | G | G | M | R | A |
| 004 UniRef90\_G7LVZ3\_7\_288 | F | L | - | A | H | F | I | R | H | L | A | Y | - | - | - | - | - | - | - | - | - | D | - | T | - | Y | A | V | - | T | E | D | D | L | N | E | Y | S | E | R | M | - | S | A | P | G | A | L | R | A |
| 005 UniRef90\_A0A2I8F4B9\_53\_324 | Y | L | - | K | W | F | H | N | S | E | A | V | - | - | - | - | - | - | - | - | - | N | - | A | - | R | A | F | - | T | N | E | V | E | E | T | Y | A | R | E | Y | - | A | M | P | G | A | L | R | A |
| 006 UniRef90\_A0A327RPK7\_10\_284 | Y | L | - | D | H | F | F | K | T | L | A | Y | - | - | - | - | - | - | - | - | - | N | - | P | - | T | S | I | - | K | E | E | A | I | A | E | Y | V | R | Q | Y | - | S | A | P | G | A | L | R | A |
| 007 UniRef90\_A0A6L3SWG4\_20\_294 | Y | L | - | E | R | F | Y | L | D | L | A | F | - | - | - | - | - | - | - | - | - | D | - | I | - | E | A | I | - | G | A | E | A | V | A | R | Y | V | R | A | F | - | T | R | P | G | A | M | R | A |
| 008 UniRef90\_A0A401ZLI5\_4\_287 | L | L | - | Q | W | F | F | S | R | - | A | R | - | - | - | - | - | - | - | - | - | N | - | S | - | A | A | F | - | T | Q | E | D | I | D | E | Y | V | R | C | Y | - | S | G | R | D | A | L | R | A |
| 009 UniRef90\_A0A4V2U6R1\_12\_297 | Y | L | - | R | S | F | Y | L | R | L | C | A | - | - | - | - | - | - | - | - | - | N | - | P | - | D | A | I | - | S | I | A | D | A | E | R | Y | V | A | D | Y | - | T | A | P | G | A | M | R | A |
| 010 UniRef90\_A0A5A5T922\_6\_282 | Y | L | - | S | W | F | Y | E | N | L | P | A | - | - | - | - | - | - | - | - | - | V | - | K | - | G | A | I | - | T | E | A | D | I | D | E | Y | V | R | T | Y | - | S | D | P | G | A | L | R | A |
| 011 UniRef90\_A0A2V6UIN9\_3\_273 | Y | F | - | E | H | F | W | N | D | F | A | A | - | - | - | - | - | - | - | - | - | N | - | P | A | H | S | V | - | S | E | A | N | R | Q | L | Y | A | A | A | Y | - | A | Q | P | G | S | M | R | A |
| 012 UniRef90\_UPI001669B3C5\_41\_314 | Y | L | - | E | H | F | W | N | D | F | A | A | - | - | - | - | - | - | - | - | - | D | - | R | N | H | S | V | - | S | E | A | D | R | R | I | Y | A | R | A | Y | - | A | Q | P | G | A | M | R | A |
| 013 UniRef90\_A0A4R2Z7A8\_3\_305 | Y | L | - | D | W | F | L | R | R | K | T | A | - | - | - | - | - | - | - | - | - | A | - | P | - | D | A | F | - | T | D | E | D | I | S | E | Y | L | R | V | F | - | L | K | S | G | G | L | R | A |
| 014 UniRef90\_A0A5C5SVV1\_11\_281 | Y | V | - | S | T | F | I | F | D | R | A | F | - | - | - | - | - | - | - | - | - | D | - | H | - | G | A | H | - | A | L | E | D | I | E | V | F | A | R | A | F | - | A | Q | P | G | R | T | R | G |
| 015 UniRef90\_L9WLS3\_16\_284 | Y | L | - | E | W | F | Y | K | E | G | A | Y | - | - | - | - | - | - | - | - | - | D | - | P | - | A | A | I | - | D | S | D | A | R | D | E | Y | V | R | C | Y | - | S | Q | P | G | G | L | R | G |
| 016 UniRef90\_UPI00131EC7B5\_30\_311 | Y | L | - | D | W | F | L | K | R | K | T | A | - | - | - | - | - | - | - | - | - | C | - | P | - | D | V | F | - | S | A | E | D | M | A | E | Y | L | R | I | L | - | M | L | N | G | G | L | R | A |
| 017 UniRef90\_C7QAM5\_50\_332 | W | T | - | S | K | F | V | D | D | L | E | V | - | - | - | - | - | - | - | - | - | V | - | K | - | G | A | V | - | T | P | N | D | I | D | V | F | S | S | Y | L | - | K | D | P | A | H | L | E | A |
| 018 UniRef90\_A0A1Q3SXP6\_11\_288 | Y | L | - | E | H | F | W | N | D | F | A | A | - | - | - | - | - | - | - | - | - | D | - | K | T | K | S | V | - | P | E | Q | D | R | R | F | Y | A | E | S | Y | - | A | Q | P | G | A | M | K | A |
| 019 UniRef90\_UPI00149248BF\_39\_315 | Y | L | - | R | W | L | V | G | Q | L | A | F | - | - | - | - | - | - | - | - | - | R | - | P | - | D | R | V | - | - | - | - | A | V | D | E | Y | V | R | A | Y | - | S | E | P | G | A | M | K | A |
| 020 UniRef90\_UPI000361D127\_26\_313 | Y | F | - | G | Y | Q | F | A | T | K | A | A | - | - | - | - | - | - | - | - | - | T | - | P | - | T | A | I | - | P | S | T | A | V | D | V | Y | V | E | A | I | - | R | L | P | G | A | L | R | A |
| 021 UniRef90\_A0A0M4FVH2\_24\_312 | Y | L | - | D | W | F | L | R | R | K | A | A | - | - | - | - | - | - | - | - | - | N | - | P | - | E | A | F | - | S | E | A | D | I | E | E | Y | L | R | V | F | - | K | K | A | G | G | L | R | A |
| 022 UniRef90\_A0A1Q4ZL08\_9\_290 | W | V | - | D | R | F | T | D | S | M | E | V | - | - | - | - | - | - | - | - | - | Q | - | K | - | T | G | I | - | G | A | D | E | V | A | E | Y | S | R | Y | L | - | Q | D | E | A | H | L | R | A |
| 023 UniRef90\_S3CY91\_25\_307 | Y | L | - | K | H | F | Y | D | R | L | C | V | - | - | - | - | - | - | - | - | - | N | - | P | - | A | A | I | - | T | P | A | D | L | D | H | Y | V | T | S | F | - | A | Q | P | G | G | M | R | C |
| 024 UniRef90\_UPI0010F95BB2\_18\_294 | F | I | - | R | H | M | F | H | R | D | K | V | G | L | - | - | - | - | - | - | W | D | - | R | - | T | A | I | - | L | E | A | D | I | A | V | Y | A | A | A | A | - | A | A | P | G | G | L | R | S |
| 025 UniRef90\_A0A0N1GDE2\_28\_316 | F | F | - | G | Y | Q | F | A | K | K | A | A | - | - | - | - | - | - | - | - | - | T | - | P | - | D | A | I | - | P | A | Y | A | V | D | V | Y | V | D | A | I | V | A | D | P | R | G | L | R | A |
| 026 UniRef90\_A0A2E5PJQ7\_5\_284 | Y | L | - | G | W | F | Y | S | N | F | A | W | - | - | - | - | - | - | - | - | - | K | - | T | - | D | A | F | - | E | Q | P | V | I | D | E | Y | L | R | T | Y | - | T | Q | P | G | A | M | R | A |
| 027 UniRef90\_A0A1M7IC97\_44\_328 | W | T | - | A | G | F | I | G | G | F | E | A | - | - | - | - | - | - | - | - | - | V | - | P | - | G | A | V | - | T | P | A | D | I | R | V | F | A | H | Y | L | - | G | D | P | A | H | L | E | A |
| 028 UniRef90\_UPI0016149F5E\_5\_284 | Y | I | - | R | F | I | F | D | R | W | S | Y | - | - | - | - | - | - | - | - | - | R | - | R | - | D | K | V | - | - | - | - | A | V | D | V | Y | A | E | A | Y | - | A | T | P | G | A | L | R | A |
| 029 UniRef90\_UPI00156E1DFF\_8\_289 | F | L | - | T | W | W | F | N | W | L | S | A | - | - | - | - | - | - | - | - | - | V | - | P | - | G | T | F | - | P | P | K | E | V | A | A | V | A | S | S | Y | - | R | G | Y | E | A | L | R | A |
| 030 UniRef90\_A0A3A8HRU0\_15\_300 | Y | F | - | D | H | F | W | T | D | F | A | A | - | - | - | - | - | - | - | - | - | N | - | P | - | Q | A | I | - | G | E | A | E | R | Q | A | Y | T | E | A | Y | - | A | A | P | G | R | L | H | S |
| 031 UniRef90\_A0A0M3UDU6\_18\_309 | F | L | - | T | W | L | F | R | V | K | S | L | - | - | - | - | - | - | - | - | - | Q | - | P | - | W | T | I | - | T | A | E | D | I | A | V | Y | A | R | Q | L | - | A | A | P | G | A | L | R | A |
| 032 UniRef90\_A0A447J1A9\_28\_317 | Y | L | - | N | F | I | F | D | H | W | A | Y | - | - | - | - | - | - | - | - | - | C | - | R | - | D | R | V | - | - | - | - | A | A | Q | T | Y | I | D | A | Y | - | S | S | P | G | G | L | R | A |
| 033 UniRef90\_A0A5C8T429\_14\_295 | F | L | - | S | W | L | F | Q | A | K | A | S | - | - | - | - | - | - | - | - | - | N | - | P | - | A | A | I | - | S | Q | Q | A | M | D | E | Y | V | R | C | Y | - | E | A | P | G | A | W | R | C |
| 034 UniRef90\_A0A0N0TCD7\_1\_288 | Y | F | - | G | H | T | F | T | S | K | T | A | - | - | - | - | - | - | - | - | - | T | - | P | - | G | A | I | - | P | Q | H | A | V | D | V | Y | V | D | S | L | - | R | D | P | A | A | L | R | A |
| 035 UniRef90\_W9ARP2\_16\_295 | Y | I | - | N | R | F | F | D | E | L | A | Y | - | - | - | - | - | - | - | - | - | Q | - | P | - | D | A | I | - | S | I | A | D | L | D | V | Y | A | R | A | Y | - | E | A | P | G | A | M | R | A |
| 036 UniRef90\_A0A109IGY4\_11\_301 | F | F | - | G | Y | Q | F | A | R | K | A | A | - | - | - | - | - | - | - | - | - | T | - | P | - | T | T | I | - | P | G | Y | A | V | D | V | Y | V | D | A | I | V | A | D | P | R | G | L | R | A |
| 037 UniRef90\_K9DQK6\_24\_306 | F | L | - | D | R | L | W | N | Q | F | A | A | - | - | - | - | - | - | - | - | - | D | - | P | - | A | K | V | - | D | E | A | T | R | A | Y | Y | T | R | L | Y | - | A | Q | P | G | A | M | R | A |
| 038 UniRef90\_A0A1Q8IR73\_6\_294 | Y | L | - | E | W | F | F | W | S | K | T | A | - | - | - | - | - | - | - | - | - | N | - | P | - | A | C | Y | - | G | E | E | E | I | A | E | Y | L | R | T | Y | - | C | A | P | G | G | M | R | S |
| 039 UniRef90\_A0A1Y6D2S0\_29\_302 | Y | I | - | A | A | Q | I | K | Q | W | A | H | - | - | - | - | - | - | - | - | - | R | - | K | - | Q | A | I | - | T | P | Q | A | I | D | E | Y | A | K | H | Y | - | A | R | P | G | G | M | T | A |
| 040 UniRef90\_E6WJ64\_19\_301 | Y | L | - | E | W | F | L | R | R | K | T | A | - | - | - | - | - | - | - | - | - | S | - | P | - | M | V | F | - | S | D | A | D | M | A | E | Y | V | R | L | L | - | R | Q | N | G | A | L | R | A |
| 041 UniRef90\_UPI000489A94A\_43\_316 | Y | F | - | E | H | F | W | N | D | F | A | A | - | - | - | - | - | - | - | - | - | T | - | P | D | H | S | L | - | S | E | A | D | R | E | Y | Y | S | A | A | Y | - | A | R | P | G | R | M | K | A |
| 042 UniRef90\_A0A4R6HBQ4\_11\_285 | F | I | - | K | A | M | I | R | R | E | N | V | G | T | - | - | - | - | - | - | F | D | - | P | - | T | S | I | - | T | E | E | D | I | G | H | Y | A | R | A | A | - | A | A | P | G | G | L | R | G |
| 043 UniRef90\_A0A1M7QJX9\_32\_311 | F | L | - | K | G | F | W | P | V | V | G | Y | - | - | - | - | - | - | - | - | - | V | - | K | - | N | P | F | - | T | K | T | E | E | N | E | F | I | R | A | Y | - | S | V | P | G | A | T | T | G |
| 044 UniRef90\_A0A5B8WA72\_22\_300 | F | L | - | T | D | F | W | P | V | V | G | F | - | - | - | - | - | - | - | - | - | K | - | K | - | N | A | F | - | T | T | A | E | R | N | E | F | I | R | A | Y | - | S | V | P | G | A | T | T | G |
| 045 UniRef90\_A0A0G2FGE6\_47\_303 | Y | I | - | K | H | F | Y | D | R | L | C | I | - | - | - | - | - | - | - | - | - | N | - | P | - | S | A | I | - | S | P | A | D | V | D | Y | Y | A | S | M | F | - | E | K | A | G | A | M | R | A |
| 046 UniRef90\_A0A1I6BK09\_10\_296 | F | L | - | S | W | I | F | W | H | L | S | C | - | - | - | - | - | - | - | - | - | N | - | P | - | D | A | V | - | S | P | E | D | F | E | V | Y | A | R | Q | L | - | S | K | P | G | A | F | R | A |
| 047 UniRef90\_A0A4R7C9R7\_33\_310 | Y | I | - | A | A | Q | I | R | A | W | S | H | - | - | - | - | - | - | - | - | - | R | - | K | - | D | A | I | - | G | E | D | A | I | T | V | F | A | A | H | Y | - | A | Q | P | G | R | M | T | A |
| 048 UniRef90\_A0A2M9M9Q1\_18\_305 | Y | Y | - | G | D | Q | F | R | L | K | A | A | - | - | - | - | - | - | - | - | - | R | - | P | - | - | - | L | - | P | E | S | A | V | D | F | Y | V | E | T | L | A | R | D | P | E | A | L | R | A |
| 049 UniRef90\_A0A4V3T343\_11\_295 | L | L | - | D | W | L | F | A | R | A | T | A | - | - | - | - | - | - | - | - | - | D | - | P | - | A | A | V | - | D | A | H | S | R | A | V | Y | A | R | A | Y | - | D | S | P | E | A | I | R | A |
| 050 UniRef90\_UPI001616534D\_11\_303 | L | V | - | D | W | L | F | D | R | Q | A | K | - | - | - | - | - | - | - | - | - | D | - | P | - | A | S | I | - | D | E | R | S | R | R | V | Y | A | H | A | Y | - | S | T | A | D | A | V | R | A |
| 051 UniRef90\_J4PG95\_17\_290 | Y | L | - | S | F | I | F | D | K | W | S | H | - | - | - | - | - | - | - | - | - | R | - | R | - | D | R | V | - | - | - | - | A | A | D | V | Y | I | D | A | Y | - | S | A | P | G | A | L | R | S |
| 052 UniRef90\_A0A0T1T741\_1\_295 | F | F | - | G | W | Q | F | A | T | K | A | A | - | - | - | - | - | - | - | - | - | T | - | P | - | T | A | I | - | P | A | Y | A | V | D | V | Y | V | D | A | I | A | A | D | P | R | A | L | R | A |
| 053 UniRef90\_G0FSK7\_17\_310 | F | L | - | R | W | Q | F | T | H | K | A | A | - | - | - | - | - | - | - | - | - | R | - | P | - | - | - | L | - | A | E | P | A | I | E | H | Y | V | D | S | I | R | R | D | P | R | A | L | H | A |
| 054 UniRef90\_UPI00164AAB2D\_18\_303 | Y | L | - | D | R | F | W | N | E | F | A | G | - | - | - | - | - | - | - | - | - | N | - | P | - | V | K | V | - | D | E | S | T | R | A | H | Y | A | K | L | Y | - | A | A | P | G | A | M | K | A |
| 055 UniRef90\_A0A1I2MT44\_20\_306 | F | F | - | G | H | Q | F | T | A | K | A | A | - | - | - | - | - | - | - | - | - | R | - | P | - | - | - | L | - | P | D | H | A | V | Q | H | Y | V | D | T | L | A | A | D | P | A | A | L | R | A |
| 056 UniRef90\_A0A2P2CCU5\_19\_306 | F | F | - | G | Y | E | F | A | T | Q | G | - | - | - | - | - | - | - | - | - | - | - | - | - | - | G | Q | V | - | P | D | D | A | I | A | Y | Y | V | E | V | L | - | S | R | P | G | V | L | S | G |
| 057 UniRef90\_A0A2V9H5C8\_12\_298 | Y | F | - | E | H | Y | W | N | N | F | A | A | - | - | - | - | - | - | - | - | - | D | - | K | T | R | S | L | - | S | E | A | D | R | K | A | Y | T | A | A | Y | - | A | R | P | G | R | M | R | A |
| 058 UniRef90\_A0A0P4V0Y0\_7\_291 | F | L | - | S | Y | F | Y | K | T | Y | C | V | - | - | - | - | - | - | - | - | - | D | - | S | - | S | A | I | - | E | P | E | A | I | N | E | Y | L | R | T | F | - | A | T | P | A | G | I | R | G |
| 059 UniRef90\_A0A0Q4UQ58\_27\_305 | Y | I | - | R | D | Q | I | R | A | W | S | H | - | - | - | - | - | - | - | - | - | R | - | K | - | E | A | V | - | T | D | E | A | I | A | E | Y | A | R | H | Y | - | A | L | P | G | G | M | K | A |
| 060 UniRef90\_A0A6M4IM28\_20\_309 | Y | L | - | D | R | F | W | N | E | F | S | A | - | - | - | - | - | - | - | - | - | V | - | P | - | A | R | F | - | T | E | T | S | R | A | H | Y | A | A | L | Y | - | A | Q | P | G | A | M | H | A |
| 061 UniRef90\_A0A537J419\_20\_301 | Y | F | - | E | H | Y | W | N | D | F | A | A | - | - | - | - | - | - | - | - | - | D | - | K | T | R | S | L | - | S | E | A | D | R | E | A | Y | T | A | A | Y | - | A | R | P | G | R | M | R | A |
| 062 UniRef90\_A0A1V2PGN8\_6\_275 | Y | L | - | G | Y | F | F | E | R | W | T | Y | - | - | - | - | - | - | - | - | - | Q | - | R | - | Q | G | L | - | D | - | - | A | A | D | E | Y | I | R | A | F | - | S | A | P | G | A | L | R | A |
| 063 UniRef90\_A0A542JC81\_20\_314 | Y | F | - | G | Y | E | F | A | I | Q | G | - | - | - | - | - | - | - | - | - | - | - | - | - | - | G | G | V | - | P | A | E | A | I | E | Y | Y | I | G | L | V | - | S | D | P | V | A | L | T | G |
| 064 UniRef90\_UPI000424F353\_33\_308 | Y | L | - | D | R | F | W | N | E | F | S | A | - | - | - | - | - | - | - | - | - | D | - | P | - | K | N | F | - | D | E | A | S | R | E | H | Y | A | K | L | Y | - | A | K | P | G | A | M | H | S |
| 065 UniRef90\_A0A2V9W2E9\_22\_299 | Y | F | - | D | R | I | W | N | D | F | T | G | - | - | - | - | - | - | - | - | - | D | - | P | - | S | K | P | - | D | E | A | T | R | N | F | F | A | A | T | Y | - | A | Q | P | G | G | M | R | A |
| 066 UniRef90\_A0A2T6L0M5\_16\_299 | L | V | - | E | G | F | I | G | Q | F | L | G | - | - | - | - | - | - | - | - | - | D | - | Q | - | T | A | F | - | A | A | V | D | Y | E | F | Y | A | E | R | L | - | R | R | P | G | R | V | E | A |
| 067 UniRef90\_A0A261TYY2\_43\_315 | F | L | - | G | W | L | F | Q | S | K | S | A | - | - | - | - | - | - | - | - | - | G | - | V | - | Q | W | I | - | E | P | H | A | L | D | E | Y | A | R | C | Y | - | G | G | L | G | G | W | R | A |
| 068 UniRef90\_UPI0009781B11\_10\_287 | F | L | - | A | W | L | F | R | T | K | S | V | - | - | - | - | - | - | - | - | - | D | W | N | - | I | A | F | - | D | D | R | T | I | D | E | Y | A | S | A | Y | - | A | R | P | G | R | W | T | A |
| 069 UniRef90\_A0A2V9TPC1\_35\_307 | Y | F | - | E | Y | F | W | N | D | F | A | A | - | - | - | - | - | - | - | - | - | D | - | K | T | H | S | I | - | P | E | A | D | R | K | A | Y | T | A | A | Y | - | A | R | P | G | R | M | H | A |
| 070 UniRef90\_UPI00145FA88A\_6\_279 | Y | L | - | G | Y | F | F | E | R | W | T | Y | - | - | - | - | - | - | - | - | - | R | - | R | - | D | A | F | - | D | P | A | A | I | T | T | Y | V | N | A | F | - | S | A | P | G | A | L | R | A |
| 071 UniRef90\_UPI0003FADEBE\_21\_306 | Y | L | - | D | R | F | W | N | D | F | A | G | - | - | - | - | - | - | - | - | - | D | - | P | - | R | K | V | - | D | E | G | T | R | A | H | Y | A | R | L | Y | - | A | R | P | G | A | M | R | A |
| 072 UniRef90\_A0A2V8Y3P4\_78\_329 | Y | F | - | D | R | I | W | N | D | F | T | G | - | - | - | - | - | - | - | - | - | D | - | P | - | G | K | P | - | D | D | A | T | R | N | F | F | A | A | T | Y | - | A | Q | P | G | G | M | R | A |
| 073 UniRef90\_A0A285BKA7\_8\_299 | Y | F | - | G | H | Q | F | A | S | K | T | A | - | - | - | - | - | - | - | - | - | T | - | P | - | G | A | I | - | P | Q | D | A | V | D | L | Y | V | D | H | L | - | R | D | P | A | A | R | H | A |
| 074 UniRef90\_A0A0K3AVW3\_44\_319 | M | V | - | D | W | L | C | D | T | L | L | F | - | - | - | - | - | - | - | - | - | D | - | K | - | S | S | I | - | G | E | R | E | R | A | I | Y | A | A | A | W | - | D | R | P | D | A | I | R | A |
| 075 UniRef90\_A0A4D7B7N1\_7\_276 | Y | I | - | E | W | F | F | Q | R | K | T | A | - | - | - | - | - | - | - | - | - | N | - | P | A | A | T | F | - | S | A | Q | D | V | D | E | Y | E | R | V | Y | - | R | M | T | G | N | L | R | G |
| 076 UniRef90\_A0A2U0WF37\_17\_293 | Y | L | - | D | R | F | W | N | E | F | A | G | - | - | - | - | - | - | - | - | - | D | - | P | - | S | K | V | - | D | E | G | T | R | Q | H | Y | A | K | L | Y | - | A | R | P | G | S | M | R | A |
| 077 UniRef90\_UPI00135C2D49\_17\_309 | L | V | - | D | W | L | A | E | Y | L | A | K | - | - | - | - | - | - | - | - | - | D | - | P | - | E | S | L | - | D | E | R | S | R | E | V | Y | A | R | A | Y | - | S | T | A | D | A | I | R | A |
| 078 UniRef90\_UPI001473614E\_45\_322 | T | Y | - | L | G | M | I | F | D | F | S | Y | - | - | - | - | - | - | - | - | - | R | - | R | - | D | A | V | - | - | - | - | E | R | E | P | Y | Y | R | A | Y | - | A | D | P | A | R | R | T | A |
| 079 UniRef90\_J3F862\_22\_299 | Y | L | - | D | R | F | W | N | E | F | A | G | - | - | - | - | - | - | - | - | - | D | - | P | - | K | K | V | - | D | E | A | T | R | Q | H | Y | A | K | L | Y | - | A | R | P | G | A | M | H | A |
| 080 UniRef90\_A0A1A9NC01\_62\_345 | E | Q | - | D | W | I | F | T | Y | F | L | K | - | - | - | - | - | - | - | - | - | N | - | E | - | S | A | I | - | D | A | R | D | R | A | V | Y | E | H | A | Y | - | N | S | I | E | A | I | R | A |
| 081 UniRef90\_UPI00131AC11B\_45\_327 | E | Q | - | A | W | F | F | R | Y | Q | L | K | - | - | - | - | - | - | - | - | - | D | - | E | - | T | A | I | - | D | A | R | D | R | A | V | Y | A | A | A | Y | - | S | S | R | D | A | I | R | A |
| 082 UniRef90\_A0A4R8HC20\_22\_306 | L | L | - | D | W | L | F | E | N | A | A | A | - | - | - | - | - | - | - | - | - | D | - | P | - | G | S | V | - | D | A | H | A | R | A | V | Y | A | R | A | Y | - | E | A | P | E | A | V | R | A |
| 083 UniRef90\_A0A1B2HH26\_4\_280 | Y | L | - | T | W | F | W | R | Q | M | E | - | - | - | - | - | - | - | - | - | - | - | - | R | - | G | G | I | - | T | D | A | D | R | A | D | L | L | R | T | L | - | T | R | P | D | G | M | R | G |
| 084 UniRef90\_A0A517LKB1\_23\_304 | Y | L | - | T | H | F | Y | D | R | L | A | Q | - | - | - | - | - | - | - | - | - | K | - | P | - | S | A | F | - | T | N | E | D | V | Q | T | Y | V | R | Q | Y | - | S | R | P | G | A | L | R | A |
| 085 UniRef90\_A0A0N1F3N8\_17\_298 | Y | L | - | D | R | F | W | N | E | F | S | A | - | - | - | - | - | - | - | - | - | T | - | P | - | S | R | F | - | T | E | A | S | R | K | H | Y | A | A | L | Y | - | A | K | P | G | A | M | H | A |
| 086 UniRef90\_UPI0004CCFD44\_55\_340 | Y | L | - | A | L | F | Y | P | Q | V | S | F | G | G | T | A | F | G | G | T | S | D | - | R | - | S | P | F | - | T | D | A | E | I | N | E | Y | A | R | T | Y | - | R | R | P | E | V | L | S | G |
| 087 UniRef90\_A0A4D4LDP7\_7\_304 | Y | V | - | S | W | F | L | R | A | K | T | A | - | - | - | - | - | - | - | - | - | T | - | G | - | N | V | F | - | D | G | A | E | I | D | L | Y | T | K | A | L | - | I | R | D | G | G | L | H | A |
| 088 UniRef90\_A0A2V4B948\_8\_279 | Y | L | - | A | Y | F | F | E | R | W | T | H | - | - | - | - | - | - | - | - | - | R | - | R | - | D | R | M | - | E | P | D | A | I | D | E | Y | V | R | A | F | - | S | A | P | G | A | L | R | A |
| 089 UniRef90\_A0A2P2FV90\_3\_282 | Y | L | - | G | Y | F | F | E | R | W | T | H | - | - | - | - | - | - | - | - | - | Q | - | R | - | Q | G | L | - | S | R | E | A | V | D | E | Y | V | R | A | F | - | S | A | P | G | A | L | R | A |
| 090 UniRef90\_A0A4Q2J292\_17\_304 | Y | L | - | D | R | F | W | N | E | F | S | A | - | - | - | - | - | - | - | - | - | D | - | P | - | K | N | F | - | D | E | A | S | R | R | H | Y | A | K | L | Y | - | A | L | P | G | A | M | H | S |
| 091 UniRef90\_UPI0012B05B7D\_16\_287 | L | I | - | E | W | F | F | K | N | K | A | L | - | - | - | - | - | - | - | - | - | N | Y | R | - | D | T | F | - | T | E | L | D | L | D | E | Y | T | R | V | Y | - | S | A | L | G | G | M | R | G |
| 092 UniRef90\_UPI00048D4AD4\_4\_301 | L | V | - | D | W | L | F | D | R | Q | S | A | - | - | - | - | - | - | - | - | - | D | - | P | - | R | S | I | - | D | D | R | S | R | E | V | Y | A | R | A | Y | - | S | T | P | D | A | V | R | A |
| 093 UniRef90\_UPI0015F81933\_16\_310 | Y | V | - | G | W | F | L | K | A | K | A | L | - | - | - | - | - | - | - | - | - | S | - | S | - | D | T | F | - | D | D | A | E | I | E | H | Y | A | A | S | V | - | A | A | D | G | G | L | R | A |
| 094 UniRef90\_UPI00160CFF45\_55\_340 | Y | L | - | A | L | F | Y | P | Q | V | S | F | G | G | T | A | F | G | G | T | S | T | - | R | - | S | P | F | - | T | E | A | E | I | N | E | Y | A | R | T | Y | - | S | R | P | S | A | L | S | G |
| 095 UniRef90\_A0A1Q8KTW7\_17\_294 | F | W | - | S | T | W | M | K | N | E | T | Y | - | - | - | - | - | - | - | - | - | N | - | P | - | A | A | I | - | T | D | D | C | V | R | E | W | A | R | C | S | - | A | A | P | G | G | L | R | A |
| 096 UniRef90\_UPI00161A7EC4\_21\_297 | T | Y | - | L | G | F | M | F | G | F | S | Q | - | - | - | - | - | - | - | - | - | H | - | P | - | E | L | I | - | - | - | - | D | K | N | T | Y | Y | R | A | Y | - | A | D | P | A | K | R | S | A |
| 097 UniRef90\_A0A1B4WXA8\_16\_299 | Y | L | - | D | R | F | W | N | E | F | A | G | - | - | - | - | - | - | - | - | - | D | - | P | - | A | K | V | - | D | E | E | I | R | Q | H | Y | A | K | L | Y | - | A | R | P | G | Y | M | H | A |
| 098 UniRef90\_UPI000E46D2C1\_48\_330 | Y | Q | - | N | W | F | W | D | S | L | L | Y | - | - | - | - | - | - | - | - | - | N | - | Q | - | D | A | L | - | S | E | R | D | R | A | V | Y | A | A | A | Y | - | N | T | R | D | G | I | R | G |
| 099 UniRef90\_A0A2N5CFI4\_23\_293 | Y | L | - | D | R | F | W | N | E | F | S | A | - | - | - | - | - | - | - | - | - | D | - | P | - | K | S | F | - | G | E | A | S | R | Q | H | Y | A | K | F | Y | - | A | L | P | G | A | M | H | S |
| 100 UniRef90\_A0A385B2U0\_45\_333 | L | I | - | K | G | F | V | E | Q | Y | L | A | - | - | - | - | - | - | - | - | - | V | - | D | - | G | A | F | - | D | D | R | D | Y | E | F | Y | A | R | Y | L | - | R | E | P | G | R | F | K | A |
| 101 UniRef90\_A0A1Q7W147\_16\_308 | Y | V | - | D | W | F | L | T | A | K | T | L | - | - | - | - | - | - | - | - | - | S | - | P | - | G | T | F | - | D | D | A | E | R | D | H | Y | A | A | A | L | - | A | T | D | G | A | L | R | A |
| 102 UniRef90\_A0A239MTD5\_1\_271 | F | I | - | Q | E | M | I | T | A | Y | L | V | G | - | - | - | - | - | - | - | - | N | - | K | - | S | P | F | - | T | P | S | D | Y | D | Y | Y | A | H | F | L | - | K | E | P | G | R | T | T | A |
| 103 UniRef90\_UPI000561A6EF\_30\_315 | Y | F | - | G | H | Q | F | A | T | K | A | A | - | - | - | - | - | - | - | - | - | R | - | P | - | - | - | L | - | P | E | H | A | V | R | Q | Y | T | D | P | I | A | A | G | P | E | A | L | S | A |
| 104 UniRef90\_A0A4Y8RHA3\_28\_294 | Y | L | - | D | R | F | W | N | E | F | S | A | - | - | - | - | - | - | - | - | - | K | - | P | - | A | N | F | - | D | E | A | S | R | E | H | Y | A | A | L | Y | - | A | L | P | G | A | M | H | A |
| 105 UniRef90\_UPI0004DF415F\_15\_308 | Y | L | - | E | W | F | L | N | V | K | A | L | - | - | - | - | - | - | - | - | - | S | - | P | - | D | T | F | - | D | S | T | E | I | E | H | Y | A | A | A | I | - | A | A | E | G | G | L | R | A |
| 106 UniRef90\_A0A2N3KZL1\_30\_325 | F | L | - | E | H | F | I | K | E | H | A | T | - | - | - | - | - | - | - | - | - | N | - | R | - | A | A | F | - | T | D | E | L | L | D | L | Y | G | A | S | Y | - | A | K | P | H | T | L | H | A |
| 107 UniRef90\_UPI00076E3DF2\_55\_340 | Y | L | - | A | L | F | Y | P | Q | V | S | Y | E | G | S | A | F | G | G | A | S | E | - | Q | - | S | P | F | - | T | D | A | E | I | D | E | Y | A | R | T | Y | - | D | D | P | E | V | L | A | G |
| 108 UniRef90\_UPI00130E1FA7\_66\_348 | Y | Q | - | K | W | F | W | D | S | L | L | F | - | - | - | - | - | - | - | - | - | K | - | Q | - | E | A | L | - | S | E | R | D | R | A | V | Y | A | H | A | Y | - | N | S | K | D | A | I | R | A |
| 109 UniRef90\_A0A1I3FUH2\_46\_328 | Y | Q | - | E | W | F | W | D | N | L | L | Y | - | - | - | - | - | - | - | - | - | D | - | S | - | D | A | I | - | T | T | R | D | R | A | V | Y | A | N | A | Y | - | N | S | A | G | G | I | R | G |
| 110 UniRef90\_UPI000E2836BA\_28\_313 | L | V | - | D | Y | M | F | N | L | S | L | L | - | - | - | - | - | - | - | - | - | N | - | P | - | D | A | I | - | T | P | R | D | R | D | I | Y | A | R | A | Y | - | S | A | P | E | A | I | R | A |
| 111 UniRef90\_A0A653WNW3\_25\_297 | Y | L | - | D | R | F | W | N | E | F | S | A | - | - | - | - | - | - | - | - | - | T | - | P | - | S | R | F | - | S | E | A | A | R | Q | H | Y | A | A | L | Y | - | A | R | P | G | A | M | H | S |
| 112 UniRef90\_A0A5C1I8U5\_39\_321 | A | Q | - | K | Y | I | F | H | Y | L | L | A | - | - | - | - | - | - | - | - | - | D | - | D | - | R | S | I | - | G | P | F | D | R | A | V | Y | A | N | A | Y | - | N | T | R | D | A | I | R | A |
| 113 UniRef90\_UPI00165059AC\_4\_267 | Y | V | - | R | H | M | I | R | Q | W | A | H | - | - | - | - | - | - | - | - | - | I | - | - | - | - | P | L | - | P | D | E | D | V | E | E | Y | V | R | A | F | - | S | A | V | G | A | A | R | A |
| 114 UniRef90\_F8JLL5\_12\_309 | Y | V | - | G | W | F | L | K | A | K | T | L | - | - | - | - | - | - | - | - | - | S | - | S | - | D | T | F | - | D | D | T | E | T | E | H | Y | A | A | S | V | - | A | A | D | G | G | L | R | A |
| 115 UniRef90\_A0A329J6I4\_30\_314 | F | L | - | K | R | F | I | Q | S | H | A | A | - | - | - | - | - | - | - | - | - | S | - | M | - | D | A | F | - | T | P | K | L | I | D | M | Y | A | K | S | Y | - | A | K | P | Q | T | L | N | A |
| 116 UniRef90\_A0A3N1H0W7\_60\_340 | L | V | - | D | W | I | F | D | N | F | L | L | - | - | - | - | - | - | - | - | - | N | - | R | - | D | A | V | - | G | E | L | D | R | A | V | Y | A | H | H | Y | - | D | D | P | D | N | I | R | G |
| 117 UniRef90\_A0A316VRQ3\_1\_263 | Y | L | - | Q | H | L | Y | D | R | L | S | Y | - | - | - | - | - | - | - | - | - | K | - | P | - | Y | F | L | - | T | N | E | E | R | Q | V | Y | Y | E | S | F | - | R | R | S | G | R | M | R | A |
| 118 UniRef90\_A0A4P8X818\_19\_291 | Y | F | - | D | K | F | F | K | N | E | T | Y | - | - | - | - | - | - | - | - | - | D | - | P | - | - | N | I | - | P | N | D | A | M | D | E | Y | I | R | C | Y | - | S | Q | P | G | G | L | R | S |
| 119 UniRef90\_UPI0008405A69\_11\_309 | V | I | - | D | W | L | Y | R | H | M | L | L | - | - | - | - | - | - | - | - | - | E | - | Q | - | D | A | V | - | D | E | Q | A | R | R | I | Y | A | H | A | Y | - | D | S | P | D | A | I | R | A |
| 120 UniRef90\_A0A6I8M538\_3\_275 | Y | L | - | G | Y | F | Y | E | R | W | T | Y | - | - | - | - | - | - | - | - | - | Q | - | R | - | H | S | L | - | D | P | E | A | V | A | E | Y | V | R | A | F | - | S | Q | P | G | A | L | R | A |
| 121 UniRef90\_UPI000B5CCBFE\_21\_300 | L | I | - | D | W | L | Y | T | H | S | L | M | - | - | - | - | - | - | - | - | - | D | - | Q | - | E | S | I | - | T | A | F | D | R | A | V | Y | A | R | A | Y | - | D | T | A | A | G | I | R | A |
| 122 UniRef90\_A0A0M9ZDW3\_1\_266 | V | Q | - | D | W | I | I | E | H | Y | S | G | - | - | - | - | - | - | - | - | - | S | - | P | - | E | S | Y | - | T | E | E | D | R | A | I | Y | A | A | A | Y | - | N | T | S | D | A | I | R | A |
| 123 UniRef90\_UPI000E275A28\_18\_308 | F | F | - | G | Y | Q | F | T | K | I | D | T | - | - | - | - | - | - | - | - | - | - | - | P | - | H | P | L | - | P | E | Y | A | R | N | F | F | I | E | L | L | K | R | D | R | N | A | L | R | A |
| 124 UniRef90\_A0A3L8K0H7\_13\_298 | L | I | - | D | Y | M | F | D | L | S | L | L | - | - | - | - | - | - | - | - | - | N | - | P | - | A | A | I | - | P | P | H | D | R | A | V | Y | A | R | S | Y | - | T | T | P | E | A | I | R | A |
| 125 UniRef90\_A0A6N7ZB66\_18\_297 | F | V | - | Q | A | T | F | G | A | R | T | T | - | - | - | - | - | - | - | - | - | - | - | P | - | G | T | F | - | T | D | E | E | L | E | F | Y | A | R | S | Y | - | T | G | H | D | R | A | R | G |
| 126 UniRef90\_A0A2W7GM08\_52\_337 | E | Q | - | D | W | F | F | H | Y | L | T | K | - | - | - | - | - | - | - | - | - | D | - | E | - | R | Q | I | - | D | E | R | S | R | R | V | Y | A | R | A | Y | - | R | S | T | D | A | L | R | A |
| 127 UniRef90\_A0A258JI33\_23\_300 | Y | L | - | D | R | F | Y | N | E | L | S | A | - | - | - | - | - | - | - | - | - | N | - | P | - | K | A | I | - | D | E | Q | T | R | R | H | Y | A | R | L | Y | - | A | R | P | G | N | M | H | Y |
| 128 UniRef90\_A0A6A6C1N5\_26\_297 | Y | V | - | E | H | F | L | R | K | I | S | F | - | - | - | - | - | - | - | - | - | R | - | R | - | D | V | F | P | A | E | E | D | I | S | F | Y | A | D | E | Y | - | A | K | E | G | V | M | R | A |
| 129 UniRef90\_UPI00160A92C2\_5\_284 | F | L | - | R | H | F | F | T | T | W | C | Y | - | - | - | - | - | - | - | - | - | D | - | P | - | A | A | I | - | N | E | D | A | I | K | E | Y | L | R | A | Y | - | S | A | P | G | A | F | K | G |
| 130 UniRef90\_UPI0009E804D9\_8\_293 | F | F | - | T | T | T | F | T | A | L | S | - | - | - | - | - | - | - | - | - | - | N | - | P | - | G | T | F | - | T | D | K | D | L | A | F | Y | A | R | A | Y | - | Q | G | R | E | R | L | S | G |
| 131 UniRef90\_UPI00049086FA\_57\_348 | F | L | - | R | Y | F | F | Q | H | S | R | I | S | T | R | G | G | A | S | E | A | E | - | P | - | L | P | V | - | S | E | R | A | L | A | E | Y | T | R | V | Y | - | A | R | P | Q | V | L | H | A |
| 132 UniRef90\_A5FF96\_16\_286 | L | Q | - | N | Y | I | F | N | H | L | L | I | - | - | - | - | - | - | - | - | - | D | - | P | - | E | A | I | - | N | E | F | D | R | K | V | Y | A | E | A | Y | - | N | N | K | D | A | I | R | S |
| 133 UniRef90\_UPI0016621A44\_4\_265 | Y | Y | - | G | T | F | L | R | Q | S | A | G | - | - | - | - | - | - | - | - | P | A | - | P | - | L | A | L | - | P | G | D | A | E | Q | V | Y | L | D | A | Y | - | R | R | P | G | T | L | A | A |
| 134 UniRef90\_UPI000B83B944\_2\_264 | F | Q | - | D | W | L | F | Y | Y | L | A | F | G | - | - | - | - | - | - | - | - | G | - | K | - | P | P | M | - | T | Q | E | T | K | D | I | Y | Y | S | A | Y | - | S | S | P | D | A | I | R | A |
| 135 UniRef90\_A0A0B4DHQ5\_15\_286 | W | L | - | R | W | F | F | S | D | W | T | Y | - | - | - | - | - | - | - | - | - | D | - | P | - | A | T | I | - | S | G | E | A | F | D | T | Y | V | R | A | Y | - | R | S | P | G | A | V | R | G |
| 136 UniRef90\_UPI0009B31CF7\_18\_283 | Y | L | - | D | W | F | L | A | S | R | T | P | - | - | - | - | - | - | - | - | - | G | - | L | - | P | G | I | - | D | A | A | A | R | A | V | Y | A | Q | A | Y | - | A | G | R | D | A | L | R | G |
| 137 UniRef90\_UPI001661B99F\_34\_324 | F | Y | - | G | S | V | L | N | G | G | A | P | - | - | - | - | - | - | - | - | - | - | - | G | - | G | A | P | - | S | A | K | S | Q | K | E | Y | I | A | A | Y | - | S | R | P | G | A | T | R | S |
| 138 UniRef90\_UPI00104123D8\_9\_274 | Y | F | - | D | Y | F | Y | R | V | L | S | V | - | - | - | - | - | - | - | - | - | D | - | P | - | E | R | I | - | S | S | R | S | R | A | A | H | A | A | A | Y | - | G | S | A | T | A | L | A | A |
| 139 UniRef90\_A0A1M7Z6I5\_10\_283 | W | L | - | R | H | F | F | S | D | W | C | F | - | - | - | - | - | - | - | - | - | N | - | P | - | H | A | I | - | S | G | A | D | F | D | A | Y | V | A | A | Y | - | R | A | P | G | A | V | R | G |
| 140 UniRef90\_A0A431M1N9\_22\_292 | L | L | - | D | R | F | Y | N | D | L | S | A | - | - | - | - | - | - | - | - | - | D | - | P | - | S | R | I | - | D | E | Q | T | R | N | H | Y | A | A | L | Y | - | A | R | K | G | A | I | H | D |
| 141 UniRef90\_UPI0013D28676\_49\_329 | F | Y | - | L | P | F | W | A | N | - | - | - | - | - | - | - | - | - | - | - | - | S | - | N | - | G | A | I | - | S | A | A | V | Q | Q | R | Y | L | D | A | Y | - | R | R | P | A | S | T | H | A |
| 142 UniRef90\_A0A402BD85\_4\_199 | F | L | - | T | L | F | F | Q | N | S | V | P | - | - | - | - | - | - | - | - | V | Q | V | Q | - | R | S | I | - | T | Q | A | E | I | A | L | Y | A | H | Y | W | - | Q | D | P | T | H | R | H | A |
| 143 UniRef90\_A0A2V5S4I0\_55\_307 | Y | F | - | E | Y | F | W | N | V | L | A | A | - | - | - | - | - | - | - | - | - | D | - | K | T | H | S | I | - | P | E | T | D | R | K | A | Y | T | E | A | Y | - | S | R | P | G | R | M | H | S |
| 144 UniRef90\_A0A328AN40\_21\_303 | G | L | - | S | D | A | F | A | S | S | P | - | - | - | - | - | - | - | - | - | - | R | - | P | - | E | A | F | - | T | P | Q | I | M | A | R | Y | Q | E | A | W | - | R | R | P | G | A | L | T | G |
| 145 UniRef90\_A0A0M9AP20\_25\_244 | M | L | - | E | R | I | L | T | D | G | P | S | - | - | - | - | - | - | - | - | - | R | - | P | - | D | A | F | - | T | E | T | D | V | R | R | Y | K | R | A | L | - | G | Q | P | G | A | R | T | A |
| 146 UniRef90\_A0A1N6RLL9\_1\_202 | Y | F | R | E | G | M | F | N | R | F | A | L | - | - | - | - | - | - | - | - | - | H | - | K | - | D | S | I | - | T | D | S | D | V | N | H | Y | V | N | S | Y | - | G | T | S | E | K | L | R | A |
| 147 UniRef90\_A0A4R3LTR2\_48\_312 | F | L | - | R | H | H | L | A | G | Q | T | R | - | - | - | - | - | - | - | - | - | T | - | P | - | G | A | T | - | E | P | E | A | F | A | E | Y | L | R | C | Y | - | L | D | P | A | A | R | H | A |
| 148 UniRef90\_UPI0002ED95C2\_14\_285 | F | F | - | G | H | F | L | D | T | W | T | H | - | - | - | - | - | - | - | - | - | V | - | A | - | D | A | I | - | P | A | Q | I | R | A | D | Y | L | T | A | A | - | A | H | P | D | A | I | R | A |
| 149 UniRef90\_A0A1Q7MBV0\_16\_212 | R | L | - | R | R | A | L | R | Y | T | P | A | - | - | - | - | - | - | - | - | - | R | - | R | - | G | A | Y | - | D | E | A | V | I | E | A | I | V | A | A | V | - | G | D | - | - | - | R | T | A |
| 150 UniRef90\_A0A1A0KI05\_7\_212 | Y | L | - | R | H | F | W | E | H | W | S | G | - | - | - | - | - | - | - | - | - | P | - | Q | - | L | R | V | - | S | D | D | E | F | E | R | L | A | A | D | Y | - | E | S | P | G | A | F | S | A |

  
  

|  |  |  |  |  |  |  |  |  |  |  |  |  |  |  |  |  |  |  |  |  |  |  |  |  |  |  |  |  |  |  |  |  |  |  |  |  |  |  |  |  |  |  |  |  |  |  |  |  |  |  |
| --- | --- | --- | --- | --- | --- | --- | --- | --- | --- | --- | --- | --- | --- | --- | --- | --- | --- | --- | --- | --- | --- | --- | --- | --- | --- | --- | --- | --- | --- | --- | --- | --- | --- | --- | --- | --- | --- | --- | --- | --- | --- | --- | --- | --- | --- | --- | --- | --- | --- | --- |
| **001 Input\_protein\_seq** | G | F | E | - | L | Y | R | A | F | - | - | P | Q | D | E | T | R | F | K | A | - | F | - | - | M | - | K | H | - | - | K | L | P | M | P | V | L | A | L | A | G | D | K | - | - | - | - | S | - | - |
| 002 UniRef90\_UPI00158A2D6D\_3\_306 | G | F | E | - | L | Y | R | A | F | - | - | P | Q | D | E | T | Q | F | K | A | - | F | - | - | M | - | K | H | - | - | Q | L | P | M | P | V | L | A | L | A | G | D | K | - | - | - | - | S | - | - |
| 003 UniRef90\_A0A1Z4J856\_27\_318 | G | F | E | - | Y | Y | R | A | I | - | - | P | Q | V | A | K | Q | T | K | E | - | N | - | - | M | - | K | T | - | - | K | L | R | I | P | V | L | A | L | A | G | E | H | - | - | - | - | S | L | G |
| 004 UniRef90\_G7LVZ3\_7\_288 | S | F | E | - | H | Y | R | A | F | - | - | P | V | D | A | R | N | N | Q | E | - | H | - | - | A | - | R | T | - | - | K | L | P | M | P | V | L | G | I | G | G | E | Y | - | - | - | - | C | - | - |
| 005 UniRef90\_A0A2I8F4B9\_53\_324 | G | F | E | - | Y | Y | R | A | F | - | - | P | Q | D | V | K | A | N | Q | A | - | F | - | - | S | - | A | T | - | - | K | L | T | M | P | V | L | G | I | G | G | A | G | - | - | - | - | S | - | - |
| 006 UniRef90\_A0A327RPK7\_10\_284 | G | F | E | - | Y | Y | R | C | L | - | - | L | E | D | G | K | H | N | Q | K | - | Y | - | - | K | - | E | Q | - | - | K | L | D | I | P | V | L | A | Y | G | G | E | T | - | - | - | - | S | - | - |
| 007 UniRef90\_A0A6L3SWG4\_20\_294 | G | F | E | - | L | Y | R | A | F | - | - | P | A | D | A | E | R | N | R | A | - | D | L | E | R | - | E | G | - | - | K | L | P | M | P | V | L | A | V | A | G | A | H | - | - | - | - | S | T | - |
| 008 UniRef90\_A0A401ZLI5\_4\_287 | G | F | E | - | Y | Y | R | S | F | - | - | S | S | N | A | Q | L | F | K | E | - | Y | - | - | S | - | K | E | - | - | K | L | R | I | P | T | L | A | L | G | G | E | Y | - | - | - | - | S | - | - |
| 009 UniRef90\_A0A4V2U6R1\_12\_297 | G | L | E | - | L | F | R | A | F | - | - | E | Q | D | A | E | D | N | R | R | - | I | L | T | E | - | K | G | - | - | K | L | K | M | P | V | L | G | L | G | G | T | A | - | - | - | - | S | F | - |
| 010 UniRef90\_A0A5A5T922\_6\_282 | G | F | E | - | L | Y | R | A | L | - | - | P | T | D | L | K | N | N | A | M | - | S | - | - | A | - | Q | N | - | - | P | L | R | I | P | V | F | A | L | G | G | D | K | - | - | - | - | S | - | - |
| 011 UniRef90\_A0A2V6UIN9\_3\_273 | G | F | E | - | A | F | R | A | F | - | - | E | Q | D | A | T | D | F | A | A | - | F | - | - | S | - | K | T | - | - | K | L | N | M | P | M | L | V | L | T | G | E | K | - | - | - | - | A | - | - |
| 012 UniRef90\_UPI001669B3C5\_41\_314 | G | F | E | - | Y | F | H | N | F | - | - | E | K | D | A | Q | D | F | Q | Q | - | L | - | - | G | - | Q | T | - | - | K | L | T | M | P | V | L | V | I | A | G | E | K | - | - | - | - | S | - | - |
| 013 UniRef90\_A0A4R2Z7A8\_3\_305 | G | L | A | - | Y | Y | R | S | V | - | - | T | V | S | A | E | Q | N | R | K | - | L | - | N | S | - | Q | G | - | - | K | L | Q | M | P | L | L | A | V | S | A | D | Q | - | - | - | - | G | - | - |
| 014 UniRef90\_A0A5C5SVV1\_11\_281 | G | L | E | - | W | Y | R | A | F | - | - | R | K | D | H | A | D | A | L | A | - | W | - | - | K | - | R | E | - | - | R | L | T | M | P | V | L | G | L | G | G | D | G | - | - | - | - | R | - | - |
| 015 UniRef90\_L9WLS3\_16\_284 | G | F | E | - | Y | Y | R | A | Y | - | - | D | D | D | A | E | N | N | A | V | - | H | - | - | A | - | E | T | - | - | P | L | E | M | P | V | L | A | L | G | G | A | A | - | - | - | - | S | - | - |
| 016 UniRef90\_UPI00131EC7B5\_30\_311 | G | L | A | - | Y | Y | R | A | A | - | - | A | L | S | A | S | Q | N | R | E | - | R | - | V | K | - | K | G | - | - | K | L | A | M | P | I | L | A | I | S | A | E | Q | - | - | - | - | G | - | - |
| 017 UniRef90\_C7QAM5\_50\_332 | G | L | Q | - | W | F | R | T | L | - | - | P | Q | D | M | T | N | D | A | V | - | Y | - | - | Q | - | K | T | - | - | K | L | T | M | P | V | L | A | I | G | A | S | G | - | - | - | - | S | - | - |
| 018 UniRef90\_A0A1Q3SXP6\_11\_288 | G | F | E | - | V | F | R | A | F | - | - | E | R | D | A | V | D | F | E | K | - | F | - | - | S | - | S | K | - | - | K | L | T | M | P | M | L | V | L | T | G | E | K | - | - | - | - | A | - | - |
| 019 UniRef90\_UPI00149248BF\_39\_315 | G | F | E | - | Y | Y | R | A | I | - | - | P | L | T | A | Q | Q | N | E | K | - | L | - | - | R | - | K | R | - | - | K | L | T | M | P | V | L | A | I | G | G | I | R | - | - | - | - | G | - | - |
| 020 UniRef90\_UPI000361D127\_26\_313 | S | F | E | - | Y | Y | R | A | I | - | - | D | E | T | M | T | Q | T | V | L | - | R | - | - | K | - | Q | N | - | - | K | L | T | I | P | V | L | A | V | A | G | D | A | - | - | - | - | L | - | - |
| 021 UniRef90\_A0A0M4FVH2\_24\_312 | G | L | A | - | Y | Y | R | A | A | - | - | S | L | S | A | Q | Q | N | R | E | - | L | - | C | A | - | M | G | - | - | K | L | R | T | P | V | L | A | L | G | A | D | Q | - | - | - | - | G | - | - |
| 022 UniRef90\_A0A1Q4ZL08\_9\_290 | S | F | E | - | Y | F | R | A | L | - | - | P | Q | D | I | A | D | N | A | E | - | Y | - | - | A | - | K | T | - | - | K | L | A | M | P | V | L | A | L | G | A | R | A | - | - | - | - | S | - | - |
| 023 UniRef90\_S3CY91\_25\_307 | G | F | E | - | L | Y | R | A | F | - | - | P | R | D | A | R | D | N | R | R | - | M | L | E | E | - | R | G | - | - | R | S | K | V | P | A | C | S | L | S | G | E | G | - | - | - | - | S | L | - |
| 024 UniRef90\_UPI0010F95BB2\_18\_294 | M | F | A | - | Y | Y | R | T | L | L | - | S | H | D | L | Q | D | N | L | A | - | L | - | - | G | - | R | R | - | - | K | L | S | L | P | V | L | A | I | G | A | E | H | - | - | - | - | G | - | - |
| 025 UniRef90\_A0A0N1GDE2\_28\_316 | S | F | A | - | Y | Y | R | A | L | - | - | D | E | T | I | A | Q | N | E | Q | - | R | - | - | K | - | K | T | - | - | R | L | T | L | P | V | L | A | I | G | G | A | R | - | - | - | - | Y | - | - |
| 026 UniRef90\_A0A2E5PJQ7\_5\_284 | G | F | A | - | Y | Y | R | A | L | - | - | P | Q | D | V | A | A | N | R | A | - | L | - | - | L | - | E | T | G | F | R | L | P | M | P | V | L | A | M | G | G | A | M | W | E | A | R | G | - | - |
| 027 UniRef90\_A0A1M7IC97\_44\_328 | S | L | A | - | W | F | R | T | F | - | - | P | Q | D | I | R | D | D | E | R | - | F | - | - | Q | - | K | T | - | - | P | L | R | M | P | V | L | A | I | G | A | S | G | - | - | - | - | S | - | - |
| 028 UniRef90\_UPI0016149F5E\_5\_284 | G | F | A | - | Y | Y | R | A | I | - | - | P | E | T | I | R | Q | N | L | E | - | R | - | - | A | - | K | R | - | - | S | L | A | M | P | V | L | A | I | G | A | D | H | - | - | - | - | A | - | - |
| 029 UniRef90\_UPI00156E1DFF\_8\_289 | G | F | A | - | H | Y | R | T | L | - | - | L | D | D | G | R | T | N | R | T | - | W | - | - | R | - | E | T | G | G | I | L | P | M | P | V | L | A | V | G | G | E | H | - | - | - | - | S | - | - |
| 030 UniRef90\_A0A3A8HRU0\_15\_300 | T | W | G | - | Y | F | Q | A | L | - | - | D | Q | D | K | K | D | F | R | E | - | L | - | - | A | - | R | N | - | - | K | L | P | M | P | V | M | V | I | G | G | D | K | - | - | - | - | S | - | - |
| 031 UniRef90\_A0A0M3UDU6\_18\_309 | A | T | R | - | Y | Y | Q | C | A | L | S | P | E | G | V | A | A | N | R | L | - | R | - | - | A | - | E | K | - | - | P | L | D | I | P | V | L | A | L | G | A | D | R | - | - | - | - | G | - | - |
| 032 UniRef90\_A0A447J1A9\_28\_317 | G | F | A | - | Y | Y | R | A | I | - | - | P | Q | T | I | A | Q | N | K | R | - | R | - | - | A | - | E | K | - | - | K | L | T | M | P | V | L | A | I | G | A | D | H | - | - | - | - | A | - | - |
| 033 UniRef90\_A0A5C8T429\_14\_295 | A | N | S | - | Y | Y | R | A | Y | - | - | F | D | D | M | A | Q | N | R | E | - | H | - | - | A | - | R | R | - | - | K | I | R | T | P | I | L | T | V | G | G | D | V | - | - | - | - | G | - | - |
| 034 UniRef90\_A0A0N0TCD7\_1\_288 | S | F | A | - | Y | Y | R | S | L | - | - | D | E | S | A | R | Q | A | Q | R | W | R | - | - | D | - | E | G | - | - | P | L | S | V | P | V | L | A | I | G | G | E | H | - | - | - | - | S | - | - |
| 035 UniRef90\_W9ARP2\_16\_295 | M | C | E | - | V | Y | R | E | L | - | - | D | R | D | A | E | D | N | R | A | - | A | L | R | E | - | Q | G | - | - | K | L | T | V | P | V | L | A | S | G | G | A | T | - | - | - | - | N | P | - |
| 036 UniRef90\_A0A109IGY4\_11\_301 | S | F | G | - | Y | Y | R | A | L | - | - | D | E | T | I | A | Q | N | E | R | - | R | - | - | A | - | K | T | - | - | R | L | T | L | P | V | L | A | I | G | G | A | L | - | - | - | - | Y | - | - |
| 037 UniRef90\_K9DQK6\_24\_306 | S | F | A | - | Q | F | N | T | I | A | - | T | H | D | V | A | D | N | R | A | - | A | - | - | S | - | K | V | - | - | K | L | T | M | P | V | L | A | V | G | G | E | K | - | - | - | - | S | - | - |
| 038 UniRef90\_A0A1Q8IR73\_6\_294 | G | L | A | - | F | Y | R | A | A | - | - | A | L | S | V | E | Q | N | R | A | - | L | - | S | R | - | D | R | - | - | K | L | A | M | P | V | L | G | L | S | A | D | Q | - | - | - | - | G | - | - |
| 039 UniRef90\_A0A1Y6D2S0\_29\_302 | G | F | N | - | Y | Y | R | A | L | - | - | L | D | D | A | Q | F | V | A | A | - | Y | - | - | A | - | D | Q | - | - | K | F | A | M | P | V | L | A | V | A | G | R | Y | - | - | - | - | G | - | - |
| 040 UniRef90\_E6WJ64\_19\_301 | G | L | A | - | P | Y | R | V | V | - | - | T | Q | S | A | A | Q | N | R | A | - | L | - | R | E | - | Q | G | - | - | K | L | T | L | P | L | L | A | I | S | A | D | Q | - | - | - | - | G | - | - |
| 041 UniRef90\_UPI000489A94A\_43\_316 | G | F | E | - | Y | F | H | A | F | - | - | G | Q | D | A | T | D | F | A | A | - | F | - | - | A | - | Q | T | - | - | P | L | T | M | P | M | L | V | V | A | G | E | K | - | - | - | - | A | - | - |
| 042 UniRef90\_A0A4R6HBQ4\_11\_285 | M | F | A | - | H | Y | R | A | L | I | - | P | Q | D | R | D | D | N | L | R | - | L | - | - | G | - | A | S | - | - | P | L | L | C | P | V | L | A | V | G | G | D | H | - | - | - | - | G | - | - |
| 043 UniRef90\_A0A1M7QJX9\_32\_311 | A | F | H | - | W | F | G | E | F | - | - | N | H | D | A | K | D | N | L | E | - | L | - | - | M | - | K | T | - | - | K | L | R | M | P | L | L | S | L | G | A | D | H | - | - | - | - | S | - | - |
| 044 UniRef90\_A0A5B8WA72\_22\_300 | A | F | H | - | W | F | G | Y | F | - | - | N | Q | D | A | K | D | N | V | E | - | L | - | - | A | - | R | H | - | - | K | L | T | M | P | V | L | A | M | G | S | E | H | - | - | - | - | F | - | - |
| 045 UniRef90\_A0A0G2FGE6\_47\_303 | G | F | D | - | V | Y | R | A | F | - | - | H | K | D | A | E | E | N | R | D | - | W | V | A | N | - | N | G | - | - | K | C | T | V | P | C | M | S | L | N | G | E | G | - | - | - | - | S | F | - |
| 046 UniRef90\_A0A1I6BK09\_10\_296 | G | I | N | - | L | Y | A | A | V | - | - | W | T | D | A | E | H | N | R | A | - | S | - | - | A | - | Q | R | - | - | K | L | P | M | P | V | L | A | V | G | G | A | C | - | - | - | - | S | - | - |
| 047 UniRef90\_A0A4R7C9R7\_33\_310 | G | F | N | - | Y | Y | R | A | L | - | - | R | E | D | A | P | L | A | A | E | - | L | - | - | R | - | G | R | - | - | R | L | P | M | P | V | M | A | V | A | G | R | Q | - | - | - | - | S | - | - |
| 048 UniRef90\_A0A2M9M9Q1\_18\_305 | S | F | D | - | Y | Y | R | A | T | - | - | D | E | N | I | A | Q | N | G | R | - | R | - | - | G | - | R | R | - | - | P | L | P | M | P | V | L | G | V | A | G | A | R | - | - | - | - | G | - | - |
| 049 UniRef90\_A0A4V3T343\_11\_295 | S | N | D | - | W | Y | R | A | F | - | - | P | Q | D | I | E | D | L | A | G | - | Y | - | - | - | - | - | P | - | - | P | V | T | A | P | L | L | A | L | G | G | E | F | - | - | - | - | S | - | - |
| 050 UniRef90\_UPI001616534D\_11\_303 | G | N | G | - | W | Y | Q | T | F | - | - | T | R | D | I | D | D | E | Q | T | - | Y | - | - | - | - | - | G | - | - | R | V | S | A | P | V | L | A | L | G | G | D | E | - | - | - | - | S | - | - |
| 051 UniRef90\_J4PG95\_17\_290 | G | F | A | - | Y | Y | R | A | I | - | - | P | E | T | I | R | Q | N | Q | E | - | R | - | - | S | - | K | T | - | - | R | L | R | M | P | T | L | A | I | G | T | E | H | - | - | - | - | A | - | - |
| 052 UniRef90\_A0A0T1T741\_1\_295 | S | F | A | - | Y | Y | R | A | L | - | - | D | E | T | I | A | Q | N | E | Q | - | R | - | - | G | - | R | T | - | - | R | L | T | L | P | V | L | A | A | G | G | A | L | - | - | - | - | W | - | - |
| 053 UniRef90\_G0FSK7\_17\_310 | S | F | G | - | C | Y | R | A | I | - | - | G | D | T | V | A | Q | N | A | E | - | R | - | - | V | - | K | N | - | - | R | L | D | L | P | I | L | T | I | A | G | E | R | - | - | - | - | S | - | - |
| 054 UniRef90\_UPI00164AAB2D\_18\_303 | A | F | N | - | Q | F | L | S | I | - | - | R | K | D | V | D | D | N | A | E | - | S | - | - | M | - | K | T | - | - | K | L | N | M | P | V | L | A | I | G | G | E | K | - | - | - | - | S | - | - |
| 055 UniRef90\_A0A1I2MT44\_20\_306 | S | F | A | - | F | Y | R | S | L | - | - | D | E | T | I | A | Q | N | E | Q | - | R | - | - | A | - | A | R | - | - | R | L | T | L | P | V | L | A | V | G | G | A | Q | - | - | - | - | N | - | - |
| 056 UniRef90\_A0A2P2CCU5\_19\_306 | S | F | G | - | F | Y | R | S | W | - | - | D | E | T | M | A | Q | N | G | E | - | R | - | - | A | - | T | R | - | - | M | L | T | M | P | V | L | A | I | G | G | E | T | - | - | - | - | S | - | - |
| 057 UniRef90\_A0A2V9H5C8\_12\_298 | G | W | A | - | Y | F | V | S | F | - | - | P | Q | A | A | K | D | F | V | Q | - | L | - | - | A | - | K | T | - | - | K | L | T | I | P | V | L | A | I | G | G | E | K | - | - | - | - | S | - | - |
| 058 UniRef90\_A0A0P4V0Y0\_7\_291 | A | L | G | - | V | Y | R | A | I | - | - | F | E | S | V | Q | Q | T | E | A | - | I | - | - | A | - | N | H | - | - | P | I | Q | T | P | V | L | A | L | G | G | E | K | - | - | - | - | S | - | - |
| 059 UniRef90\_A0A0Q4UQ58\_27\_305 | G | F | N | - | Y | Y | R | A | L | - | - | K | D | D | A | A | L | A | A | S | - | F | - | - | A | - | G | R | - | - | R | L | A | M | P | V | L | A | I | T | G | R | Y | - | - | - | - | G | - | - |
| 060 UniRef90\_A0A6M4IM28\_20\_309 | G | F | E | - | Q | F | R | A | F | - | - | D | Q | D | A | I | D | N | K | A | - | F | - | L | Q | - | R | G | - | - | K | L | T | M | P | V | L | A | V | G | G | E | K | - | - | - | - | S | - | - |
| 061 UniRef90\_A0A537J419\_20\_301 | G | W | A | - | Y | F | I | A | F | - | - | P | Q | T | A | K | D | F | A | Q | - | L | - | - | S | - | K | T | - | - | A | L | T | I | P | V | L | V | I | G | G | E | K | - | - | - | - | A | - | - |
| 062 UniRef90\_A0A1V2PGN8\_6\_275 | G | F | D | - | D | Y | R | A | S | F | - | P | A | D | D | E | H | D | E | A | - | D | - | - | A | - | G | R | - | - | R | L | A | M | P | V | L | A | M | W | G | E | T | - | - | - | - | G | L | - |
| 063 UniRef90\_A0A542JC81\_20\_314 | S | L | G | - | F | Y | R | A | W | - | - | E | T | T | M | G | Q | N | A | E | - | R | - | - | A | - | K | S | - | - | K | L | P | M | P | V | L | A | V | G | G | E | R | - | - | - | - | S | - | - |
| 064 UniRef90\_UPI000424F353\_33\_308 | G | F | E | - | Q | F | K | A | F | - | - | D | Q | D | A | V | D | N | K | A | - | F | - | L | A | - | K | G | - | - | K | L | P | M | P | V | L | A | V | G | G | E | K | - | - | - | - | S | - | - |
| 065 UniRef90\_A0A2V9W2E9\_22\_299 | G | F | A | - | Q | F | A | A | F | - | - | S | Q | D | A | K | D | N | E | V | - | F | - | - | E | - | R | V | - | - | K | L | T | M | P | V | L | A | V | G | G | E | K | - | - | - | - | S | - | - |
| 066 UniRef90\_A0A2T6L0M5\_16\_299 | W | V | K | - | M | Y | R | S | L | - | - | A | V | N | I | Q | Q | N | K | Q | - | L | - | Q | A | - | A | G | - | - | P | I | G | M | P | I | L | A | V | G | G | E | K | - | - | - | - | S | - | - |
| 067 UniRef90\_A0A261TYY2\_43\_315 | G | A | S | - | Y | Y | R | A | L | - | - | F | Q | D | M | E | Q | N | R | A | - | H | - | - | A | - | R | A | - | - | R | L | T | M | P | V | L | A | M | G | G | E | A | - | - | - | - | A | - | - |
| 068 UniRef90\_UPI0009781B11\_10\_287 | G | L | A | - | Y | Y | R | S | I | - | - | F | E | S | I | A | Q | N | K | V | - | S | - | - | S | - | R | I | - | - | P | L | P | M | P | V | L | A | I | G | G | D | F | - | - | - | - | G | - | - |
| 069 UniRef90\_A0A2V9TPC1\_35\_307 | G | W | A | - | Y | F | V | S | F | - | - | Q | Q | A | A | K | D | F | A | Q | - | L | - | - | S | - | Q | T | - | - | K | L | H | M | P | V | L | T | I | G | G | E | K | - | - | - | - | S | - | - |
| 070 UniRef90\_UPI00145FA88A\_6\_279 | G | F | D | - | D | Y | R | A | S | F | - | P | T | D | S | E | H | D | D | A | - | D | A | D | S | - | G | R | - | - | R | L | V | M | P | V | L | A | L | W | G | G | N | - | - | - | - | G | L | - |
| 071 UniRef90\_UPI0003FADEBE\_21\_306 | A | F | V | - | Q | F | Q | S | I | - | - | R | K | D | A | Q | D | N | A | E | - | L | - | - | V | - | R | T | - | - | R | L | T | M | P | V | L | A | I | G | G | E | K | - | - | - | - | S | - | - |
| 072 UniRef90\_A0A2V8Y3P4\_78\_329 | G | F | A | - | Q | F | A | A | F | - | - | S | Q | D | A | K | D | N | K | V | - | F | - | - | E | - | Q | T | - | - | K | L | T | M | P | V | M | A | V | G | A | E | K | - | - | - | - | S | - | - |
| 073 UniRef90\_A0A285BKA7\_8\_299 | S | F | E | - | Y | Y | R | T | L | - | - | D | T | T | A | E | H | I | Q | R | W | R | - | - | D | - | Q | G | - | - | P | L | A | I | P | V | L | A | I | G | G | Q | Y | - | - | - | - | S | - | - |
| 074 UniRef90\_A0A0K3AVW3\_44\_319 | G | N | A | - | W | Y | Q | G | F | - | - | Y | Q | D | I | E | D | E | K | T | - | Y | - | - | - | - | - | G | - | - | K | I | T | A | P | M | L | G | L | A | A | S | - | - | - | - | - | - | - | - |
| 075 UniRef90\_A0A4D7B7N1\_7\_276 | A | L | G | - | Y | Y | R | A | V | - | - | F | E | D | I | E | Q | N | K | H | - | L | - | - | A | - | N | I | - | - | R | L | K | T | P | I | L | A | L | G | G | D | V | - | - | - | - | G | - | - |
| 076 UniRef90\_A0A2U0WF37\_17\_293 | A | F | A | - | Q | F | R | S | I | - | - | R | Q | D | A | V | D | N | E | A | - | S | - | - | M | - | K | T | - | - | K | L | T | M | P | V | L | A | I | G | G | E | K | - | - | - | - | S | - | - |
| 077 UniRef90\_UPI00135C2D49\_17\_309 | G | N | S | - | W | Y | R | A | F | - | - | T | T | D | I | A | D | E | R | T | - | Y | - | - | - | - | - | G | - | - | P | L | R | A | P | L | L | A | L | G | G | D | L | - | - | - | - | S | - | - |
| 078 UniRef90\_UPI001473614E\_45\_322 | G | Y | E | - | Y | Y | R | A | F | - | - | A | A | D | A | E | N | N | T | A | - | N | - | - | A | - | A | K | - | - | R | L | P | M | P | V | L | G | I | G | G | Q | Y | - | - | - | - | S | - | - |
| 079 UniRef90\_J3F862\_22\_299 | A | F | A | - | Q | F | R | A | I | - | - | R | Q | D | A | V | D | N | E | A | - | S | - | - | M | - | K | T | - | - | K | L | K | M | P | V | L | A | V | G | G | E | K | - | - | - | - | S | - | - |
| 080 UniRef90\_A0A1A9NC01\_62\_345 | S | D | G | - | W | Y | Q | A | L | - | - | P | Q | D | V | V | D | Y | R | N | - | Y | - | - | - | - | - | G | - | - | K | L | D | M | P | V | L | A | M | G | G | P | - | - | - | - | - | - | - | - |
| 081 UniRef90\_UPI00131AC11B\_45\_327 | G | N | G | - | W | Y | Q | A | F | - | - | S | R | D | I | A | D | S | R | T | - | Y | - | - | - | - | - | Q | - | - | P | L | A | M | P | V | L | G | L | G | G | T | - | - | - | - | - | - | - | - |
| 082 UniRef90\_A0A4R8HC20\_22\_306 | A | N | D | - | W | Y | R | A | F | - | - | T | R | D | I | A | D | L | A | G | - | Y | - | - | - | - | - | A | - | - | P | V | A | A | P | I | L | A | L | G | G | E | Y | - | - | - | - | G | - | - |
| 083 UniRef90\_A0A1B2HH26\_4\_280 | G | F | E | - | H | Y | A | S | V | - | - | A | Q | D | A | V | N | A | R | A | - | - | - | - | - | - | G | R | - | - | K | V | E | V | P | V | L | V | L | H | G | E | H | - | - | - | - | G | L | - |
| 084 UniRef90\_A0A517LKB1\_23\_304 | G | F | L | - | S | Y | K | A | F | - | - | E | T | D | G | E | D | N | Q | T | - | W | R | E | K | - | N | G | - | - | K | V | K | V | R | N | M | V | L | S | G | E | G | - | - | - | - | S | F | - |
| 085 UniRef90\_A0A0N1F3N8\_17\_298 | G | F | A | - | Q | F | A | A | F | - | - | D | Q | D | A | T | D | N | R | A | - | F | - | V | A | - | A | G | - | - | K | L | T | M | P | V | L | A | L | G | G | E | K | - | - | - | - | S | - | - |
| 086 UniRef90\_UPI0004CCFD44\_55\_340 | G | F | E | - | L | Y | R | A | L | - | - | D | K | D | V | R | D | T | T | A | - | - | - | - | - | - | A | A | - | - | P | I | R | V | P | T | L | L | M | T | A | Q | G | - | - | - | - | Q | - | - |
| 087 UniRef90\_A0A4D4LDP7\_7\_304 | A | M | E | - | Y | Y | R | A | V | - | - | P | E | S | A | A | R | N | R | R | - | L | - | L | A | - | D | R | - | - | E | L | T | M | P | V | L | A | I | D | S | E | H | - | - | - | - | G | - | - |
| 088 UniRef90\_A0A2V4B948\_8\_279 | G | F | D | - | D | Y | R | A | S | F | - | P | A | D | A | E | H | D | D | A | - | D | A | T | T | - | G | R | - | - | K | L | H | A | P | V | L | V | L | W | G | A | Q | - | - | - | - | G | L | - |
| 089 UniRef90\_A0A2P2FV90\_3\_282 | G | F | D | - | D | Y | R | A | S | F | - | P | H | D | A | E | A | D | D | A | - | D | F | D | A | - | G | R | - | - | R | L | E | L | P | V | L | A | L | W | G | A | S | - | - | - | - | G | L | - |
| 090 UniRef90\_A0A4Q2J292\_17\_304 | G | F | E | - | Q | F | H | A | F | - | - | D | Q | D | A | I | D | N | K | A | - | F | - | L | A | - | K | G | - | - | L | L | Q | M | P | V | L | A | I | G | G | E | K | - | - | - | - | S | - | - |
| 091 UniRef90\_UPI0012B05B7D\_16\_287 | M | L | G | - | Y | Y | R | S | V | - | - | L | E | D | M | E | Q | N | R | V | - | F | - | - | G | - | Q | Q | - | - | L | L | K | I | P | V | L | A | L | G | G | D | K | - | - | - | - | G | - | - |
| 092 UniRef90\_UPI00048D4AD4\_4\_301 | G | N | A | - | W | Y | Q | A | F | - | - | N | A | D | I | A | D | S | L | T | - | Y | - | - | - | - | - | R | - | - | P | V | R | A | P | I | L | A | L | G | G | D | R | - | - | - | - | A | - | - |
| 093 UniRef90\_UPI0015F81933\_16\_310 | S | L | A | - | Y | Y | R | D | A | - | - | A | E | S | A | Q | K | N | H | E | - | A | - | L | E | - | R | Q | - | - | R | L | T | V | P | I | L | G | I | S | S | S | H | - | - | - | - | G | - | - |
| 094 UniRef90\_UPI00160CFF45\_55\_340 | G | F | E | - | L | Y | R | S | L | - | - | D | K | D | V | S | D | A | K | A | - | - | - | - | - | - | A | K | - | - | P | V | R | V | P | A | L | V | M | S | A | Q | G | - | - | - | - | Q | - | - |
| 095 UniRef90\_A0A1Q8KTW7\_17\_294 | I | F | E | - | V | Y | R | A | T | - | - | F | T | N | V | E | L | N | T | E | - | W | - | - | A | - | R | T | - | - | P | L | P | M | P | V | L | T | I | G | S | S | H | - | - | - | - | F | - | - |
| 096 UniRef90\_UPI00161A7EC4\_21\_297 | G | Y | E | - | Y | Y | R | A | F | - | - | A | E | D | G | T | N | N | T | A | - | N | - | - | A | - | S | R | - | - | R | L | T | Q | P | V | L | A | L | G | G | A | A | - | - | - | - | S | - | - |
| 097 UniRef90\_A0A1B4WXA8\_16\_299 | A | F | A | - | Q | F | R | S | I | - | - | R | Q | D | E | V | D | N | K | A | - | S | - | - | M | - | A | T | - | - | R | L | K | M | P | V | L | A | V | G | G | E | K | - | - | - | - | S | - | - |
| 098 UniRef90\_UPI000E46D2C1\_48\_330 | G | N | G | - | W | Y | Q | A | Y | - | - | P | T | D | L | D | D | Q | L | T | - | Y | - | - | - | - | P | D | - | - | K | L | E | V | P | S | L | G | I | G | G | L | - | - | - | - | - | - | - | - |
| 099 UniRef90\_A0A2N5CFI4\_23\_293 | G | F | E | - | Q | F | H | A | F | - | - | D | Q | D | V | I | D | N | K | A | - | N | - | L | A | - | K | G | - | - | A | L | P | M | P | V | L | A | I | G | G | E | K | - | - | - | - | S | - | - |
| 100 UniRef90\_A0A385B2U0\_45\_333 | W | M | N | - | M | Y | Q | A | L | - | - | H | T | D | I | A | Q | N | E | K | - | F | - | R | E | - | A | G | - | - | L | L | Q | M | P | I | L | A | V | G | G | E | E | - | - | - | - | A | - | - |
| 101 UniRef90\_A0A1Q7W147\_16\_308 | S | L | A | - | Y | Y | R | D | A | - | - | A | E | S | A | R | K | N | H | Q | - | A | - | L | R | - | R | R | - | - | H | L | T | L | P | V | L | G | I | S | S | S | H | - | - | - | - | G | - | - |
| 102 UniRef90\_A0A239MTD5\_1\_271 | W | M | N | - | V | Y | R | Q | F | - | - | R | T | N | V | Q | Q | N | K | E | - | F | - | L | A | - | R | G | - | - | K | L | K | M | P | I | L | A | I | G | A | Q | D | - | - | - | - | S | - | - |
| 103 UniRef90\_UPI000561A6EF\_30\_315 | A | F | G | - | F | Y | R | E | L | - | - | D | R | T | M | A | Q | N | Q | R | - | R | - | - | K | - | E | R | - | - | P | L | T | I | P | V | L | T | I | A | G | A | D | - | - | - | - | S | - | - |
| 104 UniRef90\_A0A4Y8RHA3\_28\_294 | G | F | A | - | Q | F | A | A | F | - | - | D | Q | D | A | V | D | N | Q | A | - | F | - | M | A | - | A | G | - | - | K | L | T | M | P | V | L | A | V | G | G | E | A | - | - | - | - | S | - | - |
| 105 UniRef90\_UPI0004DF415F\_15\_308 | S | L | A | - | Y | Y | R | D | A | - | - | A | E | S | A | R | K | N | H | E | - | A | - | L | E | - | R | Q | - | - | H | L | T | V | P | V | M | G | I | S | G | S | H | - | - | - | - | G | - | - |
| 106 UniRef90\_A0A2N3KZL1\_30\_325 | S | F | E | - | Y | Y | R | A | L | - | - | N | E | T | A | A | R | N | K | S | - | L | - | - | A | - | K | K | - | - | K | L | S | M | P | V | L | A | I | G | G | G | G | H | G | - | - | G | - | - |
| 107 UniRef90\_UPI00076E3DF2\_55\_340 | G | F | E | - | L | Y | R | A | L | - | - | D | E | D | V | R | D | T | G | E | - | - | - | - | - | - | A | A | - | - | P | V | D | V | P | T | L | L | M | T | A | E | G | - | - | - | - | Q | - | - |
| 108 UniRef90\_UPI00130E1FA7\_66\_348 | G | D | G | - | W | Y | Q | S | F | - | - | P | Q | D | L | E | D | N | R | K | - | F | - | - | - | - | P | E | - | - | K | I | D | V | P | S | L | G | I | G | G | I | - | - | - | - | - | - | - | - |
| 109 UniRef90\_A0A1I3FUH2\_46\_328 | G | N | G | - | W | Y | K | A | Y | - | - | P | Q | D | L | V | D | N | A | A | - | Y | - | - | - | - | P | E | - | - | R | I | E | G | P | T | L | G | I | G | G | L | - | - | - | - | - | - | - | - |
| 110 UniRef90\_UPI000E2836BA\_28\_313 | G | Q | G | - | W | F | Q | A | Y | - | - | R | E | D | I | A | D | F | N | A | - | Y | - | - | - | - | - | P | - | - | K | V | T | A | P | M | L | G | L | A | Y | G | - | - | - | - | - | A | - | - |
| 111 UniRef90\_A0A653WNW3\_25\_297 | G | F | A | - | Q | F | A | A | F | - | - | D | Q | D | A | I | D | N | K | A | - | F | - | L | A | Q | Q | G | - | - | K | L | A | M | P | I | L | A | L | G | G | E | K | - | - | - | - | S | - | - |
| 112 UniRef90\_A0A5C1I8U5\_39\_321 | G | N | G | - | W | Y | Q | A | F | - | - | S | K | D | I | A | D | Y | K | T | - | Y | - | - | - | - | - | E | - | - | K | L | T | M | P | V | L | G | I | G | G | P | - | - | - | - | - | - | - | - |
| 113 UniRef90\_UPI00165059AC\_4\_267 | G | F | A | - | D | Y | R | A | A | F | - | A | G | D | A | E | H | D | E | D | - | D | - | - | A | - | G | R | - | - | R | L | T | M | P | V | R | V | L | W | G | E | H | - | - | - | - | S | F | - |
| 114 UniRef90\_F8JLL5\_12\_309 | S | L | A | - | Y | Y | R | D | A | - | - | A | E | S | A | R | K | N | H | E | - | A | - | L | Q | - | Q | Q | - | - | R | L | T | V | P | V | L | G | I | S | S | S | H | - | - | - | - | G | - | - |
| 115 UniRef90\_A0A329J6I4\_30\_314 | S | F | E | - | Y | Y | R | A | L | - | - | N | T | S | I | A | E | N | A | E | - | L | - | - | S | - | K | T | - | - | S | L | N | M | P | V | L | A | I | G | G | G | G | R | N | - | - | G | - | - |
| 116 UniRef90\_A0A3N1H0W7\_60\_340 | G | N | A | - | W | Y | G | T | F | - | - | G | Q | D | V | V | D | L | A | D | - | Y | - | - | - | - | - | G | - | - | R | V | T | A | P | V | L | G | M | V | S | N | L | A | D | G | - | A | - | - |
| 117 UniRef90\_A0A316VRQ3\_1\_263 | G | F | G | - | V | Y | R | A | F | - | - | R | Q | D | D | K | D | V | K | E | - | N | I | A | S | - | K | G | - | - | K | L | S | I | P | I | L | A | T | G | G | S | E | - | - | - | - | S | V | - |
| 118 UniRef90\_A0A4P8X818\_19\_291 | M | F | E | - | V | Y | R | A | T | - | - | L | E | D | G | E | W | N | K | K | - | A | - | - | A | - | E | T | - | - | K | L | P | M | P | V | L | A | V | G | S | K | H | - | - | - | - | F | - | - |
| 119 UniRef90\_UPI0008405A69\_11\_309 | G | N | G | - | W | Y | Q | T | M | - | - | R | Q | D | I | A | D | L | D | T | - | Y | - | - | - | - | - | P | - | - | P | V | T | L | P | I | L | T | L | S | G | E | Q | - | - | - | - | S | - | - |
| 120 UniRef90\_A0A6I8M538\_3\_275 | G | F | D | - | D | Y | R | A | S | F | - | P | D | D | A | E | L | D | D | A | - | D | F | A | A | - | G | K | - | - | R | V | T | Q | P | L | L | A | L | W | G | A | N | - | - | - | - | G | L | - |
| 121 UniRef90\_UPI000B5CCBFE\_21\_300 | T | N | G | - | Y | Y | Q | A | Y | - | - | H | Q | D | I | E | D | L | K | G | - | Y | - | - | - | - | - | D | - | - | K | V | T | V | P | I | L | A | L | A | A | P | T | - | - | - | - | - | - | - |
| 122 UniRef90\_A0A0M9ZDW3\_1\_266 | G | N | A | - | W | Y | Q | E | W | - | - | H | Q | D | M | V | D | D | K | E | - | Y | - | - | P | - | E | T | - | - | P | I | T | V | P | T | L | F | L | A | G | K | - | - | - | - | - | - | - | - |
| 123 UniRef90\_UPI000E275A28\_18\_308 | S | F | E | - | Y | Y | R | A | I | - | - | D | D | D | L | P | Q | N | Y | A | - | R | - | - | K | - | K | T | - | - | K | L | P | M | P | V | L | G | F | A | G | A | L | - | - | - | - | A | - | - |
| 124 UniRef90\_A0A3L8K0H7\_13\_298 | S | Q | G | - | W | F | R | S | Y | - | - | R | Q | D | I | D | D | F | N | T | - | Y | - | - | - | - | - | D | - | - | K | I | T | A | P | M | L | G | L | A | Y | G | - | - | - | - | - | T | - | - |
| 125 UniRef90\_A0A6N7ZB66\_18\_297 | G | F | E | - | H | Y | R | A | L | - | - | L | E | D | G | R | E | N | T | A | - | V | - | L | E | - | K | G | - | - | K | I | T | T | P | L | L | L | I | G | G | K | D | - | - | - | - | S | - | - |
| 126 UniRef90\_A0A2W7GM08\_52\_337 | G | N | A | - | W | Y | Q | A | F | - | - | A | Q | D | I | V | D | Y | R | A | - | Y | - | - | - | - | - | G | - | - | N | L | E | M | P | V | L | A | L | G | G | P | - | - | - | - | - | - | - | - |
| 127 UniRef90\_A0A258JI33\_23\_300 | A | F | E | - | Q | F | A | T | F | N | - | T | K | D | R | A | D | N | L | A | - | F | - | Q | A | - | K | G | - | - | K | L | T | M | P | I | L | A | L | G | A | D | H | - | - | - | - | S | - | - |
| 128 UniRef90\_A0A6A6C1N5\_26\_297 | A | M | G | - | W | Y | A | A | F | - | - | E | T | D | A | E | E | N | L | E | - | W | V | R | R | - | E | G | - | - | K | C | A | V | P | T | M | V | L | S | G | E | R | - | - | - | - | S | W | - |
| 129 UniRef90\_UPI00160A92C2\_5\_284 | A | F | N | - | D | Y | R | A | G | - | - | P | I | D | L | Q | Q | D | Q | E | - | D | - | - | S | - | D | H | - | - | L | I | E | C | P | V | L | V | M | W | G | R | D | F | - | - | - | E | L | - |
| 130 UniRef90\_UPI0009E804D9\_8\_293 | G | F | L | - | H | Y | R | D | L | - | - | L | E | D | G | R | E | N | R | T | - | L | - | L | Q | - | N | R | - | - | P | L | T | M | P | V | L | A | I | G | G | G | D | - | - | - | - | R | - | - |
| 131 UniRef90\_UPI00049086FA\_57\_348 | G | F | E | - | L | Y | R | A | W | - | - | A | Q | D | E | A | E | N | A | R | - | L | - | - | Q | - | D | T | - | - | P | L | T | I | P | V | R | L | L | A | Q | D | G | - | - | - | - | - | - | - |
| 132 UniRef90\_A5FF96\_16\_286 | S | N | G | - | W | Y | Q | A | F | - | - | T | E | D | I | Q | D | I | K | Q | - | Y | - | - | - | - | - | K | - | - | K | V | Q | I | P | A | I | G | I | G | S | Q | E | - | - | - | - | - | - | - |
| 133 UniRef90\_UPI0016621A44\_4\_265 | G | L | G | - | Y | Y | R | T | A | - | - | Q | A | D | A | A | A | V | R | T | - | R | - | - | S | - | H | H | - | - | P | L | D | T | P | V | L | A | I | G | G | R | Y | - | - | - | - | G | - | - |
| 134 UniRef90\_UPI000B83B944\_2\_264 | S | N | G | - | W | Y | N | T | F | - | - | R | Q | D | I | D | D | L | K | T | - | Y | - | - | - | - | - | K | - | - | K | L | S | V | P | A | L | G | I | A | G | W | D | - | - | - | - | S | - | - |
| 135 UniRef90\_A0A0B4DHQ5\_15\_286 | A | M | A | - | D | Y | R | A | N | - | - | A | V | D | V | E | Q | D | K | E | - | D | - | - | A | - | D | V | - | - | K | I | N | C | P | T | L | A | L | W | G | A | D | F | - | - | - | G | A | - |
| 136 UniRef90\_UPI0009B31CF7\_18\_283 | G | F | E | - | H | Y | R | A | M | - | - | P | S | S | A | L | Q | I | Q | S | - | L | - | - | A | D | N | H | - | - | R | L | Q | Q | P | T | L | A | I | S | G | G | V | - | - | - | - | - | - | - |
| 137 UniRef90\_UPI001661B99F\_34\_324 | G | F | D | - | F | F | R | Q | Q | - | - | D | K | G | E | R | E | V | Q | A | - | L | - | L | D | - | D | N | - | - | P | L | T | M | P | V | L | G | I | G | G | Q | H | - | - | - | - | S | - | - |
| 138 UniRef90\_UPI00104123D8\_9\_274 | G | F | D | - | L | Y | R | A | F | - | - | P | Q | D | V | R | D | N | I | A | - | L | - | - | A | E | A | A | - | - | P | V | D | T | P | L | L | Y | V | R | G | D | G | - | - | - | - | E | - | - |
| 139 UniRef90\_A0A1M7Z6I5\_10\_283 | A | M | A | - | D | Y | R | A | N | - | - | A | E | D | N | A | Q | D | L | V | - | D | - | - | A | - | D | V | - | - | K | I | G | C | P | V | L | S | L | W | G | A | D | F | - | - | - | G | A | - |
| 140 UniRef90\_A0A431M1N9\_22\_292 | A | F | S | G | Q | F | A | A | F | - | - | A | Q | D | A | E | D | N | K | Q | - | L | - | F | A | R | N | G | - | - | K | L | G | M | P | V | L | A | I | G | G | D | H | - | - | - | - | S | - | - |
| 141 UniRef90\_UPI0013D28676\_49\_329 | G | F | E | - | L | F | R | Q | Q | - | - | D | A | G | E | Q | Q | V | G | A | - | L | V | T | K | - | D | G | - | - | K | L | A | I | P | T | L | G | V | G | G | Q | N | - | - | - | - | S | - | - |
| 142 UniRef90\_A0A402BD85\_4\_199 | Y | I | S | - | Y | F | - | - | - | - | - | - | - | - | - | - | - | - | - | - | - | - | - | - | - | - | - | - | - | - | - | - | - | - | - | - | - | - | - | - | - | - | - | - | - | - | - | - | - | - |
| 143 UniRef90\_A0A2V5S4I0\_55\_307 | G | W | A | - | Y | F | V | S | W | - | - | P | Q | L | A | K | D | F | A | Q | - | L | - | - | S | - | Q | T | - | - | K | L | T | T | P | V | L | S | I | G | R | R | E | - | - | - | - | R | - | - |
| 144 UniRef90\_A0A328AN40\_21\_303 | G | F | N | - | W | Y | R | A | L | - | - | F | R | - | - | A | E | L | A | V | - | P | - | - | A | - | K | G | - | - | S | I | G | V | P | T | L | V | L | W | G | D | Q | - | - | - | - | D | P | - |
| 145 UniRef90\_A0A0M9AP20\_25\_244 | A | V | N | - | Y | Y | R | A | L | - | - | A | R | R | N | A | K | L | T | L | - | T | - | - | A | - | G | - | - | - | - | - | - | - | - | - | - | - | - | - | - | - | - | - | - | - | - | - | - | - |
| 146 UniRef90\_A0A1N6RLL9\_1\_202 | G | L | E | - | F | Y | R | A | F | - | - | P | A | N | E | K | F | N | A | A | - | - | - | - | K | - | R | N | - | - | V | L | N | N | P | I | V | L | A | G | G | D | K | - | - | - | - | A | - | - |
| 147 UniRef90\_A0A4R3LTR2\_48\_312 | I | C | E | - | D | Y | R | A | A | A | - | G | I | D | L | V | H | D | A | A | - | D | - | - | R | - | E | T | - | - | R | I | A | A | P | L | L | A | L | W | G | A | K | - | - | - | - | G | V | - |
| 148 UniRef90\_UPI0002ED95C2\_14\_285 | V | C | A | - | D | Y | R | A | S | A | - | Y | V | D | P | A | H | D | E | A | - | D | R | R | D | - | G | R | - | - | R | L | E | M | P | V | A | A | L | W | Q | D | P | - | - | - | - | G | E | - |
| 149 UniRef90\_A0A1Q7MBV0\_16\_212 | P | I | N | - | Y | Y | R | A | A | - | - | - | - | - | - | - | - | - | - | - | - | - | - | - | - | - | - | - | - | - | - | - | - | - | - | - | - | - | - | - | - | - | - | - | - | - | - | - | - | - |
| 150 UniRef90\_A0A1A0KI05\_7\_212 | S | I | A | - | W | Y | R | A | G | - | - | - | - | - | - | - | - | - | - | - | - | - | - | - | - | - | - | - | - | - | - | - | - | - | - | - | - | - | - | - | - | - | - | - | - | - | - | - | - | - |

  
  

|  |  |  |  |  |  |  |  |  |  |  |  |  |  |  |  |  |  |  |  |  |  |  |  |  |  |  |  |  |  |  |  |  |  |  |  |  |  |  |  |  |  |  |  |  |  |  |  |  |  |  |
| --- | --- | --- | --- | --- | --- | --- | --- | --- | --- | --- | --- | --- | --- | --- | --- | --- | --- | --- | --- | --- | --- | --- | --- | --- | --- | --- | --- | --- | --- | --- | --- | --- | --- | --- | --- | --- | --- | --- | --- | --- | --- | --- | --- | --- | --- | --- | --- | --- | --- | --- |
| **001 Input\_protein\_seq** | - | - | - | N | G | A | - | - | - | K | E | L | D | M | A | R | E | L | A | - | - | L | D | - | - | V | R | G | A | V | A | P | N | T | G | H | W | L | P | D | E | N | P | A | F | L | T | R | Q | L |
| 002 UniRef90\_UPI00158A2D6D\_3\_306 | - | - | - | N | G | T | - | - | - | R | E | L | D | M | A | R | E | L | A | - | - | V | D | - | - | V | R | G | A | V | A | P | N | T | G | H | W | L | P | D | E | N | P | A | F | L | T | R | Q | L |
| 003 UniRef90\_A0A1Z4J856\_27\_318 | N | P | A | L | G | D | - | - | - | P | A | R | T | S | I | Q | L | L | A | - | - | E | N | - | - | V | R | Y | S | A | I | E | N | C | G | H | W | I | P | E | E | R | P | A | Y | L | V | Q | Q | L |
| 004 UniRef90\_G7LVZ3\_7\_288 | - | - | - | M | G | D | - | - | - | Q | V | G | H | I | M | R | P | L | A | - | - | D | N | - | - | V | Q | T | A | V | V | E | R | S | G | H | W | I | P | E | E | Q | P | E | Q | L | A | A | L | L |
| 005 UniRef90\_A0A2I8F4B9\_53\_324 | - | - | - | F | G | P | - | - | - | I | I | G | D | H | L | R | H | V | A | - | - | T | N | - | - | V | E | A | I | N | V | Q | G | S | G | H | W | V | A | E | E | Q | P | A | V | V | T | G | A | L |
| 006 UniRef90\_A0A327RPK7\_10\_284 | - | - | - | T | G | D | - | - | - | N | F | R | K | S | L | S | L | I | A | - | - | N | H | - | - | V | E | G | G | S | I | P | E | C | G | H | Y | I | A | E | E | Q | P | E | F | L | I | K | V | L |
| 007 UniRef90\_A0A6L3SWG4\_20\_294 | - | - | - | F | A | R | - | - | - | S | M | D | G | M | M | R | E | V | A | - | - | Q | D | - | - | V | T | F | Q | V | I | E | Q | A | N | H | W | I | P | E | E | N | A | E | A | L | A | A | A | V |
| 008 UniRef90\_A0A401ZLI5\_4\_287 | - | - | - | G | A | G | - | - | - | W | P | F | Y | S | L | A | Q | L | A | - | - | E | N | - | - | V | S | S | G | I | I | S | Q | C | G | H | Y | I | A | E | E | Q | P | E | E | L | L | Q | R | L |
| 009 UniRef90\_A0A4V2U6R1\_12\_297 | - | - | - | F | L | P | - | - | - | I | A | E | Q | M | L | L | E | V | A | - | - | E | K | - | - | V | T | V | R | P | I | E | D | C | G | H | W | I | A | E | E | Q | P | E | R | L | L | Q | Q | L |
| 010 UniRef90\_A0A5A5T922\_6\_282 | - | - | - | L | G | P | - | - | - | K | T | R | Q | A | L | E | V | L | A | - | - | T | D | - | - | V | R | G | G | I | I | K | N | C | G | H | F | V | P | E | E | Y | P | D | L | L | A | S | Q | L |
| 011 UniRef90\_A0A2V6UIN9\_3\_273 | - | - | - | S | G | E | - | - | - | F | L | I | T | Q | G | R | L | V | A | - | - | E | K | - | - | V | E | G | V | V | I | K | G | S | G | H | W | L | I | D | E | A | P | E | Q | V | I | P | K | L |
| 012 UniRef90\_UPI001669B3C5\_41\_314 | - | - | - | G | S | T | - | - | - | F | L | V | D | Q | V | K | L | V | A | - | - | T | D | - | - | V | K | G | E | V | I | P | N | S | G | H | W | L | M | E | E | A | P | E | K | V | I | P | L | L |
| 013 UniRef90\_A0A4R2Z7A8\_3\_305 | - | - | - | S | I | P | - | - | - | D | M | A | V | P | L | R | D | F | A | - | - | E | N | - | - | V | T | G | I | I | I | A | N | S | G | H | F | I | P | E | E | Q | P | E | A | L | A | R | E | L |
| 014 UniRef90\_A0A5C5SVV1\_11\_281 | - | - | - | W | G | P | - | - | - | Q | I | V | A | I | L | Q | E | F | A | - | - | E | D | - | - | V | R | G | G | S | I | V | D | C | N | H | W | L | P | E | E | R | P | R | E | T | A | D | A | L |
| 015 UniRef90\_L9WLS3\_16\_284 | - | - | - | F | R | S | - | - | - | L | P | I | E | D | M | N | A | V | A | - | - | T | D | - | - | V | E | G | E | V | L | E | R | A | G | H | W | I | P | E | E | R | P | E | Y | F | V | E | R | V |
| 016 UniRef90\_UPI00131EC7B5\_30\_311 | - | - | - | S | I | A | - | - | - | D | M | A | T | P | L | R | P | F | A | - | - | E | D | - | - | V | A | G | V | L | V | T | H | C | G | H | F | L | P | E | E | Q | P | Q | V | I | A | R | E | L |
| 017 UniRef90\_C7QAM5\_50\_332 | - | - | - | L | G | K | - | - | - | A | V | P | D | Q | V | R | Q | Y | A | - | - | K | H | - | - | V | T | G | V | V | V | P | D | S | G | H | W | M | Y | E | E | H | P | A | E | M | A | H | I | L |
| 018 UniRef90\_A0A1Q3SXP6\_11\_288 | - | - | - | S | G | E | - | - | - | F | L | I | E | Q | G | R | Q | V | N | - | - | N | N | - | - | I | Q | G | K | I | I | K | N | A | G | H | W | L | M | E | E | A | P | Q | Q | V | I | P | E | L |
| 019 UniRef90\_UPI00149248BF\_39\_315 | - | - | - | A | G | Q | - | - | - | L | T | I | E | T | M | K | L | V | S | - | - | D | N | - | - | V | Q | G | Q | I | I | E | R | C | G | H | Y | T | P | E | E | C | P | A | E | L | L | H | L | L |
| 020 UniRef90\_UPI000361D127\_26\_313 | - | - | - | G | G | E | - | - | - | N | V | E | R | E | V | R | S | L | A | - | - | D | D | - | - | V | T | G | V | V | I | A | N | C | G | H | F | V | A | E | E | A | P | E | A | F | L | N | A | L |
| 021 UniRef90\_A0A0M4FVH2\_24\_312 | - | - | - | S | I | A | - | - | - | D | M | V | T | P | L | K | A | F | A | - | - | E | D | - | - | V | Q | G | G | T | I | S | F | C | G | H | F | L | P | E | E | Q | P | E | A | V | S | R | E | L |
| 022 UniRef90\_A0A1Q4ZL08\_9\_290 | - | - | - | L | G | D | - | - | - | A | V | S | Q | Q | A | A | R | Y | A | - | - | S | N | - | - | V | S | G | G | V | V | E | D | C | G | H | W | I | F | E | E | R | P | A | E | L | T | S | Q | L |
| 023 UniRef90\_S3CY91\_25\_307 | - | - | - | L | L | Q | - | - | - | V | A | E | E | Q | T | R | E | F | Y | - | - | E | S | - | - | V | E | V | A | T | V | E | G | S | G | H | W | C | A | E | E | N | P | G | G | L | V | R | K | V |
| 024 UniRef90\_UPI0010F95BB2\_18\_294 | - | - | - | Y | R | A | - | - | - | A | S | L | S | T | M | E | R | V | A | - | - | T | N | - | - | P | E | G | W | I | V | P | N | C | G | H | Y | V | P | E | E | C | P | D | Q | L | A | E | R | L |
| 025 UniRef90\_A0A0N1GDE2\_28\_316 | - | - | - | T | G | V | - | - | - | M | V | A | E | T | M | R | L | A | A | - | - | D | D | - | - | V | T | G | V | V | L | D | D | C | G | H | Y | A | A | E | E | Q | P | A | R | F | T | E | I | L |
| 026 UniRef90\_A0A2E5PJQ7\_5\_284 | - | - | - | R | G | E | - | - | - | E | P | E | K | S | M | R | R | V | A | - | - | E | N | - | - | V | T | G | M | V | A | A | E | S | G | H | F | I | P | E | E | Q | P | D | F | V | A | E | K | L |
| 027 UniRef90\_A0A1M7IC97\_44\_328 | - | - | - | L | G | R | - | - | - | A | V | G | D | Q | A | E | Q | Y | A | - | - | S | D | - | - | V | T | G | V | T | I | P | D | S | G | H | W | I | Y | E | E | H | P | E | E | L | T | T | M | L |
| 028 UniRef90\_UPI0016149F5E\_5\_284 | - | - | - | T | N | D | - | - | - | A | P | L | L | T | M | Q | G | K | A | - | - | S | I | - | - | L | Q | G | A | I | V | T | E | C | G | H | F | I | M | E | E | Q | P | E | A | F | I | A | H | L |
| 029 UniRef90\_UPI00156E1DFF\_8\_289 | - | - | - | T | G | T | - | - | - | Q | L | A | D | S | L | N | T | V | A | - | - | Q | R | - | - | L | A | G | V | V | I | P | G | S | G | H | F | V | P | E | E | C | P | D | A | F | A | R | E | L |
| 030 UniRef90\_A0A3A8HRU0\_15\_300 | - | - | - | M | G | E | - | - | - | P | L | V | A | Q | A | R | A | V | A | - | - | A | Q | - | - | V | E | P | H | I | L | R | D | T | G | H | W | V | T | E | E | R | P | E | E | V | R | Q | L | L |
| 031 UniRef90\_A0A0M3UDU6\_18\_309 | - | - | - | V | G | D | - | - | - | H | I | V | K | A | L | Q | A | L | A | - | - | T | N | - | - | V | Q | G | D | V | I | L | D | A | G | H | Y | L | P | E | E | A | A | N | R | I | A | D | L | L |
| 032 UniRef90\_A0A447J1A9\_28\_317 | - | - | - | T | R | D | - | - | - | A | P | Q | L | T | M | Q | G | R | A | - | - | V | N | - | - | L | Q | G | A | I | L | S | E | C | G | H | F | V | T | E | E | C | P | E | Q | L | M | D | V | V |
| 033 UniRef90\_A0A5C8T429\_14\_295 | - | - | - | L | G | S | - | - | - | M | M | E | P | M | M | R | P | V | A | - | - | E | D | - | - | V | T | G | L | V | V | P | D | C | G | H | Y | V | P | E | E | A | P | N | F | L | A | E | H | L |
| 034 UniRef90\_A0A0N0TCD7\_1\_288 | - | - | - | T | G | T | - | - | - | M | P | E | E | V | M | R | M | V | A | - | - | T | D | - | - | V | T | G | L | I | I | P | A | A | G | H | F | L | P | E | E | A | P | Q | A | L | T | T | A | L |
| 035 UniRef90\_W9ARP2\_16\_295 | - | - | - | L | A | Q | - | - | - | N | F | R | E | M | C | E | E | I | A | - | - | D | S | - | - | V | T | S | Q | L | V | P | D | C | G | H | W | V | P | E | E | Q | P | E | Y | F | T | N | M | F |
| 036 UniRef90\_A0A109IGY4\_11\_301 | - | - | - | S | G | A | - | - | - | L | V | A | R | T | M | R | L | A | A | - | - | A | D | - | - | V | T | E | A | I | I | E | D | C | G | H | Y | A | A | E | E | Q | P | A | R | F | T | G | I | L |
| 037 UniRef90\_K9DQK6\_24\_306 | - | - | - | F | G | P | - | - | - | M | M | A | T | V | M | R | N | A | A | - | - | L | D | - | - | V | R | Q | A | V | V | P | G | A | G | H | W | M | M | E | E | N | P | D | A | T | V | E | L | I |
| 038 UniRef90\_A0A1Q8IR73\_6\_294 | - | - | - | S | I | P | - | - | - | D | M | S | A | A | L | R | P | F | A | - | - | N | E | - | - | V | K | G | E | T | I | A | N | C | G | H | F | Q | P | E | E | Q | P | E | A | V | A | D | A | L |
| 039 UniRef90\_A0A1Y6D2S0\_29\_302 | - | - | - | V | A | D | - | - | - | K | L | S | K | A | L | Q | A | K | A | - | - | D | I | - | - | I | K | S | A | I | A | E | D | S | G | H | F | V | P | E | E | A | P | A | F | L | V | E | Q | L |
| 040 UniRef90\_E6WJ64\_19\_301 | - | - | - | S | I | P | - | - | - | D | M | A | T | P | L | R | Q | F | A | - | - | D | D | - | - | V | T | G | I | T | L | A | H | S | G | H | F | I | P | D | E | Q | P | L | A | L | A | A | A | L |
| 041 UniRef90\_UPI000489A94A\_43\_316 | - | - | - | S | G | T | - | - | - | F | L | V | D | Q | A | K | L | V | D | - | - | T | H | - | - | V | Q | A | V | V | V | P | G | A | G | H | W | L | M | E | E | A | P | A | P | T | M | S | A | I |
| 042 UniRef90\_A0A4R6HBQ4\_11\_285 | - | - | - | Y | G | E | - | - | - | A | A | L | N | T | M | K | R | V | A | - | - | T | E | - | - | P | E | G | A | I | I | K | D | C | G | H | Y | V | P | E | E | R | P | R | E | L | V | E | T | M |
| 043 UniRef90\_A0A1M7QJX9\_32\_311 | - | - | - | A | G | S | - | - | - | Y | L | A | A | H | S | R | L | V | A | - | - | I | N | - | - | V | K | E | V | I | I | K | G | S | G | H | W | V | V | Q | E | Q | T | A | Q | V | Q | K | A | L |
| 044 UniRef90\_A0A5B8WA72\_22\_300 | - | - | - | A | G | A | - | - | - | F | L | A | T | H | T | R | L | V | A | - | - | T | N | - | - | V | Q | E | S | I | I | K | D | S | G | H | W | V | V | Q | E | N | T | P | Q | V | Q | K | D | L |
| 045 UniRef90\_A0A0G2FGE6\_47\_303 | - | - | - | L | A | N | - | - | - | I | A | E | E | Q | N | L | E | A | Y | - | - | Q | A | - | - | T | E | T | A | T | I | P | G | A | G | H | W | C | A | E | E | N | P | G | A | F | T | Q | T | V |
| 046 UniRef90\_A0A1I6BK09\_10\_296 | - | - | - | G | G | A | - | - | - | Y | I | A | Q | A | F | A | V | A | A | - | - | E | D | - | - | V | R | P | L | V | L | E | G | A | G | H | W | L | A | E | E | Q | P | A | A | L | A | T | H | F |
| 047 UniRef90\_A0A4R7C9R7\_33\_310 | - | - | - | G | G | S | - | - | - | R | L | A | D | S | L | R | A | E | A | - | - | P | S | - | - | L | T | S | V | I | I | D | D | C | G | H | F | V | A | E | E | A | P | D | P | F | - | - | - | - |
| 048 UniRef90\_A0A2M9M9Q1\_18\_305 | - | - | - | Q | G | D | - | - | - | R | L | A | A | L | L | G | P | V | A | - | - | E | R | - | - | F | T | G | A | V | L | P | D | C | G | H | Y | V | P | E | E | C | P | D | A | L | L | D | H | L |
| 049 UniRef90\_A0A4V3T343\_11\_295 | - | - | - | N | Y | D | - | - | - | L | L | T | E | A | V | P | P | L | G | - | - | T | D | - | - | T | R | V | V | R | V | D | K | S | G | H | Y | L | P | E | E | R | P | E | V | V | V | E | E | L |
| 050 UniRef90\_UPI001616534D\_11\_303 | - | - | - | N | Y | V | - | - | - | H | L | R | E | L | M | P | S | K | G | - | - | T | D | - | - | V | Q | V | A | E | V | A | D | C | G | H | Y | I | P | E | E | Q | P | Q | A | V | I | D | A | L |
| 051 UniRef90\_J4PG95\_17\_290 | - | - | - | T | R | D | - | - | - | A | P | L | I | T | M | R | D | N | A | - | - | T | D | - | - | L | T | G | V | I | I | A | D | C | G | H | F | V | T | E | E | C | H | E | A | F | S | E | H | L |
| 052 UniRef90\_A0A0T1T741\_1\_295 | - | - | - | S | G | A | - | - | - | N | A | A | R | T | M | R | L | T | A | - | - | D | D | - | - | V | T | G | V | V | L | D | D | C | G | H | Y | P | A | E | E | Q | P | A | R | C | A | E | I | L |
| 053 UniRef90\_G0FSK7\_17\_310 | - | - | - | T | G | P | - | - | - | L | V | E | R | T | M | I | P | A | A | - | - | A | D | - | - | V | R | G | V | V | L | P | G | C | G | H | Y | P | A | E | E | A | P | Q | A | M | L | A | A | L |
| 054 UniRef90\_UPI00164AAB2D\_18\_303 | - | - | - | F | G | A | - | - | - | N | E | A | A | V | M | R | N | A | A | - | - | T | N | - | - | V | T | E | K | V | I | P | N | A | G | H | W | L | M | E | E | A | P | D | A | T | I | E | A | V |
| 055 UniRef90\_A0A1I2MT44\_20\_306 | - | - | - | L | G | A | - | - | - | A | V | A | A | T | M | D | R | A | A | - | - | D | D | - | - | V | T | G | V | V | I | P | G | C | G | H | F | P | P | E | E | T | P | Q | E | L | L | S | A | L |
| 056 UniRef90\_A0A2P2CCU5\_19\_306 | - | - | - | W | G | G | - | - | - | A | V | G | G | A | M | S | A | L | A | - | - | G | H | - | - | V | E | T | L | V | V | P | G | T | G | H | W | V | A | E | A | A | P | E | A | V | I | E | A | L |
| 057 UniRef90\_A0A2V9H5C8\_12\_298 | - | - | - | L | G | E | - | - | - | F | L | G | E | Q | M | K | L | V | A | - | - | A | D | - | - | V | T | A | V | V | L | K | D | T | G | H | W | V | L | E | E | N | P | K | E | T | T | D | A | L |
| 058 UniRef90\_A0A0P4V0Y0\_7\_291 | - | - | - | I | G | E | - | - | - | K | V | K | L | M | M | Q | S | V | A | - | - | T | D | - | - | V | R | G | G | S | V | D | R | C | G | H | F | I | P | D | E | R | P | D | A | L | I | D | Q | L |
| 059 UniRef90\_A0A0Q4UQ58\_27\_305 | - | - | - | V | G | S | - | - | - | R | L | S | D | A | L | S | R | E | S | - | - | D | R | - | - | L | E | T | V | I | V | E | E | S | G | H | F | V | A | E | E | A | P | E | T | F | - | - | - | - |
| 060 UniRef90\_A0A6M4IM28\_20\_309 | - | - | - | F | G | A | - | - | - | V | M | A | T | V | M | R | F | V | A | - | - | T | D | - | - | V | T | E | A | I | V | P | G | S | G | H | W | I | M | E | E | N | P | T | A | T | I | A | I | V |
| 061 UniRef90\_A0A537J419\_20\_301 | - | - | - | L | G | E | - | - | - | L | L | G | Q | Q | M | K | L | V | A | - | - | S | D | - | - | V | T | V | V | V | L | K | D | T | G | H | W | V | L | E | E | R | P | K | E | T | T | D | A | L |
| 062 UniRef90\_A0A1V2PGN8\_6\_275 | - | - | - | L | G | K | - | - | L | P | T | A | D | I | W | R | E | Y | A | - | - | E | D | - | - | V | T | G | Y | A | I | A | E | C | G | H | F | L | A | E | E | Q | P | S | T | V | A | G | H | L |
| 063 UniRef90\_A0A542JC81\_20\_314 | - | - | - | Y | G | H | - | - | - | H | V | E | E | N | M | R | L | L | A | - | - | D | D | - | - | V | E | G | V | V | I | A | G | A | G | H | W | V | A | E | E | A | P | E | Q | L | L | A | A | L |
| 064 UniRef90\_UPI000424F353\_33\_308 | - | - | - | F | G | P | - | - | - | L | M | A | T | V | M | R | A | A | A | - | - | T | N | - | - | V | Q | E | A | V | V | P | H | A | G | H | W | L | M | E | E | N | P | N | A | T | I | K | F | V |
| 065 UniRef90\_A0A2V9W2E9\_22\_299 | - | - | - | F | G | P | - | - | - | L | Q | A | V | I | M | R | H | V | A | - | - | T | N | - | - | V | Q | E | A | V | V | T | G | S | G | H | W | L | M | E | E | R | P | A | Y | T | V | T | L | I |
| 066 UniRef90\_A0A2T6L0M5\_16\_299 | - | - | - | L | G | K | - | - | - | S | I | G | T | Q | L | E | R | Y | A | - | - | T | Q | - | - | V | D | T | R | V | L | A | G | A | G | H | W | V | T | E | E | R | P | H | E | V | T | E | L | L |
| 067 UniRef90\_A0A261TYY2\_43\_315 | - | - | - | L | G | G | - | - | - | M | M | E | T | M | L | S | R | V | A | - | - | D | D | - | - | V | T | G | - | V | I | A | P | C | G | H | Y | V | P | E | E | A | P | E | F | L | V | A | Q | L |
| 068 UniRef90\_UPI0009781B11\_10\_287 | - | - | - | V | G | E | - | - | - | N | M | H | R | S | I | V | G | A | A | - | - | L | D | - | - | V | R | G | T | V | I | A | N | C | G | H | Y | V | P | E | E | K | P | S | E | L | I | D | A | M |
| 069 UniRef90\_A0A2V9TPC1\_35\_307 | - | - | - | L | G | E | - | - | - | A | L | G | Q | Q | T | K | L | V | A | - | - | T | D | - | - | V | T | V | V | V | L | K | D | T | G | H | W | V | L | E | E | R | P | K | E | T | T | E | A | L |
| 070 UniRef90\_UPI00145FA88A\_6\_279 | - | - | - | L | G | K | - | - | L | P | A | L | E | I | W | Q | D | Y | A | - | - | D | D | - | - | V | R | G | W | A | I | A | D | C | G | H | F | L | P | E | E | Q | P | G | E | T | L | R | A | L |
| 071 UniRef90\_UPI0003FADEBE\_21\_306 | - | - | - | F | G | A | - | - | - | N | E | A | V | V | M | R | N | A | A | - | - | S | D | - | - | V | T | E | L | V | V | P | G | T | G | H | W | L | M | E | E | A | P | D | A | T | V | K | A | V |
| 072 UniRef90\_A0A2V8Y3P4\_78\_329 | - | - | - | F | G | A | - | - | - | L | Q | A | V | I | M | R | H | V | A | - | - | N | N | - | - | V | Q | E | E | V | V | A | G | S | G | H | W | L | M | E | E | R | P | A | Y | T | V | A | L | I |
| 073 UniRef90\_A0A285BKA7\_8\_299 | - | - | - | T | G | T | - | - | - | M | P | A | D | T | M | R | L | V | A | - | - | T | D | - | - | V | T | E | L | V | I | P | Q | A | G | H | F | L | P | E | E | V | P | Q | E | L | T | K | H | L |
| 074 UniRef90\_A0A0K3AVW3\_44\_319 | - | - | - | Y | N | D | - | - | - | T | L | E | H | S | L | P | D | K | G | - | - | T | D | - | - | V | R | L | G | K | I | E | N | S | G | H | F | L | P | E | E | Q | P | D | A | V | L | K | A | L |
| 075 UniRef90\_A0A4D7B7N1\_7\_276 | - | - | - | M | S | P | - | - | - | N | I | Y | D | A | M | K | P | L | G | - | - | E | N | - | - | V | Q | G | G | I | V | A | D | C | G | H | Y | M | P | E | E | Q | P | E | V | I | A | E | R | M |
| 076 UniRef90\_A0A2U0WF37\_17\_293 | - | - | - | F | G | G | - | - | - | N | E | A | I | V | M | R | N | A | A | - | - | D | D | - | - | V | T | E | L | V | V | P | G | A | G | H | W | L | M | E | E | A | P | G | E | L | I | - | - | - |
| 077 UniRef90\_UPI00135C2D49\_17\_309 | - | - | - | N | H | A | - | - | - | S | L | S | G | V | L | P | A | K | G | - | - | T | D | - | - | V | R | V | L | R | V | D | D | C | G | H | Y | I | P | E | E | Q | P | A | A | V | I | E | A | L |
| 078 UniRef90\_UPI001473614E\_45\_322 | - | - | - | F | G | P | - | - | - | G | V | A | D | S | F | R | Q | V | A | - | - | D | D | - | - | V | R | T | V | V | A | P | D | S | G | H | F | V | P | E | E | N | P | R | F | M | A | - | - | - |
| 079 UniRef90\_J3F862\_22\_299 | - | - | - | F | G | N | - | - | - | N | E | A | I | V | M | R | N | A | A | - | - | D | N | - | - | V | T | E | L | V | I | A | G | A | G | H | W | L | M | E | E | A | P | V | E | T | T | Q | A | I |
| 080 UniRef90\_A0A1A9NC01\_62\_345 | - | - | - | A | Y | G | - | - | - | W | M | K | K | V | I | S | Q | K | A | - | - | T | N | - | - | L | N | A | V | E | V | Q | D | S | G | H | F | V | Q | E | E | Q | P | E | L | V | A | K | T | M |
| 081 UniRef90\_UPI00131AC11B\_45\_327 | - | - | - | G | Y | P | - | - | - | R | L | K | A | S | L | D | A | S | A | - | - | P | G | - | - | S | S | T | F | R | I | D | G | S | G | H | F | I | A | E | E | K | P | F | V | L | L | F | Y | L |
| 082 UniRef90\_A0A4R8HC20\_22\_306 | - | - | - | N | H | E | - | - | - | S | L | V | T | A | V | P | A | L | G | - | - | T | D | - | - | T | R | V | V | R | V | D | G | S | G | H | Y | L | P | E | E | R | P | E | V | V | V | E | A | L |
| 083 UniRef90\_A0A1B2HH26\_4\_280 | - | - | - | P | A | D | - | - | - | V | L | L | T | G | A | R | E | A | A | - | - | T | D | - | - | V | R | A | E | I | V | P | D | A | A | H | T | F | A | A | D | N | P | S | A | T | A | D | V | L |
| 084 UniRef90\_A0A517LKB1\_23\_304 | - | - | - | L | K | G | - | - | - | G | A | E | S | I | A | R | E | F | Y | - | - | E | N | - | - | P | V | V | G | L | V | E | G | S | G | H | W | L | A | E | E | N | P | E | G | F | A | K | E | V |
| 085 UniRef90\_A0A0N1F3N8\_17\_298 | - | - | - | F | G | L | - | - | - | T | M | A | E | V | M | K | F | A | A | - | - | S | D | - | - | V | Q | G | A | I | V | P | D | S | G | H | W | I | M | E | E | N | P | A | A | T | I | K | L | V |
| 086 UniRef90\_UPI0004CCFD44\_55\_340 | - | - | - | L | A | P | - | - | - | V | R | A | T | A | A | Q | R | M | T | - | - | N | I | - | - | V | R | A | V | D | V | P | N | A | G | H | W | L | V | E | E | N | P | Q | F | V | T | T | E | L |
| 087 UniRef90\_A0A4D4LDP7\_7\_304 | - | - | - | S | I | P | - | - | - | D | M | A | A | P | L | T | P | F | V | - | - | P | H | - | - | V | R | R | A | S | I | A | G | A | G | H | F | I | P | D | E | Q | P | A | A | L | A | Q | I | L |
| 088 UniRef90\_A0A2V4B948\_8\_279 | - | - | - | L | G | S | - | - | L | P | A | E | E | I | W | R | D | Y | A | - | - | T | D | - | - | V | T | G | F | P | V | A | D | C | G | H | F | L | P | E | E | Q | P | E | V | V | T | E | R | L |
| 089 UniRef90\_A0A2P2FV90\_3\_282 | - | - | - | L | G | K | - | - | L | P | T | L | E | I | W | K | D | Y | A | - | - | N | D | - | - | V | S | G | T | A | I | E | E | C | G | H | F | L | A | E | E | R | P | R | A | L | L | E | N | L |
| 090 UniRef90\_A0A4Q2J292\_17\_304 | - | - | - | F | G | S | - | - | - | T | M | E | T | V | V | R | F | A | A | - | - | S | N | - | - | V | Q | G | A | V | I | P | G | S | G | H | W | L | M | E | E | Q | P | K | A | T | V | A | V | I |
| 091 UniRef90\_UPI0012B05B7D\_16\_287 | - | - | - | S | A | P | - | - | - | D | L | H | D | R | I | K | Q | L | A | - | - | I | D | - | - | V | Y | G | G | H | I | K | D | S | G | H | Y | I | P | E | E | Q | P | L | A | L | V | E | E | I |
| 092 UniRef90\_UPI00048D4AD4\_4\_301 | - | - | - | G | Y | R | - | - | - | W | L | R | T | I | M | P | A | K | G | - | - | T | D | - | - | V | R | V | V | E | V | S | D | C | G | H | Y | I | P | E | E | Q | P | H | A | V | I | D | E | L |
| 093 UniRef90\_UPI0015F81933\_16\_310 | - | - | - | S | I | P | - | - | - | D | M | A | A | S | I | R | P | W | A | - | - | D | H | - | - | A | T | G | T | V | V | P | D | A | G | H | F | I | P | D | E | Q | P | D | A | V | A | A | A | L |
| 094 UniRef90\_UPI00160CFF45\_55\_340 | - | - | - | I | K | A | - | - | - | I | R | A | T | V | S | P | R | L | T | - | - | N | I | - | - | V | R | A | V | D | V | P | K | A | G | H | W | L | I | E | E | N | P | Q | F | V | T | K | E | L |
| 095 UniRef90\_A0A1Q8KTW7\_17\_294 | - | - | - | I | G | E | - | - | - | E | S | R | R | Q | M | E | R | T | A | - | - | T | D | - | - | V | R | Y | V | E | I | A | E | C | G | H | S | L | A | L | E | R | P | A | E | L | A | R | L | L |
| 096 UniRef90\_UPI00161A7EC4\_21\_297 | - | - | - | F | G | P | - | - | - | G | V | A | D | S | F | R | L | V | A | - | - | D | D | - | - | V | R | P | V | V | V | P | D | S | G | H | Y | I | A | E | E | N | S | S | Y | L | G | - | - | - |
| 097 UniRef90\_A0A1B4WXA8\_16\_299 | - | - | - | F | G | S | - | - | - | N | E | A | I | V | M | R | N | A | A | - | - | D | K | - | - | V | T | E | V | V | I | P | G | A | G | H | W | L | M | E | E | A | P | T | Q | T | I | A | A | I |
| 098 UniRef90\_UPI000E46D2C1\_48\_330 | - | - | - | G | N | V | - | - | - | L | L | V | E | F | L | Q | G | R | F | - | - | V | N | - | - | P | R | L | E | H | L | P | E | A | G | H | Y | V | A | E | E | A | P | E | E | V | T | A | S | L |
| 099 UniRef90\_A0A2N5CFI4\_23\_293 | - | - | - | F | G | V | - | - | - | M | M | E | T | V | A | R | A | A | A | - | - | S | N | - | - | V | Q | G | V | V | I | P | N | A | G | H | W | L | M | E | E | Q | Q | Q | A | - | - | - | - | - |
| 100 UniRef90\_A0A385B2U0\_45\_333 | - | - | - | L | S | S | - | - | - | A | V | G | T | Q | W | Q | G | Y | A | - | - | A | N | - | - | V | E | T | Q | V | M | T | D | T | G | H | W | L | T | E | E | R | P | Q | E | L | T | T | M | L |
| 101 UniRef90\_A0A1Q7W147\_16\_308 | - | - | - | S | I | P | - | - | - | D | M | A | A | S | I | S | P | W | A | - | - | K | N | - | - | A | T | G | I | V | V | P | D | A | G | H | F | I | P | D | E | Q | P | E | A | V | A | A | A | L |
| 102 UniRef90\_A0A239MTD5\_1\_271 | - | - | - | F | G | G | - | - | - | I | V | V | D | Q | W | R | D | Y | A | - | - | V | N | - | - | V | D | G | R | V | L | K | N | S | G | H | F | V | T | E | E | K | P | Q | E | V | T | A | M | L |
| 103 UniRef90\_UPI000561A6EF\_30\_315 | - | - | - | L | G | G | - | - | - | A | V | G | G | A | M | R | L | A | A | - | - | E | D | - | - | V | R | E | V | I | L | P | Q | C | G | H | Y | P | A | E | E | A | P | E | A | T | L | A | A | - |
| 104 UniRef90\_A0A4Y8RHA3\_28\_294 | - | - | - | F | G | T | - | - | - | T | M | A | A | V | T | G | F | A | A | - | - | S | N | - | - | V | K | G | A | V | I | K | G | A | G | H | W | L | M | E | E | Q | P | A | A | T | V | - | - | - |
| 105 UniRef90\_UPI0004DF415F\_15\_308 | - | - | - | S | T | P | - | - | - | D | M | A | A | S | L | G | P | W | A | - | - | K | N | - | - | T | T | G | T | V | I | P | D | S | G | H | F | I | P | D | E | Q | P | G | A | L | V | D | A | L |
| 106 UniRef90\_A0A2N3KZL1\_30\_325 | - | - | - | M | G | Q | - | - | - | L | E | A | D | Q | L | G | E | Y | G | - | - | T | H | - | - | V | K | G | L | V | I | P | D | C | G | H | W | L | P | E | E | C | A | K | P | L | N | D | A | V |
| 107 UniRef90\_UPI00076E3DF2\_55\_340 | - | - | - | L | D | T | - | - | - | V | R | A | T | V | D | S | H | I | T | - | - | N | V | - | - | V | R | A | V | D | V | P | N | A | G | H | W | L | I | E | E | N | P | E | Y | V | A | E | E | L |
| 108 UniRef90\_UPI00130E1FA7\_66\_348 | - | - | - | G | N | G | - | - | - | L | L | V | D | F | M | K | A | R | I | - | - | K | T | - | - | P | Q | F | K | P | L | P | D | T | G | H | Y | V | A | E | E | E | P | E | K | V | G | Q | Y | I |
| 109 UniRef90\_A0A1I3FUH2\_46\_328 | - | - | - | G | H | V | - | - | - | I | L | A | D | F | M | E | K | R | H | - | - | V | D | - | - | P | E | L | V | H | L | Q | E | S | G | H | Y | V | A | E | E | A | P | D | E | V | T | R | L | I |
| 110 UniRef90\_UPI000E2836BA\_28\_313 | - | - | - | F | Y | D | - | - | - | Y | M | R | R | A | L | P | A | Q | G | - | - | T | D | - | - | V | E | V | K | E | I | K | N | T | R | N | Y | L | V | E | E | Q | P | E | A | V | T | E | A | L |
| 111 UniRef90\_A0A653WNW3\_25\_297 | - | - | - | F | G | L | - | - | - | T | M | A | V | V | M | R | F | A | A | - | - | T | D | - | - | V | S | E | G | I | V | P | D | S | G | H | W | I | M | E | E | N | P | K | A | T | V | A | A | - |
| 112 UniRef90\_A0A5C1I8U5\_39\_321 | - | - | - | G | Y | D | - | - | - | W | L | R | F | T | L | P | N | K | T | - | - | T | D | - | - | L | K | I | E | K | I | E | N | S | G | H | F | I | A | E | E | Q | P | E | T | V | I | R | D | I |
| 113 UniRef90\_UPI00165059AC\_4\_267 | - | - | - | L | Q | T | - | - | L | D | P | V | A | I | W | R | D | Y | A | - | - | E | D | - | - | V | T | G | T | V | I | E | D | C | G | H | F | L | A | E | E | Q | P | A | R | V | L | E | E | L |
| 114 UniRef90\_F8JLL5\_12\_309 | - | - | - | S | I | R | - | - | - | D | M | A | A | S | I | K | P | W | A | - | - | D | H | - | - | A | T | G | I | V | V | P | D | A | G | H | F | I | P | D | E | Q | P | D | A | V | A | A | A | L |
| 115 UniRef90\_A0A329J6I4\_30\_314 | - | - | - | L | G | Q | - | - | - | F | Q | I | D | Q | T | K | K | Y | A | - | - | T | N | - | - | V | K | G | E | I | L | P | G | C | G | H | W | L | P | E | E | C | A | A | K | L | N | A | V | V |
| 116 UniRef90\_A0A3N1H0W7\_60\_340 | - | - | - | F | A | A | - | - | - | Q | M | R | A | T | L | P | N | Q | A | - | - | T | D | - | - | V | R | V | V | V | V | P | D | S | G | H | Y | L | V | E | E | S | P | Q | T | V | I | D | H | F |
| 117 UniRef90\_A0A316VRQ3\_1\_263 | - | - | - | F | C | K | - | - | - | Y | I | E | Q | I | G | K | E | I | G | - | - | G | N | - | - | M | Q | F | K | P | V | E | D | A | S | H | W | V | P | E | E | N | P | V | G | L | A | N | L | T |
| 118 UniRef90\_A0A4P8X818\_19\_291 | - | - | - | I | A | D | - | - | - | E | V | Q | R | Q | M | E | R | V | A | - | - | S | N | - | - | V | T | G | - | V | I | L | D | Y | G | H | Q | L | A | E | E | C | P | E | D | F | A | H | V | L |
| 119 UniRef90\_UPI0008405A69\_11\_309 | - | - | - | Y | P | Q | - | - | - | L | R | E | Q | M | S | A | D | K | G | - | - | T | D | - | - | I | R | V | V | E | I | K | G | S | G | H | Y | L | P | E | E | Q | P | E | Q | L | L | R | E | L |
| 120 UniRef90\_A0A6I8M538\_3\_275 | - | - | - | L | G | T | - | - | L | P | T | L | E | I | W | Q | E | Y | A | - | - | N | D | - | - | V | T | G | A | A | L | A | E | C | G | H | F | L | P | E | E | Q | P | D | E | V | V | S | H | L |
| 121 UniRef90\_UPI000B5CCBFE\_21\_300 | - | - | - | Q | Y | D | - | - | - | Q | V | Q | D | Q | L | S | R | S | A | - | - | T | D | - | - | V | R | M | V | L | V | E | K | S | M | H | W | L | A | E | D | A | S | D | L | V | A | R | E | L |
| 122 UniRef90\_A0A0M9ZDW3\_1\_266 | - | - | - | S | A | P | - | - | - | L | I | F | P | M | I | E | K | K | A | - | - | A | D | - | - | I | R | T | V | E | L | A | G | A | G | H | F | L | S | D | E | R | P | D | D | L | L | A | V | L |
| 123 UniRef90\_UPI000E275A28\_18\_308 | - | - | - | C | G | E | - | - | - | L | V | E | E | Q | L | R | R | V | A | - | - | T | D | - | - | V | Q | C | T | I | I | P | D | C | G | H | F | I | P | E | E | C | P | E | D | L | L | A | V | L |
| 124 UniRef90\_A0A3L8K0H7\_13\_298 | - | - | - | F | H | D | - | - | - | Y | M | S | Q | V | L | P | T | Q | G | - | - | T | D | - | - | V | R | V | A | K | I | E | N | T | R | N | Y | L | V | E | E | Q | P | E | A | V | A | G | A | L |
| 125 UniRef90\_A0A6N7ZB66\_18\_297 | - | - | - | T | G | D | - | - | - | Q | T | I | K | A | L | A | P | H | A | - | - | E | K | - | - | L | T | S | - | Y | V | A | D | T | S | H | F | V | A | E | E | A | P | E | W | F | V | E | K | L |
| 126 UniRef90\_A0A2W7GM08\_52\_337 | - | - | - | G | F | D | - | - | - | W | L | K | A | T | L | A | P | K | V | - | - | A | N | - | - | L | S | V | V | K | V | - | D | S | G | H | F | I | P | E | E | I | P | D | V | L | L | E | H | L |
| 127 UniRef90\_A0A258JI33\_23\_300 | - | - | - | F | G | A | - | - | - | M | Q | A | T | V | M | R | E | V | G | - | - | S | N | - | - | V | E | G | G | I | I | A | N | S | G | H | W | I | M | E | E | Q | P | A | Q | T | V | D | K | I |
| 128 UniRef90\_A0A6A6C1N5\_26\_297 | - | - | - | Q | R | E | - | - | - | E | A | E | G | M | V | R | E | V | T | - | - | E | D | G | V | V | E | V | G | V | V | E | G | V | G | H | Y | L | A | E | E | G | P | E | E | F | V | E | V | V |
| 129 UniRef90\_UPI00160A92C2\_5\_284 | - | - | - | V | G | G | L | - | Y | D | V | L | A | V | W | Q | G | M | A | - | - | N | D | - | - | V | R | G | L | A | I | P | E | C | G | H | L | P | Q | E | E | Q | P | Q | R | V | N | Q | E | L |
| 130 UniRef90\_UPI0009E804D9\_8\_293 | - | - | - | M | G | T | - | - | - | G | V | A | D | A | L | R | P | H | A | - | - | P | R | - | - | L | A | G | - | L | V | A | P | T | G | H | F | V | V | E | E | D | P | T | W | F | L | Q | A | L |
| 131 UniRef90\_UPI00049086FA\_57\_348 | - | - | - | F | A | S | - | - | - | V | M | L | P | A | V | R | A | A | A | - | - | P | A | - | - | A | T | G | A | D | V | P | G | A | G | H | W | M | L | D | Q | H | P | D | Q | V | V | A | E | I |
| 132 UniRef90\_A5FF96\_16\_286 | - | - | - | G | A | K | - | - | - | M | L | S | Y | F | F | S | V | Y | L | - | - | E | N | - | - | F | S | I | E | E | I | E | N | S | G | H | F | I | H | E | E | K | P | E | L | T | A | L | L | I |
| 133 UniRef90\_UPI0016621A44\_4\_265 | - | - | - | M | G | T | - | - | - | A | V | T | K | C | L | A | Q | L | A | - | - | T | D | - | - | V | Q | D | L | Q | V | A | Q | A | G | H | Y | P | A | E | Q | D | P | A | T | V | N | R | E | L |
| 134 UniRef90\_UPI000B83B944\_2\_264 | - | - | - | T | A | H | - | - | - | T | L | E | E | F | L | K | Q | N | A | - | - | T | D | - | - | M | K | M | V | K | I | N | K | A | G | H | W | I | S | E | Q | S | P | Q | E | T | V | K | L | L |
| 135 UniRef90\_A0A0B4DHQ5\_15\_286 | - | - | - | V | G | K | M | - | F | D | M | P | K | V | W | A | E | M | A | - | - | S | N | - | - | L | K | A | V | P | I | E | R | C | G | H | L | P | H | E | E | R | P | E | E | V | N | A | L | L |
| 136 UniRef90\_UPI0009B31CF7\_18\_283 | - | - | - | V | G | D | - | - | - | A | L | A | H | Q | L | R | P | H | T | - | - | C | T | - | - | L | H | T | Q | R | I | S | G | C | G | H | N | I | P | E | E | Q | P | E | A | L | S | S | A | L |
| 137 UniRef90\_UPI001661B99F\_34\_324 | - | - | - | M | G | E | - | - | - | K | V | G | E | D | L | K | H | V | A | V | R | A | N | - | - | V | T | T | A | V | V | P | N | A | N | H | W | V | T | E | E | N | P | Q | F | V | I | D | Q | L |
| 138 UniRef90\_UPI00104123D8\_9\_274 | - | - | - | G | G | D | I | - | T | R | Y | A | Q | G | F | R | D | A | G | V | - | R | N | - | - | L | R | T | A | L | V | P | D | A | G | H | F | A | Q | E | E | D | P | I | G | V | - | - | - | - |
| 139 UniRef90\_A0A1M7Z6I5\_10\_283 | - | - | - | V | G | K | T | - | F | D | M | A | Q | V | W | A | E | M | A | - | - | D | D | - | - | L | I | A | V | P | V | E | R | C | G | H | L | P | H | E | E | R | P | D | V | V | N | P | L | L |
| 140 UniRef90\_A0A431M1N9\_22\_292 | - | - | - | Y | G | A | - | - | - | G | M | A | D | E | L | G | F | A | A | - | - | T | N | - | - | V | Q | G | A | V | I | T | N | S | G | H | W | I | M | E | E | Q | P | Q | Q | - | - | - | - | - |
| 141 UniRef90\_UPI0013D28676\_49\_329 | - | - | - | M | N | A | - | - | - | L | V | G | K | D | L | G | D | V | A | - | - | T | H | - | - | V | T | T | A | V | V | P | D | A | N | H | W | V | M | E | E | D | P | G | L | V | L | Q | L | L |
| 142 UniRef90\_A0A402BD85\_4\_199 | - | - | - | - | - | - | - | - | - | - | - | - | - | - | - | - | - | - | - | - | - | - | - | - | - | - | - | - | - | - | - | - | - | - | - | - | - | - | - | - | - | - | - | - | - | - | - | - | - | - |
| 143 UniRef90\_A0A2V5S4I0\_55\_307 | - | - | - | C | R | S | - | - | - | C | C | L | E | R | - | - | - | - | - | - | - | - | - | - | - | - | - | - | - | - | - | - | - | - | - | H | R | T | L | D | F | R | - | - | - | - | - | - | - | - |
| 144 UniRef90\_A0A328AN40\_21\_303 | - | - | - | F | A | E | - | - | A | K | L | A | E | E | S | A | A | L | C | - | - | A | S | - | - | A | E | V | V | H | F | P | H | A | S | H | W | L | P | H | D | E | P | D | G | V | S | Q | R | L |
| 145 UniRef90\_A0A0M9AP20\_25\_244 | - | - | - | - | - | - | - | - | - | - | - | - | - | - | - | - | - | - | - | - | - | - | - | - | - | - | - | - | - | - | - | - | - | - | - | - | - | - | - | - | - | - | - | - | - | - | - | - | - | - |
| 146 UniRef90\_A0A1N6RLL9\_1\_202 | - | - | - | A | G | P | T | L | L | K | M | A | E | S | L | R | K | Y | G | C | - | T | N | - | - | V | T | T | K | I | I | Q | N | S | G | H | F | V | S | E | E | Q | P | E | I | V | I | G | L | I |
| 147 UniRef90\_A0A4R3LTR2\_48\_312 | - | - | - | V | G | D | L | - | Y | D | V | L | E | T | W | R | E | K | A | - | - | R | D | - | - | V | R | G | H | A | L | - | D | C | G | H | L | V | Q | E | E | R | P | E | E | T | L | A | A | L |
| 148 UniRef90\_UPI0002ED95C2\_14\_285 | - | - | V | I | L | P | - | - | F | D | P | L | E | I | W | S | T | W | A | - | - | P | N | - | - | L | R | T | A | T | V | - | N | C | G | H | F | L | P | E | E | Q | P | G | V | V | - | - | - | - |
| 149 UniRef90\_A0A1Q7MBV0\_16\_212 | - | - | - | - | - | - | - | - | - | - | - | - | - | - | - | - | - | - | - | - | - | - | - | - | - | - | - | - | - | - | - | - | - | - | - | - | - | - | - | - | - | - | - | - | - | - | - | - | - | - |
| 150 UniRef90\_A0A1A0KI05\_7\_212 | - | - | - | - | - | - | - | - | - | - | - | - | - | - | - | - | - | - | - | - | - | - | - | - | - | - | - | - | - | - | - | - | - | - | - | - | - | - | - | - | - | - | - | - | - | - | - | - | - | - |

  
  

|  |  |  |  |  |  |  |  |  |  |  |  |
| --- | --- | --- | --- | --- | --- | --- | --- | --- | --- | --- | --- |
| **001 Input\_protein\_seq** | L | D | F | F | R | E | A | A | S | G | R |
| 002 UniRef90\_UPI00158A2D6D\_3\_306 | I | A | F | F | R | E | A | V | P | G | R |
| 003 UniRef90\_A0A1Z4J856\_27\_318 | L | N | F | F | G | E | - | - | - | - | - |
| 004 UniRef90\_G7LVZ3\_7\_288 | V | E | F | F | T | T | T | S | A | G | - |
| 005 UniRef90\_A0A2I8F4B9\_53\_324 | L | R | F | L | - | - | - | - | - | - | - |
| 006 UniRef90\_A0A327RPK7\_10\_284 | T | E | F | L | - | - | - | - | - | - | - |
| 007 UniRef90\_A0A6L3SWG4\_20\_294 | R | S | F | L | S | E | - | - | - | - | - |
| 008 UniRef90\_A0A401ZLI5\_4\_287 | N | A | F | F | S | E | - | - | - | - | - |
| 009 UniRef90\_A0A4V2U6R1\_12\_297 | R | E | F | V | S | E | T | - | - | - | - |
| 010 UniRef90\_A0A5A5T922\_6\_282 | L | E | F | F | A | E | - | - | - | - | - |
| 011 UniRef90\_A0A2V6UIN9\_3\_273 | V | A | F | F | - | - | - | - | - | - | - |
| 012 UniRef90\_UPI001669B3C5\_41\_314 | K | D | F | I | N | - | - | - | - | - | - |
| 013 UniRef90\_A0A4R2Z7A8\_3\_305 | H | T | F | F | - | - | - | - | - | - | - |
| 014 UniRef90\_A0A5C5SVV1\_11\_281 | L | A | F | L | R | G | - | - | - | - | - |
| 015 UniRef90\_L9WLS3\_16\_284 | E | S | F | L | E | S | A | - | - | - | - |
| 016 UniRef90\_UPI00131EC7B5\_30\_311 | S | G | F | F | - | - | - | - | - | - | - |
| 017 UniRef90\_C7QAM5\_50\_332 | L | D | F | L | - | - | - | - | - | - | - |
| 018 UniRef90\_A0A1Q3SXP6\_11\_288 | V | S | F | L | T | - | - | - | - | - | - |
| 019 UniRef90\_UPI00149248BF\_39\_315 | K | S | F | L | - | - | - | - | - | - | - |
| 020 UniRef90\_UPI000361D127\_26\_313 | R | P | F | - | - | - | - | - | - | - | - |
| 021 UniRef90\_A0A0M4FVH2\_24\_312 | K | A | F | F | - | - | - | - | - | - | - |
| 022 UniRef90\_A0A1Q4ZL08\_9\_290 | L | E | F | L | Q | P | - | - | - | - | - |
| 023 UniRef90\_S3CY91\_25\_307 | M | E | F | V | - | - | - | - | - | - | - |
| 024 UniRef90\_UPI0010F95BB2\_18\_294 | V | S | F | F | R | N | S | - | - | - | - |
| 025 UniRef90\_A0A0N1GDE2\_28\_316 | E | D | F | L | - | - | - | - | - | - | - |
| 026 UniRef90\_A0A2E5PJQ7\_5\_284 | L | E | F | F | K | D | - | - | - | - | - |
| 027 UniRef90\_A0A1M7IC97\_44\_328 | L | D | F | L | - | - | - | - | - | - | - |
| 028 UniRef90\_UPI0016149F5E\_5\_284 | L | P | F | L | A | - | - | - | - | - | - |
| 029 UniRef90\_UPI00156E1DFF\_8\_289 | L | H | F | L | - | - | - | - | - | - | - |
| 030 UniRef90\_A0A3A8HRU0\_15\_300 | G | D | F | L | R | - | - | - | - | - | - |
| 031 UniRef90\_A0A0M3UDU6\_18\_309 | V | D | F | L | K | - | - | - | - | - | - |
| 032 UniRef90\_A0A447J1A9\_28\_317 | L | P | F | L | R | - | - | - | - | - | - |
| 033 UniRef90\_A0A5C8T429\_14\_295 | I | N | F | L | - | - | - | - | - | - | - |
| 034 UniRef90\_A0A0N0TCD7\_1\_288 | L | D | F | L | R | - | - | - | - | - | - |
| 035 UniRef90\_W9ARP2\_16\_295 | C | E | F | D | A | - | - | - | - | - | - |
| 036 UniRef90\_A0A109IGY4\_11\_301 | A | D | F | L | - | - | - | - | - | - | - |
| 037 UniRef90\_K9DQK6\_24\_306 | D | D | F | L | T | A | - | - | - | - | - |
| 038 UniRef90\_A0A1Q8IR73\_6\_294 | A | R | F | F | A | - | - | - | - | - | - |
| 039 UniRef90\_A0A1Y6D2S0\_29\_302 | S | S | F | L | A | P | A | - | - | - | - |
| 040 UniRef90\_E6WJ64\_19\_301 | A | R | F | F | R | - | - | - | - | - | - |
| 041 UniRef90\_UPI000489A94A\_43\_316 | A | S | F | L | - | - | - | - | - | - | - |
| 042 UniRef90\_A0A4R6HBQ4\_11\_285 | V | S | F | F | A | - | - | - | - | - | - |
| 043 UniRef90\_A0A1M7QJX9\_32\_311 | L | D | F | F | L | - | - | - | - | - | - |
| 044 UniRef90\_A0A5B8WA72\_22\_300 | L | A | F | L | - | - | - | - | - | - | - |
| 045 UniRef90\_A0A0G2FGE6\_47\_303 | L | A | - | - | - | - | - | - | - | - | - |
| 046 UniRef90\_A0A1I6BK09\_10\_296 | L | D | F | F | G | E | A | D | - | - | - |
| 047 UniRef90\_A0A4R7C9R7\_33\_310 | - | - | - | - | - | - | - | - | - | - | - |
| 048 UniRef90\_A0A2M9M9Q1\_18\_305 | L | P | F | L | - | - | - | - | - | - | - |
| 049 UniRef90\_A0A4V3T343\_11\_295 | L | R | F | L | - | - | - | - | - | - | - |
| 050 UniRef90\_UPI001616534D\_11\_303 | T | - | - | - | - | - | - | - | - | - | - |
| 051 UniRef90\_J4PG95\_17\_290 | L | A | F | L | - | - | - | - | - | - | - |
| 052 UniRef90\_A0A0T1T741\_1\_295 | E | D | F | L | - | - | - | - | - | - | - |
| 053 UniRef90\_G0FSK7\_17\_310 | S | E | F | L | A | P | W | A | D | - | - |
| 054 UniRef90\_UPI00164AAB2D\_18\_303 | L | T | F | I | R | - | - | - | - | - | - |
| 055 UniRef90\_A0A1I2MT44\_20\_306 | R | P | F | - | - | - | - | - | - | - | - |
| 056 UniRef90\_A0A2P2CCU5\_19\_306 | T | T | F | L | - | - | - | - | - | - | - |
| 057 UniRef90\_A0A2V9H5C8\_12\_298 | V | K | F | - | - | - | - | - | - | - | - |
| 058 UniRef90\_A0A0P4V0Y0\_7\_291 | F | T | F | F | Q | S | V | - | - | - | - |
| 059 UniRef90\_A0A0Q4UQ58\_27\_305 | - | - | - | - | - | - | - | - | - | - | - |
| 060 UniRef90\_A0A6M4IM28\_20\_309 | R | K | F | I | G | D | A | - | - | - | - |
| 061 UniRef90\_A0A537J419\_20\_301 | L | K | F | - | - | - | - | - | - | - | - |
| 062 UniRef90\_A0A1V2PGN8\_6\_275 | I | R | F | - | - | - | - | - | - | - | - |
| 063 UniRef90\_A0A542JC81\_20\_314 | I | S | F | L | E | P | F | R | A | A | A |
| 064 UniRef90\_UPI000424F353\_33\_308 | T | D | F | L | - | - | - | - | - | - | - |
| 065 UniRef90\_A0A2V9W2E9\_22\_299 | R | N | F | - | - | - | - | - | - | - | - |
| 066 UniRef90\_A0A2T6L0M5\_16\_299 | R | T | F | L | R | - | - | - | - | - | - |
| 067 UniRef90\_A0A261TYY2\_43\_315 | R | A | F | - | - | - | - | - | - | - | - |
| 068 UniRef90\_UPI0009781B11\_10\_287 | L | P | F | L | S | E | - | - | - | - | - |
| 069 UniRef90\_A0A2V9TPC1\_35\_307 | Q | K | F | - | - | - | - | - | - | - | - |
| 070 UniRef90\_UPI00145FA88A\_6\_279 | Q | G | F | - | - | - | - | - | - | - | - |
| 071 UniRef90\_UPI0003FADEBE\_21\_306 | Q | E | F | L | - | - | - | - | - | - | - |
| 072 UniRef90\_A0A2V8Y3P4\_78\_329 | - | - | - | - | - | - | - | - | - | - | - |
| 073 UniRef90\_A0A285BKA7\_8\_299 | L | D | F | L | R | - | - | - | - | - | - |
| 074 UniRef90\_A0A0K3AVW3\_44\_319 | V | D | F | L | R | - | - | - | - | - | - |
| 075 UniRef90\_A0A4D7B7N1\_7\_276 | L | A | F | F | G | E | - | - | - | - | - |
| 076 UniRef90\_A0A2U0WF37\_17\_293 | - | - | - | - | - | - | - | - | - | - | - |
| 077 UniRef90\_UPI00135C2D49\_17\_309 | E | D | F | L | R | - | - | - | - | - | - |
| 078 UniRef90\_UPI001473614E\_45\_322 | - | - | - | - | - | - | - | - | - | - | - |
| 079 UniRef90\_J3F862\_22\_299 | R | R | F | - | - | - | - | - | - | - | - |
| 080 UniRef90\_A0A1A9NC01\_62\_345 | L | D | F | L | Q | - | - | - | - | - | - |
| 081 UniRef90\_UPI00131AC11B\_45\_327 | N | D | F | L | - | - | - | - | - | - | - |
| 082 UniRef90\_A0A4R8HC20\_22\_306 | T | R | F | - | - | - | - | - | - | - | - |
| 083 UniRef90\_A0A1B2HH26\_4\_280 | T | H | F | F | R | - | - | - | - | - | - |
| 084 UniRef90\_A0A517LKB1\_23\_304 | L | K | F | V | - | - | - | - | - | - | - |
| 085 UniRef90\_A0A0N1F3N8\_17\_298 | R | - | - | - | - | - | - | - | - | - | - |
| 086 UniRef90\_UPI0004CCFD44\_55\_340 | L | R | F | L | - | - | - | - | - | - | - |
| 087 UniRef90\_A0A4D4LDP7\_7\_304 | L | R | F | A | - | - | - | - | - | - | - |
| 088 UniRef90\_A0A2V4B948\_8\_279 | R | A | F | - | - | - | - | - | - | - | - |
| 089 UniRef90\_A0A2P2FV90\_3\_282 | K | P | F | - | - | - | - | - | - | - | - |
| 090 UniRef90\_A0A4Q2J292\_17\_304 | A | D | F | L | - | - | - | - | - | - | - |
| 091 UniRef90\_UPI0012B05B7D\_16\_287 | I | E | F | D | R | - | - | - | - | - | - |
| 092 UniRef90\_UPI00048D4AD4\_4\_301 | T | S | F | - | - | - | - | - | - | - | - |
| 093 UniRef90\_UPI0015F81933\_16\_310 | T | D | F | I | T | E | - | - | - | - | - |
| 094 UniRef90\_UPI00160CFF45\_55\_340 | L | R | F | L | - | - | - | - | - | - | - |
| 095 UniRef90\_A0A1Q8KTW7\_17\_294 | G | E | F | F | A | D | - | - | - | - | - |
| 096 UniRef90\_UPI00161A7EC4\_21\_297 | - | - | - | - | - | - | - | - | - | - | - |
| 097 UniRef90\_A0A1B4WXA8\_16\_299 | R | N | F | - | - | - | - | - | - | - | - |
| 098 UniRef90\_UPI000E46D2C1\_48\_330 | R | A | F | L | - | - | - | - | - | - | - |
| 099 UniRef90\_A0A2N5CFI4\_23\_293 | - | - | - | - | - | - | - | - | - | - | - |
| 100 UniRef90\_A0A385B2U0\_45\_333 | L | K | F | L | R | - | - | - | - | - | - |
| 101 UniRef90\_A0A1Q7W147\_16\_308 | A | D | F | V | - | - | - | - | - | - | - |
| 102 UniRef90\_A0A239MTD5\_1\_271 | Q | S | F | L | Q | E | - | - | - | - | - |
| 103 UniRef90\_UPI000561A6EF\_30\_315 | - | - | - | - | - | - | - | - | - | - | - |
| 104 UniRef90\_A0A4Y8RHA3\_28\_294 | - | - | - | - | - | - | - | - | - | - | - |
| 105 UniRef90\_UPI0004DF415F\_15\_308 | T | A | F | I | - | - | - | - | - | - | - |
| 106 UniRef90\_A0A2N3KZL1\_30\_325 | L | D | F | L | - | - | - | - | - | - | - |
| 107 UniRef90\_UPI00076E3DF2\_55\_340 | L | L | F | L | - | - | - | - | - | - | - |
| 108 UniRef90\_UPI00130E1FA7\_66\_348 | T | E | F | L | - | - | - | - | - | - | - |
| 109 UniRef90\_A0A1I3FUH2\_46\_328 | L | G | F | I | - | - | - | - | - | - | - |
| 110 UniRef90\_UPI000E2836BA\_28\_313 | L | D | F | F | - | - | - | - | - | - | - |
| 111 UniRef90\_A0A653WNW3\_25\_297 | - | - | - | - | - | - | - | - | - | - | - |
| 112 UniRef90\_A0A5C1I8U5\_39\_321 | L | Q | F | L | - | - | - | - | - | - | - |
| 113 UniRef90\_UPI00165059AC\_4\_267 | - | - | - | - | - | - | - | - | - | - | - |
| 114 UniRef90\_F8JLL5\_12\_309 | T | D | F | V | T | E | G | - | - | - | - |
| 115 UniRef90\_A0A329J6I4\_30\_314 | V | D | F | L | - | - | - | - | - | - | - |
| 116 UniRef90\_A0A3N1H0W7\_60\_340 | T | E | F | F | - | - | - | - | - | - | - |
| 117 UniRef90\_A0A316VRQ3\_1\_263 | V | K | F | L | Q | E | - | - | - | - | - |
| 118 UniRef90\_A0A4P8X818\_19\_291 | D | E | F | F | - | - | - | - | - | - | - |
| 119 UniRef90\_UPI0008405A69\_11\_309 | F | S | F | F | A | P | S | A | T | - | - |
| 120 UniRef90\_A0A6I8M538\_3\_275 | R | K | F | - | - | - | - | - | - | - | - |
| 121 UniRef90\_UPI000B5CCBFE\_21\_300 | L | D | F | L | - | - | - | - | - | - | - |
| 122 UniRef90\_A0A0M9ZDW3\_1\_266 | E | D | F | F | - | - | - | - | - | - | - |
| 123 UniRef90\_UPI000E275A28\_18\_308 | K | P | F | F | E | - | - | - | - | - | - |
| 124 UniRef90\_A0A3L8K0H7\_13\_298 | L | E | F | F | - | - | - | - | - | - | - |
| 125 UniRef90\_A0A6N7ZB66\_18\_297 | T | D | F | L | A | N | A | G | - | - | - |
| 126 UniRef90\_A0A2W7GM08\_52\_337 | G | A | F | L | A | Q | - | - | - | - | - |
| 127 UniRef90\_A0A258JI33\_23\_300 | - | - | - | - | - | - | - | - | - | - | - |
| 128 UniRef90\_A0A6A6C1N5\_26\_297 | L | E | F | L | - | - | - | - | - | - | - |
| 129 UniRef90\_UPI00160A92C2\_5\_284 | R | E | F | L | R | - | - | - | - | - | - |
| 130 UniRef90\_UPI0009E804D9\_8\_293 | D | G | F | L | - | - | - | - | - | - | - |
| 131 UniRef90\_UPI00049086FA\_57\_348 | N | A | F | Y | P | A | A | A | - | - | - |
| 132 UniRef90\_A5FF96\_16\_286 | - | - | - | - | - | - | - | - | - | - | - |
| 133 UniRef90\_UPI0016621A44\_4\_265 | T | S | F | L | - | - | - | - | - | - | - |
| 134 UniRef90\_UPI000B83B944\_2\_264 | T | E | F | F | - | - | - | - | - | - | - |
| 135 UniRef90\_A0A0B4DHQ5\_15\_286 | L | E | F | L | Q | - | - | - | - | - | - |
| 136 UniRef90\_UPI0009B31CF7\_18\_283 | L | A | F | F | A | E | S | - | - | - | - |
| 137 UniRef90\_UPI001661B99F\_34\_324 | R | Q | - | - | - | - | - | - | - | - | - |
| 138 UniRef90\_UPI00104123D8\_9\_274 | - | - | - | - | - | - | - | - | - | - | - |
| 139 UniRef90\_A0A1M7Z6I5\_10\_283 | L | D | F | L | E | - | - | - | - | - | - |
| 140 UniRef90\_A0A431M1N9\_22\_292 | - | - | - | - | - | - | - | - | - | - | - |
| 141 UniRef90\_UPI0013D28676\_49\_329 | S | T | F | F | - | - | - | - | - | - | - |
| 142 UniRef90\_A0A402BD85\_4\_199 | - | - | - | - | - | - | - | - | - | - | - |
| 143 UniRef90\_A0A2V5S4I0\_55\_307 | - | - | - | - | - | - | - | - | - | - | - |
| 144 UniRef90\_A0A328AN40\_21\_303 | L | A | F | L | R | - | - | - | - | - | - |
| 145 UniRef90\_A0A0M9AP20\_25\_244 | - | - | - | - | - | - | - | - | - | - | - |
| 146 UniRef90\_A0A1N6RLL9\_1\_202 | - | - | - | - | - | - | - | - | - | - | - |
| 147 UniRef90\_A0A4R3LTR2\_48\_312 | L | A | F | - | - | - | - | - | - | - | - |
| 148 UniRef90\_UPI0002ED95C2\_14\_285 | - | - | - | - | - | - | - | - | - | - | - |
| 149 UniRef90\_A0A1Q7MBV0\_16\_212 | - | - | - | - | - | - | - | - | - | - | - |
| 150 UniRef90\_A0A1A0KI05\_7\_212 | - | - | - | - | - | - | - | - | - | - | - |

  
  

|  |  |  |  |  |  |  |  |  |  |  |  |  |  |  |  |  |  |
| --- | --- | --- | --- | --- | --- | --- | --- | --- | --- | --- | --- | --- | --- | --- | --- | --- | --- |
| |  |  |  |  |  |  |  |  |  | | --- | --- | --- | --- | --- | --- | --- | --- | --- | | 1 | 2 | 3 | 4 | 5 | 6 | 7 | 8 | 9 |   |  |  |  |  |  |  | | --- | --- | --- | --- | --- | --- | | **Variable** |  | **Average** |  | **Conserved** | | | |  |  |  | | --- | --- | | **X | - Insufficient data - the calculation for this site was performed on less than 10% of the sequences. |** |
