# Supplementary material for: Biochemical and structural characterization of two cif-like epoxide hydrolases from Burkholderia cenocepacia
Source: Curr Res Struct Biol. 2021 Feb 21;3:72–84. doi: 10.1016/j.crstbi.2021.02.002 (PMC8244358; doi:10.1016/j.crstbi.2021.02.002)
Supplement: Multimedia component 6 [file mmc6.zip › Cfl2_MSA.html]

ConSurf Color-Coded MSA

# ConSurf Color-Coded MSA

|  |  |  |  |  |  |  |  |  |  |  |  |  |  |  |  |  |  |  |  |  |  |  |  |  |  |  |  |  |  |  |  |  |  |  |  |  |  |  |  |  |  |  |  |  |  |  |  |  |  |  |
| --- | --- | --- | --- | --- | --- | --- | --- | --- | --- | --- | --- | --- | --- | --- | --- | --- | --- | --- | --- | --- | --- | --- | --- | --- | --- | --- | --- | --- | --- | --- | --- | --- | --- | --- | --- | --- | --- | --- | --- | --- | --- | --- | --- | --- | --- | --- | --- | --- | --- | --- |
| **001 Input\_protein\_seq** | M | Y | Q | H | Q | S | T | E | A | A | S | H | L | E | A | T | P | - | Y | F | R | E | D | P | R | L | T | G | F | R | H | R | F | D | T | V | D | G | V | R | L | H | F | V | E | G | G | - | - | - |
| 002 UniRef90\_UPI001430CB85\_4\_307 | - | - | - | - | Q | S | S | T | A | P | K | I | V | D | F | G | P | - | V | S | V | P | D | L | N | L | S | G | F | E | H | R | F | E | T | I | D | G | I | R | L | H | Y | V | T | G | G | - | - | - |
| 003 UniRef90\_UPI00055F8D66\_10\_306 | - | - | - | - | - | - | - | - | - | - | - | T | I | E | A | P | P | - | S | S | R | P | E | R | E | L | A | G | F | K | H | L | F | Q | T | V | D | G | M | R | L | H | Y | V | A | G | G | - | - | - |
| 004 UniRef90\_UPI00161D8BA7\_15\_312 | - | - | - | - | - | - | - | - | - | - | R | V | V | D | T | L | P | - | V | S | L | P | E | T | S | L | A | G | F | E | H | R | Y | E | T | V | G | G | T | R | L | H | Y | V | E | G | G | - | - | - |
| 005 UniRef90\_A0A4R7GLU7\_7\_305 | - | - | - | - | - | - | - | - | - | S | S | V | V | S | A | D | P | - | F | A | V | A | D | P | S | L | P | G | F | T | H | R | Y | A | S | V | E | G | V | R | L | H | Y | V | S | G | G | - | - | - |
| 006 UniRef90\_UPI00041CD164\_24\_309 | - | - | - | - | - | - | - | - | - | - | - | - | - | - | - | - | - | - | - | - | - | - | - | P | L | P | S | G | F | E | H | R | F | A | T | V | D | G | V | G | L | H | Y | V | A | G | G | - | - | - |
| 007 UniRef90\_I0QSF8\_15\_298 | - | - | - | - | - | - | - | - | - | - | - | - | - | - | - | - | - | - | - | - | - | - | - | - | - | - | K | G | F | S | H | Y | Y | R | L | V | D | G | V | R | I | H | Y | V | A | G | G | - | - | - |
| 008 UniRef90\_A0A2N5ENH7\_9\_301 | - | - | - | - | - | - | - | - | - | - | - | - | - | - | - | - | P | - | W | S | Q | P | V | A | G | L | P | G | F | A | H | Y | Y | A | T | V | E | G | V | R | L | H | Y | V | R | G | G | - | - | - |
| 009 UniRef90\_A0A0Q8E2M6\_29\_312 | - | - | - | - | - | - | - | - | - | - | - | - | - | - | - | - | - | - | - | - | - | - | - | - | - | - | P | D | F | T | H | R | F | A | N | V | D | G | V | R | L | H | Y | V | R | G | G | - | - | - |
| 010 UniRef90\_A0A0F5XY46\_6\_287 | - | - | - | - | - | - | - | - | - | - | - | - | - | - | - | - | - | - | - | - | - | - | - | - | - | - | - | H | Y | Q | H | H | F | A | S | V | D | Q | L | R | L | H | Y | L | S | A | G | - | - | - |
| 011 UniRef90\_A0A3N4NUX5\_4\_284 | - | - | - | - | - | - | - | - | - | - | - | - | - | - | - | - | - | - | - | - | - | - | - | - | - | - | - | - | I | E | H | H | F | A | Q | V | N | G | L | R | I | H | Y | L | S | A | G | - | - | - |
| 012 UniRef90\_UPI000DDED2D9\_11\_293 | - | - | - | - | - | - | - | - | - | - | - | - | - | - | - | - | - | - | - | - | - | - | - | - | - | I | D | G | F | T | R | G | F | L | T | V | D | G | V | R | I | H | Y | V | S | G | G | - | - | - |
| 013 UniRef90\_UPI000DDED53B\_10\_292 | - | - | - | - | - | - | - | - | - | - | - | - | - | - | - | - | - | - | - | - | - | - | - | - | - | - | Q | G | F | M | H | R | F | A | S | P | K | G | I | R | L | H | Y | V | E | G | G | - | - | - |
| 014 UniRef90\_A0A1G4JKM0\_6\_288 | - | - | - | - | - | - | - | - | - | - | - | - | - | - | - | - | - | - | - | - | - | - | - | - | - | - | - | - | L | T | H | K | F | T | L | V | A | G | T | R | I | H | Y | V | T | A | G | - | - | L |
| 015 UniRef90\_UPI0016728815\_21\_305 | - | - | - | - | - | - | - | - | - | - | - | - | - | - | - | - | - | - | - | - | - | - | - | P | D | L | A | G | F | T | H | R | W | A | D | A | D | G | V | R | L | H | A | V | E | G | G | - | - | - |
| 016 UniRef90\_A0A3N2H8D2\_14\_297 | - | - | - | - | - | - | - | - | - | - | - | - | - | - | - | - | - | - | - | - | - | - | - | - | H | L | T | G | F | T | H | R | W | V | D | A | D | G | V | R | L | H | A | V | E | G | G | - | - | - |
| 017 UniRef90\_UPI0005644DD1\_9\_307 | - | - | - | - | - | - | - | - | - | Q | H | V | S | E | A | H | P | V | R | D | L | P | L | H | D | L | D | G | F | T | H | R | W | V | D | A | D | G | I | R | L | H | A | V | E | G | G | - | - | - |
| 018 UniRef90\_A0A6B2VDS0\_18\_308 | - | - | - | - | - | - | - | - | - | - | - | - | - | - | - | - | - | - | R | D | L | P | L | Q | H | L | A | G | F | T | H | R | W | V | D | A | D | G | V | R | L | H | A | V | E | G | G | - | - | - |
| 019 UniRef90\_A0A6G3RZZ1\_25\_307 | - | - | - | - | - | - | - | - | - | - | - | - | - | - | - | - | - | - | - | - | - | - | - | - | - | L | A | G | F | T | H | R | W | V | D | A | D | G | I | R | L | H | T | V | R | G | G | - | - | - |
| 020 UniRef90\_A0A209C7X3\_21\_305 | - | - | - | - | - | - | - | - | - | - | - | - | - | - | - | - | - | - | - | - | - | - | - | P | G | L | A | G | F | T | H | R | W | V | D | A | G | G | V | G | L | H | A | V | E | G | G | - | - | - |
| 021 UniRef90\_UPI001269E708\_26\_309 | - | - | - | - | - | - | - | - | - | - | - | - | - | - | - | - | - | - | - | - | - | - | - | - | D | L | A | G | F | T | H | R | W | V | D | A | D | G | V | R | L | H | A | V | E | G | G | - | - | - |
| 022 UniRef90\_A0A2M9J7W9\_25\_306 | - | - | - | - | - | - | - | - | - | - | - | - | - | - | - | - | - | - | - | - | - | - | - | - | - | L | A | G | F | T | H | R | W | V | D | A | E | G | I | R | L | H | A | V | E | G | G | - | - | - |
| 023 UniRef90\_A0A6G9F3J8\_23\_307 | - | - | - | - | - | - | - | - | - | - | - | - | - | - | - | - | - | - | - | - | - | - | - | - | - | L | A | G | F | A | H | R | W | V | D | A | G | G | T | R | L | H | A | V | E | G | G | - | - | - |
| 024 UniRef90\_A0A1H2UZ41\_14\_292 | - | - | - | - | - | - | - | - | - | - | - | - | - | - | - | - | - | - | - | - | - | - | - | - | - | - | - | - | F | T | H | S | M | V | E | A | E | G | I | R | F | H | T | V | S | G | G | - | - | - |
| 025 UniRef90\_UPI0012B05B7D\_16\_286 | - | - | - | - | - | - | - | - | - | - | - | - | - | - | - | - | - | - | - | - | - | - | - | - | - | - | - | - | - | - | - | - | - | - | - | F | D | N | L | R | L | H | Y | L | E | S | G | - | - | - |
| 026 UniRef90\_A0A5C8T429\_18\_294 | - | - | - | - | - | - | - | - | - | - | - | - | - | - | - | - | - | - | - | - | - | - | - | - | - | - | - | - | F | Q | H | R | V | Q | G | A | V | G | N | R | L | H | Y | V | T | G | G | - | - | - |
| 027 UniRef90\_UPI0012E86E3E\_10\_309 | - | - | - | - | - | - | - | - | - | - | - | V | A | D | T | V | P | - | P | F | D | P | E | T | F | W | T | L | F | H | H | A | T | V | A | V | N | G | V | R | L | H | F | V | E | G | G | - | - | - |
| 028 UniRef90\_A0A1A9HTL2\_13\_300 | - | - | - | - | - | - | - | - | - | - | - | - | - | - | - | - | - | - | - | - | - | - | - | - | - | - | - | - | - | H | H | G | A | V | V | V | N | G | V | K | L | H | Y | V | E | G | G | - | - | E |
| 029 UniRef90\_A0A0R1V0C7\_7\_281 | - | - | - | - | - | - | - | - | - | - | - | - | - | - | - | - | - | - | - | - | - | - | - | - | - | - | - | - | - | - | - | - | - | A | T | K | D | N | I | T | W | H | I | Y | E | D | G | - | - | - |
| 030 UniRef90\_A0A3N1M9Y1\_5\_282 | - | - | - | - | - | - | - | - | - | - | - | - | - | - | - | - | - | - | - | - | - | - | - | - | - | - | - | - | - | E | H | H | F | A | R | L | S | Q | V | A | M | H | Y | V | T | A | G | - | - | - |
| 031 UniRef90\_A0A158JUX7\_17\_294 | - | - | - | - | - | - | - | - | - | - | - | - | - | - | - | - | - | - | - | - | - | - | - | - | - | - | - | - | - | H | H | E | R | R | W | V | N | G | V | R | I | H | F | V | T | A | G | - | - | - |
| 032 UniRef90\_A0A0U3LIL8\_12\_296 | - | - | - | - | - | - | - | - | - | - | - | - | - | - | - | - | - | - | - | - | - | - | - | - | - | - | - | - | L | H | H | K | L | I | D | V | D | G | I | R | L | H | Y | V | E | A | G | - | - | - |
| 033 UniRef90\_A0A248JS33\_7\_301 | - | - | - | - | - | - | - | - | - | - | - | - | - | - | - | - | - | - | - | - | - | - | D | R | F | W | Q | R | F | R | H | G | L | V | T | A | N | G | V | R | L | H | Y | V | E | G | G | P | E | G |
| 034 UniRef90\_A0A1Y0REZ4\_12\_287 | - | - | - | - | - | - | - | - | - | - | - | - | - | - | - | - | - | - | - | - | - | - | - | - | - | - | - | - | F | S | H | H | T | A | F | V | N | G | I | R | Q | H | Y | V | I | G | G | - | - | - |
| 035 UniRef90\_A0A0M2WRU5\_3\_280 | - | - | - | - | - | - | - | - | - | - | - | - | - | - | - | - | - | - | - | - | - | - | - | - | - | - | - | E | F | T | H | D | Y | V | R | I | D | G | Q | R | L | H | C | V | I | A | G | - | - | - |
| 036 UniRef90\_A0A0N8GFM1\_6\_279 | - | - | - | - | - | - | - | - | - | - | - | - | - | - | - | - | - | - | - | - | - | - | - | - | - | - | - | - | - | - | S | H | F | V | D | L | D | G | V | S | L | H | Y | L | T | A | G | - | - | - |
| 037 UniRef90\_A0A252EMP1\_7\_277 | - | - | - | - | - | - | - | - | - | - | - | - | - | - | - | - | - | - | - | - | - | - | - | - | - | - | - | - | - | T | H | A | I | A | Q | I | G | D | V | H | L | H | Y | V | R | A | G | - | - | - |
| 038 UniRef90\_W0A6K5\_40\_320 | - | - | - | - | - | - | - | - | - | - | - | - | - | - | - | - | - | - | - | - | - | - | - | - | N | E | T | D | F | Q | H | H | Y | A | K | V | A | G | Q | K | I | H | Y | V | T | A | G | - | - | - |
| 039 UniRef90\_A0A3S1SM66\_23\_304 | - | - | - | - | - | - | - | - | - | - | - | - | - | - | - | - | - | - | - | - | - | - | - | - | - | - | - | - | - | - | H | G | T | V | I | A | N | G | L | H | F | H | Y | V | E | G | G | - | - | - |
| 040 UniRef90\_A0A2E5L5C6\_7\_286 | - | - | - | - | - | - | - | - | - | - | - | - | - | - | - | - | - | - | - | - | - | - | - | - | - | - | - | - | V | K | H | N | F | A | D | L | G | D | V | L | I | H | Y | V | M | A | G | - | - | - |
| 041 UniRef90\_M5D1B4\_14\_289 | - | - | - | - | - | - | - | - | - | - | - | - | - | - | - | - | - | - | - | - | - | - | - | - | - | - | - | S | L | R | H | H | Y | L | R | I | D | G | Q | R | V | H | C | V | S | T | G | - | - | - |
| 042 UniRef90\_UPI0016845BA7\_31\_303 | - | - | - | - | - | - | - | - | - | - | - | - | - | - | - | - | - | - | - | - | - | - | - | - | - | - | - | T | F | T | H | H | T | A | D | V | N | G | V | R | L | H | Y | V | M | G | G | - | - | - |
| 043 UniRef90\_UPI0014648199\_14\_291 | - | - | - | - | - | - | - | - | - | - | - | - | - | - | - | - | - | - | - | - | - | - | - | - | - | - | - | E | F | T | H | H | Y | V | R | L | D | G | K | K | M | H | C | V | M | A | G | - | - | - |
| 044 UniRef90\_K9RUM7\_12\_289 | - | - | - | - | - | - | - | - | - | - | - | - | - | - | - | - | - | - | - | - | - | - | - | - | - | - | P | G | F | E | H | T | F | T | T | V | G | D | V | T | L | H | A | I | E | G | G | - | - | - |
| 045 UniRef90\_A0A1Q2U086\_13\_290 | - | - | - | - | - | - | - | - | - | - | - | - | - | - | - | - | - | - | - | - | - | - | - | - | - | - | P | G | F | E | H | T | Y | S | T | V | D | G | V | T | L | H | A | I | E | G | G | - | - | - |
| 046 UniRef90\_B9JMN1\_3\_282 | - | - | - | - | - | - | - | - | - | - | - | - | - | - | - | - | - | - | - | - | - | - | - | - | - | - | T | S | F | E | H | H | T | V | R | V | K | D | I | R | M | H | Y | V | R | A | G | - | - | - |
| 047 UniRef90\_A0A2X1TAZ7\_33\_319 | - | - | - | - | - | - | - | - | - | - | - | - | - | - | - | - | - | - | T | P | A | I | Q | A | N | E | A | D | F | Q | H | H | Y | T | D | V | A | G | Q | R | I | H | Y | V | T | A | G | - | - | - |
| 048 UniRef90\_UPI00131A8152\_6\_279 | - | - | - | - | - | - | - | - | - | - | - | - | - | - | - | - | - | - | - | - | - | - | - | - | - | - | - | - | - | - | H | H | T | A | D | L | S | N | A | R | L | H | F | V | T | A | G | - | - | - |
| 049 UniRef90\_F7YB04\_47\_316 | - | - | - | - | - | - | - | - | - | - | - | - | - | - | - | - | - | - | - | - | - | - | - | - | - | - | - | - | - | - | - | - | - | - | D | V | N | G | V | A | Y | H | Y | L | L | A | A | - | - | - |
| 050 UniRef90\_A0A1A3M1Z8\_5\_294 | - | - | - | - | - | - | - | - | - | - | - | - | - | - | - | - | - | - | - | - | - | - | - | - | - | - | - | - | - | R | H | N | V | F | D | T | A | D | V | R | I | H | Y | V | T | A | G | - | - | - |
| 051 UniRef90\_UPI0004C32CE1\_32\_312 | - | - | - | - | - | - | - | - | - | - | - | - | - | - | - | - | - | - | - | - | - | - | E | A | D | L | P | G | F | K | H | N | Y | A | Q | V | N | G | V | R | L | H | Y | V | T | G | G | - | - | - |
| 052 UniRef90\_A0A0J6NQJ9\_12\_287 | - | - | - | - | - | - | - | - | - | - | - | - | - | - | - | - | - | - | - | - | - | - | - | - | - | - | - | D | F | T | H | D | Y | V | R | L | D | G | Q | R | V | H | V | V | T | A | G | - | - | - |
| 053 UniRef90\_A0A534ESC2\_32\_321 | - | - | - | - | - | - | - | - | - | - | - | - | - | - | - | - | - | - | - | - | - | - | - | - | - | - | - | - | - | T | H | H | V | V | R | V | D | G | L | R | F | H | Y | V | T | A | G | - | - | - |
| 054 UniRef90\_A0A1H1Z302\_21\_301 | - | - | - | - | - | - | - | - | - | - | - | - | - | - | - | - | - | - | - | - | - | - | - | - | - | - | G | V | F | T | S | R | I | V | E | L | D | G | L | R | L | H | A | V | T | G | G | - | - | - |
| 055 UniRef90\_A0A158DLE6\_24\_295 | - | - | - | - | - | - | - | - | - | - | - | - | - | - | - | - | - | - | - | - | - | - | - | - | - | - | - | - | - | - | - | - | - | - | R | V | N | G | L | R | I | H | T | R | V | A | G | - | - | - |
| 056 UniRef90\_A0A163VVR8\_16\_280 | - | - | - | - | - | - | - | - | - | - | - | - | - | - | - | - | - | - | - | - | - | - | - | - | - | - | - | - | - | E | H | V | Y | L | Q | V | N | G | R | R | L | H | C | V | V | A | G | - | - | - |
| 057 UniRef90\_UPI000369F0DF\_7\_289 | - | - | - | - | - | - | - | - | - | - | - | - | - | - | - | - | - | - | - | - | - | P | D | T | R | D | E | G | F | A | H | H | Y | I | R | L | D | G | E | R | L | H | C | V | V | L | G | - | - | - |
| 058 UniRef90\_UPI00098F4FB4\_43\_309 | - | - | - | - | - | - | - | - | - | - | - | - | - | - | - | - | - | - | - | - | - | - | - | - | - | - | - | - | - | - | - | - | - | - | D | V | D | G | V | S | Y | H | Y | L | L | A | R | - | - | - |
| 059 UniRef90\_UPI0015A1AD76\_10\_292 | - | - | - | - | - | - | - | - | - | - | - | - | - | - | - | - | - | - | - | - | - | - | - | - | - | - | - | - | - | - | H | H | R | A | Q | V | G | D | V | S | L | H | Y | L | Q | A | G | - | - | - |
| 060 UniRef90\_UPI001689F051\_63\_318 | - | - | - | - | - | - | - | - | - | - | - | - | - | - | - | - | - | - | - | - | - | - | - | - | - | - | - | - | - | - | - | - | - | - | - | - | - | - | - | - | - | - | - | - | - | - | - | - | - | - |
| 061 UniRef90\_UPI000DD77D66\_43\_313 | - | - | - | - | - | - | - | - | - | - | - | - | - | - | - | - | - | - | - | - | - | - | - | - | - | - | - | - | - | - | - | - | - | - | D | V | D | G | V | S | Y | H | Y | L | L | A | R | - | - | - |
| 062 UniRef90\_A0A3M0I8T4\_31\_307 | - | - | - | - | - | - | - | - | - | - | - | - | - | - | - | - | - | - | - | - | - | - | - | - | - | - | - | - | F | T | S | R | Y | V | D | A | G | D | V | R | L | H | A | V | V | G | G | - | - | - |
| 063 UniRef90\_UPI000377E2D1\_4\_271 | - | - | - | - | - | - | - | - | - | - | - | - | - | - | - | - | - | - | - | - | - | - | - | - | - | - | - | - | H | R | H | S | Y | V | D | V | G | E | V | V | L | H | V | A | E | V | G | - | - | - |
| 064 UniRef90\_UPI0016198B53\_15\_286 | - | - | - | - | - | - | - | - | - | - | - | - | - | - | - | - | - | - | - | - | - | - | - | - | - | - | - | - | - | - | - | - | - | - | R | V | G | D | V | R | L | H | T | V | Q | G | G | - | - | - |
| 065 UniRef90\_UPI0013C0217D\_48\_325 | - | - | - | - | - | - | - | - | - | - | - | - | - | - | - | - | - | - | - | - | - | - | - | - | - | - | G | G | F | R | S | R | Y | A | T | V | N | G | V | R | L | H | Y | V | T | G | G | - | - | - |
| 066 UniRef90\_C7PGN2\_5\_260 | - | - | - | - | - | - | - | - | - | - | - | - | - | - | - | - | - | - | - | - | - | - | - | - | - | - | - | - | - | - | - | - | - | - | - | - | - | - | - | - | - | - | - | - | - | - | - | - | - | - |
| 067 UniRef90\_A0A1F7Q7Q2\_7\_275 | - | - | - | - | - | - | - | - | - | - | - | - | - | - | - | - | - | - | - | - | - | - | - | - | - | - | - | - | - | - | - | - | - | G | T | R | D | G | I | R | L | H | Y | R | R | L | G | - | - | - |
| 068 UniRef90\_A0A2V7F1K9\_9\_274 | - | - | - | - | - | - | - | - | - | - | - | - | - | - | - | - | - | - | - | - | - | - | - | - | - | - | - | - | - | - | - | - | - | - | - | K | D | R | V | R | I | H | Y | R | R | M | G | - | - | - |
| 069 UniRef90\_UPI001616C592\_31\_308 | - | - | - | - | - | - | - | - | - | - | - | - | - | - | - | - | - | - | - | - | - | - | - | - | - | - | - | - | F | A | S | R | L | V | N | V | D | G | V | H | L | H | V | V | T | G | G | - | - | - |
| 070 UniRef90\_UPI001456849A\_71\_360 | - | - | - | - | - | - | - | - | - | - | - | - | - | - | - | - | - | - | - | - | - | - | S | L | A | G | S | E | F | S | H | H | R | V | V | V | G | D | V | R | I | H | Y | V | R | G | G | - | - | - |
| 071 UniRef90\_A0A2S8J6S6\_18\_282 | - | - | - | - | - | - | - | - | - | - | - | - | - | - | - | - | - | - | - | - | - | - | - | - | - | - | - | - | - | - | - | - | - | - | - | - | P | D | V | S | L | H | A | V | V | A | G | - | - | - |
| 072 UniRef90\_A0A2I2GC12\_18\_280 | - | - | - | - | - | - | - | - | - | - | - | - | - | - | - | - | - | - | - | - | - | - | - | - | - | - | - | - | - | - | - | - | - | - | - | - | - | - | - | - | - | - | - | - | V | G | G | - | - | - |
| 073 UniRef90\_A0A549T469\_41\_312 | - | - | - | - | - | - | - | - | - | - | - | - | - | - | - | - | - | - | - | - | - | - | - | - | - | - | - | G | A | E | S | R | F | L | E | V | D | G | L | R | L | H | F | V | T | L | G | - | - | - |
| 074 UniRef90\_A0A5C4LJC6\_5\_278 | - | - | - | - | - | - | - | - | - | - | - | - | - | - | - | - | - | - | - | - | - | - | - | - | - | L | Q | G | F | S | Q | Q | L | V | P | A | N | G | I | R | L | N | A | V | T | G | G | - | - | - |
| 075 UniRef90\_UPI001619D806\_9\_283 | - | - | - | - | - | - | - | - | - | - | - | - | - | - | - | - | - | - | - | - | - | - | - | - | - | - | - | - | - | - | - | - | - | A | T | V | N | G | I | R | M | H | Y | R | R | A | G | - | - | - |
| 076 UniRef90\_A0A239P1A5\_69\_347 | - | - | - | - | - | - | - | - | - | - | - | - | - | - | - | - | - | - | - | - | - | - | - | - | - | - | - | T | F | T | S | R | Y | V | D | V | D | K | M | R | L | H | A | V | T | G | G | - | - | - |
| 077 UniRef90\_UPI0005262A70\_5\_280 | - | - | - | - | - | - | - | - | - | - | - | - | - | - | - | - | - | - | - | - | - | - | - | - | - | - | - | G | I | T | H | H | T | A | T | I | N | G | D | D | L | H | W | V | S | A | G | - | - | - |
| 078 UniRef90\_A0A241XUQ6\_27\_314 | - | - | - | - | - | - | - | - | - | - | - | - | - | - | - | - | - | - | - | - | - | E | F | P | V | P | N | G | F | E | S | A | Y | R | E | V | D | G | V | K | L | H | Y | V | K | G | G | - | - | - |
| 079 UniRef90\_A0A160FQ65\_32\_312 | - | - | - | - | - | - | - | - | - | - | - | - | - | - | - | - | - | - | - | - | - | - | - | - | - | - | - | - | F | E | S | Y | F | V | P | A | N | G | L | N | L | H | A | V | I | G | G | - | - | - |
| 080 UniRef90\_UPI0006AE4C23\_4\_281 | - | - | - | - | - | - | - | - | - | - | - | - | - | - | - | - | - | - | - | - | - | - | - | - | E | L | D | G | F | E | H | A | Y | A | R | V | N | G | V | R | L | H | Y | V | I | G | G | - | - | - |
| 081 UniRef90\_T0H3T7\_11\_281 | - | - | - | - | - | - | - | - | - | - | - | - | - | - | - | - | - | - | - | - | - | - | - | - | - | - | - | - | - | - | - | - | - | - | - | A | N | G | I | R | Q | N | Y | I | D | A | G | - | - | - |
| 082 UniRef90\_A0A4R2IH15\_5\_277 | - | - | - | - | - | - | - | - | - | - | - | - | - | - | - | - | - | - | - | - | - | - | - | - | - | - | - | G | F | T | E | R | Y | V | E | L | T | D | V | R | L | H | V | V | T | G | G | - | - | - |
| 083 UniRef90\_UPI00140B863E\_4\_276 | - | - | - | - | - | - | - | - | - | - | - | - | - | - | - | - | - | - | - | - | - | - | - | - | - | - | - | - | - | R | H | L | R | V | D | A | N | G | V | G | I | H | T | V | V | A | G | - | - | - |
| 084 UniRef90\_A0A3R9UXB7\_22\_297 | - | - | - | - | - | - | - | - | - | - | - | - | - | - | - | - | - | - | - | - | - | - | - | - | - | - | - | - | - | - | - | A | H | A | D | V | N | G | T | R | L | H | Y | V | E | G | G | - | - | - |
| 085 UniRef90\_UPI000F8D1465\_20\_303 | - | - | - | - | - | - | - | - | - | - | - | - | - | - | - | - | - | - | - | - | - | - | D | D | F | W | T | R | F | E | S | R | R | I | A | V | N | G | V | T | L | H | A | V | T | G | G | - | - | - |
| 086 UniRef90\_A0A6J4HYT7\_23\_307 | - | - | - | - | - | - | - | - | - | - | - | - | - | - | - | - | - | - | - | - | - | - | - | - | - | L | P | G | F | V | N | G | Y | A | R | V | N | G | V | T | L | H | Y | V | A | G | G | - | - | - |
| 087 UniRef90\_A0A1D8SMR3\_19\_296 | - | - | - | - | - | - | - | - | - | - | - | - | - | - | - | - | - | - | - | - | - | - | - | - | - | - | - | - | F | T | S | R | T | V | D | V | D | G | V | E | L | H | A | V | V | G | G | - | - | - |
| 088 UniRef90\_A0A4V2HUZ4\_48\_320 | - | - | - | - | - | - | - | - | - | - | - | - | - | - | - | - | - | - | - | - | - | - | - | - | - | - | A | G | M | S | S | R | F | V | D | V | N | G | I | R | M | H | Y | V | A | M | G | - | - | - |
| 089 UniRef90\_UPI0010582A1B\_5\_279 | - | - | - | - | - | - | - | - | - | - | - | - | - | - | - | - | - | - | - | - | - | - | - | - | - | L | K | G | F | T | Q | Q | L | M | P | V | N | G | I | R | I | N | V | V | T | G | G | - | - | - |
| 090 UniRef90\_UPI00166CE541\_54\_338 | - | - | - | - | - | - | - | - | - | - | - | - | - | - | - | - | - | - | - | - | - | E | P | R | V | P | A | G | F | R | E | K | R | V | R | V | G | D | V | A | I | N | H | V | R | G | G | - | - | - |
| 091 UniRef90\_A0A5C1HX54\_34\_307 | - | - | - | - | - | - | - | - | - | - | - | - | - | - | - | - | - | - | - | - | - | - | - | - | - | - | A | G | F | K | H | Q | Y | A | T | V | N | G | V | K | I | H | Y | V | I | G | G | - | - | - |
| 092 UniRef90\_A0A4D4KET2\_34\_306 | - | - | - | - | - | - | - | - | - | - | - | - | - | - | - | - | - | - | - | - | - | - | - | - | - | - | - | - | - | - | - | R | S | V | D | T | G | R | V | R | L | H | A | V | T | G | G | - | - | - |
| 093 UniRef90\_A0A1I1XP17\_9\_276 | - | - | - | - | - | - | - | - | - | - | - | - | - | - | - | - | P | - | - | - | - | - | - | - | - | - | - | - | - | - | - | - | - | - | V | A | E | G | V | R | L | H | Y | L | E | D | G | - | - | - |
| 094 UniRef90\_A0A1D7VX60\_23\_299 | - | - | - | - | - | - | - | - | - | - | - | - | - | - | - | - | - | - | - | - | - | - | - | - | - | - | - | T | F | T | S | R | F | V | Q | A | N | G | I | R | Q | H | V | V | V | G | G | - | - | - |
| 095 UniRef90\_A0A2T4ZAU7\_6\_296 | - | - | - | - | - | - | - | - | - | - | - | - | - | - | - | - | - | - | - | - | - | - | D | F | R | A | C | G | L | K | H | C | H | A | K | V | N | N | I | Q | L | H | F | V | E | G | G | - | - | - |
| 096 UniRef90\_UPI00143A9489\_23\_300 | - | - | - | - | - | - | - | - | - | - | - | - | - | - | - | - | - | - | - | - | - | - | P | P | Y | P | A | G | F | R | T | Q | Q | V | Q | V | D | G | A | T | L | H | V | R | I | G | G | - | - | - |
| 097 UniRef90\_A0A7C7NVI2\_6\_287 | - | - | - | - | - | - | - | - | - | - | - | - | - | - | - | - | - | - | - | - | - | - | - | - | - | - | - | - | - | D | H | R | M | A | L | V | N | G | I | N | I | H | Y | V | L | E | G | - | - | - |
| 098 UniRef90\_UPI001661A3FF\_52\_332 | - | - | - | - | - | - | - | - | - | - | - | - | - | - | - | - | - | - | - | - | - | - | - | - | - | - | A | G | F | T | E | H | R | A | R | V | A | G | I | G | L | H | Y | V | T | G | G | - | - | - |
| 099 UniRef90\_A0A6G4V4F1\_2\_276 | - | - | - | - | - | - | - | - | - | - | - | - | - | - | - | - | - | - | - | - | - | - | - | - | - | L | E | G | F | E | D | R | Y | A | D | V | N | G | T | R | L | H | Y | V | I | G | G | - | - | - |
| 100 UniRef90\_A0A260W032\_16\_294 | - | - | - | - | - | - | - | - | - | - | - | - | - | - | - | - | - | - | - | - | - | - | - | - | - | - | - | - | - | E | H | H | T | I | T | A | N | G | I | A | H | H | V | V | T | A | G | - | - | - |
| 101 UniRef90\_UPI00036BBA85\_7\_295 | - | - | - | - | - | - | - | - | - | - | - | - | - | - | - | - | - | - | - | - | - | - | - | - | - | - | - | - | - | E | H | H | R | A | L | V | N | G | I | H | M | H | Y | V | L | A | G | - | - | - |
| 102 UniRef90\_A0A4R5QBW7\_9\_278 | - | - | - | - | - | - | - | - | - | - | - | - | - | - | - | - | - | - | - | - | - | - | - | - | - | - | - | - | - | - | - | - | - | - | D | L | G | E | V | R | L | H | Y | V | T | A | G | - | - | - |
| 103 UniRef90\_UPI0012FB7881\_21\_292 | - | - | - | - | - | - | - | - | - | - | - | - | - | - | - | - | - | - | - | - | - | - | - | - | - | - | - | - | - | - | - | D | R | Y | D | V | G | D | V | R | L | H | A | V | T | G | G | - | - | - |
| 104 UniRef90\_UPI00131B39C2\_13\_285 | - | - | - | - | - | - | - | - | - | - | - | - | - | - | - | - | - | - | - | - | - | - | - | - | - | - | - | G | F | Q | T | Q | N | I | R | T | N | G | T | T | L | H | V | R | V | G | G | - | - | - |
| 105 UniRef90\_M2VKF7\_34\_311 | - | - | - | - | - | - | - | - | - | - | - | - | - | - | - | - | - | - | - | - | - | - | - | - | - | - | - | - | - | - | - | H | S | V | D | T | G | K | L | R | L | H | A | V | I | G | G | - | - | - |
| 106 UniRef90\_UPI0016124E74\_10\_283 | - | - | - | - | - | - | - | - | - | - | - | - | - | - | - | - | - | - | - | - | - | - | - | - | - | - | - | - | - | - | - | - | - | - | M | V | N | G | I | R | M | H | Y | R | R | A | G | - | - | - |
| 107 UniRef90\_A0A263DFR4\_27\_299 | - | - | - | - | - | - | - | - | - | - | - | - | - | - | - | - | - | - | - | - | - | - | - | - | - | - | - | - | - | - | - | - | - | - | - | T | P | R | L | R | H | H | A | V | V | G | G | - | - | - |
| 108 UniRef90\_A0A0U5F3A6\_35\_313 | - | - | - | - | - | - | - | - | - | - | - | - | - | - | - | - | - | - | - | - | - | - | - | - | - | - | - | - | F | K | S | Y | L | I | D | I | G | A | V | R | L | H | A | V | I | G | G | - | - | - |
| 109 UniRef90\_A0A2N3KZL1\_38\_324 | - | - | - | - | - | - | - | - | - | - | - | - | - | - | - | - | - | - | - | - | - | E | F | P | V | P | A | G | F | E | S | S | Y | A | E | I | N | G | V | R | L | H | Y | V | K | G | G | - | - | - |
| 110 UniRef90\_A0A1X7C0M0\_25\_283 | - | - | - | - | - | - | - | - | - | - | - | - | - | - | - | - | - | - | - | - | - | - | - | - | - | - | - | - | - | - | - | - | - | - | - | - | - | - | - | - | - | S | V | A | V | G | G | - | - | - |
| 111 UniRef90\_A0A078M0V2\_4\_284 | - | - | - | - | - | - | - | - | - | - | - | - | - | - | - | - | - | - | - | - | - | - | - | - | - | - | - | - | - | - | - | H | Y | I | T | I | D | N | T | K | L | H | Y | V | T | G | G | - | - | - |
| 112 UniRef90\_A0A0F4Z6H8\_20\_271 | - | - | - | - | - | - | - | - | - | - | - | - | - | - | - | - | - | - | - | - | - | - | - | - | - | - | - | - | - | - | - | - | - | - | - | - | - | - | - | - | - | - | - | - | - | - | - | - | - | - |
| 113 UniRef90\_UPI00161D2319\_22\_292 | - | - | - | - | - | - | - | - | - | - | - | - | - | - | - | - | - | - | - | - | - | - | - | - | - | - | - | - | - | - | - | H | T | V | E | T | C | E | L | T | L | H | T | V | S | G | G | - | - | - |
| 114 UniRef90\_A0A1Q8LX67\_38\_327 | - | - | - | - | - | - | - | - | - | - | - | - | - | - | - | - | - | - | T | R | D | D | P | D | F | D | G | T | F | R | H | G | F | A | D | V | D | G | V | R | M | H | Y | V | A | G | G | - | - | - |
| 115 UniRef90\_A0A1Q9S2P6\_18\_309 | V | A | - | - | - | - | - | - | - | - | - | - | - | - | - | - | - | - | T | R | D | D | P | G | F | S | A | R | F | R | H | E | F | T | E | V | D | G | V | R | M | H | Y | V | T | G | G | - | - | - |
| 116 UniRef90\_A0A5M7BQA6\_8\_281 | - | - | - | - | - | - | - | - | - | - | - | - | - | - | - | - | - | - | - | - | - | - | - | - | - | - | - | - | - | - | - | - | - | - | Q | V | N | G | L | R | M | H | Y | A | R | A | G | - | - | - |
| 117 UniRef90\_A0A3N6GJS2\_33\_314 | - | - | - | - | - | - | - | - | - | - | - | - | - | - | - | - | - | - | - | - | - | - | - | - | - | - | - | G | F | S | S | E | Y | A | T | V | N | G | L | R | L | H | Y | V | T | G | G | - | - | - |
| 118 UniRef90\_A0A0Q6MZD2\_36\_308 | - | - | - | - | - | - | - | - | - | - | - | - | - | - | - | - | - | - | - | - | - | - | - | - | - | - | - | - | F | S | T | R | E | V | T | T | N | G | A | T | I | H | V | R | S | G | G | - | - | - |
| 119 UniRef90\_UPI00052520C0\_22\_293 | - | - | - | - | - | - | - | - | - | - | - | - | - | - | - | - | - | - | - | - | - | - | - | - | - | - | - | - | - | - | - | G | R | Y | D | L | G | D | V | T | L | H | A | V | T | G | G | - | - | - |
| 120 UniRef90\_A0A1Q4XCF8\_2\_276 | - | - | - | - | - | - | - | - | - | - | - | - | - | - | - | - | - | - | - | - | - | - | - | - | - | - | - | - | - | - | - | - | - | - | T | V | E | G | L | R | L | H | Y | V | A | G | G | - | - | - |
| 121 UniRef90\_A0A328LZX6\_33\_305 | - | - | - | - | - | - | - | - | - | - | - | - | - | - | - | - | - | - | - | - | - | - | - | - | - | - | - | S | F | Q | T | R | D | I | A | T | N | G | T | T | L | H | V | R | V | G | G | - | - | - |
| 122 UniRef90\_A0A2V8S6L4\_40\_311 | - | - | - | - | - | - | - | - | - | - | - | - | - | - | - | - | - | - | - | - | - | - | - | - | - | - | P | G | F | S | D | R | F | A | E | V | N | G | T | R | L | H | Y | L | I | G | G | - | - | - |
| 123 UniRef90\_A0A1I5EB88\_8\_281 | - | - | - | - | - | - | - | - | - | - | - | - | - | - | - | - | - | - | - | - | - | - | - | - | - | - | - | - | - | - | - | - | - | - | L | V | N | G | L | R | V | H | Y | T | T | A | G | - | - | - |
| 124 UniRef90\_UPI0014147ABD\_39\_305 | - | - | - | - | - | - | - | - | - | - | - | - | - | - | - | - | - | - | - | - | - | - | - | - | - | - | - | - | - | - | - | - | - | - | - | - | G | E | L | R | L | H | A | V | V | G | G | - | - | - |
| 125 UniRef90\_A0A1I7DL13\_6\_274 | - | - | - | - | - | - | - | - | - | - | - | - | - | - | - | - | - | - | - | - | - | - | - | - | - | - | - | - | - | - | - | H | R | V | P | V | E | G | G | F | L | H | A | L | S | S | G | - | - | - |
| 126 UniRef90\_A0A3N7EDS3\_31\_305 | - | - | - | - | - | - | - | - | - | - | - | - | - | - | - | - | - | - | - | - | - | - | - | - | - | - | - | A | F | K | T | R | E | I | A | V | N | G | T | T | L | H | V | R | V | G | G | - | - | - |
| 127 UniRef90\_UPI0016225F9B\_4\_275 | - | - | - | - | - | - | - | - | - | - | - | - | - | - | - | - | - | - | - | - | - | - | - | - | - | - | - | - | - | - | - | - | - | - | - | V | G | G | L | R | M | H | Y | L | I | E | G | - | - | - |
| 128 UniRef90\_UPI000997AE50\_107\_382 | - | - | - | - | - | - | - | - | - | - | - | - | - | - | - | - | - | - | - | - | - | - | - | - | - | - | - | E | F | R | H | C | F | T | T | V | D | G | I | Q | M | H | Y | V | I | G | G | - | - | - |
| 129 UniRef90\_A0A0N7F3H5\_6\_275 | - | - | - | - | - | - | - | - | - | - | - | - | - | - | - | - | - | - | - | - | - | - | - | - | - | - | - | - | - | - | - | - | - | A | T | V | N | G | I | R | M | H | Y | L | R | G | G | - | - | - |
| 130 UniRef90\_A0A3Q8F441\_11\_281 | - | - | - | - | - | - | - | - | - | - | - | - | - | - | - | - | - | - | - | - | - | - | - | - | - | - | - | - | - | - | - | - | - | - | - | A | N | G | I | R | Q | N | F | I | D | A | G | - | - | - |
| 131 UniRef90\_UPI001487D2FB\_23\_300 | - | - | - | - | - | - | - | - | - | - | - | - | - | - | - | - | - | - | - | - | - | - | Q | P | F | P | S | S | F | R | A | Q | Q | V | Q | S | E | G | A | T | L | H | V | M | V | G | G | - | - | - |
| 132 UniRef90\_A0A437GNC7\_38\_307 | - | - | - | - | - | - | - | - | - | - | - | - | - | - | - | - | - | - | - | - | - | - | - | - | - | - | - | - | - | - | - | - | - | - | - | - | - | E | I | E | L | H | A | V | I | G | G | - | - | - |
| 133 UniRef90\_A0A1H3SWJ7\_6\_283 | - | - | - | - | - | - | - | - | - | - | - | - | - | - | - | - | - | - | - | - | - | - | - | - | - | - | - | - | - | R | E | E | T | R | T | V | D | G | L | R | F | H | V | R | R | A | G | - | - | - |
| 134 UniRef90\_UPI0013DD6281\_49\_329 | - | - | - | - | - | - | - | - | - | - | - | - | - | - | - | - | - | - | - | - | - | - | - | - | - | - | - | R | F | R | H | R | T | A | D | V | D | G | V | R | M | H | Y | V | T | G | G | - | - | - |
| 135 UniRef90\_I0L7C1\_27\_272 | - | - | - | - | - | - | - | - | - | - | - | - | - | - | - | - | - | - | - | - | - | - | - | - | - | - | - | - | - | - | - | - | - | - | - | - | - | - | - | - | - | - | - | - | - | - | G | - | - | - |
| 136 UniRef90\_A0A260DLM1\_45\_326 | - | - | - | - | - | - | - | - | - | - | - | - | - | - | - | - | - | - | - | - | - | - | - | - | - | - | R | L | F | R | H | E | F | A | T | V | R | G | M | Q | M | H | Y | V | V | G | G | - | - | - |
| 137 UniRef90\_UPI0005BDD2EC\_7\_280 | - | - | - | - | - | - | - | - | - | - | - | - | - | - | - | - | - | - | - | - | - | - | - | - | - | - | - | - | - | - | - | - | - | - | K | V | D | G | L | R | M | H | Y | R | R | A | G | - | - | - |
| 138 UniRef90\_A0A6G9YBC1\_27\_296 | - | - | - | - | - | - | - | - | - | - | - | - | - | - | - | - | - | - | - | - | - | - | - | - | - | - | - | - | - | - | - | - | - | - | S | V | N | G | I | R | V | H | Y | R | I | S | G | - | - | - |
| 139 UniRef90\_UPI00145FA88A\_8\_280 | - | - | - | - | - | - | - | - | - | - | - | - | - | - | - | - | - | - | - | - | - | - | - | - | - | - | - | - | - | - | - | - | - | - | - | V | N | G | I | R | M | H | Y | L | R | G | G | - | - | - |
| 140 UniRef90\_A0A2W0B1K8\_46\_299 | - | - | - | - | - | - | - | - | - | - | - | - | - | - | - | - | - | - | - | - | - | - | - | - | - | - | - | - | - | - | - | - | - | - | - | - | - | - | - | - | - | - | - | - | - | G | G | - | - | - |
| 141 UniRef90\_A0A5B8G622\_28\_299 | - | - | - | - | - | - | - | - | - | - | - | - | - | - | - | - | - | - | - | - | - | - | - | - | - | - | - | - | F | D | A | R | D | I | A | T | N | A | T | T | L | H | V | R | V | G | G | - | - | - |
| 142 UniRef90\_A0A4S1XGQ8\_21\_302 | - | - | - | - | - | - | - | - | - | - | - | - | - | - | - | - | - | - | - | - | E | V | P | A | Y | P | A | S | F | E | T | R | E | I | T | A | N | G | T | V | L | H | V | R | I | G | G | - | - | - |
| 143 UniRef90\_A0A251YJ72\_8\_294 | - | - | - | - | - | - | - | - | - | - | - | - | - | - | - | - | P | - | I | A | A | P | G | A | T | V | P | G | T | Q | H | H | R | A | D | V | G | G | T | S | L | H | V | V | T | A | G | - | - | - |
| 144 UniRef90\_UPI000A0527D2\_65\_318 | - | - | - | - | - | - | - | - | - | - | - | - | - | - | - | - | - | - | - | - | - | - | - | - | - | - | - | - | - | - | - | - | - | - | - | - | - | - | - | - | - | - | - | - | - | - | - | - | - | - |
| 145 UniRef90\_A0A4V2S7H9\_8\_280 | - | - | - | - | - | - | - | - | - | - | - | - | - | - | - | - | - | - | - | - | - | - | - | - | - | - | - | - | - | - | - | - | - | - | T | V | N | G | I | R | M | S | Y | Q | R | V | G | - | - | - |
| 146 UniRef90\_UPI00131B216A\_18\_247 | - | - | - | - | - | - | - | - | - | - | - | - | - | - | - | - | - | - | - | - | - | - | - | - | - | - | - | - | F | E | S | R | H | A | E | V | N | G | T | D | L | H | Y | V | I | G | G | - | - | - |
| 147 UniRef90\_A0A1G8T9Y6\_5\_243 | - | - | - | - | - | - | - | - | - | - | - | - | - | - | - | - | - | - | - | - | - | - | - | - | - | - | - | - | - | - | - | - | - | - | - | - | - | - | - | - | - | - | - | - | - | - | - | - | - | - |
| 148 UniRef90\_UPI00161F613F\_5\_254 | - | - | - | - | - | - | - | - | - | - | - | - | - | - | - | - | - | - | - | - | - | - | - | - | - | - | - | - | - | - | - | - | - | - | - | I | N | G | T | T | L | H | Y | V | E | A | G | - | - | - |
| 149 UniRef90\_UPI00142071FA\_80\_362 | - | - | - | - | - | - | - | - | - | - | - | - | - | - | - | - | - | - | - | - | - | - | - | - | - | - | - | - | F | T | S | R | F | V | E | A | N | G | I | R | Q | H | V | V | I | G | G | - | - | - |
| 150 UniRef90\_A0A6G7Q8F6\_7\_219 | - | - | - | - | - | - | - | - | - | - | - | - | - | - | - | - | - | - | - | - | - | - | - | - | - | - | - | - | - | S | H | R | H | V | D | L | R | G | L | R | L | H | V | A | E | A | G | - | - | - |

  
  

|  |  |  |  |  |  |  |  |  |  |  |  |  |  |  |  |  |  |  |  |  |  |  |  |  |  |  |  |  |  |  |  |  |  |  |  |  |  |  |  |  |  |  |  |  |  |  |  |  |  |  |
| --- | --- | --- | --- | --- | --- | --- | --- | --- | --- | --- | --- | --- | --- | --- | --- | --- | --- | --- | --- | --- | --- | --- | --- | --- | --- | --- | --- | --- | --- | --- | --- | --- | --- | --- | --- | --- | --- | --- | --- | --- | --- | --- | --- | --- | --- | --- | --- | --- | --- | --- |
| **001 Input\_protein\_seq** | R | A | - | - | - | - | - | D | - | - | - | G | E | - | T | - | I | V | L | L | A | G | F | P | E | S | W | Y | A | W | R | R | V | M | P | L | L | A | D | E | - | F | R | I | V | A | P | D | L | P |
| 002 UniRef90\_UPI001430CB85\_4\_307 | K | D | - | - | - | - | - | S | - | - | - | G | G | - | T | - | V | V | L | L | A | G | Y | P | E | S | W | F | A | W | R | K | V | M | P | A | L | A | E | H | - | Y | R | V | I | V | P | D | L | P |
| 003 UniRef90\_UPI00055F8D66\_10\_306 | N | E | - | - | - | - | - | D | - | - | - | G | D | - | V | - | V | V | L | L | A | G | F | P | E | S | W | Y | A | W | R | K | I | M | P | L | L | A | P | T | - | Y | K | L | I | A | L | D | L | P |
| 004 UniRef90\_UPI00161D8BA7\_15\_312 | Q | S | - | - | - | - | - | D | - | - | - | G | D | - | I | - | V | V | L | L | A | G | F | P | E | S | W | F | A | W | R | K | V | I | P | Q | L | A | P | N | - | F | K | V | I | A | I | D | L | P |
| 005 UniRef90\_A0A4R7GLU7\_7\_305 | N | P | - | - | - | - | - | E | - | - | - | G | K | - | V | - | L | L | L | L | A | G | F | P | Q | T | W | Y | A | W | R | G | V | M | Q | A | L | A | N | D | - | F | W | L | I | A | P | D | L | P |
| 006 UniRef90\_UPI00041CD164\_24\_309 | K | A | - | - | - | - | - | D | - | - | - | G | D | - | V | - | V | V | L | L | A | G | F | P | E | S | G | Y | A | W | R | K | V | M | P | L | L | A | G | S | - | F | R | V | I | A | P | D | L | P |
| 007 UniRef90\_I0QSF8\_15\_298 | N | P | - | - | - | - | - | H | - | - | - | G | E | - | T | - | L | V | L | L | A | G | F | P | E | S | W | Y | S | W | R | K | I | M | P | A | L | A | D | H | - | Y | W | I | I | A | P | D | L | P |
| 008 UniRef90\_A0A2N5ENH7\_9\_301 | N | P | - | - | - | - | - | Q | - | - | - | G | E | - | T | - | L | L | L | L | A | G | F | P | Q | S | G | Y | A | W | H | K | V | M | A | R | L | A | D | R | - | Y | H | I | I | A | P | D | L | P |
| 009 UniRef90\_A0A0Q8E2M6\_29\_312 | Q | P | - | - | - | - | - | G | - | - | - | G | D | - | T | - | I | V | F | L | A | G | F | P | Q | S | W | Y | A | W | R | K | V | M | P | Y | L | A | S | T | - | R | D | V | I | A | I | D | L | P |
| 010 UniRef90\_A0A0F5XY46\_6\_287 | C | E | - | - | - | - | - | N | - | - | - | A | E | - | V | - | V | V | L | L | A | G | F | P | Q | S | S | Y | A | W | R | E | V | I | P | G | L | A | D | H | - | F | Q | V | I | A | P | D | L | P |
| 011 UniRef90\_A0A3N4NUX5\_4\_284 | D | P | - | - | - | - | - | Q | - | - | - | Q | D | - | T | - | L | V | L | L | A | G | F | P | Q | S | S | Y | A | W | R | D | I | M | P | L | L | T | D | R | - | F | Y | V | I | A | P | D | M | P |
| 012 UniRef90\_UPI000DDED2D9\_11\_293 | C | A | - | - | - | - | - | G | - | - | - | G | E | - | T | - | I | I | L | F | P | G | F | P | Q | S | W | Y | A | W | R | K | I | L | P | M | L | G | E | R | - | Y | R | V | I | A | P | D | L | P |
| 013 UniRef90\_UPI000DDED53B\_10\_292 | N | K | - | - | - | - | - | N | - | - | - | G | P | - | T | - | I | V | L | L | A | G | F | P | E | N | W | L | A | W | R | K | V | M | I | S | L | A | P | T | - | F | Y | V | V | A | I | D | L | P |
| 014 UniRef90\_A0A1G4JKM0\_6\_288 | K | S | - | - | - | - | - | S | - | - | - | G | D | - | T | N | L | V | L | L | A | G | F | P | E | S | W | Y | A | W | R | Q | V | I | P | E | L | A | K | N | - | F | H | V | V | A | I | D | L | P |
| 015 UniRef90\_UPI0016728815\_21\_305 | R | P | - | - | - | - | - | D | - | - | - | G | P | - | A | - | V | V | L | L | A | G | F | P | Q | T | W | W | A | W | R | K | V | M | P | D | L | A | G | R | - | F | R | V | I | A | I | D | L | P |
| 016 UniRef90\_A0A3N2H8D2\_14\_297 | R | T | - | - | - | - | - | G | - | - | - | G | P | - | A | - | V | V | L | L | A | G | F | P | Q | T | W | W | A | W | R | K | V | M | P | R | L | A | E | R | - | F | H | V | I | A | I | D | L | P |
| 017 UniRef90\_UPI0005644DD1\_9\_307 | Q | P | - | - | - | - | - | A | - | - | - | G | P | - | A | - | V | V | L | L | A | G | F | P | Q | T | W | R | A | W | R | K | V | M | P | G | L | A | D | R | - | F | R | V | I | A | V | E | L | P |
| 018 UniRef90\_A0A6B2VDS0\_18\_308 | R | P | - | - | - | - | - | S | - | - | - | G | P | - | A | - | V | V | L | L | A | G | F | P | Q | T | W | W | A | W | R | K | V | M | P | G | L | A | H | R | - | F | H | V | I | A | I | D | L | P |
| 019 UniRef90\_A0A6G3RZZ1\_25\_307 | R | P | - | - | - | - | - | A | - | - | - | G | P | - | T | - | V | V | L | L | A | G | F | P | Q | T | W | W | A | W | R | K | V | M | P | G | L | A | E | R | - | F | H | V | V | A | V | D | L | P |
| 020 UniRef90\_A0A209C7X3\_21\_305 | R | P | - | - | - | - | - | D | - | - | - | G | P | - | A | - | V | V | L | L | A | G | F | P | Q | T | W | W | A | W | R | K | V | M | P | N | L | A | G | R | - | C | R | V | I | A | I | D | L | P |
| 021 UniRef90\_UPI001269E708\_26\_309 | R | P | - | - | - | - | - | A | - | - | - | G | P | - | V | - | V | V | L | L | A | G | F | P | Q | T | W | W | A | W | R | K | V | M | P | L | L | A | E | R | - | F | R | V | I | A | I | D | L | P |
| 022 UniRef90\_A0A2M9J7W9\_25\_306 | R | P | - | - | - | - | - | E | - | - | - | G | P | - | T | - | V | V | L | L | A | G | F | P | Q | T | W | W | A | W | R | E | V | M | T | G | L | A | E | R | - | F | H | V | I | A | V | D | L | P |
| 023 UniRef90\_A0A6G9F3J8\_23\_307 | R | S | - | - | - | - | N | G | - | - | - | G | P | - | T | - | V | V | L | L | A | G | F | P | Q | T | W | W | A | W | R | K | V | M | P | T | L | A | D | R | - | Y | H | V | I | A | I | D | L | P |
| 024 UniRef90\_A0A1H2UZ41\_14\_292 | - | - | - | - | - | - | - | Q | - | - | - | G | F | - | P | - | V | L | L | M | A | G | F | P | Q | S | W | Y | A | W | R | R | V | M | P | L | L | C | E | H | - | F | H | V | V | A | I | D | L | P |
| 025 UniRef90\_UPI0012B05B7D\_16\_286 | - | - | - | - | - | - | - | T | - | - | - | G | P | - | V | - | I | L | L | V | A | G | F | P | Q | S | C | Y | A | W | R | K | V | I | P | L | L | A | N | K | - | Y | R | V | I | A | L | D | L | P |
| 026 UniRef90\_A0A5C8T429\_18\_294 | - | - | - | - | - | - | - | Q | - | - | - | G | A | - | P | - | V | L | L | V | P | G | W | P | Q | T | W | Y | A | W | R | H | V | M | P | L | L | A | K | R | - | F | T | V | V | A | V | D | L | P |
| 027 UniRef90\_UPI0012E86E3E\_10\_309 | - | - | - | - | - | - | - | S | - | - | - | G | A | - | P | - | I | L | L | I | P | G | W | P | Q | S | W | Y | T | W | R | Y | V | M | P | L | L | A | A | A | G | R | R | V | I | A | L | D | P | R |
| 028 UniRef90\_A0A1A9HTL2\_13\_300 | G | S | - | - | - | - | - | K | - | - | - | G | D | - | P | - | I | L | L | I | P | G | W | P | E | S | W | Y | A | W | R | F | V | M | P | R | L | I | A | E | G | H | P | V | V | A | L | D | L | R |
| 029 UniRef90\_A0A0R1V0C7\_7\_281 | K | E | - | - | - | - | - | T | - | - | - | A | P | - | T | - | I | F | L | I | A | G | F | P | Q | S | A | Y | A | W | R | K | V | W | P | L | L | A | K | Q | - | Y | H | V | L | A | I | D | L | P |
| 030 UniRef90\_A0A3N1M9Y1\_5\_282 | - | - | - | - | - | - | - | E | - | - | - | G | P | - | P | - | V | V | L | L | H | G | W | P | S | T | W | Y | E | W | R | H | V | I | D | R | L | A | P | H | - | H | R | V | I | A | P | D | L | R |
| 031 UniRef90\_A0A158JUX7\_17\_294 | - | - | - | - | - | - | - | S | - | - | - | G | Q | - | P | - | V | L | L | I | H | G | W | P | E | T | W | Y | A | W | R | K | V | I | P | L | L | A | G | R | - | Y | F | V | I | A | P | D | M | R |
| 032 UniRef90\_A0A0U3LIL8\_12\_296 | - | - | - | - | - | - | - | T | - | - | - | G | A | - | P | - | V | L | L | I | P | G | W | P | Q | S | W | F | A | W | R | D | V | I | P | R | L | A | R | A | G | R | R | V | I | A | I | D | P | R |
| 033 UniRef90\_A0A248JS33\_7\_301 | E | Q | - | - | - | - | - | Q | - | - | - | G | P | - | P | - | V | L | L | I | P | G | W | P | Q | S | W | Y | A | W | R | H | V | M | P | L | L | A | D | A | G | R | R | V | I | A | V | D | P | R |
| 034 UniRef90\_A0A1Y0REZ4\_12\_287 | - | - | - | - | - | - | - | K | - | - | - | G | D | - | P | - | V | V | L | L | H | G | W | P | Q | T | W | Y | E | W | R | R | I | M | P | A | L | A | E | K | - | Y | T | V | I | A | P | D | L | R |
| 035 UniRef90\_A0A0M2WRU5\_3\_280 | T | G | - | - | - | - | - | N | - | - | - | G | K | - | P | - | V | L | L | I | P | G | W | P | Q | T | W | Y | A | W | R | H | V | M | A | A | L | A | A | N | G | Y | Q | A | V | A | V | D | P | P |
| 036 UniRef90\_A0A0N8GFM1\_6\_279 | - | - | - | - | - | - | - | Q | - | - | - | G | D | - | P | - | V | L | L | V | H | G | I | P | Q | T | S | Y | E | W | R | H | I | M | P | R | L | A | E | R | - | Y | T | V | I | A | P | D | L | R |
| 037 UniRef90\_A0A252EMP1\_7\_277 | - | - | - | - | - | - | - | K | - | - | - | G | E | - | P | - | V | L | L | V | H | G | W | P | Q | T | W | Y | E | W | H | R | V | I | P | H | L | V | E | A | G | H | E | V | I | A | V | D | M | R |
| 038 UniRef90\_W0A6K5\_40\_320 | - | - | - | - | - | - | - | K | - | - | - | G | E | - | P | - | V | L | L | I | P | G | W | P | Q | T | W | Y | A | W | R | H | V | M | T | K | L | A | T | E | G | Y | M | A | I | A | V | D | P | P |
| 039 UniRef90\_A0A3S1SM66\_23\_304 | - | - | - | - | - | - | - | Q | - | - | - | G | R | - | P | - | V | L | L | I | P | G | W | P | Q | S | W | Y | A | W | R | H | V | M | P | F | L | I | A | A | G | R | R | V | I | A | I | D | P | R |
| 040 UniRef90\_A0A2E5L5C6\_7\_286 | - | - | - | - | - | - | - | A | - | - | - | G | P | - | A | - | I | V | L | L | H | G | W | P | Q | T | W | W | E | W | R | H | I | I | P | S | L | A | M | N | - | Y | T | V | I | A | P | D | L | R |
| 041 UniRef90\_M5D1B4\_14\_289 | - | - | - | - | - | - | - | H | - | - | - | G | E | - | P | - | V | L | L | I | P | G | W | P | Q | T | W | Y | A | W | R | H | V | L | H | A | L | A | A | A | G | F | E | A | I | A | V | D | P | P |
| 042 UniRef90\_UPI0016845BA7\_31\_303 | - | - | - | - | - | - | - | Q | - | - | - | G | D | - | P | - | L | V | L | L | H | G | W | T | Q | T | S | Y | A | W | N | R | I | M | P | A | L | A | E | R | - | Y | T | V | I | A | P | D | T | R |
| 043 UniRef90\_UPI0014648199\_14\_291 | - | - | - | - | - | - | - | T | - | - | - | G | E | - | P | - | V | L | L | I | P | G | W | P | Q | T | W | Y | A | F | R | H | V | M | Q | A | L | A | E | Q | G | F | Q | A | I | A | V | D | P | P |
| 044 UniRef90\_K9RUM7\_12\_289 | - | - | - | - | - | - | - | Q | - | - | - | G | P | - | T | - | L | L | L | L | G | G | W | P | Q | T | C | Y | V | W | R | L | L | W | Q | Q | L | G | E | K | - | F | R | V | I | S | L | D | M | R |
| 045 UniRef90\_A0A1Q2U086\_13\_290 | - | - | - | - | - | - | - | Q | - | - | - | G | T | - | P | - | L | L | L | L | G | G | W | P | Q | T | C | Y | V | W | R | L | L | L | E | P | L | G | Q | N | - | F | H | V | I | A | L | D | M | R |
| 046 UniRef90\_B9JMN1\_3\_282 | - | - | - | - | - | - | - | K | - | - | - | G | E | - | P | - | L | V | L | L | H | G | W | P | Q | N | W | A | S | W | K | R | I | I | P | V | L | S | D | H | - | F | T | V | I | A | P | D | M | R |
| 047 UniRef90\_A0A2X1TAZ7\_33\_319 | - | - | - | - | - | - | - | K | - | - | - | G | E | - | P | - | V | L | L | I | P | G | W | P | Q | T | W | Y | T | W | R | Y | V | M | T | E | L | A | A | Q | G | Y | M | A | I | A | V | D | P | P |
| 048 UniRef90\_UPI00131A8152\_6\_279 | - | - | - | - | - | - | - | E | - | - | - | G | E | - | P | - | L | V | L | L | H | G | W | P | Q | T | W | Y | C | W | R | R | V | I | P | L | L | A | D | R | - | Y | R | I | I | A | P | D | L | T |
| 049 UniRef90\_F7YB04\_47\_316 | - | - | - | - | - | - | - | G | - | - | - | G | P | K | T | - | V | V | L | L | H | G | W | G | T | T | S | Y | M | W | R | F | V | M | P | Q | L | V | A | R | G | Y | T | V | L | A | P | D | L | R |
| 050 UniRef90\_A0A1A3M1Z8\_5\_294 | T | P | P | S | D | G | G | D | - | - | - | D | R | - | A | - | I | V | L | L | H | G | W | P | Q | T | W | W | E | W | R | H | I | M | E | P | L | H | S | D | G | W | F | V | V | A | P | D | Y | R |
| 051 UniRef90\_UPI0004C32CE1\_32\_312 | - | - | - | - | - | - | - | R | - | - | - | G | E | - | P | - | L | V | L | L | P | G | W | P | Q | T | W | W | E | Y | H | Q | I | M | P | A | L | A | T | R | - | F | R | V | I | A | V | D | L | R |
| 052 UniRef90\_A0A0J6NQJ9\_12\_287 | - | - | - | - | - | - | - | A | - | - | - | G | R | - | P | - | V | L | L | I | P | G | W | P | Q | T | W | F | A | W | R | H | V | M | R | A | L | A | A | R | G | F | Q | A | I | A | V | D | P | P |
| 053 UniRef90\_A0A534ESC2\_32\_321 | - | - | - | - | - | - | - | S | - | - | - | G | E | - | P | - | V | V | L | L | P | G | W | P | E | S | W | I | A | W | R | K | L | T | P | L | L | V | K | A | G | R | Q | V | Y | V | L | D | P | R |
| 054 UniRef90\_A0A1H1Z302\_21\_301 | - | - | - | - | - | - | - | D | - | - | - | G | P | - | P | - | L | L | L | V | G | G | W | P | Q | T | W | Y | A | W | R | E | V | M | P | A | L | A | R | R | - | H | T | V | V | A | V | D | S | R |
| 055 UniRef90\_A0A158DLE6\_24\_295 | - | - | - | - | - | - | - | A | - | - | - | G | P | - | A | - | F | L | L | L | H | G | W | P | Q | T | S | Y | A | W | R | K | L | M | P | L | L | S | P | H | - | A | T | V | I | A | P | D | L | R |
| 056 UniRef90\_A0A163VVR8\_16\_280 | - | - | - | - | - | - | - | E | - | - | - | G | R | - | P | - | V | L | L | I | P | G | W | P | Q | T | W | Y | T | W | R | H | V | M | Q | A | L | A | A | A | G | F | Q | A | I | A | V | D | P | P |
| 057 UniRef90\_UPI000369F0DF\_7\_289 | - | - | - | - | - | - | - | E | - | - | - | G | K | - | P | - | A | L | L | I | P | G | W | P | Q | T | W | Y | A | W | R | H | V | M | P | A | L | A | R | N | G | F | R | A | I | A | V | D | P | I |
| 058 UniRef90\_UPI00098F4FB4\_43\_309 | - | - | - | - | - | - | - | G | - | - | - | G | P | K | T | - | V | V | L | L | H | G | W | G | T | T | S | Y | M | W | R | Y | V | M | P | Q | L | A | A | L | G | F | T | V | I | A | P | D | L | R |
| 059 UniRef90\_UPI0015A1AD76\_10\_292 | - | - | - | - | - | - | - | Q | P | Q | P | G | R | A | P | - | L | V | L | L | H | G | F | P | Q | H | S | H | M | W | R | R | I | M | P | A | L | A | E | H | - | Y | L | V | I | A | P | D | L | R |
| 060 UniRef90\_UPI001689F051\_63\_318 | - | - | - | - | - | - | - | T | - | - | - | G | E | - | P | - | A | L | L | L | H | G | W | P | Q | S | S | Y | A | W | R | A | V | I | P | L | L | - | D | R | - | Y | T | L | V | V | P | D | L | P |
| 061 UniRef90\_UPI000DD77D66\_43\_313 | - | - | - | - | - | - | - | G | - | - | - | G | P | Q | T | - | V | V | L | L | H | G | W | G | T | T | S | Y | M | W | R | Y | V | M | P | E | L | T | G | R | G | Y | T | V | L | A | P | D | I | R |
| 062 UniRef90\_A0A3M0I8T4\_31\_307 | - | - | - | - | - | - | - | D | - | - | - | G | P | - | P | - | L | L | L | L | A | G | W | P | Q | T | W | Y | A | W | R | L | L | M | P | A | L | A | E | D | - | F | R | V | V | A | V | D | P | R |
| 063 UniRef90\_UPI000377E2D1\_4\_271 | - | - | - | - | - | - | - | E | - | - | - | G | A | - | P | - | V | L | L | I | H | G | F | P | Q | T | W | Y | S | W | T | Q | V | A | P | L | L | V | D | A | G | Y | R | C | V | I | P | D | L | R |
| 064 UniRef90\_UPI0016198B53\_15\_286 | - | - | - | - | - | - | - | A | - | - | - | G | H | - | P | - | L | L | L | V | G | G | W | P | Q | N | W | Y | V | W | R | H | V | M | P | R | L | A | G | H | - | F | R | V | I | A | A | D | P | R |
| 065 UniRef90\_UPI0013C0217D\_48\_325 | - | - | - | - | - | - | - | H | - | - | - | G | Q | - | P | - | L | L | L | V | P | G | W | P | Q | T | W | W | A | Y | R | K | V | M | P | Q | L | A | R | R | - | H | R | V | I | A | V | D | L | R |
| 066 UniRef90\_C7PGN2\_5\_260 | - | - | - | - | - | - | - | - | - | - | - | - | K | - | V | - | I | L | L | L | H | G | Y | P | Q | T | S | Y | A | W | R | F | V | T | P | L | L | A | K | E | G | Y | K | V | I | A | P | D | L | P |
| 067 UniRef90\_A0A1F7Q7Q2\_7\_275 | - | - | - | - | - | - | - | R | - | - | - | G | P | - | G | - | L | V | L | L | H | G | Y | P | Q | T | G | H | M | W | R | K | V | M | P | V | L | A | E | Q | - | F | T | V | V | A | P | D | L | R |
| 068 UniRef90\_A0A2V7F1K9\_9\_274 | - | - | - | - | - | - | - | A | - | - | - | G | R | - | G | - | M | V | L | L | H | G | Y | P | Q | T | G | H | M | W | R | K | V | M | P | A | L | A | E | R | - | F | T | V | V | A | P | D | L | R |
| 069 UniRef90\_UPI001616C592\_31\_308 | - | - | - | - | - | - | - | D | - | - | - | G | P | - | P | - | V | L | L | L | G | G | W | P | Q | F | W | Y | Q | W | R | L | I | M | P | A | L | A | E | D | - | H | T | V | I | A | V | D | P | R |
| 070 UniRef90\_UPI001456849A\_71\_360 | - | - | - | - | - | - | - | K | - | - | - | G | E | - | P | - | L | I | L | L | H | G | W | P | A | T | W | W | E | W | K | K | V | M | P | G | L | A | K | D | - | F | D | V | I | A | V | D | T | R |
| 071 UniRef90\_A0A2S8J6S6\_18\_282 | - | - | - | - | - | - | - | A | - | - | - | G | P | - | A | - | V | F | L | L | H | G | F | P | Q | T | W | H | E | W | T | P | V | I | D | E | L | A | Q | H | - | H | T | V | V | A | V | D | L | K |
| 072 UniRef90\_A0A2I2GC12\_18\_280 | - | - | - | - | - | - | - | Q | - | - | - | G | P | - | P | - | V | V | L | V | A | G | W | P | Q | T | A | E | A | Y | S | D | L | F | E | P | L | S | K | H | - | H | Q | F | F | A | L | D | P | P |
| 073 UniRef90\_A0A549T469\_41\_312 | - | - | - | - | - | - | - | A | - | - | - | G | P | - | A | - | V | V | L | L | H | G | W | P | Q | T | W | F | A | W | A | A | T | M | E | R | L | A | R | R | - | F | T | V | I | A | P | D | L | R |
| 074 UniRef90\_A0A5C4LJC6\_5\_278 | - | - | - | - | - | - | - | A | - | - | - | G | P | - | P | - | I | L | L | L | H | G | W | P | E | T | W | W | E | W | H | H | V | M | P | V | L | A | E | R | - | F | S | V | V | A | L | D | L | R |
| 075 UniRef90\_UPI001619D806\_9\_283 | - | - | - | - | - | - | - | D | - | - | - | G | P | - | P | - | V | V | L | L | H | G | W | P | Q | T | W | F | C | W | R | N | V | I | E | S | L | A | K | R | - | H | T | V | I | A | P | D | L | R |
| 076 UniRef90\_A0A239P1A5\_69\_347 | - | - | - | - | - | - | - | D | - | - | - | G | P | - | P | - | I | L | F | I | G | G | W | P | Q | T | W | Y | A | W | R | H | V | M | P | A | F | A | R | D | - | F | S | I | V | A | I | D | S | R |
| 077 UniRef90\_UPI0005262A70\_5\_280 | - | - | - | - | - | - | - | S | G | - | - | G | T | - | P | - | V | L | L | V | H | G | F | P | E | S | W | W | A | F | R | R | L | I | P | L | L | A | A | E | - | H | R | V | I | A | V | D | L | P |
| 078 UniRef90\_A0A241XUQ6\_27\_314 | - | - | - | - | - | - | - | Q | - | - | - | G | P | - | L | - | V | M | L | V | H | G | F | G | Q | T | W | Y | E | W | H | Q | L | M | P | E | L | A | K | R | - | F | T | V | I | A | P | D | L | P |
| 079 UniRef90\_A0A160FQ65\_32\_312 | - | - | - | - | - | - | - | K | - | - | - | G | A | - | P | - | L | L | L | L | A | G | W | P | Q | N | W | F | A | W | R | Y | M | M | L | P | L | A | Q | S | - | F | T | V | I | A | V | D | P | R |
| 080 UniRef90\_UPI0006AE4C23\_4\_281 | - | - | - | - | - | - | - | S | - | - | - | G | D | - | P | - | L | V | L | L | P | G | W | P | R | T | W | W | Q | Y | R | K | V | M | P | A | L | A | E | H | - | F | R | V | I | V | C | E | Y | R |
| 081 UniRef90\_T0H3T7\_11\_281 | - | - | - | - | - | - | - | E | - | - | - | G | A | - | P | - | V | V | L | L | H | G | F | P | E | T | N | F | A | W | R | F | Q | I | P | V | L | G | E | L | - | Y | R | I | I | A | P | D | L | R |
| 082 UniRef90\_A0A4R2IH15\_5\_277 | - | - | - | - | - | - | - | E | - | - | - | G | P | - | P | - | V | L | L | I | A | G | W | P | Q | T | W | Y | A | W | R | L | V | M | P | E | L | A | R | H | - | R | Q | V | V | A | V | D | T | R |
| 083 UniRef90\_UPI00140B863E\_4\_276 | - | - | - | - | - | - | - | R | - | - | - | G | S | - | P | - | V | V | L | L | H | G | W | P | V | T | W | Y | H | W | R | T | T | I | S | V | L | A | E | H | - | H | L | V | I | A | P | D | L | R |
| 084 UniRef90\_A0A3R9UXB7\_22\_297 | - | - | - | - | - | - | - | S | G | P | - | G | A | - | P | - | V | V | L | L | G | G | W | P | Q | T | W | W | Q | W | H | K | V | L | P | E | L | A | R | H | - | H | R | V | L | A | V | D | L | R |
| 085 UniRef90\_UPI000F8D1465\_20\_303 | - | - | - | - | - | - | - | K | - | - | - | G | P | - | P | - | L | L | L | I | N | G | W | P | Q | T | W | Y | V | W | R | F | L | M | P | V | L | A | A | H | - | Y | S | L | V | V | A | E | P | R |
| 086 UniRef90\_A0A6J4HYT7\_23\_307 | - | - | - | - | - | - | - | Q | - | - | - | G | T | - | P | - | L | V | L | L | P | G | W | P | Q | T | W | W | A | Y | H | K | V | M | P | L | L | A | A | R | - | F | R | V | I | A | V | D | I | R |
| 087 UniRef90\_A0A1D8SMR3\_19\_296 | - | - | - | - | - | - | - | T | - | - | - | G | P | - | A | - | L | L | L | L | P | A | W | P | Q | F | W | Y | G | W | R | L | V | M | P | A | L | A | E | H | - | F | T | V | V | A | A | D | M | R |
| 088 UniRef90\_A0A4V2HUZ4\_48\_320 | - | - | - | - | - | - | - | S | - | - | - | G | P | - | V | - | L | V | L | L | H | G | W | P | E | T | W | F | A | W | R | G | I | M | P | R | L | A | R | K | - | F | T | V | V | A | A | D | L | R |
| 089 UniRef90\_UPI0010582A1B\_5\_279 | - | - | - | - | - | - | - | S | - | - | - | G | P | - | P | - | I | L | L | L | H | G | W | P | E | T | W | W | E | W | H | H | V | M | P | L | L | A | A | H | - | F | S | V | V | A | M | D | L | R |
| 090 UniRef90\_UPI00166CE541\_54\_338 | - | - | - | - | - | - | - | H | - | - | - | G | P | - | T | - | L | V | L | L | H | G | Y | P | Q | T | W | Y | T | W | R | E | V | L | P | E | L | A | K | H | - | Y | T | V | I | A | P | D | L | R |
| 091 UniRef90\_A0A5C1HX54\_34\_307 | - | - | - | - | - | - | - | Q | - | - | - | G | E | - | P | - | L | L | L | V | H | G | F | G | Q | N | W | Y | M | W | N | R | L | L | P | E | L | S | K | H | - | F | T | I | V | A | P | D | L | R |
| 092 UniRef90\_A0A4D4KET2\_34\_306 | - | - | - | - | - | - | - | E | - | - | - | G | P | - | A | - | L | L | L | L | C | G | W | P | Q | T | W | Y | A | W | R | L | L | M | P | A | L | A | R | D | - | F | H | V | V | A | P | D | P | R |
| 093 UniRef90\_A0A1I1XP17\_9\_276 | - | - | - | - | - | - | - | A | - | - | - | G | P | R | T | - | V | V | L | L | H | G | F | P | Q | T | C | H | A | W | R | K | V | A | P | R | L | V | A | Q | G | L | R | V | I | A | P | D | Y | R |
| 094 UniRef90\_A0A1D7VX60\_23\_299 | - | - | - | - | - | - | - | E | - | - | - | G | P | - | P | - | L | L | L | I | H | G | W | P | E | T | W | Y | A | W | R | L | V | M | P | A | L | A | R | H | - | F | T | V | I | A | V | D | Q | R |
| 095 UniRef90\_A0A2T4ZAU7\_6\_296 | - | - | - | - | - | - | - | E | H | H | - | T | E | - | T | - | V | I | L | L | H | G | F | P | Q | S | W | V | I | W | R | Y | V | I | F | D | L | L | K | R | - | Y | H | V | I | A | V | D | L | R |
| 096 UniRef90\_UPI00143A9489\_23\_300 | - | - | - | - | - | - | - | Q | - | - | - | G | P | - | A | - | V | L | M | I | H | G | Y | G | E | T | G | D | M | W | S | P | L | A | A | A | L | A | A | D | - | H | T | L | I | A | P | D | L | R |
| 097 UniRef90\_A0A7C7NVI2\_6\_287 | - | - | - | - | - | - | - | Q | - | - | - | G | N | - | P | - | V | V | L | L | H | G | W | P | E | F | W | Y | G | W | R | K | Q | I | P | V | L | S | R | R | - | F | Q | T | I | V | P | D | M | R |
| 098 UniRef90\_UPI001661A3FF\_52\_332 | - | - | - | - | - | - | - | H | - | - | - | G | P | - | T | - | L | V | L | L | H | G | Y | P | Q | T | W | Y | E | W | H | G | I | M | P | A | L | A | E | H | - | Y | T | V | I | A | P | D | L | P |
| 099 UniRef90\_A0A6G4V4F1\_2\_276 | - | - | - | - | - | - | - | E | - | - | - | G | E | - | P | - | L | V | L | M | D | G | F | P | R | T | W | H | G | L | H | K | I | M | T | P | L | A | E | H | - | F | R | V | I | A | V | D | Y | R |
| 100 UniRef90\_A0A260W032\_16\_294 | - | - | - | - | - | - | - | S | - | - | - | G | P | - | P | - | V | Y | L | L | H | G | F | P | E | T | W | F | G | W | R | K | Q | I | A | V | L | A | K | T | - | Y | T | V | I | A | P | D | L | R |
| 101 UniRef90\_UPI00036BBA85\_7\_295 | P | E | - | - | - | - | - | D | - | - | - | A | P | - | P | - | V | V | L | L | H | G | W | P | E | T | W | Y | G | W | R | K | Q | I | P | V | L | A | E | K | - | Y | R | L | I | V | P | D | L | R |
| 102 UniRef90\_A0A4R5QBW7\_9\_278 | - | - | - | - | - | - | - | A | - | - | - | G | P | - | A | - | V | L | L | L | H | G | W | P | Q | S | W | R | M | W | E | A | I | I | P | G | L | A | A | R | - | H | R | V | V | A | P | D | L | R |
| 103 UniRef90\_UPI0012FB7881\_21\_292 | - | - | - | - | - | - | - | T | - | - | - | G | P | - | A | - | L | L | L | V | G | G | W | P | Q | F | W | W | Q | W | R | K | V | M | P | R | L | A | G | H | - | F | T | V | V | A | V | D | P | R |
| 104 UniRef90\_UPI00131B39C2\_13\_285 | - | - | - | - | - | - | - | Q | - | - | - | G | P | - | A | - | A | I | L | L | H | G | Y | G | E | T | G | D | M | W | A | P | L | A | T | E | L | A | H | D | - | H | T | V | I | A | P | D | L | R |
| 105 UniRef90\_M2VKF7\_34\_311 | - | - | - | - | - | - | - | E | - | - | - | G | E | - | P | - | L | L | L | H | C | G | W | P | Q | S | W | Y | A | W | R | Y | L | M | L | P | L | A | R | H | - | F | K | V | I | A | V | D | P | R |
| 106 UniRef90\_UPI0016124E74\_10\_283 | - | - | - | - | - | - | - | S | - | - | - | G | P | - | L | - | V | V | L | L | H | G | W | P | Q | T | S | Y | C | W | R | H | V | M | P | A | L | A | E | R | - | C | T | V | L | A | P | D | L | R |
| 107 UniRef90\_A0A263DFR4\_27\_299 | - | - | - | - | - | - | - | R | - | - | - | G | P | - | A | - | L | L | L | I | N | G | W | P | Q | T | W | Y | A | W | R | H | V | L | P | V | L | A | E | H | - | A | T | V | V | A | V | E | P | R |
| 108 UniRef90\_A0A0U5F3A6\_35\_313 | - | - | - | - | - | - | - | Q | - | - | - | G | P | - | A | - | L | L | L | L | G | G | W | P | Q | N | W | F | A | W | R | A | L | M | L | P | L | A | E | R | - | F | T | V | I | A | V | D | P | R |
| 109 UniRef90\_A0A2N3KZL1\_38\_324 | - | - | - | - | - | - | - | S | - | - | - | G | P | - | L | - | A | F | L | V | H | G | F | G | Q | S | W | Y | E | W | H | Q | L | M | P | L | L | A | K | T | - | H | S | V | V | A | V | D | L | P |
| 110 UniRef90\_A0A1X7C0M0\_25\_283 | - | - | - | - | - | - | - | D | - | - | - | G | P | - | V | - | L | V | L | L | H | G | W | P | Q | T | S | R | A | W | A | R | V | M | P | D | L | A | E | R | - | H | T | V | V | V | P | D | L | R |
| 111 UniRef90\_A0A078M0V2\_4\_284 | I | D | - | - | - | - | - | D | - | - | - | G | P | - | V | - | C | L | L | L | H | G | F | P | Q | S | W | I | T | W | R | N | V | I | P | H | L | E | E | H | - | Y | K | I | I | A | V | D | L | R |
| 112 UniRef90\_A0A0F4Z6H8\_20\_271 | - | - | - | - | - | - | - | - | - | - | - | - | - | - | - | - | - | - | L | S | T | G | I | P | K | T | S | Y | H | W | R | K | V | I | P | F | L | T | P | S | - | F | T | V | V | V | P | D | M | R |
| 113 UniRef90\_UPI00161D2319\_22\_292 | - | - | - | - | - | - | - | D | - | - | - | G | P | - | P | - | L | L | L | L | P | G | W | P | Q | F | W | Y | S | W | R | L | I | M | P | A | L | A | E | R | - | F | T | V | V | A | A | D | L | R |
| 114 UniRef90\_A0A1Q8LX67\_38\_327 | - | - | - | - | - | - | - | T | - | - | - | G | P | - | P | - | V | V | L | L | H | G | W | P | Q | S | W | Y | A | W | W | P | I | M | P | A | L | A | E | H | - | H | T | V | Y | A | V | D | L | P |
| 115 UniRef90\_A0A1Q9S2P6\_18\_309 | - | - | - | - | - | - | - | S | - | - | - | G | P | - | P | - | L | V | L | L | H | G | W | P | Q | T | W | F | A | W | R | D | V | M | P | A | L | A | D | H | - | F | T | V | Y | A | L | D | L | P |
| 116 UniRef90\_A0A5M7BQA6\_8\_281 | - | - | - | - | - | - | - | S | - | - | - | G | P | - | G | - | L | V | L | L | H | G | W | P | Q | T | S | H | C | W | R | K | L | I | P | E | L | A | R | T | - | H | T | V | I | A | P | D | L | R |
| 117 UniRef90\_A0A3N6GJS2\_33\_314 | - | - | - | - | - | - | - | E | - | - | - | G | T | - | P | - | L | V | L | L | P | G | W | P | E | T | W | W | E | F | R | K | V | M | P | A | L | A | A | A | G | R | R | V | I | V | L | D | L | P |
| 118 UniRef90\_A0A0Q6MZD2\_36\_308 | - | - | - | - | - | - | - | K | - | - | - | G | P | - | A | - | V | V | L | L | H | G | Y | G | E | T | G | D | M | W | V | P | L | A | A | D | L | A | R | D | - | H | T | V | I | V | P | D | L | R |
| 119 UniRef90\_UPI00052520C0\_22\_293 | - | - | - | - | - | - | - | D | - | - | - | G | P | - | P | - | L | L | L | V | G | G | W | P | Q | F | W | W | Q | W | R | K | I | M | P | R | L | A | E | D | - | Y | T | V | I | A | V | D | P | R |
| 120 UniRef90\_A0A1Q4XCF8\_2\_276 | - | - | - | - | - | - | - | E | - | - | - | G | R | - | P | - | L | V | L | L | P | G | W | P | Q | T | W | W | E | F | R | K | V | M | P | A | L | A | E | A | G | R | R | V | I | V | L | D | L | P |
| 121 UniRef90\_A0A328LZX6\_33\_305 | - | - | - | - | - | - | - | S | - | - | - | G | P | - | A | - | V | V | L | L | H | G | Y | G | E | T | G | D | M | W | A | P | L | A | A | E | L | A | R | N | - | H | R | V | I | V | P | D | L | R |
| 122 UniRef90\_A0A2V8S6L4\_40\_311 | - | - | - | - | - | - | - | K | - | - | - | G | S | - | P | - | V | V | L | L | H | G | F | A | E | T | G | H | M | W | L | P | I | M | P | L | L | A | K | S | - | H | T | V | V | V | P | D | L | R |
| 123 UniRef90\_A0A1I5EB88\_8\_281 | - | - | - | - | - | - | - | S | - | - | - | G | P | - | G | - | L | V | L | L | H | G | W | P | Q | T | S | H | C | W | R | K | L | I | P | E | L | A | R | T | - | H | T | V | I | A | P | D | L | R |
| 124 UniRef90\_UPI0014147ABD\_39\_305 | - | - | - | - | - | - | - | N | - | - | - | G | P | - | P | - | L | L | L | V | H | G | W | P | E | T | W | Y | A | W | R | L | L | M | P | A | L | A | R | D | - | F | E | V | V | A | V | D | Q | R |
| 125 UniRef90\_A0A1I7DL13\_6\_274 | - | - | - | - | - | - | - | H | - | - | - | G | D | E | T | - | I | V | L | L | H | G | W | P | Q | T | S | R | E | W | D | R | V | S | A | L | L | D | G | D | - | Y | R | L | I | V | P | D | L | R |
| 126 UniRef90\_A0A3N7EDS3\_31\_305 | - | - | - | - | - | - | - | T | - | - | - | G | P | - | A | - | V | T | L | L | H | G | Y | G | E | T | G | D | M | W | A | P | M | A | N | E | F | A | R | D | - | H | T | V | V | V | P | D | L | R |
| 127 UniRef90\_UPI0016225F9B\_4\_275 | - | - | - | - | - | - | - | E | - | - | - | G | P | - | L | - | L | M | L | V | H | G | W | P | Q | T | S | H | C | W | R | H | L | I | G | P | L | A | E | H | - | F | T | V | V | A | P | D | L | R |
| 128 UniRef90\_UPI000997AE50\_107\_382 | - | - | - | - | - | - | - | S | - | - | - | G | P | Q | T | - | T | V | L | L | H | G | W | P | E | N | W | Y | A | Y | R | G | I | M | P | K | L | L | P | G | - | R | T | V | I | A | V | D | L | P |
| 129 UniRef90\_A0A0N7F3H5\_6\_275 | - | - | - | - | - | - | - | E | - | - | - | G | P | - | P | - | V | V | L | L | H | G | W | P | Q | T | S | H | C | W | H | K | I | F | D | D | L | A | D | K | - | H | T | V | I | A | P | D | L | R |
| 130 UniRef90\_A0A3Q8F441\_11\_281 | - | - | - | - | - | - | - | N | - | - | - | G | A | - | P | - | V | V | L | L | H | G | F | P | E | T | N | Y | A | W | R | H | Q | I | P | V | L | A | A | N | - | H | R | V | I | A | P | D | L | R |
| 131 UniRef90\_UPI001487D2FB\_23\_300 | - | - | - | - | - | - | - | K | - | - | - | G | P | - | A | - | V | V | L | L | H | G | F | G | D | T | G | A | M | W | S | P | L | A | A | E | L | A | A | R | - | H | T | V | V | V | P | D | L | R |
| 132 UniRef90\_A0A437GNC7\_38\_307 | - | - | - | - | - | - | - | E | - | - | - | G | P | - | P | - | L | L | L | L | G | G | W | P | Q | S | W | Y | I | W | R | D | V | M | L | P | L | A | E | R | - | F | T | L | V | V | P | D | P | R |
| 133 UniRef90\_A0A1H3SWJ7\_6\_283 | - | - | - | - | - | - | - | E | - | - | - | G | P | - | L | - | I | L | F | L | H | G | W | P | Q | T | S | Y | A | W | R | R | V | L | P | V | L | A | R | D | - | H | T | V | V | A | P | D | L | R |
| 134 UniRef90\_UPI0013DD6281\_49\_329 | - | - | - | - | - | - | - | T | - | - | - | G | K | - | P | - | L | V | L | L | H | G | W | P | Q | S | W | Y | A | W | R | G | I | M | P | A | L | A | E | R | - | H | T | V | Y | A | L | D | L | P |
| 135 UniRef90\_I0L7C1\_27\_272 | - | - | - | - | - | - | - | T | - | - | - | G | P | - | P | - | V | L | L | M | H | G | W | P | H | T | W | Q | V | W | R | E | V | L | P | L | L | G | G | R | - | R | R | V | I | A | P | D | L | R |
| 136 UniRef90\_A0A260DLM1\_45\_326 | - | - | - | - | - | - | - | T | - | - | - | G | P | - | A | - | M | V | L | L | H | G | W | P | Q | T | W | F | E | W | R | E | I | M | P | A | L | A | R | T | - | H | T | V | Y | A | V | D | L | P |
| 137 UniRef90\_UPI0005BDD2EC\_7\_280 | - | - | - | - | - | - | - | S | - | - | - | G | P | - | L | - | V | V | L | L | H | G | W | P | Q | T | G | Y | C | W | R | K | I | T | D | E | L | A | R | T | - | H | T | V | V | A | P | D | L | R |
| 138 UniRef90\_A0A6G9YBC1\_27\_296 | - | - | - | - | - | - | - | S | - | - | - | G | P | - | A | - | L | V | L | L | H | G | W | P | Q | T | G | L | C | W | H | R | I | A | P | D | L | A | A | T | - | H | T | V | I | A | P | D | L | R |
| 139 UniRef90\_UPI00145FA88A\_8\_280 | - | - | - | - | - | - | - | D | - | - | - | G | P | - | P | - | L | V | L | L | H | G | W | P | Q | T | S | H | C | W | R | F | I | A | G | P | L | C | E | T | - | H | T | V | I | V | P | D | L | R |
| 140 UniRef90\_A0A2W0B1K8\_46\_299 | - | - | - | - | - | - | - | K | - | - | - | G | P | - | V | - | V | V | L | I | H | G | Y | A | E | N | S | D | S | W | A | P | L | A | A | D | L | M | K | D | - | H | T | V | V | V | P | D | L | R |
| 141 UniRef90\_A0A5B8G622\_28\_299 | - | - | - | - | - | - | - | S | - | - | - | G | P | - | A | - | V | V | L | L | H | G | Y | G | E | T | G | D | M | W | S | P | L | A | E | D | L | A | R | D | - | H | T | V | I | V | P | D | L | R |
| 142 UniRef90\_A0A4S1XGQ8\_21\_302 | - | - | - | - | - | - | - | K | - | - | - | G | P | - | A | - | V | L | L | L | H | G | Y | G | E | T | G | D | M | W | A | P | L | A | A | A | L | M | R | D | - | H | Q | V | I | V | P | D | L | R |
| 143 UniRef90\_A0A251YJ72\_8\_294 | - | - | - | - | - | - | - | S | T | - | - | G | S | - | T | - | V | L | L | V | H | G | F | P | E | S | W | W | A | F | H | R | L | I | P | L | L | A | S | E | - | H | R | V | V | A | V | D | L | R |
| 144 UniRef90\_UPI000A0527D2\_65\_318 | - | - | - | - | - | - | - | D | - | - | - | R | A | - | T | - | V | M | L | L | H | G | A | P | Q | T | S | H | A | W | R | K | V | V | V | P | L | A | A | A | G | Y | R | V | I | A | P | D | F | R |
| 145 UniRef90\_A0A4V2S7H9\_8\_280 | - | - | - | - | - | - | - | D | - | - | - | G | P | - | A | - | L | V | L | L | H | G | W | P | Q | T | S | Y | C | W | H | K | V | I | E | P | L | A | R | T | - | H | T | V | I | A | P | D | L | R |
| 146 UniRef90\_UPI00131B216A\_18\_247 | - | - | - | - | - | - | - | Q | - | - | - | G | D | - | P | - | L | L | L | L | G | G | W | P | Q | T | W | W | Q | F | R | K | I | M | P | S | L | A | Q | R | - | H | Q | V | I | A | V | D | L | R |
| 147 UniRef90\_A0A1G8T9Y6\_5\_243 | - | - | - | - | - | - | - | - | - | - | - | - | - | - | - | - | - | - | - | - | - | - | - | - | - | - | - | - | - | - | - | - | I | L | P | A | L | A | K | H | - | Y | T | V | I | A | P | D | L | R |
| 148 UniRef90\_UPI00161F613F\_5\_254 | - | - | - | - | - | - | - | T | R | - | - | G | E | - | P | - | I | L | L | V | H | G | F | P | E | T | S | Y | A | F | H | R | V | M | P | L | L | A | G | N | - | H | R | V | F | A | V | D | L | R |
| 149 UniRef90\_UPI00142071FA\_80\_362 | - | - | - | - | - | - | - | G | - | - | - | G | P | - | P | - | L | L | L | I | H | G | W | P | E | N | W | Y | A | W | R | F | L | M | P | A | L | A | R | D | - | F | T | V | I | V | P | D | Q | R |
| 150 UniRef90\_A0A6G7Q8F6\_7\_219 | P | G | - | - | - | - | - | D | - | - | - | G | P | - | P | - | T | I | L | L | H | G | F | P | E | F | W | F | G | W | R | H | Q | I | A | P | L | A | G | N | G | L | R | L | L | I | P | D | Q | R |

  
  

|  |  |  |  |  |  |  |  |  |  |  |  |  |  |  |  |  |  |  |  |  |  |  |  |  |  |  |  |  |  |  |  |  |  |  |  |  |  |  |  |  |  |  |  |  |  |  |  |  |  |  |
| --- | --- | --- | --- | --- | --- | --- | --- | --- | --- | --- | --- | --- | --- | --- | --- | --- | --- | --- | --- | --- | --- | --- | --- | --- | --- | --- | --- | --- | --- | --- | --- | --- | --- | --- | --- | --- | --- | --- | --- | --- | --- | --- | --- | --- | --- | --- | --- | --- | --- | --- |
| **001 Input\_protein\_seq** | G | Q | G | D | S | D | R | P | L | - | - | V | G | Y | D | T | Q | T | V | A | A | T | L | A | R | L | L | - | E | R | Q | N | I | A | R | - | - | - | F | Y | L | A | A | H | D | V | G | A | W | V |
| 002 UniRef90\_UPI001430CB85\_4\_307 | G | Q | G | D | S | D | R | P | L | - | - | D | G | Y | D | T | K | S | L | A | T | V | L | H | A | L | L | - | G | K | L | G | I | Q | R | - | - | - | Y | Y | L | A | A | H | D | V | G | A | W | V |
| 003 UniRef90\_UPI00055F8D66\_10\_306 | G | Q | G | D | S | D | R | P | A | - | - | V | G | Y | D | T | K | T | L | A | I | T | V | H | K | F | L | - | E | Q | L | G | T | K | R | - | - | - | Y | F | L | A | A | H | D | V | G | A | W | V |
| 004 UniRef90\_UPI00161D8BA7\_15\_312 | G | Q | G | D | S | D | R | P | L | - | - | D | G | Y | D | T | Q | T | L | A | I | K | V | H | G | L | L | - | E | Q | L | N | I | K | R | - | - | - | Y | F | M | A | A | H | D | V | G | A | W | V |
| 005 UniRef90\_A0A4R7GLU7\_7\_305 | G | Q | G | D | S | D | R | P | E | - | - | S | G | Y | D | T | Q | N | L | A | Q | K | I | H | G | L | M | - | Q | L | L | G | H | S | H | - | - | - | Y | S | L | A | A | H | D | V | G | A | W | V |
| 006 UniRef90\_UPI00041CD164\_24\_309 | G | Q | G | D | S | D | H | P | E | - | - | G | G | Y | D | T | Q | S | L | A | I | A | V | H | G | L | L | - | H | Q | I | G | A | S | R | - | - | - | Y | F | L | A | A | H | D | I | G | A | W | V |
| 007 UniRef90\_I0QSF8\_15\_298 | G | Q | G | D | S | D | K | P | L | - | - | E | G | Y | D | T | Q | S | L | A | T | A | V | H | S | L | L | - | Q | Q | L | D | V | K | D | - | - | - | Y | Y | L | A | A | H | D | V | G | A | W | V |
| 008 UniRef90\_A0A2N5ENH7\_9\_301 | G | Q | G | D | S | D | K | P | Q | - | - | S | G | Y | D | T | L | A | L | A | A | K | V | Q | G | L | M | - | H | T | L | E | R | G | R | - | - | - | Y | Y | L | A | A | H | D | V | G | A | W | V |
| 009 UniRef90\_A0A0Q8E2M6\_29\_312 | G | Q | G | D | S | D | R | L | D | - | - | G | G | Y | D | T | D | A | L | A | G | K | V | H | A | L | L | - | Q | Q | L | G | V | H | R | - | - | - | Y | G | L | V | A | H | D | V | G | A | W | V |
| 010 UniRef90\_A0A0F5XY46\_6\_287 | G | Q | G | D | S | D | F | P | A | - | - | E | G | Y | D | T | D | T | V | A | Q | R | V | M | A | L | L | - | Q | Q | L | G | I | T | R | - | - | - | F | H | L | V | G | H | D | I | G | A | W | V |
| 011 UniRef90\_A0A3N4NUX5\_4\_284 | G | Q | G | D | S | D | L | P | Q | - | - | Q | G | Y | D | T | G | T | L | A | G | H | I | D | A | L | L | - | Q | Q | L | N | L | P | A | - | - | - | V | F | L | A | G | H | D | V | G | A | W | V |
| 012 UniRef90\_UPI000DDED2D9\_11\_293 | G | Q | G | D | S | D | R | P | L | - | - | R | G | C | D | T | L | A | I | A | R | L | M | R | A | V | V | - | E | R | L | G | V | E | T | - | - | - | H | Y | L | A | A | H | D | I | G | A | W | V |
| 013 UniRef90\_UPI000DDED53B\_10\_292 | G | Q | G | D | S | D | R | P | T | - | - | Y | G | Y | D | T | Q | T | V | A | E | R | V | H | D | L | V | - | N | H | L | G | F | K | R | - | - | - | Y | G | L | A | A | H | D | V | G | A | W | V |
| 014 UniRef90\_A0A1G4JKM0\_6\_288 | G | Q | G | D | S | D | K | P | A | - | - | D | G | Y | D | T | L | T | V | S | N | I | V | H | E | V | I | - | R | K | L | E | M | T | H | - | - | - | Y | F | L | A | A | H | D | I | G | A | W | V |
| 015 UniRef90\_UPI0016728815\_21\_305 | G | Q | G | H | S | A | R | P | D | - | - | I | S | Y | D | T | H | T | V | A | A | H | V | H | A | A | V | - | E | A | L | G | V | S | A | - | - | - | Y | R | L | A | A | H | D | I | G | A | W | V |
| 016 UniRef90\_A0A3N2H8D2\_14\_297 | G | Q | G | H | S | E | R | P | E | - | - | R | G | Y | D | T | H | T | A | A | A | H | V | H | A | A | V | - | K | A | L | G | V | S | S | - | - | - | Y | C | L | V | A | H | D | I | G | A | W | V |
| 017 UniRef90\_UPI0005644DD1\_9\_307 | G | Q | G | H | S | E | R | P | E | - | - | L | S | Y | D | T | H | T | V | A | A | H | V | H | A | A | V | - | K | A | L | G | V | S | T | - | - | - | Y | W | L | A | A | H | D | I | G | A | W | V |
| 018 UniRef90\_A0A6B2VDS0\_18\_308 | G | Q | G | H | S | E | R | P | E | - | - | R | S | Y | A | T | H | A | V | A | A | H | V | H | T | A | V | - | K | A | L | G | V | S | A | - | - | - | Y | S | L | I | A | H | D | I | G | A | W | V |
| 019 UniRef90\_A0A6G3RZZ1\_25\_307 | G | Q | G | H | S | E | R | P | Q | - | - | G | A | Y | D | T | H | T | A | A | S | R | V | Q | A | A | L | - | D | A | L | G | V | P | T | - | - | - | Y | W | L | V | A | H | D | I | G | A | W | V |
| 020 UniRef90\_A0A209C7X3\_21\_305 | G | Q | G | H | S | E | R | P | D | - | - | I | S | Y | D | T | H | T | V | A | A | H | V | H | A | A | V | - | E | A | L | G | V | S | T | - | - | - | Y | W | L | V | A | H | D | I | G | A | W | V |
| 021 UniRef90\_UPI001269E708\_26\_309 | G | Q | G | H | S | E | R | P | E | - | - | S | G | Y | D | T | H | T | V | A | R | R | V | H | A | A | V | - | R | I | L | G | A | S | T | - | - | - | Y | W | L | V | A | H | D | I | G | A | W | V |
| 022 UniRef90\_A0A2M9J7W9\_25\_306 | G | Q | G | H | S | E | R | P | R | - | - | G | S | Y | D | T | H | T | V | A | S | R | V | Q | A | A | L | - | T | A | L | D | V | S | T | - | - | - | Y | W | L | A | A | H | D | I | G | A | W | V |
| 023 UniRef90\_A0A6G9F3J8\_23\_307 | G | Q | G | H | S | E | R | P | D | - | - | G | S | Y | D | T | H | T | V | A | A | Y | V | H | A | A | V | - | R | A | L | G | V | P | S | - | - | - | Y | W | L | A | A | H | D | I | G | A | W | V |
| 024 UniRef90\_A0A1H2UZ41\_14\_292 | G | Q | G | D | S | D | K | P | I | - | - | D | G | Y | D | T | R | T | T | G | D | R | V | H | A | L | A | - | K | T | L | G | F | E | R | - | - | - | Y | H | I | G | S | H | D | I | G | S | W | V |
| 025 UniRef90\_UPI0012B05B7D\_16\_286 | G | Q | G | D | S | D | K | P | P | - | - | G | G | Y | D | T | Q | T | T | A | E | R | I | H | G | F | V | - | E | K | L | G | L | E | N | - | - | - | F | L | Y | V | G | H | D | I | G | A | W | V |
| 026 UniRef90\_A0A5C8T429\_18\_294 | G | M | G | D | S | D | K | P | I | - | - | D | G | Y | D | T | G | T | V | A | M | R | L | H | D | L | T | - | R | A | L | G | W | R | Q | - | - | - | F | D | F | V | G | H | D | I | G | C | W | L |
| 027 UniRef90\_UPI0012E86E3E\_10\_309 | G | M | G | D | S | D | R | P | A | - | - | S | G | Y | E | M | R | T | A | A | A | D | L | H | G | F | V | - | E | A | L | G | L | T | V | N | G | P | L | D | V | V | G | H | D | V | G | T | W | I |
| 028 UniRef90\_A0A1A9HTL2\_13\_300 | G | M | G | D | S | D | H | P | P | - | - | E | G | Y | D | S | K | T | I | S | A | D | I | H | A | F | V | - | A | A | K | G | L | A | K | K | G | N | L | H | V | A | G | H | D | V | G | A | W | M |
| 029 UniRef90\_A0A0R1V0C7\_7\_281 | G | Q | G | Y | S | S | V | P | R | - | - | S | G | Y | D | T | K | T | T | A | H | R | I | H | E | L | V | - | I | Q | L | G | L | K | K | - | - | - | I | L | Y | V | G | H | D | V | G | S | W | V |
| 030 UniRef90\_A0A3N1M9Y1\_5\_282 | G | L | G | D | S | S | R | P | A | - | - | D | G | Y | D | K | R | T | V | A | A | D | V | W | E | L | V | - | E | H | L | G | L | G | R | - | - | - | F | H | L | V | G | H | D | W | G | G | P | T |
| 031 UniRef90\_A0A158JUX7\_17\_294 | G | M | G | D | S | S | K | P | L | - | - | E | G | Y | D | K | K | S | V | A | T | D | L | H | E | L | V | - | R | S | L | G | F | D | R | - | - | - | V | L | L | V | G | H | D | M | G | G | Q | V |
| 032 UniRef90\_A0A0U3LIL8\_12\_296 | G | M | G | D | S | A | H | P | Q | - | - | D | G | F | T | P | G | I | V | A | A | E | I | H | R | F | A | - | Q | L | T | G | L | L | E | A | G | K | L | D | V | A | G | H | D | V | G | A | W | I |
| 033 UniRef90\_A0A248JS33\_7\_301 | G | F | G | E | S | D | R | P | A | - | - | G | G | Y | D | L | G | T | A | A | A | D | M | H | G | L | V | - | D | V | L | G | L | L | K | A | G | P | L | D | V | V | G | H | D | V | G | T | W | I |
| 034 UniRef90\_A0A1Y0REZ4\_12\_287 | G | M | G | D | S | S | K | P | S | - | - | A | G | Y | D | K | R | T | V | A | E | D | I | H | Q | L | V | - | H | L | L | G | F | Q | Q | - | - | - | T | F | L | I | G | H | D | I | G | A | M | V |
| 035 UniRef90\_A0A0M2WRU5\_3\_280 | G | S | G | H | S | D | R | P | A | - | - | G | G | Y | D | T | G | A | V | A | T | T | L | H | R | A | M | - | L | A | L | G | H | A | Q | - | - | - | Y | D | V | V | G | H | D | I | G | M | W | V |
| 036 UniRef90\_A0A0N8GFM1\_6\_279 | G | L | G | D | S | S | R | P | A | - | - | T | G | Y | D | K | K | T | I | A | A | D | L | W | A | L | T | - | R | R | L | G | I | D | R | - | - | - | F | H | L | A | G | H | D | W | G | G | P | V |
| 037 UniRef90\_A0A252EMP1\_7\_277 | G | A | G | D | S | S | R | P | A | - | - | S | G | Y | D | S | N | T | V | A | D | E | L | H | A | L | V | - | R | H | L | G | F | A | S | - | - | - | I | R | L | V | A | H | D | N | G | A | R | V |
| 038 UniRef90\_W0A6K5\_40\_320 | G | T | G | Y | S | D | R | P | D | - | - | R | G | Y | D | T | G | A | V | A | T | T | L | H | G | M | M | - | N | Q | L | G | H | K | K | - | - | - | Y | S | V | V | G | H | D | I | G | M | W | V |
| 039 UniRef90\_A0A3S1SM66\_23\_304 | G | T | G | Q | S | D | R | P | A | - | - | H | G | Y | D | L | M | T | A | A | A | D | V | H | A | I | A | - | E | A | L | G | L | L | S | D | G | P | I | D | V | A | G | H | D | V | G | T | W | I |
| 040 UniRef90\_A0A2E5L5C6\_7\_286 | G | L | G | D | S | S | R | P | L | - | - | N | G | Y | D | K | K | T | V | A | N | D | I | W | R | L | V | N | E | I | L | G | Y | K | S | - | - | - | F | F | L | V | G | H | D | W | G | G | P | T |
| 041 UniRef90\_M5D1B4\_14\_289 | G | I | G | E | S | D | R | P | V | - | - | H | G | Y | D | T | G | S | A | A | A | V | L | H | Q | T | M | - | Q | A | L | G | H | E | R | - | - | - | Y | Q | V | V | G | H | D | I | G | M | W | I |
| 042 UniRef90\_UPI0016845BA7\_31\_303 | G | L | G | D | S | S | K | P | D | - | - | S | G | Y | D | K | R | T | M | A | E | D | I | Y | Q | L | V | - | Q | H | L | G | F | D | D | - | - | - | I | S | L | V | G | H | D | I | G | G | Q | I |
| 043 UniRef90\_UPI0014648199\_14\_291 | G | L | G | N | S | D | R | P | E | - | - | R | G | Y | D | T | G | N | I | A | D | V | L | H | R | T | M | - | Q | G | L | G | L | T | R | - | - | - | Y | H | V | V | G | H | D | V | G | M | W | V |
| 044 UniRef90\_K9RUM7\_12\_289 | G | Q | G | D | S | D | I | P | A | - | - | G | P | Y | D | C | G | T | A | A | Q | E | I | K | D | F | L | - | A | L | K | Q | I | N | Q | - | - | - | F | Y | L | V | G | H | D | V | G | A | W | V |
| 045 UniRef90\_A0A1Q2U086\_13\_290 | G | Q | G | D | S | N | I | P | E | - | - | G | S | Y | D | C | G | T | A | A | Q | E | I | I | A | Y | L | - | Q | Q | K | G | I | T | S | - | - | - | F | Y | L | V | G | H | D | V | G | A | W | V |
| 046 UniRef90\_B9JMN1\_3\_282 | G | F | G | A | T | S | K | A | E | - | - | A | G | Y | D | T | N | N | V | A | D | D | I | R | E | L | V | - | G | S | L | G | F | E | R | - | - | - | I | F | L | A | G | H | D | W | G | A | A | V |
| 047 UniRef90\_A0A2X1TAZ7\_33\_319 | G | T | G | Y | S | A | R | P | E | - | - | S | G | Y | D | T | G | A | V | A | M | V | L | H | T | M | M | - | H | Q | L | G | Y | K | T | - | - | - | Y | S | V | V | G | H | D | I | G | M | W | V |
| 048 UniRef90\_UPI00131A8152\_6\_279 | G | L | G | H | S | L | S | K | T | - | - | E | R | Y | D | K | R | S | I | A | A | D | I | V | E | L | T | H | L | H | L | G | L | G | K | - | - | - | F | N | L | V | G | H | D | W | G | G | G | V |
| 049 UniRef90\_F7YB04\_47\_316 | G | L | G | D | T | A | K | P | A | - | - | A | G | Y | E | K | A | A | I | A | E | D | I | R | A | L | V | - | N | Q | L | N | L | G | P | - | - | V | V | N | L | V | G | H | D | M | G | G | M | V |
| 050 UniRef90\_A0A1A3M1Z8\_5\_294 | G | A | G | G | S | S | K | P | S | - | - | S | G | Y | D | K | Q | T | M | A | G | D | I | K | G | L | L | - | E | H | L | G | V | Q | K | - | - | P | I | T | L | V | G | H | D | L | G | L | M | V |
| 051 UniRef90\_UPI0004C32CE1\_32\_312 | G | M | G | G | S | E | K | P | A | - | - | G | G | Y | D | K | K | T | M | A | A | D | I | H | A | L | V | - | K | H | L | G | Y | E | K | - | - | - | V | G | L | A | G | H | D | I | G | S | Q | V |
| 052 UniRef90\_A0A0J6NQJ9\_12\_287 | G | T | G | V | S | S | R | P | D | - | - | S | G | Y | D | T | G | A | V | A | A | A | L | H | Q | V | M | - | L | R | L | G | H | A | R | - | - | - | Y | Q | V | V | G | H | D | V | G | M | W | L |
| 053 UniRef90\_A0A534ESC2\_32\_321 | G | F | G | D | S | D | K | P | Q | - | - | S | G | Y | D | L | D | T | A | A | R | D | L | H | G | F | L | - | A | A | T | G | L | I | R | P | A | G | V | D | I | V | A | H | D | V | G | T | W | I |
| 054 UniRef90\_A0A1H1Z302\_21\_301 | G | S | G | L | S | G | K | P | E | - | - | G | G | Y | D | A | G | T | L | A | A | D | M | V | A | L | M | - | A | A | L | G | H | D | R | - | - | - | F | D | V | V | G | H | D | I | G | M | W | T |
| 055 UniRef90\_A0A158DLE6\_24\_295 | G | F | G | D | S | S | K | P | A | - | - | T | G | Y | D | K | K | T | V | A | S | D | L | A | A | L | L | - | D | A | L | H | I | D | K | - | - | - | A | C | V | V | G | H | D | M | G | G | Q | V |
| 056 UniRef90\_A0A163VVR8\_16\_280 | G | I | G | D | S | D | K | P | A | - | - | G | G | Y | D | T | G | N | L | G | A | T | L | H | A | M | M | - | A | Q | L | G | H | E | R | - | - | - | Y | Q | L | V | G | H | D | I | G | M | W | I |
| 057 UniRef90\_UPI000369F0DF\_7\_289 | G | S | G | Y | S | S | R | P | E | - | - | R | G | Y | D | T | G | S | A | A | R | L | L | H | R | L | M | - | I | Q | L | G | H | D | R | - | - | - | Y | R | V | A | G | H | D | V | G | M | W | I |
| 058 UniRef90\_UPI00098F4FB4\_43\_309 | G | L | G | D | T | A | K | P | V | - | - | D | G | Y | E | K | A | L | I | A | E | D | I | R | K | L | L | - | R | N | L | D | V | G | P | - | - | V | V | N | L | V | G | H | D | M | G | G | M | V |
| 059 UniRef90\_UPI0015A1AD76\_10\_292 | G | V | G | G | S | S | I | L | P | - | - | T | G | Y | D | K | R | T | L | A | A | D | V | Y | G | L | M | - | T | Q | L | G | H | A | E | - | - | - | I | N | L | V | G | Y | D | L | G | A | G | V |
| 060 UniRef90\_UPI001689F051\_63\_318 | G | F | G | A | S | S | R | L | A | - | - | S | G | Y | D | K | K | S | I | A | G | I | L | H | R | L | M | - | L | R | L | G | F | Q | R | - | - | - | Y | H | V | A | G | H | D | V | G | G | Q | V |
| 061 UniRef90\_UPI000DD77D66\_43\_313 | G | L | G | D | T | A | K | P | A | - | - | A | G | Y | E | K | A | A | V | A | Q | D | I | W | K | L | V | - | N | N | L | D | L | G | P | - | - | T | V | N | L | V | G | H | D | M | G | G | M | V |
| 062 UniRef90\_A0A3M0I8T4\_31\_307 | G | V | G | L | S | D | K | P | R | - | - | D | G | Y | D | T | A | T | L | A | D | D | M | A | E | L | M | - | T | A | L | G | H | E | R | - | - | - | F | A | M | V | G | H | D | V | G | M | W | T |
| 063 UniRef90\_UPI000377E2D1\_4\_271 | G | L | G | D | S | T | R | P | L | - | - | H | G | Y | D | K | Q | T | L | G | E | D | L | V | R | L | L | - | D | A | L | G | I | D | R | - | - | - | C | A | V | V | G | H | D | W | G | G | V | V |
| 064 UniRef90\_UPI0016198B53\_15\_286 | G | V | G | R | S | D | K | P | H | - | - | G | G | Y | D | T | A | T | V | A | A | E | L | V | A | L | M | - | R | T | L | G | H | D | R | - | - | - | F | T | M | A | G | H | D | V | G | M | W | I |
| 065 UniRef90\_UPI0013C0217D\_48\_325 | G | M | G | G | S | D | K | P | A | - | - | G | G | Y | D | K | K | T | M | A | G | D | L | Y | A | L | V | - | R | Q | L | G | H | C | R | - | - | - | V | N | I | A | G | H | D | I | G | S | K | V |
| 066 UniRef90\_C7PGN2\_5\_260 | G | L | G | D | T | A | N | P | V | - | - | - | S | F | D | K | K | T | I | A | V | L | I | H | D | M | M | - | K | A | D | G | Y | N | D | - | - | - | I | Y | L | V | G | H | D | W | G | A | S | V |
| 067 UniRef90\_A0A1F7Q7Q2\_7\_275 | G | Y | G | D | S | D | R | P | A | - | - | A | G | Y | D | K | R | T | M | A | A | D | V | A | D | V | I | - | A | A | L | G | L | A | P | - | - | - | V | T | L | V | G | H | D | R | G | A | R | V |
| 068 UniRef90\_A0A2V7F1K9\_9\_274 | G | Y | G | D | S | D | R | P | A | - | - | T | G | Y | D | K | R | T | M | A | A | D | I | A | E | V | I | - | T | A | L | T | L | A | P | - | - | - | V | I | L | V | G | H | D | R | G | A | R | V |
| 069 UniRef90\_UPI001616C592\_31\_308 | G | A | G | L | S | D | K | P | A | - | - | E | G | Y | D | S | G | T | L | A | R | E | T | H | R | L | M | - | Q | V | L | G | Y | D | R | - | - | - | F | A | M | I | A | H | D | V | G | G | W | T |
| 070 UniRef90\_UPI001456849A\_71\_360 | G | L | G | D | S | S | R | P | E | - | - | A | G | Y | D | K | D | Q | I | G | E | D | I | V | K | L | A | - | S | Q | L | G | L | T | R | - | - | - | F | S | V | A | G | H | D | L | G | G | Q | V |
| 071 UniRef90\_A0A2S8J6S6\_18\_282 | G | A | G | N | S | S | K | P | L | - | - | I | G | Y | D | K | V | T | M | A | A | E | L | D | G | L | R | - | Q | A | L | G | F | E | T | - | - | - | V | Q | V | V | G | H | D | I | G | G | M | V |
| 072 UniRef90\_A0A2I2GC12\_18\_280 | G | L | G | D | S | A | P | S | P | - | - | N | G | Y | D | T | A | A | I | S | K | T | L | A | E | S | L | - | N | D | V | I | K | E | P | - | - | - | Y | H | L | V | G | H | D | I | G | G | W | I |
| 073 UniRef90\_A0A549T469\_41\_312 | G | V | G | L | S | D | R | P | P | - | - | S | G | Y | D | K | R | R | I | A | E | D | I | A | A | L | I | - | D | R | A | A | G | G | R | - | - | - | A | Y | V | V | G | H | D | M | G | G | K | A |
| 074 UniRef90\_A0A5C4LJC6\_5\_278 | G | A | G | F | S | D | C | P | L | - | - | D | G | Y | D | K | A | T | M | A | R | D | A | H | E | V | M | - | V | A | L | G | H | E | R | - | - | - | Y | A | V | C | G | H | D | I | G | G | M | V |
| 075 UniRef90\_UPI001619D806\_9\_283 | G | Y | G | R | T | D | K | P | L | - | - | G | G | Y | D | K | R | T | M | A | G | D | V | S | E | L | V | - | S | G | L | G | F | D | R | - | - | - | V | A | V | A | G | H | D | R | G | G | R | V |
| 076 UniRef90\_A0A239P1A5\_69\_347 | G | V | G | L | S | S | K | P | A | - | - | S | G | Y | D | S | R | T | L | A | G | D | A | V | K | L | M | - | K | A | L | G | H | D | R | - | - | - | F | A | L | V | T | H | D | V | G | S | W | T |
| 077 UniRef90\_UPI0005262A70\_5\_280 | G | F | G | D | S | - | - | A | V | - | - | G | D | F | T | S | A | T | M | A | E | S | L | C | A | L | I | - | V | H | L | G | L | G | P | - | - | - | V | H | L | T | G | Q | D | I | S | G | T | P |
| 078 UniRef90\_A0A241XUQ6\_27\_314 | G | L | G | Q | S | E | P | P | K | - | - | T | G | Y | S | G | E | Q | V | A | V | Y | L | H | K | L | A | - | R | Q | F | S | P | D | R | - | - | P | F | D | L | V | A | H | D | I | G | I | W | N |
| 079 UniRef90\_A0A160FQ65\_32\_312 | G | V | G | L | S | D | K | P | T | - | - | S | G | Y | D | S | R | T | L | S | A | D | M | F | A | L | M | - | D | T | L | G | Y | E | H | - | - | - | F | A | M | A | G | H | D | I | G | M | W | T |
| 080 UniRef90\_UPI0006AE4C23\_4\_281 | G | M | G | D | S | E | K | P | E | - | - | A | G | Y | G | K | A | N | M | A | Q | D | I | Y | E | L | V | - | R | H | L | G | Y | D | Q | - | - | - | V | N | I | A | G | E | D | V | G | S | W | I |
| 081 UniRef90\_T0H3T7\_11\_281 | G | Y | G | E | T | D | K | P | A | - | - | T | G | Y | D | K | R | N | M | A | K | D | L | A | A | L | L | - | D | E | L | G | I | D | R | - | - | - | I | A | L | V | G | H | D | R | G | A | R | V |
| 082 UniRef90\_A0A4R2IH15\_5\_277 | G | V | G | L | S | S | K | P | H | - | - | T | G | Y | D | T | G | T | L | A | R | D | M | V | E | L | M | - | D | A | L | G | H | E | R | - | - | - | F | A | V | V | G | H | D | I | G | M | W | I |
| 083 UniRef90\_UPI00140B863E\_4\_276 | G | L | G | D | S | D | R | P | A | - | - | G | G | Y | D | K | R | T | L | A | D | D | V | V | A | V | A | - | A | H | V | G | A | D | R | - | - | - | F | A | L | V | G | H | D | F | G | G | S | V |
| 084 UniRef90\_A0A3R9UXB7\_22\_297 | G | M | G | G | S | A | K | P | E | - | - | T | G | Y | D | K | R | T | M | A | A | D | V | H | A | L | L | - | G | R | L | G | I | A | S | - | - | - | A | H | L | V | G | H | D | I | G | A | M | V |
| 085 UniRef90\_UPI000F8D1465\_20\_303 | G | L | G | R | S | D | K | P | D | - | - | G | A | Y | D | T | A | A | L | A | G | D | L | A | G | L | M | - | S | T | L | G | H | E | R | - | - | - | F | A | V | V | G | H | D | V | G | M | W | V |
| 086 UniRef90\_A0A6J4HYT7\_23\_307 | G | M | G | S | S | E | K | P | P | - | - | G | G | Y | D | K | K | T | M | A | A | D | V | H | A | L | I | - | R | H | L | G | Y | E | R | - | - | - | V | H | V | A | G | H | D | I | G | A | H | V |
| 087 UniRef90\_A0A1D8SMR3\_19\_296 | G | M | G | A | S | S | K | P | A | - | - | G | G | Y | D | A | V | T | L | A | D | E | M | T | A | L | M | - | A | S | L | G | H | D | S | - | - | - | F | H | V | A | G | Y | D | M | G | M | M | V |
| 088 UniRef90\_A0A4V2HUZ4\_48\_320 | G | S | G | L | S | E | R | T | D | - | - | A | G | Y | D | K | Q | T | I | A | E | D | I | R | A | L | I | - | A | H | L | G | P | A | P | - | - | - | A | H | V | V | G | H | D | M | G | G | K | A |
| 089 UniRef90\_UPI0010582A1B\_5\_279 | G | A | G | F | S | D | C | P | L | - | - | D | G | Y | D | K | A | T | M | A | R | D | A | H | Q | V | M | - | V | A | L | G | H | G | R | - | - | - | Y | A | V | C | G | H | D | I | G | G | M | V |
| 090 UniRef90\_UPI00166CE541\_54\_338 | G | A | G | K | S | D | A | P | A | - | - | S | G | Y | D | K | K | T | L | A | A | D | L | H | G | L | L | - | T | T | L | G | L | K | L | - | - | H | I | R | L | V | G | H | D | I | G | T | M | V |
| 091 UniRef90\_A0A5C1HX54\_34\_307 | G | V | G | E | S | A | K | P | V | - | - | G | G | Y | D | K | K | T | M | S | T | D | L | H | E | L | M | - | K | S | L | G | F | K | N | - | - | - | I | N | L | V | G | H | D | I | G | L | M | V |
| 092 UniRef90\_A0A4D4KET2\_34\_306 | G | V | G | L | S | G | K | P | L | - | - | D | G | Y | D | T | G | T | L | A | T | D | M | V | A | L | M | - | E | A | L | G | H | R | R | - | - | - | F | A | M | V | G | H | D | V | G | M | W | T |
| 093 UniRef90\_A0A1I1XP17\_9\_276 | G | A | G | H | S | S | K | P | P | - | - | A | G | Y | D | K | W | T | I | A | A | D | I | H | A | L | L | - | E | Q | A | A | A | E | E | - | - | R | I | A | L | V | G | H | D | I | G | A | M | V |
| 094 UniRef90\_A0A1D7VX60\_23\_299 | G | I | G | L | T | A | K | P | K | - | - | S | G | Y | D | T | R | T | L | A | D | D | M | V | T | L | M | - | A | A | L | G | Y | Q | R | - | - | - | F | A | A | V | G | H | D | T | G | M | P | I |
| 095 UniRef90\_A0A2T4ZAU7\_6\_296 | G | Y | G | D | S | D | K | P | E | G | M | A | G | Y | D | K | A | N | M | A | R | D | I | Y | V | L | M | - | K | H | L | Q | L | A | K | - | - | - | V | A | L | V | G | H | D | R | G | A | R | V |
| 096 UniRef90\_UPI00143A9489\_23\_300 | G | M | G | L | S | S | K | P | E | - | - | G | G | Y | D | K | R | T | Q | G | V | D | M | A | R | M | L | - | D | A | L | G | V | A | R | - | - | - | V | D | L | V | T | H | D | I | G | N | M | V |
| 097 UniRef90\_A0A7C7NVI2\_6\_287 | G | F | G | Y | S | D | K | P | L | - | - | S | G | Y | D | T | R | S | A | A | S | D | I | Y | E | L | V | - | R | Q | L | G | L | Q | Q | - | - | - | V | D | I | V | A | H | D | I | G | V | R | V |
| 098 UniRef90\_UPI001661A3FF\_52\_332 | G | A | G | Q | S | D | A | P | P | - | - | S | G | Y | D | K | K | T | M | A | A | E | L | H | A | L | L | - | V | S | L | G | K | D | D | - | - | D | V | R | I | V | G | H | D | I | G | T | M | V |
| 099 UniRef90\_A0A6G4V4F1\_2\_276 | G | Q | G | E | S | D | K | P | E | - | - | G | G | Y | D | K | K | T | M | A | K | D | I | Y | E | L | V | - | R | G | L | G | H | E | Q | - | - | - | V | N | I | A | G | A | D | I | G | A | M | V |
| 100 UniRef90\_A0A260W032\_16\_294 | G | Y | G | A | T | D | K | P | A | - | - | T | G | Y | D | K | R | T | M | A | A | D | V | V | A | I | M | - | D | A | L | G | H | E | K | - | - | - | I | A | L | V | T | H | D | R | G | S | R | V |
| 101 UniRef90\_UPI00036BBA85\_7\_295 | G | Y | G | E | T | D | K | P | A | - | - | D | N | Y | D | K | R | N | M | A | K | D | I | K | E | L | I | - | E | H | L | G | Y | S | K | - | - | - | V | V | M | V | G | H | D | R | G | A | R | V |
| 102 UniRef90\_A0A4R5QBW7\_9\_278 | G | L | G | D | S | S | R | P | A | - | - | G | G | Y | D | K | K | T | V | S | N | D | L | W | R | L | M | A | E | V | L | G | E | R | R | - | - | - | F | F | L | V | G | H | D | W | G | G | P | T |
| 103 UniRef90\_UPI0012FB7881\_21\_292 | G | T | G | R | S | D | K | P | E | - | - | T | G | Y | D | S | T | T | A | G | A | D | L | A | R | L | M | - | T | V | L | G | H | D | S | - | - | - | F | G | V | I | G | H | D | V | G | M | V | Q |
| 104 UniRef90\_UPI00131B39C2\_13\_285 | G | M | G | L | S | S | R | P | E | - | - | Y | G | Y | D | K | K | T | Q | G | G | D | V | A | G | L | L | - | D | E | L | G | I | E | R | - | - | - | V | D | L | V | T | H | D | I | G | N | M | V |
| 105 UniRef90\_M2VKF7\_34\_311 | G | L | G | I | S | D | K | P | V | - | - | D | G | F | D | V | D | T | L | A | A | D | M | F | E | L | M | - | D | V | L | G | H | E | R | - | - | - | F | A | L | A | G | H | D | I | G | V | M | V |
| 106 UniRef90\_UPI0016124E74\_10\_283 | G | Y | G | R | S | D | K | P | P | - | - | H | G | Y | D | K | R | T | M | A | T | D | V | S | D | L | I | - | E | S | L | G | F | A | E | - | - | - | A | S | V | V | G | H | D | R | G | A | R | V |
| 107 UniRef90\_A0A263DFR4\_27\_299 | G | S | G | R | S | D | K | P | G | - | - | D | G | Y | D | T | G | S | L | A | A | D | L | A | A | V | M | - | E | E | L | G | H | E | E | - | - | - | Y | A | V | L | G | H | D | V | G | M | W | I |
| 108 UniRef90\_A0A0U5F3A6\_35\_313 | G | V | G | L | S | D | K | P | E | - | - | D | G | Y | D | A | D | T | L | S | A | D | M | F | A | L | M | - | D | A | L | G | H | E | R | - | - | - | F | A | M | V | G | H | D | I | G | M | W | T |
| 109 UniRef90\_A0A2N3KZL1\_38\_324 | G | L | G | Q | S | A | V | P | K | - | - | S | - | Y | V | G | Q | D | I | S | P | I | L | Y | G | L | A | - | K | Q | F | S | P | D | A | - | - | P | F | D | L | V | A | H | D | I | G | I | W | N |
| 110 UniRef90\_A0A1X7C0M0\_25\_283 | G | A | G | D | S | D | R | P | E | - | - | G | G | Y | A | K | T | D | Q | A | D | D | I | R | G | I | L | - | T | A | L | N | L | S | G | - | - | P | V | G | V | A | G | H | D | I | G | A | M | V |
| 111 UniRef90\_A0A078M0V2\_4\_284 | G | Y | G | D | S | D | K | P | E | G | I | E | N | Y | T | N | K | I | M | A | Q | D | I | A | E | I | L | - | A | A | Q | N | V | T | D | - | - | - | A | Y | V | V | G | H | D | R | G | A | R | V |
| 112 UniRef90\_A0A0F4Z6H8\_20\_271 | G | I | G | D | S | D | H | P | A | - | - | N | G | Y | D | M | A | T | V | A | D | D | L | A | E | L | M | - | T | T | L | G | H | E | T | - | - | - | F | H | A | C | G | E | D | W | G | G | A | A |
| 113 UniRef90\_UPI00161D2319\_22\_292 | G | M | D | A | S | D | K | P | A | - | - | T | G | Y | D | A | A | A | L | A | D | D | M | A | A | L | M | - | T | A | L | G | H | D | R | - | - | - | F | A | V | V | G | Y | D | L | G | M | L | V |
| 114 UniRef90\_A0A1Q8LX67\_38\_327 | G | L | G | D | S | E | G | V | P | - | - | T | G | Y | D | K | A | T | L | A | R | Y | V | H | G | L | V | A | G | H | L | G | L | R | E | - | - | - | V | H | V | V | G | H | D | L | G | A | G | V |
| 115 UniRef90\_A0A1Q9S2P6\_18\_309 | G | L | G | D | S | E | G | A | P | - | - | P | S | Y | D | K | A | T | L | A | R | Y | V | H | G | M | V | H | G | E | F | G | L | R | D | - | - | - | V | R | L | V | G | H | D | L | G | A | A | V |
| 116 UniRef90\_A0A5M7BQA6\_8\_281 | G | Y | G | R | T | D | K | P | R | - | - | G | G | Y | D | K | R | T | M | A | S | D | V | S | E | L | V | - | A | S | L | G | F | E | S | - | - | - | V | G | V | V | G | H | D | R | G | A | R | V |
| 117 UniRef90\_A0A3N6GJS2\_33\_314 | G | M | G | G | S | D | K | P | A | - | - | T | G | Y | D | K | K | T | M | A | G | V | V | H | G | F | V | - | R | T | L | G | Y | D | Q | - | - | - | V | D | I | A | G | H | D | V | G | A | Q | V |
| 118 UniRef90\_A0A0Q6MZD2\_36\_308 | G | L | G | L | S | S | K | P | A | - | - | G | G | F | D | K | K | T | Q | A | G | D | V | S | G | V | L | - | A | A | Y | G | V | Q | Q | - | - | - | A | D | V | V | A | H | D | I | G | N | M | V |
| 119 UniRef90\_UPI00052520C0\_22\_293 | G | F | G | D | S | D | K | P | E | - | - | T | G | Y | D | S | V | T | A | A | G | D | L | A | R | L | M | - | T | V | L | G | H | E | R | - | - | - | F | R | V | V | G | H | D | V | G | M | I | I |
| 120 UniRef90\_A0A1Q4XCF8\_2\_276 | G | M | G | G | S | D | K | P | A | - | - | T | G | Y | D | K | K | T | M | A | G | V | V | H | G | F | V | - | R | A | L | G | Y | D | Q | - | - | - | V | D | I | A | G | H | D | V | G | S | Q | V |
| 121 UniRef90\_A0A328LZX6\_33\_305 | G | L | G | L | S | A | R | P | A | - | - | T | G | Y | D | K | K | T | Q | G | E | D | V | A | G | L | L | - | D | A | L | K | V | E | H | - | - | - | V | D | V | V | A | H | D | I | G | N | M | V |
| 122 UniRef90\_A0A2V8S6L4\_40\_311 | G | A | G | E | S | A | K | P | D | - | - | T | G | Y | D | K | K | T | M | A | V | D | I | H | E | L | T | - | K | S | L | K | F | D | R | - | - | - | A | T | I | V | G | H | D | I | G | L | M | V |
| 123 UniRef90\_A0A1I5EB88\_8\_281 | G | Y | G | R | T | D | K | P | R | - | - | D | G | Y | D | K | R | T | M | A | A | D | M | A | E | L | V | - | A | A | L | G | F | D | S | - | - | - | V | S | V | V | G | H | D | R | G | A | R | V |
| 124 UniRef90\_UPI0014147ABD\_39\_305 | G | M | G | L | S | D | K | P | D | - | - | G | G | Y | D | T | G | S | L | A | D | D | L | A | A | V | M | - | D | A | L | G | H | Q | R | - | - | - | F | A | V | V | G | H | D | T | G | F | A | I |
| 125 UniRef90\_A0A1I7DL13\_6\_274 | G | C | G | D | S | F | K | P | M | - | - | T | G | Y | D | A | L | T | Q | A | D | D | I | L | A | L | I | - | Q | H | F | G | V | G | R | - | - | - | V | H | L | V | G | H | D | L | G | G | P | V |
| 126 UniRef90\_A0A3N7EDS3\_31\_305 | G | M | G | L | S | A | R | A | T | - | - | A | G | F | D | K | K | T | Q | G | Q | D | I | A | Q | L | L | - | D | K | L | G | V | A | K | - | - | - | T | Q | L | V | T | H | D | I | G | N | M | V |
| 127 UniRef90\_UPI0016225F9B\_4\_275 | G | Y | G | R | T | D | K | P | L | - | - | T | G | F | D | K | R | T | M | A | S | D | V | R | E | L | A | - | R | Q | L | G | H | E | R | - | - | - | L | T | L | V | G | H | D | R | G | A | R | V |
| 128 UniRef90\_UPI000997AE50\_107\_382 | G | L | G | D | S | T | G | A | P | - | - | S | N | Y | A | A | T | T | M | A | T | Y | V | H | D | L | L | - | D | R | I | G | K | R | H | - | - | G | V | E | L | V | A | H | D | I | G | A | G | V |
| 129 UniRef90\_A0A0N7F3H5\_6\_275 | G | Y | G | R | T | D | K | P | R | - | - | D | G | Y | D | K | R | T | M | A | S | D | V | S | E | L | V | - | R | G | L | G | F | E | Q | - | - | - | V | S | V | V | G | H | D | R | G | G | R | V |
| 130 UniRef90\_A0A3Q8F441\_11\_281 | G | Y | G | E | T | E | K | P | A | - | - | T | G | Y | D | K | R | S | M | A | Q | D | I | V | A | L | L | - | D | T | L | G | I | D | K | - | - | - | V | A | L | V | G | H | D | R | G | A | R | V |
| 131 UniRef90\_UPI001487D2FB\_23\_300 | G | M | G | L | S | S | H | P | E | - | - | G | G | Y | D | K | W | T | Q | A | G | D | V | R | A | V | L | - | S | R | L | G | I | D | R | - | - | - | A | A | V | V | G | H | D | I | G | T | M | V |
| 132 UniRef90\_A0A437GNC7\_38\_307 | G | L | G | I | S | D | K | P | E | - | - | E | G | Y | D | K | G | T | L | G | R | D | L | F | G | L | M | - | T | A | L | G | H | E | R | - | - | - | F | A | L | V | G | H | D | C | G | M | W | V |
| 133 UniRef90\_A0A1H3SWJ7\_6\_283 | G | Y | G | A | S | S | R | P | R | - | - | G | G | Y | D | K | R | T | M | S | A | D | M | S | G | L | M | - | H | E | L | G | Y | T | Q | - | - | - | A | V | V | V | G | H | D | R | G | A | R | V |
| 134 UniRef90\_UPI0013DD6281\_49\_329 | G | L | G | D | S | E | G | A | P | - | - | P | S | Y | D | K | A | T | L | A | R | L | V | H | G | L | L | A | G | R | L | G | L | R | D | - | - | - | I | D | L | V | G | H | D | L | G | A | G | V |
| 135 UniRef90\_I0L7C1\_27\_272 | G | L | G | D | S | A | R | A | A | - | - | D | G | F | D | A | A | T | G | A | D | D | M | L | G | L | L | - | D | A | L | G | V | E | Q | - | - | - | T | E | I | V | A | L | D | A | G | V | P | A |
| 136 UniRef90\_A0A260DLM1\_45\_326 | G | L | G | D | S | R | G | T | P | - | - | P | S | L | D | K | A | T | L | A | T | Y | V | H | D | L | V | A | D | Q | L | G | Q | R | D | - | - | - | I | D | I | V | G | H | D | L | G | A | A | V |
| 137 UniRef90\_UPI0005BDD2EC\_7\_280 | G | Y | G | L | T | D | K | P | Q | - | - | S | G | Y | G | K | R | R | M | A | A | D | I | S | E | L | I | - | H | G | L | G | F | D | R | - | - | A | A | A | V | I | G | H | D | R | G | A | R | V |
| 138 UniRef90\_A0A6G9YBC1\_27\_296 | G | Y | G | R | T | D | K | P | V | - | - | G | G | Y | D | K | R | T | M | A | A | D | I | A | G | L | L | - | E | H | L | G | V | R | T | - | - | - | A | A | V | V | G | H | D | R | G | A | R | V |
| 139 UniRef90\_UPI00145FA88A\_8\_280 | G | Y | G | R | S | D | K | P | L | - | - | G | G | Y | D | K | R | T | M | A | T | D | V | A | E | L | V | - | G | S | L | G | F | D | R | - | - | - | V | S | V | V | G | H | D | R | G | A | R | V |
| 140 UniRef90\_A0A2W0B1K8\_46\_299 | G | I | G | K | S | S | K | P | E | - | - | G | G | Y | D | K | K | T | Q | A | K | D | I | R | A | V | V | - | T | A | L | G | Y | D | K | - | - | - | T | F | V | V | A | H | D | I | G | N | M | V |
| 141 UniRef90\_A0A5B8G622\_28\_299 | G | L | G | L | S | A | R | P | A | - | - | G | G | Y | D | K | K | T | Q | G | E | D | I | A | G | V | L | - | D | A | L | G | V | G | E | - | - | - | V | D | L | V | A | H | D | I | G | N | M | V |
| 142 UniRef90\_A0A4S1XGQ8\_21\_302 | G | M | G | L | S | A | V | A | P | - | - | G | G | Y | D | K | K | T | Q | A | A | D | M | V | A | L | L | - | D | A | L | K | V | T | Q | - | - | - | A | D | V | V | A | H | D | I | G | N | M | V |
| 143 UniRef90\_A0A251YJ72\_8\_294 | G | F | G | D | S | D | V | A | G | - | - | P | D | H | D | S | A | T | T | A | A | D | L | H | A | L | I | - | D | A | L | G | R | G | P | - | - | - | V | H | L | L | A | Q | D | I | A | G | G | A |
| 144 UniRef90\_UPI000A0527D2\_65\_318 | G | A | G | A | S | T | R | P | P | - | - | G | G | Y | D | K | A | T | M | S | A | D | L | H | A | L | I | H | N | V | L | D | I | D | G | - | - | P | V | S | V | V | G | H | D | L | G | S | M | L |
| 145 UniRef90\_A0A4V2S7H9\_8\_280 | G | Y | G | H | T | D | K | P | R | - | - | H | G | Y | D | K | R | T | M | A | T | D | V | A | E | L | L | - | R | S | L | G | F | D | T | - | - | - | A | K | V | V | G | H | D | R | G | G | R | V |
| 146 UniRef90\_UPI00131B216A\_18\_247 | G | M | G | S | S | G | K | P | E | - | - | S | G | Y | D | K | K | T | M | A | A | D | V | L | A | L | A | - | R | H | L | G | H | E | Q | - | - | - | V | D | I | A | G | H | D | I | G | A | L | V |
| 147 UniRef90\_A0A1G8T9Y6\_5\_243 | G | A | G | R | S | D | A | P | A | - | - | G | G | Y | D | K | K | T | M | A | A | D | I | H | G | L | L | - | D | R | L | G | L | D | R | - | - | D | I | R | L | V | G | H | D | I | G | T | M | V |
| 148 UniRef90\_UPI00161F613F\_5\_254 | G | F | G | D | S | - | - | A | A | - | - | G | D | N | D | S | A | T | A | A | E | D | L | H | Q | L | I | - | A | H | F | G | V | G | P | - | - | - | V | H | L | V | G | Q | D | I | A | G | G | A |
| 149 UniRef90\_UPI00142071FA\_80\_362 | G | I | G | L | T | E | K | A | P | - | - | Y | G | Y | D | T | A | T | L | A | D | D | L | A | A | L | M | - | T | A | L | G | H | Q | R | - | - | - | F | A | V | V | G | H | D | T | G | Y | I | I |
| 150 UniRef90\_A0A6G7Q8F6\_7\_219 | G | Y | G | L | S | D | R | P | K | G | I | A | A | Y | H | L | D | R | L | A | Q | D | V | I | A | L | A | - | D | A | C | G | L | A | R | - | - | - | F | N | L | V | G | H | D | W | G | G | L | V |

  
  

|  |  |  |  |  |  |  |  |  |  |  |  |  |  |  |  |  |  |  |  |  |  |  |  |  |  |  |  |  |  |  |  |  |  |  |  |  |  |  |  |  |  |  |  |  |  |  |  |  |  |  |
| --- | --- | --- | --- | --- | --- | --- | --- | --- | --- | --- | --- | --- | --- | --- | --- | --- | --- | --- | --- | --- | --- | --- | --- | --- | --- | --- | --- | --- | --- | --- | --- | --- | --- | --- | --- | --- | --- | --- | --- | --- | --- | --- | --- | --- | --- | --- | --- | --- | --- | --- |
| **001 Input\_protein\_seq** | A | Y | P | F | A | A | M | Y | P | E | S | V | K | R | L | A | L | L | D | - | A | G | I | P | G | V | T | - | - | - | - | L | P | A | A | L | - | - | - | - | - | - | - | P | I | E | - | - | - | - |
| 002 UniRef90\_UPI001430CB85\_4\_307 | A | Y | P | Y | A | V | L | F | G | N | E | V | Q | R | L | A | L | L | D | - | A | G | I | P | G | V | T | - | - | - | - | L | P | D | A | L | - | - | - | - | - | - | - | P | V | A | - | - | - | - |
| 003 UniRef90\_UPI00055F8D66\_10\_306 | A | Y | T | Y | A | A | L | F | G | D | E | V | R | S | L | A | L | M | D | - | A | G | I | P | G | I | T | - | - | - | - | L | P | D | A | L | - | - | - | - | - | - | - | P | T | A | - | - | - | - |
| 004 UniRef90\_UPI00161D8BA7\_15\_312 | A | Y | P | Y | A | M | L | F | S | D | E | V | E | R | L | A | L | F | D | - | A | G | I | P | G | V | T | - | - | - | - | L | P | D | A | L | - | - | - | - | - | - | - | P | I | A | - | - | - | - |
| 005 UniRef90\_A0A4R7GLU7\_7\_305 | A | Y | P | Y | A | A | M | Y | G | D | E | V | R | R | L | A | L | L | D | - | A | G | I | P | G | I | T | - | - | - | - | L | P | D | A | L | - | - | - | - | - | - | - | P | V | T | - | - | - | - |
| 006 UniRef90\_UPI00041CD164\_24\_309 | A | Y | P | L | A | A | M | F | G | T | E | V | R | R | L | A | L | L | D | - | A | A | I | P | G | I | T | - | - | - | - | L | P | E | A | L | - | - | - | - | - | - | - | P | I | A | - | - | - | - |
| 007 UniRef90\_I0QSF8\_15\_298 | A | Y | P | Y | A | V | L | F | G | D | E | I | K | K | L | A | I | L | D | - | A | G | I | P | G | I | T | - | - | - | - | L | P | D | A | L | - | - | - | - | - | - | - | P | H | S | - | - | - | - |
| 008 UniRef90\_A0A2N5ENH7\_9\_301 | A | W | P | Y | A | L | R | Y | S | P | Q | V | I | K | L | A | L | L | D | - | A | G | I | P | G | I | T | - | - | - | - | L | P | D | A | L | - | - | - | - | - | - | - | P | A | T | - | - | - | - |
| 009 UniRef90\_A0A0Q8E2M6\_29\_312 | A | Y | P | L | A | A | R | Y | S | D | E | V | Q | A | L | A | L | L | D | - | A | G | I | P | G | V | T | - | - | - | - | L | P | D | R | L | - | - | - | - | - | - | - | P | M | A | - | - | - | - |
| 010 UniRef90\_A0A0F5XY46\_6\_287 | A | W | S | L | A | A | Q | F | P | Q | H | V | S | R | L | A | L | L | D | - | G | G | I | P | G | I | T | - | - | - | - | L | P | E | S | L | - | - | - | - | - | - | - | P | V | A | - | - | - | - |
| 011 UniRef90\_A0A3N4NUX5\_4\_284 | A | F | A | L | A | A | Q | F | P | E | R | V | K | K | I | A | L | L | D | - | A | G | I | P | G | V | T | - | - | - | - | L | P | D | A | L | - | - | - | - | - | - | - | P | V | T | - | - | - | - |
| 012 UniRef90\_UPI000DDED2D9\_11\_293 | A | Y | P | Y | A | A | S | Y | G | N | S | V | R | G | L | A | I | L | D | - | T | G | I | P | G | I | S | - | - | - | - | L | P | D | M | L | - | - | - | - | - | - | - | P | W | A | - | - | - | - |
| 013 UniRef90\_UPI000DDED53B\_10\_292 | A | F | P | Y | S | L | L | Y | G | D | E | V | G | A | L | V | L | M | D | - | A | G | I | P | G | I | T | - | - | - | - | L | P | D | M | L | - | - | - | - | - | - | - | P | S | T | - | - | - | - |
| 014 UniRef90\_A0A1G4JKM0\_6\_288 | A | F | P | Y | A | L | M | F | S | D | E | I | E | G | L | A | L | L | D | - | A | G | I | P | G | V | T | - | - | - | - | L | P | S | L | I | - | - | - | - | - | - | - | P | T | S | - | - | - | - |
| 015 UniRef90\_UPI0016728815\_21\_305 | A | F | S | L | A | L | K | Y | P | G | E | L | R | G | L | A | L | L | D | - | A | G | I | P | G | I | T | - | - | - | - | L | P | D | A | I | - | - | - | - | - | - | - | P | T | D | - | - | - | - |
| 016 UniRef90\_A0A3N2H8D2\_14\_297 | A | F | S | L | A | L | N | F | E | N | H | L | R | R | V | A | L | L | D | - | A | G | I | P | G | V | T | - | - | - | - | L | P | E | T | I | - | - | - | - | - | - | - | F | T | D | - | - | - | - |
| 017 UniRef90\_UPI0005644DD1\_9\_307 | A | F | S | L | A | L | N | H | E | D | Q | L | R | G | V | A | L | L | D | - | A | G | I | P | G | I | T | - | - | - | - | L | P | E | S | I | - | - | - | - | - | - | - | P | T | D | - | - | - | - |
| 018 UniRef90\_A0A6B2VDS0\_18\_308 | A | F | S | L | A | L | N | F | E | S | H | L | H | R | V | A | L | L | D | - | A | G | I | P | G | I | T | - | - | - | - | L | P | E | A | I | - | - | - | - | - | - | - | P | T | D | - | - | - | - |
| 019 UniRef90\_A0A6G3RZZ1\_25\_307 | A | F | S | L | A | L | K | H | E | E | R | L | H | G | V | A | L | L | D | - | A | G | I | P | G | I | T | - | - | - | - | L | P | D | A | V | - | - | - | - | - | - | - | P | T | D | - | - | - | - |
| 020 UniRef90\_A0A209C7X3\_21\_305 | A | F | S | L | A | L | Q | H | P | G | R | L | H | G | L | A | L | L | D | - | A | G | I | P | G | I | T | - | - | - | - | L | P | D | A | I | - | - | - | - | - | - | - | P | T | D | - | - | - | - |
| 021 UniRef90\_UPI001269E708\_26\_309 | A | F | S | L | A | L | T | Y | E | D | R | L | G | G | V | V | L | L | D | - | A | G | I | P | G | V | S | - | - | - | - | L | P | A | A | V | - | - | - | - | - | - | - | P | T | D | - | - | - | - |
| 022 UniRef90\_A0A2M9J7W9\_25\_306 | A | F | S | L | A | L | T | Y | E | E | R | L | H | G | V | A | L | L | D | - | A | G | I | P | G | I | T | - | - | - | - | L | P | D | S | I | - | - | - | - | - | - | - | P | T | D | - | - | - | - |
| 023 UniRef90\_A0A6G9F3J8\_23\_307 | A | F | S | L | A | L | E | Y | E | S | Q | L | H | G | V | A | L | V | D | - | A | G | I | P | G | I | T | - | - | - | - | L | P | D | A | V | - | - | - | - | - | - | - | P | L | D | - | - | - | - |
| 024 UniRef90\_A0A1H2UZ41\_14\_292 | A | Y | P | F | V | V | R | F | A | E | E | V | G | R | L | V | M | L | D | - | A | N | I | P | G | V | T | - | - | - | - | L | K | S | T | I | - | - | - | - | - | - | - | E | V | G | - | - | - | - |
| 025 UniRef90\_UPI0012B05B7D\_16\_286 | G | Y | A | F | G | H | L | Y | A | S | S | L | R | G | I | V | L | L | D | - | A | N | I | P | G | V | T | - | - | - | - | L | Q | D | T | I | - | - | - | - | - | - | - | T | L | G | - | - | - | - |
| 026 UniRef90\_A0A5C8T429\_18\_294 | G | Y | P | F | V | A | T | Y | P | D | S | V | R | K | L | A | L | I | D | - | A | T | V | P | G | L | A | - | - | - | - | P | A | E | A | Y | - | - | - | - | - | - | - | A | F | A | - | - | - | - |
| 027 UniRef90\_UPI0012E86E3E\_10\_309 | G | Y | A | Y | A | A | E | W | P | E | D | V | K | R | L | A | V | F | D | - | A | G | I | P | G | I | T | - | - | - | - | P | P | A | P | A | - | - | - | - | - | - | - | G | I | P | - | - | - | - |
| 028 UniRef90\_A0A1A9HTL2\_13\_300 | A | Y | A | Y | A | A | D | W | P | R | E | V | R | T | L | T | V | M | E | - | A | A | L | P | G | I | T | - | - | - | - | P | P | A | P | A | - | - | - | - | - | - | - | G | I | P | - | - | - | - |
| 029 UniRef90\_A0A0R1V0C7\_7\_281 | G | F | T | Y | A | H | L | F | P | D | D | L | L | G | I | T | L | I | D | - | A | N | I | P | G | V | T | - | - | - | - | L | Q | K | Q | I | - | - | - | - | - | - | - | P | L | D | - | - | - | - |
| 030 UniRef90\_A0A3N1M9Y1\_5\_282 | A | F | A | L | A | A | A | H | P | E | A | I | R | T | L | S | I | V | D | - | V | T | L | P | G | I | G | - | - | - | - | P | D | I | - | - | - | - | - | - | - | - | - | - | - | - | - | - | - | - |
| 031 UniRef90\_A0A158JUX7\_17\_294 | A | Y | A | H | A | A | Q | W | P | Q | E | V | R | K | F | A | F | I | E | - | S | G | L | P | A | F | G | - | - | - | - | Q | E | A | S | M | - | - | - | - | - | - | - | D | I | A | - | - | - | - |
| 032 UniRef90\_A0A0U3LIL8\_12\_296 | A | Y | A | Y | A | A | D | W | P | E | D | V | R | T | L | S | L | L | D | - | A | A | L | P | G | I | S | - | - | - | - | P | L | P | T | T | - | - | - | - | - | - | - | E | I | P | - | - | - | - |
| 033 UniRef90\_A0A248JS33\_7\_301 | G | Q | A | Y | A | A | D | W | P | G | D | V | R | R | L | A | L | V | D | - | A | A | L | P | G | I | T | - | - | - | - | P | P | P | P | P | - | - | - | - | - | - | - | G | I | P | - | - | - | - |
| 034 UniRef90\_A0A1Y0REZ4\_12\_287 | A | Y | A | Y | A | A | A | Y | P | H | H | V | R | R | L | V | F | A | E | - | S | L | L | P | G | F | G | - | - | - | - | L | E | E | M | M | - | - | - | - | - | - | - | D | V | W | - | - | - | - |
| 035 UniRef90\_A0A0M2WRU5\_3\_280 | G | Y | A | L | A | S | D | F | P | Q | A | V | T | K | L | A | L | T | E | - | A | V | I | P | G | L | A | - | - | - | - | P | A | P | P | I | - | - | - | - | - | - | - | F | V | A | - | - | - | - |
| 036 UniRef90\_A0A0N8GFM1\_6\_279 | A | F | S | L | A | A | H | H | R | E | A | V | K | S | L | A | I | L | D | - | V | V | I | P | G | D | G | - | - | - | - | A | D | F | - | - | - | - | - | - | - | - | - | - | - | - | - | - | - | - |
| 037 UniRef90\_A0A252EMP1\_7\_277 | A | Y | A | Y | A | A | R | H | P | T | E | V | K | S | L | V | F | L | E | - | S | K | I | L | G | I | E | - | - | - | - | S | D | D | - | - | - | - | - | - | - | - | - | - | - | - | - | - | - | - |
| 038 UniRef90\_W0A6K5\_40\_320 | G | Y | A | L | A | S | D | Y | P | A | D | I | K | K | I | A | L | T | E | - | A | V | I | P | G | L | A | - | - | - | - | P | A | P | S | I | - | - | - | - | - | - | - | F | V | D | - | - | - | - |
| 039 UniRef90\_A0A3S1SM66\_23\_304 | A | Y | A | Y | A | A | D | W | P | S | D | V | R | R | L | A | V | M | D | - | A | L | L | P | G | L | S | - | - | - | - | A | P | R | - | I | - | - | - | - | - | - | - | D | L | S | - | - | - | - |
| 040 UniRef90\_A0A2E5L5C6\_7\_286 | A | Y | A | L | A | A | A | H | P | E | A | V | E | K | M | V | I | L | D | - | V | T | I | P | G | C | G | - | - | - | - | G | D | F | - | - | - | - | - | - | - | - | - | - | - | - | - | - | - | - |
| 041 UniRef90\_M5D1B4\_14\_289 | A | Y | A | L | A | S | D | Q | P | Q | A | V | Q | Q | L | A | V | T | E | - | A | V | I | P | G | L | A | - | - | - | - | P | E | P | G | I | - | - | - | - | - | - | - | F | A | A | - | - | - | - |
| 042 UniRef90\_UPI0016845BA7\_31\_303 | A | Y | A | Y | A | N | A | Y | S | E | D | V | N | H | L | A | I | L | E | - | V | P | I | P | G | L | E | - | - | - | - | G | W | E | N | V | - | - | - | - | - | - | - | S | S | E | - | - | - | - |
| 043 UniRef90\_UPI0014648199\_14\_291 | G | Y | A | L | A | S | D | Q | P | Q | A | V | R | T | L | T | L | T | E | - | A | V | I | P | G | L | A | - | - | - | - | P | A | P | P | I | - | - | - | - | - | - | - | F | V | A | - | - | - | - |
| 044 UniRef90\_K9RUM7\_12\_289 | A | F | T | V | L | K | T | F | P | E | S | I | L | G | A | G | L | I | D | - | A | A | I | P | G | L | V | - | - | - | - | S | P | D | F | F | - | - | - | - | - | - | - | G | V | V | - | - | - | - |
| 045 UniRef90\_A0A1Q2U086\_13\_290 | A | F | T | I | L | K | L | F | P | Q | S | V | L | G | A | G | L | I | D | - | A | A | I | P | G | L | V | - | - | - | - | S | D | D | F | F | - | - | - | - | - | - | - | S | V | V | - | - | - | - |
| 046 UniRef90\_B9JMN1\_3\_282 | A | Y | S | Y | A | A | Q | F | Q | Q | Q | V | R | K | L | A | I | F | E | - | M | V | L | P | G | F | G | - | - | - | - | I | M | E | E | A | - | - | - | - | - | - | - | M | T | P | - | - | - | - |
| 047 UniRef90\_A0A2X1TAZ7\_33\_319 | G | Y | A | L | A | A | D | Y | P | A | D | I | K | R | I | A | L | T | E | - | A | V | I | P | G | L | A | - | - | - | - | P | A | P | A | I | - | - | - | - | - | - | - | F | V | D | - | - | - | - |
| 048 UniRef90\_UPI00131A8152\_6\_279 | A | W | A | V | A | A | H | H | P | G | H | A | K | T | L | T | L | V | D | - | I | A | I | P | G | D | G | - | - | - | - | N | P | N | I | - | - | - | - | - | - | - | - | - | - | - | - | - | - | - |
| 049 UniRef90\_F7YB04\_47\_316 | A | Y | S | Y | A | A | Q | H | P | G | E | V | T | T | L | A | I | V | D | - | V | P | L | P | G | I | E | - | - | - | - | P | W | N | E | L | - | - | - | - | - | - | - | I | Q | G | - | - | - | - |
| 050 UniRef90\_A0A1A3M1Z8\_5\_294 | A | Y | A | Y | A | S | L | Y | P | A | D | I | E | R | L | V | L | M | E | - | A | P | I | P | G | T | K | - | - | - | - | S | M | E | S | V | - | - | - | - | - | - | - | A | T | T | - | - | - | - |
| 051 UniRef90\_UPI0004C32CE1\_32\_312 | A | F | S | F | A | A | N | Y | P | A | A | T | R | K | V | A | F | M | D | - | V | L | H | P | D | E | G | - | - | - | - | Y | Y | Q | L | P | - | - | - | - | - | - | - | L | L | P | - | - | - | - |
| 052 UniRef90\_A0A0J6NQJ9\_12\_287 | A | Y | A | L | A | S | D | H | P | G | V | V | S | R | L | A | L | T | E | - | A | V | I | P | G | L | A | - | - | - | - | P | A | P | S | I | - | - | - | - | - | - | - | F | A | P | - | - | - | - |
| 053 UniRef90\_A0A534ESC2\_32\_321 | A | V | A | H | A | Q | V | Y | P | R | E | V | K | R | L | V | V | T | E | - | S | N | V | P | G | V | S | - | - | - | - | P | P | A | P | A | - | - | - | - | - | - | - | G | I | P | - | - | - | - |
| 054 UniRef90\_A0A1H1Z302\_21\_301 | A | Y | A | L | A | A | D | H | P | E | R | V | G | R | L | A | V | V | D | - | A | V | I | P | G | V | T | - | - | - | - | P | V | P | S | F | - | - | - | - | - | - | - | F | S | P | - | - | - | - |
| 055 UniRef90\_A0A158DLE6\_24\_295 | G | Y | A | F | A | A | L | Y | P | Q | L | T | D | R | F | V | F | I | E | - | S | G | L | P | G | F | G | - | - | - | - | Q | E | N | A | M | - | - | - | - | - | - | - | N | V | A | - | - | - | - |
| 056 UniRef90\_A0A163VVR8\_16\_280 | G | Y | A | M | A | S | D | F | P | Q | A | V | E | R | L | V | L | T | E | - | A | V | I | P | G | L | A | - | - | - | - | P | P | P | P | I | - | - | - | - | - | - | - | F | V | A | - | - | - | - |
| 057 UniRef90\_UPI000369F0DF\_7\_289 | G | Y | A | L | A | T | D | H | P | E | A | V | E | R | M | A | L | T | E | - | A | V | I | P | G | L | A | - | - | - | - | P | S | P | P | I | - | - | - | - | - | - | - | F | V | P | - | - | - | - |
| 058 UniRef90\_UPI00098F4FB4\_43\_309 | V | Y | A | Y | A | A | Q | H | P | N | D | V | E | T | L | S | I | M | D | - | V | P | L | P | G | I | A | - | - | - | - | P | W | D | E | I | - | - | - | - | - | - | - | V | Q | G | - | - | - | - |
| 059 UniRef90\_UPI0015A1AD76\_10\_292 | A | Y | A | F | A | A | A | H | P | E | A | V | R | Q | L | V | F | M | E | - | F | A | L | A | G | F | G | - | - | - | - | V | W | E | Q | G | - | - | - | - | - | - | - | I | T | P | - | - | - | - |
| 060 UniRef90\_UPI001689F051\_63\_318 | A | Y | P | L | A | G | Q | Y | P | D | A | V | R | T | L | T | L | I | E | - | A | G | V | I | G | L | G | - | - | - | - | R | S | V | A | A | - | - | - | - | - | - | - | - | - | - | - | - | - | - |
| 061 UniRef90\_UPI000DD77D66\_43\_313 | A | Y | A | Y | A | A | Q | H | P | A | N | V | R | T | L | A | I | L | D | - | V | P | L | P | G | I | E | - | - | - | - | P | W | D | E | W | - | - | - | - | - | - | - | V | Q | G | - | - | - | - |
| 062 UniRef90\_A0A3M0I8T4\_31\_307 | A | Y | A | L | A | A | D | H | P | D | R | V | V | R | L | A | V | A | E | - | A | A | I | P | G | V | S | - | - | - | - | P | S | P | P | L | - | - | - | - | - | - | - | F | G | S | - | - | - | - |
| 063 UniRef90\_UPI000377E2D1\_4\_271 | A | F | S | I | A | A | R | H | P | E | R | V | S | R | L | G | V | V | D | - | V | A | I | P | G | D | G | - | - | - | - | Q | P | N | I | - | - | - | - | - | - | - | - | - | - | - | - | - | - | - |
| 064 UniRef90\_UPI0016198B53\_15\_286 | G | Y | A | L | A | A | D | H | P | A | A | V | D | R | L | V | L | I | D | - | A | T | I | P | G | L | A | - | - | - | - | P | D | I | P | I | - | - | - | - | - | - | - | F | G | P | - | - | - | - |
| 065 UniRef90\_UPI0013C0217D\_48\_325 | A | F | S | F | A | V | N | H | P | A | A | T | R | R | A | A | M | L | D | - | A | L | H | P | D | D | R | - | - | - | - | F | Y | R | L | P | - | - | - | - | - | - | - | L | M | P | - | - | - | - |
| 066 UniRef90\_C7PGN2\_5\_260 | A | Y | S | Y | A | A | Q | F | G | E | E | V | K | R | L | V | L | M | D | - | V | P | P | V | G | E | Y | - | - | - | - | L | K | K | T | P | - | - | - | - | - | - | - | L | L | P | - | - | - | - |
| 067 UniRef90\_A0A1F7Q7Q2\_7\_275 | A | H | R | L | A | L | D | H | P | A | L | L | T | R | L | V | L | L | D | - | - | I | A | P | T | Y | D | - | - | - | - | V | F | E | R | - | - | - | - | - | - | - | - | - | T | D | - | - | - | - |
| 068 UniRef90\_A0A2V7F1K9\_9\_274 | A | H | R | F | A | L | D | H | P | T | L | L | T | H | L | V | L | L | D | - | - | I | A | P | T | Y | D | - | - | - | - | V | L | E | S | - | - | - | - | - | - | - | - | - | L | D | - | - | - | - |
| 069 UniRef90\_UPI001616C592\_31\_308 | A | Y | A | M | V | A | D | N | P | G | P | V | T | R | L | A | I | A | E | - | M | L | I | P | G | I | S | - | - | - | - | P | S | P | P | L | - | - | - | - | - | - | - | I | P | E | - | - | - | - |
| 070 UniRef90\_UPI001456849A\_71\_360 | A | F | A | I | A | R | N | H | P | D | M | V | K | R | L | A | I | L | D | - | V | P | L | M | G | L | P | - | - | - | - | Y | A | D | T | L | - | - | - | - | - | - | - | - | - | - | - | - | - | - |
| 071 UniRef90\_A0A2S8J6S6\_18\_282 | A | Y | A | W | A | A | T | H | R | D | T | V | T | R | L | A | I | F | D | - | V | P | I | P | G | A | E | - | - | - | - | L | W | D | T | I | - | - | - | - | - | - | - | Y | A | D | - | - | - | - |
| 072 UniRef90\_A0A2I2GC12\_18\_280 | A | Y | P | W | A | A | Q | F | P | S | R | I | K | S | L | S | I | L | D | - | A | A | V | P | G | L | T | - | - | - | - | P | P | Q | Q | F | - | - | - | - | - | - | - | P | L | S | - | - | - | - |
| 073 UniRef90\_A0A549T469\_41\_312 | A | F | M | L | A | L | L | H | P | E | K | V | A | R | L | V | L | V | D | - | C | L | V | P | G | T | E | - | - | - | - | N | M | D | A | R | - | - | - | - | - | - | - | R | - | - | - | - | - | - |
| 074 UniRef90\_A0A5C4LJC6\_5\_278 | A | L | P | Q | A | A | L | Y | R | E | A | V | T | H | L | A | V | L | D | - | V | P | L | P | G | W | T | - | - | - | - | E | W | E | A | T | - | - | - | - | - | - | - | T | A | - | - | - | - | - |
| 075 UniRef90\_UPI001619D806\_9\_283 | A | H | R | W | A | L | D | R | P | D | Q | V | E | R | L | A | V | L | D | - | - | I | I | P | T | R | E | - | - | - | - | M | W | K | R | - | - | - | - | - | - | - | - | - | L | D | - | - | - | - |
| 076 UniRef90\_A0A239P1A5\_69\_347 | G | Y | A | L | A | S | D | H | P | Q | S | V | E | R | Y | V | A | M | E | - | T | I | T | P | G | L | T | - | - | - | - | E | P | G | A | L | - | - | - | - | - | - | - | F | L | P | - | - | - | - |
| 077 UniRef90\_UPI0005262A70\_5\_280 | T | F | R | L | A | A | R | H | P | E | L | L | R | S | F | T | A | I | E | - | T | T | L | P | G | Y | G | - | - | - | - | L | E | T | L | A | - | - | - | - | - | - | - | D | V | V | - | - | - | - |
| 078 UniRef90\_A0A241XUQ6\_27\_314 | T | Y | P | M | V | V | K | N | Q | A | D | I | A | R | L | V | Y | M | E | - | A | P | I | P | D | A | R | - | - | - | - | I | Y | R | F | P | - | - | - | - | - | - | - | A | F | T | - | - | - | - |
| 079 UniRef90\_A0A160FQ65\_32\_312 | G | F | A | M | A | A | D | Q | P | G | R | I | E | R | I | A | L | G | E | - | A | I | I | P | G | V | S | - | - | - | - | D | S | P | P | L | - | - | - | - | - | - | - | L | S | D | - | - | - | - |
| 080 UniRef90\_UPI0006AE4C23\_4\_281 | A | Y | F | F | A | A | T | H | P | E | S | T | W | R | L | A | M | W | D | - | P | G | A | P | D | P | V | - | - | - | - | L | A | S | L | S | - | - | - | - | - | - | - | A | L | P | - | - | - | - |
| 081 UniRef90\_T0H3T7\_11\_281 | A | T | R | F | A | K | D | Y | P | E | R | V | D | R | L | V | V | M | D | - | - | N | V | P | T | R | I | - | - | - | - | V | A | R | D | - | - | - | - | - | - | - | - | - | I | N | - | - | - | - |
| 082 UniRef90\_A0A4R2IH15\_5\_277 | G | Y | A | L | A | A | D | H | P | E | R | I | E | R | L | A | V | A | E | - | A | A | V | P | G | L | T | - | - | - | - | P | S | P | G | L | - | - | - | - | - | - | - | F | E | S | - | - | - | - |
| 083 UniRef90\_UPI00140B863E\_4\_276 | A | Y | A | L | A | A | A | Y | R | D | N | V | S | H | L | V | V | E | E | - | E | L | L | P | G | L | D | - | - | - | - | V | S | P | A | L | - | - | - | - | - | - | - | V | N | E | - | - | - | - |
| 084 UniRef90\_A0A3R9UXB7\_22\_297 | A | H | A | H | A | A | N | H | P | E | A | T | R | S | V | T | L | L | D | - | V | S | H | P | D | D | G | - | - | - | - | W | A | E | L | T | - | - | - | - | - | - | - | L | L | P | - | - | - | - |
| 085 UniRef90\_UPI000F8D1465\_20\_303 | G | Y | A | L | A | A | D | F | P | A | R | V | E | R | L | A | V | M | D | - | A | N | I | P | G | V | L | - | - | - | - | P | S | P | P | L | - | - | - | - | - | - | - | F | S | E | - | - | - | - |
| 086 UniRef90\_A0A6J4HYT7\_23\_307 | A | F | S | F | A | A | N | F | P | E | A | T | A | R | L | V | M | L | D | - | T | P | H | P | D | E | S | - | - | - | - | M | Y | R | L | P | - | - | - | - | - | - | - | M | L | P | L | A | E | V |
| 087 UniRef90\_A0A1D8SMR3\_19\_296 | G | Y | V | L | A | A | R | H | R | D | R | V | T | R | L | V | L | A | E | - | A | V | L | A | G | F | T | - | - | - | - | P | L | P | P | L | - | - | - | - | - | - | - | L | M | P | - | - | - | - |
| 088 UniRef90\_A0A4V2HUZ4\_48\_320 | A | Y | V | L | A | H | L | Y | P | Q | A | V | S | R | L | V | L | V | D | - | C | L | L | P | G | T | E | - | - | - | - | N | L | D | A | L | - | - | - | - | - | - | - | R | - | - | - | - | - | - |
| 089 UniRef90\_UPI0010582A1B\_5\_279 | A | L | S | Q | A | A | M | H | R | Q | A | V | T | R | L | A | V | L | D | - | V | P | L | P | G | W | T | - | - | - | - | G | W | E | A | T | - | - | - | - | - | - | - | I | A | - | - | - | - | - |
| 090 UniRef90\_UPI00166CE541\_54\_338 | A | Y | A | Y | A | A | A | H | P | G | D | V | T | K | L | V | L | S | E | - | A | P | I | P | D | K | A | - | - | - | - | L | Y | T | F | P | - | - | - | - | - | - | - | S | L | T | - | - | - | - |
| 091 UniRef90\_A0A5C1HX54\_34\_307 | A | Y | A | Y | A | A | Q | Y | P | E | D | V | K | K | I | A | L | L | D | - | A | L | L | P | G | V | E | - | - | - | - | - | - | - | - | - | - | - | - | - | - | - | - | P | V | W | - | - | - | - |
| 092 UniRef90\_A0A4D4KET2\_34\_306 | G | Y | A | L | A | A | D | H | P | D | R | L | D | R | L | A | V | A | E | - | A | A | I | P | G | L | S | - | - | - | - | P | S | P | P | L | - | - | - | - | - | - | - | F | G | S | - | - | - | - |
| 093 UniRef90\_A0A1I1XP17\_9\_276 | A | L | A | Y | A | F | R | F | R | D | Q | V | S | H | L | V | L | I | D | - | A | P | L | Q | G | T | Q | - | - | - | - | A | M | E | A | M | - | - | - | - | - | - | - | R | G | D | - | - | - | - |
| 094 UniRef90\_A0A1D7VX60\_23\_299 | A | Y | A | L | A | A | D | H | P | E | R | V | A | R | L | A | V | A | E | - | A | F | L | P | A | V | T | - | - | - | - | P | S | P | P | L | - | - | - | - | - | - | - | V | G | T | - | - | - | - |
| 095 UniRef90\_A0A2T4ZAU7\_6\_296 | A | R | R | F | A | L | D | Y | P | D | H | V | V | G | L | G | L | I | D | - | - | I | L | P | L | E | Y | - | - | - | - | V | Y | D | H | L | - | - | - | - | - | - | - | S | T | S | - | - | - | - |
| 096 UniRef90\_UPI00143A9489\_23\_300 | G | Y | A | F | A | A | Q | Y | P | E | R | V | T | R | F | V | L | M | D | - | A | P | I | P | G | V | G | - | - | - | - | P | W | D | E | I | - | - | - | - | - | - | - | L | K | S | - | - | - | - |
| 097 UniRef90\_A0A7C7NVI2\_6\_287 | A | Y | R | F | A | L | D | H | E | E | T | V | R | R | L | V | L | L | D | - | S | T | P | P | M | E | Q | - | - | - | - | L | G | P | Q | S | - | - | - | - | - | - | - | - | - | - | - | - | - | - |
| 098 UniRef90\_UPI001661A3FF\_52\_332 | A | Y | S | Y | A | A | Q | Y | P | A | T | V | E | K | L | V | L | S | E | - | A | P | I | P | D | L | S | - | - | - | - | I | Y | Q | I | P | - | - | - | - | - | - | - | S | L | T | - | - | - | - |
| 099 UniRef90\_A0A6G4V4F1\_2\_276 | A | Y | S | F | A | A | N | Y | P | Q | A | T | K | K | L | A | L | W | E | - | G | A | P | F | S | Q | A | - | - | - | - | F | Q | H | F | A | - | - | - | - | - | - | K | P | F | P | - | - | - | - |
| 100 UniRef90\_A0A260W032\_16\_294 | G | T | R | F | A | K | D | H | P | D | K | L | D | R | L | V | V | M | D | - | - | N | I | P | T | R | I | - | - | - | - | V | A | E | T | Y | - | - | - | - | - | - | - | D | V | A | - | - | - | - |
| 101 UniRef90\_UPI00036BBA85\_7\_295 | T | T | R | F | A | K | D | Y | P | E | M | V | D | R | I | C | T | M | D | - | - | N | I | P | T | R | I | - | - | - | - | V | F | E | T | M | S | G | R | P | I | G | T | D | I | S | - | - | - | - |
| 102 UniRef90\_A0A4R5QBW7\_9\_278 | A | F | S | L | A | A | Q | H | R | D | A | V | R | R | M | A | I | F | D | - | A | P | V | P | G | D | G | - | - | - | - | S | P | V | F | H | - | - | - | - | - | - | - | - | - | - | - | - | - | - |
| 103 UniRef90\_UPI0012FB7881\_21\_292 | A | Y | A | L | A | A | D | H | P | G | R | V | T | R | L | V | L | A | E | - | A | A | L | P | G | V | S | - | - | - | - | P | M | P | A | V | - | - | - | - | - | - | - | L | P | P | - | - | - | - |
| 104 UniRef90\_UPI00131B39C2\_13\_285 | G | Y | A | F | A | A | Q | Y | P | H | R | V | T | R | F | V | V | M | D | - | A | P | L | P | G | I | G | - | - | - | - | P | W | E | E | I | - | - | - | - | - | - | - | L | K | H | - | - | - | - |
| 105 UniRef90\_M2VKF7\_34\_311 | G | Y | A | M | A | A | L | Q | P | Q | R | L | T | R | L | A | L | G | E | - | G | T | I | P | G | A | S | - | - | - | - | P | S | P | E | L | - | - | - | - | - | - | - | I | P | D | - | - | - | - |
| 106 UniRef90\_UPI0016124E74\_10\_283 | A | H | R | W | A | L | D | R | P | E | Q | V | Q | R | L | A | V | L | D | - | - | I | A | P | T | R | A | - | - | - | - | M | W | Q | R | - | - | - | - | - | - | - | - | - | M | D | - | - | - | - |
| 107 UniRef90\_A0A263DFR4\_27\_299 | G | F | A | L | A | A | D | H | P | E | R | V | T | R | L | A | V | T | E | - | A | A | I | P | G | V | S | - | - | - | - | A | D | P | A | F | - | - | - | - | - | - | - | F | G | S | - | - | - | - |
| 108 UniRef90\_A0A0U5F3A6\_35\_313 | G | Y | A | M | A | A | D | R | P | E | R | I | A | R | I | A | L | G | E | - | A | I | I | P | G | L | S | - | - | - | - | A | S | P | P | L | - | - | - | - | - | - | - | I | S | D | - | - | - | - |
| 109 UniRef90\_A0A2N3KZL1\_38\_324 | T | Y | P | M | A | V | S | H | Q | K | D | I | R | K | L | I | F | M | E | - | A | P | I | P | D | H | R | - | - | - | - | L | Y | D | F | P | - | - | - | - | - | - | - | A | F | T | - | - | - | - |
| 110 UniRef90\_A0A1X7C0M0\_25\_283 | A | F | A | W | A | A | A | R | P | E | D | I | S | H | L | V | L | I | D | - | A | L | L | P | G | L | G | - | - | - | - | L | E | E | A | M | - | - | - | - | - | - | - | N | V | A | - | - | - | - |
| 111 UniRef90\_A0A078M0V2\_4\_284 | A | R | R | L | A | Y | D | N | P | E | L | I | N | R | L | A | L | I | E | - | - | I | M | P | M | E | Y | - | - | - | - | V | Y | S | L | S | - | - | - | - | - | - | - | - | - | - | - | - | - | - |
| 112 UniRef90\_A0A0F4Z6H8\_20\_271 | L | Y | Q | L | A | V | R | H | P | S | R | V | K | S | L | I | F | Q | E | - | M | M | L | P | G | F | G | - | - | - | - | L | E | D | W | A | - | - | - | - | - | - | - | K | F | D | - | - | - | - |
| 113 UniRef90\_UPI00161D2319\_22\_292 | G | Y | A | L | A | A | S | H | R | E | R | V | T | G | L | V | G | A | E | - | S | I | L | P | G | L | S | - | - | - | - | P | S | P | P | L | - | - | - | - | - | - | - | L | S | D | - | - | - | - |
| 114 UniRef90\_A0A1Q8LX67\_38\_327 | G | F | R | Y | A | A | Q | Y | P | D | E | V | L | R | Y | A | H | L | D | - | Y | P | L | P | G | P | A | - | - | - | - | L | S | A | A | - | - | - | - | - | - | - | - | - | - | - | - | - | - | - |
| 115 UniRef90\_A0A1Q9S2P6\_18\_309 | A | F | Q | Y | A | V | Q | F | P | A | G | V | A | A | L | A | Y | L | D | - | L | P | L | P | G | P | V | - | - | - | - | V | D | A | A | - | - | - | - | - | - | - | - | - | - | - | - | - | - | - |
| 116 UniRef90\_A0A5M7BQA6\_8\_281 | A | H | R | W | G | L | D | R | P | D | Q | V | E | R | V | A | L | L | D | - | - | I | V | P | T | R | E | - | - | - | - | M | W | R | R | - | - | - | - | - | - | - | - | - | M | D | - | - | - | - |
| 117 UniRef90\_A0A3N6GJS2\_33\_314 | A | F | S | F | A | V | N | H | P | E | A | T | R | R | V | A | L | L | D | - | V | L | H | P | D | A | S | - | - | - | - | M | Y | Q | I | P | - | - | - | - | - | - | - | V | L | P | - | - | - | - |
| 118 UniRef90\_A0A0Q6MZD2\_36\_308 | A | F | Q | F | A | A | Q | H | P | E | Q | V | R | R | L | V | L | I | D | - | A | P | V | P | G | V | G | - | - | - | - | P | W | E | E | I | - | - | - | - | - | - | - | L | K | N | - | - | - | - |
| 119 UniRef90\_UPI00052520C0\_22\_293 | S | Y | A | L | A | A | D | H | R | D | R | V | T | G | L | V | L | A | E | - | A | A | L | P | G | I | S | - | - | - | - | D | V | P | S | I | - | - | - | - | - | - | - | I | P | A | - | - | - | - |
| 120 UniRef90\_A0A1Q4XCF8\_2\_276 | A | F | S | F | A | A | N | H | P | Q | A | V | R | K | V | A | L | L | D | - | V | L | H | P | D | A | S | - | - | - | - | L | Y | Q | I | P | - | - | - | - | - | - | - | M | L | P | - | - | - | - |
| 121 UniRef90\_A0A328LZX6\_33\_305 | G | Y | A | F | V | A | E | H | R | D | R | V | R | R | F | V | L | M | D | - | A | P | I | P | G | I | G | - | - | - | - | P | W | D | E | I | - | - | - | - | - | - | - | L | K | S | - | - | - | - |
| 122 UniRef90\_A0A2V8S6L4\_40\_311 | A | Y | A | Y | A | A | Q | F | P | Q | E | T | D | R | L | V | L | M | D | - | A | F | L | P | G | I | G | - | - | - | - | N | W | K | D | V | - | - | - | - | - | - | - | W | L | M | - | - | - | - |
| 123 UniRef90\_A0A1I5EB88\_8\_281 | A | H | R | W | G | L | D | R | P | D | Q | V | D | R | L | A | L | L | D | - | - | I | V | P | T | R | E | - | - | - | - | M | W | R | L | - | - | - | - | - | - | - | - | - | M | N | - | - | - | - |
| 124 UniRef90\_UPI0014147ABD\_39\_305 | A | Y | A | L | A | A | D | H | P | D | R | V | D | R | V | A | L | A | E | I | P | G | P | P | G | A | A | - | - | - | - | P | S | P | P | L | - | - | - | - | - | - | - | F | S | P | - | - | - | - |
| 125 UniRef90\_A0A1I7DL13\_6\_274 | A | Y | A | F | A | A | K | Y | R | D | R | C | K | S | L | T | L | I | E | - | A | P | L | W | G | I | V | - | - | - | - | S | D | E | V | P | - | - | - | - | - | - | - | D | L | A | - | - | - | - |
| 126 UniRef90\_A0A3N7EDS3\_31\_305 | G | Y | A | F | A | A | Q | N | R | D | R | V | T | R | F | V | L | I | D | - | A | P | V | P | G | V | G | - | - | - | - | P | W | E | E | I | - | - | - | - | - | - | - | L | K | N | - | - | - | - |
| 127 UniRef90\_UPI0016225F9B\_4\_275 | A | H | R | Y | A | L | D | H | P | D | E | V | E | R | L | V | V | M | D | - | - | I | I | P | T | R | E | - | - | - | - | M | W | R | R | F | - | - | - | - | - | - | - | D | M | D | - | - | - | - |
| 128 UniRef90\_UPI000997AE50\_107\_382 | A | Y | P | L | A | A | E | Y | R | Q | Q | V | S | G | L | F | M | I | D | - | F | P | L | V | G | K | N | - | - | - | - | L | R | F | A | - | - | - | - | - | - | - | - | - | - | - | - | - | - | - |
| 129 UniRef90\_A0A0N7F3H5\_6\_275 | A | H | R | W | A | L | D | R | P | D | E | V | T | R | L | A | I | L | D | - | - | I | L | P | T | R | D | - | - | - | - | M | W | Q | R | - | - | - | - | - | - | - | - | - | M | T | - | - | - | - |
| 130 UniRef90\_A0A3Q8F441\_11\_281 | A | T | R | L | A | K | D | H | P | Q | R | V | D | R | L | V | V | M | D | - | - | N | V | P | T | R | I | - | - | - | - | V | A | R | E | - | - | - | - | - | - | - | - | - | L | N | - | - | - | - |
| 131 UniRef90\_UPI001487D2FB\_23\_300 | A | F | A | Y | A | A | R | Y | P | E | K | T | E | R | L | V | V | M | D | - | A | P | V | P | G | I | G | - | - | - | - | P | W | D | E | I | - | - | - | - | - | - | - | V | R | N | - | - | - | - |
| 132 UniRef90\_A0A437GNC7\_38\_307 | G | Y | A | M | A | A | D | Q | P | E | R | I | A | R | I | A | L | G | E | - | A | I | I | P | G | V | A | - | - | - | - | V | S | P | P | L | - | - | - | - | - | - | - | I | S | D | - | - | - | - |
| 133 UniRef90\_A0A1H3SWJ7\_6\_283 | A | H | R | W | G | L | E | R | P | H | E | I | S | R | L | M | L | L | S | - | - | V | V | P | T | L | E | - | - | - | - | M | W | R | H | - | - | - | - | - | - | - | - | - | M | D | - | - | - | - |
| 134 UniRef90\_UPI0013DD6281\_49\_329 | G | F | Q | Y | A | A | Q | Y | P | G | E | V | R | A | Y | A | H | L | D | - | Y | P | L | P | G | P | A | - | - | - | - | L | S | A | A | - | - | - | - | - | - | - | - | - | - | - | - | - | - | - |
| 135 UniRef90\_I0L7C1\_27\_272 | A | F | L | L | G | A | L | H | P | A | R | V | R | R | L | V | L | M | E | - | A | T | L | P | G | L | P | - | - | - | - | - | - | - | - | - | - | - | - | - | - | - | G | G | F | P | - | - | - | - |
| 136 UniRef90\_A0A260DLM1\_45\_326 | A | F | R | Y | A | A | Q | Y | P | S | E | V | D | Q | L | A | Y | L | D | - | L | P | L | P | G | P | A | - | - | - | - | I | D | G | Q | - | - | - | - | - | - | - | - | - | - | - | - | - | - | - |
| 137 UniRef90\_UPI0005BDD2EC\_7\_280 | A | H | R | W | G | L | D | R | P | Q | E | V | E | R | L | A | L | L | D | - | - | I | I | P | T | R | E | - | - | - | - | M | L | A | R | - | - | - | - | - | - | - | - | - | M | D | - | - | - | - |
| 138 UniRef90\_A0A6G9YBC1\_27\_296 | A | H | R | W | A | L | D | R | P | D | Q | V | T | K | L | A | V | L | D | - | - | V | I | P | T | R | E | - | - | - | - | V | W | R | L | - | - | - | - | - | - | - | - | - | M | N | - | - | - | - |
| 139 UniRef90\_UPI00145FA88A\_8\_280 | G | H | R | W | A | L | D | R | P | D | Q | V | E | R | L | S | V | L | D | - | - | I | V | P | T | R | E | - | - | - | - | M | W | R | R | - | - | - | - | - | - | - | - | - | W | N | - | - | - | - |
| 140 UniRef90\_A0A2W0B1K8\_46\_299 | A | Y | A | Y | A | A | M | Y | P | D | K | V | E | R | L | V | V | M | D | - | A | P | I | P | G | I | G | - | - | - | - | P | W | K | E | I | - | - | - | - | - | - | - | L | L | N | - | - | - | - |
| 141 UniRef90\_A0A5B8G622\_28\_299 | G | F | A | F | A | A | E | H | R | D | R | V | K | R | L | V | L | M | D | - | A | P | L | P | G | V | G | - | - | - | - | P | W | E | E | I | - | - | - | - | - | - | - | L | K | N | - | - | - | - |
| 142 UniRef90\_A0A4S1XGQ8\_21\_302 | A | Y | A | F | A | V | Q | N | Q | K | R | V | T | R | L | V | L | I | D | - | A | P | V | P | G | I | G | - | - | - | - | P | W | E | E | I | - | - | - | - | - | - | - | L | K | N | - | - | - | - |
| 143 UniRef90\_A0A251YJ72\_8\_294 | A | Y | R | L | A | A | D | H | P | E | S | V | R | S | L | T | A | V | E | - | M | G | L | A | G | F | G | - | - | - | - | L | E | G | L | G | - | - | - | - | - | - | - | D | I | T | - | - | - | - |
| 144 UniRef90\_UPI000A0527D2\_65\_318 | A | L | G | Y | A | L | R | Y | R | D | D | V | V | S | L | T | T | M | E | - | A | P | L | P | G | S | D | - | - | - | - | Y | Y | E | E | R | - | - | - | - | - | - | - | K | V | A | - | - | - | - |
| 145 UniRef90\_A0A4V2S7H9\_8\_280 | A | H | R | W | A | L | D | R | P | A | E | V | E | R | L | A | V | L | D | - | - | I | I | P | T | R | E | - | - | - | - | M | W | R | R | - | - | - | - | - | - | - | - | - | L | D | - | - | - | - |
| 146 UniRef90\_UPI00131B216A\_18\_247 | A | Y | S | F | A | A | N | H | P | E | A | A | R | R | I | A | L | L | D | - | V | M | H | P | D | E | S | - | - | - | - | W | F | Q | H | T | - | - | - | - | - | - | - | V | L | P | - | - | - | - |
| 147 UniRef90\_A0A1G8T9Y6\_5\_243 | A | Y | A | Y | A | A | A | H | P | R | D | V | R | R | L | V | L | S | E | - | A | P | I | P | D | K | S | - | - | - | - | L | Y | G | F | A | - | - | - | - | - | - | - | S | L | T | - | - | - | - |
| 148 UniRef90\_UPI00161F613F\_5\_254 | V | Y | R | L | A | A | T | H | P | E | D | V | R | T | L | I | A | V | E | - | M | G | L | A | G | Y | G | - | - | - | - | L | E | A | L | A | - | - | - | - | - | - | - | D | V | T | - | - | - | - |
| 149 UniRef90\_UPI00142071FA\_80\_362 | G | Y | A | L | A | A | D | H | R | D | R | V | S | R | L | V | V | A | E | I | P | G | P | P | G | V | E | D | P | E | H | P | A | P | P | L | - | - | - | - | - | - | - | F | L | P | - | - | - | - |
| 150 UniRef90\_A0A6G7Q8F6\_7\_219 | A | F | W | V | A | S | F | H | P | E | R | V | E | R | L | V | A | L | N | - | A | C | H | P | G | V | F | - | - | - | - | G | P | Y | L | R | - | - | - | - | - | - | - | R | H | P | - | - | - | - |

  
  

|  |  |  |  |  |  |  |  |  |  |  |  |  |  |  |  |  |  |  |  |  |  |  |  |  |  |  |  |  |  |  |  |  |  |  |  |  |  |  |  |  |  |  |  |  |  |  |  |  |  |  |
| --- | --- | --- | --- | --- | --- | --- | --- | --- | --- | --- | --- | --- | --- | --- | --- | --- | --- | --- | --- | --- | --- | --- | --- | --- | --- | --- | --- | --- | --- | --- | --- | --- | --- | --- | --- | --- | --- | --- | --- | --- | --- | --- | --- | --- | --- | --- | --- | --- | --- | --- |
| **001 Input\_protein\_seq** | - | - | - | - | P | - | G | N | A | - | W | R | T | W | H | F | A | F | H | T | V | - | A | D | - | L | P | E | T | L | I | A | G | K | E | R | E | Y | L | - | D | W | F | L | R | R | K | - | - | - |
| 002 UniRef90\_UPI001430CB85\_4\_307 | - | - | - | - | P | - | D | R | A | - | W | R | T | W | H | F | A | F | H | S | I | - | P | D | - | L | P | E | L | L | I | A | G | K | E | R | A | Y | L | - | D | W | F | L | R | R | K | - | - | - |
| 003 UniRef90\_UPI00055F8D66\_10\_306 | - | - | - | - | P | - | E | R | A | - | W | R | T | W | H | F | A | F | H | A | V | - | A | D | - | L | P | E | M | L | I | A | G | R | E | R | E | Y | L | - | D | W | F | L | R | R | K | - | - | - |
| 004 UniRef90\_UPI00161D8BA7\_15\_312 | - | - | - | - | P | - | D | R | A | - | W | R | T | W | H | F | A | F | H | A | I | - | P | D | - | L | P | E | L | L | I | A | G | H | E | R | E | Y | L | - | D | W | F | L | R | R | K | - | - | - |
| 005 UniRef90\_A0A4R7GLU7\_7\_305 | - | - | - | - | P | - | D | T | S | - | W | K | T | W | H | F | A | F | H | T | L | - | P | D | - | L | P | E | A | L | I | T | G | N | E | R | V | Y | L | - | D | W | F | L | R | R | K | - | - | - |
| 006 UniRef90\_UPI00041CD164\_24\_309 | - | - | - | - | A | - | D | R | A | - | W | R | T | W | H | F | A | F | H | A | L | - | P | D | - | L | P | E | T | L | I | T | G | R | E | R | E | Y | L | - | E | W | F | L | R | R | K | - | - | - |
| 007 UniRef90\_I0QSF8\_15\_298 | - | - | - | - | G | - | D | K | G | - | W | R | T | W | H | F | A | F | H | A | I | - | A | D | - | L | P | E | E | L | I | A | G | K | E | R | I | Y | L | - | N | W | F | L | R | R | K | - | - | - |
| 008 UniRef90\_A0A2N5ENH7\_9\_301 | - | - | - | - | P | - | D | N | A | - | W | K | T | W | H | F | A | F | H | L | L | - | P | D | - | L | P | E | A | L | I | T | G | R | E | E | I | Y | L | - | D | W | F | L | R | R | K | - | - | - |
| 009 UniRef90\_A0A0Q8E2M6\_29\_312 | - | - | - | - | P | - | E | Q | A | - | W | R | T | W | H | F | A | F | H | V | I | - | P | D | - | L | P | E | M | L | I | T | G | H | E | R | Q | Y | L | - | A | W | F | L | R | R | K | - | - | - |
| 010 UniRef90\_A0A0F5XY46\_6\_287 | - | - | - | - | G | - | G | N | A | - | W | K | T | W | H | F | A | F | H | C | V | - | S | D | - | L | P | E | A | L | I | A | G | R | E | A | I | Y | L | - | D | W | F | F | N | R | K | - | - | - |
| 011 UniRef90\_A0A3N4NUX5\_4\_284 | - | - | - | - | S | - | Q | S | A | - | W | K | T | W | H | F | A | F | H | L | V | - | E | D | - | L | P | E | T | L | I | S | G | R | E | A | I | Y | L | - | D | W | F | F | R | R | K | - | - | - |
| 012 UniRef90\_UPI000DDED2D9\_11\_293 | - | - | - | - | S | - | D | V | A | - | W | R | T | W | H | V | A | F | H | N | L | - | P | D | - | L | P | E | A | L | I | R | D | R | E | D | I | Y | L | - | R | W | F | L | Q | R | K | - | - | - |
| 013 UniRef90\_UPI000DDED53B\_10\_292 | - | - | - | - | S | - | D | K | S | - | W | K | T | W | H | F | G | F | H | A | V | - | P | D | - | L | P | E | I | L | L | E | G | R | E | R | A | Y | L | - | E | W | F | F | R | T | K | - | - | - |
| 014 UniRef90\_A0A1G4JKM0\_6\_288 | - | - | - | - | S | - | D | S | A | - | W | R | L | W | H | F | P | F | H | A | V | - | L | D | - | L | P | E | L | L | I | R | G | K | E | R | V | Y | L | - | E | W | F | L | K | R | K | - | - | - |
| 015 UniRef90\_UPI0016728815\_21\_305 | - | - | - | - | P | - | E | R | A | - | W | K | T | W | H | F | A | F | H | L | V | - | P | D | - | L | P | E | T | L | L | A | G | R | E | R | E | Y | V | - | G | W | F | L | K | A | K | - | - | - |
| 016 UniRef90\_A0A3N2H8D2\_14\_297 | - | - | - | - | P | - | D | R | A | - | W | K | T | W | H | F | A | F | H | T | V | - | P | E | - | L | P | E | T | L | L | A | G | R | E | R | E | Y | V | - | G | W | F | L | T | V | K | - | - | - |
| 017 UniRef90\_UPI0005644DD1\_9\_307 | - | - | - | - | P | - | A | R | A | - | W | K | T | W | H | F | A | F | H | L | V | - | P | D | - | L | P | E | T | L | L | A | G | R | E | R | E | Y | V | - | S | W | F | L | K | A | K | - | - | - |
| 018 UniRef90\_A0A6B2VDS0\_18\_308 | - | - | - | - | P | - | D | R | A | - | W | K | T | W | H | F | A | F | H | M | V | - | P | D | - | L | P | E | T | L | L | A | G | R | E | R | D | Y | V | - | G | W | F | L | K | M | K | - | - | - |
| 019 UniRef90\_A0A6G3RZZ1\_25\_307 | - | - | - | - | P | - | E | Q | A | - | W | K | T | W | H | F | A | F | H | L | V | - | P | E | - | L | P | E | T | L | L | T | G | R | E | R | E | Y | V | - | D | W | F | L | K | A | K | - | - | - |
| 020 UniRef90\_A0A209C7X3\_21\_305 | - | - | - | - | P | - | E | Q | A | - | W | K | T | W | H | F | A | F | H | L | V | - | P | D | - | L | P | E | T | L | L | A | G | R | E | R | E | Y | V | - | G | W | F | L | K | A | K | - | - | - |
| 021 UniRef90\_UPI001269E708\_26\_309 | - | - | - | - | P | - | E | Q | A | - | W | K | T | W | H | F | A | F | H | L | V | - | P | D | - | L | P | E | R | L | L | A | G | R | E | G | E | Y | V | - | G | W | F | L | K | A | K | - | - | - |
| 022 UniRef90\_A0A2M9J7W9\_25\_306 | - | - | - | - | P | - | E | R | A | - | W | K | T | W | H | F | A | F | H | L | V | - | P | E | - | L | P | E | T | L | L | T | G | R | E | R | D | Y | V | - | G | W | F | L | K | T | K | - | - | - |
| 023 UniRef90\_A0A6G9F3J8\_23\_307 | - | - | - | - | P | - | E | R | A | - | W | K | T | W | H | F | A | F | H | L | V | - | P | G | - | L | P | E | T | L | L | T | G | R | E | H | E | Y | V | - | G | W | F | L | K | A | K | - | - | - |
| 024 UniRef90\_A0A1H2UZ41\_14\_292 | - | - | - | - | P | - | - | H | N | - | W | K | A | W | H | F | F | F | H | Q | V | - | A | D | - | L | P | E | I | L | I | T | G | R | E | R | S | Y | I | - | E | W | F | F | Q | R | K | - | - | - |
| 025 UniRef90\_UPI0012B05B7D\_16\_286 | - | - | - | - | P | - | - | D | N | - | W | R | N | W | H | F | L | F | N | P | I | - | A | D | - | L | P | E | A | L | L | A | G | R | E | R | I | L | I | - | E | W | F | F | K | N | K | - | - | - |
| 026 UniRef90\_A0A5C8T429\_18\_294 | - | - | - | - | P | - | E | R | I | - | G | R | N | W | H | F | F | F | N | A | L | - | S | D | - | L | P | E | T | L | L | A | G | R | E | R | E | F | L | - | S | W | L | F | Q | A | K | - | - | - |
| 027 UniRef90\_UPI0012E86E3E\_10\_309 | - | - | - | - | S | T | E | A | N | - | V | K | T | W | H | F | A | F | N | R | L | - | D | D | - | L | P | E | I | L | L | Q | G | R | E | R | E | F | L | - | T | W | L | F | R | T | K | - | - | - |
| 028 UniRef90\_A0A1A9HTL2\_13\_300 | - | - | - | - | G | D | D | A | N | - | L | K | S | W | H | F | A | F | N | R | L | - | P | D | - | L | P | E | I | L | V | Q | G | H | E | R | A | Y | L | - | G | W | L | F | A | Q | K | - | - | - |
| 029 UniRef90\_A0A0R1V0C7\_7\_281 | - | - | - | - | A | - | - | E | S | - | W | R | S | W | H | F | L | F | N | V | I | - | P | D | - | L | P | E | E | L | L | K | G | K | E | R | V | L | L | - | E | W | F | F | S | N | K | - | - | - |
| 030 UniRef90\_A0A3N1M9Y1\_5\_282 | - | - | - | - | - | - | S | Q | G | - | G | R | R | W | H | H | A | F | H | R | T | - | L | D | - | L | P | E | A | L | V | T | G | R | E | R | P | Y | L | - | S | W | F | Y | A | E | F | - | - | - |
| 031 UniRef90\_A0A158JUX7\_17\_294 | - | - | - | - | K | - | - | - | - | - | G | G | S | W | H | F | G | F | N | M | Q | - | G | D | - | L | A | E | A | L | V | S | G | R | E | R | L | F | L | - | E | Y | L | F | K | R | D | R | I | G |
| 032 UniRef90\_A0A0U3LIL8\_12\_296 | - | - | - | - | T | D | E | I | N | - | K | R | T | W | H | F | S | F | N | R | L | - | P | D | - | L | P | E | I | L | V | R | G | H | E | R | P | Y | L | - | T | W | L | F | T | Q | K | - | - | - |
| 033 UniRef90\_A0A248JS33\_7\_301 | - | - | - | - | S | D | E | A | N | - | A | R | T | W | H | F | A | F | N | R | L | - | D | D | - | L | P | E | L | L | I | Q | G | R | E | R | A | Y | L | - | G | H | L | F | N | A | K | - | - | - |
| 034 UniRef90\_A0A1Y0REZ4\_12\_287 | - | - | - | - | R | - | - | - | - | - | G | G | S | W | I | L | G | F | N | M | T | - | P | D | - | I | P | E | A | L | V | A | G | R | E | R | L | Y | F | E | S | A | L | Y | K | P | A | - | - | - |
| 035 UniRef90\_A0A0M2WRU5\_3\_280 | - | - | - | - | P | - | S | D | N | - | I | F | L | W | H | F | M | F | N | Q | V | - | L | D | - | L | P | E | M | L | T | A | G | K | E | R | E | Y | I | - | R | F | I | F | D | R | W | - | - | - |
| 036 UniRef90\_A0A0N8GFM1\_6\_279 | - | - | - | - | - | - | S | Q | G | - | G | R | R | W | H | H | A | F | F | R | T | - | L | D | - | L | P | E | A | L | C | I | G | R | E | E | L | V | L | - | G | W | L | F | D | N | Y | - | - | - |
| 037 UniRef90\_A0A252EMP1\_7\_277 | - | - | - | - | - | - | D | A | S | - | K | E | Y | W | H | F | G | F | H | Q | E | - | A | D | - | L | P | E | A | L | L | A | G | R | E | R | E | Y | L | - | S | F | F | F | K | R | Y | - | - | - |
| 038 UniRef90\_W0A6K5\_40\_320 | - | - | - | - | P | - | Q | D | N | - | I | F | L | W | H | F | M | F | N | Q | V | - | Q | D | - | L | P | E | T | L | T | A | G | K | E | K | E | Y | L | - | N | F | I | F | D | H | W | - | - | - |
| 039 UniRef90\_A0A3S1SM66\_23\_304 | - | - | - | - | V | H | E | T | N | - | L | R | S | W | H | F | P | F | N | Q | L | - | D | D | - | L | P | E | L | L | L | A | G | R | E | K | A | F | L | - | T | W | L | F | R | A | K | - | - | - |
| 040 UniRef90\_A0A2E5L5C6\_7\_286 | - | - | - | - | - | - | S | E | G | - | S | R | R | W | H | H | Q | F | H | S | T | - | P | D | - | L | A | E | A | L | T | Q | G | R | E | G | I | Y | L | - | S | W | F | Y | R | T | F | - | - | - |
| 041 UniRef90\_M5D1B4\_14\_289 | - | - | - | - | P | - | A | D | N | - | I | F | L | W | H | F | M | F | N | Q | V | - | A | D | - | L | P | E | A | L | I | T | G | R | E | R | A | Y | L | - | E | F | M | F | D | R | W | - | - | - |
| 042 UniRef90\_UPI0016845BA7\_31\_303 | - | - | - | - | - | - | - | - | - | - | Q | S | A | W | H | F | Q | F | H | A | V | - | P | D | - | L | P | E | A | L | V | T | G | R | E | R | I | Y | F | - | S | H | F | Y | E | N | Y | - | - | - |
| 043 UniRef90\_UPI0014648199\_14\_291 | - | - | - | - | P | - | E | Q | N | - | I | F | L | W | H | F | L | F | N | Q | V | - | Q | D | - | L | P | E | A | L | I | A | G | R | E | E | T | Y | L | - | N | F | M | F | D | R | W | - | - | - |
| 044 UniRef90\_K9RUM7\_12\_289 | - | - | - | - | - | - | - | N | A | - | A | K | V | W | Q | F | Y | F | H | K | V | - | P | D | - | L | A | D | S | L | V | A | G | K | E | L | E | Y | L | - | S | W | Y | F | T | N | K | - | - | - |
| 045 UniRef90\_A0A1Q2U086\_13\_290 | - | - | - | - | - | - | - | N | A | - | A | K | V | W | Q | F | Y | F | H | K | V | - | P | D | - | L | A | D | S | L | V | A | G | K | E | K | E | Y | L | - | S | W | Y | F | T | N | K | - | - | - |
| 046 UniRef90\_B9JMN1\_3\_282 | - | - | - | - | Q | - | P | N | G | - | N | F | L | W | H | M | G | F | Q | S | V | - | P | D | - | I | P | F | T | L | I | S | G | R | E | D | L | Y | L | - | R | W | F | F | Q | T | Y | - | - | - |
| 047 UniRef90\_A0A2X1TAZ7\_33\_319 | - | - | - | - | P | - | E | E | N | - | I | F | L | W | H | F | M | F | N | Q | V | - | P | D | - | L | P | E | M | L | T | A | G | K | E | Q | D | Y | L | - | N | F | I | F | D | H | W | - | - | - |
| 048 UniRef90\_UPI00131A8152\_6\_279 | - | - | - | - | - | - | S | Q | G | - | G | A | R | W | H | H | A | F | H | N | T | - | I | D | - | L | P | E | E | L | I | A | G | R | E | E | I | Y | L | - | G | W | F | Y | R | N | Y | - | - | - |
| 049 UniRef90\_F7YB04\_47\_316 | - | - | - | - | - | - | - | - | - | - | P | R | T | W | H | F | R | F | H | S | V | - | R | D | - | V | P | E | M | L | I | A | G | R | E | L | E | Y | L | - | K | W | F | H | N | A | E | - | - | - |
| 050 UniRef90\_A0A1A3M1Z8\_5\_294 | - | - | - | - | N | - | F | R | E | - | N | K | L | W | H | F | H | M | H | G | A | - | A | D | N | V | A | E | T | L | I | A | G | N | E | F | P | Y | L | - | R | S | F | Y | V | R | L | - | - | - |
| 051 UniRef90\_UPI0004C32CE1\_32\_312 | - | - | - | - | P | - | P | G | Q | - | F | S | L | W | W | F | A | F | N | Q | V | - | Q | T | - | L | P | E | Q | L | L | A | G | R | F | R | H | L | I | - | D | W | L | F | A | N | T | - | - | - |
| 052 UniRef90\_A0A0J6NQJ9\_12\_287 | - | - | - | - | P | - | E | E | N | - | I | F | L | W | H | F | M | F | N | Q | L | - | A | D | - | L | P | E | T | L | I | A | G | R | E | R | A | Y | L | - | D | F | M | F | Q | H | W | - | - | - |
| 053 UniRef90\_A0A534ESC2\_32\_321 | - | - | - | - | S | E | A | A | N | - | L | K | S | W | Q | F | A | F | N | R | L | - | D | D | - | L | P | E | I | L | V | Q | G | R | E | R | A | Y | L | - | A | W | I | Y | A | T | K | - | - | - |
| 054 UniRef90\_A0A1H1Z302\_21\_301 | - | - | - | - | A | - | A | A | S | - | Q | R | L | W | H | F | G | F | N | R | L | - | T | D | - | L | N | E | E | L | V | R | G | R | E | R | L | F | F | - | G | Y | Q | F | A | K | K | - | - | - |
| 055 UniRef90\_A0A158DLE6\_24\_295 | - | - | - | - | T | - | - | - | - | - | G | G | S | W | H | F | G | F | N | M | A | - | G | D | - | I | S | E | E | L | V | R | G | R | E | A | L | F | V | - | R | H | F | V | R | R | D | T | V | G |
| 056 UniRef90\_A0A163VVR8\_16\_280 | - | - | - | - | P | - | E | E | N | - | I | F | L | W | H | F | M | F | N | Q | L | - | A | D | - | L | P | E | T | L | T | Q | G | R | E | Q | E | Y | L | - | G | Y | I | F | N | R | W | - | - | - |
| 057 UniRef90\_UPI000369F0DF\_7\_289 | - | - | - | - | P | - | S | D | N | - | I | F | L | W | H | F | L | F | N | Q | I | - | L | D | - | L | P | E | F | L | T | S | G | R | E | K | E | Y | L | - | G | F | I | F | D | K | W | - | - | - |
| 058 UniRef90\_UPI00098F4FB4\_43\_309 | - | - | - | - | - | - | - | - | - | - | P | R | T | W | H | F | R | F | H | S | V | - | R | D | - | V | P | E | M | L | I | A | G | R | E | L | E | Y | L | - | K | W | F | H | N | A | E | - | - | - |
| 059 UniRef90\_UPI0015A1AD76\_10\_292 | - | - | - | G | P | D | W | H | N | - | G | V | N | W | H | A | A | L | F | T | L | - | P | D | - | V | A | E | S | F | M | G | G | Q | E | R | K | F | L | - | S | W | I | F | W | H | L | - | - | - |
| 060 UniRef90\_UPI001689F051\_63\_318 | - | - | - | - | A | N | P | L | T | - | G | G | S | W | H | F | G | F | N | M | V | - | P | D | - | L | P | E | L | L | L | A | G | R | E | R | A | Y | L | - | E | F | M | F | H | R | D | - | - | - |
| 061 UniRef90\_UPI000DD77D66\_43\_313 | - | - | - | - | - | - | - | - | - | - | P | R | T | W | H | F | R | F | H | A | V | - | R | D | - | V | P | E | M | L | I | S | G | R | E | F | E | Y | L | - | K | W | F | H | N | A | E | - | - | - |
| 062 UniRef90\_A0A3M0I8T4\_31\_307 | - | - | - | - | R | - | E | G | N | - | T | R | L | W | H | F | A | F | N | R | L | - | A | E | - | L | N | E | E | L | V | R | G | R | E | H | L | Y | F | - | G | A | Q | F | A | T | K | - | - | - |
| 063 UniRef90\_UPI000377E2D1\_4\_271 | - | - | - | - | - | - | S | Q | G | - | G | R | R | W | H | H | A | F | L | G | T | - | L | D | - | L | P | E | A | L | I | S | G | R | E | E | V | F | L | - | R | W | F | Y | E | H | Y | - | - | - |
| 064 UniRef90\_UPI0016198B53\_15\_286 | - | - | - | - | E | - | A | Q | N | - | D | L | L | W | H | F | S | F | N | R | R | - | R | S | - | V | N | E | L | L | V | R | G | R | E | H | I | Y | Y | - | G | D | Q | F | Q | L | K | - | - | - |
| 065 UniRef90\_UPI0013C0217D\_48\_325 | - | - | - | - | K | - | P | G | T | G | F | P | L | W | W | Y | A | F | N | Q | V | - | Q | G | - | L | P | E | Q | L | V | A | G | R | V | R | H | L | I | - | D | W | F | F | A | Q | S | - | - | - |
| 066 UniRef90\_C7PGN2\_5\_260 | - | - | - | - | P | - | - | N | N | - | K | G | L | W | W | F | A | F | H | Q | V | - | H | Q | - | L | P | E | L | L | V | A | G | K | E | R | E | Y | L | - | Q | W | F | Y | H | N | S | - | - | - |
| 067 UniRef90\_A0A1F7Q7Q2\_7\_275 | - | - | - | - | R | - | V | S | A | - | R | A | R | W | H | W | F | F | H | Q | V | - | P | D | - | L | P | E | A | L | T | A | G | R | E | D | V | Y | L | - | R | F | L | Y | R | S | W | - | - | - |
| 068 UniRef90\_A0A2V7F1K9\_9\_274 | - | - | - | - | R | - | A | G | A | - | R | A | R | W | H | W | F | F | H | Q | V | - | P | D | - | L | P | E | A | L | T | A | G | R | E | D | V | Y | L | - | R | Y | M | Y | K | A | W | - | - | - |
| 069 UniRef90\_UPI001616C592\_31\_308 | - | - | - | - | D | R | W | S | S | - | D | F | A | W | H | Y | N | F | N | R | T | - | T | D | - | I | N | E | R | L | V | E | G | R | E | H | L | Y | F | - | G | H | Q | F | A | T | K | - | - | - |
| 070 UniRef90\_UPI001456849A\_71\_360 | - | - | - | - | - | - | - | - | - | - | - | A | P | W | H | F | A | F | N | K | V | - | P | D | - | V | P | E | M | L | T | Q | G | R | E | R | L | Y | L | - | D | L | F | W | T | G | A | - | - | - |
| 071 UniRef90\_A0A2S8J6S6\_18\_282 | - | - | - | - | - | - | - | - | - | - | P | T | V | W | H | F | A | F | H | Q | N | - | R | D | - | L | P | E | F | L | I | S | G | R | E | Y | G | Y | V | - | E | S | F | L | R | N | R | - | - | - |
| 072 UniRef90\_A0A2I2GC12\_18\_280 | - | - | - | - | Y | - | E | T | N | - | L | R | L | W | Q | F | S | F | N | A | L | - | P | E | - | L | P | E | I | L | T | Q | G | R | E | R | E | L | L | - | T | W | F | F | N | L | K | - | - | - |
| 073 UniRef90\_A0A549T469\_41\_312 | - | - | - | - | - | - | - | - | - | - | G | G | A | W | H | Y | G | F | H | M | A | - | R | E | - | I | P | E | L | L | T | K | G | R | E | R | D | Y | I | - | T | A | Q | I | R | A | W | - | - | - |
| 074 UniRef90\_A0A5C4LJC6\_5\_278 | - | - | - | - | - | - | - | - | - | - | - | R | L | W | H | F | G | F | H | S | H | - | R | D | - | L | P | E | R | L | I | H | G | R | E | Y | D | Y | L | - | S | T | F | M | A | E | R | - | - | - |
| 075 UniRef90\_UPI001619D806\_9\_283 | - | - | - | - | A | - | D | V | G | - | R | G | Y | W | H | W | L | F | H | L | Q | - | P | D | - | L | P | E | R | L | A | G | Q | D | V | A | G | Y | L | - | G | Y | F | F | E | R | W | - | - | - |
| 076 UniRef90\_A0A239P1A5\_69\_347 | - | - | - | - | G | - | Q | L | N | - | S | L | L | W | H | F | P | F | N | R | L | - | E | G | - | I | N | E | Q | L | V | R | G | R | E | D | I | Y | F | - | R | Y | Q | F | A | T | K | - | - | - |
| 077 UniRef90\_UPI0005262A70\_5\_280 | - | - | - | - | N | - | - | - | - | - | G | G | S | W | H | V | G | F | L | A | A | - | P | G | - | I | P | E | L | L | L | A | G | R | E | R | E | F | L | T | G | F | A | F | P | A | M | - | - | - |
| 078 UniRef90\_A0A241XUQ6\_27\_314 | - | - | - | - | A | - | Q | G | E | - | S | L | V | W | H | F | S | F | F | A | A | - | D | D | R | L | A | E | T | L | I | A | G | K | E | R | F | F | L | - | E | H | F | I | K | S | H | - | - | - |
| 079 UniRef90\_A0A160FQ65\_32\_312 | - | - | - | - | D | R | W | L | S | - | D | L | L | W | H | F | N | F | N | R | A | - | R | E | - | V | N | E | R | L | V | E | G | R | E | E | I | Y | F | - | G | H | Q | F | A | S | K | - | - | - |
| 080 UniRef90\_UPI0006AE4C23\_4\_281 | - | - | - | - | A | - | S | A | E | - | T | N | A | W | H | F | G | F | N | Q | L | - | D | N | - | L | P | E | Q | L | L | A | D | R | Y | H | I | L | M | - | D | W | L | A | E | E | F | - | - | - |
| 081 UniRef90\_T0H3T7\_11\_281 | - | - | - | - | A | - | E | I | A | - | K | S | Y | W | F | F | L | F | H | L | I | - | P | D | - | L | P | E | T | L | I | A | G | K | E | E | Q | W | L | - | R | W | F | F | S | D | W | - | - | - |
| 082 UniRef90\_A0A4R2IH15\_5\_277 | - | - | - | - | - | - | - | - | - | - | P | R | L | W | H | F | G | F | N | R | L | - | P | E | - | L | N | E | V | L | V | R | G | R | E | E | E | F | F | - | G | Y | Q | F | T | A | K | - | - | - |
| 083 UniRef90\_UPI00140B863E\_4\_276 | - | - | - | - | R | - | - | - | - | - | Y | P | R | W | H | N | A | F | H | A | A | - | D | D | - | V | P | E | L | L | I | T | G | R | E | Q | E | Y | L | - | G | I | F | W | A | L | T | - | - | - |
| 084 UniRef90\_A0A3R9UXB7\_22\_297 | - | - | - | - | Q | - | D | G | R | - | R | H | L | W | W | F | A | F | N | Q | V | - | H | G | - | L | P | E | A | L | L | A | G | R | Y | R | L | L | L | - | D | W | L | F | T | H | G | - | - | - |
| 085 UniRef90\_UPI000F8D1465\_20\_303 | - | - | - | - | A | - | P | L | N | - | R | R | L | W | H | F | A | F | N | R | L | - | D | G | - | L | N | E | R | M | V | E | G | R | E | E | I | Y | F | - | G | D | Q | L | A | S | K | - | - | - |
| 086 UniRef90\_A0A6J4HYT7\_23\_307 | A | A | E | K | Q | - | A | G | H | - | P | Y | P | W | W | L | A | F | N | Q | V | - | K | E | - | L | P | E | Q | L | L | A | G | R | F | R | F | V | L | - | D | Y | V | F | K | H | A | - | - | - |
| 087 UniRef90\_A0A1D8SMR3\_19\_296 | - | - | - | - | P | - | E | V | N | - | E | L | A | W | H | F | V | F | N | R | L | - | A | E | - | I | N | E | R | M | V | S | G | R | E | E | I | Y | F | - | G | Y | Q | F | A | T | K | - | - | - |
| 088 UniRef90\_A0A4V2HUZ4\_48\_320 | - | - | - | - | - | - | - | - | - | - | G | G | A | W | H | Y | G | F | H | M | A | - | S | E | - | F | P | E | M | L | T | M | G | R | E | R | D | Y | I | - | R | A | Q | I | R | A | W | - | - | - |
| 089 UniRef90\_UPI0010582A1B\_5\_279 | - | - | - | - | - | - | - | - | - | - | - | R | L | W | H | F | G | F | H | M | N | - | R | D | - | L | P | E | R | L | I | H | G | R | E | Y | D | Y | V | - | S | T | F | M | A | E | R | - | - | - |
| 090 UniRef90\_UPI00166CE541\_54\_338 | - | - | - | - | - | - | A | K | G | - | P | G | F | W | N | F | G | Y | F | S | L | - | T | N | G | L | P | E | R | M | V | N | G | R | E | E | Q | W | V | - | E | R | F | V | D | S | L | - | - | - |
| 091 UniRef90\_A0A5C1HX54\_34\_307 | - | - | - | - | S | - | Q | V | R | - | S | Q | A | W | W | F | G | F | F | G | M | - | P | H | - | S | G | E | - | I | V | D | G | K | A | G | L | F | L | - | T | N | F | W | P | I | V | - | - | - |
| 092 UniRef90\_A0A4D4KET2\_34\_306 | - | - | - | - | G | - | D | A | N | - | D | R | L | W | H | F | G | F | N | R | L | - | P | D | - | L | N | E | Q | L | V | S | G | R | E | E | L | Y | F | - | G | H | Q | F | A | T | K | - | - | - |
| 093 UniRef90\_A0A1I1XP17\_9\_276 | - | - | - | - | - | - | - | - | - | - | P | R | G | W | H | V | A | F | H | G | A | - | R | D | - | I | A | E | R | L | V | E | G | R | E | R | V | Y | L | - | R | Y | M | I | D | V | R | - | - | - |
| 094 UniRef90\_A0A1D7VX60\_23\_299 | - | - | - | - | A | - | Q | V | N | - | K | R | L | W | H | I | P | F | N | R | L | - | T | D | - | V | N | E | Q | L | V | R | G | R | E | G | I | Y | F | - | G | W | Q | F | A | T | K | - | - | - |
| 095 UniRef90\_A0A2T4ZAU7\_6\_296 | - | - | - | - | - | - | E | A | A | - | K | K | Y | W | H | W | V | F | Q | I | V | - | P | E | - | L | P | E | E | L | I | A | G | R | E | E | S | Y | L | - | S | F | L | F | R | R | A | - | - | - |
| 096 UniRef90\_UPI00143A9489\_23\_300 | - | - | - | - | - | - | - | - | - | - | P | L | L | W | H | F | R | F | G | G | - | - | P | D | - | - | M | E | R | L | V | A | G | R | E | R | I | Y | L | - | D | R | F | W | N | D | F | - | - | - |
| 097 UniRef90\_A0A7C7NVI2\_6\_287 | - | - | - | - | P | - | A | V | V | - | R | E | R | W | H | S | Y | F | H | Q | Q | - | S | D | - | L | P | E | K | L | I | E | G | R | E | E | V | Y | L | - | R | H | I | L | R | D | W | - | - | - |
| 098 UniRef90\_UPI001661A3FF\_52\_332 | - | - | - | - | - | - | E | R | G | - | P | G | V | W | N | F | G | F | F | N | L | - | T | D | G | L | P | E | N | M | I | R | G | R | E | V | S | W | T | - | A | G | F | I | G | G | F | - | - | - |
| 099 UniRef90\_A0A6G4V4F1\_2\_276 | - | - | - | - | G | - | P | G | D | - | L | N | L | W | T | Y | P | L | S | Q | I | - | E | G | - | L | P | A | K | L | L | A | G | R | F | R | H | V | I | - | D | W | S | A | E | H | L | - | - | - |
| 100 UniRef90\_A0A260W032\_16\_294 | - | - | - | - | - | - | K | A | N | - | A | G | Y | W | F | F | T | F | L | A | V | - | P | D | - | L | P | E | T | L | I | A | G | K | E | R | E | F | L | - | T | Y | F | Y | R | T | W | - | - | - |
| 101 UniRef90\_UPI00036BBA85\_7\_295 | - | - | - | - | P | G | Q | M | A | - | K | G | Y | W | F | F | N | F | N | Q | L | - | V | D | - | L | P | E | A | L | I | T | G | R | E | E | I | W | L | - | R | H | W | L | S | S | W | - | - | - |
| 102 UniRef90\_A0A4R5QBW7\_9\_278 | - | - | - | - | - | - | - | - | - | - | A | G | R | W | H | H | G | F | H | W | E | - | D | G | - | L | A | E | A | L | T | A | G | R | E | E | V | Y | L | - | R | F | F | Y | R | T | F | - | - | - |
| 103 UniRef90\_UPI0012FB7881\_21\_292 | - | - | - | - | S | N | Q | V | A | - | D | M | T | W | H | F | L | F | N | R | L | - | T | D | - | V | N | E | R | L | V | E | G | R | E | E | I | F | F | - | G | H | Q | F | A | V | K | - | - | - |
| 104 UniRef90\_UPI00131B39C2\_13\_285 | - | - | - | - | - | - | - | - | - | - | P | L | L | W | H | F | R | F | G | G | - | - | P | D | - | - | M | E | R | L | V | A | G | R | E | R | I | Y | L | - | D | R | F | W | N | E | F | - | - | - |
| 105 UniRef90\_M2VKF7\_34\_311 | - | - | - | - | N | R | I | L | S | - | D | F | L | W | H | F | N | F | N | R | A | - | L | E | - | V | N | E | R | L | V | Q | G | R | E | D | V | Y | F | - | N | Y | Q | F | A | S | K | - | - | - |
| 106 UniRef90\_UPI0016124E74\_10\_283 | - | - | - | - | F | - | A | I | A | - | N | G | Y | W | H | W | L | F | H | L | Q | - | P | D | - | L | P | E | R | L | A | G | Q | D | V | S | G | Y | L | - | G | Y | F | F | E | R | W | - | - | - |
| 107 UniRef90\_A0A263DFR4\_27\_299 | - | - | - | - | D | - | A | A | N | - | D | R | L | F | H | F | G | F | N | R | L | - | G | R | - | L | N | E | E | L | V | R | G | R | E | A | L | Y | F | - | G | Y | Q | F | A | T | K | - | - | - |
| 108 UniRef90\_A0A0U5F3A6\_35\_313 | - | - | - | - | D | R | R | T | S | - | D | F | L | W | H | F | N | F | N | R | A | - | L | G | - | V | N | E | A | L | V | Q | G | R | E | A | I | Y | F | - | G | Y | Q | F | A | T | K | - | - | - |
| 109 UniRef90\_A0A2N3KZL1\_38\_324 | - | - | - | - | P | - | E | G | E | - | S | L | V | W | H | F | S | F | F | A | A | G | N | N | - | L | A | E | T | L | V | T | G | H | E | R | M | F | L | - | E | H | F | I | K | E | H | - | - | - |
| 110 UniRef90\_A0A1X7C0M0\_25\_283 | - | - | - | - | E | - | - | - | - | - | G | G | M | W | H | F | G | F | F | M | T | - | E | H | - | L | P | E | M | L | F | D | G | H | E | R | E | F | I | - | A | A | T | F | T | A | - | - | - | - |
| 111 UniRef90\_A0A078M0V2\_4\_284 | - | - | - | - | A | Q | E | A | A | - | K | K | Y | W | H | W | V | F | Q | V | V | - | P | D | - | L | P | E | T | L | I | S | G | K | E | E | E | Y | L | - | K | F | L | L | S | R | G | - | - | - |
| 112 UniRef90\_A0A0F4Z6H8\_20\_271 | - | - | - | - | P | N | R | P | G | - | P | H | L | W | Q | V | G | F | Y | A | V | - | P | D | - | V | P | E | L | L | L | R | G | R | E | P | E | Y | F | - | S | W | F | I | K | N | E | - | - | - |
| 113 UniRef90\_UPI00161D2319\_22\_292 | - | - | - | - | P | - | A | T | N | - | E | F | L | W | H | F | A | F | N | R | L | - | A | D | - | I | N | E | R | M | V | A | G | R | E | E | I | Y | F | - | G | Y | N | L | T | S | K | - | - | - |
| 114 UniRef90\_A0A1Q8LX67\_38\_327 | - | - | - | - | - | - | R | Y | R | - | T | F | S | W | H | I | A | F | H | T | K | - | R | V | - | V | P | E | A | V | V | A | D | D | V | R | E | Y | L | - | S | L | F | Y | A | Q | V | - | - | - |
| 115 UniRef90\_A0A1Q9S2P6\_18\_309 | - | - | - | - | - | - | T | Y | R | - | T | L | S | W | H | I | A | F | H | T | Q | - | P | R | - | V | P | E | A | V | V | G | D | D | V | R | E | Y | L | - | A | L | F | Y | P | Q | V | - | - | - |
| 116 UniRef90\_A0A5M7BQA6\_8\_281 | - | - | - | - | A | - | E | A | G | - | T | A | Y | W | H | W | L | F | H | L | Q | - | P | D | - | L | P | E | L | L | A | G | K | D | V | A | G | Y | L | - | G | Y | F | F | E | R | W | - | - | - |
| 117 UniRef90\_A0A3N6GJS2\_33\_314 | - | - | - | - | A | - | P | G | L | P | F | F | P | W | W | F | A | F | N | Q | V | - | V | G | - | L | P | E | Q | L | L | A | G | R | A | H | F | L | I | - | D | W | V | F | D | N | F | - | - | - |
| 118 UniRef90\_A0A0Q6MZD2\_36\_308 | - | - | - | - | - | - | - | - | - | - | P | L | L | W | H | F | R | F | G | G | - | - | P | D | - | - | M | E | R | L | V | A | G | R | E | R | I | Y | L | - | D | R | F | W | N | E | F | - | - | - |
| 119 UniRef90\_UPI00052520C0\_22\_293 | - | - | - | - | A | N | R | P | V | - | E | A | L | W | H | F | M | F | N | R | L | - | D | S | - | L | N | E | R | L | V | E | G | R | E | D | L | Y | F | - | G | E | Q | F | R | T | K | - | - | - |
| 120 UniRef90\_A0A1Q4XCF8\_2\_276 | - | - | - | - | A | - | P | G | L | P | F | H | P | W | W | F | A | F | N | S | V | - | A | G | - | L | P | E | Q | L | L | A | G | R | A | R | H | L | V | - | D | W | V | F | D | G | F | - | - | - |
| 121 UniRef90\_A0A328LZX6\_33\_305 | - | - | - | - | - | - | - | - | - | - | P | M | L | W | H | F | H | F | G | G | - | - | P | D | - | - | M | E | R | L | V | A | G | R | E | R | I | Y | L | - | D | R | F | W | N | D | F | - | - | - |
| 122 UniRef90\_A0A2V8S6L4\_40\_311 | - | - | - | - | - | - | - | - | - | - | R | D | L | W | H | F | H | F | Y | G | E | - | - | - | - | T | P | L | A | L | V | T | G | R | E | R | I | Y | L | - | E | H | F | W | N | D | F | - | - | - |
| 123 UniRef90\_A0A1I5EB88\_8\_281 | - | - | - | - | T | - | E | A | G | - | T | A | Y | W | H | W | F | F | H | L | Q | - | P | D | - | L | P | E | R | L | V | G | N | D | V | A | G | Y | L | - | G | Y | F | F | E | R | W | - | - | - |
| 124 UniRef90\_UPI0014147ABD\_39\_305 | - | - | - | - | G | - | P | L | N | - | D | R | L | W | H | L | P | F | N | R | V | - | D | R | - | L | P | E | Q | L | V | A | G | R | E | D | V | F | F | - | G | Y | E | F | A | V | Q | - | - | - |
| 125 UniRef90\_A0A1I7DL13\_6\_274 | - | - | - | - | - | - | - | - | - | - | T | L | F | W | H | L | K | F | H | Q | D | - | V | D | - | M | A | T | K | M | I | G | S | D | I | P | A | Y | L | - | N | H | F | Y | R | D | F | - | - | - |
| 126 UniRef90\_A0A3N7EDS3\_31\_305 | - | - | - | - | - | - | - | - | - | - | P | L | L | W | H | F | R | F | G | G | - | - | P | D | - | - | M | E | R | L | V | A | G | R | E | R | I | Y | L | - | D | R | F | W | N | E | F | - | - | - |
| 127 UniRef90\_UPI0016225F9B\_4\_275 | - | - | - | - | S | - | G | I | A | - | L | G | T | W | H | W | T | F | H | L | Q | - | P | D | - | L | P | E | R | L | A | G | A | D | V | R | G | Y | L | - | G | Y | F | F | E | K | W | - | - | - |
| 128 UniRef90\_UPI000997AE50\_107\_382 | - | - | - | - | - | - | D | F | S | - | N | V | S | F | H | F | L | F | N | Q | Q | - | S | P | - | L | A | E | Q | L | V | T | G | R | E | K | Q | F | F | - | G | Y | F | Y | P | T | F | - | - | - |
| 129 UniRef90\_A0A0N7F3H5\_6\_275 | - | - | - | - | A | - | D | I | A | - | R | A | Y | W | H | W | L | F | H | L | Q | - | P | D | - | L | P | E | L | L | A | G | Q | N | I | A | T | Y | L | - | G | Y | F | F | E | R | W | - | - | - |
| 130 UniRef90\_A0A3Q8F441\_11\_281 | - | - | - | - | A | - | S | I | A | - | K | A | Y | W | F | F | L | F | H | L | V | - | P | D | - | L | P | E | A | L | I | A | G | N | E | A | K | W | L | - | R | W | F | F | S | D | W | - | - | - |
| 131 UniRef90\_UPI001487D2FB\_23\_300 | - | - | - | - | - | - | - | - | - | - | P | L | L | W | H | F | S | F | G | G | - | - | P | D | - | - | A | E | R | L | V | A | G | R | E | R | I | Y | L | - | D | R | F | W | N | E | F | - | - | - |
| 132 UniRef90\_A0A437GNC7\_38\_307 | - | - | - | - | D | R | Q | L | S | - | D | F | L | W | H | N | N | F | C | R | V | - | R | G | - | I | N | E | A | L | V | V | G | R | E | E | I | F | F | - | D | Y | Q | F | - | T | K | - | - | - |
| 133 UniRef90\_A0A1H3SWJ7\_6\_283 | - | - | - | - | A | - | R | V | G | - | A | A | Y | W | H | W | L | F | H | L | V | - | P | D | - | L | P | E | F | L | T | E | G | K | G | G | E | Y | V | - | R | F | M | L | A | A | G | - | - | - |
| 134 UniRef90\_UPI0013DD6281\_49\_329 | - | - | - | - | - | - | K | Y | R | - | T | L | S | W | H | L | G | F | H | G | Q | - | D | D | - | F | P | E | A | I | V | A | D | D | V | R | E | Y | L | - | A | L | F | Y | A | Y | V | - | - | - |
| 135 UniRef90\_I0L7C1\_27\_272 | - | - | - | - | - | - | - | - | - | - | A | P | P | W | W | F | G | F | H | S | A | - | P | G | - | L | A | E | T | V | L | E | G | H | E | A | E | Y | L | - | D | W | F | L | T | A | G | - | - | - |
| 136 UniRef90\_A0A260DLM1\_45\_326 | - | - | - | - | - | - | Q | Y | R | - | S | L | S | W | H | I | A | F | H | S | Q | - | P | S | - | V | P | E | M | L | V | T | G | R | V | R | S | Y | L | - | A | D | F | Y | P | R | V | - | - | - |
| 137 UniRef90\_UPI0005BDD2EC\_7\_280 | - | - | - | - | L | - | L | L | A | - | S | S | H | W | H | W | L | F | H | L | Q | - | P | D | - | L | P | E | R | L | A | G | R | D | V | R | G | Y | L | - | E | Y | F | Y | E | R | W | - | - | - |
| 138 UniRef90\_A0A6G9YBC1\_27\_296 | - | - | - | - | A | - | D | I | A | - | R | R | Y | F | H | W | L | F | H | L | Q | - | P | D | - | L | P | E | L | L | V | G | Q | N | I | S | G | Y | L | - | R | Y | F | F | E | H | W | - | - | - |
| 139 UniRef90\_UPI00145FA88A\_8\_280 | - | - | - | - | T | - | E | V | G | - | A | A | Y | W | H | W | M | F | H | L | Q | - | P | D | - | L | P | E | L | L | A | G | K | D | V | A | G | Y | L | - | G | Y | F | F | E | R | W | - | - | - |
| 140 UniRef90\_A0A2W0B1K8\_46\_299 | - | - | - | - | - | - | - | - | - | - | P | G | V | W | H | F | N | F | H | G | - | - | P | D | - | - | A | E | R | L | V | A | G | R | E | R | I | Y | F | - | D | R | I | W | N | D | F | - | - | - |
| 141 UniRef90\_A0A5B8G622\_28\_299 | - | - | - | - | - | - | - | - | - | - | P | L | L | W | H | F | R | F | G | G | - | - | P | D | - | - | M | E | R | L | V | A | G | R | E | R | I | Y | L | - | D | R | F | W | N | E | F | - | - | - |
| 142 UniRef90\_A0A4S1XGQ8\_21\_302 | - | - | - | - | - | - | - | - | - | - | P | L | L | W | H | F | R | F | G | G | - | - | P | D | - | - | M | E | R | L | V | A | G | R | E | R | I | Y | L | - | D | R | F | W | N | E | F | - | - | - |
| 143 UniRef90\_A0A251YJ72\_8\_294 | - | - | - | - | H | - | - | - | - | - | G | G | S | W | H | I | G | A | L | A | A | - | P | G | - | I | P | E | L | L | L | A | G | R | E | R | E | L | L | G | S | W | A | F | P | T | M | - | - | - |
| 144 UniRef90\_UPI000A0527D2\_65\_318 | - | - | - | - | - | - | - | - | - | - | K | S | A | W | H | F | D | F | H | S | N | - | P | D | - | I | A | V | H | L | V | H | G | R | E | R | W | Y | I | - | Q | R | F | Y | D | D | L | - | - | - |
| 145 UniRef90\_A0A4V2S7H9\_8\_280 | - | - | - | - | A | - | P | V | A | - | R | G | Y | W | H | W | L | F | H | L | Q | - | P | D | - | L | P | E | L | L | A | G | Q | N | V | A | G | Y | L | - | G | Y | F | F | E | R | W | - | - | - |
| 146 UniRef90\_UPI00131B216A\_18\_247 | - | - | - | - | R | - | P | G | R | - | P | L | L | W | W | F | A | F | N | Q | L | - | R | G | - | L | P | E | Q | L | I | T | G | R | S | R | Y | L | I | - | D | F | V | H | D | E | S | - | - | - |
| 147 UniRef90\_A0A1G8T9Y6\_5\_243 | - | - | - | - | - | - | A | Q | G | - | P | G | A | W | H | F | G | F | F | S | L | - | T | N | G | L | P | E | Q | L | I | D | G | N | E | E | V | W | V | - | D | R | F | T | D | S | L | - | - | - |
| 148 UniRef90\_UPI00161F613F\_5\_254 | - | - | - | - | H | - | - | - | - | - | G | G | A | W | H | I | G | V | L | A | A | - | P | G | - | V | P | E | M | L | L | A | G | H | E | R | E | F | L | - | E | Y | L | L | - | - | - | - | - | - |
| 149 UniRef90\_UPI00142071FA\_80\_362 | - | - | - | - | E | - | F | L | N | - | N | R | L | W | H | I | P | F | N | R | V | - | D | D | E | L | I | V | D | M | V | R | S | N | A | D | A | F | Y | - | R | Y | E | F | A | I | Q | - | - | - |
| 150 UniRef90\_A0A6G7Q8F6\_7\_219 | - | - | - | - | - | - | G | Q | A | - | L | R | S | A | Y | A | G | F | F | Q | L | - | P | L | - | L | P | E | R | L | L | S | A | R | R | G | H | L | L | - | R | A | L | M | R | G | - | - | - | - |

  
  

|  |  |  |  |  |  |  |  |  |  |  |  |  |  |  |  |  |  |  |  |  |  |  |  |  |  |  |  |  |  |  |  |  |  |  |  |  |  |  |  |  |  |  |  |  |  |  |  |  |  |  |
| --- | --- | --- | --- | --- | --- | --- | --- | --- | --- | --- | --- | --- | --- | --- | --- | --- | --- | --- | --- | --- | --- | --- | --- | --- | --- | --- | --- | --- | --- | --- | --- | --- | --- | --- | --- | --- | --- | --- | --- | --- | --- | --- | --- | --- | --- | --- | --- | --- | --- | --- |
| **001 Input\_protein\_seq** | A | A | N | P | - | - | - | - | - | - | - | - | - | E | S | F | - | S | D | A | D | V | D | E | Y | L | R | V | F | - | T | R | D | G | G | L | R | A | G | L | A | F | Y | R | A | V | - | - | S | E |
| 002 UniRef90\_UPI001430CB85\_4\_307 | T | A | N | P | - | - | - | - | - | - | - | - | - | Q | T | F | - | S | D | A | D | M | D | E | Y | V | R | V | F | - | T | R | E | G | G | L | R | A | G | L | A | Y | Y | R | A | V | - | - | A | E |
| 003 UniRef90\_UPI00055F8D66\_10\_306 | A | A | N | P | - | - | - | - | - | - | - | - | - | E | A | F | - | S | E | A | D | I | E | E | Y | L | R | V | F | - | K | K | P | G | G | L | R | A | G | L | A | Y | Y | R | D | A | - | - | S | L |
| 004 UniRef90\_UPI00161D8BA7\_15\_312 | T | A | N | P | - | - | - | - | - | - | - | - | - | A | T | F | - | S | D | G | D | I | E | E | Y | L | R | I | F | - | K | K | D | G | G | L | R | A | G | L | A | Y | Y | R | S | A | - | - | A | Q |
| 005 UniRef90\_A0A4R7GLU7\_7\_305 | T | A | A | P | - | - | - | - | - | - | - | - | - | D | A | F | - | S | E | D | D | I | S | E | Y | L | R | V | F | - | L | K | S | G | G | L | R | A | G | L | A | Y | Y | R | S | V | - | - | T | K |
| 006 UniRef90\_UPI00041CD164\_24\_309 | A | A | D | P | - | - | - | - | - | - | - | - | - | E | V | F | - | T | S | A | D | I | D | E | Y | V | R | V | L | - | K | K | D | G | G | L | R | A | G | L | A | Y | Y | R | A | V | - | - | E | Q |
| 007 UniRef90\_I0QSF8\_15\_298 | T | A | A | P | - | - | - | - | - | - | - | - | - | D | A | F | - | T | E | E | D | I | N | E | Y | L | R | I | F | - | T | Q | S | G | A | V | R | A | G | M | A | Y | Y | R | A | L | - | - | G | L |
| 008 UniRef90\_A0A2N5ENH7\_9\_301 | T | A | S | P | - | - | - | - | - | - | - | - | - | M | A | F | - | S | D | A | D | M | A | E | Y | V | R | L | L | - | K | Q | N | G | A | L | R | A | G | L | A | P | Y | R | D | V | - | - | S | V |
| 009 UniRef90\_A0A0Q8E2M6\_29\_312 | A | A | D | P | - | - | - | - | - | - | - | - | - | S | V | F | - | S | E | Q | D | L | D | E | Y | L | R | V | F | - | T | L | P | G | G | L | R | S | G | L | A | Y | Y | R | A | T | - | - | P | E |
| 010 UniRef90\_A0A0F5XY46\_6\_287 | A | A | S | P | - | - | - | - | - | - | - | - | - | H | R | I | - | D | A | A | A | R | A | E | Y | L | R | L | Y | - | Q | Q | P | G | A | L | R | A | G | L | A | Y | Y | R | A | V | - | - | E | Q |
| 011 UniRef90\_A0A3N4NUX5\_4\_284 | T | A | N | P | - | - | - | - | - | - | - | - | - | L | T | F | - | D | A | T | K | M | Q | E | Y | L | R | I | F | - | T | R | P | G | A | L | R | A | G | L | A | C | Y | R | A | V | - | - | T | Q |
| 012 UniRef90\_UPI000DDED2D9\_11\_293 | A | A | S | P | - | - | - | - | - | - | - | - | - | A | Y | F | - | S | E | D | D | F | A | E | Y | L | R | V | F | - | R | I | - | N | G | L | K | G | G | L | S | Y | Y | R | A | V | - | - | S | Q |
| 013 UniRef90\_UPI000DDED53B\_10\_292 | T | A | N | P | - | - | - | - | - | - | - | - | - | N | C | V | - | G | E | E | E | I | D | E | Y | L | R | I | F | - | L | A | P | G | G | I | R | A | G | L | S | F | Y | R | S | L | - | - | A | L |
| 014 UniRef90\_A0A1G4JKM0\_6\_288 | A | A | D | P | - | - | - | - | - | - | - | - | - | T | V | F | - | S | E | T | D | L | E | E | Y | E | R | I | L | - | T | Q | P | G | A | L | R | A | G | L | A | Y | Y | R | A | C | - | - | D | K |
| 015 UniRef90\_UPI0016728815\_21\_305 | A | R | S | V | - | - | - | - | - | - | - | - | - | G | T | F | - | D | D | T | E | I | E | Q | Y | A | A | S | L | - | A | A | D | G | G | L | R | A | S | L | A | Y | Y | R | D | A | - | - | A | E |
| 016 UniRef90\_A0A3N2H8D2\_14\_297 | A | L | S | P | - | - | - | - | - | - | - | - | - | S | T | F | - | D | D | T | E | L | D | H | Y | A | A | A | V | - | A | A | D | G | G | L | R | A | S | L | A | Y | Y | R | D | A | - | - | A | E |
| 017 UniRef90\_UPI0005644DD1\_9\_307 | A | L | S | P | - | - | - | - | - | - | - | - | - | D | T | F | - | D | G | A | D | L | D | H | Y | A | A | A | L | - | A | A | D | G | G | L | H | A | S | L | A | Y | Y | R | D | A | - | - | A | E |
| 018 UniRef90\_A0A6B2VDS0\_18\_308 | A | L | S | P | - | - | - | - | - | - | - | - | - | D | T | F | - | D | D | T | E | L | D | H | Y | A | A | A | V | - | A | A | D | G | G | L | R | A | S | L | A | Y | Y | R | D | A | - | - | G | E |
| 019 UniRef90\_A0A6G3RZZ1\_25\_307 | T | L | L | P | - | - | - | - | - | - | - | - | - | D | T | F | - | D | D | A | E | V | D | H | Y | A | A | A | L | - | A | A | E | G | G | L | R | A | S | L | A | Y | Y | R | D | A | - | - | T | E |
| 020 UniRef90\_A0A209C7X3\_21\_305 | T | L | S | S | - | - | - | - | - | - | - | - | - | D | T | F | - | D | D | A | E | I | E | R | Y | A | A | A | V | - | A | A | D | G | G | L | R | A | S | L | A | Y | Y | R | D | A | - | - | A | E |
| 021 UniRef90\_UPI001269E708\_26\_309 | A | H | S | P | - | - | - | - | - | - | - | - | - | D | T | F | - | D | A | A | E | I | D | H | Y | T | T | A | V | - | A | A | G | S | G | L | R | A | F | L | A | Y | Y | R | D | A | - | - | A | E |
| 022 UniRef90\_A0A2M9J7W9\_25\_306 | A | L | S | P | - | - | - | - | - | - | - | - | - | D | T | F | - | D | D | A | E | I | G | H | Y | A | A | A | V | - | A | A | E | G | G | L | T | A | S | L | A | Y | Y | R | D | A | - | - | A | E |
| 023 UniRef90\_A0A6G9F3J8\_23\_307 | A | L | S | P | - | - | - | - | - | - | - | - | - | A | A | F | - | D | D | A | E | I | E | Q | Y | A | A | A | L | - | A | A | D | G | G | L | R | A | S | L | A | Y | Y | R | D | A | - | - | A | A |
| 024 UniRef90\_A0A1H2UZ41\_14\_292 | T | Y | N | P | A | - | - | - | - | - | - | - | - | G | T | F | - | S | Q | A | D | I | D | E | Y | E | R | V | Y | - | K | Q | P | G | N | L | R | G | A | L | A | Y | Y | R | A | V | - | - | L | E |
| 025 UniRef90\_UPI0012B05B7D\_16\_286 | A | L | N | Y | R | - | - | - | - | - | - | - | - | D | T | F | - | T | E | L | D | L | D | E | Y | T | R | V | Y | - | S | A | L | G | G | M | R | G | M | L | G | Y | Y | R | S | V | - | - | L | E |
| 026 UniRef90\_A0A5C8T429\_18\_294 | A | S | N | P | - | - | - | - | - | - | - | - | - | A | A | I | - | S | Q | Q | A | M | D | E | Y | V | R | C | Y | - | E | A | P | G | A | W | R | C | A | N | S | Y | Y | R | A | Y | - | - | F | D |
| 027 UniRef90\_UPI0012E86E3E\_10\_309 | A | V | R | P | - | - | - | - | - | - | - | - | - | W | T | I | - | T | P | A | D | L | D | E | Y | V | R | V | N | - | A | A | P | G | A | T | R | A | A | L | S | Y | Y | R | H | K | L | G | P | E |
| 028 UniRef90\_A0A1A9HTL2\_13\_300 | S | V | K | R | - | - | - | - | - | - | - | - | - | E | V | F | - | T | T | E | A | L | D | E | Y | T | R | V | F | - | A | Q | P | G | G | A | R | A | A | F | N | Y | Y | R | A | A | F | S | D | T |
| 029 UniRef90\_A0A0R1V0C7\_7\_281 | A | R | N | W | R | - | - | - | - | - | - | - | - | T | A | F | - | T | K | E | D | I | A | E | Y | V | Q | E | Y | - | R | Q | L | G | V | M | R | G | M | L | G | Y | Y | R | A | V | - | - | L | D |
| 030 UniRef90\_A0A3N1M9Y1\_5\_282 | S | W | Q | Q | - | - | - | - | - | - | - | - | - | G | A | I | - | T | D | A | D | I | D | E | Y | V | R | T | Y | - | S | K | P | G | A | L | R | A | G | F | A | Y | Y | R | N | I | - | - | P | Q |
| 031 UniRef90\_A0A158JUX7\_17\_294 | L | V | D | P | - | - | - | - | - | - | - | - | - | T | A | V | - | S | D | A | D | L | E | I | Y | A | D | A | W | - | R | Q | P | G | A | L | R | A | S | F | A | Y | Y | Q | Q | L | L | - | G | Q |
| 032 UniRef90\_A0A0U3LIL8\_12\_296 | A | A | R | P | - | - | - | - | - | - | - | - | - | W | V | F | - | T | P | A | V | L | D | E | Y | T | R | I | Y | - | T | Q | P | G | G | T | R | T | A | F | A | Y | Y | R | E | S | F | S | R | Q |
| 033 UniRef90\_A0A248JS33\_7\_301 | A | L | R | R | - | - | - | - | - | - | - | - | - | W | A | I | - | G | P | A | D | L | D | E | Y | V | R | V | L | - | S | L | P | G | A | L | R | S | T | F | A | Y | Y | R | E | V | F | R | P | A |
| 034 UniRef90\_A0A1Y0REZ4\_12\_287 | A | Y | N | P | - | - | - | - | - | - | - | - | - | D | A | I | - | A | K | A | D | I | E | E | Y | V | R | C | Y | - | S | A | P | G | G | L | R | G | G | F | E | H | Y | R | A | F | - | - | F | E |
| 035 UniRef90\_A0A0M2WRU5\_3\_280 | S | Y | R | R | - | - | - | - | - | - | - | - | - | D | K | V | - | - | - | - | A | V | D | V | Y | A | D | A | Y | - | A | T | P | G | A | L | R | A | G | F | A | Y | Y | R | A | I | - | - | P | E |
| 036 UniRef90\_A0A0N8GFM1\_6\_279 | G | H | R | P | - | - | - | - | - | - | - | - | - | N | A | I | - | P | P | A | D | Q | E | E | Y | F | R | S | Y | - | R | K | F | G | T | F | R | A | M | L | E | F | Y | R | A | L | - | - | P | T |
| 037 UniRef90\_A0A252EMP1\_7\_277 | A | F | D | P | - | - | - | - | - | - | - | - | - | R | S | I | - | T | E | A | D | I | T | E | Y | V | R | C | Y | - | S | G | L | G | G | M | R | A | G | F | N | Y | Y | R | A | F | - | - | P | E |
| 038 UniRef90\_W0A6K5\_40\_320 | S | Y | R | R | - | - | - | - | - | - | - | - | - | D | R | V | - | - | - | - | A | A | Q | T | Y | I | D | A | Y | - | S | S | P | G | G | L | R | A | G | F | A | Y | Y | R | A | I | - | - | P | Q |
| 039 UniRef90\_A0A3S1SM66\_23\_304 | S | V | R | P | - | - | - | - | - | - | - | - | - | W | A | I | - | T | P | E | D | I | D | E | Y | A | H | Q | M | - | A | V | P | G | A | V | R | A | A | T | A | Y | Y | K | A | A | F | S | A | E |
| 040 UniRef90\_A0A2E5L5C6\_7\_286 | A | Y | K | P | - | - | - | - | - | - | - | - | - | D | S | I | - | T | N | E | D | L | K | E | Y | L | R | T | Y | - | V | Q | P | G | A | M | R | A | G | F | S | Y | Y | R | A | I | - | - | P | Q |
| 041 UniRef90\_M5D1B4\_14\_289 | S | Y | R | R | - | - | - | - | - | - | - | - | - | D | A | V | - | - | - | - | A | A | D | T | Y | I | A | A | Y | - | S | R | P | G | A | L | R | A | G | F | A | W | Y | R | A | I | - | - | P | E |
| 042 UniRef90\_UPI0016845BA7\_31\_303 | A | Y | D | P | - | - | - | - | - | - | - | - | - | T | A | I | - | T | E | A | D | I | N | E | Y | L | R | T | Y | - | S | N | P | D | A | L | H | A | G | F | E | Y | Y | R | A | F | - | - | P | E |
| 043 UniRef90\_UPI0014648199\_14\_291 | S | F | H | R | - | - | - | - | - | - | - | - | - | D | A | V | - | - | - | - | A | S | D | V | Y | I | R | A | Y | - | S | T | P | G | G | L | R | A | G | F | D | Y | Y | R | A | I | - | - | P | E |
| 044 UniRef90\_K9RUM7\_12\_289 | S | K | R | K | - | - | - | - | - | - | - | - | - | Q | N | L | - | T | P | E | V | M | D | Y | Y | A | K | Y | Y | - | S | Q | P | G | A | M | K | A | G | F | L | W | Y | A | A | L | - | - | E | E |
| 045 UniRef90\_A0A1Q2U086\_13\_290 | S | K | R | K | - | - | - | - | - | - | - | - | - | D | N | L | - | T | P | E | V | M | D | Y | Y | A | R | Y | Y | - | S | R | P | G | A | M | K | A | G | F | L | W | Y | S | A | L | - | - | E | E |
| 046 UniRef90\_B9JMN1\_3\_282 | A | Y | D | P | - | - | - | - | - | - | - | - | - | S | A | I | - | T | S | T | E | T | E | E | Y | V | Q | A | M | - | T | N | V | G | A | L | R | A | G | L | Q | Y | Y | Q | S | Y | - | - | F | T |
| 047 UniRef90\_A0A2X1TAZ7\_33\_319 | A | Y | R | R | - | - | - | - | - | - | - | - | - | D | R | V | - | - | - | - | A | A | Q | T | Y | I | A | A | Y | - | S | S | P | G | G | L | R | A | G | F | A | Y | Y | R | A | I | - | - | P | Q |
| 048 UniRef90\_UPI00131A8152\_6\_279 | G | A | R | E | - | - | - | - | - | - | - | - | - | D | A | I | - | D | S | E | E | I | E | E | Y | L | R | S | Y | - | R | R | A | S | V | L | H | A | G | F | E | Y | Y | R | A | V | - | - | G | Q |
| 049 UniRef90\_F7YB04\_47\_316 | G | V | N | T | - | - | - | - | - | - | - | - | - | R | A | F | - | D | T | E | A | D | E | V | Y | G | R | S | Y | - | A | Q | P | G | A | L | R | A | G | F | E | Y | Y | R | A | F | - | - | P | R |
| 050 UniRef90\_A0A1A3M1Z8\_5\_294 | S | A | N | P | - | - | - | - | - | - | - | - | - | D | A | I | - | S | L | A | D | A | Q | R | Y | V | A | D | Y | - | T | A | P | G | A | M | R | A | G | I | E | L | F | R | A | F | - | - | E | K |
| 051 UniRef90\_UPI0004C32CE1\_32\_312 | A | F | N | P | - | - | - | - | - | - | - | - | - | D | A | I | - | D | E | R | S | R | Q | I | Y | A | R | A | Y | - | N | S | P | D | A | I | R | A | S | N | G | W | Y | Q | A | F | - | - | H | Q |
| 052 UniRef90\_A0A0J6NQJ9\_12\_287 | S | W | R | R | - | - | - | - | - | - | - | - | - | D | R | V | - | - | - | - | A | A | E | V | Y | I | D | A | Y | - | S | S | P | G | G | L | R | G | G | F | A | Y | Y | R | A | I | - | - | P | E |
| 053 UniRef90\_A0A534ESC2\_32\_321 | S | T | R | T | - | - | - | - | - | - | - | - | - | Y | A | I | - | E | P | A | A | L | E | E | Y | V | R | V | Y | - | S | L | P | G | A | M | R | A | G | F | A | W | Y | R | V | N | F | S | A | E |
| 054 UniRef90\_A0A1H1Z302\_21\_301 | A | A | T | P | - | - | - | - | - | - | - | - | - | D | T | I | - | P | A | Y | A | V | D | V | Y | V | D | A | I | V | A | D | P | R | G | L | G | A | S | F | E | Y | Y | R | A | L | - | - | D | E |
| 055 UniRef90\_A0A158DLE6\_24\_295 | T | F | D | P | - | - | - | - | - | - | - | - | - | S | S | I | - | T | E | E | D | I | N | V | Y | A | T | A | L | - | T | Q | P | G | A | L | R | A | S | F | S | Y | Y | R | T | L | - | - | F | V |
| 056 UniRef90\_A0A163VVR8\_16\_280 | S | Y | R | R | - | - | - | - | - | - | - | - | - | D | R | V | - | - | - | - | A | A | Q | V | Y | I | A | A | Y | - | S | A | P | G | G | L | R | A | G | F | D | Y | Y | R | A | I | - | - | P | E |
| 057 UniRef90\_UPI000369F0DF\_7\_289 | S | H | R | R | - | - | - | - | - | - | - | - | - | D | R | V | - | - | - | - | A | A | D | V | Y | A | A | A | Y | - | G | T | P | G | G | L | R | A | G | F | A | Y | Y | R | A | I | - | - | P | E |
| 058 UniRef90\_UPI00098F4FB4\_43\_309 | G | V | N | T | - | - | - | - | - | - | - | - | - | R | A | F | - | D | N | E | A | D | E | I | Y | G | R | S | Y | - | A | Q | P | G | A | L | R | A | G | F | E | Y | Y | R | A | F | - | - | P | K |
| 059 UniRef90\_UPI0015A1AD76\_10\_292 | S | C | N | P | - | - | - | - | - | - | - | - | - | D | A | I | - | S | Q | K | D | F | E | L | Y | A | R | Q | L | - | S | K | P | G | A | F | R | A | G | I | N | L | Y | A | A | V | - | - | W | T |
| 060 UniRef90\_UPI001689F051\_63\_318 | S | V | G | L | Y | - | - | - | - | - | - | V | T | D | A | I | - | G | E | D | D | L | D | V | Y | A | R | A | L | - | A | G | P | G | A | V | R | A | T | M | G | Y | Y | R | T | L | - | - | P | A |
| 061 UniRef90\_UPI000DD77D66\_43\_313 | G | V | N | S | - | - | - | - | - | - | - | - | - | R | A | F | - | D | N | Q | A | D | E | I | Y | G | R | S | Y | - | S | Q | P | G | A | L | R | A | G | F | E | Y | Y | R | A | F | - | - | P | Q |
| 062 UniRef90\_A0A3M0I8T4\_31\_307 | A | A | R | P | - | - | - | - | - | - | - | - | - | - | - | L | - | P | E | S | A | V | R | Y | Y | I | D | T | L | A | S | D | P | D | A | L | R | A | S | F | D | F | Y | R | A | L | - | - | D | E |
| 063 UniRef90\_UPI000377E2D1\_4\_271 | G | H | H | Q | - | - | - | - | - | - | - | - | - | K | V | L | - | P | D | D | A | V | A | E | Y | L | R | C | Y | - | T | A | A | G | A | L | R | A | G | F | E | L | Y | R | T | V | - | - | P | L |
| 064 UniRef90\_UPI0016198B53\_15\_286 | A | A | R | P | - | - | - | - | - | - | - | - | - | - | - | L | - | P | A | E | A | V | A | F | Y | V | E | S | I | A | R | D | P | E | A | L | R | A | S | F | D | Y | Y | R | A | T | - | - | D | D |
| 065 UniRef90\_UPI0013C0217D\_48\_325 | L | P | D | Q | - | - | - | - | - | - | - | - | - | S | L | V | - | G | D | F | D | R | R | V | Y | T | R | A | Y | - | D | S | A | E | A | V | R | A | S | N | G | W | Y | R | S | F | - | - | H | Q |
| 066 UniRef90\_C7PGN2\_5\_260 | S | F | K | K | - | - | - | - | - | - | - | - | - | E | V | F | - | D | N | D | T | I | N | E | Y | Y | R | T | Y | - | S | Q | A | D | K | M | S | N | G | F | N | Y | Y | R | T | I | - | - | L | Q |
| 067 UniRef90\_A0A1F7Q7Q2\_7\_275 | A | W | N | P | - | - | - | - | - | - | - | - | - | A | A | I | - | E | E | E | A | I | Q | E | Y | L | R | C | F | - | R | Q | P | G | A | M | R | A | G | F | E | D | Y | R | A | A | A | - | S | L |
| 068 UniRef90\_A0A2V7F1K9\_9\_274 | A | Y | N | P | - | - | - | - | - | - | - | - | - | A | A | I | - | E | E | E | A | V | Q | E | Y | L | R | C | F | - | R | Q | P | G | A | M | R | A | G | F | E | D | Y | R | A | A | A | - | T | L |
| 069 UniRef90\_UPI001616C592\_31\_308 | A | A | T | P | - | - | - | - | - | - | - | - | - | T | A | V | - | P | A | A | V | V | D | V | Y | V | R | A | L | - | R | I | P | G | A | L | R | A | S | F | E | F | Y | R | A | I | - | - | D | E |
| 070 UniRef90\_UPI001456849A\_71\_360 | T | Y | N | S | - | - | - | - | - | - | - | - | - | R | G | F | - | D | E | A | D | I | Q | E | F | M | R | T | Y | - | A | A | A | G | A | M | R | A | G | F | N | Y | Y | R | T | F | - | - | E | Q |
| 071 UniRef90\_A0A2S8J6S6\_18\_282 | A | T | N | H | - | - | - | - | - | - | - | - | - | G | A | F | - | T | D | R | D | V | E | L | Y | A | R | A | F | - | A | Q | P | G | A | I | R | S | T | L | E | W | Y | R | A | F | - | - | E | R |
| 072 UniRef90\_A0A2I2GC12\_18\_280 | T | A | H | P | - | - | - | - | - | - | - | - | - | E | T | F | - | P | K | E | R | L | E | S | Y | I | Q | S | Y | - | A | R | P | G | A | M | S | R | G | F | E | Y | Y | R | A | V | - | - | G | T |
| 073 UniRef90\_A0A549T469\_41\_312 | S | F | R | E | - | - | - | - | - | - | - | - | - | D | A | V | - | S | A | A | A | I | D | E | F | A | R | H | Y | - | A | T | P | G | G | M | T | A | G | F | E | Y | Y | R | A | L | - | - | P | E |
| 074 UniRef90\_A0A5C4LJC6\_5\_278 | F | H | D | H | - | - | - | - | - | - | - | - | - | G | T | F | - | D | P | A | D | V | E | I | Y | A | Q | A | M | - | A | R | P | G | R | T | R | G | G | M | E | W | Y | R | T | L | - | - | A | A |
| 075 UniRef90\_UPI001619D806\_9\_283 | T | Y | R | R | - | - | - | - | - | - | - | - | - | D | V | F | - | T | S | E | V | V | D | E | Y | V | R | A | F | - | S | A | P | G | A | L | R | A | G | F | D | D | Y | R | A | S | F | - | P | D |
| 076 UniRef90\_A0A239P1A5\_69\_347 | G | T | T | P | - | - | - | - | - | - | - | - | - | T | S | M | - | P | A | T | A | V | Q | V | Y | V | D | A | L | T | S | S | P | Q | A | L | R | S | S | F | E | F | Y | R | A | I | - | - | D | E |
| 077 UniRef90\_UPI0005262A70\_5\_280 | N | G | T | P | - | - | - | - | - | - | - | - | - | G | A | I | - | T | D | R | D | V | D | E | F | T | R | V | L | - | A | R | P | G | G | L | R | A | P | A | A | F | Y | A | S | M | - | - | L | R |
| 078 UniRef90\_A0A241XUQ6\_27\_314 | A | S | N | T | - | - | - | - | - | - | - | - | - | E | V | F | - | S | E | R | L | L | D | L | Y | A | R | S | Y | - | A | K | P | H | S | L | N | A | S | F | E | Y | Y | R | A | L | - | - | N | E |
| 079 UniRef90\_A0A160FQ65\_32\_312 | A | G | S | P | - | - | - | - | - | - | - | - | - | N | A | V | - | P | A | Y | A | Q | S | F | Y | I | E | Q | L | K | R | D | S | Q | A | L | R | A | S | F | D | Y | Y | R | A | I | - | - | D | E |
| 080 UniRef90\_UPI0006AE4C23\_4\_281 | A | V | D | P | - | - | - | - | - | - | - | - | - | E | A | I | - | D | R | E | S | R | A | I | Y | A | R | A | Y | - | D | S | P | E | A | I | R | A | V | A | G | W | Y | R | T | L | - | - | S | Q |
| 081 UniRef90\_T0H3T7\_11\_281 | A | Y | D | P | - | - | - | - | - | - | - | - | - | S | S | I | - | S | G | E | A | F | D | T | Y | V | R | A | Y | - | R | A | P | G | A | V | R | G | A | M | A | D | Y | R | A | N | - | - | A | E |
| 082 UniRef90\_A0A4R2IH15\_5\_277 | A | T | Q | P | - | - | - | - | - | - | - | - | - | - | - | L | - | P | E | Q | A | I | Q | V | Y | V | D | A | L | - | K | D | P | D | A | L | R | A | S | F | E | F | Y | R | A | L | - | - | D | T |
| 083 UniRef90\_UPI00140B863E\_4\_276 | A | A | G | - | - | - | - | - | - | - | - | - | - | H | P | L | - | P | P | D | V | V | E | E | Y | R | R | A | Y | - | M | R | P | G | S | L | R | V | A | L | A | Y | Y | R | T | A | - | - | I | D |
| 084 UniRef90\_A0A3R9UXB7\_22\_297 | A | A | D | P | - | - | - | - | - | - | - | - | - | D | R | I | - | D | A | R | S | R | A | V | Y | A | R | A | Y | - | D | S | A | E | A | V | R | A | G | N | D | W | Y | R | A | F | - | - | P | Q |
| 085 UniRef90\_UPI000F8D1465\_20\_303 | G | A | T | P | - | - | - | - | - | - | - | - | - | D | A | I | - | P | A | E | A | A | E | I | Y | I | A | P | I | K | A | S | R | Q | A | L | K | A | C | F | E | F | Y | R | A | I | - | - | E | I |
| 086 UniRef90\_A0A6J4HYT7\_23\_307 | S | A | D | E | - | - | - | - | - | - | - | - | - | S | S | L | - | S | P | F | D | R | E | V | Y | A | A | A | Y | - | D | R | A | E | A | I | R | A | G | N | A | W | Y | Q | A | F | - | - | P | Q |
| 087 UniRef90\_A0A1D8SMR3\_19\_296 | A | A | S | P | - | - | - | - | - | - | - | - | - | E | A | I | - | P | Q | T | A | V | D | V | Y | V | D | A | L | - | R | D | P | A | A | L | H | A | S | F | E | F | Y | R | A | G | - | - | E | S |
| 088 UniRef90\_A0A4V2HUZ4\_48\_320 | S | H | N | K | - | - | - | - | - | - | - | - | - | M | A | I | - | S | E | D | A | K | S | E | F | A | Y | H | Y | - | A | R | P | G | G | M | T | A | G | F | N | Y | Y | R | A | L | - | - | R | Q |
| 089 UniRef90\_UPI0010582A1B\_5\_279 | F | Y | D | H | - | - | - | - | - | - | - | - | - | S | A | F | - | N | P | A | D | I | E | I | Y | A | K | A | L | - | A | L | P | G | R | T | R | G | G | M | E | W | Y | R | T | L | - | - | T | A |
| 090 UniRef90\_UPI00166CE541\_54\_338 | E | V | K | K | - | - | - | - | - | - | - | - | - | N | G | V | - | S | D | E | Q | V | R | E | Y | A | R | H | L | - | R | D | D | A | H | L | R | A | S | F | E | W | F | R | A | L | - | - | P | K |
| 091 UniRef90\_A0A5C1HX54\_34\_307 | G | Y | V | K | - | - | - | - | - | - | - | - | - | E | P | F | - | T | K | A | E | T | D | E | F | I | R | A | Y | - | S | V | P | G | A | T | T | G | A | F | N | W | F | A | A | F | - | - | N | Q |
| 092 UniRef90\_A0A4D4KET2\_34\_306 | A | A | K | A | - | - | - | - | - | - | - | - | - | - | - | L | - | P | D | H | A | V | R | H | Y | V | D | T | L | A | A | D | P | E | A | L | R | S | S | F | A | F | Y | R | A | L | - | - | D | T |
| 093 UniRef90\_A0A1I1XP17\_9\_276 | A | F | D | P | - | - | - | - | - | - | - | - | - | S | A | I | - | A | P | A | D | F | E | C | Y | V | E | A | Y | - | A | A | A | G | A | M | R | A | A | F | E | L | Y | R | A | F | - | - | D | E |
| 094 UniRef90\_A0A1D7VX60\_23\_299 | A | V | R | K | - | - | - | - | - | - | - | - | - | - | - | L | - | P | D | Y | A | V | D | Y | Y | V | R | I | L | A | S | Q | H | D | A | L | R | G | S | F | G | W | Y | R | A | L | - | - | D | T |
| 095 UniRef90\_A0A2T4ZAU7\_6\_296 | P | H | L | L | - | - | - | - | - | - | - | - | - | E | R | L | K | A | D | G | A | W | D | E | Y | V | R | I | W | - | K | Q | P | G | S | Y | Q | A | A | L | N | D | Y | R | A | T | H | - | E | I |
| 096 UniRef90\_UPI00143A9489\_23\_300 | S | A | D | P | - | - | - | - | - | - | - | - | - | A | R | F | - | D | E | A | S | R | E | H | Y | A | R | L | Y | - | A | Q | P | G | A | M | R | A | G | F | A | Q | F | L | A | F | - | - | D | Q |
| 097 UniRef90\_A0A7C7NVI2\_6\_287 | T | V | N | K | - | - | - | - | - | - | - | - | - | Y | P | P | - | T | N | E | E | I | E | E | Y | V | K | A | Y | - | S | Q | P | G | A | L | R | G | G | F | S | Y | Y | R | A | A | A | - | Y | E |
| 098 UniRef90\_UPI001661A3FF\_52\_332 | E | G | V | P | - | - | - | - | - | - | - | - | - | G | A | V | - | S | Q | D | D | I | R | V | F | A | H | Y | L | - | R | D | P | A | H | L | A | A | S | L | A | W | F | R | V | F | - | - | P | Q |
| 099 UniRef90\_A0A6G4V4F1\_2\_276 | A | R | H | P | - | - | - | - | - | - | - | - | - | E | R | I | - | S | E | E | S | R | A | I | Y | A | R | A | Y | - | D | T | P | E | A | L | T | A | G | F | G | V | F | H | T | F | - | - | H | Q |
| 100 UniRef90\_A0A260W032\_16\_294 | S | Y | N | P | - | - | - | - | - | - | - | - | - | E | M | L | - | T | P | A | E | I | D | V | Y | V | R | A | Y | - | Q | Q | P | G | T | V | R | G | S | C | A | D | Y | R | A | A | - | - | A | E |
| 101 UniRef90\_UPI00036BBA85\_7\_295 | S | Y | N | R | - | - | - | - | - | - | - | - | - | E | L | F | - | S | D | E | E | V | A | E | Y | V | K | A | Y | - | S | R | P | G | A | L | R | G | S | F | N | D | Y | R | A | A | - | - | P | V |
| 102 UniRef90\_A0A4R5QBW7\_9\_278 | G | G | R | P | - | - | - | - | - | - | - | - | - | D | C | V | - | P | E | A | A | Q | R | D | Y | I | R | A | Y | - | S | Q | P | G | A | M | H | A | G | F | E | Y | Y | R | A | M | - | - | R | Q |
| 103 UniRef90\_UPI0012FB7881\_21\_292 | G | P | - | - | - | - | - | - | - | - | - | - | - | - | - | L | - | P | D | E | T | V | A | V | Y | V | D | A | L | - | R | R | P | G | A | L | H | A | S | F | Q | S | Y | R | A | L | - | - | D | V |
| 104 UniRef90\_UPI00131B39C2\_13\_285 | S | A | D | P | - | - | - | - | - | - | - | - | - | K | S | F | - | G | E | A | S | R | D | H | Y | A | R | L | Y | - | A | Q | P | G | A | M | H | A | G | F | C | Q | F | A | A | F | - | - | D | Q |
| 105 UniRef90\_M2VKF7\_34\_311 | A | G | S | P | - | - | - | - | - | - | - | - | - | D | G | V | - | P | K | Y | A | R | D | F | Y | I | E | L | L | R | R | V | P | G | T | L | T | S | S | F | N | Y | Y | R | A | I | - | - | D | Q |
| 106 UniRef90\_UPI0016124E74\_10\_283 | T | F | N | R | - | - | - | - | - | - | - | - | - | A | G | L | - | E | P | E | A | V | A | E | Y | V | Q | A | F | - | S | A | P | G | A | L | R | A | G | F | D | D | Y | R | G | A | L | - | N | G |
| 107 UniRef90\_A0A263DFR4\_27\_299 | T | A | P | G | - | - | - | - | - | - | - | - | - | R | Q | L | - | D | P | H | A | V | E | V | Y | V | A | A | L | T | E | D | P | E | A | L | R | A | S | F | E | P | Y | R | A | T | - | - | E | E |
| 108 UniRef90\_A0A0U5F3A6\_35\_313 | A | G | S | P | - | - | - | - | - | - | - | - | - | E | A | L | - | P | A | A | A | R | D | F | Y | I | E | Q | L | R | R | D | P | K | A | L | R | A | S | F | E | Y | Y | R | A | I | - | - | D | A |
| 109 UniRef90\_A0A2N3KZL1\_38\_324 | A | T | N | R | - | - | - | - | - | - | - | - | - | A | A | F | - | T | D | E | L | L | D | L | Y | G | A | S | Y | - | A | K | P | H | T | L | H | A | S | F | E | Y | Y | R | A | L | - | - | N | E |
| 110 UniRef90\_A0A1X7C0M0\_25\_283 | M | S | N | P | - | - | - | - | - | - | - | - | - | G | T | F | - | T | E | E | D | L | T | T | Y | A | R | A | Y | - | T | G | R | D | R | L | R | G | G | F | A | H | Y | R | T | L | - | - | L | D |
| 111 UniRef90\_A0A078M0V2\_4\_284 | D | G | L | F | - | - | - | - | - | - | - | - | - | E | L | L | S | S | D | G | S | W | D | E | Y | L | S | H | W | - | K | K | P | G | A | V | K | A | A | L | S | D | Y | R | A | S | Y | - | K | F |
| 112 UniRef90\_A0A0F4Z6H8\_20\_271 | A | A | D | P | - | - | - | - | - | - | - | - | - | A | S | I | - | D | D | D | A | V | D | E | Y | V | R | T | Y | - | S | Q | P | G | A | L | R | S | M | C | S | F | F | R | A | R | - | - | A | T |
| 113 UniRef90\_UPI00161D2319\_22\_292 | T | A | A | P | - | - | - | - | - | - | - | - | - | G | A | I | - | P | Q | Q | A | I | D | V | Y | V | D | Q | L | - | R | D | P | A | A | L | H | A | S | F | G | Y | Y | R | T | L | - | - | D | T |
| 114 UniRef90\_A0A1Q8LX67\_38\_327 | A | L | D | G | E | S | F | G | G | S | R | T | A | P | P | F | - | D | E | R | E | I | D | E | F | T | R | T | Y | - | S | R | P | Q | V | L | S | G | G | F | E | L | Y | R | A | L | - | - | D | R |
| 115 UniRef90\_A0A1Q9S2P6\_18\_309 | A | F | N | G | T | A | F | G | G | A | G | A | R | S | P | F | - | D | D | A | E | V | D | E | Y | A | R | T | Y | - | S | R | P | E | V | L | H | G | G | F | E | L | Y | R | T | L | - | - | G | R |
| 116 UniRef90\_A0A5M7BQA6\_8\_281 | T | V | N | R | - | - | - | - | - | - | - | - | - | H | G | L | - | E | P | E | A | I | D | E | Y | V | R | A | F | - | S | A | P | G | A | L | R | A | G | F | D | D | Y | R | A | S | F | - | P | L |
| 117 UniRef90\_A0A3N6GJS2\_33\_314 | L | L | D | P | - | - | - | - | - | - | - | - | - | G | A | V | - | T | P | A | D | R | A | V | Y | T | R | A | Y | - | D | T | A | D | G | I | R | G | G | N | G | W | Y | Q | T | F | - | - | G | Q |
| 118 UniRef90\_A0A0Q6MZD2\_36\_308 | S | A | T | P | - | - | - | - | - | - | - | - | - | A | R | F | - | S | E | T | A | R | Q | H | Y | A | K | L | Y | - | A | R | P | G | A | M | H | S | G | F | A | Q | F | A | A | F | - | - | D | Q |
| 119 UniRef90\_UPI00052520C0\_22\_293 | G | T | - | - | - | - | - | - | - | - | - | - | - | - | - | M | - | P | E | D | V | I | A | V | Y | V | D | A | L | - | R | R | P | G | A | L | H | A | S | F | Q | Y | Y | R | A | L | - | - | D | R |
| 120 UniRef90\_A0A1Q4XCF8\_2\_276 | L | L | D | R | - | - | - | - | - | - | - | - | - | G | A | V | - | D | E | Q | A | R | A | V | Y | T | R | A | Y | - | D | T | A | D | G | I | R | G | G | N | G | W | Y | Q | T | F | - | - | G | Q |
| 121 UniRef90\_A0A328LZX6\_33\_305 | S | A | D | P | - | - | - | - | - | - | - | - | - | R | R | F | - | T | E | A | D | R | R | H | Y | A | R | L | Y | - | A | S | P | G | A | M | H | A | G | F | A | Q | F | A | A | F | - | - | E | Q |
| 122 UniRef90\_A0A2V8S6L4\_40\_311 | A | A | D | R | K | - | - | - | - | - | - | - | - | H | S | V | - | P | E | A | D | R | Q | I | Y | A | R | A | F | - | A | Q | P | G | A | I | H | A | G | F | E | Y | F | R | N | F | - | - | E | R |
| 123 UniRef90\_A0A1I5EB88\_8\_281 | T | V | N | R | - | - | - | - | - | - | - | - | - | H | G | L | - | E | T | S | A | I | E | E | Y | V | R | A | F | - | S | A | P | G | A | L | R | A | G | F | D | D | Y | R | A | S | F | - | P | L |
| 124 UniRef90\_UPI0014147ABD\_39\_305 | G | G | D | - | - | - | - | - | - | - | - | - | - | - | - | L | - | P | D | E | V | I | D | Y | Y | V | G | I | L | - | S | D | P | D | A | L | R | G | S | F | G | W | Y | R | A | L | - | - | D | A |
| 125 UniRef90\_A0A1I7DL13\_6\_274 | A | F | N | P | - | - | - | - | - | - | - | - | - | N | A | I | - | T | P | N | E | A | A | D | Y | I | R | A | Y | - | S | G | V | G | A | L | R | A | S | L | M | Q | Y | H | A | I | - | - | P | E |
| 126 UniRef90\_A0A3N7EDS3\_31\_305 | S | A | T | P | - | - | - | - | - | - | - | - | - | A | R | F | - | S | E | A | A | R | Q | H | Y | A | R | L | Y | - | A | R | P | G | N | M | H | G | G | F | S | Q | F | A | A | F | - | - | D | Q |
| 127 UniRef90\_UPI0016225F9B\_4\_275 | T | V | Q | R | - | - | - | - | - | - | - | - | - | A | A | L | - | D | A | E | - | - | - | E | Y | Y | R | A | F | - | S | Q | P | G | A | L | R | A | G | F | D | D | Y | R | A | S | F | - | P | D |
| 128 UniRef90\_UPI000997AE50\_107\_382 | S | H | S | P | - | - | - | - | - | - | - | - | - | H | A | V | P | S | P | G | A | V | K | E | Y | V | R | T | Y | - | S | R | P | Q | V | L | H | G | G | F | E | F | Y | R | Y | W | - | - | N | Q |
| 129 UniRef90\_A0A0N7F3H5\_6\_275 | T | Y | Q | R | - | - | - | - | - | - | - | - | - | Q | G | L | - | - | - | D | A | A | D | E | Y | I | R | A | F | - | S | A | P | G | A | L | R | A | G | F | D | D | Y | R | A | S | F | - | P | T |
| 130 UniRef90\_A0A3Q8F441\_11\_281 | A | Y | D | P | - | - | - | - | - | - | - | - | - | A | A | I | - | S | G | E | A | F | D | T | Y | V | R | A | Y | - | S | A | P | G | A | V | R | G | A | M | S | D | Y | R | A | N | - | - | A | V |
| 131 UniRef90\_UPI001487D2FB\_23\_300 | A | G | N | P | - | - | - | - | - | - | - | - | - | A | K | V | - | D | E | A | T | R | V | Y | Y | A | R | L | Y | - | A | R | P | G | A | M | R | S | A | F | A | Q | F | R | S | F | - | - | P | Q |
| 132 UniRef90\_A0A437GNC7\_38\_307 | I | P | V | P | - | - | - | - | - | - | - | - | - | N | P | I | - | P | A | D | I | R | A | F | Y | I | D | L | I | K | R | D | R | R | T | L | R | A | S | F | D | Y | Y | R | A | I | - | - | D | D |
| 133 UniRef90\_A0A1H3SWJ7\_6\_283 | A | F | R | R | - | - | - | - | - | - | - | - | - | E | A | F | - | T | E | A | D | L | R | H | Y | A | E | A | Y | - | A | Q | P | G | A | L | R | A | S | F | D | D | Y | R | A | A | R | - | H | E |
| 134 UniRef90\_UPI0013DD6281\_49\_329 | A | Y | G | G | K | S | F | G | G | P | G | A | K | S | P | F | - | T | D | R | Q | E | D | E | F | A | R | T | Y | - | R | R | P | Q | V | L | T | G | G | F | E | L | Y | R | T | L | - | - | E | R |
| 135 UniRef90\_I0L7C1\_27\_272 | T | F | E | R | - | - | - | - | - | - | - | - | - | R | G | V | - | P | S | E | V | R | D | A | F | V | S | A | Y | - | T | G | R | E | A | L | R | C | G | F | G | Y | Y | R | A | A | - | - | E | Q |
| 136 UniRef90\_A0A260DLM1\_45\_326 | A | Y | R | G | T | A | F | G | G | P | G | A | T | S | P | F | - | T | D | A | E | I | D | E | Y | A | R | T | Y | - | D | R | P | E | V | L | R | A | G | F | D | L | Y | R | S | L | - | - | D | R |
| 137 UniRef90\_UPI0005BDD2EC\_7\_280 | T | Y | N | R | - | - | - | - | - | - | - | - | - | H | G | L | - | E | P | E | T | V | D | T | Y | V | R | A | F | - | S | R | P | G | A | L | R | A | G | F | D | D | Y | R | A | L | - | - | E | E |
| 138 UniRef90\_A0A6G9YBC1\_27\_296 | T | V | N | R | - | - | - | - | - | - | - | - | - | H | G | L | - | T | E | E | A | V | D | E | Y | I | R | A | F | - | S | V | P | G | A | L | R | A | G | F | D | D | Y | R | A | A | - | - | E | L |
| 139 UniRef90\_UPI00145FA88A\_8\_280 | T | Y | R | R | - | - | - | - | - | - | - | - | - | D | A | F | - | D | P | A | A | I | T | T | Y | V | N | A | F | - | S | A | P | G | A | L | R | A | G | F | D | D | Y | R | A | S | F | - | P | T |
| 140 UniRef90\_A0A2W0B1K8\_46\_299 | T | G | D | P | - | - | - | - | - | - | - | - | - | S | K | P | - | D | E | A | T | R | D | F | F | A | A | T | Y | - | A | Q | P | G | G | M | R | A | G | F | A | Q | F | T | A | F | - | - | S | Q |
| 141 UniRef90\_A0A5B8G622\_28\_299 | S | A | T | P | - | - | - | - | - | - | - | - | - | A | K | F | - | T | E | A | S | R | E | H | Y | A | A | L | Y | - | A | L | P | G | A | M | H | A | G | F | E | Q | F | K | A | F | - | - | D | Q |
| 142 UniRef90\_A0A4S1XGQ8\_21\_302 | S | A | D | P | - | - | - | - | - | - | - | - | - | A | H | F | - | D | E | A | S | R | A | H | Y | A | A | I | Y | - | A | R | P | G | H | M | H | A | G | F | A | Q | F | A | A | F | - | - | D | Q |
| 143 UniRef90\_A0A251YJ72\_8\_294 | T | A | V | A | - | - | - | - | - | - | - | - | - | G | A | V | - | T | D | A | D | V | D | E | F | A | R | G | Y | - | A | R | P | G | G | W | A | G | A | V | G | L | Y | R | S | M | - | - | L | A |
| 144 UniRef90\_UPI000A0527D2\_65\_318 | A | Y | Q | P | - | - | - | - | - | - | - | - | - | G | A | I | - | T | E | S | D | I | D | V | Y | A | R | A | F | - | E | A | P | G | A | I | R | A | L | C | E | I | Y | R | E | L | - | - | D | H |
| 145 UniRef90\_A0A4V2S7H9\_8\_280 | T | H | Q | R | - | - | - | - | - | - | - | - | - | S | G | L | - | T | K | D | A | V | D | E | Y | V | R | A | F | - | S | A | P | G | A | L | R | A | G | F | D | D | Y | R | A | S | F | - | P | D |
| 146 UniRef90\_UPI00131B216A\_18\_247 | T | P | D | P | - | - | - | - | - | - | - | - | - | A | A | I | - | G | D | F | D | R | A | V | Y | A | H | A | Y | - | S | T | P | E | A | I | R | A | S | N | S | W | Y | R | A | F | - | - | G | Q |
| 147 UniRef90\_A0A1G8T9Y6\_5\_243 | E | V | R | K | - | - | - | - | - | - | - | - | - | G | A | I | - | G | P | A | Q | V | R | E | F | A | G | Y | L | - | R | D | D | A | H | L | R | A | S | L | E | W | F | R | A | F | - | - | P | A |
| 148 UniRef90\_UPI00161F613F\_5\_254 | - | - | - | - | - | - | - | - | - | - | - | - | - | - | - | - | - | P | A | G | D | V | G | E | F | A | R | T | Y | - | A | R | P | G | G | W | R | G | A | A | G | L | Y | R | S | M | - | - | L | R |
| 149 UniRef90\_UPI00142071FA\_80\_362 | G | G | G | A | - | - | - | - | - | - | - | - | - | - | T | L | - | P | D | H | A | I | E | Y | Y | V | S | L | Y | N | R | D | R | S | T | L | R | A | S | F | G | L | Y | R | A | W | - | - | D | A |
| 150 UniRef90\_A0A6G7Q8F6\_7\_219 | S | S | R | A | - | - | - | - | - | - | - | - | - | G | T | F | - | S | E | A | D | L | D | L | Y | A | R | E | W | - | L | R | P | G | A | V | T | A | M | L | N | W | Y | R | A | L | - | - | A | R |

  
  

|  |  |  |  |  |  |  |  |  |  |  |  |  |  |  |  |  |  |  |  |  |  |  |  |  |  |  |  |  |  |  |  |  |  |  |  |  |  |  |  |  |  |  |  |  |  |  |  |  |  |  |
| --- | --- | --- | --- | --- | --- | --- | --- | --- | --- | --- | --- | --- | --- | --- | --- | --- | --- | --- | --- | --- | --- | --- | --- | --- | --- | --- | --- | --- | --- | --- | --- | --- | --- | --- | --- | --- | --- | --- | --- | --- | --- | --- | --- | --- | --- | --- | --- | --- | --- | --- |
| **001 Input\_protein\_seq** | S | S | A | Q | N | R | K | L | - | Q | A | L | G | K | L | K | M | P | V | L | A | V | S | A | D | Q | - | - | - | - | - | - | - | - | - | - | - | - | G | S | - | - | - | - | - | - | - | I | P | D |
| 002 UniRef90\_UPI001430CB85\_4\_307 | S | A | R | Q | N | Q | E | L | - | S | A | Q | G | K | L | R | M | P | L | L | A | L | S | A | D | Q | - | - | - | - | - | - | - | - | - | - | - | - | G | S | - | - | - | - | - | - | - | I | P | D |
| 003 UniRef90\_UPI00055F8D66\_10\_306 | S | A | Q | Q | N | R | E | L | - | S | A | M | G | K | L | K | T | P | V | L | A | L | G | S | D | Q | - | - | - | - | - | - | - | - | - | - | - | - | G | S | - | - | - | - | - | - | - | I | T | D |
| 004 UniRef90\_UPI00161D8BA7\_15\_312 | S | A | I | Q | N | K | A | L | - | S | G | K | G | K | L | K | A | P | L | L | A | V | G | S | D | Q | - | - | - | - | - | - | - | - | - | - | - | - | G | S | - | - | - | - | - | - | - | I | A | D |
| 005 UniRef90\_A0A4R7GLU7\_7\_305 | S | A | E | Q | N | R | E | L | - | N | R | R | G | K | L | P | M | P | L | L | A | V | S | A | S | Q | - | - | - | - | - | - | - | - | - | - | - | - | G | S | - | - | - | - | - | - | - | I | P | D |
| 006 UniRef90\_UPI00041CD164\_24\_309 | S | A | Q | Q | N | R | E | L | - | S | R | G | G | K | L | A | M | P | V | L | G | M | G | S | D | Q | - | - | - | - | - | - | - | - | - | - | - | - | G | S | - | - | - | - | - | - | - | L | P | D |
| 007 UniRef90\_I0QSF8\_15\_298 | S | S | R | Q | N | A | H | F | - | K | T | L | G | K | L | T | T | P | V | L | A | F | S | A | E | Q | - | - | - | - | - | - | - | - | - | - | - | - | G | S | - | - | - | - | - | - | - | I | A | D |
| 008 UniRef90\_A0A2N5ENH7\_9\_301 | S | A | A | Q | N | R | A | L | - | C | E | Q | G | K | L | T | L | P | L | L | A | V | S | A | D | Q | - | - | - | - | - | - | - | - | - | - | - | - | G | S | - | - | - | - | - | - | - | I | P | D |
| 009 UniRef90\_A0A0Q8E2M6\_29\_312 | S | A | R | Q | N | R | A | F | - | A | A | R | E | K | L | A | M | P | V | L | A | L | S | A | D | Q | - | - | - | - | - | - | - | - | - | - | - | - | G | S | - | - | - | - | - | - | - | I | P | D |
| 010 UniRef90\_A0A0F5XY46\_6\_287 | S | A | G | Q | N | R | Q | R | - | A | Q | A | G | K | L | A | L | P | L | L | A | V | S | A | D | Q | - | - | - | - | - | - | - | - | - | - | - | - | G | S | - | - | - | - | - | - | - | I | P | D |
| 011 UniRef90\_A0A3N4NUX5\_4\_284 | S | A | A | Q | N | R | A | L | - | L | Q | T | G | P | L | D | L | P | L | L | A | V | S | A | D | Q | - | - | - | - | - | - | - | - | - | - | - | - | G | S | - | - | - | - | - | - | - | I | A | D |
| 012 UniRef90\_UPI000DDED2D9\_11\_293 | S | A | E | Q | N | R | K | L | - | S | E | S | S | K | L | Q | M | P | V | L | A | I | R | A | D | Q | - | - | - | - | - | - | - | - | - | - | - | - | G | S | - | - | - | - | - | - | - | M | P | D |
| 013 UniRef90\_UPI000DDED53B\_10\_292 | S | A | N | Q | N | R | T | L | - | L | E | H | G | G | L | K | M | P | T | L | G | L | G | A | D | Q | - | - | - | - | - | - | - | - | - | - | - | - | G | S | - | - | - | - | - | - | - | I | P | D |
| 014 UniRef90\_A0A1G4JKM0\_6\_288 | S | A | R | Q | N | K | A | L | - | L | D | E | G | K | I | L | P | P | L | L | A | V | S | S | D | Q | - | - | - | - | - | - | - | - | - | - | - | - | G | S | - | - | - | - | - | - | - | I | P | S |
| 015 UniRef90\_UPI0016728815\_21\_305 | S | A | R | R | N | H | E | A | - | L | A | R | Q | R | L | S | V | P | V | L | G | I | S | G | S | H | - | - | - | - | - | - | - | - | - | - | - | - | G | S | - | - | - | - | - | - | - | V | P | D |
| 016 UniRef90\_A0A3N2H8D2\_14\_297 | S | A | R | R | N | R | E | A | - | L | Q | Q | R | R | L | T | V | P | V | L | G | I | S | S | S | H | - | - | - | - | - | - | - | - | - | - | - | - | G | S | - | - | - | - | - | - | - | I | P | D |
| 017 UniRef90\_UPI0005644DD1\_9\_307 | S | A | R | R | N | H | Q | A | - | L | S | R | K | H | L | T | L | P | V | L | G | I | S | S | S | H | - | - | - | - | - | - | - | - | - | - | - | - | G | S | - | - | - | - | - | - | - | I | P | D |
| 018 UniRef90\_A0A6B2VDS0\_18\_308 | S | A | R | K | N | R | E | A | - | L | T | S | R | R | L | T | V | P | I | L | G | I | S | S | S | H | - | - | - | - | - | - | - | - | - | - | - | - | G | S | - | - | - | - | - | - | - | I | P | D |
| 019 UniRef90\_A0A6G3RZZ1\_25\_307 | S | A | R | R | N | H | D | A | - | L | E | R | G | N | L | S | V | P | V | L | G | I | S | G | S | H | - | - | - | - | - | - | - | - | - | - | - | - | G | S | - | - | - | - | - | - | - | I | P | D |
| 020 UniRef90\_A0A209C7X3\_21\_305 | S | A | R | K | N | H | E | A | - | L | E | R | Q | R | L | T | V | P | V | L | G | I | S | S | S | N | - | - | - | - | - | - | - | - | - | - | - | - | G | S | - | - | - | - | - | - | - | I | P | D |
| 021 UniRef90\_UPI001269E708\_26\_309 | S | A | R | R | N | H | E | A | - | L | E | R | Q | R | L | T | V | P | V | L | G | I | S | S | S | H | - | - | - | - | - | - | - | - | - | - | - | - | G | S | - | - | - | - | - | - | - | I | P | D |
| 022 UniRef90\_A0A2M9J7W9\_25\_306 | S | A | R | R | N | H | E | A | - | L | E | R | G | H | L | T | V | P | V | L | G | I | S | S | S | H | - | - | - | - | - | - | - | - | - | - | - | - | G | S | - | - | - | - | - | - | - | I | P | D |
| 023 UniRef90\_A0A6G9F3J8\_23\_307 | S | A | R | G | N | H | A | A | - | L | D | R | Q | H | L | T | L | P | V | L | G | I | S | S | S | H | - | - | - | - | - | - | - | - | - | - | - | - | G | S | - | - | - | - | - | - | - | I | P | D |
| 024 UniRef90\_A0A1H2UZ41\_14\_292 | D | V | E | Q | N | K | S | L | - | - | A | T | T | K | I | D | T | P | T | L | A | L | G | G | D | V | - | - | - | - | - | - | - | - | - | - | - | - | G | M | - | - | - | - | - | - | - | S | P | D |
| 025 UniRef90\_UPI0012B05B7D\_16\_286 | D | M | E | Q | N | R | V | F | - | - | G | Q | Q | L | L | K | I | P | V | L | A | L | G | G | D | K | - | - | - | - | - | - | - | - | - | - | - | - | G | S | - | - | - | - | - | - | - | A | P | D |
| 026 UniRef90\_A0A5C8T429\_18\_294 | D | M | A | Q | N | R | E | H | - | - | A | R | R | K | I | R | T | P | I | L | T | V | G | G | D | V | - | - | - | - | - | - | - | - | - | - | - | - | G | L | - | - | - | - | - | - | - | G | S | M |
| 027 UniRef90\_UPI0012E86E3E\_10\_309 | G | L | A | H | S | H | A | R | - | - | A | E | R | K | L | A | M | P | V | L | A | F | G | A | E | T | - | - | - | - | - | - | - | - | - | - | - | - | G | V | - | - | - | - | - | - | - | G | A | M |
| 028 UniRef90\_A0A1A9HTL2\_13\_300 | G | L | Q | Q | N | R | L | R | - | - | A | A | T | P | L | T | I | P | V | L | A | I | G | G | Q | H | - | - | - | - | - | - | - | - | - | - | - | - | S | V | - | - | - | - | - | - | - | G | A | G |
| 029 UniRef90\_A0A0R1V0C7\_7\_281 | D | F | K | I | N | E | P | L | - | - | C | T | K | K | I | V | I | P | L | L | T | I | N | G | E | F | - | - | - | - | - | - | - | - | - | - | - | - | G | S | - | - | - | - | - | - | - | S | R | D |
| 030 UniRef90\_A0A3N1M9Y1\_5\_282 | D | A | A | D | N | V | R | L | L | E | S | G | F | R | L | P | M | P | V | L | A | L | G | G | A | R | - | - | - | - | - | - | - | - | - | - | - | - | T | E | - | - | A | R | A | - | R | G | L | E |
| 031 UniRef90\_A0A158JUX7\_17\_294 | D | T | A | D | N | K | R | F | - | - | G | A | T | K | L | P | M | P | V | L | A | I | G | A | E | Y | - | - | - | - | - | - | - | - | - | - | - | - | G | Y | - | - | - | - | - | - | - | K | D | S |
| 032 UniRef90\_A0A0U3LIL8\_12\_296 | A | L | Q | A | N | Q | A | R | - | - | A | A | R | K | L | E | M | P | V | M | A | I | G | G | Q | L | - | - | - | - | - | - | - | - | - | - | - | - | S | V | - | - | - | - | - | - | - | A | S | S |
| 033 UniRef90\_A0A248JS33\_7\_301 | E | L | A | R | A | K | A | R | - | - | A | G | K | G | L | M | L | P | I | L | A | V | G | A | R | T | - | - | - | - | - | - | - | - | - | - | - | - | G | V | - | - | - | - | - | - | - | G | T | V |
| 034 UniRef90\_A0A1Y0REZ4\_12\_287 | D | A | K | H | N | R | E | S | - | - | G | K | T | K | L | Q | M | P | V | L | A | L | G | G | D | S | - | - | - | - | - | - | - | - | - | - | - | - | C | L | - | - | - | - | - | - | - | G | D | L |
| 035 UniRef90\_A0A0M2WRU5\_3\_280 | T | I | R | Q | N | L | Q | R | - | - | A | K | N | S | L | A | M | P | V | L | A | I | G | A | D | H | - | - | - | - | - | - | - | - | - | - | - | - | A | T | - | - | - | - | - | - | - | N | D | A |
| 036 UniRef90\_A0A0N8GFM1\_6\_279 | D | A | A | D | N | R | A | N | L | D | R | L | G | K | L | T | M | P | V | L | A | L | G | G | E | K | - | - | - | - | - | - | - | - | - | - | - | - | A | F | - | - | - | - | G | - | R | G | M | E |
| 037 UniRef90\_A0A252EMP1\_7\_277 | T | A | V | Q | S | R | E | L | - | - | A | R | T | K | L | T | I | P | V | L | A | Y | G | G | S | H | - | - | - | - | - | - | - | - | - | - | - | - | C | M | - | - | - | - | - | - | - | S | E | I |
| 038 UniRef90\_W0A6K5\_40\_320 | T | I | A | Q | N | K | Q | R | - | - | A | E | K | K | L | M | I | P | V | L | A | I | G | A | D | H | - | - | - | - | - | - | - | - | - | - | - | - | A | T | - | - | - | - | - | - | - | R | D | A |
| 039 UniRef90\_A0A3S1SM66\_23\_304 | G | T | A | A | N | R | A | R | - | - | A | E | T | L | L | E | A | P | V | L | A | L | G | A | E | R | - | - | - | - | - | - | - | - | - | - | - | - | G | V | - | - | - | - | - | - | - | G | P | A |
| 040 UniRef90\_A0A2E5L5C6\_7\_286 | D | I | E | D | N | K | K | N | I | - | Q | K | M | K | L | P | M | P | V | L | A | I | G | G | G | V | - | - | - | - | - | - | - | - | - | - | - | - | S | Y | P | N | G | R | G | - | R | G | K | Q |
| 041 UniRef90\_M5D1B4\_14\_289 | T | I | R | Q | N | Q | L | R | - | - | A | Q | R | R | L | A | M | P | V | L | A | I | G | A | E | H | - | - | - | - | - | - | - | - | - | - | - | - | A | T | - | - | - | - | - | - | - | A | D | A |
| 042 UniRef90\_UPI0016845BA7\_31\_303 | D | T | R | Y | N | R | E | H | - | - | - | I | K | M | L | D | M | P | V | L | A | L | G | G | E | S | - | - | - | - | - | - | - | - | - | - | - | - | T | L | - | - | - | - | - | - | - | G | D | F |
| 043 UniRef90\_UPI0014648199\_14\_291 | T | I | R | Q | N | L | D | R | - | - | A | T | R | M | L | P | M | P | V | L | A | I | G | A | E | H | - | - | - | - | - | - | - | - | - | - | - | - | A | T | - | - | - | - | - | - | - | N | D | A |
| 044 UniRef90\_K9RUM7\_12\_289 | T | M | A | A | N | T | L | A | - | - | P | E | T | K | F | T | Q | P | I | F | A | M | G | G | E | F | - | - | - | - | - | - | - | - | - | - | - | - | A | T | - | - | - | - | - | - | - | K | T | L |
| 045 UniRef90\_A0A1Q2U086\_13\_290 | T | I | A | T | N | A | L | E | - | - | P | G | V | R | F | S | Q | P | I | F | A | M | G | G | E | F | - | - | - | - | - | - | - | - | - | - | - | - | A | T | - | - | - | - | - | - | - | K | N | L |
| 046 UniRef90\_B9JMN1\_3\_282 | S | A | A | Q | N | A | Q | H | - | - | K | Q | H | K | L | T | I | P | V | S | A | W | A | G | E | A | - | - | - | - | - | - | - | - | - | - | - | - | C | L | - | - | - | - | - | - | - | G | P | L |
| 047 UniRef90\_A0A2X1TAZ7\_33\_319 | T | I | L | Q | N | K | Q | R | - | - | A | E | K | K | L | T | M | P | V | L | A | I | G | A | D | H | - | - | - | - | - | - | - | - | - | - | - | - | A | T | - | - | - | - | - | - | - | R | D | A |
| 048 UniRef90\_UPI00131A8152\_6\_279 | D | I | E | D | N | S | S | R | - | A | L | H | Y | R | P | D | I | P | V | L | A | I | G | G | G | K | - | - | - | - | - | - | - | - | - | - | - | - | S | W | - | - | - | - | G | - | R | R | A | Q |
| 049 UniRef90\_F7YB04\_47\_316 | D | E | E | Q | N | K | A | F | - | - | A | K | H | K | L | T | M | P | V | L | G | I | S | G | S | G | - | - | - | - | - | - | - | - | - | - | - | - | G | L | - | - | - | - | - | - | - | A | S | M |
| 050 UniRef90\_A0A1A3M1Z8\_5\_294 | D | A | V | D | N | Q | R | V | M | S | E | K | G | K | L | T | M | P | V | L | G | L | G | G | N | A | - | - | - | - | - | - | - | - | - | - | - | - | S | F | - | - | - | - | - | - | F | L | P | I |
| 051 UniRef90\_UPI0004C32CE1\_32\_312 | D | I | A | D | G | K | - | - | - | - | T | Y | A | K | L | T | P | P | V | L | G | L | A | S | V | L | - | - | - | - | - | - | - | - | - | - | - | - | S | - | - | - | - | - | - | - | - | I | E | Q |
| 052 UniRef90\_A0A0J6NQJ9\_12\_287 | T | M | R | Q | N | Q | R | R | - | - | A | Q | S | K | L | T | M | P | V | L | A | I | G | A | E | H | - | - | - | - | - | - | - | - | - | - | - | - | A | T | - | - | - | - | - | - | - | G | N | A |
| 053 UniRef90\_A0A534ESC2\_32\_321 | G | L | A | Q | A | K | V | R | - | - | A | A | Q | R | L | P | M | P | V | L | A | L | G | G | D | E | - | - | - | - | - | - | - | - | - | - | - | - | G | V | - | - | - | - | - | - | - | G | D | A |
| 054 UniRef90\_A0A1H1Z302\_21\_301 | T | I | A | Q | N | E | Q | R | - | - | R | K | T | R | L | T | L | P | V | L | A | I | G | G | A | L | - | - | - | - | - | - | - | - | - | - | - | - | Y | S | - | - | - | - | - | - | - | G | A | M |
| 055 UniRef90\_A0A158DLE6\_24\_295 | D | R | D | D | N | L | Q | F | - | - | G | L | K | K | L | E | M | P | V | L | A | V | G | C | D | F | - | - | - | - | - | - | - | - | - | - | - | - | G | Y | - | - | - | - | - | - | - | G | G | G |
| 056 UniRef90\_A0A163VVR8\_16\_280 | T | V | R | Q | N | R | L | R | - | - | A | A | T | P | L | T | M | P | V | L | T | V | G | G | E | H | - | - | - | - | - | - | - | - | - | - | - | - | A | T | - | - | - | - | - | - | - | G | D | A |
| 057 UniRef90\_UPI000369F0DF\_7\_289 | T | I | R | Q | N | R | E | R | - | - | A | S | R | P | L | T | A | P | V | L | A | I | G | C | E | H | - | - | - | - | - | - | - | - | - | - | - | - | A | T | - | - | - | - | - | - | - | A | D | A |
| 058 UniRef90\_UPI00098F4FB4\_43\_309 | D | V | E | T | N | R | A | L | - | - | A | A | R | K | L | T | M | P | V | L | A | M | S | G | A | G | - | - | - | - | - | - | - | - | - | - | - | - | G | M | - | - | - | - | - | - | - | G | S | M |
| 059 UniRef90\_UPI0015A1AD76\_10\_292 | D | G | A | H | N | R | E | N | - | V | Q | Q | G | K | L | S | M | P | V | L | G | V | G | G | A | C | - | - | - | - | - | - | - | - | - | - | - | - | S | G | - | - | - | - | - | - | - | G | P | Y |
| 060 UniRef90\_UPI001689F051\_63\_318 | D | I | E | H | N | R | T | L | - | S | A | A | G | P | I | T | M | P | T | A | V | V | G | A | R | H | - | - | - | - | - | - | - | - | - | - | - | - | G | V | - | - | - | - | - | - | - | G | L | S |
| 061 UniRef90\_UPI000DD77D66\_43\_313 | D | V | V | A | N | R | V | F | - | - | A | Q | S | K | L | E | M | P | V | L | A | M | S | G | I | G | - | - | - | - | - | - | - | - | - | - | - | - | G | L | - | - | - | - | - | - | - | G | T | V |
| 062 UniRef90\_A0A3M0I8T4\_31\_307 | T | I | A | Q | N | A | L | R | - | - | K | T | T | R | L | T | L | P | V | L | A | I | A | G | A | A | - | - | - | - | - | - | - | - | - | - | - | - | N | S | - | - | - | - | - | - | - | G | E | L |
| 063 UniRef90\_UPI000377E2D1\_4\_271 | D | I | A | A | N | E | T | L | - | - | - | - | A | K | T | D | V | P | V | L | A | V | G | G | A | T | - | - | - | - | - | - | - | - | - | - | - | - | S | F | - | - | - | - | G | - | R | G | A | E |
| 064 UniRef90\_UPI0016198B53\_15\_286 | N | I | A | Q | N | H | R | R | - | - | R | Q | R | P | L | T | L | P | V | L | G | V | S | G | D | R | - | - | - | - | - | - | - | - | - | - | - | - | G | Q | - | - | - | - | - | - | - | G | E | R |
| 065 UniRef90\_UPI0013C0217D\_48\_325 | D | I | E | D | M | R | - | - | - | - | S | Y | G | R | V | T | A | P | L | L | G | V | A | S | E | V | - | - | - | - | - | - | - | - | - | - | - | - | S | - | - | - | - | - | - | - | - | H | G | E |
| 066 UniRef90\_C7PGN2\_5\_260 | D | I | N | D | N | K | A | F | - | - | G | Q | N | L | L | Q | M | P | V | L | A | I | G | G | E | H | - | - | - | - | - | - | - | - | - | - | - | - | G | L | - | - | - | - | - | - | - | G | Q | L |
| 067 UniRef90\_A0A1F7Q7Q2\_7\_275 | D | F | E | H | D | G | A | D | - | - | R | D | R | K | I | G | V | P | T | L | V | L | W | G | G | A | - | - | - | - | - | - | - | - | - | - | - | G | R | V | - | - | - | - | P | - | Q | A | A | D |
| 068 UniRef90\_A0A2V7F1K9\_9\_274 | D | L | E | H | D | R | A | D | - | - | R | D | K | K | I | A | M | P | T | L | V | L | W | G | E | G | - | - | - | - | - | - | - | - | - | - | - | - | R | S | - | - | - | - | P | - | Q | A | A | D |
| 069 UniRef90\_UPI001616C592\_31\_308 | I | V | E | Q | S | A | H | R | - | - | K | K | T | P | V | E | I | P | I | L | A | I | S | G | S | A | - | - | - | - | - | - | - | - | - | - | - | - | G | - | - | - | - | - | - | - | - | G | L | D |
| 070 UniRef90\_UPI001456849A\_71\_360 | D | G | A | A | N | K | A | W | L | G | S | G | N | K | L | K | M | P | V | L | W | L | G | G | E | G | T | P | E | A | A | M | A | T | V | G | V | I | S | T | - | - | - | - | - | - | - | G | D | F |
| 071 UniRef90\_A0A2S8J6S6\_18\_282 | D | A | E | D | N | R | K | F | - | - | K | Q | E | P | L | T | I | P | V | L | A | L | G | G | E | N | - | - | - | - | - | - | - | - | - | - | - | - | R | W | - | - | - | - | - | - | - | G | P | K |
| 072 UniRef90\_A0A2I2GC12\_18\_280 | S | A | K | Q | N | V | E | F | - | - | A | K | K | P | L | D | V | P | V | L | A | L | G | G | S | K | - | - | - | - | - | - | - | - | - | - | - | - | S | V | - | - | - | - | - | - | - | G | G | N |
| 073 UniRef90\_A0A549T469\_41\_312 | D | A | R | S | V | A | A | L | - | - | A | D | R | R | L | D | M | P | V | L | A | I | A | G | R | H | - | - | - | - | - | - | - | - | - | - | - | - | G | V | - | - | - | - | - | - | - | G | E | R |
| 074 UniRef90\_A0A5C4LJC6\_5\_278 | D | H | A | A | A | L | A | Y | - | - | K | K | R | P | L | E | I | P | V | L | G | L | G | G | D | Q | - | - | - | - | - | - | - | - | - | - | - | - | R | F | - | - | - | - | - | - | - | G | P | R |
| 075 UniRef90\_UPI001619D806\_9\_283 | D | A | E | H | D | D | A | D | A | E | A | G | H | R | L | T | M | P | V | L | A | M | W | G | S | A | - | - | - | - | - | - | - | - | - | - | - | - | G | L | - | - | - | - | L | - | G | S | L | P |
| 076 UniRef90\_A0A239P1A5\_69\_347 | I | I | A | Q | N | T | E | R | - | - | R | K | T | K | L | K | M | P | V | L | T | I | A | G | E | I | - | - | - | - | - | - | - | - | - | - | - | - | A | V | - | - | - | - | - | - | - | G | T | G |
| 077 UniRef90\_UPI0005262A70\_5\_280 | E | G | D | E | I | R | S | I | - | A | A | E | G | K | L | T | V | P | V | L | A | V | D | G | G | S | - | - | - | - | - | - | - | - | - | - | - | - | - | - | - | - | - | - | - | - | - | G | P | F |
| 078 UniRef90\_A0A241XUQ6\_27\_314 | S | V | R | Q | N | A | E | L | - | - | A | K | T | R | L | Q | M | P | T | M | T | L | A | G | G | G | H | G | - | - | - | - | - | - | - | - | - | - | G | M | - | - | - | - | - | - | - | G | T | F |
| 079 UniRef90\_A0A160FQ65\_32\_312 | S | I | P | Q | N | R | E | R | - | L | T | R | G | K | L | T | L | P | V | L | A | F | A | G | E | L | - | - | - | - | - | - | - | - | - | - | - | - | C | C | - | - | - | - | - | - | - | G | G | L |
| 080 UniRef90\_UPI0006AE4C23\_4\_281 | D | I | Q | E | A | A | - | - | - | - | H | H | P | K | L | S | M | P | V | L | V | P | G | G | K | F | - | - | - | - | - | - | - | - | - | - | - | - | - | - | - | - | - | - | - | - | - | L | E | L |
| 081 UniRef90\_T0H3T7\_11\_281 | D | V | A | Q | D | L | E | D | - | - | A | D | V | K | I | A | C | P | T | L | A | L | W | G | E | S | F | G | - | - | - | - | - | - | - | - | - | - | A | V | - | - | - | - | G | - | K | M | F | D |
| 082 UniRef90\_A0A4R2IH15\_5\_277 | T | I | E | Q | N | T | R | R | - | - | K | Q | T | R | L | T | L | P | V | L | T | I | A | G | A | N | - | - | - | - | - | - | - | - | - | - | - | - | S | L | - | - | - | - | - | - | - | N | D | A |
| 083 UniRef90\_UPI00140B863E\_4\_276 | D | A | E | L | N | R | R | T | - | - | A | E | E | P | L | T | T | P | V | L | A | V | G | G | D | Q | - | - | - | - | - | - | - | - | - | - | - | - | A | M | - | - | - | - | - | - | - | A | G | A |
| 084 UniRef90\_A0A3R9UXB7\_22\_297 | D | V | A | D | L | R | - | - | - | - | T | Y | D | P | V | T | V | P | L | L | A | L | G | G | D | R | - | - | - | - | - | - | - | - | - | - | - | - | S | N | - | - | - | - | - | - | - | Y | E | A |
| 085 UniRef90\_UPI000F8D1465\_20\_303 | N | I | L | L | N | G | R | R | - | - | K | M | R | M | L | E | M | P | V | L | A | I | G | G | A | L | - | - | - | - | - | - | - | - | - | - | - | - | G | L | - | - | - | - | - | - | - | G | D | R |
| 086 UniRef90\_A0A6J4HYT7\_23\_307 | D | I | R | D | G | K | - | - | - | - | T | Y | G | K | L | G | M | P | V | L | G | I | G | G | S | G | - | - | - | - | - | - | - | - | - | - | - | - | - | - | - | - | - | - | - | - | - | F | E | L |
| 087 UniRef90\_A0A1D8SMR3\_19\_296 | G | D | Q | V | V | A | L | A | - | - | A | E | G | P | L | D | I | P | V | L | A | I | G | G | E | Y | - | - | - | - | - | - | - | - | - | - | - | - | G | M | - | - | - | - | - | - | - | R | D | G |
| 088 UniRef90\_A0A4V2HUZ4\_48\_320 | D | A | R | F | A | E | A | L | - | - | R | G | R | K | L | T | P | P | V | M | T | I | T | G | R | Y | - | - | - | - | - | - | - | - | - | - | - | - | G | V | - | - | - | - | - | - | - | G | D | K |
| 089 UniRef90\_UPI0010582A1B\_5\_279 | D | H | A | A | A | L | E | Y | - | - | K | K | Q | P | L | E | I | P | V | L | G | L | G | G | D | Q | - | - | - | - | - | - | - | - | - | - | - | - | R | F | - | - | - | - | - | - | - | G | A | Q |
| 090 UniRef90\_UPI00166CE541\_54\_338 | D | V | A | D | N | S | A | Y | - | - | R | T | K | K | L | T | M | P | V | L | A | I | G | A | R | G | - | - | - | - | - | - | - | - | - | - | - | - | S | L | - | - | - | - | - | - | - | G | G | F |
| 091 UniRef90\_A0A5C1HX54\_34\_307 | D | A | V | D | N | V | E | L | - | - | M | K | K | K | L | P | M | P | L | L | A | M | G | S | D | H | - | - | - | - | - | - | - | - | - | - | - | - | L | A | - | - | - | - | - | - | - | G | A | F |
| 092 UniRef90\_A0A4D4KET2\_34\_306 | T | I | A | Q | N | Q | Q | R | - | - | K | T | R | R | L | T | L | P | V | L | T | L | A | G | A | E | - | - | - | - | - | - | - | - | - | - | - | - | N | L | - | - | - | - | - | - | - | G | E | A |
| 093 UniRef90\_A0A1I1XP17\_9\_276 | D | A | A | R | I | K | A | E | L | A | R | A | G | K | L | R | M | P | V | L | A | I | G | G | A | A | - | - | - | - | - | - | - | - | - | - | - | G | G | L | - | - | - | - | - | - | - | G | E | A |
| 094 UniRef90\_A0A1D7VX60\_23\_299 | T | S | A | Q | D | R | Q | R | - | - | R | A | R | P | L | T | L | P | V | L | A | I | G | G | A | G | - | - | - | - | - | - | - | - | - | - | - | - | S | L | - | - | - | - | - | - | - | G | E | N |
| 095 UniRef90\_A0A2T4ZAU7\_6\_296 | D | L | P | R | Y | R | M | E | K | E | A | N | R | K | M | D | T | P | C | L | L | L | W | G | K | D | - | - | - | - | - | - | - | - | - | - | - | - | G | N | - | - | - | - | L | - | A | E | Q | P |
| 096 UniRef90\_UPI00143A9489\_23\_300 | D | A | V | D | N | R | A | L | L | - | A | R | G | P | L | T | M | P | V | L | A | I | G | G | A | A | - | - | - | - | - | - | - | - | - | - | - | - | S | L | - | - | - | - | - | - | - | G | P | V |
| 097 UniRef90\_A0A7C7NVI2\_6\_287 | D | P | P | H | W | Q | A | D | - | - | A | G | R | Q | L | P | H | Q | V | L | F | L | Y | G | S | R | - | - | - | - | - | - | - | - | - | - | - | - | R | V | R | T | A | E | A | Q | G | A | G | P |
| 098 UniRef90\_UPI001661A3FF\_52\_332 | D | I | R | D | N | A | Q | F | - | - | Q | R | T | P | L | P | M | P | V | L | A | I | G | A | S | G | - | - | - | - | - | - | - | - | - | - | - | - | S | L | - | - | - | - | - | - | - | G | S | A |
| 099 UniRef90\_A0A6G4V4F1\_2\_276 | D | V | A | D | N | D | - | - | - | - | S | Y | P | P | L | E | M | P | V | L | A | I | G | G | D | - | - | - | - | - | - | - | - | - | - | - | - | - | - | - | - | - | - | - | - | - | - | - | - | F |
| 100 UniRef90\_A0A260W032\_16\_294 | D | V | A | Q | D | E | Q | D | - | - | L | D | T | L | I | Q | C | P | L | L | S | M | W | G | E | D | F | A | - | - | - | - | - | - | - | - | - | - | A | V | - | - | - | - | G | - | G | A | Y | D |
| 101 UniRef90\_UPI00036BBA85\_7\_295 | D | T | A | Q | D | M | E | D | - | - | A | H | V | K | I | K | C | P | I | L | T | M | W | G | A | D | F | D | - | - | - | - | - | - | - | - | - | - | L | V | - | - | - | - | G | - | Q | W | F | D |
| 102 UniRef90\_A0A4R5QBW7\_9\_278 | D | V | A | D | N | E | A | M | L | A | R | D | G | T | L | R | M | P | V | L | C | Y | G | G | P | L | - | - | - | - | - | - | - | - | - | - | - | - | G | R | - | - | - | - | G | - | R | G | L | G |
| 103 UniRef90\_UPI0012FB7881\_21\_292 | S | I | A | Q | N | T | E | R | - | - | A | R | T | P | L | P | V | P | V | L | A | V | G | G | S | R | - | - | - | - | - | - | - | - | - | - | - | - | S | R | - | - | - | - | - | - | - | G | A | A |
| 104 UniRef90\_UPI00131B39C2\_13\_285 | D | V | I | D | N | R | A | F | L | - | A | R | G | K | L | A | M | P | V | L | A | V | G | G | E | K | - | - | - | - | - | - | - | - | - | - | - | - | S | F | - | - | - | - | - | - | - | G | S | T |
| 105 UniRef90\_M2VKF7\_34\_311 | T | I | P | Q | V | R | V | H | - | - | M | A | N | K | V | Q | V | P | V | L | A | F | A | G | A | L | - | - | - | - | - | - | - | - | - | - | - | - | A | C | - | - | - | - | - | - | - | G | P | M |
| 106 UniRef90\_UPI0016124E74\_10\_283 | D | S | E | H | D | E | E | S | A | A | A | G | Q | R | L | T | M | P | V | L | A | L | W | G | S | A | - | - | - | - | - | - | - | - | - | - | - | - | G | L | - | - | - | - | P | - | G | R | L | P |
| 107 UniRef90\_A0A263DFR4\_27\_299 | T | V | R | Q | N | A | R | R | - | - | R | G | T | R | L | S | L | P | V | L | A | V | G | G | S | A | - | - | - | - | - | - | - | - | - | - | - | - | S | V | - | - | - | - | - | - | - | G | A | A |
| 108 UniRef90\_A0A0U5F3A6\_35\_313 | S | I | P | Q | Y | R | R | R | - | - | M | A | R | R | I | A | L | P | V | L | A | F | S | G | A | L | - | - | - | - | - | - | - | - | - | - | - | - | A | C | - | - | - | - | - | - | - | S | D | M |
| 109 UniRef90\_A0A2N3KZL1\_38\_324 | T | A | A | R | N | K | S | L | - | - | A | K | K | K | L | S | M | P | V | L | A | I | G | G | G | G | H | G | - | - | - | - | - | - | - | - | - | - | G | M | - | - | - | - | - | - | - | G | Q | L |
| 110 UniRef90\_A0A1X7C0M0\_25\_283 | D | G | R | E | N | R | A | L | - | L | A | R | R | R | L | P | M | P | V | L | A | I | G | S | T | H | - | - | - | - | - | - | - | - | - | - | - | - | S | - | - | - | - | - | - | - | - | G | T | A |
| 111 UniRef90\_A0A078M0V2\_4\_284 | D | L | P | E | Y | Q | E | K | L | E | S | A | D | K | L | N | I | D | T | L | L | L | W | G | E | K | - | - | - | - | - | - | - | - | - | - | - | - | G | N | - | - | - | - | L | - | S | G | L | P |
| 112 UniRef90\_A0A0F4Z6H8\_20\_271 | I | Q | Q | N | H | E | A | A | - | - | A | R | T | K | L | T | V | P | V | L | A | I | G | A | E | S | - | - | - | - | - | - | - | - | - | - | - | - | F | I | - | - | - | - | - | - | - | G | R | E |
| 113 UniRef90\_UPI00161D2319\_22\_292 | S | A | E | Q | I | L | R | W | - | R | D | E | G | P | L | S | I | P | V | L | A | I | G | G | Q | H | - | - | - | - | - | - | - | - | - | - | - | - | S | T | - | - | - | - | - | - | - | G | T | M |
| 114 UniRef90\_A0A1Q8LX67\_38\_327 | D | E | R | D | N | T | - | - | - | - | A | A | A | P | V | P | T | P | T | M | L | M | T | A | Q | G | - | - | - | - | - | - | - | - | - | - | - | - | L | L | - | - | - | - | - | - | - | E | S | T |
| 115 UniRef90\_A0A1Q9S2P6\_18\_309 | D | A | A | D | N | T | - | - | - | - | A | A | A | P | I | T | T | P | T | L | L | L | T | A | Q | G | - | - | - | - | - | - | - | - | - | - | - | - | L | L | - | - | - | - | - | - | - | E | P | T |
| 116 UniRef90\_A0A5M7BQA6\_8\_281 | D | S | E | H | D | D | A | D | A | A | A | G | R | K | L | T | M | P | V | L | A | L | W | G | S | A | - | - | - | - | - | - | - | - | - | - | - | - | G | L | - | - | - | - | P | - | A | K | L | P |
| 117 UniRef90\_A0A3N6GJS2\_33\_314 | D | I | A | D | L | G | - | - | - | - | T | Y | G | Q | V | T | A | P | M | L | G | L | V | S | N | L | - | - | - | - | - | - | - | - | - | - | - | - | A | D | - | - | - | - | G | M | F | A | K | Q |
| 118 UniRef90\_A0A0Q6MZD2\_36\_308 | D | A | V | D | N | R | A | F | V | - | A | N | N | R | L | K | M | P | L | L | A | L | G | G | E | K | - | - | - | - | - | - | - | - | - | - | - | - | S | F | - | - | - | - | - | - | - | G | L | Q |
| 119 UniRef90\_UPI00052520C0\_22\_293 | T | I | P | Q | N | V | E | R | - | - | A | R | T | P | L | A | I | P | V | L | A | V | G | G | A | G | - | - | - | - | - | - | - | - | - | - | - | - | S | R | - | - | - | - | - | - | - | G | A | S |
| 120 UniRef90\_A0A1Q4XCF8\_2\_276 | D | I | V | D | L | G | - | - | - | - | T | Y | G | K | I | G | A | P | V | L | G | L | V | S | N | L | - | - | - | - | - | - | - | - | - | - | - | - | A | D | - | - | - | - | G | L | F | A | A | Q |
| 121 UniRef90\_A0A328LZX6\_33\_305 | D | A | K | D | N | Q | A | F | L | - | A | Q | G | K | L | T | M | P | L | L | A | I | G | G | E | K | - | - | - | - | - | - | - | - | - | - | - | - | S | F | - | - | - | - | - | - | - | G | P | T |
| 122 UniRef90\_A0A2V8S6L4\_40\_311 | D | A | Q | D | F | A | Q | W | - | - | G | A | A | R | L | A | M | P | V | L | V | L | T | G | E | K | - | - | - | - | - | - | - | - | - | - | - | - | A | S | - | - | - | - | - | - | - | G | E | F |
| 123 UniRef90\_A0A1I5EB88\_8\_281 | D | S | E | H | D | D | A | D | A | E | A | G | R | K | L | T | M | P | V | L | A | L | W | G | S | A | - | - | - | - | - | - | - | - | - | - | - | - | G | L | - | - | - | - | P | - | A | K | L | P |
| 124 UniRef90\_UPI0014147ABD\_39\_305 | T | V | A | Q | N | Q | Q | R | - | - | R | N | Q | P | L | T | M | P | V | L | A | V | G | G | E | A | - | - | - | - | - | - | - | - | - | - | - | - | S | Y | - | - | - | - | - | - | - | G | P | H |
| 125 UniRef90\_A0A1I7DL13\_6\_274 | I | G | E | Q | L | K | E | L | - | - | S | A | Q | K | L | T | I | P | V | A | S | Y | G | G | A | M | - | - | - | - | - | - | - | - | - | - | - | - | V | M | - | - | - | - | - | - | - | A | D | Y |
| 126 UniRef90\_A0A3N7EDS3\_31\_305 | D | A | I | D | N | K | A | F | M | - | A | Q | G | K | L | E | M | P | V | L | A | L | G | G | E | K | - | - | - | - | - | - | - | - | - | - | - | - | S | F | - | - | - | - | - | - | - | G | S | T |
| 127 UniRef90\_UPI0016225F9B\_4\_275 | D | A | A | H | D | D | A | D | Y | D | A | G | K | R | L | E | M | P | V | L | A | L | W | G | E | A | - | - | - | - | - | - | - | - | - | - | - | - | G | L | - | - | - | - | P | - | A | R | V | G |
| 128 UniRef90\_UPI000997AE50\_107\_382 | D | E | I | D | N | K | R | L | - | - | Q | Q | K | P | L | T | I | P | V | R | M | I | S | E | E | G | - | - | - | - | - | - | - | - | - | - | - | - | - | - | - | - | - | - | - | - | F | L | D | V |
| 129 UniRef90\_A0A0N7F3H5\_6\_275 | D | H | D | H | D | E | A | D | - | - | A | G | R | K | L | T | M | P | V | L | A | M | W | G | E | A | - | - | - | - | - | - | - | - | - | - | - | - | G | L | - | - | - | - | L | - | G | K | L | P |
| 130 UniRef90\_A0A3Q8F441\_11\_281 | D | V | Q | Q | D | L | E | D | - | - | A | H | V | R | I | A | C | P | T | L | A | L | W | G | A | E | F | E | - | - | - | - | - | - | - | - | - | - | A | V | - | - | - | - | G | - | R | M | F | D |
| 131 UniRef90\_UPI001487D2FB\_23\_300 | D | A | E | D | N | R | K | A | L | - | A | G | K | K | L | R | M | P | V | L | A | I | G | G | A | K | - | - | - | - | - | - | - | - | - | - | - | - | S | F | - | - | - | - | - | - | - | G | A | N |
| 132 UniRef90\_A0A437GNC7\_38\_307 | D | I | P | A | N | R | E | R | - | - | M | K | T | R | L | T | M | P | V | L | G | F | A | G | E | L | - | - | - | - | - | - | - | - | - | - | - | - | A | C | - | - | - | - | - | - | - | A | G | A |
| 133 UniRef90\_A0A1H3SWJ7\_6\_283 | D | L | E | A | D | E | A | D | A | A | A | G | R | R | L | T | M | P | V | H | V | A | W | G | A | E | - | - | - | - | - | - | - | - | - | - | - | - | G | L | - | - | - | - | V | - | G | S | L | P |
| 134 UniRef90\_UPI0013DD6281\_49\_329 | D | E | R | D | N | T | - | - | - | - | A | A | A | P | I | A | T | P | T | M | L | M | T | A | E | G | - | - | - | - | - | - | - | - | - | - | - | - | Q | L | - | - | - | - | - | - | - | P | F | A |
| 135 UniRef90\_I0L7C1\_27\_272 | N | A | A | A | I | A | - | - | - | - | A | A | G | R | L | T | M | P | T | L | A | V | G | G | N | T | - | - | - | - | - | - | - | - | - | - | - | - | - | - | - | - | - | - | - | - | V | G | D | L |
| 136 UniRef90\_A0A260DLM1\_45\_326 | D | V | D | A | N | V | - | - | - | - | A | A | R | P | I | S | T | P | T | L | V | M | A | A | E | G | - | - | - | - | - | - | - | - | - | - | - | - | Q | A | - | - | - | - | - | - | - | E | S | V |
| 137 UniRef90\_UPI0005BDD2EC\_7\_280 | D | R | A | L | D | D | A | D | F | A | A | G | R | R | L | E | M | P | V | L | A | L | W | G | S | E | - | - | - | - | - | - | - | - | - | - | - | - | G | F | - | - | - | - | P | - | A | R | L | P |
| 138 UniRef90\_A0A6G9YBC1\_27\_296 | D | A | E | H | D | D | A | D | - | - | A | E | R | S | L | R | Q | P | V | L | A | L | W | G | S | G | - | - | - | - | - | - | - | - | - | - | - | - | S | F | - | - | - | - | L | - | E | K | L | P |
| 139 UniRef90\_UPI00145FA88A\_8\_280 | D | S | E | H | D | D | A | D | A | D | S | G | R | R | L | V | M | P | V | L | A | L | W | G | G | N | - | - | - | - | - | - | - | - | - | - | - | - | G | L | - | - | - | - | L | - | G | K | L | P |
| 140 UniRef90\_A0A2W0B1K8\_46\_299 | D | A | K | D | N | E | A | F | - | - | E | R | V | K | L | T | M | P | V | L | A | V | G | G | E | K | - | - | - | - | - | - | - | - | - | - | - | - | S | F | - | - | - | - | - | - | - | G | P | L |
| 141 UniRef90\_A0A5B8G622\_28\_299 | D | A | L | D | N | Q | A | F | L | - | A | E | G | K | L | A | M | P | V | L | A | V | G | G | D | H | - | - | - | - | - | - | - | - | - | - | - | - | S | F | - | - | - | - | - | - | - | G | S | G |
| 142 UniRef90\_A0A4S1XGQ8\_21\_302 | D | A | I | D | N | R | G | F | L | A | A | Q | G | K | L | G | M | P | V | L | A | L | G | G | E | K | - | - | - | - | - | - | - | - | - | - | - | - | S | F | - | - | - | - | - | - | - | A | G | T |
| 143 UniRef90\_A0A251YJ72\_8\_294 | E | G | A | E | L | R | A | R | - | - | A | E | V | P | L | A | I | P | A | L | A | V | G | G | S | G | - | - | - | - | - | - | - | - | - | - | - | - | - | - | - | - | - | - | - | - | - | G | P | F |
| 144 UniRef90\_UPI000A0527D2\_65\_318 | D | A | K | V | H | R | A | S | V | K | D | H | G | K | L | T | A | P | V | L | A | T | G | G | G | A | - | - | - | - | - | - | - | - | - | - | - | Q | S | L | - | - | - | - | - | - | - | A | A | N |
| 145 UniRef90\_A0A4V2S7H9\_8\_280 | D | A | A | H | D | D | E | D | A | A | A | G | R | R | L | T | M | P | V | L | A | L | W | G | A | T | - | - | - | - | - | - | - | - | - | - | - | - | G | L | - | - | - | - | L | - | G | S | L | P |
| 146 UniRef90\_UPI00131B216A\_18\_247 | D | I | E | D | E | K | - | - | - | - | G | Y | G | P | L | T | P | P | I | L | A | L | T | G | E | - | - | - | - | - | - | - | - | - | - | - | - | - | - | - | - | - | - | - | - | - | - | - | - | - |
| 147 UniRef90\_A0A1G8T9Y6\_5\_243 | D | I | A | D | N | A | R | Y | - | - | R | R | T | K | L | T | M | P | V | L | A | I | G | A | S | G | - | - | - | - | - | - | - | - | - | - | - | - | S | L | - | - | - | - | - | - | - | G | D | L |
| 148 UniRef90\_UPI00161F613F\_5\_254 | E | G | D | E | L | R | A | L | - | - | - | - | P | T | L | T | M | P | V | L | A | V | - | - | - | - | - | - | - | - | - | - | - | - | - | - | - | - | - | - | - | - | - | - | - | - | - | G | P | F |
| 149 UniRef90\_UPI00142071FA\_80\_362 | I | L | A | Q | N | M | E | R | - | - | Q | K | V | P | L | T | L | P | V | L | G | I | G | G | A | N | - | - | - | - | - | - | - | - | - | - | - | - | S | W | - | - | - | - | - | - | - | G | P | A |
| 150 UniRef90\_A0A6G7Q8F6\_7\_219 | - | - | - | - | - | - | - | - | - | - | - | - | - | - | - | - | L | P | - | - | - | - | - | - | - | - | - | - | - | - | - | - | - | - | - | - | - | - | - | - | - | - | - | - | - | - | - | - | - | - |

  
  

|  |  |  |  |  |  |  |  |  |  |  |  |  |  |  |  |  |  |  |  |  |  |  |  |  |  |  |  |  |  |  |  |  |  |  |  |  |  |  |  |  |  |  |  |  |  |  |  |
| --- | --- | --- | --- | --- | --- | --- | --- | --- | --- | --- | --- | --- | --- | --- | --- | --- | --- | --- | --- | --- | --- | --- | --- | --- | --- | --- | --- | --- | --- | --- | --- | --- | --- | --- | --- | --- | --- | --- | --- | --- | --- | --- | --- | --- | --- | --- | --- |
| **001 Input\_protein\_seq** | M | A | G | P | L | E | H | V | - | A | E | E | V | T | A | A | T | I | - | - | - | - | - | A | Y | S | G | H | F | I | P | E | E | Q | P | Q | A | L | A | R | E | L | R | D | F | F | R |
| 002 UniRef90\_UPI001430CB85\_4\_307 | M | A | G | P | L | R | A | Y | - | A | E | D | V | R | G | V | T | I | - | - | - | - | - | S | H | C | G | H | F | L | P | E | E | Q | S | T | A | V | A | G | E | L | L | E | F | F | - |
| 003 UniRef90\_UPI00055F8D66\_10\_306 | M | V | T | P | L | K | A | F | - | V | Q | D | V | R | G | G | A | I | - | - | - | - | - | S | Y | C | G | H | F | L | P | E | E | Q | P | E | V | V | A | E | E | L | A | D | F | F | - |
| 004 UniRef90\_UPI00161D8BA7\_15\_312 | M | A | S | P | L | R | A | Y | - | A | D | D | V | R | G | L | I | I | - | - | - | - | - | A | N | C | G | H | F | L | P | E | E | Q | P | K | A | V | A | E | E | L | I | A | F | F | - |
| 005 UniRef90\_A0A4R7GLU7\_7\_305 | M | A | L | P | L | R | A | F | - | A | E | N | V | T | G | I | I | I | - | - | - | - | - | A | H | S | G | H | F | I | P | E | E | Q | P | V | V | L | A | R | E | L | H | N | F | F | - |
| 006 UniRef90\_UPI00041CD164\_24\_309 | M | A | A | P | L | R | V | C | - | A | Q | D | V | R | G | M | R | I | - | - | - | - | - | A | R | C | G | H | F | L | P | E | E | Q | P | S | A | V | A | G | A | L | T | A | F | F | - |
| 007 UniRef90\_I0QSF8\_15\_298 | M | A | T | P | L | R | P | Y | - | F | E | N | V | E | G | V | M | I | - | - | - | - | - | E | N | S | G | H | F | L | Q | E | E | Q | P | E | A | I | A | K | E | F | R | N | F | F | K |
| 008 UniRef90\_A0A2N5ENH7\_9\_301 | M | A | V | P | L | R | R | F | - | A | D | D | V | T | G | I | T | I | - | - | - | - | - | S | H | S | G | H | F | I | P | D | E | Q | P | E | A | L | A | E | A | L | A | R | F | F | R |
| 009 UniRef90\_A0A0Q8E2M6\_29\_312 | M | A | A | P | L | R | A | F | - | A | D | D | V | H | G | V | R | I | - | - | - | - | - | A | D | C | G | H | F | M | P | E | E | Q | P | A | A | V | A | S | A | L | A | A | F | F | Q |
| 010 UniRef90\_A0A0F5XY46\_6\_287 | M | A | T | P | L | R | A | V | - | A | E | Q | V | S | G | A | V | I | - | - | - | - | - | G | E | C | G | H | F | I | P | D | E | Q | P | E | K | L | A | E | I | L | T | D | F | F | - |
| 011 UniRef90\_A0A3N4NUX5\_4\_284 | M | A | E | P | L | R | Q | F | - | A | R | H | V | Y | G | V | K | V | - | - | - | - | - | E | R | C | G | H | F | I | P | E | E | Q | P | A | V | L | A | K | H | F | S | A | F | F | - |
| 012 UniRef90\_UPI000DDED2D9\_11\_293 | L | V | A | Q | L | Q | K | I | - | A | T | D | V | T | G | T | N | I | - | - | - | - | - | E | Q | C | G | H | Y | L | A | E | E | Q | P | A | A | L | A | S | A | L | M | R | F | F | - |
| 013 UniRef90\_UPI000DDED53B\_10\_292 | L | A | G | A | I | R | P | F | - | A | A | R | L | E | G | E | V | I | - | - | - | - | - | A | N | C | G | H | F | Q | P | E | E | Q | P | D | A | V | A | D | A | L | T | G | F | F | - |
| 014 UniRef90\_A0A1G4JKM0\_6\_288 | M | A | K | S | L | E | A | F | - | S | N | Q | V | V | D | V | T | I | - | - | - | - | - | K | D | C | G | H | Y | I | T | E | E | Q | P | F | E | L | S | D | E | L | R | S | F | I | - |
| 015 UniRef90\_UPI0016728815\_21\_305 | M | A | A | S | V | R | P | W | - | A | D | H | A | T | G | V | V | V | - | - | - | - | - | P | D | A | G | H | F | I | P | D | E | Q | P | A | A | V | A | A | A | L | S | G | F | - | - |
| 016 UniRef90\_A0A3N2H8D2\_14\_297 | M | A | A | S | L | S | P | C | - | A | D | N | T | T | G | I | V | V | - | - | - | - | - | P | D | A | G | H | F | I | P | D | E | Q | P | D | A | V | A | D | A | L | T | G | F | - | - |
| 017 UniRef90\_UPI0005644DD1\_9\_307 | M | A | A | S | I | R | P | W | - | A | D | N | T | T | G | V | V | V | - | - | - | - | - | P | D | A | G | H | F | I | P | D | E | Q | P | E | A | V | A | A | A | I | A | G | F | - | - |
| 018 UniRef90\_A0A6B2VDS0\_18\_308 | M | A | A | S | L | S | P | W | - | A | D | N | I | T | S | V | I | V | - | - | - | - | - | P | D | A | G | H | F | I | P | D | E | Q | P | E | A | V | A | A | A | I | E | N | F | I | - |
| 019 UniRef90\_A0A6G3RZZ1\_25\_307 | M | A | A | S | L | G | P | W | - | A | D | T | V | T | G | A | V | I | - | - | - | - | - | P | R | A | G | H | F | I | P | D | E | Q | P | D | A | T | V | D | A | L | T | A | F | - | - |
| 020 UniRef90\_A0A209C7X3\_21\_305 | M | A | A | S | I | R | P | W | - | A | D | R | A | T | G | V | V | V | - | - | - | - | - | P | D | A | G | H | F | I | P | D | E | Q | P | A | A | V | V | A | A | L | T | A | F | - | - |
| 021 UniRef90\_UPI001269E708\_26\_309 | M | A | A | S | L | S | P | W | - | A | E | Q | V | T | G | V | V | V | - | - | - | - | - | P | D | A | G | H | F | I | P | D | E | Q | P | E | A | V | A | A | A | I | A | G | F | - | - |
| 022 UniRef90\_A0A2M9J7W9\_25\_306 | M | A | A | S | I | G | P | W | - | A | R | N | T | T | G | V | S | I | - | - | - | - | - | P | R | A | G | H | F | I | P | D | E | Q | P | A | A | T | V | D | A | L | T | A | - | - | - |
| 023 UniRef90\_A0A6G9F3J8\_23\_307 | M | A | S | S | L | R | P | W | - | A | G | N | A | A | G | V | V | V | - | - | - | - | - | P | D | A | G | H | F | V | P | D | E | Q | P | G | A | V | A | A | A | L | V | D | F | I | - |
| 024 UniRef90\_A0A1H2UZ41\_14\_292 | I | F | E | A | M | K | P | L | - | T | S | H | I | E | G | G | I | V | - | - | - | - | - | R | N | C | G | H | Y | M | P | E | E | Q | P | E | E | I | G | E | K | M | I | D | F | F | K |
| 025 UniRef90\_UPI0012B05B7D\_16\_286 | L | H | D | R | I | K | Q | L | - | A | I | D | V | Y | G | G | H | I | - | - | - | - | - | K | D | S | G | H | Y | I | P | E | E | Q | P | L | A | L | V | E | E | I | I | E | F | D | - |
| 026 UniRef90\_A0A5C8T429\_18\_294 | M | E | P | M | M | R | P | V | - | A | E | D | V | T | G | L | V | V | - | - | - | - | - | P | D | C | G | H | Y | V | P | E | E | A | P | N | F | L | A | E | H | L | I | N | F | - | - |
| 027 UniRef90\_UPI0012E86E3E\_10\_309 | L | V | D | T | M | R | L | V | - | A | T | D | V | D | G | G | V | F | - | - | - | - | - | E | G | C | G | H | Y | M | P | E | E | A | P | R | A | V | A | E | Q | I | M | R | F | - | - |
| 028 UniRef90\_A0A1A9HTL2\_13\_300 | M | E | K | T | M | R | L | V | - | A | T | D | V | Q | G | A | T | L | - | - | - | - | - | P | G | V | G | H | F | V | L | E | E | S | P | G | E | V | A | A | Q | L | N | A | F | M | R |
| 029 UniRef90\_A0A0R1V0C7\_7\_281 | L | F | E | K | L | K | T | L | - | G | K | S | V | N | G | R | T | I | - | - | - | - | - | K | E | S | G | H | Y | I | P | E | E | K | P | A | D | L | A | A | Q | I | L | E | F | I | - |
| 030 UniRef90\_A0A3N1M9Y1\_5\_282 | P | A | E | S | L | G | C | I | - | A | D | R | V | E | G | G | T | I | - | - | - | - | - | A | E | S | G | H | F | V | P | E | E | Q | P | D | A | L | S | A | R | L | M | E | F | W | R |
| 031 UniRef90\_A0A158JUX7\_17\_294 | A | E | R | T | M | Q | A | V | - | A | L | N | V | Q | G | A | I | I | - | - | - | - | - | S | N | S | G | H | Y | I | P | E | E | Q | P | L | A | L | V | R | L | L | L | D | F | F | - |
| 032 UniRef90\_A0A0U3LIL8\_12\_296 | L | F | Q | T | L | Q | G | I | - | A | S | N | V | C | G | Q | V | I | - | - | - | - | - | A | D | C | G | H | F | V | M | E | E | C | P | E | E | V | A | G | H | L | L | D | F | I | - |
| 033 UniRef90\_A0A248JS33\_7\_301 | L | A | D | T | L | R | G | H | - | A | A | D | V | R | A | Q | V | L | - | - | - | - | - | - | D | C | G | H | Y | V | P | E | E | A | P | D | E | L | A | A | V | L | V | D | F | L | - |
| 034 UniRef90\_A0A1Y0REZ4\_12\_287 | V | L | K | S | M | Q | Q | V | - | A | A | N | I | R | G | V | V | L | - | - | - | - | - | E | R | C | G | H | W | L | A | E | E | R | P | D | Y | L | V | E | L | L | L | S | F | F | - |
| 035 UniRef90\_A0A0M2WRU5\_3\_280 | P | L | L | T | M | Q | G | K | - | A | D | N | L | Q | G | A | I | V | - | - | - | - | - | A | E | C | G | H | F | I | M | E | E | Q | P | E | A | F | I | A | H | L | L | P | F | - | - |
| 036 UniRef90\_A0A0N8GFM1\_6\_279 | C | I | E | S | L | R | R | V | - | A | E | D | V | R | G | G | S | V | - | - | - | - | - | P | E | C | G | H | W | I | P | E | E | Q | P | E | F | L | A | E | A | L | M | Q | F | F | - |
| 037 UniRef90\_A0A252EMP1\_7\_277 | P | L | R | S | M | K | L | V | - | A | N | N | V | Q | G | G | V | I | - | - | - | - | - | P | D | C | G | H | W | V | P | D | E | K | P | E | W | I | A | R | E | I | N | R | F | - | - |
| 038 UniRef90\_W0A6K5\_40\_320 | P | Q | Q | T | M | Q | G | R | - | A | V | N | L | Q | G | A | M | L | - | - | - | - | - | S | E | C | G | H | F | V | T | E | E | C | P | E | Q | L | M | T | V | L | L | P | F | L | R |
| 039 UniRef90\_A0A3S1SM66\_23\_304 | I | L | D | T | F | R | P | L | - | A | R | D | V | S | G | R | T | I | - | - | - | - | - | L | N | C | G | H | Y | M | P | E | E | C | P | E | E | V | A | A | D | L | I | L | F | F | - |
| 040 UniRef90\_A0A2E5L5C6\_7\_286 | T | S | E | S | L | A | R | V | - | A | D | N | V | T | G | E | V | F | - | - | - | - | - | D | Y | C | G | H | F | I | P | E | E | A | P | S | R | L | N | D | S | L | A | R | F | F | - |
| 041 UniRef90\_M5D1B4\_14\_289 | P | M | L | T | L | Q | P | H | - | A | D | D | L | R | G | S | I | V | - | - | - | - | - | P | G | C | G | H | F | I | M | E | E | A | P | Q | A | F | L | E | Q | L | L | P | F | - | - |
| 042 UniRef90\_UPI0016845BA7\_31\_303 | P | L | I | Q | L | Q | T | V | - | A | K | N | I | R | G | G | T | M | - | - | - | - | - | Q | G | C | G | H | Y | I | P | D | E | C | A | E | A | L | T | E | E | L | L | S | F | - | - |
| 043 UniRef90\_UPI0014648199\_14\_291 | P | L | V | T | L | Q | G | H | - | A | S | D | L | R | G | A | I | I | - | - | - | - | - | A | D | C | G | H | F | V | M | E | E | A | P | E | A | F | L | R | H | L | L | G | F | L | R |
| 044 UniRef90\_K9RUM7\_12\_289 | I | Y | N | G | M | T | P | Y | - | C | E | N | L | T | S | Y | L | I | - | - | - | - | - | E | Q | C | G | H | Y | I | P | E | E | A | P | E | E | I | F | Q | A | I | S | D | T | F | - |
| 045 UniRef90\_A0A1Q2U086\_13\_290 | I | Y | S | G | L | E | P | Y | - | C | D | R | L | S | P | Y | L | I | - | - | - | - | - | D | G | C | G | H | Y | I | P | E | E | A | P | A | E | I | V | Q | A | I | I | E | T | F | - |
| 046 UniRef90\_B9JMN1\_3\_282 | T | K | Q | C | L | D | M | A | - | A | T | D | V | T | G | G | V | I | - | - | - | - | - | E | R | C | G | H | W | I | G | E | E | R | P | D | F | V | A | S | Q | L | V | E | F | F | - |
| 047 UniRef90\_A0A2X1TAZ7\_33\_319 | P | Q | L | T | M | Q | G | R | - | A | A | D | L | Q | G | A | M | L | - | - | - | - | - | S | E | C | G | H | F | V | T | E | E | C | P | E | Q | L | M | G | V | L | L | P | F | L | R |
| 048 UniRef90\_UPI00131A8152\_6\_279 | V | A | E | S | A | R | R | M | - | A | D | N | V | T | D | A | I | I | - | - | - | - | - | E | D | A | G | H | W | I | P | E | E | Q | P | A | E | L | A | D | L | I | D | S | F | - | - |
| 049 UniRef90\_F7YB04\_47\_316 | Y | E | G | H | L | R | H | V | - | A | E | N | V | R | A | V | V | V | - | - | - | - | - | E | G | S | G | H | W | V | P | E | E | Q | P | A | A | V | T | R | A | L | I | E | F | - | - |
| 050 UniRef90\_A0A1A3M1Z8\_5\_294 | A | E | E | M | L | A | E | V | - | A | E | N | F | T | V | R | P | I | - | - | - | - | - | E | A | S | G | H | W | I | A | E | E | Q | P | E | T | L | L | L | R | L | R | E | F | I | - |
| 051 UniRef90\_UPI0004C32CE1\_32\_312 | F | R | A | A | L | P | N | L | - | A | T | D | F | K | A | V | Q | I | - | - | - | - | - | D | G | A | G | H | W | L | A | E | E | Q | P | D | A | V | A | R | E | L | T | Q | F | F | - |
| 052 UniRef90\_A0A0J6NQJ9\_12\_287 | P | L | D | T | M | R | G | H | - | A | D | D | L | R | G | V | V | I | - | - | - | - | - | P | D | C | G | H | F | I | M | E | E | A | A | E | E | F | I | A | E | L | L | P | F | - | - |
| 053 UniRef90\_A0A534ESC2\_32\_321 | L | R | A | T | V | A | T | I | - | G | D | R | V | K | G | G | A | V | K | G | S | P | A | E | G | C | G | H | F | L | P | E | E | C | P | N | E | L | A | A | A | I | L | G | F | W | R |
| 054 UniRef90\_A0A1H1Z302\_21\_301 | V | A | E | T | M | R | L | A | - | A | D | D | V | T | G | V | V | I | - | - | - | - | - | D | D | C | G | H | Y | A | A | E | E | Q | P | A | R | F | T | E | V | L | E | D | F | L | - |
| 055 UniRef90\_A0A158DLE6\_24\_295 | S | K | A | T | M | E | R | V | - | A | S | N | V | R | G | V | E | L | - | - | - | - | - | S | E | S | G | H | Y | P | A | E | E | Q | P | Q | Q | L | A | S | V | L | L | D | F | L | - |
| 056 UniRef90\_A0A163VVR8\_16\_280 | P | L | T | T | L | R | G | N | - | A | H | D | L | R | G | E | T | V | - | - | - | - | - | A | G | C | G | H | F | I | T | E | E | C | H | E | - | - | - | - | - | - | - | - | - | - | - |
| 057 UniRef90\_UPI000369F0DF\_7\_289 | P | I | E | T | L | R | P | H | - | A | T | D | L | R | G | E | I | I | - | - | - | - | - | R | D | C | G | H | F | V | M | E | E | A | P | R | E | F | N | A | Q | L | I | P | F | F | - |
| 058 UniRef90\_UPI00098F4FB4\_43\_309 | Y | E | G | H | I | R | N | V | - | A | K | N | V | R | G | V | V | V | - | - | - | - | - | E | G | S | G | H | W | I | P | E | E | K | P | Q | D | V | I | D | E | L | - | - | - | - | - |
| 059 UniRef90\_UPI0015A1AD76\_10\_292 | I | A | Q | F | L | A | T | V | - | A | D | D | V | R | P | L | V | L | - | - | - | - | - | E | G | A | G | H | W | L | V | E | E | Q | P | A | A | L | T | R | E | L | L | D | F | F | - |
| 060 UniRef90\_UPI001689F051\_63\_318 | W | I | D | T | V | Q | E | V | - | A | T | H | V | S | G | H | L | I | - | - | - | - | - | E | E | C | G | H | Y | V | P | E | E | Q | P | G | Q | L | A | D | L | L | - | - | - | - | - |
| 061 UniRef90\_UPI000DD77D66\_43\_313 | Y | G | E | H | I | R | H | I | - | A | K | N | V | R | A | L | V | V | - | - | - | - | - | E | G | A | G | H | W | I | P | E | E | Q | P | A | A | V | T | A | A | L | I | D | F | L | - |
| 062 UniRef90\_A0A3M0I8T4\_31\_307 | V | A | T | T | M | R | L | A | - | A | E | H | V | D | S | L | I | I | - | - | - | - | - | P | D | C | G | H | Y | P | A | E | E | A | P | E | E | M | L | A | A | L | S | D | F | L | - |
| 063 UniRef90\_UPI000377E2D1\_4\_271 | V | E | E | S | L | R | R | M | - | A | H | N | V | T | G | H | V | L | - | - | - | - | - | D | D | C | G | H | W | V | P | E | E | K | P | R | E | L | A | E | - | - | - | - | - | - | - |
| 064 UniRef90\_UPI0016198B53\_15\_286 | L | G | A | L | L | A | P | V | - | T | E | R | L | S | T | A | V | L | - | - | - | - | - | P | G | C | G | H | Y | V | P | E | E | S | P | D | A | L | L | G | H | L | L | P | F | L | R |
| 065 UniRef90\_UPI0013C0217D\_48\_325 | F | Q | Q | V | L | P | A | L | - | G | N | D | V | R | V | V | K | L | - | - | - | - | - | A | N | T | V | H | Y | L | P | E | E | A | P | D | R | L | A | R | T | L | T | D | F | F | - |
| 066 UniRef90\_C7PGN2\_5\_260 | A | Y | N | I | T | K | P | L | - | V | A | D | I | T | G | A | V | I | - | - | - | - | - | P | D | C | G | H | Y | I | Q | E | E | Q | P | D | F | L | V | K | E | L | I | Q | F | F | - |
| 067 UniRef90\_A0A1F7Q7Q2\_7\_275 | M | L | G | V | W | R | A | R | C | A | G | P | V | T | G | H | A | V | - | - | - | - | - | P | D | S | G | H | F | I | P | E | E | Q | P | Q | A | V | V | D | - | - | - | - | - | - | - |
| 068 UniRef90\_A0A2V7F1K9\_9\_274 | M | V | G | V | W | K | A | R | C | A | S | T | V | A | G | H | P | V | - | - | - | - | - | S | G | S | G | H | F | I | P | E | E | Q | P | Q | A | V | L | D | - | - | - | - | - | - | - |
| 069 UniRef90\_UPI001616C592\_31\_308 | V | A | A | E | M | R | V | G | - | A | T | D | V | T | G | V | V | L | - | - | - | - | - | - | D | G | G | H | Y | I | S | E | E | A | P | E | A | F | L | A | A | V | E | T | F | L | R |
| 070 UniRef90\_UPI001456849A\_71\_360 | L | G | R | Q | L | Q | T | V | - | A | T | D | L | R | G | E | S | M | - | - | - | - | - | A | G | C | G | H | W | L | A | S | E | C | P | E | R | T | E | K | H | L | S | L | F | F | - |
| 071 UniRef90\_A0A2S8J6S6\_18\_282 | I | V | D | M | V | S | E | F | - | G | S | N | V | T | G | G | S | I | - | - | - | - | - | P | D | C | G | H | W | V | V | E | E | R | P | N | A | V | L | V | A | L | A | N | F | - | - |
| 072 UniRef90\_A0A2I2GC12\_18\_280 | M | V | R | L | A | Q | N | L | - | A | S | N | V | Q | G | G | E | I | - | - | - | - | - | E | D | C | G | H | F | V | V | E | E | K | P | D | D | V | A | E | K | L | M | G | F | F | K |
| 073 UniRef90\_A0A549T469\_41\_312 | L | A | E | A | L | R | P | K | - | A | R | A | L | S | S | I | I | V | - | - | - | - | - | E | E | S | G | H | F | V | A | D | E | A | P | E | V | F | W | R | E | A | E | R | F | - | - |
| 074 UniRef90\_A0A5C4LJC6\_5\_278 | M | V | P | M | L | Q | E | F | - | A | G | T | V | T | G | G | A | I | - | - | - | - | - | A | R | C | G | H | Y | V | A | D | E | R | P | H | E | V | A | A | A | L | I | A | F | - | - |
| 075 UniRef90\_UPI001619D806\_9\_283 | S | A | D | I | W | R | G | Y | - | A | D | H | L | T | A | A | E | I | - | - | - | - | - | S | E | C | G | H | F | L | P | E | E | Q | P | D | V | V | A | G | Q | L | R | D | F | F | - |
| 076 UniRef90\_A0A239P1A5\_69\_347 | M | E | T | E | M | R | A | V | - | A | E | N | V | R | S | V | L | I | - | - | - | - | - | P | K | A | G | H | F | L | M | D | E | N | P | T | Q | V | I | D | A | L | K | P | F | - | - |
| 077 UniRef90\_UPI0005262A70\_5\_280 | T | S | G | T | M | A | Q | V | - | A | D | R | V | T | S | I | R | L | - | - | - | - | - | D | G | V | G | H | L | V | A | L | E | A | P | D | T | L | A | K | E | L | L | T | F | Y | R |
| 078 UniRef90\_A0A241XUQ6\_27\_314 | Q | L | E | Q | M | K | A | Y | - | A | D | D | V | E | G | H | V | L | - | - | - | - | - | P | G | C | G | H | W | L | P | E | E | C | A | A | P | M | N | R | L | V | I | D | F | - | - |
| 079 UniRef90\_A0A160FQ65\_32\_312 | V | E | A | E | L | R | T | V | - | A | E | D | V | Q | S | A | I | I | - | - | - | - | - | A | G | A | G | H | Y | P | A | E | E | K | P | E | A | T | L | K | S | L | Q | D | F | F | - |
| 080 UniRef90\_UPI0006AE4C23\_4\_281 | S | R | A | S | N | E | H | R | - | A | T | D | I | R | F | V | E | I | - | - | - | - | - | P | G | A | G | H | Y | L | S | E | E | R | P | E | Q | L | S | R | E | L | I | T | F | F | - |
| 081 UniRef90\_T0H3T7\_11\_281 | M | P | K | V | W | A | E | M | - | A | D | N | L | K | A | V | A | I | - | - | - | - | - | P | R | C | G | H | L | P | H | E | E | R | P | E | A | V | N | A | L | L | L | E | F | - | - |
| 082 UniRef90\_A0A4R2IH15\_5\_277 | V | E | R | T | M | R | L | A | - | A | D | D | V | R | A | M | V | L | - | - | - | - | - | P | D | C | G | H | Y | P | A | E | E | Q | P | E | L | M | V | K | A | L | I | D | F | L | - |
| 083 UniRef90\_UPI00140B863E\_4\_276 | V | E | A | S | I | G | Q | A | - | A | S | D | V | T | G | V | V | L | - | - | - | - | - | P | D | C | G | H | Y | P | A | E | E | H | P | Q | R | F | A | A | L | V | A | D | F | I | - |
| 084 UniRef90\_A0A3R9UXB7\_22\_297 | L | R | A | A | V | P | A | H | - | G | T | D | T | R | V | V | R | I | - | - | - | - | - | D | D | C | G | H | Y | L | P | E | E | Q | P | E | A | V | L | A | E | L | L | P | F | L | R |
| 085 UniRef90\_UPI000F8D1465\_20\_303 | V | G | E | Q | M | K | L | V | - | A | R | D | V | T | A | L | T | L | - | - | - | - | - | E | G | C | G | H | Y | V | P | E | E | A | P | D | A | T | L | A | A | L | L | P | F | - | - |
| 086 UniRef90\_A0A6J4HYT7\_23\_307 | L | A | L | S | L | P | D | R | - | A | T | H | P | K | L | V | Q | V | - | - | - | - | - | E | G | S | G | H | F | I | A | E | E | Q | P | H | Q | T | A | R | L | M | I | D | F | L | - |
| 087 UniRef90\_A0A1D8SMR3\_19\_296 | V | E | Q | A | V | R | Q | V | - | A | S | D | T | S | G | A | I | A | - | - | - | - | - | P | G | A | A | H | F | L | P | E | E | A | P | E | W | M | A | R | T | L | I | D | F | F | - |
| 088 UniRef90\_A0A4V2HUZ4\_48\_320 | L | A | E | S | L | R | G | Q | - | A | A | D | L | T | S | V | V | A | - | - | - | - | - | E | D | S | G | H | F | V | A | E | E | V | P | D | F | F | C | E | K | L | E | T | F | - | - |
| 089 UniRef90\_UPI0010582A1B\_5\_279 | M | V | P | M | L | K | E | F | - | A | T | S | V | T | G | G | S | I | - | - | - | - | - | M | R | C | S | H | Y | V | A | D | E | R | P | D | E | V | A | A | A | L | I | E | F | V | - |
| 090 UniRef90\_UPI00166CE541\_54\_338 | V | P | T | Q | V | R | R | Y | - | A | R | D | V | T | G | I | I | I | - | - | - | - | - | E | N | S | G | H | W | I | F | E | E | Q | P | E | Q | L | T | R | T | L | L | R | F | - | - |
| 091 UniRef90\_A0A5C1HX54\_34\_307 | L | A | D | H | S | K | L | V | - | A | E | N | V | K | E | V | I | I | - | - | - | - | - | H | D | S | G | H | W | I | V | Q | E | Q | T | A | Q | V | Q | K | G | L | L | D | F | F | - |
| 092 UniRef90\_A0A4D4KET2\_34\_306 | V | G | N | T | M | R | L | A | - | A | D | D | V | E | S | H | I | L | - | - | - | - | - | P | G | C | G | H | Y | P | A | E | E | V | P | E | A | M | L | A | A | L | T | V | F | - | - |
| 093 UniRef90\_A0A1I1XP17\_9\_276 | M | R | P | M | V | Q | Q | L | - | A | G | H | A | R | Y | L | A | A | - | - | - | - | - | P | G | C | A | H | W | V | P | E | E | N | P | T | F | L | C | - | - | - | - | - | - | - | - |
| 094 UniRef90\_A0A1D7VX60\_23\_299 | V | A | D | T | M | K | R | V | - | A | H | D | V | R | S | V | V | I | - | - | - | - | - | P | G | T | G | H | W | V | A | E | E | A | P | Q | E | V | L | A | A | L | T | T | F | - | - |
| 095 UniRef90\_A0A2T4ZAU7\_6\_296 | V | L | D | I | W | N | Q | V | - | A | T | T | V | T | G | R | E | I | - | - | - | - | - | A | N | C | G | H | Y | V | P | E | E | Q | P | A | V | V | V | R | E | I | M | P | F | - | - |
| 096 UniRef90\_UPI00143A9489\_23\_300 | M | G | A | V | A | R | G | A | - | A | T | N | V | E | E | L | I | I | - | - | - | - | - | P | N | S | G | H | W | L | M | E | E | Q | P | E | A | T | V | A | A | I | R | A | F | - | - |
| 097 UniRef90\_A0A7C7NVI2\_6\_287 | L | E | D | A | W | R | G | V | - | F | P | N | V | Q | G | K | D | M | - | - | - | - | - | G | N | Y | G | H | F | L | Q | W | E | A | P | E | D | T | N | R | E | I | M | D | F | F | - |
| 098 UniRef90\_UPI001661A3FF\_52\_332 | E | G | E | Q | M | K | A | Y | - | A | S | H | V | T | G | V | T | I | - | - | - | - | - | P | N | S | G | H | W | I | Y | E | E | R | P | E | E | L | T | A | L | L | L | D | F | L | - |
| 099 UniRef90\_A0A6G4V4F1\_2\_276 | M | R | P | M | M | E | D | K | - | A | N | D | I | R | Y | S | G | I | - | - | - | - | - | E | D | S | G | H | F | V | A | E | E | Q | P | E | A | L | A | A | E | L | E | K | F | F | - |
| 100 UniRef90\_A0A260W032\_16\_294 | V | A | A | I | W | Q | S | M | - | G | T | N | V | R | T | V | A | I | - | - | - | - | - | P | R | C | G | H | L | C | H | E | E | R | P | D | V | V | N | H | E | L | L | E | F | L | - |
| 101 UniRef90\_UPI00036BBA85\_7\_295 | V | E | A | V | W | K | T | M | - | G | D | D | V | K | A | V | P | L | - | - | - | - | - | P | E | C | G | H | L | C | Q | E | E | R | P | D | L | V | N | A | E | L | L | E | F | - | - |
| 102 UniRef90\_A0A4R5QBW7\_9\_278 | A | I | E | S | W | R | R | V | - | A | E | D | V | R | G | G | I | A | - | - | - | - | - | E | G | C | G | H | W | I | P | E | E | R | P | D | W | V | T | A | Q | L | L | E | F | F | - |
| 103 UniRef90\_UPI0012FB7881\_21\_292 | V | A | A | D | V | R | R | I | - | A | T | E | V | T | E | L | V | L | - | - | - | - | - | E | D | C | G | H | Y | V | A | E | E | A | P | D | A | F | S | T | A | V | L | A | F | F | - |
| 104 UniRef90\_UPI00131B39C2\_13\_285 | M | A | V | V | M | R | A | A | - | A | T | D | V | D | E | L | V | I | - | - | - | - | - | A | A | S | G | H | W | L | M | E | E | Q | P | M | A | T | V | A | A | V | R | A | F | - | - |
| 105 UniRef90\_M2VKF7\_34\_311 | V | E | N | E | Q | R | N | L | - | A | S | D | V | Q | A | T | I | I | - | - | - | - | - | P | D | C | G | H | F | P | A | E | E | Q | P | E | A | L | L | A | I | L | L | D | F | F | K |
| 106 UniRef90\_UPI0016124E74\_10\_283 | M | L | D | I | W | R | E | Y | - | A | E | Q | V | T | G | I | A | I | - | - | - | - | - | E | E | C | G | H | F | L | P | E | E | R | P | E | A | V | L | G | H | L | R | D | F | L | - |
| 107 UniRef90\_A0A263DFR4\_27\_299 | V | G | A | T | M | S | A | V | - | A | D | D | V | T | A | R | V | L | - | - | - | - | - | E | G | C | G | H | F | P | A | E | E | Q | P | E | E | F | L | A | E | L | L | P | F | L | R |
| 108 UniRef90\_A0A0U5F3A6\_35\_313 | V | E | Q | E | L | R | S | V | - | A | D | D | V | Q | S | V | I | I | - | - | - | - | - | P | D | S | G | H | Y | P | A | E | E | Q | P | E | V | L | L | A | A | L | Q | A | F | - | - |
| 109 UniRef90\_A0A2N3KZL1\_38\_324 | E | A | D | Q | L | G | E | Y | - | G | T | H | V | K | G | L | V | I | - | - | - | - | - | P | D | C | G | H | W | L | P | E | E | C | A | K | P | L | N | D | A | V | L | D | F | - | - |
| 110 UniRef90\_A0A1X7C0M0\_25\_283 | T | A | Q | A | L | T | P | H | - | A | D | D | V | Q | G | V | V | - | - | - | - | - | - | A | P | T | G | H | F | V | A | E | E | D | P | D | W | F | T | K | T | V | T | T | - | - | - |
| 111 UniRef90\_A0A078M0V2\_4\_284 | V | E | D | I | W | Q | Q | S | - | I | N | N | V | T | G | Y | E | L | - | - | - | - | - | K | S | C | G | H | Y | L | L | E | E | K | G | E | E | V | A | Q | H | I | L | E | F | - | - |
| 112 UniRef90\_A0A0F4Z6H8\_20\_271 | V | K | R | Q | M | E | R | V | - | A | Q | N | V | Q | Y | A | E | F | - | - | - | - | - | - | D | Y | G | H | Q | L | A | E | E | C | P | E | M | L | S | Q | R | Y | L | S | F | - | - |
| 113 UniRef90\_UPI00161D2319\_22\_292 | P | E | E | T | M | R | L | V | - | A | T | D | V | T | G | L | V | I | - | - | - | - | - | P | G | A | G | H | F | L | P | E | E | A | P | E | D | W | A | R | R | - | - | - | - | - | - |
| 114 UniRef90\_A0A1Q8LX67\_38\_327 | R | P | S | V | E | S | R | L | - | P | N | L | V | R | A | V | E | V | - | - | - | - | - | P | G | S | G | H | W | L | P | E | E | N | P | G | F | V | L | A | E | L | L | A | F | - | - |
| 115 UniRef90\_A0A1Q9S2P6\_18\_309 | R | A | A | I | A | P | R | A | - | T | T | I | V | H | A | A | E | V | - | - | - | - | - | P | G | A | G | H | W | L | M | E | E | N | P | G | F | V | T | T | E | L | I | R | F | - | - |
| 116 UniRef90\_A0A5M7BQA6\_8\_281 | M | R | D | I | W | R | E | Y | - | A | E | D | V | S | C | A | E | I | - | - | - | - | - | A | D | C | G | H | F | L | P | E | E | R | P | E | Q | V | L | A | H | L | R | E | F | L | - |
| 117 UniRef90\_A0A3N6GJS2\_33\_314 | M | E | A | T | L | P | T | Q | - | G | S | D | V | R | V | V | L | V | - | - | - | - | - | P | D | A | G | H | Y | F | V | E | E | A | P | Q | A | V | I | D | A | F | N | A | F | F | - |
| 118 UniRef90\_A0A0Q6MZD2\_36\_308 | M | A | A | V | M | R | A | G | - | A | E | D | V | T | E | G | V | V | - | - | - | - | - | P | G | S | G | H | W | I | M | E | E | N | P | S | A | T | G | K | L | V | R | D | F | I | - |
| 119 UniRef90\_UPI00052520C0\_22\_293 | V | A | A | D | V | R | K | V | - | A | G | Q | V | T | E | L | V | I | - | - | - | - | - | D | D | C | G | H | Y | V | A | E | E | A | P | E | N | F | T | A | A | I | L | D | F | L | - |
| 120 UniRef90\_A0A1Q4XCF8\_2\_276 | M | Q | A | T | L | P | T | Q | - | A | G | D | V | R | V | V | V | V | - | - | - | - | - | P | D | A | G | H | Y | F | V | E | E | A | P | Q | A | V | I | D | E | F | N | A | F | F | - |
| 121 UniRef90\_A0A328LZX6\_33\_305 | M | A | V | V | M | R | A | T | - | A | T | N | V | T | E | R | V | I | - | - | - | - | - | P | D | A | G | H | W | L | M | E | E | Q | P | K | A | T | I | A | A | V | R | D | F | - | - |
| 122 UniRef90\_A0A2V8S6L4\_40\_311 | L | I | E | Q | A | R | L | V | - | A | T | D | V | Q | G | V | V | I | - | - | - | - | - | K | G | S | G | H | W | L | M | E | E | A | P | D | Q | V | I | P | Q | L | V | - | - | - | - |
| 123 UniRef90\_A0A1I5EB88\_8\_281 | M | R | E | I | W | R | D | Y | - | A | E | H | P | T | C | A | E | I | - | - | - | - | - | A | D | C | G | H | F | L | A | E | E | R | P | E | Q | V | L | A | H | L | R | E | F | L | - |
| 124 UniRef90\_UPI0014147ABD\_39\_305 | V | A | E | A | M | E | P | L | - | A | D | D | V | R | G | E | V | I | - | - | - | - | - | A | G | T | G | H | W | L | A | E | Q | S | P | D | Q | L | L | A | A | L | T | A | F | - | - |
| 125 UniRef90\_A0A1I7DL13\_6\_274 | S | G | N | A | A | K | L | V | - | A | E | K | V | D | A | G | I | V | - | - | - | - | - | E | N | C | G | H | W | V | P | E | E | R | P | E | F | V | A | D | L | I | R | - | - | - | - |
| 126 UniRef90\_A0A3N7EDS3\_31\_305 | M | A | T | V | M | R | F | A | - | A | S | D | V | T | E | G | I | V | - | - | - | - | - | P | D | S | G | H | W | I | M | E | E | N | P | K | A | T | T | Q | L | V | G | D | F | L | R |
| 127 UniRef90\_UPI0016225F9B\_4\_275 | V | E | E | V | W | R | D | Y | - | A | H | D | V | R | G | A | E | I | - | - | - | - | - | P | D | C | G | H | F | L | A | E | E | R | P | D | L | V | L | D | H | L | R | D | F | L | - |
| 128 UniRef90\_UPI000997AE50\_107\_382 | M | L | A | G | A | Q | G | A | - | S | P | S | A | T | G | V | E | V | - | - | - | - | - | A | G | S | G | H | W | L | A | E | E | A | P | R | R | I | I | E | E | I | N | A | F | Y | - |
| 129 UniRef90\_A0A0N7F3H5\_6\_275 | T | A | D | I | W | R | A | Y | - | A | E | D | V | T | A | Q | A | I | - | - | - | - | - | T | E | C | G | H | F | L | A | E | E | Q | P | S | T | V | A | G | H | L | V | R | F | - | - |
| 130 UniRef90\_A0A3Q8F441\_11\_281 | M | P | Q | V | W | A | E | M | - | A | E | N | L | R | A | V | A | I | - | - | - | - | - | E | R | C | G | H | L | P | H | E | E | R | P | E | V | V | N | G | L | L | M | E | F | - | - |
| 131 UniRef90\_UPI001487D2FB\_23\_300 | E | A | M | V | M | R | H | A | - | A | T | D | V | T | E | V | V | I | - | - | - | - | - | P | D | A | G | H | W | L | M | E | E | A | P | A | A | T | I | A | A | I | R | A | F | - | - |
| 132 UniRef90\_A0A437GNC7\_38\_307 | V | E | E | Q | L | R | Q | V | - | A | D | D | V | R | C | T | I | I | - | - | - | - | - | P | E | C | G | H | F | V | P | E | E | T | P | G | A | L | L | A | L | L | R | P | F | L | - |
| 133 UniRef90\_A0A1H3SWJ7\_6\_283 | V | L | D | L | W | R | D | Y | - | A | T | R | L | E | G | A | Q | I | - | - | - | - | - | A | S | C | G | H | F | P | A | E | E | Q | P | D | V | L | V | A | R | I | R | A | F | - | - |
| 134 UniRef90\_UPI0013DD6281\_49\_329 | R | S | T | V | E | P | R | M | - | S | R | I | T | R | A | V | E | V | - | - | - | - | - | P | K | A | G | H | W | L | T | E | E | N | P | K | F | V | T | A | E | L | L | A | F | - | - |
| 135 UniRef90\_I0L7C1\_27\_272 | L | N | R | Q | L | T | P | I | - | A | E | D | L | S | G | E | L | I | - | - | - | - | - | A | R | S | G | H | L | V | P | L | D | R | P | D | A | V | A | R | L | L | R | - | - | - | - |
| 136 UniRef90\_A0A260DLM1\_45\_326 | R | A | T | L | T | S | V | V | - | T | T | I | E | H | S | V | D | V | - | - | - | - | - | P | H | S | G | H | W | L | V | E | E | N | P | E | F | V | T | R | T | L | S | G | F | - | - |
| 137 UniRef90\_UPI0005BDD2EC\_7\_280 | A | L | D | I | W | R | E | Y | - | A | K | D | V | M | G | A | Q | I | - | - | - | - | - | P | D | C | G | H | F | I | A | E | E | R | P | G | E | V | L | A | H | V | R | K | F | L | - |
| 138 UniRef90\_A0A6G9YBC1\_27\_296 | A | L | D | I | W | R | K | Y | - | A | D | D | V | Q | G | G | P | I | - | - | - | - | - | P | D | C | G | H | F | I | A | E | E | R | P | E | V | L | V | D | R | L | R | A | F | - | - |
| 139 UniRef90\_UPI00145FA88A\_8\_280 | A | L | E | I | W | Q | D | Y | - | A | D | D | V | R | G | W | A | I | - | - | - | - | - | A | D | C | G | H | F | L | P | E | E | Q | P | G | E | T | L | R | A | L | Q | G | F | L | - |
| 140 UniRef90\_A0A2W0B1K8\_46\_299 | Q | A | V | I | M | R | H | V | - | A | T | N | V | Q | E | A | V | V | - | - | - | - | - | N | G | S | G | H | W | L | M | E | E | R | P | A | Y | T | V | T | L | I | R | N | F | - | - |
| 141 UniRef90\_A0A5B8G622\_28\_299 | M | A | D | V | V | R | F | A | - | A | T | D | V | Q | G | L | V | I | - | - | - | - | - | G | D | A | G | H | W | L | M | E | E | Q | P | D | A | T | V | A | A | V | R | A | F | - | - |
| 142 UniRef90\_A0A4S1XGQ8\_21\_302 | M | A | T | V | M | R | A | A | - | A | T | N | V | T | Q | G | I | V | - | - | - | - | - | P | A | S | G | H | W | I | M | E | E | N | P | D | A | T | V | K | L | V | T | D | F | L | - |
| 143 UniRef90\_A0A251YJ72\_8\_294 | T | V | A | T | L | E | G | V | M | A | G | P | V | A | S | V | Q | L | - | - | - | - | - | D | G | V | G | H | H | V | A | L | E | A | P | G | P | L | A | D | A | V | L | A | F | L | - |
| 144 UniRef90\_UPI000A0527D2\_65\_318 | Y | G | P | M | C | R | D | V | - | A | E | N | V | T | T | E | L | I | - | - | - | - | - | P | D | A | G | H | W | V | P | E | E | Q | P | E | A | L | I | - | - | - | - | - | - | - | - |
| 145 UniRef90\_A0A4V2S7H9\_8\_280 | T | L | E | I | W | R | D | Y | - | G | T | S | V | T | G | E | A | I | - | - | - | - | - | S | E | C | G | H | F | L | A | E | E | Q | P | A | A | V | L | D | R | L | L | P | F | - | - |
| 146 UniRef90\_UPI00131B216A\_18\_247 | - | - | - | - | - | - | - | - | - | - | - | - | - | - | - | - | - | - | - | - | - | - | - | - | - | - | - | - | - | - | - | - | - | - | - | - | - | - | - | - | - | - | - | - | - | - | - |
| 147 UniRef90\_A0A1G8T9Y6\_5\_243 | V | P | T | Q | V | R | S | Y | - | A | T | H | V | T | G | L | V | I | - | - | - | - | - | A | D | S | G | H | W | I | Y | E | E | H | P | A | E | L | T | R | R | L | L | A | F | - | - |
| 148 UniRef90\_UPI00161F613F\_5\_254 | A | V | A | T | M | R | Q | V | - | A | Q | Q | V | T | A | V | E | L | - | - | - | - | - | D | G | V | G | H | Y | V | A | L | E | A | P | E | R | L | A | H | A | V | L | D | F | I | R |
| 149 UniRef90\_UPI00142071FA\_80\_362 | A | A | A | G | M | T | P | A | - | A | S | D | V | Q | A | A | V | I | - | - | - | - | - | A | G | A | G | H | W | V | A | E | Q | A | P | A | Q | M | L | E | L | L | R | A | F | - | - |
| 150 UniRef90\_A0A6G7Q8F6\_7\_219 | - | - | - | - | - | - | - | - | - | - | - | - | - | - | - | - | - | - | - | - | - | - | - | - | - | - | - | - | - | - | - | - | - | - | - | - | - | - | - | - | - | - | - | - | - | - | - |

  
  

|  |  |  |  |  |  |  |  |  |  |  |  |  |  |  |  |  |  |
| --- | --- | --- | --- | --- | --- | --- | --- | --- | --- | --- | --- | --- | --- | --- | --- | --- | --- |
| |  |  |  |  |  |  |  |  |  | | --- | --- | --- | --- | --- | --- | --- | --- | --- | | 1 | 2 | 3 | 4 | 5 | 6 | 7 | 8 | 9 |   |  |  |  |  |  |  | | --- | --- | --- | --- | --- | --- | | **Variable** |  | **Average** |  | **Conserved** | | | |  |  |  | | --- | --- | | **X | - Insufficient data - the calculation for this site was performed on less than 10% of the sequences. |** |
