# Supplementary material for: Biochemical and structural characterization of two cif-like epoxide hydrolases from Burkholderia cenocepacia
Source: Curr Res Struct Biol. 2021 Feb 21;3:72–84. doi: 10.1016/j.crstbi.2021.02.002 (PMC8244358; doi:10.1016/j.crstbi.2021.02.002)
Supplement: Multimedia component 7 [file mmc7.zip › Cif_MSA.html]

ConSurf Color-Coded MSA

# ConSurf Color-Coded MSA

|  |  |  |  |  |  |  |  |  |  |  |  |  |  |  |  |  |  |  |  |  |  |  |  |  |  |  |  |  |  |  |  |  |  |  |  |  |  |  |  |  |  |  |  |  |  |  |  |  |  |  |
| --- | --- | --- | --- | --- | --- | --- | --- | --- | --- | --- | --- | --- | --- | --- | --- | --- | --- | --- | --- | --- | --- | --- | --- | --- | --- | --- | --- | --- | --- | --- | --- | --- | --- | --- | --- | --- | --- | --- | --- | --- | --- | --- | --- | --- | --- | --- | --- | --- | --- | --- |
| **001 Input\_protein\_seq** | M | I | L | D | R | L | C | R | G | - | L | L | A | G | I | A | L | T | F | S | L | G | G | F | A | A | E | E | F | P | V | P | N | G | F | E | S | - | A | Y | R | E | V | D | G | V | K | L | H | Y |
| 002 UniRef90\_A0A2G0Y5Q3\_5\_314 | I | - | - | - | - | - | - | K | G | - | V | L | A | A | V | A | L | S | F | S | G | W | A | L | A | E | T | E | F | P | L | P | P | G | F | K | S | - | E | Y | K | E | V | D | G | V | K | M | H | Y |
| 003 UniRef90\_A0A172YJZ3\_2\_306 | - | - | - | - | - | - | - | - | - | - | - | L | L | G | T | T | L | A | L | A | A | P | A | F | A | A | D | E | F | P | L | P | E | G | F | T | S | - | G | Y | Q | T | I | D | G | V | K | L | H | Y |
| 004 UniRef90\_A0A4Z1C7D3\_9\_314 | - | - | - | - | - | - | - | - | - | - | A | L | I | A | A | L | T | T | T | F | A | H | A | Q | T | E | P | E | F | P | V | P | A | G | F | E | S | - | E | F | A | E | I | D | G | V | T | L | H | Y |
| 005 UniRef90\_A0A0A3Z1S4\_7\_319 | M | - | - | - | - | L | C | G | A | L | L | S | T | A | M | V | S | A | A | S | I | A | A | A | P | A | D | E | F | P | V | P | A | G | F | S | S | - | E | Y | K | R | V | D | G | V | K | L | H | Y |
| 006 UniRef90\_A0A329J6I4\_9\_315 | - | - | - | - | - | - | - | - | - | - | L | A | T | A | A | M | A | M | A | L | P | Y | L | A | Q | A | A | E | F | P | V | P | K | G | F | T | S | - | E | F | R | T | V | D | G | V | K | L | H | Y |
| 007 UniRef90\_A0A4V1G750\_8\_314 | - | - | - | - | - | - | - | - | A | - | L | C | G | A | L | A | L | L | S | S | V | S | A | C | A | E | E | E | F | P | I | P | T | G | F | T | S | - | H | Y | Q | T | V | D | G | V | K | L | H | Y |
| 008 UniRef90\_T2L220\_17\_309 | - | - | - | - | - | - | - | - | - | - | - | - | - | - | - | - | - | - | - | - | - | - | - | - | P | A | D | E | F | P | I | P | D | G | F | T | S | - | G | Y | Q | T | I | D | G | V | K | L | H | Y |
| 009 UniRef90\_UPI000C2FE79C\_12\_314 | - | - | - | - | - | - | - | - | - | - | - | - | - | S | S | A | A | L | L | S | T | L | T | F | A | A | D | E | F | P | V | P | A | N | F | S | S | - | Q | Y | K | T | I | D | G | V | R | L | H | Y |
| 010 UniRef90\_UPI00102FACD2\_13\_314 | - | - | - | - | - | - | - | - | - | - | - | - | - | - | L | S | L | F | L | A | A | P | V | F | A | A | N | T | F | P | V | P | A | G | F | T | S | - | E | Y | K | V | I | D | G | V | R | M | H | Y |
| 011 UniRef90\_UPI0014749FF6\_10\_312 | - | - | - | - | - | - | - | - | - | - | - | - | - | - | - | G | A | M | C | S | F | S | V | M | A | T | D | E | F | P | I | P | Q | G | F | K | S | - | G | F | E | E | V | N | G | I | K | M | H | Y |
| 012 UniRef90\_A0A3M4V4S1\_41\_332 | - | - | - | - | - | - | - | - | - | - | - | - | - | - | - | - | - | - | - | - | - | - | - | - | - | - | A | E | F | P | I | P | E | G | F | T | S | - | A | Y | E | T | V | D | G | V | K | L | H | F |
| 013 UniRef90\_A0A3L8C981\_31\_324 | - | - | - | - | - | - | - | - | - | - | - | - | - | - | - | - | - | - | - | - | - | - | - | - | V | Q | E | E | F | P | I | P | K | G | F | T | G | - | K | Y | E | N | V | D | G | V | K | L | H | F |
| 014 UniRef90\_A0A1X0N2L9\_32\_323 | - | - | - | - | - | - | - | - | - | - | - | - | - | - | - | - | - | - | - | - | - | - | - | - | - | Q | E | E | F | P | I | P | D | G | F | S | S | - | G | Y | E | N | V | D | G | V | E | L | H | F |
| 015 UniRef90\_UPI00166CAA4C\_15\_313 | - | - | - | - | - | - | - | - | - | - | - | - | L | L | A | I | T | A | S | L | S | S | A | R | A | A | A | P | A | P | V | P | D | G | Y | R | S | - | A | F | A | T | V | D | G | L | R | L | H | Y |
| 016 UniRef90\_UPI001661A3FF\_47\_334 | - | - | - | - | - | - | - | - | - | - | - | - | - | - | - | - | - | - | - | - | - | - | - | - | - | - | - | G | E | R | I | P | A | G | F | T | E | - | H | R | A | R | V | A | G | I | G | L | H | Y |
| 017 UniRef90\_A0A1H5MZC9\_42\_331 | - | - | - | - | - | - | - | - | - | - | - | - | - | - | - | - | - | - | - | - | - | - | - | - | A | A | V | Q | E | D | L | P | A | G | F | T | Q | - | H | R | T | Q | V | G | A | L | G | L | N | Y |
| 018 UniRef90\_UPI000690E8EC\_26\_310 | - | - | - | - | - | - | - | - | - | - | - | - | - | - | - | - | - | - | - | - | - | - | - | - | - | - | A | A | E | K | M | P | A | G | F | S | E | - | H | K | - | - | - | - | A | V | G | I | H | Y |
| 019 UniRef90\_R4LNI7\_31\_317 | - | - | - | - | - | - | - | - | - | - | - | - | - | - | - | - | - | - | - | - | - | - | - | - | - | - | - | R | S | R | V | P | A | G | F | T | E | - | Q | R | A | S | V | G | G | L | H | I | N | Y |
| 020 UniRef90\_A0A4R2C838\_67\_350 | - | - | - | - | - | - | - | - | - | - | - | - | - | - | - | - | - | - | - | - | - | - | - | - | - | - | - | - | - | - | - | P | V | G | F | I | E | - | R | K | A | N | V | N | G | I | G | I | N | Y |
| 021 UniRef90\_A0A2P9HD85\_25\_319 | - | - | - | - | - | - | - | - | - | - | - | - | - | - | - | - | - | - | - | - | - | - | - | - | - | - | - | Q | Y | A | V | P | E | G | F | E | S | - | G | M | V | E | T | D | G | I | R | L | H | Y |
| 022 UniRef90\_UPI000D14D87C\_43\_332 | - | - | - | - | - | - | - | - | - | - | - | - | - | - | - | - | - | - | - | - | - | - | - | - | A | A | P | A | E | Q | I | P | T | G | F | S | E | - | H | K | T | H | V | A | G | I | G | I | N | Y |
| 023 UniRef90\_A0A1Q4ZL08\_6\_289 | - | - | - | - | - | - | - | - | - | - | - | - | - | - | - | - | - | - | - | - | - | - | - | - | - | - | - | - | - | - | I | P | P | G | F | T | E | - | Q | V | A | R | V | G | E | I | T | I | N | Y |
| 024 UniRef90\_A0A1I2GGE7\_30\_316 | - | - | - | - | - | - | - | - | - | - | - | - | - | - | - | - | - | - | - | - | - | - | - | - | - | - | - | D | A | R | L | P | K | G | F | T | E | - | Q | K | V | R | V | G | D | V | G | I | N | Y |
| 025 UniRef90\_UPI0005580D51\_18\_305 | - | - | - | - | - | - | - | - | - | - | - | - | - | - | - | - | - | - | - | - | - | - | - | - | - | G | R | E | R | R | V | P | Y | G | F | T | E | - | Q | K | V | S | V | G | T | V | G | I | N | Y |
| 026 UniRef90\_A0A2W2F1P0\_6\_289 | - | - | - | - | - | - | - | - | - | - | - | - | - | - | - | - | - | - | - | - | - | - | - | - | - | - | - | - | - | - | V | P | A | G | F | A | E | - | Q | R | T | Q | V | G | D | V | A | V | N | Y |
| 027 UniRef90\_UPI0010F9F40F\_6\_288 | - | - | - | - | - | - | - | - | - | - | - | - | - | - | - | - | - | - | - | - | - | - | - | - | - | - | - | - | - | - | V | P | A | G | F | A | E | - | Q | R | A | Q | V | D | D | I | T | V | N | Y |
| 028 UniRef90\_A0A3E2YQE6\_6\_289 | - | - | - | - | - | - | - | - | - | - | - | - | - | - | - | - | - | - | - | - | - | - | - | - | - | - | - | - | - | - | V | P | K | G | F | S | E | - | R | R | V | R | V | G | E | V | M | I | N | H |
| 029 UniRef90\_A0A1A9ACL3\_6\_288 | - | - | - | - | - | - | - | - | - | - | - | - | - | - | - | - | - | - | - | - | - | - | - | - | - | - | - | - | - | - | M | P | T | G | F | T | E | - | Q | R | A | R | V | G | D | V | T | W | N | Y |
| 030 UniRef90\_UPI00174B5D19\_38\_323 | G | - | - | - | - | - | - | - | - | - | - | - | - | - | - | - | - | - | - | - | - | - | - | - | - | - | - | - | V | E | P | P | R | G | Y | R | S | - | E | F | A | Q | V | N | G | F | R | M | H | Y |
| 031 UniRef90\_A0A495JCH5\_50\_333 | - | - | - | - | - | - | - | - | - | - | - | - | - | - | - | - | - | - | - | - | - | - | - | - | - | - | - | - | - | - | P | P | A | G | F | T | E | - | R | K | V | V | V | G | D | I | G | I | N | F |
| 032 UniRef90\_UPI0013D14F7B\_11\_294 | - | - | - | - | - | - | - | - | - | - | - | - | - | - | - | - | - | - | - | - | - | - | - | - | - | - | - | - | - | Q | T | P | E | G | L | T | R | - | D | K | V | D | V | G | G | L | G | I | D | Y |
| 033 UniRef90\_UPI00036EC6E3\_56\_342 | - | - | - | - | - | - | - | - | - | - | - | - | - | - | - | - | - | - | - | - | - | - | - | - | - | - | - | - | P | S | P | P | A | G | F | V | E | - | R | K | V | K | V | N | G | I | G | I | N | Y |
| 034 UniRef90\_A0A1H3P9Q0\_6\_290 | - | - | - | - | - | - | - | - | - | - | - | - | - | - | - | - | - | - | - | - | - | - | - | - | - | - | - | - | - | - | V | P | S | G | F | T | E | - | Q | T | A | R | V | G | D | V | T | L | H | Y |
| 035 UniRef90\_A0A385B2U0\_41\_332 | G | - | - | - | - | - | - | - | - | - | - | - | - | - | - | - | - | - | - | - | - | - | A | S | A | R | L | P | Y | R | L | P | D | G | F | R | S | - | E | Y | A | Q | V | N | D | F | R | M | H | Y |
| 036 UniRef90\_UPI001430CB85\_25\_306 | - | - | - | - | - | - | - | - | - | - | - | - | - | - | - | - | - | - | - | - | - | - | - | - | - | - | - | - | - | - | - | - | S | G | F | E | H | - | R | F | E | T | I | D | G | I | R | L | H | Y |
| 037 UniRef90\_A0A109IHW8\_6\_288 | - | - | - | - | - | - | - | - | - | - | - | - | - | - | - | - | - | - | - | - | - | - | - | - | - | - | - | - | - | - | V | P | A | G | F | T | E | - | Q | R | A | Q | V | G | D | V | A | I | N | Y |
| 038 UniRef90\_A0A2T6L0M5\_14\_298 | - | - | - | - | - | - | - | - | - | - | - | - | - | - | - | - | - | - | - | - | - | - | - | - | - | - | - | - | T | R | V | P | A | G | Y | E | S | - | H | Y | A | H | V | N | G | F | R | M | H | Y |
| 039 UniRef90\_A0A1C4Z0V4\_6\_288 | - | - | - | - | - | - | - | - | - | - | - | - | - | - | - | - | - | - | - | - | - | - | - | - | - | - | - | - | - | - | V | P | A | G | F | T | E | - | Q | R | I | Q | V | G | A | V | A | I | N | Y |
| 040 UniRef90\_A0A4U3M272\_82\_365 | - | - | - | - | - | - | - | - | - | - | - | - | - | - | - | - | - | - | - | - | - | - | - | - | - | - | - | - | - | - | - | P | P | G | F | V | E | - | R | K | V | K | V | N | G | I | G | I | N | Y |
| 041 UniRef90\_UPI00052728DD\_22\_294 | - | - | - | - | - | - | - | - | - | - | - | - | - | - | - | - | - | - | - | - | - | - | - | - | - | - | - | - | - | - | - | - | - | R | F | E | H | - | R | F | E | M | V | D | G | I | R | L | H | Y |
| 042 UniRef90\_A0A6B8MSH7\_52\_336 | - | - | - | - | - | - | - | - | - | - | - | - | - | - | - | - | - | - | - | - | - | - | - | - | - | - | - | - | Y | A | Y | S | Q | G | L | E | S | - | K | F | I | E | T | D | G | V | R | L | H | Y |
| 043 UniRef90\_I4VSM6\_28\_303 | - | - | - | - | - | - | - | - | - | - | - | - | - | - | - | - | - | - | - | - | - | - | - | - | - | - | - | - | - | - | - | S | P | G | F | V | H | - | R | F | A | N | V | D | G | V | R | L | H | Y |
| 044 UniRef90\_A0A1I3BFK1\_40\_314 | - | - | - | - | - | - | - | - | - | - | - | - | - | - | - | - | - | - | - | - | - | - | - | - | - | - | - | - | - | P | N | L | S | G | F | K | H | - | Y | F | E | T | V | D | G | V | R | L | H | Y |
| 045 UniRef90\_A0A1Z4J856\_42\_316 | - | - | - | - | - | - | - | - | - | - | - | - | - | - | - | - | - | - | - | - | - | - | - | - | - | - | - | - | - | - | - | - | - | - | - | - | - | - | - | - | A | K | V | N | D | V | R | L | H | Y |
| 046 UniRef90\_A0A0M4FVH2\_27\_314 | - | - | - | - | - | - | - | - | - | - | - | - | - | - | - | - | - | - | - | - | - | - | - | - | - | - | - | - | - | P | S | L | D | G | F | K | H | - | R | F | Q | T | V | D | G | V | R | L | H | Y |
| 047 UniRef90\_UPI001032135D\_25\_299 | - | - | - | - | - | - | - | - | - | - | - | - | - | - | - | - | - | - | - | - | - | - | - | - | - | - | - | - | - | - | - | - | P | G | F | E | H | - | R | Y | A | T | I | E | G | V | R | I | H | Y |
| 048 UniRef90\_D5WL63\_24\_300 | - | - | - | - | - | - | - | - | - | - | - | - | - | - | - | - | - | - | - | - | - | - | - | - | - | - | - | P | V | P | G | L | E | G | F | E | H | - | K | F | A | T | V | D | G | L | R | L | H | Y |
| 049 UniRef90\_A0A1H3E943\_18\_291 | - | - | - | - | - | - | - | - | - | - | - | - | - | - | - | - | - | - | - | - | - | - | - | - | - | - | - | - | - | - | - | - | - | - | - | - | - | - | - | - | I | D | A | G | G | I | R | F | H | T |
| 050 UniRef90\_A0A346N7R3\_30\_317 | - | - | - | - | - | - | - | - | - | - | - | - | - | - | - | - | - | L | F | Q | A | P | A | H | - | - | - | A | A | S | K | S | P | A | P | Q | S | - | L | E | A | S | V | N | G | V | Q | L | H | Y |
| 051 UniRef90\_A0A537Q000\_40\_318 | - | - | - | - | - | - | - | - | - | - | - | - | - | - | - | - | - | - | - | - | - | - | - | - | - | - | - | - | - | - | - | - | L | G | F | K | S | - | D | Y | A | E | V | N | G | V | K | L | H | Y |
| 052 UniRef90\_E6V5X5\_24\_320 | - | - | - | - | - | - | - | - | - | - | V | A | V | G | A | A | L | T | M | G | G | T | A | H | A | A | A | A | A | D | A | V | P | G | F | R | S | - | G | S | A | D | V | N | G | T | R | I | H | Y |
| 053 UniRef90\_A0A484PNM3\_14\_291 | - | - | - | - | - | - | - | - | - | - | - | - | - | - | - | - | - | - | - | - | - | - | - | - | - | - | - | - | - | - | - | - | - | G | F | T | H | - | H | M | Q | G | A | P | G | N | R | L | H | Y |
| 054 UniRef90\_UPI0011BDEFB8\_30\_311 | - | - | - | - | - | - | - | - | - | - | - | - | - | - | - | - | - | - | - | - | - | - | - | - | - | - | - | - | - | - | - | - | E | G | F | V | H | - | R | Y | A | T | V | D | G | V | R | L | H | Y |
| 055 UniRef90\_A0A1H2UZ41\_16\_293 | - | - | - | - | - | - | - | - | - | - | - | - | - | - | - | - | - | - | - | - | - | - | - | - | - | - | - | - | - | - | - | - | - | - | - | - | H | - | S | M | V | E | A | E | G | I | R | F | H | T |
| 056 UniRef90\_A0A1M5ZT81\_29\_302 | - | - | - | - | - | - | - | - | - | - | - | - | - | - | - | - | - | - | - | - | - | - | - | - | - | - | - | - | - | - | - | - | - | G | F | K | H | - | C | F | E | T | V | E | G | V | R | L | H | Y |
| 057 UniRef90\_A0A135GL34\_14\_283 | - | - | - | - | - | - | - | - | - | - | - | - | - | - | - | - | - | - | - | - | - | - | - | - | - | - | - | - | - | - | - | - | - | - | - | - | - | - | - | - | I | D | L | D | E | V | R | L | H | Y |
| 058 UniRef90\_A0A484UEV2\_23\_280 | - | - | - | - | - | - | - | - | - | - | - | - | - | - | - | - | - | - | - | - | - | - | - | - | - | - | - | - | - | - | - | - | - | - | - | - | - | - | - | - | - | - | - | A | G | N | R | L | H | Y |
| 059 UniRef90\_A0A495NUG6\_51\_333 | - | - | - | - | - | - | - | - | - | - | - | - | - | - | - | - | - | - | - | - | - | - | - | - | - | - | - | - | - | - | P | P | A | G | F | T | S | - | H | D | V | D | V | D | G | T | S | M | H | Y |
| 060 UniRef90\_A0A1M5MA45\_15\_292 | - | - | - | - | - | - | - | - | - | - | - | - | - | - | - | - | - | - | - | - | - | - | - | - | - | - | - | - | - | - | P | P | E | N | F | R | H | - | Q | T | A | S | V | N | G | I | T | M | H | Y |
| 061 UniRef90\_UPI000EF95C7F\_30\_312 | - | - | - | - | - | - | - | - | - | - | - | - | - | - | - | - | - | - | - | - | - | - | - | - | - | - | - | - | - | - | - | T | N | T | F | T | S | - | A | L | T | D | V | D | G | V | Q | L | H | I |
| 062 UniRef90\_L9WLS3\_15\_271 | - | - | - | - | - | - | - | - | - | - | - | - | - | - | - | - | - | - | - | - | - | - | - | - | - | - | - | - | - | - | - | - | - | - | - | - | - | - | - | - | E | R | V | N | G | V | S | I | H | Y |
| 063 UniRef90\_UPI0005D31B04\_21\_288 | - | - | - | - | - | - | - | - | - | - | - | - | - | - | - | - | - | - | - | - | - | - | - | - | - | - | - | - | - | - | - | - | - | - | - | - | - | - | - | - | - | - | V | N | G | V | R | L | H | Y |
| 064 UniRef90\_UPI001408C682\_10\_298 | - | - | - | - | - | - | - | - | - | - | - | - | - | - | - | - | - | - | T | T | L | A | L | A | G | G | F | A | S | A | A | P | E | G | F | T | D | - | Q | Y | A | Q | V | N | G | V | K | L | H | Y |
| 065 UniRef90\_A0A1Y6KKD8\_22\_314 | - | - | - | - | - | - | - | - | - | - | - | - | - | - | - | - | - | - | - | - | - | - | - | - | F | A | R | V | E | A | S | L | P | G | F | T | H | - | R | F | E | T | V | D | G | L | R | L | H | Y |
| 066 UniRef90\_A0A0X3RYG7\_65\_349 | - | - | - | - | - | - | - | - | - | - | - | - | - | - | - | - | - | - | - | - | - | - | - | - | - | - | - | - | - | - | P | P | A | G | F | R | S | - | K | F | A | E | V | N | G | F | R | M | H | Y |
| 067 UniRef90\_A0A2T0T762\_22\_302 | - | - | - | - | - | - | - | - | - | - | - | - | - | - | - | - | - | - | - | - | - | - | - | - | - | - | - | - | - | - | - | - | - | V | F | T | S | - | R | L | V | E | V | D | G | L | R | L | H | A |
| 068 UniRef90\_A0A2V6QCX2\_10\_286 | - | - | - | - | - | - | - | - | - | - | - | - | - | - | - | - | - | - | - | - | - | - | - | - | - | - | - | - | - | - | Q | P | A | G | F | T | E | - | R | F | A | K | V | N | G | V | R | L | R | Y |
| 069 UniRef90\_D8MTC4\_28\_309 | - | - | - | - | - | - | - | - | - | - | - | - | - | - | - | - | - | - | - | - | - | - | - | - | - | - | - | - | - | - | - | - | - | G | F | V | H | - | R | Y | A | S | V | E | G | V | R | L | H | Y |
| 070 UniRef90\_UPI0012FADC80\_10\_303 | - | - | - | - | - | - | - | - | - | - | - | A | L | L | V | A | V | A | L | A | F | S | A | L | A | Q | V | P | A | S | P | P | S | N | F | K | H | - | Q | T | A | A | V | N | G | V | N | I | H | Y |
| 071 UniRef90\_A0A2E5PAM0\_2\_278 | - | - | - | - | - | - | - | - | - | - | - | - | - | - | - | - | - | - | - | - | - | - | - | - | - | - | - | - | - | - | - | - | - | - | F | E | H | - | H | T | A | E | I | D | D | V | R | L | H | Y |
| 072 UniRef90\_T0HVL8\_12\_269 | - | - | - | - | - | - | - | - | - | - | - | - | - | - | - | - | - | - | - | - | - | - | - | - | - | - | - | - | - | - | - | - | - | - | - | - | - | - | - | - | A | E | V | G | D | V | R | L | H | Y |
| 073 UniRef90\_A0A3N4T2I8\_17\_298 | - | - | - | - | - | - | - | - | - | - | - | - | - | - | - | - | - | - | - | - | - | - | - | - | - | - | - | - | - | - | - | - | - | - | F | T | S | - | R | L | V | E | V | N | G | L | R | L | H | A |
| 074 UniRef90\_A0A433IAT3\_19\_312 | - | - | - | - | - | - | - | - | - | - | - | - | - | F | V | V | L | L | L | A | C | A | S | L | S | A | T | A | S | E | I | P | A | G | F | T | E | - | H | F | A | E | V | N | G | V | R | L | H | Y |
| 075 UniRef90\_A0A226WR47\_30\_304 | - | - | - | - | - | - | - | - | - | - | - | - | - | - | - | - | - | - | - | - | - | - | - | - | - | - | - | - | - | - | - | - | T | G | F | A | H | - | R | F | E | T | V | N | G | V | R | L | H | Y |
| 076 UniRef90\_UPI000DD77D66\_25\_314 | - | - | - | - | - | - | - | - | - | - | - | - | - | - | - | - | - | - | - | - | - | - | S | S | A | E | E | S | F | T | S | S | S | S | V | V | D | - | G | K | S | D | V | D | G | V | S | Y | H | Y |
| 077 UniRef90\_A0A2A5QV31\_6\_277 | - | - | - | - | - | - | - | - | - | - | - | - | - | - | - | - | - | - | - | - | - | - | - | - | - | - | - | - | - | - | - | - | - | D | F | E | H | - | G | Q | A | R | V | N | G | V | K | L | H | Y |
| 078 UniRef90\_A0A127EY60\_8\_296 | - | - | - | - | - | - | - | - | - | - | - | G | L | L | A | V | L | A | A | A | V | A | S | L | A | P | A | A | N | A | A | E | P | R | I | E | S | - | R | F | A | E | V | N | G | V | K | L | H | Y |
| 079 UniRef90\_UPI0004952425\_6\_308 | K | - | - | - | - | - | I | C | R | - | A | I | L | L | V | M | L | M | A | L | A | V | A | L | L | N | P | G | A | A | S | A | Q | A | P | Q | S | - | R | F | A | E | V | N | G | V | K | L | H | Y |
| 080 UniRef90\_A0A0D0JP71\_56\_330 | - | - | - | - | - | - | - | - | - | - | - | - | - | - | - | - | - | - | - | - | - | - | - | - | - | - | - | - | - | - | P | P | A | N | F | K | H | - | A | Y | E | E | V | N | G | V | K | I | H | Y |
| 081 UniRef90\_A0A6A7LIC2\_1\_257 | - | - | - | - | - | - | - | - | - | - | - | - | - | - | - | - | - | - | - | - | - | - | - | - | - | - | - | - | - | - | - | - | - | - | - | - | - | - | - | - | - | - | - | - | - | - | - | M | H | Y |
| 082 UniRef90\_UPI00097BCF81\_14\_312 | A | - | - | - | - | - | - | A | A | - | A | G | I | G | A | T | F | V | L | G | D | P | A | H | - | A | A | S | T | D | T | V | A | G | F | R | S | - | R | T | A | D | V | N | G | T | R | I | H | Y |
| 083 UniRef90\_UPI001591AA5D\_7\_297 | - | - | - | - | - | - | - | - | - | - | - | - | - | - | - | - | - | - | - | - | - | - | - | S | L | P | L | P | G | G | F | T | D | V | F | T | S | - | R | L | V | E | L | D | G | L | R | L | H | A |
| 084 UniRef90\_A0A4P8YCR5\_18\_300 | - | - | - | - | - | - | - | - | - | - | - | - | - | - | - | - | - | - | - | - | - | - | - | - | - | - | - | - | - | - | - | L | P | G | F | E | H | - | R | Y | A | T | V | D | G | L | R | L | H | Y |
| 085 UniRef90\_A0A4R0GJE6\_14\_295 | - | - | - | - | - | - | - | - | - | - | - | - | - | - | - | - | - | - | - | - | - | - | - | - | - | - | - | - | - | - | - | - | - | - | F | T | S | - | R | L | V | K | V | N | E | L | R | L | H | A |
| 086 UniRef90\_A0A502C146\_32\_301 | - | - | - | - | - | - | - | - | - | - | - | - | - | - | - | - | - | - | - | - | - | - | - | - | - | - | - | - | - | - | - | - | - | - | - | - | - | - | - | - | S | V | V | N | G | V | K | L | H | Y |
| 087 UniRef90\_A0A4Y9SAQ9\_19\_291 | - | - | - | - | - | - | - | - | - | - | - | - | - | - | - | - | - | - | - | - | - | - | - | T | L | P | A | Y | A | A | A | P | A | G | Y | T | D | - | K | T | A | S | V | N | G | T | Q | I | H | Y |
| 088 UniRef90\_A0A327RPK7\_10\_286 | - | - | - | - | - | - | - | - | - | - | - | - | - | - | - | - | - | - | - | - | - | - | - | - | - | - | - | - | - | - | - | - | - | - | Y | E | H | - | R | I | A | R | V | N | N | I | N | L | H | Y |
| 089 UniRef90\_A0A239MTD5\_1\_269 | - | - | - | - | - | - | - | - | - | - | - | - | - | - | - | - | - | - | - | - | - | - | - | - | - | - | - | - | - | - | - | - | - | - | - | - | - | - | - | - | - | - | - | - | - | - | - | M | H | Y |
| 090 UniRef90\_UPI00098F4FB4\_42\_302 | - | - | - | - | - | - | - | - | - | - | - | - | - | - | - | - | - | - | - | - | - | - | - | - | - | - | - | - | - | - | - | - | - | - | - | - | - | - | - | - | S | D | V | D | G | V | S | Y | H | Y |
| 091 UniRef90\_A0A261TYY2\_38\_305 | - | - | - | - | - | - | - | - | - | - | - | - | - | - | - | - | - | - | - | - | - | - | - | - | - | - | - | - | - | - | - | - | P | H | F | E | H | - | R | M | Q | G | E | P | G | Q | R | L | H | Y |
| 092 UniRef90\_UPI0015F81933\_27\_309 | - | - | - | - | - | - | - | - | - | - | - | - | - | - | - | - | - | - | - | - | - | - | - | - | - | - | - | - | - | - | - | - | - | G | F | T | H | - | R | W | V | D | A | D | G | V | R | L | H | A |
| 093 UniRef90\_A0A0C7N0E5\_9\_278 | - | - | - | - | - | - | - | - | - | - | - | - | - | - | - | - | - | - | - | - | - | - | - | - | - | - | - | - | - | - | - | - | - | - | - | - | - | - | K | F | K | T | V | E | G | I | R | I | H | Y |
| 094 UniRef90\_UPI00112BEE4D\_14\_300 | - | - | - | - | - | - | - | - | - | - | - | - | - | - | - | - | - | - | - | - | - | - | - | - | - | - | - | - | - | - | T | P | E | G | F | V | H | - | R | H | A | L | V | D | G | L | R | L | H | Y |
| 095 UniRef90\_A0A401Z9D4\_17\_277 | - | - | - | - | - | - | - | - | - | - | - | - | - | - | - | - | - | - | - | - | - | - | - | - | - | - | - | - | - | - | - | - | - | - | - | - | - | - | - | T | A | Q | V | N | G | V | R | L | H | Y |
| 096 UniRef90\_A0A5J6MKN6\_7\_281 | - | - | - | - | - | - | - | - | - | - | - | - | - | - | - | - | - | - | - | - | - | - | - | - | - | - | - | - | A | P | L | P | E | G | F | I | E | - | K | T | A | T | V | D | A | V | S | I | N | Y |
| 097 UniRef90\_A0A4Q7XSL0\_10\_307 | - | - | - | - | - | - | - | G | A | - | S | L | L | A | A | S | A | L | S | A | T | V | P | A | F | A | G | P | V | P | Y | P | A | S | F | Q | T | - | Q | V | V | N | A | D | G | V | A | L | H | V |
| 098 UniRef90\_UPI0016110D13\_9\_294 | - | - | - | - | - | - | - | - | - | - | - | - | - | - | - | - | - | - | - | - | - | - | - | - | - | - | - | - | - | - | A | P | P | G | F | A | H | - | R | F | T | R | V | D | G | L | R | F | H | Y |
| 099 UniRef90\_A0A520GLB7\_17\_313 | - | - | - | - | - | - | - | - | - | - | A | G | I | G | V | S | L | T | V | G | S | E | V | H | A | V | P | T | A | D | A | V | A | G | F | R | S | - | R | T | A | E | V | N | G | T | R | I | H | Y |
| 100 UniRef90\_A0A252EMP1\_9\_266 | - | - | - | - | - | - | - | - | - | - | - | - | - | - | - | - | - | - | - | - | - | - | - | - | - | - | - | - | - | - | - | - | - | - | - | - | - | - | A | I | A | Q | I | G | D | V | H | L | H | Y |
| 101 UniRef90\_UPI001668C4B3\_34\_306 | - | - | - | - | - | - | - | - | - | - | - | - | - | - | - | - | - | - | - | - | - | - | - | - | - | - | - | - | - | - | - | - | - | - | F | T | S | - | H | H | V | V | T | G | G | P | R | L | H | A |
| 102 UniRef90\_UPI00135BC32E\_12\_265 | - | - | - | - | - | - | - | - | - | - | - | - | - | - | - | - | - | - | - | - | - | - | - | - | - | - | - | - | - | - | - | - | - | - | - | - | - | - | - | - | A | Y | V | N | G | V | D | L | H | Y |
| 103 UniRef90\_A0A2V9DES3\_17\_290 | - | - | - | - | - | - | - | - | - | - | - | - | - | - | - | - | - | - | - | - | - | - | - | - | - | - | - | - | - | - | - | - | - | - | - | Q | S | - | R | F | A | N | V | N | S | V | R | L | H | Y |
| 104 UniRef90\_A0A6P0DAX4\_34\_314 | - | - | - | - | - | - | - | - | - | - | - | - | - | - | - | - | - | - | - | - | - | - | - | - | - | - | - | - | - | - | - | S | N | S | I | V | D | - | G | K | S | D | V | D | G | V | N | Y | H | Y |
| 105 UniRef90\_A0A1M5KPU7\_13\_307 | - | - | - | - | - | - | - | C | I | - | A | L | L | L | V | - | - | - | - | - | - | V | A | L | L | Y | P | G | K | A | F | A | Q | A | P | Q | G | - | K | F | A | D | V | N | G | V | R | L | H | Y |
| 106 UniRef90\_A0A2I8DLX4\_17\_295 | - | - | - | - | - | - | - | - | - | - | - | - | - | - | - | - | - | - | - | - | - | - | - | - | - | - | - | - | - | - | - | - | - | P | F | R | H | - | A | R | V | E | V | D | G | L | G | Y | H | V |
| 107 UniRef90\_A0A5S4WMZ6\_36\_313 | - | - | - | - | - | - | - | - | - | - | - | - | - | - | - | - | - | - | - | - | - | - | - | - | - | - | - | - | - | - | - | - | - | Q | F | T | H | - | A | Y | I | E | A | N | G | V | R | L | H | Y |
| 108 UniRef90\_UPI0003744601\_9\_294 | - | - | - | - | - | - | - | - | - | - | - | - | - | - | - | - | - | - | - | - | - | - | - | - | - | A | L | R | A | P | V | P | D | G | A | E | S | - | R | Y | A | T | V | N | G | V | R | L | H | Y |
| 109 UniRef90\_A0A0H3KYM5\_9\_289 | - | - | - | - | - | - | - | - | - | - | - | - | - | - | - | - | - | - | - | - | - | - | - | - | - | - | - | - | - | - | A | P | D | G | F | S | H | - | H | F | S | R | I | E | G | L | R | F | H | Y |
| 110 UniRef90\_UPI001616C592\_11\_307 | - | - | - | - | - | - | - | - | - | - | - | - | - | - | V | G | G | F | G | S | V | S | G | T | P | G | L | P | D | G | F | E | E | V | F | A | S | - | R | L | V | N | V | D | G | V | H | L | H | V |
| 111 UniRef90\_A0A1U9ZZS0\_24\_308 | - | - | - | - | - | - | - | - | - | - | - | - | - | - | - | - | - | - | - | - | - | - | - | - | - | - | - | - | - | - | - | - | - | G | F | T | H | - | R | W | V | D | A | D | G | V | R | L | H | A |
| 112 UniRef90\_UPI00146E50DC\_33\_308 | - | - | - | - | - | - | - | - | - | - | - | - | - | - | - | - | - | - | - | - | - | - | - | - | - | - | - | - | - | - | - | - | - | A | I | A | S | - | K | F | A | E | A | N | G | V | R | L | H | Y |
| 113 UniRef90\_C3KLY5\_13\_287 | - | - | - | - | - | - | - | - | - | - | - | - | - | - | - | - | - | - | - | - | - | - | - | - | - | - | - | - | - | - | - | - | E | G | F | E | R | - | G | Y | A | T | V | D | G | V | R | L | H | Y |
| 114 UniRef90\_A0A2V6STQ7\_27\_303 | - | - | - | - | - | - | - | - | - | - | - | - | - | - | - | - | - | - | - | - | - | - | - | - | - | - | - | - | - | - | L | P | K | G | F | T | E | - | K | R | A | D | V | D | G | V | T | I | N | Y |
| 115 UniRef90\_UPI0008D5DA8A\_10\_290 | - | - | - | - | - | - | - | - | - | - | - | - | - | - | - | - | - | - | - | - | - | - | - | - | - | - | - | - | - | - | A | F | S | A | F | T | H | - | Q | Y | V | N | V | N | G | N | R | I | H | C |
| 116 UniRef90\_A0A3N2H8D2\_16\_299 | - | - | - | - | - | - | - | - | - | - | - | - | - | - | - | - | - | - | - | - | - | - | - | - | - | - | - | - | - | - | - | - | T | G | F | T | H | - | R | W | V | D | A | D | G | V | R | L | H | A |
| 117 UniRef90\_A0A379Z3Q1\_16\_292 | - | - | - | - | - | - | - | - | - | - | - | - | - | - | - | - | - | - | - | - | - | - | - | - | - | - | - | - | - | - | - | - | - | - | - | E | H | - | V | Y | A | R | V | K | G | N | R | I | H | C |
| 118 UniRef90\_A0A4R5QBW7\_8\_277 | - | - | - | - | - | - | - | - | - | - | - | - | - | - | - | - | - | - | - | - | - | - | - | - | - | - | - | - | - | - | - | - | - | - | - | - | - | - | - | - | A | D | L | G | E | V | R | L | H | Y |
| 119 UniRef90\_A0A163VVR8\_16\_291 | - | - | - | - | - | - | - | - | - | - | - | - | - | - | - | - | - | - | - | - | - | - | - | - | - | - | - | - | - | - | - | - | - | - | - | E | H | - | V | Y | L | Q | V | N | G | R | R | L | H | C |
| 120 UniRef90\_G0FSK7\_29\_305 | - | - | - | - | - | - | - | - | - | - | - | - | - | - | - | - | - | - | - | - | - | - | - | - | - | - | - | - | - | - | - | - | - | - | F | T | S | - | R | W | I | A | V | G | E | L | R | L | H | A |
| 121 UniRef90\_A0A4Q7FLU1\_10\_276 | - | - | - | - | - | - | - | - | - | - | - | - | - | - | - | - | - | - | - | - | - | - | - | - | - | - | - | - | - | - | - | - | - | - | - | - | - | - | - | - | - | E | L | G | D | V | R | I | H | Y |
| 122 UniRef90\_A0A2E3NGG1\_11\_269 | - | - | - | - | - | - | - | - | - | - | - | - | - | - | - | - | - | - | - | - | - | - | - | - | - | - | - | - | - | - | - | - | - | - | - | - | - | - | - | - | A | F | V | N | G | Q | Q | I | H | Y |
| 123 UniRef90\_A0A2J9ERN8\_9\_287 | - | - | - | - | - | - | - | - | - | - | - | - | - | - | - | - | - | - | - | - | - | - | - | - | - | - | - | - | - | - | A | P | E | G | F | S | H | - | K | F | A | T | V | N | G | L | R | F | H | Y |
| 124 UniRef90\_UPI001677C581\_8\_288 | - | - | - | - | - | - | - | - | - | - | - | - | - | - | - | - | - | - | - | - | - | - | - | - | - | - | - | - | - | P | D | H | D | N | Y | T | H | - | R | Y | V | R | I | D | G | Q | R | I | H | C |
| 125 UniRef90\_UPI0003039C4F\_8\_269 | - | - | - | - | - | - | - | - | - | - | - | - | - | - | - | - | - | - | - | - | - | - | - | - | - | - | - | - | - | - | - | - | - | - | - | - | - | - | - | Y | A | D | L | G | D | V | R | L | H | Y |
| 126 UniRef90\_A0A1B1YXM9\_17\_276 | - | - | - | - | - | - | - | - | - | - | - | - | - | - | - | - | - | - | - | - | - | - | - | - | - | - | - | - | - | - | - | - | - | - | - | - | - | - | - | - | - | Q | L | A | A | V | R | L | H | Y |
| 127 UniRef90\_A0A534ZXC6\_31\_288 | - | - | - | - | - | - | - | - | - | - | - | - | - | - | - | - | - | - | - | - | - | - | - | - | - | - | - | - | - | - | - | - | - | - | - | - | - | - | R | S | A | D | V | A | G | V | T | L | H | Y |
| 128 UniRef90\_A0A2X1TAZ7\_42\_319 | - | - | - | - | - | - | - | - | - | - | - | - | - | - | - | - | - | - | - | - | - | - | - | - | - | - | - | - | - | - | - | - | - | D | F | Q | H | - | H | Y | T | D | V | A | G | Q | R | I | H | Y |
| 129 UniRef90\_A0A4Q5QRH4\_8\_282 | - | - | - | - | - | - | - | - | - | - | - | - | - | - | - | - | - | - | - | - | - | - | - | - | - | - | - | - | - | P | A | P | A | G | F | A | H | - | H | F | A | T | A | G | G | L | R | F | H | Y |
| 130 UniRef90\_A0A0D6HQT0\_17\_294 | - | - | - | - | - | - | - | - | - | - | - | - | - | - | - | - | - | - | - | - | - | - | - | - | - | - | - | - | - | - | - | - | - | - | F | R | H | - | R | R | V | R | V | D | G | L | G | Y | H | V |
| 131 UniRef90\_A0A1G4JCM6\_9\_275 | - | - | - | - | - | - | - | - | - | - | - | - | - | - | - | - | - | - | - | - | - | - | - | - | - | - | - | - | - | - | - | - | - | - | - | - | - | - | M | F | K | A | V | E | G | I | R | M | H | Y |
| 132 UniRef90\_UPI001269E708\_28\_301 | - | - | - | - | - | - | - | - | - | - | - | - | - | - | - | - | - | - | - | - | - | - | - | - | - | - | - | - | - | - | - | - | A | G | F | T | H | - | R | W | V | D | A | D | G | V | R | L | H | A |
| 133 UniRef90\_A0A538SUN7\_3\_303 | - | - | - | - | - | - | - | R | C | - | F | L | I | G | L | A | A | L | F | L | A | S | P | A | I | A | R | V | E | P | F | P | A | G | F | R | S | - | Q | A | I | A | T | N | G | T | S | L | Y | V |
| 134 UniRef90\_UPI001646B9D9\_15\_288 | - | - | - | - | - | - | - | - | - | - | - | - | - | - | - | - | - | - | - | - | - | - | - | - | - | - | - | - | - | - | - | - | - | - | - | - | - | - | V | Y | V | Q | V | D | G | L | R | L | H | C |
| 135 UniRef90\_K9DQK6\_17\_307 | - | - | - | - | - | - | - | - | - | - | - | - | - | - | - | - | - | - | - | - | A | L | A | A | Q | A | A | E | P | S | M | P | A | G | F | K | T | A | S | V | Q | T | A | D | G | A | T | I | H | V |
| 136 UniRef90\_A0A2N9BM66\_25\_297 | - | - | - | - | - | - | - | - | - | - | - | - | - | - | - | - | - | - | - | - | - | - | - | - | - | - | - | - | - | - | - | - | - | - | - | - | - | - | - | - | - | K | T | T | E | L | T | L | H | A |
| 137 UniRef90\_A0A370LBV7\_5\_266 | - | - | - | - | - | - | - | - | - | - | - | - | - | - | - | - | - | - | - | - | - | - | - | - | - | - | - | - | - | - | - | - | - | - | I | E | S | - | R | F | A | E | V | D | G | I | K | L | H | Y |
| 138 UniRef90\_A0A2V9ZM06\_3\_258 | - | - | - | - | - | - | - | - | - | - | - | - | - | - | - | - | - | - | - | - | - | - | - | - | - | - | - | - | - | - | - | - | - | - | - | - | - | - | - | - | - | E | V | D | G | V | K | L | H | Y |
| 139 UniRef90\_UPI00101F418B\_26\_310 | - | - | - | - | - | - | - | - | - | - | - | - | - | - | - | - | - | - | - | - | - | - | - | - | - | - | - | - | - | - | - | - | - | - | F | Q | H | - | R | E | A | Q | V | G | S | I | R | L | H | Y |
| 140 UniRef90\_A0A4V2YYI8\_27\_305 | - | - | - | - | - | - | - | - | - | - | - | - | - | - | - | - | - | - | - | - | - | - | - | - | - | - | - | - | - | - | - | - | D | T | F | T | S | - | R | Y | V | D | I | G | E | L | R | L | H | A |
| 141 UniRef90\_UPI001487D2FB\_8\_292 | - | - | - | - | - | - | - | - | - | - | - | - | - | A | A | I | A | L | A | L | A | M | P | A | S | A | E | V | Q | P | F | P | S | S | F | R | A | - | Q | Q | V | Q | S | E | G | A | T | L | H | V |
| 142 UniRef90\_B9XAH7\_320\_607 | - | - | - | - | - | - | - | - | - | - | - | - | - | - | - | - | - | - | - | - | - | - | - | - | - | - | - | - | - | - | A | P | S | G | V | Q | H | - | K | T | V | L | V | N | G | V | K | L | H | Y |
| 143 UniRef90\_UPI0013696204\_44\_306 | - | - | - | - | - | - | - | - | - | - | - | - | - | - | - | - | - | - | - | - | - | - | - | - | - | - | - | - | - | - | - | - | - | - | - | - | - | - | - | - | - | - | P | D | G | L | R | L | H | A |
| 144 UniRef90\_W5WIM6\_13\_292 | - | - | - | - | - | - | - | - | - | - | - | - | - | - | - | - | - | - | - | - | - | - | - | - | - | - | - | - | - | - | - | - | D | T | F | R | S | - | A | W | V | Q | V | G | D | L | R | L | H | A |
| 145 UniRef90\_A0A2X2GG63\_10\_290 | - | - | - | - | - | - | - | - | - | - | - | - | - | - | - | - | - | - | - | - | - | - | - | - | - | - | - | - | - | L | N | G | P | E | P | E | H | - | V | Y | A | R | V | N | G | N | R | I | H | C |
| 146 UniRef90\_A0A2E2YI39\_6\_274 | - | - | - | - | - | - | - | - | - | - | - | - | - | - | - | - | - | - | - | - | - | - | - | - | - | - | - | - | - | - | - | - | - | G | V | X | H | - | Q | Y | A | D | L | G | D | V | M | L | H | Y |
| 147 UniRef90\_A0A2W7G3K6\_24\_312 | - | - | - | - | - | - | - | - | - | - | - | - | - | - | - | - | - | - | - | - | - | - | - | - | - | - | - | - | - | - | - | - | N | T | F | R | H | - | G | T | A | N | V | D | G | V | R | L | H | Y |
| 148 UniRef90\_A0A6B2RWJ8\_28\_298 | - | - | - | - | - | - | - | - | - | - | - | - | - | - | - | - | - | - | - | - | - | - | - | - | - | - | - | - | - | - | - | - | - | G | F | A | H | - | R | W | A | D | A | E | G | I | R | L | H | A |
| 149 UniRef90\_A0A537A198\_6\_270 | - | - | - | - | - | - | - | - | - | - | - | - | - | - | - | - | - | - | - | - | - | - | - | - | - | - | - | - | - | - | - | - | - | - | - | - | - | - | - | - | - | - | - | - | - | V | T | L | H | V |
| 150 UniRef90\_UPI00135F2885\_48\_334 | - | - | - | - | - | - | - | - | - | - | - | - | - | - | - | - | - | - | - | - | - | - | - | - | - | - | - | - | - | - | - | - | T | G | F | R | H | - | H | R | T | A | V | G | D | V | R | L | H | Y |

  
  

|  |  |  |  |  |  |  |  |  |  |  |  |  |  |  |  |  |  |  |  |  |  |  |  |  |  |  |  |  |  |  |  |  |  |  |  |  |  |  |  |  |  |  |  |  |  |  |  |  |  |  |
| --- | --- | --- | --- | --- | --- | --- | --- | --- | --- | --- | --- | --- | --- | --- | --- | --- | --- | --- | --- | --- | --- | --- | --- | --- | --- | --- | --- | --- | --- | --- | --- | --- | --- | --- | --- | --- | --- | --- | --- | --- | --- | --- | --- | --- | --- | --- | --- | --- | --- | --- |
| **001 Input\_protein\_seq** | V | K | G | G | - | - | - | - | Q | G | - | P | - | L | V | M | L | V | H | G | F | G | Q | T | W | Y | E | W | H | Q | L | M | P | E | L | - | A | K | R | - | F | T | V | I | A | P | D | L | P | G |
| 002 UniRef90\_A0A2G0Y5Q3\_5\_314 | V | T | G | G | - | - | - | - | S | G | - | P | - | L | V | M | L | V | H | G | F | G | Q | T | W | Y | E | W | H | Q | L | M | P | E | L | - | A | R | N | - | F | T | V | V | A | P | D | L | P | G |
| 003 UniRef90\_A0A172YJZ3\_2\_306 | V | K | G | G | - | - | - | - | S | G | - | P | - | L | V | Y | L | V | H | G | F | G | Q | T | W | Y | E | W | H | Q | L | M | P | E | L | - | A | K | R | - | F | T | V | V | A | P | D | L | P | G |
| 004 UniRef90\_A0A4Z1C7D3\_9\_314 | V | T | G | G | - | - | - | - | S | G | - | P | - | L | V | Y | L | V | H | G | F | G | Q | S | W | Y | E | W | H | Q | L | M | P | E | L | - | A | K | T | - | H | M | V | V | A | V | D | L | P | G |
| 005 UniRef90\_A0A0A3Z1S4\_7\_319 | V | K | G | G | - | - | - | - | K | G | - | P | - | L | V | Y | L | V | H | G | F | G | Q | S | W | Y | E | W | H | Q | L | M | P | E | L | - | A | A | R | - | H | T | V | V | A | V | D | L | P | G |
| 006 UniRef90\_A0A329J6I4\_9\_315 | V | K | G | G | - | - | - | - | S | G | - | P | - | L | V | F | L | A | H | G | F | G | Q | A | W | Y | E | W | H | N | L | M | P | E | L | - | A | R | N | - | H | T | V | V | A | V | D | L | P | G |
| 007 UniRef90\_A0A4V1G750\_8\_314 | V | K | G | G | - | - | - | - | S | G | - | P | - | L | V | F | L | V | H | G | F | G | Q | T | W | Y | E | W | H | Q | L | M | P | L | L | - | A | Q | H | - | Y | T | V | V | A | P | D | L | P | G |
| 008 UniRef90\_T2L220\_17\_309 | V | K | G | G | - | - | - | - | S | G | - | P | - | L | V | Y | L | V | H | G | F | G | Q | T | W | Y | E | W | H | Q | L | M | P | A | L | - | A | K | S | - | F | T | V | V | A | P | D | L | P | G |
| 009 UniRef90\_UPI000C2FE79C\_12\_314 | V | K | G | G | - | - | - | - | S | G | - | P | - | L | V | Y | L | V | H | G | F | G | Q | T | W | Y | E | W | H | Q | L | M | P | E | L | - | A | K | N | - | H | T | V | V | A | V | D | L | P | G |
| 010 UniRef90\_UPI00102FACD2\_13\_314 | V | K | G | G | - | - | - | - | Q | G | - | P | - | L | V | Y | L | V | H | G | F | G | Q | T | W | Y | E | W | H | H | L | M | P | E | L | - | A | R | N | - | H | T | V | V | A | P | D | L | P | G |
| 011 UniRef90\_UPI0014749FF6\_10\_312 | V | A | G | G | - | - | - | - | S | G | - | P | - | V | I | L | M | V | H | G | F | A | Q | S | W | Y | E | W | N | D | L | M | P | Q | L | - | A | K | T | - | H | T | V | I | A | V | D | L | P | G |
| 012 UniRef90\_A0A3M4V4S1\_41\_332 | V | R | G | G | - | - | - | - | K | G | - | P | - | L | V | L | L | V | H | G | F | G | Q | T | W | Y | E | W | N | S | L | M | P | Q | L | - | A | E | R | - | Y | T | V | V | A | V | D | L | P | G |
| 013 UniRef90\_A0A3L8C981\_31\_324 | V | H | G | G | - | - | - | - | K | G | - | P | - | L | V | L | L | V | H | G | F | G | Q | T | W | Y | E | W | N | E | L | M | Q | Q | L | - | A | E | R | - | Y | T | V | V | A | V | D | L | P | G |
| 014 UniRef90\_A0A1X0N2L9\_32\_323 | V | R | G | G | - | - | - | - | R | G | - | P | - | L | V | M | L | V | H | G | F | G | Q | T | W | Y | E | W | N | S | L | M | S | Q | L | - | A | E | R | - | Y | T | V | L | A | V | D | L | P | G |
| 015 UniRef90\_UPI00166CAA4C\_15\_313 | V | I | G | G | - | - | - | - | R | G | - | T | - | P | L | L | L | I | H | G | W | G | S | T | W | Y | M | W | R | K | V | M | P | A | L | - | A | Q | Q | - | H | T | T | I | V | V | D | L | P | G |
| 016 UniRef90\_UPI001661A3FF\_47\_334 | V | T | G | G | - | - | - | - | H | G | - | P | - | T | L | V | L | L | H | G | Y | P | Q | T | W | Y | E | W | H | G | I | M | P | A | L | - | A | E | H | - | Y | T | V | I | A | P | D | L | P | G |
| 017 UniRef90\_A0A1H5MZC9\_42\_331 | V | I | G | G | - | - | - | - | H | G | - | P | - | T | L | V | L | L | H | G | Y | P | Q | T | W | Y | E | W | R | H | V | M | P | A | L | - | A | E | H | - | Y | T | V | I | A | P | D | L | P | G |
| 018 UniRef90\_UPI000690E8EC\_26\_310 | V | I | G | G | - | - | - | - | H | G | - | P | - | T | L | V | L | L | H | G | Y | P | Q | T | W | Y | E | W | H | D | I | L | P | A | L | - | A | R | H | - | Y | T | V | I | A | P | D | L | P | G |
| 019 UniRef90\_R4LNI7\_31\_317 | V | R | G | G | - | - | - | - | H | G | - | P | - | T | L | V | L | L | H | G | F | P | E | S | W | Y | M | W | R | D | V | L | P | A | L | - | A | E | H | - | Y | T | V | I | A | P | D | L | P | G |
| 020 UniRef90\_A0A4R2C838\_67\_350 | V | R | G | G | - | - | - | - | H | G | - | P | - | T | L | L | L | L | H | G | Y | P | Q | T | W | Y | T | W | N | H | I | L | P | A | L | - | A | R | H | - | Y | T | V | V | A | P | D | L | P | G |
| 021 UniRef90\_A0A2P9HD85\_25\_319 | V | R | G | G | E | G | - | - | K | G | - | D | - | P | I | I | F | V | H | G | F | G | S | T | W | K | M | W | Q | P | A | L | E | K | F | - | S | A | N | - | H | Q | V | I | A | I | D | L | P | G |
| 022 UniRef90\_UPI000D14D87C\_43\_332 | V | I | G | G | - | - | - | - | H | G | - | P | - | T | L | V | L | I | H | G | Y | P | Q | T | W | Y | E | W | H | G | I | M | P | A | L | - | A | E | H | - | Y | T | V | I | A | P | D | L | P | G |
| 023 UniRef90\_A0A1Q4ZL08\_6\_289 | V | R | G | G | - | - | - | - | Q | G | - | G | - | T | L | V | L | L | H | G | Y | P | Q | T | W | F | M | W | R | K | V | L | P | E | L | - | A | K | R | - | Y | T | V | I | A | P | D | L | R | G |
| 024 UniRef90\_A0A1I2GGE7\_30\_316 | V | R | G | G | - | - | - | - | R | G | - | P | - | V | L | V | L | V | H | G | Y | P | Q | T | W | Y | E | W | H | E | I | M | P | A | L | - | A | E | H | - | Y | T | V | V | A | P | D | L | R | G |
| 025 UniRef90\_UPI0005580D51\_18\_305 | V | R | G | G | - | - | - | - | S | G | - | P | - | T | L | V | L | V | H | G | F | P | Q | T | W | Y | E | W | R | G | L | L | P | E | L | - | A | K | H | - | Y | T | V | I | A | P | D | L | R | G |
| 026 UniRef90\_A0A2W2F1P0\_6\_289 | V | R | G | G | - | - | - | - | N | G | - | P | - | T | L | V | L | L | H | G | Y | P | Q | S | W | Y | M | W | R | H | V | L | P | E | L | - | G | Q | S | - | Y | E | V | I | A | P | D | L | R | G |
| 027 UniRef90\_UPI0010F9F40F\_6\_288 | V | R | G | G | - | - | - | - | S | G | - | P | - | T | L | V | L | L | H | G | Y | P | Q | T | W | Y | M | W | R | H | V | L | P | E | L | - | G | R | S | - | F | E | V | I | A | P | D | L | R | G |
| 028 UniRef90\_A0A3E2YQE6\_6\_289 | V | R | G | G | - | - | - | - | Q | G | - | P | - | P | L | V | L | L | H | G | Y | P | E | S | W | Y | M | W | R | A | L | L | P | K | L | - | A | R | H | - | F | D | V | V | A | P | D | L | R | G |
| 029 UniRef90\_A0A1A9ACL3\_6\_288 | V | R | G | G | - | - | - | - | R | G | - | P | - | T | V | V | L | L | H | G | Y | P | Q | C | W | R | M | W | R | H | L | L | P | E | L | - | A | E | S | - | Y | E | V | V | A | P | D | L | R | G |
| 030 UniRef90\_UPI00174B5D19\_38\_323 | L | R | G | G | - | - | - | - | K | G | - | S | - | P | V | V | L | I | H | G | F | P | Q | T | S | A | E | W | R | P | Q | L | D | A | L | - | A | R | K | - | H | D | V | I | A | V | D | L | R | G |
| 031 UniRef90\_A0A495JCH5\_50\_333 | V | E | G | G | - | - | - | - | H | G | - | K | - | T | L | V | L | L | H | G | Y | P | Q | T | W | Y | E | W | H | D | I | L | P | E | L | - | A | Q | H | - | Y | H | V | I | A | P | D | L | R | G |
| 032 UniRef90\_UPI0013D14F7B\_11\_294 | V | T | G | G | - | - | - | - | S | G | - | P | - | T | L | V | L | L | H | G | Y | P | Q | T | W | Y | E | W | R | H | V | L | P | A | L | - | A | E | H | - | Y | T | V | V | A | P | S | L | R | G |
| 033 UniRef90\_UPI00036EC6E3\_56\_342 | V | R | G | G | - | - | - | - | H | G | - | P | - | T | L | L | L | L | H | G | Y | P | Q | T | W | Y | T | W | D | H | I | L | P | A | L | - | A | Q | H | - | Y | T | V | V | A | P | D | L | P | G |
| 034 UniRef90\_A0A1H3P9Q0\_6\_290 | V | H | G | G | - | - | - | - | R | G | - | R | - | T | V | V | L | L | H | G | Y | P | Q | T | W | Y | M | W | R | K | V | L | P | E | L | - | A | A | H | - | Y | T | I | I | A | P | D | L | R | G |
| 035 UniRef90\_A0A385B2U0\_41\_332 | L | R | G | G | - | - | - | - | S | G | - | S | - | P | V | V | L | L | H | G | F | P | Q | T | S | A | E | W | E | P | Q | L | E | A | L | - | A | K | D | - | H | T | V | I | A | V | D | L | R | G |
| 036 UniRef90\_UPI001430CB85\_25\_306 | V | T | G | G | K | D | - | - | S | G | - | G | - | T | V | V | L | L | A | G | Y | P | E | S | W | F | A | W | R | K | V | M | P | A | L | - | A | E | H | - | Y | R | V | I | V | P | D | L | P | G |
| 037 UniRef90\_A0A109IHW8\_6\_288 | V | R | G | G | - | - | - | - | R | G | - | P | - | T | L | V | L | L | H | G | Y | P | Q | C | W | R | M | W | R | G | L | L | P | E | L | - | A | A | T | - | Y | H | V | V | A | P | D | L | R | G |
| 038 UniRef90\_A0A2T6L0M5\_14\_298 | L | R | G | G | - | - | - | - | S | G | - | S | - | P | V | L | L | L | H | G | F | P | Q | T | W | A | E | W | H | H | Q | L | G | P | L | - | A | A | D | - | H | T | V | I | A | V | D | L | R | G |
| 039 UniRef90\_A0A1C4Z0V4\_6\_288 | V | R | G | G | - | - | - | - | R | G | - | P | - | T | L | V | L | L | H | G | Y | P | Q | C | W | R | M | W | R | G | L | L | P | E | L | - | A | D | S | - | Y | H | V | I | A | P | D | L | R | G |
| 040 UniRef90\_A0A4U3M272\_82\_365 | V | R | G | G | - | - | - | - | H | G | - | P | - | T | L | L | L | L | H | G | Y | P | Q | T | W | F | T | W | D | H | V | L | P | A | L | - | A | Q | H | - | Y | T | V | V | A | P | D | L | P | G |
| 041 UniRef90\_UPI00052728DD\_22\_294 | V | T | G | G | K | V | - | - | D | G | - | E | - | T | V | V | L | L | A | G | Y | P | Q | S | W | F | A | W | R | K | V | M | P | L | L | - | A | A | R | - | Y | R | I | V | A | P | D | L | P | G |
| 042 UniRef90\_A0A6B8MSH7\_52\_336 | V | E | G | G | N | - | - | - | S | G | - | T | - | P | I | I | F | I | H | G | F | G | S | T | W | K | M | W | E | P | V | M | E | K | F | - | K | A | T | - | H | K | V | I | A | I | D | L | P | G |
| 043 UniRef90\_I4VSM6\_28\_303 | V | R | G | G | R | P | - | - | G | G | - | D | - | T | I | V | L | L | A | G | F | P | Q | S | W | Y | A | W | R | Q | V | M | P | C | L | - | A | S | T | - | H | D | V | I | A | I | D | L | P | G |
| 044 UniRef90\_A0A1I3BFK1\_40\_314 | V | E | G | G | N | V | - | - | D | G | - | E | - | V | I | V | L | L | A | G | F | P | E | S | W | F | A | W | R | K | V | M | P | L | L | - | A | S | A | - | Y | R | I | I | A | L | D | L | P | G |
| 045 UniRef90\_A0A1Z4J856\_42\_316 | V | I | G | G | - | - | - | - | K | G | - | E | - | P | L | V | L | L | H | G | F | P | T | T | W | Y | E | W | R | H | V | M | P | T | L | - | A | E | R | - | Y | T | V | I | V | P | D | M | R | G |
| 046 UniRef90\_A0A0M4FVH2\_27\_314 | V | V | G | G | K | D | - | - | D | G | - | D | - | V | V | V | L | L | P | G | F | P | E | S | W | F | T | W | H | K | V | M | P | L | L | - | A | P | T | - | Y | K | L | I | V | L | D | L | P | G |
| 047 UniRef90\_UPI001032135D\_25\_299 | V | T | G | G | N | P | - | - | A | G | - | D | - | T | L | V | L | L | A | G | F | P | Q | S | W | Y | A | W | R | K | V | M | Q | Q | M | - | G | A | T | - | Y | H | I | V | A | P | D | L | P | G |
| 048 UniRef90\_D5WL63\_24\_300 | V | T | G | G | N | P | - | - | Q | G | - | D | - | V | I | V | L | L | A | G | F | P | Q | S | W | F | A | W | R | N | V | M | A | L | L | - | A | K | T | - | Y | R | I | V | A | P | D | L | P | G |
| 049 UniRef90\_A0A1H3E943\_18\_291 | V | S | G | G | - | - | - | - | T | G | - | E | - | P | L | L | L | I | A | G | F | P | Q | T | W | Y | A | W | R | R | M | M | P | L | L | - | A | E | K | - | Y | N | V | I | A | I | D | L | P | G |
| 050 UniRef90\_A0A346N7R3\_30\_317 | L | R | A | G | - | - | - | - | S | G | - | P | - | A | V | V | L | L | H | G | Y | A | Q | T | S | R | M | W | L | P | L | I | P | E | L | - | A | K | T | - | H | T | V | I | A | P | D | L | R | G |
| 051 UniRef90\_A0A537Q000\_40\_318 | V | A | G | G | - | - | - | - | E | G | R | K | - | A | L | L | L | I | P | G | W | P | Q | T | W | Y | V | W | R | K | V | M | P | E | L | - | A | K | E | - | Y | R | V | I | A | V | D | T | R | G |
| 052 UniRef90\_E6V5X5\_24\_320 | R | I | G | G | - | - | - | - | S | G | - | P | - | T | V | V | L | L | H | G | Y | A | E | T | G | H | M | W | N | P | L | M | P | L | L | - | A | K | T | - | H | T | V | V | V | P | D | L | R | G |
| 053 UniRef90\_A0A484PNM3\_14\_291 | V | I | G | G | - | - | - | - | Q | G | - | D | - | P | V | L | L | V | P | G | W | P | Q | T | W | Y | A | W | R | K | I | M | P | A | L | - | A | E | K | - | H | T | V | V | A | V | D | P | P | G |
| 054 UniRef90\_UPI0011BDEFB8\_30\_311 | V | T | G | G | N | P | - | - | D | G | - | E | - | T | L | V | L | L | A | G | F | P | Q | S | W | Y | A | W | R | Q | V | M | P | - | L | - | M | N | T | - | F | H | I | I | A | P | D | L | P | G |
| 055 UniRef90\_A0A1H2UZ41\_16\_293 | V | S | G | G | - | - | - | - | Q | G | - | F | - | P | V | L | L | M | A | G | F | P | Q | S | W | Y | A | W | R | R | V | M | P | L | L | - | C | E | H | - | F | H | V | V | A | I | D | L | P | G |
| 056 UniRef90\_A0A1M5ZT81\_29\_302 | V | A | G | G | K | A | - | - | D | G | - | Q | - | T | V | V | L | L | A | G | Y | P | E | S | W | F | A | W | R | K | V | M | P | M | L | - | S | A | N | - | Y | R | V | I | V | P | D | L | P | G |
| 057 UniRef90\_A0A135GL34\_14\_283 | V | R | G | G | - | - | - | - | D | G | - | P | - | P | L | V | L | L | H | G | W | G | S | T | W | Y | M | W | R | K | V | M | S | Q | L | - | A | D | H | - | Y | T | V | I | V | P | D | L | R | G |
| 058 UniRef90\_A0A484UEV2\_23\_280 | V | T | G | G | - | - | - | - | K | G | - | D | - | P | I | L | L | V | P | G | W | P | Q | T | W | Y | A | W | R | K | I | M | P | A | L | - | A | E | K | - | Y | T | V | V | A | V | D | P | P | G |
| 059 UniRef90\_A0A495NUG6\_51\_333 | L | I | G | G | - | - | - | - | S | G | - | P | - | T | L | V | L | V | H | G | Y | P | Q | T | S | Y | E | W | Y | S | V | M | P | A | L | - | A | E | H | - | Y | T | V | V | V | P | D | L | P | G |
| 060 UniRef90\_A0A1M5MA45\_15\_292 | V | I | G | G | - | - | - | - | E | G | - | P | - | P | L | V | L | V | H | G | F | G | Q | N | W | F | M | W | N | R | L | L | P | E | F | - | S | K | H | - | F | T | V | I | A | P | E | L | R | G |
| 061 UniRef90\_UPI000EF95C7F\_30\_312 | V | T | G | G | - | - | - | - | D | G | - | P | - | P | L | L | L | I | G | G | W | P | Q | F | W | Y | Q | W | R | L | V | M | P | S | L | - | A | E | H | - | F | T | V | I | A | A | D | P | R | G |
| 062 UniRef90\_L9WLS3\_15\_271 | V | T | A | G | - | - | - | - | S | G | - | P | - | P | L | V | L | L | H | G | W | P | Q | T | W | Y | E | W | R | D | V | I | P | S | F | - | A | A | E | - | H | T | V | I | A | P | D | L | R | G |
| 063 UniRef90\_UPI0005D31B04\_21\_288 | V | M | A | G | - | - | - | - | S | G | - | E | - | P | L | V | L | L | H | G | W | P | Q | T | W | R | E | W | H | S | M | I | P | A | L | - | A | R | Q | - | F | T | V | I | A | P | D | M | R | G |
| 064 UniRef90\_UPI001408C682\_10\_298 | Q | V | G | G | - | - | - | - | K | G | - | S | - | P | V | V | L | L | H | G | Y | A | Q | T | G | H | M | W | G | P | A | M | A | E | L | - | V | K | N | - | H | T | V | I | V | P | D | M | R | G |
| 065 UniRef90\_A0A1Y6KKD8\_22\_314 | V | S | G | G | R | P | - | - | E | G | - | E | - | V | V | V | L | L | A | G | F | P | Q | S | W | F | A | W | R | K | V | M | P | L | L | - | G | A | R | - | H | R | V | I | A | V | D | F | P | G |
| 066 UniRef90\_A0A0X3RYG7\_65\_349 | V | R | G | G | - | - | - | - | K | G | - | S | - | P | V | V | M | I | H | G | F | P | E | D | W | S | E | W | R | K | E | M | V | P | L | - | S | K | N | - | H | T | V | I | A | V | D | L | R | G |
| 067 UniRef90\_A0A2T0T762\_22\_302 | V | T | G | G | - | - | - | - | D | G | - | P | - | P | L | L | L | I | S | G | W | P | Q | T | W | Y | A | W | R | E | M | M | P | A | L | - | A | R | E | - | H | T | V | V | A | V | D | S | R | G |
| 068 UniRef90\_A0A2V6QCX2\_10\_286 | L | V | G | G | - | - | - | - | R | G | - | S | - | A | V | V | L | L | H | G | Y | A | E | T | G | H | M | W | R | P | L | M | P | L | L | - | A | E | R | - | H | T | V | V | V | P | D | L | R | G |
| 069 UniRef90\_D8MTC4\_28\_309 | V | S | G | G | N | P | - | - | Q | G | - | E | - | T | L | V | L | L | A | G | F | P | Q | S | W | F | A | W | R | Q | V | M | A | R | L | - | A | S | T | - | Y | R | I | I | A | P | D | L | P | G |
| 070 UniRef90\_UPI0012FADC80\_10\_303 | V | I | G | G | - | - | - | - | K | G | - | A | - | P | L | M | L | V | H | G | F | G | Q | N | W | Y | M | W | N | R | L | L | P | E | L | - | S | K | H | - | F | T | V | I | A | P | D | L | R | G |
| 071 UniRef90\_A0A2E5PAM0\_2\_278 | V | T | V | G | - | - | - | - | N | A | - | P | K | V | L | C | L | L | H | G | W | P | Q | T | W | W | E | W | R | R | V | I | P | T | L | - | A | E | H | - | F | T | V | V | A | P | D | L | X | G |
| 072 UniRef90\_T0HVL8\_12\_269 | V | R | G | G | - | - | - | - | Q | G | - | E | - | P | V | L | L | V | H | G | W | P | Q | T | W | F | S | W | R | H | V | I | P | R | L | I | A | A | G | - | H | D | V | I | A | V | D | M | R | G |
| 073 UniRef90\_A0A3N4T2I8\_17\_298 | V | T | G | G | - | - | - | - | D | G | - | P | - | P | L | L | L | I | G | G | W | P | Q | T | W | Y | A | W | R | E | V | M | P | A | L | - | A | R | R | - | H | T | V | V | A | V | D | S | R | G |
| 074 UniRef90\_A0A433IAT3\_19\_312 | L | I | G | G | - | - | - | - | Q | G | - | N | - | P | V | V | L | L | H | G | Y | A | E | T | G | H | M | W | L | P | L | M | P | L | L | - | A | K | N | - | H | T | V | I | V | P | D | L | R | G |
| 075 UniRef90\_A0A226WR47\_30\_304 | V | T | G | D | K | I | - | - | D | G | - | D | - | T | V | V | L | L | A | G | F | P | E | S | W | Y | A | W | R | K | V | M | P | L | L | - | A | E | R | - | Y | K | I | V | A | L | D | L | P | G |
| 076 UniRef90\_UPI000DD77D66\_25\_314 | L | L | A | R | - | - | - | - | G | G | - | P | Q | T | V | V | L | L | H | G | W | G | T | T | S | Y | M | W | R | Y | V | M | P | E | L | - | T | G | R | G | Y | T | V | L | A | P | D | I | R | G |
| 077 UniRef90\_A0A2A5QV31\_6\_277 | V | T | A | G | - | - | - | - | E | G | - | P | - | P | L | V | L | L | H | G | W | P | Q | T | W | Y | E | W | R | D | V | I | P | E | L | - | A | A | D | - | H | T | V | I | A | P | D | L | R | G |
| 078 UniRef90\_A0A127EY60\_8\_296 | L | V | A | G | - | - | - | - | Q | G | - | S | - | P | V | I | L | L | H | G | Y | A | Q | N | S | H | M | W | R | P | L | I | P | K | L | - | A | A | T | - | H | T | V | I | A | P | D | L | R | G |
| 079 UniRef90\_UPI0004952425\_6\_308 | L | V | A | G | - | - | - | - | K | G | - | D | - | P | V | V | L | L | H | G | Y | A | Q | T | S | H | M | W | L | P | L | I | A | K | L | - | A | D | K | - | H | T | V | I | A | P | D | L | R | G |
| 080 UniRef90\_A0A0D0JP71\_56\_330 | V | I | G | G | - | - | - | - | K | G | - | D | - | P | L | V | L | V | H | G | F | G | Q | N | W | Y | M | W | N | R | L | L | P | E | L | - | S | K | H | - | F | T | V | I | A | P | D | L | R | G |
| 081 UniRef90\_A0A6A7LIC2\_1\_257 | V | I | G | G | - | - | - | - | Q | G | - | D | - | P | V | V | L | L | H | G | W | P | Q | T | W | Y | E | W | R | H | V | M | P | A | L | - | A | K | Y | - | Y | T | V | V | A | P | D | L | R | G |
| 082 UniRef90\_UPI00097BCF81\_14\_312 | R | I | G | G | - | - | - | - | T | G | - | P | - | A | V | V | L | L | H | G | Y | A | E | T | S | H | M | W | N | P | L | M | P | L | L | - | A | K | T | - | H | T | V | V | V | P | D | L | R | G |
| 083 UniRef90\_UPI001591AA5D\_7\_297 | V | T | G | G | - | - | - | - | D | G | - | P | - | P | L | L | L | V | G | G | W | P | Q | T | W | Y | A | W | R | R | V | M | P | A | L | - | A | R | R | - | H | T | V | V | A | V | D | P | R | G |
| 084 UniRef90\_A0A4P8YCR5\_18\_300 | V | A | G | G | N | E | - | - | E | G | - | A | - | T | L | V | L | F | A | G | F | P | Q | S | W | Y | A | W | H | K | V | M | K | A | L | - | A | S | R | - | F | Y | I | I | A | P | D | L | P | G |
| 085 UniRef90\_A0A4R0GJE6\_14\_295 | V | T | G | G | - | - | - | - | N | G | - | P | - | A | L | L | L | V | G | G | W | P | Q | T | W | Y | A | W | R | Q | V | M | P | A | L | - | A | R | E | - | H | T | V | V | A | V | D | S | R | G |
| 086 UniRef90\_A0A502C146\_32\_301 | M | K | A | G | - | - | - | - | K | G | - | P | - | A | I | I | L | L | H | G | Y | A | E | T | S | H | M | W | L | P | L | I | P | L | L | - | A | K | N | - | H | T | V | I | A | P | D | L | R | G |
| 087 UniRef90\_A0A4Y9SAQ9\_19\_291 | Q | I | G | G | - | - | - | - | K | G | - | T | - | P | V | V | L | L | H | G | Y | T | Q | T | G | H | M | W | Q | P | I | M | A | E | L | - | A | K | T | - | H | T | V | I | V | P | D | L | R | G |
| 088 UniRef90\_A0A327RPK7\_10\_286 | V | I | A | G | - | - | - | - | S | G | - | D | - | P | I | V | L | L | H | G | W | P | Q | T | W | Y | E | W | K | D | I | I | P | T | L | - | A | K | S | - | Y | T | V | I | A | P | D | L | R | G |
| 089 UniRef90\_A0A239MTD5\_1\_269 | M | E | G | G | - | - | - | - | Q | G | - | S | - | P | V | V | M | I | H | G | F | P | Q | T | W | A | E | W | R | Q | Q | M | G | P | L | - | S | R | T | - | H | T | V | I | A | V | D | L | R | G |
| 090 UniRef90\_UPI00098F4FB4\_42\_302 | L | L | A | R | - | - | - | - | G | G | - | P | K | T | V | V | L | L | H | G | W | G | T | T | S | Y | M | W | R | Y | V | M | P | Q | L | - | A | A | L | G | F | T | V | I | A | P | D | L | R | G |
| 091 UniRef90\_A0A261TYY2\_38\_305 | V | I | G | G | - | - | - | - | Q | G | - | E | - | P | V | L | L | V | P | G | W | P | Q | T | W | Y | A | W | R | K | T | M | R | A | L | - | A | R | H | - | Y | T | V | V | A | I | D | P | P | G |
| 092 UniRef90\_UPI0015F81933\_27\_309 | V | E | G | G | R | P | - | - | S | G | - | P | - | A | V | V | L | L | A | G | F | P | Q | T | W | W | A | W | R | K | V | M | P | G | L | - | A | H | R | - | F | H | V | I | A | I | D | L | P | G |
| 093 UniRef90\_A0A0C7N0E5\_9\_278 | V | T | A | G | T | K | G | N | G | Q | - | P | - | P | I | I | L | L | A | G | F | P | E | S | W | Y | A | W | R | K | L | M | P | I | L | - | A | E | S | - | F | F | V | V | A | I | D | L | P | G |
| 094 UniRef90\_UPI00112BEE4D\_14\_300 | V | E | G | G | R | A | - | - | D | A | - | P | - | P | L | L | L | L | A | G | F | P | E | S | W | Y | A | W | R | H | V | L | P | L | L | - | A | P | H | - | H | R | L | L | A | P | D | L | P | G |
| 095 UniRef90\_A0A401Z9D4\_17\_277 | V | I | G | G | - | - | - | - | Q | G | - | F | - | P | V | L | L | L | H | G | W | P | Q | T | W | Y | A | W | R | K | I | M | P | A | L | - | A | E | K | - | Y | T | V | I | A | P | D | S | R | G |
| 096 UniRef90\_A0A5J6MKN6\_7\_281 | K | I | G | G | - | - | - | - | R | G | - | P | - | V | V | V | L | L | H | G | Y | A | Q | T | S | H | M | W | T | P | L | L | P | L | L | - | A | T | T | - | H | T | V | I | A | P | D | L | R | G |
| 097 UniRef90\_A0A4Q7XSL0\_10\_307 | R | V | G | G | - | - | - | - | N | G | - | P | - | A | V | V | L | I | H | G | F | G | D | T | G | D | M | W | A | P | L | A | V | A | L | - | A | K | E | - | H | R | V | I | V | P | D | L | R | G |
| 098 UniRef90\_UPI0016110D13\_9\_294 | V | V | G | G | A | D | - | - | A | P | - | E | - | I | I | V | L | L | A | G | F | P | E | S | W | Y | A | W | R | H | V | M | P | M | L | - | G | E | R | - | Y | R | V | L | A | I | D | L | P | G |
| 099 UniRef90\_A0A520GLB7\_17\_313 | R | I | G | G | - | - | - | - | S | G | - | P | - | A | V | V | L | L | H | G | Y | A | E | T | G | H | M | W | N | P | V | M | P | L | L | - | A | K | T | - | H | T | V | I | V | P | D | L | R | G |
| 100 UniRef90\_A0A252EMP1\_9\_266 | V | R | A | G | - | - | - | - | K | G | - | E | - | P | V | L | L | V | H | G | W | P | Q | T | W | Y | E | W | H | R | V | I | P | H | L | - | V | E | A | G | H | E | V | I | A | V | D | M | R | G |
| 101 UniRef90\_UPI001668C4B3\_34\_306 | V | V | G | G | - | - | - | - | D | G | - | P | - | P | L | L | L | L | A | G | W | P | Q | T | W | Y | A | W | R | L | V | M | P | A | L | - | A | R | D | - | F | R | V | V | A | V | D | P | R | G |
| 102 UniRef90\_UPI00135BC32E\_12\_265 | V | R | A | G | - | - | - | - | E | G | - | P | - | P | L | L | L | L | H | G | W | P | Q | T | W | Y | E | W | R | E | V | I | G | P | L | - | A | Q | Q | - | Y | T | V | I | A | P | D | L | R | G |
| 103 UniRef90\_A0A2V9DES3\_17\_290 | L | V | G | G | - | - | - | - | K | G | - | D | - | P | V | V | L | L | H | G | Y | A | E | T | S | H | M | W | R | P | L | I | A | E | L | - | A | K | T | - | H | T | V | I | A | P | D | L | R | G |
| 104 UniRef90\_A0A6P0DAX4\_34\_314 | L | L | A | Q | - | - | - | - | G | G | - | P | Q | T | V | V | L | L | H | G | W | G | T | T | S | Y | M | W | R | Y | V | M | P | E | L | - | V | A | R | G | Y | T | V | L | A | P | D | L | R | G |
| 105 UniRef90\_A0A1M5KPU7\_13\_307 | L | I | A | G | - | - | - | - | R | G | - | D | - | P | V | V | L | L | H | G | Y | A | E | T | S | H | M | W | L | P | L | I | A | K | L | - | A | D | K | - | H | T | V | I | A | P | D | L | R | G |
| 106 UniRef90\_A0A2I8DLX4\_17\_295 | V | E | G | G | - | - | - | - | E | G | - | P | - | A | L | I | L | L | A | G | F | P | Q | S | W | Y | A | W | R | R | V | M | P | L | L | - | A | P | H | - | F | R | V | Y | A | V | D | L | P | G |
| 107 UniRef90\_A0A5S4WMZ6\_36\_313 | V | V | G | G | P | A | - | - | S | G | - | E | - | M | I | I | L | L | H | G | W | P | Q | T | W | Y | T | W | R | H | V | M | P | A | L | - | A | A | A | G | F | R | V | V | A | V | D | Y | R | G |
| 108 UniRef90\_UPI0003744601\_9\_294 | V | I | A | G | - | - | - | - | S | G | - | D | - | P | V | L | L | L | H | G | W | P | E | T | W | Y | A | W | R | K | V | I | P | A | L | - | A | D | R | - | F | T | V | I | A | P | D | M | R | G |
| 109 UniRef90\_A0A0H3KYM5\_9\_289 | V | T | G | G | R | E | - | - | D | G | - | F | - | P | L | V | L | L | A | G | F | P | E | S | W | F | A | W | R | H | V | M | L | P | L | - | A | D | Q | - | Y | R | I | I | A | V | D | L | P | G |
| 110 UniRef90\_UPI001616C592\_11\_307 | V | T | G | G | - | - | - | - | D | G | - | P | - | P | V | L | L | L | G | G | W | P | Q | F | W | Y | Q | W | R | L | I | M | P | A | L | - | A | E | D | - | H | T | V | I | A | V | D | P | R | G |
| 111 UniRef90\_A0A1U9ZZS0\_24\_308 | V | E | G | G | Q | P | - | - | A | D | - | P | - | A | V | V | L | L | A | G | F | P | Q | T | W | W | A | W | R | K | V | M | P | R | L | - | A | A | R | - | F | R | V | I | A | I | D | L | P | G |
| 112 UniRef90\_UPI00146E50DC\_33\_308 | L | V | A | G | - | - | - | - | K | G | - | E | - | P | M | V | L | L | H | G | Y | A | Q | T | S | H | M | W | R | P | L | I | K | E | L | - | A | K | T | - | H | L | V | I | A | P | D | M | R | G |
| 113 UniRef90\_C3KLY5\_13\_287 | V | S | G | G | R | A | - | - | D | G | - | E | - | V | I | L | L | F | A | G | F | P | Q | S | W | Y | A | W | R | K | I | L | P | I | L | - | G | A | R | - | Y | R | I | I | A | P | D | L | P | G |
| 114 UniRef90\_A0A2V6STQ7\_27\_303 | K | I | G | G | - | - | - | - | E | G | - | P | - | G | V | V | L | L | H | G | Y | A | Q | T | S | H | M | W | T | P | L | M | P | R | L | - | A | T | S | - | H | T | V | M | A | P | D | L | R | G |
| 115 UniRef90\_UPI0008D5DA8A\_10\_290 | A | V | A | G | - | - | - | - | S | G | - | T | - | P | V | L | L | I | P | G | W | P | Q | T | W | Y | T | W | R | H | I | L | A | A | L | - | A | Q | A | G | Y | Q | A | I | A | V | D | P | P | G |
| 116 UniRef90\_A0A3N2H8D2\_16\_299 | V | E | G | G | R | T | - | - | G | G | - | P | - | A | V | V | L | L | A | G | F | P | Q | T | W | W | A | W | R | K | V | M | P | R | L | - | A | E | R | - | F | H | V | I | A | I | D | L | P | G |
| 117 UniRef90\_A0A379Z3Q1\_16\_292 | V | T | L | G | - | - | - | - | E | G | - | Q | - | P | V | L | L | I | P | G | W | P | Q | T | W | F | T | W | R | H | V | M | Q | A | L | - | A | A | A | G | F | K | A | I | A | V | D | P | P | G |
| 118 UniRef90\_A0A4R5QBW7\_8\_277 | V | T | A | G | - | - | - | - | A | G | - | P | - | A | V | L | L | L | H | G | W | P | Q | S | W | R | M | W | E | A | I | I | P | G | L | - | A | A | R | - | H | R | V | V | A | P | D | L | R | G |
| 119 UniRef90\_A0A163VVR8\_16\_291 | V | V | A | G | - | - | - | - | E | G | - | R | - | P | V | L | L | I | P | G | W | P | Q | T | W | Y | T | W | R | H | V | M | Q | A | L | - | A | A | A | G | F | Q | A | I | A | V | D | P | P | G |
| 120 UniRef90\_G0FSK7\_29\_305 | V | V | G | G | - | - | - | - | D | G | - | P | - | P | L | L | L | I | P | G | W | P | Q | T | W | Y | A | W | R | L | L | M | P | E | L | - | A | R | D | - | F | T | V | V | A | V | D | P | R | G |
| 121 UniRef90\_A0A4Q7FLU1\_10\_276 | V | T | A | G | - | - | - | - | S | G | - | P | - | P | L | V | L | L | H | G | W | P | Q | T | W | Y | T | W | R | H | V | I | G | E | L | - | A | D | Q | - | Y | F | I | V | A | P | D | L | R | G |
| 122 UniRef90\_A0A2E3NGG1\_11\_269 | V | R | A | G | - | - | - | - | E | G | - | P | - | A | V | A | L | L | H | G | W | P | Q | T | W | Y | M | W | R | K | V | I | P | A | L | - | A | E | R | - | Y | T | V | L | A | P | D | L | R | G |
| 123 UniRef90\_A0A2J9ERN8\_9\_287 | V | E | G | G | A | P | - | - | G | E | - | P | - | T | V | V | L | M | A | G | F | P | E | S | W | Y | A | W | R | H | V | M | P | Q | L | - | G | R | R | - | F | H | I | I | A | L | D | L | P | G |
| 124 UniRef90\_UPI001677C581\_8\_288 | V | T | A | G | - | - | - | - | S | G | - | E | - | P | V | L | L | I | P | G | W | P | Q | T | W | F | A | W | R | H | V | M | Q | A | L | - | A | D | K | G | F | M | A | I | A | V | D | P | P | G |
| 125 UniRef90\_UPI0003039C4F\_8\_269 | V | A | A | G | - | - | - | - | S | G | - | P | - | T | V | V | L | L | H | G | W | P | Q | T | W | Y | M | W | R | D | V | I | H | G | L | - | A | S | R | - | Y | R | V | I | A | P | D | L | R | G |
| 126 UniRef90\_A0A1B1YXM9\_17\_276 | L | T | A | G | - | - | - | - | D | G | - | P | - | A | L | L | L | L | H | G | W | P | Q | S | S | H | E | W | R | H | L | I | P | T | L | - | A | Q | R | - | H | R | V | I | A | P | D | L | R | G |
| 127 UniRef90\_A0A534ZXC6\_31\_288 | L | T | A | G | - | - | - | - | T | G | - | P | - | A | V | I | L | L | H | G | Y | T | Q | T | S | R | M | W | K | P | V | I | P | K | L | - | A | T | R | - | F | T | V | I | A | P | D | L | P | G |
| 128 UniRef90\_A0A2X1TAZ7\_42\_319 | V | T | A | G | - | - | - | - | K | G | - | E | - | P | V | L | L | I | P | G | W | P | Q | T | W | Y | T | W | R | Y | V | M | T | E | L | - | A | A | Q | G | Y | M | A | I | A | V | D | P | P | G |
| 129 UniRef90\_A0A4Q5QRH4\_8\_282 | V | A | G | G | V | E | - | - | T | A | - | T | - | T | V | V | L | L | A | G | F | P | E | S | W | Y | A | W | R | H | V | M | P | V | L | - | G | E | R | - | F | R | V | I | A | I | D | L | P | G |
| 130 UniRef90\_A0A0D6HQT0\_17\_294 | V | E | G | G | - | - | - | - | A | G | - | P | - | A | L | I | L | L | A | G | F | P | Q | S | W | Y | A | W | R | R | V | M | P | L | L | - | A | P | H | - | F | R | L | Y | A | I | D | L | P | G |
| 131 UniRef90\_A0A1G4JCM6\_9\_275 | V | T | A | G | Q | K | - | - | G | K | - | P | - | A | V | V | L | L | A | G | F | P | Q | S | W | Y | A | W | R | K | V | I | P | Y | L | - | S | Q | S | - | Y | Q | V | F | A | I | D | L | P | G |
| 132 UniRef90\_UPI001269E708\_28\_301 | V | E | G | G | R | P | - | - | A | G | - | P | - | V | V | V | L | L | A | G | F | P | Q | T | W | W | A | W | R | K | V | M | P | L | L | - | A | E | R | - | F | R | V | I | A | I | D | L | P | G |
| 133 UniRef90\_A0A538SUN7\_3\_303 | R | V | G | G | - | - | - | - | K | G | - | P | - | A | V | V | L | L | H | G | F | G | D | T | G | D | M | W | A | L | L | A | A | K | L | - | M | K | D | - | H | T | V | I | V | P | D | L | R | G |
| 134 UniRef90\_UPI001646B9D9\_15\_288 | A | V | M | G | - | - | - | - | E | G | - | E | - | P | V | L | L | V | P | G | W | P | Q | T | W | Y | A | W | R | H | V | M | P | I | L | - | A | A | R | G | F | R | A | I | A | I | D | P | P | G |
| 135 UniRef90\_K9DQK6\_17\_307 | R | S | G | G | - | - | - | - | K | G | - | P | - | A | V | V | L | I | H | G | F | G | D | T | G | D | M | W | G | P | L | A | A | R | L | - | A | R | N | - | H | T | V | I | V | P | D | L | R | G |
| 136 UniRef90\_A0A2N9BM66\_25\_297 | V | V | S | G | - | - | - | - | D | G | - | P | - | P | L | L | L | L | P | G | W | P | Q | F | W | Y | S | W | R | L | V | M | P | A | L | - | A | E | H | - | F | T | V | I | A | P | D | L | R | G |
| 137 UniRef90\_A0A370LBV7\_5\_266 | L | A | A | G | - | - | - | - | Q | G | - | E | - | P | V | L | L | L | H | G | Y | A | Q | N | S | H | M | W | R | P | L | I | A | K | L | - | A | E | T | - | H | L | V | I | A | P | D | L | R | G |
| 138 UniRef90\_A0A2V9ZM06\_3\_258 | M | T | A | G | - | - | - | - | H | G | - | T | - | P | L | I | L | L | H | G | Y | A | E | T | S | L | M | W | K | P | I | I | P | L | L | - | A | E | R | - | F | T | V | I | A | P | D | L | P | G |
| 139 UniRef90\_UPI00101F418B\_26\_310 | V | E | G | G | - | - | - | - | Q | G | - | R | - | P | L | L | L | I | P | G | W | P | Q | S | W | Y | T | W | R | H | V | M | P | A | L | - | A | A | S | D | R | R | V | I | A | I | D | P | R | G |
| 140 UniRef90\_A0A4V2YYI8\_27\_305 | V | I | G | G | - | - | - | - | E | G | - | P | - | A | L | L | L | L | S | G | W | P | Q | T | W | Y | A | W | R | F | L | M | P | A | L | - | A | R | D | - | F | T | V | V | A | A | D | P | R | G |
| 141 UniRef90\_UPI001487D2FB\_8\_292 | M | V | G | G | - | - | - | - | K | G | - | P | - | A | V | V | L | L | H | G | F | G | D | T | G | A | M | W | S | P | L | A | A | E | L | - | A | A | R | - | H | T | V | V | V | P | D | L | R | G |
| 142 UniRef90\_B9XAH7\_320\_607 | V | V | A | G | - | - | - | - | D | G | - | E | - | P | V | V | L | V | P | G | W | P | E | S | W | F | A | W | R | R | V | I | P | E | L | - | V | R | S | G | R | R | V | Y | A | I | D | P | R | G |
| 143 UniRef90\_UPI0013696204\_44\_306 | V | T | G | G | - | - | - | - | D | G | - | P | - | P | L | L | L | L | G | G | W | P | Q | T | W | Y | A | W | R | L | L | M | P | A | L | - | A | R | D | - | H | Q | V | V | A | V | D | P | R | G |
| 144 UniRef90\_W5WIM6\_13\_292 | V | T | G | G | - | - | - | - | D | G | - | P | - | P | L | L | L | L | A | G | W | P | Q | T | W | Y | A | W | R | E | L | M | P | A | L | - | A | R | D | - | F | S | V | V | A | A | D | P | R | G |
| 145 UniRef90\_A0A2X2GG63\_10\_290 | V | T | L | G | - | - | - | - | E | G | - | Q | - | P | V | L | L | I | P | G | W | P | Q | T | W | Y | T | W | R | H | V | M | Q | A | L | - | A | A | A | G | F | K | A | I | A | V | D | P | P | G |
| 146 UniRef90\_A0A2E2YI39\_6\_274 | V | L | A | G | - | - | - | - | E | G | - | P | - | P | V | V | L | L | H | G | W | P | Q | T | W | W | E | W | R | H | I | I | P | V | L | - | A | K | N | - | Y | T | V | I | A | P | D | L | R | G |
| 147 UniRef90\_A0A2W7G3K6\_24\_312 | V | E | G | G | - | - | - | - | Q | G | - | A | - | P | V | L | L | L | P | G | W | P | Q | S | W | Y | V | W | R | Y | V | M | P | L | L | V | A | A | G | - | R | R | V | I | A | V | D | P | R | G |
| 148 UniRef90\_A0A6B2RWJ8\_28\_298 | V | E | G | G | L | P | - | - | N | G | - | P | - | T | V | V | L | L | A | G | F | P | Q | T | W | W | A | W | R | K | V | M | P | G | L | - | A | E | R | - | F | H | V | I | A | I | D | L | P | G |
| 149 UniRef90\_A0A537A198\_6\_270 | V | T | A | G | - | - | - | - | S | G | - | V | - | P | I | V | L | L | H | G | W | P | Q | T | W | Y | E | W | R | K | L | I | P | L | L | - | A | D | R | - | Y | R | L | V | A | P | D | L | P | G |
| 150 UniRef90\_UPI00135F2885\_48\_334 | V | R | G | G | - | - | - | - | E | G | - | E | - | P | L | I | L | L | H | G | W | P | T | T | W | W | E | W | H | K | I | M | P | G | L | - | A | E | T | - | F | D | V | I | A | V | D | T | R | G |

  
  

|  |  |  |  |  |  |  |  |  |  |  |  |  |  |  |  |  |  |  |  |  |  |  |  |  |  |  |  |  |  |  |  |  |  |  |  |  |  |  |  |  |  |  |  |  |  |  |  |  |  |  |
| --- | --- | --- | --- | --- | --- | --- | --- | --- | --- | --- | --- | --- | --- | --- | --- | --- | --- | --- | --- | --- | --- | --- | --- | --- | --- | --- | --- | --- | --- | --- | --- | --- | --- | --- | --- | --- | --- | --- | --- | --- | --- | --- | --- | --- | --- | --- | --- | --- | --- | --- |
| **001 Input\_protein\_seq** | L | G | Q | S | - | - | - | E | P | - | P | K | - | - | T | G | Y | S | G | E | Q | V | A | V | Y | L | H | K | L | A | - | R | Q | F | S | P | - | - | D | R | P | F | D | L | V | A | H | D | I | G |
| 002 UniRef90\_A0A2G0Y5Q3\_5\_314 | L | G | Q | S | - | - | - | E | P | - | P | K | - | - | - | A | Y | S | G | E | D | V | A | P | Y | L | Y | K | L | A | - | K | N | F | S | P | - | - | D | K | P | F | N | L | V | A | H | D | I | G |
| 003 UniRef90\_A0A172YJZ3\_2\_306 | L | G | Q | S | - | - | - | A | V | - | P | P | - | - | - | S | Y | R | A | T | D | V | S | A | L | L | Y | E | L | A | - | M | R | F | S | G | - | - | G | E | K | F | D | L | V | A | H | D | I | G |
| 004 UniRef90\_A0A4Z1C7D3\_9\_314 | L | G | L | S | - | - | - | S | Q | - | P | P | - | - | - | S | Y | A | G | Q | D | V | A | P | I | L | H | K | L | A | - | K | E | F | S | P | - | - | D | A | P | F | D | L | V | A | H | D | I | G |
| 005 UniRef90\_A0A0A3Z1S4\_7\_319 | L | G | L | S | - | - | - | Q | P | - | P | A | - | - | T | S | Y | S | G | E | D | V | A | N | T | L | Y | K | L | A | - | D | S | L | R | G | - | - | D | Q | K | F | T | L | V | A | H | D | I | G |
| 006 UniRef90\_A0A329J6I4\_9\_315 | L | G | E | S | - | - | - | Q | P | - | P | K | - | - | T | G | Y | S | G | T | A | V | S | K | Y | L | F | D | L | A | - | T | Q | L | S | G | - | - | N | Q | P | F | D | L | V | A | H | D | I | G |
| 007 UniRef90\_A0A4V1G750\_8\_314 | L | G | Q | S | - | - | - | A | P | - | A | P | - | - | - | S | L | R | G | R | D | V | A | P | L | L | F | K | L | A | - | T | R | F | S | G | - | - | E | Q | K | F | D | L | V | A | H | D | I | G |
| 008 UniRef90\_T2L220\_17\_309 | L | G | Q | S | - | - | - | A | V | - | P | P | - | - | - | S | Y | R | A | T | D | I | A | V | L | L | Y | K | L | A | - | K | Q | F | S | G | - | - | N | Q | T | F | D | L | V | A | H | D | I | G |
| 009 UniRef90\_UPI000C2FE79C\_12\_314 | L | G | Q | S | - | - | - | A | V | - | P | K | - | - | - | S | Y | R | A | I | D | V | A | P | L | L | Y | Q | L | A | - | K | N | I | S | G | - | - | G | Q | R | F | D | L | V | A | H | D | I | G |
| 010 UniRef90\_UPI00102FACD2\_13\_314 | L | G | D | S | - | - | - | E | V | - | P | P | - | - | - | S | Y | R | A | T | D | V | A | P | L | L | Y | K | L | A | - | M | Q | F | S | G | - | - | G | K | P | F | D | L | V | S | H | D | I | G |
| 011 UniRef90\_UPI0014749FF6\_10\_312 | L | G | L | S | - | - | - | E | P | - | L | K | - | - | T | T | Y | T | G | E | D | V | S | P | Y | I | Y | A | F | A | - | K | R | F | S | P | - | - | D | A | P | F | K | L | V | A | H | D | I | G |
| 012 UniRef90\_A0A3M4V4S1\_41\_332 | L | G | L | S | - | - | - | A | P | - | P | K | - | - | T | T | Y | T | G | V | D | I | S | E | Y | L | Y | K | L | A | - | K | Q | I | S | A | - | - | D | K | P | F | Y | L | V | A | H | D | I | G |
| 013 UniRef90\_A0A3L8C981\_31\_324 | L | G | L | S | - | - | - | Q | A | - | P | Q | - | - | T | S | Y | T | G | V | D | V | S | E | Y | L | Y | K | L | A | - | K | R | L | S | P | - | - | E | K | P | F | Y | L | V | A | H | D | I | G |
| 014 UniRef90\_A0A1X0N2L9\_32\_323 | L | G | L | S | - | - | - | G | - | - | P | A | - | - | Q | T | F | T | G | V | D | I | S | E | Y | L | H | K | L | A | - | K | R | V | S | A | - | - | G | K | A | F | Y | L | V | A | H | D | I | G |
| 015 UniRef90\_UPI00166CAA4C\_15\_313 | F | G | E | S | - | - | - | D | V | - | P | T | - | - | A | G | F | D | K | A | T | T | A | M | R | V | H | A | L | M | - | K | A | I | G | V | - | - | - | E | S | A | N | V | V | G | H | D | I | G |
| 016 UniRef90\_UPI001661A3FF\_47\_334 | A | G | Q | S | - | - | - | D | A | - | P | P | - | - | S | G | Y | D | K | K | T | M | A | A | E | L | H | A | L | L | - | V | S | L | G | K | - | - | D | D | D | V | R | I | V | G | H | D | I | G |
| 017 UniRef90\_A0A1H5MZC9\_42\_331 | A | G | L | S | - | - | - | D | A | - | P | V | - | - | S | G | Y | D | K | K | S | M | A | A | D | I | H | G | L | L | - | A | Q | I | G | H | - | - | D | K | D | I | R | L | V | G | H | D | I | G |
| 018 UniRef90\_UPI000690E8EC\_26\_310 | A | G | Q | S | - | - | - | D | A | - | P | A | - | - | A | G | Y | D | K | K | S | M | A | A | D | I | H | A | M | L | - | A | S | I | G | R | - | - | D | H | D | I | R | L | V | G | H | D | I | G |
| 019 UniRef90\_R4LNI7\_31\_317 | S | G | R | S | - | - | - | D | A | - | P | A | - | - | T | G | Y | D | K | K | T | L | A | A | D | L | Y | G | L | L | - | V | Q | L | G | R | - | - | Q | H | H | I | R | L | A | G | H | D | I | G |
| 020 UniRef90\_A0A4R2C838\_67\_350 | A | G | L | S | - | - | - | D | A | - | P | A | S | S | A | A | Y | T | K | K | A | M | A | A | D | I | H | A | L | M | - | V | E | L | G | L | - | - | S | R | N | I | R | I | V | A | H | D | I | G |
| 021 UniRef90\_A0A2P9HD85\_25\_319 | L | G | Q | S | - | - | - | E | P | - | S | A | - | - | K | R | Y | D | A | E | Q | M | S | A | Y | L | L | G | A | I | - | K | S | L | T | D | - | - | N | Q | P | F | T | Y | V | C | H | D | L | C |
| 022 UniRef90\_UPI000D14D87C\_43\_332 | A | G | R | S | - | - | - | D | T | - | P | V | - | - | S | G | Y | D | K | K | T | M | A | A | K | L | H | A | L | L | - | V | S | L | G | K | - | - | D | Q | D | V | N | V | V | G | H | D | I | G |
| 023 UniRef90\_A0A1Q4ZL08\_6\_289 | A | G | G | S | - | - | - | D | A | - | P | A | - | - | G | G | Y | D | K | K | T | L | A | R | D | V | H | D | L | L | - | A | Q | L | G | L | - | - | E | R | D | I | C | L | V | G | H | D | I | G |
| 024 UniRef90\_A0A1I2GGE7\_30\_316 | A | G | K | S | - | - | - | D | A | - | P | A | - | - | G | G | Y | D | K | K | T | M | A | A | D | I | H | G | L | L | - | V | K | L | G | L | - | - | N | R | D | I | R | L | V | G | H | D | I | G |
| 025 UniRef90\_UPI0005580D51\_18\_305 | A | G | G | S | - | - | - | D | A | - | P | A | - | - | G | G | Y | D | K | K | T | M | A | D | D | I | H | R | L | L | - | V | K | L | G | L | - | - | N | H | D | I | R | L | V | G | H | D | I | G |
| 026 UniRef90\_A0A2W2F1P0\_6\_289 | F | G | D | S | - | - | - | D | A | - | P | S | - | - | G | G | Y | D | K | K | T | L | A | G | D | V | H | G | L | L | - | T | S | L | G | L | - | - | D | R | D | L | R | L | V | G | H | D | I | G |
| 027 UniRef90\_UPI0010F9F40F\_6\_288 | F | G | G | S | - | - | - | D | A | - | P | A | - | - | D | G | Y | D | K | K | T | L | A | G | D | L | H | G | L | L | - | T | S | L | G | L | - | - | D | R | D | L | H | L | V | G | H | D | L | G |
| 028 UniRef90\_A0A3E2YQE6\_6\_289 | F | G | D | S | - | - | - | D | A | - | P | A | - | - | A | G | Y | D | K | K | T | L | A | A | D | L | H | G | L | L | - | D | E | L | G | V | - | - | T | G | D | I | R | L | V | G | H | D | V | G |
| 029 UniRef90\_A0A1A9ACL3\_6\_288 | F | G | D | S | - | - | - | D | A | - | P | P | - | - | G | G | Y | D | K | K | T | V | A | A | E | L | H | G | L | L | - | T | G | L | G | L | - | - | A | G | E | I | R | L | V | G | H | D | L | G |
| 030 UniRef90\_UPI00174B5D19\_38\_323 | T | G | N | S | - | - | - | G | V | - | P | A | - | - | T | G | Y | D | T | V | Q | L | A | E | D | V | H | A | L | L | - | V | K | L | G | V | - | - | N | N | G | V | Q | V | V | A | H | D | V | G |
| 031 UniRef90\_A0A495JCH5\_50\_333 | A | G | G | S | - | - | - | D | A | - | P | A | - | - | A | G | Y | D | K | K | T | L | A | D | D | V | H | K | L | L | - | V | T | L | H | R | - | - | D | Q | D | I | R | L | V | G | H | D | I | G |
| 032 UniRef90\_UPI0013D14F7B\_11\_294 | A | G | R | S | - | - | - | D | A | - | P | P | - | - | A | G | Y | D | K | K | T | M | A | A | D | I | H | G | L | L | - | V | A | L | G | L | - | - | D | S | R | V | R | M | V | G | H | D | I | G |
| 033 UniRef90\_UPI00036EC6E3\_56\_342 | A | G | L | S | - | - | - | D | A | - | P | A | P | A | A | N | Y | T | K | K | A | M | A | A | D | I | Y | A | L | M | - | V | K | L | G | L | - | - | S | H | D | L | R | I | V | A | H | D | I | G |
| 034 UniRef90\_A0A1H3P9Q0\_6\_290 | S | G | G | S | - | - | - | D | A | - | P | A | - | - | E | G | Y | D | K | K | T | L | A | G | D | V | H | G | L | L | - | T | Q | L | G | L | - | - | D | R | D | V | S | L | V | G | H | D | I | G |
| 035 UniRef90\_A0A385B2U0\_41\_332 | T | G | D | S | - | - | - | S | V | - | P | Q | - | - | Q | G | Y | D | T | A | Q | L | A | D | D | V | H | E | L | L | - | T | R | L | N | L | - | - | N | K | G | V | Q | I | V | A | H | D | I | G |
| 036 UniRef90\_UPI001430CB85\_25\_306 | Q | G | D | S | - | - | - | D | R | - | P | L | - | - | D | G | Y | D | T | K | S | L | A | T | V | L | H | A | L | L | - | G | K | L | G | I | - | - | - | Q | R | Y | Y | L | A | A | H | D | V | G |
| 037 UniRef90\_A0A109IHW8\_6\_288 | F | G | G | S | - | - | - | D | A | - | P | P | - | - | D | G | Y | D | K | L | T | V | A | A | D | L | H | G | L | L | - | T | G | L | G | L | - | - | A | D | D | I | R | L | V | G | H | D | V | G |
| 038 UniRef90\_A0A2T6L0M5\_14\_298 | T | G | D | S | - | - | - | E | V | - | T | S | - | - | D | G | Y | D | T | V | Q | M | A | D | D | V | H | A | L | L | - | T | Q | L | G | L | - | - | N | D | G | I | Q | I | V | A | H | D | I | G |
| 039 UniRef90\_A0A1C4Z0V4\_6\_288 | F | G | D | S | - | - | - | D | A | - | P | P | - | - | G | G | Y | D | K | K | T | L | A | A | D | L | Y | G | L | L | - | T | D | L | G | L | - | - | A | G | G | I | R | L | V | G | H | D | V | G |
| 040 UniRef90\_A0A4U3M272\_82\_365 | A | G | L | S | - | - | - | D | A | - | P | A | S | S | A | A | Y | T | K | K | A | M | A | A | D | I | Y | A | L | M | - | V | K | L | G | L | - | - | S | H | N | L | R | V | V | G | H | D | I | G |
| 041 UniRef90\_UPI00052728DD\_22\_294 | Q | G | D | S | - | - | - | D | R | - | P | L | - | - | D | G | Y | D | T | Q | T | L | A | T | R | L | H | A | L | L | - | R | K | L | D | V | - | - | - | Q | R | C | Y | L | V | A | H | D | V | G |
| 042 UniRef90\_A0A6B8MSH7\_52\_336 | L | G | Q | S | - | - | - | S | P | I | K | N | - | - | E | D | Y | S | A | Q | N | V | S | T | I | L | L | S | A | I | - | K | K | I | A | G | - | - | D | E | P | I | Y | F | V | T | H | D | L | G |
| 043 UniRef90\_I4VSM6\_28\_303 | Q | G | D | S | - | - | - | D | R | - | P | D | - | - | G | G | Y | D | T | G | A | L | A | A | K | V | H | A | L | L | - | Q | Q | L | G | V | - | - | - | N | R | Y | G | L | A | A | H | D | V | G |
| 044 UniRef90\_A0A1I3BFK1\_40\_314 | Q | G | D | S | - | - | - | D | R | - | P | A | - | - | T | G | Y | D | T | M | T | L | A | T | T | V | H | K | L | L | - | Q | Q | L | G | T | - | - | - | K | R | Y | F | L | A | A | H | D | V | G |
| 045 UniRef90\_A0A1Z4J856\_42\_316 | L | G | D | S | - | - | - | S | K | - | P | L | - | - | T | G | Y | D | A | R | T | V | A | D | D | I | Y | Q | L | V | - | G | K | L | G | F | - | - | - | K | R | I | F | L | V | G | H | D | I | G |
| 046 UniRef90\_A0A0M4FVH2\_27\_314 | Q | G | D | S | - | - | - | D | R | - | P | A | - | - | D | G | Y | D | T | K | T | L | A | T | T | V | H | K | F | L | - | Q | Q | L | G | T | - | - | - | K | R | Y | F | L | A | A | H | D | V | G |
| 047 UniRef90\_UPI001032135D\_25\_299 | Q | G | D | S | - | - | - | D | R | - | P | E | - | - | T | G | Y | D | T | R | S | L | A | E | K | V | H | G | L | L | - | R | Q | L | R | I | - | - | - | H | R | Y | F | L | A | A | H | D | V | G |
| 048 UniRef90\_D5WL63\_24\_300 | Q | G | D | S | - | - | - | D | R | - | P | M | - | - | D | G | Y | D | T | Q | T | L | A | S | H | V | H | G | L | L | - | Q | Q | L | G | V | - | - | - | S | R | Y | F | L | A | G | H | D | I | G |
| 049 UniRef90\_A0A1H3E943\_18\_291 | Q | G | S | S | - | - | - | D | K | - | P | A | - | - | N | G | Y | D | T | R | T | T | G | D | R | I | H | K | L | M | - | Q | V | M | G | N | - | - | - | N | R | Y | S | I | L | A | H | D | I | G |
| 050 UniRef90\_A0A346N7R3\_30\_317 | A | G | G | S | - | - | - | S | K | - | P | E | - | - | S | G | Y | T | K | K | N | M | A | Q | D | I | H | A | L | T | - | A | A | L | G | F | - | - | - | H | Q | V | K | I | V | G | H | D | I | G |
| 051 UniRef90\_A0A537Q000\_40\_318 | M | G | D | S | - | - | - | S | R | - | P | E | - | - | T | G | Y | D | T | E | T | V | A | K | D | L | S | D | L | M | - | Q | R | L | G | A | - | - | - | D | H | Y | S | V | I | G | H | D | V | G |
| 052 UniRef90\_E6V5X5\_24\_320 | A | G | N | S | - | - | - | S | K | - | P | E | - | - | T | G | Y | G | K | K | N | M | A | V | D | I | H | E | L | V | - | R | S | L | G | I | - | - | - | R | S | V | S | I | V | G | H | D | I | G |
| 053 UniRef90\_A0A484PNM3\_14\_291 | L | G | D | S | - | - | - | D | K | - | P | A | - | - | G | G | Y | D | T | R | A | V | A | Q | R | L | H | D | F | T | - | R | A | L | G | W | - | - | - | D | G | F | H | F | V | G | H | D | V | G |
| 054 UniRef90\_UPI0011BDEFB8\_30\_311 | Q | G | D | S | - | - | - | D | R | - | P | D | - | - | S | G | Y | D | T | Q | A | L | A | E | K | I | H | G | L | L | - | A | Q | L | K | I | - | - | - | D | R | Y | F | L | A | A | H | D | V | G |
| 055 UniRef90\_A0A1H2UZ41\_16\_293 | Q | G | D | S | - | - | - | D | K | - | P | I | - | - | D | G | Y | D | T | R | T | T | G | D | R | V | H | A | L | A | - | K | T | L | G | F | - | - | - | E | R | Y | H | I | G | S | H | D | I | G |
| 056 UniRef90\_A0A1M5ZT81\_29\_302 | Q | G | D | S | - | - | - | D | R | - | P | E | - | - | G | G | Y | D | T | Q | S | L | A | L | T | L | H | G | M | L | - | Q | Q | L | G | V | - | - | - | A | R | C | R | L | A | A | H | D | V | G |
| 057 UniRef90\_A0A135GL34\_14\_283 | L | G | D | S | G | V | P | E | K | - | P | L | - | - | T | G | Y | D | K | K | T | V | A | Q | D | I | H | Q | L | V | - | E | K | L | E | L | - | - | - | G | P | V | H | V | A | G | H | D | H | G |
| 058 UniRef90\_A0A484UEV2\_23\_280 | L | G | D | S | - | - | - | D | K | - | P | A | - | - | G | G | Y | D | T | R | A | V | A | Q | R | L | H | D | F | T | - | R | A | L | G | W | - | - | - | D | R | F | H | Y | V | G | H | D | V | G |
| 059 UniRef90\_A0A495NUG6\_51\_333 | A | G | Q | S | - | - | - | G | A | - | P | R | - | - | S | G | Y | D | K | V | S | M | A | K | S | I | H | G | M | L | - | S | A | A | G | H | - | - | D | T | N | V | R | M | V | G | H | D | I | G |
| 060 UniRef90\_A0A1M5MA45\_15\_292 | L | G | E | S | - | - | - | D | K | - | P | A | - | - | G | G | Y | D | K | K | T | M | A | S | D | I | H | Q | L | V | - | V | S | L | G | L | - | - | - | K | R | I | K | L | V | G | H | D | I | G |
| 061 UniRef90\_UPI000EF95C7F\_30\_312 | S | G | L | S | - | - | - | D | K | - | P | E | - | - | S | G | Y | D | S | T | T | S | A | A | E | F | H | R | L | M | - | E | I | L | G | H | - | - | - | D | R | F | S | V | I | G | H | D | I | G |
| 062 UniRef90\_L9WLS3\_15\_271 | L | G | D | S | - | - | - | E | T | - | P | A | - | - | S | G | Y | D | K | D | T | V | A | T | D | V | R | E | L | V | - | H | A | L | G | H | - | G | D | E | R | I | A | L | V | G | H | D | W | G |
| 063 UniRef90\_UPI0005D31B04\_21\_288 | F | G | D | S | - | - | - | D | K | - | P | G | - | - | S | G | Y | D | K | R | T | V | A | Q | D | I | H | Q | L | V | - | H | H | L | G | F | - | - | - | H | E | I | N | L | V | G | H | D | I | G |
| 064 UniRef90\_UPI001408C682\_10\_298 | N | G | D | S | - | - | - | E | K | - | P | A | - | - | S | G | Y | D | K | K | T | M | A | T | D | I | H | E | L | V | - | K | T | I | S | K | - | - | - | E | P | A | A | V | V | G | H | D | I | G |
| 065 UniRef90\_A0A1Y6KKD8\_22\_314 | Q | G | D | S | - | - | - | D | R | - | P | A | - | - | D | G | Y | D | T | R | T | L | A | T | F | V | H | R | L | L | - | R | Q | L | G | V | - | - | - | T | S | H | H | L | V | G | H | D | I | G |
| 066 UniRef90\_A0A0X3RYG7\_65\_349 | A | G | Q | S | - | - | - | Q | V | - | T | K | - | - | S | G | Y | D | A | V | Q | L | S | K | D | V | H | Q | L | L | - | T | Q | L | K | L | - | - | N | N | G | V | Q | V | V | A | H | D | I | G |
| 067 UniRef90\_A0A2T0T762\_22\_302 | A | G | L | S | - | - | - | D | K | - | P | D | - | - | G | G | Y | D | A | G | T | L | A | A | D | L | V | A | L | M | - | S | A | L | G | H | - | - | - | D | R | F | D | V | V | G | H | D | I | G |
| 068 UniRef90\_A0A2V6QCX2\_10\_286 | A | G | G | S | - | - | - | A | K | - | P | A | - | - | T | G | Y | D | K | R | N | M | A | V | D | I | H | E | L | T | - | A | S | L | K | L | - | - | - | D | R | V | R | I | V | G | H | D | I | G |
| 069 UniRef90\_D8MTC4\_28\_309 | Q | G | D | S | - | - | - | D | R | - | P | E | - | - | T | G | Y | D | T | R | S | L | A | T | K | V | H | G | L | L | - | S | S | L | A | I | - | - | - | T | R | Y | Y | L | A | A | H | D | V | G |
| 070 UniRef90\_UPI0012FADC80\_10\_303 | V | G | E | S | - | - | - | S | K | - | P | A | - | - | S | G | Y | D | K | R | T | M | A | T | D | L | H | E | L | A | - | K | K | L | G | Y | - | - | - | S | S | I | N | L | A | G | H | D | I | G |
| 071 UniRef90\_A0A2E5PAM0\_2\_278 | L | G | D | S | - | - | - | S | C | - | P | P | - | - | G | G | Y | D | K | R | N | V | A | N | D | I | W | R | L | M | Y | E | V | L | G | H | - | - | - | E | Q | F | C | L | A | G | H | D | W | G |
| 072 UniRef90\_T0HVL8\_12\_269 | T | G | D | S | - | - | - | S | R | - | P | K | - | - | T | G | Y | D | S | D | T | V | A | A | D | L | H | A | L | V | - | Q | H | L | G | I | - | - | - | P | R | I | R | L | V | G | H | D | N | G |
| 073 UniRef90\_A0A3N4T2I8\_17\_298 | A | G | L | S | - | - | - | D | K | - | P | D | - | - | D | G | Y | D | A | G | T | L | A | A | D | L | V | A | L | M | - | T | A | L | G | H | - | - | - | D | R | F | D | V | V | G | H | D | I | G |
| 074 UniRef90\_A0A433IAT3\_19\_312 | A | G | N | S | - | - | - | S | K | - | P | D | - | - | S | G | Y | D | K | K | N | M | A | V | D | I | H | D | L | T | - | S | S | L | G | F | - | - | - | D | R | V | S | I | V | G | H | D | I | G |
| 075 UniRef90\_A0A226WR47\_30\_304 | Q | G | D | S | - | - | - | D | R | - | P | A | - | - | D | G | Y | D | T | Q | S | L | A | V | M | V | H | G | L | L | - | Q | Q | L | G | I | - | - | - | G | E | Y | F | L | A | A | H | D | V | G |
| 076 UniRef90\_UPI000DD77D66\_25\_314 | L | G | D | T | - | - | - | A | K | - | P | A | - | - | A | G | Y | E | K | A | A | V | A | Q | D | I | W | K | L | V | - | N | N | L | D | L | - | - | G | P | T | V | N | L | V | G | H | D | M | G |
| 077 UniRef90\_A0A2A5QV31\_6\_277 | L | G | D | S | - | - | - | E | A | - | P | V | - | - | S | G | Y | D | K | D | T | V | A | T | D | V | R | E | L | V | - | S | H | L | G | F | - | D | D | E | P | I | A | L | V | G | H | D | W | G |
| 078 UniRef90\_A0A127EY60\_8\_296 | F | G | G | S | - | - | - | G | K | - | P | A | - | - | S | G | Y | D | K | K | T | M | A | Q | D | V | H | A | L | T | - | Q | S | L | G | L | - | - | - | K | R | V | G | I | A | G | H | D | I | G |
| 079 UniRef90\_UPI0004952425\_6\_308 | F | G | Q | S | - | - | - | S | A | - | P | A | - | - | D | G | Y | T | K | A | A | M | A | Q | D | I | H | A | L | V | - | K | S | L | K | Y | - | - | - | D | R | I | R | L | V | G | H | D | I | G |
| 080 UniRef90\_A0A0D0JP71\_56\_330 | V | G | E | S | - | - | - | D | K | - | P | E | - | - | G | G | Y | D | K | K | N | M | A | K | D | I | H | E | L | V | - | K | K | L | G | Y | - | - | - | N | N | I | N | L | A | G | H | D | I | G |
| 081 UniRef90\_A0A6A7LIC2\_1\_257 | F | G | E | S | - | - | - | S | K | - | P | V | - | - | T | G | Y | D | G | N | T | T | A | E | D | L | Y | Q | L | V | - | S | Q | L | G | F | - | - | D | K | K | I | Y | L | V | G | H | D | V | G |
| 082 UniRef90\_UPI00097BCF81\_14\_312 | A | G | G | S | - | - | - | S | K | - | P | D | - | - | A | G | Y | E | K | K | N | M | A | V | D | I | H | E | L | V | - | K | S | L | G | I | - | - | - | A | K | A | S | I | V | G | H | D | I | G |
| 083 UniRef90\_UPI001591AA5D\_7\_297 | A | G | L | S | - | - | - | G | K | - | P | D | - | - | D | G | Y | D | A | G | T | M | A | A | D | L | A | A | L | M | - | T | A | L | G | H | - | - | - | H | R | F | D | V | V | G | H | D | V | G |
| 084 UniRef90\_A0A4P8YCR5\_18\_300 | Q | G | D | S | - | - | - | D | K | - | P | L | - | - | D | G | Y | D | T | L | T | L | A | D | K | A | H | G | L | L | - | A | Q | L | G | V | - | - | - | H | R | Y | Y | L | A | G | H | D | V | G |
| 085 UniRef90\_A0A4R0GJE6\_14\_295 | A | G | L | S | - | - | - | D | K | - | P | D | - | - | D | G | Y | D | A | G | T | L | A | A | D | L | V | A | L | M | - | A | A | L | G | H | - | - | - | D | R | F | D | V | V | G | H | D | I | G |
| 086 UniRef90\_A0A502C146\_32\_301 | A | G | D | S | - | - | - | G | K | - | P | E | - | - | T | G | Y | E | K | K | A | M | A | N | D | I | H | A | L | A | - | R | S | L | G | I | - | - | - | S | H | A | Q | V | V | G | H | D | I | G |
| 087 UniRef90\_A0A4Y9SAQ9\_19\_291 | A | G | A | S | - | - | - | A | K | - | A | A | - | - | D | G | Y | E | K | T | N | M | A | R | D | I | H | E | L | V | - | Q | S | V | T | K | - | - | - | Q | P | A | T | V | V | G | H | D | I | G |
| 088 UniRef90\_A0A327RPK7\_10\_286 | A | G | L | S | - | - | - | D | K | - | P | K | - | - | T | G | Y | D | K | L | T | L | A | Q | D | I | Y | L | L | V | - | E | Q | L | G | F | - | - | - | K | E | I | S | L | V | G | H | D | I | G |
| 089 UniRef90\_A0A239MTD5\_1\_269 | T | G | N | S | - | - | - | Q | V | - | T | E | - | - | S | G | Y | Q | A | A | Q | L | A | E | D | V | H | E | L | L | - | K | Q | L | G | L | - | - | N | N | D | I | Q | V | V | A | H | D | V | G |
| 090 UniRef90\_UPI00098F4FB4\_42\_302 | L | G | D | T | - | - | - | A | K | - | P | V | - | - | D | G | Y | E | K | A | L | I | A | E | D | I | R | K | L | L | - | R | N | L | D | V | - | - | G | P | V | V | N | L | V | G | H | D | M | G |
| 091 UniRef90\_A0A261TYY2\_38\_305 | L | G | D | S | - | - | - | D | R | - | P | P | - | - | G | G | Y | D | T | D | A | A | A | D | R | L | H | A | F | T | - | Q | S | L | G | W | - | - | - | D | R | F | H | Y | V | G | H | D | V | G |
| 092 UniRef90\_UPI0015F81933\_27\_309 | Q | G | H | S | - | - | - | E | R | - | P | E | - | - | R | G | Y | D | T | H | T | V | A | A | H | V | H | A | A | V | - | K | A | L | G | V | - | - | - | S | T | Y | W | L | V | A | H | D | I | G |
| 093 UniRef90\_A0A0C7N0E5\_9\_278 | Q | G | D | S | - | - | - | D | K | - | P | G | - | - | D | G | Y | D | T | S | T | V | A | K | I | V | H | G | L | V | - | E | K | L | Q | I | - | - | - | P | R | Y | H | L | V | A | H | D | V | G |
| 094 UniRef90\_UPI00112BEE4D\_14\_300 | Q | G | D | S | - | - | - | D | R | - | P | L | - | - | E | G | Y | D | T | A | A | L | A | N | A | L | H | A | L | V | - | R | K | L | G | W | - | - | - | G | R | H | A | V | V | G | H | D | V | G |
| 095 UniRef90\_A0A401Z9D4\_17\_277 | M | G | D | S | - | - | - | E | R | - | T | D | - | - | S | G | Y | D | A | S | T | L | A | E | D | A | F | S | L | V | - | R | S | L | G | F | - | - | - | Q | H | I | F | L | I | S | H | D | L | G |
| 096 UniRef90\_A0A5J6MKN6\_7\_281 | A | G | A | S | - | - | - | Q | R | - | P | P | - | - | D | G | Y | D | K | K | T | M | A | R | D | I | H | G | L | V | - | R | Q | L | G | H | - | - | - | D | R | V | Q | M | V | G | H | D | I | G |
| 097 UniRef90\_A0A4Q7XSL0\_10\_307 | L | G | L | S | - | - | - | S | K | - | P | E | - | - | G | G | Y | D | K | K | T | Q | A | G | D | I | R | A | I | L | - | D | K | L | G | V | - | - | - | D | K | A | D | I | V | G | H | D | I | G |
| 098 UniRef90\_UPI0016110D13\_9\_294 | Q | G | D | S | - | - | - | D | R | - | P | L | - | - | D | G | Y | D | T | Q | T | V | A | E | R | V | H | G | L | L | - | Q | V | I | G | V | - | - | - | T | R | Y | S | L | V | A | H | D | V | G |
| 099 UniRef90\_A0A520GLB7\_17\_313 | A | G | G | S | - | - | - | S | K | - | P | A | - | - | S | G | Y | E | K | K | N | M | A | L | D | V | H | E | L | V | - | R | S | L | G | I | - | - | - | G | H | A | S | V | V | G | H | D | I | G |
| 100 UniRef90\_A0A252EMP1\_9\_266 | A | G | D | S | - | - | - | S | R | - | P | A | - | - | S | G | Y | D | S | N | T | V | A | D | E | L | H | A | L | V | - | R | H | L | G | F | - | - | - | A | S | I | R | L | V | A | H | D | N | G |
| 101 UniRef90\_UPI001668C4B3\_34\_306 | V | G | L | S | - | - | - | D | K | - | P | R | - | - | D | G | Y | D | T | G | T | L | A | R | D | A | V | A | L | M | - | E | A | L | G | H | - | - | - | R | R | F | A | M | V | G | H | D | I | G |
| 102 UniRef90\_UPI00135BC32E\_12\_265 | M | G | D | S | - | - | - | G | K | - | P | R | - | - | E | G | Y | D | K | R | T | V | A | D | D | T | R | A | L | V | - | H | E | L | G | F | - | - | - | E | E | V | A | V | V | G | H | D | W | G |
| 103 UniRef90\_A0A2V9DES3\_17\_290 | A | G | Q | S | - | - | - | S | T | - | P | A | - | - | D | G | Y | T | K | A | E | M | A | Q | D | I | Q | A | L | A | - | R | K | L | G | Y | - | - | - | E | R | I | R | I | V | G | H | D | I | G |
| 104 UniRef90\_A0A6P0DAX4\_34\_314 | L | G | D | T | - | - | - | A | K | - | P | A | - | - | A | G | Y | E | K | A | A | I | A | Q | D | I | R | K | L | V | - | S | N | L | G | L | - | - | G | P | A | V | N | L | V | G | H | D | M | G |
| 105 UniRef90\_A0A1M5KPU7\_13\_307 | F | G | E | S | - | - | - | S | A | - | P | P | - | - | D | G | Y | T | K | A | A | M | A | R | D | I | H | A | L | V | - | K | G | L | N | Y | - | - | - | D | R | I | R | L | V | G | H | D | I | G |
| 106 UniRef90\_A0A2I8DLX4\_17\_295 | Q | G | D | S | - | - | - | D | K | - | P | L | - | - | D | G | Y | D | T | R | T | A | G | E | R | L | R | A | L | F | - | H | T | L | G | L | - | - | - | T | R | Y | A | L | A | G | H | D | I | G |
| 107 UniRef90\_A0A5S4WMZ6\_36\_313 | A | G | D | S | - | - | - | D | K | - | P | Q | - | - | G | G | Y | D | K | A | T | M | A | A | D | I | R | S | M | V | - | H | Q | F | G | R | - | - | - | D | R | I | D | L | V | G | R | D | I | G |
| 108 UniRef90\_UPI0003744601\_9\_294 | Y | G | D | S | - | - | - | E | R | - | P | A | - | - | V | G | Y | D | K | V | T | V | A | T | D | L | H | E | L | M | - | R | T | L | G | F | - | - | - | G | R | I | H | L | V | V | Q | D | L | G |
| 109 UniRef90\_A0A0H3KYM5\_9\_289 | Q | G | D | T | - | - | - | D | K | - | P | L | - | - | S | G | Y | D | T | Q | T | V | A | T | R | L | H | A | F | I | - | D | S | L | G | L | - | - | - | G | S | Y | F | L | A | A | H | D | V | G |
| 110 UniRef90\_UPI001616C592\_11\_307 | A | G | L | S | - | - | - | D | K | - | P | A | - | - | E | G | Y | D | S | G | T | L | A | R | E | T | H | R | L | M | - | Q | V | L | G | Y | - | - | - | D | R | F | A | M | I | A | H | D | V | G |
| 111 UniRef90\_A0A1U9ZZS0\_24\_308 | Q | G | H | S | - | - | - | E | R | - | P | E | - | - | R | G | Y | D | T | H | T | V | A | A | H | V | H | A | A | V | - | R | A | L | G | V | - | - | - | P | A | Y | W | L | A | A | H | D | I | G |
| 112 UniRef90\_UPI00146E50DC\_33\_308 | A | G | S | S | - | - | - | A | A | - | P | A | - | - | D | G | Y | G | K | A | A | L | A | K | D | I | H | A | L | A | - | T | S | L | K | L | - | - | - | G | P | V | K | L | V | G | H | D | I | G |
| 113 UniRef90\_C3KLY5\_13\_287 | Q | G | D | S | - | - | - | D | R | - | P | I | - | - | S | G | C | D | T | M | N | I | A | K | I | A | R | G | L | V | - | Q | K | L | G | V | - | - | - | D | R | H | F | L | V | A | H | D | I | G |
| 114 UniRef90\_A0A2V6STQ7\_27\_303 | A | G | G | S | - | - | - | A | R | - | P | P | - | - | D | G | Y | D | K | K | T | L | A | R | D | I | R | G | L | V | - | R | Q | L | G | H | - | - | - | A | R | V | Q | L | V | G | H | D | I | G |
| 115 UniRef90\_UPI0008D5DA8A\_10\_290 | T | G | D | S | - | - | - | D | R | - | P | T | - | - | E | G | Y | D | T | G | A | V | A | A | T | L | H | Q | L | M | - | S | Q | L | G | H | - | - | - | A | R | Y | Q | V | V | G | H | D | I | G |
| 116 UniRef90\_A0A3N2H8D2\_16\_299 | Q | G | H | S | - | - | - | E | R | - | P | E | - | - | R | G | Y | D | T | H | T | A | A | A | H | V | H | A | A | V | - | K | A | L | G | V | - | - | - | S | S | Y | C | L | V | A | H | D | I | G |
| 117 UniRef90\_A0A379Z3Q1\_16\_292 | I | G | D | S | - | - | - | D | K | - | P | I | - | - | G | G | Y | D | T | G | S | V | A | A | T | L | H | Q | T | M | - | L | Q | L | G | H | - | - | - | E | R | Y | Q | L | I | G | H | D | I | G |
| 118 UniRef90\_A0A4R5QBW7\_8\_277 | L | G | D | S | - | - | - | S | R | - | P | A | - | - | G | G | Y | D | K | K | T | V | S | N | D | L | W | R | L | M | A | E | V | L | G | E | - | - | - | R | R | F | F | L | V | G | H | D | W | G |
| 119 UniRef90\_A0A163VVR8\_16\_291 | I | G | D | S | - | - | - | D | K | - | P | A | - | - | G | G | Y | D | T | G | N | L | G | A | T | L | H | A | M | M | - | A | Q | L | G | H | - | - | - | E | R | Y | Q | L | V | G | H | D | I | G |
| 120 UniRef90\_G0FSK7\_29\_305 | T | G | L | S | - | - | - | G | Q | - | P | G | - | - | D | G | Y | D | T | A | T | L | A | A | D | L | T | G | L | M | - | A | A | L | G | H | - | - | - | E | R | F | A | V | A | G | H | D | V | G |
| 121 UniRef90\_A0A4Q7FLU1\_10\_276 | L | G | D | S | - | - | - | S | R | - | P | V | - | - | S | G | Y | D | K | R | T | I | A | D | D | V | W | R | V | I | S | C | D | L | E | L | - | - | - | Q | S | F | Y | L | V | G | H | D | W | G |
| 122 UniRef90\_A0A2E3NGG1\_11\_269 | F | G | Q | S | - | - | - | S | K | - | P | L | - | - | D | G | Y | D | K | R | T | V | A | V | D | I | Y | E | L | V | - | R | S | L | G | F | - | - | - | E | D | V | A | L | V | G | H | D | M | G |
| 123 UniRef90\_A0A2J9ERN8\_9\_287 | Q | G | D | S | - | - | - | D | R | - | P | L | - | - | D | G | Y | D | T | Q | T | I | A | R | R | T | H | D | L | L | - | A | H | L | G | V | - | - | - | K | R | Y | R | L | A | A | H | D | V | G |
| 124 UniRef90\_UPI001677C581\_8\_288 | T | G | H | S | - | - | - | D | R | - | P | L | - | - | D | G | Y | D | T | G | N | T | A | A | R | L | H | R | V | M | - | A | Q | L | G | H | - | - | - | D | R | Y | D | V | V | G | H | D | V | G |
| 125 UniRef90\_UPI0003039C4F\_8\_269 | L | G | D | S | - | - | - | S | R | - | P | L | - | - | G | G | Y | D | T | K | T | V | S | Q | D | I | W | R | L | V | H | D | V | L | G | E | - | - | - | D | R | F | F | V | V | G | H | D | W | G |
| 126 UniRef90\_A0A1B1YXM9\_17\_276 | L | G | D | S | - | - | - | S | R | - | P | V | - | - | S | G | Y | D | K | M | T | V | A | A | D | L | R | N | L | L | R | D | E | L | G | I | - | - | - | A | Q | A | A | V | V | G | H | D | W | G |
| 127 UniRef90\_A0A534ZXC6\_31\_288 | I | G | D | S | - | - | - | A | I | - | P | A | - | - | D | G | L | N | M | K | T | A | A | V | R | V | H | A | L | A | - | R | A | L | G | I | - | - | - | D | K | A | R | V | V | G | H | D | V | G |
| 128 UniRef90\_A0A2X1TAZ7\_42\_319 | T | G | Y | S | - | - | - | A | R | - | P | E | - | - | S | G | Y | D | T | G | A | V | A | M | V | L | H | T | M | M | - | H | Q | L | G | Y | - | - | - | K | T | Y | S | V | V | G | H | D | I | G |
| 129 UniRef90\_A0A4Q5QRH4\_8\_282 | Q | G | D | S | - | - | - | D | K | - | P | L | - | - | D | G | Y | D | T | Q | A | V | A | R | R | V | H | D | L | V | - | A | H | L | G | L | - | - | - | G | R | Y | C | L | A | A | H | D | V | G |
| 130 UniRef90\_A0A0D6HQT0\_17\_294 | Q | G | D | S | - | - | - | D | K | - | P | L | - | - | D | G | Y | D | T | R | S | A | G | E | R | L | R | A | L | F | - | H | T | L | G | L | - | - | - | T | R | Y | A | L | A | G | H | G | I | G |
| 131 UniRef90\_A0A1G4JCM6\_9\_275 | Q | G | D | S | - | - | - | D | K | - | P | G | - | - | D | G | Y | D | T | Q | T | I | A | N | R | V | H | G | V | V | - | K | G | L | G | L | - | - | - | S | R | Y | S | L | A | A | H | D | I | G |
| 132 UniRef90\_UPI001269E708\_28\_301 | Q | G | H | S | - | - | - | E | R | - | P | E | - | - | S | G | Y | D | T | H | T | V | A | R | R | V | H | A | A | V | - | R | I | L | G | A | - | - | - | S | T | Y | W | L | V | A | H | D | I | G |
| 133 UniRef90\_A0A538SUN7\_3\_303 | M | G | L | S | - | - | - | A | H | - | P | E | - | - | T | G | Y | T | K | K | N | Q | G | V | D | I | V | G | V | L | - | D | H | F | K | I | - | - | - | R | K | A | D | L | V | T | H | D | I | G |
| 134 UniRef90\_UPI001646B9D9\_15\_288 | L | G | D | S | - | - | - | D | R | - | P | E | - | - | Q | G | Y | D | T | G | N | V | A | R | L | L | H | A | A | M | - | T | R | L | G | H | - | - | - | E | R | Y | H | L | V | G | H | D | V | G |
| 135 UniRef90\_K9DQK6\_17\_307 | L | G | L | S | - | - | - | S | K | - | A | A | - | - | G | G | Y | D | K | K | S | Q | A | A | D | I | R | A | V | V | - | E | R | L | G | Q | - | - | - | D | R | A | D | V | V | G | H | D | I | G |
| 136 UniRef90\_A0A2N9BM66\_25\_297 | M | G | A | S | - | - | - | D | K | - | P | A | - | - | T | G | Y | D | A | A | T | L | A | D | D | M | A | A | L | M | - | T | A | L | G | H | - | - | - | D | R | F | A | V | V | G | Y | D | L | G |
| 137 UniRef90\_A0A370LBV7\_5\_266 | F | G | A | S | - | - | - | D | K | - | P | E | - | - | A | G | Y | D | K | K | T | M | A | G | D | V | H | A | L | A | - | A | S | L | G | L | - | - | - | K | S | A | A | V | V | G | H | D | I | G |
| 138 UniRef90\_A0A2V9ZM06\_3\_258 | I | G | D | S | - | - | - | A | I | - | P | P | - | - | D | G | L | D | M | K | T | A | A | L | R | I | H | A | L | A | - | R | S | F | G | V | - | - | - | Q | K | A | E | V | V | G | H | D | I | G |
| 139 UniRef90\_UPI00101F418B\_26\_310 | L | G | D | S | - | - | - | D | K | - | P | I | - | - | A | G | Y | D | L | A | T | V | A | Q | D | L | H | Q | F | A | - | L | Q | T | G | L | M | S | H | G | A | L | D | V | A | G | H | D | I | G |
| 140 UniRef90\_A0A4V2YYI8\_27\_305 | V | G | L | S | - | - | - | D | K | - | P | L | - | - | D | G | Y | D | T | G | T | L | A | T | D | M | V | A | L | M | - | N | A | L | G | H | - | - | - | E | R | F | A | M | V | G | H | D | I | G |
| 141 UniRef90\_UPI001487D2FB\_8\_292 | M | G | L | S | - | - | - | S | H | - | P | E | - | - | G | G | Y | D | K | W | T | Q | A | G | D | V | R | A | V | L | - | S | R | L | G | I | - | - | - | D | R | A | A | V | V | G | H | D | I | G |
| 142 UniRef90\_B9XAH7\_320\_607 | F | G | D | S | - | - | - | E | K | - | P | L | - | - | T | G | Y | D | P | A | T | A | A | Q | D | L | H | A | F | I | - | E | T | L | D | L | A | K | G | R | G | V | D | V | V | G | H | D | I | G |
| 143 UniRef90\_UPI0013696204\_44\_306 | V | G | L | S | - | - | - | D | K | - | P | R | - | - | D | G | Y | D | T | G | T | L | A | A | D | M | V | A | L | M | - | G | A | L | G | H | - | - | - | E | R | F | A | M | V | G | H | D | I | G |
| 144 UniRef90\_W5WIM6\_13\_292 | V | G | L | S | - | - | - | G | K | - | P | D | - | - | Q | G | Y | D | T | G | T | L | A | A | D | M | V | G | L | M | - | S | A | L | G | H | - | - | - | H | R | F | A | M | V | G | H | D | V | G |
| 145 UniRef90\_A0A2X2GG63\_10\_290 | I | G | D | S | - | - | - | D | K | - | P | C | - | - | G | G | Y | D | T | G | S | V | A | A | T | L | H | Q | T | M | - | L | Q | L | G | H | - | - | - | A | R | Y | Q | L | V | G | H | D | I | G |
| 146 UniRef90\_A0A2E2YI39\_6\_274 | L | G | D | S | - | - | - | S | R | - | P | I | - | - | G | G | Y | D | K | M | T | V | A | N | D | I | W | K | L | V | S | E | E | L | G | Y | - | - | - | S | S | F | L | L | V | G | H | D | W | G |
| 147 UniRef90\_A0A2W7G3K6\_24\_312 | I | G | D | S | - | - | - | D | R | - | P | A | - | - | N | G | Y | D | L | R | T | V | A | A | E | L | H | G | L | I | - | G | T | L | G | L | A | H | E | G | G | I | D | V | V | G | H | D | I | G |
| 148 UniRef90\_A0A6B2RWJ8\_28\_298 | Q | G | H | S | - | - | - | D | R | - | P | Q | - | - | G | G | Y | D | T | H | T | V | A | S | R | V | Q | A | A | L | - | A | A | L | K | V | - | - | - | P | E | Y | W | L | V | A | H | D | I | G |
| 149 UniRef90\_A0A537A198\_6\_270 | L | G | E | S | - | - | - | S | R | - | P | A | - | - | N | G | Y | D | Q | N | S | I | A | S | D | V | R | E | M | C | - | Q | Q | L | D | L | - | - | - | G | R | F | H | L | V | G | H | D | F | G |
| 150 UniRef90\_UPI00135F2885\_48\_334 | L | G | D | S | - | - | - | T | R | - | P | E | - | - | S | G | Y | E | K | D | V | I | G | E | D | I | V | A | L | A | - | R | Q | L | G | L | - | - | - | T | R | F | S | I | A | G | H | D | L | G |

  
  

|  |  |  |  |  |  |  |  |  |  |  |  |  |  |  |  |  |  |  |  |  |  |  |  |  |  |  |  |  |  |  |  |  |  |  |  |  |  |  |  |  |  |  |  |  |  |  |  |  |  |  |
| --- | --- | --- | --- | --- | --- | --- | --- | --- | --- | --- | --- | --- | --- | --- | --- | --- | --- | --- | --- | --- | --- | --- | --- | --- | --- | --- | --- | --- | --- | --- | --- | --- | --- | --- | --- | --- | --- | --- | --- | --- | --- | --- | --- | --- | --- | --- | --- | --- | --- | --- |
| **001 Input\_protein\_seq** | I | W | N | T | Y | P | M | V | V | K | N | Q | A | D | I | A | R | L | V | Y | M | E | A | P | I | P | D | A | R | I | Y | R | F | P | A | - | - | F | T | A | Q | - | G | E | S | L | V | W | H | F |
| 002 UniRef90\_A0A2G0Y5Q3\_5\_314 | I | W | N | T | Y | P | M | V | V | K | N | Q | A | D | I | A | K | L | V | Y | M | E | A | P | I | P | D | A | K | A | Y | D | F | P | A | - | - | F | T | P | E | - | G | E | S | L | V | W | H | F |
| 003 UniRef90\_A0A172YJZ3\_2\_306 | I | W | N | T | Y | P | M | L | V | Q | H | Q | D | D | I | R | R | A | I | Y | M | E | A | P | I | P | D | D | S | I | Y | S | F | P | A | - | - | F | T | P | Q | - | G | E | S | L | V | W | H | F |
| 004 UniRef90\_A0A4Z1C7D3\_9\_314 | I | W | N | T | Y | P | M | A | A | N | H | Q | D | D | I | R | R | L | V | F | M | E | A | P | I | P | D | D | N | I | Y | D | F | P | A | - | - | F | T | P | Q | - | G | E | S | L | V | W | H | F |
| 005 UniRef90\_A0A0A3Z1S4\_7\_319 | I | W | N | T | Y | P | M | A | V | K | H | Q | D | K | I | D | R | L | I | Y | M | E | A | P | I | P | D | R | R | M | Y | D | F | P | A | - | - | F | S | P | E | - | G | E | S | L | V | W | H | F |
| 006 UniRef90\_A0A329J6I4\_9\_315 | I | W | N | T | Y | P | L | A | V | M | H | Q | S | Q | I | K | K | L | V | F | M | E | A | P | I | P | D | K | S | V | Y | D | F | P | A | - | - | F | S | P | E | - | G | E | S | L | V | W | H | F |
| 007 UniRef90\_A0A4V1G750\_8\_314 | I | W | N | T | W | P | M | L | V | Q | H | Q | A | Q | I | R | K | A | V | Y | M | E | A | P | I | P | D | D | S | I | Y | E | F | P | A | - | - | F | T | P | E | - | G | E | S | L | V | W | H | F |
| 008 UniRef90\_T2L220\_17\_309 | I | W | N | T | Y | P | M | L | V | Q | H | Q | N | A | I | R | R | V | I | Y | M | E | A | P | I | P | G | D | S | I | Y | S | F | P | A | - | - | F | T | P | E | - | G | E | S | L | V | W | H | F |
| 009 UniRef90\_UPI000C2FE79C\_12\_314 | I | W | N | T | Y | P | M | L | V | N | H | Q | A | D | I | R | R | A | V | Y | M | E | A | P | I | P | D | D | S | I | Y | K | F | A | A | - | - | F | S | P | K | - | G | E | S | L | V | W | H | F |
| 010 UniRef90\_UPI00102FACD2\_13\_314 | N | W | N | T | Y | P | F | V | V | T | H | E | N | N | L | R | R | V | V | F | M | E | A | P | I | P | D | D | T | L | Y | A | F | P | A | - | - | F | T | P | Q | - | G | E | S | L | V | W | H | F |
| 011 UniRef90\_UPI0014749FF6\_10\_312 | I | W | A | T | Y | P | M | L | S | Q | H | Q | K | D | I | S | Q | V | A | Y | L | E | A | V | I | P | D | S | R | I | Y | G | F | P | A | - | - | Y | T | P | Q | - | G | E | S | T | A | W | H | F |
| 012 UniRef90\_A0A3M4V4S1\_41\_332 | I | W | N | S | Y | P | M | V | A | R | H | P | G | D | V | V | K | A | A | F | I | E | A | T | I | P | D | D | T | L | Y | S | L | P | A | - | - | F | V | A | T | - | G | E | A | P | G | W | H | H |
| 013 UniRef90\_A0A3L8C981\_31\_324 | I | W | N | S | Y | P | M | V | A | R | H | P | E | D | I | V | K | A | A | F | I | E | A | T | I | P | D | D | T | L | Y | S | L | P | A | - | - | F | V | A | T | - | G | E | A | P | G | W | H | H |
| 014 UniRef90\_A0A1X0N2L9\_32\_323 | I | W | N | S | Y | P | M | V | A | R | H | P | E | D | I | I | K | A | A | F | I | E | A | T | I | P | D | D | T | L | Y | S | L | P | A | - | - | F | V | A | T | - | G | E | A | P | G | W | H | H |
| 015 UniRef90\_UPI00166CAA4C\_15\_313 | V | M | V | A | F | A | Y | A | N | E | F | P | R | E | T | R | T | L | T | L | V | E | A | P | I | P | D | T | S | V | F | Q | A | P | A | - | - | L | T | A | - | - | Q | G | P | Y | L | W | N | F |
| 016 UniRef90\_UPI001661A3FF\_47\_334 | T | M | V | A | Y | S | Y | A | A | Q | Y | P | A | T | V | E | K | L | V | L | S | E | A | P | I | P | D | L | S | I | Y | Q | I | P | S | - | - | L | T | E | - | - | R | G | P | G | V | W | N | F |
| 017 UniRef90\_A0A1H5MZC9\_42\_331 | T | M | V | A | Y | S | Y | A | A | A | H | P | A | D | V | R | K | L | V | L | S | E | A | P | I | P | D | P | G | I | Y | T | F | P | S | - | - | L | T | A | - | - | D | G | P | G | A | W | H | F |
| 018 UniRef90\_UPI000690E8EC\_26\_310 | T | M | V | A | Y | S | Y | A | A | A | H | R | A | D | V | T | Q | L | V | L | S | E | A | P | I | P | D | P | G | I | Y | Q | F | P | A | - | - | L | T | A | - | - | R | G | P | G | V | W | N | F |
| 019 UniRef90\_R4LNI7\_31\_317 | T | M | V | A | Y | A | Y | A | A | A | H | P | G | D | V | D | K | L | V | L | S | E | A | P | I | P | D | E | E | V | Y | T | Y | P | A | - | - | L | T | A | - | - | R | G | P | Y | L | W | N | F |
| 020 UniRef90\_A0A4R2C838\_67\_350 | T | T | V | A | Y | P | Y | A | A | A | H | P | N | D | V | V | K | L | V | L | S | E | A | P | I | P | D | P | T | I | Y | T | F | P | S | - | - | L | T | P | - | - | N | G | P | G | L | W | W | F |
| 021 UniRef90\_A0A2P9HD85\_25\_319 | N | S | A | S | Y | P | M | V | A | N | N | Q | D | I | I | K | K | V | V | F | M | D | S | P | I | P | D | K | A | M | W | T | Y | P | G | - | - | L | T | P | N | - | G | P | G | L | G | W | H | F |
| 022 UniRef90\_UPI000D14D87C\_43\_332 | T | M | V | A | Y | S | Y | A | A | Q | Y | P | H | S | V | K | K | L | V | L | S | E | A | P | I | P | D | Q | G | I | Y | A | F | P | S | - | - | L | T | A | - | - | Q | G | P | G | V | W | N | F |
| 023 UniRef90\_A0A1Q4ZL08\_6\_289 | T | M | V | A | Y | A | Y | A | A | A | Y | G | N | Q | V | S | K | L | V | L | T | E | A | P | I | P | D | Q | G | L | Y | Q | A | P | S | - | - | L | T | P | - | - | S | G | P | G | L | W | N | F |
| 024 UniRef90\_A0A1I2GGE7\_30\_316 | T | M | V | A | Y | A | Y | A | A | A | N | P | T | E | V | K | K | L | V | L | S | E | A | P | I | P | D | E | S | I | Y | S | F | P | A | - | - | L | P | K | - | - | T | G | R | G | V | W | N | F |
| 025 UniRef90\_UPI0005580D51\_18\_305 | T | M | V | A | Y | A | Y | A | A | A | H | P | G | E | V | T | K | L | V | L | S | E | A | P | I | P | D | A | N | L | Y | T | F | P | S | - | - | L | T | D | - | - | K | G | P | G | A | W | H | F |
| 026 UniRef90\_A0A2W2F1P0\_6\_289 | T | M | V | A | Y | A | Y | A | A | A | H | P | D | R | I | S | R | L | V | L | S | E | A | P | I | P | D | E | S | I | Y | T | F | P | A | - | - | L | T | A | - | - | A | G | P | A | V | W | N | F |
| 027 UniRef90\_UPI0010F9F40F\_6\_288 | T | M | V | A | Y | A | Y | A | A | A | H | P | D | R | V | R | R | L | V | L | S | E | A | P | I | P | D | E | S | I | Y | T | L | P | A | - | - | L | T | T | - | - | A | G | P | A | V | W | N | F |
| 028 UniRef90\_A0A3E2YQE6\_6\_289 | T | M | V | A | Y | A | Y | A | A | A | H | P | D | R | V | S | R | L | V | L | S | E | A | P | I | P | D | E | S | V | Y | A | F | P | A | - | - | L | T | P | - | - | A | G | P | G | V | W | N | F |
| 029 UniRef90\_A0A1A9ACL3\_6\_288 | T | M | V | A | Y | A | Y | A | A | A | H | P | D | S | V | A | R | L | V | L | T | E | A | P | I | P | D | E | S | I | Y | T | I | P | A | - | - | L | T | A | - | - | A | G | P | A | V | W | N | F |
| 030 UniRef90\_UPI00174B5D19\_38\_323 | A | W | I | A | Y | P | Y | A | A | M | W | P | A | E | V | S | R | I | V | V | M | E | G | P | I | V | D | E | S | L | Y | G | F | P | A | - | - | L | A | P | E | - | G | G | L | Y | V | W | H | F |
| 031 UniRef90\_A0A495JCH5\_50\_333 | T | M | V | A | Y | A | Y | A | A | A | H | P | Q | D | V | T | R | L | V | L | T | E | A | P | I | P | D | Q | T | I | Y | N | F | P | A | - | - | L | T | S | - | - | Q | G | P | G | F | W | N | F |
| 032 UniRef90\_UPI0013D14F7B\_11\_294 | T | M | V | A | Y | A | Y | A | V | A | H | P | D | E | V | T | K | L | V | L | S | E | A | P | I | P | D | P | A | I | Y | S | F | P | S | - | - | L | T | P | - | - | D | G | P | G | A | W | H | F |
| 033 UniRef90\_UPI00036EC6E3\_56\_342 | T | T | V | A | Y | P | Y | A | A | A | H | P | D | D | I | V | K | L | V | L | S | E | A | P | I | P | D | P | T | I | Y | T | F | P | S | - | - | L | T | P | - | - | N | G | P | G | L | W | W | F |
| 034 UniRef90\_A0A1H3P9Q0\_6\_290 | A | M | V | A | Y | A | Y | A | A | A | H | A | G | D | V | R | H | L | V | L | T | E | A | P | I | P | D | Q | S | L | Y | Q | A | P | S | - | - | L | T | P | - | - | N | G | P | G | W | W | N | L |
| 035 UniRef90\_A0A385B2U0\_41\_332 | A | W | I | A | Y | P | Y | A | A | M | W | P | N | E | V | S | R | M | V | V | M | E | G | P | I | P | D | R | S | L | Y | T | F | P | A | - | - | F | P | A | E | - | G | G | L | S | T | W | H | L |
| 036 UniRef90\_UPI001430CB85\_25\_306 | A | W | V | A | Y | P | Y | A | V | L | F | G | N | E | V | Q | R | L | A | L | L | D | A | G | I | P | G | V | T | L | P | D | A | L | P | - | - | V | A | P | D | - | R | A | W | R | T | W | H | F |
| 037 UniRef90\_A0A109IHW8\_6\_288 | T | M | V | A | Y | A | Y | A | A | A | H | P | D | T | V | S | R | L | V | L | T | E | A | P | I | P | D | E | S | I | Y | T | F | P | A | - | - | L | T | E | - | - | A | G | P | A | V | W | N | F |
| 038 UniRef90\_A0A2T6L0M5\_14\_298 | A | W | V | A | Y | P | Y | A | A | R | W | P | S | E | V | D | R | M | A | L | L | E | G | P | L | P | D | E | T | V | Y | D | Y | R | A | - | - | L | D | P | D | - | G | G | P | S | I | W | H | Y |
| 039 UniRef90\_A0A1C4Z0V4\_6\_288 | T | M | V | A | Y | A | Y | A | A | A | H | P | D | E | V | S | R | L | V | L | T | E | A | P | I | P | D | E | S | I | Y | A | F | P | A | - | - | L | T | A | - | - | A | G | P | G | V | W | N | F |
| 040 UniRef90\_A0A4U3M272\_82\_365 | T | T | V | A | Y | P | Y | A | A | A | H | R | D | D | V | V | K | L | V | L | S | E | A | P | I | P | D | P | V | I | Y | T | F | P | S | - | - | L | T | P | - | - | N | G | P | G | L | W | W | F |
| 041 UniRef90\_UPI00052728DD\_22\_294 | A | W | V | A | Y | P | Y | A | A | L | F | G | D | T | V | M | R | L | A | L | L | D | A | G | I | P | G | I | T | L | P | D | A | L | P | - | - | I | A | P | E | - | R | A | W | R | T | W | H | F |
| 042 UniRef90\_A0A6B8MSH7\_52\_336 | N | S | A | S | Y | P | L | V | A | N | N | Q | G | Y | I | K | K | V | V | F | M | D | S | P | I | P | D | K | S | M | F | E | Y | A | G | - | - | Y | T | P | S | - | G | P | G | L | G | W | H | F |
| 043 UniRef90\_I4VSM6\_28\_303 | A | W | V | A | Y | P | L | A | A | R | Y | S | S | E | V | R | A | L | A | L | L | D | A | G | I | P | G | V | T | L | P | D | R | L | P | - | - | V | A | P | E | - | Q | A | W | R | T | W | H | F |
| 044 UniRef90\_A0A1I3BFK1\_40\_314 | A | W | V | A | Y | P | Y | A | A | L | F | S | D | E | V | Q | R | L | A | L | M | D | A | G | I | P | G | I | T | L | P | D | A | L | S | - | - | T | A | P | E | - | Q | A | W | R | T | W | H | F |
| 045 UniRef90\_A0A1Z4J856\_42\_316 | A | P | V | A | Y | A | Y | A | A | A | H | P | E | D | V | R | R | L | A | V | L | E | L | V | L | A | G | A | G | L | E | E | L | I | N | - | - | - | - | - | K | - | A | S | A | A | L | W | H | F |
| 046 UniRef90\_A0A0M4FVH2\_27\_314 | A | W | V | A | Y | T | Y | A | A | L | F | G | D | E | V | R | R | L | A | L | L | D | A | G | I | P | G | I | T | M | P | D | A | L | P | - | - | T | A | P | E | - | R | A | W | K | T | W | H | F |
| 047 UniRef90\_UPI001032135D\_25\_299 | A | W | V | A | W | P | C | A | A | R | Y | P | D | E | V | R | K | L | A | L | L | D | A | G | I | P | G | V | T | L | P | E | A | L | P | - | - | V | S | P | D | - | N | A | W | K | T | W | H | F |
| 048 UniRef90\_D5WL63\_24\_300 | A | W | V | A | F | P | Y | A | V | M | F | A | D | E | V | R | R | V | A | L | L | D | A | G | I | P | G | V | T | L | P | D | A | L | P | - | - | I | A | P | E | - | R | A | W | R | T | W | H | F |
| 049 UniRef90\_A0A1H3E943\_18\_291 | A | W | V | A | Y | P | Y | I | A | R | F | S | D | E | V | R | K | L | V | I | L | D | A | N | I | P | G | V | T | L | S | N | T | I | T | - | - | V | G | R | D | - | - | N | W | K | A | W | H | F |
| 050 UniRef90\_A0A346N7R3\_30\_317 | L | M | V | A | Y | S | Y | A | A | Q | Y | P | D | D | V | D | R | I | V | L | M | D | A | F | L | P | G | V | G | D | W | K | N | V | W | - | - | - | - | - | - | - | L | M | R | D | L | W | H | F |
| 051 UniRef90\_A0A537Q000\_40\_318 | M | W | I | A | Y | P | L | A | A | Q | H | R | E | A | V | D | K | L | I | V | T | E | A | L | I | P | G | V | T | P | T | P | P | M | L | - | - | L | P | P | D | - | A | T | A | G | L | T | Q | F |
| 052 UniRef90\_E6V5X5\_24\_320 | L | M | V | A | Y | A | Y | A | A | Q | F | P | S | E | T | D | K | V | V | L | M | D | A | F | L | P | G | I | G | A | W | Q | N | V | W | - | - | - | - | - | - | - | L | L | R | D | L | W | H | F |
| 053 UniRef90\_A0A484PNM3\_14\_291 | V | W | I | G | Y | P | Y | A | R | L | F | P | E | Q | V | R | K | L | V | L | I | D | A | T | V | P | G | I | V | P | R | E | A | Y | A | - | - | F | E | Q | A | - | R | I | S | K | N | W | H | F |
| 054 UniRef90\_UPI0011BDEFB8\_30\_311 | A | W | V | A | W | P | F | T | A | L | Y | G | D | E | V | K | R | L | A | L | L | D | A | G | I | P | G | V | T | L | P | D | A | L | P | - | - | V | T | P | D | - | K | A | W | K | T | W | H | F |
| 055 UniRef90\_A0A1H2UZ41\_16\_293 | S | W | V | A | Y | P | F | V | V | R | F | A | E | E | V | G | R | L | V | M | L | D | A | N | I | P | G | V | T | L | K | S | T | I | E | - | - | V | G | P | H | - | - | N | W | K | A | W | H | F |
| 056 UniRef90\_A0A1M5ZT81\_29\_302 | A | W | V | A | Y | P | Y | A | A | L | F | D | G | E | V | E | R | L | A | L | L | D | A | G | I | P | G | I | T | L | P | D | A | L | P | - | - | I | T | A | E | - | R | V | W | R | T | W | H | F |
| 057 UniRef90\_A0A135GL34\_14\_283 | A | A | V | A | Y | A | Y | A | A | T | H | R | D | E | V | R | S | L | V | F | C | E | M | A | L | K | G | V | A | G | D | K | G | I | E | Y | F | M | D | Q | R | - | D | E | L | R | L | W | H | L |
| 058 UniRef90\_A0A484UEV2\_23\_280 | V | W | I | G | Y | P | Y | A | R | L | Y | P | E | Q | V | L | T | L | T | L | I | D | A | T | V | P | G | I | V | P | Q | E | A | Y | A | - | - | F | E | Q | Q | - | R | I | G | K | N | W | H | F |
| 059 UniRef90\_A0A495NUG6\_51\_333 | T | M | V | A | Y | A | Y | A | A | T | Y | R | S | S | V | E | R | L | M | L | T | E | A | P | I | P | D | K | V | V | Y | S | Y | P | S | - | - | L | T | P | - | - | Q | G | P | G | F | W | N | F |
| 060 UniRef90\_A0A1M5MA45\_15\_292 | L | M | V | S | Y | A | Y | A | A | Q | Y | S | D | E | V | E | K | L | A | L | L | D | A | F | I | P | G | V | E | P | - | - | - | - | - | - | - | V | W | S | R | - | M | S | T | T | I | W | H | F |
| 061 UniRef90\_UPI000EF95C7F\_30\_312 | M | W | T | G | Y | A | M | A | A | D | R | P | G | P | I | E | R | M | V | L | V | D | A | I | I | P | G | V | S | P | S | P | P | L | L | - | - | S | D | R | R | - | T | S | D | F | L | W | H | F |
| 062 UniRef90\_L9WLS3\_15\_271 | M | P | T | A | Y | A | Y | A | A | Q | Y | R | E | E | V | A | A | L | C | V | L | E | A | G | L | P | G | V | R | E | D | - | - | - | - | - | - | - | - | - | - | - | E | K | R | H | F | W | H | T |
| 063 UniRef90\_UPI0005D31B04\_21\_288 | M | M | V | A | Y | E | Y | A | S | A | H | P | D | E | V | R | K | L | A | V | L | E | A | G | L | P | G | L | G | L | E | A | L | H | D | - | - | - | - | S | A | - | A | F | P | Q | F | W | H | F |
| 064 UniRef90\_UPI001408C682\_10\_298 | L | M | V | A | Y | G | Y | A | A | Q | F | P | A | D | T | S | R | V | V | F | M | D | A | F | V | P | G | I | G | D | W | R | N | A | F | - | - | - | - | - | - | - | P | A | K | A | V | W | H | F |
| 065 UniRef90\_A0A1Y6KKD8\_22\_314 | A | W | V | A | Y | P | Y | A | A | L | F | G | D | A | V | Q | S | L | A | L | L | D | A | G | I | P | G | V | T | L | P | E | A | L | P | - | - | Y | A | P | D | - | R | A | W | R | T | W | H | F |
| 066 UniRef90\_A0A0X3RYG7\_65\_349 | M | W | V | A | Y | G | Y | A | A | Q | Y | R | S | Q | V | R | S | M | A | V | M | E | A | P | V | P | D | K | S | I | Y | T | Y | A | A | - | - | L | N | A | D | P | N | R | P | S | P | W | H | F |
| 067 UniRef90\_A0A2T0T762\_22\_302 | M | W | T | G | Y | A | L | A | A | D | H | P | E | R | V | G | R | L | A | V | V | D | A | I | I | P | G | L | T | P | A | P | P | F | F | - | - | S | P | A | P | - | V | T | Q | R | F | W | H | F |
| 068 UniRef90\_A0A2V6QCX2\_10\_286 | L | M | V | A | Y | A | Y | A | A | Q | F | P | E | A | T | E | R | V | V | L | M | D | A | F | L | P | G | I | G | N | W | K | N | V | W | - | - | - | - | - | - | - | L | L | R | D | L | W | H | F |
| 069 UniRef90\_D8MTC4\_28\_309 | A | W | V | A | W | P | Y | A | A | L | Y | G | D | E | V | K | R | L | A | L | L | D | A | G | I | P | G | I | T | L | P | D | A | L | P | - | - | V | S | P | D | - | A | A | W | K | T | W | H | F |
| 070 UniRef90\_UPI0012FADC80\_10\_303 | L | M | V | A | Y | A | Y | A | A | Q | Y | P | A | D | V | K | K | V | A | L | M | D | A | L | L | P | G | V | E | P | - | - | - | - | - | - | - | V | W | H | Q | - | V | S | T | T | A | W | W | F |
| 071 UniRef90\_A0A2E5PAM0\_2\_278 | G | P | V | A | Y | A | L | A | H | A | H | G | S | A | V | T | R | L | A | I | L | D | V | V | I | P | F | G | S | P | D | D | L | T | - | - | - | - | - | - | - | - | W | G | G | R | R | W | H | H |
| 072 UniRef90\_T0HVL8\_12\_269 | G | R | V | A | Y | A | Y | A | A | N | Y | R | E | E | V | A | S | L | V | F | L | E | S | K | V | L | G | I | E | S | D | D | D | - | - | - | - | - | - | - | - | - | A | Q | K | E | Y | W | H | F |
| 073 UniRef90\_A0A3N4T2I8\_17\_298 | T | W | T | A | Y | A | L | T | A | D | H | P | E | R | V | G | R | L | A | I | V | E | A | V | I | P | G | L | T | P | S | P | P | F | F | - | - | G | P | A | A | - | A | N | Q | R | L | W | Q | F |
| 074 UniRef90\_A0A433IAT3\_19\_312 | L | M | V | A | Y | A | Y | A | A | Q | F | P | Q | A | T | E | R | V | V | L | M | D | A | F | L | P | G | I | G | N | W | K | D | V | W | - | - | - | - | - | - | - | L | M | R | D | L | W | H | F |
| 075 UniRef90\_A0A226WR47\_30\_304 | A | W | V | A | Y | P | Y | A | A | L | F | G | N | E | V | R | R | L | A | L | L | D | A | G | I | P | G | I | T | L | P | D | A | L | P | - | - | V | A | P | E | - | R | A | W | R | T | W | H | F |
| 076 UniRef90\_UPI000DD77D66\_25\_314 | G | M | V | A | Y | A | Y | A | A | Q | H | P | A | N | V | R | T | L | A | I | L | D | V | P | L | P | G | I | E | P | W | D | E | W | V | - | - | - | - | - | - | - | Q | G | P | R | T | W | H | F |
| 077 UniRef90\_A0A2A5QV31\_6\_277 | M | P | T | A | Y | A | Y | A | A | Q | Y | R | E | D | V | R | A | L | C | V | L | E | A | G | L | P | G | I | N | E | D | - | - | - | - | - | - | - | - | - | - | - | E | K | L | K | L | W | H | T |
| 078 UniRef90\_A0A127EY60\_8\_296 | L | M | V | A | Y | A | Y | A | A | Q | Y | P | D | E | V | D | R | I | V | L | M | D | A | F | L | P | G | I | G | D | T | T | G | I | F | - | - | - | - | - | - | - | L | L | R | D | L | W | H | F |
| 079 UniRef90\_UPI0004952425\_6\_308 | L | M | V | A | Y | A | Y | A | A | Q | Y | P | T | E | V | D | R | I | V | L | M | E | A | F | L | P | G | V | G | E | W | N | N | V | F | - | - | - | - | - | - | - | L | L | R | D | L | W | H | F |
| 080 UniRef90\_A0A0D0JP71\_56\_330 | L | M | V | A | Y | A | Y | A | A | Q | Y | G | S | E | V | K | K | L | A | L | M | D | A | L | L | P | G | I | E | P | - | - | - | - | - | - | - | V | W | S | Q | - | V | S | A | S | A | W | W | F |
| 081 UniRef90\_A0A6A7LIC2\_1\_257 | A | Q | T | A | Y | S | Y | A | A | I | H | P | D | N | V | S | K | L | V | I | M | D | A | V | F | P | G | F | F | P | P | N | - | - | - | - | - | - | - | - | - | - | F | E | G | A | C | W | W | C |
| 082 UniRef90\_UPI00097BCF81\_14\_312 | L | M | V | A | Y | A | Y | A | A | Q | F | P | S | E | T | D | R | V | V | L | M | D | A | F | L | P | G | I | G | P | W | Q | N | V | W | - | - | - | - | - | - | - | L | L | R | D | L | W | H | F |
| 083 UniRef90\_UPI001591AA5D\_7\_297 | M | W | T | G | Y | A | L | A | A | D | H | P | E | R | V | G | R | L | T | V | V | D | A | I | I | P | G | L | T | P | A | P | P | V | F | - | - | G | T | A | A | - | V | N | Q | R | L | W | H | F |
| 084 UniRef90\_A0A4P8YCR5\_18\_300 | A | W | V | A | W | P | F | A | N | R | Y | R | D | E | V | K | R | L | A | L | L | D | A | G | I | P | G | I | T | L | P | D | A | L | P | - | - | L | S | P | D | - | K | A | W | K | T | W | H | F |
| 085 UniRef90\_A0A4R0GJE6\_14\_295 | T | W | T | G | Y | A | L | A | A | D | H | P | E | Q | V | G | R | L | A | V | L | E | A | V | I | P | G | L | S | P | S | P | P | F | F | - | - | G | P | A | A | - | A | S | Q | K | L | W | Q | F |
| 086 UniRef90\_A0A502C146\_32\_301 | L | M | V | A | Y | A | Y | A | A | Q | Y | P | T | E | T | D | R | V | V | L | M | D | A | F | L | P | G | V | G | D | W | T | H | V | W | - | - | - | - | - | - | - | L | L | H | D | L | W | H | F |
| 087 UniRef90\_A0A4Y9SAQ9\_19\_291 | L | M | V | A | Y | A | Y | A | A | Q | Y | P | G | D | T | S | K | L | A | L | M | D | A | F | L | P | G | I | G | N | W | K | D | M | F | - | - | - | - | - | - | - | L | L | R | D | L | W | H | F |
| 088 UniRef90\_A0A327RPK7\_10\_286 | A | M | V | A | F | T | Y | A | A | E | Y | R | E | Y | V | K | K | L | V | L | L | D | V | L | L | P | G | F | G | L | E | K | L | M | D | - | - | - | - | - | V | - | A | N | G | G | M | W | H | F |
| 089 UniRef90\_A0A239MTD5\_1\_269 | V | W | V | A | Y | A | Y | A | A | Q | W | P | S | E | V | R | R | M | A | V | M | E | A | P | I | A | D | G | S | A | Y | S | Y | P | A | - | - | L | N | A | D | P | E | K | P | S | P | W | H | W |
| 090 UniRef90\_UPI00098F4FB4\_42\_302 | G | M | V | V | Y | A | Y | A | A | Q | H | P | N | D | V | E | T | L | S | I | M | D | V | P | L | P | G | I | A | P | W | D | E | I | V | - | - | - | - | - | - | - | Q | G | P | R | T | W | H | F |
| 091 UniRef90\_A0A261TYY2\_38\_305 | V | W | I | G | Y | A | Y | A | S | R | H | G | H | T | L | N | K | L | A | L | I | D | A | T | I | P | G | L | T | P | P | E | A | Y | A | - | - | F | E | P | Q | - | R | I | S | K | N | W | H | F |
| 092 UniRef90\_UPI0015F81933\_27\_309 | A | W | V | A | F | S | L | A | L | K | Y | Q | S | R | L | R | G | L | A | L | L | D | A | G | I | P | G | I | T | L | P | D | A | I | P | - | - | T | D | P | E | - | Q | A | W | K | T | W | H | F |
| 093 UniRef90\_A0A0C7N0E5\_9\_278 | A | W | V | A | F | P | Y | A | L | L | F | S | E | E | I | E | R | L | A | L | L | D | A | G | I | P | G | V | T | L | P | D | K | L | P | - | - | T | S | P | D | - | T | A | W | R | I | W | H | F |
| 094 UniRef90\_UPI00112BEE4D\_14\_300 | A | W | V | A | Y | A | Y | A | G | M | F | A | A | D | V | E | R | L | A | L | I | D | A | G | I | P | G | A | T | L | P | D | A | L | P | - | - | S | A | P | E | - | V | A | W | K | T | W | H | F |
| 095 UniRef90\_A0A401Z9D4\_17\_277 | V | S | A | A | Y | A | Y | A | A | R | Y | R | E | A | V | Q | R | L | V | I | L | D | A | P | L | E | G | F | G | R | E | E | Y | - | - | - | - | - | - | - | A | - | A | K | N | S | V | W | H | Y |
| 096 UniRef90\_A0A5J6MKN6\_7\_281 | L | M | V | A | Y | A | Y | A | A | Q | F | P | A | E | V | S | K | V | A | L | M | D | A | F | L | P | G | V | G | D | W | K | D | V | W | - | - | - | - | - | - | - | L | M | R | D | L | W | H | F |
| 097 UniRef90\_A0A4Q7XSL0\_10\_307 | T | M | V | A | Y | A | Y | A | A | R | Y | P | D | K | T | T | R | L | V | V | M | D | A | P | V | P | G | V | P | P | W | E | Q | I | V | - | - | - | - | - | - | - | R | A | P | V | L | W | H | F |
| 098 UniRef90\_UPI0016110D13\_9\_294 | A | W | I | A | F | P | Y | A | L | L | F | G | D | E | I | G | A | L | V | L | M | D | A | G | I | P | A | V | T | M | P | E | M | L | P | - | - | S | S | P | D | - | K | S | W | K | T | W | H | F |
| 099 UniRef90\_A0A520GLB7\_17\_313 | L | M | V | A | Y | A | Y | A | A | Q | F | P | G | E | T | D | K | V | V | L | M | D | A | F | L | P | G | I | G | A | W | Q | N | V | W | - | - | - | - | - | - | - | L | L | R | D | L | W | H | F |
| 100 UniRef90\_A0A252EMP1\_9\_266 | A | R | V | A | Y | A | Y | A | A | R | H | P | T | E | V | K | S | L | V | F | L | E | S | K | I | L | G | I | E | S | D | D | D | - | - | - | - | - | - | - | - | - | A | S | K | E | Y | W | H | F |
| 101 UniRef90\_UPI001668C4B3\_34\_306 | M | W | T | G | Y | A | L | A | A | D | H | P | E | R | L | A | R | L | A | V | A | E | A | A | I | P | G | L | S | P | S | P | P | L | F | - | - | G | S | G | E | - | A | N | D | R | L | W | H | F |
| 102 UniRef90\_UPI00135BC32E\_12\_265 | M | P | T | A | Y | A | Y | A | A | R | Y | R | E | E | V | Q | A | L | A | V | M | E | A | M | L | P | G | V | R | E | D | D | - | - | - | - | - | - | - | - | - | - | K | L | G | M | L | W | H | V |
| 103 UniRef90\_A0A2V9DES3\_17\_290 | L | M | V | A | Y | A | Y | A | A | Q | Y | P | S | E | V | D | R | I | A | L | M | D | A | F | L | P | G | V | G | N | W | R | D | V | W | - | - | - | - | - | - | - | L | M | R | D | L | W | H | F |
| 104 UniRef90\_A0A6P0DAX4\_34\_314 | G | M | V | V | Y | A | Y | A | A | Q | H | P | G | E | V | K | T | L | A | I | L | D | V | P | L | P | G | I | E | P | W | D | E | W | V | - | - | - | - | - | - | - | Q | G | P | R | T | W | H | F |
| 105 UniRef90\_A0A1M5KPU7\_13\_307 | L | M | V | A | Y | A | Y | A | A | Q | Y | P | S | E | V | D | R | L | V | L | M | E | A | F | L | P | G | V | G | D | W | N | S | V | F | - | - | - | - | - | - | - | L | L | R | D | L | W | H | F |
| 106 UniRef90\_A0A2I8DLX4\_17\_295 | A | W | I | A | Y | P | Y | A | A | R | H | A | D | E | V | E | R | V | V | L | L | D | A | N | I | P | G | V | T | L | K | P | A | I | E | - | - | L | G | P | D | - | - | N | W | K | S | W | H | F |
| 107 UniRef90\_A0A5S4WMZ6\_36\_313 | A | M | V | A | Y | A | Y | A | A | Q | W | P | A | E | I | S | K | L | A | L | L | D | V | P | L | P | G | T | Q | V | W | D | E | A | L | - | - | - | - | A | K | - | Q | D | P | Q | I | W | H | F |
| 108 UniRef90\_UPI0003744601\_9\_294 | G | P | V | G | F | A | Y | A | A | S | F | P | A | D | V | R | D | F | I | F | I | E | S | A | V | P | G | F | G | L | E | A | A | M | D | - | - | - | - | - | V | - | A | H | G | G | S | W | H | M |
| 109 UniRef90\_A0A0H3KYM5\_9\_289 | A | W | V | A | F | T | Y | A | M | L | Y | G | E | E | I | R | A | L | T | L | M | D | A | G | I | P | G | I | S | L | P | D | K | L | P | - | - | S | A | S | D | - | K | S | W | K | T | W | H | F |
| 110 UniRef90\_UPI001616C592\_11\_307 | G | W | T | A | Y | A | M | V | A | D | N | P | G | P | V | T | R | L | A | I | A | E | M | L | I | P | G | I | S | P | S | P | P | L | I | - | P | E | D | R | W | - | S | S | D | F | A | W | H | Y |
| 111 UniRef90\_A0A1U9ZZS0\_24\_308 | A | W | V | A | F | S | L | A | L | R | F | E | R | H | L | R | G | V | A | L | L | D | A | G | I | P | G | I | T | L | P | D | A | I | P | - | - | T | D | P | D | - | R | A | W | K | T | W | H | F |
| 112 UniRef90\_UPI00146E50DC\_33\_308 | L | M | V | A | Y | A | Y | A | A | Q | Y | P | A | E | V | K | R | I | V | L | M | D | A | F | L | P | G | V | G | N | W | K | D | V | W | - | - | - | - | - | - | - | L | L | R | D | L | W | H | F |
| 113 UniRef90\_C3KLY5\_13\_287 | A | W | V | A | Y | P | Y | A | A | L | Y | G | S | S | V | R | G | L | A | I | L | D | T | G | I | P | G | I | S | L | P | D | A | L | P | - | - | W | S | S | D | - | V | A | W | R | T | W | H | V |
| 114 UniRef90\_A0A2V6STQ7\_27\_303 | L | M | V | A | Y | A | Y | A | A | Q | Y | P | G | A | V | S | K | V | V | L | M | D | A | F | L | P | G | V | G | K | W | K | D | V | W | - | - | - | - | - | - | - | L | L | R | D | L | W | H | F |
| 115 UniRef90\_UPI0008D5DA8A\_10\_290 | M | W | V | A | Y | A | L | A | S | D | F | P | A | A | V | S | R | L | A | L | T | E | A | V | I | P | G | L | A | P | A | P | T | I | F | - | - | A | P | P | E | - | E | N | I | F | L | W | H | F |
| 116 UniRef90\_A0A3N2H8D2\_16\_299 | A | W | V | A | F | S | L | A | L | N | F | E | N | H | L | R | R | V | A | L | L | D | A | G | I | P | G | V | T | L | P | E | T | I | F | - | - | T | D | P | D | - | R | A | W | K | T | W | H | F |
| 117 UniRef90\_A0A379Z3Q1\_16\_292 | M | W | V | G | Y | A | M | A | S | D | F | P | Q | A | V | T | G | L | V | L | T | E | A | V | I | P | G | L | A | P | A | P | P | I | F | - | - | V | P | A | D | - | Q | N | I | F | L | W | H | F |
| 118 UniRef90\_A0A4R5QBW7\_8\_277 | G | P | T | A | F | S | L | A | A | Q | H | R | D | A | V | R | R | M | A | I | F | D | A | P | V | P | G | D | G | S | P | V | - | - | - | - | - | - | - | - | - | - | F | H | A | G | R | W | H | H |
| 119 UniRef90\_A0A163VVR8\_16\_291 | M | W | I | G | Y | A | M | A | S | D | F | P | Q | A | V | E | R | L | V | L | T | E | A | V | I | P | G | L | A | P | P | P | P | I | F | - | - | V | A | P | E | - | E | N | I | F | L | W | H | F |
| 120 UniRef90\_G0FSK7\_29\_305 | M | W | I | G | Y | A | M | A | A | D | H | P | G | R | V | A | R | L | A | L | A | E | A | L | I | P | G | L | S | P | S | P | P | L | F | - | - | A | P | R | D | - | V | V | E | R | L | W | H | F |
| 121 UniRef90\_A0A4Q7FLU1\_10\_276 | G | P | V | A | Y | A | L | A | V | A | H | P | E | A | V | S | R | L | A | I | L | D | V | T | I | P | G | D | G | T | A | N | F | S | - | - | - | - | - | - | - | - | Q | S | G | K | R | W | H | H |
| 122 UniRef90\_A0A2E3NGG1\_11\_269 | G | P | T | S | Y | A | Y | A | C | A | H | P | D | E | V | R | R | L | C | I | L | D | V | A | I | T | I | D | E | S | E | Q | A | - | - | - | - | - | - | - | E | - | Y | Y | K | R | L | F | H | L |
| 123 UniRef90\_A0A2J9ERN8\_9\_287 | A | W | V | A | F | P | Y | A | H | L | F | A | Q | E | V | E | A | L | A | L | M | D | A | G | I | P | G | V | T | L | P | D | R | L | P | - | - | A | A | S | P | - | D | A | W | K | T | W | H | F |
| 124 UniRef90\_UPI001677C581\_8\_288 | M | W | V | A | Y | A | L | A | S | D | Q | P | D | A | V | R | R | L | A | I | S | E | A | V | I | P | G | L | A | P | A | P | D | I | F | - | - | A | P | P | E | - | Q | N | I | F | L | W | H | F |
| 125 UniRef90\_UPI0003039C4F\_8\_269 | G | P | V | A | F | A | L | A | A | Q | H | R | K | A | I | L | G | L | A | A | F | D | V | P | V | P | G | D | G | A | P | L | - | - | - | - | - | - | - | - | - | - | A | A | M | A | R | W | H | F |
| 126 UniRef90\_A0A1B1YXM9\_17\_276 | G | A | V | A | Y | A | L | A | A | Q | D | R | A | L | V | T | Q | L | A | I | L | D | M | L | L | P | G | I | E | L | P | G | - | - | - | - | - | L | G | A | N | - | A | L | A | S | Y | W | H | F |
| 127 UniRef90\_A0A534ZXC6\_31\_288 | L | M | V | A | Y | A | Y | A | T | Q | F | P | A | E | V | E | K | L | V | V | M | D | A | F | L | P | G | V | A | G | W | E | R | V | Y | - | - | - | - | - | - | - | D | D | P | G | I | W | H | F |
| 128 UniRef90\_A0A2X1TAZ7\_42\_319 | M | W | V | G | Y | A | L | A | A | D | Y | P | A | D | I | K | R | I | A | L | T | E | A | V | I | P | G | L | A | P | A | P | A | I | F | - | - | V | D | P | E | - | E | N | I | F | L | W | H | F |
| 129 UniRef90\_A0A4Q5QRH4\_8\_282 | A | W | V | A | F | P | Y | A | L | M | F | G | N | E | I | E | A | L | A | L | M | D | A | G | I | P | G | V | T | L | P | D | M | L | P | - | - | S | A | S | D | - | K | S | W | K | T | W | H | F |
| 130 UniRef90\_A0A0D6HQT0\_17\_294 | A | W | I | A | Y | P | Y | A | A | R | H | A | D | E | V | E | R | L | V | L | L | D | A | N | I | P | G | V | T | L | P | A | S | F | E | - | - | L | G | P | D | - | - | N | W | K | R | W | H | F |
| 131 UniRef90\_A0A1G4JCM6\_9\_275 | A | W | V | A | F | P | Y | A | A | M | F | G | N | E | V | E | R | L | A | M | L | D | A | G | I | P | G | V | T | L | P | D | M | L | P | - | - | S | D | P | D | - | R | A | W | R | T | W | H | F |
| 132 UniRef90\_UPI001269E708\_28\_301 | A | W | V | A | F | S | L | A | L | T | Y | E | D | R | L | G | G | V | V | L | L | D | A | G | I | P | G | V | S | L | P | A | A | V | P | - | - | T | D | P | E | - | Q | A | W | K | T | W | H | F |
| 133 UniRef90\_A0A538SUN7\_3\_303 | N | M | V | G | Y | A | L | A | T | Q | Y | P | T | Q | I | A | R | W | V | V | I | D | A | P | L | P | G | I | G | D | W | E | H | I | V | - | - | - | - | - | - | - | C | S | P | Q | V | W | H | F |
| 134 UniRef90\_UPI001646B9D9\_15\_288 | M | W | V | A | Y | A | L | A | S | D | I | P | Q | A | V | R | S | L | A | V | A | E | A | V | I | P | G | L | A | E | A | P | T | I | F | - | - | V | A | P | Q | - | E | N | I | F | L | W | H | F |
| 135 UniRef90\_K9DQK6\_17\_307 | T | M | V | A | Y | A | Y | A | V | R | Y | P | D | K | V | T | R | L | V | V | M | D | A | P | V | P | G | V | A | P | W | D | E | L | V | - | - | - | - | - | - | - | L | D | P | R | L | W | H | F |
| 136 UniRef90\_A0A2N9BM66\_25\_297 | M | L | V | G | Y | A | L | A | A | S | Y | R | D | R | V | T | R | L | V | V | S | E | A | I | L | P | G | L | S | P | S | P | P | L | L | - | - | S | D | P | A | - | T | N | E | M | L | W | H | F |
| 137 UniRef90\_A0A370LBV7\_5\_266 | L | M | V | A | Y | A | Y | A | A | Q | Y | P | D | E | V | S | R | L | A | L | M | D | A | F | L | P | G | V | G | D | W | T | N | V | W | - | - | - | - | - | - | - | L | L | R | D | L | W | H | F |
| 138 UniRef90\_A0A2V9ZM06\_3\_258 | L | M | V | A | Y | A | Y | A | A | Q | F | P | A | E | V | A | K | L | V | V | M | D | A | F | L | P | G | V | A | G | W | E | A | V | Y | - | - | - | - | - | - | - | N | N | P | S | I | W | H | F |
| 139 UniRef90\_UPI00101F418B\_26\_310 | A | W | I | S | Y | A | W | V | A | D | W | P | G | D | I | G | R | L | A | L | Y | D | A | A | L | P | G | I | T | P | P | P | P | A | G | - | V | L | S | E | E | - | G | N | I | R | T | W | H | F |
| 140 UniRef90\_A0A4V2YYI8\_27\_305 | M | W | T | G | Y | A | L | A | A | D | H | P | D | R | L | E | R | L | V | V | A | E | A | L | I | P | G | L | S | P | S | P | P | L | F | - | - | S | G | R | E | - | A | N | T | R | L | W | H | F |
| 141 UniRef90\_UPI001487D2FB\_8\_292 | T | M | V | A | F | A | Y | A | A | R | Y | P | E | K | T | E | R | L | V | V | M | D | A | P | V | P | G | I | G | P | W | D | E | I | V | - | - | - | - | - | - | - | R | N | P | L | L | W | H | F |
| 142 UniRef90\_B9XAH7\_320\_607 | T | W | I | A | F | A | H | A | T | A | F | P | H | D | V | R | R | L | V | L | S | E | A | A | I | P | G | I | S | S | L | P | G | G | A | - | - | P | D | S | A | - | T | N | L | K | T | W | H | F |
| 143 UniRef90\_UPI0013696204\_44\_306 | M | W | T | G | Y | A | L | A | A | D | H | P | G | R | L | D | R | L | V | V | A | E | A | A | I | P | G | L | A | P | S | P | P | L | F | - | - | G | D | R | E | - | L | N | D | R | L | W | H | F |
| 144 UniRef90\_W5WIM6\_13\_292 | M | W | T | G | Y | A | L | A | A | D | H | P | E | R | L | E | R | L | A | V | A | E | A | V | I | P | G | L | A | P | S | P | P | L | F | - | - | A | P | Q | E | - | A | I | E | R | L | W | H | F |
| 145 UniRef90\_A0A2X2GG63\_10\_290 | M | W | I | G | Y | A | M | A | S | D | F | P | Q | A | V | T | G | L | V | L | T | E | A | V | I | P | G | L | A | P | A | P | P | I | F | - | - | V | P | A | E | - | Q | N | I | F | L | W | H | F |
| 146 UniRef90\_A0A2E2YI39\_6\_274 | G | P | T | A | Y | A | L | A | A | S | H | P | Q | A | V | E | K | L | V | I | L | D | V | V | V | P | G | C | G | G | D | F | S | - | - | - | - | - | - | - | - | - | E | G | G | R | R | W | H | H |
| 147 UniRef90\_A0A2W7G3K6\_24\_312 | T | W | I | G | Y | A | L | A | A | D | W | P | A | D | V | R | R | L | A | V | F | D | A | A | L | P | G | I | S | A | P | P | P | A | G | - | I | P | S | A | E | - | A | N | V | K | T | W | H | F |
| 148 UniRef90\_A0A6B2RWJ8\_28\_298 | A | W | V | A | F | S | M | A | L | K | Y | E | E | R | L | H | G | V | A | L | L | D | A | G | I | P | G | I | T | L | P | D | S | I | P | - | - | T | D | P | D | - | R | A | W | K | T | W | H | F |
| 149 UniRef90\_A0A537A198\_6\_270 | G | P | C | G | F | A | L | A | C | A | A | P | Q | S | V | R | T | L | T | I | L | D | V | T | I | P | G | I | G | P | D | I | S | - | - | - | - | - | - | - | - | - | Q | G | G | L | R | W | H | H |
| 150 UniRef90\_UPI00135F2885\_48\_334 | G | Q | V | A | F | A | I | A | R | N | H | P | E | M | V | Q | R | L | A | I | L | D | V | P | L | M | G | M | P | Y | S | E | A | L | A | - | - | - | - | - | - | - | - | - | - | - | P | W | H | F |

  
  

|  |  |  |  |  |  |  |  |  |  |  |  |  |  |  |  |  |  |  |  |  |  |  |  |  |  |  |  |  |  |  |  |  |  |  |  |  |  |  |  |  |  |  |  |  |  |  |  |  |  |  |
| --- | --- | --- | --- | --- | --- | --- | --- | --- | --- | --- | --- | --- | --- | --- | --- | --- | --- | --- | --- | --- | --- | --- | --- | --- | --- | --- | --- | --- | --- | --- | --- | --- | --- | --- | --- | --- | --- | --- | --- | --- | --- | --- | --- | --- | --- | --- | --- | --- | --- | --- |
| **001 Input\_protein\_seq** | S | - | F | F | A | A | D | D | R | L | A | E | T | L | I | A | G | K | E | R | F | F | L | E | H | F | I | K | - | S | H | A | S | - | N | T | - | E | V | F | S | E | R | L | L | D | L | Y | A | R |
| 002 UniRef90\_A0A2G0Y5Q3\_5\_314 | S | - | F | F | A | A | N | G | K | L | A | E | T | L | I | T | G | K | E | K | F | F | L | S | H | F | I | K | - | S | H | G | T | - | N | T | - | S | V | F | T | E | K | L | L | D | L | Y | A | K |
| 003 UniRef90\_A0A172YJZ3\_2\_306 | S | - | F | F | A | A | G | E | R | L | A | E | T | L | I | A | G | N | E | R | V | F | F | E | H | F | I | K | - | E | H | A | T | - | N | R | - | D | V | F | T | P | E | L | L | D | L | Y | A | A |
| 004 UniRef90\_A0A4Z1C7D3\_9\_314 | S | - | F | F | A | A | S | E | N | L | A | E | T | L | V | T | G | N | E | R | M | F | L | E | H | F | I | K | - | E | H | A | T | - | N | R | - | E | A | F | T | D | E | V | L | D | L | Y | A | A |
| 005 UniRef90\_A0A0A3Z1S4\_7\_319 | S | - | F | F | A | A | K | N | Q | L | A | E | T | L | I | K | G | H | E | K | F | F | L | T | H | F | I | L | - | E | H | A | T | - | N | K | - | A | A | F | T | P | A | L | L | D | R | Y | A | K |
| 006 UniRef90\_A0A329J6I4\_9\_315 | S | - | F | F | A | A | G | D | H | M | A | E | T | L | I | K | G | H | E | K | F | F | L | K | R | F | I | Q | - | S | H | A | A | - | S | M | - | D | A | F | T | P | K | L | I | D | M | Y | A | K |
| 007 UniRef90\_A0A4V1G750\_8\_314 | S | - | F | F | A | A | D | G | Q | L | A | E | K | L | I | T | G | K | E | R | L | F | F | E | H | F | I | K | - | T | H | A | V | - | N | K | - | A | V | F | T | P | K | L | L | D | M | Y | A | K |
| 008 UniRef90\_T2L220\_17\_309 | S | - | F | F | A | A | E | Q | Q | L | A | E | K | L | V | S | G | K | E | R | I | F | F | E | H | F | I | K | - | V | H | A | A | - | N | K | - | E | V | F | T | P | E | L | L | D | L | Y | A | S |
| 009 UniRef90\_UPI000C2FE79C\_12\_314 | S | - | F | F | S | A | D | N | Q | L | A | E | T | L | I | K | G | K | E | R | Y | F | F | E | H | F | I | K | - | V | H | A | T | - | N | T | - | S | V | F | T | P | Q | L | L | D | L | Y | G | K |
| 010 UniRef90\_UPI00102FACD2\_13\_314 | S | - | F | F | T | A | K | N | N | L | A | E | T | L | I | A | G | K | E | R | L | F | F | E | H | F | I | K | - | E | H | A | T | - | N | T | - | S | V | F | T | S | Q | L | L | D | L | Y | A | N |
| 011 UniRef90\_UPI0014749FF6\_10\_312 | S | - | F | F | S | A | D | G | A | L | P | E | T | L | I | S | G | N | E | K | V | F | F | S | H | F | I | K | - | K | H | A | E | - | K | P | - | E | A | F | T | E | K | M | L | D | L | Y | A | K |
| 012 UniRef90\_A0A3M4V4S1\_41\_332 | S | - | F | F | A | A | S | G | Q | L | A | D | A | M | V | K | G | N | E | R | L | F | L | T | H | F | I | R | - | H | H | A | S | - | N | Q | - | A | V | F | T | D | E | L | L | E | S | Y | V | H |
| 013 UniRef90\_A0A3L8C981\_31\_324 | S | - | F | F | A | A | S | D | Q | L | A | D | A | M | V | K | G | N | E | R | L | F | L | T | H | F | I | R | - | H | H | A | S | - | N | Q | - | E | A | L | S | D | E | L | I | D | R | Y | V | R |
| 014 UniRef90\_A0A1X0N2L9\_32\_323 | S | - | F | F | A | A | S | D | Q | L | A | D | A | M | V | K | G | N | E | R | L | F | L | T | H | F | I | R | - | H | H | A | S | - | N | Q | - | A | V | F | T | D | D | L | I | E | R | Y | V | R |
| 015 UniRef90\_UPI00166CAA4C\_15\_313 | G | - | F | F | A | T | - | P | V | M | P | E | F | L | T | R | G | R | E | Q | A | F | L | E | R | F | V | R | - | D | R | T | V | - | N | Q | - | E | A | F | T | A | A | D | F | N | E | Y | G | R |
| 016 UniRef90\_UPI001661A3FF\_47\_334 | G | - | F | F | N | L | T | D | G | L | P | E | N | M | I | R | G | R | E | V | S | W | T | A | G | F | I | G | - | G | F | E | G | - | V | P | - | G | A | V | S | Q | D | D | I | R | V | F | A | H |
| 017 UniRef90\_A0A1H5MZC9\_42\_331 | G | - | F | F | A | L | G | N | G | L | P | E | D | L | I | Q | G | R | E | E | V | W | T | D | R | F | I | D | - | D | L | E | V | - | R | K | - | G | A | V | T | P | E | D | V | S | V | F | A | G |
| 018 UniRef90\_UPI000690E8EC\_26\_310 | G | - | F | F | S | L | V | N | G | L | P | E | G | V | V | S | G | R | E | A | L | W | V | R | K | F | I | D | - | G | L | E | V | - | Q | K | - | G | S | V | T | P | G | D | V | A | V | F | A | H |
| 019 UniRef90\_R4LNI7\_31\_317 | G | - | F | F | S | L | T | N | G | L | P | E | D | L | V | R | G | R | E | A | R | W | V | Q | L | F | T | D | - | A | L | E | V | - | N | K | - | D | G | I | G | P | A | E | V | A | E | F | A | R |
| 020 UniRef90\_A0A4R2C838\_67\_350 | G | - | L | F | A | E | K | D | G | L | A | E | D | L | M | T | G | R | E | Q | Q | W | V | T | D | F | M | P | - | T | S | E | V | - | V | K | - | G | A | V | T | A | C | D | M | D | I | Y | A | H |
| 021 UniRef90\_A0A2P9HD85\_25\_319 | G | - | Y | F | S | F | - | G | D | I | A | E | K | M | V | S | T | D | P | V | L | F | M | S | Y | F | I | K | - | E | Y | A | G | - | K | R | - | D | V | F | T | P | E | L | L | D | E | L | I | E |
| 022 UniRef90\_UPI000D14D87C\_43\_332 | G | - | F | F | S | L | Q | T | G | L | P | E | S | L | I | R | G | R | E | L | T | W | T | T | G | F | M | R | - | G | I | A | V | - | H | K | - | D | A | L | T | P | E | D | L | Q | V | F | S | S |
| 023 UniRef90\_A0A1Q4ZL08\_6\_289 | G | - | F | F | S | L | P | N | G | L | P | E | Q | I | V | A | G | R | E | E | L | W | V | D | R | F | T | D | - | S | M | E | V | - | Q | K | - | T | G | I | G | A | D | E | V | A | E | Y | S | R |
| 024 UniRef90\_A0A1I2GGE7\_30\_316 | G | - | F | F | S | L | T | N | G | L | P | E | Q | M | V | D | G | R | E | E | L | W | V | K | L | F | T | D | - | Q | L | M | D | - | V | K | - | G | A | I | G | P | E | D | V | K | V | Y | A | K |
| 025 UniRef90\_UPI0005580D51\_18\_305 | G | - | F | F | S | L | T | N | G | L | P | E | Q | L | V | Q | G | R | E | E | V | W | V | D | R | F | T | D | - | S | L | E | V | - | Q | K | - | N | A | I | G | P | V | E | V | R | E | F | A | R |
| 026 UniRef90\_A0A2W2F1P0\_6\_289 | G | - | F | F | S | L | T | N | G | L | P | E | Q | L | V | A | G | R | E | A | L | W | V | D | R | F | T | D | - | S | L | M | I | - | R | K | - | G | S | L | D | A | T | D | I | E | E | Y | A | R |
| 027 UniRef90\_UPI0010F9F40F\_6\_288 | G | - | F | F | S | L | T | N | G | L | P | E | Q | L | V | A | G | R | E | V | L | W | V | D | R | F | T | D | - | S | L | M | L | - | R | K | - | G | S | L | D | A | A | D | I | E | E | Y | A | R |
| 028 UniRef90\_A0A3E2YQE6\_6\_289 | G | - | F | F | S | L | T | N | G | L | P | E | Q | L | V | S | G | R | E | T | L | W | V | D | R | F | T | D | - | S | M | M | V | - | R | K | - | G | S | I | G | P | D | D | V | E | E | Y | A | R |
| 029 UniRef90\_A0A1A9ACL3\_6\_288 | G | - | F | F | T | V | P | N | G | L | P | E | Q | L | V | V | G | R | E | A | L | W | V | D | R | F | S | D | - | S | I | M | V | - | N | K | - | G | S | L | G | P | D | D | I | E | E | Y | A | R |
| 030 UniRef90\_UPI00174B5D19\_38\_323 | G | - | L | F | Q | - | - | K | K | L | A | E | Q | L | V | R | G | H | E | R | E | F | V | E | G | F | V | D | - | Q | Y | L | A | - | V | K | - | D | A | F | G | P | A | D | Y | E | Y | Y | A | R |
| 031 UniRef90\_A0A495JCH5\_50\_333 | G | - | F | F | N | V | T | N | G | L | P | E | Q | T | V | K | G | R | E | T | Q | W | I | E | R | F | T | D | - | M | L | E | Y | - | N | K | - | D | G | V | T | P | G | D | A | A | I | Y | G | Y |
| 032 UniRef90\_UPI0013D14F7B\_11\_294 | G | - | F | F | N | L | A | N | G | F | A | D | E | L | I | A | G | N | E | K | L | W | V | T | K | F | I | D | - | A | L | E | M | - | I | K | - | G | S | V | T | D | E | D | I | S | I | Y | A | E |
| 033 UniRef90\_UPI00036EC6E3\_56\_342 | G | - | L | F | A | E | K | N | G | L | A | E | D | L | M | T | G | R | E | Q | Q | W | V | T | D | F | M | P | - | T | S | E | V | - | V | K | - | G | A | V | T | A | C | D | M | D | I | Y | A | H |
| 034 UniRef90\_A0A1H3P9Q0\_6\_290 | G | - | F | F | S | V | P | N | G | L | P | E | F | I | V | E | G | R | E | A | L | W | V | D | R | F | T | D | - | S | M | L | V | - | Q | K | - | T | G | I | R | A | A | D | V | A | E | Y | A | R |
| 035 UniRef90\_A0A385B2U0\_41\_332 | G | - | F | F | Q | - | - | K | D | F | A | Q | D | L | V | R | G | H | E | R | D | L | I | K | G | F | V | E | - | Q | Y | L | A | - | V | D | - | G | A | F | D | D | R | D | Y | E | F | Y | A | R |
| 036 UniRef90\_UPI001430CB85\_25\_306 | A | - | F | H | S | I | - | P | D | L | P | E | L | L | I | A | G | K | E | R | A | Y | L | D | W | F | L | R | - | R | K | T | A | - | N | P | - | Q | T | F | S | D | A | D | M | D | E | Y | V | R |
| 037 UniRef90\_A0A109IHW8\_6\_288 | G | - | F | F | N | L | T | N | G | L | P | E | E | L | V | T | G | R | E | A | R | W | V | D | R | F | T | D | - | S | I | M | V | - | H | K | - | G | S | I | G | A | D | D | I | E | E | Y | A | R |
| 038 UniRef90\_A0A2T6L0M5\_14\_298 | G | - | F | F | Q | - | - | A | D | I | A | E | G | L | I | A | G | N | E | R | A | L | V | E | G | F | I | G | - | Q | F | L | G | - | D | Q | - | T | A | F | A | A | V | D | Y | E | F | Y | A | E |
| 039 UniRef90\_A0A1C4Z0V4\_6\_288 | G | - | F | F | N | L | A | N | G | L | P | E | E | L | V | A | G | R | E | S | L | W | V | D | R | F | T | D | - | S | L | M | V | - | R | K | - | G | S | I | G | A | D | D | I | E | E | Y | A | R |
| 040 UniRef90\_A0A4U3M272\_82\_365 | G | - | L | F | A | E | K | N | G | L | A | E | D | L | M | T | G | R | E | Q | Q | W | V | T | D | F | M | P | - | T | S | E | V | - | V | K | - | G | A | L | T | P | C | D | L | A | I | Y | A | H |
| 041 UniRef90\_UPI00052728DD\_22\_294 | A | - | F | H | A | I | - | P | D | L | P | E | L | L | I | E | G | K | E | R | A | Y | L | D | W | F | L | R | - | R | K | T | A | - | N | P | - | Q | T | F | S | E | A | D | M | D | E | Y | G | R |
| 042 UniRef90\_A0A6B8MSH7\_52\_336 | G | - | Y | F | S | F | - | G | D | I | A | E | K | Q | I | A | S | D | P | S | L | F | F | S | Y | F | I | K | - | T | Y | A | G | - | K | K | - | D | I | F | T | P | Q | L | L | S | E | L | I | E |
| 043 UniRef90\_I4VSM6\_28\_303 | A | - | F | H | V | I | - | P | D | L | P | E | M | L | I | A | G | H | E | R | E | Y | L | A | W | F | L | R | - | R | K | A | A | - | D | P | - | S | V | F | S | E | Q | D | L | D | E | Y | L | R |
| 044 UniRef90\_A0A1I3BFK1\_40\_314 | A | - | F | H | A | V | - | P | D | L | P | E | I | L | I | S | G | R | E | R | E | Y | L | E | W | F | L | R | - | R | K | A | A | - | N | P | - | E | V | F | S | E | A | D | I | E | E | Y | L | R |
| 045 UniRef90\_A0A1Z4J856\_42\_316 | S | - | F | Q | A | T | - | R | D | L | P | E | A | L | V | E | G | K | E | R | L | Y | L | S | V | F | L | R | - | P | F | T | Y | - | N | P | - | A | A | I | T | E | D | A | M | D | E | Y | V | R |
| 046 UniRef90\_A0A0M4FVH2\_27\_314 | A | - | F | H | V | I | - | P | D | L | P | E | M | L | I | A | G | R | E | R | E | Y | L | D | W | F | L | R | - | R | K | A | A | - | N | P | - | E | A | F | S | E | A | D | I | E | E | Y | L | R |
| 047 UniRef90\_UPI001032135D\_25\_299 | A | - | F | H | T | L | - | P | D | L | P | E | A | L | I | A | G | R | E | R | L | Y | L | E | W | F | L | K | - | R | K | T | A | - | A | P | - | D | T | F | S | D | E | D | I | N | E | Y | L | R |
| 048 UniRef90\_D5WL63\_24\_300 | A | - | F | H | V | L | - | P | D | L | P | E | V | L | L | A | G | K | E | R | E | Y | L | D | W | F | L | R | - | R | K | T | A | - | N | P | - | D | T | F | T | D | A | D | I | D | E | Y | L | R |
| 049 UniRef90\_A0A1H3E943\_18\_291 | L | - | F | H | P | V | - | S | D | L | P | E | I | L | I | T | G | R | E | R | E | Y | I | E | W | F | F | Q | - | R | K | T | Y | - | N | P | K | A | T | F | S | Q | E | D | I | D | V | Y | V | N |
| 050 UniRef90\_A0A346N7R3\_30\_317 | H | - | F | Y | G | - | - | - | E | V | P | L | K | L | V | K | G | R | E | R | I | Y | F | E | H | F | W | N | - | D | F | A | A | - | D | R | N | H | S | V | S | E | A | D | R | Q | F | Y | A | K |
| 051 UniRef90\_A0A537Q000\_40\_318 | M | - | F | N | Q | L | - | R | D | L | P | E | F | L | V | A | G | R | E | D | A | Y | M | R | W | V | V | D | - | H | L | A | Y | - | R | P | - | E | R | V | A | - | - | - | V | D | E | Y | T | R |
| 052 UniRef90\_E6V5X5\_24\_320 | H | - | F | H | G | - | - | - | A | T | P | L | A | L | V | K | G | R | E | R | I | Y | F | E | H | F | W | N | - | D | F | A | A | - | D | P | K | H | S | V | P | E | A | D | R | Q | F | Y | A | R |
| 053 UniRef90\_A0A484PNM3\_14\_291 | F | - | F | L | A | M | - | P | D | L | A | E | A | L | L | T | G | R | E | R | A | F | L | A | W | L | F | Q | - | S | K | S | A | - | N | V | - | D | W | L | D | A | R | A | L | D | E | Y | V | R |
| 054 UniRef90\_UPI0011BDEFB8\_30\_311 | A | - | F | H | S | I | - | A | D | L | P | E | A | L | I | T | G | R | E | R | I | Y | L | E | W | F | L | K | - | R | K | T | A | - | C | P | - | D | V | F | T | E | D | D | L | A | E | Y | L | R |
| 055 UniRef90\_A0A1H2UZ41\_16\_293 | F | - | F | H | Q | V | - | A | D | L | P | E | I | L | I | T | G | R | E | R | S | Y | I | E | W | F | F | Q | - | R | K | T | Y | - | N | P | A | G | T | F | S | Q | A | D | I | D | E | Y | E | R |
| 056 UniRef90\_A0A1M5ZT81\_29\_302 | A | - | F | H | A | I | - | P | D | L | P | E | M | L | I | A | G | R | E | R | E | Y | L | E | W | F | L | R | - | R | K | A | A | - | N | P | - | E | T | F | S | D | A | D | I | D | E | Y | L | R |
| 057 UniRef90\_A0A135GL34\_14\_283 | S | - | F | H | A | A | - | S | D | V | A | E | M | L | I | S | G | K | E | R | E | Y | L | R | W | F | Y | R | - | M | Q | M | Y | - | N | L | - | D | G | I | T | D | E | D | I | D | V | Y | A | R |
| 058 UniRef90\_A0A484UEV2\_23\_280 | F | - | F | L | A | M | - | P | D | L | A | E | A | L | L | T | G | R | E | R | E | F | L | S | W | L | F | H | - | S | K | S | A | - | N | V | - | D | W | L | D | A | A | A | L | D | E | Y | V | R |
| 059 UniRef90\_A0A495NUG6\_51\_333 | G | - | F | F | S | L | T | N | G | L | P | E | Q | T | I | D | Q | H | E | S | E | W | V | A | G | F | V | D | - | W | L | T | V | - | D | K | - | E | A | F | T | R | E | D | T | D | V | Y | A | C |
| 060 UniRef90\_A0A1M5MA45\_15\_292 | G | - | F | F | S | - | - | R | P | I | A | G | D | L | V | A | G | K | E | K | E | F | L | Y | D | F | W | P | D | Q | Y | G | V | - | N | K | - | E | P | F | T | Q | A | E | K | D | E | F | V | R |
| 061 UniRef90\_UPI000EF95C7F\_30\_312 | N | - | F | N | R | A | - | H | G | I | N | E | Q | L | V | T | G | R | E | D | L | Y | F | G | H | Q | F | A | - | T | K | A | A | - | T | P | - | T | S | I | P | A | E | A | V | D | T | Y | V | Q |
| 062 UniRef90\_L9WLS3\_15\_271 | R | - | F | H | G | V | - | R | D | L | P | E | R | L | V | A | G | R | E | R | M | Y | L | E | W | F | Y | K | - | E | G | A | Y | - | D | P | - | A | A | I | D | S | D | A | R | D | E | Y | V | R |
| 063 UniRef90\_UPI0005D31B04\_21\_288 | G | - | F | F | R | A | - | P | N | L | A | E | A | L | I | A | G | R | E | K | M | F | I | T | H | F | I | R | - | H | L | A | Y | - | D | T | - | Y | A | L | S | D | A | D | L | N | E | Y | A | E |
| 064 UniRef90\_UPI001408C682\_10\_298 | H | - | F | Y | G | - | - | - | E | T | P | L | A | L | V | Q | G | R | E | R | I | Y | F | E | H | F | W | N | - | D | F | S | A | - | D | K | T | K | S | V | K | E | A | D | R | K | I | Y | A | A |
| 065 UniRef90\_A0A1Y6KKD8\_22\_314 | A | - | F | H | V | V | - | A | D | L | P | E | M | L | I | Q | G | R | E | R | D | Y | L | A | W | F | L | R | - | R | K | G | A | - | N | P | - | E | V | F | S | D | S | D | L | D | E | Y | A | R |
| 066 UniRef90\_A0A0X3RYG7\_65\_349 | G | - | L | F | Q | - | - | M | P | L | A | E | K | L | I | A | G | H | E | R | V | I | V | H | D | M | M | L | - | E | Y | V | N | R | D | K | - | S | P | F | T | A | S | D | F | D | Y | Y | S | H |
| 067 UniRef90\_A0A2T0T762\_22\_302 | A | - | F | N | R | L | - | P | D | L | N | E | E | L | V | R | G | R | E | R | L | F | F | G | Y | Q | F | A | - | T | K | A | A | - | T | P | - | T | T | I | P | E | H | A | V | D | V | Y | V | D |
| 068 UniRef90\_A0A2V6QCX2\_10\_286 | H | - | F | Y | G | - | - | - | D | V | P | L | A | L | V | K | G | R | E | R | I | Y | L | E | H | F | W | N | - | D | F | A | A | - | D | R | T | R | S | V | P | E | A | D | R | R | L | Y | A | R |
| 069 UniRef90\_D8MTC4\_28\_309 | A | - | F | H | V | I | - | P | D | L | P | E | A | L | I | A | G | N | E | R | V | Y | L | E | W | F | L | K | - | R | K | T | A | - | C | P | - | D | V | F | S | E | T | D | M | E | E | Y | L | R |
| 070 UniRef90\_UPI0012FADC80\_10\_303 | G | - | F | F | A | - | - | R | P | I | A | G | S | L | V | A | G | H | E | K | E | F | L | T | D | F | W | P | - | Q | V | G | Y | - | V | K | - | N | P | F | T | Q | T | E | K | D | E | F | I | R |
| 071 UniRef90\_A0A2E5PAM0\_2\_278 | G | - | F | H | W | V | - | Q | D | L | P | E | A | L | V | A | G | R | E | R | X | Y | L | S | W | F | Y | K | - | N | L | A | Y | - | N | P | - | S | A | I | X | P | K | D | I | D | E | Y | E | R |
| 072 UniRef90\_T0HVL8\_12\_269 | G | - | F | H | Q | E | - | A | D | L | P | D | L | L | L | A | G | R | E | R | E | Y | L | S | F | F | Y | K | - | R | Y | A | F | - | D | S | - | R | A | I | N | A | E | D | I | D | E | Y | V | R |
| 073 UniRef90\_A0A3N4T2I8\_17\_298 | G | - | F | N | R | L | - | D | D | L | N | E | E | L | V | R | G | R | E | Q | L | F | F | G | W | Q | F | A | - | T | K | A | A | - | T | P | - | T | A | I | P | A | Y | A | V | D | V | Y | V | D |
| 074 UniRef90\_A0A433IAT3\_19\_312 | H | - | F | Y | G | - | - | - | E | V | P | L | A | L | V | N | G | R | E | R | I | Y | F | E | H | F | W | N | - | D | F | A | A | - | D | P | H | H | S | I | P | E | A | D | R | R | F | Y | A | E |
| 075 UniRef90\_A0A226WR47\_30\_304 | A | - | F | H | A | I | - | P | E | L | P | E | M | L | I | T | G | R | E | R | E | Y | L | D | W | F | L | R | - | R | K | T | A | - | N | P | - | E | S | F | P | D | A | D | V | E | E | Y | L | R |
| 076 UniRef90\_UPI000DD77D66\_25\_314 | R | - | F | H | A | V | - | R | D | V | P | E | M | L | I | S | G | R | E | F | E | Y | L | K | W | F | H | N | - | A | E | G | V | - | N | S | - | R | A | F | D | N | Q | A | D | E | I | Y | G | R |
| 077 UniRef90\_A0A2A5QV31\_6\_277 | R | - | F | H | S | V | - | R | D | L | P | E | R | L | V | A | G | R | E | R | L | Y | L | D | W | F | Y | G | - | E | G | A | Y | - | D | P | - | T | A | I | D | D | D | A | R | E | E | Y | V | R |
| 078 UniRef90\_A0A127EY60\_8\_296 | H | - | F | Y | G | - | - | - | E | T | P | L | K | L | V | A | G | R | E | R | I | Y | F | E | H | F | W | N | - | D | F | A | A | - | N | P | K | K | S | V | S | E | T | D | R | R | F | Y | T | K |
| 079 UniRef90\_UPI0004952425\_6\_308 | H | - | F | Y | G | - | - | - | K | T | P | L | A | L | V | T | G | R | E | R | I | Y | L | E | H | F | W | N | - | D | F | A | A | - | D | P | T | K | S | V | S | E | A | D | R | Q | F | Y | A | K |
| 080 UniRef90\_A0A0D0JP71\_56\_330 | G | - | F | F | A | - | - | W | P | A | S | G | D | I | V | K | G | K | E | K | E | F | L | T | N | F | W | P | - | M | V | G | H | - | V | K | - | N | P | F | T | A | E | E | T | A | E | F | V | R |
| 081 UniRef90\_A0A6A7LIC2\_1\_257 | S | - | F | H | Q | T | - | R | D | V | P | E | L | L | T | A | G | N | E | R | E | Y | L | S | W | H | Y | L | - | G | L | A | Y | - | N | P | - | E | A | I | T | E | A | D | I | D | T | Y | V | S |
| 082 UniRef90\_UPI00097BCF81\_14\_312 | H | - | F | H | G | - | - | - | P | T | P | L | A | L | V | K | G | R | E | R | T | Y | F | E | H | F | W | N | - | D | F | A | A | - | D | P | K | R | S | V | P | E | A | D | R | R | V | Y | A | K |
| 083 UniRef90\_UPI001591AA5D\_7\_297 | G | - | F | N | R | L | - | D | G | L | N | E | E | L | V | R | G | R | E | R | L | F | F | G | S | Q | F | A | - | T | K | A | A | - | T | P | - | A | A | I | P | S | D | A | V | D | V | Y | V | D |
| 084 UniRef90\_A0A4P8YCR5\_18\_300 | A | - | F | H | M | I | - | A | D | L | P | E | A | L | I | S | G | R | E | R | L | Y | L | D | W | F | L | R | - | R | K | T | A | - | F | P | - | D | A | F | S | E | Q | D | I | D | E | Y | L | R |
| 085 UniRef90\_A0A4R0GJE6\_14\_295 | G | - | F | N | R | L | - | T | D | L | N | E | E | L | V | R | G | R | E | R | L | F | F | G | W | Q | F | A | - | T | K | A | A | - | T | P | - | T | T | I | P | E | Y | A | V | D | V | Y | V | D |
| 086 UniRef90\_A0A502C146\_32\_301 | H | - | F | Y | G | - | - | - | E | T | P | L | K | L | V | D | G | R | E | R | I | Y | F | D | H | F | W | N | - | D | F | A | A | - | D | R | N | H | S | I | S | E | A | D | R | A | F | Y | T | K |
| 087 UniRef90\_A0A4Y9SAQ9\_19\_291 | H | - | F | F | G | - | - | - | E | T | P | E | R | L | V | Q | G | R | E | R | I | Y | F | E | H | F | W | N | - | D | F | A | A | - | D | R | N | H | S | I | S | E | Q | D | R | K | L | Y | A | A |
| 088 UniRef90\_A0A327RPK7\_10\_286 | G | - | F | H | M | V | - | H | D | L | P | E | A | L | I | T | G | R | E | H | I | Y | L | D | H | F | F | K | - | T | L | A | Y | - | N | P | - | T | S | I | K | E | E | A | I | A | E | Y | V | R |
| 089 UniRef90\_A0A239MTD5\_1\_269 | G | - | M | F | Q | - | - | M | P | L | A | E | H | L | I | A | G | H | E | R | V | F | I | Q | E | M | I | T | - | A | Y | L | V | G | N | K | - | S | P | F | T | P | S | D | Y | D | Y | Y | A | H |
| 090 UniRef90\_UPI00098F4FB4\_42\_302 | R | - | F | H | S | V | - | R | D | V | P | E | M | L | I | A | G | R | E | L | E | Y | L | K | W | F | H | N | - | A | E | G | V | - | N | T | - | R | A | F | D | N | E | A | D | E | I | Y | G | R |
| 091 UniRef90\_A0A261TYY2\_38\_305 | Y | - | F | F | A | M | - | P | D | L | A | E | A | L | L | T | G | R | E | R | E | F | L | G | W | L | F | Q | - | S | K | S | A | - | G | V | - | Q | W | I | E | P | H | A | L | D | E | Y | A | R |
| 092 UniRef90\_UPI0015F81933\_27\_309 | A | - | F | H | L | V | - | P | D | L | P | E | T | L | L | A | G | R | E | R | E | Y | V | G | W | F | L | K | - | A | K | A | L | - | S | S | - | D | T | F | D | D | A | E | I | E | H | Y | A | A |
| 093 UniRef90\_A0A0C7N0E5\_9\_278 | P | - | F | H | A | V | - | P | D | L | P | E | L | L | I | S | D | K | E | R | V | Y | L | D | W | F | L | R | - | R | K | S | A | - | D | P | - | T | I | F | T | E | A | D | L | D | E | Y | E | R |
| 094 UniRef90\_UPI00112BEE4D\_14\_300 | A | - | F | H | A | L | - | P | D | L | P | E | A | L | L | A | G | R | E | R | L | Y | L | E | W | F | L | K | - | R | K | A | A | - | D | P | - | G | T | F | S | A | A | D | I | D | E | Y | L | R |
| 095 UniRef90\_A0A401Z9D4\_17\_277 | G | L | F | Q | A | P | - | R | S | L | A | E | S | L | V | E | G | R | E | H | I | L | L | Q | W | F | F | A | - | R | - | A | R | - | N | S | - | A | A | F | T | Q | E | D | I | A | E | Y | V | R |
| 096 UniRef90\_A0A5J6MKN6\_7\_281 | H | - | F | Y | G | - | - | - | E | T | P | L | A | L | V | A | G | R | E | R | I | Y | F | E | H | F | W | N | - | D | F | A | A | - | D | R | T | K | S | V | S | E | A | D | R | Q | L | Y | A | A |
| 097 UniRef90\_A0A4Q7XSL0\_10\_307 | D | - | F | G | G | - | - | - | K | D | A | L | R | L | V | Q | G | R | E | R | I | Y | L | D | R | F | W | N | - | D | F | A | G | - | D | A | - | S | K | I | D | E | A | T | R | Q | H | Y | A | R |
| 098 UniRef90\_UPI0016110D13\_9\_294 | A | - | F | H | A | V | - | P | D | L | P | E | A | L | I | A | G | R | E | R | E | Y | L | G | W | F | F | R | - | E | K | T | V | - | N | P | - | S | C | Y | T | E | E | A | M | A | E | Y | L | R |
| 099 UniRef90\_A0A520GLB7\_17\_313 | H | - | F | H | G | - | - | - | P | T | P | L | A | L | V | K | G | R | E | R | I | Y | F | E | H | F | W | N | - | D | F | A | A | - | D | P | K | H | S | I | P | E | A | D | R | R | L | Y | A | R |
| 100 UniRef90\_A0A252EMP1\_9\_266 | G | - | F | H | Q | E | - | A | D | L | P | E | A | L | L | A | G | R | E | R | E | Y | L | S | F | F | F | K | - | R | Y | A | F | - | D | P | - | R | S | I | T | E | A | D | I | T | E | Y | V | R |
| 101 UniRef90\_UPI001668C4B3\_34\_306 | G | - | F | N | R | L | - | D | G | V | N | E | Q | L | V | R | G | R | E | R | L | F | F | G | H | Q | F | A | - | S | K | A | A | - | R | P | - | D | A | L | P | A | H | A | V | D | L | Y | V | R |
| 102 UniRef90\_UPI00135BC32E\_12\_265 | R | - | F | H | Q | V | - | R | D | L | P | E | R | L | V | S | G | N | E | E | T | Y | L | K | W | F | Y | R | - | E | G | A | Y | - | D | P | - | S | A | I | D | R | E | A | L | Q | E | Y | V | R |
| 103 UniRef90\_A0A2V9DES3\_17\_290 | H | - | F | Y | G | - | - | - | K | T | P | L | A | L | V | H | G | R | E | R | I | Y | F | E | H | F | W | N | - | D | F | A | A | - | D | S | T | H | S | V | P | E | R | D | R | R | I | Y | A | K |
| 104 UniRef90\_A0A6P0DAX4\_34\_314 | R | - | F | H | A | L | - | R | D | V | P | E | M | L | I | A | G | R | E | L | E | Y | L | K | W | F | H | N | - | V | E | G | V | - | N | S | - | R | A | F | D | N | E | A | D | E | I | Y | G | R |
| 105 UniRef90\_A0A1M5KPU7\_13\_307 | H | - | F | Y | G | - | - | - | K | T | P | L | A | L | V | T | G | R | E | R | I | Y | L | D | H | F | W | N | - | D | F | A | A | - | D | P | N | K | S | L | S | E | S | D | R | Q | F | Y | A | K |
| 106 UniRef90\_A0A2I8DLX4\_17\_295 | L | - | F | N | S | V | - | P | D | L | P | E | A | L | L | Q | G | R | E | R | I | L | I | E | W | F | F | S | - | R | K | T | A | - | N | K | P | G | V | F | S | A | A | D | I | D | E | Y | E | R |
| 107 UniRef90\_A0A5S4WMZ6\_36\_313 | G | - | L | H | Q | Q | - | R | D | V | A | E | M | L | V | A | G | R | E | H | A | Y | V | S | D | F | Y | K | - | K | R | L | A | - | A | P | - | - | - | V | A | D | E | D | V | A | V | Y | A | R |
| 108 UniRef90\_UPI0003744601\_9\_294 | G | - | F | N | M | A | - | Q | G | I | N | E | V | L | V | A | G | R | E | R | P | F | I | E | Y | F | Y | R | - | R | G | T | L | - | H | P | - | D | A | L | T | P | A | D | I | D | E | Y | A | R |
| 109 UniRef90\_A0A0H3KYM5\_9\_289 | A | - | F | H | A | V | - | A | D | L | P | E | A | L | L | A | G | R | E | R | I | Y | L | E | W | F | F | R | - | E | K | A | A | - | N | H | - | S | C | Y | G | E | T | E | L | S | E | Y | E | R |
| 110 UniRef90\_UPI001616C592\_11\_307 | N | - | F | N | R | T | - | T | D | I | N | E | R | L | V | E | G | R | E | H | L | Y | F | G | H | Q | F | A | - | T | K | A | A | - | T | P | - | T | A | V | P | A | A | V | V | D | V | Y | V | R |
| 111 UniRef90\_A0A1U9ZZS0\_24\_308 | A | - | F | H | L | V | - | P | D | L | P | E | T | L | L | A | G | R | E | R | D | Y | V | G | W | F | L | R | - | M | K | A | L | - | S | P | - | G | T | F | D | D | A | E | I | D | H | Y | A | A |
| 112 UniRef90\_UPI00146E50DC\_33\_308 | H | - | F | Y | G | - | - | - | T | T | P | L | A | L | V | S | G | R | E | R | L | Y | F | E | H | F | W | N | - | D | F | A | A | - | N | K | T | K | S | L | S | E | A | D | R | R | F | Y | A | K |
| 113 UniRef90\_C3KLY5\_13\_287 | A | - | F | H | N | V | - | P | D | L | P | E | A | L | I | E | G | R | E | R | T | Y | L | N | W | F | L | Q | - | R | K | A | A | - | N | P | - | Q | V | F | S | D | A | D | F | E | E | Y | L | R |
| 114 UniRef90\_A0A2V6STQ7\_27\_303 | H | - | F | H | G | - | - | - | E | T | P | L | A | L | V | K | G | R | E | R | I | Y | F | E | H | F | W | N | - | D | F | A | A | - | D | R | T | R | S | V | P | E | A | D | R | R | I | Y | A | A |
| 115 UniRef90\_UPI0008D5DA8A\_10\_290 | M | - | F | N | Q | L | - | H | D | L | P | E | T | L | I | A | G | K | E | R | A | Y | L | T | Y | M | F | D | - | K | W | S | H | - | R | R | - | D | R | V | A | - | - | - | A | E | V | Y | I | E |
| 116 UniRef90\_A0A3N2H8D2\_16\_299 | A | - | F | H | T | V | - | P | E | L | P | E | T | L | L | A | G | R | E | R | E | Y | V | G | W | F | L | T | - | V | K | A | L | - | S | P | - | S | T | F | D | D | T | E | L | D | H | Y | A | A |
| 117 UniRef90\_A0A379Z3Q1\_16\_292 | M | - | F | N | Q | L | - | A | D | L | P | E | T | L | T | L | G | R | E | R | E | Y | L | S | Y | I | F | N | - | R | W | S | Y | - | R | R | - | D | R | V | A | - | - | - | A | E | T | Y | I | A |
| 118 UniRef90\_A0A4R5QBW7\_8\_277 | G | - | F | H | W | E | - | D | G | L | A | E | A | L | T | A | G | R | E | E | V | Y | L | R | F | F | Y | R | - | T | F | G | G | - | R | P | - | D | C | V | P | E | A | A | Q | R | D | Y | I | R |
| 119 UniRef90\_A0A163VVR8\_16\_291 | M | - | F | N | Q | L | - | A | D | L | P | E | T | L | T | Q | G | R | E | Q | E | Y | L | G | Y | I | F | N | - | R | W | S | Y | - | R | R | - | D | R | V | A | - | - | - | A | Q | V | Y | I | A |
| 120 UniRef90\_G0FSK7\_29\_305 | A | - | F | N | Q | L | - | D | D | L | N | E | Q | L | V | A | G | R | E | D | V | F | L | R | W | Q | F | T | - | H | K | A | A | - | R | P | - | - | - | L | A | E | P | A | I | E | H | Y | V | D |
| 121 UniRef90\_A0A4Q7FLU1\_10\_276 | G | - | F | H | A | T | - | R | D | L | P | E | T | L | I | A | G | R | E | D | V | Y | L | E | W | F | F | R | - | N | F | G | A | - | T | P | - | T | A | V | S | P | D | D | I | A | E | Y | L | R |
| 122 UniRef90\_A0A2E3NGG1\_11\_269 | S | - | F | N | A | E | - | P | D | I | A | I | Q | L | V | T | G | R | E | R | E | F | L | T | H | F | Y | R | - | R | - | C | Y | - | N | P | - | G | A | F | S | L | E | D | I | D | E | Y | V | A |
| 123 UniRef90\_A0A2J9ERN8\_9\_287 | A | - | F | H | A | V | - | P | D | L | P | E | I | L | L | E | G | R | E | R | A | Y | L | E | W | F | F | W | - | T | K | T | A | - | S | P | - | A | C | Y | G | E | E | E | I | A | E | Y | L | R |
| 124 UniRef90\_UPI001677C581\_8\_288 | M | - | F | N | Q | L | - | A | D | L | P | E | T | L | I | T | G | R | E | K | A | Y | L | D | F | M | F | D | - | R | W | S | H | - | R | R | - | D | S | V | A | - | - | - | V | D | V | Y | V | A |
| 125 UniRef90\_UPI0003039C4F\_8\_269 | G | - | F | H | G | E | - | P | D | F | P | E | A | L | V | A | G | R | E | E | L | Y | L | S | F | F | Y | R | - | K | A | G | A | - | R | P | - | D | A | I | S | E | E | A | R | R | E | Y | L | R |
| 126 UniRef90\_A0A1B1YXM9\_17\_276 | A | - | F | H | G | V | - | R | D | L | A | E | M | L | V | A | G | R | E | R | A | Y | I | S | W | F | F | Q | - | N | F | A | Y | - | N | P | - | R | A | V | S | E | A | D | V | D | E | Y | A | R |
| 127 UniRef90\_A0A534ZXC6\_31\_288 | R | - | F | N | G | - | - | - | P | T | P | E | A | L | V | R | G | R | E | R | V | Y | F | E | H | F | W | N | - | D | F | A | A | - | D | P | R | R | S | V | P | E | A | D | R | A | V | Y | A | A |
| 128 UniRef90\_A0A2X1TAZ7\_42\_319 | M | - | F | N | Q | V | - | P | D | L | P | E | M | L | T | A | G | K | E | Q | D | Y | L | N | F | I | F | D | - | H | W | A | Y | - | R | R | - | D | R | V | A | - | - | - | A | Q | T | Y | I | A |
| 129 UniRef90\_A0A4Q5QRH4\_8\_282 | A | - | F | H | A | V | - | P | D | L | P | E | I | L | L | E | G | R | E | R | P | Y | L | E | W | F | F | W | - | N | K | T | V | - | N | P | - | R | V | Y | G | E | A | E | I | N | E | Y | L | R |
| 130 UniRef90\_A0A0D6HQT0\_17\_294 | L | - | F | N | T | V | - | P | D | L | P | E | A | L | L | Q | G | R | E | R | V | L | I | E | W | F | F | S | - | R | K | T | A | - | N | K | P | G | V | F | S | G | A | D | L | D | E | Y | E | R |
| 131 UniRef90\_A0A1G4JCM6\_9\_275 | P | - | F | H | A | V | - | P | D | L | P | E | L | L | I | R | D | K | E | R | T | Y | L | E | W | F | L | R | - | R | K | T | A | - | N | P | - | T | I | F | T | E | A | D | L | D | E | Y | E | R |
| 132 UniRef90\_UPI001269E708\_28\_301 | A | - | F | H | L | V | - | P | D | L | P | E | R | L | L | A | G | R | E | G | E | Y | V | G | W | F | L | K | - | A | K | A | H | - | S | P | - | D | T | F | D | A | A | E | I | D | H | Y | T | T |
| 133 UniRef90\_A0A538SUN7\_3\_303 | N | - | F | R | G | - | - | - | P | D | E | E | R | L | V | A | G | R | E | R | I | Y | L | D | R | F | W | N | - | E | L | S | A | - | D | P | - | K | A | I | D | E | Q | T | R | R | H | Y | A | L |
| 134 UniRef90\_UPI001646B9D9\_15\_288 | L | - | F | N | Q | V | - | R | D | L | P | E | F | L | V | A | G | R | E | E | G | Y | L | N | F | I | F | D | - | R | W | A | V | - | R | R | - | H | A | V | A | - | - | - | S | D | V | Y | I | R |
| 135 UniRef90\_K9DQK6\_17\_307 | N | - | F | G | G | - | - | - | K | E | A | E | R | L | V | A | G | R | E | R | I | F | L | D | R | L | W | N | - | Q | F | A | A | - | D | P | - | A | K | V | D | E | A | T | R | A | Y | Y | T | R |
| 136 UniRef90\_A0A2N9BM66\_25\_297 | A | - | F | N | R | L | - | A | D | I | N | E | R | M | V | A | G | R | E | E | I | Y | F | G | H | T | F | A | - | S | K | T | A | - | A | P | - | G | A | I | P | Q | H | A | I | D | V | Y | V | D |
| 137 UniRef90\_A0A370LBV7\_5\_266 | H | - | F | Y | G | - | - | - | K | T | P | L | A | L | V | D | G | R | E | R | I | Y | F | E | H | F | W | N | - | D | F | A | A | - | N | P | E | K | S | V | S | E | A | D | R | Q | L | Y | A | A |
| 138 UniRef90\_A0A2V9ZM06\_3\_258 | R | - | F | N | G | - | - | - | P | T | P | E | A | L | V | Q | G | R | E | R | T | Y | F | E | Y | F | W | N | - | D | L | A | A | - | D | K | T | R | S | L | P | E | A | D | R | Q | A | Y | T | T |
| 139 UniRef90\_UPI00101F418B\_26\_310 | G | - | F | N | R | L | - | A | D | L | P | E | L | L | V | E | G | R | E | R | A | Y | L | D | W | L | F | R | - | A | K | L | R | - | I | S | - | S | A | I | T | A | A | D | L | D | E | Y | T | R |
| 140 UniRef90\_A0A4V2YYI8\_27\_305 | A | - | F | N | R | L | - | A | D | M | N | E | E | L | V | S | G | R | E | R | L | Y | F | G | H | Q | F | A | - | T | K | A | G | - | H | P | - | - | - | L | P | D | Y | A | V | Q | Y | Y | V | D |
| 141 UniRef90\_UPI001487D2FB\_8\_292 | S | - | F | G | G | - | - | - | P | D | A | E | R | L | V | A | G | R | E | R | I | Y | L | D | R | F | W | N | - | E | F | A | G | - | N | P | - | A | K | V | D | E | A | T | R | V | Y | Y | A | R |
| 142 UniRef90\_B9XAH7\_320\_607 | G | - | F | N | R | L | - | N | D | L | P | E | I | L | V | Q | G | H | E | R | A | F | L | S | W | F | F | T | - | N | K | I | L | - | R | K | - | G | A | I | E | P | A | A | L | D | E | Y | V | R |
| 143 UniRef90\_UPI0013696204\_44\_306 | G | - | F | N | R | L | - | G | E | L | N | E | L | L | V | R | G | R | E | H | A | F | F | S | H | Q | F | A | - | S | K | A | A | - | S | P | - | T | A | L | P | A | H | A | V | Q | V | Y | V | E |
| 144 UniRef90\_W5WIM6\_13\_292 | G | - | F | N | R | L | - | G | S | L | N | E | Q | L | V | Q | G | R | E | R | L | F | F | G | H | Q | F | T | - | T | K | A | A | - | Q | P | - | - | - | L | P | E | H | A | V | E | L | Y | I | E |
| 145 UniRef90\_A0A2X2GG63\_10\_290 | M | - | F | N | Q | L | - | A | D | L | P | E | A | L | T | L | G | R | E | R | E | Y | L | S | Y | I | F | N | - | R | W | S | Y | - | R | R | - | D | R | V | A | - | - | - | A | E | T | Y | I | A |
| 146 UniRef90\_A0A2E2YI39\_6\_274 | Q | - | F | H | L | T | - | P | D | L | P | E | E | L | I | N | G | R | E | K | V | Y | L | S | W | F | Y | R | - | T | F | A | Y | - | K | P | - | G | C | I | T | K | N | D | L | N | E | Y | V | R |
| 147 UniRef90\_A0A2W7G3K6\_24\_312 | G | - | F | N | R | L | - | D | D | L | P | E | L | L | L | E | G | R | E | R | A | F | L | T | W | L | F | R | - | A | K | A | A | - | R | A | - | W | T | V | V | P | A | D | L | D | E | Y | V | R |
| 148 UniRef90\_A0A6B2RWJ8\_28\_298 | A | - | F | H | L | V | - | P | E | L | P | E | T | L | L | T | G | R | E | R | D | Y | V | G | W | F | L | K | - | A | K | A | L | - | S | P | - | D | A | F | D | D | A | E | I | D | H | Y | A | A |
| 149 UniRef90\_A0A537A198\_6\_270 | A | - | F | H | M | T | - | P | E | L | P | E | A | L | V | A | A | R | E | R | E | Y | L | G | W | F | Y | R | - | E | F | C | W | - | Q | R | - | G | A | I | G | H | A | D | I | D | E | Y | L | R |
| 150 UniRef90\_UPI00135F2885\_48\_334 | A | - | F | N | A | V | - | P | D | L | P | E | A | L | T | Q | G | R | E | R | I | F | L | D | I | F | W | S | - | G | F | T | Y | - | N | P | - | R | G | F | D | E | A | D | I | Q | E | F | L | R |

  
  

|  |  |  |  |  |  |  |  |  |  |  |  |  |  |  |  |  |  |  |  |  |  |  |  |  |  |  |  |  |  |  |  |  |  |  |  |  |  |  |  |  |  |  |  |  |  |  |  |  |  |  |
| --- | --- | --- | --- | --- | --- | --- | --- | --- | --- | --- | --- | --- | --- | --- | --- | --- | --- | --- | --- | --- | --- | --- | --- | --- | --- | --- | --- | --- | --- | --- | --- | --- | --- | --- | --- | --- | --- | --- | --- | --- | --- | --- | --- | --- | --- | --- | --- | --- | --- | --- |
| **001 Input\_protein\_seq** | S | Y | A | K | - | P | H | S | L | N | A | S | F | E | Y | Y | R | A | L | N | - | E | S | - | - | V | R | Q | N | A | E | - | L | - | A | - | K | T | - | - | R | L | Q | M | P | T | M | T | L | A |
| 002 UniRef90\_A0A2G0Y5Q3\_5\_314 | S | Y | S | K | - | P | H | S | L | N | A | S | F | E | Y | Y | R | A | L | N | - | K | S | - | - | I | E | Q | N | I | K | - | L | - | S | - | K | T | - | - | K | L | T | M | P | T | M | A | L | S |
| 003 UniRef90\_A0A172YJZ3\_2\_306 | S | Y | A | K | - | P | H | S | L | N | A | A | F | E | Y | Y | R | V | L | N | - | Q | G | - | - | V | Q | D | N | A | E | - | L | - | A | - | Q | T | - | - | R | I | T | I | P | V | L | A | I | G |
| 004 UniRef90\_A0A4Z1C7D3\_9\_314 | S | Y | A | K | - | P | H | T | L | N | A | S | F | E | Y | Y | R | S | L | N | - | E | T | - | - | V | E | R | N | R | P | - | L | - | A | - | K | A | - | - | P | L | T | M | P | I | L | A | I | G |
| 005 UniRef90\_A0A0A3Z1S4\_7\_319 | S | Y | A | K | - | P | H | T | L | T | A | S | F | E | Y | Y | R | A | L | N | - | T | S | - | - | I | E | Q | N | R | K | - | L | - | A | - | Q | T | - | - | K | L | T | L | P | V | M | A | I | G |
| 006 UniRef90\_A0A329J6I4\_9\_315 | S | Y | A | K | - | P | Q | T | L | N | A | S | F | E | Y | Y | R | A | L | N | - | T | S | - | - | I | A | E | N | A | E | - | L | - | S | - | K | T | - | - | S | L | N | M | P | V | L | A | I | G |
| 007 UniRef90\_A0A4V1G750\_8\_314 | S | Y | A | K | - | P | H | S | L | A | A | A | F | G | Y | Y | R | E | L | N | - | Q | N | - | - | V | Q | D | N | R | K | - | L | - | S | - | G | T | - | - | P | L | T | L | P | V | L | A | I | G |
| 008 UniRef90\_T2L220\_17\_309 | S | Y | A | K | - | S | H | S | L | N | A | S | F | E | Y | Y | R | V | L | N | - | Q | G | - | - | V | K | D | N | N | E | - | L | - | A | - | R | T | - | - | K | V | T | I | P | A | L | A | I | G |
| 009 UniRef90\_UPI000C2FE79C\_12\_314 | S | Y | A | K | - | P | H | T | L | H | A | A | F | E | Y | Y | R | V | L | N | - | Q | G | - | - | V | I | D | N | K | V | - | L | - | A | - | Q | T | - | - | K | I | S | I | P | M | L | A | I | G |
| 010 UniRef90\_UPI00102FACD2\_13\_314 | A | Y | A | K | - | P | H | S | L | H | A | A | F | A | Y | Y | H | V | L | N | - | Q | G | - | - | V | E | D | N | K | R | - | L | - | S | - | Q | S | - | - | K | I | S | L | P | V | L | A | I | G |
| 011 UniRef90\_UPI0014749FF6\_10\_312 | S | Y | S | K | - | P | H | T | L | H | A | A | F | E | Y | Y | R | A | L | P | - | A | S | - | - | V | Q | Q | N | E | A | L | L | - | T | - | K | G | - | - | K | L | N | M | P | L | L | A | V | S |
| 012 UniRef90\_A0A3M4V4S1\_41\_332 | S | Y | S | K | - | P | Q | T | F | H | H | A | F | E | Y | Y | R | A | L | P | - | Q | S | - | - | I | K | Q | N | E | K | - | L | - | V | - | A | T | - | - | K | L | T | V | P | V | L | A | V | G |
| 013 UniRef90\_A0A3L8C981\_31\_324 | S | Y | S | K | - | P | K | T | F | H | H | A | F | E | Y | Y | R | A | L | P | - | Q | S | - | - | I | A | Q | N | E | K | - | L | - | A | - | T | T | - | - | K | I | T | L | P | V | L | A | V | G |
| 014 UniRef90\_A0A1X0N2L9\_32\_323 | S | Y | S | K | - | P | Q | T | F | H | Y | A | F | E | Y | Y | R | A | L | P | - | Q | T | - | - | I | A | Q | N | E | Q | - | L | - | A | - | K | T | - | - | R | L | K | M | P | V | L | A | V | G |
| 015 UniRef90\_UPI00166CAA4C\_15\_313 | S | Y | G | D | - | P | A | R | M | S | A | S | F | N | Y | F | R | T | F | N | - | Q | D | - | - | V | A | Q | T | A | I | - | Y | - | A | - | K | H | - | - | K | L | N | L | P | V | L | A | I | G |
| 016 UniRef90\_UPI001661A3FF\_47\_334 | Y | L | R | D | - | P | A | H | L | A | A | S | L | A | W | F | R | V | F | P | - | Q | D | - | - | I | R | D | N | A | Q | - | F | - | Q | - | R | T | - | - | P | L | P | M | P | V | L | A | I | G |
| 017 UniRef90\_A0A1H5MZC9\_42\_331 | H | L | K | D | - | P | A | H | L | E | A | S | L | A | W | F | R | T | L | P | - | Q | D | - | - | M | K | N | D | A | V | - | Y | - | Q | - | K | R | - | - | K | L | T | M | P | V | L | A | V | G |
| 018 UniRef90\_UPI000690E8EC\_26\_310 | Y | L | K | D | - | P | A | H | L | R | A | S | F | A | W | F | R | A | F | P | - | Q | D | - | - | I | Q | D | N | A | V | - | Y | - | R | - | Q | T | - | - | K | L | T | M | P | V | L | A | I | G |
| 019 UniRef90\_R4LNI7\_31\_317 | P | L | R | D | - | P | A | H | L | R | A | S | F | E | Y | F | R | A | L | P | - | Q | D | - | - | I | D | D | N | A | G | - | Y | - | A | - | R | T | - | - | E | L | P | M | P | V | L | A | I | G |
| 020 UniRef90\_A0A4R2C838\_67\_350 | A | L | A | Q | - | P | G | H | L | R | A | S | I | D | W | F | A | T | L | P | - | Q | D | - | - | V | K | D | N | A | I | - | Y | - | Q | - | K | T | - | - | K | L | S | M | P | V | L | A | I | G |
| 021 UniRef90\_A0A2P9HD85\_25\_319 | P | Y | S | T | - | R | A | N | L | R | A | A | F | G | Y | Y | Q | S | H | S | - | D | S | - | - | A | R | Q | N | E | A | - | L | L | A | A | G | K | - | - | Q | L | T | I | P | V | Y | S | I | S |
| 022 UniRef90\_UPI000D14D87C\_43\_332 | Y | L | R | D | - | Y | A | H | L | E | A | S | F | A | W | F | R | T | F | P | - | Q | D | - | - | I | A | D | N | A | R | - | F | - | R | - | K | K | - | - | P | L | T | M | P | V | L | A | I | G |
| 023 UniRef90\_A0A1Q4ZL08\_6\_289 | Y | L | Q | D | - | E | A | H | L | R | A | S | F | E | Y | F | R | A | L | P | - | Q | D | - | - | I | A | D | N | A | E | - | Y | - | A | - | K | T | - | - | K | L | A | M | P | V | L | A | L | G |
| 024 UniRef90\_A0A1I2GGE7\_30\_316 | Y | L | K | D | - | D | A | H | L | R | A | S | Y | E | W | F | R | A | F | P | - | K | D | - | - | V | R | D | N | E | T | - | N | - | I | - | K | T | - | - | K | L | P | M | P | V | L | A | I | G |
| 025 UniRef90\_UPI0005580D51\_18\_305 | Y | L | R | D | - | P | A | H | L | K | A | S | F | G | W | F | R | A | F | P | - | A | D | - | - | I | A | D | N | L | T | - | Y | - | A | - | E | V | - | - | K | L | T | M | P | V | L | A | I | G |
| 026 UniRef90\_A0A2W2F1P0\_6\_289 | H | L | R | D | - | E | A | H | L | R | A | S | F | E | C | F | R | A | F | G | - | Q | D | - | - | V | A | D | N | A | G | - | Y | - | R | - | K | S | - | - | K | L | P | M | P | V | L | A | I | G |
| 027 UniRef90\_UPI0010F9F40F\_6\_288 | H | L | R | D | - | E | A | H | L | R | A | S | F | E | C | F | R | A | F | A | - | Q | D | - | - | V | A | D | N | A | G | - | Y | - | R | - | R | S | - | - | K | L | A | M | P | V | L | A | I | G |
| 028 UniRef90\_A0A3E2YQE6\_6\_289 | H | L | R | D | - | E | A | H | L | R | A | S | F | A | Y | F | R | A | F | G | - | E | D | - | - | I | S | D | N | A | A | - | Y | - | R | - | A | T | - | - | P | L | P | M | P | V | L | A | V | G |
| 029 UniRef90\_A0A1A9ACL3\_6\_288 | H | L | R | D | - | E | A | H | L | R | A | S | F | G | Y | F | R | A | F | G | - | Q | D | - | - | V | A | D | N | A | A | - | Y | - | R | - | S | T | - | - | R | L | P | M | P | V | L | A | V | G |
| 030 UniRef90\_UPI00174B5D19\_38\_323 | Y | L | R | Q | - | P | G | R | F | R | A | W | M | Q | M | Y | R | A | I | D | - | T | D | - | - | V | R | Q | T | A | E | - | L | S | A | - | A | G | - | - | P | L | R | M | P | V | L | A | V | G |
| 031 UniRef90\_A0A495JCH5\_50\_333 | Y | L | K | Q | - | P | G | H | T | R | A | S | F | E | W | F | R | T | L | N | - | Q | D | - | - | V | A | D | N | A | V | - | N | - | A | - | K | T | - | - | K | L | T | M | P | V | L | A | L | G |
| 032 UniRef90\_UPI0013D14F7B\_11\_294 | Y | L | S | D | - | P | A | H | L | R | A | N | L | A | W | F | R | S | L | P | - | Q | D | - | - | M | A | D | N | T | E | - | F | - | G | - | K | T | - | - | K | L | A | M | P | V | L | A | I | G |
| 033 UniRef90\_UPI00036EC6E3\_56\_342 | F | L | A | Q | - | P | G | H | L | R | A | S | I | E | W | F | A | T | L | P | - | R | D | - | - | V | K | D | N | A | V | - | Y | - | Q | - | Q | T | - | - | K | L | T | M | P | V | L | A | I | G |
| 034 UniRef90\_A0A1H3P9Q0\_6\_290 | H | L | R | D | - | S | A | H | L | R | A | S | F | A | Y | F | R | A | M | P | - | R | D | - | - | V | A | D | N | A | E | - | Y | - | G | - | A | T | - | - | K | L | P | M | P | V | L | A | L | G |
| 035 UniRef90\_A0A385B2U0\_41\_332 | Y | L | R | E | - | P | G | R | F | K | A | W | M | N | M | Y | Q | A | L | H | - | T | D | - | - | I | A | Q | N | E | K | - | F | R | E | - | A | G | - | - | L | L | Q | M | P | I | L | A | V | G |
| 036 UniRef90\_UPI001430CB85\_25\_306 | V | F | T | R | - | E | G | G | L | R | A | G | L | A | Y | Y | R | A | V | A | - | E | S | - | - | A | R | Q | N | Q | E | - | L | S | A | - | Q | G | - | - | K | L | R | M | P | L | L | A | L | S |
| 037 UniRef90\_A0A109IHW8\_6\_288 | H | L | S | D | - | P | G | H | L | R | A | S | F | D | Y | F | R | A | F | A | - | Q | D | - | - | V | A | D | N | T | A | - | Y | - | R | - | K | N | - | - | P | L | P | M | P | V | L | A | V | G |
| 038 UniRef90\_A0A2T6L0M5\_14\_298 | R | L | R | R | - | P | G | R | V | E | A | W | V | K | M | Y | R | S | L | A | - | V | N | - | - | I | Q | Q | N | K | Q | - | L | Q | A | - | A | G | - | - | P | I | G | M | P | I | L | A | V | G |
| 039 UniRef90\_A0A1C4Z0V4\_6\_288 | H | L | R | D | - | P | A | H | L | R | A | S | F | A | Y | F | R | A | F | G | - | Q | D | - | - | V | A | D | N | A | A | - | Y | - | R | - | K | R | - | - | P | L | P | M | P | V | L | A | V | G |
| 040 UniRef90\_A0A4U3M272\_82\_365 | F | L | A | L | - | P | G | H | L | R | A | S | I | E | W | F | A | T | L | P | - | Q | D | - | - | V | K | D | N | A | I | - | S | - | Q | - | Q | T | - | - | K | L | S | M | P | V | L | A | I | G |
| 041 UniRef90\_UPI00052728DD\_22\_294 | V | F | T | R | - | E | G | G | L | R | A | G | L | A | Y | Y | R | A | A | D | - | E | S | - | - | A | R | Q | N | R | A | - | L | S | A | - | K | G | - | - | K | L | Q | M | P | V | L | A | L | S |
| 042 UniRef90\_A0A6B8MSH7\_52\_336 | P | Y | S | T | - | R | E | K | L | H | A | A | F | G | Y | Y | K | S | H | A | - | K | S | - | - | I | A | Q | N | E | Q | - | L | L | A | E | G | K | - | - | K | L | S | I | P | S | M | A | L | T |
| 043 UniRef90\_I4VSM6\_28\_303 | V | F | T | L | - | P | G | G | L | R | S | G | L | A | Y | Y | R | A | T | P | - | E | S | - | - | A | R | Q | N | R | A | - | F | A | D | - | T | G | - | - | K | L | A | M | P | V | L | A | L | S |
| 044 UniRef90\_A0A1I3BFK1\_40\_314 | V | F | K | K | - | V | G | G | L | R | A | G | L | A | Y | Y | R | D | A | S | - | I | S | - | - | A | Q | Q | N | R | D | - | L | S | A | - | L | G | - | - | K | L | R | T | P | I | L | A | L | G |
| 045 UniRef90\_A0A1Z4J856\_42\_316 | T | Y | S | A | - | P | G | G | M | R | A | G | F | E | Y | Y | R | A | I | P | - | Q | V | - | - | A | K | Q | T | K | E | - | N | - | M | - | K | T | - | - | K | L | R | I | P | V | L | A | L | A |
| 046 UniRef90\_A0A0M4FVH2\_27\_314 | V | F | K | K | - | A | G | G | L | R | A | G | L | A | Y | Y | R | A | A | S | - | L | S | - | - | A | Q | Q | N | R | E | - | L | C | A | - | M | G | - | - | K | L | R | T | P | V | L | A | L | G |
| 047 UniRef90\_UPI001032135D\_25\_299 | V | F | C | S | - | S | G | G | L | R | A | G | L | A | F | Y | R | A | V | T | - | Q | S | - | - | A | Q | Q | N | R | D | - | L | Q | A | - | K | G | - | - | K | L | K | M | P | I | L | A | V | S |
| 048 UniRef90\_D5WL63\_24\_300 | I | L | K | K | - | D | G | G | L | R | A | G | L | A | Y | Y | R | A | A | H | - | V | S | - | - | A | R | Q | N | A | E | - | L | K | Q | - | R | G | - | - | M | L | D | L | P | L | L | T | L | G |
| 049 UniRef90\_A0A1H3E943\_18\_291 | A | Y | Q | Q | - | P | G | N | L | R | G | A | L | S | Y | Y | R | A | V | F | - | D | D | - | - | I | E | Q | N | K | A | - | L | - | A | - | T | Q | - | - | K | V | K | T | P | I | L | A | L | G |
| 050 UniRef90\_A0A346N7R3\_30\_317 | A | Y | A | Q | - | P | G | G | M | R | A | G | F | E | Y | F | R | A | F | E | - | Q | D | - | - | A | K | D | N | A | E | - | L | - | S | - | K | V | - | - | K | L | N | M | P | M | L | V | L | T |
| 051 UniRef90\_A0A537Q000\_40\_318 | A | Y | S | V | - | P | G | A | M | K | A | G | F | A | Y | Y | R | A | I | P | - | Q | T | - | - | V | Q | E | N | Q | E | - | L | - | R | - | R | S | - | - | K | L | A | M | P | V | L | A | I | G |
| 052 UniRef90\_E6V5X5\_24\_320 | A | Y | A | Q | - | P | G | G | M | R | A | G | F | E | Y | F | K | A | F | E | - | Q | D | - | - | A | A | E | F | A | E | - | L | - | G | - | K | T | - | - | P | L | P | M | P | M | L | V | L | S |
| 053 UniRef90\_A0A484PNM3\_14\_291 | C | Y | G | A | - | L | G | A | W | R | A | A | A | S | Y | Y | R | G | L | F | - | E | D | - | - | M | A | Q | N | Q | A | - | L | - | A | - | R | E | - | - | P | L | K | M | P | V | M | A | M | G |
| 054 UniRef90\_UPI0011BDEFB8\_30\_311 | V | L | T | R | - | D | G | G | L | R | A | G | L | A | Y | Y | R | A | A | E | - | L | S | - | - | A | R | Q | N | K | A | - | L | L | A | - | K | G | - | - | K | L | P | M | P | V | L | A | I | S |
| 055 UniRef90\_A0A1H2UZ41\_16\_293 | V | Y | K | Q | - | P | G | N | L | R | G | A | L | A | Y | Y | R | A | V | L | - | E | D | - | - | V | E | Q | N | K | S | - | L | - | A | - | T | T | - | - | K | I | D | T | P | T | L | A | L | G |
| 056 UniRef90\_A0A1M5ZT81\_29\_302 | V | F | R | K | - | Y | G | G | L | R | A | G | L | A | Y | Y | R | C | A | A | - | L | S | - | - | A | Q | Q | N | R | R | - | L | F | A | - | K | G | - | - | R | L | K | P | P | V | L | A | L | S |
| 057 UniRef90\_A0A135GL34\_14\_283 | S | Y | S | G | - | P | G | G | L | R | - | - | L | E | L | Y | R | A | F | Y | - | Q | D | - | - | G | I | D | N | A | E | - | L | - | S | - | K | T | - | - | K | L | T | I | P | V | L | A | L | G |
| 058 UniRef90\_A0A484UEV2\_23\_280 | C | Y | G | T | - | L | G | G | W | R | T | A | A | A | Y | Y | R | A | L | F | - | Q | D | - | - | M | A | Q | N | R | E | - | L | - | A | - | R | E | - | - | K | L | R | M | P | V | M | A | M | G |
| 059 UniRef90\_A0A495NUG6\_51\_333 | F | L | R | A | - | D | A | H | L | R | A | S | F | E | W | F | R | A | F | P | - | R | D | - | - | N | T | Q | V | A | R | - | M | - | G | - | R | R | - | - | P | L | A | M | P | V | L | A | V | G |
| 060 UniRef90\_A0A1M5MA45\_15\_292 | A | Y | A | T | - | H | G | S | T | T | G | S | F | H | W | F | G | S | F | R | - | Q | D | - | - | V | K | D | N | Q | E | - | L | - | T | - | K | K | - | - | K | L | K | M | P | V | L | A | M | G |
| 061 UniRef90\_UPI000EF95C7F\_30\_312 | A | L | R | L | - | P | G | A | L | K | A | S | F | E | Y | Y | R | A | I | D | - | Q | I | - | - | L | E | Q | T | R | T | - | R | - | L | - | Q | K | - | - | K | I | D | I | P | V | L | A | V | A |
| 062 UniRef90\_L9WLS3\_15\_271 | C | Y | S | Q | - | P | G | G | L | R | G | G | F | E | Y | Y | R | A | Y | D | - | D | D | - | - | A | E | N | N | A | V | - | H | - | A | - | E | T | - | - | P | L | E | M | P | V | L | A | L | G |
| 063 UniRef90\_UPI0005D31B04\_21\_288 | R | L | S | A | - | P | G | A | L | R | A | S | F | E | H | Y | R | A | F | D | - | I | D | - | - | A | V | N | N | R | Q | - | N | - | A | - | L | T | - | - | K | L | S | M | P | V | L | A | V | G |
| 064 UniRef90\_UPI001408C682\_10\_298 | A | Y | A | Q | - | P | G | A | M | R | A | G | F | A | Y | F | S | D | F | E | - | R | D | - | - | A | A | D | F | G | P | - | M | - | S | - | K | T | - | - | K | L | T | M | P | V | L | T | L | A |
| 065 UniRef90\_A0A1Y6KKD8\_22\_314 | V | L | T | K | - | A | G | G | L | R | A | G | L | A | Y | Y | R | A | A | E | - | L | S | - | - | A | R | Q | N | R | E | - | L | K | A | - | R | G | - | - | K | L | K | P | P | V | L | A | V | G |
| 066 UniRef90\_A0A0X3RYG7\_65\_349 | F | L | K | E | - | P | G | R | A | T | A | W | M | N | V | Y | R | G | L | R | - | T | D | - | - | V | Q | Q | N | K | E | - | F | L | A | - | Q | G | - | - | K | L | K | M | P | I | L | A | I | G |
| 067 UniRef90\_A0A2T0T762\_22\_302 | A | I | V | A | D | P | R | G | L | R | A | S | F | S | Y | Y | R | A | L | D | - | D | T | - | - | I | A | Q | N | E | Q | - | R | - | R | - | K | T | - | - | R | L | T | L | P | V | L | A | I | G |
| 068 UniRef90\_A0A2V6QCX2\_10\_286 | A | Y | A | Q | - | P | G | A | M | R | A | G | F | E | Y | F | R | N | F | E | - | R | D | - | - | A | E | D | F | A | R | - | M | - | G | - | S | T | - | - | R | L | T | M | P | L | L | V | L | S |
| 069 UniRef90\_D8MTC4\_28\_309 | I | M | M | K | - | S | G | G | L | R | A | G | L | A | Y | Y | R | S | A | A | - | L | S | - | - | A | Q | Q | N | R | E | - | L | Q | A | - | N | G | - | - | K | L | S | M | P | I | L | A | I | S |
| 070 UniRef90\_UPI0012FADC80\_10\_303 | A | Y | A | R | - | P | G | A | T | T | G | S | F | H | W | F | G | A | F | P | - | Q | D | - | - | A | K | D | N | L | L | - | L | - | M | - | K | H | - | - | K | L | P | M | P | L | L | A | M | G |
| 071 UniRef90\_A0A2E5PAM0\_2\_278 | A | Y | S | Q | - | P | G | V | L | R | A | G | F | E | Y | Y | R | A | T | P | - | K | D | - | - | I | S | H | N | N | E | - | N | - | M | - | K | T | - | - | K | L | A | M | P | V | L | A | L | G |
| 072 UniRef90\_T0HVL8\_12\_269 | C | Y | A | S | - | L | G | G | M | R | A | G | F | E | Y | Y | R | A | F | P | - | E | T | - | - | A | C | Q | S | R | E | - | L | - | A | - | R | T | - | - | K | L | T | I | P | V | L | A | Y | G |
| 073 UniRef90\_A0A3N4T2I8\_17\_298 | A | I | T | A | D | P | R | A | L | R | A | S | F | A | Y | Y | R | A | L | D | - | E | T | - | - | I | A | Q | N | E | Q | - | R | - | S | - | K | T | - | - | R | L | T | L | P | V | L | G | V | G |
| 074 UniRef90\_A0A433IAT3\_19\_312 | A | Y | A | Q | - | P | G | G | M | R | A | G | F | E | Y | F | R | N | F | E | - | R | D | - | - | A | K | D | F | A | Q | - | L | - | S | - | A | T | - | - | P | L | S | M | P | V | L | V | L | T |
| 075 UniRef90\_A0A226WR47\_30\_304 | I | F | K | K | - | E | G | G | L | R | A | G | L | A | Y | Y | R | A | A | A | - | L | S | - | - | A | Q | Q | N | R | A | - | L | C | A | - | K | G | - | - | K | L | K | M | P | V | L | A | L | G |
| 076 UniRef90\_UPI000DD77D66\_25\_314 | S | Y | S | Q | - | P | G | A | L | R | A | G | F | E | Y | Y | R | A | F | P | - | Q | D | - | - | V | V | A | N | R | V | - | F | - | A | - | Q | S | - | - | K | L | E | M | P | V | L | A | M | S |
| 077 UniRef90\_A0A2A5QV31\_6\_277 | C | Y | A | Q | - | A | G | G | L | R | G | G | F | E | Y | Y | R | A | Y | D | - | A | D | - | - | A | E | H | N | Q | D | - | H | - | A | - | E | D | - | - | P | L | E | M | P | V | L | A | L | G |
| 078 UniRef90\_A0A127EY60\_8\_296 | A | Y | A | Q | - | P | G | A | M | R | A | G | F | E | V | F | R | A | F | D | - | Q | D | - | - | A | K | D | F | A | E | - | L | - | A | - | K | T | - | - | K | L | K | M | P | M | L | V | L | S |
| 079 UniRef90\_UPI0004952425\_6\_308 | A | Y | A | Q | - | P | G | H | M | K | A | G | M | E | V | F | R | A | F | P | - | K | D | - | - | A | E | D | F | A | G | - | F | - | A | - | R | T | - | - | K | L | T | M | P | M | L | V | L | S |
| 080 UniRef90\_A0A0D0JP71\_56\_330 | A | Y | A | V | - | K | D | G | A | K | S | S | F | K | W | F | G | A | F | P | - | Q | D | - | - | G | K | D | N | L | I | - | L | - | A | - | K | T | - | - | K | L | K | M | P | L | L | A | M | G |
| 081 UniRef90\_A0A6A7LIC2\_1\_257 | S | Y | S | A | - | P | G | G | M | R | A | G | F | E | Y | Y | R | A | F | P | - | M | N | - | - | A | E | Q | N | K | A | - | L | - | S | - | E | T | - | - | K | L | Q | I | P | V | L | A | L | G |
| 082 UniRef90\_UPI00097BCF81\_14\_312 | A | Y | A | Q | - | P | G | G | M | R | A | G | F | E | Y | F | K | A | F | E | - | R | D | - | - | A | A | E | F | A | E | - | L | - | G | - | K | T | - | - | P | L | P | M | P | M | L | V | L | S |
| 083 UniRef90\_UPI001591AA5D\_7\_297 | A | I | V | A | D | P | R | G | L | R | A | S | F | G | Y | Y | R | A | L | D | - | E | T | - | - | I | A | Q | N | E | K | - | R | - | K | - | K | T | - | - | P | L | T | L | P | V | L | A | V | G |
| 084 UniRef90\_A0A4P8YCR5\_18\_300 | V | F | T | T | - | S | G | G | L | R | A | G | L | A | Y | Y | R | A | A | D | - | L | S | - | - | A | R | Q | N | R | E | - | L | Q | Q | - | A | G | - | - | P | L | N | V | P | V | L | A | L | S |
| 085 UniRef90\_A0A4R0GJE6\_14\_295 | A | I | A | A | D | P | R | A | L | R | A | S | F | A | Y | Y | R | A | L | D | - | E | T | - | - | I | A | Q | N | Q | Q | - | R | - | S | - | K | T | - | - | R | L | T | I | P | V | L | A | V | G |
| 086 UniRef90\_A0A502C146\_32\_301 | A | Y | S | Q | - | P | G | G | M | R | A | G | F | E | Y | F | K | A | F | P | - | Q | D | - | - | A | K | D | F | E | E | - | L | - | S | - | K | T | - | - | K | L | T | M | P | M | L | V | L | S |
| 087 UniRef90\_A0A4Y9SAQ9\_19\_291 | A | Y | A | Q | - | P | G | G | I | R | A | G | F | Q | Y | F | K | T | F | P | - | K | D | - | - | A | E | Q | F | A | E | - | L | - | G | - | K | T | - | - | K | L | Q | I | P | V | L | V | L | A |
| 088 UniRef90\_A0A327RPK7\_10\_286 | Q | Y | S | A | - | P | G | A | L | R | A | G | F | E | Y | Y | R | C | L | L | - | E | D | - | - | G | K | H | N | Q | K | - | Y | - | K | - | E | Q | - | - | K | L | D | I | P | V | L | A | Y | G |
| 089 UniRef90\_A0A239MTD5\_1\_269 | F | L | K | E | - | P | G | R | T | T | A | W | M | N | V | Y | R | Q | F | R | - | T | N | - | - | V | Q | Q | N | K | E | - | F | L | A | - | R | G | - | - | K | L | K | M | P | I | L | A | I | G |
| 090 UniRef90\_UPI00098F4FB4\_42\_302 | S | Y | A | Q | - | P | G | A | L | R | A | G | F | E | Y | Y | R | A | F | P | - | K | D | - | - | V | E | T | N | R | A | - | L | - | A | - | A | R | - | - | K | L | T | M | P | V | L | A | M | S |
| 091 UniRef90\_A0A261TYY2\_38\_305 | C | Y | G | G | - | L | G | G | W | R | A | G | A | S | Y | Y | R | A | L | F | - | Q | D | - | - | M | E | Q | N | R | A | - | H | - | A | - | R | A | - | - | R | L | T | M | P | V | L | A | M | G |
| 092 UniRef90\_UPI0015F81933\_27\_309 | S | V | A | A | - | D | G | G | L | R | A | S | L | A | Y | Y | R | D | A | A | - | E | S | - | - | A | Q | K | N | H | E | - | A | L | E | - | R | Q | - | - | R | L | T | V | P | I | L | G | I | S |
| 093 UniRef90\_A0A0C7N0E5\_9\_278 | I | F | L | Q | - | P | G | A | L | R | A | G | L | A | Y | Y | R | A | C | G | - | K | S | - | - | A | Q | Q | N | K | E | - | L | L | K | - | N | G | - | - | K | L | A | P | P | L | L | A | V | S |
| 094 UniRef90\_UPI00112BEE4D\_14\_300 | G | F | A | G | - | M | G | A | L | R | A | G | L | G | Y | Y | R | A | A | G | - | R | S | - | - | A | E | Q | N | R | E | - | R | A | R | - | A | G | - | - | K | L | A | V | P | L | L | A | I | S |
| 095 UniRef90\_A0A401Z9D4\_17\_277 | C | Y | S | G | - | R | D | A | L | R | A | G | F | E | Y | Y | R | S | F | S | - | T | N | - | - | A | Q | Q | F | R | A | - | Y | - | S | - | K | E | - | - | K | L | H | I | P | V | L | A | L | G |
| 096 UniRef90\_A0A5J6MKN6\_7\_281 | A | Y | A | R | - | D | G | G | M | R | A | G | F | E | Y | F | R | N | F | E | - | Q | D | - | - | A | K | D | F | A | A | - | L | - | S | - | V | M | - | - | K | L | T | M | P | F | L | I | L | T |
| 097 UniRef90\_A0A4Q7XSL0\_10\_307 | L | Y | A | R | - | P | G | A | M | K | A | S | F | A | Q | F | Q | S | I | R | - | K | D | - | - | A | E | D | N | A | E | - | F | - | V | - | K | T | - | - | K | L | T | M | P | V | L | A | V | G |
| 098 UniRef90\_UPI0016110D13\_9\_294 | I | F | R | A | - | P | G | G | L | R | A | G | L | S | F | Y | R | S | A | T | - | K | S | - | - | A | E | Q | N | R | S | - | L | L | K | - | E | R | - | - | K | L | T | M | P | V | L | G | L | S |
| 099 UniRef90\_A0A520GLB7\_17\_313 | A | Y | A | Q | - | P | G | G | M | R | A | G | F | A | Y | F | Q | A | F | E | - | Q | D | - | - | A | A | E | F | A | E | - | L | - | G | - | K | T | - | - | P | L | P | M | P | M | L | V | L | S |
| 100 UniRef90\_A0A252EMP1\_9\_266 | C | Y | S | G | - | L | G | G | M | R | A | G | F | N | Y | Y | R | A | F | P | - | E | T | - | - | A | V | Q | S | R | E | - | L | - | A | - | R | T | - | - | K | L | T | I | P | V | L | A | Y | G |
| 101 UniRef90\_UPI001668C4B3\_34\_306 | T | L | A | A | D | P | E | A | L | R | S | S | F | A | C | Y | R | A | L | D | - | A | T | - | - | I | A | Q | N | A | A | - | R | - | A | - | R | R | - | - | R | L | A | L | P | V | L | T | I | A |
| 102 UniRef90\_UPI00135BC32E\_12\_265 | A | Y | S | A | - | P | G | G | L | R | G | G | F | E | Y | Y | R | A | Y | D | - | E | D | - | - | A | E | H | N | K | E | - | H | - | T | - | Q | Q | - | - | A | L | E | M | P | V | L | A | L | G |
| 103 UniRef90\_A0A2V9DES3\_17\_290 | A | Y | A | R | - | P | G | G | M | R | S | G | F | E | Y | F | R | A | F | E | - | K | D | - | - | A | K | D | F | A | R | - | F | - | A | - | Q | T | - | - | P | L | R | M | P | M | L | V | L | T |
| 104 UniRef90\_A0A6P0DAX4\_34\_314 | S | Y | A | Q | - | P | G | A | L | R | A | G | F | E | Y | Y | R | A | F | P | - | E | D | - | - | A | V | T | N | R | A | - | F | - | A | - | E | S | - | - | K | L | E | M | P | V | L | A | M | S |
| 105 UniRef90\_A0A1M5KPU7\_13\_307 | A | Y | A | Q | - | P | G | H | M | A | A | G | M | E | V | F | R | A | F | P | - | K | D | - | - | A | E | D | F | A | Q | - | F | - | A | - | K | T | - | - | Q | L | T | M | P | V | L | V | L | S |
| 106 UniRef90\_A0A2I8DLX4\_17\_295 | V | Y | Q | T | - | L | G | G | L | R | G | M | L | G | Y | Y | R | A | A | H | - | Q | D | - | - | A | L | Q | N | R | E | - | L | - | R | - | D | A | - | - | P | L | T | V | P | V | L | A | L | G |
| 107 UniRef90\_A0A5S4WMZ6\_36\_313 | A | Y | T | A | - | P | G | G | L | R | A | G | F | E | L | Y | R | A | F | P | - | E | D | - | - | E | Q | R | F | K | T | - | F | - | M | - | S | R | - | - | K | L | S | M | P | V | L | A | L | A |
| 108 UniRef90\_UPI0003744601\_9\_294 | S | Y | A | A | - | - | G | G | L | V | A | S | F | N | Y | Y | R | T | L | L | - | D | D | - | - | A | K | V | N | R | D | - | K | L | A | - | V | K | - | - | R | L | A | L | P | V | L | S | L | A |
| 109 UniRef90\_A0A0H3KYM5\_9\_289 | L | L | K | T | - | P | G | G | L | R | A | G | L | A | F | Y | R | A | L | S | - | Q | S | - | - | A | A | Q | N | A | E | - | L | V | R | - | Q | G | - | - | R | L | V | M | P | V | L | G | L | S |
| 110 UniRef90\_UPI001616C592\_11\_307 | A | L | R | I | - | P | G | A | L | R | A | S | F | E | F | Y | R | A | I | D | - | E | I | - | - | V | E | Q | S | A | H | - | R | - | K | - | K | T | - | - | P | V | E | I | P | I | L | A | I | S |
| 111 UniRef90\_A0A1U9ZZS0\_24\_308 | A | I | A | A | - | D | G | G | L | R | A | S | L | A | Y | Y | R | D | A | A | - | E | S | - | - | A | R | R | N | H | E | - | A | L | K | - | R | Q | - | - | H | L | T | V | P | V | L | G | I | S |
| 112 UniRef90\_UPI00146E50DC\_33\_308 | E | Y | A | K | - | P | G | H | M | K | A | G | M | E | Y | F | R | N | F | E | - | Q | D | - | - | A | K | E | F | A | V | - | F | - | A | - | K | T | - | - | P | L | P | M | P | M | L | V | L | A |
| 113 UniRef90\_C3KLY5\_13\_287 | V | F | L | Y | - | - | S | G | L | K | G | G | L | A | Y | Y | R | A | V | S | - | L | S | - | - | A | Q | Q | N | R | E | - | L | S | A | - | K | G | - | - | K | L | E | M | P | V | L | A | V | R |
| 114 UniRef90\_A0A2V6STQ7\_27\_303 | A | Y | A | R | - | A | D | G | M | R | A | G | F | E | Y | F | K | A | F | E | - | Q | D | - | - | A | K | D | F | A | A | - | F | - | A | - | A | T | - | - | R | L | D | M | P | F | L | V | L | T |
| 115 UniRef90\_UPI0008D5DA8A\_10\_290 | A | Y | S | A | - | P | G | G | L | R | G | G | F | S | Y | Y | R | A | I | P | - | E | T | - | - | I | R | Q | N | A | L | - | R | - | A | - | A | R | - | - | R | L | E | M | P | T | L | A | I | G |
| 116 UniRef90\_A0A3N2H8D2\_16\_299 | A | V | A | A | - | D | G | G | L | R | A | S | L | A | Y | Y | R | D | A | A | - | E | S | - | - | A | R | R | N | R | E | - | A | L | Q | - | Q | R | - | - | R | L | T | V | P | V | L | G | I | S |
| 117 UniRef90\_A0A379Z3Q1\_16\_292 | A | Y | S | A | - | P | G | S | L | R | A | G | F | D | Y | Y | R | A | I | P | - | E | T | - | - | I | R | Q | N | Q | R | - | R | - | A | - | E | T | - | - | R | L | T | M | P | V | M | T | I | G |
| 118 UniRef90\_A0A4R5QBW7\_8\_277 | A | Y | S | Q | - | P | G | A | M | H | A | G | F | E | Y | Y | R | A | M | R | - | Q | D | - | - | V | A | D | N | E | A | - | M | L | A | R | D | G | - | - | T | L | R | M | P | V | L | C | Y | G |
| 119 UniRef90\_A0A163VVR8\_16\_291 | A | Y | S | A | - | P | G | G | L | R | A | G | F | D | Y | Y | R | A | I | P | - | E | T | - | - | V | R | Q | N | R | L | - | R | - | A | - | A | T | - | - | P | L | T | M | P | V | L | T | V | G |
| 120 UniRef90\_G0FSK7\_29\_305 | S | I | R | R | D | P | R | A | L | H | A | S | F | G | C | Y | R | A | I | G | - | D | T | - | - | V | A | Q | N | A | E | - | R | - | V | - | K | N | - | - | R | L | D | L | P | I | L | T | I | A |
| 121 UniRef90\_A0A4Q7FLU1\_10\_276 | T | Y | R | M | - | P | G | A | L | R | A | G | F | E | Y | Y | R | A | L | E | - | R | D | - | - | T | I | D | N | R | S | - | - | - | - | - | N | P | - | - | R | L | E | M | P | V | L | A | L | G |
| 122 UniRef90\_A0A2E3NGG1\_11\_269 | A | F | S | A | - | P | G | A | M | R | G | S | M | A | H | Y | G | A | L | P | - | L | D | - | - | L | K | H | N | Q | E | - | N | - | A | - | E | T | - | - | K | L | E | M | P | V | L | A | L | G |
| 123 UniRef90\_A0A2J9ERN8\_9\_287 | I | Y | G | A | - | P | G | G | M | R | A | G | L | A | F | Y | R | S | A | A | - | L | S | - | - | A | E | Q | N | R | A | - | L | A | Q | - | K | G | - | - | E | L | T | M | P | V | L | G | L | S |
| 124 UniRef90\_UPI001677C581\_8\_288 | A | Y | A | A | - | P | G | G | L | R | G | G | F | A | Y | Y | R | A | I | P | - | E | T | - | - | I | R | Q | N | R | E | - | R | - | A | - | K | R | - | - | K | L | A | M | P | V | L | A | I | G |
| 125 UniRef90\_UPI0003039C4F\_8\_269 | T | Y | S | Q | - | P | G | A | M | R | A | G | F | N | Y | Y | R | A | L | P | - | Q | D | - | - | I | A | D | N | Q | Q | - | F | I | A | - | E | G | - | - | K | L | E | M | P | V | L | V | Y | G |
| 126 UniRef90\_A0A1B1YXM9\_17\_276 | C | L | T | Q | - | P | G | A | L | R | A | G | F | E | Y | Y | R | A | A | G | - | Q | D | - | - | A | V | D | F | A | A | - | A | - | A | - | R | E | - | - | P | L | S | C | P | V | L | A | L | G |
| 127 UniRef90\_A0A534ZXC6\_31\_288 | A | Y | A | R | - | P | G | R | M | R | A | G | W | A | Y | F | V | S | F | Q | - | Q | T | - | - | A | K | D | F | A | E | - | L | - | S | - | K | T | - | - | K | L | T | M | P | V | L | A | I | G |
| 128 UniRef90\_A0A2X1TAZ7\_42\_319 | A | Y | S | S | - | P | G | G | L | R | A | G | F | A | Y | Y | R | A | I | P | - | Q | T | - | - | I | L | Q | N | K | Q | - | R | - | A | - | E | K | - | - | K | L | T | M | P | V | L | A | I | G |
| 129 UniRef90\_A0A4Q5QRH4\_8\_282 | I | Y | S | A | - | P | G | G | M | R | A | G | L | A | F | Y | R | A | I | D | - | R | S | - | - | A | E | Q | N | R | T | - | L | T | A | - | K | G | - | - | K | L | S | M | P | V | L | G | L | S |
| 130 UniRef90\_A0A0D6HQT0\_17\_294 | V | Y | R | T | - | L | G | G | L | R | G | M | L | G | Y | Y | R | A | A | H | - | Q | S | - | - | A | E | Q | N | R | A | - | L | - | R | - | E | T | - | - | P | L | T | L | P | V | L | A | L | G |
| 131 UniRef90\_A0A1G4JCM6\_9\_275 | I | F | T | Q | - | P | G | S | L | R | A | G | L | A | Y | Y | R | A | C | A | - | K | S | - | - | A | E | Q | N | R | A | - | L | V | K | - | N | D | - | - | P | L | N | M | D | V | L | A | V | S |
| 132 UniRef90\_UPI001269E708\_28\_301 | A | V | A | A | - | G | S | G | L | R | A | F | L | A | Y | Y | R | D | A | A | - | E | S | - | - | A | R | R | N | H | E | - | A | L | E | - | R | Q | - | - | R | L | T | V | P | V | L | G | I | S |
| 133 UniRef90\_A0A538SUN7\_3\_303 | L | Y | A | R | - | P | H | A | I | H | D | A | F | N | Q | F | G | A | F | S | - | Q | D | - | - | A | I | D | N | K | A | L | L | - | A | - | K | G | - | - | K | L | T | M | P | V | L | A | I | G |
| 134 UniRef90\_UPI001646B9D9\_15\_288 | A | Y | S | S | - | P | G | G | M | R | G | G | L | A | Y | Y | R | A | I | P | - | E | T | - | - | I | R | Q | N | R | E | - | R | - | A | - | R | R | - | - | P | L | T | M | P | V | L | A | I | G |
| 135 UniRef90\_K9DQK6\_17\_307 | L | Y | A | Q | - | P | G | A | M | R | A | S | F | A | Q | F | N | T | I | A | T | H | D | - | - | V | A | D | N | R | A | - | A | - | S | - | K | V | - | - | K | L | T | M | P | V | L | A | V | G |
| 136 UniRef90\_A0A2N9BM66\_25\_297 | S | L | R | D | - | P | A | A | L | R | A | S | F | E | Y | Y | R | T | L | D | - | T | S | - | - | A | E | H | V | L | R | W | R | - | D | - | E | G | - | - | P | L | T | I | P | V | L | A | I | G |
| 137 UniRef90\_A0A370LBV7\_5\_266 | A | Y | G | Q | - | P | G | A | M | R | A | G | F | E | V | F | R | N | F | E | - | Q | D | - | - | A | K | D | F | A | G | - | F | - | A | - | A | T | - | - | K | L | K | V | P | M | L | V | L | T |
| 138 UniRef90\_A0A2V9ZM06\_3\_258 | A | Y | S | R | - | P | G | R | M | R | A | G | W | A | Y | F | V | S | F | Q | - | Q | A | - | - | A | K | D | F | A | Q | - | L | - | S | - | Q | T | - | - | K | L | T | M | P | V | L | V | I | G |
| 139 UniRef90\_UPI00101F418B\_26\_310 | V | F | S | A | - | P | G | A | A | R | A | G | F | A | Y | Y | R | A | L | F | - | N | G | D | G | L | E | Q | N | R | R | - | R | - | A | - | T | R | - | - | R | L | S | I | P | V | M | A | W | G |
| 140 UniRef90\_A0A4V2YYI8\_27\_305 | I | L | A | T | V | P | E | A | L | R | A | S | F | E | S | Y | R | A | L | D | - | T | S | - | - | I | A | Q | N | E | Q | - | R | - | K | - | A | R | - | - | R | L | I | L | P | I | L | A | I | A |
| 141 UniRef90\_UPI001487D2FB\_8\_292 | L | Y | A | R | - | P | G | A | M | R | S | A | F | A | Q | F | R | S | F | P | - | Q | D | - | - | A | E | D | N | R | K | A | L | - | A | - | G | K | - | - | K | L | R | M | P | V | L | A | I | G |
| 142 UniRef90\_B9XAH7\_320\_607 | L | F | S | N | - | P | D | S | A | R | A | G | F | T | Y | Y | R | D | F | F | - | D | E | A | G | Q | A | Q | M | K | D | - | G | - | A | - | A | R | - | - | R | L | A | M | P | V | L | A | L | G |
| 143 UniRef90\_UPI0013696204\_44\_306 | A | L | A | A | G | P | E | A | L | R | C | S | F | E | F | Y | R | A | L | D | - | T | T | - | - | M | E | Q | N | S | R | - | R | - | K | - | E | R | - | - | R | L | T | L | P | V | L | T | L | A |
| 144 UniRef90\_W5WIM6\_13\_292 | T | L | A | S | D | R | E | A | L | R | A | S | F | E | F | Y | R | A | I | D | - | T | T | - | - | I | E | Q | N | T | L | - | R | - | R | - | Q | Q | - | - | K | L | T | M | P | V | L | T | I | A |
| 145 UniRef90\_A0A2X2GG63\_10\_290 | A | Y | S | A | - | P | G | S | L | R | A | G | F | D | Y | Y | R | A | I | P | - | E | T | - | - | I | R | Q | N | Q | R | - | R | - | A | - | E | T | - | - | P | L | G | M | P | V | M | T | I | G |
| 146 UniRef90\_A0A2E2YI39\_6\_274 | T | Y | K | Q | - | P | G | A | L | R | A | G | F | S | Y | Y | R | T | M | V | - | Q | D | - | - | A | K | V | N | A | E | - | I | I | R | - | K | F | - | - | K | X | P | M | P | V | L | A | I | G |
| 147 UniRef90\_A0A2W7G3K6\_24\_312 | V | N | E | A | - | P | G | A | T | R | A | A | L | S | Y | Y | R | H | A | F | - | S | T | A | G | L | E | Q | N | R | T | - | R | - | G | - | T | H | - | - | K | L | A | M | P | V | L | A | Y | G |
| 148 UniRef90\_A0A6B2RWJ8\_28\_298 | A | L | A | A | - | E | G | G | L | R | A | S | L | A | Y | Y | R | D | A | A | - | E | S | - | - | A | R | S | N | H | E | - | A | L | E | - | R | G | - | - | H | L | S | I | P | V | L | G | I | S |
| 149 UniRef90\_A0A537A198\_6\_270 | S | Y | T | Q | - | P | G | A | M | R | A | G | F | E | Y | Y | R | S | L | P | - | R | T | - | - | K | A | D | N | R | A | - | L | - | A | - | D | S | G | F | R | L | S | M | P | V | L | A | L | G |
| 150 UniRef90\_UPI00135F2885\_48\_334 | S | Y | T | A | - | V | G | A | M | H | A | G | F | N | Y | Y | R | A | F | D | - | R | D | - | - | A | A | A | N | R | A | W | F | E | A | - | G | N | - | - | K | L | D | M | P | V | L | W | L | G |

  
  

|  |  |  |  |  |  |  |  |  |  |  |  |  |  |  |  |  |  |  |  |  |  |  |  |  |  |  |  |  |  |  |  |  |  |  |  |  |  |  |  |  |  |  |  |  |  |  |  |  |  |  |
| --- | --- | --- | --- | --- | --- | --- | --- | --- | --- | --- | --- | --- | --- | --- | --- | --- | --- | --- | --- | --- | --- | --- | --- | --- | --- | --- | --- | --- | --- | --- | --- | --- | --- | --- | --- | --- | --- | --- | --- | --- | --- | --- | --- | --- | --- | --- | --- | --- | --- | --- |
| **001 Input\_protein\_seq** | G | G | - | - | - | - | - | G | H | G | G | - | - | - | - | - | - | - | - | - | - | - | - | M | G | T | F | Q | L | E | Q | M | K | - | - | - | - | A | Y | A | E | D | V | E | G | H | - | V | L | P |
| 002 UniRef90\_A0A2G0Y5Q3\_5\_314 | G | G | - | - | - | - | - | G | H | G | G | - | - | - | - | - | - | - | - | - | - | - | - | M | G | Q | F | Q | I | D | Q | M | K | - | - | - | - | E | Y | A | N | D | V | E | G | H | - | I | L | P |
| 003 UniRef90\_A0A172YJZ3\_2\_306 | G | G | - | - | - | - | - | G | H | G | G | - | - | - | - | - | - | - | - | - | - | - | - | M | G | Q | F | Q | V | D | Q | M | Q | - | - | - | - | R | Y | A | T | D | V | T | G | L | - | V | M | P |
| 004 UniRef90\_A0A4Z1C7D3\_9\_314 | G | G | - | - | - | - | - | G | H | G | G | - | - | - | - | - | - | - | - | - | - | - | - | M | G | Q | F | Q | V | D | Q | L | R | - | - | - | - | Q | Y | G | T | D | V | E | G | L | - | V | I | P |
| 005 UniRef90\_A0A0A3Z1S4\_7\_319 | G | G | - | - | - | - | - | G | H | G | G | - | - | - | - | - | - | - | - | - | - | - | - | M | G | Q | F | Q | V | D | Q | M | K | - | - | - | - | D | Y | A | T | K | V | E | G | H | - | V | L | A |
| 006 UniRef90\_A0A329J6I4\_9\_315 | G | G | - | - | - | - | - | G | R | N | G | - | - | - | - | - | - | - | - | - | - | - | - | L | G | Q | F | Q | I | D | Q | T | K | - | - | - | - | K | Y | A | T | N | V | K | G | E | - | I | L | P |
| 007 UniRef90\_A0A4V1G750\_8\_314 | G | G | - | - | - | - | - | G | H | G | G | - | - | - | - | - | - | - | - | - | - | - | - | M | G | Q | F | Q | V | D | Q | L | R | - | - | - | - | R | Y | A | S | N | V | T | G | K | - | V | L | P |
| 008 UniRef90\_T2L220\_17\_309 | G | G | - | - | - | - | - | D | H | G | G | - | - | - | - | - | - | - | - | - | - | - | - | M | G | Q | Y | Q | I | D | M | M | R | - | - | - | - | H | Y | A | S | D | V | T | G | I | - | V | L | H |
| 009 UniRef90\_UPI000C2FE79C\_12\_314 | G | G | - | - | - | - | - | G | N | G | G | - | - | - | - | - | - | - | - | - | - | - | - | M | G | E | Y | E | S | Q | V | V | S | - | - | - | - | H | Y | A | D | N | V | T | A | K | - | V | L | P |
| 010 UniRef90\_UPI00102FACD2\_13\_314 | G | G | - | - | - | - | - | G | H | G | G | - | - | - | - | - | - | - | - | - | - | - | - | M | G | E | L | Q | V | N | Q | I | R | - | - | - | - | H | F | A | T | N | V | T | G | K | - | V | L | P |
| 011 UniRef90\_UPI0014749FF6\_10\_312 | G | S | - | - | - | - | - | G | R | G | G | - | - | - | - | - | - | - | - | - | - | - | - | L | G | Q | T | Q | I | D | Q | M | N | - | - | - | - | E | Y | A | T | N | V | Q | G | H | - | V | L | N |
| 012 UniRef90\_A0A3M4V4S1\_41\_332 | G | G | - | - | - | - | - | G | N | G | G | - | - | - | - | - | - | - | - | - | - | - | - | F | G | A | Q | Q | P | E | N | I | R | - | - | - | - | R | Y | A | T | N | V | E | S | H | - | M | L | P |
| 013 UniRef90\_A0A3L8C981\_31\_324 | G | G | - | - | - | - | - | G | N | G | G | - | - | - | - | - | - | - | - | - | - | - | - | F | G | A | Q | Q | P | E | N | I | H | - | - | - | - | R | Y | A | T | N | V | E | A | H | - | V | L | Q |
| 014 UniRef90\_A0A1X0N2L9\_32\_323 | G | G | - | - | - | - | - | G | N | G | G | - | - | - | - | - | - | - | - | - | - | - | - | F | G | A | M | Q | P | E | N | I | R | - | - | - | - | R | Y | A | T | H | V | E | A | H | - | V | L | S |
| 015 UniRef90\_UPI00166CAA4C\_15\_313 | A | A | - | - | - | - | - | - | - | G | S | - | - | - | - | - | - | - | - | - | - | - | - | L | G | E | H | V | P | E | Q | V | R | - | - | - | - | Q | Y | A | T | N | V | E | G | H | - | V | V | D |
| 016 UniRef90\_UPI001661A3FF\_47\_334 | A | S | - | - | - | - | - | - | - | G | S | - | - | - | - | - | - | - | - | - | - | - | - | L | G | S | A | E | G | E | Q | M | K | - | - | - | - | A | Y | A | S | H | V | T | G | V | - | T | I | P |
| 017 UniRef90\_A0A1H5MZC9\_42\_331 | A | E | - | - | - | - | - | - | - | G | S | - | - | - | - | - | - | - | - | - | - | - | - | L | G | D | S | V | P | E | Q | V | K | - | - | - | - | K | Y | A | T | H | V | T | G | V | - | V | V | P |
| 018 UniRef90\_UPI000690E8EC\_26\_310 | A | D | - | - | - | - | - | - | - | G | S | - | - | - | - | - | - | - | - | - | - | - | - | L | G | A | Q | V | V | R | Q | V | C | - | - | - | - | R | Y | A | T | N | V | T | G | V | - | V | V | P |
| 019 UniRef90\_R4LNI7\_31\_317 | A | A | - | - | - | - | - | - | - | G | S | - | - | - | - | - | - | - | - | - | - | - | - | L | G | R | T | V | P | D | Q | V | E | - | - | - | - | R | Y | A | T | H | V | T | G | A | - | V | V | P |
| 020 UniRef90\_A0A4R2C838\_67\_350 | A | S | - | - | - | - | - | - | - | G | N | - | - | - | - | - | - | - | - | - | - | - | - | L | G | T | R | E | A | T | W | V | R | - | - | - | - | K | Y | A | T | N | V | T | G | L | - | V | I | P |
| 021 UniRef90\_A0A2P9HD85\_25\_319 | G | A | - | - | - | - | - | - | - | K | G | - | - | - | - | - | - | - | - | - | - | - | - | V | N | D | V | L | P | K | Q | L | A | A | R | F | V | K | D | P | S | K | L | G | S | T | - | I | L | P |
| 022 UniRef90\_UPI000D14D87C\_43\_332 | A | S | - | - | - | - | - | - | - | G | S | - | - | - | - | - | - | - | - | - | - | - | - | L | G | S | S | V | A | K | Q | V | R | - | - | - | - | H | Y | A | A | H | V | T | P | A | - | V | I | P |
| 023 UniRef90\_A0A1Q4ZL08\_6\_289 | A | R | - | - | - | - | - | - | - | A | S | - | - | - | - | - | - | - | - | - | - | - | - | L | G | D | A | V | S | Q | Q | A | A | - | - | - | - | R | Y | A | S | N | V | S | G | G | - | V | V | E |
| 024 UniRef90\_A0A1I2GGE7\_30\_316 | A | D | - | - | - | - | - | - | - | G | S | - | - | - | - | - | - | - | - | - | - | - | - | L | G | G | N | I | E | G | Q | V | R | - | - | - | - | Q | Y | A | V | N | V | K | G | A | - | V | I | A |
| 025 UniRef90\_UPI0005580D51\_18\_305 | A | D | - | - | - | - | - | - | - | G | S | - | - | - | - | - | - | - | - | - | - | - | - | L | R | D | T | E | E | T | Q | V | R | - | - | - | - | Q | Y | A | T | N | V | T | G | A | - | V | I | A |
| 026 UniRef90\_A0A2W2F1P0\_6\_289 | A | Q | - | - | - | - | - | - | - | A | S | - | - | - | - | - | - | - | - | - | - | - | - | L | G | G | Q | V | A | E | Q | V | R | - | - | - | - | R | Y | A | D | T | V | T | G | Q | - | V | V | E |
| 027 UniRef90\_UPI0010F9F40F\_6\_288 | A | Q | - | - | - | - | - | - | - | A | S | - | - | - | - | - | - | - | - | - | - | - | - | L | G | G | Q | V | A | E | Q | V | R | - | - | - | - | R | Y | A | D | S | V | T | G | E | - | V | V | E |
| 028 UniRef90\_A0A3E2YQE6\_6\_289 | G | R | - | - | - | - | - | - | - | A | S | - | - | - | - | - | - | - | - | - | - | - | - | L | G | G | Q | V | A | D | Q | V | R | - | - | - | - | R | Y | A | T | T | V | R | G | E | - | V | I | E |
| 029 UniRef90\_A0A1A9ACL3\_6\_288 | A | Q | - | - | - | - | - | - | - | A | S | - | - | - | - | - | - | - | - | - | - | - | - | L | G | P | Q | V | A | D | Q | V | R | - | - | - | - | R | Y | A | G | T | V | T | G | E | - | V | V | D |
| 030 UniRef90\_UPI00174B5D19\_38\_323 | G | E | - | - | - | - | - | - | - | K | A | - | - | - | - | - | - | - | - | - | - | - | - | L | G | G | S | V | G | A | Q | W | S | - | - | - | - | K | Y | A | T | N | V | D | T | R | - | V | L | A |
| 031 UniRef90\_A0A495JCH5\_50\_333 | A | Q | - | - | - | - | - | - | - | Y | S | - | - | - | - | - | - | - | - | - | - | - | - | L | G | G | S | V | P | D | Q | V | R | - | - | - | - | K | Y | A | T | N | V | T | G | D | - | V | V | Q |
| 032 UniRef90\_UPI0013D14F7B\_11\_294 | A | E | - | - | - | - | - | - | - | G | S | - | - | - | - | - | - | - | - | - | - | - | - | L | A | D | F | V | G | I | Q | V | K | - | - | - | - | N | Y | A | S | D | V | T | P | A | - | V | I | A |
| 033 UniRef90\_UPI00036EC6E3\_56\_342 | A | S | - | - | - | - | - | - | - | A | N | - | - | - | - | - | - | - | - | - | - | - | - | L | G | S | R | E | A | T | W | V | S | - | - | - | - | Q | Y | A | T | N | V | T | G | V | - | V | I | P |
| 034 UniRef90\_A0A1H3P9Q0\_6\_290 | G | R | - | - | - | - | - | - | - | A | S | - | - | - | - | - | - | - | - | - | - | - | - | L | G | D | R | V | A | G | Q | A | A | - | - | - | - | Q | Y | A | T | T | V | T | G | G | - | V | I | E |
| 035 UniRef90\_A0A385B2U0\_41\_332 | G | E | - | - | - | - | - | - | - | E | A | - | - | - | - | - | - | - | - | - | - | - | - | L | S | S | A | V | G | T | Q | W | Q | - | - | - | - | G | Y | A | A | N | V | E | T | Q | - | V | M | T |
| 036 UniRef90\_UPI001430CB85\_25\_306 | A | D | - | - | - | - | - | - | - | Q | G | - | - | - | - | - | - | - | - | - | - | - | - | S | I | P | D | M | A | G | P | L | R | - | - | - | - | A | Y | A | E | D | V | R | G | V | - | T | I | S |
| 037 UniRef90\_A0A109IHW8\_6\_288 | A | A | - | - | - | - | - | - | - | A | S | - | - | - | - | - | - | - | - | - | - | - | - | L | G | G | Q | V | A | E | Q | V | R | - | - | - | - | R | Y | A | R | T | V | D | G | R | - | V | V | A |
| 038 UniRef90\_A0A2T6L0M5\_14\_298 | G | E | - | - | - | - | - | - | - | K | S | - | - | - | - | - | - | - | - | - | - | - | - | L | G | K | S | I | G | T | Q | L | E | - | - | - | - | R | Y | A | T | Q | V | D | T | R | - | V | L | A |
| 039 UniRef90\_A0A1C4Z0V4\_6\_288 | A | A | - | - | - | - | - | - | - | A | S | - | - | - | - | - | - | - | - | - | - | - | - | L | G | G | Q | V | A | D | Q | V | R | - | - | - | - | R | Y | A | R | T | V | E | G | R | - | V | V | E |
| 040 UniRef90\_A0A4U3M272\_82\_365 | A | S | - | - | - | - | - | - | - | G | N | - | - | - | - | - | - | - | - | - | - | - | - | L | G | T | R | E | A | T | W | V | R | - | - | - | - | Q | Y | A | T | N | V | T | G | L | - | V | I | P |
| 041 UniRef90\_UPI00052728DD\_22\_294 | A | D | - | - | - | - | - | - | - | Q | G | - | - | - | - | - | - | - | - | - | - | - | - | S | I | P | N | M | A | A | P | L | E | - | - | - | - | A | C | A | T | N | V | E | G | V | - | T | I | G |
| 042 UniRef90\_A0A6B8MSH7\_52\_336 | G | E | - | - | - | - | - | - | - | K | G | - | - | - | - | - | - | - | - | - | - | - | - | V | N | D | V | L | V | K | E | M | R | S | R | F | V | Q | D | P | Q | N | F | K | G | V | - | I | L | P |
| 043 UniRef90\_I4VSM6\_28\_303 | A | D | - | - | - | - | - | - | - | Q | G | - | - | - | - | - | - | - | - | - | - | - | - | S | I | P | D | M | A | A | P | L | R | - | - | - | - | A | F | A | E | D | V | R | G | G | - | R | I | A |
| 044 UniRef90\_A0A1I3BFK1\_40\_314 | A | D | - | - | - | - | - | - | - | Q | G | - | - | - | - | - | - | - | - | - | - | - | - | S | I | V | D | M | V | T | P | L | R | - | - | - | - | A | F | A | Q | D | V | Q | G | S | - | N | I | R |
| 045 UniRef90\_A0A1Z4J856\_42\_316 | G | E | - | - | - | - | - | - | - | H | S | - | - | - | - | - | L | G | - | - | N | P | A | L | G | D | P | A | R | T | S | I | Q | - | - | - | - | L | L | A | E | N | V | R | Y | S | - | A | I | E |
| 046 UniRef90\_A0A0M4FVH2\_27\_314 | A | D | - | - | - | - | - | - | - | Q | G | - | - | - | - | - | - | - | - | - | - | - | - | S | I | A | D | M | V | T | P | L | K | - | - | - | - | A | F | A | E | D | V | Q | G | G | - | T | I | S |
| 047 UniRef90\_UPI001032135D\_25\_299 | A | G | - | - | - | - | - | - | - | Q | G | - | - | - | - | - | - | - | - | - | - | - | - | S | I | A | D | M | A | A | P | L | R | - | - | - | - | A | F | A | D | E | V | T | G | V | - | V | V | P |
| 048 UniRef90\_D5WL63\_24\_300 | S | D | - | - | - | - | - | - | - | Q | G | - | - | - | - | - | - | - | - | - | - | - | - | S | I | A | D | M | A | T | P | L | R | - | - | - | - | A | F | F | T | D | V | Q | G | R | - | T | I | S |
| 049 UniRef90\_A0A1H3E943\_18\_291 | G | A | - | - | - | - | - | - | - | Q | G | - | - | - | - | - | - | - | - | - | - | - | - | M | S | P | D | L | F | E | A | M | K | - | - | - | - | P | L | A | E | N | L | S | G | G | - | V | I | D |
| 050 UniRef90\_A0A346N7R3\_30\_317 | G | E | - | - | - | - | - | - | - | R | A | - | - | - | - | - | - | - | - | - | - | - | - | S | G | T | F | L | I | D | Q | G | K | - | - | - | - | L | V | D | S | N | V | Q | G | I | - | V | I | K |
| 051 UniRef90\_A0A537Q000\_40\_318 | G | V | - | - | - | - | - | - | - | I | G | - | - | - | - | - | - | - | - | - | - | - | - | A | G | K | T | T | I | E | T | M | K | - | - | - | - | L | V | S | D | H | V | E | G | A | - | I | V | E |
| 052 UniRef90\_E6V5X5\_24\_320 | G | E | - | - | - | - | - | - | - | K | A | - | - | - | - | - | - | - | - | - | - | - | - | G | G | T | F | L | I | E | Q | G | K | - | - | - | - | M | V | A | T | Q | V | Q | G | V | - | I | V | K |
| 053 UniRef90\_A0A484PNM3\_14\_291 | G | G | - | - | - | - | - | - | - | A | A | - | - | - | - | - | - | - | - | - | - | - | - | L | G | A | M | M | G | R | M | M | A | - | - | - | - | E | V | A | N | D | V | T | G | - | - | V | V | A |
| 054 UniRef90\_UPI0011BDEFB8\_30\_311 | A | D | - | - | - | - | - | - | - | H | G | - | - | - | - | - | - | - | - | - | - | - | - | S | I | A | D | M | A | A | P | L | R | - | - | - | - | P | F | A | E | N | V | S | G | I | - | R | V | P |
| 055 UniRef90\_A0A1H2UZ41\_16\_293 | G | D | - | - | - | - | - | - | - | V | G | - | - | - | - | - | - | - | - | - | - | - | - | M | S | P | D | I | F | E | A | M | K | - | - | - | - | P | L | T | S | H | I | E | G | G | - | I | V | R |
| 056 UniRef90\_A0A1M5ZT81\_29\_302 | A | D | - | - | - | - | - | - | - | Q | G | - | - | - | - | - | - | - | - | - | - | - | - | S | I | P | D | M | A | G | P | L | R | - | - | - | - | E | Y | A | D | D | V | R | G | A | - | T | I | A |
| 057 UniRef90\_A0A135GL34\_14\_283 | G | A | - | - | - | - | - | - | - | N | S | - | - | - | - | - | - | - | - | - | - | - | - | I | R | D | L | C | V | S | S | M | E | - | - | - | - | A | V | A | E | N | V | H | G | V | - | V | V | P |
| 058 UniRef90\_A0A484UEV2\_23\_280 | G | G | - | - | - | - | - | - | - | G | A | - | - | - | - | - | - | - | - | - | - | - | - | L | G | A | L | M | G | R | M | L | G | - | - | - | - | A | V | A | D | D | V | T | N | - | - | V | V | A |
| 059 UniRef90\_A0A495NUG6\_51\_333 | A | D | - | - | - | - | - | - | - | H | S | - | - | - | - | - | - | - | - | - | - | - | - | L | A | S | S | V | G | T | Q | A | R | - | - | - | - | S | Y | A | R | D | V | T | T | R | - | V | V | K |
| 060 UniRef90\_A0A1M5MA45\_15\_292 | G | E | - | - | - | - | - | - | - | F | S | - | - | - | - | - | - | - | - | - | - | - | - | C | A | P | F | L | A | D | H | A | R | - | - | - | - | L | V | A | E | N | V | E | E | V | - | R | I | A |
| 061 UniRef90\_UPI000EF95C7F\_30\_312 | G | D | - | - | - | - | - | - | - | A | L | - | - | - | - | - | - | - | - | - | - | - | - | G | G | E | N | M | E | K | E | V | R | - | - | - | - | T | I | A | S | N | V | T | G | A | V | V | I | P |
| 062 UniRef90\_L9WLS3\_15\_271 | G | A | - | - | - | - | - | - | - | A | S | - | - | - | - | - | - | - | - | - | - | - | - | F | R | S | L | P | I | E | D | M | N | - | - | - | - | A | V | A | T | D | V | E | G | E | - | V | L | E |
| 063 UniRef90\_UPI0005D31B04\_21\_288 | G | E | - | - | - | - | - | - | - | F | C | - | - | - | - | - | - | - | - | - | - | - | - | M | N | V | Q | M | G | H | I | M | R | - | - | - | - | P | L | A | N | D | V | Q | S | V | - | V | I | K |
| 064 UniRef90\_UPI001408C682\_10\_298 | G | E | - | - | - | - | - | - | - | Y | S | - | - | - | - | - | - | - | - | - | - | - | - | A | G | E | F | L | G | K | Q | I | G | - | - | - | - | S | V | A | T | N | V | Q | S | T | - | I | I | K |
| 065 UniRef90\_A0A1Y6KKD8\_22\_314 | A | D | - | - | - | - | - | - | - | Q | G | - | - | - | - | - | - | - | - | - | - | - | - | S | I | V | D | M | A | A | P | L | R | - | - | - | - | E | V | A | D | H | V | E | G | L | - | T | I | A |
| 066 UniRef90\_A0A0X3RYG7\_65\_349 | G | Q | - | - | - | - | - | - | - | Y | S | - | - | - | - | - | - | - | - | - | - | - | - | F | G | S | G | V | P | N | Q | W | H | - | - | - | - | E | Y | A | E | D | V | Q | G | R | - | V | L | A |
| 067 UniRef90\_A0A2T0T762\_22\_302 | G | A | - | - | - | - | - | - | - | R | Y | - | - | - | - | - | - | - | - | - | - | - | - | S | G | A | M | V | A | E | T | M | R | - | - | - | - | L | A | A | D | D | V | T | E | V | - | V | I | D |
| 068 UniRef90\_A0A2V6QCX2\_10\_286 | G | E | - | - | - | - | - | - | - | K | A | - | - | - | - | - | - | - | - | - | - | - | - | G | G | A | F | L | I | E | Q | A | K | - | - | - | - | L | V | A | S | D | V | R | G | T | - | V | V | T |
| 069 UniRef90\_D8MTC4\_28\_309 | A | E | - | - | - | - | - | - | - | Q | G | - | - | - | - | - | - | - | - | - | - | - | - | S | I | P | D | M | A | A | P | L | R | - | - | - | - | P | F | A | A | D | V | T | G | V | - | V | V | P |
| 070 UniRef90\_UPI0012FADC80\_10\_303 | G | Q | - | - | - | - | - | - | - | H | Y | - | - | - | - | - | - | - | - | - | - | - | - | A | A | A | F | L | A | D | H | S | R | - | - | - | - | L | V | A | T | N | V | T | E | A | - | T | I | P |
| 071 UniRef90\_A0A2E5PAM0\_2\_278 | G | S | - | - | - | - | - | - | - | G | G | - | - | - | - | - | K | G | - | - | - | - | - | R | G | L | Q | V | M | E | N | M | K | - | - | - | - | E | L | A | T | K | V | K | G | G | - | E | I | A |
| 072 UniRef90\_T0HVL8\_12\_269 | G | S | - | - | - | - | - | - | - | H | C | - | - | - | - | - | - | - | - | - | - | - | - | M | G | E | I | P | L | R | S | M | Q | - | - | - | - | R | V | A | Q | H | V | E | G | G | - | I | I | P |
| 073 UniRef90\_A0A3N4T2I8\_17\_298 | G | A | - | - | - | - | - | - | - | L | W | - | - | - | - | - | - | - | - | - | - | - | - | S | G | A | N | A | A | Q | T | M | R | - | - | - | - | L | A | A | D | D | V | T | G | V | - | V | L | D |
| 074 UniRef90\_A0A433IAT3\_19\_312 | G | E | - | - | - | - | - | - | - | K | A | - | - | - | - | - | - | - | - | - | - | - | - | S | G | N | F | L | I | E | Q | T | K | - | - | - | - | L | V | A | S | N | V | Q | G | Q | - | V | V | M |
| 075 UniRef90\_A0A226WR47\_30\_304 | S | D | - | - | - | - | - | - | - | Q | G | - | - | - | - | - | - | - | - | - | - | - | - | S | I | P | D | M | A | A | P | L | R | - | - | - | - | A | F | A | E | D | V | H | G | S | - | T | I | A |
| 076 UniRef90\_UPI000DD77D66\_25\_314 | G | I | - | - | - | - | - | - | - | G | G | - | - | - | - | - | - | - | - | - | - | - | - | L | G | T | V | Y | G | E | H | I | R | - | - | - | - | H | I | A | K | N | V | R | A | L | - | V | V | E |
| 077 UniRef90\_A0A2A5QV31\_6\_277 | G | A | - | - | - | - | - | - | - | A | S | - | - | - | - | - | - | - | - | - | - | - | - | F | R | E | L | P | I | R | D | M | E | - | - | - | - | A | V | A | T | D | V | E | S | E | - | V | V | E |
| 078 UniRef90\_A0A127EY60\_8\_296 | G | E | - | - | - | - | - | - | - | K | A | - | - | - | - | - | - | - | - | - | - | - | - | G | G | Q | F | L | I | D | Q | G | K | - | - | - | - | M | V | A | D | N | V | E | G | V | - | I | V | K |
| 079 UniRef90\_UPI0004952425\_6\_308 | G | E | - | - | - | - | - | - | - | K | A | - | - | - | - | - | - | - | - | - | - | - | - | G | G | P | F | L | I | E | Q | G | K | - | - | - | - | M | V | A | T | N | V | E | G | V | - | L | V | K |
| 080 UniRef90\_A0A0D0JP71\_56\_330 | G | E | - | - | - | - | - | - | - | Y | F | - | - | - | - | - | - | - | - | - | - | - | - | A | A | A | F | L | K | E | H | S | K | - | - | - | - | L | V | A | E | N | V | T | E | S | - | K | I | A |
| 081 UniRef90\_A0A6A7LIC2\_1\_257 | G | D | I | Y | P | A | F | G | G | D | A | - | - | - | - | - | - | - | - | - | - | - | - | P | G | N | F | A | L | D | S | T | Q | - | - | - | - | G | L | A | E | N | V | K | G | V | - | I | V | P |
| 082 UniRef90\_UPI00097BCF81\_14\_312 | G | E | - | - | - | - | - | - | - | K | A | - | - | - | - | - | - | - | - | - | - | - | - | G | G | S | F | L | I | D | Q | G | K | - | - | - | - | M | V | A | T | N | V | K | G | V | - | I | V | V |
| 083 UniRef90\_UPI001591AA5D\_7\_297 | G | A | - | - | - | - | - | - | - | R | Y | - | - | - | - | - | - | - | - | - | - | - | - | C | G | P | Q | V | A | E | T | M | R | - | - | - | - | P | A | A | G | D | V | T | E | V | - | V | L | D |
| 084 UniRef90\_A0A4P8YCR5\_18\_300 | S | D | - | - | - | - | - | - | - | Q | G | - | - | - | - | - | - | - | - | - | - | - | - | S | I | P | D | M | A | A | P | L | R | - | - | - | - | P | F | V | T | R | I | E | G | Q | - | T | I | A |
| 085 UniRef90\_A0A4R0GJE6\_14\_295 | G | A | - | - | - | - | - | - | - | L | W | - | - | - | - | - | - | - | - | - | - | - | - | S | G | P | N | A | A | Q | T | M | R | - | - | - | - | L | A | A | D | D | V | T | G | V | - | V | L | D |
| 086 UniRef90\_A0A502C146\_32\_301 | G | E | - | - | - | - | - | - | - | K | A | - | - | - | - | - | - | - | - | - | - | - | - | G | G | T | F | L | V | D | Q | A | R | - | - | - | - | L | V | A | T | Q | V | S | G | V | - | I | I | K |
| 087 UniRef90\_A0A4Y9SAQ9\_19\_291 | G | E | - | - | - | - | - | - | - | K | S | - | - | - | - | - | - | - | - | - | - | - | - | G | G | E | F | L | I | N | Q | V | K | - | - | - | - | L | V | A | N | N | V | D | G | K | - | V | I | K |
| 088 UniRef90\_A0A327RPK7\_10\_286 | G | E | - | - | - | - | - | - | - | T | S | - | - | - | - | - | - | - | - | - | - | - | - | T | G | D | N | F | R | K | S | L | S | - | - | - | - | L | I | A | N | H | V | E | G | G | - | S | I | P |
| 089 UniRef90\_A0A239MTD5\_1\_269 | A | Q | - | - | - | - | - | - | - | D | S | - | - | - | - | - | - | - | - | - | - | - | - | F | G | G | I | V | V | D | Q | W | R | - | - | - | - | D | Y | A | V | N | V | D | G | R | - | V | L | K |
| 090 UniRef90\_UPI00098F4FB4\_42\_302 | G | A | - | - | - | - | - | - | - | G | G | - | - | - | - | - | - | - | - | - | - | - | - | M | G | S | M | Y | E | G | H | I | R | - | - | - | - | N | V | A | K | N | V | R | G | V | - | V | V | E |
| 091 UniRef90\_A0A261TYY2\_38\_305 | G | E | - | - | - | - | - | - | - | A | A | - | - | - | - | - | - | - | - | - | - | - | - | L | G | G | M | M | E | T | M | L | S | - | - | - | - | R | V | A | D | D | V | T | G | - | - | V | I | A |
| 092 UniRef90\_UPI0015F81933\_27\_309 | S | S | - | - | - | - | - | - | - | H | G | - | - | - | - | - | - | - | - | - | - | - | - | S | I | P | D | M | A | A | S | I | R | - | - | - | - | P | W | A | D | H | A | T | G | T | - | V | V | P |
| 093 UniRef90\_A0A0C7N0E5\_9\_278 | S | D | - | - | - | - | - | - | - | Q | G | - | - | - | - | - | - | - | - | - | - | - | - | S | I | P | N | M | A | K | S | L | E | - | - | - | - | A | F | S | S | E | V | V | G | A | - | T | I | E |
| 094 UniRef90\_UPI00112BEE4D\_14\_300 | A | D | - | - | - | - | - | - | - | Q | G | - | - | - | - | - | - | - | - | - | - | - | - | S | I | R | D | M | A | A | P | L | R | - | - | - | - | A | C | F | E | H | V | E | A | A | - | T | I | A |
| 095 UniRef90\_A0A401Z9D4\_17\_277 | G | E | - | - | - | - | - | - | - | Y | S | - | - | - | - | - | - | - | - | - | - | - | - | G | A | G | W | P | F | Y | S | F | A | - | - | - | - | Q | L | A | D | N | V | S | G | G | - | I | I | P |
| 096 UniRef90\_A0A5J6MKN6\_7\_281 | G | E | - | - | - | - | - | - | - | K | A | - | - | - | - | - | - | - | - | - | - | - | - | S | G | T | F | L | I | D | Q | A | K | - | - | - | - | I | V | A | S | N | V | T | G | V | - | I | V | K |
| 097 UniRef90\_A0A4Q7XSL0\_10\_307 | G | E | - | - | - | - | - | - | - | K | S | - | - | - | - | - | - | - | - | - | - | - | - | F | G | A | N | E | A | A | V | M | R | - | - | - | - | N | A | A | T | N | V | T | E | V | - | V | V | P |
| 098 UniRef90\_UPI0016110D13\_9\_294 | A | D | - | - | - | - | - | - | - | Q | G | - | - | - | - | - | - | - | - | - | - | - | - | S | I | P | D | I | A | A | A | V | H | - | - | - | - | P | Y | A | T | D | V | S | G | D | - | V | I | K |
| 099 UniRef90\_A0A520GLB7\_17\_313 | G | E | - | - | - | - | - | - | - | K | A | - | - | - | - | - | - | - | - | - | - | - | - | G | G | A | F | L | I | E | Q | G | K | - | - | - | - | M | V | A | T | N | V | Q | G | V | - | I | V | K |
| 100 UniRef90\_A0A252EMP1\_9\_266 | G | S | - | - | - | - | - | - | - | H | C | - | - | - | - | - | - | - | - | - | - | - | - | M | S | E | I | P | L | R | S | M | K | - | - | - | - | L | V | A | N | N | V | Q | G | G | - | V | I | P |
| 101 UniRef90\_UPI001668C4B3\_34\_306 | G | A | - | - | - | - | - | - | - | Q | C | - | - | - | - | - | - | - | - | - | - | - | - | S | G | E | L | V | G | D | T | M | R | - | - | - | - | L | A | A | E | D | V | E | S | V | - | I | L | P |
| 102 UniRef90\_UPI00135BC32E\_12\_265 | G | E | - | - | - | - | - | - | - | A | S | - | - | - | - | - | - | - | - | - | - | - | - | F | G | S | M | P | V | E | D | M | E | - | - | - | - | A | V | A | T | D | V | D | G | E | - | V | V | E |
| 103 UniRef90\_A0A2V9DES3\_17\_290 | G | E | - | - | - | - | - | - | - | K | A | - | - | - | - | - | - | - | - | - | - | - | - | S | G | D | F | L | I | Q | Q | G | R | - | - | - | - | L | V | A | T | N | V | E | G | V | - | V | V | R |
| 104 UniRef90\_A0A6P0DAX4\_34\_314 | G | I | - | - | - | - | - | - | - | G | G | - | - | - | - | - | - | - | - | - | - | - | - | L | G | S | I | Y | G | G | H | I | R | - | - | - | - | H | V | A | N | N | V | R | A | V | - | V | I | D |
| 105 UniRef90\_A0A1M5KPU7\_13\_307 | G | E | - | - | - | - | - | - | - | K | A | - | - | - | - | - | - | - | - | - | - | - | - | G | G | P | F | L | I | E | Q | G | K | - | - | - | - | M | V | A | T | N | V | E | G | I | - | L | V | Q |
| 106 UniRef90\_A0A2I8DLX4\_17\_295 | G | D | - | - | - | - | - | - | - | R | G | - | - | - | - | - | - | - | - | - | - | - | - | S | A | P | N | L | Y | E | A | L | K | - | - | - | - | P | L | A | R | D | L | Q | G | G | - | V | L | S |
| 107 UniRef90\_A0A5S4WMZ6\_36\_313 | G | E | - | - | - | - | - | - | - | K | S | - | - | - | - | - | - | - | - | - | - | - | - | N | G | W | T | E | T | A | M | A | K | - | - | - | - | E | V | A | Q | D | V | H | G | G | - | V | A | P |
| 108 UniRef90\_UPI0003744601\_9\_294 | A | E | - | - | - | - | - | - | - | Q | G | - | - | - | - | - | - | - | - | - | - | - | - | F | G | D | F | S | H | A | S | I | A | - | - | - | - | Q | V | A | D | L | V | E | R | Q | - | T | I | A |
| 109 UniRef90\_A0A0H3KYM5\_9\_289 | A | D | - | - | - | - | - | - | - | Q | G | - | - | - | - | - | - | - | - | - | - | - | - | S | I | A | D | M | A | A | T | L | K | - | - | - | - | A | Y | G | D | D | I | H | G | Q | - | H | I | A |
| 110 UniRef90\_UPI001616C592\_11\_307 | G | S | - | - | - | - | - | - | - | A | G | - | - | - | - | - | - | - | - | - | - | - | - | - | G | L | D | V | A | A | E | M | R | - | - | - | - | V | G | A | T | D | V | T | G | V | - | V | L | - |
| 111 UniRef90\_A0A1U9ZZS0\_24\_308 | S | S | - | - | - | - | - | - | - | H | G | - | - | - | - | - | - | - | - | - | - | - | - | S | I | P | D | M | A | A | S | L | S | - | - | - | - | P | W | A | D | H | V | T | G | V | - | V | V | P |
| 112 UniRef90\_UPI00146E50DC\_33\_308 | G | E | - | - | - | - | - | - | - | K | A | - | - | - | - | - | - | - | - | - | - | - | - | S | G | E | S | L | V | N | Q | G | K | - | - | - | - | L | V | A | T | N | V | E | G | M | - | V | V | K |
| 113 UniRef90\_C3KLY5\_13\_287 | A | D | - | - | - | - | - | - | - | Q | G | - | - | - | - | - | - | - | - | - | - | - | - | S | M | R | D | L | V | A | Q | L | Q | - | - | - | - | K | I | A | T | N | V | R | G | T | - | A | I | A |
| 114 UniRef90\_A0A2V6STQ7\_27\_303 | G | E | - | - | - | - | - | - | - | K | A | - | - | - | - | - | - | - | - | - | - | - | - | S | G | T | F | L | I | E | Q | V | K | - | - | - | - | M | V | A | S | H | V | S | G | T | - | V | V | K |
| 115 UniRef90\_UPI0008D5DA8A\_10\_290 | A | E | - | - | - | - | - | - | - | H | A | - | - | - | - | - | - | - | - | - | - | - | - | T | R | D | A | P | L | I | T | M | R | - | - | - | - | E | N | A | S | D | L | R | G | A | - | I | I | P |
| 116 UniRef90\_A0A3N2H8D2\_16\_299 | S | S | - | - | - | - | - | - | - | H | G | - | - | - | - | - | - | - | - | - | - | - | - | S | I | P | D | M | A | A | S | L | S | - | - | - | - | P | C | A | D | N | T | T | G | I | - | V | V | P |
| 117 UniRef90\_A0A379Z3Q1\_16\_292 | A | E | - | - | - | - | - | - | - | H | A | - | - | - | - | - | - | - | - | - | - | - | - | T | G | E | A | P | F | V | T | L | R | - | - | - | - | D | N | A | V | D | L | R | G | E | - | T | V | A |
| 118 UniRef90\_A0A4R5QBW7\_8\_277 | G | P | - | - | - | - | - | - | - | L | G | - | - | - | - | - | R | G | - | - | - | - | - | R | G | L | G | A | I | E | S | W | R | - | - | - | - | R | V | A | E | D | V | R | G | G | - | I | A | E |
| 119 UniRef90\_A0A163VVR8\_16\_291 | G | E | - | - | - | - | - | - | - | H | A | - | - | - | - | - | - | - | - | - | - | - | - | T | G | D | A | P | L | T | T | L | R | - | - | - | - | G | N | A | H | D | L | R | G | E | - | T | V | A |
| 120 UniRef90\_G0FSK7\_29\_305 | G | E | - | - | - | - | - | - | - | R | S | - | - | - | - | - | - | - | - | - | - | - | - | T | G | P | L | V | E | R | T | M | I | - | - | - | - | P | A | A | A | D | V | R | G | V | - | V | L | P |
| 121 UniRef90\_A0A4Q7FLU1\_10\_276 | G | A | - | - | - | - | - | - | - | C | S | - | - | - | - | - | W | G | - | - | - | - | - | R | G | L | E | V | V | E | S | L | R | - | - | - | - | R | V | A | R | D | V | R | G | G | - | V | V | E |
| 122 UniRef90\_A0A2E3NGG1\_11\_269 | G | A | - | - | - | - | - | - | - | M | S | - | - | - | - | - | - | - | - | - | - | - | - | F | G | G | G | V | K | K | S | L | E | - | - | - | - | A | V | A | N | D | V | R | G | G | - | V | I | Q |
| 123 UniRef90\_A0A2J9ERN8\_9\_287 | A | D | - | - | - | - | - | - | - | Q | G | - | - | - | - | - | - | - | - | - | - | - | - | S | I | P | D | M | S | L | L | L | R | - | - | - | - | A | F | A | A | D | V | V | G | E | - | T | I | T |
| 124 UniRef90\_UPI001677C581\_8\_288 | A | E | - | - | - | - | - | - | - | H | A | - | - | - | - | - | - | - | - | - | - | - | - | T | N | D | A | P | L | V | T | M | R | - | - | - | - | D | N | A | T | D | L | R | G | V | - | V | V | P |
| 125 UniRef90\_UPI0003039C4F\_8\_269 | G | G | - | - | - | - | - | - | - | A | P | - | - | - | - | G | I | G | - | - | - | - | - | R | G | M | A | A | M | E | S | W | Q | - | - | - | - | R | V | A | A | N | V | R | G | G | - | V | A | E |
| 126 UniRef90\_A0A1B1YXM9\_17\_276 | G | E | - | - | - | - | - | - | - | R | S | - | - | - | - | - | - | - | - | - | - | - | - | I | G | A | A | V | K | L | C | M | Q | - | - | - | - | Q | V | A | G | D | V | R | G | G | - | V | M | P |
| 127 UniRef90\_A0A534ZXC6\_31\_288 | G | A | - | - | - | - | - | - | - | K | A | - | - | - | - | - | - | - | - | - | - | - | - | N | G | E | L | L | G | Q | Q | M | K | - | - | - | - | L | V | A | S | D | A | T | T | V | - | V | L | P |
| 128 UniRef90\_A0A2X1TAZ7\_42\_319 | A | D | - | - | - | - | - | - | - | H | A | - | - | - | - | - | - | - | - | - | - | - | - | T | R | D | A | P | Q | L | T | M | Q | - | - | - | - | G | R | A | A | D | L | Q | G | A | - | M | L | S |
| 129 UniRef90\_A0A4Q5QRH4\_8\_282 | A | D | - | - | - | - | - | - | - | Q | G | - | - | - | - | - | - | - | - | - | - | - | - | S | I | R | D | M | A | A | A | I | R | - | - | - | - | P | F | G | A | D | V | R | G | E | - | T | I | K |
| 130 UniRef90\_A0A0D6HQT0\_17\_294 | G | E | - | - | - | - | - | - | - | H | G | - | - | - | - | - | - | - | - | - | - | - | - | S | A | P | D | L | Y | E | A | L | G | - | - | - | - | P | L | A | R | D | L | R | G | G | - | V | L | A |
| 131 UniRef90\_A0A1G4JCM6\_9\_275 | A | D | - | - | - | - | - | - | - | Q | G | - | - | - | - | - | - | - | - | - | - | - | - | S | I | P | N | M | A | K | P | L | E | - | - | - | - | V | F | A | P | K | V | E | G | V | - | T | I | N |
| 132 UniRef90\_UPI001269E708\_28\_301 | S | S | - | - | - | - | - | - | - | H | G | - | - | - | - | - | - | - | - | - | - | - | - | S | I | P | D | M | A | A | S | L | S | - | - | - | - | P | W | A | E | Q | V | T | G | V | - | V | V | P |
| 133 UniRef90\_A0A538SUN7\_3\_303 | A | E | - | - | - | - | - | - | - | K | S | - | - | - | - | - | - | - | - | - | - | - | - | F | G | T | A | M | A | D | D | I | R | - | - | - | - | F | V | A | T | N | V | Q | M | G | - | V | V | P |
| 134 UniRef90\_UPI001646B9D9\_15\_288 | A | A | - | - | - | - | - | - | - | Q | A | - | - | - | - | - | - | - | - | - | - | - | - | T | G | D | A | P | L | R | T | L | Q | - | - | - | - | G | H | A | R | S | V | R | G | V | - | I | V | P |
| 135 UniRef90\_K9DQK6\_17\_307 | G | E | - | - | - | - | - | - | - | K | S | - | - | - | - | - | - | - | - | - | - | - | - | F | G | P | M | M | A | T | V | M | R | - | - | - | - | N | A | A | L | D | V | R | Q | A | - | V | V | P |
| 136 UniRef90\_A0A2N9BM66\_25\_297 | G | Q | - | - | - | - | - | - | - | Y | S | - | - | - | - | - | - | - | - | - | - | - | - | T | G | T | M | P | E | E | T | M | R | - | - | - | - | L | V | A | P | D | V | T | G | L | - | V | I | P |
| 137 UniRef90\_A0A370LBV7\_5\_266 | G | E | - | - | - | - | - | - | - | K | A | - | - | - | - | - | - | - | - | - | - | - | - | S | G | E | F | L | I | A | Q | G | R | - | - | - | - | L | V | A | E | S | V | E | G | V | - | V | V | K |
| 138 UniRef90\_A0A2V9ZM06\_3\_258 | G | E | - | - | - | - | - | - | - | K | S | - | - | - | - | - | - | - | - | - | - | - | - | L | G | D | A | L | A | Q | Q | M | K | - | - | - | - | S | V | A | S | N | V | T | V | V | - | V | L | K |
| 139 UniRef90\_UPI00101F418B\_26\_310 | A | S | - | - | - | - | - | - | - | D | G | - | - | - | - | - | - | - | - | - | - | - | - | V | G | N | I | L | L | D | T | I | R | - | - | - | - | Y | I | A | D | D | V | R | G | G | - | T | I | E |
| 140 UniRef90\_A0A4V2YYI8\_27\_305 | G | A | - | - | - | - | - | - | - | K | S | - | - | - | - | - | - | - | - | - | - | - | - | A | G | E | S | V | S | D | T | M | R | - | - | - | - | L | A | A | D | D | V | E | S | V | - | V | L | T |
| 141 UniRef90\_UPI001487D2FB\_8\_292 | G | A | - | - | - | - | - | - | - | K | S | - | - | - | - | - | - | - | - | - | - | - | - | F | G | A | N | E | A | M | V | M | R | - | - | - | - | H | A | A | T | D | V | T | E | V | - | V | I | P |
| 142 UniRef90\_B9XAH7\_320\_607 | G | E | - | - | - | - | - | - | - | G | G | - | - | - | - | - | - | - | - | - | - | - | - | V | G | S | A | L | L | K | A | V | Q | - | - | - | - | P | L | G | D | N | V | H | G | G | - | V | L | V |
| 143 UniRef90\_UPI0013696204\_44\_306 | G | A | - | - | - | - | - | - | - | E | C | - | - | - | - | - | - | - | - | - | - | - | - | S | G | E | L | V | G | N | T | M | R | - | - | - | - | L | A | A | D | H | V | E | S | R | - | I | L | P |
| 144 UniRef90\_W5WIM6\_13\_292 | G | A | - | - | - | - | - | - | - | R | S | - | - | - | - | - | - | - | - | - | - | - | - | V | G | E | L | V | G | A | T | M | A | - | - | - | - | P | A | A | V | D | V | T | S | V | - | V | L | P |
| 145 UniRef90\_A0A2X2GG63\_10\_290 | A | E | - | - | - | - | - | - | - | H | A | - | - | - | - | - | - | - | - | - | - | - | - | T | G | D | A | P | F | V | T | L | R | - | - | - | - | D | N | A | V | N | L | R | G | E | - | T | V | A |
| 146 UniRef90\_A0A2E2YI39\_6\_274 | G | G | - | - | - | - | - | - | - | V | S | - | Y | P | N | G | R | G | - | - | - | - | - | R | G | K | D | P | E | A | S | L | R | - | - | - | - | R | V | A | I | N | V | K | G | E | - | I | F | P |
| 147 UniRef90\_A0A2W7G3K6\_24\_312 | A | D | - | - | - | - | - | - | - | G | G | - | - | - | - | - | - | - | - | - | - | - | - | V | G | G | G | L | A | A | T | M | Q | - | - | - | - | L | A | A | E | Y | V | E | G | G | - | V | F | G |
| 148 UniRef90\_A0A6B2RWJ8\_28\_298 | S | S | - | - | - | - | - | - | - | H | G | - | - | - | - | - | - | - | - | - | - | - | - | S | I | P | D | M | A | A | S | I | R | - | - | - | - | P | W | A | E | N | A | T | G | A | - | F | I | P |
| 149 UniRef90\_A0A537A198\_6\_270 | G | A | - | - | - | - | - | - | - | K | A | - | - | - | E | A | R | G | - | - | - | - | - | R | G | E | E | P | L | D | S | L | R | - | - | - | - | A | I | A | S | N | V | T | G | G | - | A | I | P |
| 150 UniRef90\_UPI00135F2885\_48\_334 | G | E | - | - | - | - | - | - | - | G | T | A | E | A | D | I | A | G | A | G | L | I | S | T | G | N | L | L | E | L | Q | L | E | - | - | - | - | N | A | A | T | D | L | R | G | Q | - | S | L | A |

  
  

|  |  |  |  |  |  |  |  |  |  |  |  |  |  |  |  |  |  |  |  |  |  |  |  |  |  |  |
| --- | --- | --- | --- | --- | --- | --- | --- | --- | --- | --- | --- | --- | --- | --- | --- | --- | --- | --- | --- | --- | --- | --- | --- | --- | --- | --- |
| **001 Input\_protein\_seq** | G | C | G | H | W | L | P | E | E | C | A | A | P | M | N | R | L | V | I | D | F | L | S | R | G | R |
| 002 UniRef90\_A0A2G0Y5Q3\_5\_314 | G | C | G | H | W | L | P | E | E | C | A | A | P | M | N | E | L | V | V | K | F | L | N | K | - | - |
| 003 UniRef90\_A0A172YJZ3\_2\_306 | G | C | G | H | W | L | P | E | E | C | T | A | Q | L | N | S | A | V | S | D | F | L | Q | - | - | - |
| 004 UniRef90\_A0A4Z1C7D3\_9\_314 | D | C | G | H | W | L | P | E | E | C | A | A | P | L | N | E | A | V | V | Q | F | L | T | - | - | - |
| 005 UniRef90\_A0A0A3Z1S4\_7\_319 | G | C | G | H | W | L | P | E | E | C | P | R | E | L | N | P | L | V | V | N | F | I | N | - | - | - |
| 006 UniRef90\_A0A329J6I4\_9\_315 | G | C | G | H | W | L | P | E | E | C | A | A | K | L | N | A | V | V | V | D | F | L | D | - | - | - |
| 007 UniRef90\_A0A4V1G750\_8\_314 | G | C | G | H | W | L | P | E | E | C | P | G | P | L | N | S | A | V | I | D | F | L | N | - | - | - |
| 008 UniRef90\_T2L220\_17\_309 | G | G | G | H | W | L | P | E | E | C | P | E | A | L | N | K | A | V | M | E | F | L | N | R | - | - |
| 009 UniRef90\_UPI000C2FE79C\_12\_314 | D | C | G | H | W | L | P | E | E | C | A | A | P | L | N | D | A | V | I | G | F | L | S | - | - | - |
| 010 UniRef90\_UPI00102FACD2\_13\_314 | G | C | G | H | W | L | P | E | E | C | S | F | A | L | N | S | A | V | I | T | F | L | D | - | - | - |
| 011 UniRef90\_UPI0014749FF6\_10\_312 | G | C | G | H | W | L | M | E | E | C | P | S | L | V | K | P | L | V | V | E | F | L | N | - | - | - |
| 012 UniRef90\_A0A3M4V4S1\_41\_332 | G | C | G | H | W | V | P | E | E | C | A | T | A | L | N | P | L | I | N | S | F | L | D | R | - | - |
| 013 UniRef90\_A0A3L8C981\_31\_324 | G | C | G | H | W | V | T | E | E | C | A | P | A | L | N | P | L | I | S | S | F | L | A | R | - | - |
| 014 UniRef90\_A0A1X0N2L9\_32\_323 | G | C | G | H | W | V | P | E | E | C | S | S | V | L | N | P | L | I | S | S | F | L | A | R | - | - |
| 015 UniRef90\_UPI00166CAA4C\_15\_313 | Q | S | G | H | W | V | P | E | E | R | P | D | T | L | L | K | L | L | E | P | F | L | - | - | - | - |
| 016 UniRef90\_UPI001661A3FF\_47\_334 | N | S | G | H | W | I | Y | E | E | R | P | E | E | L | T | A | L | L | L | D | F | L | G | R | - | - |
| 017 UniRef90\_A0A1H5MZC9\_42\_331 | D | S | G | H | W | L | Y | E | E | Q | P | A | A | M | T | R | I | L | L | T | F | L | K | - | - | - |
| 018 UniRef90\_UPI000690E8EC\_26\_310 | D | S | G | H | W | I | Y | E | E | H | P | E | E | L | T | R | T | L | L | G | F | L | N | R | - | - |
| 019 UniRef90\_R4LNI7\_31\_317 | D | S | G | H | W | L | Y | E | E | R | P | A | E | L | T | A | L | L | L | T | F | L | G | - | - | - |
| 020 UniRef90\_A0A4R2C838\_67\_350 | N | S | G | H | W | L | Y | Q | E | H | P | A | E | L | T | S | V | L | L | Q | F | L | - | - | - | - |
| 021 UniRef90\_A0A2P9HD85\_25\_319 | D | T | G | H | W | L | L | E | E | S | A | T | E | V | N | A | L | L | A | D | F | I | D | - | - | - |
| 022 UniRef90\_UPI000D14D87C\_43\_332 | D | S | G | H | W | I | Y | E | E | H | P | E | E | T | T | D | L | L | L | H | F | L | G | - | - | - |
| 023 UniRef90\_A0A1Q4ZL08\_6\_289 | D | C | G | H | W | I | F | E | E | R | P | A | E | L | T | S | Q | L | L | E | F | L | Q | - | - | - |
| 024 UniRef90\_A0A1I2GGE7\_30\_316 | G | S | G | H | W | I | Y | E | E | H | P | D | E | M | T | G | I | L | L | S | F | L | G | - | - | - |
| 025 UniRef90\_UPI0005580D51\_18\_305 | N | S | G | H | W | I | Y | E | E | H | P | A | E | L | T | R | L | L | L | T | F | L | - | - | - | - |
| 026 UniRef90\_A0A2W2F1P0\_6\_289 | D | S | G | H | W | L | F | E | E | Q | P | A | E | L | T | A | L | L | L | P | F | L | Q | - | - | - |
| 027 UniRef90\_UPI0010F9F40F\_6\_288 | D | S | G | H | W | L | Y | E | E | Q | P | A | E | L | T | A | L | L | L | P | F | L | - | - | - | - |
| 028 UniRef90\_A0A3E2YQE6\_6\_289 | D | C | G | H | W | L | Y | E | E | R | P | E | E | L | L | A | L | L | L | D | F | L | R | - | - | - |
| 029 UniRef90\_A0A1A9ACL3\_6\_288 | N | C | G | H | W | L | F | E | E | R | P | A | E | L | A | A | L | L | L | P | F | L | - | - | - | - |
| 030 UniRef90\_UPI00174B5D19\_38\_323 | G | T | G | H | W | V | T | E | E | R | P | E | E | V | T | K | L | L | A | E | F | L | - | - | - | - |
| 031 UniRef90\_A0A495JCH5\_50\_333 | D | S | G | H | W | M | W | E | E | K | P | E | E | V | T | N | R | L | L | T | F | L | N | - | - | - |
| 032 UniRef90\_UPI0013D14F7B\_11\_294 | G | A | G | H | W | I | Y | E | E | R | P | D | E | M | T | Q | V | L | L | T | F | L | - | - | - | - |
| 033 UniRef90\_UPI00036EC6E3\_56\_342 | N | S | G | H | W | L | Y | Q | E | H | P | A | E | L | T | T | I | L | L | Q | F | L | - | - | - | - |
| 034 UniRef90\_A0A1H3P9Q0\_6\_290 | E | C | G | H | W | L | F | E | E | Q | P | A | Q | L | T | E | Q | L | R | Q | F | L | P | A | - | - |
| 035 UniRef90\_A0A385B2U0\_41\_332 | D | T | G | H | W | L | T | E | E | R | P | Q | E | L | T | T | M | L | L | K | F | L | - | - | - | - |
| 036 UniRef90\_UPI001430CB85\_25\_306 | H | C | G | H | F | L | P | E | E | Q | S | T | A | V | A | G | E | L | L | E | F | - | - | - | - | - |
| 037 UniRef90\_A0A109IHW8\_6\_288 | D | C | G | H | W | L | F | E | E | R | P | A | E | L | T | A | L | L | L | P | F | L | - | - | - | - |
| 038 UniRef90\_A0A2T6L0M5\_14\_298 | G | A | G | H | W | V | T | E | E | R | P | H | E | V | T | E | L | L | R | T | F | L | - | - | - | - |
| 039 UniRef90\_A0A1C4Z0V4\_6\_288 | D | S | G | H | W | L | F | E | E | R | P | A | E | L | T | G | L | L | L | P | F | L | - | - | - | - |
| 040 UniRef90\_A0A4U3M272\_82\_365 | N | S | G | H | W | L | Y | Q | E | H | P | A | E | L | T | S | V | L | L | Q | F | L | - | - | - | - |
| 041 UniRef90\_UPI00052728DD\_22\_294 | H | C | G | H | F | L | P | E | E | Q | P | A | A | - | - | - | - | - | - | - | - | - | - | - | - | - |
| 042 UniRef90\_A0A6B8MSH7\_52\_336 | D | T | G | H | W | M | V | E | E | N | P | T | A | V | - | - | - | - | - | - | - | - | - | - | - | - |
| 043 UniRef90\_I4VSM6\_28\_303 | N | C | G | H | F | M | P | E | E | Q | P | A | A | V | - | - | - | - | - | - | - | - | - | - | - | - |
| 044 UniRef90\_A0A1I3BFK1\_40\_314 | F | C | G | H | F | L | P | E | E | Q | P | - | - | - | - | - | - | - | - | - | - | - | - | - | - | - |
| 045 UniRef90\_A0A1Z4J856\_42\_316 | N | C | G | H | W | I | P | E | E | R | P | A | Y | L | V | Q | Q | L | L | N | F | F | - | - | - | - |
| 046 UniRef90\_A0A0M4FVH2\_27\_314 | F | C | G | H | F | L | P | E | E | Q | P | E | A | V | S | R | E | L | K | A | F | F | S | L | - | - |
| 047 UniRef90\_UPI001032135D\_25\_299 | H | C | G | H | F | L | P | E | E | Q | P | Q | A | I | - | - | - | - | - | - | - | - | - | - | - | - |
| 048 UniRef90\_D5WL63\_24\_300 | H | C | G | H | F | I | P | E | E | Q | P | - | - | - | - | - | - | - | - | - | - | - | - | - | - | - |
| 049 UniRef90\_A0A1H3E943\_18\_291 | Q | C | G | H | Y | M | P | E | E | Q | P | D | V | I | S | Q | M | V | L | K | F | L | A | - | - | - |
| 050 UniRef90\_A0A346N7R3\_30\_317 | G | S | G | H | W | L | M | D | E | A | P | A | Q | V | I | P | L | L | L | E | F | L | N | - | - | - |
| 051 UniRef90\_A0A537Q000\_40\_318 | D | C | G | H | Y | T | P | E | E | C | P | D | R | F | L | D | L | V | K | P | F | L | A | - | - | - |
| 052 UniRef90\_E6V5X5\_24\_320 | G | S | G | H | W | L | M | E | E | A | P | E | Q | V | I | P | A | L | V | N | F | L | - | - | - | - |
| 053 UniRef90\_A0A484PNM3\_14\_291 | P | C | G | H | Y | V | P | E | E | A | P | D | F | L | V | R | H | L | R | D | F | L | - | - | - | - |
| 054 UniRef90\_UPI0011BDEFB8\_30\_311 | H | C | G | H | F | L | P | E | E | Q | P | Q | A | I | A | R | E | L | S | D | F | F | - | - | - | - |
| 055 UniRef90\_A0A1H2UZ41\_16\_293 | N | C | G | H | Y | M | P | E | E | Q | P | E | E | I | G | E | K | M | I | D | F | F | K | K | - | - |
| 056 UniRef90\_A0A1M5ZT81\_29\_302 | C | C | G | H | F | L | P | E | E | Q | P | A | A | V | - | - | - | - | - | - | - | - | - | - | - | - |
| 057 UniRef90\_A0A135GL34\_14\_283 | N | C | G | H | W | L | P | E | E | R | P | A | E | F | L | E | - | - | - | - | - | - | - | - | - | - |
| 058 UniRef90\_A0A484UEV2\_23\_280 | P | C | G | H | Y | V | P | E | E | A | P | - | - | - | - | - | - | - | - | - | - | - | - | - | - | - |
| 059 UniRef90\_A0A495NUG6\_51\_333 | S | S | G | H | W | I | W | E | E | Q | P | R | A | M | T | R | M | T | L | R | F | M | - | - | - | - |
| 060 UniRef90\_A0A1M5MA45\_15\_292 | G | S | G | H | W | M | V | Q | E | Q | T | A | Q | V | Q | K | G | L | M | D | F | L | R | - | - | - |
| 061 UniRef90\_UPI000EF95C7F\_30\_312 | D | C | G | H | Y | V | P | E | E | A | P | A | A | F | V | R | A | V | L | P | F | L | E | - | - | - |
| 062 UniRef90\_L9WLS3\_15\_271 | R | A | G | H | W | I | P | E | E | R | P | E | - | - | - | - | - | - | - | - | - | - | - | - | - | - |
| 063 UniRef90\_UPI0005D31B04\_21\_288 | R | S | G | H | W | L | T | E | E | Q | P | E | Q | L | T | A | A | L | L | E | F | - | - | - | - | - |
| 064 UniRef90\_UPI001408C682\_10\_298 | G | S | G | H | W | L | I | D | E | A | P | E | Q | V | V | P | A | L | V | Q | F | L | - | - | - | - |
| 065 UniRef90\_A0A1Y6KKD8\_22\_314 | S | C | G | H | Y | I | P | E | E | Q | P | A | V | V | A | E | E | L | M | G | F | F | S | R | - | - |
| 066 UniRef90\_A0A0X3RYG7\_65\_349 | K | T | G | H | F | V | N | E | E | R | P | K | E | V | T | A | A | L | Q | S | F | L | - | - | - | - |
| 067 UniRef90\_A0A2T0T762\_22\_302 | D | C | G | H | Y | A | A | E | E | Q | P | A | R | F | T | E | A | L | E | D | F | L | A | - | - | - |
| 068 UniRef90\_A0A2V6QCX2\_10\_286 | G | V | G | H | W | L | M | E | E | A | P | D | T | V | I | P | A | I | S | D | F | V | - | - | - | - |
| 069 UniRef90\_D8MTC4\_28\_309 | Y | C | G | H | F | L | P | E | E | Q | P | E | A | I | A | R | E | L | N | D | F | F | - | - | - | - |
| 070 UniRef90\_UPI0012FADC80\_10\_303 | G | A | G | H | W | L | V | Q | E | N | T | P | A | V | Q | Q | A | L | L | S | F | - | - | - | - | - |
| 071 UniRef90\_A0A2E5PAM0\_2\_278 | H | C | G | H | W | L | P | E | E | Q | P | D | T | V | A | R | E | M | L | N | F | F | - | - | - | - |
| 072 UniRef90\_T0HVL8\_12\_269 | Q | C | G | H | W | I | P | D | E | K | P | E | - | - | - | - | - | - | - | - | - | - | - | - | - | - |
| 073 UniRef90\_A0A3N4T2I8\_17\_298 | D | C | G | H | Y | P | A | E | E | Q | P | A | R | F | V | E | I | L | E | D | F | L | A | A | N | - |
| 074 UniRef90\_A0A433IAT3\_19\_312 | G | S | G | H | W | L | M | E | E | A | P | Q | A | V | I | P | A | I | I | A | F | L | - | - | - | - |
| 075 UniRef90\_A0A226WR47\_30\_304 | F | C | G | H | F | L | P | E | E | Q | P | A | A | V | - | - | - | - | - | - | - | - | - | - | - | - |
| 076 UniRef90\_UPI000DD77D66\_25\_314 | G | A | G | H | W | I | P | E | E | Q | P | A | A | V | T | A | A | L | I | D | F | L | P | - | - | - |
| 077 UniRef90\_A0A2A5QV31\_6\_277 | R | A | G | H | W | I | P | E | E | R | P | E | Y | F | V | E | R | L | T | E | F | - | - | - | - | - |
| 078 UniRef90\_A0A127EY60\_8\_296 | G | S | G | H | W | L | I | D | E | A | P | D | Q | V | I | - | - | - | - | - | - | - | - | - | - | - |
| 079 UniRef90\_UPI0004952425\_6\_308 | G | S | G | H | W | L | M | E | E | A | P | D | Q | V | I | P | K | L | V | E | F | L | N | R | - | - |
| 080 UniRef90\_A0A0D0JP71\_56\_330 | G | S | G | H | W | L | V | Q | E | N | T | A | Q | V | Q | K | D | L | L | A | F | - | - | - | - | - |
| 081 UniRef90\_A0A6A7LIC2\_1\_257 | L | S | G | H | Y | I | A | E | E | R | P | D | - | - | - | - | - | - | - | - | - | - | - | - | - | - |
| 082 UniRef90\_UPI00097BCF81\_14\_312 | G | S | G | H | W | L | M | E | E | A | P | D | Q | V | I | P | A | L | V | D | F | L | - | - | - | - |
| 083 UniRef90\_UPI001591AA5D\_7\_297 | D | C | G | H | Y | V | A | E | E | Q | P | V | P | F | T | E | A | L | E | D | F | L | G | - | - | - |
| 084 UniRef90\_A0A4P8YCR5\_18\_300 | H | C | G | H | F | I | P | E | E | Q | P | L | A | T | A | R | A | L | S | D | F | - | - | - | - | - |
| 085 UniRef90\_A0A4R0GJE6\_14\_295 | D | C | G | H | Y | P | A | E | E | Q | P | A | R | F | V | E | I | L | E | N | F | L | A | A | N | - |
| 086 UniRef90\_A0A502C146\_32\_301 | G | S | G | H | W | L | L | E | E | A | P | T | Q | V | I | P | L | L | V | D | F | L | N | Q | - | - |
| 087 UniRef90\_A0A4Y9SAQ9\_19\_291 | G | S | G | H | W | V | M | E | E | A | P | - | - | - | - | - | - | - | - | - | - | - | - | - | - | - |
| 088 UniRef90\_A0A327RPK7\_10\_286 | E | C | G | H | Y | I | A | E | E | Q | P | E | F | L | I | K | V | L | T | E | F | L | S | H | - | - |
| 089 UniRef90\_A0A239MTD5\_1\_269 | N | S | G | H | F | V | T | E | E | K | P | Q | E | V | T | A | M | L | Q | S | F | L | - | - | - | - |
| 090 UniRef90\_UPI00098F4FB4\_42\_302 | G | S | G | H | W | I | P | E | E | K | P | - | - | - | - | - | - | - | - | - | - | - | - | - | - | - |
| 091 UniRef90\_A0A261TYY2\_38\_305 | P | C | G | H | Y | V | P | E | E | A | P | - | - | - | - | - | - | - | - | - | - | - | - | - | - | - |
| 092 UniRef90\_UPI0015F81933\_27\_309 | D | A | G | H | F | I | P | D | E | Q | P | D | A | V | A | A | A | L | T | D | F | I | T | - | - | - |
| 093 UniRef90\_A0A0C7N0E5\_9\_278 | D | C | G | H | F | I | P | E | E | Q | P | S | - | - | - | - | - | - | - | - | - | - | - | - | - | - |
| 094 UniRef90\_UPI00112BEE4D\_14\_300 | D | C | G | H | Y | V | P | E | E | Q | P | G | A | L | A | G | L | L | R | D | F | L | E | R | - | - |
| 095 UniRef90\_A0A401Z9D4\_17\_277 | E | C | G | H | Y | I | A | E | E | Q | P | E | E | L | - | - | - | - | - | - | - | - | - | - | - | - |
| 096 UniRef90\_A0A5J6MKN6\_7\_281 | G | S | G | H | W | L | I | D | E | A | P | D | Q | V | I | P | A | L | - | - | - | - | - | - | - | - |
| 097 UniRef90\_A0A4Q7XSL0\_10\_307 | G | A | G | H | W | L | M | E | E | A | T | A | E | T | V | K | T | V | Q | S | F | L | - | - | - | - |
| 098 UniRef90\_UPI0016110D13\_9\_294 | N | C | G | H | F | Q | P | E | E | Q | P | E | A | V | A | T | T | I | V | R | Y | L | E | - | - | - |
| 099 UniRef90\_A0A520GLB7\_17\_313 | G | S | G | H | W | L | M | E | E | A | P | D | Q | V | I | P | A | L | I | G | F | L | - | - | - | - |
| 100 UniRef90\_A0A252EMP1\_9\_266 | D | C | G | H | W | V | P | D | E | K | - | - | - | - | - | - | - | - | - | - | - | - | - | - | - | - |
| 101 UniRef90\_UPI001668C4B3\_34\_306 | D | C | G | H | Y | P | A | E | E | A | P | E | A | V | L | A | - | - | - | - | - | - | - | - | - | - |
| 102 UniRef90\_UPI00135BC32E\_12\_265 | R | A | G | H | W | I | P | E | E | R | - | - | - | - | - | - | - | - | - | - | - | - | - | - | - | - |
| 103 UniRef90\_A0A2V9DES3\_17\_290 | N | S | G | H | W | L | M | E | E | A | P | D | Q | V | I | P | K | L | V | E | F | L | N | R | - | - |
| 104 UniRef90\_A0A6P0DAX4\_34\_314 | G | A | G | H | W | I | P | E | E | Q | P | V | A | V | T | T | A | L | T | D | F | L | P | - | - | - |
| 105 UniRef90\_A0A1M5KPU7\_13\_307 | G | S | G | H | W | L | M | E | E | A | P | N | Q | V | I | P | K | L | V | E | F | L | N | R | - | - |
| 106 UniRef90\_A0A2I8DLX4\_17\_295 | D | C | G | H | Y | I | P | E | E | Q | P | R | E | L | A | R | R | M | L | A | F | L | - | - | - | - |
| 107 UniRef90\_A0A5S4WMZ6\_36\_313 | K | T | G | H | W | L | P | D | E | N | P | D | Y | L | S | R | Q | L | L | A | F | L | - | - | - | - |
| 108 UniRef90\_UPI0003744601\_9\_294 | G | A | K | H | F | L | V | Q | D | Q | P | Q | A | A | T | E | A | I | L | S | F | L | T | R | - | - |
| 109 UniRef90\_A0A0H3KYM5\_9\_289 | N | C | G | H | F | Q | P | E | E | Q | P | E | A | T | A | N | A | L | - | - | - | - | - | - | - | - |
| 110 UniRef90\_UPI001616C592\_11\_307 | D | G | G | H | Y | I | S | E | E | A | P | E | A | F | L | A | A | V | E | T | F | L | - | - | - | - |
| 111 UniRef90\_A0A1U9ZZS0\_24\_308 | G | A | G | H | F | I | P | D | E | Q | P | E | A | V | A | A | A | V | A | D | F | I | L | A | G | - |
| 112 UniRef90\_UPI00146E50DC\_33\_308 | D | A | G | H | W | L | M | E | E | A | P | T | V | V | I | P | K | L | V | D | F | L | N | R | - | - |
| 113 UniRef90\_C3KLY5\_13\_287 | S | C | G | H | Y | L | A | E | E | Q | P | D | A | L | A | - | - | - | - | - | - | - | - | - | - | - |
| 114 UniRef90\_A0A2V6STQ7\_27\_303 | G | S | G | H | W | L | M | E | E | A | T | D | Q | V | V | P | A | I | V | A | F | L | - | - | - | - |
| 115 UniRef90\_UPI0008D5DA8A\_10\_290 | D | C | G | H | F | V | T | E | E | C | H | E | A | F | L | E | I | L | L | P | F | L | S | - | - | - |
| 116 UniRef90\_A0A3N2H8D2\_16\_299 | D | A | G | H | F | I | P | D | E | Q | P | D | A | V | A | D | A | L | T | G | F | L | A | - | - | - |
| 117 UniRef90\_A0A379Z3Q1\_16\_292 | G | C | G | H | F | I | T | E | E | C | H | E | E | F | I | A | L | V | I | P | F | L | S | G | - | - |
| 118 UniRef90\_A0A4R5QBW7\_8\_277 | G | C | G | H | W | I | P | E | E | R | P | D | W | V | T | A | Q | L | L | E | F | - | - | - | - | - |
| 119 UniRef90\_A0A163VVR8\_16\_291 | G | C | G | H | F | I | T | E | E | C | H | E | E | F | I | A | L | I | T | P | F | L | A | - | - | - |
| 120 UniRef90\_G0FSK7\_29\_305 | G | C | G | H | Y | P | A | E | E | A | P | Q | A | M | L | A | A | L | S | E | F | L | - | - | - | - |
| 121 UniRef90\_A0A4Q7FLU1\_10\_276 | G | A | G | H | W | I | P | E | E | Q | P | A | Q | L | A | K | A | F | Q | T | F | - | - | - | - | - |
| 122 UniRef90\_A0A2E3NGG1\_11\_269 | G | C | A | H | W | I | A | E | E | Q | P | E | R | L | - | - | - | - | - | - | - | - | - | - | - | - |
| 123 UniRef90\_A0A2J9ERN8\_9\_287 | E | C | G | H | F | L | P | E | E | K | P | D | A | V | A | D | - | - | - | - | - | - | - | - | - | - |
| 124 UniRef90\_UPI001677C581\_8\_288 | D | C | G | H | F | I | M | E | E | A | P | E | A | F | I | A | H | L | V | P | F | L | - | - | - | - |
| 125 UniRef90\_UPI0003039C4F\_8\_269 | G | C | G | H | W | I | P | E | E | L | P | D | - | - | - | - | - | - | - | - | - | - | - | - | - | - |
| 126 UniRef90\_A0A1B1YXM9\_17\_276 | R | C | G | H | W | I | A | E | E | Q | P | Q | - | - | - | - | - | - | - | - | - | - | - | - | - | - |
| 127 UniRef90\_A0A534ZXC6\_31\_288 | D | T | G | H | W | V | L | E | E | K | - | - | - | - | - | - | - | - | - | - | - | - | - | - | - | - |
| 128 UniRef90\_A0A2X1TAZ7\_42\_319 | E | C | G | H | F | V | T | E | E | C | P | E | Q | L | M | G | V | L | L | P | F | L | R | - | - | - |
| 129 UniRef90\_A0A4Q5QRH4\_8\_282 | D | C | G | H | F | Q | P | E | E | Q | P | - | - | - | - | - | - | - | - | - | - | - | - | - | - | - |
| 130 UniRef90\_A0A0D6HQT0\_17\_294 | D | C | G | H | Y | L | P | E | E | Q | P | R | E | L | A | R | R | M | L | A | F | L | - | - | - | - |
| 131 UniRef90\_A0A1G4JCM6\_9\_275 | E | C | G | H | Y | I | P | E | E | Q | P | - | - | - | - | - | - | - | - | - | - | - | - | - | - | - |
| 132 UniRef90\_UPI001269E708\_28\_301 | D | A | G | H | F | I | P | D | E | Q | P | E | A | - | - | - | - | - | - | - | - | - | - | - | - | - |
| 133 UniRef90\_A0A538SUN7\_3\_303 | H | S | G | H | W | V | M | E | E | N | P | K | A | T | M | A | L | V | M | D | F | L | S | K | - | - |
| 134 UniRef90\_UPI001646B9D9\_15\_288 | D | C | G | H | F | V | M | E | E | A | P | V | P | F | N | E | H | L | L | A | F | L | Q | - | - | - |
| 135 UniRef90\_K9DQK6\_17\_307 | G | A | G | H | W | M | M | E | E | N | P | D | A | T | V | E | L | I | D | D | F | L | T | A | G | - |
| 136 UniRef90\_A0A2N9BM66\_25\_297 | G | A | G | H | F | L | P | E | E | A | P | E | E | V | S | R | A | L | L | D | F | L | - | - | - | - |
| 137 UniRef90\_A0A370LBV7\_5\_266 | G | S | G | H | W | L | M | D | E | A | P | - | - | - | - | - | - | - | - | - | - | - | - | - | - | - |
| 138 UniRef90\_A0A2V9ZM06\_3\_258 | D | T | G | H | W | V | L | E | E | R | P | - | - | - | - | - | - | - | - | - | - | - | - | - | - | - |
| 139 UniRef90\_UPI00101F418B\_26\_310 | A | C | G | H | Y | V | P | E | E | A | P | E | T | V | I | R | Q | L | A | E | F | F | - | - | - | - |
| 140 UniRef90\_A0A4V2YYI8\_27\_305 | D | C | G | H | Y | P | A | E | E | A | P | E | A | M | L | S | A | L | T | A | F | L | - | - | - | - |
| 141 UniRef90\_UPI001487D2FB\_8\_292 | D | A | G | H | W | L | M | E | E | A | P | A | A | - | - | - | - | - | - | - | - | - | - | - | - | - |
| 142 UniRef90\_B9XAH7\_320\_607 | G | F | G | H | Y | L | P | E | E | C | P | E | E | L | T | R | A | I | F | E | F | W | - | - | - | - |
| 143 UniRef90\_UPI0013696204\_44\_306 | D | C | G | H | Y | P | A | E | E | A | P | G | A | - | - | - | - | - | - | - | - | - | - | - | - | - |
| 144 UniRef90\_W5WIM6\_13\_292 | D | C | G | H | F | P | A | E | E | A | P | Q | D | M | L | A | A | L | S | E | F | L | A | - | - | - |
| 145 UniRef90\_A0A2X2GG63\_10\_290 | D | C | G | H | F | I | T | E | E | C | H | E | A | F | I | A | L | V | V | P | F | L | - | - | - | - |
| 146 UniRef90\_A0A2E2YI39\_6\_274 | E | C | G | H | F | I | P | E | E | K | - | - | - | - | - | - | - | - | - | - | - | - | - | - | - | - |
| 147 UniRef90\_A0A2W7G3K6\_24\_312 | N | C | G | H | Y | V | P | E | E | A | P | R | A | L | T | E | R | L | L | R | F | L | D | R | - | - |
| 148 UniRef90\_A0A6B2RWJ8\_28\_298 | E | A | G | H | F | I | P | D | E | Q | P | - | - | - | - | - | - | - | - | - | - | - | - | - | - | - |
| 149 UniRef90\_A0A537A198\_6\_270 | E | C | G | H | F | I | P | E | E | Q | P | A | V | L | A | E | R | L | L | - | - | - | - | - | - | - |
| 150 UniRef90\_UPI00135F2885\_48\_334 | G | C | G | H | W | L | A | S | E | C | P | E | R | T | E | Q | L | L | N | D | F | F | S | - | - | - |

  
  

|  |  |  |  |  |  |  |  |  |  |  |  |  |  |  |  |  |  |
| --- | --- | --- | --- | --- | --- | --- | --- | --- | --- | --- | --- | --- | --- | --- | --- | --- | --- |
| |  |  |  |  |  |  |  |  |  | | --- | --- | --- | --- | --- | --- | --- | --- | --- | | 1 | 2 | 3 | 4 | 5 | 6 | 7 | 8 | 9 |   |  |  |  |  |  |  | | --- | --- | --- | --- | --- | --- | | **Variable** |  | **Average** |  | **Conserved** | | | |  |  |  | | --- | --- | | **X | - Insufficient data - the calculation for this site was performed on less than 10% of the sequences. |** |
