# Supplementary material for: Biochemical and structural characterization of two cif-like epoxide hydrolases from Burkholderia cenocepacia
Source: Curr Res Struct Biol. 2021 Feb 21;3:72–84. doi: 10.1016/j.crstbi.2021.02.002 (PMC8244358; doi:10.1016/j.crstbi.2021.02.002)
Supplement: Multimedia component 8 [file mmc8.zip › aCif_MSA.html]

ConSurf Color-Coded MSA

# ConSurf Color-Coded MSA

|  |  |  |  |  |  |  |  |  |  |  |  |  |  |  |  |  |  |  |  |  |  |  |  |  |  |  |  |  |  |  |  |  |  |  |  |  |  |  |  |  |  |  |  |  |  |  |  |  |  |  |
| --- | --- | --- | --- | --- | --- | --- | --- | --- | --- | --- | --- | --- | --- | --- | --- | --- | --- | --- | --- | --- | --- | --- | --- | --- | --- | --- | --- | --- | --- | --- | --- | --- | --- | --- | --- | --- | --- | --- | --- | --- | --- | --- | --- | --- | --- | --- | --- | --- | --- | --- |
| **001 Input\_protein\_seq** | M | K | K | F | F | K | V | M | T | L | S | A | A | M | G | M | F | G | L | N | Q | A | N | A | E | - | Y | D | P | N | L | K | S | I | D | T | P | P | A | V | S | Q | Q | M | F | N | K | V | K | S |
| 002 UniRef90\_D8JE57\_1\_349 | M | N | R | Y | F | K | N | I | S | I | S | T | A | F | I | L | V | S | S | S | Y | A | N | A | E | - | Y | D | P | N | L | K | S | I | D | T | P | P | A | V | S | Q | Q | M | F | N | E | V | K | S |
| 003 UniRef90\_A0A6B8MSH7\_6\_345 | - | - | - | - | - | - | - | - | T | L | F | T | T | M | L | F | A | S | V | S | S | T | Y | A | E | - | Y | D | P | N | L | K | S | I | D | T | P | P | A | V | S | Q | Q | K | F | D | L | V | K | S |
| 004 UniRef90\_A0A6B2FNS7\_11\_352 | - | - | - | - | - | - | - | - | L | I | A | T | A | V | S | L | S | A | T | H | A | A | R | A | E | - | Y | D | P | N | L | K | S | I | D | T | P | P | Q | V | S | Q | R | G | F | D | Q | V | K | A |
| 005 UniRef90\_UPI00073D803C\_6\_344 | - | - | - | - | - | - | - | - | A | L | L | A | A | M | V | F | A | A | L | N | S | A | H | A | E | - | Y | D | P | N | L | K | S | I | D | T | P | A | E | V | S | Q | Q | Q | F | N | Q | V | Q | S |
| 006 UniRef90\_A0A285K5K8\_3\_346 | - | - | - | - | F | K | - | V | A | L | L | S | S | L | L | L | S | A | S | G | V | A | V | A | E | - | Y | D | P | A | L | Q | S | I | D | T | P | A | A | E | S | Q | Q | Q | F | N | A | V | K | S |
| 007 UniRef90\_A0A379I9D6\_1\_346 | M | K | K | V | L | S | - | - | A | V | L | L | S | S | L | L | T | G | S | S | M | V | W | A | G | - | Y | D | P | A | M | K | S | I | D | T | P | P | G | V | S | R | Q | Q | F | D | A | V | K | S |
| 008 UniRef90\_A0A6B2FTI5\_15\_352 | - | - | - | - | - | - | - | - | - | - | - | - | L | L | S | L | S | T | T | S | S | A | R | A | Q | - | Y | D | P | G | L | K | S | I | D | T | P | P | Q | E | S | Q | R | Q | F | R | L | I | K | A |
| 009 UniRef90\_A0A2P9HD85\_2\_318 | - | - | - | - | - | - | - | - | - | - | - | - | - | - | - | - | - | - | - | - | - | - | - | - | - | - | - | - | - | - | - | P | S | I | N | T | P | P | E | V | S | K | A | N | L | A | K | V | K | A |
| 010 UniRef90\_A0A1T1H7K2\_15\_316 | - | - | - | - | - | - | - | - | - | - | - | - | - | - | - | - | - | - | - | - | - | - | - | - | - | - | - | - | - | - | - | - | - | - | - | - | - | - | - | - | A | Q | S | V | I | A | A | P | Q | L |
| 011 UniRef90\_A0A1A9HKQ2\_27\_313 | - | - | - | - | - | - | - | - | - | - | - | - | - | - | - | - | - | - | - | - | - | - | - | - | - | - | - | - | - | - | - | - | - | - | - | - | - | - | - | - | - | - | - | - | - | - | - | - | - | - |
| 012 UniRef90\_A0A0J6GRA0\_22\_303 | - | - | - | - | - | - | - | - | - | - | - | - | - | - | - | - | - | - | - | - | - | - | - | - | - | - | - | - | - | - | - | - | - | - | - | - | - | - | - | - | - | - | - | - | - | - | - | - | - | - |
| 013 UniRef90\_UPI000F5BD18A\_25\_314 | - | - | - | - | - | - | - | - | - | - | - | - | - | - | - | - | - | - | - | - | - | - | - | - | - | - | - | - | - | - | - | - | - | - | - | - | - | - | - | - | - | - | - | - | - | - | - | - | - | - |
| 014 UniRef90\_A0A4V1G750\_26\_311 | - | - | - | - | - | - | - | - | - | - | - | - | - | - | - | - | - | - | - | - | - | - | - | - | - | - | - | - | - | - | - | - | - | - | - | - | - | - | - | - | - | - | - | - | - | - | - | - | - | - |
| 015 UniRef90\_U3U231\_21\_300 | - | - | - | - | - | - | - | - | - | - | - | - | - | - | - | - | - | - | - | - | - | - | - | - | - | - | - | - | - | - | - | - | - | - | - | - | - | - | - | - | - | - | - | - | - | - | - | - | - | G |
| 016 UniRef90\_F2LPZ2\_26\_313 | - | - | - | - | - | - | - | - | - | - | - | - | - | - | - | - | - | - | - | - | - | - | - | - | - | - | - | - | - | - | - | - | - | - | - | - | - | - | - | - | - | - | - | - | - | - | - | - | - | - |
| 017 UniRef90\_A0A3M4V4S1\_6\_330 | - | - | - | - | F | H | A | I | A | L | A | V | A | A | L | A | M | G | V | T | Q | A | N | A | A | P | I | N | P | A | - | - | - | - | - | - | P | P | - | - | - | - | - | - | - | - | P | A | V | S |
| 018 UniRef90\_A0A178LP42\_49\_332 | - | - | - | - | - | - | - | - | - | - | - | - | - | - | - | - | - | - | - | - | - | - | - | - | - | - | - | - | - | - | - | - | - | - | - | - | - | - | - | - | - | - | - | - | - | - | - | - | - | - |
| 019 UniRef90\_T2L220\_20\_307 | - | - | - | - | - | - | - | - | - | - | - | - | - | - | - | - | - | - | - | - | - | - | - | - | - | - | - | - | - | - | - | - | - | - | - | - | - | - | - | - | - | - | - | - | - | - | - | - | - | - |
| 020 UniRef90\_UPI0014749FF6\_22\_304 | - | - | - | - | - | - | - | - | - | - | - | - | - | - | - | - | - | - | - | - | - | - | - | - | - | - | - | - | - | - | - | - | - | - | - | - | - | - | - | - | - | - | - | - | - | - | - | - | - | - |
| 021 UniRef90\_A0A172YJZ3\_18\_305 | - | - | - | - | - | - | - | - | - | - | - | - | - | - | - | - | - | - | - | - | - | - | - | - | - | - | - | - | - | - | - | - | - | - | - | - | - | - | - | - | - | - | - | - | - | - | - | - | - | - |
| 022 UniRef90\_UPI000737C6BF\_26\_313 | - | - | - | - | - | - | - | - | - | - | - | - | - | - | - | - | - | - | - | - | - | - | - | - | - | - | - | - | - | - | - | - | - | - | - | - | - | - | - | - | - | - | - | - | - | - | - | - | - | - |
| 023 UniRef90\_A0A329J6I4\_26\_309 | - | - | - | - | - | - | - | - | - | - | - | - | - | - | - | - | - | - | - | - | - | - | - | - | - | - | - | - | - | - | - | - | - | - | - | - | - | - | - | - | - | - | - | - | - | - | - | - | - | - |
| 024 UniRef90\_A0A2N3KZL1\_30\_325 | - | - | - | - | - | - | - | - | - | - | - | - | - | - | - | - | - | - | - | - | - | - | - | - | - | - | - | - | - | - | - | - | - | - | - | - | - | - | - | - | - | - | - | - | - | - | K | P | Q | S |
| 025 UniRef90\_UPI0006CA847F\_27\_323 | - | - | - | - | - | - | - | - | - | - | - | - | - | - | - | - | - | - | - | - | - | - | - | - | - | - | - | - | - | - | - | - | - | - | - | - | - | - | - | - | - | - | - | - | - | - | - | - | - | S |
| 026 UniRef90\_UPI00054E0E46\_26\_312 | - | - | - | - | - | - | - | - | - | - | - | - | - | - | - | - | - | - | - | - | - | - | - | - | - | - | - | - | - | - | - | - | - | - | - | - | - | - | - | - | - | - | - | - | - | - | - | - | - | - |
| 027 UniRef90\_A0A0S9PRE2\_29\_311 | - | - | - | - | - | - | - | - | - | - | - | - | - | - | - | - | - | - | - | - | - | - | - | - | - | - | - | - | - | - | - | - | - | - | - | - | - | - | - | - | - | - | - | - | - | - | - | - | - | - |
| 028 UniRef90\_W0AA74\_37\_318 | - | - | - | - | - | - | - | - | - | - | - | - | - | - | - | - | - | - | - | - | - | - | - | - | - | - | - | - | - | - | - | - | - | - | - | - | - | - | - | - | - | - | - | - | - | - | - | - | - | - |
| 029 UniRef90\_A0A2G0Y5Q3\_27\_312 | - | - | - | - | - | - | - | - | - | - | - | - | - | - | - | - | - | - | - | - | - | - | - | - | - | - | - | - | - | - | - | - | - | - | - | - | - | - | - | - | - | - | - | - | - | - | - | - | - | - |
| 030 UniRef90\_A0A1M6LZ92\_62\_349 | - | - | - | - | - | - | - | - | - | - | - | - | - | - | - | - | - | - | - | - | - | - | - | - | - | - | - | - | - | - | - | - | - | - | - | - | - | - | - | - | - | - | - | - | - | - | - | - | - | - |
| 031 UniRef90\_A0A4Z1C7D3\_28\_313 | - | - | - | - | - | - | - | - | - | - | - | - | - | - | - | - | - | - | - | - | - | - | - | - | - | - | - | - | - | - | - | - | - | - | - | - | - | - | - | - | - | - | - | - | - | - | - | - | - | - |
| 032 UniRef90\_A0A367MBG0\_273\_561 | - | - | - | - | - | - | - | - | - | - | - | - | - | - | - | - | - | - | - | - | - | - | - | - | - | - | - | - | - | - | - | - | - | - | - | - | - | - | - | - | - | - | - | - | - | - | - | - | - | - |
| 033 UniRef90\_UPI00067CEA54\_35\_318 | - | - | - | - | - | - | - | - | - | - | - | - | - | - | - | - | - | - | - | - | - | - | - | - | - | - | - | - | - | - | - | - | - | - | - | - | - | - | - | - | - | - | - | - | - | - | - | - | - | - |
| 034 UniRef90\_A0A1Y0L5M5\_31\_311 | - | - | - | - | - | - | - | - | - | - | - | - | - | - | - | - | - | - | - | - | - | - | - | - | - | - | - | - | - | - | - | - | - | - | - | - | - | - | - | - | - | - | - | - | - | - | - | - | - | - |
| 035 UniRef90\_UPI000C2FE79C\_29\_313 | - | - | - | - | - | - | - | - | - | - | - | - | - | - | - | - | - | - | - | - | - | - | - | - | - | - | - | - | - | - | - | - | - | - | - | - | - | - | - | - | - | - | - | - | - | - | - | - | - | - |
| 036 UniRef90\_A0A4P8SJX7\_14\_295 | - | - | - | - | - | - | - | - | - | - | - | - | - | - | - | - | - | - | - | - | - | - | - | - | - | - | - | - | - | - | - | - | - | - | - | - | - | - | - | - | - | - | - | - | - | - | - | - | - | - |
| 037 UniRef90\_A0A1I1BZT3\_28\_315 | - | - | - | - | - | - | - | - | - | - | - | - | - | - | - | - | - | - | - | - | - | - | - | - | - | - | - | - | - | - | - | - | - | - | - | - | - | - | - | - | - | - | - | - | - | - | - | - | - | - |
| 038 UniRef90\_UPI000DF0D36E\_15\_290 | - | - | - | - | - | - | - | - | - | - | - | - | - | - | - | - | - | - | - | - | - | - | - | - | - | - | - | - | - | - | - | - | - | - | - | - | - | - | - | - | - | - | - | - | - | - | - | - | - | - |
| 039 UniRef90\_UPI00136B8797\_37\_318 | - | - | - | - | - | - | - | - | - | - | - | - | - | - | - | - | - | - | - | - | - | - | - | - | - | - | - | - | - | - | - | - | - | - | - | - | - | - | - | - | - | - | - | - | - | - | - | - | - | - |
| 040 UniRef90\_A0A1H4CLJ1\_28\_313 | - | - | - | - | - | - | - | - | - | - | - | - | - | - | - | - | - | - | - | - | - | - | - | - | - | - | - | - | - | - | - | - | - | - | - | - | - | - | - | - | - | - | - | - | - | - | - | - | - | - |
| 041 UniRef90\_UPI000E1FE28F\_29\_313 | - | - | - | - | - | - | - | - | - | - | - | - | - | - | - | - | - | - | - | - | - | - | - | - | - | - | - | - | - | - | - | - | - | - | - | - | - | - | - | - | - | - | - | - | - | - | - | - | - | - |
| 042 UniRef90\_A0A556AX95\_26\_313 | - | - | - | - | - | - | - | - | - | - | - | - | - | - | - | - | - | - | - | - | - | - | - | - | - | - | - | - | - | - | - | - | - | - | - | - | - | - | - | - | - | - | - | - | - | - | - | - | - | - |
| 043 UniRef90\_A0A6M8HNY7\_38\_322 | - | - | - | - | - | - | - | - | - | - | - | - | - | - | - | - | - | - | - | - | - | - | - | - | - | - | - | - | - | - | - | - | - | - | - | - | - | - | - | - | - | - | - | - | - | - | - | - | - | - |
| 044 UniRef90\_UPI001612B72A\_24\_301 | - | - | - | - | - | - | - | - | - | - | - | - | - | - | - | - | - | - | - | - | - | - | - | - | - | - | - | - | - | - | - | - | - | - | - | - | - | - | - | - | - | - | - | - | - | - | - | - | - | - |
| 045 UniRef90\_UPI0012F66D09\_46\_328 | - | - | - | - | - | - | - | - | - | - | - | - | - | - | - | - | - | - | - | - | - | - | - | - | - | - | - | - | - | - | - | - | - | - | - | - | - | - | - | - | - | - | - | - | - | - | - | - | - | - |
| 046 UniRef90\_UPI000A94940B\_29\_316 | - | - | - | - | - | - | - | - | - | - | - | - | - | - | - | - | - | - | - | - | - | - | - | - | - | - | - | - | - | - | - | - | - | - | - | - | - | - | - | - | - | - | - | - | - | - | - | - | - | - |
| 047 UniRef90\_UPI00102FACD2\_29\_312 | - | - | - | - | - | - | - | - | - | - | - | - | - | - | - | - | - | - | - | - | - | - | - | - | - | - | - | - | - | - | - | - | - | - | - | - | - | - | - | - | - | - | - | - | - | - | - | - | - | - |
| 048 UniRef90\_A0A1S8YNF4\_19\_297 | - | - | - | - | - | - | - | - | - | - | - | - | - | - | - | - | - | - | - | - | - | - | - | - | - | - | - | - | - | - | - | - | - | - | - | - | - | - | - | - | - | - | - | - | - | - | - | - | - | - |
| 049 UniRef90\_UPI0015755320\_14\_301 | - | - | - | - | - | - | - | - | - | - | - | - | - | - | - | - | - | - | - | - | - | - | - | - | - | - | - | - | - | - | - | - | - | - | - | - | - | - | - | - | - | - | - | - | - | - | - | - | - | - |
| 050 UniRef90\_A0A427Q4N3\_22\_313 | - | - | - | - | - | - | - | - | - | - | - | - | - | - | - | - | - | - | - | - | - | - | - | - | - | - | - | - | - | - | - | - | - | - | - | - | - | - | - | - | - | - | - | - | - | - | - | - | - | - |
| 051 UniRef90\_A0A0A3Z1S4\_30\_318 | - | - | - | - | - | - | - | - | - | - | - | - | - | - | - | - | - | - | - | - | - | - | - | - | - | - | - | - | - | - | - | - | - | - | - | - | - | - | - | - | - | - | - | - | - | - | - | - | - | - |
| 052 UniRef90\_A0A3L8C981\_35\_323 | - | - | - | - | - | - | - | - | - | - | - | - | - | - | - | - | - | - | - | - | - | - | - | - | - | - | - | - | - | - | - | - | - | - | - | - | - | - | - | - | - | - | - | - | - | - | - | - | - | - |
| 053 UniRef90\_A0A6L5BLL6\_28\_324 | - | - | - | - | - | - | - | - | - | - | - | - | - | - | - | - | - | - | - | - | - | - | - | - | - | - | - | - | - | - | - | - | - | - | - | - | - | - | - | - | - | - | - | - | - | - | P | I | K | A |
| 054 UniRef90\_A0A6B1J288\_27\_314 | - | - | - | - | - | - | - | - | - | - | - | - | - | - | - | - | - | - | - | - | - | - | - | - | - | - | - | - | - | - | - | - | - | - | - | - | - | - | - | - | - | - | - | - | - | - | - | - | - | - |
| 055 UniRef90\_A0A263NNG9\_22\_274 | - | - | - | - | - | - | - | - | - | - | - | - | - | - | - | - | - | - | - | - | - | - | - | - | - | - | - | - | - | - | - | - | - | - | - | - | - | - | - | - | - | - | - | - | - | - | - | - | - | - |
| 056 UniRef90\_W0HXQ2\_33\_307 | - | - | - | - | - | - | - | - | - | - | - | - | - | - | - | - | - | - | - | - | - | - | - | - | - | - | - | - | - | - | - | - | - | - | - | - | - | - | - | - | - | - | - | - | - | - | - | - | - | - |
| 057 UniRef90\_A0A031HKS8\_40\_323 | - | - | - | - | - | - | - | - | - | - | - | - | - | - | - | - | - | - | - | - | - | - | - | - | - | - | - | - | - | - | - | - | - | - | - | - | - | - | - | - | - | - | - | - | - | - | - | - | - | - |
| 058 UniRef90\_A0A4R2FY05\_27\_320 | - | - | - | - | - | - | - | - | - | - | - | - | - | - | - | - | - | - | - | - | - | - | - | - | - | - | - | - | - | - | - | - | - | - | - | - | - | - | - | - | - | - | - | - | - | - | - | - | - | S |
| 059 UniRef90\_A0A1X0N2L9\_36\_321 | - | - | - | - | - | - | - | - | - | - | - | - | - | - | - | - | - | - | - | - | - | - | - | - | - | - | - | - | - | - | - | - | - | - | - | - | - | - | - | - | - | - | - | - | - | - | - | - | - | - |
| 060 UniRef90\_A0A4R1U0K9\_37\_322 | - | - | - | - | - | - | - | - | - | - | - | - | - | - | - | - | - | - | - | - | - | - | - | - | - | - | - | - | - | - | - | - | - | - | - | - | - | - | - | - | - | - | - | - | - | - | - | - | - | - |
| 061 UniRef90\_A0A0Q8TEQ0\_42\_323 | - | - | - | - | - | - | - | - | - | - | - | - | - | - | - | - | - | - | - | - | - | - | - | - | - | - | - | - | - | - | - | - | - | - | - | - | - | - | - | - | - | - | - | - | - | - | - | - | - | - |
| 062 UniRef90\_A0A0Q6ZEJ0\_41\_323 | - | - | - | - | - | - | - | - | - | - | - | - | - | - | - | - | - | - | - | - | - | - | - | - | - | - | - | - | - | - | - | - | - | - | - | - | - | - | - | - | - | - | - | - | - | - | - | - | - | - |
| 063 UniRef90\_UPI0004C0CBB1\_54\_338 | - | - | - | - | - | - | - | - | - | - | - | - | - | - | - | - | - | - | - | - | - | - | - | - | - | - | - | - | - | - | - | - | - | - | - | - | - | - | - | - | - | - | - | - | - | - | - | - | - | - |
| 064 UniRef90\_UPI0004CAB4F8\_56\_339 | - | - | - | - | - | - | - | - | - | - | - | - | - | - | - | - | - | - | - | - | - | - | - | - | - | - | - | - | - | - | - | - | - | - | - | - | - | - | - | - | - | - | - | - | - | - | - | - | - | - |
| 065 UniRef90\_UPI00049002CC\_60\_343 | - | - | - | - | - | - | - | - | - | - | - | - | - | - | - | - | - | - | - | - | - | - | - | - | - | - | - | - | - | - | - | - | - | - | - | - | - | - | - | - | - | - | - | - | - | - | - | - | - | - |
| 066 UniRef90\_A0A561IX74\_59\_327 | - | - | - | - | - | - | - | - | - | - | - | - | - | - | - | - | - | - | - | - | - | - | - | - | - | - | - | - | - | - | - | - | - | - | - | - | - | - | - | - | - | - | - | - | - | - | - | - | - | - |
| 067 UniRef90\_A0A0X3RYG7\_67\_350 | - | - | - | - | - | - | - | - | - | - | - | - | - | - | - | - | - | - | - | - | - | - | - | - | - | - | - | - | - | - | - | - | - | - | - | - | - | - | - | - | - | - | - | - | - | - | - | - | - | - |
| 068 UniRef90\_A0A2W2E1M5\_55\_338 | - | - | - | - | - | - | - | - | - | - | - | - | - | - | - | - | - | - | - | - | - | - | - | - | - | - | - | - | - | - | - | - | - | - | - | - | - | - | - | - | - | - | - | - | - | - | - | - | - | - |
| 069 UniRef90\_A0A2V7YB42\_28\_306 | - | - | - | - | - | - | - | - | - | - | - | - | - | - | - | - | - | - | - | - | - | - | - | - | - | - | - | - | - | - | - | - | - | - | - | - | - | - | - | - | - | - | - | - | - | - | - | - | - | - |
| 070 UniRef90\_A0A544XC49\_52\_336 | - | - | - | - | - | - | - | - | - | - | - | - | - | - | - | - | - | - | - | - | - | - | - | - | - | - | - | - | - | - | - | - | - | - | - | - | - | - | - | - | - | - | - | - | - | - | - | - | - | - |
| 071 UniRef90\_A0A101PQ90\_46\_324 | - | - | - | - | - | - | - | - | - | - | - | - | - | - | - | - | - | - | - | - | - | - | - | - | - | - | - | - | - | - | - | - | - | - | - | - | - | - | - | - | - | - | - | - | - | - | - | - | - | - |
| 072 UniRef90\_A0A1C4VK67\_14\_288 | - | - | - | - | - | - | - | - | - | - | - | - | - | - | - | - | - | - | - | - | - | - | - | - | - | - | - | - | - | - | - | - | - | - | - | - | - | - | - | - | - | - | - | - | - | - | - | - | - | - |
| 073 UniRef90\_A0A0X3RPC2\_57\_340 | - | - | - | - | - | - | - | - | - | - | - | - | - | - | - | - | - | - | - | - | - | - | - | - | - | - | - | - | - | - | - | - | - | - | - | - | - | - | - | - | - | - | - | - | - | - | - | - | - | - |
| 074 UniRef90\_A0A4R2VWW4\_55\_319 | - | - | - | - | - | - | - | - | - | - | - | - | - | - | - | - | - | - | - | - | - | - | - | - | - | - | - | - | - | - | - | - | - | - | - | - | - | - | - | - | - | - | - | - | - | - | - | - | - | - |
| 075 UniRef90\_A0A1Q7VB11\_36\_307 | - | - | - | - | - | - | - | - | - | - | - | - | - | - | - | - | - | - | - | - | - | - | - | - | - | - | - | - | - | - | - | - | - | - | - | - | - | - | - | - | - | - | - | - | - | - | - | - | - | - |
| 076 UniRef90\_C7PGQ3\_38\_305 | - | - | - | - | - | - | - | - | - | - | - | - | - | - | - | - | - | - | - | - | - | - | - | - | - | - | - | - | - | - | - | - | - | - | - | - | - | - | - | - | - | - | - | - | - | - | - | - | - | - |
| 077 UniRef90\_A0A1N7R4E6\_62\_325 | - | - | - | - | - | - | - | - | - | - | - | - | - | - | - | - | - | - | - | - | - | - | - | - | - | - | - | - | - | - | - | - | - | - | - | - | - | - | - | - | - | - | - | - | - | - | - | - | - | - |
| 078 UniRef90\_A0A2H2Y4V3\_41\_307 | - | - | - | - | - | - | - | - | - | - | - | - | - | - | - | - | - | - | - | - | - | - | - | - | - | - | - | - | - | - | - | - | - | - | - | - | - | - | - | - | - | - | - | - | - | - | - | - | - | - |
| 079 UniRef90\_A0A448SCU6\_8\_276 | - | - | - | - | - | - | - | - | - | - | - | - | - | - | - | - | - | - | - | - | - | - | - | - | - | - | - | - | - | - | - | - | - | - | - | - | - | - | - | - | - | - | - | - | - | - | - | - | - | - |
| 080 UniRef90\_A0A365Y5E2\_33\_303 | - | - | - | - | - | - | - | - | - | - | - | - | - | - | - | - | - | - | - | - | - | - | - | - | - | - | - | - | - | - | - | - | - | - | - | - | - | - | - | - | - | - | - | - | - | - | - | - | - | - |
| 081 UniRef90\_A0A1I2CFD2\_63\_327 | - | - | - | - | - | - | - | - | - | - | - | - | - | - | - | - | - | - | - | - | - | - | - | - | - | - | - | - | - | - | - | - | - | - | - | - | - | - | - | - | - | - | - | - | - | - | - | - | - | - |
| 082 UniRef90\_A0A1G7Z850\_54\_322 | - | - | - | - | - | - | - | - | - | - | - | - | - | - | - | - | - | - | - | - | - | - | - | - | - | - | - | - | - | - | - | - | - | - | - | - | - | - | - | - | - | - | - | - | - | - | - | - | - | - |
| 083 UniRef90\_A0A2V9XG70\_35\_308 | - | - | - | - | - | - | - | - | - | - | - | - | - | - | - | - | - | - | - | - | - | - | - | - | - | - | - | - | - | - | - | - | - | - | - | - | - | - | - | - | - | - | - | - | - | - | - | - | - | - |
| 084 UniRef90\_A0A0Q4GSS4\_39\_308 | - | - | - | - | - | - | - | - | - | - | - | - | - | - | - | - | - | - | - | - | - | - | - | - | - | - | - | - | - | - | - | - | - | - | - | - | - | - | - | - | - | - | - | - | - | - | - | - | - | - |
| 085 UniRef90\_A0A0M9ZTU1\_1\_271 | - | - | - | - | - | - | - | - | - | - | - | - | - | - | - | - | - | - | - | - | - | - | - | - | - | - | - | - | - | - | - | - | - | - | - | - | - | - | - | - | - | - | - | - | - | - | - | - | - | - |
| 086 UniRef90\_A0A239P597\_1\_270 | - | - | - | - | - | - | - | - | - | - | - | - | - | - | - | - | - | - | - | - | - | - | - | - | - | - | - | - | - | - | - | - | - | - | - | - | - | - | - | - | - | - | - | - | - | - | - | - | - | - |
| 087 UniRef90\_UPI00128E3D53\_19\_294 | - | - | - | - | - | - | - | - | - | - | - | - | - | - | - | - | - | - | - | - | - | - | - | - | - | - | - | - | - | - | - | - | - | - | - | - | - | - | - | - | - | - | - | - | - | - | - | - | - | - |
| 088 UniRef90\_A0A1V9E1W0\_57\_321 | - | - | - | - | - | - | - | - | - | - | - | - | - | - | - | - | - | - | - | - | - | - | - | - | - | - | - | - | - | - | - | - | - | - | - | - | - | - | - | - | - | - | - | - | - | - | - | - | - | - |
| 089 UniRef90\_A0A239MTD5\_1\_269 | - | - | - | - | - | - | - | - | - | - | - | - | - | - | - | - | - | - | - | - | - | - | - | - | - | - | - | - | - | - | - | - | - | - | - | - | - | - | - | - | - | - | - | - | - | - | - | - | - | - |
| 090 UniRef90\_UPI0009FCBBE8\_54\_317 | - | - | - | - | - | - | - | - | - | - | - | - | - | - | - | - | - | - | - | - | - | - | - | - | - | - | - | - | - | - | - | - | - | - | - | - | - | - | - | - | - | - | - | - | - | - | - | - | - | - |
| 091 UniRef90\_A0A1A9HTL2\_19\_299 | - | - | - | - | - | - | - | - | - | - | - | - | - | - | - | - | - | - | - | - | - | - | - | - | - | - | - | - | - | - | - | - | - | - | - | - | - | - | - | - | - | - | - | - | - | - | - | - | - | - |
| 092 UniRef90\_A0A4R7CM41\_23\_285 | - | - | - | - | - | - | - | - | - | - | - | - | - | - | - | - | - | - | - | - | - | - | - | - | - | - | - | - | - | - | - | - | - | - | - | - | - | - | - | - | - | - | - | - | - | - | - | - | - | - |
| 093 UniRef90\_A0A562QYK6\_25\_307 | - | - | - | - | - | - | - | - | - | - | - | - | - | - | - | - | - | - | - | - | - | - | - | - | - | - | - | - | - | - | - | - | - | - | - | - | - | - | - | - | - | - | - | - | - | - | - | - | - | - |
| 094 UniRef90\_A0A2V9ZM06\_3\_269 | - | - | - | - | - | - | - | - | - | - | - | - | - | - | - | - | - | - | - | - | - | - | - | - | - | - | - | - | - | - | - | - | - | - | - | - | - | - | - | - | - | - | - | - | - | - | - | - | - | - |
| 095 UniRef90\_A0A4P8YF40\_11\_271 | - | - | - | - | - | - | - | - | - | - | - | - | - | - | - | - | - | - | - | - | - | - | - | - | - | - | - | - | - | - | - | - | - | - | - | - | - | - | - | - | - | - | - | - | - | - | - | - | - | - |
| 096 UniRef90\_A0A2V6E8I4\_30\_303 | - | - | - | - | - | - | - | - | - | - | - | - | - | - | - | - | - | - | - | - | - | - | - | - | - | - | - | - | - | - | - | - | - | - | - | - | - | - | - | - | - | - | - | - | - | - | - | - | - | - |
| 097 UniRef90\_A0A2V9BFT0\_23\_295 | - | - | - | - | - | - | - | - | - | - | - | - | - | - | - | - | - | - | - | - | - | - | - | - | - | - | - | - | - | - | - | - | - | - | - | - | - | - | - | - | - | - | - | - | - | - | - | - | - | - |
| 098 UniRef90\_A0A2V9H5C8\_29\_298 | - | - | - | - | - | - | - | - | - | - | - | - | - | - | - | - | - | - | - | - | - | - | - | - | - | - | - | - | - | - | - | - | - | - | - | - | - | - | - | - | - | - | - | - | - | - | - | - | - | - |
| 099 UniRef90\_A0A101CRV3\_44\_308 | - | - | - | - | - | - | - | - | - | - | - | - | - | - | - | - | - | - | - | - | - | - | - | - | - | - | - | - | - | - | - | - | - | - | - | - | - | - | - | - | - | - | - | - | - | - | - | - | - | - |
| 100 UniRef90\_A0A5M6CFP9\_57\_321 | - | - | - | - | - | - | - | - | - | - | - | - | - | - | - | - | - | - | - | - | - | - | - | - | - | - | - | - | - | - | - | - | - | - | - | - | - | - | - | - | - | - | - | - | - | - | - | - | - | - |
| 101 UniRef90\_A0A401Z9D4\_19\_284 | - | - | - | - | - | - | - | - | - | - | - | - | - | - | - | - | - | - | - | - | - | - | - | - | - | - | - | - | - | - | - | - | - | - | - | - | - | - | - | - | - | - | - | - | - | - | - | - | - | - |
| 102 UniRef90\_A0A2V8XJ58\_32\_308 | - | - | - | - | - | - | - | - | - | - | - | - | - | - | - | - | - | - | - | - | - | - | - | - | - | - | - | - | - | - | - | - | - | - | - | - | - | - | - | - | - | - | - | - | - | - | - | - | - | - |
| 103 UniRef90\_A0A252EMP1\_15\_277 | - | - | - | - | - | - | - | - | - | - | - | - | - | - | - | - | - | - | - | - | - | - | - | - | - | - | - | - | - | - | - | - | - | - | - | - | - | - | - | - | - | - | - | - | - | - | - | - | - | - |
| 104 UniRef90\_A0A1H3H759\_34\_310 | - | - | - | - | - | - | - | - | - | - | - | - | - | - | - | - | - | - | - | - | - | - | - | - | - | - | - | - | - | - | - | - | - | - | - | - | - | - | - | - | - | - | - | - | - | - | - | - | - | - |
| 105 UniRef90\_A0A4D7B7N1\_3\_270 | - | - | - | - | - | - | - | - | - | - | - | - | - | - | - | - | - | - | - | - | - | - | - | - | - | - | - | - | - | - | - | - | - | - | - | - | - | - | - | - | - | - | - | - | - | - | - | - | - | - |
| 106 UniRef90\_A0A1Q7MUT6\_34\_308 | - | - | - | - | - | - | - | - | - | - | - | - | - | - | - | - | - | - | - | - | - | - | - | - | - | - | - | - | - | - | - | - | - | - | - | - | - | - | - | - | - | - | - | - | - | - | - | - | - | - |
| 107 UniRef90\_A0A2V6KQW0\_24\_297 | - | - | - | - | - | - | - | - | - | - | - | - | - | - | - | - | - | - | - | - | - | - | - | - | - | - | - | - | - | - | - | - | - | - | - | - | - | - | - | - | - | - | - | - | - | - | - | - | - | - |
| 108 UniRef90\_A0A4R6LFZ5\_44\_308 | - | - | - | - | - | - | - | - | - | - | - | - | - | - | - | - | - | - | - | - | - | - | - | - | - | - | - | - | - | - | - | - | - | - | - | - | - | - | - | - | - | - | - | - | - | - | - | - | - | - |
| 109 UniRef90\_A0A1H7UU25\_49\_318 | - | - | - | - | - | - | - | - | - | - | - | - | - | - | - | - | - | - | - | - | - | - | - | - | - | - | - | - | - | - | - | - | - | - | - | - | - | - | - | - | - | - | - | - | - | - | - | - | - | - |
| 110 UniRef90\_UPI0008326796\_14\_259 | - | - | - | - | - | - | - | - | - | - | - | - | - | - | - | - | - | - | - | - | - | - | - | - | - | - | - | - | - | - | - | - | - | - | - | - | - | - | - | - | - | - | - | - | - | - | - | - | - | - |
| 111 UniRef90\_A0A2V5S8F2\_33\_306 | - | - | - | - | - | - | - | - | - | - | - | - | - | - | - | - | - | - | - | - | - | - | - | - | - | - | - | - | - | - | - | - | - | - | - | - | - | - | - | - | - | - | - | - | - | - | - | - | - | - |
| 112 UniRef90\_A0A3S3VTN8\_36\_308 | - | - | - | - | - | - | - | - | - | - | - | - | - | - | - | - | - | - | - | - | - | - | - | - | - | - | - | - | - | - | - | - | - | - | - | - | - | - | - | - | - | - | - | - | - | - | - | - | - | - |
| 113 UniRef90\_A0A6J4PSU4\_16\_248 | - | - | - | - | - | - | - | - | - | - | - | - | - | - | - | - | - | - | - | - | - | - | - | - | - | - | - | - | - | - | - | - | - | - | - | - | - | - | - | - | - | - | - | - | - | - | - | - | - | - |
| 114 UniRef90\_A0A534ZXC6\_26\_300 | - | - | - | - | - | - | - | - | - | - | - | - | - | - | - | - | - | - | - | - | - | - | - | - | - | - | - | - | - | - | - | - | - | - | - | - | - | - | - | - | - | - | - | - | - | - | - | - | - | - |
| 115 UniRef90\_A0A2A9KBX9\_78\_358 | - | - | - | - | - | - | - | - | - | - | - | - | - | - | - | - | - | - | - | - | - | - | - | - | - | - | - | - | - | - | - | - | - | - | - | - | - | - | - | - | - | - | - | - | - | - | - | - | - | - |
| 116 UniRef90\_A0A2W0BWH7\_16\_287 | - | - | - | - | - | - | - | - | - | - | - | - | - | - | - | - | - | - | - | - | - | - | - | - | - | - | - | - | - | - | - | - | - | - | - | - | - | - | - | - | - | - | - | - | - | - | - | - | - | - |
| 117 UniRef90\_A0A1R1RMU1\_30\_310 | - | - | - | - | - | - | - | - | - | - | - | - | - | - | - | - | - | - | - | - | - | - | - | - | - | - | - | - | - | - | - | - | - | - | - | - | - | - | - | - | - | - | - | - | - | - | - | - | - | - |
| 118 UniRef90\_A0A3A1NH59\_38\_305 | - | - | - | - | - | - | - | - | - | - | - | - | - | - | - | - | - | - | - | - | - | - | - | - | - | - | - | - | - | - | - | - | - | - | - | - | - | - | - | - | - | - | - | - | - | - | - | - | - | - |
| 119 UniRef90\_A0A0Q6WP84\_43\_317 | - | - | - | - | - | - | - | - | - | - | - | - | - | - | - | - | - | - | - | - | - | - | - | - | - | - | - | - | - | - | - | - | - | - | - | - | - | - | - | - | - | - | - | - | - | - | - | - | - | - |
| 120 UniRef90\_A0A2V6P1A8\_15\_289 | - | - | - | - | - | - | - | - | - | - | - | - | - | - | - | - | - | - | - | - | - | - | - | - | - | - | - | - | - | - | - | - | - | - | - | - | - | - | - | - | - | - | - | - | - | - | - | - | - | - |
| 121 UniRef90\_A0A2V5NC55\_35\_307 | - | - | - | - | - | - | - | - | - | - | - | - | - | - | - | - | - | - | - | - | - | - | - | - | - | - | - | - | - | - | - | - | - | - | - | - | - | - | - | - | - | - | - | - | - | - | - | - | - | - |
| 122 UniRef90\_A0A2V9BM40\_39\_318 | - | - | - | - | - | - | - | - | - | - | - | - | - | - | - | - | - | - | - | - | - | - | - | - | - | - | - | - | - | - | - | - | - | - | - | - | - | - | - | - | - | - | - | - | - | - | - | - | - | - |
| 123 UniRef90\_A0A5B8WA72\_34\_302 | - | - | - | - | - | - | - | - | - | - | - | - | - | - | - | - | - | - | - | - | - | - | - | - | - | - | - | - | - | - | - | - | - | - | - | - | - | - | - | - | - | - | - | - | - | - | - | - | - | - |
| 124 UniRef90\_A0A1Y6D2S0\_25\_299 | - | - | - | - | - | - | - | - | - | - | - | - | - | - | - | - | - | - | - | - | - | - | - | - | - | - | - | - | - | - | - | - | - | - | - | - | - | - | - | - | - | - | - | - | - | - | - | - | - | - |
| 125 UniRef90\_A0A494W0L3\_12\_284 | - | - | - | - | - | - | - | - | - | - | - | - | - | - | - | - | - | - | - | - | - | - | - | - | - | - | - | - | - | - | - | - | - | - | - | - | - | - | - | - | - | - | - | - | - | - | - | - | - | - |
| 126 UniRef90\_UPI0015CD0065\_39\_309 | - | - | - | - | - | - | - | - | - | - | - | - | - | - | - | - | - | - | - | - | - | - | - | - | - | - | - | - | - | - | - | - | - | - | - | - | - | - | - | - | - | - | - | - | - | - | - | - | - | - |
| 127 UniRef90\_UPI00166E2F5D\_15\_289 | - | - | - | - | - | - | - | - | - | - | - | - | - | - | - | - | - | - | - | - | - | - | - | - | - | - | - | - | - | - | - | - | - | - | - | - | - | - | - | - | - | - | - | - | - | - | - | - | - | - |
| 128 UniRef90\_A0A372NPX1\_36\_305 | - | - | - | - | - | - | - | - | - | - | - | - | - | - | - | - | - | - | - | - | - | - | - | - | - | - | - | - | - | - | - | - | - | - | - | - | - | - | - | - | - | - | - | - | - | - | - | - | - | - |
| 129 UniRef90\_A0A317JA08\_13\_279 | - | - | - | - | - | - | - | - | - | - | - | - | - | - | - | - | - | - | - | - | - | - | - | - | - | - | - | - | - | - | - | - | - | - | - | - | - | - | - | - | - | - | - | - | - | - | - | - | - | - |
| 130 UniRef90\_A0A2V6N4R6\_10\_283 | - | - | - | - | - | - | - | - | - | - | - | - | - | - | - | - | - | - | - | - | - | - | - | - | - | - | - | - | - | - | - | - | - | - | - | - | - | - | - | - | - | - | - | - | - | - | - | - | - | - |
| 131 UniRef90\_A0A2V5IZ05\_29\_302 | - | - | - | - | - | - | - | - | - | - | - | - | - | - | - | - | - | - | - | - | - | - | - | - | - | - | - | - | - | - | - | - | - | - | - | - | - | - | - | - | - | - | - | - | - | - | - | - | - | - |
| 132 UniRef90\_A0A521E7V8\_39\_308 | - | - | - | - | - | - | - | - | - | - | - | - | - | - | - | - | - | - | - | - | - | - | - | - | - | - | - | - | - | - | - | - | - | - | - | - | - | - | - | - | - | - | - | - | - | - | - | - | - | - |
| 133 UniRef90\_UPI001665F59F\_18\_290 | - | - | - | - | - | - | - | - | - | - | - | - | - | - | - | - | - | - | - | - | - | - | - | - | - | - | - | - | - | - | - | - | - | - | - | - | - | - | - | - | - | - | - | - | - | - | - | - | - | - |
| 134 UniRef90\_A0A0Q9GGK2\_19\_284 | - | - | - | - | - | - | - | - | - | - | - | - | - | - | - | - | - | - | - | - | - | - | - | - | - | - | - | - | - | - | - | - | - | - | - | - | - | - | - | - | - | - | - | - | - | - | - | - | - | - |
| 135 UniRef90\_A0A329YBZ0\_37\_320 | - | - | - | - | - | - | - | - | - | - | - | - | - | - | - | - | - | - | - | - | - | - | - | - | - | - | - | - | - | - | - | - | - | - | - | - | - | - | - | - | - | - | - | - | - | - | - | - | - | - |
| 136 UniRef90\_A0A2E0H9P2\_13\_236 | - | - | - | - | - | - | - | - | - | - | - | - | - | - | - | - | - | - | - | - | - | - | - | - | - | - | - | - | - | - | - | - | - | - | - | - | - | - | - | - | - | - | - | - | - | - | - | - | - | - |
| 137 UniRef90\_A0A095SYG7\_44\_308 | - | - | - | - | - | - | - | - | - | - | - | - | - | - | - | - | - | - | - | - | - | - | - | - | - | - | - | - | - | - | - | - | - | - | - | - | - | - | - | - | - | - | - | - | - | - | - | - | - | - |
| 138 UniRef90\_A0A2U0ZNC3\_54\_322 | - | - | - | - | - | - | - | - | - | - | - | - | - | - | - | - | - | - | - | - | - | - | - | - | - | - | - | - | - | - | - | - | - | - | - | - | - | - | - | - | - | - | - | - | - | - | - | - | - | - |
| 139 UniRef90\_A0A2H2XXU1\_20\_287 | - | - | - | - | - | - | - | - | - | - | - | - | - | - | - | - | - | - | - | - | - | - | - | - | - | - | - | - | - | - | - | - | - | - | - | - | - | - | - | - | - | - | - | - | - | - | - | - | - | - |
| 140 UniRef90\_A0A0P4V0Y0\_11\_278 | - | - | - | - | - | - | - | - | - | - | - | - | - | - | - | - | - | - | - | - | - | - | - | - | - | - | - | - | - | - | - | - | - | - | - | - | - | - | - | - | - | - | - | - | - | - | - | - | - | - |
| 141 UniRef90\_A0A2D7FF16\_11\_288 | - | - | - | - | - | - | - | - | - | - | - | - | - | - | - | - | - | - | - | - | - | - | - | - | - | - | - | - | - | - | - | - | - | - | - | - | - | - | - | - | - | - | - | - | - | - | - | - | - | - |
| 142 UniRef90\_UPI0009FE04F9\_77\_354 | - | - | - | - | - | - | - | - | - | - | - | - | - | - | - | - | - | - | - | - | - | - | - | - | - | - | - | - | - | - | - | - | - | - | - | - | - | - | - | - | - | - | - | - | - | - | - | - | - | - |
| 143 UniRef90\_UPI00148816F0\_34\_297 | - | - | - | - | - | - | - | - | - | - | - | - | - | - | - | - | - | - | - | - | - | - | - | - | - | - | - | - | - | - | - | - | - | - | - | - | - | - | - | - | - | - | - | - | - | - | - | - | - | - |
| 144 UniRef90\_A0A2V5MXX8\_31\_304 | - | - | - | - | - | - | - | - | - | - | - | - | - | - | - | - | - | - | - | - | - | - | - | - | - | - | - | - | - | - | - | - | - | - | - | - | - | - | - | - | - | - | - | - | - | - | - | - | - | - |
| 145 UniRef90\_Q1W504\_15\_282 | - | - | - | - | - | - | - | - | - | - | - | - | - | - | - | - | - | - | - | - | - | - | - | - | - | - | - | - | - | - | - | - | - | - | - | - | - | - | - | - | - | - | - | - | - | - | - | - | - | - |
| 146 UniRef90\_UPI001661900D\_10\_259 | - | - | - | - | - | - | - | - | - | - | - | - | - | - | - | - | - | - | - | - | - | - | - | - | - | - | - | - | - | - | - | - | - | - | - | - | - | - | - | - | - | - | - | - | - | - | - | - | - | - |
| 147 UniRef90\_A0A2D6SJL3\_11\_235 | - | - | - | - | - | - | - | - | - | - | - | - | - | - | - | - | - | - | - | - | - | - | - | - | - | - | - | - | - | - | - | - | - | - | - | - | - | - | - | - | - | - | - | - | - | - | - | - | - | - |
| 148 UniRef90\_A0A2V6J615\_3\_225 | - | - | - | - | - | - | - | - | - | - | - | - | - | - | - | - | - | - | - | - | - | - | - | - | - | - | - | - | - | - | - | - | - | - | - | - | - | - | - | - | - | - | - | - | - | - | - | - | - | - |

  
  

|  |  |  |  |  |  |  |  |  |  |  |  |  |  |  |  |  |  |  |  |  |  |  |  |  |  |  |  |  |  |  |  |  |  |  |  |  |  |  |  |  |  |  |  |  |  |  |  |  |  |  |
| --- | --- | --- | --- | --- | --- | --- | --- | --- | --- | --- | --- | --- | --- | --- | --- | --- | --- | --- | --- | --- | --- | --- | --- | --- | --- | --- | --- | --- | --- | --- | --- | --- | --- | --- | --- | --- | --- | --- | --- | --- | --- | --- | --- | --- | --- | --- | --- | --- | --- | --- |
| **001 Input\_protein\_seq** | N | G | L | G | Q | Y | A | Y | A | K | G | - | - | - | - | L | S | S | K | F | I | E | S | - | E | G | V | K | L | H | Y | V | E | G | G | S | - | - | - | K | G | T | - | P | I | V | F | I | H | G |
| 002 UniRef90\_D8JE57\_1\_349 | N | G | L | G | Q | Y | A | Y | A | K | G | - | - | - | - | L | S | S | K | F | I | E | S | - | E | G | V | K | L | H | Y | V | E | G | G | S | - | - | - | K | G | T | - | P | I | V | F | I | H | G |
| 003 UniRef90\_A0A6B8MSH7\_6\_345 | E | K | L | G | Q | Y | A | Y | S | Q | G | - | - | - | - | L | E | S | K | F | I | E | T | - | D | G | V | R | L | H | Y | V | E | G | G | N | - | - | - | S | G | T | - | P | I | I | F | I | H | G |
| 004 UniRef90\_A0A6B2FNS7\_11\_352 | Q | K | R | G | Q | Y | A | V | A | K | G | - | - | - | - | L | T | S | K | F | I | D | V | - | N | G | Q | R | L | H | Y | V | E | G | G | N | - | - | - | S | G | T | - | P | I | I | F | I | H | G |
| 005 UniRef90\_UPI00073D803C\_6\_344 | E | Q | R | G | Q | Y | A | Y | G | E | G | - | - | - | - | L | K | S | Q | F | I | N | V | - | N | G | V | R | L | H | Y | V | E | G | G | N | - | - | - | S | G | T | - | P | I | V | F | I | H | G |
| 006 UniRef90\_A0A285K5K8\_3\_346 | E | Q | R | G | Q | Y | A | N | A | Q | G | - | - | - | - | L | T | S | K | F | I | E | V | - | N | G | V | R | L | H | Y | V | E | G | G | E | - | - | - | T | G | T | - | P | I | V | F | L | H | G |
| 007 UniRef90\_A0A379I9D6\_1\_346 | N | H | Q | G | Q | Y | Q | Y | A | A | G | - | - | - | - | L | S | S | K | F | V | E | V | - | D | G | V | R | I | H | Y | V | E | G | G | R | - | - | - | G | G | T | - | P | V | I | F | L | H | G |
| 008 UniRef90\_A0A6B2FTI5\_15\_352 | Q | K | R | G | Q | Y | A | V | A | Q | G | - | - | - | - | L | S | S | K | F | I | N | V | - | E | G | Q | R | L | H | Y | V | E | G | G | S | - | - | - | S | G | T | - | P | I | V | F | I | H | G |
| 009 UniRef90\_A0A2P9HD85\_2\_318 | E | K | R | G | Q | Y | A | V | P | E | G | - | - | - | - | F | E | S | G | M | V | E | T | - | D | G | I | R | L | H | Y | V | R | G | G | E | G | - | - | K | G | D | - | P | I | I | F | V | H | G |
| 010 UniRef90\_A0A1T1H7K2\_15\_316 | N | T | T | H | E | F | P | V | M | K | G | - | - | - | - | Y | S | S | Y | Y | D | D | I | - | D | G | V | K | L | H | Y | V | K | G | G | K | - | - | - | - | G | P | - | L | V | F | L | V | H | G |
| 011 UniRef90\_A0A1A9HKQ2\_27\_313 | - | - | - | - | - | F | P | L | P | A | G | - | - | - | - | F | T | S | E | Y | Q | T | V | - | D | G | V | K | L | H | Y | V | K | G | G | S | - | - | - | - | G | P | - | L | V | Y | L | V | H | G |
| 012 UniRef90\_A0A0J6GRA0\_22\_303 | - | Q | V | A | E | F | P | V | P | A | G | - | - | - | - | F | K | S | E | F | K | T | I | - | D | G | V | K | L | H | Y | V | K | G | G | S | - | - | - | - | G | P | - | L | V | Y | L | V | H | G |
| 013 UniRef90\_UPI000F5BD18A\_25\_314 | - | - | - | D | E | F | P | V | P | S | G | - | - | - | - | F | S | S | D | Y | Q | T | V | - | D | G | V | R | L | H | Y | V | K | G | G | S | - | - | - | - | G | P | - | L | V | Y | L | V | H | G |
| 014 UniRef90\_A0A4V1G750\_26\_311 | - | - | - | - | E | F | P | I | P | T | G | - | - | - | - | F | T | S | H | Y | Q | T | V | - | D | G | V | K | L | H | Y | V | K | G | G | S | - | - | - | - | G | P | - | L | V | F | L | V | H | G |
| 015 UniRef90\_U3U231\_21\_300 | N | A | A | D | E | F | P | L | P | T | G | - | - | - | - | F | T | S | S | Y | Q | T | V | - | D | G | V | R | L | H | Y | V | K | G | G | S | - | - | - | - | G | P | - | L | V | Y | L | V | H | G |
| 016 UniRef90\_F2LPZ2\_26\_313 | - | - | - | - | E | F | P | L | P | P | G | - | - | - | - | F | T | S | G | Y | Q | T | I | - | D | G | V | R | L | H | Y | V | K | G | G | S | - | - | - | - | G | P | - | L | V | Y | L | V | H | G |
| 017 UniRef90\_A0A3M4V4S1\_6\_330 | A | Q | Q | A | E | F | P | I | P | E | G | - | - | - | - | F | T | S | A | Y | E | T | V | - | D | G | V | K | L | H | F | V | R | G | G | K | - | - | - | - | G | P | - | L | V | L | L | V | H | G |
| 018 UniRef90\_A0A178LP42\_49\_332 | - | - | - | - | - | - | - | - | P | A | G | - | - | - | - | F | V | S | A | Y | Q | T | V | - | D | G | V | K | L | H | Y | V | K | G | G | N | - | - | - | - | G | P | - | L | V | L | L | V | H | G |
| 019 UniRef90\_T2L220\_20\_307 | - | - | - | - | E | F | P | I | P | D | G | - | - | - | - | F | T | S | G | Y | Q | T | I | - | D | G | V | K | L | H | Y | V | K | G | G | S | - | - | - | - | G | P | - | L | V | Y | L | V | H | G |
| 020 UniRef90\_UPI0014749FF6\_22\_304 | - | - | - | - | E | F | P | I | P | Q | G | - | - | - | - | F | K | S | G | F | E | E | V | - | N | G | I | K | M | H | Y | V | A | G | G | S | - | - | - | - | G | P | - | V | I | L | M | V | H | G |
| 021 UniRef90\_A0A172YJZ3\_18\_305 | - | - | - | - | E | F | P | L | P | E | G | - | - | - | - | F | T | S | G | Y | Q | T | I | - | D | G | V | K | L | H | Y | V | K | G | G | S | - | - | - | - | G | P | - | L | V | Y | L | V | H | G |
| 022 UniRef90\_UPI000737C6BF\_26\_313 | - | - | - | - | E | F | P | L | P | E | G | - | - | - | - | F | T | S | G | Y | R | T | I | - | D | G | V | D | L | H | Y | V | K | G | G | S | - | - | - | - | G | P | - | L | V | Y | L | V | H | G |
| 023 UniRef90\_A0A329J6I4\_26\_309 | - | - | - | - | E | F | P | V | P | K | G | - | - | - | - | F | T | S | E | F | R | T | V | - | D | G | V | K | L | H | Y | V | K | G | G | S | - | - | - | - | G | P | - | L | V | F | L | A | H | G |
| 024 UniRef90\_A0A2N3KZL1\_30\_325 | Q | P | E | S | E | F | P | V | P | A | G | - | - | - | - | F | E | S | S | Y | A | E | I | - | N | G | V | R | L | H | Y | V | K | G | G | S | - | - | - | - | G | P | - | L | A | F | L | V | H | G |
| 025 UniRef90\_UPI0006CA847F\_27\_323 | Q | A | N | S | E | F | P | I | P | A | G | - | - | - | - | F | E | S | S | F | K | E | I | - | N | G | V | K | L | H | Y | V | E | G | G | S | - | - | - | - | G | P | - | L | V | L | M | V | H | G |
| 026 UniRef90\_UPI00054E0E46\_26\_312 | - | - | - | - | E | F | P | V | P | A | G | - | - | - | - | F | T | S | E | Y | K | D | I | - | D | G | V | R | L | H | Y | V | K | G | G | Q | - | - | - | - | G | P | - | L | V | Y | L | V | H | G |
| 027 UniRef90\_A0A0S9PRE2\_29\_311 | - | - | - | - | - | - | - | - | - | A | G | - | - | - | - | F | T | P | G | F | Q | T | V | - | N | G | V | S | M | H | Y | L | K | G | G | Q | - | - | - | - | G | P | - | L | V | L | L | V | H | G |
| 028 UniRef90\_W0AA74\_37\_318 | - | - | - | - | - | - | - | - | - | - | G | - | - | - | - | F | T | P | A | Y | Q | T | V | - | D | G | V | K | L | H | Y | L | K | G | G | K | - | - | - | - | G | P | - | L | V | L | L | V | H | G |
| 029 UniRef90\_A0A2G0Y5Q3\_27\_312 | - | - | - | - | - | - | P | L | P | P | G | - | - | - | - | F | K | S | E | Y | K | E | V | - | D | G | V | K | M | H | Y | V | T | G | G | S | - | - | - | - | G | P | - | L | V | M | L | V | H | G |
| 030 UniRef90\_A0A1M6LZ92\_62\_349 | - | - | - | - | E | F | P | V | P | A | G | - | - | - | - | F | A | S | D | Y | Q | T | V | - | D | G | V | R | L | H | Y | V | K | G | G | S | - | - | - | - | G | P | - | L | V | Y | L | V | H | G |
| 031 UniRef90\_A0A4Z1C7D3\_28\_313 | - | - | - | - | - | - | P | V | P | A | G | - | - | - | - | F | E | S | E | F | A | E | I | - | D | G | V | T | L | H | Y | V | T | G | G | S | - | - | - | - | G | P | - | L | V | Y | L | V | H | G |
| 032 UniRef90\_A0A367MBG0\_273\_561 | - | - | - | - | E | F | P | V | P | N | G | - | - | - | - | F | E | S | A | Y | R | E | V | - | D | G | V | K | L | H | Y | V | K | G | G | Q | - | - | - | - | G | P | - | L | V | M | L | V | H | G |
| 033 UniRef90\_UPI00067CEA54\_35\_318 | - | - | - | - | - | - | - | - | P | E | G | - | - | - | - | F | T | P | A | Y | Q | T | V | - | D | G | V | K | I | H | Y | L | K | G | G | K | - | - | - | - | G | P | - | L | V | L | L | V | H | G |
| 034 UniRef90\_A0A1Y0L5M5\_31\_311 | - | - | - | - | - | F | P | V | P | Q | G | - | - | - | - | F | N | S | G | Y | E | T | I | - | D | G | I | R | L | H | Y | V | K | G | G | Q | - | - | - | - | G | P | - | L | V | Y | L | V | H | G |
| 035 UniRef90\_UPI000C2FE79C\_29\_313 | - | - | - | - | - | - | - | V | P | A | N | - | - | - | - | F | S | S | Q | Y | K | T | I | - | D | G | V | R | L | H | Y | V | K | G | G | S | - | - | - | - | G | P | - | L | V | Y | L | V | H | G |
| 036 UniRef90\_A0A4P8SJX7\_14\_295 | - | - | - | - | E | F | P | I | P | Q | G | - | - | - | - | F | S | S | E | V | K | T | V | - | D | G | I | K | L | H | Y | V | Q | G | G | K | - | - | - | - | G | P | - | L | V | Y | L | V | H | G |
| 037 UniRef90\_A0A1I1BZT3\_28\_315 | - | - | - | - | - | F | P | V | P | A | G | - | - | - | - | F | E | S | A | Y | S | E | I | - | D | S | V | K | L | H | Y | V | K | G | G | S | - | - | - | - | G | P | - | L | A | F | L | V | H | G |
| 038 UniRef90\_UPI000DF0D36E\_15\_290 | - | - | - | - | - | F | P | V | P | Q | G | - | - | - | - | F | N | S | E | Y | T | T | I | - | D | G | V | R | L | H | Y | V | K | G | G | Q | - | - | - | - | G | P | - | L | V | Y | L | V | H | G |
| 039 UniRef90\_UPI00136B8797\_37\_318 | - | - | - | - | - | - | - | - | - | - | G | - | - | - | - | F | T | P | A | Y | Q | T | V | - | D | G | V | K | L | H | Y | L | K | G | G | Q | - | - | - | - | G | P | - | M | V | L | L | V | H | G |
| 040 UniRef90\_A0A1H4CLJ1\_28\_313 | - | - | - | - | - | - | P | I | P | A | G | - | - | - | - | F | K | S | E | F | Q | I | V | - | D | G | V | K | I | H | Y | V | I | G | G | N | - | - | - | - | G | P | - | L | V | Y | L | V | H | G |
| 041 UniRef90\_UPI000E1FE28F\_29\_313 | - | - | - | - | - | - | - | T | P | T | G | - | - | - | - | F | T | S | A | Y | Q | T | V | - | D | G | V | R | L | H | Y | V | K | G | G | S | - | - | - | - | G | P | - | L | V | Y | L | V | H | G |
| 042 UniRef90\_A0A556AX95\_26\_313 | - | - | - | - | E | F | P | V | P | E | G | - | - | - | - | F | A | S | G | Y | Q | T | I | - | D | G | V | R | L | H | Y | V | K | G | G | S | - | - | - | - | G | P | - | L | V | Y | L | V | H | G |
| 043 UniRef90\_A0A6M8HNY7\_38\_322 | - | - | - | - | - | - | - | I | P | A | G | - | - | - | - | F | S | P | S | Y | R | E | V | - | D | G | I | R | L | H | Y | V | K | G | G | A | - | - | - | - | G | P | - | L | V | L | L | V | H | G |
| 044 UniRef90\_UPI001612B72A\_24\_301 | - | - | - | E | E | F | P | L | P | E | G | - | - | - | - | F | T | S | G | F | R | E | V | - | D | G | V | K | L | H | Y | V | Q | G | G | S | - | - | - | - | G | P | - | L | V | L | L | V | H | G |
| 045 UniRef90\_UPI0012F66D09\_46\_328 | - | - | - | - | - | - | - | - | - | A | G | - | - | - | - | F | T | P | G | Y | A | E | V | - | D | G | V | K | L | H | Y | L | K | G | G | A | - | - | - | - | G | P | - | L | V | L | L | V | H | G |
| 046 UniRef90\_UPI000A94940B\_29\_316 | - | - | - | - | E | F | P | L | P | A | G | - | - | - | - | F | S | S | G | Y | Q | T | I | - | D | G | V | K | L | H | Y | V | K | G | G | T | - | - | - | - | G | P | - | L | V | Y | L | V | H | G |
| 047 UniRef90\_UPI00102FACD2\_29\_312 | - | - | - | - | - | - | - | V | P | A | G | - | - | - | - | F | T | S | E | Y | K | V | I | - | D | G | V | R | M | H | Y | V | K | G | G | Q | - | - | - | - | G | P | - | L | V | Y | L | V | H | G |
| 048 UniRef90\_A0A1S8YNF4\_19\_297 | - | - | - | P | E | F | P | L | P | Q | G | - | - | - | - | F | S | S | E | Y | Q | T | V | - | D | G | I | N | M | H | Y | V | K | G | G | H | - | - | - | - | G | P | - | L | V | Y | L | V | H | G |
| 049 UniRef90\_UPI0015755320\_14\_301 | - | - | - | - | - | F | P | L | P | E | G | - | - | - | - | F | T | G | H | F | R | E | I | - | D | G | V | N | L | H | Y | V | A | G | G | Q | - | - | - | - | G | P | - | L | A | L | L | V | H | G |
| 050 UniRef90\_A0A427Q4N3\_22\_313 | H | A | A | D | E | F | P | V | P | K | G | - | - | - | - | F | T | S | H | Y | Q | T | V | - | D | G | V | K | L | H | Y | V | K | G | G | S | - | - | - | - | G | P | - | L | V | Y | L | V | H | G |
| 051 UniRef90\_A0A0A3Z1S4\_30\_318 | - | - | - | - | E | F | P | V | P | A | G | - | - | - | - | F | S | S | E | Y | K | R | V | - | D | G | V | K | L | H | Y | V | K | G | G | K | - | - | - | - | G | P | - | L | V | Y | L | V | H | G |
| 052 UniRef90\_A0A3L8C981\_35\_323 | - | - | - | - | - | F | P | I | P | K | G | - | - | - | - | F | T | G | K | Y | E | N | V | - | D | G | V | K | L | H | F | V | H | G | G | K | - | - | - | - | G | P | - | L | V | L | L | V | H | G |
| 053 UniRef90\_A0A6L5BLL6\_28\_324 | V | A | Q | E | E | F | P | I | P | D | G | - | - | - | - | F | T | G | G | Y | E | K | V | - | D | G | V | N | L | H | F | V | R | G | G | K | - | - | - | - | G | P | - | L | V | L | L | V | H | G |
| 054 UniRef90\_A0A6B1J288\_27\_314 | - | - | - | - | - | F | P | L | P | P | G | - | - | - | - | F | S | S | E | Y | Q | T | I | - | D | G | V | N | M | H | Y | V | K | G | G | A | - | - | - | - | G | P | - | L | V | Y | L | V | H | G |
| 055 UniRef90\_A0A263NNG9\_22\_274 | - | - | - | - | - | - | - | - | - | - | - | - | - | - | - | - | - | - | - | - | - | - | - | - | - | - | - | - | - | - | - | - | - | - | - | - | - | - | - | - | - | - | - | - | - | - | A | V | H | G |
| 056 UniRef90\_W0HXQ2\_33\_307 | - | - | - | - | - | - | - | - | - | A | G | - | - | - | - | F | T | S | A | Y | Q | T | I | - | N | H | V | R | L | H | Y | V | K | G | G | Q | - | - | - | - | G | P | - | L | V | L | L | V | H | G |
| 057 UniRef90\_A0A031HKS8\_40\_323 | - | - | - | - | - | - | - | - | P | A | G | - | - | - | - | F | T | P | G | Y | R | E | A | - | G | G | V | R | L | H | Y | L | K | G | G | K | - | - | - | - | G | P | - | L | V | L | L | V | H | G |
| 058 UniRef90\_A0A4R2FY05\_27\_320 | Q | A | D | S | E | F | P | I | P | S | G | - | - | - | - | F | E | S | S | F | K | E | V | - | N | G | V | K | L | H | Y | V | I | G | G | E | - | - | - | - | G | P | - | L | L | V | M | V | H | G |
| 059 UniRef90\_A0A1X0N2L9\_36\_321 | - | - | - | - | - | - | P | I | P | D | G | - | - | - | - | F | S | S | G | Y | E | N | V | - | D | G | V | E | L | H | F | V | R | G | G | R | - | - | - | - | G | P | - | L | V | M | L | V | H | G |
| 060 UniRef90\_A0A4R1U0K9\_37\_322 | - | - | - | - | - | - | - | I | P | D | G | - | - | - | - | F | S | G | G | Y | E | T | I | - | D | G | V | K | L | H | F | V | R | G | G | R | - | - | - | - | G | P | - | L | V | L | L | V | H | G |
| 061 UniRef90\_A0A0Q8TEQ0\_42\_323 | - | - | - | - | - | - | - | - | P | A | G | - | - | - | - | F | A | P | G | F | R | S | V | - | D | G | V | N | L | H | Y | L | K | G | G | K | - | - | - | - | G | K | - | L | V | L | L | V | H | G |
| 062 UniRef90\_A0A0Q6ZEJ0\_41\_323 | - | - | - | - | - | - | P | S | P | S | G | - | - | - | - | F | D | P | G | Y | K | E | V | - | D | G | V | S | L | H | Y | V | K | G | G | G | - | - | - | - | G | P | - | L | V | L | L | V | H | G |
| 063 UniRef90\_UPI0004C0CBB1\_54\_338 | - | - | - | - | - | - | - | - | P | P | G | - | - | - | - | F | R | S | K | Y | A | E | V | - | N | G | F | R | M | H | Y | M | Q | G | G | K | - | - | - | - | G | S | - | P | L | V | L | I | H | G |
| 064 UniRef90\_UPI0004CAB4F8\_56\_339 | - | - | - | - | - | - | - | - | P | P | G | - | - | - | - | F | Q | S | K | Y | A | E | V | - | N | G | F | R | M | H | Y | L | Q | G | G | K | - | - | - | - | G | S | - | P | V | V | L | I | H | G |
| 065 UniRef90\_UPI00049002CC\_60\_343 | - | - | - | - | - | - | - | - | - | A | G | - | - | - | - | F | Q | S | K | Y | A | E | V | - | N | G | F | R | M | H | Y | M | R | G | G | K | - | - | - | - | G | S | - | P | V | V | L | I | H | G |
| 066 UniRef90\_A0A561IX74\_59\_327 | - | - | - | - | - | - | - | - | - | - | - | - | - | - | - | - | - | - | A | Y | A | R | V | - | N | G | V | N | I | H | Y | V | A | G | G | S | - | - | - | - | G | E | - | P | L | V | L | I | H | G |
| 067 UniRef90\_A0A0X3RYG7\_67\_350 | - | - | - | - | - | - | - | - | - | A | G | - | - | - | - | F | R | S | K | F | A | E | V | - | N | G | F | R | M | H | Y | V | R | G | G | K | - | - | - | - | G | S | - | P | V | V | M | I | H | G |
| 068 UniRef90\_A0A2W2E1M5\_55\_338 | - | - | - | - | - | - | - | - | - | A | G | - | - | - | - | F | R | S | E | Y | A | K | V | - | N | G | F | R | M | H | Y | V | R | G | G | R | - | - | - | - | G | S | - | P | V | V | M | I | H | G |
| 069 UniRef90\_A0A2V7YB42\_28\_306 | - | - | - | - | - | Q | A | A | A | A | S | - | - | - | - | I | S | S | R | T | A | E | V | - | E | G | V | K | L | H | Y | L | T | A | G | R | - | - | - | - | G | A | - | P | V | I | L | L | H | G |
| 070 UniRef90\_A0A544XC49\_52\_336 | - | - | - | - | - | - | - | - | P | A | G | - | - | - | - | F | R | S | K | Y | A | E | V | - | N | G | F | R | M | H | Y | V | R | G | G | K | - | - | - | - | G | S | - | P | V | V | M | I | H | G |
| 071 UniRef90\_A0A101PQ90\_46\_324 | - | - | - | - | - | - | - | - | - | - | G | - | - | - | - | F | S | E | H | K | T | Q | V | - | A | G | T | G | I | N | Y | V | I | G | G | Q | - | - | - | - | G | P | - | T | L | V | L | I | H | G |
| 072 UniRef90\_A0A1C4VK67\_14\_288 | - | - | - | - | - | - | - | - | - | - | - | - | - | - | - | - | - | - | - | Q | A | R | V | - | R | D | V | T | L | N | Y | V | R | G | G | S | - | - | - | - | G | P | - | T | L | V | L | L | H | G |
| 073 UniRef90\_A0A0X3RPC2\_57\_340 | - | - | - | - | - | - | - | - | - | A | G | - | - | - | - | F | R | S | K | Y | A | E | V | - | N | G | F | R | M | H | Y | V | R | G | G | K | - | - | - | - | G | P | - | P | V | V | M | I | H | G |
| 074 UniRef90\_A0A4R2VWW4\_55\_319 | - | - | - | - | - | - | - | - | - | - | - | - | - | - | - | - | - | - | - | - | - | - | V | - | N | G | I | D | V | H | Y | V | I | G | G | S | - | - | - | - | G | E | - | P | L | V | L | I | H | G |
| 075 UniRef90\_A0A1Q7VB11\_36\_307 | - | - | - | - | - | - | - | - | - | - | - | - | - | - | - | F | V | S | R | V | A | E | V | - | E | G | V | K | L | H | Y | T | T | G | G | H | - | - | - | - | G | T | - | P | L | I | L | L | H | G |
| 076 UniRef90\_C7PGQ3\_38\_305 | - | - | - | - | - | - | - | - | - | - | - | - | - | - | - | - | - | - | - | Y | A | K | V | - | N | G | I | N | I | H | Y | V | I | G | G | S | - | - | - | - | G | E | - | P | L | V | L | L | H | G |
| 077 UniRef90\_A0A1N7R4E6\_62\_325 | - | - | - | - | - | - | - | - | - | - | - | - | - | - | - | - | - | - | - | - | - | - | - | - | N | G | I | A | I | H | Y | V | V | G | G | T | - | - | - | - | G | E | - | P | L | V | L | L | H | G |
| 078 UniRef90\_A0A2H2Y4V3\_41\_307 | - | - | - | - | - | - | - | - | - | - | - | - | - | - | - | - | - | - | - | - | - | D | V | - | N | G | V | R | L | H | Y | V | I | A | G | K | - | - | - | - | G | A | - | P | V | V | L | L | H | G |
| 079 UniRef90\_A0A448SCU6\_8\_276 | - | - | - | - | - | - | - | - | - | - | - | - | - | - | - | - | - | - | E | I | A | Q | V | - | N | G | I | R | L | H | Y | V | R | G | G | T | - | - | - | - | G | E | - | P | V | L | L | V | H | G |
| 080 UniRef90\_A0A365Y5E2\_33\_303 | - | - | - | - | - | - | - | - | - | - | - | - | - | - | - | - | A | S | K | V | M | T | V | E | N | G | L | T | I | H | Y | V | I | G | G | K | - | - | - | - | G | L | - | P | L | L | L | L | H | G |
| 081 UniRef90\_A0A1I2CFD2\_63\_327 | - | - | - | - | - | - | - | - | - | - | - | - | - | - | - | - | - | - | - | - | - | - | V | - | N | G | I | D | I | H | Y | V | V | G | G | E | - | - | - | - | G | E | - | P | L | V | L | L | H | G |
| 082 UniRef90\_A0A1G7Z850\_54\_322 | - | - | - | - | - | - | - | - | - | - | - | - | - | - | - | - | - | - | A | Y | A | K | V | - | N | G | I | D | I | H | Y | V | I | G | G | S | - | - | - | - | G | E | - | P | L | V | L | L | H | G |
| 083 UniRef90\_A0A2V9XG70\_35\_308 | - | - | - | - | - | - | - | - | - | - | T | - | - | - | - | I | S | S | R | D | A | E | V | - | D | G | V | K | L | H | Y | L | T | A | G | H | - | - | - | - | G | T | - | P | L | I | L | L | H | G |
| 084 UniRef90\_A0A0Q4GSS4\_39\_308 | - | - | - | - | - | - | - | - | - | - | - | - | - | - | - | - | - | - | Q | Y | A | S | V | - | N | G | V | K | L | H | Y | V | I | G | G | K | - | - | - | - | G | E | - | P | L | L | L | I | H | G |
| 085 UniRef90\_A0A0M9ZTU1\_1\_271 | - | - | - | - | - | - | - | - | - | - | - | - | - | - | - | - | - | - | - | - | - | - | - | - | - | - | - | - | M | H | Y | V | R | G | G | K | - | - | - | - | G | S | - | P | V | V | L | I | H | G |
| 086 UniRef90\_A0A239P597\_1\_270 | - | - | - | - | - | - | - | - | - | - | - | - | - | - | - | - | - | - | - | - | - | - | - | - | - | - | - | - | M | H | Y | V | R | G | G | K | - | - | - | - | G | P | - | P | V | V | M | I | H | G |
| 087 UniRef90\_UPI00128E3D53\_19\_294 | - | - | - | - | - | - | - | M | P | K | G | - | - | - | - | A | R | S | E | F | V | E | V | - | N | D | I | Q | L | H | Y | V | T | M | G | S | - | - | - | - | G | A | - | P | M | I | L | L | H | G |
| 088 UniRef90\_A0A1V9E1W0\_57\_321 | - | - | - | - | - | - | - | - | - | - | - | - | - | - | - | - | - | - | - | - | - | - | V | - | N | G | I | D | V | H | Y | V | I | G | G | S | - | - | - | - | G | E | - | P | L | V | L | L | H | G |
| 089 UniRef90\_A0A239MTD5\_1\_269 | - | - | - | - | - | - | - | - | - | - | - | - | - | - | - | - | - | - | - | - | - | - | - | - | - | - | - | - | M | H | Y | M | E | G | G | Q | - | - | - | - | G | S | - | P | V | V | M | I | H | G |
| 090 UniRef90\_UPI0009FCBBE8\_54\_317 | - | - | - | - | - | - | - | - | - | - | G | - | - | - | - | F | R | E | G | F | V | K | V | - | D | G | G | S | L | H | Y | V | R | G | G | S | - | - | - | - | G | P | - | A | L | V | L | L | H | G |
| 091 UniRef90\_A0A1A9HTL2\_19\_299 | - | - | - | - | - | - | - | - | - | - | - | - | - | - | - | - | - | - | - | - | - | - | V | - | N | G | V | K | L | H | Y | V | E | G | G | E | G | - | S | K | G | D | - | P | I | L | L | I | P | G |
| 092 UniRef90\_A0A4R7CM41\_23\_285 | - | - | - | - | - | - | - | - | - | - | - | - | - | - | - | - | - | - | - | - | - | - | - | - | N | G | V | D | I | H | Y | V | I | G | G | K | - | - | - | - | G | R | - | P | L | V | L | I | H | G |
| 093 UniRef90\_A0A562QYK6\_25\_307 | - | A | L | G | G | S | A | S | A | Q | D | - | - | - | - | I | A | S | K | F | A | E | A | - | N | G | V | R | L | H | Y | L | V | A | G | K | - | - | - | - | G | A | - | P | I | V | L | L | H | G |
| 094 UniRef90\_A0A2V9ZM06\_3\_269 | - | - | - | - | - | - | - | - | - | - | - | - | - | - | - | - | - | - | - | - | - | E | V | - | D | G | V | K | L | H | Y | M | T | A | G | H | - | - | - | - | G | T | - | P | L | I | L | L | H | G |
| 095 UniRef90\_A0A4P8YF40\_11\_271 | - | - | - | - | - | - | - | - | - | - | - | - | - | - | - | - | - | - | - | - | - | - | V | - | N | G | V | R | L | H | Y | V | R | G | G | A | - | - | - | - | G | E | - | P | V | L | L | V | H | G |
| 096 UniRef90\_A0A2V6E8I4\_30\_303 | - | - | - | - | - | - | - | - | - | K | N | - | - | - | - | I | V | S | C | T | A | E | V | - | D | G | V | Q | L | H | Y | L | T | A | G | H | - | - | - | - | G | P | - | T | V | I | L | L | H | G |
| 097 UniRef90\_A0A2V9BFT0\_23\_295 | - | - | - | - | - | - | - | - | - | - | Q | - | - | - | - | F | V | S | R | T | A | D | V | - | E | G | V | K | I | H | Y | T | T | G | G | H | - | - | - | - | G | P | - | T | V | I | L | L | H | G |
| 098 UniRef90\_A0A2V9H5C8\_29\_298 | - | - | - | - | - | - | - | - | - | - | - | - | - | - | - | - | - | S | H | T | T | E | I | - | D | G | L | K | L | H | Y | T | T | G | G | H | - | - | - | - | G | P | - | A | L | I | L | L | H | G |
| 099 UniRef90\_A0A101CRV3\_44\_308 | - | - | - | - | - | - | - | - | - | - | - | - | - | - | - | - | - | - | - | - | - | - | V | - | N | N | V | D | I | H | Y | V | I | G | G | K | - | - | - | - | G | E | - | P | L | V | L | V | H | G |
| 100 UniRef90\_A0A5M6CFP9\_57\_321 | - | - | - | - | - | - | - | - | - | - | - | - | - | - | - | - | - | - | - | - | - | - | V | - | N | G | V | N | I | H | Y | V | I | G | G | K | - | - | - | - | G | E | - | P | L | V | L | I | H | G |
| 101 UniRef90\_A0A401Z9D4\_19\_284 | - | - | - | - | - | - | - | - | - | - | - | - | - | - | - | - | - | - | - | - | - | Q | V | - | N | G | V | R | L | H | Y | V | I | G | G | Q | - | - | - | - | G | F | - | P | V | L | L | L | H | G |
| 102 UniRef90\_A0A2V8XJ58\_32\_308 | - | - | - | - | - | - | - | A | E | Q | A | - | - | - | - | F | V | S | R | S | A | E | I | - | D | G | V | K | L | R | Y | T | T | G | G | H | - | - | - | - | G | P | - | S | V | I | L | L | H | G |
| 103 UniRef90\_A0A252EMP1\_15\_277 | - | - | - | - | - | - | - | - | - | - | - | - | - | - | - | - | - | - | - | - | - | - | - | - | - | D | V | H | L | H | Y | V | R | A | G | K | - | - | - | - | G | E | - | P | V | L | L | V | H | G |
| 104 UniRef90\_A0A1H3H759\_34\_310 | - | - | - | - | - | - | - | - | - | - | - | - | - | - | - | F | R | S | R | W | M | Q | V | - | G | D | L | R | L | H | A | V | T | G | G | E | - | - | - | - | G | P | - | P | L | L | L | L | A | G |
| 105 UniRef90\_A0A4D7B7N1\_3\_270 | - | - | - | - | - | - | - | - | - | - | - | - | - | - | - | - | - | - | - | - | - | T | A | - | A | G | L | R | F | H | T | V | E | G | G | S | - | - | - | - | G | A | - | P | V | V | L | L | A | G |
| 106 UniRef90\_A0A1Q7MUT6\_34\_308 | - | - | - | - | - | - | - | - | - | N | A | - | - | - | - | I | V | S | R | D | A | E | V | - | E | G | V | K | L | H | Y | T | T | A | G | H | - | - | - | - | G | P | - | A | V | I | L | L | H | G |
| 107 UniRef90\_A0A2V6KQW0\_24\_297 | - | - | - | - | - | - | - | - | - | K | N | - | - | - | - | I | V | S | R | T | A | E | I | - | D | G | V | H | L | H | Y | L | T | A | G | H | - | - | - | - | G | P | - | A | V | I | L | L | H | G |
| 108 UniRef90\_A0A4R6LFZ5\_44\_308 | - | - | - | - | - | - | - | - | - | - | - | - | - | - | - | - | - | - | - | - | - | - | V | - | N | N | V | D | I | H | Y | V | I | G | G | K | - | - | - | - | G | E | - | P | L | V | L | V | H | G |
| 109 UniRef90\_A0A1H7UU25\_49\_318 | - | - | - | - | - | - | - | - | - | - | - | - | - | - | - | - | - | S | A | T | V | N | I | - | N | G | T | G | I | H | Y | V | I | G | G | S | - | - | - | - | G | E | - | P | L | L | L | I | H | G |
| 110 UniRef90\_UPI0008326796\_14\_259 | - | - | - | - | - | - | - | - | - | S | G | - | - | - | - | F | Q | H | Q | F | E | T | V | - | R | G | V | R | L | H | F | V | E | S | G | N | P | - | - | H | G | D | - | T | L | V | L | L | A | G |
| 111 UniRef90\_A0A2V5S8F2\_33\_306 | - | - | - | - | - | - | - | - | - | K | D | - | - | - | - | I | V | S | R | T | A | D | I | - | D | G | V | Q | L | H | Y | M | T | A | G | H | - | - | - | - | G | P | - | T | V | I | L | L | P | G |
| 112 UniRef90\_A0A3S3VTN8\_36\_308 | - | - | - | - | - | - | - | - | - | - | G | - | - | - | - | F | K | N | Q | Y | A | T | V | - | N | G | V | K | I | H | Y | V | I | G | G | N | - | - | - | - | G | E | - | P | L | L | L | V | H | G |
| 113 UniRef90\_A0A6J4PSU4\_16\_248 | - | - | - | - | - | - | - | - | - | - | - | - | - | - | - | - | - | - | - | - | - | R | A | - | N | G | V | R | M | H | Y | V | M | G | G | E | A | G | G | A | G | D | - | P | V | I | L | L | H | G |
| 114 UniRef90\_A0A534ZXC6\_26\_300 | - | - | - | - | - | - | - | - | - | A | T | - | - | - | - | V | S | S | R | S | A | D | V | - | A | G | V | T | L | H | Y | L | T | A | G | T | - | - | - | - | G | P | - | A | V | I | L | L | H | G |
| 115 UniRef90\_A0A2A9KBX9\_78\_358 | - | - | - | - | - | - | - | - | - | - | - | - | - | - | - | - | - | - | A | E | I | T | V | - | N | G | I | K | I | H | Y | V | D | G | G | T | - | - | - | - | G | A | - | P | I | L | L | I | P | G |
| 116 UniRef90\_A0A2W0BWH7\_16\_287 | - | - | - | - | - | - | - | - | - | - | - | - | - | - | - | - | A | S | R | A | A | D | V | - | D | G | V | S | I | H | Y | M | T | A | G | Q | - | - | - | - | G | P | - | A | V | V | L | L | H | G |
| 117 UniRef90\_A0A1R1RMU1\_30\_310 | - | - | - | - | - | - | - | - | - | - | G | - | - | - | - | F | K | H | Y | F | Q | T | V | - | D | G | V | R | L | H | Y | V | E | G | G | N | E | - | - | N | G | E | - | V | V | V | L | L | A | G |
| 118 UniRef90\_A0A3A1NH59\_38\_305 | - | - | - | - | - | - | - | - | - | - | - | - | - | - | - | - | - | - | - | - | - | N | V | - | N | G | I | N | L | H | Y | V | I | G | G | K | - | - | - | - | G | E | - | P | L | V | L | L | H | G |
| 119 UniRef90\_A0A0Q6WP84\_43\_317 | - | - | - | - | - | - | - | - | P | A | G | - | - | - | - | F | A | S | K | T | A | A | V | - | N | G | T | K | I | H | Y | L | V | G | G | K | - | - | - | - | G | T | - | P | V | V | L | L | H | G |
| 120 UniRef90\_A0A2V6P1A8\_15\_289 | - | - | - | - | - | - | - | - | V | K | T | - | - | - | - | I | V | S | H | D | V | K | T | - | E | S | V | Q | L | H | Y | L | T | A | G | H | - | - | - | - | G | P | - | A | V | I | L | L | H | G |
| 121 UniRef90\_A0A2V5NC55\_35\_307 | - | - | - | - | - | - | - | - | - | - | - | - | - | - | - | I | A | L | R | K | A | T | I | - | D | N | V | E | I | H | Y | L | T | T | G | Q | - | - | - | - | G | P | - | A | V | I | L | L | H | G |
| 122 UniRef90\_A0A2V9BM40\_39\_318 | - | - | - | G | A | D | A | A | A | N | V | - | - | - | - | I | A | S | R | T | A | E | V | - | E | G | I | K | L | H | Y | F | T | A | G | Q | - | - | - | - | G | P | - | A | V | I | L | L | H | G |
| 123 UniRef90\_A0A5B8WA72\_34\_302 | - | - | - | - | - | - | - | - | - | - | - | - | - | - | - | - | - | - | - | - | A | T | V | - | N | N | V | K | I | H | Y | V | I | G | G | K | - | - | - | - | G | E | - | P | L | L | L | I | H | G |
| 124 UniRef90\_A0A1Y6D2S0\_25\_299 | - | - | - | - | - | - | - | - | P | K | G | - | - | - | - | A | K | S | G | F | A | Q | V | - | N | G | I | Q | M | H | Y | V | K | M | G | K | - | - | - | - | G | P | - | L | L | L | L | L | H | G |
| 125 UniRef90\_A0A494W0L3\_12\_284 | - | - | - | - | - | - | - | - | - | - | G | - | - | - | - | F | K | H | Q | Y | A | T | V | - | N | G | V | K | I | H | Y | V | T | G | G | K | - | - | - | - | G | E | - | P | L | L | L | V | H | G |
| 126 UniRef90\_UPI0015CD0065\_39\_309 | - | - | - | - | - | - | R | L | A | P | G | - | - | - | - | F | S | H | G | K | V | S | V | - | T | G | G | T | L | H | Y | V | R | G | G | S | - | - | - | - | G | P | - | A | L | V | L | L | H | G |
| 127 UniRef90\_UPI00166E2F5D\_15\_289 | - | - | - | - | - | - | - | - | - | - | - | - | - | - | - | - | - | - | K | E | V | K | V | - | K | G | I | P | M | H | V | V | E | A | G | L | E | - | - | S | N | P | - | T | V | F | F | I | H | G |
| 128 UniRef90\_A0A372NPX1\_36\_305 | - | - | - | - | - | - | - | - | - | - | - | - | - | - | - | - | - | - | - | Y | A | T | V | - | H | G | V | K | L | H | Y | V | I | G | G | K | - | - | - | - | G | E | - | P | L | L | L | I | H | G |
| 129 UniRef90\_A0A317JA08\_13\_279 | - | - | - | - | - | - | - | - | - | - | - | - | - | - | - | - | - | S | R | V | A | R | I | - | E | G | K | K | L | H | Y | L | T | A | G | E | - | - | - | - | G | P | - | A | L | I | L | L | H | G |
| 130 UniRef90\_A0A2V6N4R6\_10\_283 | - | - | - | - | - | - | - | - | - | K | N | - | - | - | - | V | V | S | R | T | A | E | I | - | N | G | Q | Q | M | H | Y | L | Y | A | G | H | - | - | - | - | G | P | - | A | V | L | L | L | H | G |
| 131 UniRef90\_A0A2V5IZ05\_29\_302 | - | - | - | - | - | - | - | - | - | - | A | - | - | - | - | I | A | S | R | T | A | T | V | - | D | G | V | K | L | Y | Y | L | T | A | G | R | - | - | - | - | G | P | - | T | V | I | L | L | H | G |
| 132 UniRef90\_A0A521E7V8\_39\_308 | - | - | - | - | - | - | - | - | - | - | - | - | - | - | - | - | - | - | - | Y | A | T | V | - | N | G | V | K | I | H | Y | V | I | G | G | K | - | - | - | - | G | E | - | P | L | L | L | V | H | G |
| 133 UniRef90\_UPI001665F59F\_18\_290 | - | - | - | - | - | - | - | - | - | - | - | - | - | - | - | - | - | - | - | - | - | M | V | - | S | G | I | P | I | H | V | V | E | S | G | S | I | - | - | S | K | P | - | A | L | F | F | I | H | G |
| 134 UniRef90\_A0A0Q9GGK2\_19\_284 | - | - | - | - | - | - | - | - | - | - | - | - | - | - | - | - | - | - | - | - | - | - | - | - | - | N | I | N | L | H | Y | V | I | A | G | S | - | - | - | - | G | D | - | P | I | V | L | L | H | G |
| 135 UniRef90\_A0A329YBZ0\_37\_320 | - | - | - | - | - | - | G | L | P | Q | G | F | E | T | Q | F | S | H | H | Y | A | D | A | - | N | G | V | R | L | H | Y | V | M | G | G | A | A | - | - | N | G | P | - | L | V | V | L | L | H | G |
| 136 UniRef90\_A0A2E0H9P2\_13\_236 | - | - | - | - | - | - | - | - | - | - | - | - | - | - | - | - | - | - | - | - | - | D | L | - | G | D | V | M | L | H | Y | V | T | A | G | K | - | - | - | - | G | P | - | P | V | V | L | L | H | G |
| 137 UniRef90\_A0A095SYG7\_44\_308 | - | - | - | - | - | - | - | - | - | - | - | - | - | - | - | - | - | - | - | - | - | - | V | - | N | G | V | N | I | H | Y | V | I | G | G | K | - | - | - | - | G | E | - | P | L | V | L | V | H | G |
| 138 UniRef90\_A0A2U0ZNC3\_54\_322 | - | - | - | - | - | - | - | - | - | - | - | - | - | - | - | - | - | - | Q | V | A | E | V | - | N | G | V | K | I | H | Y | V | I | G | G | Q | - | - | - | - | G | E | - | P | L | V | L | I | H | G |
| 139 UniRef90\_A0A2H2XXU1\_20\_287 | - | - | - | - | - | - | - | - | - | - | - | - | - | - | - | - | - | - | - | - | - | - | V | - | N | G | V | Q | L | H | Y | V | M | G | G | K | - | - | - | - | G | A | - | P | V | V | L | L | H | G |
| 140 UniRef90\_A0A0P4V0Y0\_11\_278 | - | - | - | - | - | - | - | - | - | - | - | - | - | - | - | - | - | - | H | T | T | E | A | - | N | G | V | K | L | H | Y | V | I | G | G | S | - | - | - | - | G | E | - | P | I | L | L | W | H | G |
| 141 UniRef90\_A0A2D7FF16\_11\_288 | - | - | - | - | - | - | - | - | - | - | - | - | - | - | - | - | - | - | H | F | A | E | V | - | G | E | V | M | L | H | Y | V | T | A | G | S | - | - | - | - | G | P | - | P | I | V | L | L | H | G |
| 142 UniRef90\_UPI0009FE04F9\_77\_354 | - | - | - | - | - | - | - | - | - | - | - | - | - | - | - | - | - | - | C | F | T | T | V | - | D | G | V | Q | M | H | Y | V | I | G | G | S | - | - | - | - | G | P | Q | P | L | V | L | L | H | G |
| 143 UniRef90\_UPI00148816F0\_34\_297 | - | - | - | - | - | - | - | - | - | - | - | - | - | - | - | - | - | - | - | F | T | T | I | - | D | D | L | Q | M | H | Y | V | V | G | G | D | - | - | - | - | G | P | T | P | I | V | L | L | H | G |
| 144 UniRef90\_A0A2V5MXX8\_31\_304 | - | - | - | - | - | - | - | - | - | - | - | - | - | - | - | - | - | - | - | - | - | S | I | - | D | N | V | R | L | H | Y | L | T | A | G | H | - | - | - | - | G | P | - | A | V | I | L | L | H | G |
| 145 UniRef90\_Q1W504\_15\_282 | - | - | - | - | - | - | - | - | - | - | - | - | - | - | - | - | - | - | - | - | - | - | - | - | N | G | V | R | L | R | Y | S | I | S | G | S | - | - | - | - | G | E | - | P | L | V | L | L | H | G |
| 146 UniRef90\_UPI001661900D\_10\_259 | - | - | - | - | - | - | - | - | - | - | - | - | - | - | - | - | - | - | - | - | - | - | - | - | N | G | V | E | L | N | V | A | L | A | G | S | - | - | - | - | G | P | - | A | V | L | L | L | H | G |
| 147 UniRef90\_A0A2D6SJL3\_11\_235 | - | - | - | - | - | - | - | - | - | - | - | - | - | - | - | - | - | - | - | F | A | D | L | - | G | D | V | I | L | H | Y | V | X | X | G | X | - | - | - | - | G | P | - | P | V | V | L | L | H | G |
| 148 UniRef90\_A0A2V6J615\_3\_225 | - | - | - | - | - | - | - | - | - | - | - | - | - | - | - | - | - | - | - | - | - | - | - | - | - | - | - | - | - | - | - | - | - | - | - | - | - | - | - | - | - | - | - | - | - | - | - | - | - | - |

  
  

|  |  |  |  |  |  |  |  |  |  |  |  |  |  |  |  |  |  |  |  |  |  |  |  |  |  |  |  |  |  |  |  |  |  |  |  |  |  |  |  |  |  |  |  |  |  |  |  |  |  |  |
| --- | --- | --- | --- | --- | --- | --- | --- | --- | --- | --- | --- | --- | --- | --- | --- | --- | --- | --- | --- | --- | --- | --- | --- | --- | --- | --- | --- | --- | --- | --- | --- | --- | --- | --- | --- | --- | --- | --- | --- | --- | --- | --- | --- | --- | --- | --- | --- | --- | --- | --- |
| **001 Input\_protein\_seq** | F | G | S | T | W | K | M | W | E | P | V | M | L | S | Y | - | M | K | D | - | H | K | V | I | A | I | D | L | P | G | L | G | Q | S | G | P | I | L | N | D | - | - | - | - | - | - | - | D | Y | S |
| 002 UniRef90\_D8JE57\_1\_349 | F | G | S | T | W | K | M | W | E | P | V | M | L | S | Y | - | M | K | D | - | H | K | V | I | A | I | D | L | P | G | L | G | Q | S | S | P | I | L | N | D | - | - | - | - | - | - | - | D | Y | S |
| 003 UniRef90\_A0A6B8MSH7\_6\_345 | F | G | S | T | W | K | M | W | E | P | V | M | E | K | F | - | K | A | T | - | H | K | V | I | A | I | D | L | P | G | L | G | Q | S | S | P | I | K | N | E | - | - | - | - | - | - | - | D | Y | S |
| 004 UniRef90\_A0A6B2FNS7\_11\_352 | F | G | S | T | W | K | M | W | E | P | V | M | E | K | F | - | K | A | N | - | N | K | V | I | A | I | D | L | P | G | L | G | Q | S | S | P | I | A | N | G | - | - | - | - | - | - | - | D | Y | S |
| 005 UniRef90\_UPI00073D803C\_6\_344 | F | G | S | T | W | K | M | W | E | P | I | M | E | K | F | - | K | A | S | - | N | K | V | I | A | I | D | L | P | G | L | G | Q | S | S | A | V | A | N | D | - | - | - | - | - | - | - | D | Y | S |
| 006 UniRef90\_A0A285K5K8\_3\_346 | F | G | S | T | W | K | M | W | E | P | V | M | E | K | F | - | K | A | N | - | H | K | V | I | A | I | D | L | P | G | L | G | Q | S | A | A | I | P | D | G | - | - | - | - | - | - | - | D | Y | S |
| 007 UniRef90\_A0A379I9D6\_1\_346 | F | G | S | T | W | K | M | W | E | P | V | M | Q | V | F | - | A | K | K | - | R | T | V | I | A | V | D | L | P | G | L | G | Q | S | G | P | I | K | G | D | - | - | - | - | - | - | - | D | Y | S |
| 008 UniRef90\_A0A6B2FTI5\_15\_352 | F | G | S | T | W | K | M | W | E | P | V | M | E | K | F | - | K | A | N | - | N | K | V | I | A | I | D | L | P | G | L | G | Q | S | A | P | I | A | N | D | - | - | - | - | - | - | - | D | Y | S |
| 009 UniRef90\_A0A2P9HD85\_2\_318 | F | G | S | T | W | K | M | W | Q | P | A | L | E | K | F | - | S | A | N | - | H | Q | V | I | A | I | D | L | P | G | L | G | Q | S | E | P | S | A | K | - | - | - | - | - | - | - | - | R | Y | D |
| 010 UniRef90\_A0A1T1H7K2\_15\_316 | F | G | Q | S | W | Y | E | W | H | Q | L | M | P | T | L | - | E | K | N | - | Y | T | V | V | A | V | D | L | P | G | L | G | L | S | Q | P | P | - | K | - | - | - | - | - | - | - | - | S | F | K |
| 011 UniRef90\_A0A1A9HKQ2\_27\_313 | F | G | Q | S | W | Y | E | W | H | Q | L | M | P | R | L | - | A | E | K | - | F | T | V | V | A | P | D | L | P | G | L | G | Q | S | A | V | P | - | P | - | - | - | - | - | - | - | - | S | F | R |
| 012 UniRef90\_A0A0J6GRA0\_22\_303 | F | G | Q | A | W | Y | E | W | H | S | L | M | P | E | L | - | A | K | T | - | H | T | V | V | A | V | D | L | P | G | L | G | E | S | E | P | P | K | T | - | - | - | - | - | - | - | - | S | Y | T |
| 013 UniRef90\_UPI000F5BD18A\_25\_314 | F | G | Q | T | W | Y | E | W | H | D | L | M | P | E | L | - | A | K | N | - | H | T | V | V | A | V | D | L | P | G | L | G | Q | S | E | E | P | - | K | - | - | - | - | - | - | - | - | S | Y | R |
| 014 UniRef90\_A0A4V1G750\_26\_311 | F | G | Q | T | W | Y | E | W | H | Q | L | M | P | L | L | - | A | Q | H | - | Y | T | V | V | A | P | D | L | P | G | L | G | Q | S | A | P | A | - | P | - | - | - | - | - | - | - | - | S | L | R |
| 015 UniRef90\_U3U231\_21\_300 | F | G | Q | T | W | Y | E | W | H | Q | L | M | P | E | L | - | A | K | N | - | H | T | V | V | A | V | D | L | P | G | L | G | Q | S | D | L | P | - | H | - | - | - | - | - | - | - | - | S | Y | R |
| 016 UniRef90\_F2LPZ2\_26\_313 | F | G | Q | T | W | Y | E | W | H | Q | L | M | P | E | L | - | A | R | N | - | H | T | V | V | A | P | D | L | P | G | L | G | Q | S | A | E | P | - | G | - | - | - | - | - | - | - | - | S | Y | R |
| 017 UniRef90\_A0A3M4V4S1\_6\_330 | F | G | Q | T | W | Y | E | W | N | S | L | M | P | Q | L | - | A | E | R | - | Y | T | V | V | A | V | D | L | P | G | L | G | L | S | A | P | P | K | T | - | - | - | - | - | - | - | - | T | Y | T |
| 018 UniRef90\_A0A178LP42\_49\_332 | F | A | Q | T | W | Y | E | W | N | Q | L | M | P | L | L | - | A | N | T | - | H | T | V | V | A | I | D | L | P | G | L | G | Q | S | D | P | P | - | K | - | - | - | - | - | - | - | - | S | Y | V |
| 019 UniRef90\_T2L220\_20\_307 | F | G | Q | T | W | Y | E | W | H | Q | L | M | P | A | L | - | A | K | S | - | F | T | V | V | A | P | D | L | P | G | L | G | Q | S | A | V | P | - | P | - | - | - | - | - | - | - | - | S | Y | R |
| 020 UniRef90\_UPI0014749FF6\_22\_304 | F | A | Q | S | W | Y | E | W | N | D | L | M | P | Q | L | - | A | K | T | - | H | T | V | I | A | V | D | L | P | G | L | G | L | S | E | P | L | K | T | - | - | - | - | - | - | - | - | T | Y | T |
| 021 UniRef90\_A0A172YJZ3\_18\_305 | F | G | Q | T | W | Y | E | W | H | Q | L | M | P | E | L | - | A | K | R | - | F | T | V | V | A | P | D | L | P | G | L | G | Q | S | A | V | P | - | P | - | - | - | - | - | - | - | - | S | Y | R |
| 022 UniRef90\_UPI000737C6BF\_26\_313 | F | G | Q | T | W | Y | E | W | H | Q | L | M | P | A | L | - | A | E | R | - | F | T | V | V | A | P | D | L | P | G | L | G | Q | S | S | V | P | - | P | - | - | - | - | - | - | - | - | S | Y | R |
| 023 UniRef90\_A0A329J6I4\_26\_309 | F | G | Q | A | W | Y | E | W | H | N | L | M | P | E | L | - | A | R | N | - | H | T | V | V | A | V | D | L | P | G | L | G | E | S | Q | P | P | K | T | - | - | - | - | - | - | - | - | G | Y | S |
| 024 UniRef90\_A0A2N3KZL1\_30\_325 | F | G | Q | S | W | Y | E | W | H | Q | L | M | P | L | L | - | A | K | T | - | H | S | V | V | A | V | D | L | P | G | L | G | Q | S | A | V | P | - | K | - | - | - | - | - | - | - | - | S | Y | V |
| 025 UniRef90\_UPI0006CA847F\_27\_323 | Y | G | Q | T | W | Y | E | W | H | Q | L | M | P | K | L | - | A | K | D | - | H | R | V | V | A | V | D | M | P | G | L | G | Q | S | E | P | L | D | S | - | - | - | - | - | - | - | - | T | Y | S |
| 026 UniRef90\_UPI00054E0E46\_26\_312 | F | G | Q | S | W | Y | E | W | H | R | L | M | P | E | L | - | A | K | S | - | H | T | V | V | A | P | D | L | P | G | L | G | E | S | D | V | P | - | P | - | - | - | - | - | - | - | - | S | Y | R |
| 027 UniRef90\_A0A0S9PRE2\_29\_311 | F | G | Q | T | W | Y | E | W | H | R | L | M | P | E | L | - | A | K | D | - | H | T | V | V | A | V | E | L | P | G | L | G | Q | S | G | P | A | - | K | - | - | - | - | - | - | - | - | S | Y | A |
| 028 UniRef90\_W0AA74\_37\_318 | F | A | Q | T | W | Y | E | W | T | Q | I | M | P | L | L | - | A | K | N | - | Y | T | V | V | A | I | D | L | P | G | L | G | Q | S | E | A | P | - | K | - | - | - | - | - | - | - | - | S | Y | L |
| 029 UniRef90\_A0A2G0Y5Q3\_27\_312 | F | G | Q | T | W | Y | E | W | H | Q | L | M | P | E | L | - | A | R | N | - | F | T | V | V | A | P | D | L | P | G | L | G | Q | S | E | P | P | - | K | - | - | - | - | - | - | - | - | A | Y | S |
| 030 UniRef90\_A0A1M6LZ92\_62\_349 | F | G | Q | S | W | Y | E | W | H | Q | L | M | P | V | L | - | A | K | D | - | H | T | V | V | A | V | D | L | P | G | L | G | Q | S | G | V | P | - | A | - | - | - | - | - | - | - | - | S | Y | R |
| 031 UniRef90\_A0A4Z1C7D3\_28\_313 | F | G | Q | S | W | Y | E | W | H | Q | L | M | P | E | L | - | A | K | T | - | H | M | V | V | A | V | D | L | P | G | L | G | L | S | S | Q | P | - | P | - | - | - | - | - | - | - | - | S | Y | A |
| 032 UniRef90\_A0A367MBG0\_273\_561 | F | G | Q | T | W | Y | E | W | H | Q | L | M | P | E | L | - | A | K | R | - | F | T | V | I | A | P | D | L | P | G | L | G | Q | S | E | P | P | K | T | - | - | - | - | - | - | - | - | G | Y | S |
| 033 UniRef90\_UPI00067CEA54\_35\_318 | F | A | Q | T | W | Y | E | W | N | E | L | M | P | L | L | - | A | K | N | - | H | T | V | V | A | I | D | L | P | G | L | G | Q | S | D | A | P | - | K | - | - | - | - | - | - | - | - | S | Y | A |
| 034 UniRef90\_A0A1Y0L5M5\_31\_311 | F | G | Q | S | W | F | E | W | H | Q | I | M | P | E | L | - | A | K | T | - | H | T | V | V | A | V | D | L | P | G | L | G | L | S | Q | P | P | A | S | - | - | - | - | - | - | - | - | S | Y | T |
| 035 UniRef90\_UPI000C2FE79C\_29\_313 | F | G | Q | T | W | Y | E | W | H | Q | L | M | P | E | L | - | A | K | N | - | H | T | V | V | A | V | D | L | P | G | L | G | Q | S | A | V | P | - | K | - | - | - | - | - | - | - | - | S | Y | R |
| 036 UniRef90\_A0A4P8SJX7\_14\_295 | F | G | Q | S | W | Y | E | W | H | Q | L | M | P | E | L | - | A | K | N | - | H | T | V | V | A | V | D | L | P | G | L | G | L | S | Q | P | P | A | T | - | - | - | - | - | - | - | - | S | Y | S |
| 037 UniRef90\_A0A1I1BZT3\_28\_315 | F | G | Q | S | W | Y | E | W | H | Q | L | M | P | E | L | - | A | K | T | - | H | T | V | V | A | V | D | L | P | G | L | G | Q | S | T | P | P | - | K | - | - | - | - | - | - | - | - | S | Y | A |
| 038 UniRef90\_UPI000DF0D36E\_15\_290 | F | G | Q | S | W | Y | E | W | H | Q | L | M | P | E | L | - | A | K | T | - | H | T | V | V | A | V | D | L | P | G | L | G | L | S | Q | P | P | A | S | - | - | - | - | - | - | - | - | S | Y | T |
| 039 UniRef90\_UPI00136B8797\_37\_318 | F | A | Q | T | W | Y | E | W | H | Q | I | M | P | L | L | - | A | K | T | - | H | T | V | V | A | I | D | L | P | G | L | G | Q | S | E | A | P | - | K | - | - | - | - | - | - | - | - | S | Y | A |
| 040 UniRef90\_A0A1H4CLJ1\_28\_313 | F | G | Q | S | W | Y | E | W | H | Q | L | M | P | E | L | - | S | K | K | - | Y | T | V | V | A | P | D | L | P | G | L | G | Q | S | Q | I | T | - | T | - | - | - | - | - | - | - | - | S | Y | R |
| 041 UniRef90\_UPI000E1FE28F\_29\_313 | F | G | Q | T | W | Y | E | W | H | Q | L | M | P | L | L | - | A | K | H | - | H | T | V | V | A | V | D | L | P | G | L | G | Q | S | A | V | P | - | P | - | - | - | - | - | - | - | - | S | Y | R |
| 042 UniRef90\_A0A556AX95\_26\_313 | F | G | Q | S | W | Y | E | W | H | Q | L | M | P | E | L | - | A | K | Q | - | F | T | V | V | A | P | D | L | P | G | L | G | Q | S | A | V | P | - | P | - | - | - | - | - | - | - | - | S | Y | R |
| 043 UniRef90\_A0A6M8HNY7\_38\_322 | F | G | Q | T | W | Y | E | W | H | Q | L | M | P | L | L | - | A | K | N | - | H | S | V | I | A | V | D | L | P | A | L | G | L | S | D | A | P | - | R | - | - | - | - | - | - | - | - | S | Y | A |
| 044 UniRef90\_UPI001612B72A\_24\_301 | F | G | Q | A | W | Y | E | W | R | Q | L | M | P | L | L | - | A | K | T | - | H | A | V | V | A | V | D | L | P | A | L | G | L | S | E | P | P | - | K | - | - | - | - | - | - | - | - | S | Y | A |
| 045 UniRef90\_UPI0012F66D09\_46\_328 | F | G | Q | S | W | Y | E | W | H | Q | L | M | P | L | L | - | A | K | T | - | H | T | V | V | A | V | D | L | P | A | L | G | L | S | G | V | P | - | K | - | - | - | - | - | - | - | - | S | Y | A |
| 046 UniRef90\_UPI000A94940B\_29\_316 | F | G | Q | T | W | Y | E | W | H | Q | L | M | P | E | L | - | A | K | H | - | Y | T | V | V | A | P | D | L | P | G | L | G | Q | S | A | V | P | - | T | - | - | - | - | - | - | - | - | S | Y | R |
| 047 UniRef90\_UPI00102FACD2\_29\_312 | F | G | Q | T | W | Y | E | W | H | H | L | M | P | E | L | - | A | R | N | - | H | T | V | V | A | P | D | L | P | G | L | G | D | S | E | V | P | - | P | - | - | - | - | - | - | - | - | S | Y | R |
| 048 UniRef90\_A0A1S8YNF4\_19\_297 | F | G | Q | A | W | Y | E | W | H | Q | L | M | P | Q | L | - | A | T | N | - | H | T | V | V | A | V | D | L | P | G | L | G | M | S | Q | P | P | L | T | - | - | - | - | - | - | - | - | G | Y | S |
| 049 UniRef90\_UPI0015755320\_14\_301 | F | G | Q | T | W | Y | E | W | H | Q | L | M | P | L | L | - | A | R | Q | - | F | T | V | V | A | P | D | L | P | G | L | G | L | S | A | P | P | A | T | - | - | - | - | - | - | - | - | S | Y | T |
| 050 UniRef90\_A0A427Q4N3\_22\_313 | F | G | Q | T | W | Y | E | W | H | Q | L | M | P | E | L | - | A | R | H | - | F | T | V | V | A | P | D | L | P | G | L | G | Q | S | Q | T | P | - | P | - | - | - | - | - | - | - | - | S | Y | R |
| 051 UniRef90\_A0A0A3Z1S4\_30\_318 | F | G | Q | S | W | Y | E | W | H | Q | L | M | P | E | L | - | A | A | R | - | H | T | V | V | A | V | D | L | P | G | L | G | L | S | Q | P | P | A | T | - | - | - | - | - | - | - | - | S | Y | S |
| 052 UniRef90\_A0A3L8C981\_35\_323 | F | G | Q | T | W | Y | E | W | N | E | L | M | Q | Q | L | - | A | E | R | - | Y | T | V | V | A | V | D | L | P | G | L | G | L | S | Q | A | P | Q | T | - | - | - | - | - | - | - | - | S | Y | T |
| 053 UniRef90\_A0A6L5BLL6\_28\_324 | F | G | Q | T | W | Y | E | W | N | R | L | M | P | Q | L | - | A | E | H | - | Y | T | V | V | A | V | D | L | P | G | L | G | L | S | D | P | P | Q | T | - | - | - | - | - | - | - | - | S | Y | T |
| 054 UniRef90\_A0A6B1J288\_27\_314 | F | G | Q | T | W | Y | E | W | H | Q | L | M | P | E | L | - | A | R | K | - | Y | T | V | V | A | P | D | L | P | G | L | G | Q | S | S | V | P | - | A | - | - | - | - | - | - | - | - | S | Y | R |
| 055 UniRef90\_A0A263NNG9\_22\_274 | F | G | Q | S | W | Y | E | W | H | Q | L | M | R | E | L | - | A | K | K | - | Y | R | V | I | A | V | D | L | P | G | L | G | M | S | E | P | L | K | T | - | - | - | - | - | - | - | - | S | Y | S |
| 056 UniRef90\_W0HXQ2\_33\_307 | F | G | Q | S | W | Y | E | W | R | T | L | M | P | L | L | - | A | A | Q | - | H | T | V | I | A | V | D | L | P | G | L | G | D | S | A | P | P | V | S | - | - | - | - | - | - | - | - | G | Y | S |
| 057 UniRef90\_A0A031HKS8\_40\_323 | F | G | Q | S | W | Y | E | W | H | Q | L | M | P | M | L | - | A | R | T | - | H | T | V | V | A | V | D | L | P | A | L | G | L | S | G | A | P | - | K | - | - | - | - | - | - | - | - | S | Y | A |
| 058 UniRef90\_A0A4R2FY05\_27\_320 | F | A | Q | S | W | Y | E | W | H | Q | L | M | P | K | L | - | A | E | D | - | Y | Q | V | I | A | M | D | L | P | G | L | G | L | S | E | P | L | D | S | - | - | - | - | - | - | - | - | T | Y | T |
| 059 UniRef90\_A0A1X0N2L9\_36\_321 | F | G | Q | T | W | Y | E | W | N | S | L | M | S | Q | L | - | A | E | R | - | Y | T | V | L | A | V | D | L | P | G | L | G | L | S | G | P | A | Q | - | - | - | - | - | - | - | - | - | T | F | T |
| 060 UniRef90\_A0A4R1U0K9\_37\_322 | F | G | Q | T | W | Y | E | W | N | R | L | M | P | K | L | - | A | G | R | - | Y | T | V | V | A | V | E | L | P | G | L | G | L | S | E | A | P | R | T | - | - | - | - | - | - | - | - | S | Y | T |
| 061 UniRef90\_A0A0Q8TEQ0\_42\_323 | Y | G | Q | T | W | Y | E | W | R | Y | L | M | P | E | L | - | A | K | S | - | H | T | V | V | A | V | D | L | P | A | L | G | Q | S | G | S | T | - | K | - | - | - | - | - | - | - | - | S | Y | A |
| 062 UniRef90\_A0A0Q6ZEJ0\_41\_323 | Y | G | Q | T | W | Y | E | W | R | Y | L | M | P | E | L | - | A | K | T | - | H | T | V | I | A | V | D | L | P | A | L | G | Q | S | G | T | T | - | M | - | - | - | - | - | - | - | - | S | Y | A |
| 063 UniRef90\_UPI0004C0CBB1\_54\_338 | F | P | Q | T | W | A | E | W | R | N | Q | M | G | P | L | - | S | K | N | - | H | T | V | I | A | V | D | L | R | G | M | G | N | S | E | V | T | K | S | - | - | - | - | - | - | - | - | G | Y | Q |
| 064 UniRef90\_UPI0004CAB4F8\_56\_339 | F | P | Q | T | W | A | E | W | R | H | Q | M | G | P | L | - | S | K | T | - | H | T | V | I | A | V | D | L | R | G | A | G | N | S | E | V | T | K | S | - | - | - | - | - | - | - | - | G | Y | E |
| 065 UniRef90\_UPI00049002CC\_60\_343 | F | P | Q | T | W | A | E | W | R | Q | Q | M | G | P | L | - | S | K | T | - | H | T | V | I | A | V | D | L | R | G | T | G | N | S | Q | V | T | K | S | - | - | - | - | - | - | - | - | G | Y | Q |
| 066 UniRef90\_A0A561IX74\_59\_327 | F | G | Q | N | W | Y | M | W | N | R | L | L | P | E | L | - | S | K | H | - | F | T | V | I | A | P | D | L | P | G | L | G | E | S | D | K | P | D | S | - | - | - | - | - | - | - | - | G | Y | D |
| 067 UniRef90\_A0A0X3RYG7\_67\_350 | F | P | E | D | W | S | E | W | R | K | E | M | V | P | L | - | S | K | N | - | H | T | V | I | A | V | D | L | R | G | A | G | Q | S | Q | V | T | K | S | - | - | - | - | - | - | - | - | G | Y | D |
| 068 UniRef90\_A0A2W2E1M5\_55\_338 | F | P | Q | N | W | S | E | W | R | Q | E | M | V | P | L | - | S | K | T | - | H | T | V | I | A | V | D | L | R | G | A | G | E | S | E | V | T | K | S | - | - | - | - | - | - | - | - | G | Y | Q |
| 069 UniRef90\_A0A2V7YB42\_28\_306 | Y | A | E | T | S | R | M | W | E | P | I | L | P | R | L | - | A | E | R | - | F | T | V | I | A | P | D | L | P | G | I | G | D | S | A | I | P | A | D | - | - | - | - | - | - | - | - | G | L | D |
| 070 UniRef90\_A0A544XC49\_52\_336 | F | P | E | D | W | S | E | W | R | Q | E | M | G | P | L | - | S | K | T | - | H | T | V | I | A | V | D | L | R | G | A | G | E | S | Q | V | T | E | S | - | - | - | - | - | - | - | - | G | Y | Q |
| 071 UniRef90\_A0A101PQ90\_46\_324 | Y | P | Q | T | W | Y | E | W | H | G | I | M | P | A | L | - | A | K | H | - | Y | T | V | I | A | P | D | L | P | G | A | G | K | S | D | A | P | A | S | - | - | - | - | - | - | - | - | G | Y | D |
| 072 UniRef90\_A0A1C4VK67\_14\_288 | Y | P | Q | C | W | H | M | W | R | P | V | L | P | E | L | - | A | R | H | - | F | E | V | I | A | P | D | L | R | G | F | G | G | S | D | A | P | E | G | - | - | - | - | - | - | - | - | G | Y | D |
| 073 UniRef90\_A0A0X3RPC2\_57\_340 | F | P | Q | S | W | T | E | W | R | Q | Q | M | V | P | L | - | S | K | T | - | H | T | V | I | A | V | D | L | R | G | T | G | N | S | Q | V | T | K | S | - | - | - | - | - | - | - | - | G | Y | D |
| 074 UniRef90\_A0A4R2VWW4\_55\_319 | F | G | Q | N | W | Y | M | W | N | R | L | L | P | E | F | - | S | K | H | - | F | T | V | I | A | P | D | L | P | G | L | G | E | S | G | K | P | D | S | - | - | - | - | - | - | - | - | G | Y | D |
| 075 UniRef90\_A0A1Q7VB11\_36\_307 | Y | A | E | T | S | R | M | W | T | P | I | L | P | V | L | - | G | E | K | - | F | T | V | I | A | P | D | L | P | G | I | G | E | S | S | I | P | A | D | - | - | - | - | - | - | - | - | G | L | D |
| 076 UniRef90\_C7PGQ3\_38\_305 | F | G | Q | N | W | Y | M | W | N | R | L | L | P | E | L | - | S | K | H | - | F | T | V | I | A | P | D | L | P | G | L | G | E | S | G | K | P | A | G | - | - | - | - | - | - | - | - | G | Y | D |
| 077 UniRef90\_A0A1N7R4E6\_62\_325 | F | G | Q | N | W | Y | M | W | N | R | L | L | P | E | L | - | S | K | H | - | F | T | V | I | A | P | D | L | P | G | L | G | E | S | G | K | P | D | S | - | - | - | - | - | - | - | - | G | Y | D |
| 078 UniRef90\_A0A2H2Y4V3\_41\_307 | F | P | Q | T | W | Y | E | W | R | K | V | M | P | A | L | - | A | Q | R | - | Y | T | V | I | A | P | D | L | R | G | L | G | K | S | S | K | P | E | N | - | - | - | - | - | - | - | - | G | Y | D |
| 079 UniRef90\_A0A448SCU6\_8\_276 | W | P | Q | T | W | Y | E | W | R | Q | V | I | P | R | L | I | A | A | G | - | H | E | V | I | A | V | D | M | R | G | S | G | D | S | D | K | P | A | T | - | - | - | - | - | - | - | - | G | Y | D |
| 080 UniRef90\_A0A365Y5E2\_33\_303 | F | G | Q | N | W | Y | M | W | E | R | L | L | P | E | L | - | S | K | H | - | F | T | V | I | A | P | D | L | P | G | L | G | E | S | G | K | P | A | D | - | - | - | - | - | - | - | - | G | Y | D |
| 081 UniRef90\_A0A1I2CFD2\_63\_327 | F | G | Q | N | W | Y | M | W | N | R | L | L | P | E | L | - | S | K | H | - | F | T | V | I | A | P | D | L | P | G | L | G | E | S | G | K | P | D | S | - | - | - | - | - | - | - | - | G | Y | D |
| 082 UniRef90\_A0A1G7Z850\_54\_322 | F | G | Q | N | W | Y | M | W | N | R | L | L | P | E | F | - | S | K | H | - | F | T | V | I | A | P | D | L | P | G | L | G | E | S | G | K | P | D | S | - | - | - | - | - | - | - | - | G | Y | D |
| 083 UniRef90\_A0A2V9XG70\_35\_308 | Y | A | E | T | S | L | M | W | K | P | I | I | P | G | L | - | A | E | R | - | F | T | V | I | A | P | D | L | P | G | I | G | D | S | A | I | P | A | D | - | - | - | - | - | - | - | - | G | L | D |
| 084 UniRef90\_A0A0Q4GSS4\_39\_308 | F | G | Q | N | W | F | M | W | N | R | L | L | P | E | L | - | S | K | H | - | F | T | V | I | A | P | D | M | R | G | V | G | E | S | G | K | T | A | A | - | - | - | - | - | - | - | - | G | Y | D |
| 085 UniRef90\_A0A0M9ZTU1\_1\_271 | F | P | Q | T | W | T | E | W | R | Q | Q | M | S | P | L | - | A | K | E | - | H | T | V | I | A | V | D | L | R | G | T | G | D | S | Q | V | M | K | G | - | - | - | - | - | - | - | - | G | Y | Q |
| 086 UniRef90\_A0A239P597\_1\_270 | F | P | E | E | W | S | E | W | R | Q | E | M | V | P | L | - | S | K | T | - | H | T | V | I | A | V | D | L | R | G | A | G | E | S | Q | V | T | E | S | - | - | - | - | - | - | - | - | G | Y | D |
| 087 UniRef90\_UPI00128E3D53\_19\_294 | W | P | Q | T | W | W | S | W | R | H | V | M | E | R | F | - | S | D | R | - | Y | T | V | I | A | A | D | L | R | G | C | G | L | S | E | I | T | K | T | - | - | - | - | - | - | - | - | G | Y | D |
| 088 UniRef90\_A0A1V9E1W0\_57\_321 | F | G | Q | N | W | Y | M | W | N | R | L | L | P | E | F | - | S | K | H | - | F | T | V | I | T | P | D | L | P | G | L | G | E | S | G | K | P | D | S | - | - | - | - | - | - | - | - | G | Y | D |
| 089 UniRef90\_A0A239MTD5\_1\_269 | F | P | Q | T | W | A | E | W | R | Q | Q | M | G | P | L | - | S | R | T | - | H | T | V | I | A | V | D | L | R | G | T | G | N | S | Q | V | T | E | S | - | - | - | - | - | - | - | - | G | Y | Q |
| 090 UniRef90\_UPI0009FCBBE8\_54\_317 | W | P | N | T | W | Y | E | W | H | S | V | M | P | E | L | - | A | K | T | - | H | T | V | I | A | F | D | L | P | G | L | G | G | S | A | V | S | T | D | G | - | - | - | - | - | - | - | K | Y | D |
| 091 UniRef90\_A0A1A9HTL2\_19\_299 | W | P | E | S | W | Y | A | W | R | F | V | M | P | R | L | I | A | E | G | - | H | P | V | V | A | L | D | L | R | G | M | G | D | S | D | H | P | P | E | - | - | - | - | - | - | - | - | G | Y | D |
| 092 UniRef90\_A0A4R7CM41\_23\_285 | F | G | Q | T | W | Y | M | W | N | R | L | L | P | V | L | - | S | R | H | - | F | T | V | I | A | P | D | L | R | G | V | G | E | S | G | K | P | E | D | - | - | - | - | - | - | - | - | G | Y | D |
| 093 UniRef90\_A0A562QYK6\_25\_307 | Y | A | E | T | S | H | M | W | R | P | L | M | K | E | L | - | A | K | T | - | N | L | V | I | A | P | D | L | R | G | A | G | S | S | A | A | P | D | E | - | - | - | - | - | - | - | - | G | Y | G |
| 094 UniRef90\_A0A2V9ZM06\_3\_269 | Y | A | E | T | S | L | M | W | K | P | I | I | P | L | L | - | A | E | R | - | F | T | V | I | A | P | D | L | P | G | I | G | D | S | A | I | P | P | D | - | - | - | - | - | - | - | - | G | L | D |
| 095 UniRef90\_A0A4P8YF40\_11\_271 | W | P | Q | T | W | Y | E | W | H | R | V | I | P | R | L | I | A | G | G | - | H | E | V | I | A | V | D | M | R | G | S | G | D | S | D | K | P | D | T | - | - | - | - | - | - | - | - | G | Y | D |
| 096 UniRef90\_A0A2V6E8I4\_30\_303 | Y | T | Q | T | S | R | M | W | K | P | I | I | P | L | L | - | A | E | R | - | F | T | V | I | A | P | D | L | P | G | I | G | D | S | A | V | P | A | N | - | - | - | - | - | - | - | - | G | L | D |
| 097 UniRef90\_A0A2V9BFT0\_23\_295 | Y | A | E | T | S | R | M | W | T | P | I | L | P | V | L | - | G | E | K | - | F | M | V | I | A | P | D | L | P | G | I | G | D | S | S | I | P | A | D | - | - | - | - | - | - | - | - | G | L | D |
| 098 UniRef90\_A0A2V9H5C8\_29\_298 | Y | A | E | T | S | R | M | W | T | P | I | L | P | L | F | - | G | E | K | - | F | T | V | I | A | P | D | L | P | G | I | G | D | S | A | I | P | T | D | - | - | - | - | - | - | - | - | G | L | D |
| 099 UniRef90\_A0A101CRV3\_44\_308 | F | G | Q | N | W | Y | M | W | N | R | L | L | P | E | L | - | S | K | H | - | F | T | V | I | A | P | D | L | R | G | V | G | E | S | G | K | P | E | S | - | - | - | - | - | - | - | - | G | Y | D |
| 100 UniRef90\_A0A5M6CFP9\_57\_321 | F | G | Q | N | W | F | M | W | N | R | L | L | P | E | F | - | A | K | H | - | Y | T | V | I | A | P | D | L | P | G | L | G | E | S | G | K | P | E | T | - | - | - | - | - | - | - | - | G | Y | D |
| 101 UniRef90\_A0A401Z9D4\_19\_284 | W | P | Q | T | W | Y | A | W | R | K | I | M | P | A | L | - | A | E | K | - | Y | T | V | I | A | P | D | S | R | G | M | G | D | S | E | R | T | D | S | - | - | - | - | - | - | - | - | G | Y | D |
| 102 UniRef90\_A0A2V8XJ58\_32\_308 | Y | A | E | T | S | R | M | W | T | P | I | L | P | V | L | - | G | E | K | - | F | T | V | I | A | P | D | L | P | G | I | G | E | S | S | I | P | A | D | - | - | - | - | - | - | - | - | G | L | D |
| 103 UniRef90\_A0A252EMP1\_15\_277 | W | P | Q | T | W | Y | E | W | H | R | V | I | P | H | L | V | E | A | G | - | H | E | V | I | A | V | D | M | R | G | A | G | D | S | S | R | P | A | S | - | - | - | - | - | - | - | - | G | Y | D |
| 104 UniRef90\_A0A1H3H759\_34\_310 | W | P | Q | T | W | Y | A | W | R | E | L | M | P | A | L | - | A | R | D | - | F | T | V | V | A | A | D | P | R | G | V | G | L | S | G | K | P | D | D | - | - | - | - | - | - | - | - | G | Y | D |
| 105 UniRef90\_A0A4D7B7N1\_3\_270 | F | P | Q | S | W | Y | A | W | R | R | V | M | P | L | I | - | A | E | R | - | H | K | V | I | A | I | D | L | P | G | Q | G | D | S | D | K | P | V | D | - | - | - | - | - | - | - | - | G | Y | D |
| 106 UniRef90\_A0A1Q7MUT6\_34\_308 | Y | A | E | T | S | L | M | W | K | P | M | I | P | L | L | - | A | Q | R | - | F | T | V | I | A | P | D | L | P | G | I | G | D | S | A | I | P | A | D | - | - | - | - | - | - | - | - | G | L | D |
| 107 UniRef90\_A0A2V6KQW0\_24\_297 | Y | A | E | T | S | R | M | W | K | P | I | I | P | L | L | - | A | E | R | - | F | T | V | I | A | P | D | L | P | G | I | G | D | S | G | I | P | A | N | - | - | - | - | - | - | - | - | G | I | D |
| 108 UniRef90\_A0A4R6LFZ5\_44\_308 | F | G | Q | N | W | Y | M | W | N | R | L | L | P | E | L | - | S | K | H | - | F | T | V | I | A | P | D | L | R | G | V | G | E | S | G | K | P | A | N | - | - | - | - | - | - | - | - | G | Y | D |
| 109 UniRef90\_A0A1H7UU25\_49\_318 | F | G | Q | N | W | Y | M | W | N | R | L | L | P | E | L | - | S | K | H | - | F | T | V | I | A | P | D | L | P | G | L | G | E | S | G | K | P | D | S | - | - | - | - | - | - | - | - | G | Y | T |
| 110 UniRef90\_UPI0008326796\_14\_259 | Y | P | E | S | W | Y | S | W | R | K | V | M | P | L | L | - | A | E | K | - | F | R | L | I | A | I | D | L | P | G | Q | G | D | S | D | K | P | T | D | - | - | - | - | - | - | - | - | G | Y | D |
| 111 UniRef90\_A0A2V5S8F2\_33\_306 | Y | A | E | T | S | R | M | W | K | P | I | I | P | V | L | - | S | E | R | - | F | T | V | I | A | P | D | L | P | G | I | G | D | S | A | I | P | A | D | - | - | - | - | - | - | - | - | G | L | D |
| 112 UniRef90\_A0A3S3VTN8\_36\_308 | F | G | Q | N | W | Y | M | W | N | R | L | L | P | E | L | - | S | K | H | - | F | T | V | I | A | P | D | M | R | G | V | G | E | S | G | K | P | A | D | - | - | - | - | - | - | - | - | G | Y | S |
| 113 UniRef90\_A0A6J4PSU4\_16\_248 | W | T | Q | T | W | R | G | W | R | K | V | M | P | L | L | - | A | E | R | - | H | T | V | I | A | P | D | L | R | G | M | G | D | S | E | K | P | L | G | - | - | - | - | - | - | - | - | G | Y | D |
| 114 UniRef90\_A0A534ZXC6\_26\_300 | Y | T | Q | T | S | R | M | W | K | P | V | I | P | K | L | - | A | T | R | - | F | T | V | I | A | P | D | L | P | G | I | G | D | S | A | I | P | A | D | - | - | - | - | - | - | - | - | G | L | N |
| 115 UniRef90\_A0A2A9KBX9\_78\_358 | W | P | Q | S | W | Y | A | W | R | F | I | M | P | A | L | - | A | A | S | G | R | R | V | I | A | I | D | P | R | G | M | G | D | S | S | H | P | L | E | - | - | - | - | - | - | - | - | G | Y | D |
| 116 UniRef90\_A0A2W0BWH7\_16\_287 | F | A | E | T | S | L | M | W | K | P | V | I | P | A | L | - | A | R | R | - | F | T | V | I | A | P | D | L | P | G | I | G | E | S | A | I | P | K | E | - | - | - | - | - | - | - | - | G | L | D |
| 117 UniRef90\_A0A1R1RMU1\_30\_310 | F | P | E | S | W | F | A | W | R | K | V | M | P | L | L | - | A | P | T | - | F | K | L | I | A | L | D | L | P | G | Q | G | D | S | D | R | P | A | V | - | - | - | - | - | - | - | - | G | Y | D |
| 118 UniRef90\_A0A3A1NH59\_38\_305 | F | G | Q | N | W | Y | M | W | N | R | L | L | P | E | L | - | S | K | H | - | F | T | V | I | A | P | D | L | R | G | I | G | E | S | D | K | P | E | S | - | - | - | - | - | - | - | - | G | Y | D |
| 119 UniRef90\_A0A0Q6WP84\_43\_317 | Y | T | Q | T | G | H | M | W | K | P | I | M | G | E | L | - | A | K | T | - | H | T | V | I | V | P | D | M | R | G | V | G | G | S | E | K | A | A | D | - | - | - | - | - | - | - | - | G | Y | L |
| 120 UniRef90\_A0A2V6P1A8\_15\_289 | Y | A | E | T | S | R | M | W | R | P | L | I | P | L | L | - | A | E | K | - | F | T | V | I | A | P | D | L | P | G | I | G | D | S | S | I | P | K | D | - | - | - | - | - | - | - | - | K | I | D |
| 121 UniRef90\_A0A2V5NC55\_35\_307 | Y | A | E | T | S | R | M | W | R | P | I | I | P | L | L | - | A | K | K | - | F | T | V | I | A | P | D | L | P | G | I | G | D | S | A | I | P | S | D | - | - | - | - | - | - | - | - | G | I | D |
| 122 UniRef90\_A0A2V9BM40\_39\_318 | Y | T | Q | T | S | R | M | W | R | P | I | I | P | L | L | - | A | E | K | - | F | T | V | I | A | P | D | L | P | G | I | G | D | S | E | I | P | A | T | - | - | - | - | - | - | - | - | G | L | D |
| 123 UniRef90\_A0A5B8WA72\_34\_302 | F | G | Q | N | W | Y | M | W | N | R | L | L | P | E | L | - | S | K | H | - | Y | T | V | I | A | P | D | M | R | G | V | G | E | S | G | K | P | T | A | - | - | - | - | - | - | - | - | G | Y | D |
| 124 UniRef90\_A0A1Y6D2S0\_25\_299 | W | P | Q | T | W | Y | E | W | H | R | I | M | P | M | L | - | A | G | K | - | Y | T | V | V | A | P | D | L | R | G | L | G | L | S | E | K | T | R | T | - | - | - | - | - | - | - | - | G | Y | D |
| 125 UniRef90\_A0A494W0L3\_12\_284 | F | G | Q | N | W | Y | M | W | N | R | L | L | P | E | L | - | S | K | H | - | F | T | I | I | A | P | D | L | R | G | V | G | E | S | E | K | T | A | G | - | - | - | - | - | - | - | - | G | Y | D |
| 126 UniRef90\_UPI0015CD0065\_39\_309 | W | P | Q | T | W | Q | M | W | E | K | V | M | P | T | L | - | A | R | E | - | H | T | V | I | A | F | D | L | P | G | L | G | E | S | S | I | P | S | G | - | - | - | - | - | - | - | - | G | Y | D |
| 127 UniRef90\_UPI00166E2F5D\_15\_289 | W | P | T | C | W | L | E | F | E | S | V | M | K | W | L | - | V | K | D | - | F | H | V | V | A | I | D | L | P | G | I | G | G | S | K | I | P | L | Q | - | - | - | - | - | - | - | - | S | Y | S |
| 128 UniRef90\_A0A372NPX1\_36\_305 | F | G | Q | N | W | Y | M | W | N | R | L | L | P | E | L | - | S | K | H | - | F | T | V | I | A | P | D | M | R | G | V | G | E | S | Q | K | T | T | A | - | - | - | - | - | - | - | - | G | Y | D |
| 129 UniRef90\_A0A317JA08\_13\_279 | Y | T | Q | T | S | R | M | W | R | P | L | I | D | K | L | - | K | D | K | - | F | T | I | I | A | S | D | L | P | G | I | G | D | S | D | I | P | K | D | - | - | - | - | - | - | - | - | G | C | D |
| 130 UniRef90\_A0A2V6N4R6\_10\_283 | F | A | E | T | S | R | M | W | R | P | L | I | P | L | L | - | A | E | K | - | F | T | V | I | A | P | D | L | P | A | I | G | D | S | S | I | P | R | D | - | - | - | - | - | - | - | - | K | I | D |
| 131 UniRef90\_A0A2V5IZ05\_29\_302 | Y | T | Q | T | S | R | M | W | K | A | I | I | P | F | L | - | A | N | K | - | F | T | V | I | A | P | D | L | P | G | I | G | E | S | E | I | P | A | S | - | - | - | - | - | - | - | - | G | L | D |
| 132 UniRef90\_A0A521E7V8\_39\_308 | F | G | Q | N | W | Y | M | W | N | R | L | L | P | E | L | - | S | N | H | - | F | T | V | I | A | P | D | L | R | G | V | G | E | S | E | K | T | T | G | - | - | - | - | - | - | - | - | G | Y | D |
| 133 UniRef90\_UPI001665F59F\_18\_290 | W | P | T | C | W | L | E | F | E | T | V | M | T | I | L | - | S | E | D | - | Y | H | V | I | A | I | D | L | P | G | I | G | E | S | E | V | P | L | K | - | - | - | - | - | - | - | - | S | Y | S |
| 134 UniRef90\_A0A0Q9GGK2\_19\_284 | W | P | Q | T | W | Y | A | W | K | D | I | I | P | E | L | - | A | K | S | - | H | T | I | I | V | P | D | L | R | G | A | G | L | S | D | K | P | K | T | - | - | - | - | - | - | - | - | G | Y | D |
| 135 UniRef90\_A0A329YBZ0\_37\_320 | W | P | Q | T | W | Y | T | W | R | N | I | M | P | S | L | - | A | K | A | G | Y | R | V | V | A | V | D | Y | R | G | A | G | E | S | E | K | P | L | A | - | - | - | - | - | - | - | - | G | Y | D |
| 136 UniRef90\_A0A2E0H9P2\_13\_236 | W | P | Q | T | W | W | E | W | R | H | V | I | P | E | L | - | S | K | D | - | Y | T | V | I | A | P | D | L | R | G | L | G | D | S | S | K | P | F | D | - | - | - | - | - | - | - | - | G | Y | D |
| 137 UniRef90\_A0A095SYG7\_44\_308 | F | G | Q | N | W | Y | M | W | N | R | L | L | P | E | L | - | S | K | H | - | F | T | V | I | A | P | D | L | R | G | V | G | E | S | D | K | P | A | T | - | - | - | - | - | - | - | - | G | Y | D |
| 138 UniRef90\_A0A2U0ZNC3\_54\_322 | F | G | Q | N | W | F | M | W | N | R | L | L | P | E | L | - | S | K | H | - | F | T | V | I | A | P | D | L | R | G | V | G | E | S | E | V | T | E | G | - | - | - | - | - | - | - | - | G | Y | D |
| 139 UniRef90\_A0A2H2XXU1\_20\_287 | W | P | Q | T | W | Y | E | W | R | K | I | M | P | A | L | - | A | K | N | - | Y | T | V | I | A | P | D | M | R | G | L | G | D | S | S | K | P | D | G | - | - | - | - | - | - | - | - | G | Y | D |
| 140 UniRef90\_A0A0P4V0Y0\_11\_278 | F | L | E | T | W | Y | C | W | R | K | I | M | P | A | L | - | A | E | R | - | Y | T | V | I | V | P | D | M | R | G | Y | G | D | S | D | K | P | E | T | - | - | - | - | - | - | - | - | G | Y | D |
| 141 UniRef90\_A0A2D7FF16\_11\_288 | W | P | Q | T | W | Y | E | W | R | H | I | I | P | R | L | - | A | E | R | - | F | T | V | I | A | P | D | L | R | G | L | G | D | S | S | R | P | L | S | - | - | - | - | - | - | - | - | G | Y | D |
| 142 UniRef90\_UPI0009FE04F9\_77\_354 | W | P | E | S | W | Y | E | Y | H | A | V | M | P | R | L | - | L | A | G | - | R | T | V | I | A | I | D | L | P | G | L | G | D | T | T | G | E | P | P | - | - | - | - | - | - | - | - | S | Y | T |
| 143 UniRef90\_UPI00148816F0\_34\_297 | F | T | E | T | W | Y | G | W | L | P | I | M | T | D | L | - | L | P | G | - | H | T | V | I | A | I | D | L | P | G | L | G | D | S | T | G | S | L | P | - | - | - | - | - | - | - | - | S | H | D |
| 144 UniRef90\_A0A2V5MXX8\_31\_304 | F | A | E | T | S | R | M | W | R | P | I | I | P | L | L | - | A | Q | R | - | F | T | V | I | A | P | D | L | P | G | I | G | D | S | S | I | P | A | D | N | S | E | N | F | R | E | R | G | I | D |
| 145 UniRef90\_Q1W504\_15\_282 | W | P | Q | S | R | R | E | W | R | H | V | I | P | S | L | - | A | S | H | - | F | T | V | I | A | P | D | M | R | G | F | G | D | S | D | K | P | S | S | - | - | - | - | - | - | - | - | G | Y | D |
| 146 UniRef90\_UPI001661900D\_10\_259 | F | P | H | T | W | E | V | W | T | D | V | M | A | D | L | - | A | G | R | - | Y | R | V | I | A | P | D | L | R | G | F | G | A | S | G | P | A | A | S | - | - | - | - | - | - | - | - | G | Y | D |
| 147 UniRef90\_A0A2D6SJL3\_11\_235 | W | P | Q | T | W | W | E | W | R | H | V | I | P | V | L | - | A | K | N | - | H | T | V | I | A | P | D | L | R | G | L | G | D | S | S | R | P | L | N | - | - | - | - | - | - | - | - | G | Y | D |
| 148 UniRef90\_A0A2V6J615\_3\_225 | - | - | - | - | - | - | - | - | - | - | - | - | - | - | - | - | - | - | - | - | - | - | V | I | A | P | D | L | P | G | I | G | D | S | S | I | P | - | D | - | - | - | - | - | - | - | - | K | I | D |

  
  

|  |  |  |  |  |  |  |  |  |  |  |  |  |  |  |  |  |  |  |  |  |  |  |  |  |  |  |  |  |  |  |  |  |  |  |  |  |  |  |  |  |  |  |  |  |  |  |  |  |  |  |
| --- | --- | --- | --- | --- | --- | --- | --- | --- | --- | --- | --- | --- | --- | --- | --- | --- | --- | --- | --- | --- | --- | --- | --- | --- | --- | --- | --- | --- | --- | --- | --- | --- | --- | --- | --- | --- | --- | --- | --- | --- | --- | --- | --- | --- | --- | --- | --- | --- | --- | --- |
| **001 Input\_protein\_seq** | A | E | N | T | S | K | I | L | I | G | A | I | K | - | K | I | A | G | K | - | - | G | P | I | Y | Y | V | S | H | D | L | G | N | T | A | S | Y | P | L | V | A | N | N | Q | G | - | Y | I | K | K |
| 002 UniRef90\_D8JE57\_1\_349 | A | E | N | V | S | K | I | L | L | S | A | I | K | - | Q | I | T | K | N | - | - | E | P | I | Y | Y | V | S | H | D | I | G | N | T | A | S | Y | P | L | V | A | N | N | Q | D | - | Y | V | K | K |
| 003 UniRef90\_A0A6B8MSH7\_6\_345 | A | Q | N | V | S | T | I | L | L | S | A | I | K | - | K | I | A | G | D | - | - | E | P | I | Y | F | V | T | H | D | L | G | N | S | A | S | Y | P | L | V | A | N | N | Q | G | - | Y | I | K | K |
| 004 UniRef90\_A0A6B2FNS7\_11\_352 | A | E | N | V | S | R | I | L | L | A | T | I | K | - | K | I | T | D | S | - | - | K | Q | I | Y | Y | V | S | H | D | L | G | N | S | A | S | Y | P | L | V | A | N | N | Q | G | S | Y | I | K | K |
| 005 UniRef90\_UPI00073D803C\_6\_344 | A | E | N | I | S | A | I | L | L | T | A | I | K | - | Q | I | A | G | N | - | - | G | P | I | Y | Y | V | S | H | D | L | G | N | S | A | S | Y | P | L | V | A | N | N | Q | G | - | Y | I | S | K |
| 006 UniRef90\_A0A285K5K8\_3\_346 | A | K | N | V | S | D | I | L | L | K | A | M | K | - | Q | V | A | G | T | - | - | K | P | V | Y | F | V | S | H | D | L | G | N | S | A | S | Y | P | L | V | A | E | N | Q | D | - | Y | I | K | K |
| 007 UniRef90\_A0A379I9D6\_1\_346 | A | E | N | V | S | R | L | L | L | G | A | V | K | - | K | I | T | A | A | - | - | Q | S | I | D | F | V | S | H | D | L | G | N | S | A | S | Y | P | F | V | A | R | N | Q | G | S | F | I | K | K |
| 008 UniRef90\_A0A6B2FTI5\_15\_352 | A | E | N | V | S | R | I | L | L | A | A | I | K | - | Q | I | V | G | N | - | - | K | P | I | Y | Y | V | S | H | D | L | G | N | S | A | S | Y | P | L | V | A | K | N | Q | G | N | V | V | K | K |
| 009 UniRef90\_A0A2P9HD85\_2\_318 | A | E | Q | M | S | A | Y | L | L | G | A | I | K | - | S | L | T | D | N | - | - | Q | P | F | T | Y | V | C | H | D | L | C | N | S | A | S | Y | P | M | V | A | N | N | Q | D | - | I | I | K | K |
| 010 UniRef90\_A0A1T1H7K2\_15\_316 | G | S | D | V | S | E | I | L | Y | K | L | A | T | - | K | L | S | N | D | - | - | Q | P | F | Y | L | V | A | H | D | I | G | I | W | N | S | Y | P | M | A | V | K | H | Q | D | - | K | I | K | K |
| 011 UniRef90\_A0A1A9HKQ2\_27\_313 | A | T | D | V | A | P | L | L | Y | G | L | A | K | - | N | L | S | G | G | - | - | Q | K | F | D | L | V | A | H | D | I | G | I | W | N | T | Y | P | M | L | V | A | H | Q | D | - | Q | I | R | R |
| 012 UniRef90\_A0A0J6GRA0\_22\_303 | G | T | D | V | S | K | Y | L | F | A | L | A | T | - | Q | L | S | G | N | - | - | Q | P | F | D | L | V | A | H | D | I | G | I | W | N | T | Y | P | M | A | V | N | H | Q | S | - | Q | I | R | K |
| 013 UniRef90\_UPI000F5BD18A\_25\_314 | A | I | D | V | A | P | L | L | Y | Q | L | A | E | - | K | F | S | G | G | - | - | K | K | F | D | L | V | A | H | D | I | G | I | W | N | T | Y | P | M | L | V | K | N | Q | D | - | N | I | R | R |
| 014 UniRef90\_A0A4V1G750\_26\_311 | G | R | D | V | A | P | L | L | F | K | L | A | T | - | R | F | S | G | E | - | - | Q | K | F | D | L | V | A | H | D | I | G | I | W | N | T | W | P | M | L | V | Q | H | Q | A | - | Q | I | R | K |
| 015 UniRef90\_U3U231\_21\_300 | A | T | D | V | A | P | L | L | Y | Q | L | A | Q | - | H | F | A | G | S | - | - | K | K | F | D | L | V | A | Y | D | I | G | I | W | N | T | Y | P | L | L | V | K | H | Q | D | - | S | I | R | R |
| 016 UniRef90\_F2LPZ2\_26\_313 | A | T | E | V | A | P | L | L | H | Q | L | A | L | - | R | F | S | G | Q | - | - | R | P | F | D | L | V | A | H | D | I | G | I | W | N | T | Y | P | M | L | V | R | H | P | E | - | D | I | R | R |
| 017 UniRef90\_A0A3M4V4S1\_6\_330 | G | V | D | I | S | E | Y | L | Y | K | L | A | K | - | Q | I | S | A | D | - | - | K | P | F | Y | L | V | A | H | D | I | G | I | W | N | S | Y | P | M | V | A | R | H | P | G | - | D | V | V | K |
| 018 UniRef90\_A0A178LP42\_49\_332 | G | Q | D | V | A | E | L | I | Y | R | F | A | K | - | S | Q | S | P | Q | - | - | G | Q | I | D | L | V | S | H | D | I | N | N | W | N | T | F | P | M | V | A | E | H | Q | A | - | D | F | E | H |
| 019 UniRef90\_T2L220\_20\_307 | A | T | D | I | A | V | L | L | Y | K | L | A | K | - | Q | F | S | G | N | - | - | Q | T | F | D | L | V | A | H | D | I | G | I | W | N | T | Y | P | M | L | V | Q | H | Q | N | - | A | I | R | R |
| 020 UniRef90\_UPI0014749FF6\_22\_304 | G | E | D | V | S | P | Y | I | Y | A | F | A | K | - | R | F | S | P | D | - | - | A | P | F | K | L | V | A | H | D | I | G | I | W | A | T | Y | P | M | L | S | Q | H | Q | K | - | D | I | S | Q |
| 021 UniRef90\_A0A172YJZ3\_18\_305 | A | T | D | V | S | A | L | L | Y | E | L | A | M | - | R | F | S | G | G | - | - | E | K | F | D | L | V | A | H | D | I | G | I | W | N | T | Y | P | M | L | V | Q | H | Q | D | - | D | I | R | R |
| 022 UniRef90\_UPI000737C6BF\_26\_313 | A | T | D | I | A | P | L | L | H | A | L | A | L | - | Q | F | S | G | G | - | - | E | R | F | D | L | V | A | H | D | I | G | I | W | N | T | Y | P | M | L | V | Q | H | Q | G | - | D | I | R | R |
| 023 UniRef90\_A0A329J6I4\_26\_309 | G | T | A | V | S | K | Y | L | F | D | L | A | T | - | Q | L | S | G | N | - | - | Q | P | F | D | L | V | A | H | D | I | G | I | W | N | T | Y | P | L | A | V | M | H | Q | S | - | Q | I | K | K |
| 024 UniRef90\_A0A2N3KZL1\_30\_325 | G | Q | D | I | S | P | I | L | Y | G | L | A | K | - | Q | F | S | P | D | - | - | A | P | F | D | L | V | A | H | D | I | G | I | W | N | T | Y | P | M | A | V | S | H | Q | K | - | D | I | R | K |
| 025 UniRef90\_UPI0006CA847F\_27\_323 | G | L | D | V | Y | P | Y | L | H | G | L | A | K | - | S | F | S | P | D | - | - | E | P | F | D | V | V | A | H | D | T | G | V | W | M | T | Y | P | M | I | A | Q | H | Q | D | - | D | I | S | K |
| 026 UniRef90\_UPI00054E0E46\_26\_312 | A | T | D | V | A | S | L | L | Y | K | L | A | L | - | Q | F | N | G | N | - | - | R | P | F | D | L | V | S | H | D | I | G | N | W | N | T | Y | P | F | V | V | K | H | E | N | - | N | L | R | R |
| 027 UniRef90\_A0A0S9PRE2\_29\_311 | G | Q | D | V | A | P | L | L | H | T | F | A | K | - | G | F | S | P | D | - | - | A | P | F | D | L | V | A | H | D | I | G | I | W | N | T | Y | P | M | L | A | A | H | P | K | - | D | V | R | R |
| 028 UniRef90\_W0AA74\_37\_318 | G | Q | D | I | S | K | I | I | Y | K | F | A | K | - | S | H | S | Q | D | - | - | G | K | I | N | L | V | S | H | D | I | G | N | W | N | T | F | P | T | V | A | E | H | Q | A | - | D | F | T | H |
| 029 UniRef90\_A0A2G0Y5Q3\_27\_312 | G | E | D | V | A | P | Y | L | Y | K | L | A | K | - | N | F | S | P | D | - | - | K | P | F | N | L | V | A | H | D | I | G | I | W | N | T | Y | P | M | V | V | K | N | Q | A | - | D | I | A | K |
| 030 UniRef90\_A0A1M6LZ92\_62\_349 | A | T | D | V | A | P | L | L | Y | Q | L | A | E | - | K | F | S | G | G | - | - | R | K | F | D | L | V | A | H | D | I | G | I | W | N | T | Y | P | M | L | V | R | H | Q | D | - | N | I | R | R |
| 031 UniRef90\_A0A4Z1C7D3\_28\_313 | G | Q | D | V | A | P | I | L | H | K | L | A | K | - | E | F | S | P | D | - | - | A | P | F | D | L | V | A | H | D | I | G | I | W | N | T | Y | P | M | A | A | N | H | Q | D | - | D | I | R | R |
| 032 UniRef90\_A0A367MBG0\_273\_561 | G | E | Q | V | A | V | Y | L | H | K | L | A | R | - | Q | F | S | P | D | - | - | R | P | F | D | L | V | A | H | D | I | G | I | W | N | T | Y | P | M | V | V | K | N | Q | A | - | D | I | A | R |
| 033 UniRef90\_UPI00067CEA54\_35\_318 | G | Q | D | V | A | K | L | I | Y | Q | F | A | K | - | S | H | S | Q | N | - | - | G | Q | M | D | L | V | S | H | D | I | G | N | W | N | T | F | P | M | V | A | E | H | Q | A | - | D | F | K | H |
| 034 UniRef90\_A0A1Y0L5M5\_31\_311 | G | E | D | I | S | E | Y | L | Y | K | L | A | I | - | N | F | S | G | D | - | - | N | K | F | S | L | V | A | H | D | I | G | I | W | N | S | Y | P | M | A | I | K | H | Q | D | - | K | I | S | K |
| 035 UniRef90\_UPI000C2FE79C\_29\_313 | A | I | D | V | A | P | L | L | Y | Q | L | A | K | - | N | I | S | G | G | - | - | Q | R | F | D | L | V | A | H | D | I | G | I | W | N | T | Y | P | M | L | V | N | H | Q | A | - | D | I | R | R |
| 036 UniRef90\_A0A4P8SJX7\_14\_295 | G | E | D | V | S | Q | Y | F | Y | K | L | A | D | - | S | L | S | P | G | - | - | E | K | F | T | L | V | A | H | D | I | G | I | W | S | T | Y | P | M | A | A | S | H | Q | D | - | R | I | S | K |
| 037 UniRef90\_A0A1I1BZT3\_28\_315 | G | Q | D | V | A | P | I | L | H | K | L | A | K | - | Q | F | S | P | T | - | - | A | P | F | D | L | V | A | H | D | I | G | I | W | N | T | Y | P | M | A | V | T | H | Q | D | - | D | I | R | K |
| 038 UniRef90\_UPI000DF0D36E\_15\_290 | G | E | D | V | S | E | Y | L | Y | K | L | A | I | - | N | F | S | G | D | - | - | R | T | F | S | L | V | A | H | D | I | G | I | W | N | T | Y | P | M | A | A | K | H | Q | D | - | K | I | N | K |
| 039 UniRef90\_UPI00136B8797\_37\_318 | G | Q | D | V | A | K | L | I | H | Q | F | A | K | - | S | H | S | P | D | - | - | A | P | F | D | L | V | S | H | D | I | G | N | W | N | T | F | P | M | V | A | E | H | P | K | - | D | F | K | H |
| 040 UniRef90\_A0A1H4CLJ1\_28\_313 | A | T | D | I | S | A | L | L | Y | H | H | A | K | - | S | H | S | M | G | - | - | K | K | F | D | L | V | A | H | D | I | G | I | W | V | T | Y | P | M | L | V | Q | N | Q | K | - | D | I | R | S |
| 041 UniRef90\_UPI000E1FE28F\_29\_313 | A | I | D | V | A | K | L | L | Y | Q | L | A | Q | - | H | F | S | G | G | - | - | Q | K | F | D | L | V | A | H | D | I | G | I | W | N | T | Y | P | M | L | V | K | H | Q | K | - | A | I | R | S |
| 042 UniRef90\_A0A556AX95\_26\_313 | A | T | D | I | A | P | L | L | Y | K | L | A | V | - | Q | F | S | G | G | - | - | E | K | F | D | L | V | A | H | D | I | G | I | W | N | T | Y | P | M | L | V | Q | R | Q | D | - | D | I | R | R |
| 043 UniRef90\_A0A6M8HNY7\_38\_322 | G | Q | D | V | A | P | V | L | Y | D | F | A | K | - | S | F | S | P | K | - | - | A | P | F | D | L | V | A | H | D | I | G | I | W | N | T | Y | P | M | A | V | A | H | Q | S | - | D | I | H | R |
| 044 UniRef90\_UPI001612B72A\_24\_301 | G | Q | D | V | A | V | L | L | H | K | F | A | K | - | S | F | S | P | S | - | - | E | P | F | D | L | V | A | H | D | I | G | I | W | N | T | F | P | M | A | V | Q | H | Q | G | - | D | I | R | R |
| 045 UniRef90\_UPI0012F66D09\_46\_328 | G | Q | D | V | A | P | L | L | Y | Q | F | A | K | - | Q | F | S | P | N | - | - | A | P | F | D | L | V | A | H | D | I | G | I | W | N | T | Y | P | M | A | V | E | H | Q | A | - | D | I | R | R |
| 046 UniRef90\_UPI000A94940B\_29\_316 | A | T | E | V | A | P | L | L | Y | K | L | A | E | - | Q | F | S | G | G | - | - | Q | K | F | D | L | V | A | H | D | I | G | I | W | N | T | Y | P | M | L | V | Q | H | Q | D | - | T | I | G | R |
| 047 UniRef90\_UPI00102FACD2\_29\_312 | A | T | D | V | A | P | L | L | Y | K | L | A | M | - | Q | F | S | G | G | - | - | K | P | F | D | L | V | S | H | D | I | G | N | W | N | T | Y | P | F | V | V | T | H | E | N | - | N | L | R | R |
| 048 UniRef90\_A0A1S8YNF4\_19\_297 | G | E | Q | V | A | E | Y | L | Y | K | L | A | E | - | T | L | S | G | D | - | - | E | K | F | T | L | I | A | H | D | I | G | I | W | N | T | Y | P | M | A | V | K | H | Q | D | - | K | I | D | K |
| 049 UniRef90\_UPI0015755320\_14\_301 | G | Q | D | V | S | V | Y | L | H | K | L | A | R | - | S | L | S | P | D | - | - | A | P | F | H | L | V | A | H | D | I | G | I | W | N | T | Y | P | M | A | V | Q | H | Q | A | - | D | I | A | R |
| 050 UniRef90\_A0A427Q4N3\_22\_313 | A | T | D | V | A | P | L | L | Y | K | L | A | A | - | Q | M | S | N | G | - | - | Q | K | F | D | L | V | A | H | D | I | G | I | W | N | T | F | P | M | L | V | Q | N | Q | A | - | N | I | R | R |
| 051 UniRef90\_A0A0A3Z1S4\_30\_318 | G | E | D | V | A | N | T | L | Y | K | L | A | D | - | S | L | R | G | D | - | - | Q | K | F | T | L | V | A | H | D | I | G | I | W | N | T | Y | P | M | A | V | K | H | Q | D | - | K | I | D | R |
| 052 UniRef90\_A0A3L8C981\_35\_323 | G | V | D | V | S | E | Y | L | Y | K | L | A | K | - | R | L | S | P | E | - | - | K | P | F | Y | L | V | A | H | D | I | G | I | W | N | S | Y | P | M | V | A | R | H | P | E | - | D | I | V | K |
| 053 UniRef90\_A0A6L5BLL6\_28\_324 | G | V | D | I | S | E | Y | L | H | K | L | A | K | - | R | L | S | H | D | - | - | K | P | F | Y | L | V | A | H | D | I | G | I | W | N | S | Y | P | M | V | A | R | Y | P | T | - | D | I | V | K |
| 054 UniRef90\_A0A6B1J288\_27\_314 | A | T | D | V | A | P | I | L | Y | K | L | A | R | - | Q | F | S | G | D | - | - | Q | K | F | D | L | V | A | H | D | I | G | I | W | N | T | Y | P | M | L | V | E | H | Q | D | - | T | I | R | R |
| 055 UniRef90\_A0A263NNG9\_22\_274 | G | Q | E | V | S | P | Y | I | Y | A | F | A | K | - | R | F | S | P | D | - | - | A | P | F | N | L | M | A | H | D | I | G | I | W | A | T | Y | P | M | L | A | Q | H | P | K | - | D | I | A | K |
| 056 UniRef90\_W0HXQ2\_33\_307 | G | A | A | I | A | D | A | L | Y | R | L | A | L | - | H | P | S | Q | G | - | - | Q | P | F | S | L | V | A | H | D | I | G | I | W | N | T | Y | P | L | A | V | K | H | P | R | - | H | I | D | K |
| 057 UniRef90\_A0A031HKS8\_40\_323 | G | Q | D | V | A | P | V | L | H | E | F | A | K | - | S | F | S | P | D | - | - | A | P | F | D | L | V | A | H | D | I | G | I | W | N | T | Y | P | M | V | A | A | H | Q | D | - | D | V | R | R |
| 058 UniRef90\_A0A4R2FY05\_27\_320 | G | Q | D | V | S | P | Y | I | H | S | L | A | K | - | T | F | S | P | D | - | - | D | K | F | N | V | I | A | H | D | I | G | G | W | V | T | Y | P | M | L | A | Q | H | Q | N | - | D | I | D | K |
| 059 UniRef90\_A0A1X0N2L9\_36\_321 | G | V | D | I | S | E | Y | L | H | K | L | A | K | - | R | V | S | A | G | - | - | K | A | F | Y | L | V | A | H | D | I | G | I | W | N | S | Y | P | M | V | A | R | H | P | E | - | D | I | I | K |
| 060 UniRef90\_A0A4R1U0K9\_37\_322 | G | V | D | I | S | D | Y | L | Y | K | F | A | K | - | R | F | S | P | D | - | - | K | P | F | F | L | V | A | H | D | I | G | I | W | N | S | Y | P | M | V | A | R | H | P | A | - | D | I | V | K |
| 061 UniRef90\_A0A0Q8TEQ0\_42\_323 | G | Q | D | V | A | K | L | L | H | K | F | A | K | - | S | F | S | P | D | - | - | A | P | F | D | L | V | A | H | D | I | G | I | W | N | T | Y | P | M | V | V | Q | N | Q | D | - | D | V | R | R |
| 062 UniRef90\_A0A0Q6ZEJ0\_41\_323 | G | Q | D | V | A | D | L | L | H | K | F | A | K | - | S | F | S | P | D | - | - | A | P | F | D | L | V | A | H | D | I | G | I | W | N | T | Y | P | M | V | V | Q | N | Q | D | - | D | V | R | R |
| 063 UniRef90\_UPI0004C0CBB1\_54\_338 | T | A | Q | M | A | G | D | V | H | E | L | L | K | - | Q | L | K | L | N | - | - | K | G | V | Q | L | V | G | H | D | I | G | L | W | V | S | Y | A | Y | A | A | Q | W | P | S | - | E | V | R | R |
| 064 UniRef90\_UPI0004CAB4F8\_56\_339 | K | A | Q | M | A | G | D | V | H | E | L | L | K | - | Q | L | K | L | N | - | - | N | G | V | Q | I | V | G | H | D | V | G | L | W | V | S | Y | A | Y | A | A | Q | W | P | S | - | E | V | R | S |
| 065 UniRef90\_UPI00049002CC\_60\_343 | A | A | Q | L | A | K | D | V | H | Q | L | L | K | - | Q | L | G | L | N | - | - | N | G | I | Q | V | V | G | H | D | V | G | V | W | V | T | Y | A | Y | A | A | Q | W | P | S | - | E | V | R | R |
| 066 UniRef90\_A0A561IX74\_59\_327 | K | K | A | L | A | G | Y | I | H | G | L | V | K | - | E | L | G | Y | N | - | - | - | N | I | N | L | A | G | H | D | I | G | L | M | V | A | Y | A | Y | A | A | Q | F | S | G | - | E | V | K | K |
| 067 UniRef90\_A0A0X3RYG7\_67\_350 | A | V | Q | L | S | K | D | V | H | Q | L | L | T | - | Q | L | K | L | N | - | - | N | G | V | Q | V | V | A | H | D | I | G | M | W | V | A | Y | G | Y | A | A | Q | Y | R | S | - | Q | V | R | S |
| 068 UniRef90\_A0A2W2E1M5\_55\_338 | A | A | Q | L | A | K | D | V | H | Q | L | L | T | - | Q | L | K | L | N | - | - | N | G | V | Q | V | V | G | H | D | I | G | V | W | V | A | Y | D | Y | A | A | Q | Y | R | S | - | E | V | R | S |
| 069 UniRef90\_A0A2V7YB42\_28\_306 | M | K | N | A | A | I | R | I | H | A | L | A | R | - | S | L | G | L | E | - | - | - | K | A | R | V | V | G | H | D | I | G | L | M | V | A | Y | A | Y | A | A | Q | F | P | A | - | E | V | E | K |
| 070 UniRef90\_A0A544XC49\_52\_336 | A | A | Q | L | A | K | D | V | H | Q | L | L | T | - | Q | L | K | L | N | - | - | N | G | V | Q | V | V | A | H | D | I | G | V | W | V | A | Y | D | Y | A | A | Q | F | R | S | - | E | V | R | S |
| 071 UniRef90\_A0A101PQ90\_46\_324 | K | K | T | M | A | A | K | L | H | A | L | L | V | - | S | L | G | K | A | - | - | D | D | V | N | V | V | G | H | D | I | G | T | M | V | A | Y | S | Y | A | A | Q | Y | P | H | - | S | V | R | K |
| 072 UniRef90\_A0A1C4VK67\_14\_288 | K | K | T | V | A | A | D | L | D | G | L | L | D | - | Q | L | G | L | T | - | - | G | D | L | Q | M | V | G | H | D | L | G | T | M | V | A | Y | A | Y | A | A | A | H | P | Q | - | R | V | S | R |
| 073 UniRef90\_A0A0X3RPC2\_57\_340 | A | A | Q | L | A | R | D | V | H | Q | L | L | K | - | Q | L | G | L | N | - | - | K | G | V | Q | V | V | A | H | D | L | G | V | F | V | A | Y | G | Y | A | A | Q | W | P | S | - | E | V | R | S |
| 074 UniRef90\_A0A4R2VWW4\_55\_319 | K | K | T | L | A | A | Y | I | R | G | L | V | K | - | Q | L | G | Y | D | - | - | - | K | I | N | L | A | G | H | D | I | G | L | M | V | A | Y | A | Y | A | A | Q | F | S | G | - | E | V | K | K |
| 075 UniRef90\_A0A1Q7VB11\_36\_307 | M | K | T | A | A | T | R | I | H | A | L | A | K | - | S | L | G | V | A | - | - | - | K | V | R | V | V | G | H | D | I | G | L | M | V | A | Y | A | Y | A | A | Q | F | P | S | - | E | V | E | K |
| 076 UniRef90\_C7PGQ3\_38\_305 | K | K | T | M | A | T | Y | I | H | G | V | V | K | - | E | L | G | Y | N | - | - | - | H | I | K | L | A | G | H | D | I | G | L | M | V | A | Y | A | Y | A | A | Q | Y | S | D | - | E | V | T | K |
| 077 UniRef90\_A0A1N7R4E6\_62\_325 | K | K | S | L | A | V | Y | I | H | G | L | V | K | - | Q | L | G | Y | T | - | - | - | N | I | N | L | A | G | H | D | I | G | L | M | V | A | Y | A | Y | A | A | Q | F | S | G | - | E | V | K | K |
| 078 UniRef90\_A0A2H2Y4V3\_41\_307 | A | T | T | V | A | E | D | I | F | Q | L | V | N | - | R | L | G | F | Q | - | - | - | S | I | Y | L | V | G | H | D | I | A | G | A | V | A | Y | A | Y | A | A | K | H | P | N | - | A | V | Q | R |
| 079 UniRef90\_A0A448SCU6\_8\_276 | S | N | T | V | A | G | D | L | H | A | L | V | N | - | H | L | G | F | E | - | - | - | R | I | R | V | V | A | H | D | N | G | A | R | V | A | Y | A | Y | A | A | N | Y | R | H | - | Q | V | K | S |
| 080 UniRef90\_A0A365Y5E2\_33\_303 | K | K | T | M | A | L | H | I | H | A | L | M | Q | - | K | L | G | F | N | - | - | - | N | I | M | L | A | G | H | D | I | G | L | M | V | A | Y | A | Y | A | R | Q | Y | E | N | - | E | V | T | K |
| 081 UniRef90\_A0A1I2CFD2\_63\_327 | K | K | A | L | A | G | Y | I | H | G | L | V | K | - | Q | L | G | Y | N | - | - | - | N | I | N | L | A | G | H | D | I | G | L | M | I | A | Y | A | Y | A | A | Q | F | S | G | - | E | V | K | K |
| 082 UniRef90\_A0A1G7Z850\_54\_322 | K | K | T | M | A | A | Y | I | H | G | V | V | K | - | Q | L | G | Y | D | - | - | - | H | I | N | L | A | G | H | D | I | G | L | M | V | A | Y | A | Y | A | A | Q | F | S | N | - | E | V | N | K |
| 083 UniRef90\_A0A2V9XG70\_35\_308 | M | K | T | S | A | I | R | I | H | A | L | A | R | - | S | L | G | V | Q | - | - | - | K | A | E | V | V | G | H | D | I | G | L | M | V | A | Y | A | Y | A | A | Q | F | P | A | - | E | V | Q | K |
| 084 UniRef90\_A0A0Q4GSS4\_39\_308 | K | K | N | M | A | K | D | M | H | E | L | M | N | - | K | L | G | Y | K | - | - | - | N | V | N | V | V | G | H | D | I | G | L | M | V | G | Y | A | Y | A | A | Q | F | P | K | - | E | V | K | K |
| 085 UniRef90\_A0A0M9ZTU1\_1\_271 | A | A | Q | L | A | G | D | V | H | Q | L | L | K | - | Q | L | G | L | N | - | - | N | G | I | Q | V | V | G | H | D | L | G | V | W | V | S | Y | A | Y | A | A | Q | W | P | N | - | E | V | R | R |
| 086 UniRef90\_A0A239P597\_1\_270 | A | A | Q | L | A | K | D | V | H | Q | L | L | A | - | Q | L | K | F | N | - | - | N | G | V | Q | V | V | A | H | D | I | G | V | W | V | A | Y | A | Y | A | A | Q | F | R | S | - | E | V | R | S |
| 087 UniRef90\_UPI00128E3D53\_19\_294 | K | A | N | L | A | K | D | I | A | E | L | I | D | - | K | V | A | G | G | - | - | - | K | A | I | V | V | S | H | D | M | G | G | K | T | S | Y | M | L | A | H | L | Y | P | D | - | R | I | E | K |
| 088 UniRef90\_A0A1V9E1W0\_57\_321 | K | K | T | L | A | T | Y | I | H | G | V | V | K | - | Q | L | G | Y | D | - | - | - | H | I | N | L | A | G | H | D | I | G | L | M | V | A | Y | A | Y | A | A | Q | F | S | N | - | E | V | N | K |
| 089 UniRef90\_A0A239MTD5\_1\_269 | A | A | Q | L | A | E | D | V | H | E | L | L | K | - | Q | L | G | L | N | - | - | N | D | I | Q | V | V | A | H | D | V | G | V | W | V | A | Y | A | Y | A | A | Q | W | P | S | - | E | V | R | R |
| 090 UniRef90\_UPI0009FCBBE8\_54\_317 | T | I | S | V | A | S | K | V | H | Q | G | V | T | - | G | L | G | Y | Q | - | - | - | S | V | A | I | L | A | H | D | V | G | S | L | V | A | Y | P | Y | A | R | N | F | P | G | - | E | V | S | R |
| 091 UniRef90\_A0A1A9HTL2\_19\_299 | S | K | T | I | S | A | D | I | H | A | F | V | A | - | A | K | G | L | A | K | K | G | N | L | H | V | A | G | H | D | V | G | A | W | M | A | Y | A | Y | A | A | D | W | P | R | - | E | V | R | T |
| 092 UniRef90\_A0A4R7CM41\_23\_285 | K | K | T | M | A | T | D | I | Y | A | L | V | K | - | K | L | G | Y | K | - | - | - | N | I | S | L | A | G | H | D | I | G | F | M | V | A | Y | A | Y | A | A | Q | Y | G | A | - | D | V | M | K |
| 093 UniRef90\_A0A562QYK6\_25\_307 | K | V | A | L | A | R | D | I | H | A | L | V | T | - | G | L | K | L | G | - | - | - | P | I | K | L | V | G | H | D | I | G | L | M | V | A | Y | A | Y | A | A | Q | Y | P | S | - | E | V | Q | R |
| 094 UniRef90\_A0A2V9ZM06\_3\_269 | M | K | T | A | A | L | R | I | H | A | L | A | R | - | S | F | G | V | Q | - | - | - | K | A | E | V | V | G | H | D | I | G | L | M | V | A | Y | A | Y | A | A | Q | F | P | A | - | E | V | A | K |
| 095 UniRef90\_A0A4P8YF40\_11\_271 | S | N | T | V | A | D | D | L | H | A | L | V | A | - | H | L | G | F | Q | - | - | - | K | I | R | L | V | A | H | D | N | G | A | R | V | A | Y | A | Y | A | A | N | Y | R | Q | - | E | V | K | S |
| 096 UniRef90\_A0A2V6E8I4\_30\_303 | M | K | T | S | A | I | R | I | H | A | L | A | R | - | S | L | G | V | E | - | - | - | K | A | K | V | V | G | H | D | I | G | L | M | V | A | Y | A | Y | A | A | Q | F | P | A | - | E | V | E | K |
| 097 UniRef90\_A0A2V9BFT0\_23\_295 | M | K | T | A | A | I | R | I | H | A | L | A | R | - | S | L | G | V | E | - | - | - | K | A | R | V | V | G | H | D | I | G | L | M | V | A | Y | A | Y | A | A | Q | F | P | S | - | E | V | E | K |
| 098 UniRef90\_A0A2V9H5C8\_29\_298 | M | K | T | A | A | M | R | I | H | A | L | A | R | - | S | L | G | V | A | - | - | - | K | A | R | V | V | G | H | D | I | G | L | M | V | A | Y | A | Y | A | A | Q | F | P | S | - | E | V | E | K |
| 099 UniRef90\_A0A101CRV3\_44\_308 | K | K | T | M | A | S | D | I | H | E | L | V | K | - | K | L | G | Y | K | - | - | - | N | I | N | I | A | G | H | D | I | G | L | M | V | A | Y | A | Y | A | A | Q | Y | P | G | - | E | V | K | K |
| 100 UniRef90\_A0A5M6CFP9\_57\_321 | K | K | T | M | A | S | V | I | H | G | L | I | K | - | E | L | G | Y | K | - | - | - | Q | V | N | L | A | G | H | D | I | G | L | M | V | A | Y | A | Y | A | A | Q | Y | G | S | - | E | V | K | K |
| 101 UniRef90\_A0A401Z9D4\_19\_284 | A | S | T | L | A | E | D | A | F | S | L | V | R | - | S | L | G | F | Q | - | - | - | H | I | F | L | I | S | H | D | L | G | V | S | A | A | Y | A | Y | A | A | R | Y | R | E | - | A | V | Q | R |
| 102 UniRef90\_A0A2V8XJ58\_32\_308 | L | K | T | A | A | I | R | I | H | A | L | A | R | - | S | L | G | V | E | - | - | - | K | A | R | V | V | G | H | D | I | G | L | M | V | A | Y | A | Y | A | A | Q | F | P | G | - | E | V | E | K |
| 103 UniRef90\_A0A252EMP1\_15\_277 | S | N | T | V | A | D | E | L | H | A | L | V | R | - | H | L | G | F | A | - | - | - | S | I | R | L | V | A | H | D | N | G | A | R | V | A | Y | A | Y | A | A | R | H | P | T | - | E | V | K | S |
| 104 UniRef90\_A0A1H3H759\_34\_310 | T | G | T | L | A | A | D | M | A | G | L | M | S | - | A | L | G | H | D | - | - | - | R | F | A | M | V | G | H | D | V | G | M | W | T | G | Y | A | L | A | A | D | H | P | G | - | R | V | E | R |
| 105 UniRef90\_A0A4D7B7N1\_3\_270 | T | R | T | T | G | E | R | I | H | A | L | L | Q | - | T | L | G | H | D | - | - | - | R | Y | F | I | G | A | H | D | I | G | A | W | V | A | Y | P | Y | V | A | R | Y | A | D | - | E | V | R | R |
| 106 UniRef90\_A0A1Q7MUT6\_34\_308 | M | K | T | A | A | I | R | I | H | A | L | A | R | - | S | L | G | V | Q | - | - | - | K | A | E | V | V | G | H | D | I | G | L | M | V | A | Y | A | Y | A | A | Q | F | P | A | - | E | V | E | K |
| 107 UniRef90\_A0A2V6KQW0\_24\_297 | M | K | T | S | A | N | R | I | H | A | L | V | R | - | S | L | G | I | E | - | - | - | K | A | R | V | V | G | H | D | I | G | L | M | V | A | Y | A | Y | A | T | Q | F | P | A | - | E | T | E | K |
| 108 UniRef90\_A0A4R6LFZ5\_44\_308 | K | K | T | M | A | N | D | I | H | E | L | V | K | - | K | L | G | Y | K | - | - | - | N | I | N | I | A | G | H | D | I | G | L | M | V | A | Y | A | Y | A | A | Q | F | P | G | - | E | V | K | K |
| 109 UniRef90\_A0A1H7UU25\_49\_318 | K | K | Q | L | A | Q | D | L | H | G | L | V | K | - | Q | L | G | Y | S | - | - | - | N | I | D | V | V | G | H | D | I | G | L | M | V | A | Y | A | Y | A | A | Q | Y | S | N | - | E | V | K | K |
| 110 UniRef90\_UPI0008326796\_14\_259 | T | E | T | V | A | R | Y | V | H | G | L | L | S | - | Q | K | G | I | T | - | - | - | H | Y | G | L | V | A | H | D | V | G | A | W | V | A | F | P | Y | A | A | M | Y | Q | N | - | E | L | S | G |
| 111 UniRef90\_A0A2V5S8F2\_33\_306 | M | K | T | S | A | I | R | I | H | A | L | A | R | - | S | L | G | V | E | - | - | - | K | A | R | V | V | G | H | D | I | G | L | M | V | A | Y | A | Y | A | A | Q | F | A | G | - | E | T | E | K |
| 112 UniRef90\_A0A3S3VTN8\_36\_308 | K | K | N | M | A | V | D | M | H | E | L | M | K | - | K | L | G | Y | Q | - | - | - | H | I | N | I | A | G | H | D | I | G | L | M | V | A | Y | A | Y | A | A | Q | F | P | A | - | D | V | K | K |
| 113 UniRef90\_A0A6J4PSU4\_16\_248 | G | L | T | V | A | E | D | V | R | Q | L | V | L | - | G | L | G | F | A | - | - | - | S | F | H | L | V | G | H | D | I | G | G | W | A | A | Y | P | L | A | A | S | H | P | E | - | A | V | R | T |
| 114 UniRef90\_A0A534ZXC6\_26\_300 | M | K | T | A | A | V | R | V | H | A | L | A | R | - | A | L | G | I | D | - | - | - | K | A | R | V | V | G | H | D | V | G | L | M | V | A | Y | A | Y | A | T | Q | F | P | A | - | E | V | E | K |
| 115 UniRef90\_A0A2A9KBX9\_78\_358 | L | K | T | V | A | N | D | V | H | Q | F | V | E | - | A | L | G | L | A | H | D | G | Q | L | Y | V | A | G | H | D | V | G | A | W | I | A | Y | A | Y | A | A | D | W | S | A | - | D | V | K | R |
| 116 UniRef90\_A0A2W0BWH7\_16\_287 | M | K | S | A | A | V | R | M | H | A | L | V | R | - | S | L | G | F | E | - | - | - | K | A | E | V | V | G | H | D | I | G | L | M | V | A | Y | A | Y | A | A | Q | F | P | A | - | E | I | T | K |
| 117 UniRef90\_A0A1R1RMU1\_30\_310 | T | K | T | L | A | I | M | V | H | K | F | L | Q | - | Q | L | G | T | K | - | - | - | R | Y | F | L | A | A | H | D | V | G | A | W | V | A | Y | P | Y | A | A | L | F | G | D | - | E | V | Q | R |
| 118 UniRef90\_A0A3A1NH59\_38\_305 | K | K | T | M | A | V | D | I | H | E | M | V | K | - | Q | L | G | Y | E | - | - | - | K | I | N | L | V | G | H | D | I | G | M | M | V | A | Y | A | Y | A | A | Q | F | G | N | - | E | V | K | K |
| 119 UniRef90\_A0A0Q6WP84\_43\_317 | K | T | N | M | A | K | D | I | H | E | L | V | G | - | S | I | S | R | E | - | - | - | P | A | T | V | V | G | H | D | I | G | L | M | V | A | Y | A | Y | A | A | Q | Y | P | A | - | D | T | K | K |
| 120 UniRef90\_A0A2V6P1A8\_15\_289 | M | I | T | S | A | E | R | I | H | A | L | V | R | - | S | L | G | V | E | - | - | - | K | A | R | V | V | G | H | D | I | G | L | M | V | A | Y | A | Y | A | A | K | F | P | A | - | E | T | E | K |
| 121 UniRef90\_A0A2V5NC55\_35\_307 | M | I | T | S | A | K | R | I | H | A | L | V | R | - | S | L | G | V | E | - | - | - | K | A | R | V | V | G | H | D | I | G | L | M | V | A | Y | A | Y | A | A | Q | F | P | A | - | E | T | E | K |
| 122 UniRef90\_A0A2V9BM40\_39\_318 | M | K | A | A | A | I | R | I | H | A | L | A | R | - | S | L | G | V | E | - | - | - | K | A | R | V | V | G | H | D | I | G | L | M | V | A | Y | A | Y | A | A | Q | F | P | A | - | E | V | E | K |
| 123 UniRef90\_A0A5B8WA72\_34\_302 | K | K | N | M | A | V | D | M | H | E | L | M | K | - | K | L | G | Y | Q | - | - | - | H | V | N | V | V | G | H | D | I | G | L | M | V | A | Y | A | Y | A | A | Q | F | P | D | - | D | V | K | K |
| 124 UniRef90\_A0A1Y6D2S0\_25\_299 | K | P | T | I | A | N | D | I | A | A | L | I | Q | - | H | L | G | R | G | - | - | - | P | A | F | V | V | G | H | D | M | G | G | K | A | A | Y | V | L | G | L | V | H | P | E | - | L | V | A | K |
| 125 UniRef90\_A0A494W0L3\_12\_284 | K | K | T | M | A | V | D | L | H | E | L | I | K | - | K | L | G | Y | K | - | - | - | N | I | N | L | A | G | H | D | I | G | L | M | V | A | Y | A | Y | A | V | Q | F | P | S | - | E | V | K | K |
| 126 UniRef90\_UPI0015CD0065\_39\_309 | K | A | A | T | A | R | R | I | H | E | A | V | V | - | K | L | G | L | K | - | - | - | Q | V | G | I | I | G | H | D | L | G | A | L | I | A | Y | P | Y | A | R | D | Y | P | G | - | E | V | S | R |
| 127 UniRef90\_UPI00166E2F5D\_15\_289 | K | R | H | I | A | M | Y | V | R | G | V | M | D | - | T | M | A | L | T | - | - | - | D | V | T | L | V | G | C | D | V | G | G | Q | V | T | Y | A | F | L | K | E | F | P | N | - | R | I | A | R |
| 128 UniRef90\_A0A372NPX1\_36\_305 | K | K | N | M | A | V | D | M | H | E | L | M | Q | - | K | L | G | Y | K | - | - | - | H | V | N | V | A | G | H | D | I | G | L | M | V | A | Y | A | Y | A | A | Q | Y | P | A | - | E | V | K | K |
| 129 UniRef90\_A0A317JA08\_13\_279 | M | K | T | A | A | I | R | I | H | A | L | A | K | - | S | I | G | V | T | - | - | - | N | A | R | V | V | G | H | D | I | G | L | M | V | A | Y | A | Y | A | A | Q | F | S | R | - | E | V | E | K |
| 130 UniRef90\_A0A2V6N4R6\_10\_283 | M | I | T | S | A | N | R | I | H | A | L | V | H | - | S | L | G | I | E | - | - | - | K | A | R | V | V | G | H | D | I | G | L | M | V | A | Y | A | Y | A | A | Q | F | P | A | - | E | T | E | K |
| 131 UniRef90\_A0A2V5IZ05\_29\_302 | M | K | T | S | A | I | R | I | H | A | L | A | K | - | S | L | G | V | E | - | - | - | K | A | S | V | V | G | H | D | I | G | L | M | V | A | Y | A | Y | A | A | Q | F | P | A | - | E | T | E | K |
| 132 UniRef90\_A0A521E7V8\_39\_308 | K | K | N | M | A | L | D | L | H | E | L | M | K | - | K | L | G | Y | S | - | - | - | H | I | S | L | A | G | H | D | I | G | L | M | V | A | Y | C | Y | A | A | Q | F | P | A | - | D | V | K | K |
| 133 UniRef90\_UPI001665F59F\_18\_290 | K | R | T | I | A | A | C | V | R | G | V | M | E | - | T | M | D | L | T | - | - | - | D | V | T | L | V | G | C | D | V | G | G | Q | I | T | Y | A | F | L | K | N | Y | P | T | - | I | I | S | R |
| 134 UniRef90\_A0A0Q9GGK2\_19\_284 | K | L | T | L | A | Q | D | I | H | A | L | S | E | - | K | L | G | F | N | - | - | - | Q | I | S | L | V | G | H | D | L | G | G | M | V | A | F | T | Y | A | A | E | Y | P | E | - | N | V | K | K |
| 135 UniRef90\_A0A329YBZ0\_37\_320 | K | A | T | M | A | A | D | I | R | A | L | I | D | - | G | L | G | A | T | - | - | - | K | V | H | L | V | G | R | D | I | G | V | M | V | A | Y | A | Y | A | A | Q | W | P | D | - | E | V | A | S |
| 136 UniRef90\_A0A2E0H9P2\_13\_236 | K | M | T | V | A | N | D | I | W | K | L | V | S | E | K | L | G | Y | S | - | - | - | S | F | L | L | V | G | H | D | W | G | G | P | T | A | Y | A | L | A | A | S | H | P | E | - | A | I | E | K |
| 137 UniRef90\_A0A095SYG7\_44\_308 | K | K | T | M | A | I | D | I | H | E | L | V | K | - | K | L | G | Y | N | - | - | - | K | I | N | L | A | G | H | D | I | G | L | M | V | A | Y | A | Y | A | A | Q | F | P | S | - | E | V | K | K |
| 138 UniRef90\_A0A2U0ZNC3\_54\_322 | K | K | T | M | A | V | D | I | H | E | L | M | K | - | K | L | G | Y | N | - | - | - | S | I | N | L | V | G | H | D | I | G | L | M | V | A | Y | A | Y | A | A | Q | Y | G | N | - | E | V | K | K |
| 139 UniRef90\_A0A2H2XXU1\_20\_287 | K | R | T | V | A | E | D | I | Y | Q | L | I | K | - | S | L | G | F | K | - | - | - | Q | I | N | L | V | G | H | D | L | G | G | M | V | A | Y | A | Y | A | T | A | Y | P | E | - | S | V | R | R |
| 140 UniRef90\_A0A0P4V0Y0\_11\_278 | A | R | S | L | S | E | D | F | R | Q | L | I | Q | - | Q | L | G | F | E | - | - | - | K | I | H | I | V | A | H | D | M | G | A | P | P | A | L | I | Y | T | N | D | Y | P | D | - | E | V | L | S |
| 141 UniRef90\_A0A2D7FF16\_11\_288 | K | K | T | V | S | G | D | V | W | R | L | V | S | E | Q | L | G | H | E | - | - | - | T | F | H | L | V | G | H | D | W | G | G | P | T | A | F | A | L | A | A | A | H | P | E | - | S | I | R | T |
| 142 UniRef90\_UPI0009FE04F9\_77\_354 | K | T | T | L | A | R | Y | V | S | A | L | L | T | - | K | I | G | V | G | - | - | H | D | V | R | V | V | A | H | D | F | G | V | G | V | A | Y | A | L | A | A | E | H | R | E | - | Q | I | A | G |
| 143 UniRef90\_UPI00148816F0\_34\_297 | K | V | T | L | A | G | Y | V | H | R | L | L | D | - | L | L | G | F | T | - | - | S | G | V | Q | L | V | S | H | D | A | G | S | G | I | A | F | A | L | V | T | Q | W | R | D | - | Q | F | T | G |
| 144 UniRef90\_A0A2V5MXX8\_31\_304 | I | L | S | S | A | T | R | I | H | G | L | V | H | - | S | L | G | I | E | - | - | - | K | A | R | V | V | G | H | D | I | G | L | M | V | A | Y | A | Y | A | T | Q | F | P | A | - | E | T | E | K |
| 145 UniRef90\_Q1W504\_15\_282 | K | R | T | V | A | K | D | I | H | E | L | I | H | - | Q | L | G | F | E | - | - | - | K | I | Y | L | V | G | H | D | I | G | L | M | V | A | Y | E | Y | A | A | S | H | P | N | - | E | V | K | K |
| 146 UniRef90\_UPI001661900D\_10\_259 | A | G | T | L | A | E | D | A | A | A | L | L | A | - | A | L | G | V | S | - | - | - | S | A | A | V | V | G | I | D | A | G | A | P | A | A | F | L | L | A | L | R | H | P | D | - | L | V | R | R |
| 147 UniRef90\_A0A2D6SJL3\_11\_235 | K | K | T | V | A | N | D | V | W | R | L | M | S | E | V | L | G | F | S | - | - | - | T | F | F | V | V | G | H | D | W | G | G | P | T | A | Y | A | L | A | A | A | H | P | D | - | A | V | T | K |
| 148 UniRef90\_A0A2V6J615\_3\_225 | M | L | E | A | A | K | Q | I | H | D | L | V | R | - | S | L | K | I | D | - | - | - | K | A | R | V | V | G | H | D | I | G | L | M | V | A | Y | A | Y | A | A | Q | F | P | G | - | E | T | E | K |

  
  

|  |  |  |  |  |  |  |  |  |  |  |  |  |  |  |  |  |  |  |  |  |  |  |  |  |  |  |  |  |  |  |  |  |  |  |  |  |  |  |  |  |  |  |  |  |  |  |  |  |  |  |
| --- | --- | --- | --- | --- | --- | --- | --- | --- | --- | --- | --- | --- | --- | --- | --- | --- | --- | --- | --- | --- | --- | --- | --- | --- | --- | --- | --- | --- | --- | --- | --- | --- | --- | --- | --- | --- | --- | --- | --- | --- | --- | --- | --- | --- | --- | --- | --- | --- | --- | --- |
| **001 Input\_protein\_seq** | A | V | F | M | D | S | P | I | P | D | R | A | - | M | F | E | Y | P | G | Y | T | - | - | A | D | G | P | G | - | L | G | W | H | F | G | Y | F | S | F | G | - | D | I | A | E | K | Q | I | A | N |
| 002 UniRef90\_D8JE57\_1\_349 | V | V | F | M | D | S | P | I | P | D | M | E | - | T | F | H | H | P | G | Y | S | - | - | P | K | G | P | G | - | L | G | W | H | F | G | Y | F | S | F | G | - | D | I | A | E | K | Q | I | A | N |
| 003 UniRef90\_A0A6B8MSH7\_6\_345 | V | V | F | M | D | S | P | I | P | D | K | S | - | M | F | E | Y | A | G | Y | T | - | - | P | S | G | P | G | - | L | G | W | H | F | G | Y | F | S | F | G | - | D | I | A | E | K | Q | I | A | S |
| 004 UniRef90\_A0A6B2FNS7\_11\_352 | A | V | F | M | D | S | P | I | P | D | R | A | - | M | F | T | Y | P | G | Y | T | - | - | P | Q | G | P | G | - | L | G | W | H | F | G | Y | F | S | F | G | - | D | I | A | E | K | Q | I | A | A |
| 005 UniRef90\_UPI00073D803C\_6\_344 | V | V | F | M | D | S | P | I | P | D | K | A | - | M | F | E | Y | P | G | Y | T | - | - | P | S | G | P | G | - | L | G | W | H | F | G | Y | F | S | F | G | - | D | I | A | E | K | Q | V | G | S |
| 006 UniRef90\_A0A285K5K8\_3\_346 | V | V | F | M | D | S | P | I | P | D | N | A | - | M | F | A | Y | P | G | Y | T | - | - | P | G | G | P | G | - | L | G | W | H | F | G | Y | F | S | F | G | - | D | I | A | E | K | Q | I | A | Q |
| 007 UniRef90\_A0A379I9D6\_1\_346 | V | V | F | M | D | S | P | I | P | D | R | E | - | M | F | T | Y | P | G | Y | T | - | - | S | S | G | P | G | - | L | G | W | H | F | G | Y | F | S | F | G | - | D | I | A | E | K | Q | I | A | A |
| 008 UniRef90\_A0A6B2FTI5\_15\_352 | V | V | F | M | D | S | P | I | P | D | R | A | - | M | Y | T | Y | P | G | Y | T | - | - | P | N | G | P | G | - | L | G | W | H | F | G | Y | F | S | F | G | - | D | I | A | E | K | Q | I | S | A |
| 009 UniRef90\_A0A2P9HD85\_2\_318 | V | V | F | M | D | S | P | I | P | D | K | A | - | M | W | T | Y | P | G | L | T | - | - | P | N | G | P | G | - | L | G | W | H | F | G | Y | F | S | F | G | - | D | I | A | E | K | M | V | S | T |
| 010 UniRef90\_A0A1T1H7K2\_15\_316 | L | V | F | M | E | A | P | I | P | D | K | S | - | M | Y | E | F | P | A | F | S | - | - | E | E | G | E | S | - | L | V | W | H | F | S | F | F | A | A | K | Q | N | L | A | E | T | L | I | K | G |
| 011 UniRef90\_A0A1A9HKQ2\_27\_313 | V | V | Y | M | E | A | P | I | P | D | E | S | - | M | Y | T | F | P | A | F | T | - | - | A | E | G | E | S | - | L | V | W | H | F | S | F | F | A | A | G | G | N | M | A | E | K | L | I | A | G |
| 012 UniRef90\_A0A0J6GRA0\_22\_303 | L | V | Y | M | E | A | P | I | P | D | K | R | - | I | Y | D | F | P | A | F | S | - | - | P | Q | G | E | S | - | L | V | W | H | F | S | F | F | A | A | G | N | Q | L | A | E | K | L | I | T | G |
| 013 UniRef90\_UPI000F5BD18A\_25\_314 | A | V | Y | M | E | A | P | I | P | D | D | S | - | I | Y | D | F | P | A | F | T | - | - | P | K | G | E | S | - | L | V | W | H | F | S | F | F | D | A | D | N | D | L | A | Q | N | L | I | A | G |
| 014 UniRef90\_A0A4V1G750\_26\_311 | A | V | Y | M | E | A | P | I | P | D | D | S | - | I | Y | E | F | P | A | F | T | - | - | P | E | G | E | S | - | L | V | W | H | F | S | F | F | A | A | D | G | Q | L | A | E | K | L | I | T | G |
| 015 UniRef90\_U3U231\_21\_300 | A | I | F | M | E | A | P | I | P | D | E | S | - | I | Y | Q | F | P | A | F | T | - | - | P | E | G | E | S | - | L | V | W | H | F | S | F | F | A | A | Q | G | Q | L | A | E | Q | L | I | A | G |
| 016 UniRef90\_F2LPZ2\_26\_313 | A | V | Y | M | E | A | P | I | P | D | E | S | - | I | Y | S | F | P | A | F | T | - | - | P | Q | G | E | S | - | L | V | W | H | F | S | F | F | A | A | D | E | D | L | A | E | T | L | I | A | G |
| 017 UniRef90\_A0A3M4V4S1\_6\_330 | A | A | F | I | E | A | T | I | P | D | D | T | - | L | Y | S | L | P | A | F | V | - | - | A | T | G | E | A | - | P | G | W | H | H | S | F | F | A | A | S | G | Q | L | A | D | A | M | V | K | G |
| 018 UniRef90\_A0A178LP42\_49\_332 | A | V | F | M | E | A | P | L | P | D | E | G | - | I | Y | T | F | P | A | F | T | - | - | P | Q | G | E | S | - | P | M | W | H | F | S | F | F | A | A | G | D | N | L | A | E | T | M | V | A | G |
| 019 UniRef90\_T2L220\_20\_307 | V | I | Y | M | E | A | P | I | P | G | D | S | - | I | Y | S | F | P | A | F | T | - | - | P | E | G | E | S | - | L | V | W | H | F | S | F | F | A | A | E | Q | Q | L | A | E | K | L | V | S | G |
| 020 UniRef90\_UPI0014749FF6\_22\_304 | V | A | Y | L | E | A | V | I | P | D | S | R | - | I | Y | G | F | P | A | Y | T | - | - | P | Q | G | E | S | - | T | A | W | H | F | S | F | F | S | A | D | G | A | L | P | E | T | L | I | S | G |
| 021 UniRef90\_A0A172YJZ3\_18\_305 | A | I | Y | M | E | A | P | I | P | D | D | S | - | I | Y | S | F | P | A | F | T | - | - | P | Q | G | E | S | - | L | V | W | H | F | S | F | F | A | A | G | E | R | L | A | E | T | L | I | A | G |
| 022 UniRef90\_UPI000737C6BF\_26\_313 | V | I | Y | M | E | A | P | I | P | D | E | S | - | I | Y | T | F | P | A | F | T | - | - | A | Q | G | E | S | - | L | V | W | H | F | S | F | F | A | A | E | G | R | L | A | E | T | L | V | A | G |
| 023 UniRef90\_A0A329J6I4\_26\_309 | L | V | F | M | E | A | P | I | P | D | K | S | - | V | Y | D | F | P | A | F | S | - | - | P | E | G | E | S | - | L | V | W | H | F | S | F | F | A | A | G | D | H | M | A | E | T | L | I | K | G |
| 024 UniRef90\_A0A2N3KZL1\_30\_325 | L | I | F | M | E | A | P | I | P | D | H | R | - | L | Y | D | F | P | A | F | T | - | - | P | E | G | E | S | - | L | V | W | H | F | S | F | F | A | A | G | N | N | L | A | E | T | L | V | T | G |
| 025 UniRef90\_UPI0006CA847F\_27\_323 | V | I | Y | F | D | S | I | I | P | D | D | T | - | I | Y | T | F | P | S | F | T | - | - | P | E | G | E | A | - | S | T | W | H | W | S | F | Y | T | A | E | N | N | F | A | E | N | I | I | E | G |
| 026 UniRef90\_UPI00054E0E46\_26\_312 | V | V | F | M | E | A | P | I | P | D | E | S | - | L | Y | S | F | P | A | F | T | - | - | S | E | G | E | S | - | L | V | W | H | F | S | F | F | T | A | R | N | D | L | A | E | T | L | I | A | G |
| 027 UniRef90\_A0A0S9PRE2\_29\_311 | V | V | F | M | E | A | P | I | P | D | D | G | - | L | Y | A | Y | P | A | F | T | - | - | P | Q | G | E | S | - | L | V | W | H | F | S | F | F | T | A | A | N | D | L | A | R | T | L | I | A | G |
| 028 UniRef90\_W0AA74\_37\_318 | V | V | F | M | E | A | P | L | P | D | E | G | - | L | Y | A | F | P | S | F | T | - | - | P | Q | G | E | S | - | P | M | W | H | F | S | F | F | A | A | P | G | N | F | A | E | K | M | V | A | G |
| 029 UniRef90\_A0A2G0Y5Q3\_27\_312 | L | V | Y | M | E | A | P | I | P | D | A | K | - | A | Y | D | F | P | A | F | T | - | - | P | E | G | E | S | - | L | V | W | H | F | S | F | F | A | A | N | G | K | L | A | E | T | L | I | T | G |
| 030 UniRef90\_A0A1M6LZ92\_62\_349 | A | V | Y | M | E | A | P | I | P | D | D | S | - | I | Y | S | F | P | A | F | T | - | - | S | K | G | E | S | - | P | V | W | H | F | S | F | F | D | A | D | G | Q | L | A | E | T | L | I | T | G |
| 031 UniRef90\_A0A4Z1C7D3\_28\_313 | L | V | F | M | E | A | P | I | P | D | D | N | - | I | Y | D | F | P | A | F | T | - | - | P | Q | G | E | S | - | L | V | W | H | F | S | F | F | A | A | S | E | N | L | A | E | T | L | V | T | G |
| 032 UniRef90\_A0A367MBG0\_273\_561 | L | V | Y | M | E | A | P | I | P | D | A | R | - | I | Y | R | F | P | A | F | T | - | - | A | Q | G | E | S | - | L | V | W | H | F | S | F | F | A | A | D | D | R | L | A | E | T | L | I | A | G |
| 033 UniRef90\_UPI00067CEA54\_35\_318 | V | V | F | M | E | A | P | L | P | D | E | G | - | I | Y | T | F | P | S | F | T | - | - | P | Q | G | E | S | - | P | M | W | H | F | S | F | F | A | A | G | N | N | L | A | E | T | L | V | A | G |
| 034 UniRef90\_A0A1Y0L5M5\_31\_311 | L | I | F | M | E | A | P | I | P | D | K | R | - | M | Y | D | F | P | A | F | S | - | - | P | E | G | E | S | - | L | V | W | H | F | S | F | F | A | A | K | Q | Q | L | A | E | T | L | I | R | G |
| 035 UniRef90\_UPI000C2FE79C\_29\_313 | A | V | Y | M | E | A | P | I | P | D | D | S | - | I | Y | K | F | A | A | F | S | - | - | P | K | G | E | S | - | L | V | W | H | F | S | F | F | S | A | D | N | Q | L | A | E | T | L | I | K | G |
| 036 UniRef90\_A0A4P8SJX7\_14\_295 | L | V | Y | M | E | A | P | I | P | D | K | R | - | M | Y | D | F | P | A | F | S | - | - | L | E | G | E | S | - | L | V | W | H | F | S | F | F | A | A | K | N | K | L | A | E | T | L | I | K | G |
| 037 UniRef90\_A0A1I1BZT3\_28\_315 | L | V | F | M | E | A | P | I | P | D | D | R | - | I | Y | A | F | P | A | F | T | - | - | P | E | G | E | S | - | L | V | W | H | F | S | F | F | A | A | A | G | N | L | A | E | T | L | V | T | G |
| 038 UniRef90\_UPI000DF0D36E\_15\_290 | L | I | F | M | E | A | P | I | P | D | K | R | - | M | Y | D | F | P | A | F | S | - | - | P | E | G | E | S | - | L | V | W | H | F | S | F | F | A | A | K | Q | Q | L | A | E | K | L | I | K | G |
| 039 UniRef90\_UPI00136B8797\_37\_318 | V | V | F | M | E | A | P | L | P | D | E | G | - | I | Y | T | F | P | S | F | T | - | - | P | Q | G | E | S | - | P | M | W | H | F | S | F | F | A | A | G | G | N | F | A | E | K | L | V | A | G |
| 040 UniRef90\_A0A1H4CLJ1\_28\_313 | V | I | Y | M | E | A | P | I | P | D | E | S | - | I | Y | N | Y | P | A | F | T | - | - | P | Q | G | E | S | - | L | V | W | H | F | S | F | F | A | A | K | G | R | L | A | E | T | L | I | A | G |
| 041 UniRef90\_UPI000E1FE28F\_29\_313 | A | I | Y | M | E | A | P | I | P | D | P | S | - | I | Y | K | F | P | A | F | T | - | - | P | K | G | E | S | - | L | V | W | H | F | S | F | F | A | A | D | D | N | L | A | E | T | L | V | T | G |
| 042 UniRef90\_A0A556AX95\_26\_313 | V | V | Y | M | E | A | P | I | P | D | D | S | - | I | Y | S | F | P | A | F | T | - | - | P | E | G | E | S | - | L | V | W | H | F | S | F | F | A | A | D | Q | R | L | A | E | T | L | I | T | G |
| 043 UniRef90\_A0A6M8HNY7\_38\_322 | L | V | Y | M | E | A | P | I | P | D | D | R | - | L | Y | Q | F | P | A | F | T | - | - | P | R | G | E | S | - | L | V | W | H | F | S | F | F | S | A | N | D | D | L | A | D | R | L | V | T | G |
| 044 UniRef90\_UPI001612B72A\_24\_301 | L | V | Y | M | E | A | P | I | P | D | Q | R | - | L | Y | Q | F | P | A | F | T | - | - | P | E | G | E | S | - | L | V | W | H | F | S | F | F | A | A | G | D | L | L | P | E | R | L | V | S | G |
| 045 UniRef90\_UPI0012F66D09\_46\_328 | L | V | Y | M | E | A | P | I | P | D | E | R | - | L | Y | Q | F | P | A | F | T | - | - | P | Q | G | E | S | - | L | V | W | H | F | S | F | F | A | A | G | N | Q | L | P | E | T | L | V | T | G |
| 046 UniRef90\_UPI000A94940B\_29\_316 | V | V | Y | M | E | A | P | I | P | N | D | T | - | I | Y | S | F | P | A | F | T | - | - | P | Q | G | E | S | - | L | V | W | H | F | S | F | F | A | A | D | Q | R | L | A | E | T | L | I | T | G |
| 047 UniRef90\_UPI00102FACD2\_29\_312 | V | V | F | M | E | A | P | I | P | D | D | T | - | L | Y | A | F | P | A | F | T | - | - | P | Q | G | E | S | - | L | V | W | H | F | S | F | F | T | A | K | N | N | L | A | E | T | L | I | A | G |
| 048 UniRef90\_A0A1S8YNF4\_19\_297 | V | I | Y | M | E | A | P | V | P | D | Q | R | - | M | Y | D | F | P | A | F | S | - | - | P | Q | G | E | S | - | L | V | W | H | F | S | F | F | A | A | Q | N | R | L | A | E | T | L | I | A | G |
| 049 UniRef90\_UPI0015755320\_14\_301 | L | V | Y | M | E | A | P | I | P | D | Q | R | - | I | H | G | F | P | A | F | T | - | - | P | E | G | E | S | - | L | V | W | H | F | S | F | F | A | A | G | N | R | L | A | E | T | L | I | A | G |
| 050 UniRef90\_A0A427Q4N3\_22\_313 | V | I | Y | M | E | A | P | I | P | D | D | S | - | L | Y | Q | F | P | A | F | T | - | - | P | Q | G | E | S | - | L | V | W | H | F | S | F | F | A | A | E | P | N | L | A | E | T | L | I | A | G |
| 051 UniRef90\_A0A0A3Z1S4\_30\_318 | L | I | Y | M | E | A | P | I | P | D | R | R | - | M | Y | D | F | P | A | F | S | - | - | P | E | G | E | S | - | L | V | W | H | F | S | F | F | A | A | K | N | Q | L | A | E | T | L | I | K | G |
| 052 UniRef90\_A0A3L8C981\_35\_323 | A | A | F | I | E | A | T | I | P | D | D | T | - | L | Y | S | L | P | A | F | V | - | - | A | T | G | E | A | - | P | G | W | H | H | S | F | F | A | A | S | D | Q | L | A | D | A | M | V | K | G |
| 053 UniRef90\_A0A6L5BLL6\_28\_324 | A | A | F | L | E | A | T | I | P | D | D | T | - | L | Y | S | L | P | A | F | V | - | - | S | T | G | E | A | - | P | G | W | H | H | S | F | F | A | A | S | D | Q | L | A | D | A | M | V | K | G |
| 054 UniRef90\_A0A6B1J288\_27\_314 | A | V | Y | M | E | A | P | I | P | E | E | S | - | I | Y | S | F | P | A | F | T | - | - | P | E | G | E | S | - | L | V | W | H | F | S | F | F | A | A | Q | N | R | L | A | E | T | L | I | A | G |
| 055 UniRef90\_A0A263NNG9\_22\_274 | V | A | Y | L | E | S | V | I | P | D | E | L | - | M | Y | N | F | P | A | F | T | - | - | P | Q | G | E | S | - | T | A | W | H | F | S | F | F | S | A | D | D | K | L | A | E | T | L | I | A | G |
| 056 UniRef90\_W0HXQ2\_33\_307 | V | V | F | M | E | A | P | I | P | D | K | R | - | L | Y | D | F | P | A | F | S | - | - | P | Q | G | E | S | - | L | V | W | H | F | S | F | F | A | A | G | D | N | L | A | E | T | L | I | A | G |
| 057 UniRef90\_A0A031HKS8\_40\_323 | V | V | F | M | E | A | P | I | P | D | D | R | - | L | Y | Q | F | P | A | F | T | - | - | P | E | G | E | S | - | L | V | W | H | F | S | F | F | A | A | D | N | L | L | P | E | R | L | V | R | G |
| 058 UniRef90\_A0A4R2FY05\_27\_320 | V | A | L | L | E | T | I | V | P | S | D | L | - | I | Y | T | F | P | A | Y | T | - | - | P | Q | G | E | S | - | T | A | W | H | F | S | F | F | S | A | K | N | E | L | A | E | S | L | I | S | G |
| 059 UniRef90\_A0A1X0N2L9\_36\_321 | A | A | F | I | E | A | T | I | P | D | D | T | - | L | Y | S | L | P | A | F | V | - | - | A | T | G | E | A | - | P | G | W | H | H | S | F | F | A | A | S | D | Q | L | A | D | A | M | V | K | G |
| 060 UniRef90\_A0A4R1U0K9\_37\_322 | A | A | F | I | E | A | T | V | P | D | D | T | - | L | Y | S | L | P | A | F | V | - | - | A | T | G | E | A | - | A | G | W | H | H | S | F | F | A | A | D | D | Q | L | A | D | A | M | V | K | G |
| 061 UniRef90\_A0A0Q8TEQ0\_42\_323 | V | A | F | M | E | A | P | I | P | D | E | G | - | L | Y | G | W | P | A | F | T | - | - | D | K | G | A | S | - | L | A | W | H | F | S | F | F | N | L | A | A | P | L | P | E | M | M | L | A | G |
| 062 UniRef90\_A0A0Q6ZEJ0\_41\_323 | L | I | Y | M | E | A | P | I | P | D | E | G | - | L | Y | T | W | P | A | F | T | - | - | D | K | G | A | S | - | L | A | W | H | F | S | F | F | N | L | A | A | P | L | P | E | K | M | L | A | G |
| 063 UniRef90\_UPI0004C0CBB1\_54\_338 | M | A | V | M | E | A | P | I | P | D | E | S | - | I | Y | T | Y | P | A | L | E | - | A | D | P | N | K | P | - | S | A | W | H | F | G | M | F | Q | L | P | - | - | L | A | E | Q | L | I | T | G |
| 064 UniRef90\_UPI0004CAB4F8\_56\_339 | M | A | V | M | E | A | P | I | P | D | D | S | - | L | Y | S | F | P | A | L | E | - | A | N | P | D | T | P | - | S | M | W | H | F | G | L | F | Q | L | P | - | - | L | A | E | Q | L | I | A | G |
| 065 UniRef90\_UPI00049002CC\_60\_343 | V | A | V | M | E | A | P | I | A | D | D | R | - | A | F | T | Y | P | A | L | N | - | A | D | P | K | K | P | - | S | P | W | H | W | G | M | F | Q | L | P | - | - | L | A | E | H | L | I | A | G |
| 066 UniRef90\_A0A561IX74\_59\_327 | L | A | L | M | D | A | L | L | P | G | V | E | - | P | V | W | - | - | - | - | - | - | - | S | Q | V | K | S | - | A | A | W | W | F | G | F | F | S | F | P | - | - | A | S | G | E | L | V | A | G |
| 067 UniRef90\_A0A0X3RYG7\_67\_350 | M | A | V | M | E | A | P | V | P | D | K | S | - | I | Y | T | Y | A | A | L | N | - | A | D | P | N | R | P | - | S | P | W | H | F | G | L | F | Q | M | P | - | - | L | A | E | K | L | I | A | G |
| 068 UniRef90\_A0A2W2E1M5\_55\_338 | M | A | V | M | E | A | P | I | P | D | E | S | - | V | Y | S | Y | P | V | L | N | - | A | D | P | N | K | P | - | A | P | W | H | F | G | L | F | Q | L | P | - | - | L | A | E | H | L | I | A | G |
| 069 UniRef90\_A0A2V7YB42\_28\_306 | L | V | L | M | D | A | F | L | P | G | V | D | - | E | W | E | T | - | - | - | - | - | - | V | Y | D | N | P | - | A | I | W | H | F | R | F | H | G | - | - | - | P | T | P | E | A | L | V | Q | G |
| 070 UniRef90\_A0A544XC49\_52\_336 | M | A | V | M | E | A | P | I | P | D | K | S | - | I | Y | G | Y | P | V | L | N | - | A | D | P | N | K | P | - | A | A | W | H | F | G | L | F | Q | L | P | - | - | L | A | E | R | L | I | A | G |
| 071 UniRef90\_A0A101PQ90\_46\_324 | L | V | L | S | E | A | P | I | P | D | P | G | - | I | Y | K | F | P | A | L | - | - | - | T | A | K | G | P | - | G | V | W | N | F | G | F | F | S | L | Q | T | G | L | P | E | S | L | I | Q | G |
| 072 UniRef90\_A0A1C4VK67\_14\_288 | L | V | L | S | E | A | P | I | P | D | E | G | - | I | Y | A | L | P | A | L | - | - | - | T | A | V | G | P | - | G | V | W | N | F | G | F | F | S | L | G | N | G | L | P | E | Q | I | V | T | G |
| 073 UniRef90\_A0A0X3RPC2\_57\_340 | M | A | V | M | E | A | P | I | P | D | D | S | - | V | F | S | F | P | V | L | N | - | A | D | P | N | K | P | - | A | A | W | H | F | G | L | F | Q | L | P | - | - | L | A | E | H | L | I | A | G |
| 074 UniRef90\_A0A4R2VWW4\_55\_319 | L | A | L | M | D | A | L | L | P | G | V | E | - | P | V | W | - | - | - | - | - | - | - | S | Q | V | K | G | - | S | A | W | W | F | G | F | F | S | F | P | - | - | H | S | G | E | L | V | A | G |
| 075 UniRef90\_A0A1Q7VB11\_36\_307 | L | V | V | M | D | A | F | L | P | G | V | P | - | G | W | E | A | - | - | - | - | - | - | V | Y | N | N | P | - | A | I | W | H | F | R | F | N | G | - | - | - | P | T | P | E | E | L | V | R | G |
| 076 UniRef90\_C7PGQ3\_38\_305 | L | A | L | M | D | A | L | L | P | G | V | E | - | P | V | W | - | - | - | - | - | - | - | S | Q | V | K | G | - | A | A | W | W | F | G | F | F | S | F | P | - | - | A | S | G | E | L | V | A | G |
| 077 UniRef90\_A0A1N7R4E6\_62\_325 | L | A | L | M | D | A | L | L | P | G | V | E | - | P | V | W | - | - | - | - | - | - | - | S | Q | V | K | G | - | A | A | W | W | F | G | F | F | S | F | P | - | - | A | S | G | E | L | V | A | G |
| 078 UniRef90\_A0A2H2Y4V3\_41\_307 | L | V | I | L | E | S | T | I | P | G | F | E | - | A | Q | D | T | - | - | - | - | - | - | T | G | R | L | S | - | K | V | W | H | P | A | F | H | Q | E | P | - | D | L | P | E | A | L | V | T | G |
| 079 UniRef90\_A0A448SCU6\_8\_276 | L | V | F | L | E | S | K | I | L | G | I | E | - | S | E | - | - | - | - | - | - | - | - | D | D | A | E | K | - | E | Y | W | H | F | G | F | H | Q | A | P | - | D | L | A | Q | Q | L | V | S | G |
| 080 UniRef90\_A0A365Y5E2\_33\_303 | L | V | L | M | D | A | L | L | P | G | V | E | - | P | V | W | - | - | - | - | - | - | - | S | Q | V | R | G | - | A | A | W | W | F | G | F | F | A | W | P | - | - | T | S | P | D | I | V | E | G |
| 081 UniRef90\_A0A1I2CFD2\_63\_327 | L | A | L | M | D | A | L | L | P | G | V | E | - | P | V | W | - | - | - | - | - | - | - | S | Q | V | K | A | - | S | A | W | W | F | G | F | F | S | F | P | - | - | A | S | G | E | L | V | A | G |
| 082 UniRef90\_A0A1G7Z850\_54\_322 | L | A | L | M | D | A | L | L | P | G | V | E | - | P | V | W | - | - | - | - | - | - | - | S | Q | V | K | G | - | A | A | W | W | F | G | F | F | S | F | P | - | - | A | S | G | E | L | V | A | G |
| 083 UniRef90\_A0A2V9XG70\_35\_308 | L | V | V | M | D | A | F | L | P | G | V | G | - | E | W | E | T | - | - | - | - | - | - | I | Y | N | D | P | - | G | F | W | H | F | R | F | N | G | - | - | - | P | T | P | Q | A | L | V | Q | G |
| 084 UniRef90\_A0A0Q4GSS4\_39\_308 | L | A | L | L | D | A | L | L | P | G | I | E | - | P | V | W | - | - | - | - | - | - | - | S | Q | V | K | S | - | Q | A | W | W | F | G | F | F | A | Q | P | - | - | N | S | G | K | L | V | S | G |
| 085 UniRef90\_A0A0M9ZTU1\_1\_271 | M | A | V | M | E | A | P | I | A | D | D | G | - | I | Y | R | F | P | A | V | N | - | A | D | P | K | K | P | - | S | P | W | H | W | G | M | F | L | Q | P | - | - | S | A | E | R | L | I | S | G |
| 086 UniRef90\_A0A239P597\_1\_270 | M | A | V | M | E | A | P | I | P | D | Q | S | - | I | Y | H | Y | P | V | L | N | - | A | D | P | N | K | P | - | A | A | W | H | F | G | L | F | Q | L | P | - | - | L | A | E | K | L | I | A | G |
| 087 UniRef90\_UPI00128E3D53\_19\_294 | L | V | M | S | D | C | Q | V | P | G | K | E | - | N | P | - | - | - | - | - | - | - | - | N | T | L | D | G | - | R | G | W | H | F | G | F | H | Q | A | E | - | N | F | P | E | M | L | T | E | G |
| 088 UniRef90\_A0A1V9E1W0\_57\_321 | L | A | L | M | D | A | L | L | P | G | I | E | - | P | V | W | - | - | - | - | - | - | - | T | Q | V | K | G | - | S | A | W | W | F | G | F | F | S | F | P | - | - | A | S | G | E | L | V | A | G |
| 089 UniRef90\_A0A239MTD5\_1\_269 | M | A | V | M | E | A | P | I | A | D | G | S | - | A | Y | S | Y | P | A | L | N | - | A | D | P | E | K | P | - | S | P | W | H | W | G | M | F | Q | M | P | - | - | L | A | E | H | L | I | A | G |
| 090 UniRef90\_UPI0009FCBBE8\_54\_317 | M | A | V | L | E | A | P | L | N | G | F | G | - | L | E | G | A | Y | G | - | - | - | - | - | - | - | - | - | - | L | S | W | H | F | M | F | N | M | T | G | N | P | V | P | E | Q | L | I | N | D |
| 091 UniRef90\_A0A1A9HTL2\_19\_299 | L | T | V | M | E | A | A | L | P | G | I | T | - | P | P | A | P | A | G | I | P | - | G | D | D | A | N | L | - | K | S | W | H | F | A | F | N | R | L | P | - | D | L | P | E | I | L | V | Q | G |
| 092 UniRef90\_A0A4R7CM41\_23\_285 | L | A | L | M | D | A | I | L | P | G | V | E | - | P | V | W | - | - | - | - | - | - | - | S | Q | K | K | S | - | Q | A | W | W | F | G | F | F | A | W | P | - | - | V | S | A | R | L | V | A | G |
| 093 UniRef90\_A0A562QYK6\_25\_307 | L | V | L | M | D | A | F | L | P | G | I | G | - | N | W | R | D | - | - | - | - | - | - | V | W | L | L | R | - | D | L | W | H | F | H | F | Y | G | - | - | - | P | T | P | L | A | L | V | A | G |
| 094 UniRef90\_A0A2V9ZM06\_3\_269 | L | V | V | M | D | A | F | L | P | G | V | A | - | G | W | E | A | - | - | - | - | - | - | V | Y | N | N | P | - | S | I | W | H | F | R | F | N | G | - | - | - | P | T | P | E | A | L | V | Q | G |
| 095 UniRef90\_A0A4P8YF40\_11\_271 | L | V | F | L | E | S | K | I | L | G | I | E | - | S | E | - | - | - | - | - | - | - | - | D | D | A | E | K | - | E | Y | W | H | F | G | F | H | Q | A | P | - | D | L | A | Q | L | L | V | T | G |
| 096 UniRef90\_A0A2V6E8I4\_30\_303 | L | V | V | M | D | A | F | L | P | G | V | A | - | G | W | E | P | - | - | - | - | - | - | I | Y | N | D | P | - | N | I | W | H | F | R | F | H | G | - | - | - | P | T | P | E | A | L | V | K | G |
| 097 UniRef90\_A0A2V9BFT0\_23\_295 | L | V | V | M | D | A | F | L | P | G | V | P | - | G | W | E | A | - | - | - | - | - | - | I | Y | N | N | P | - | N | I | W | H | F | R | F | N | G | - | - | - | P | T | P | E | A | L | V | H | G |
| 098 UniRef90\_A0A2V9H5C8\_29\_298 | L | V | V | M | D | A | F | L | P | G | V | G | - | E | W | E | A | - | - | - | - | - | - | V | Y | N | N | P | - | G | F | W | H | F | R | F | H | G | - | - | - | P | T | P | E | A | L | V | E | G |
| 099 UniRef90\_A0A101CRV3\_44\_308 | L | A | L | M | D | A | L | L | P | G | V | E | - | P | V | W | - | - | - | - | - | - | - | S | Q | V | S | A | - | S | A | W | W | F | G | F | F | S | W | P | - | - | S | S | G | A | I | V | E | G |
| 100 UniRef90\_A0A5M6CFP9\_57\_321 | L | A | L | M | D | A | L | L | P | G | I | E | - | P | V | W | - | - | - | - | - | - | - | S | Q | V | K | G | - | S | A | W | W | F | G | F | F | G | W | K | - | - | A | S | G | E | I | V | A | G |
| 101 UniRef90\_A0A401Z9D4\_19\_284 | L | V | I | L | D | A | P | L | E | G | F | G | - | R | E | E | - | - | - | - | - | - | - | Y | A | A | K | N | - | S | V | W | H | Y | G | L | F | Q | A | P | R | S | L | A | E | S | L | V | E | G |
| 102 UniRef90\_A0A2V8XJ58\_32\_308 | L | V | V | M | D | A | F | L | P | G | V | G | - | E | W | G | T | - | - | - | - | - | - | V | Y | N | N | P | - | A | I | W | H | F | R | F | N | G | - | - | - | P | T | P | E | A | L | V | H | G |
| 103 UniRef90\_A0A252EMP1\_15\_277 | L | V | F | L | E | S | K | I | L | G | I | E | - | S | D | - | - | - | - | - | - | - | - | D | D | A | S | K | - | E | Y | W | H | F | G | F | H | Q | E | A | - | D | L | P | E | A | L | L | A | G |
| 104 UniRef90\_A0A1H3H759\_34\_310 | L | A | L | A | E | A | V | I | P | G | L | A | - | P | S | P | - | P | L | F | A | - | P | Q | E | A | V | D | - | R | L | W | H | F | G | F | N | R | L | G | - | S | L | N | E | E | L | V | A | G |
| 105 UniRef90\_A0A4D7B7N1\_3\_270 | L | V | L | L | D | A | N | I | P | G | V | T | - | L | R | P | T | V | D | V | - | - | - | G | P | D | N | W | - | K | S | W | H | F | F | F | H | P | V | P | - | D | L | P | E | A | L | I | T | G |
| 106 UniRef90\_A0A1Q7MUT6\_34\_308 | L | V | V | M | D | A | F | L | P | G | V | A | - | G | W | G | A | - | - | - | - | - | - | I | Y | N | N | P | - | D | F | W | H | F | R | F | H | G | - | - | - | P | T | P | E | A | L | V | Q | G |
| 107 UniRef90\_A0A2V6KQW0\_24\_297 | L | V | V | M | D | A | F | L | P | G | V | P | - | G | W | E | A | - | - | - | - | - | - | I | Y | N | A | P | - | T | V | W | H | F | R | F | N | G | - | - | - | E | Y | P | E | A | L | V | K | G |
| 108 UniRef90\_A0A4R6LFZ5\_44\_308 | L | A | L | M | D | A | L | L | P | G | V | E | - | P | V | W | - | - | - | - | - | - | - | S | Q | V | S | A | - | S | A | W | W | F | G | F | F | S | W | P | - | - | S | S | G | A | I | V | N | G |
| 109 UniRef90\_A0A1H7UU25\_49\_318 | V | A | L | L | D | A | L | L | P | G | V | E | - | P | V | W | - | - | - | - | - | - | - | S | E | V | R | A | - | K | A | W | W | F | G | F | F | A | L | P | - | - | A | S | A | E | V | V | A | G |
| 110 UniRef90\_UPI0008326796\_14\_259 | L | V | L | M | D | A | G | I | P | G | V | T | - | L | P | E | M | L | P | A | A | - | - | P | D | R | S | W | - | K | T | W | H | F | S | F | H | M | L | P | - | D | L | P | E | A | L | I | A | G |
| 111 UniRef90\_A0A2V5S8F2\_33\_306 | L | V | V | M | D | A | F | L | P | G | V | T | - | G | W | E | P | - | - | - | - | - | - | I | Y | N | D | P | - | A | I | W | H | F | R | F | H | G | - | - | - | P | T | P | E | A | L | V | K | G |
| 112 UniRef90\_A0A3S3VTN8\_36\_308 | I | A | L | M | D | A | L | L | P | G | I | E | - | P | V | W | - | - | - | - | - | - | - | S | Q | V | K | A | - | Q | A | W | W | F | G | F | F | A | Q | P | - | - | H | S | G | E | L | V | S | G |
| 113 UniRef90\_A0A6J4PSU4\_16\_248 | L | A | L | V | D | S | L | P | P | G | L | G | - | F | D | E | L | S | - | - | - | - | - | V | T | E | E | M | - | P | L | W | H | T | G | F | H | T | A | R | - | D | V | A | E | M | L | V | S | G |
| 114 UniRef90\_A0A534ZXC6\_26\_300 | L | V | V | M | D | A | F | L | P | G | V | A | - | G | W | E | R | - | - | - | - | - | - | V | Y | D | D | P | - | G | I | W | H | F | R | F | N | G | - | - | - | P | T | P | E | A | L | V | R | G |
| 115 UniRef90\_A0A2A9KBX9\_78\_358 | L | V | V | L | E | A | A | L | P | G | I | T | - | P | P | A | P | A | G | V | P | - | S | D | N | A | N | T | - | R | T | W | H | F | G | F | N | R | L | D | - | D | L | P | E | I | L | V | Q | G |
| 116 UniRef90\_A0A2W0BWH7\_16\_287 | L | V | V | M | D | A | F | L | P | G | V | G | - | G | W | E | E | - | - | - | - | - | - | V | Y | N | N | P | - | A | I | W | H | F | R | F | N | G | - | - | - | P | T | P | E | A | L | V | Q | G |
| 117 UniRef90\_A0A1R1RMU1\_30\_310 | L | A | L | M | D | A | G | I | P | G | I | T | - | L | P | D | A | L | P | T | A | - | - | P | E | H | A | W | - | R | T | W | H | F | A | F | H | S | V | P | - | D | L | P | E | L | L | I | A | G |
| 118 UniRef90\_A0A3A1NH59\_38\_305 | I | A | L | L | D | A | L | L | P | G | V | E | - | P | V | W | - | - | - | - | - | - | - | Q | Q | L | S | T | - | T | V | W | H | F | G | F | F | S | R | P | - | - | V | A | G | E | V | V | A | G |
| 119 UniRef90\_A0A0Q6WP84\_43\_317 | V | A | L | M | D | A | F | L | P | G | I | G | - | N | W | T | N | - | - | - | - | - | - | M | F | L | L | R | - | D | L | W | H | F | H | F | F | G | - | - | - | E | T | P | E | K | L | V | A | G |
| 120 UniRef90\_A0A2V6P1A8\_15\_289 | L | A | V | M | D | A | F | L | P | G | V | P | - | G | W | E | P | - | - | - | - | - | - | I | Y | N | S | P | - | N | V | W | H | F | R | F | N | G | - | - | - | E | Y | P | E | A | L | V | K | G |
| 121 UniRef90\_A0A2V5NC55\_35\_307 | L | V | L | M | D | A | F | L | P | G | V | P | - | G | W | E | P | - | - | - | - | - | - | I | Y | N | N | P | - | N | I | W | H | F | R | F | N | G | - | - | - | E | Y | P | E | A | L | V | Q | G |
| 122 UniRef90\_A0A2V9BM40\_39\_318 | L | V | V | M | D | A | F | L | P | G | V | A | - | G | W | E | N | - | - | - | - | - | - | V | Y | N | N | P | - | G | I | W | H | F | R | F | N | G | - | - | - | P | T | P | E | A | L | V | R | G |
| 123 UniRef90\_A0A5B8WA72\_34\_302 | L | A | L | M | D | A | L | L | P | G | I | E | - | P | V | W | - | - | - | - | - | - | - | S | Q | V | K | A | - | Q | A | W | W | F | G | F | F | A | Q | P | - | - | H | A | G | E | L | V | S | G |
| 124 UniRef90\_A0A1Y6D2S0\_25\_299 | L | V | L | V | D | C | M | P | P | G | T | E | - | N | M | - | - | - | - | - | - | - | - | D | S | A | K | G | - | G | M | W | H | Y | G | F | H | M | A | A | - | G | F | P | E | M | L | T | K | N |
| 125 UniRef90\_A0A494W0L3\_12\_284 | V | A | L | M | D | A | L | L | P | G | I | E | - | P | V | W | - | - | - | - | - | - | - | S | Q | V | R | A | - | Q | A | W | W | F | G | F | F | S | Q | P | - | - | H | S | G | E | I | V | A | G |
| 126 UniRef90\_UPI0015CD0065\_39\_309 | I | A | V | I | E | T | P | L | A | G | F | G | - | L | E | D | L | Y | G | - | - | - | - | - | - | - | - | - | - | I | S | W | H | F | R | F | N | M | S | P | A | P | I | P | E | T | I | M | D | N |
| 127 UniRef90\_UPI00166E2F5D\_15\_289 | A | V | I | M | N | V | V | I | P | G | I | P | - | P | W | D | T | - | - | - | - | - | - | V | K | R | N | P | - | Y | I | W | H | F | A | F | H | A | I | P | - | Q | L | P | E | K | L | V | A | G |
| 128 UniRef90\_A0A372NPX1\_36\_305 | L | A | L | M | D | A | L | L | P | G | I | E | - | P | V | W | - | - | - | - | - | - | - | S | Q | V | K | A | - | Q | A | W | W | F | G | F | F | A | Q | P | - | - | H | A | G | E | L | V | A | G |
| 129 UniRef90\_A0A317JA08\_13\_279 | L | V | V | M | D | A | F | L | P | G | V | A | - | G | W | E | L | - | - | - | - | - | - | A | Y | N | N | D | - | N | L | W | H | F | R | F | H | G | - | - | - | P | A | P | E | A | L | V | K | G |
| 130 UniRef90\_A0A2V6N4R6\_10\_283 | L | V | V | M | D | A | F | L | P | G | V | A | - | G | W | E | P | - | - | - | - | - | - | I | Y | N | A | P | - | N | I | W | H | F | R | F | N | G | - | - | - | E | Y | P | E | K | L | V | Q | G |
| 131 UniRef90\_A0A2V5IZ05\_29\_302 | L | V | L | M | D | A | F | L | P | G | V | T | - | D | W | E | A | - | - | - | - | - | - | V | Y | N | N | P | - | R | I | W | H | F | R | F | N | G | - | - | - | P | T | P | E | A | L | V | R | G |
| 132 UniRef90\_A0A521E7V8\_39\_308 | L | A | L | M | D | A | L | L | P | G | I | E | - | P | V | W | - | - | - | - | - | - | - | S | Q | V | K | A | - | Q | A | W | W | F | G | F | F | G | Q | P | - | - | H | S | G | E | L | V | S | G |
| 133 UniRef90\_UPI001665F59F\_18\_290 | A | V | I | M | N | V | V | I | P | G | V | E | - | P | W | D | T | - | - | - | - | - | - | V | K | S | N | P | - | Y | I | W | H | F | A | F | H | S | I | P | - | E | L | P | E | R | L | V | S | G |
| 134 UniRef90\_A0A0Q9GGK2\_19\_284 | L | V | I | L | D | V | L | I | P | G | L | G | - | L | E | Q | I | M | - | - | - | - | - | D | V | A | N | G | - | G | M | W | H | F | G | F | H | M | V | P | - | D | L | P | E | A | L | I | T | G |
| 135 UniRef90\_A0A329YBZ0\_37\_320 | I | T | M | L | D | V | P | I | P | A | T | A | - | A | W | N | E | A | K | - | - | - | - | H | K | P | D | P | - | E | L | W | H | F | G | L | F | Q | Q | R | - | D | I | A | E | M | V | V | A | G |
| 136 UniRef90\_A0A2E0H9P2\_13\_236 | L | V | I | L | D | V | V | I | P | G | C | G | - | G | D | - | - | - | - | - | - | - | - | F | S | E | G | G | - | R | R | W | H | H | Q | F | H | M | T | A | - | D | L | P | E | D | L | I | S | G |
| 137 UniRef90\_A0A095SYG7\_44\_308 | L | A | L | M | D | A | L | L | P | G | I | E | - | P | V | W | - | - | - | - | - | - | - | S | Q | V | S | A | - | S | A | W | W | F | G | F | F | G | W | S | - | - | A | S | G | D | I | V | A | G |
| 138 UniRef90\_A0A2U0ZNC3\_54\_322 | L | A | L | L | D | A | L | L | P | G | V | E | - | P | V | W | - | - | - | - | - | - | - | S | D | L | Y | N | - | K | L | W | W | F | G | F | F | A | R | P | - | - | V | S | G | E | L | V | A | G |
| 139 UniRef90\_A0A2H2XXU1\_20\_287 | L | V | L | A | E | F | W | L | P | G | F | G | - | L | E | E | G | M | - | - | - | - | - | D | V | A | K | R | - | G | L | W | H | F | G | F | H | M | T | P | - | K | I | P | E | M | L | T | Q | G |
| 140 UniRef90\_A0A0P4V0Y0\_11\_278 | L | T | Y | L | E | E | P | V | L | L | Q | A | H | L | Q | Q | I | F | Q | F | S | P | Q | S | T | Q | N | G | - | G | L | W | W | W | T | F | F | L | A | P | - | E | L | P | E | T | L | I | A | G |
| 141 UniRef90\_A0A2D7FF16\_11\_288 | L | T | I | V | D | V | V | I | P | G | D | G | - | G | D | - | - | - | - | - | - | - | - | F | S | E | G | G | - | R | R | W | H | H | Q | F | H | I | T | P | - | D | L | P | E | A | L | I | T | G |
| 142 UniRef90\_UPI0009FE04F9\_77\_354 | L | L | L | M | D | F | P | L | V | G | K | N | - | L | G | F | - | - | - | - | - | - | - | A | A | V | Q | P | - | L | S | W | H | F | S | F | N | V | Q | N | - | P | L | A | E | N | L | V | T | G |
| 143 UniRef90\_UPI00148816F0\_34\_297 | W | L | M | M | D | F | P | I | V | G | G | S | - | L | R | Y | - | - | - | - | - | - | - | D | Q | V | R | P | - | L | S | F | H | F | A | F | H | E | Q | E | - | P | L | F | E | D | L | V | A | G |
| 144 UniRef90\_A0A2V5MXX8\_31\_304 | L | A | V | M | D | A | F | L | P | G | V | P | - | G | W | E | A | - | - | - | - | - | - | I | Y | N | N | P | - | N | L | W | H | F | R | F | N | G | - | - | - | D | Y | P | E | A | L | V | K | G |
| 145 UniRef90\_Q1W504\_15\_282 | L | V | V | M | E | A | A | I | P | G | L | G | - | L | E | A | L | Q | - | - | - | - | - | D | T | A | R | F | P | H | L | W | H | F | G | F | F | I | A | P | - | G | V | A | E | S | L | V | T | G |
| 146 UniRef90\_UPI001661900D\_10\_259 | L | V | V | M | E | S | L | L | G | A | L | P | - | G | A | E | D | - | - | - | - | - | - | F | L | A | D | G | - | P | P | W | W | F | G | F | H | R | A | A | P | G | L | A | E | T | V | L | E | G |
| 147 UniRef90\_A0A2D6SJL3\_11\_235 | L | V | I | L | D | V | T | X | P | G | C | G | - | G | D | - | - | - | - | - | - | - | - | F | S | E | G | G | - | R | R | W | X | H | Q | F | H | S | T | L | - | D | L | P | E | A | X | I | H | G |
| 148 UniRef90\_A0A2V6J615\_3\_225 | L | A | V | M | D | A | F | L | P | G | V | A | - | G | W | E | P | - | - | - | - | - | - | I | Y | N | N | P | - | N | T | W | H | F | R | F | N | G | - | - | - | E | Y | P | E | K | L | V | K | G |

  
  

|  |  |  |  |  |  |  |  |  |  |  |  |  |  |  |  |  |  |  |  |  |  |  |  |  |  |  |  |  |  |  |  |  |  |  |  |  |  |  |  |  |  |  |  |  |  |  |  |  |  |  |
| --- | --- | --- | --- | --- | --- | --- | --- | --- | --- | --- | --- | --- | --- | --- | --- | --- | --- | --- | --- | --- | --- | --- | --- | --- | --- | --- | --- | --- | --- | --- | --- | --- | --- | --- | --- | --- | --- | --- | --- | --- | --- | --- | --- | --- | --- | --- | --- | --- | --- | --- |
| **001 Input\_protein\_seq** | D | P | - | - | N | L | F | F | S | Y | F | I | K | T | Y | A | - | - | - | - | - | - | - | - | - | G | K | K | - | E | I | F | T | P | E | L | L | A | E | L | I | E | P | Y | S | T | R | - | D | K |
| 002 UniRef90\_D8JE57\_1\_349 | D | P | - | - | N | L | F | L | S | Y | F | I | K | T | Y | A | - | - | - | - | - | - | - | - | - | G | K | K | - | D | I | F | K | P | E | L | L | A | E | L | I | E | P | Y | S | T | R | - | D | K |
| 003 UniRef90\_A0A6B8MSH7\_6\_345 | D | P | - | - | S | L | F | F | S | Y | F | I | K | T | Y | A | - | - | - | - | - | - | - | - | - | G | K | K | - | D | I | F | T | P | Q | L | L | S | E | L | I | E | P | Y | S | T | R | - | E | K |
| 004 UniRef90\_A0A6B2FNS7\_11\_352 | D | P | - | - | S | L | F | F | S | Y | F | I | K | T | Y | A | - | - | - | - | - | - | - | - | - | G | K | K | - | E | I | F | T | P | E | L | L | S | E | L | I | E | P | Y | S | T | R | - | A | R |
| 005 UniRef90\_UPI00073D803C\_6\_344 | D | P | - | - | A | L | F | F | S | Y | F | I | K | T | Y | A | - | - | - | - | - | - | - | - | - | G | K | K | - | D | V | F | T | P | T | L | L | T | E | L | I | E | P | Y | S | T | R | - | E | K |
| 006 UniRef90\_A0A285K5K8\_3\_346 | D | P | - | - | A | L | F | F | S | Y | F | I | K | T | Y | A | - | - | - | - | - | - | - | - | - | G | K | K | - | A | L | F | T | P | E | L | L | S | E | L | I | E | P | Y | S | T | R | - | E | K |
| 007 UniRef90\_A0A379I9D6\_1\_346 | D | P | - | - | N | L | F | F | S | Y | F | I | K | T | Y | A | - | - | - | - | - | - | - | - | - | G | K | K | - | E | L | F | T | P | Q | L | L | S | E | L | I | E | P | Y | S | T | R | - | E | R |
| 008 UniRef90\_A0A6B2FTI5\_15\_352 | D | P | - | - | A | L | F | F | S | Y | F | I | T | T | Y | A | - | - | - | - | - | - | - | - | - | G | K | K | - | E | K | F | T | P | A | L | L | S | E | L | T | E | P | F | S | T | R | - | E | K |
| 009 UniRef90\_A0A2P9HD85\_2\_318 | D | P | - | - | V | L | F | M | S | Y | F | I | K | E | Y | A | - | - | - | - | - | - | - | - | - | G | K | R | - | D | V | F | T | P | E | L | L | D | E | L | I | E | P | Y | S | T | R | - | A | N |
| 010 UniRef90\_A0A1T1H7K2\_15\_316 | H | E | - | - | K | F | F | L | T | H | F | I | H | E | H | A | - | - | - | - | - | - | - | - | - | T | N | K | - | T | V | F | T | P | Q | V | L | D | M | Y | A | K | S | Y | S | K | P | - | H | T |
| 011 UniRef90\_A0A1A9HKQ2\_27\_313 | K | E | - | - | R | F | F | F | E | H | F | I | K | V | H | A | - | - | - | - | - | - | - | - | - | T | N | K | - | E | V | F | T | P | K | L | L | D | L | Y | A | R | S | Y | A | K | P | - | H | S |
| 012 UniRef90\_A0A0J6GRA0\_22\_303 | H | E | - | - | K | L | F | L | S | H | F | I | K | E | H | A | - | - | - | - | - | - | - | - | - | T | N | K | - | E | A | F | T | P | E | L | L | D | L | Y | A | K | S | Y | A | K | P | - | H | T |
| 013 UniRef90\_UPI000F5BD18A\_25\_314 | K | E | - | - | R | L | F | F | E | H | F | I | K | S | H | A | - | - | - | - | - | - | - | - | - | T | N | T | - | S | V | F | T | P | E | L | L | D | L | Y | G | K | S | Y | S | K | P | - | H | S |
| 014 UniRef90\_A0A4V1G750\_26\_311 | K | E | - | - | R | L | F | F | E | H | F | I | K | T | H | A | - | - | - | - | - | - | - | - | - | V | N | K | - | A | V | F | T | P | K | L | L | D | M | Y | A | K | S | Y | A | K | P | - | H | S |
| 015 UniRef90\_U3U231\_21\_300 | K | E | - | - | R | L | F | F | E | H | F | I | K | E | H | A | - | - | - | - | - | - | - | - | - | A | N | K | - | A | V | F | T | P | A | L | L | D | L | Y | S | K | S | Y | A | K | P | - | H | S |
| 016 UniRef90\_F2LPZ2\_26\_313 | K | Q | - | - | R | F | F | L | E | H | F | I | K | Q | H | A | - | - | - | - | - | - | - | - | - | S | N | Q | - | A | V | F | T | P | A | L | L | D | R | Y | A | R | S | Y | S | K | P | - | H | S |
| 017 UniRef90\_A0A3M4V4S1\_6\_330 | N | E | - | - | R | L | F | L | T | H | F | I | R | H | H | A | - | - | - | - | - | - | - | - | - | S | N | Q | - | A | V | F | T | D | E | L | L | E | S | Y | V | H | S | Y | S | K | P | - | Q | T |
| 018 UniRef90\_A0A178LP42\_49\_332 | R | E | - | - | G | P | F | F | E | H | F | I | R | S | H | A | - | - | - | - | - | - | - | - | - | V | N | K | - | A | V | F | T | P | E | L | L | N | A | Y | A | R | S | Y | A | K | P | - | Q | S |
| 019 UniRef90\_T2L220\_20\_307 | K | E | - | - | R | I | F | F | E | H | F | I | K | V | H | A | - | - | - | - | - | - | - | - | - | A | N | K | - | E | V | F | T | P | E | L | L | D | L | Y | A | S | S | Y | A | K | S | - | H | S |
| 020 UniRef90\_UPI0014749FF6\_22\_304 | N | E | - | - | K | V | F | F | S | H | F | I | K | K | H | A | - | - | - | - | - | - | - | - | - | E | K | P | - | E | A | F | T | E | K | M | L | D | L | Y | A | K | S | Y | S | K | P | - | H | T |
| 021 UniRef90\_A0A172YJZ3\_18\_305 | N | E | - | - | R | V | F | F | E | H | F | I | K | E | H | A | - | - | - | - | - | - | - | - | - | T | N | R | - | D | V | F | T | P | E | L | L | D | L | Y | A | A | S | Y | A | K | P | - | H | S |
| 022 UniRef90\_UPI000737C6BF\_26\_313 | N | E | - | - | R | M | F | F | E | H | F | I | K | E | H | A | - | - | - | - | - | - | - | - | - | T | N | K | - | D | V | F | T | P | E | L | L | D | L | Y | A | A | S | Y | A | K | P | - | H | S |
| 023 UniRef90\_A0A329J6I4\_26\_309 | H | E | - | - | K | F | F | L | K | R | F | I | Q | S | H | A | - | - | - | - | - | - | - | - | - | A | S | M | - | D | A | F | T | P | K | L | I | D | M | Y | A | K | S | Y | A | K | P | - | Q | T |
| 024 UniRef90\_A0A2N3KZL1\_30\_325 | H | E | - | - | R | M | F | L | E | H | F | I | K | E | H | A | - | - | - | - | - | - | - | - | - | T | N | R | - | A | A | F | T | D | E | L | L | D | L | Y | G | A | S | Y | A | K | P | - | H | T |
| 025 UniRef90\_UPI0006CA847F\_27\_323 | K | E | - | - | K | L | F | L | S | H | I | I | K | K | H | A | - | - | - | - | - | - | - | - | - | T | N | K | - | E | V | F | T | D | E | L | L | D | L | Y | A | K | S | Y | A | K | P | - | E | T |
| 026 UniRef90\_UPI00054E0E46\_26\_312 | K | E | - | - | R | L | F | F | E | H | F | I | K | E | H | A | - | - | - | - | - | - | - | - | - | T | N | K | - | A | V | F | T | P | Q | L | L | D | L | Y | A | R | S | Y | A | K | P | - | H | S |
| 027 UniRef90\_A0A0S9PRE2\_29\_311 | K | E | - | - | R | F | F | L | E | Y | F | I | K | V | H | A | - | - | - | - | - | - | - | - | - | T | N | T | - | V | V | F | T | P | E | V | L | D | L | Y | A | H | S | Y | S | K | P | - | G | A |
| 028 UniRef90\_W0AA74\_37\_318 | R | E | - | - | R | M | F | F | E | H | F | I | K | S | H | A | - | - | - | - | - | - | - | - | - | V | N | K | - | A | V | F | T | P | E | L | L | D | R | Y | S | R | S | Y | A | K | T | - | H | S |
| 029 UniRef90\_A0A2G0Y5Q3\_27\_312 | K | E | - | - | K | F | F | L | S | H | F | I | K | S | H | G | - | - | - | - | - | - | - | - | - | T | N | T | - | S | V | F | T | E | K | L | L | D | L | Y | A | K | S | Y | S | K | P | - | H | S |
| 030 UniRef90\_A0A1M6LZ92\_62\_349 | K | E | - | - | R | F | F | L | E | Y | F | V | K | V | H | A | - | - | - | - | - | - | - | - | - | A | N | K | - | A | V | F | T | P | Q | L | L | D | L | Y | G | K | S | Y | A | K | P | - | Q | S |
| 031 UniRef90\_A0A4Z1C7D3\_28\_313 | N | E | - | - | R | M | F | L | E | H | F | I | K | E | H | A | - | - | - | - | - | - | - | - | - | T | N | R | - | E | A | F | T | D | E | V | L | D | L | Y | A | A | S | Y | A | K | P | - | H | T |
| 032 UniRef90\_A0A367MBG0\_273\_561 | K | E | - | - | R | F | F | L | E | H | F | I | K | S | H | A | - | - | - | - | - | - | - | - | - | S | N | T | - | E | V | F | S | E | R | L | L | D | L | Y | A | R | S | Y | A | K | P | - | H | S |
| 033 UniRef90\_UPI00067CEA54\_35\_318 | R | E | - | - | V | S | F | F | E | H | F | I | K | S | H | A | - | - | - | - | - | - | - | - | - | V | N | K | - | E | V | F | T | S | E | L | I | G | L | Y | A | R | S | Y | S | K | P | - | Q | S |
| 034 UniRef90\_A0A1Y0L5M5\_31\_311 | N | E | - | - | E | F | F | L | T | H | F | I | K | E | H | A | - | - | - | - | - | - | - | - | - | S | N | K | - | D | A | F | T | P | E | L | L | H | M | Y | A | E | S | Y | A | K | P | - | H | T |
| 035 UniRef90\_UPI000C2FE79C\_29\_313 | K | E | - | - | R | Y | F | F | E | H | F | I | K | V | H | A | - | - | - | - | - | - | - | - | - | T | N | T | - | S | V | F | T | P | Q | L | L | D | L | Y | G | K | S | Y | A | K | P | - | H | T |
| 036 UniRef90\_A0A4P8SJX7\_14\_295 | H | E | - | - | K | L | F | L | T | H | F | I | M | E | H | A | - | - | - | - | - | - | - | - | - | T | H | K | - | A | V | F | T | P | A | L | L | D | M | Y | A | T | S | Y | A | K | P | - | H | T |
| 037 UniRef90\_A0A1I1BZT3\_28\_315 | H | E | - | - | R | M | F | L | E | H | F | I | K | E | H | A | - | - | - | - | - | - | - | - | - | T | N | R | - | D | A | F | S | D | E | L | L | D | L | Y | A | A | S | Y | A | K | P | - | Q | T |
| 038 UniRef90\_UPI000DF0D36E\_15\_290 | N | E | - | - | K | F | F | L | T | H | F | I | K | E | H | A | - | - | - | - | - | - | - | - | - | S | N | K | - | D | A | F | T | P | N | L | L | D | M | Y | A | K | S | Y | A | K | P | - | H | T |
| 039 UniRef90\_UPI00136B8797\_37\_318 | R | E | - | - | D | I | F | F | D | H | F | I | K | S | H | A | - | - | - | - | - | - | - | - | - | V | N | K | - | A | V | F | T | P | E | L | I | G | A | Y | A | R | S | Y | A | K | P | - | Q | S |
| 040 UniRef90\_A0A1H4CLJ1\_28\_313 | H | E | - | - | A | Y | F | F | A | H | F | I | K | V | H | A | - | - | - | - | - | - | - | - | - | G | K | P | - | E | V | F | T | N | E | L | L | D | M | Y | A | K | S | Y | S | K | P | - | Q | S |
| 041 UniRef90\_UPI000E1FE28F\_29\_313 | K | E | - | - | R | F | F | F | S | H | F | I | K | V | H | A | - | - | - | - | - | - | - | - | - | T | N | K | - | A | V | F | T | P | A | L | L | D | L | Y | G | K | S | Y | A | K | P | - | H | S |
| 042 UniRef90\_A0A556AX95\_26\_313 | K | E | - | - | R | V | F | F | E | H | F | I | K | E | H | A | - | - | - | - | - | - | - | - | - | T | N | K | - | D | V | F | T | P | E | L | L | D | L | Y | A | K | S | Y | A | K | P | - | H | T |
| 043 UniRef90\_A0A6M8HNY7\_38\_322 | H | E | - | - | R | F | F | I | E | H | F | I | K | V | H | A | - | - | - | - | - | - | - | - | - | T | N | K | - | D | V | F | T | P | K | L | L | D | L | Y | G | H | S | Y | A | K | P | - | Q | T |
| 044 UniRef90\_UPI001612B72A\_24\_301 | K | E | - | - | R | F | F | L | E | H | F | I | S | Q | H | A | - | - | - | - | - | - | - | - | - | T | N | K | - | D | A | F | T | P | E | L | F | D | L | Y | G | R | S | Y | A | K | P | - | R | T |
| 045 UniRef90\_UPI0012F66D09\_46\_328 | H | E | - | - | R | F | F | I | E | H | F | I | K | E | H | A | - | - | - | - | - | - | - | - | - | T | N | K | - | N | V | F | T | P | A | L | L | D | M | Y | G | R | S | Y | A | K | P | - | Q | T |
| 046 UniRef90\_UPI000A94940B\_29\_316 | K | E | - | - | R | V | F | F | E | H | F | I | K | E | H | A | - | - | - | - | - | - | - | - | - | T | H | K | - | A | V | F | T | P | E | L | L | D | L | Y | A | A | S | Y | A | K | P | - | H | S |
| 047 UniRef90\_UPI00102FACD2\_29\_312 | K | E | - | - | R | L | F | F | E | H | F | I | K | E | H | A | - | - | - | - | - | - | - | - | - | T | N | T | - | S | V | F | T | S | Q | L | L | D | L | Y | A | N | A | Y | A | K | P | - | H | S |
| 048 UniRef90\_A0A1S8YNF4\_19\_297 | N | E | - | - | G | F | F | L | T | H | F | I | Q | Q | H | A | - | - | - | - | - | - | - | - | - | A | N | K | - | E | A | F | T | P | A | L | L | E | M | Y | S | R | S | Y | S | K | P | - | H | S |
| 049 UniRef90\_UPI0015755320\_14\_301 | K | E | - | - | R | F | F | F | E | H | F | I | R | V | H | A | - | - | - | - | - | - | - | - | - | V | N | Q | - | A | A | F | T | P | E | L | L | D | L | Y | A | R | S | Y | A | K | P | - | H | S |
| 050 UniRef90\_A0A427Q4N3\_22\_313 | K | E | - | - | R | V | F | F | E | H | F | I | K | Q | H | A | - | - | - | - | - | - | - | - | - | T | N | T | - | S | V | F | T | P | Q | L | L | D | L | Y | A | K | S | Y | A | K | P | - | H | S |
| 051 UniRef90\_A0A0A3Z1S4\_30\_318 | H | E | - | - | K | F | F | L | T | H | F | I | L | E | H | A | - | - | - | - | - | - | - | - | - | T | N | K | - | A | A | F | T | P | A | L | L | D | R | Y | A | K | S | Y | A | K | P | - | H | T |
| 052 UniRef90\_A0A3L8C981\_35\_323 | N | E | - | - | R | L | F | L | T | H | F | I | R | H | H | A | - | - | - | - | - | - | - | - | - | S | N | Q | - | E | A | L | S | D | E | L | I | D | R | Y | V | R | S | Y | S | K | P | - | K | T |
| 053 UniRef90\_A0A6L5BLL6\_28\_324 | N | E | - | - | R | L | F | L | T | H | F | I | R | H | H | A | - | - | - | - | - | - | - | - | - | S | N | Q | - | E | V | F | T | D | D | L | V | D | S | Y | V | R | S | Y | S | K | A | - | Q | T |
| 054 UniRef90\_A0A6B1J288\_27\_314 | K | E | - | - | R | F | F | I | E | H | F | I | K | E | H | A | - | - | - | - | - | - | - | - | - | S | N | K | - | A | A | F | T | S | E | L | L | N | L | Y | G | K | S | Y | A | K | P | - | H | S |
| 055 UniRef90\_A0A263NNG9\_22\_274 | K | E | - | - | K | L | F | L | S | H | F | I | K | K | H | A | - | - | - | - | - | - | - | - | - | E | K | Q | - | S | V | F | T | D | E | L | L | G | L | Y | A | K | S | Y | A | K | P | - | Q | T |
| 056 UniRef90\_W0HXQ2\_33\_307 | H | E | - | - | K | T | F | L | T | H | F | I | R | S | H | A | - | - | - | - | - | - | - | - | - | T | R | Q | - | A | A | F | T | P | Q | L | L | D | G | Y | A | A | A | Y | A | K | P | - | S | S |
| 057 UniRef90\_A0A031HKS8\_40\_323 | N | E | - | - | R | F | F | I | E | H | F | I | K | V | H | A | - | - | - | - | - | - | - | - | - | T | N | K | - | D | A | F | T | P | G | L | L | D | L | Y | G | R | S | Y | A | K | P | - | T | T |
| 058 UniRef90\_A0A4R2FY05\_27\_320 | N | E | - | - | D | K | F | F | K | H | F | I | L | E | H | A | - | - | - | - | - | - | - | - | - | E | N | K | - | N | A | F | S | D | E | L | F | G | M | Y | A | E | S | Y | K | K | P | - | Q | T |
| 059 UniRef90\_A0A1X0N2L9\_36\_321 | N | E | - | - | R | L | F | L | T | H | F | I | R | H | H | A | - | - | - | - | - | - | - | - | - | S | N | Q | - | A | V | F | T | D | D | L | I | E | R | Y | V | R | S | Y | S | K | P | - | Q | T |
| 060 UniRef90\_A0A4R1U0K9\_37\_322 | N | E | - | - | R | L | F | L | T | H | F | I | R | H | H | A | - | - | - | - | - | - | - | - | - | S | N | Q | - | A | V | F | T | D | G | L | I | D | R | Y | V | R | S | Y | A | K | P | - | Q | T |
| 061 UniRef90\_A0A0Q8TEQ0\_42\_323 | K | E | - | - | R | V | F | L | E | D | F | I | L | T | R | A | - | - | - | - | - | - | - | - | - | V | N | K | - | K | A | F | P | P | D | V | L | D | M | Y | A | K | S | W | A | K | P | - | A | S |
| 062 UniRef90\_A0A0Q6ZEJ0\_41\_323 | N | E | - | - | R | A | F | L | E | D | F | I | L | K | R | A | - | - | - | - | - | - | - | - | - | G | N | K | - | D | A | F | P | S | E | V | L | D | M | Y | A | K | S | W | A | K | P | - | R | S |
| 063 UniRef90\_UPI0004C0CBB1\_54\_338 | H | E | - | - | R | A | F | V | E | G | F | V | G | E | Y | L | G | - | - | - | - | - | - | - | - | S | N | K | - | S | A | F | T | P | S | D | Y | D | F | Y | A | H | Y | L | K | Q | P | - | D | R |
| 064 UniRef90\_UPI0004CAB4F8\_56\_339 | H | E | - | - | R | P | F | V | Q | G | F | I | G | E | L | L | - | - | - | - | - | - | - | - | - | A | N | K | - | S | A | F | S | P | A | D | Y | D | F | Y | A | H | Y | L | K | Q | P | - | G | H |
| 065 UniRef90\_UPI00049002CC\_60\_343 | H | E | - | - | R | V | F | V | Q | D | L | I | T | E | Y | L | A | - | - | - | - | - | - | - | - | G | N | K | - | S | P | F | T | P | S | D | Y | D | Y | Y | A | H | F | L | K | E | P | - | G | R |
| 066 UniRef90\_A0A561IX74\_59\_327 | R | E | - | - | R | L | F | L | T | N | F | W | P | V | V | G | - | - | - | - | - | - | - | - | - | H | V | K | - | E | A | F | T | A | E | E | A | N | E | F | I | R | A | Y | S | T | K | - | G | S |
| 067 UniRef90\_A0A0X3RYG7\_67\_350 | H | E | - | - | R | V | I | V | H | D | M | M | L | E | Y | V | N | - | - | - | - | - | - | - | - | R | D | K | - | S | P | F | T | A | S | D | F | D | Y | Y | S | H | F | L | K | E | P | - | G | R |
| 068 UniRef90\_A0A2W2E1M5\_55\_338 | H | E | - | - | R | V | L | V | H | D | M | I | A | E | Y | L | A | - | - | - | - | - | - | - | - | G | A | K | - | S | P | F | T | A | S | D | Y | D | Y | Y | A | H | F | L | K | E | P | - | G | R |
| 069 UniRef90\_A0A2V7YB42\_28\_306 | R | E | - | - | R | T | Y | F | E | H | F | W | N | D | F | A | - | - | - | - | - | - | - | - | - | A | D | K | T | R | S | I | P | E | A | E | R | S | A | Y | A | A | A | Y | A | R | P | - | G | R |
| 070 UniRef90\_A0A544XC49\_52\_336 | H | E | - | - | R | V | L | V | H | D | M | M | T | E | Y | L | A | - | - | - | - | - | - | - | - | G | D | K | - | S | P | F | T | A | S | D | F | D | F | Y | A | H | F | L | K | E | P | - | G | R |
| 071 UniRef90\_A0A101PQ90\_46\_324 | R | E | - | - | L | T | W | T | S | G | F | M | R | G | I | A | - | - | - | - | - | - | - | - | - | V | H | K | - | D | A | L | T | P | E | D | L | E | V | F | S | S | Y | L | R | D | Y | - | A | H |
| 072 UniRef90\_A0A1C4VK67\_14\_288 | R | E | - | - | A | L | W | V | N | R | F | T | D | A | L | M | - | - | - | - | - | - | - | - | - | V | R | K | - | G | S | I | G | T | P | D | I | E | E | Y | A | S | H | L | R | D | P | - | A | H |
| 073 UniRef90\_A0A0X3RPC2\_57\_340 | H | E | - | - | R | V | L | V | E | D | M | I | T | E | Y | L | A | - | - | - | - | - | - | - | - | G | N | K | - | S | P | F | T | A | S | D | Y | D | F | Y | A | H | Y | L | K | E | P | - | G | R |
| 074 UniRef90\_A0A4R2VWW4\_55\_319 | Q | E | - | - | R | L | F | L | T | N | F | W | P | V | V | G | - | - | - | - | - | - | - | - | - | H | V | K | - | D | P | F | T | P | E | E | V | N | E | F | V | R | A | Y | T | V | K | - | G | G |
| 075 UniRef90\_A0A1Q7VB11\_36\_307 | R | E | - | - | R | I | Y | F | E | H | F | W | N | N | F | A | - | - | - | - | - | - | - | - | - | A | D | K | T | H | S | I | P | E | A | D | R | V | A | Y | A | A | A | Y | A | R | P | - | G | R |
| 076 UniRef90\_C7PGQ3\_38\_305 | R | E | - | - | R | L | F | L | T | N | F | W | P | V | V | G | - | - | - | - | - | - | - | - | - | H | A | K | - | D | P | F | T | P | E | E | V | N | E | F | V | R | A | Y | S | T | K | - | G | S |
| 077 UniRef90\_A0A1N7R4E6\_62\_325 | R | E | - | - | R | L | F | L | T | N | F | W | P | V | V | G | - | - | - | - | - | - | - | - | - | H | V | K | - | N | A | F | T | T | E | E | T | N | E | F | V | R | A | Y | S | T | K | - | G | S |
| 078 UniRef90\_A0A2H2Y4V3\_41\_307 | R | E | - | - | R | T | Y | L | K | Y | F | F | T | K | Y | A | - | - | - | - | - | - | - | - | - | Y | Y | K | - | N | A | V | G | S | D | D | L | S | E | Y | V | R | Y | Y | S | A | P | - | G | A |
| 079 UniRef90\_A0A448SCU6\_8\_276 | R | E | - | - | R | E | Y | L | S | Y | F | F | T | T | Y | A | - | - | - | - | - | - | - | - | - | Y | D | P | - | R | A | I | T | A | A | D | L | D | E | F | V | R | C | Y | S | A | A | - | G | G |
| 080 UniRef90\_A0A365Y5E2\_33\_303 | K | A | - | - | N | A | F | L | T | G | F | W | P | V | V | G | - | - | - | - | - | - | - | - | - | H | V | K | - | D | P | F | T | K | Q | E | S | D | E | F | I | R | A | Y | S | V | H | - | G | S |
| 081 UniRef90\_A0A1I2CFD2\_63\_327 | R | E | - | - | R | L | F | L | T | N | F | W | P | V | V | G | - | - | - | - | - | - | - | - | - | H | V | K | - | N | A | F | T | T | E | E | T | N | E | F | V | R | A | Y | S | T | K | - | G | S |
| 082 UniRef90\_A0A1G7Z850\_54\_322 | R | E | - | - | R | L | F | L | T | N | F | W | P | V | V | G | - | - | - | - | - | - | - | - | - | H | V | K | - | D | A | F | T | T | E | E | T | N | E | F | V | R | A | Y | A | V | K | - | G | G |
| 083 UniRef90\_A0A2V9XG70\_35\_308 | R | E | - | - | R | T | Y | F | E | H | F | W | N | D | F | A | - | - | - | - | - | - | - | - | - | A | D | K | T | R | S | L | P | E | A | D | R | K | A | Y | T | A | A | Y | S | R | P | - | G | R |
| 084 UniRef90\_A0A0Q4GSS4\_39\_308 | K | V | - | - | S | L | F | F | T | D | F | W | P | T | V | G | - | - | - | - | - | - | - | - | - | F | K | K | - | N | A | F | S | A | A | E | T | A | E | F | I | R | A | Y | S | V | N | - | G | A |
| 085 UniRef90\_A0A0M9ZTU1\_1\_271 | N | E | - | - | R | V | L | V | Q | T | L | F | S | E | Y | L | A | - | - | - | - | - | - | - | P | G | N | K | - | S | T | F | T | P | S | D | I | D | Y | Y | A | H | F | L | K | E | P | - | G | R |
| 086 UniRef90\_A0A239P597\_1\_270 | H | E | - | - | R | V | L | V | H | D | M | M | L | E | Y | V | A | - | - | - | - | - | - | - | - | G | D | K | - | S | P | F | T | A | S | D | F | D | F | Y | A | H | F | L | K | E | P | - | G | R |
| 087 UniRef90\_UPI00128E3D53\_19\_294 | R | E | - | - | R | A | Y | I | G | A | Q | L | H | N | W | S | - | - | - | - | - | - | - | - | - | Y | S | K | - | T | L | F | S | E | E | E | I | T | E | Y | A | R | Q | Y | T | R | P | - | G | G |
| 088 UniRef90\_A0A1V9E1W0\_57\_321 | R | E | - | - | R | L | F | L | T | N | F | W | P | V | V | G | - | - | - | - | - | - | - | - | - | H | V | K | - | D | P | F | T | T | E | E | V | N | E | F | I | R | A | Y | T | V | K | - | G | G |
| 089 UniRef90\_A0A239MTD5\_1\_269 | H | E | - | - | R | V | F | I | Q | E | M | I | T | A | Y | L | V | - | - | - | - | - | - | - | - | G | N | K | - | S | P | F | T | P | S | D | Y | D | Y | Y | A | H | F | L | K | E | P | - | G | R |
| 090 UniRef90\_UPI0009FCBBE8\_54\_317 | R | A | H | V | A | T | Y | L | G | W | L | F | S | S | A | - | - | - | - | - | - | - | - | - | - | K | Y | P | - | N | S | I | A | Q | - | - | - | S | T | Y | I | D | A | Y | S | D | A | - | A | H |
| 091 UniRef90\_A0A1A9HTL2\_19\_299 | H | E | - | - | R | A | Y | L | G | W | L | F | A | Q | K | S | - | - | - | - | - | - | - | - | - | V | K | R | - | E | V | F | T | T | E | A | L | D | E | Y | T | R | V | F | A | Q | P | - | G | G |
| 092 UniRef90\_A0A4R7CM41\_23\_285 | N | V | - | - | P | L | F | L | A | N | F | W | S | T | K | A | - | - | - | - | - | - | - | - | - | H | Q | Q | - | D | A | F | T | K | E | E | W | T | E | F | T | R | A | Y | M | Q | P | - | G | A |
| 093 UniRef90\_A0A562QYK6\_25\_307 | R | E | - | - | R | I | Y | F | E | H | F | W | N | D | F | A | - | - | - | - | - | - | - | - | - | A | D | K | T | K | S | L | S | E | A | D | R | R | F | Y | A | K | E | Y | G | K | P | - | G | H |
| 094 UniRef90\_A0A2V9ZM06\_3\_269 | R | E | - | - | R | T | Y | F | E | Y | F | W | N | D | L | A | - | - | - | - | - | - | - | - | - | A | D | K | T | R | S | L | P | E | A | D | R | Q | A | Y | T | T | A | Y | S | R | P | - | G | R |
| 095 UniRef90\_A0A4P8YF40\_11\_271 | R | E | - | - | R | E | Y | L | S | Y | F | F | K | T | Y | A | - | - | - | - | - | - | - | - | - | Y | N | P | - | L | A | V | T | E | S | D | I | D | E | F | V | R | C | Y | S | A | S | - | G | G |
| 096 UniRef90\_A0A2V6E8I4\_30\_303 | R | E | - | - | R | T | Y | F | E | Y | F | W | N | D | L | A | - | - | - | - | - | - | - | - | - | A | D | K | T | R | S | I | P | E | A | D | R | K | A | Y | T | E | A | Y | S | R | P | - | G | R |
| 097 UniRef90\_A0A2V9BFT0\_23\_295 | R | E | - | - | Q | I | Y | F | A | Y | F | W | N | D | L | A | - | - | - | - | - | - | - | - | - | A | D | K | T | R | S | L | P | E | G | D | R | K | A | Y | V | E | A | Y | S | R | P | - | G | R |
| 098 UniRef90\_A0A2V9H5C8\_29\_298 | R | E | - | - | R | T | Y | F | E | H | Y | W | N | N | F | A | - | - | - | - | - | - | - | - | - | A | D | K | T | R | S | L | S | E | A | D | R | K | A | Y | T | A | A | Y | A | R | P | - | G | R |
| 099 UniRef90\_A0A101CRV3\_44\_308 | K | E | - | - | K | E | F | L | T | G | F | W | P | V | V | A | - | - | - | - | - | - | - | - | - | F | D | K | - | N | A | F | T | K | E | E | T | A | E | F | I | R | A | Y | A | V | K | - | G | A |
| 100 UniRef90\_A0A5M6CFP9\_57\_321 | K | E | - | - | R | E | F | L | T | N | F | W | P | V | V | G | - | - | - | - | - | - | - | - | - | H | V | K | - | D | P | F | T | K | E | E | A | N | E | F | I | R | A | Y | H | V | K | - | G | A |
| 101 UniRef90\_A0A401Z9D4\_19\_284 | R | E | - | - | H | I | L | L | Q | W | F | F | A | R | - | A | - | - | - | - | - | - | - | - | - | R | N | S | - | A | A | F | T | Q | E | D | I | A | E | Y | V | R | C | Y | S | G | R | - | D | A |
| 102 UniRef90\_A0A2V8XJ58\_32\_308 | R | E | - | - | R | L | Y | F | E | Y | F | W | N | D | L | A | - | - | - | - | - | - | - | - | - | A | D | R | T | R | S | L | P | E | A | D | R | K | A | Y | A | E | A | Y | A | R | P | - | G | R |
| 103 UniRef90\_A0A252EMP1\_15\_277 | R | E | - | - | R | E | Y | L | S | F | F | F | K | R | Y | A | - | - | - | - | - | - | - | - | - | F | D | P | - | R | S | I | T | E | A | D | I | T | E | Y | V | R | C | Y | S | G | L | - | G | G |
| 104 UniRef90\_A0A1H3H759\_34\_310 | R | E | - | - | R | L | F | F | G | Y | Q | F | A | T | K | A | - | - | - | - | - | - | - | - | - | A | R | K | - | - | - | L | P | E | H | A | V | D | L | Y | I | E | A | L | A | A | A | R | D | A |
| 105 UniRef90\_A0A4D7B7N1\_3\_270 | R | E | - | - | R | L | Y | I | E | W | F | F | Q | R | K | T | - | - | - | - | - | - | - | - | - | A | N | P | A | A | T | F | S | A | Q | D | V | D | E | Y | E | R | V | Y | R | M | T | - | G | N |
| 106 UniRef90\_A0A1Q7MUT6\_34\_308 | R | E | - | - | R | T | Y | F | E | H | F | W | N | D | F | A | - | - | - | - | - | - | - | - | - | A | D | K | T | H | S | I | P | D | A | D | R | K | A | Y | T | A | A | Y | S | R | P | - | G | R |
| 107 UniRef90\_A0A2V6KQW0\_24\_297 | R | E | - | - | R | I | Y | F | E | Y | F | W | N | V | F | A | - | - | - | - | - | - | - | - | - | A | D | K | T | R | S | I | P | E | A | D | R | K | A | Y | T | E | A | Y | S | K | P | - | G | R |
| 108 UniRef90\_A0A4R6LFZ5\_44\_308 | K | E | - | - | K | E | F | L | T | G | F | W | P | V | V | A | - | - | - | - | - | - | - | - | - | F | N | K | - | E | S | F | T | K | E | E | T | A | E | F | I | R | A | Y | S | V | N | - | G | A |
| 109 UniRef90\_A0A1H7UU25\_49\_318 | K | E | - | - | K | E | F | L | I | G | F | W | P | V | V | G | - | - | - | - | - | - | - | - | - | H | V | Q | - | N | A | F | T | K | E | E | T | D | E | F | I | R | A | Y | R | T | P | - | R | S |
| 110 UniRef90\_UPI0008326796\_14\_259 | K | E | - | - | R | V | Y | L | D | W | F | L | R | R | K | T | - | - | - | - | - | - | - | - | - | A | N | P | - | F | S | F | S | E | E | D | L | D | E | Y | E | R | I | F | T | L | P | - | G | A |
| 111 UniRef90\_A0A2V5S8F2\_33\_306 | R | E | - | - | R | I | F | F | E | Y | F | W | N | D | L | A | - | - | - | - | - | - | - | - | - | A | D | K | T | R | S | L | P | E | A | D | R | K | A | Y | A | E | A | Y | S | R | P | - | G | R |
| 112 UniRef90\_A0A3S3VTN8\_36\_308 | K | V | - | - | G | L | F | F | T | D | F | W | P | T | V | G | - | - | - | - | - | - | - | - | - | F | K | N | - | N | S | F | T | Q | A | E | K | N | E | F | I | R | A | Y | S | V | P | - | G | A |
| 113 UniRef90\_A0A6J4PSU4\_16\_248 | K | E | - | - | R | E | Y | L | S | H | F | Y | R | T | Y | S | - | - | - | - | - | - | - | - | - | Y | D | P | - | A | A | I | G | E | E | D | V | D | E | Y | V | T | R | Y | S | A | P | - | G | G |
| 114 UniRef90\_A0A534ZXC6\_26\_300 | R | E | - | - | R | V | Y | F | E | H | F | W | N | D | F | A | - | - | - | - | - | - | - | - | - | A | D | P | R | R | S | V | P | E | A | D | R | A | V | Y | A | A | A | Y | A | R | P | - | G | R |
| 115 UniRef90\_A0A2A9KBX9\_78\_358 | H | E | - | - | R | A | Y | L | S | W | I | F | Q | Q | K | A | - | - | - | - | - | - | - | - | - | T | R | N | - | W | V | F | T | P | D | V | I | D | E | Y | V | R | V | F | I | Q | P | - | G | G |
| 116 UniRef90\_A0A2W0BWH7\_16\_287 | R | E | - | - | G | T | Y | F | E | Y | F | W | N | D | L | A | - | - | - | - | - | - | - | - | - | A | N | K | A | R | S | I | P | E | S | D | R | R | A | Y | V | A | A | Y | S | R | P | - | G | R |
| 117 UniRef90\_A0A1R1RMU1\_30\_310 | R | E | - | - | R | E | Y | L | D | W | F | L | R | R | K | A | - | - | - | - | - | - | - | - | - | A | N | P | - | E | V | F | S | E | A | D | I | E | E | Y | L | R | V | F | K | K | A | - | G | G |
| 118 UniRef90\_A0A3A1NH59\_38\_305 | K | E | - | - | E | M | F | F | K | D | F | W | T | T | V | T | - | - | - | - | - | - | - | - | - | P | M | K | - | R | P | F | S | K | D | E | E | D | E | F | I | R | A | Y | S | V | K | - | G | S |
| 119 UniRef90\_A0A0Q6WP84\_43\_317 | R | E | - | - | R | T | Y | F | E | H | F | W | N | D | F | A | - | - | - | - | - | - | - | - | - | A | D | K | S | K | S | I | K | E | A | D | R | K | L | Y | A | A | A | Y | A | Q | P | - | G | G |
| 120 UniRef90\_A0A2V6P1A8\_15\_289 | R | E | - | - | R | I | Y | F | E | Y | F | W | N | V | L | A | - | - | - | - | - | - | - | - | - | A | D | K | T | H | S | I | P | E | A | D | R | K | A | Y | T | E | A | Y | S | K | P | - | G | R |
| 121 UniRef90\_A0A2V5NC55\_35\_307 | R | E | - | - | R | T | Y | F | E | Y | F | W | N | V | F | A | - | - | - | - | - | - | - | - | - | A | D | K | N | H | S | I | P | E | A | E | R | K | A | Y | T | E | A | Y | S | K | P | - | G | R |
| 122 UniRef90\_A0A2V9BM40\_39\_318 | R | E | - | - | R | I | Y | F | E | H | F | W | N | N | F | A | - | - | - | - | - | - | - | - | - | A | D | K | T | H | S | I | P | E | A | D | R | V | A | Y | T | A | A | Y | A | R | P | - | G | R |
| 123 UniRef90\_A0A5B8WA72\_34\_302 | K | V | - | - | G | L | F | L | T | D | F | W | P | V | V | G | - | - | - | - | - | - | - | - | - | F | K | K | - | N | A | F | T | T | A | E | R | N | E | F | I | R | A | Y | S | V | P | - | G | A |
| 124 UniRef90\_A0A1Y6D2S0\_25\_299 | R | E | - | - | R | E | Y | I | A | A | Q | I | K | Q | W | A | - | - | - | - | - | - | - | - | - | H | R | K | - | Q | A | I | T | P | Q | A | I | D | E | Y | A | K | H | Y | A | R | P | - | G | G |
| 125 UniRef90\_A0A494W0L3\_12\_284 | K | A | - | - | G | L | F | L | T | D | F | W | P | V | V | G | - | - | - | - | - | - | - | - | - | Y | I | K | - | K | P | F | T | K | A | E | T | A | E | F | I | R | A | Y | S | V | P | - | G | A |
| 126 UniRef90\_UPI0015CD0065\_39\_309 | D | D | - | V | R | T | Y | L | G | M | M | F | G | - | F | S | - | - | - | - | - | - | - | - | - | H | Q | P | - | G | A | I | N | A | - | - | - | E | P | Y | F | Q | A | Y | A | D | P | - | A | R |
| 127 UniRef90\_UPI00166E2F5D\_15\_289 | N | E | - | - | L | L | Y | F | S | Y | F | Y | D | V | L | A | - | - | - | - | - | - | - | - | - | G | K | G | - | K | K | L | G | E | T | L | K | K | S | F | T | E | A | Y | T | R | P | - | G | A |
| 128 UniRef90\_A0A372NPX1\_36\_305 | K | V | - | - | G | L | F | L | T | D | F | W | P | V | V | G | - | - | - | - | - | - | - | - | - | F | Q | K | - | N | A | F | T | A | A | E | R | K | E | F | I | R | A | Y | S | V | P | - | G | A |
| 129 UniRef90\_A0A317JA08\_13\_279 | R | E | - | - | K | I | Y | F | A | Y | F | W | N | D | L | A | - | - | - | - | - | - | - | - | - | A | D | K | G | R | S | L | S | T | P | D | R | K | A | Y | V | A | A | Y | A | R | P | - | G | R |
| 130 UniRef90\_A0A2V6N4R6\_10\_283 | R | E | - | - | R | T | Y | F | E | Y | F | W | N | V | L | A | - | - | - | - | - | - | - | - | - | A | D | K | T | H | S | I | P | E | S | D | R | K | A | Y | T | E | A | Y | S | K | P | - | G | R |
| 131 UniRef90\_A0A2V5IZ05\_29\_302 | R | E | - | - | R | I | Y | F | E | H | F | W | N | D | F | A | - | - | - | - | - | - | - | - | - | A | D | K | N | R | S | L | S | E | A | D | R | A | G | Y | T | A | A | Y | S | R | P | - | G | R |
| 132 UniRef90\_A0A521E7V8\_39\_308 | K | V | - | - | G | L | F | F | T | D | F | W | P | V | V | G | - | - | - | - | - | - | - | - | - | F | Q | K | - | N | A | F | T | T | A | E | T | A | E | F | I | R | S | Y | S | V | P | - | G | A |
| 133 UniRef90\_UPI001665F59F\_18\_290 | N | E | - | - | F | L | Y | F | S | Y | F | Y | D | V | L | A | - | - | - | - | - | - | - | - | - | G | K | D | - | K | K | M | D | D | A | R | K | K | L | Y | T | D | A | Y | I | R | P | - | D | A |
| 134 UniRef90\_A0A0Q9GGK2\_19\_284 | R | E | - | - | R | L | Y | L | D | Y | F | F | K | H | V | T | - | - | - | - | - | - | - | - | - | Y | N | P | - | A | S | I | K | E | E | D | L | A | E | Y | V | R | A | Y | S | A | P | - | G | A |
| 135 UniRef90\_A0A329YBZ0\_37\_320 | H | E | - | - | Y | A | F | I | R | D | F | Y | L | K | R | A | - | - | - | - | - | - | - | - | - | Y | R | Q | - | - | - | V | A | D | E | D | I | A | V | Y | A | K | A | Y | A | A | P | - | G | G |
| 136 UniRef90\_A0A2E0H9P2\_13\_236 | R | E | - | - | E | I | Y | L | S | W | F | Y | K | T | F | A | - | - | - | - | - | - | - | - | - | Y | K | P | - | N | S | I | T | Q | K | D | I | E | E | Y | V | R | T | Y | R | Q | P | - | G | A |
| 137 UniRef90\_A0A095SYG7\_44\_308 | K | E | - | - | K | E | F | L | T | N | F | W | P | Q | V | A | - | - | - | - | - | - | - | - | - | F | A | K | - | D | S | F | T | K | E | E | T | A | E | F | I | R | A | Y | A | V | K | - | G | A |
| 138 UniRef90\_A0A2U0ZNC3\_54\_322 | Q | A | - | - | G | L | F | L | T | D | F | W | T | V | V | G | - | - | - | - | - | - | - | - | - | H | V | P | - | N | A | F | T | K | E | E | K | D | E | F | I | R | A | Y | S | V | K | - | G | A |
| 139 UniRef90\_A0A2H2XXU1\_20\_287 | R | E | - | - | R | E | Y | L | S | T | I | A | Y | K | S | G | - | - | - | - | - | - | - | - | - | Y | N | P | - | T | A | I | T | E | A | D | I | D | E | Y | V | R | H | Y | A | S | P | - | G | G |
| 140 UniRef90\_A0A0P4V0Y0\_11\_278 | R | E | - | - | Q | E | F | L | S | Y | F | Y | K | T | Y | C | - | - | - | - | - | - | - | - | - | V | D | S | - | S | A | I | E | P | E | A | I | N | E | Y | L | R | T | F | A | T | P | - | A | G |
| 141 UniRef90\_A0A2D7FF16\_11\_288 | R | E | - | - | R | T | Y | L | Q | W | F | Y | Q | T | F | S | - | - | - | - | - | - | - | - | - | Y | K | S | - | D | A | I | D | D | D | A | L | N | E | F | V | R | T | Y | S | Q | R | - | G | A |
| 142 UniRef90\_UPI0009FE04F9\_77\_354 | R | V | - | - | E | T | F | L | D | Y | F | L | R | H | S | E | V | S | E | Q | A | R | A | S | S | A | H | E | - | L | P | V | T | P | R | A | L | A | E | Y | V | R | V | Y | S | R | P | - | Q | V |
| 143 UniRef90\_UPI00148816F0\_34\_297 | R | E | - | - | R | L | Y | L | D | Y | F | Y | R | V | Q | A | - | - | - | - | - | - | - | - | - | G | G | G | E | L | P | M | T | P | G | V | L | E | E | Y | V | R | A | Y | A | R | P | - | A | A |
| 144 UniRef90\_A0A2V5MXX8\_31\_304 | R | E | - | - | R | A | Y | F | E | Y | F | W | N | V | F | A | - | - | - | - | - | - | - | - | - | A | D | K | T | R | S | I | P | E | G | D | R | K | A | Y | T | E | A | Y | S | R | P | - | G | R |
| 145 UniRef90\_Q1W504\_15\_282 | H | E | - | - | E | V | F | L | R | Y | F | I | Q | H | L | A | - | - | - | - | - | - | - | - | - | Y | D | A | - | S | A | I | T | D | E | D | I | A | H | Y | S | T | N | M | K | T | P | - | G | G |
| 146 UniRef90\_UPI001661900D\_10\_259 | H | E | - | - | A | E | Y | V | D | W | F | L | R | S | G | T | - | - | - | - | - | - | - | - | - | L | G | - | - | E | G | V | R | P | A | L | R | E | A | F | V | R | A | Y | T | G | R | - | E | A |
| 147 UniRef90\_A0A2D6SJL3\_11\_235 | X | E | - | - | K | V | Y | L | G | W | F | Y | S | T | F | A | - | - | - | - | - | - | - | - | - | Y | V | P | - | N | A | I | T | E | V | D | I | N | E | Y | V | R | T | Y | S | Q | P | - | G | A |
| 148 UniRef90\_A0A2V6J615\_3\_225 | R | E | - | - | R | T | Y | F | E | Y | F | W | N | V | L | A | - | - | - | - | - | - | - | - | - | A | D | K | T | R | S | I | P | E | A | D | R | R | A | Y | T | E | A | Y | A | K | P | - | G | R |

  
  

|  |  |  |  |  |  |  |  |  |  |  |  |  |  |  |  |  |  |  |  |  |  |  |  |  |  |  |  |  |  |  |  |  |  |  |  |  |  |  |  |  |  |  |  |  |  |  |  |  |  |  |
| --- | --- | --- | --- | --- | --- | --- | --- | --- | --- | --- | --- | --- | --- | --- | --- | --- | --- | --- | --- | --- | --- | --- | --- | --- | --- | --- | --- | --- | --- | --- | --- | --- | --- | --- | --- | --- | --- | --- | --- | --- | --- | --- | --- | --- | --- | --- | --- | --- | --- | --- |
| **001 Input\_protein\_seq** | L | K | A | A | F | G | Y | Y | R | S | H | A | D | S | I | - | - | R | Q | N | E | A | L | L | A | N | G | K | K | L | T | I | P | S | M | A | L | T | G | Q | - | - | - | K | G | V | - | - | - | - |
| 002 UniRef90\_D8JE57\_1\_349 | L | K | A | A | F | G | Y | Y | R | S | K | K | D | S | A | - | - | L | Q | N | E | A | L | L | A | N | N | K | K | L | F | I | P | A | M | A | L | S | G | E | - | - | - | K | G | V | - | - | - | - |
| 003 UniRef90\_A0A6B8MSH7\_6\_345 | L | H | A | A | F | G | Y | Y | K | S | H | A | K | S | I | - | - | A | Q | N | E | Q | L | L | A | E | G | K | K | L | S | I | P | S | M | A | L | T | G | E | - | - | - | K | G | V | - | - | - | - |
| 004 UniRef90\_A0A6B2FNS7\_11\_352 | L | R | A | A | F | G | Y | Y | K | S | H | A | D | S | V | - | - | Q | Q | N | E | A | L | L | A | A | G | K | T | L | R | I | P | S | M | A | L | T | G | A | - | - | - | K | G | V | - | - | - | - |
| 005 UniRef90\_UPI00073D803C\_6\_344 | L | H | A | A | F | G | Y | Y | K | S | H | A | R | S | V | - | - | K | Q | N | E | A | L | L | A | A | G | K | T | L | S | I | P | S | M | A | L | T | G | A | - | - | - | S | G | V | - | - | - | - |
| 006 UniRef90\_A0A285K5K8\_3\_346 | L | K | A | A | F | G | Y | Y | K | S | H | A | D | S | I | - | - | K | Q | N | E | S | L | L | A | A | G | K | K | I | S | I | P | S | M | A | L | T | G | Q | - | - | - | K | G | V | - | - | - | - |
| 007 UniRef90\_A0A379I9D6\_1\_346 | L | H | A | A | F | G | Y | Y | K | S | H | A | N | S | I | - | - | A | Q | N | E | A | L | L | A | D | G | K | L | L | K | I | P | S | M | A | L | T | G | Q | - | - | - | Q | G | V | - | - | - | - |
| 008 UniRef90\_A0A6B2FTI5\_15\_352 | I | H | A | A | L | G | Y | Y | H | S | K | T | R | S | I | - | - | A | Q | N | E | A | L | L | A | A | G | K | T | L | H | I | P | S | M | A | L | T | G | A | - | - | - | K | G | V | - | - | - | - |
| 009 UniRef90\_A0A2P9HD85\_2\_318 | L | R | A | A | F | G | Y | Y | Q | S | H | S | D | S | A | - | - | R | Q | N | E | A | L | L | A | A | G | K | Q | L | T | I | P | V | Y | S | I | S | G | A | - | - | - | K | G | V | - | - | - | - |
| 010 UniRef90\_A0A1T1H7K2\_15\_316 | L | N | A | S | F | E | Y | Y | R | S | L | N | D | S | I | - | - | K | E | N | E | T | I | S | K | T | - | - | K | L | N | M | P | V | L | A | I | G | G | G | G | H | - | G | G | M | - | - | - | - |
| 011 UniRef90\_A0A1A9HKQ2\_27\_313 | L | N | A | A | F | E | Y | Y | R | V | V | N | Q | G | V | - | - | Q | D | N | K | V | L | A | N | R | - | - | K | I | T | V | P | A | M | A | I | G | G | G | G | H | - | G | G | L | - | - | - | - |
| 012 UniRef90\_A0A0J6GRA0\_22\_303 | L | N | A | S | F | E | Y | Y | R | A | L | N | Q | S | V | - | - | T | E | N | V | E | L | S | K | T | - | - | P | L | N | M | P | V | L | A | I | G | G | G | G | H | - | G | G | M | - | - | - | - |
| 013 UniRef90\_UPI000F5BD18A\_25\_314 | L | K | A | A | F | E | Y | Y | R | V | L | N | Q | G | V | - | - | L | D | N | K | E | L | S | K | Q | - | - | K | I | D | I | P | M | L | A | I | G | G | G | G | N | - | G | G | M | - | - | - | - |
| 014 UniRef90\_A0A4V1G750\_26\_311 | L | A | A | A | F | G | Y | Y | R | E | L | N | Q | N | V | - | - | Q | D | N | R | K | L | S | G | T | - | - | P | L | T | L | P | V | L | A | I | G | G | G | G | H | - | G | G | M | - | - | - | - |
| 015 UniRef90\_U3U231\_21\_300 | L | N | A | A | F | E | Y | Y | R | V | L | N | Q | G | V | - | - | A | D | N | K | V | L | S | R | E | - | - | K | I | A | V | P | M | L | A | I | G | G | G | G | H | - | G | G | L | - | - | - | - |
| 016 UniRef90\_F2LPZ2\_26\_313 | L | H | A | A | F | G | Y | Y | R | E | L | N | Q | D | V | - | - | R | D | N | A | E | L | S | R | S | - | - | K | L | T | M | P | M | L | A | I | G | G | G | G | H | - | G | G | L | - | - | - | - |
| 017 UniRef90\_A0A3M4V4S1\_6\_330 | F | H | H | A | F | E | Y | Y | R | A | L | P | Q | S | I | - | - | K | Q | N | E | K | L | V | A | T | - | - | K | L | T | V | P | V | L | A | V | G | G | G | G | N | - | G | G | F | - | - | - | - |
| 018 UniRef90\_A0A178LP42\_49\_332 | F | H | A | A | M | E | Y | Y | R | A | L | P | E | T | Q | - | - | R | R | N | V | P | L | F | E | N | - | - | K | L | T | M | E | T | M | A | I | G | G | G | S | P | - | V | S | V | - | - | - | - |
| 019 UniRef90\_T2L220\_20\_307 | L | N | A | S | F | E | Y | Y | R | V | L | N | Q | G | V | - | - | K | D | N | N | E | L | A | R | T | - | - | K | V | T | I | P | A | L | A | I | G | G | G | D | H | - | G | G | M | - | - | - | - |
| 020 UniRef90\_UPI0014749FF6\_22\_304 | L | H | A | A | F | E | Y | Y | R | A | L | P | A | S | V | - | - | Q | Q | N | E | A | L | L | T | K | - | G | K | L | N | M | P | L | L | A | V | S | G | S | G | R | - | G | G | L | - | - | - | - |
| 021 UniRef90\_A0A172YJZ3\_18\_305 | L | N | A | A | F | E | Y | Y | R | V | L | N | Q | G | V | - | - | Q | D | N | A | E | L | A | Q | T | - | - | R | I | T | I | P | V | L | A | I | G | G | G | G | H | - | G | G | M | - | - | - | - |
| 022 UniRef90\_UPI000737C6BF\_26\_313 | L | N | A | A | F | E | Y | Y | R | V | L | N | Q | G | V | - | - | Q | D | N | L | V | L | A | Q | T | - | - | R | V | T | I | P | A | L | A | I | G | G | G | G | H | - | G | G | M | - | - | - | - |
| 023 UniRef90\_A0A329J6I4\_26\_309 | L | N | A | S | F | E | Y | Y | R | A | L | N | T | S | I | - | - | A | E | N | A | E | L | S | K | T | - | - | S | L | N | M | P | V | L | A | I | G | G | G | G | R | - | N | G | L | - | - | - | - |
| 024 UniRef90\_A0A2N3KZL1\_30\_325 | L | H | A | S | F | E | Y | Y | R | A | L | N | E | T | A | - | - | A | R | N | K | S | L | A | K | K | - | - | K | L | S | M | P | V | L | A | I | G | G | G | G | H | - | G | G | M | - | - | - | - |
| 025 UniRef90\_UPI0006CA847F\_27\_323 | L | H | A | A | F | E | Y | Y | R | S | F | T | T | S | A | - | - | E | Q | N | K | E | I | V | K | D | - | H | K | L | K | M | P | V | L | V | I | P | G | D | S | N | G | G | G | L | - | - | - | - |
| 026 UniRef90\_UPI00054E0E46\_26\_312 | L | H | A | A | F | E | Y | Y | R | V | L | N | Q | G | V | - | - | E | D | N | K | A | L | S | R | T | - | - | K | I | S | L | P | V | L | A | I | G | G | G | G | H | - | G | G | M | - | - | - | - |
| 027 UniRef90\_A0A0S9PRE2\_29\_311 | F | D | A | A | F | A | Y | Y | R | A | L | N | E | T | A | - | - | R | R | N | K | P | L | A | S | P | - | - | R | L | A | M | P | I | L | A | I | G | G | G | G | H | - | G | G | F | - | - | - | - |
| 028 UniRef90\_W0AA74\_37\_318 | F | N | A | A | M | E | Y | Y | R | A | L | P | E | T | Q | - | - | R | R | N | I | P | L | F | K | N | - | - | K | L | T | M | K | T | M | A | I | G | G | G | S | P | - | V | S | V | - | - | - | - |
| 029 UniRef90\_A0A2G0Y5Q3\_27\_312 | L | N | A | S | F | E | Y | Y | R | A | L | N | K | S | I | - | - | E | Q | N | I | K | L | S | K | T | - | - | K | L | T | M | P | T | M | A | L | S | G | G | G | H | - | G | G | M | - | - | - | - |
| 030 UniRef90\_A0A1M6LZ92\_62\_349 | L | H | A | A | F | E | Y | F | R | V | L | N | Q | D | V | - | - | L | D | N | K | Q | L | S | R | Q | - | - | K | I | E | I | P | M | L | A | I | G | G | G | G | N | - | G | G | M | - | - | - | - |
| 031 UniRef90\_A0A4Z1C7D3\_28\_313 | L | N | A | S | F | E | Y | Y | R | S | L | N | E | T | V | - | - | E | R | N | R | P | L | A | K | A | - | - | P | L | T | M | P | I | L | A | I | G | G | G | G | H | - | G | G | M | - | - | - | - |
| 032 UniRef90\_A0A367MBG0\_273\_561 | L | N | A | S | F | E | Y | Y | R | A | L | N | E | S | V | - | - | R | Q | N | A | E | L | A | K | T | - | - | R | L | Q | M | P | T | M | T | L | A | G | G | G | H | - | G | G | M | - | - | - | - |
| 033 UniRef90\_UPI00067CEA54\_35\_318 | F | H | A | A | M | E | Y | Y | R | A | L | P | E | T | Q | - | - | Q | R | N | I | P | L | F | K | N | - | - | K | L | T | M | K | T | M | A | I | G | G | G | S | P | - | V | S | V | - | - | - | - |
| 034 UniRef90\_A0A1Y0L5M5\_31\_311 | L | T | A | S | F | E | Y | Y | R | A | L | N | Q | S | I | - | - | E | Q | N | K | I | L | A | K | D | - | - | K | L | N | I | P | V | M | A | I | G | G | G | G | H | - | G | G | M | - | - | - | - |
| 035 UniRef90\_UPI000C2FE79C\_29\_313 | L | H | A | A | F | E | Y | Y | R | V | L | N | Q | G | V | - | - | I | D | N | K | V | L | A | Q | T | - | - | K | I | S | I | P | M | L | A | I | G | G | G | G | N | - | G | G | M | - | - | - | - |
| 036 UniRef90\_A0A4P8SJX7\_14\_295 | L | T | A | S | F | E | Y | Y | R | A | L | N | T | S | I | - | - | G | Q | N | K | K | L | S | E | T | - | - | K | L | T | M | P | V | M | A | I | G | G | G | G | H | - | G | G | M | - | - | - | - |
| 037 UniRef90\_A0A1I1BZT3\_28\_315 | L | N | A | S | F | E | Y | Y | R | S | L | N | E | T | T | - | - | E | R | N | K | P | L | A | E | T | - | - | K | L | R | M | P | A | L | A | I | G | G | G | G | H | - | G | G | M | - | - | - | - |
| 038 UniRef90\_UPI000DF0D36E\_15\_290 | L | T | A | S | F | E | Y | Y | R | A | L | N | Q | S | I | - | - | E | Q | N | K | T | L | A | K | E | - | - | K | L | N | M | P | V | M | A | I | G | G | G | G | H | - | G | G | M | - | - | - | - |
| 039 UniRef90\_UPI00136B8797\_37\_318 | F | H | A | A | M | E | Y | Y | R | A | L | P | E | T | Q | - | - | V | R | N | I | P | L | F | K | N | - | - | K | L | T | M | K | T | M | A | I | G | G | G | S | P | - | V | S | V | - | - | - | - |
| 040 UniRef90\_A0A1H4CLJ1\_28\_313 | L | N | A | A | F | E | Y | Y | Q | V | L | T | K | D | V | - | - | K | D | N | K | E | I | S | K | T | - | - | K | I | T | I | P | A | M | A | I | G | G | G | G | N | - | G | G | F | - | - | - | - |
| 041 UniRef90\_UPI000E1FE28F\_29\_313 | L | H | A | A | F | E | Y | Y | R | V | L | N | E | D | V | - | - | K | D | N | Q | Q | L | A | K | Q | - | - | K | I | E | V | P | M | L | A | I | G | G | G | G | N | - | G | G | M | - | - | - | - |
| 042 UniRef90\_A0A556AX95\_26\_313 | L | N | A | A | F | E | Y | Y | R | V | L | N | Q | G | V | - | - | Q | D | N | K | Q | L | A | Q | T | - | - | K | V | T | I | P | A | L | A | I | G | G | G | G | H | - | G | G | M | - | - | - | - |
| 043 UniRef90\_A0A6M8HNY7\_38\_322 | L | H | G | S | F | E | Y | Y | R | A | L | N | E | T | V | - | - | R | R | N | K | P | L | A | A | T | - | - | K | L | T | M | P | V | L | A | I | G | G | G | G | N | - | G | G | L | - | - | - | - |
| 044 UniRef90\_UPI001612B72A\_24\_301 | L | H | G | S | F | E | Y | Y | R | A | L | N | E | T | A | - | - | R | R | N | A | P | L | A | A | T | - | - | K | L | T | M | P | V | L | A | I | G | G | G | G | H | - | G | G | L | - | - | - | - |
| 045 UniRef90\_UPI0012F66D09\_46\_328 | L | H | G | S | F | E | Y | Y | R | A | L | N | E | T | A | - | - | R | R | N | K | P | L | S | A | T | - | - | K | L | T | M | P | V | L | A | I | G | G | G | G | H | - | G | G | M | - | - | - | - |
| 046 UniRef90\_UPI000A94940B\_29\_316 | L | N | A | A | F | E | Y | Y | R | V | L | N | Q | G | V | - | - | E | D | N | K | R | L | S | Q | T | - | - | Q | I | T | I | P | A | L | A | I | G | G | G | G | H | - | G | G | M | - | - | - | - |
| 047 UniRef90\_UPI00102FACD2\_29\_312 | L | H | A | A | F | A | Y | Y | H | V | L | N | Q | G | V | - | - | E | D | N | K | R | L | S | Q | S | - | - | K | I | S | L | P | V | L | A | I | G | G | G | G | H | - | G | G | M | - | - | - | - |
| 048 UniRef90\_A0A1S8YNF4\_19\_297 | L | T | A | S | F | E | Y | Y | R | A | L | N | V | S | I | - | - | A | Q | N | R | K | L | A | E | T | - | - | K | L | E | M | P | V | M | A | I | G | G | G | G | H | - | G | G | M | - | - | - | - |
| 049 UniRef90\_UPI0015755320\_14\_301 | L | N | A | A | F | E | Y | Y | R | A | L | N | E | T | A | - | - | K | G | N | A | A | L | S | K | N | - | - | K | L | R | M | P | V | L | A | I | G | G | G | G | H | - | G | G | M | - | - | - | - |
| 050 UniRef90\_A0A427Q4N3\_22\_313 | L | N | A | A | F | E | Y | Y | R | V | L | N | Q | N | A | - | - | R | D | N | K | Q | L | S | K | T | - | - | R | I | S | I | P | A | L | A | I | G | G | G | G | N | - | G | G | L | - | - | - | - |
| 051 UniRef90\_A0A0A3Z1S4\_30\_318 | L | T | A | S | F | E | Y | Y | R | A | L | N | T | S | I | - | - | E | Q | N | R | K | L | A | Q | T | - | - | K | L | T | L | P | V | M | A | I | G | G | G | G | H | - | G | G | M | - | - | - | - |
| 052 UniRef90\_A0A3L8C981\_35\_323 | F | H | H | A | F | E | Y | Y | R | A | L | P | Q | S | I | - | - | A | Q | N | E | K | L | A | T | T | - | - | K | I | T | L | P | V | L | A | V | G | G | G | G | N | - | G | G | F | - | - | - | - |
| 053 UniRef90\_A0A6L5BLL6\_28\_324 | F | H | S | A | F | E | Y | Y | R | A | L | P | R | S | I | - | - | V | Q | N | Q | E | L | A | K | T | - | - | K | V | T | M | P | V | L | A | V | G | G | G | G | N | - | G | G | F | - | - | - | - |
| 054 UniRef90\_A0A6B1J288\_27\_314 | L | N | A | S | F | E | Y | Y | R | V | L | N | Q | G | V | - | - | K | D | N | Q | Q | L | A | K | T | - | - | K | I | T | I | P | M | L | A | I | G | G | G | G | H | - | G | G | M | - | - | - | - |
| 055 UniRef90\_A0A263NNG9\_22\_274 | L | H | A | A | F | E | Y | Y | R | A | L | P | Q | S | V | - | - | Q | Q | N | K | A | L | L | A | Q | - | G | K | L | S | M | P | L | L | A | V | A | G | D | G | R | - | G | S | L | - | - | - | - |
| 056 UniRef90\_W0HXQ2\_33\_307 | L | H | A | S | F | E | Y | Y | R | A | L | N | I | S | I | - | - | A | Q | N | A | V | L | G | Q | H | - | - | P | L | P | M | P | V | M | A | I | G | G | G | G | H | - | G | G | M | - | - | - | - |
| 057 UniRef90\_A0A031HKS8\_40\_323 | L | H | G | S | F | E | Y | Y | R | A | L | N | E | T | A | - | - | R | R | N | R | P | L | A | A | T | - | - | K | L | R | M | P | V | L | A | I | G | G | G | G | H | - | G | G | M | - | - | - | - |
| 058 UniRef90\_A0A4R2FY05\_27\_320 | L | N | A | A | F | E | Y | Y | R | A | L | P | E | S | V | - | - | I | Q | N | D | N | L | S | E | N | - | N | L | L | K | M | P | I | L | T | I | S | G | N | G | Q | - | G | S | L | - | - | - | - |
| 059 UniRef90\_A0A1X0N2L9\_36\_321 | F | H | Y | A | F | E | Y | Y | R | A | L | P | Q | T | I | - | - | A | Q | N | E | Q | L | A | K | T | - | - | R | L | K | M | P | V | L | A | V | G | G | G | G | N | - | G | G | F | - | - | - | - |
| 060 UniRef90\_A0A4R1U0K9\_37\_322 | F | H | H | A | F | E | Y | Y | R | A | L | P | R | S | I | - | - | V | Q | N | Q | A | L | A | A | S | - | - | K | L | T | M | P | V | L | A | V | G | G | G | G | N | - | G | G | F | - | - | - | - |
| 061 UniRef90\_A0A0Q8TEQ0\_42\_323 | L | I | A | S | L | E | Y | Y | R | A | L | N | E | T | A | - | - | Q | R | N | K | P | L | A | A | T | - | - | K | L | T | M | P | V | L | A | I | G | G | G | - | - | - | N | S | M | - | - | - | - |
| 062 UniRef90\_A0A0Q6ZEJ0\_41\_323 | L | T | A | S | L | E | Y | Y | R | A | L | N | E | T | A | - | - | Q | R | N | K | P | L | A | E | T | - | - | K | L | T | M | P | V | L | A | I | G | G | G | - | - | - | N | S | M | - | - | - | - |
| 063 UniRef90\_UPI0004C0CBB1\_54\_338 | T | T | A | W | M | S | V | Y | R | Q | L | R | E | D | I | - | - | R | Q | N | A | K | F | A | A | E | - | G | K | L | K | M | P | I | L | A | I | G | G | S | - | - | - | E | S | F | - | - | - | - |
| 064 UniRef90\_UPI0004CAB4F8\_56\_339 | T | T | A | W | M | N | M | Y | R | Q | L | R | E | D | V | - | - | R | Q | N | K | K | F | L | A | E | - | G | K | L | R | M | P | I | L | A | I | G | G | Q | - | - | - | K | S | F | - | - | - | - |
| 065 UniRef90\_UPI00049002CC\_60\_343 | T | T | A | W | M | D | I | Y | R | Q | I | R | T | N | V | - | - | Q | Q | N | K | E | F | L | A | Q | - | G | K | L | K | M | P | V | L | A | I | G | G | Q | - | - | - | D | S | F | - | - | - | - |
| 066 UniRef90\_A0A561IX74\_59\_327 | T | A | G | A | F | H | W | F | G | A | F | E | Q | D | A | - | - | R | D | N | K | E | L | M | K | T | - | - | K | L | K | M | P | L | L | A | M | G | G | E | - | - | - | Y | F | G | - | - | - | - |
| 067 UniRef90\_A0A0X3RYG7\_67\_350 | A | T | A | W | M | N | V | Y | R | G | L | R | T | D | V | - | - | Q | Q | N | K | E | F | L | A | Q | - | G | K | L | K | M | P | I | L | A | I | G | G | Q | - | - | - | Y | S | F | - | - | - | - |
| 068 UniRef90\_A0A2W2E1M5\_55\_338 | T | T | A | W | M | S | V | Y | R | G | L | R | A | D | V | - | - | Q | Q | N | K | E | F | L | A | Q | - | G | K | L | K | M | P | I | L | A | I | G | G | Q | - | - | - | N | S | F | - | - | - | - |
| 069 UniRef90\_A0A2V7YB42\_28\_306 | M | R | A | G | W | A | Y | F | V | S | F | P | Q | A | A | - | - | K | D | F | A | Q | L | S | R | T | - | - | R | L | T | M | P | V | L | A | I | G | G | E | - | - | - | K | A | N | - | - | - | - |
| 070 UniRef90\_A0A544XC49\_52\_336 | T | T | A | W | M | N | V | Y | R | G | L | R | T | D | V | - | - | H | R | N | K | E | F | L | A | Q | - | G | K | L | K | M | P | I | L | A | I | G | G | Q | - | - | - | N | S | F | - | - | - | - |
| 071 UniRef90\_A0A101PQ90\_46\_324 | L | E | A | S | F | A | W | F | R | T | F | P | Q | D | I | - | - | A | D | N | A | R | L | Q | K | R | - | - | P | L | T | M | P | V | L | A | I | G | A | D | - | - | - | G | S | L | - | - | - | - |
| 072 UniRef90\_A0A1C4VK67\_14\_288 | L | R | A | S | F | G | Y | F | R | A | F | G | Q | D | I | - | - | A | D | N | T | A | Y | R | A | T | - | - | K | L | P | M | P | V | L | A | V | G | A | R | - | - | - | A | S | L | - | - | - | - |
| 073 UniRef90\_A0A0X3RPC2\_57\_340 | T | T | A | W | M | S | V | Y | R | Q | I | R | T | D | V | - | - | Q | Q | N | K | E | F | L | A | Q | - | G | K | L | K | M | P | I | L | A | V | G | G | E | - | - | - | D | S | F | - | - | - | - |
| 074 UniRef90\_A0A4R2VWW4\_55\_319 | T | T | G | A | F | H | W | F | G | A | F | E | Q | D | A | - | - | K | D | N | K | E | F | M | K | T | - | - | K | L | K | M | P | L | L | A | M | G | G | E | - | - | - | Y | F | G | - | - | - | - |
| 075 UniRef90\_A0A1Q7VB11\_36\_307 | M | R | A | G | W | A | Y | F | V | S | F | Q | Q | A | A | - | - | K | D | F | A | E | L | S | Q | T | - | - | K | L | T | M | P | V | L | A | I | G | G | E | - | - | - | K | S | L | - | - | - | - |
| 076 UniRef90\_C7PGQ3\_38\_305 | T | T | G | A | F | H | W | F | G | A | F | E | Q | D | A | - | - | K | D | N | Q | E | F | M | K | T | - | - | K | L | K | M | P | L | L | A | M | G | G | E | - | - | - | Y | F | G | - | - | - | - |
| 077 UniRef90\_A0A1N7R4E6\_62\_325 | T | A | G | A | F | H | W | F | G | A | F | E | Q | D | A | - | - | K | D | N | Q | V | F | M | Q | T | - | - | K | L | K | M | P | L | L | A | M | G | G | E | - | - | - | Y | F | G | - | - | - | - |
| 078 UniRef90\_A0A2H2Y4V3\_41\_307 | L | R | A | A | F | A | Y | Y | R | A | F | P | Q | A | I | - | - | E | Q | N | K | E | Y | A | K | T | - | - | K | L | Q | M | P | V | L | A | L | G | G | E | - | - | - | S | V | L | - | - | - | - |
| 079 UniRef90\_A0A448SCU6\_8\_276 | L | R | A | G | F | E | F | Y | K | A | F | P | E | S | A | - | - | R | Q | S | R | E | L | A | A | R | - | - | K | L | D | I | P | V | M | A | W | G | G | S | - | - | - | H | C | M | - | - | - | - |
| 080 UniRef90\_A0A365Y5E2\_33\_303 | I | A | G | S | F | H | W | F | A | A | F | P | Q | D | A | - | - | R | D | N | Q | E | F | M | K | H | - | - | K | L | K | M | P | L | L | A | M | A | G | E | - | - | - | - | F | A | - | - | - | - |
| 081 UniRef90\_A0A1I2CFD2\_63\_327 | T | A | G | A | F | Q | W | F | A | A | F | E | Q | D | T | - | - | K | D | N | K | V | F | M | K | T | - | - | K | L | K | M | P | L | L | A | M | G | G | E | - | - | - | Y | F | G | - | - | - | - |
| 082 UniRef90\_A0A1G7Z850\_54\_322 | T | T | G | A | F | H | W | F | G | A | F | D | Q | D | A | - | - | K | D | N | K | V | F | M | K | T | - | - | K | L | K | M | P | L | L | A | M | G | G | E | - | - | - | Y | F | G | - | - | - | - |
| 083 UniRef90\_A0A2V9XG70\_35\_308 | M | R | A | G | W | A | Y | F | V | S | F | Q | Q | A | A | - | - | K | D | F | A | Q | L | S | Q | T | - | - | K | L | P | M | P | V | L | V | I | G | G | E | - | - | - | K | A | L | - | - | - | - |
| 084 UniRef90\_A0A0Q4GSS4\_39\_308 | T | T | G | A | F | H | W | F | G | A | F | T | Q | D | A | - | - | K | D | N | L | E | L | A | K | T | - | - | K | L | S | M | P | V | L | A | M | G | S | D | - | - | - | H | F | A | - | - | - | - |
| 085 UniRef90\_A0A0M9ZTU1\_1\_271 | L | T | A | W | L | S | V | Y | R | A | V | H | A | D | V | - | - | Q | Q | N | K | E | F | L | A | H | - | G | K | L | R | M | P | I | L | A | V | G | G | Q | - | - | - | Y | S | F | - | - | - | - |
| 086 UniRef90\_A0A239P597\_1\_270 | T | T | A | W | M | S | V | Y | R | G | L | R | T | D | V | - | - | Q | Q | N | K | K | F | L | A | Q | - | G | K | L | K | M | P | I | L | A | I | G | G | E | - | - | - | R | S | F | - | - | - | - |
| 087 UniRef90\_UPI00128E3D53\_19\_294 | M | T | A | G | F | N | L | Y | K | A | M | P | Q | D | S | - | - | A | L | A | E | T | F | H | G | K | - | - | P | L | P | F | P | V | M | T | V | A | G | R | - | - | - | H | G | A | - | - | - | - |
| 088 UniRef90\_A0A1V9E1W0\_57\_321 | T | T | G | A | F | H | W | F | G | A | F | E | Q | D | A | - | - | K | D | N | K | V | F | M | K | T | - | - | K | L | K | M | P | L | L | A | M | G | G | E | - | - | - | Y | S | A | - | - | - | - |
| 089 UniRef90\_A0A239MTD5\_1\_269 | T | T | A | W | M | N | V | Y | R | Q | F | R | T | N | V | - | - | Q | Q | N | K | E | F | L | A | R | - | G | K | L | K | M | P | I | L | A | I | G | A | Q | - | - | - | D | S | F | - | - | - | - |
| 090 UniRef90\_UPI0009FCBBE8\_54\_317 | R | S | A | G | F | D | Y | Y | R | A | Y | P | T | N | A | - | - | A | D | N | K | R | L | A | E | Q | - | - | K | L | T | I | P | V | M | A | M | G | A | S | - | - | - | D | V | F | - | - | - | - |
| 091 UniRef90\_A0A1A9HTL2\_19\_299 | A | R | A | A | F | N | Y | Y | R | A | A | F | S | D | T | G | L | Q | Q | N | R | L | R | A | A | T | - | - | P | L | T | I | P | V | L | A | I | G | G | Q | - | - | - | H | S | V | - | - | - | - |
| 092 UniRef90\_A0A4R7CM41\_23\_285 | M | E | G | S | F | H | W | Y | S | T | F | E | T | D | A | - | - | L | H | N | K | Q | F | M | E | Q | - | - | K | L | T | M | P | V | L | A | M | G | G | E | - | - | - | F | D | T | - | - | - | - |
| 093 UniRef90\_A0A562QYK6\_25\_307 | M | K | A | G | M | A | Y | F | S | N | F | E | Q | D | A | - | - | K | D | F | A | E | L | G | K | T | - | - | K | L | P | M | P | L | L | V | I | A | G | E | - | - | - | K | A | S | - | - | - | - |
| 094 UniRef90\_A0A2V9ZM06\_3\_269 | M | R | A | G | W | A | Y | F | V | S | F | Q | Q | A | A | - | - | K | D | F | A | Q | L | S | Q | T | - | - | K | L | T | M | P | V | L | V | I | G | G | E | - | - | - | K | S | L | - | - | - | - |
| 095 UniRef90\_A0A4P8YF40\_11\_271 | L | R | A | G | F | E | F | Y | K | A | F | P | E | S | A | - | - | R | Q | S | R | E | L | A | A | H | - | - | K | L | D | I | P | V | M | A | W | G | G | S | - | - | - | H | C | M | - | - | - | - |
| 096 UniRef90\_A0A2V6E8I4\_30\_303 | M | R | A | A | W | A | Y | F | A | S | W | P | E | L | A | - | - | K | D | F | A | Q | L | S | Q | T | - | - | K | L | T | M | P | V | L | S | I | G | G | E | - | - | - | K | S | L | - | - | - | - |
| 097 UniRef90\_A0A2V9BFT0\_23\_295 | M | K | A | G | W | A | Y | F | V | S | F | Q | Q | A | A | - | - | R | D | F | E | K | L | S | Q | T | - | - | K | L | T | M | P | V | L | S | I | G | G | D | - | - | - | K | S | L | - | - | - | - |
| 098 UniRef90\_A0A2V9H5C8\_29\_298 | M | R | A | G | W | A | Y | F | V | S | F | P | Q | A | A | - | - | K | D | F | V | Q | L | A | K | T | - | - | K | L | T | I | P | V | L | A | I | G | G | E | - | - | - | K | S | L | - | - | - | - |
| 099 UniRef90\_A0A101CRV3\_44\_308 | T | T | G | S | F | H | W | F | G | A | F | A | Q | D | A | - | - | K | D | N | T | E | L | M | K | T | - | - | K | L | N | M | P | V | L | T | M | G | G | Q | - | - | - | Y | F | A | - | - | - | - |
| 100 UniRef90\_A0A5M6CFP9\_57\_321 | T | T | G | A | F | H | W | F | G | A | F | E | Q | D | A | - | - | A | D | N | K | E | F | M | K | H | - | - | K | L | K | M | P | V | L | A | M | G | G | E | - | - | - | Y | F | G | - | - | - | - |
| 101 UniRef90\_A0A401Z9D4\_19\_284 | L | R | A | G | F | E | Y | Y | R | S | F | S | T | N | A | - | - | Q | Q | F | R | A | Y | S | K | E | - | - | K | L | H | I | P | V | L | A | L | G | G | E | - | - | - | Y | S | G | - | - | - | - |
| 102 UniRef90\_A0A2V8XJ58\_32\_308 | M | R | A | G | W | S | Y | F | V | S | F | Q | Q | A | A | - | - | K | D | F | A | Q | L | S | Q | T | - | - | K | L | T | M | P | V | L | A | I | G | G | D | - | - | - | K | S | L | - | - | - | - |
| 103 UniRef90\_A0A252EMP1\_15\_277 | M | R | A | G | F | N | Y | Y | R | A | F | P | E | T | A | - | - | V | Q | S | R | E | L | A | R | T | - | - | K | L | T | I | P | V | L | A | Y | G | G | S | - | - | - | H | C | M | - | - | - | - |
| 104 UniRef90\_A0A1H3H759\_34\_310 | L | R | A | S | F | E | F | Y | R | A | I | D | T | T | I | - | - | E | Q | N | A | R | R | K | E | R | - | - | K | L | T | M | P | V | L | T | I | A | G | A | - | - | - | R | S | V | - | - | - | - |
| 105 UniRef90\_A0A4D7B7N1\_3\_270 | L | R | G | A | L | G | Y | Y | R | A | V | F | E | D | I | - | - | E | Q | N | K | H | L | A | N | I | - | - | R | L | K | T | P | I | L | A | L | G | G | D | - | - | - | V | G | M | - | - | - | - |
| 106 UniRef90\_A0A1Q7MUT6\_34\_308 | M | R | A | A | W | A | Y | F | V | S | F | Q | Q | A | A | - | - | K | D | F | A | Q | L | S | Q | T | - | - | K | L | T | M | P | V | L | V | I | G | G | E | - | - | - | K | S | L | - | - | - | - |
| 107 UniRef90\_A0A2V6KQW0\_24\_297 | M | R | A | A | W | A | Y | F | A | S | W | P | Q | L | A | - | - | K | E | F | A | Q | L | S | Q | T | - | - | K | L | T | M | P | V | L | S | I | G | G | D | - | - | - | K | S | L | - | - | - | - |
| 108 UniRef90\_A0A4R6LFZ5\_44\_308 | T | T | G | S | F | H | W | F | G | A | F | A | E | D | G | - | - | K | D | N | L | E | F | M | K | T | - | - | K | L | N | M | P | V | L | T | M | G | G | Q | - | - | - | Y | F | A | - | - | - | - |
| 109 UniRef90\_A0A1H7UU25\_49\_318 | T | Y | G | A | F | H | W | F | A | A | F | N | D | D | A | - | - | L | D | N | K | E | F | M | K | H | - | - | K | L | K | M | P | L | L | A | M | A | G | E | - | - | - | Y | - | A | - | - | - | - |
| 110 UniRef90\_UPI0008326796\_14\_259 | L | R | A | G | L | A | F | Y | R | S | A | A | H | S | A | - | - | D | Q | N | K | V | L | A | A | G | - | G | K | L | T | V | P | L | L | A | L | S | A | D | - | - | - | Q | G | S | - | - | - | - |
| 111 UniRef90\_A0A2V5S8F2\_33\_306 | M | R | A | G | W | A | Y | F | V | S | F | Q | Q | A | A | - | - | K | D | F | A | Q | L | S | Q | T | - | - | K | L | T | M | P | V | L | S | I | G | G | E | - | - | - | K | S | L | - | - | - | - |
| 112 UniRef90\_A0A3S3VTN8\_36\_308 | T | T | G | A | F | H | W | F | G | E | F | A | N | D | G | - | - | K | D | N | V | E | F | A | K | T | - | - | K | L | T | I | P | L | L | A | M | G | S | D | - | - | - | H | F | A | - | - | - | - |
| 113 UniRef90\_A0A6J4PSU4\_16\_248 | L | R | G | G | F | E | H | Y | R | T | F | F | Q | S | V | - | - | R | Q | N | R | E | L | A | G | R | - | G | K | L | G | M | P | V | L | A | L | G | G | D | - | - | - | H | S | V | - | - | - | - |
| 114 UniRef90\_A0A534ZXC6\_26\_300 | M | R | A | G | W | A | Y | F | V | S | F | Q | Q | T | A | - | - | K | D | F | A | E | L | S | K | T | - | - | K | L | T | M | P | V | L | A | I | G | G | A | - | - | - | K | A | N | - | - | - | - |
| 115 UniRef90\_A0A2A9KBX9\_78\_358 | P | R | A | A | F | S | Y | Y | R | V | A | F | S | D | S | G | L | K | Q | N | R | L | R | A | Q | T | - | - | K | L | T | M | P | V | L | A | V | G | G | Q | - | - | - | F | G | V | - | - | - | - |
| 116 UniRef90\_A0A2W0BWH7\_16\_287 | M | H | A | A | W | A | Y | F | V | S | F | Q | Q | A | A | - | - | K | D | F | A | E | L | S | Q | T | - | - | K | L | T | M | P | V | L | A | I | G | G | E | - | - | - | K | S | L | - | - | - | - |
| 117 UniRef90\_A0A1R1RMU1\_30\_310 | L | R | A | G | L | A | Y | Y | R | D | A | S | M | S | A | - | - | Q | Q | N | R | E | L | S | A | L | - | G | K | L | K | T | P | I | L | A | L | G | A | D | - | - | - | Q | G | S | - | - | - | - |
| 118 UniRef90\_A0A3A1NH59\_38\_305 | T | T | G | S | F | H | W | F | G | D | F | Q | K | D | V | - | - | I | D | N | H | E | F | M | K | H | - | - | K | L | E | M | P | M | L | A | M | G | G | E | - | - | - | Y | F | S | - | - | - | - |
| 119 UniRef90\_A0A0Q6WP84\_43\_317 | V | R | A | G | F | Q | Y | F | K | T | F | P | Q | D | A | - | - | E | Q | F | A | E | L | G | K | T | - | - | K | L | T | M | P | M | L | V | L | A | G | E | - | - | - | K | S | G | - | - | - | - |
| 120 UniRef90\_A0A2V6P1A8\_15\_289 | M | R | A | A | W | A | Y | F | A | S | W | P | E | L | A | - | - | K | D | F | A | Q | L | S | Q | T | - | - | K | L | T | M | P | V | L | S | I | G | G | E | - | - | - | K | S | L | - | - | - | - |
| 121 UniRef90\_A0A2V5NC55\_35\_307 | M | R | A | A | W | A | Y | F | A | S | W | P | Q | L | A | - | - | T | Q | F | A | Q | L | S | K | T | - | - | K | L | T | M | P | V | L | S | I | G | G | E | - | - | - | K | S | L | - | - | - | - |
| 122 UniRef90\_A0A2V9BM40\_39\_318 | M | R | A | G | W | A | Y | F | V | S | F | Q | Q | A | A | - | - | K | D | F | A | E | L | S | Q | T | - | - | K | L | T | M | P | V | L | A | I | G | G | G | - | - | - | K | A | N | - | - | - | - |
| 123 UniRef90\_A0A5B8WA72\_34\_302 | T | T | G | A | F | H | W | F | G | Y | F | N | Q | D | A | - | - | K | D | N | V | E | L | A | R | H | - | - | K | L | T | M | P | V | L | A | M | G | S | E | - | - | - | H | F | A | - | - | - | - |
| 124 UniRef90\_A0A1Y6D2S0\_25\_299 | M | T | A | G | F | N | Y | Y | R | A | L | L | D | D | A | - | - | Q | F | V | A | A | Y | A | D | Q | - | - | K | F | A | M | P | V | L | A | V | A | G | R | - | - | - | Y | G | V | - | - | - | - |
| 125 UniRef90\_A0A494W0L3\_12\_284 | T | T | G | A | F | K | W | F | A | A | F | E | Q | D | A | - | - | R | D | N | V | E | F | A | K | N | - | - | K | L | P | M | P | L | L | A | M | G | S | D | - | - | - | H | L | A | - | - | - | - |
| 126 UniRef90\_UPI0015CD0065\_39\_309 | R | T | A | G | Y | E | Y | Y | R | A | F | A | A | D | A | - | - | E | N | N | K | A | G | A | G | R | - | - | K | L | T | A | P | V | L | A | V | G | G | Q | - | - | - | Y | S | F | - | - | - | - |
| 127 UniRef90\_UPI00166E2F5D\_15\_289 | L | K | A | G | F | D | F | Y | R | S | F | D | Q | D | Q | - | - | K | D | N | A | A | T | K | E | K | - | - | S | I | S | I | P | I | L | Y | L | R | G | E | - | - | - | N | E | Q | - | - | - | V |
| 128 UniRef90\_A0A372NPX1\_36\_305 | T | T | G | A | F | H | W | F | G | Y | F | D | Q | D | A | - | - | K | D | N | V | A | F | A | K | N | - | - | K | L | T | M | P | V | L | A | M | G | S | D | - | - | - | H | F | A | - | - | - | - |
| 129 UniRef90\_A0A317JA08\_13\_279 | M | R | S | G | W | A | Y | F | A | A | W | P | D | T | A | - | - | K | D | F | A | E | M | A | K | T | - | - | K | L | T | M | P | V | L | S | I | A | G | E | - | - | - | K | A | S | - | - | - | - |
| 130 UniRef90\_A0A2V6N4R6\_10\_283 | M | R | A | A | W | A | Y | F | A | S | W | P | Q | L | A | - | - | K | D | F | A | Q | L | S | Q | T | - | - | K | L | T | V | P | V | L | S | I | G | G | E | - | - | - | K | S | L | - | - | - | - |
| 131 UniRef90\_A0A2V5IZ05\_29\_302 | M | R | A | G | W | A | Y | F | V | S | F | M | Q | A | A | - | - | K | D | F | E | Q | L | S | R | T | - | - | K | L | T | M | P | V | L | A | I | G | G | E | - | - | - | K | A | N | - | - | - | - |
| 132 UniRef90\_A0A521E7V8\_39\_308 | T | T | G | A | F | H | W | F | G | Y | F | D | E | D | A | - | - | K | D | N | V | E | F | A | K | H | - | - | K | L | P | M | P | V | L | A | M | G | S | D | - | - | - | H | F | A | - | - | - | - |
| 133 UniRef90\_UPI001665F59F\_18\_290 | L | K | A | G | F | D | F | Y | R | W | F | P | Q | D | Q | - | - | K | D | N | V | A | L | K | D | Q | - | - | K | V | S | I | P | V | L | Y | L | R | G | E | - | - | - | D | E | Q | - | - | - | I |
| 134 UniRef90\_A0A0Q9GGK2\_19\_284 | L | R | A | G | F | E | S | Y | R | T | L | F | E | D | R | - | - | E | H | N | K | K | Y | L | E | K | - | - | K | L | N | M | P | I | L | A | Y | G | G | E | - | - | - | I | S | V | - | - | - | - |
| 135 UniRef90\_A0A329YBZ0\_37\_320 | L | R | A | G | F | E | L | Y | R | A | F | P | E | D | E | - | - | R | R | F | A | E | F | E | K | R | - | - | K | L | P | M | P | I | L | A | L | A | G | D | - | - | - | K | S | N | - | - | - | - |
| 136 UniRef90\_A0A2E0H9P2\_13\_236 | L | R | A | G | F | S | Y | Y | R | A | M | G | K | D | A | - | - | E | D | N | S | K | V | I | A | N | - | F | K | L | P | M | P | V | L | A | I | G | G | G | - | - | - | V | S | - | - | - | - | - |
| 137 UniRef90\_A0A095SYG7\_44\_308 | T | T | G | S | F | H | W | F | G | A | F | K | Q | D | G | - | - | I | D | N | V | E | F | M | K | T | - | - | K | L | N | M | P | I | L | T | M | G | G | Q | - | - | - | Y | F | A | - | - | - | - |
| 138 UniRef90\_A0A2U0ZNC3\_54\_322 | T | T | G | S | F | H | W | F | G | A | F | P | Q | D | A | - | - | V | D | N | K | E | L | M | K | N | - | - | K | L | K | M | P | V | L | A | M | G | A | E | - | - | - | F | G | S | - | - | - | - |
| 139 UniRef90\_A0A2H2XXU1\_20\_287 | M | R | A | G | F | E | Y | Y | R | T | L | L | K | D | G | - | - | Q | Q | N | R | E | S | A | N | T | - | - | K | L | A | V | P | V | L | V | L | T | G | S | - | D | - | S | G | I | - | - | - | - |
| 140 UniRef90\_A0A0P4V0Y0\_11\_278 | I | R | G | A | L | G | V | Y | R | A | I | F | E | S | V | - | - | Q | Q | T | E | A | I | A | N | H | - | - | P | I | Q | T | P | V | L | A | L | G | G | E | - | - | - | K | S | I | - | - | - | - |
| 141 UniRef90\_A0A2D7FF16\_11\_288 | L | R | A | G | F | N | F | Y | R | S | I | G | Q | D | A | - | - | A | D | N | A | A | L | L | K | T | G | F | K | L | P | M | P | V | L | A | I | G | G | G | - | - | - | V | S | Y | P | H | G | R |
| 142 UniRef90\_UPI0009FE04F9\_77\_354 | L | H | A | G | F | E | L | Y | R | T | W | S | A | D | E | - | - | A | E | N | K | R | L | Q | Q | T | - | - | P | L | T | I | P | V | R | M | L | T | Q | D | - | - | - | - | G | L | - | - | - | - |
| 143 UniRef90\_UPI00148816F0\_34\_297 | L | H | N | G | S | R | Y | Y | Q | A | W | P | Q | D | E | - | - | V | D | N | R | L | A | M | A | L | - | - | P | L | A | I | P | V | H | V | L | A | Q | A | - | - | - | - | P | L | - | - | - | - |
| 144 UniRef90\_A0A2V5MXX8\_31\_304 | M | R | A | A | W | T | Y | F | A | S | W | P | Q | L | A | - | - | K | D | F | A | Q | L | S | Q | T | - | - | K | L | T | I | P | V | L | S | I | G | G | D | - | - | - | K | A | N | - | - | - | - |
| 145 UniRef90\_Q1W504\_15\_282 | L | R | G | G | F | E | H | Y | R | A | F | T | V | D | A | - | - | I | N | N | K | E | N | A | S | T | - | - | K | L | P | M | P | V | L | G | I | G | G | S | - | - | - | H | G | M | - | - | - | - |
| 146 UniRef90\_UPI001661900D\_10\_259 | L | S | R | A | F | S | Y | Y | R | A | L | P | E | S | A | - | A | R | I | G | Q | A | V | A | A | R | - | - | R | L | T | V | P | T | M | A | L | G | A | Q | - | - | - | - | P | V | - | - | - | - |
| 147 UniRef90\_A0A2D6SJL3\_11\_235 | M | R | A | G | F | S | Y | Y | R | E | L | P | K | D | I | - | - | E | D | N | L | E | N | I | K | R | - | L | K | L | P | M | P | V | L | A | I | G | G | G | - | - | - | V | - | - | - | - | - | - |
| 148 UniRef90\_A0A2V6J615\_3\_225 | M | R | A | A | W | A | Y | F | A | S | W | P | Q | L | A | - | - | K | D | F | A | Q | L | S | Q | T | - | - | K | L | A | M | P | V | L | S | I | G | G | D | - | - | - | K | S | L | - | - | - | - |

  
  

|  |  |  |  |  |  |  |  |  |  |  |  |  |  |  |  |  |  |  |  |  |  |  |  |  |  |  |  |  |  |  |  |  |  |  |  |  |  |  |  |  |  |  |  |  |  |  |  |  |  |  |
| --- | --- | --- | --- | --- | --- | --- | --- | --- | --- | --- | --- | --- | --- | --- | --- | --- | --- | --- | --- | --- | --- | --- | --- | --- | --- | --- | --- | --- | --- | --- | --- | --- | --- | --- | --- | --- | --- | --- | --- | --- | --- | --- | --- | --- | --- | --- | --- | --- | --- | --- |
| **001 Input\_protein\_seq** | - | - | N | D | V | L | V | K | E | M | R | A | R | F | V | A | D | P | A | Q | Y | T | A | I | I | L | P | D | T | G | H | W | M | V | E | E | N | A | E | G | V | E | K | S | L | S | N | F | L | F |
| 002 UniRef90\_D8JE57\_1\_349 | - | - | N | D | V | L | I | K | E | M | R | A | K | F | V | E | D | P | A | K | L | E | A | V | I | L | P | N | T | G | H | W | L | I | E | E | N | P | E | G | V | E | K | S | L | S | K | F | L | F |
| 003 UniRef90\_A0A6B8MSH7\_6\_345 | - | - | N | D | V | L | V | K | E | M | R | S | R | F | V | Q | D | P | Q | N | F | K | G | V | I | L | P | D | T | G | H | W | M | V | E | E | N | P | T | A | V | S | D | E | L | S | Q | F | L | F |
| 004 UniRef90\_A0A6B2FNS7\_11\_352 | - | - | N | D | V | L | V | K | E | M | K | A | R | F | V | A | D | P | A | Q | F | T | G | I | I | L | P | D | T | G | H | W | M | V | E | E | N | A | S | G | V | T | N | A | L | S | S | F | L | F |
| 005 UniRef90\_UPI00073D803C\_6\_344 | - | - | N | D | V | L | V | K | E | M | R | S | R | F | V | K | D | R | Q | N | F | T | G | V | I | L | P | N | T | G | H | W | M | V | E | E | N | A | S | G | V | I | K | Q | L | E | N | F | L | - |
| 006 UniRef90\_A0A285K5K8\_3\_346 | - | - | N | D | V | L | V | N | E | M | R | S | R | F | V | T | D | K | Q | Q | F | K | G | V | I | L | P | E | T | G | H | W | M | V | E | E | N | A | P | G | V | I | K | E | L | E | T | F | L | F |
| 007 UniRef90\_A0A379I9D6\_1\_346 | - | - | N | D | V | L | T | K | Q | M | A | A | R | F | V | S | D | A | S | Q | Y | T | G | K | V | L | P | G | C | G | H | W | M | V | E | E | C | A | P | Q | V | T | A | E | L | S | R | F | L | - |
| 008 UniRef90\_A0A6B2FTI5\_15\_352 | - | - | N | T | V | L | V | N | E | M | K | T | R | F | V | A | N | P | A | Q | F | T | G | T | I | L | P | D | T | G | H | W | M | T | E | E | S | A | P | E | V | I | N | A | L | S | S | F | L | L |
| 009 UniRef90\_A0A2P9HD85\_2\_318 | - | - | N | D | V | L | P | K | Q | L | A | A | R | F | V | K | D | P | S | K | L | G | S | T | I | L | P | D | T | G | H | W | L | L | E | E | S | A | T | E | V | N | A | L | L | A | D | F | I | - |
| 010 UniRef90\_A0A1T1H7K2\_15\_316 | - | - | G | Q | F | Q | V | D | Q | M | K | Q | Y | - | - | - | - | A | T | N | V | T | G | K | V | I | P | N | C | G | H | W | L | P | E | E | C | A | P | E | L | N | K | T | V | G | D | F | L | - |
| 011 UniRef90\_A0A1A9HKQ2\_27\_313 | - | - | G | Q | F | Q | A | D | Q | M | K | R | Y | - | - | - | - | A | T | D | V | T | G | V | V | L | P | A | C | G | H | W | L | P | E | E | C | A | A | P | L | N | D | A | V | T | G | F | L | - |
| 012 UniRef90\_A0A0J6GRA0\_22\_303 | - | - | G | Q | L | Q | V | D | Q | M | K | E | Y | - | - | - | - | A | T | N | V | E | G | Q | V | L | P | N | C | G | H | W | L | P | E | E | C | S | A | - | - | - | - | - | - | - | - | - | - | - |
| 013 UniRef90\_UPI000F5BD18A\_25\_314 | - | - | G | D | Y | Q | R | Q | V | V | S | R | Y | - | - | - | - | A | N | N | V | T | G | K | V | L | P | D | C | G | H | W | L | P | E | E | C | T | A | P | L | N | E | A | V | L | E | F | L | S |
| 014 UniRef90\_A0A4V1G750\_26\_311 | - | - | G | Q | F | Q | V | D | Q | L | R | R | Y | - | - | - | - | A | S | N | V | T | G | K | V | L | P | G | C | G | H | W | L | P | E | E | C | P | G | P | L | N | S | A | V | I | D | - | - | - |
| 015 UniRef90\_U3U231\_21\_300 | - | - | G | A | Y | E | Q | Q | V | V | S | R | Y | - | - | - | - | A | S | Q | V | T | G | K | V | L | P | G | C | G | H | W | L | P | E | E | - | - | - | - | - | - | - | - | - | - | - | - | - | - |
| 016 UniRef90\_F2LPZ2\_26\_313 | - | - | G | Q | F | E | A | D | Q | L | R | R | Y | - | - | - | - | A | T | Q | V | S | G | R | V | L | P | D | C | G | H | W | L | P | E | E | C | A | A | P | L | N | Q | A | V | I | D | F | L | - |
| 017 UniRef90\_A0A3M4V4S1\_6\_330 | - | - | G | A | Q | Q | P | E | N | I | R | R | Y | - | - | - | - | A | T | N | V | E | S | H | M | L | P | G | C | G | H | W | V | P | E | E | C | A | T | A | L | N | P | L | I | N | S | F | L | - |
| 018 UniRef90\_A0A178LP42\_49\_332 | - | - | G | Q | F | Q | V | D | Q | L | A | K | Y | - | - | - | - | T | T | N | M | R | G | V | V | L | P | N | C | G | H | W | V | P | E | E | C | A | E | S | F | N | P | T | I | A | N | F | L | - |
| 019 UniRef90\_T2L220\_20\_307 | - | - | G | Q | Y | Q | I | D | M | M | R | H | Y | - | - | - | - | A | S | D | V | T | G | I | V | L | H | G | G | G | H | W | L | P | E | E | C | P | E | A | L | N | K | A | V | M | E | F | L | - |
| 020 UniRef90\_UPI0014749FF6\_22\_304 | - | - | G | Q | T | Q | I | D | Q | M | N | E | Y | - | - | - | - | A | T | N | V | Q | G | H | V | L | N | G | C | G | H | W | L | M | E | E | C | P | S | L | V | K | - | - | - | - | - | - | - | - |
| 021 UniRef90\_A0A172YJZ3\_18\_305 | - | - | G | Q | F | Q | V | D | Q | M | Q | R | Y | - | - | - | - | A | T | D | V | T | G | L | V | M | P | G | C | G | H | W | L | P | E | E | C | T | A | Q | L | N | S | A | V | S | D | F | L | - |
| 022 UniRef90\_UPI000737C6BF\_26\_313 | - | - | G | Q | F | Q | V | D | Q | M | R | R | Y | - | - | - | - | A | S | D | V | T | G | L | V | L | P | G | C | G | H | W | L | P | E | E | C | V | E | P | L | N | A | A | V | L | E | F | L | - |
| 023 UniRef90\_A0A329J6I4\_26\_309 | - | - | G | Q | F | Q | I | D | Q | T | K | K | Y | - | - | - | - | A | T | N | V | K | G | E | I | L | P | G | C | G | H | W | L | P | E | E | C | A | A | K | L | N | A | V | - | - | - | - | - | - |
| 024 UniRef90\_A0A2N3KZL1\_30\_325 | - | - | G | Q | L | E | A | D | Q | L | G | E | Y | - | - | - | - | G | T | H | V | K | G | L | V | I | P | D | C | G | H | W | L | P | E | E | C | A | K | P | L | N | D | A | V | L | D | F | L | - |
| 025 UniRef90\_UPI0006CA847F\_27\_323 | - | - | G | D | A | F | L | N | Q | V | K | K | Y | - | - | - | - | G | D | N | V | Q | G | E | T | I | Q | G | C | G | H | W | L | P | E | E | C | P | A | T | V | E | P | L | V | I | D | F | L | N |
| 026 UniRef90\_UPI00054E0E46\_26\_312 | - | - | G | T | Y | Q | I | E | Q | I | G | H | F | - | - | - | - | A | T | N | V | T | G | K | V | L | T | G | C | G | H | W | L | P | E | E | C | P | G | Q | L | N | N | A | V | M | S | F | - | - |
| 027 UniRef90\_A0A0S9PRE2\_29\_311 | - | - | G | E | A | Q | A | E | Q | V | R | R | Y | - | - | - | - | A | S | D | V | T | G | V | S | L | P | G | C | G | H | W | L | P | E | E | C | A | T | A | L | N | P | L | V | T | D | F | L | - |
| 028 UniRef90\_W0AA74\_37\_318 | - | - | G | R | F | Q | I | D | Q | L | A | K | Y | - | - | - | - | T | T | N | M | K | G | V | V | I | P | N | C | G | H | W | V | P | E | E | C | A | A | S | F | H | T | T | I | I | D | F | L | - |
| 029 UniRef90\_A0A2G0Y5Q3\_27\_312 | - | - | G | Q | F | Q | I | D | Q | M | K | E | Y | - | - | - | - | A | N | D | V | E | G | H | I | L | P | G | C | G | H | W | L | P | E | E | C | A | A | P | M | N | E | L | V | V | K | F | L | - |
| 030 UniRef90\_A0A1M6LZ92\_62\_349 | - | - | G | E | Y | E | N | Q | T | V | S | R | Y | - | - | - | - | A | D | H | V | T | G | K | V | L | P | D | C | G | H | W | L | P | E | E | C | A | G | P | L | N | Q | A | V | V | D | F | L | - |
| 031 UniRef90\_A0A4Z1C7D3\_28\_313 | - | - | G | Q | F | Q | V | D | Q | L | R | Q | Y | - | - | - | - | G | T | D | V | E | G | L | V | I | P | D | C | G | H | W | L | P | E | E | C | A | A | P | L | N | E | A | V | V | Q | F | L | - |
| 032 UniRef90\_A0A367MBG0\_273\_561 | - | - | G | T | F | Q | L | E | Q | M | K | A | Y | - | - | - | - | A | E | D | V | E | G | H | V | L | P | G | C | G | H | W | L | P | E | E | C | A | A | P | M | N | R | L | V | I | D | F | L | - |
| 033 UniRef90\_UPI00067CEA54\_35\_318 | - | - | G | Q | F | Q | V | D | Q | L | A | K | Y | - | - | - | - | A | T | N | M | K | G | V | V | I | P | N | C | G | H | W | M | P | E | E | C | P | A | E | L | N | S | T | I | I | N | F | L | - |
| 034 UniRef90\_A0A1Y0L5M5\_31\_311 | - | - | G | Q | F | Q | I | D | Q | M | K | E | Y | - | - | - | - | A | T | N | V | E | G | H | V | L | A | G | C | G | H | W | L | P | E | E | C | P | E | Q | L | N | - | - | - | - | - | - | - | - |
| 035 UniRef90\_UPI000C2FE79C\_29\_313 | - | - | G | E | Y | E | S | Q | V | V | S | H | Y | - | - | - | - | A | D | N | V | T | A | K | V | L | P | D | C | G | H | W | L | P | E | E | C | A | A | P | L | N | D | A | V | I | G | F | L | - |
| 036 UniRef90\_A0A4P8SJX7\_14\_295 | - | - | G | Q | F | Q | A | D | Q | M | K | N | Y | - | - | - | - | A | T | R | V | E | G | H | V | L | P | D | C | G | H | W | L | P | E | E | C | P | Q | Q | L | N | - | - | - | - | - | - | - | - |
| 037 UniRef90\_A0A1I1BZT3\_28\_315 | - | - | G | Q | L | Q | V | D | Q | L | R | E | Y | - | - | - | - | G | T | N | V | E | G | V | V | I | P | D | C | G | H | W | L | P | E | E | C | S | A | A | L | N | V | A | V | I | K | F | L | T |
| 038 UniRef90\_UPI000DF0D36E\_15\_290 | - | - | G | Q | F | Q | V | D | Q | M | K | D | Y | - | - | - | - | A | T | K | V | E | G | H | V | L | A | G | C | G | H | W | L | P | E | E | C | - | - | - | - | - | - | - | - | - | - | - | - | - |
| 039 UniRef90\_UPI00136B8797\_37\_318 | - | - | G | Q | F | Q | V | D | Q | L | A | K | Y | - | - | - | - | T | T | N | M | K | G | V | V | I | P | N | C | G | H | W | V | P | E | E | C | T | E | T | F | N | A | T | I | I | D | F | L | - |
| 040 UniRef90\_A0A1H4CLJ1\_28\_313 | - | - | G | E | N | Q | A | N | Q | M | R | K | Y | - | - | - | - | A | T | N | V | V | G | K | V | I | P | G | S | G | H | W | I | P | E | E | S | P | Q | E | L | N | S | A | V | I | E | F | L | - |
| 041 UniRef90\_UPI000E1FE28F\_29\_313 | - | - | G | A | Y | E | A | K | V | V | S | H | Y | - | - | - | - | A | E | H | V | T | G | K | V | L | D | G | C | G | H | W | L | P | E | E | C | P | V | A | L | N | K | A | V | T | R | F | L | - |
| 042 UniRef90\_A0A556AX95\_26\_313 | - | - | G | Q | Y | Q | V | D | Q | M | Q | R | Y | - | - | - | - | A | T | D | V | T | G | L | V | L | P | G | C | G | H | W | L | P | E | E | C | A | A | P | L | N | A | A | V | V | D | F | L | - |
| 043 UniRef90\_A0A6M8HNY7\_38\_322 | - | - | G | K | F | E | G | D | Q | L | R | D | Y | - | - | - | - | A | T | N | V | Q | G | E | V | L | P | G | C | G | H | W | L | P | E | E | C | P | A | T | L | D | P | M | V | V | N | F | L | - |
| 044 UniRef90\_UPI001612B72A\_24\_301 | - | - | G | A | L | Q | G | E | Q | T | R | E | Y | - | - | - | - | A | K | N | V | R | A | E | V | L | P | G | C | G | H | W | L | P | E | E | C | G | - | - | - | - | - | - | - | - | - | - | - | - |
| 045 UniRef90\_UPI0012F66D09\_46\_328 | - | - | G | K | L | Q | G | E | Q | L | S | E | Y | - | - | - | - | A | I | N | V | T | K | K | V | L | P | D | C | G | H | W | L | P | E | E | C | S | A | A | L | N | P | I | V | V | N | F | L | - |
| 046 UniRef90\_UPI000A94940B\_29\_316 | - | - | G | Q | Y | Q | I | D | M | M | K | G | F | - | - | - | - | A | T | N | V | T | G | V | V | L | P | D | C | G | H | W | L | P | E | E | C | A | A | P | L | N | A | A | V | T | K | F | L | - |
| 047 UniRef90\_UPI00102FACD2\_29\_312 | - | - | G | E | L | Q | V | N | Q | I | R | H | F | - | - | - | - | A | T | N | V | T | G | K | V | L | P | G | C | G | H | W | L | P | E | E | C | S | F | A | L | N | S | A | V | I | T | F | - | - |
| 048 UniRef90\_A0A1S8YNF4\_19\_297 | - | - | G | Q | F | Q | V | D | Q | M | K | Q | Y | - | - | - | - | A | T | D | V | Q | G | H | V | L | S | G | G | G | H | W | L | P | E | E | C | P | - | - | - | - | - | - | - | - | - | - | - | - |
| 049 UniRef90\_UPI0015755320\_14\_301 | - | - | G | Q | L | Q | I | D | Q | L | K | E | Y | - | - | - | - | A | E | D | V | Q | G | A | L | L | P | H | G | G | H | W | L | P | E | E | C | P | E | E | L | N | R | L | V | V | D | F | L | - |
| 050 UniRef90\_A0A427Q4N3\_22\_313 | - | - | G | E | F | Q | A | E | Q | M | R | R | Y | - | - | - | - | G | T | D | V | T | G | K | V | L | E | G | C | G | H | W | L | P | E | E | C | S | G | A | L | N | A | E | V | T | G | F | L | - |
| 051 UniRef90\_A0A0A3Z1S4\_30\_318 | - | - | G | Q | F | Q | V | D | Q | M | K | D | Y | - | - | - | - | A | T | K | V | E | G | H | V | L | A | G | C | G | H | W | L | P | E | E | C | P | R | E | L | N | P | L | V | V | N | F | I | - |
| 052 UniRef90\_A0A3L8C981\_35\_323 | - | - | G | A | Q | Q | P | E | N | I | H | R | Y | - | - | - | - | A | T | N | V | E | A | H | V | L | Q | G | C | G | H | W | V | T | E | E | C | A | P | A | L | N | P | L | I | S | S | F | L | A |
| 053 UniRef90\_A0A6L5BLL6\_28\_324 | - | - | G | D | Q | Q | P | E | N | I | R | R | Y | - | - | - | - | A | A | N | V | E | A | H | V | L | P | G | C | G | H | W | I | P | E | E | C | A | P | A | L | N | P | L | I | K | S | F | L | - |
| 054 UniRef90\_A0A6B1J288\_27\_314 | - | - | G | T | Y | E | T | E | M | L | R | R | Y | - | - | - | - | A | T | N | V | T | G | K | V | L | E | G | C | G | H | W | L | P | E | E | C | S | V | K | L | N | E | T | V | S | V | F | L | Y |
| 055 UniRef90\_A0A263NNG9\_22\_274 | - | - | G | Q | A | Q | I | D | Q | M | N | R | Y | - | - | - | - | A | S | N | V | Q | G | H | V | L | R | G | C | G | H | W | L | M | E | E | C | P | S | L | V | E | P | L | - | - | - | - | - | - |
| 056 UniRef90\_W0HXQ2\_33\_307 | - | - | G | Q | F | Q | V | D | Q | M | R | H | Y | - | - | - | - | A | T | R | V | T | G | H | V | L | P | G | C | G | H | W | L | P | E | E | C | P | A | T | - | - | - | - | - | - | - | - | - | - |
| 057 UniRef90\_A0A031HKS8\_40\_323 | - | - | G | R | L | E | G | D | Q | L | K | E | Y | - | - | - | - | A | I | D | V | R | G | E | V | L | P | G | C | G | H | W | L | P | E | E | C | A | A | T | L | N | P | M | V | A | A | F | L | - |
| 058 UniRef90\_A0A4R2FY05\_27\_320 | - | - | G | A | T | Q | F | N | Q | M | K | E | Y | - | - | - | - | A | N | D | V | Q | G | H | V | L | E | S | C | G | H | W | L | M | E | E | C | P | V | Q | V | E | D | L | V | I | D | F | - | - |
| 059 UniRef90\_A0A1X0N2L9\_36\_321 | - | - | G | A | M | Q | P | E | N | I | R | R | Y | - | - | - | - | A | T | H | V | E | A | H | V | L | S | G | C | G | H | W | V | P | E | E | C | S | S | V | L | N | P | L | I | S | S | F | L | - |
| 060 UniRef90\_A0A4R1U0K9\_37\_322 | - | - | G | V | S | Q | P | E | N | I | R | R | Y | - | - | - | - | A | T | N | V | K | A | H | V | L | P | G | C | G | H | W | V | P | E | E | C | A | P | A | L | D | P | L | I | T | S | F | L | - |
| 061 UniRef90\_A0A0Q8TEQ0\_42\_323 | - | - | G | A | Y | Q | G | E | Q | L | R | K | Y | - | - | - | - | A | V | N | V | Q | S | E | T | I | Q | G | C | G | H | W | L | P | E | E | C | P | N | E | L | N | S | L | V | V | N | F | L | - |
| 062 UniRef90\_A0A0Q6ZEJ0\_41\_323 | - | - | G | A | Y | Q | G | E | Q | L | G | N | Y | - | - | - | - | A | V | N | V | K | A | Q | T | V | Q | G | C | G | H | W | L | P | E | E | C | P | T | E | I | N | S | L | V | V | D | F | - | - |
| 063 UniRef90\_UPI0004C0CBB1\_54\_338 | - | - | G | D | S | I | A | D | Q | W | R | K | Y | - | - | - | - | A | V | N | V | E | G | R | V | L | N | D | S | G | H | W | V | T | E | E | K | P | Q | E | V | T | A | M | L | Q | S | F | L | Q |
| 064 UniRef90\_UPI0004CAB4F8\_56\_339 | - | - | G | G | K | I | A | D | Q | W | R | D | Y | - | - | - | - | A | V | H | V | D | G | R | V | L | K | G | S | G | H | W | V | T | E | E | K | P | Q | E | V | T | A | M | L | Q | S | F | L | Q |
| 065 UniRef90\_UPI00049002CC\_60\_343 | - | - | G | G | L | I | A | D | Q | W | R | D | Y | - | - | - | - | A | V | N | V | G | G | R | V | L | K | N | S | G | H | F | V | T | E | E | K | P | Q | E | V | T | A | L | L | Q | S | F | L | Q |
| 066 UniRef90\_A0A561IX74\_59\_327 | - | - | A | A | F | L | V | E | H | C | K | L | V | - | - | - | - | A | E | N | V | T | G | S | N | I | K | G | A | G | H | W | I | V | Q | E | N | T | E | Q | V | Q | K | D | L | L | D | F | F | - |
| 067 UniRef90\_A0A0X3RYG7\_67\_350 | - | - | G | S | G | V | P | N | Q | W | H | E | Y | - | - | - | - | A | E | D | V | Q | G | R | V | L | A | K | T | G | H | F | V | N | E | E | R | P | K | E | V | T | A | A | L | Q | S | F | L | Q |
| 068 UniRef90\_A0A2W2E1M5\_55\_338 | - | - | G | S | M | I | P | D | Q | W | R | E | Y | - | - | - | - | A | V | N | V | Q | G | R | V | L | K | N | T | G | H | F | V | T | E | E | R | P | K | E | V | T | A | M | L | Q | S | F | L | Q |
| 069 UniRef90\_A0A2V7YB42\_28\_306 | - | - | G | D | L | L | G | R | Q | A | K | R | V | - | - | - | - | A | S | N | V | T | V | V | V | L | K | D | T | G | H | W | L | M | E | E | R | P | R | E | T | M | E | A | L | S | S | F | L | - |
| 070 UniRef90\_A0A544XC49\_52\_336 | - | - | G | S | R | V | P | D | Q | W | R | E | Y | - | - | - | - | A | V | N | V | Q | G | R | V | L | K | N | T | G | H | F | V | T | E | E | R | P | K | E | V | T | A | L | L | Q | S | F | L | Q |
| 071 UniRef90\_A0A101PQ90\_46\_324 | - | - | G | S | S | V | A | K | Q | V | R | H | Y | - | - | - | - | A | T | H | V | T | P | A | V | I | P | D | S | G | H | W | I | Y | E | E | H | P | T | E | T | T | D | M | L | L | H | F | - | - |
| 072 UniRef90\_A0A1C4VK67\_14\_288 | - | - | G | E | K | L | A | E | Q | V | G | R | Y | - | - | - | - | A | D | T | V | R | G | E | V | V | E | D | C | G | H | W | L | F | E | E | R | P | D | Q | M | L | A | L | L | R | D | F | L | - |
| 073 UniRef90\_A0A0X3RPC2\_57\_340 | - | - | G | R | M | V | P | D | Q | W | R | N | Y | - | - | - | - | A | V | N | V | Q | G | R | V | L | K | N | S | G | H | F | V | T | E | E | K | P | R | E | V | T | A | M | L | Q | S | F | L | Q |
| 074 UniRef90\_A0A4R2VWW4\_55\_319 | - | - | A | A | F | L | T | E | H | C | K | L | V | - | - | - | - | A | E | N | V | K | G | S | N | I | K | G | A | G | H | W | V | V | Q | E | N | T | A | Q | V | Q | Q | D | L | L | N | F | F | - |
| 075 UniRef90\_A0A1Q7VB11\_36\_307 | - | - | G | D | A | L | G | Q | Q | M | K | L | V | - | - | - | - | A | T | D | V | T | V | V | V | L | K | D | T | G | H | W | V | L | E | E | R | P | K | E | T | T | G | A | L | V | K | F | - | - |
| 076 UniRef90\_C7PGQ3\_38\_305 | - | - | A | A | F | L | E | D | H | C | K | L | V | - | - | - | - | A | E | H | V | R | G | A | N | I | K | G | S | G | H | W | V | V | Q | E | N | T | A | Q | V | Q | K | E | L | L | D | F | F | - |
| 077 UniRef90\_A0A1N7R4E6\_62\_325 | - | - | A | A | F | L | A | D | H | C | K | L | V | - | - | - | - | A | E | N | V | K | G | S | N | I | K | G | A | G | H | W | V | V | Q | E | N | T | E | Q | V | Q | K | D | L | L | D | F | F | - |
| 078 UniRef90\_A0A2H2Y4V3\_41\_307 | - | - | G | D | L | V | V | K | S | L | Q | Q | V | - | - | - | - | A | V | N | V | R | G | G | A | I | P | R | C | G | H | W | I | A | S | E | R | P | D | Y | L | S | L | Q | L | T | K | F | - | - |
| 079 UniRef90\_A0A448SCU6\_8\_276 | - | - | G | E | I | C | L | R | S | M | Q | Q | V | - | - | - | - | A | H | H | V | E | G | G | V | I | P | E | C | G | H | W | I | A | E | E | K | P | E | F | I | A | T | E | I | I | N | F | - | - |
| 080 UniRef90\_A0A365Y5E2\_33\_303 | - | - | S | S | F | L | P | E | H | C | R | L | V | - | - | - | - | A | D | N | V | K | G | V | I | I | K | D | A | G | H | W | I | V | Q | E | N | T | P | Q | V | Q | K | E | F | L | A | F | L | - |
| 081 UniRef90\_A0A1I2CFD2\_63\_327 | - | - | A | G | F | L | V | D | H | C | K | L | I | - | - | - | - | A | E | N | V | K | G | S | N | I | K | G | A | G | H | W | I | V | Q | E | N | T | E | Q | V | Q | K | D | L | L | D | F | F | - |
| 082 UniRef90\_A0A1G7Z850\_54\_322 | - | - | A | G | F | L | V | D | H | C | K | L | V | - | - | - | - | A | E | H | V | Q | G | S | N | I | K | G | S | G | H | W | V | V | Q | E | N | T | A | Q | V | Q | K | D | L | M | D | F | F | - |
| 083 UniRef90\_A0A2V9XG70\_35\_308 | - | - | G | E | A | L | A | K | Q | M | K | L | V | - | - | - | - | A | S | D | V | T | V | V | V | L | K | D | T | G | H | W | V | L | E | E | R | P | K | E | T | T | E | A | L | Q | K | F | L | - |
| 084 UniRef90\_A0A0Q4GSS4\_39\_308 | - | - | A | P | F | L | A | T | H | V | R | L | V | - | - | - | - | A | N | Q | V | T | E | S | I | I | K | D | S | G | H | W | I | V | Q | E | N | T | P | Q | V | K | K | D | L | L | S | F | L | L |
| 085 UniRef90\_A0A0M9ZTU1\_1\_271 | - | - | G | G | I | I | A | D | Q | L | R | H | Y | - | - | - | - | A | V | N | V | D | G | R | V | L | K | N | C | G | H | F | V | T | D | E | K | P | Q | E | V | T | A | M | L | Q | S | F | L | Q |
| 086 UniRef90\_A0A239P597\_1\_270 | - | - | G | S | M | V | P | D | Q | W | R | E | Y | - | - | - | - | A | V | N | V | Q | G | R | V | L | K | G | S | G | H | F | V | N | E | E | R | P | T | E | V | T | A | L | L | Q | S | F | L | R |
| 087 UniRef90\_UPI00128E3D53\_19\_294 | - | - | N | N | Y | L | G | D | A | M | K | L | E | - | - | - | - | A | S | D | F | V | G | H | I | I | P | D | C | G | H | F | V | V | K | E | A | F | E | E | F | C | A | H | L | D | E | F | L | - |
| 088 UniRef90\_A0A1V9E1W0\_57\_321 | - | - | A | T | Y | L | A | D | H | C | K | L | V | - | - | - | - | A | E | H | V | T | G | T | N | I | K | G | A | G | H | W | I | V | Q | E | N | T | A | E | V | Q | K | G | L | L | D | F | F | - |
| 089 UniRef90\_A0A239MTD5\_1\_269 | - | - | G | G | I | V | V | D | Q | W | R | D | Y | - | - | - | - | A | V | N | V | D | G | R | V | L | K | N | S | G | H | F | V | T | E | E | K | P | Q | E | V | T | A | M | L | Q | S | F | L | - |
| 090 UniRef90\_UPI0009FCBBE8\_54\_317 | - | - | G | P | A | V | G | Q | S | F | A | N | V | - | - | - | - | A | S | D | V | R | T | V | V | A | P | D | S | G | H | W | I | P | E | E | N | P | T | F | L | - | - | - | - | - | - | - | - | - |
| 091 UniRef90\_A0A1A9HTL2\_19\_299 | - | - | G | A | G | M | E | K | T | M | R | L | V | - | - | - | - | A | T | D | V | Q | G | A | T | L | P | G | V | G | H | F | V | L | E | E | S | P | G | E | V | A | A | Q | L | N | A | F | M | - |
| 092 UniRef90\_A0A4R7CM41\_23\_285 | - | - | A | P | Y | L | A | D | H | L | K | L | V | - | - | - | - | A | H | Q | V | Q | A | S | T | I | A | N | A | G | H | W | L | V | Q | E | N | T | A | Q | V | E | K | D | L | L | A | F | - | - |
| 093 UniRef90\_A0A562QYK6\_25\_307 | - | - | G | D | V | L | V | T | Q | A | R | L | V | - | - | - | - | A | D | N | V | E | G | V | V | V | P | G | S | G | H | W | L | M | E | E | A | P | Q | V | V | I | P | K | L | V | Q | F | L | - |
| 094 UniRef90\_A0A2V9ZM06\_3\_269 | - | - | G | D | A | L | A | Q | Q | M | K | S | V | - | - | - | - | A | S | N | V | T | V | V | V | L | K | D | T | G | H | W | V | L | E | E | R | P | K | E | T | T | E | A | L | Q | S | F | L | - |
| 095 UniRef90\_A0A4P8YF40\_11\_271 | - | - | G | E | I | S | F | R | S | M | Q | Q | V | - | - | - | - | A | H | H | V | E | G | G | V | I | P | E | C | G | H | W | I | A | E | E | K | P | D | F | I | A | A | E | - | - | - | - | - | - |
| 096 UniRef90\_A0A2V6E8I4\_30\_303 | - | - | G | N | Q | L | A | Q | Q | M | K | L | V | - | - | - | - | A | S | D | V | T | V | V | V | L | K | D | T | G | H | W | V | L | E | E | R | G | R | E | T | T | D | A | L | V | K | F | - | - |
| 097 UniRef90\_A0A2V9BFT0\_23\_295 | - | - | G | D | A | L | G | Q | Q | M | K | L | V | - | - | - | - | A | T | D | V | T | V | V | V | E | K | D | A | G | H | W | I | M | E | E | Q | A | K | Q | T | M | D | E | L | V | K | F | - | - |
| 098 UniRef90\_A0A2V9H5C8\_29\_298 | - | - | G | E | F | L | G | E | Q | M | K | L | V | - | - | - | - | A | A | D | V | T | A | V | V | L | K | D | T | G | H | W | V | L | E | E | N | P | K | E | T | T | D | A | L | V | K | F | - | - |
| 099 UniRef90\_A0A101CRV3\_44\_308 | - | - | A | A | F | L | K | E | H | T | K | I | V | - | - | - | - | A | N | D | V | R | E | T | N | I | P | N | A | G | H | W | I | V | Q | E | N | T | A | A | V | Q | K | G | L | L | D | F | F | - |
| 100 UniRef90\_A0A5M6CFP9\_57\_321 | - | - | A | A | F | L | E | A | H | C | K | L | V | - | - | - | - | A | E | H | V | S | A | S | N | I | K | G | A | G | H | W | I | V | Q | E | N | T | A | Q | V | Q | K | D | L | L | D | F | F | - |
| 101 UniRef90\_A0A401Z9D4\_19\_284 | - | - | A | G | W | P | F | Y | S | F | A | Q | L | - | - | - | - | A | D | N | V | S | G | G | I | I | P | E | C | G | H | Y | I | A | E | E | Q | P | E | E | L | L | Q | R | L | N | T | F | - | - |
| 102 UniRef90\_A0A2V8XJ58\_32\_308 | - | - | G | E | F | L | G | Q | Q | M | K | L | V | - | - | - | - | A | S | D | L | S | S | V | V | L | K | N | T | G | H | W | I | L | E | E | N | S | K | E | T | T | D | A | L | V | K | F | L | - |
| 103 UniRef90\_A0A252EMP1\_15\_277 | - | - | S | E | I | P | L | R | S | M | K | L | V | - | - | - | - | A | N | N | V | Q | G | G | V | I | P | D | C | G | H | W | V | P | D | E | K | P | E | W | I | A | R | E | I | N | R | F | - | - |
| 104 UniRef90\_A0A1H3H759\_34\_310 | - | - | G | S | L | V | G | E | T | L | A | P | A | - | - | - | - | A | E | D | V | T | S | V | V | L | P | D | C | G | H | Y | P | A | E | E | A | P | Q | E | M | L | A | T | L | T | E | F | L | - |
| 105 UniRef90\_A0A4D7B7N1\_3\_270 | - | - | S | P | N | I | Y | D | A | M | K | P | L | - | - | - | - | G | E | N | V | Q | G | G | I | V | A | D | C | G | H | Y | M | P | E | E | Q | P | E | V | I | A | E | R | M | - | - | - | - | - |
| 106 UniRef90\_A0A1Q7MUT6\_34\_308 | - | - | G | D | A | L | A | Q | Q | M | K | L | V | - | - | - | - | A | S | N | V | T | V | V | V | L | K | D | T | G | H | W | V | L | E | E | R | P | K | E | T | A | E | A | L | Q | K | F | L | - |
| 107 UniRef90\_A0A2V6KQW0\_24\_297 | - | - | G | N | E | L | A | E | Q | M | K | L | V | - | - | - | - | A | D | N | V | A | V | V | V | L | K | D | T | G | H | W | I | L | E | E | R | P | K | E | T | T | D | A | L | V | K | F | - | - |
| 108 UniRef90\_A0A4R6LFZ5\_44\_308 | - | - | A | A | F | L | K | D | H | T | K | L | V | - | - | - | - | A | T | N | V | F | E | T | N | I | P | N | S | G | H | W | I | V | Q | E | N | T | A | A | V | Q | K | G | L | L | D | F | F | - |
| 109 UniRef90\_A0A1H7UU25\_49\_318 | - | - | S | A | F | L | P | E | H | C | R | L | V | - | - | - | - | A | E | H | V | T | S | S | I | I | K | G | A | G | H | W | I | V | Q | E | N | T | P | Q | V | Q | Q | D | L | L | G | F | L | L |
| 110 UniRef90\_UPI0008326796\_14\_259 | - | - | I | P | D | M | S | V | - | - | - | - | - | - | - | - | - | - | - | - | - | - | - | - | - | - | - | - | - | - | - | - | - | - | - | - | - | - | - | - | - | - | - | - | - | - | - | - | - | - |
| 111 UniRef90\_A0A2V5S8F2\_33\_306 | - | - | G | N | Q | L | A | E | Q | M | K | L | V | - | - | - | - | A | N | D | V | T | V | V | V | L | K | D | T | G | H | W | V | L | E | E | R | P | K | E | T | T | D | A | L | V | K | F | - | - |
| 112 UniRef90\_A0A3S3VTN8\_36\_308 | - | - | G | S | F | L | A | E | H | S | K | L | V | - | - | - | - | A | T | D | V | H | E | A | I | I | K | D | S | G | H | W | V | V | Q | E | Q | T | A | Q | V | Q | K | A | L | L | D | F | F | - |
| 113 UniRef90\_A0A6J4PSU4\_16\_248 | - | - | G | E | - | - | - | - | - | - | - | - | - | - | - | - | - | - | - | - | - | - | - | - | - | - | - | - | - | - | - | - | - | - | - | - | - | - | - | - | - | - | - | - | - | - | - | - | - | - |
| 114 UniRef90\_A0A534ZXC6\_26\_300 | - | - | G | E | L | L | G | Q | Q | M | K | L | V | - | - | - | - | A | S | D | A | T | T | V | V | L | P | D | T | G | H | W | V | L | E | E | K | P | D | E | T | I | D | A | L | Q | R | F | L | - |
| 115 UniRef90\_A0A2A9KBX9\_78\_358 | - | - | A | D | L | L | T | N | T | M | K | I | V | - | - | - | - | A | N | D | V | T | G | K | Q | I | N | N | C | G | H | F | V | L | E | E | C | P | K | E | V | T | E | V | L | Q | D | F | - | - |
| 116 UniRef90\_A0A2W0BWH7\_16\_287 | - | - | G | G | V | L | A | N | Q | M | K | L | V | - | - | - | - | A | T | D | V | R | A | V | V | L | N | D | T | G | H | W | V | L | E | E | N | P | K | E | T | T | A | A | L | Q | K | F | L | - |
| 117 UniRef90\_A0A1R1RMU1\_30\_310 | - | - | I | A | D | M | V | T | P | L | R | A | F | - | - | - | - | V | Q | D | V | Q | G | G | N | I | S | F | C | G | H | F | L | P | E | E | Q | P | E | V | V | A | G | E | L | T | A | F | - | - |
| 118 UniRef90\_A0A3A1NH59\_38\_305 | - | - | A | P | F | L | A | E | H | C | R | L | V | - | - | - | - | A | E | E | V | T | E | A | K | I | K | G | S | G | H | W | V | V | Q | E | Q | T | E | Q | V | Q | K | G | L | M | D | F | F | L |
| 119 UniRef90\_A0A0Q6WP84\_43\_317 | - | - | G | D | F | L | I | N | Q | G | K | L | V | - | - | - | - | A | E | N | V | D | G | R | I | I | K | G | A | G | H | W | I | M | E | E | A | P | E | Q | T | I | P | A | L | L | G | F | - | - |
| 120 UniRef90\_A0A2V6P1A8\_15\_289 | - | - | G | N | E | L | A | Q | Q | M | K | L | V | - | - | - | - | A | T | D | A | T | V | V | V | L | K | D | T | G | H | W | I | L | E | E | R | P | K | E | T | T | D | A | L | V | K | F | - | - |
| 121 UniRef90\_A0A2V5NC55\_35\_307 | - | - | G | K | E | L | G | E | Q | A | K | L | V | - | - | - | - | A | D | T | P | G | L | I | V | L | K | D | T | G | H | W | I | L | E | E | R | P | K | E | T | T | E | A | L | V | N | F | L | - |
| 122 UniRef90\_A0A2V9BM40\_39\_318 | - | - | G | D | L | L | G | Q | Q | M | K | I | V | - | - | - | - | A | S | D | A | A | M | V | V | L | K | D | A | G | H | W | V | L | E | E | K | P | K | E | T | T | D | A | L | I | K | F | - | - |
| 123 UniRef90\_A0A5B8WA72\_34\_302 | - | - | G | A | F | L | A | T | H | T | R | L | V | - | - | - | - | A | T | N | V | Q | E | S | I | I | K | D | S | G | H | W | V | V | Q | E | N | T | P | Q | V | Q | K | D | L | L | A | F | L | M |
| 124 UniRef90\_A0A1Y6D2S0\_25\_299 | - | - | A | D | K | L | S | K | A | L | Q | A | K | - | - | - | - | A | D | I | I | K | S | A | I | A | E | D | S | G | H | F | V | P | E | E | A | P | A | F | L | V | E | Q | L | S | S | F | L | - |
| 125 UniRef90\_A0A494W0L3\_12\_284 | - | - | G | S | F | L | A | A | H | S | K | L | V | - | - | - | - | A | D | N | V | Q | E | V | I | I | K | D | S | G | H | W | I | V | Q | E | Q | T | A | Q | V | Q | K | G | L | L | D | F | F | - |
| 126 UniRef90\_UPI0015CD0065\_39\_309 | - | - | G | E | G | V | G | G | S | F | G | Q | V | - | - | - | - | A | D | D | V | R | T | V | V | A | P | D | A | A | H | F | V | P | E | E | N | S | E | F | L | A | K | C | A | N | - | - | - | - |
| 127 UniRef90\_UPI00166E2F5D\_15\_289 | D | I | A | T | Y | M | D | G | F | K | E | F | G | - | - | - | - | L | R | H | I | K | A | K | L | I | E | N | C | G | H | F | S | A | E | E | N | P | E | K | V | A | Y | A | L | K | L | F | - | - |
| 128 UniRef90\_A0A372NPX1\_36\_305 | - | - | G | A | F | L | A | A | H | T | R | L | V | - | - | - | - | A | D | N | V | T | E | S | I | I | K | N | S | G | H | W | V | V | Q | E | N | T | P | Q | V | Q | K | D | L | L | D | F | F | L |
| 129 UniRef90\_A0A317JA08\_13\_279 | - | - | A | A | I | L | G | P | Q | M | K | L | V | - | - | - | - | A | T | T | V | T | A | V | E | M | K | D | T | G | H | W | L | M | E | E | R | P | E | E | T | M | G | A | L | - | - | - | - | - |
| 130 UniRef90\_A0A2V6N4R6\_10\_283 | - | - | G | N | E | L | A | A | Q | M | K | L | V | - | - | - | - | A | T | D | V | T | V | V | V | L | K | D | T | G | H | W | I | L | E | E | R | P | K | E | T | T | D | A | L | V | K | F | - | - |
| 131 UniRef90\_A0A2V5IZ05\_29\_302 | - | - | G | D | V | L | G | Q | Q | A | H | A | V | - | - | - | - | A | S | D | A | K | V | V | I | L | K | N | T | G | H | W | V | L | E | E | N | P | K | E | T | T | D | A | L | I | N | F | L | - |
| 132 UniRef90\_A0A521E7V8\_39\_308 | - | - | A | P | F | L | A | T | H | T | R | L | V | - | - | - | - | A | D | H | V | E | E | S | V | I | K | D | S | G | H | W | I | V | Q | E | N | T | P | Q | V | Q | K | D | L | L | D | F | F | L |
| 133 UniRef90\_UPI001665F59F\_18\_290 | D | I | E | T | Y | M | R | G | F | S | E | N | G | - | - | - | - | F | Q | H | I | K | A | K | V | I | E | N | C | G | H | F | S | A | E | E | E | P | E | K | V | A | G | A | I | G | E | F | I | - |
| 134 UniRef90\_A0A0Q9GGK2\_19\_284 | - | - | G | D | N | L | R | Q | S | L | L | S | V | - | - | - | - | A | N | N | V | E | G | G | S | I | P | E | C | G | H | F | I | P | E | E | K | P | E | F | L | I | K | V | L | N | S | F | F | - |
| 135 UniRef90\_A0A329YBZ0\_37\_320 | - | - | G | L | T | E | L | S | M | A | K | E | L | - | - | - | - | G | T | D | V | H | G | G | V | A | P | D | T | G | H | W | L | P | D | E | N | P | E | F | L | T | Q | Q | L | I | A | - | - | - |
| 136 UniRef90\_A0A2E0H9P2\_13\_236 | - | - | - | - | - | - | - | - | - | - | - | - | - | - | - | - | - | - | - | - | - | - | - | - | - | - | - | - | - | - | - | - | - | - | - | - | - | - | - | - | - | - | - | - | - | - | - | - | - | - |
| 137 UniRef90\_A0A095SYG7\_44\_308 | - | - | A | A | F | L | K | E | H | T | K | L | V | - | - | - | - | A | N | N | V | Y | E | T | N | I | P | N | S | G | H | W | V | V | Q | E | N | T | T | A | V | Q | Q | G | L | L | D | F | F | - |
| 138 UniRef90\_A0A2U0ZNC3\_54\_322 | - | - | G | S | F | L | A | D | H | T | R | L | V | - | - | - | - | A | D | N | V | T | E | T | K | I | A | G | S | G | H | W | I | V | Q | E | Q | T | G | Q | V | L | S | G | L | M | S | F | L | - |
| 139 UniRef90\_A0A2H2XXU1\_20\_287 | - | - | G | D | R | L | L | K | G | V | Q | A | V | - | - | - | - | A | D | N | V | Q | S | S | V | I | E | K | S | G | H | W | L | A | E | E | Q | P | A | A | L | S | E | R | L | L | T | F | - | - |
| 140 UniRef90\_A0A0P4V0Y0\_11\_278 | - | - | G | E | K | V | K | L | M | M | Q | S | V | - | - | - | - | A | T | D | V | R | G | G | S | V | D | R | C | G | H | F | I | P | D | E | R | P | D | - | - | - | - | - | - | - | - | - | - | - |
| 141 UniRef90\_A0A2D7FF16\_11\_288 | G | R | G | T | S | V | E | E | S | L | R | R | V | - | - | - | - | A | N | N | V | R | G | E | V | I | P | E | C | G | H | F | V | P | E | E | Q | P | E | K | L | T | A | L | L | L | D | F | L | - |
| 142 UniRef90\_UPI0009FE04F9\_77\_354 | - | - | S | D | V | M | V | P | A | V | R | D | A | - | - | - | - | A | P | A | V | S | G | G | E | I | D | G | A | G | H | W | L | V | D | Q | Q | P | D | R | V | V | A | E | I | D | T | - | - | - |
| 143 UniRef90\_UPI00148816F0\_34\_297 | - | - | L | D | S | F | L | Q | A | V | R | D | A | - | - | - | - | A | P | Q | A | T | G | A | A | L | - | D | T | G | H | W | M | L | H | E | A | P | E | A | V | L | T | - | - | - | - | - | - | - |
| 144 UniRef90\_A0A2V5MXX8\_31\_304 | - | - | G | N | E | L | A | Q | Q | M | K | L | V | - | - | - | - | A | A | D | V | T | V | V | V | L | K | D | T | G | H | W | I | L | E | E | R | P | K | E | T | T | D | A | L | V | K | F | - | - |
| 145 UniRef90\_Q1W504\_15\_282 | - | - | G | N | H | V | G | E | L | M | K | P | L | - | - | - | - | A | Q | N | V | K | T | I | V | I | D | R | S | G | H | W | I | P | E | E | Q | P | E | Q | L | V | N | V | L | I | N | F | L | - |
| 146 UniRef90\_UPI001661900D\_10\_259 | - | - | G | D | V | L | A | R | Q | L | R | P | L | - | - | - | - | A | D | D | L | T | A | H | L | I | E | D | C | G | H | I | I | - | - | - | - | - | - | - | - | - | - | - | - | - | - | - | - | - |
| 147 UniRef90\_A0A2D6SJL3\_11\_235 | - | - | - | - | - | - | - | - | - | - | - | - | - | - | - | - | - | - | - | - | - | - | - | - | - | - | - | - | - | - | - | - | - | - | - | - | - | - | - | - | - | - | - | - | - | - | - | - | - | - |
| 148 UniRef90\_A0A2V6J615\_3\_225 | - | - | G | N | E | L | G | A | Q | M | K | L | V | - | - | - | - | A | D | S | V | T | V | I | V | L | P | N | T | G | H | W | M | L | E | E | K | P | K | E | T | T | D | A | L | V | N | F | L | - |

  
  

|  |  |
| --- | --- |
| **001 Input\_protein\_seq** | K |
| 002 UniRef90\_D8JE57\_1\_349 | K |
| 003 UniRef90\_A0A6B8MSH7\_6\_345 | - |
| 004 UniRef90\_A0A6B2FNS7\_11\_352 | K |
| 005 UniRef90\_UPI00073D803C\_6\_344 | - |
| 006 UniRef90\_A0A285K5K8\_3\_346 | R |
| 007 UniRef90\_A0A379I9D6\_1\_346 | - |
| 008 UniRef90\_A0A6B2FTI5\_15\_352 | K |
| 009 UniRef90\_A0A2P9HD85\_2\_318 | - |
| 010 UniRef90\_A0A1T1H7K2\_15\_316 | - |
| 011 UniRef90\_A0A1A9HKQ2\_27\_313 | - |
| 012 UniRef90\_A0A0J6GRA0\_22\_303 | - |
| 013 UniRef90\_UPI000F5BD18A\_25\_314 | - |
| 014 UniRef90\_A0A4V1G750\_26\_311 | - |
| 015 UniRef90\_U3U231\_21\_300 | - |
| 016 UniRef90\_F2LPZ2\_26\_313 | - |
| 017 UniRef90\_A0A3M4V4S1\_6\_330 | - |
| 018 UniRef90\_A0A178LP42\_49\_332 | - |
| 019 UniRef90\_T2L220\_20\_307 | - |
| 020 UniRef90\_UPI0014749FF6\_22\_304 | - |
| 021 UniRef90\_A0A172YJZ3\_18\_305 | - |
| 022 UniRef90\_UPI000737C6BF\_26\_313 | - |
| 023 UniRef90\_A0A329J6I4\_26\_309 | - |
| 024 UniRef90\_A0A2N3KZL1\_30\_325 | - |
| 025 UniRef90\_UPI0006CA847F\_27\_323 | - |
| 026 UniRef90\_UPI00054E0E46\_26\_312 | - |
| 027 UniRef90\_A0A0S9PRE2\_29\_311 | - |
| 028 UniRef90\_W0AA74\_37\_318 | - |
| 029 UniRef90\_A0A2G0Y5Q3\_27\_312 | - |
| 030 UniRef90\_A0A1M6LZ92\_62\_349 | - |
| 031 UniRef90\_A0A4Z1C7D3\_28\_313 | - |
| 032 UniRef90\_A0A367MBG0\_273\_561 | - |
| 033 UniRef90\_UPI00067CEA54\_35\_318 | - |
| 034 UniRef90\_A0A1Y0L5M5\_31\_311 | - |
| 035 UniRef90\_UPI000C2FE79C\_29\_313 | - |
| 036 UniRef90\_A0A4P8SJX7\_14\_295 | - |
| 037 UniRef90\_A0A1I1BZT3\_28\_315 | - |
| 038 UniRef90\_UPI000DF0D36E\_15\_290 | - |
| 039 UniRef90\_UPI00136B8797\_37\_318 | - |
| 040 UniRef90\_A0A1H4CLJ1\_28\_313 | - |
| 041 UniRef90\_UPI000E1FE28F\_29\_313 | - |
| 042 UniRef90\_A0A556AX95\_26\_313 | - |
| 043 UniRef90\_A0A6M8HNY7\_38\_322 | - |
| 044 UniRef90\_UPI001612B72A\_24\_301 | - |
| 045 UniRef90\_UPI0012F66D09\_46\_328 | - |
| 046 UniRef90\_UPI000A94940B\_29\_316 | - |
| 047 UniRef90\_UPI00102FACD2\_29\_312 | - |
| 048 UniRef90\_A0A1S8YNF4\_19\_297 | - |
| 049 UniRef90\_UPI0015755320\_14\_301 | - |
| 050 UniRef90\_A0A427Q4N3\_22\_313 | - |
| 051 UniRef90\_A0A0A3Z1S4\_30\_318 | - |
| 052 UniRef90\_A0A3L8C981\_35\_323 | - |
| 053 UniRef90\_A0A6L5BLL6\_28\_324 | - |
| 054 UniRef90\_A0A6B1J288\_27\_314 | - |
| 055 UniRef90\_A0A263NNG9\_22\_274 | - |
| 056 UniRef90\_W0HXQ2\_33\_307 | - |
| 057 UniRef90\_A0A031HKS8\_40\_323 | - |
| 058 UniRef90\_A0A4R2FY05\_27\_320 | - |
| 059 UniRef90\_A0A1X0N2L9\_36\_321 | - |
| 060 UniRef90\_A0A4R1U0K9\_37\_322 | - |
| 061 UniRef90\_A0A0Q8TEQ0\_42\_323 | - |
| 062 UniRef90\_A0A0Q6ZEJ0\_41\_323 | - |
| 063 UniRef90\_UPI0004C0CBB1\_54\_338 | - |
| 064 UniRef90\_UPI0004CAB4F8\_56\_339 | - |
| 065 UniRef90\_UPI00049002CC\_60\_343 | - |
| 066 UniRef90\_A0A561IX74\_59\_327 | - |
| 067 UniRef90\_A0A0X3RYG7\_67\_350 | - |
| 068 UniRef90\_A0A2W2E1M5\_55\_338 | - |
| 069 UniRef90\_A0A2V7YB42\_28\_306 | - |
| 070 UniRef90\_A0A544XC49\_52\_336 | - |
| 071 UniRef90\_A0A101PQ90\_46\_324 | - |
| 072 UniRef90\_A0A1C4VK67\_14\_288 | - |
| 073 UniRef90\_A0A0X3RPC2\_57\_340 | - |
| 074 UniRef90\_A0A4R2VWW4\_55\_319 | - |
| 075 UniRef90\_A0A1Q7VB11\_36\_307 | - |
| 076 UniRef90\_C7PGQ3\_38\_305 | - |
| 077 UniRef90\_A0A1N7R4E6\_62\_325 | - |
| 078 UniRef90\_A0A2H2Y4V3\_41\_307 | - |
| 079 UniRef90\_A0A448SCU6\_8\_276 | - |
| 080 UniRef90\_A0A365Y5E2\_33\_303 | - |
| 081 UniRef90\_A0A1I2CFD2\_63\_327 | - |
| 082 UniRef90\_A0A1G7Z850\_54\_322 | - |
| 083 UniRef90\_A0A2V9XG70\_35\_308 | - |
| 084 UniRef90\_A0A0Q4GSS4\_39\_308 | - |
| 085 UniRef90\_A0A0M9ZTU1\_1\_271 | - |
| 086 UniRef90\_A0A239P597\_1\_270 | - |
| 087 UniRef90\_UPI00128E3D53\_19\_294 | - |
| 088 UniRef90\_A0A1V9E1W0\_57\_321 | - |
| 089 UniRef90\_A0A239MTD5\_1\_269 | - |
| 090 UniRef90\_UPI0009FCBBE8\_54\_317 | - |
| 091 UniRef90\_A0A1A9HTL2\_19\_299 | - |
| 092 UniRef90\_A0A4R7CM41\_23\_285 | - |
| 093 UniRef90\_A0A562QYK6\_25\_307 | - |
| 094 UniRef90\_A0A2V9ZM06\_3\_269 | - |
| 095 UniRef90\_A0A4P8YF40\_11\_271 | - |
| 096 UniRef90\_A0A2V6E8I4\_30\_303 | - |
| 097 UniRef90\_A0A2V9BFT0\_23\_295 | - |
| 098 UniRef90\_A0A2V9H5C8\_29\_298 | - |
| 099 UniRef90\_A0A101CRV3\_44\_308 | - |
| 100 UniRef90\_A0A5M6CFP9\_57\_321 | - |
| 101 UniRef90\_A0A401Z9D4\_19\_284 | - |
| 102 UniRef90\_A0A2V8XJ58\_32\_308 | - |
| 103 UniRef90\_A0A252EMP1\_15\_277 | - |
| 104 UniRef90\_A0A1H3H759\_34\_310 | - |
| 105 UniRef90\_A0A4D7B7N1\_3\_270 | - |
| 106 UniRef90\_A0A1Q7MUT6\_34\_308 | - |
| 107 UniRef90\_A0A2V6KQW0\_24\_297 | - |
| 108 UniRef90\_A0A4R6LFZ5\_44\_308 | - |
| 109 UniRef90\_A0A1H7UU25\_49\_318 | - |
| 110 UniRef90\_UPI0008326796\_14\_259 | - |
| 111 UniRef90\_A0A2V5S8F2\_33\_306 | - |
| 112 UniRef90\_A0A3S3VTN8\_36\_308 | - |
| 113 UniRef90\_A0A6J4PSU4\_16\_248 | - |
| 114 UniRef90\_A0A534ZXC6\_26\_300 | - |
| 115 UniRef90\_A0A2A9KBX9\_78\_358 | - |
| 116 UniRef90\_A0A2W0BWH7\_16\_287 | - |
| 117 UniRef90\_A0A1R1RMU1\_30\_310 | - |
| 118 UniRef90\_A0A3A1NH59\_38\_305 | K |
| 119 UniRef90\_A0A0Q6WP84\_43\_317 | - |
| 120 UniRef90\_A0A2V6P1A8\_15\_289 | - |
| 121 UniRef90\_A0A2V5NC55\_35\_307 | - |
| 122 UniRef90\_A0A2V9BM40\_39\_318 | - |
| 123 UniRef90\_A0A5B8WA72\_34\_302 | K |
| 124 UniRef90\_A0A1Y6D2S0\_25\_299 | - |
| 125 UniRef90\_A0A494W0L3\_12\_284 | - |
| 126 UniRef90\_UPI0015CD0065\_39\_309 | - |
| 127 UniRef90\_UPI00166E2F5D\_15\_289 | - |
| 128 UniRef90\_A0A372NPX1\_36\_305 | K |
| 129 UniRef90\_A0A317JA08\_13\_279 | - |
| 130 UniRef90\_A0A2V6N4R6\_10\_283 | - |
| 131 UniRef90\_A0A2V5IZ05\_29\_302 | - |
| 132 UniRef90\_A0A521E7V8\_39\_308 | K |
| 133 UniRef90\_UPI001665F59F\_18\_290 | - |
| 134 UniRef90\_A0A0Q9GGK2\_19\_284 | - |
| 135 UniRef90\_A0A329YBZ0\_37\_320 | - |
| 136 UniRef90\_A0A2E0H9P2\_13\_236 | - |
| 137 UniRef90\_A0A095SYG7\_44\_308 | - |
| 138 UniRef90\_A0A2U0ZNC3\_54\_322 | - |
| 139 UniRef90\_A0A2H2XXU1\_20\_287 | - |
| 140 UniRef90\_A0A0P4V0Y0\_11\_278 | - |
| 141 UniRef90\_A0A2D7FF16\_11\_288 | - |
| 142 UniRef90\_UPI0009FE04F9\_77\_354 | - |
| 143 UniRef90\_UPI00148816F0\_34\_297 | - |
| 144 UniRef90\_A0A2V5MXX8\_31\_304 | - |
| 145 UniRef90\_Q1W504\_15\_282 | - |
| 146 UniRef90\_UPI001661900D\_10\_259 | - |
| 147 UniRef90\_A0A2D6SJL3\_11\_235 | - |
| 148 UniRef90\_A0A2V6J615\_3\_225 | - |

  
  

|  |  |  |  |  |  |  |  |  |  |  |  |  |  |  |  |  |  |
| --- | --- | --- | --- | --- | --- | --- | --- | --- | --- | --- | --- | --- | --- | --- | --- | --- | --- |
| |  |  |  |  |  |  |  |  |  | | --- | --- | --- | --- | --- | --- | --- | --- | --- | | 1 | 2 | 3 | 4 | 5 | 6 | 7 | 8 | 9 |   |  |  |  |  |  |  | | --- | --- | --- | --- | --- | --- | | **Variable** |  | **Average** |  | **Conserved** | | | |  |  |  | | --- | --- | | **X | - Insufficient data - the calculation for this site was performed on less than 10% of the sequences. |** |
